# Small RNA sequencing reveals a novel tsRNA‐06018 playing an important role during adipogenic differentiation of hMSCs

**DOI:** 10.1111/jcmm.15858

**Published:** 2020-09-16

**Authors:** Tao Wang, Lingling Cao, Shan He, Kai Long, Xinping Wang, Hui Yu, Baicheng Ma, Xiaoyuan Xu, Weidong Li

**Affiliations:** ^1^ Key Laboratory of System Bio‐medicine of Jiangxi Province Jiujiang University Jiujiang China; ^2^ Department of Endocrinology Jiujiang Hospital Affiliated to Nanchang University Jiujiang China

**Keywords:** adipogenic differentiation, extracellular signal‐regulated kinase 1/2, human bone marrow mesenchymal stem cells, stanniocalcin 2, tRNA‐derived small RNAs

## Abstract

Transfer RNA‐derived small RNAs (tsRNAs), a novel type of non‐coding RNA derivative, are able to regulate a wide range of biological processes. What role these tsRNAs play in the regulation of human bone marrow mesenchymal stem cell (hMSCs) adipogenic differentiation remains uncertain. We induced the adipogenic differentiation of human bone marrow mesenchymal cells (hMSCs) and then performed small RNA transcriptomic sequencing, leading us to identify tsRNA‐06018 as a target of interest based upon resultant the tsRNA expression profiles. When tsRNA‐06018 was knocked down, this led to the inhibition of adipogenesis and a decrease in adipogenic marker expression. When STC2 was overexpressed, this impaired the adipogenic differentiation of these cells. We further used luciferase reporter assays to confirm that tsRNA‐06018 directly binds the 3′‐untranslated region (3′‐UTR) of STC2. In addition, we determined that both knocking down tsRNA‐06018 and overexpressing STC2 increased extracellular signal‐regulated kinase 1/2 (ERK1/2) phosphorylation within cells. We also assessed that the adipogenic differentiation of hMSCs in which tsRNA‐06018 was knocked down was further enhanced upon the addition of the ERK1/2 inhibitor U0126 as compared tsRNA‐06018 knockdown alone. Taken together, using small RNA sequencing we profiled tsRNAs in hMSCs during the process of adipogenesis, leading us to identify tsRNA‐06018 as a novel regulator of this differentiation process. This tsRNA was able to regulate adipogenic differentiation by targeting STC2 via the ERK1/2 signalling pathway.

## 1 INTRODUCTION

Adipogenesis is a process whereby pre‐adipocytic cells mature into fully fledged adipocytes that efficiently store fat.^1^ Adipose tissue dysfunction is associated with obesity and may contribute to closely related conditions including cardiovascular disease as well as type II diabetes mellitus.^2^, ^3^ As such, a better understanding of the adipogenic process has the potential to significantly benefit human health. Human bone marrow mesenchymal stem cells (hMSCs) are cells that are capable of undergoing sustained self‐renewal and of differentiating into a wide range of cell types,^4^ such as adipocytes,^5^ chondrocytes^6^ and osteoblasts.^7^ Given these differentiation characteristics, hMSCs that have been expanded in vitro represent an ideal model system for exploring the molecular mechanisms of adipogenesis in human cells.^8^

An increasingly large body of research has shown that small non‐coding RNAs (sncRNAs) are essential regulators of a wide range of biological processes such as cellular differentiation and proliferation,^9^, ^10^ as well as cell responses to stress.^11^ Research into the role of these sncRNAs has shown them to have broad and profound effects on biological systems, with miRNAs, which are the best studied subtype of sncRNA, having a marked role in governing the regulation of gene expression.^12^ There has also been substantial interest in and progress towards using these sncRNAs as tools for biological research and/or therapeutic intervention in the context of disease owing to their ability to silence gene expression in a specific and targeted manner.^13^

Advances in sequencing technologies in recent decades have led to changes in the scientific understanding of sncRNA production from tRNA loci, as several recent studies have revealed these loci to produce tRNA fragments (tRFs) that are also referred to as tRNA‐derived small RNAs (tsRNAs).^14^, ^15^, ^16^, ^17^ Previous studies have classified these tsRNAs into 6 different classes based on their specific tRNA origins.^18^, ^19^, ^20^ When tRNAs are cleaved at the anticodon site by angiogenin, they are referred to 5′‐tRNA halves, while the other halves of these molecules following such cleavage are referred to as 3′‐tRNA halves. When mature tRNAs are instead cleaved at the D‐loop or anticodon step, the resultant products are referred to as tRFs‐5, while cleavage at the T‐loop or anticodon stem yields tRFs‐3. Internal tRFs (i‐tRFs) are fragments that are contained within a mature tRNA. Lastly, tRFs‐1 are produced from fragments of the 3′ end of primary tRNAs.

As these tsRNAs are widely expressed across multiple models and biological systems, there is significant interest in determining whether they have functional relevance in the context of normal physiology and/or disease. There is evidence to support that these tsRNAs can act through different mechanisms to mediate tumour suppression,^21^ cancer cell ribosomal biogenesis,^22^ paternal epigenetic inheritance^23^, ^24^ and LTR‐retrotransposon control.^25^ However, the functional roles of tsRNAs during the adipogenic differentiation of hMSCs remain largely unclear.

In the present study, we utilized high‐throughput sequencing in order to identify tsRNAs that are differentially expressed in the context of adipogenesis, and we then subjected these tsRNAs to bioinformatics analyses to explore their potential role in this process. We additionally identified the novel tsRNA‐06018 (tRF‐3) as a tsRNA that regulates the adipogenic differentiation of hMSCs by targeting Stanniocalcin 2 (STC2) via the extracellular signal‐regulated kinase 1/2 (ERK1/2) signalling pathway. Together, these findings highlight a poorly understood role for tsRNAs in the context of adipogenic differentiation.

## 2 MATERIALS AND METHODS

### 2.1 Cell culture and adipogenesis

For this study, we utilized hMSCs (passage 2; HUXMA‐01001; Cyagen Biosciences, Guangzhou, China) that were ≥95% CD73, CD90 and CD105 positive and ≤5% CD11b, CD19, CD45, CD34 and CD HLA‐DR negative as assessed via flow cytometry. We grew these cells at 5 × 10^4^ cells/cm^2^ in OriCell hMSC Growth Media (HUXMA‐90011; Cyagen Biosciences), containing L‐glutamine, 10% FBS, and penicillin/streptomycin in a 5% CO2 37°C humidified incubator. Trypsin‐EDTA (Invitrogen, CA, USA) was used to passage cells every third day, with cells from the sixth passage being used in experiments.

For adipogenic differentiation, hMSCs were grown until confluent for 2 days, after which the media were exchanged for DMEM supplemented with 10% FBS, 10% g/mL insulin, 0.5 mmol/L 3‐isobutyl‐1‐methylxanthine and 0.5 mmol/L dexamethasone.^26^ Cells were cultured in this media for 21 total days, with cells being collected after 0, 7, 14 and 21 days for high‐throughput sequencing analysis.

### 2.2 Library construction and small RNA‐seq

Trizol (Invitrogen) was used to extract RNA from cells, after which an Agilent 2200 machine was used to assess RNA quality prior to storage at −80°C. RNA samples with an integrity score >7.0 were used to prepare cDNA libraries.

To produce small RNA sequencing libraries, we utilized an NEBNext Small RNA Library Prep Kit for Illumina. Briefly, RNA was ligated to the provided 5′ and 3′ adapters, followed by first strand cDNA synthesis. Index PCR was then used in order to apply index sequences and Illumina sequence adapters. Library purification was then performed, and a Bioanalyzer 2200 (Agilent, CA, USA) was used for quality control followed by sequencing on a HiSeq X‐ten platform (Illumina, CA, USA) using 150 bp paired‐end reads.

### 2.3 Small RNA analysis

Trim Galore was used to remove the adaptor sequences from the raw data and to eliminate low quality reads. The remaining clean reads between 12 and 50 nucleotides in length were aligned to sequences in miRBase (http://www.mirbase.org/), with BWA being used for known miRNA identification. Remaining unmapped reads were then aligned with rRNA (https://rnacentral.org/) to eliminate these rRNA fragments, after which we used an internally designed tRNA sequence database (using sequences from http://gtrnadb.ucsc.edu/ and https://cm.jefferson.edu/MINTbase/) to analyse the remaining sequences. We first removed all intronic sequences and then added CCA to the end of each tRNA sequence. Next, 50 nucleotides from the genome were added behind the CCA residues. The resultant mapped reads were considered as being possible tsRNAs and were classified according to tRFdb (http://genome.bioch.virginia.edu/trfdb/) and MINTBase (https://cm.jefferson.edu/MINTbase/).

### 2.4 Differential small RNA expression and target gene prediction

In order to filter differentially expressed miRNAs and tsRNAs, we used the EB‐Seq algorithm,^27^ after which P value and FDR significance analyses^28^ were conducted with the following cut‐off criteria: (a) Fold Change >2 or <0.5; (b) *P* < .05, FDR < 0.05. We then used the miRanda^29^ and RNAhybrid^30^ tools in order to predict the targets of tsRNAs identified in this study. A sequencing data analysis flowchart was shown in Figure S1.

### 2.5 Lentiviral transduction and hMSC positive screening

In order to knockdown tsRNA‐06018 and to overexpress STC2, we used lentiviruses from Shanghai Genechem Co., Ltd. For these experiments, we infected hMSCs using tsRNA‐06018 shRNA or STC2 overexpression lentiviral vector. Serial dilution was used to determine lentiviral titre. For transduction, 5 × 10^4^ hMSCs/cm^2^ were plated in 6‐well plates until 20%‐30% confluent, after which they were infected with 10 µL of lentivirus (1 × 10^8^ infectious units/mL; MOI = 5) in complete growth media containing 5 µg/mL polybrene (Shanghai Genechem Co., Ltd.). Cells were incubated for 10 hours prior to exchanging the transduction media for fresh media and incubating cells for a further 72 hours. After an additional 48 hours culture in 0.5 µg/mL puromycin, cells were screened for 6 days with media being replaced every 1‐2 days. The efficiency of lentiviral transduction was determined via fluorescent microscopy.

At various stages of the adipogenic differentiation process, Oil Red O staining was used to assess the formation of lipid droplets within cells. Cells were then collected to assess mRNA and protein levels of peroxisome proliferator‐activated receptor γ (PPARγ), CCAAT/enhancer‐binding protein α (C/EBPα), Fatty Acid Binding Protein‐4 (FABP4) and STC2.

### 2.6 Oil red O staining

After washing with PBS, cells were fixed for 30 minutes in 10% formalin, washed in 60% isopropanol, and then stained for 10 minutes with Oil red O (0.3%, Sigma‐Aldrich) with gentle shaking. Stained cells were then rinsed with distilled water to remove free dye and imaged under a light microscope. In addition, Oil Red O staining was quantified via treating cells with 100% isopropanol to elute the free dye, which was then analysed via spectrophotometer at 490 nm.^31^

### 2.7 Quantitative real‐time PCR (qRT‐PCR)

TRIzol (Invitrogen) was used to isolate cellular RNA based on provided directions, after which cDNA was prepared with a cDNA Reverse Transcription Kit (Thermo, CA, USA). A SYBR Premix Ex Taq kit (Toyobo, Osaka, Japan) was used for qRT‐PCR reactions on an ABI Prism 7500 real‐time PCR machine (Applied Biosystems). Thermocycler settings were as follows: 95°C for 1 min followed by 40 cycles of 95°C for 15 seconds and 60°C for 34 seconds. Primers used in this study for β‐actin, PPARγ, C/EBPα, FABP4 and STC2 are compiled in Table S1. The $2^{‐\Delta \Delta C_{t}}$ relative expression method was used to assess relative gene expression.

### 2.8 Western blotting

RIPA buffer was used to lyse cells, after which protein was boiled for 5 minutes in 5 × SDS sample buffer, after which we separated 15 µg of each sample via 10% SDS‐PAGE and transferred onto PVDF membranes (EMD Millipore). We then used 5% skim milk as a means of blocking membranes for 2 hours at room temperature, followed by probing overnight at 4°C overnight using the following antibodies: rabbit anti‐PPARγ (abcam 191407), rabbit anti‐ C/EBPα (abcam 40764), rabbit anti‐FABP4 (abcam 92501), rabbit anti‐STC2 (abcam 63057), rabbit anti‐ERK1/2(CST 9102), rabbit anti‐p‐ERK1/2(CST 9101) and mouse anti‐β‐actin (1:2,000; abcam 173838). All rabbit antibodies were diluted 1:1000. We then probed blots with appropriate secondary HRP‐linked antibodies(1:5000; CST) for 1 hours, after which enhanced chemiluminescence (BeyoECL Plus; Beyotime Institute of Biotechnology) was used to visualize proteins.

### 2.9 Luciferase reporter assay

We identified STC2 as a putative tsRNA‐06018 target, and we then used a Dual‐Luciferase Reporter Assay System (Promega, USA) to validate this prediction. Briefly, we cloned the 3′‐UTR of STC2 containing this binding site into the pGL3 vector downstream of luciferase. In addition to this wild‐type (WT) vector, we generated a vector in which this sequence had been mutated (MUT). DNA sequencing was used to validate all constructs. These WT or MUT constructs were transfected into 293T cells which were also transfected with or without a tsRNA‐06018 mimic. After 48 hours, luciferase activity was then assessed, with Renilla used to normalize luciferase activity.

### 2.10 Statistical analysis

SPSS v16.0 (SPSS, IL, USA) was used for statistical analyses. All data are expressed as mean ± standard deviation (SD). Differences within groups were analysed using Student's *t* test. In cases of multiple‐group testing, a one‐way analysis of variance was conducted. A two‐tailed *P* < .05 was the significance threshold.

## 3 RESULTS

### 3.1 tsRNAs expression profile during adipogenesis

**FIGURE 1:**
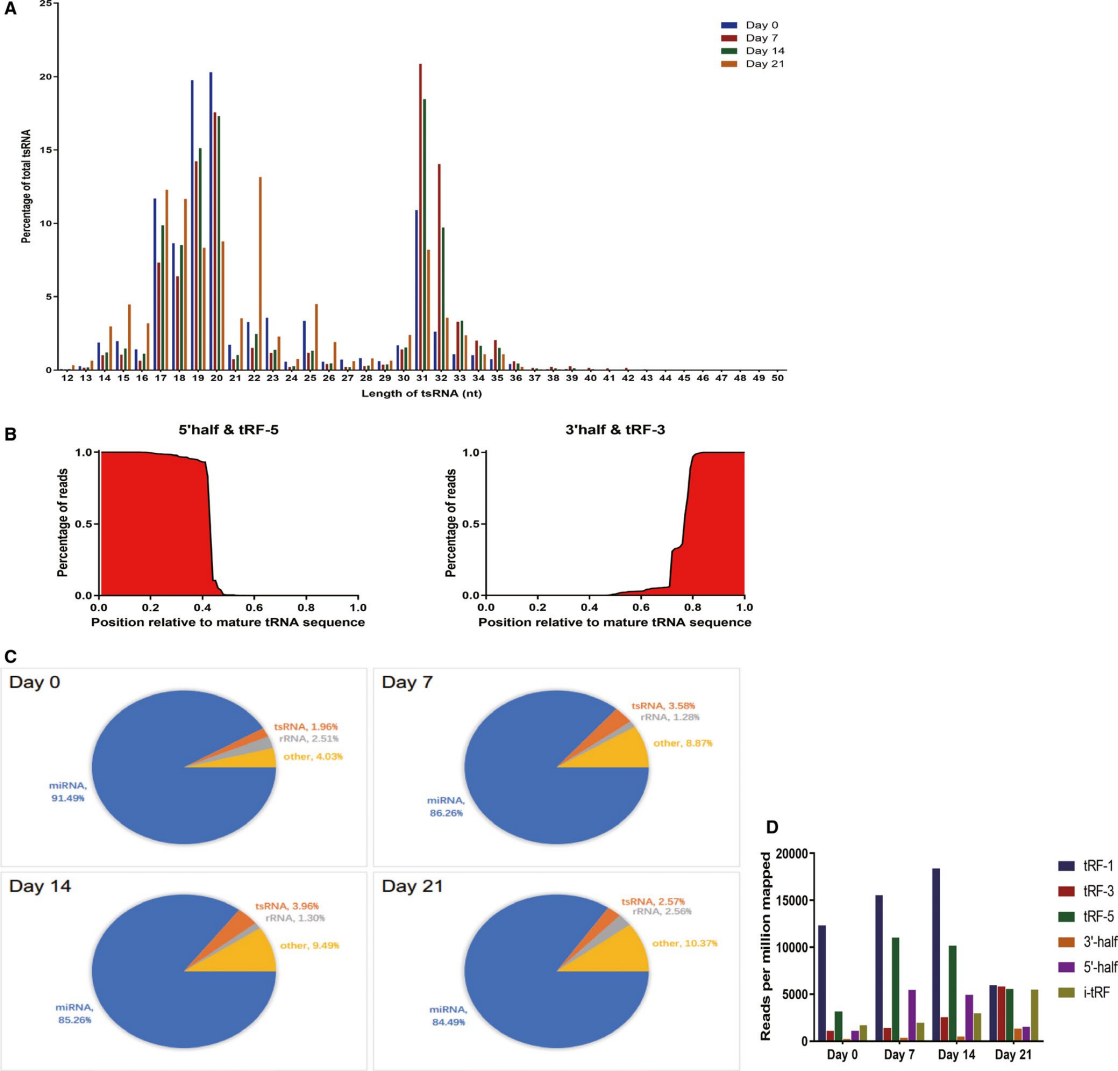
tsRNA expression profiles during adipogenic differentiation. A, Distribution of tsRNA reads during adipogenesis, with frequencies of tsRNA length variants given as a percentage of overall tsRNA reads. B, 5′‐tRF halves and tRF‐5 sequences had similar positions relative to those of the parental tRNAs, and the same was true for most 3’‐tRF halves and tRF‐3 sequences. C, tsRNA proportions among small RNA‐seq data sets during adipogenesis. tsRNA reads are given as a fraction of total library reads. D, Distribution of tRF types during adipogenesis

To determine which tsRNAs were differentially expressed in the context of adipogenesis, we collected samples from hMSCs at different stages in the differentiation process (Days 0, 7, 14 and 21) and then used an Illumina HiSeq X‐ten platform for small RNA sequencing. We then used the GtRNA and piRNA databases to filter out reads between 24 and 33 nucleotides long, allowing us to align these sequences with those present in tRFdb and tRF MINTbase to obtain tsRNA expression profiles (Figure S1). Based on the length distributions of these tsRNAs, we found them to be primarily concentrated in the 17‐23 and 30‐36 nucleotide sizes at all tested time points (Figure 1A). We found that the 5′‐tRF halves and tRF‐5 had positions similar to those of the parental tRNAs from which they were derived, and the same was true for the majority of 3′‐tRF halves and tRF‐3 (Figure 1B).

We found that the overall proportion of these tsRNAs as a fraction of overall ncRNAs at all tested time points during hMSC adipogenesis was increased (Figure 1C). We also found that all six major types of tRFs were produced in the context of adipogenesis, with the reads of these different tRF types varying over the course of days 7, 14, 21 of adipogenesis relative to the day 0 baseline values. The most pronounced increases were evident on days 7 and 14 (Figure 1D).

### 3.2 Differentially expressed tsRNAs

**FIGURE 2:**
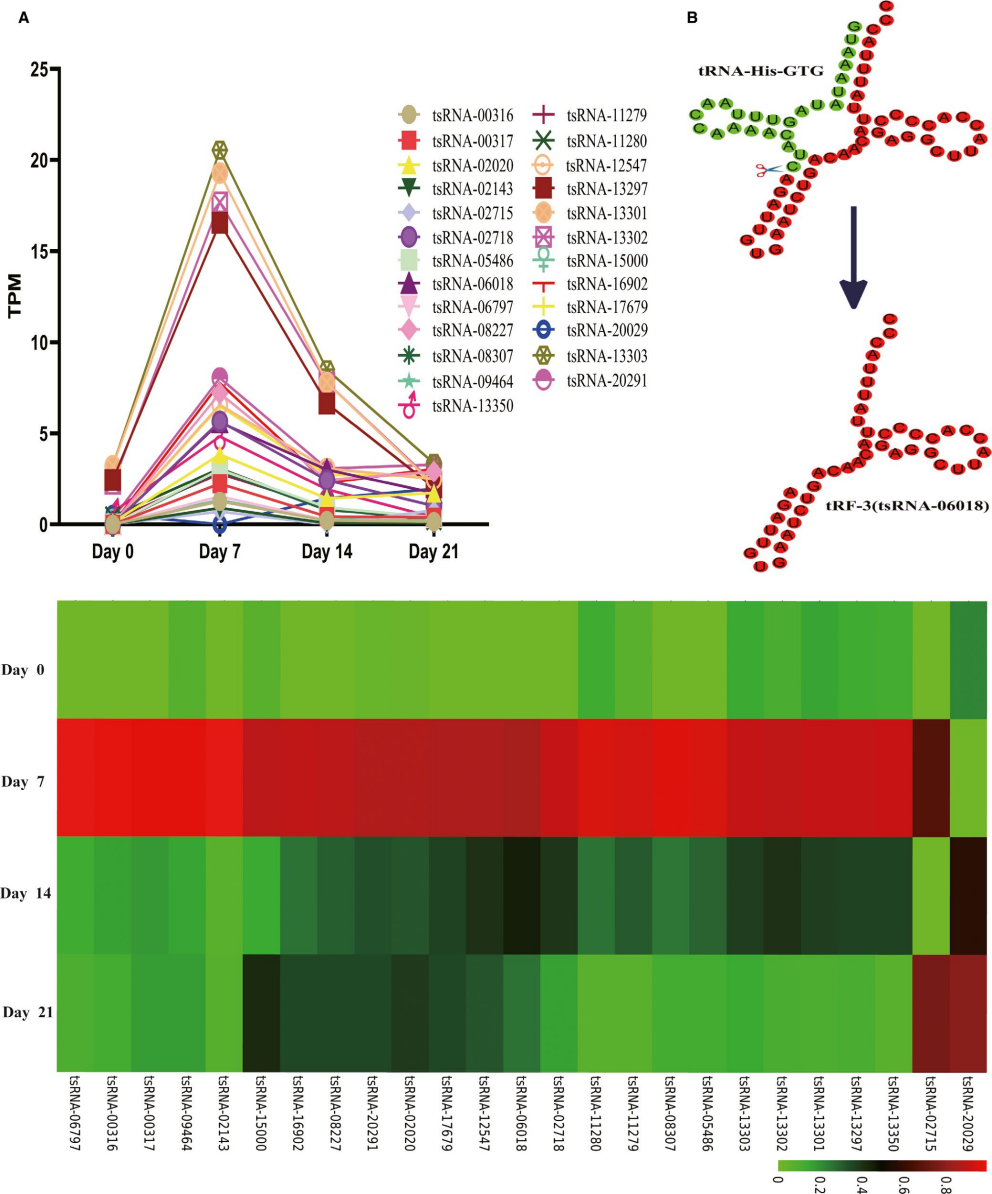
A, tsRNAs differentially expressed during adipogenesis. TPM: Transcripts Per Million Reads. B, tRNA‐His‐GTG was digested to tRF‐3(tsRNA06018; Database ID: tRF‐45‐FN7BWU2F5JYH0RYNUR)

We used the EB‐Seq algorithm in order to identify those tsRNAs that were differentially expressed during the adipogenic process in hMSCs, using Log2FC > 1 or<−1 and FDR < 0.05 as cut‐off criteria to identify tsRNAs that were expressed at different levels when comparing days 7 and 0, days 14 and 7, and days 21 and 14. As shown in Figure 2A, B, the majority of differentially expressed tsRNAs exhibited maximal expression on day 7, leading us to focus on this day 0 vs. day 7 comparison further. At this time point, we analysed six differentially expressed tRFs and focused on tsRNA‐06018 (tRF‐3; Database ID: tRF‐45‐FN7BWU2F5JYH0RYNUR) in our preliminary experiments.

### 3.3 Positive screening of stably transduced hMSCs

**FIGURE 3:**
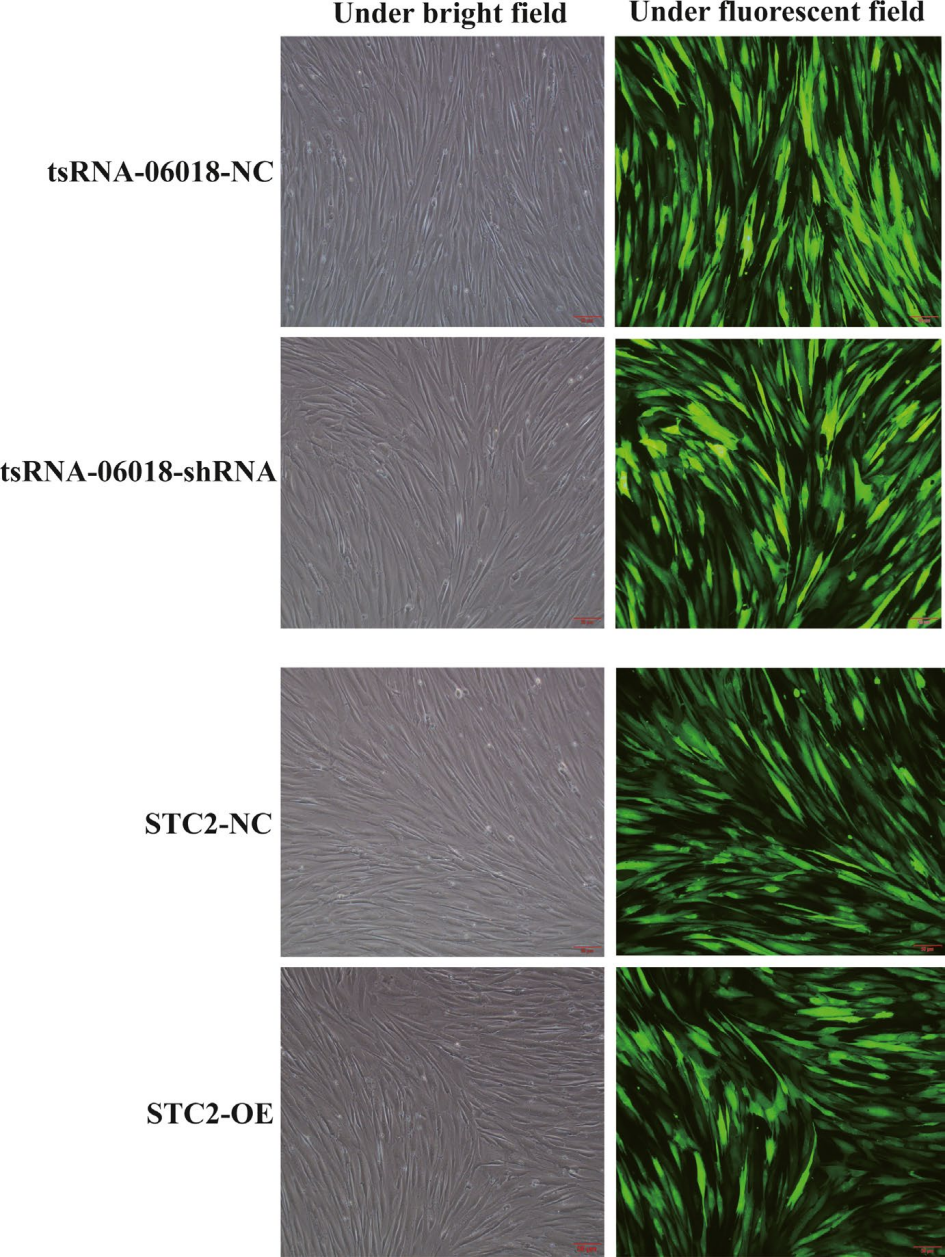
Stably transfected hMSC screening. Infected cells were analysed via light and fluorescent microscopy(10×); scale bar, 50 μm. Note: NC: negative control; OE: overexpression

After 6 days of screening, the surviving cells were resistant to puromycin, and these cells were considered to have been successfully transfected. The surviving cells began to proliferate and exhibited good growth, with clear GFP expression consistent with tsRNA‐06018‐shRNA or STC2 overexpression via stable transduction (Figure 3).

### 3.4 hMSC adipogenesis is inhibited by tsRNA‐06018 knockdown

**FIGURE 4:**
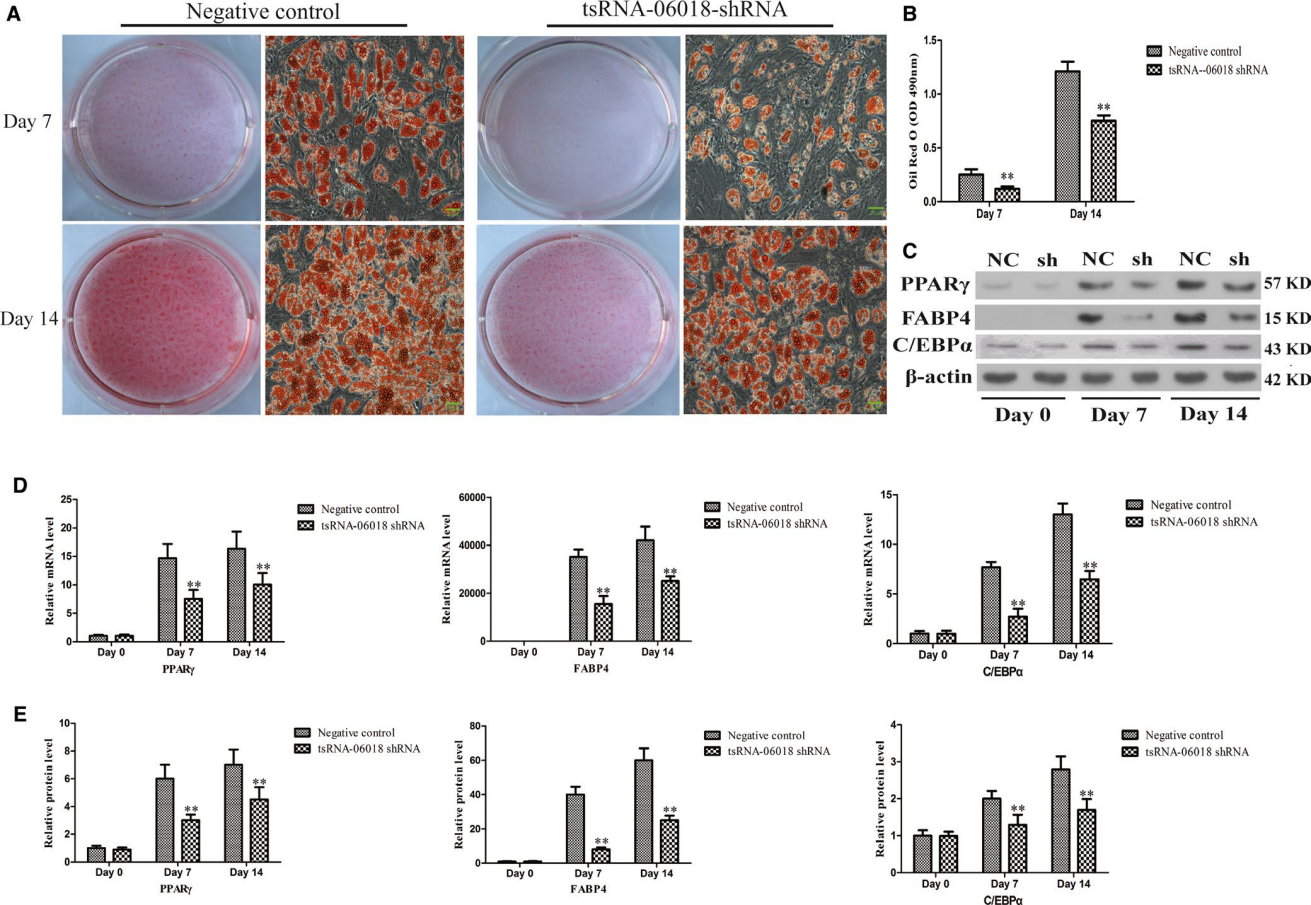
tsRNA‐06018 knockdown impairs the adipogenic differentiation of hMSCs. A, Oil red O staining of hMSCs was analysed during various differentiation stages via light microscopy (20×); scale bar, 20 μm. B, Oil Red O staining was quantified based on absorbance readings in a microplate reader. D, Expression of PPARγ, FABP4, C/EBPα and STC2 was assessed via qRT‐PCR. C, E, PPARγ, FABP4, C/EBPα and STC2 expression were assessed by Western blotting. Data are means ± SD (*n* = 3). **P < .01 vs. negative controls, respectively. Note: NC: negative control; sh: tsRNA‐06018 shRNA

To assess the impact of tsRNA‐06018 on hMSC adipogenesis, we explored the impact of tsRNA‐06018 knockdown on days 0, 7 and 14 of adipogenic differentiation. Oil Red O staining revealed that loss of this tsRNA was associated with significant inhibition of lipid accumulation and adipogenesis (Figure 4A). Consistent with this, quantification of Oil Red O staining results via isopropanol elution confirmed this difference upon knockdown (Figure 4B).

To further explore how the loss of this tsRNA impacted the adipogenic process, we assessed the expression of the adipogenic markers PPARγ, FABP4 and C/EBPα through qRT‐PCR and Western blotting, revealing that tsRNA knockdown markedly reduced PPARγ, FABP4 and C/EBPα expression (Figure 4C‐E). These results suggested a central role for tsRNA‐06018 in adipogenesis.

### 3.5 Overexpressing STC2 impairs adipogenesis

**FIGURE 5:**
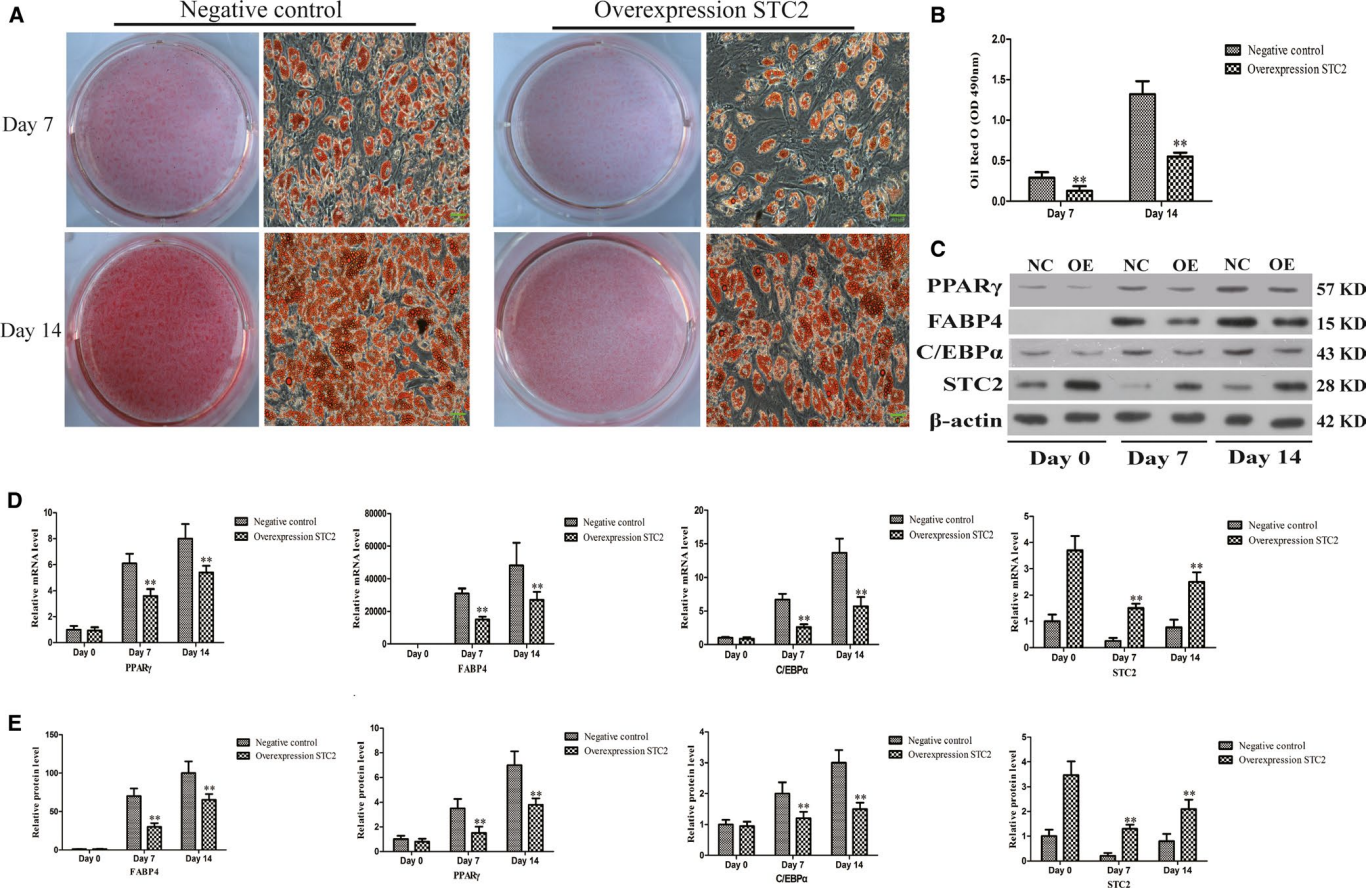
STC2 overexpression impairs the adipogenic differentiation of hMSCs. A, Oil red O staining of hMSCs was analysed during various differentiation stages via light microscopy (20×); scale bar, 20 μm. B, Oil Red O staining was quantified based on absorbance readings in a microplate reader. D, Expression of PPARγ, FABP4, C/EBPα and STC2 was assessed via qRT‐PCR. C, E, PPARγ, FABP4, C/EBPα and STC2 expression were assessed by Western blotting. Data are means ± SD (*n* = 3). **P < 0.01 vs. negative controls, respectively. Note: NC: negative control; OE: STC2 overexpression

We next used the miRanda and RNAhybrid databases to identify potential tsRNA‐06018 target genes, revealing STC2 to be one such possible target based on its sequence complementarity. Should STC2 be important for control of hMSC adipocyte differentiation, overexpression of this gene should impact the adipogenic process. Consistent with this, STC2 overexpression markedly impaired adipogenesis on days 7 and 14 (Figure 5A‐B). This overexpression was also associated with reduced expression of PPARγ, C/EBPα and FABP4 (Figure 5C‐E).

### 3.6 tsRNA‐06018 directly targets STC2

**FIGURE 6:**
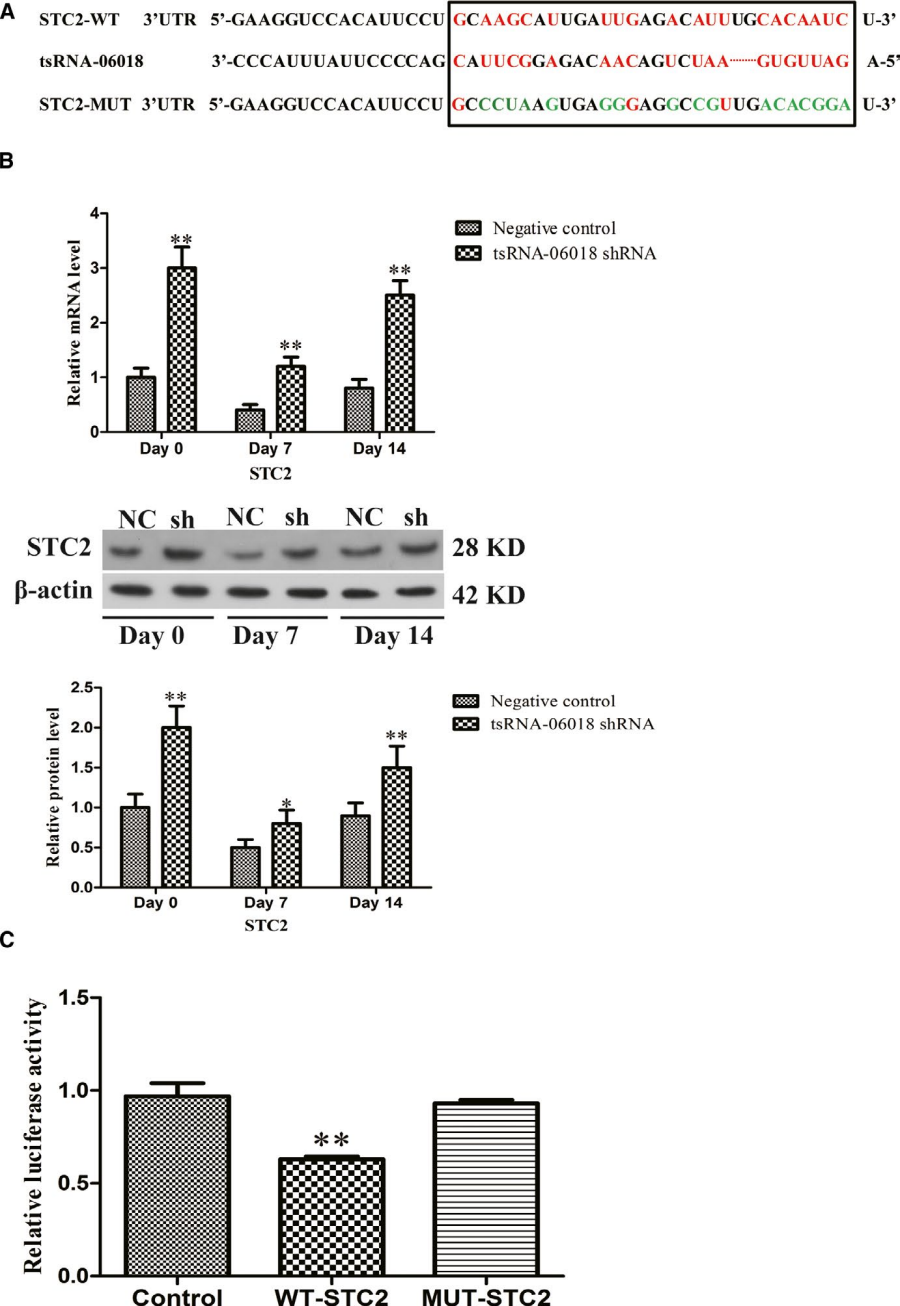
tsRNA‐06018 targets the 3′‐UTR of STC2. A, The tsRNA‐06018 binding region in the STC2 3′‐UTR. Mutated nucleotides in the mutant STC2 3'‐UTR seed sequences are represented in green. B, We assessed the impact of tsRNA‐06018 on STC2 expression during hMSC adipogenic differentiation. C, Luciferase activity assay. Data are means ± SD (*n* = 3). **P < .01 vs. negative controls, respectively. Note: NC: negative control; sh: tsRNA‐06018 shRNA

We next sought to confirm a direct interaction between tsRNA‐06018 and the STC2 mRNA, thus explaining their regulatory relationship during adipogenesis. Initially, Targetscan predictive analyses identified a tsRNA‐06018 binding site in the STC2 3′‐UTR (Figure 6A). Then we assessed the effect of tsRNA‐06018 on levels of STC2 expression during different stages of adipogenesis (Figure 6B). To confirm that this putative binding site was indeed valid, we use a luciferase reporter system. Using this system, we confirmed that tsRNA‐06018 transfection resulted in a 40% reduction in luciferase activity in cells expressing a WT but not a mutated version of this 3’‐UTR from STC2 (Figure 6C).

### 3.7 Knockdown of tsRNA‐06018 and overexpression of STC2 increased ERK1/2 phosphorylation

**FIGURE 7:**
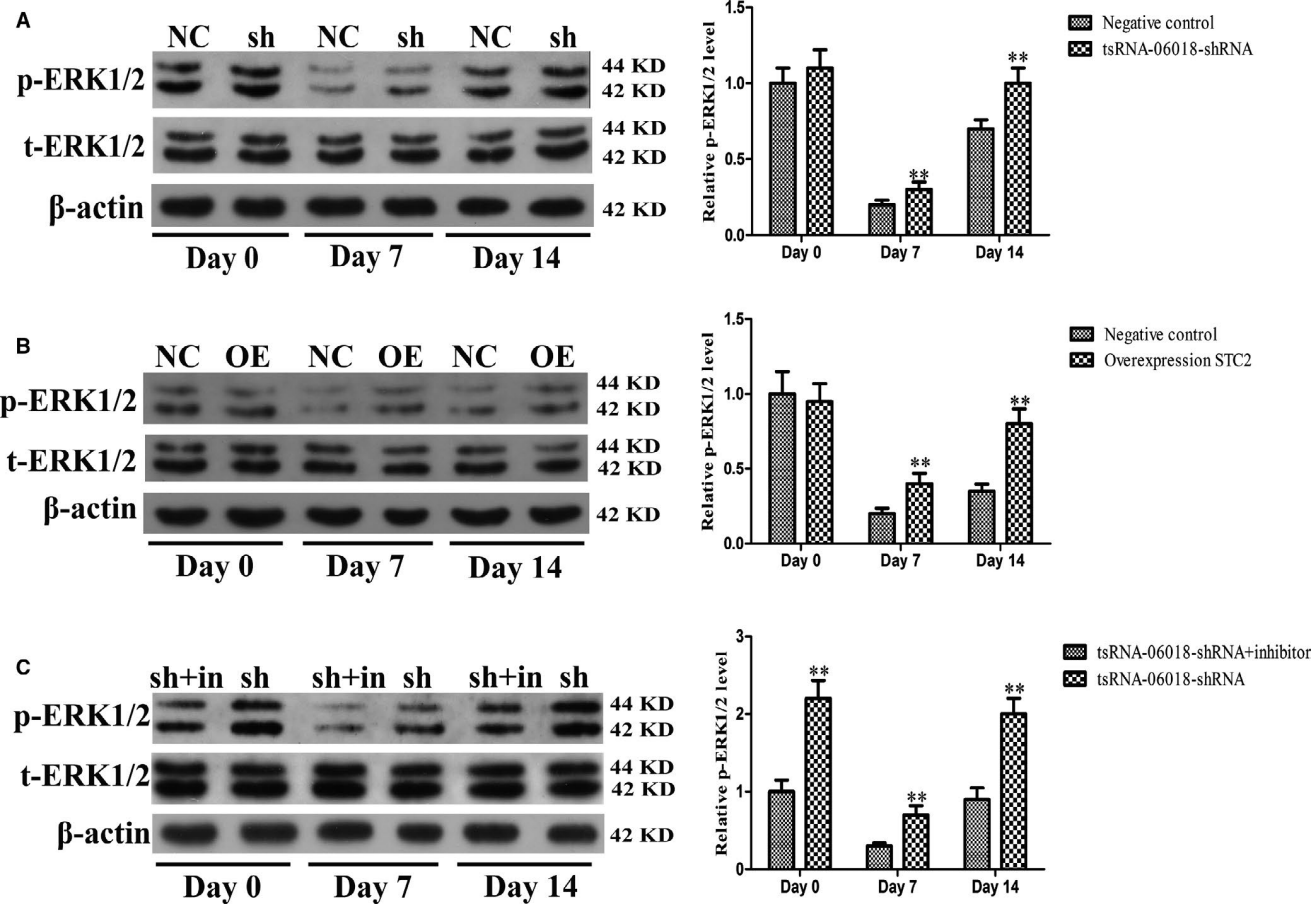
Assessment of p‐ERK1/2 levels during the adipogenic differentiation of hMSCs. A, tsRNA‐06018 shRNA enhanced p‐ERK1/2 levels. B, STC2 overexpression enhances p‐ERK1/2 levels. C, The ERK1/2 inhibitor U0126 reduced p‐ERK1/2 levels in these cells. Data are means ± SD (*n* = 3). **P < .01 vs. negative controls, or tsRNA‐06018‐shRNA + inhibitor respectively. Note: NC: negative control; sh: tsRNA‐06018‐shRNA; OE: STC2 overexpression; sh + in: tsRNA‐06018‐shRNA + inhibitor

We lastly explored whether alterations in tsRNA‐06018 and STC2 expression impacted extracellular signal‐regulated kinase 1/2 (ERK1/2) signalling during adipogenesis. To that end, we assessed p‐ERK1/2 levels in stably transduced hMSCs via Western blotting at different time points during adipogenic differentiation, revealing that both tsRNA‐06018 knockdown and STC2 overexpression were linked with elevated p‐ERK1/2 (Figure 7A‐B).

### 3.8 The ERK1/2 inhibitor U0126 reverses tsRNA‐06018 knockdown‐mediated hMSC adipogenic differentiation

**FIGURE 8:**
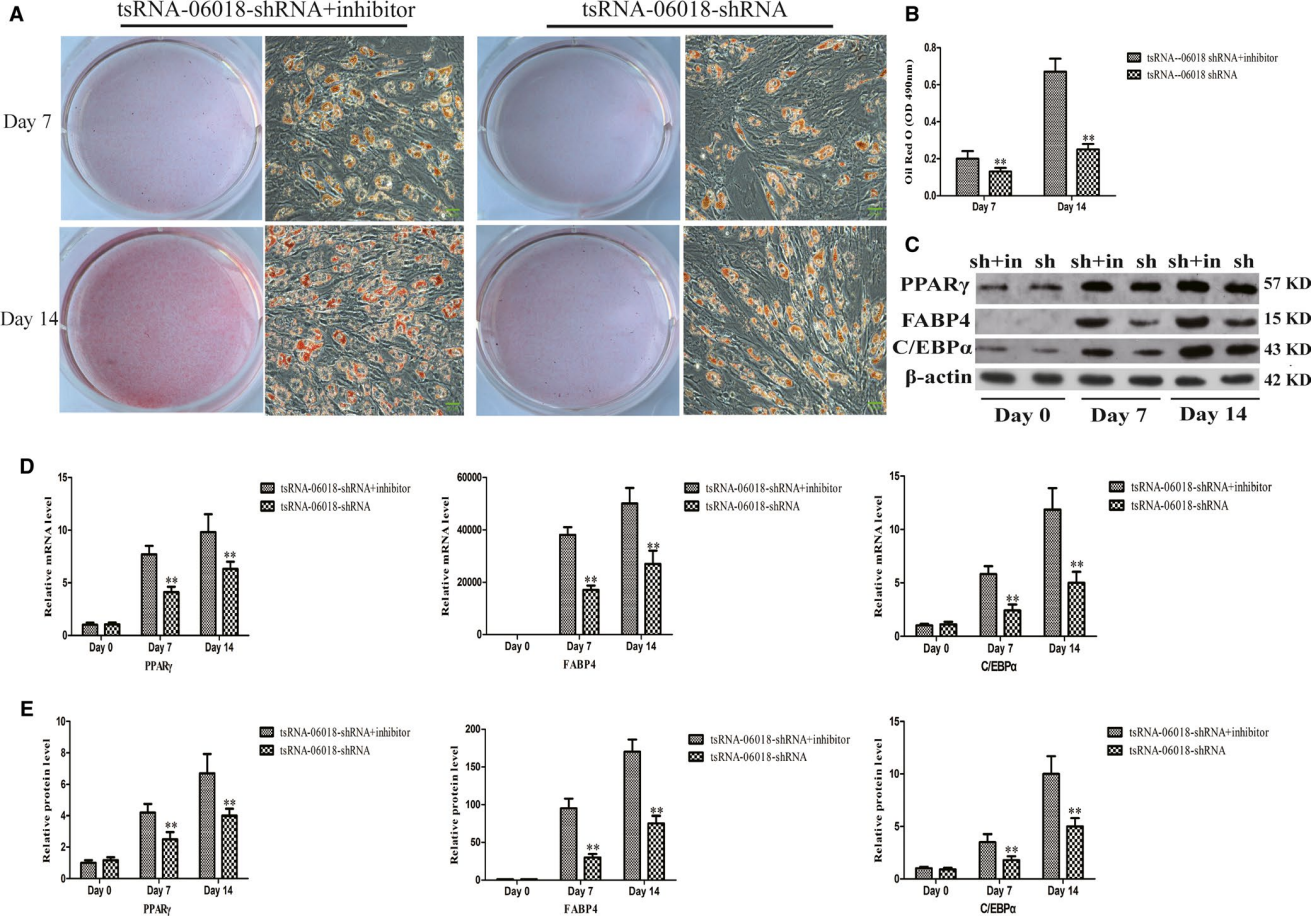
Assessment of the adipogenic differentiation of hMSCs in which tsRNA‐06018 was knocked down in parallel with ERK1/2 signalling inhibitor treatment. Oil red O staining was employed as a means of assessing lipid accumulation. Scale bar, 50μm. A, In response to treatment with an ERK1/2 signalling inhibitor, the adipogenic differentiation of hMSCs in which tsRNA‐06018 was also knocked down was associated with increased lipid accumulation relative to that which occurred in cells in which tsRNA‐06018 was knocked down but no inhibitor was added. B, Differences in Oil red O staining intensity were significant when comparing the shRNA + inhibitor and shRNA groups. D, qRT‐PCR was used to compare PPARγ, FABP4 and CEBP/α expression in the different groups. C, E, PPARγ, FABP4 and C/EBPα protein levels were assessed via Western blotting. Data are expressed as means ± SD(X ± SD, *n* = 3). **P < 0.01vs. shRNA + inhibitor, respectively. Note: sh: tsRNA‐06018 shRNA;sh + in: tsRNA‐06018‐shRNA + inhibitor

We additionally treated cells with U0126 (Sigma‐Aldrich) in order to reduce p‐ERK1/2 levels in these cells (Figure 7C), revealing that hMSC adipogenic differentiation in cells in which tsRNA‐06018 had been knocked down was stronger as compared with that in cells in which tsRNA‐06018 had been knocked down in the absence of inhibitor addition (Figure 8). These results thus further revealed that the ERK1/2 signalling pathway is downstream of tsRNA‐06018.

## 4 DISCUSSION

Recent research has clearly demonstrated diverse roles for many distinct miRNAs, lncRNA and circRNAs in adipogenesis.^32^, ^33^, ^34^ Further studies have revealed that there are many other kinds of non‐coding RNAs, including the recently discovered tsRNAs which are important for many biological processes.^21^, ^22^, ^23^, ^24^, ^25^ The function for tsRNAs in adipogenic differentiation of hMSCs remains poorly understood.

In the present study, we assessed tsRNA expression profiles during hMSC adipogenic differentiation. We found that tsRNAs present in these cells were largely between 17‐23 and 30‐36 nucleotides long, confirming that these segments were not merely random degradation products (Figure 1A). We found that the 5′‐tRF halves and tRFs‐5 present in these samples had similar positions relative to their parental tRNA sequences, and the same was true for 3′‐tRF halves and tRFs‐3 (Figure 1B), also confirming that these segments are generated by specific cleavage. These findings thus further improved our understanding of tsRNA biology and were in line with past work.^21^, ^22^, ^35^

We were able to identify 6 types of tsRNAs in our samples, with the proportion and reads of these tsRNAs largely being increased on day 7 of adipogenesis and decreased on days 14 and 21 (Figure 1C‐D). This suggested the possibility that these tsRNAs may play key roles in hMSC adipogenesis. Importantly, peak tsRNA expression was evident on day 7 of adipogenesis, leading us to focus on this time point, which has also been previously shown to be a critical period that regulates the fate of differentiating pre‐adipocytic cells.^36^, ^37^

We therefore selected the differentially expressed tsRNAs on days 7 and 14 to analyse their expression trends during adipogenic differentiation (Figure 2A,B), revealing trends consistent with the proportion and reads of these tsRNAs. Interestingly, on day 7 of adipogenesis, we observed 6 differentially expressed tsRNAs that had no expression on day 0 and yet had peak expression on day 7, suggesting that these key tsRNAs may be important drivers of adipogenesis. We were interested in exploring whether tsRNAs were able to impact the adipogenic process. In 6 differentially expressed tsRNAs, we found that tsRNA‐06018 knockdown inhibited hMSC adipogenesis (Figure 4). Interestingly, when we overexpressed tsRNA‐06018, this led to significant hMSC death (Figure S2). This suggests that tsRNA‐06018 is a key regulator of hMSC growth and proliferation. As hMSCs must be grown to confluence over 2 days prior to undergoing adipogenic differentiation, we were unable to assess the adipogenic differentiation of cells overexpressing tsRNA‐06018. Then, the remaining differentially expressed tsRNA did not imply that these tsRNAs were not involved in the process of adipogenic differentiation, possibly due to compensatory biological mechanisms.^38^, ^39^, ^40^, ^41^ However, the novel tsRNA‐06018 clearly impacted hMSC adipogenesis.

tsRNAs have been shown to play evolutionarily conserved roles in stress response in eukaryotes.^42^, ^43^, ^44^ These tsRNAs are also important in the context of cancers, neurological disease and metabolic disorders.^21^, ^22^, ^23^, ^24^, ^25^ One study recently suggested that tRF^GluTTC^ can inhibit 3T3‐L1 preadipocytes differentiation into adipocytes.^45^ Interestingly, we instead identified a distinct tsRNA that promoted hMSC adipogenesis, suggesting it acts by a distinct mechanism. We also utilized primary hMSCs rather than the 3T3‐L1 cell line model, suggesting that our cell system may be more physiologically relevant. Other recent work suggests that tsRNAs can modulate stem cell states in mouse embryonic stem cells (mESCs).^46^ This lends further credence to our results. As tsRNA studies represent a relatively new area of research, there are few experiments involving in vivo tsRNA studies.^25^, ^45^, ^46^ Similarly, human bone marrow mesenchymal stem cells were used as research objects in our study, a further verification of the in vivo experiment is also even more difficult. The adipogenic differentiation of human bone marrow mesenchymal stem cells is widely recognized as an ideal model that has been applied in vitro in many studies, and the results of these analyses are still a valuable and significant resource for future reference.^5^, ^6^, ^8^ Although no further in vivo validation experiments were performed in our study, based on our overall experimental results, we believe that this tsRNA is likely to play an important role in the adipogenic differentiation process.

The specific mechanisms whereby tsRNA‐06018 regulates adipogenesis remains to be fully established. Many different mechanisms whereby tsRNAs regulate gene expression have been proposed,^47^, ^48^, ^49^ with one study having shown that tsRNAs had functions similar to those of miRNAs, enabling them to regulate target gene expression.^50^ However, other work suggests that miRNAs and tsRNAs function in distinct manners, with tsRNA targeting sites being present in both 3′‐UTR regions as well as in CDS and 5′UTR regions of target mRNAs.^51^ Using a predictive algorithm, we identified candidate tsRNA‐06018 targets linked with adipogenesis based upon complementarity, identifying STC2 as a candidate target of this tsRNA (Figure 6A).

STC2 is a glycoprotein hormone that controls levels of calcium and phosphates.^52^, ^53^ It is essential for the osteoblastic differentiation of MC3T3‐E1 cells and is also important in the context of diabetes, cardiovascular diseases and fatty liver conditions.^54^, ^55^, ^56^, ^57^ Retinoic acid has been shown to promote STC2, and it is also a key regulator of adipogenesis.^58^, ^59^ This thus suggested a possible link between STC2 and adipogenesis. As shown in Figure 5, we found that overexpressing STC2 impaired hMSC adipogenesis, suggesting that it has a clear biological role in this process. We further identified a negative correlation between tsRNA‐06018 and STC2 expression (Figure 6B). To confirm a direct interaction between these two molecules, we further used luciferase reporter assays to confirm that tsRNA‐06018 directly binds the 3′‐ UTR region of STC2 (Figure 6C).

**FIGURE 9:**
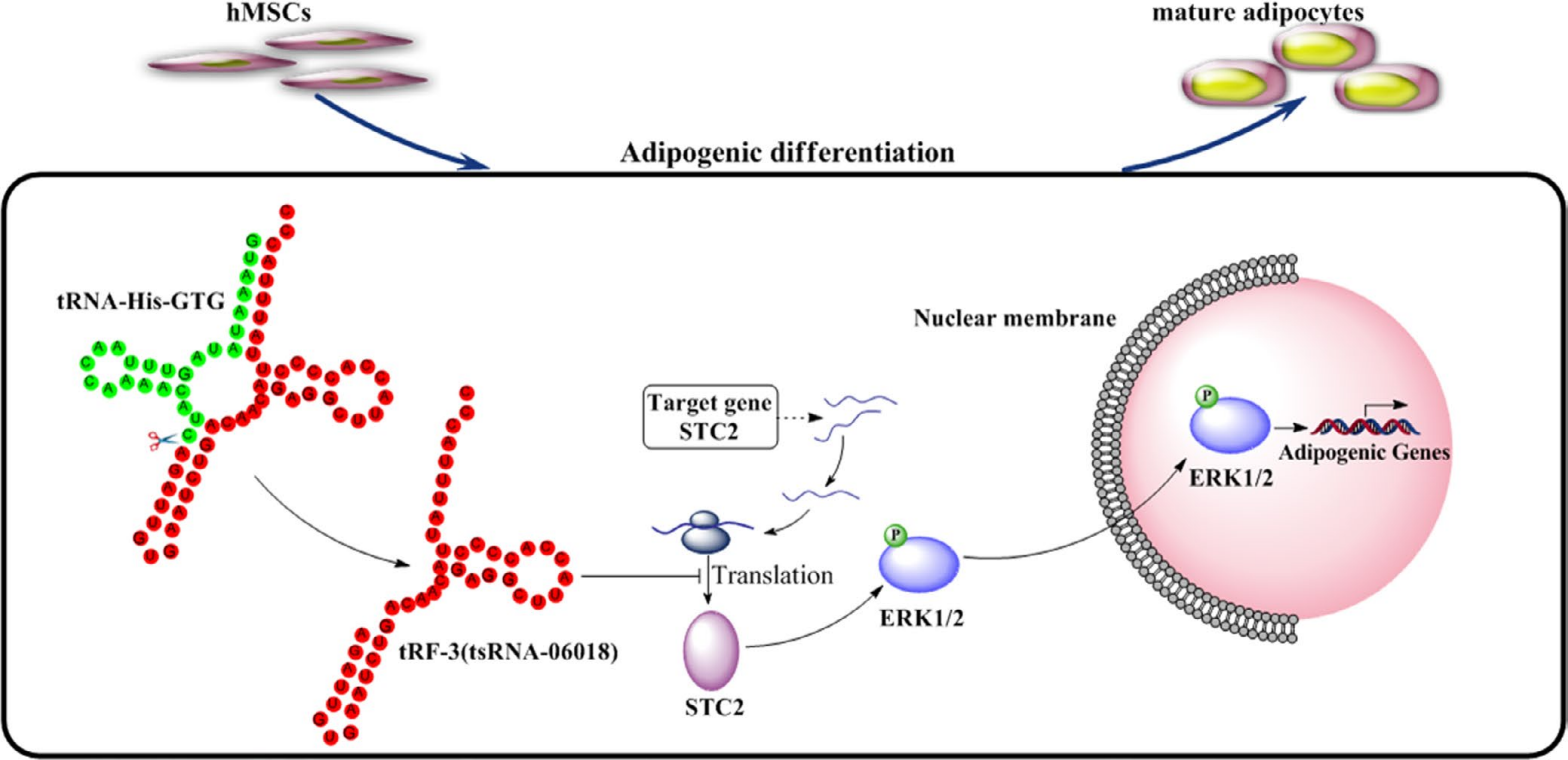
Proposed model whereby tsRNA‐06018 regulates the adipogenic differentiation of hMSCs by targeting STC2 via the ERK1/2 signalling pathway

Many different signalling pathways govern hMSC adipogenic differentiation, and thus it is vital that efforts be taken to accurately identify which pathways function downstream of STC2. Previous studies have shown that STC2 regulates the osteogenic differentiation of MC3T3‐E1 cells via the ERK1/2 signalling pathway,^54^ which is also closely related to adipogenic differentiation.^60^, ^61^ These prior results led us to hypothesize that the ERK1/2 signalling pathway is most likely to function downstream of STC2. As expected, we found that knocking down tsRNA‐06018 and overexpressing STC2 both enhanced ERK1/2 phosphorylation (Figure 7A, B). Furthermore, by adding an ERK1/2 inhibitor in order to reduce p‐ERK1/2 levels (Figure 7C), we were able to demonstrate that the adipogenic differentiation of hMSCs in which tsRNA‐06018 was also knocked down was stronger than that in cells in which tsRNA‐06018 was knocked down but to which no inhibitor was added (Figure 8). These results thus further confirmed that the ERK1/2 signalling pathway is downstream of STC2. Based on these results, we therefore speculate that tsRNA‐06018 was able to regulate adipogenic differentiation by targeting STC2 via the ERK1/2 signalling pathway (Figure 9).

In summary, we used high‐throughput sequencing to identify profiles of tsRNA expression during hMSC adipogenesis, leading us to identify the novel tsRNA‐06018 as a key regulator of this process. Together, these results may suggest new approaches for treating obesity.

## CONFLICT OF INTEREST

The authors declare that they have no conflict of interest.

## AUTHOR CONTRIBUTIONS

**Tao Wang:** Funding acquisition (equal); Methodology (equal); Project administration (lead); Resources (equal); Writing‐original draft (lead); Writing‐review & editing (lead). **Lingling Cao:** Methodology (equal); Software (equal); Validation (equal). **Shan He:** Data curation (equal); Resources (equal). **Kai Long:** Conceptualization (equal); Supervision (equal). **Xinping Wang:** Formal analysis (equal); Software (equal); Visualization (equal). **Hui Yu:** Investigation (equal); Supervision (equal). **Baicheng Ma:** Methodology (equal); Project administration (equal); Writing‐original draft (equal). **Xiaoyuan Xu:** Investigation (equal); Software (equal); Writing‐review & editing (equal). **Weidong Li:** Formal analysis (equal); Project administration (equal); Writing‐original draft (equal); Writing‐review & editing (lead).

## ETHICAL APPROVAL

Not Applicable.

## Supporting information

Figure S1

Figure S2

Table S1

Supplementary Material

## Data Availability

The data sets used and analysed during the current study are available from the corresponding author on reasonable request.
