# Supplementary figures and images for: Small RNA sequencing reveals a novel tsRNA‐06018 playing an important role during adipogenic differentiation of hMSCs
Source: J Cell Mol Med. 2020 Sep 16;24(21):12736–49. doi: 10.1111/jcmm.15858 (PMC7686998; doi:10.1111/jcmm.15858)

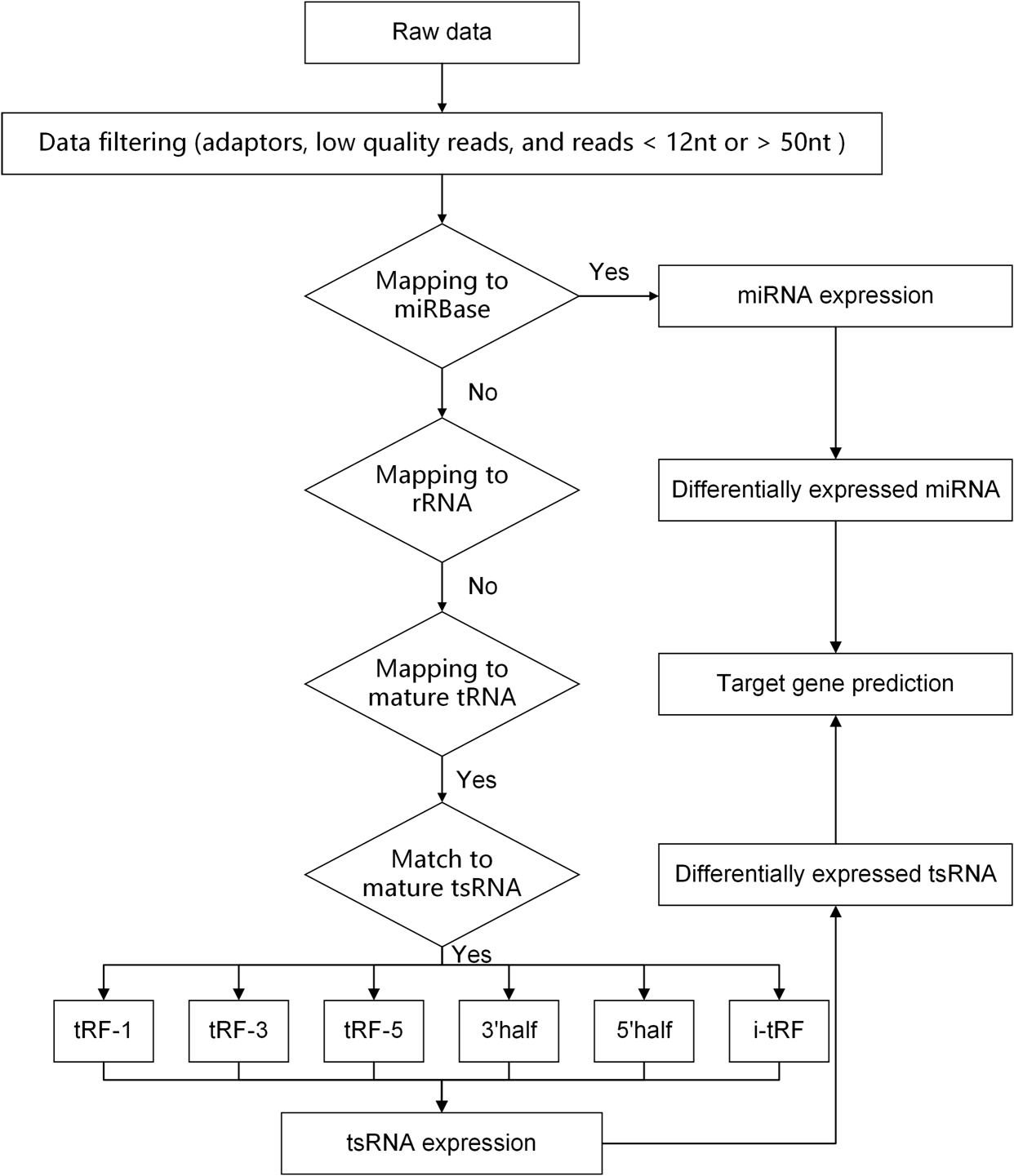

Supplement: Supplementary file 1 — Figure S1 [file JCMM-24-12736-s001.tif]

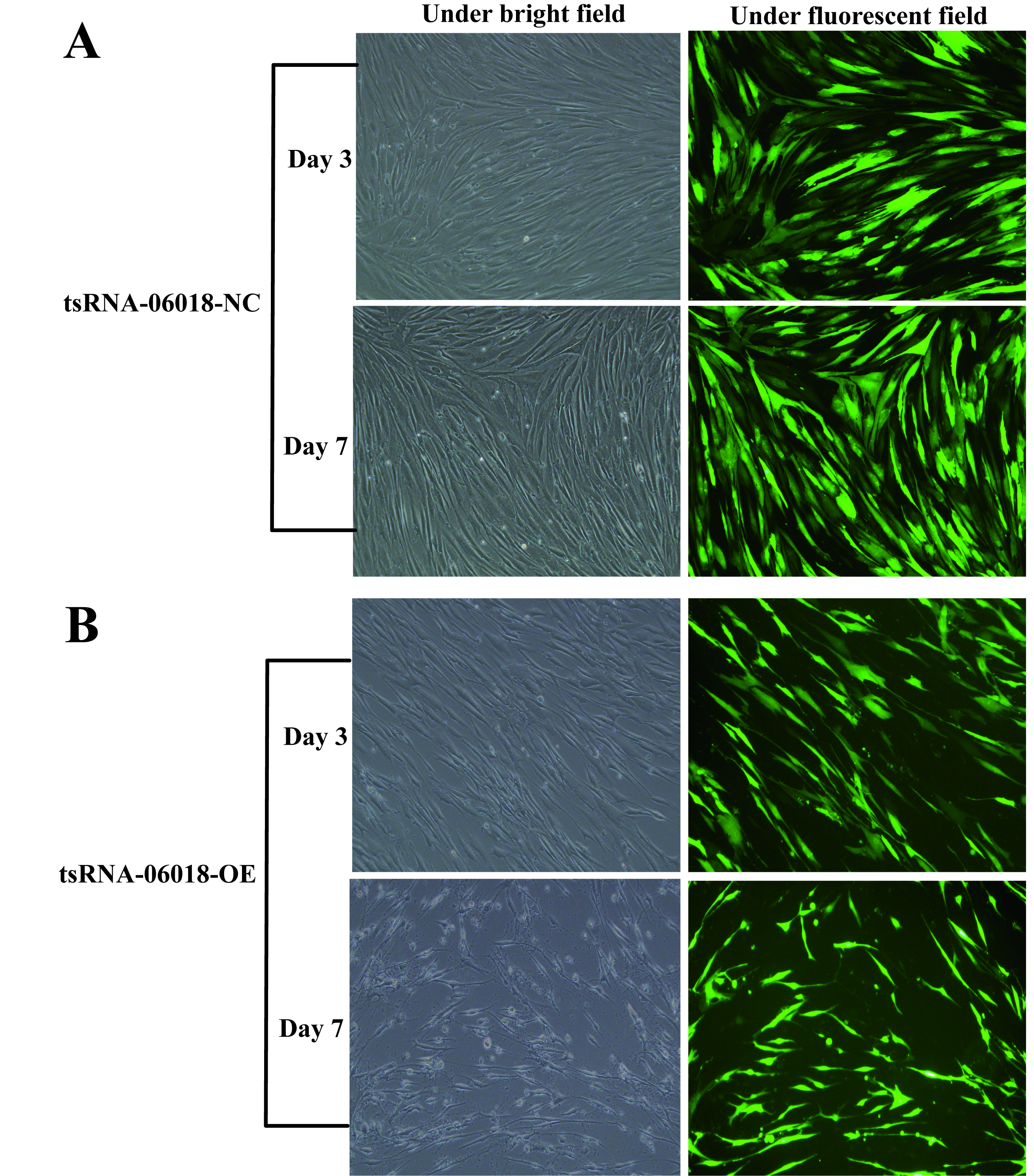

Supplement: Supplementary file 2 — Figure S2 [file JCMM-24-12736-s002.tif]
