# Supplementary material for: Small RNA sequencing reveals a novel tsRNA‐06018 playing an important role during adipogenic differentiation of hMSCs
Source: J Cell Mol Med. 2020 Sep 16;24(21):12736–49. doi: 10.1111/jcmm.15858 (PMC7686998; doi:10.1111/jcmm.15858)
Supplement: Supplementary file 3 — Table S1 [file JCMM-24-12736-s003.docx]

| Gene symbol | Forward primer | Reverse primer | Length (bp) |
| --- | --- | --- | --- |
| *PPARγ* | 5'- GGGATGTCTCATAATGCCATCAG- 3' | 5'-GCCCTCGCCTTTGCTTTG-3' | 97 |
| *CEBP/α* | 5'-CCAAGAAGTCGGTGGACAAGAAC-3' | 5'-CACCTTCTGCTGCGTCTCCA-3' | 122 |
| *FABP4* | 5-'GGATGATAAACTGGTGGTGGAATG-3' | 5'- CAGAATGTTGTAGAGTTCAATGCGA -3' | 123 |
| *STC2* | 5-'TGTGGCGTGTTTGAATGTTT-3 | 5-'CACAGGTCGTGCTTGAGGTA-3 | 245 |
| *β-actin* | 5'-GCGAGAAGATGACCCAGATCATGT-3' | 5'-TACCCCTCGTAGATGGGCACA-3' | 160 |

Table S1
