# Supplementary material for: Small RNA sequencing reveals a novel tsRNA‐06018 playing an important role during adipogenic differentiation of hMSCs
Source: J Cell Mol Med. 2020 Sep 16;24(21):12736–49. doi: 10.1111/jcmm.15858 (PMC7686998; doi:10.1111/jcmm.15858)
Supplement: Supplementary file 4 — Supplementary Material [file JCMM-24-12736-s004.pdf]

smallRNAName	smallRNASequence	AD D14	AD D21	AD D7	CY1（AD D0
tsrna-26579	AACCGAGCGTCCAAGCTCTTTCCATTTT	300	73	180	383
tsrna-26578	TGCGGTACCACTTTT	2849	898	3249	1462
tsrna-26577	GTGGGTGGCTTTTTT	78	111	68	21
tsrna-26576	TCGAGAGGGGCTGTGCTCGCAAGGTTTCTTT	17302	16412	11300	14380
tsrna-26575	AGGTGAAAGTTCCTTT	44	437	8	16
tsrna-26574	ACCTCAGAAGGTCTCACTTT	1275	507	766	854
tsrna-26572	GATATCCAACCTTCGGCTATAGGGTGGAGACTTTTT	813	430	629	561
tsrna-26571	AGGGAGGTTATGATTAACTTTT	456	777	293	270
tsrna-26570	GTGGGGTGCCTCACAGCTTCGCTGCGTGAGCATTTT	55	84	46	26
tsrna-26569	AAGAGGAGTTGTTTT	443	856	250	230
tsrna-26568	ACAAGTGCGGTTTTTT	686	342	299	270
tsrna-26567	GGAATGTCAGCTTTT	290	325	174	105
tsrna-26566	ATAGGTATTAAGGTTTT	54	7	21	34
tsrna-26565	GAGGCTTAAACTTTT	699	1534	480	447
tsrna-26564	ATGGCCGCATATATTT	42	0	19	25
tsrna-26563	TTCCGTGGGTTTGTTTT	3295	2497	2081	2030
tsrna-26562	GAGGGTTCTCACCTTCTCTCTCCGATTT	153	575	48	184
tsrna-26561	GCAACTGGTCGTTTT	3222	3249	1851	1551
tsrna-26560	GCGGGCGGACCTTTT	190	500	99	77
tsrna-26559	GAGAGCGCTCGGTTTTT	263	572	165	37
tsrna-26558	TGTGCTCCGGAGTTACCTCGTTT	2307	1241	2548	2467
tsrna-26557	GGCGATCACGTAGATTTT	1610	1503	861	930
tsrna-26556	GCACGAAAATGTGTTTT	0	0	0	0
tsrna-26555	GGTGTGGTCTGTTGTTT	0	0	1	0
tsrna-26554	TAGGGTGTGCGTGTTTTT	62	94	60	28
tsrna-26553	TTCAAAGGTGAACGTTT	125	246	50	227
tsrna-26552	GTGTAAGCAGGGTCGTTTT	261	176	259	398
tsrna-26551	ATGTGGTGGCTTACTTT	7240	2352	7221	4248
tsrna-26550	GTGTGTAGCTGCACTTTT	75	65	53	48
tsrna-26548	GAAGCGGGTGCTCTTATTTT	235868	48629	2E+05	84654
tsrna-26547	TCGCTGGTTCGAATCCGGCTCGGAGGACCA	0	51	1	0
tsrna-26544	GTAGTCGTGGCCGA	1	1	0	1
tsrna-26543	GGTTCCATGGTGTA	0	1	0	1
tsrna-26542	GGTTCCATAGTGTA	0	2	1	0
tsrna-26540	GGGGGTATAGCTCAG	1	0	0	0
tsrna-26539	GGGGGTATAGCTC	0	2	0	1
tsrna-26534	GCCCGGCTAGCTCAG	0	0	0	0
tsrna-26532	CCTTCGATAGCTCAG	0	1	0	0
tsrna-26531	GAATTCTCGCCTGCCACGCGGGAGGCCCGG	1	7	0	0
tsrna-26530	GAATTCTCGCCTGCCACGCGGGAGGCCC	2	9	3	1
tsrna-26529	GAATTCTCGCCTGCCACGCGGGAGGCC	1	9	1	1
tsrna-26528	GAATTCTCGCCTGCCACGCGGG	1	8	1	0
tsrna-26527	GAATTCTCGCCTGCCACGCGG	2	8	0	1
tsrna-26526	GAATTCTCGCCTGCCACGCG	3	5	0	0
tsrna-26525	GAATTCTCGCCTGCCACGC	4	12	1	0
tsrna-26524	GAATTCTCGCCTGCCACG	0	7	0	0
tsrna-26523	GAATTCTCGCCTGCCAC	0	0	0	0
tsrna-26522	GAATTCTCGCCTGCCA	0	1	0	0
tsrna-26517	GAATGATTTCGACTCATTAAATTATGATA	8	4	9	0
tsrna-26516	GAATGATTTCGACTCATTAAA	0	1	0	0
tsrna-26515	GAATGATTTCGACTCA	1	1	0	0
tsrna-26514	GAATCTTAGCTTTGGGTGCTAATGGTGGAGTTAAAG	2	1	1	1
tsrna-26513	GAATCTTAGCTTTGGGTGCTAATGGTGGAG	1	1	0	0
tsrna-26508	GAATCTTAGCTTTGGGTGC	0	0	0	1
tsrna-26497	GAATCTCGGTGGGACCTCCA	51	86	25	12
tsrna-26496	GAATCCTGTTCGTGACGCCA	6	0	3	1
tsrna-26494	GAATCCTGCTCACAGCGCCA	5	17	2	1
tsrna-26493	GAATCCTGCTCACAGCGCC	2	4	0	0
tsrna-26491	GAATCCTGCCGACTACGCCA	110	228	38	17
tsrna-26490	GAATCCTGCCGACTACGCC	3	14	2	0
tsrna-26489	GAATCCTGCCGACTACGC	1	1	0	0
tsrna-26488	GAATCCTGCCGACTACG	1	0	0	0
tsrna-26487	GAATCCGGCTCGGAGGACCA	1	2	0	0

tsrna-26483	GAATCCGGCTCGAAGGACCA	15	45	10	2
tsrna-26482	GAATCCGGCTCGAAGGACC	0	3	2	0
tsrna-26473	GAATCCCATCCTCGTCGCCA	4	1	3	0
tsrna-26472	GAATCCCATCCTCGTCGCC	0	1	0	1
tsrna-26471	GAATCCCATCCTCGTCGC	0	0	0	0
tsrna-26469	GAATCCCAGTAGAGCCTCCA	1	7	4	9
tsrna-26468	GAATCCCAGCGGTGCCTCCA	0	2	1	0
tsrna-26464	GAATCCCAGCGAGGCCTCCA	1	0	1	1
tsrna-26463	GAATCCCACTTCTGACACCA	5	5	3	1
tsrna-26462	GAATCCCACTTCTGACACC	0	4	0	0
tsrna-26461	GAATCCCACTTCTGACAC	2	0	0	0
tsrna-26460	GAATCCCACTTCTGACA	0	0	0	0
tsrna-26459	GAATCCCACTCCTGACACCA	72	62	48	27
tsrna-26458	GAATCCCACTCCTGACACC	1	0	1	0
tsrna-26457	GAATCCCACTCCTGACAC	1	0	0	0
tsrna-26455	GAATCCCACCTTCGTCGCCA	3	2	0	0
tsrna-26454	GAATCCCACCGCTGCCACCA	1	0	0	0
tsrna-26453	GAATCCCACCGCTGCCACC	0	0	1	0
tsrna-26450	GAATCCCACCCTCGTCGCCA	7	15	1	1
tsrna-26448	GAATCCCACCAGAGTCGCCA	1	7	0	1
tsrna-26438	GAATCACGTCGGGGTCACCA	5	6	6	5
tsrna-26437	GAATCACGTCGGGGTCACC	0	0	0	0
tsrna-26433	GAATAGTTTAAATTAGAATCTTAGCTTTGG	10	5	8	3
tsrna-26431	GAAGGTTGCGTGTTCAAGTCACGTCGGGGT	0	1	0	0
tsrna-26430	GAAGGTTGCGTGTTCAAGTCACGTC	0	1	1	0
tsrna-26429	GAAGGTTGCGTGTTCAAGTCACGT	0	1	0	0
tsrna-26428	GAAGGTTGCGTGTTCAAATCACGTCGGGGT	0	1	1	0
tsrna-26427	GAAGGTTGCGTGTTCAAATCACGTC	1	1	0	1
tsrna-26426	GAAGGTCGTGAGTTCGATCC	0	0	1	0
tsrna-26423	GAAGGTCCTGGGTTCGAGCCCCAGTGGAACCACCA	35	31	30	14
tsrna-26422	GAAGGTCCTGGGTTCGAGCCCCAGTGGAAC	2	0	1	0
tsrna-26421	GAAGGTCCTGGGTTCGAGCCCCAGTGGAA	0	2	1	0
tsrna-26419	GAAGGTCCTGGGTTCGAGCCCCA	1	0	0	0
tsrna-26418	GAAGGTCCTGGGTTCGAGCCCC	1	1	0	0
tsrna-26415	GAAGGTCCTGAGTTCGAACC	0	0	0	0
tsrna-26414	GAAGGTCCTGAGTTCGAA	0	0	0	0
tsrna-26413	GAAGGTCCTGAGTTCG	0	0	0	0
tsrna-26412	GAAGGCTGCGTGTTCGAATCACGTCGGGGT	0	0	0	0
tsrna-26410	GAAGCGTGCTGGGCCCATAACC	0	0	0	0
tsrna-26408	GAAGCCAGTTGATTAGGGTGCTTAGCTGTT	3	0	3	2
tsrna-26407	GAAGCCAGTTGATTAGGGTGCTTAGCTGT	1	0	3	1
tsrna-26402	GAAGCATTGGACTGTAAATCTAAAGACAGG	2	2	0	0
tsrna-26401	GAAGCATTGGACTGTAAATCTAAAGACA	1	0	1	0
tsrna-26400	GAAGCATTGGACTGTAAATCTAAAGA	0	0	1	0
tsrna-26398	GAAGCATTGGACTGTAAATCTAAA	0	0	0	0
tsrna-26392	GAAGCATTGGACTGTAAA	0	0	1	0
tsrna-26391	GAAGCATTGGACTGTAA	0	0	0	0
tsrna-26390	GAAGCATTGGACTGTA	0	0	0	0
tsrna-26389	GAAGCAGCTTCAAACCTGCCGGGGCTTCC	1	2	0	0
tsrna-26388	GAAGCAGCTTCAAACCTGCCGGGGCTT	0	0	0	0
tsrna-26387	GAAGCAGCTTCAAACCTGCCGGGGCT	0	0	0	0
tsrna-26382	GAAGATTGCAGGTTCGAGTCCTGCCGCGGTCGCCA	2	5	3	0
tsrna-26380	GAAGATTGAGGGTTCGAGTCCCTTCGTGGTCGCCA	5	17	2	1
tsrna-26379	GAAGATTGAGGGTTCGAGTCCCTTCGTGGTCGCC	2	4	3	1
tsrna-26378	GAAGATTGAGGGTTCGAGTCCCTTCGTGGT	1	3	1	2
tsrna-26377	GAAGATTCTAGGTTCGACTCCTGGCTGGCT	0	9	0	1
tsrna-26376	GAAGATTCTAGGTTCGACTCCTG	0	7	0	0
tsrna-26375	GAAGATTCCAGGTTCGACTCCTGGC	0	0	1	1
tsrna-26374	GAAGATTCCAGGTTCGACTCC	0	1	0	0
tsrna-26372	GAAGATCGCGGGTTCGAACCCCGTC	0	0	0	0
tsrna-26370	GAAGATCGCGGGTTCGAA	0	1	0	0
tsrna-26369	GAAGATCGCGGGTTCGA	0	1	0	0
tsrna-26368	GAAGATCGCGGGTTCG	0	1	0	0
tsrna-26367	GAAGAAGCAGCTTCAAACCTGCCGGGGCTTCC	0	4	2	0

tsrna-26366	GAAGAAGCAGCTTCAAACCTGCCGGGGCTT	0	2	1	0
tsrna-26365	GAAGAAGCAGCTTCAAACCTGCCGGGGCT	0	0	0	0
tsrna-26364	GAAGAAGCAGCTTCAAACCTGCCGGGGC	0	1	0	0
tsrna-26363	GAAGAAGCAGCTTCAAACCTGCCGGGG	0	2	0	0
tsrna-26358	GAAGAAGCAGCTTCAAACCTG	1	0	0	0
tsrna-26355	GAACTGCTAACTCATGCCCCCATGTCTAACAACATGGCTTTCT	8	3	16	0
tsrna-26354	GAACTGCTAACTCATGCCCCCATGTCTAACAACATGGCTTTCT	4	2	4	0
tsrna-26353	GAACTGCTAACTCATGCCCCCATGTCTAACAACATGGCT	0	0	0	0
tsrna-26352	GAACTGCTAACTCATGCCCCCATGTCTAACAACATGGC	1	0	2	0
tsrna-26351	GAACTGCTAACTCATGCCCCCATGTCTAACAACA	0	0	0	0
tsrna-26350	GAACTGCTAACTCATGCCCCCATGTCTAACA	0	0	0	0
tsrna-26349	GAACTGCTAACTCATGCCCCCATGTCTAAC	0	0	1	0
tsrna-26348	GAACTGCTAACTCATGCCCCCATGTCTAA	0	0	0	0
tsrna-26347	GAACTGCTAACTCATGCCCCCATGTCT	1	1	0	0
tsrna-26346	GAACTGCTAACTCATGCCCCCATGTC	0	0	0	0
tsrna-26345	GAACTGCTAACTCATGCCCCCATGT	0	0	0	0
tsrna-26344	GAACTGCTAACTCATGCCCCCATG	2	0	0	0
tsrna-26343	GAACTGCTAACTCATGCCCCCAT	0	1	0	0
tsrna-26342	GAACTGCTAACTCATGCCCCCA	0	0	1	0
tsrna-26335	GAACCTCAGAGGGGGCACCA	0	3	0	0
tsrna-26333	GAACCCTGCTCGCTGCGCCA	8	38	1	3
tsrna-26332	GAACCCTGCTCGCTGCGC	0	1	0	0
tsrna-26330	GAACCCCGTCCGTGCCTCCA	6	8	3	3
tsrna-26328	GAACCCCGTACGGGCCACCA	0	2	0	1
tsrna-26315	GAAATTTAGGTTAAATACAGACCAAGAGCC	0	1	1	0
tsrna-26313	GAAATCCAATGGGGTTTCCCC	0	1	0	0
tsrna-26312	GAAATATGTCTGATAAAAGAGTTACTTTG	0	2	0	0
tsrna-26311	GAAATATGTCTGATAAAAGAGTTACTTT	0	1	0	1
tsrna-26306	GAAATATGTCTGATAAAA	1	0	0	0
tsrna-26304	GAAATATGTCTGATAA	0	1	0	0
tsrna-26299	GAAATACAACGATGGTTTTTCATATCATTGGTCGTGGTTGTAG	1	5	4	1
tsrna-26298	GAAATACAACGATGGTTTTTCATATCATTGGTCGTGGTTGTAG	1	1	5	1
tsrna-26297	GAAATACAACGATGGTTTTTCATATCATTGGTCGTGGTTGTAG	1	1	5	1
tsrna-26296	GAAATACAACGATGGTTTTTCATATCATTGGTCGTGGTTGTAG	1	0	2	1
tsrna-26295	GAAATACAACGATGGTTTTTCATATCATTGGTCGTGGTTGTA	5	4	1	1
tsrna-26294	GAAATACAACGATGGTTTTTCATATCATTG	1	0	0	0
tsrna-26293	GAAATACAACGATGGTTTTTCATATCA	0	0	0	1
tsrna-26289	GAAATACAACGATGGTTTTT	0	0	1	0
tsrna-26285	GAAAGGTTGGTGGTTCG	0	0	0	0
tsrna-26283	GAAAGGTCCCCGGTTCGAAACCGGGCGGA	0	0	0	0
tsrna-26282	GAAAGGTCCCCGGTTCGAAACCGGGC	0	1	0	0
tsrna-26281	GAAAGGTCCCCGGTTCGAAAC	0	3	0	0
tsrna-26280	GAAAGGTCCCCGGTTCG	0	2	0	0
tsrna-26279	GAAAGGTCCCCGGTTC	0	1	0	0
tsrna-26278	GAAAGCTCACAAGAACTGCTAACTCATGCCCCCATG	6	11	0	1
tsrna-26277	GAAAGCTCACAAGAACTGCTAACTCATGCC	5	5	2	1
tsrna-26276	GAAAGCTCACAAGAACTGCTAACTCATGC	6	7	3	9
tsrna-26275	GAAAGCTCACAAGAACTGCTAACTCATG	6	4	3	2
tsrna-26274	GAAAGCTCACAAGAACTGCTAACTCA	5	7	7	5
tsrna-26273	GAAAGCTCACAAGAACTGCTAACTC	4	9	2	1
tsrna-26272	GAAAGCTCACAAGAACTGCTAACT	4	6	0	4
tsrna-26271	GAAAGCTCACAAGAACTGCTAAC	1	6	2	0
tsrna-26270	GAAAGCTCACAAGAACTGCTAA	1	3	0	0
tsrna-26269	GAAAGCTCACAAGAACTGCTA	0	1	0	0
tsrna-26268	GAAAGCTCACAAGAACTGCT	0	2	0	0
tsrna-26266	GAAAGCTCACAAGAACTG	0	0	0	1
tsrna-26265	GAAAGCTCACAAGAACT	0	0	1	0
tsrna-26263	GAAACCGGGCGGAAACACCA	272	700	104	31
tsrna-26262	GAAACCGGGCGGAAACACC	70	195	21	3
tsrna-26261	GAAACCGGGCAGAAGCACCA	30	130	14	6
tsrna-26260	GAAACCATCCTCTGCTACCA	0	3	2	1
tsrna-26258	GAAACCAGCTTTGGGGGGTTCG	2	1	1	0
tsrna-26257	GAAACCAGCTTTGGGGGGTTC	0	1	0	0
tsrna-26256	GAAAATGTTTAGACGGGCTCACATCACCCCATAAACACCA	166	44	95	24

tsrna-26255	GAAAATGTTTAGACGGGCTCACATCACCCCATAAACACC	89	70	55	14
tsrna-26254	GAAAATGTTTAGACGGGCTCACATCACCCCATAAA	95	47	33	15
tsrna-26253	GAAAATGTTTAGACGGGCTCACATCACCCCATAA	80	49	44	13
tsrna-26252	GAAAATGTTTAGACGGGCTCACATCACCCCATA	104	42	30	12
tsrna-26251	GAAAATGTTTAGACGGGCTCACATCACCCCA	73	35	29	8
tsrna-26250	GAAAATGTTTAGACGGGCTCACATCACCCC	82	42	32	8
tsrna-26249	GAAAATGTTTAGACGGGCTCACATCACCC	69	42	28	7
tsrna-26248	GAAAATGTTTAGACGGGCTCACATCACC	66	49	23	5
tsrna-26247	GAAAATGTTTAGACGGGCTCACATCA	55	37	21	3
tsrna-26246	GAAAATGTTTAGACGGGCTCACATC	40	32	15	3
tsrna-26245	GAAAATGTTTAGACGGGCTCACAT	45	26	24	5
tsrna-26244	GAAAATGTTTAGACGGGCTCACA	27	22	14	2
tsrna-26243	GAAAATGTTTAGACGGGCTCAC	27	22	13	3
tsrna-26242	GAAAATGTTTAGACGGGCTCA	14	14	2	2
tsrna-26241	GAAAATGTTTAGACGGGCTC	4	15	1	2
tsrna-26240	GAAAATGTTTAGACGGGCT	0	1	0	0
tsrna-26239	GAAAATGTTTAGACGGGC	1	0	0	0
tsrna-26236	GAAAATGTTGGTTATACCCTTCCCGTACTACCA	11	20	11	3
tsrna-26235	GAAAATGTTGGTTATACCCTTCCCGTACTA	1	0	1	0
tsrna-26234	GAAAATGTTGGTTATACCCTTCCCGTACT	0	0	0	0
tsrna-26233	GAAAATGTTGGTTATACCCTTCCCGTA	0	0	0	0
tsrna-26226	GAAAATGTTGGTTATACCC	0	0	0	0
tsrna-26223	GAAAACCTTTTTCCAAGGACACCA	1	2	1	0
tsrna-26222	GAAAACCTTTTTCCAAGGACACC	0	0	0	0
tsrna-26221	GAAAACCTTTTTCCAAGGACA	0	0	0	0
tsrna-26217	GAAAAAGTCATGGAGGCCATGGGGTTGGCTTGAAAC	91	351	80	32
tsrna-26216	GAAAAAGTCATGGAGGCCATGGGGTTGGCT	101	371	58	25
tsrna-26215	GAAAAAGTCATGGAGGCCATGGGGTTGGC	90	369	69	29
tsrna-26214	GAAAAAGTCATGGAGGCCATGGGGTTGG	96	370	70	40
tsrna-26213	GAAAAAGTCATGGAGGCCATGGGGTTG	64	355	61	32
tsrna-26212	GAAAAAGTCATGGAGGCCATGGGGTT	65	314	78	23
tsrna-26211	GAAAAAGTCATGGAGGCCATGGGGT	38	113	5	8
tsrna-26210	GAAAAAGTCATGGAGGCCATGGGG	22	90	19	12
tsrna-26209	GAAAAAGTCATGGAGGCCATGGG	15	74	17	9
tsrna-26208	GAAAAAGTCATGGAGGCCATGG	23	84	6	13
tsrna-26207	GAAAAAGTCATGGAGGCCATG	21	89	7	5
tsrna-26206	GAAAAAGTCATGGAGGCCAT	21	74	17	8
tsrna-26205	GAAAAAGTCATGGAGGCCA	22	71	13	5
tsrna-26204	GAAAAAGTCATGGAGGCC	20	64	12	11
tsrna-26203	GAAAAAGTCATGGAGGC	9	26	10	5
tsrna-26202	GAAAAAGTCATGGAGG	9	13	5	3
tsrna-26201	GAAAAACCATTTCATAACTTTGTCAAAGTT	10	4	6	2
tsrna-26198	CTTTTTCCAAGGACACCA	6	4	4	0
tsrna-26197	CTTTTTCCAAGGACACC	0	0	0	0
tsrna-26195	CTTTTCATCTGAGGGTCCAG	0	1	0	0
tsrna-26193	CTTTTAATCTGAGGGTCCGG	9	31	3	0
tsrna-26192	CTTTTAATCTGAGGGTCCAGGGTTCAAGTCCCTGTTCGGGCG	34	76	15	6
tsrna-26191	CTTTTAATCTGAGGGTCCAGGGT	8	45	11	3
tsrna-26190	CTTTTAATCTGAGGGTCCAGGG	5	19	7	1
tsrna-26189	CTTTTAATCTGAGGGTCCAGG	9	24	6	2
tsrna-26188	CTTTTAATCTGAGGGTCCAG	6	40	6	4
tsrna-26187	CTTTTAATCTGAGGGTCCA	5	23	6	4
tsrna-26186	CTTTTAATCTGAGGGTCC	6	22	2	1
tsrna-26185	CTTTTAAGTTAAAGATTAAGAGAACCAACACCTCTTTACAGTGA	10	0	20	3
tsrna-26184	CTTTTAAGTTAAAGATTAAGAGAACCAACACCTCTTTACAGTGA	8	2	3	0
tsrna-26183	CTTTTAAGTTAAAGATTAAGAGAACCAACACCTCTTTACAGTGA	9	0	2	0
tsrna-26182	CTTTTAAGTTAAAGATTAAGAGAACCAACACC	0	1	2	0
tsrna-26181	CTTTTAAGTTAAAGATTAAGAGAACCAACAC	1	1	0	0
tsrna-26180	CTTTTAAGTTAAAGATTAAGAGAACCAAC	0	0	0	0
tsrna-26179	CTTTTAAGTTAAAGATTAAGAGAACCA	0	0	0	0
tsrna-26178	CTTTTAAGTTAAAGATTAAGAGAACC	0	0	0	0
tsrna-26177	CTTTTAAGTTAAAGATTAAGAGAAC	0	0	1	0
tsrna-26174	CTTTTAAGTTAAAGATTAAGA	0	0	0	0
tsrna-26172	CTTTTAAAGGATAACAGCTATCCATTGGTC	0	0	0	0

tsrna-26169	CTTTTAAAGGATAACAGCTAT	0	0	0	1
tsrna-26165	CTTTGTCAAAGTTAAATTATAGGCTAA	5	2	1	0
tsrna-26164	CTTTGTCAAAGTTAAATTATAGGCT	1	0	2	0
tsrna-26163	CTTTGTCAAAGTTAAATTATAGGC	1	0	1	1
tsrna-26162	CTTTGTCAAAGTTAAATTATAGG	0	0	2	0
tsrna-26159	CTTTGGGTGCTAATGGTGGAGTTAAAGACTTTTTCTCTGACCA	24	61	22	16
tsrna-26158	CTTTGGGTGCTAATGGTGGAGTTAAAGACTTTTTCTCTGACC	9	26	9	5
tsrna-26157	CTTTGGGTGCTAATGGTGGAGTTAAAGACTTTTTCT	5	12	4	1
tsrna-26156	CTTTGGGTGCTAATGGTGGAGTTAAAGACT	0	8	3	0
tsrna-26155	CTTTGGGTGCTAATGGTGGAGTTAAAGAC	0	3	1	1
tsrna-26154	CTTTGGGTGCTAATGGTGGAGTTAAAGA	1	3	1	1
tsrna-26153	CTTTGGGTGCTAATGGTGGAGTTAAAG	5	1	1	0
tsrna-26152	CTTTGGGTGCTAATGGTGGAGTTAAA	0	5	1	0
tsrna-26151	CTTTGGGTGCTAATGGTGGAGTTAA	0	3	1	0
tsrna-26150	CTTTGGGTGCTAATGGTGGAGTTA	0	0	2	0
tsrna-26149	CTTTGGGTGCTAATGGTGGAGTT	0	1	0	0
tsrna-26141	CTTTGGGTGCGAGAGGTCCCGGGT	8	51	3	9
tsrna-26140	CTTTGGGTGCGAGAGGTCCCGG	0	2	0	0
tsrna-26139	CTTTGGGTGCGAGAGGTCCCG	1	2	0	0
tsrna-26138	CTTTGGGGGGTTCGATTCCTTCCTTTTTTG	0	2	0	0
tsrna-26137	CTTTGGGGGGTTCGATTCCTTCC	1	0	0	0
tsrna-26134	CTTTGCACGTATGAGGCCCCGGGT	13	22	3	12
tsrna-26133	CTTTGCACGTATGAGGCCCCGG	1	1	0	0
tsrna-26132	CTTTGCACGTATGAGGCCCCG	0	3	0	0
tsrna-26131	CTTTGCACGTATGAGGCCCC	0	0	1	0
tsrna-26130	CTTTGCACGTATGAGGCCC	0	0	0	0
tsrna-26129	CTTTGCACGCGTGGGTTCGAA	0	3	1	0
tsrna-26128	CTTTGCACGCGTGGGTTCG	0	6	2	0
tsrna-26127	CTTTGCACGCGTGGGTTC	0	4	0	0
tsrna-26126	CTTTGCACGCGTGGGTT	1	1	0	0
tsrna-26125	CTTTGCACGCGTGGGT	2	5	1	0
tsrna-26124	CTTTGATAGAGTAAATAATAGGAGCTTAAA	0	0	0	1
tsrna-26123	CTTTGATAGAGTAAATAATAGGAGCTTAA	0	0	0	0
tsrna-26122	CTTTGATAGAGTAAATAATAGGAGCTT	0	0	1	1
tsrna-26121	CTTTGATAGAGTAAATAATAGGAGCT	0	0	0	0
tsrna-26120	CTTTGATAGAGTAAATAATAGGAGC	0	0	0	0
tsrna-26110	CTTTGAATCCAGCGATCCGAGTTCA	1	2	0	1
tsrna-26109	CTTTGAATCCAGCGATCCGAGTTC	1	2	3	1
tsrna-26108	CTTTGAATCCAGCGATCCGAGTT	2	0	1	0
tsrna-26107	CTTTGAATCCAGCGATCCGAGT	1	1	1	1
tsrna-26106	CTTTGAATCCAGCGATCCGAG	3	0	1	0
tsrna-26105	CTTTGAATCCAGCGATCCGA	1	3	0	1
tsrna-26104	CTTTGAATCCAGCGATCCG	3	1	2	0
tsrna-26103	CTTTGAATCCAGCGATCC	4	1	1	0
tsrna-26102	CTTTGAATCCAGCGATC	0	0	2	1
tsrna-26101	CTTTGAATCCAGCGAT	1	3	0	0
tsrna-26100	CTTTGAATCCAGCAATCCGAGTT	5	17	5	10
tsrna-26099	CTTTGAATCCAGCAATCCGAGT	2	22	3	3
tsrna-26098	CTTTGAATCCAGCAATCCGAG	3	2	1	1
tsrna-26097	CTTTGAATCCAGCAATCCGA	1	2	2	0
tsrna-26096	CTTTCGATAGCTCAGTTGGTAGAGC	1	0	1	0
tsrna-26095	CTTTCACCGCCGCGGCCCGGGTTC	0	4	1	8
tsrna-26094	CTTTCACCGCCGCGGCCCGGGT	1	4	1	6
tsrna-26093	CTTTCACCGCCGCGGCCCG	2	3	1	5
tsrna-26092	CTTTCACCGCCGCGGCCC	0	1	1	3
tsrna-26091	CTTTCACCGCCGCGGCC	2	3	0	0
tsrna-26090	CTTTCACCGCCGCGGC	2	0	3	1
tsrna-26089	CTTTACAGTCAGAGGTTCA	0	0	0	0
tsrna-26087	CTTTACAGTCAGAGGTT	1	0	0	0
tsrna-26085	CTTTACACGCAGAAGGTCCTGG	1	0	0	0
tsrna-26084	CTTTACACGCAGAAGGTCCTG	0	1	0	0
tsrna-26083	CTTGTAGTTGAAATACAACGATGGTTTTTC	1	0	2	0
tsrna-26082	CTTGTAGTTGAAATACAACGATGGTTTTT	1	1	1	0
tsrna-26078	CTTGTAGTATAAACTAATACACCAG	0	0	1	0

tsrna-26077	CTTGTAAACCGGAGATGAAAACCTTTTTCC	2	7	4	0
tsrna-26074	CTTGTAAACCAGGGGTCGCGAGT	32	59	18	5
tsrna-26073	CTTGTAAACCAGGGGTCGCGA	1	3	2	2
tsrna-26072	CTTGTAAACCAGGGGTCGCG	0	4	1	0
tsrna-26071	CTTGTAAACAGGAGATCCTGGGT	10	82	10	7
tsrna-26068	CTTGACCGCTCTGACCA	0	1	0	0
tsrna-26066	CTTGAAACCAGCTTTGGGGGGTTC	0	0	1	0
tsrna-26065	CTTCTGTAGTGTAGTGGTTATCACGTTCGCCT	3	3	11	2
tsrna-26064	CTTCTGTAGTGTAGTGGTTATCACGTTCGCC	3	0	1	1
tsrna-26063	CTTCTGTAGTGTAGTGGTTATCACGTTCGC	2	0	0	1
tsrna-26062	CTTCTGTAGTGTAGTGGTTATCACGTTCG	2	0	1	0
tsrna-26061	CTTCTGTAGTGTAGTGGTTATCACGTTC	1	0	0	1
tsrna-26060	CTTCTGTAGTGTAGTGGTTATCACGTT	1	0	0	1
tsrna-26059	CTTCTGTAGTGTAGTGGTTATCACGT	0	0	0	0
tsrna-26058	CTTCTGTAGTGTAGTGGTTATCACG	0	1	0	1
tsrna-26057	CTTCTGTAGTGTAGTGGTTATCAC	1	0	0	1
tsrna-26056	CTTCTGTAGTGTAGTGGTTATCA	1	0	0	0
tsrna-26055	CTTCTGTAGTGTAGTGGTTATC	0	0	2	0
tsrna-26049	CTTCTAATTCAAAGGTTCCGGGTT	0	2	2	1
tsrna-26048	CTTCTAATTCAAAGGTTCCGGGT	0	0	1	0
tsrna-26047	CTTCTAATTCAAAGGTTCCGGG	0	1	0	0
tsrna-26045	CTTCGGGGGCGTGGGTTCG	0	1	0	0
tsrna-26042	CTTCGGATCAGAAGATTGCAGGTT	0	0	0	0
tsrna-26041	CTTCGGATCAGAAGATTGCAGGT	1	0	0	0
tsrna-26037	CTTCGGATCAGAAGATTGAGGGTT	4	9	2	0
tsrna-26036	CTTCGGATCAGAAGATTGAGGGT	5	10	0	0
tsrna-26030	CTTCGGAGGCGTGGGTTC	0	2	0	0
tsrna-26029	CTTCGGAGGCGTGGGTT	0	1	0	0
tsrna-26028	CTTCGGAGGCGTGGGT	0	1	0	0
tsrna-26027	CTTCGATAGCTCAGTTGGTAGAGCG	1	1	0	1
tsrna-26026	CTTCGATAGCTCAGTTGGTAGAGC	2	1	2	0
tsrna-26025	CTTCGATAGCTCAGTTGGTAGAG	3	0	4	1
tsrna-26024	CTTCGATAGCTCAGTTGGTAGA	0	0	1	0
tsrna-26023	CTTCGATAGCTCAGTTGG	1	0	1	0
tsrna-26022	CTTCGATAGCTCAGCTGGTAGAGC	2	0	2	0
tsrna-26021	CTTCGATAGCTCAGCTGGTAGAG	3	2	0	1
tsrna-26020	CTTCGATAGCTCAGCTGGTAGA	3	2	2	0
tsrna-26019	CTTCGATAGCTCAGCTGGTAG	2	2	0	2
tsrna-26018	CTTCGATAGCTCAGCTGG	0	0	0	2
tsrna-26017	CTTCGATAGCTCAGCT	2	0	1	1
tsrna-26014	CTTCCAAGCAGTTGACCCGGGTTCGATTCC	13	27	7	5
tsrna-26013	CTTCCAAGCAGTTGACCCGGGTTCG	17	21	14	2
tsrna-26012	CTTCCAAGCAGTTGACCCGGGTTC	21	16	6	8
tsrna-26011	CTTCCAAGCAGTTGACCCGGGTT	20	24	9	0
tsrna-26010	CTTCCAAGCAGTTGACCCGGGT	21	13	8	6
tsrna-26009	CTTCCAAGCAGTTGACCCGGG	1	2	0	0
tsrna-26008	CTTCCAAGCAGTTGACCCGG	0	0	0	0
tsrna-26007	CTTCCAAGCAGTTGACCCG	0	1	0	0
tsrna-26006	CTTCCAAGCAGTTGACCC	1	0	0	0
tsrna-26005	CTTCCAAGCAGTTGAC	1	0	0	0
tsrna-26004	CTTCAATAGCTCAGCTGGTAGAGC	1	0	0	0
tsrna-26002	CTTCAAAGCCCTCAGTAAGTTG	0	0	0	0
tsrna-26001	CTTCAAACCTGCCGGGGCTTCCA	6	5	4	2
tsrna-26000	CTTCAAACCTGCCGGGGCTTCC	0	1	1	0
tsrna-25999	CTTCAAACCTGCCGGGGCTTC	1	0	0	0
tsrna-25994	CTTATAATGCCGAGGTTGTGAGTTC	9	13	6	7
tsrna-25993	CTTATAATGCCGAGGTTGTGAGTT	18	25	5	8
tsrna-25992	CTTATAATGCCGAGGTTGTGAGT	14	16	12	3
tsrna-25991	CTTATAATGCCGAGGTTGTGAG	0	3	0	2
tsrna-25990	CTTATAATGCCGAGGTTGTGA	3	3	1	1
tsrna-25989	CTTATAATGCCGAGGTTGTG	3	3	1	0
tsrna-25988	CTTATAATGCCGAGGTTG	0	1	0	0
tsrna-25987	CTTAGGTCGCTGGTTCGATTCCGGCTCGA	1	0	0	0
tsrna-25986	CTTAGGTCGCTGGTTCGAATCCGGCTCGA	1	2	1	0

tsrna-25985	CTTAGGTCGCTGGTTCG	2	0	0	0
tsrna-25984	CTTAGGTCGCTGGTTC	0	1	0	0
tsrna-25983	CTTAGGAGATTTCAACTTAACTTGACCGCTCTGACCA	85	57	77	9
tsrna-25982	CTTAGGAGATTTCAACTTAACTTGACCGCTCTGACC	9	15	8	5
tsrna-25981	CTTAGGAGATTTCAACTTAACTTGACCGCTCTGAC	4	5	4	0
tsrna-25980	CTTAGGAGATTTCAACTTAACTTGACCGCTCTGA	1	5	3	1
tsrna-25979	CTTAGGAGATTTCAACTTAACTTGACCGCTCTG	1	2	1	0
tsrna-25978	CTTAGGAGATTTCAACTTAACTTGACCGCT	1	0	3	0
tsrna-25977	CTTAGGAGATTTCAACTTAACTTGACCGC	0	3	0	0
tsrna-25976	CTTAGGAGATTTCAACTTAACTTGACCG	0	1	0	0
tsrna-25975	CTTAGGAGATTTCAACTTAACTTGACC	0	1	1	0
tsrna-25974	CTTAGGAGATTTCAACTTAACTTGAC	1	0	0	0
tsrna-25973	CTTAGGAGATTTCAACTTAACTTG	1	0	0	0
tsrna-25972	CTTAGGAGATTTCAACTTAACTT	0	1	0	0
tsrna-25967	CTTAGGAGATTTCAACT	0	0	0	0
tsrna-25965	CTTAGCTTTGGGTGCTAATGGTGGAGTTA	2	3	0	1
tsrna-25962	CTTAGCTGTTAACTAAGTGTTTGTGG	0	0	0	0
tsrna-25961	CTTAGCATTAACCTTTTAAGTTAAAGATTAAGAGAA	0	0	0	0
tsrna-25960	CTTAGCATTAACCTTTTAAGTTAAAGATT	0	0	1	0
tsrna-25953	CTTACGACCCCTTATTTACCCCA	2	0	1	0
tsrna-25952	CTTACGACCCCTTATTTACCCC	1	0	0	0
tsrna-25951	CTTACGACCCCTTATTTACCC	0	0	0	0
tsrna-25945	CTTACCTCCTCAAAGCAATACACTGA	3	1	0	0
tsrna-25944	CTTACCTCCTCAAAGCAATACACT	1	4	0	1
tsrna-25943	CTTACCTCCTCAAAGCAATACA	1	0	0	0
tsrna-25942	CTTACCTCCTCAAAGCAATAC	3	0	1	0
tsrna-25941	CTTACCTCCTCAAAGCAATA	2	1	1	0
tsrna-25937	CTTACACTTAGGAGATTTCAACTTAACTTGACCGCTCTGACCA	104	47	96	14
tsrna-25936	CTTACACTTAGGAGATTTCAACTTAACTTGACCGCTCTGACC	14	10	16	1
tsrna-25935	CTTACACTTAGGAGATTTCAACTTAACTTGACCGCTCTGAC	6	2	6	0
tsrna-25934	CTTACACTTAGGAGATTTCAACTTAACTTG	0	0	0	0
tsrna-25933	CTTACACTTAGGAGATTTCAACTTAACTT	0	0	0	0
tsrna-25932	CTTACACTTAGGAGATTTCAACTTAACT	0	0	1	1
tsrna-25931	CTTACACTTAGGAGATTTCAACTTA	0	0	1	0
tsrna-25930	CTTACACTTAGGAGATTTCAACTT	0	0	0	0
tsrna-25929	CTTACACTTAGGAGATTTCAACT	0	1	0	0
tsrna-25921	CTTAATCTCAGGGTCGTGGGTTCGAGCCCCACGTTGGGCGC(	20	250	9	8
tsrna-25920	CTTAATCTCAGGGTCGTGGGT	5	5	4	3
tsrna-25919	CTTAATCTCAGGGTCGTGGG	1	5	1	0
tsrna-25918	CTTAATCTCAGGGTCGTGG	4	4	4	3
tsrna-25917	CTTAATCTCAGGGTCGTG	5	4	3	0
tsrna-25916	CTTAATCCCAGGGTCGTGGGTTCGAGCCCCACGTTGGGCGC(	17	245	8	3
tsrna-25915	CTTAATCCCAGGGTCGTGGGTT	1	0	0	0
tsrna-25913	CTTAATCCCAGGGTCGTG	1	0	0	0
tsrna-25912	CTTAATCCCAGGGTCGT	2	0	1	0
tsrna-25904	CTTAACTTGACCGCTCTGACCA	15	17	9	2
tsrna-25903	CTTAACTTGACCGCTCTGACC	1	1	0	0
tsrna-25902	CTTAACTTGACCGCTCTGAC	0	1	0	0
tsrna-25901	CTTAACTTGACCGCTCTGA	0	1	0	0
tsrna-25900	CTTAACTTGACCGCTCTG	0	0	0	0
tsrna-25897	CTTAACACAAAGCACCCAACTTACACTTAG	0	0	0	0
tsrna-25892	CTTAACACAAAGCACCCAACT	0	0	1	0
tsrna-25886	CTTAAACCCCCTTATTTCTACCA	2	1	0	0
tsrna-25885	CTTAAACCCCCTTATTTCTACC	0	2	0	0
tsrna-25883	CTTAAACCCCCTTATTTCTA	0	1	0	0
tsrna-25882	CTTAAACCCCCTTATTTCT	0	1	1	0
tsrna-25881	CTTAAACCCCCTTATTT	0	0	1	0
tsrna-25880	CTTAAAACTTTACAGTCAGAGGTTCA	0	0	1	0
tsrna-25879	CTTAAAACTTTACAGTCAGAGGTTC	1	0	0	0
tsrna-25878	CTTAAAACTTTACAGTCAGAGGTT	0	1	0	0
tsrna-25877	CTTAAAACTTTACAGTCAGAGGT	0	3	0	0
tsrna-25876	CTTAAAACTTTACAGTCAGAGG	0	2	0	0
tsrna-25872	CTGTTAACTGAAAGGTTGGTGGT	0	1	0	0
tsrna-25869	CTGTTAACTAAGTGTTTGTGGGTTTAAGTC	1	1	0	1

tsrna-25868	CTGTTAACTAAGTGTTTGTGGGTTTA	1	0	0	0
tsrna-25867	CTGTTAACTAAGTGTTTGTGGGTTT	0	1	0	1
tsrna-25866	CTGTTAACTAAGTGTTTGTGGGTT	0	1	0	0
tsrna-25865	CTGTTAACTAAGTGTTTGTGGGT	1	0	0	0
tsrna-25863	CTGTTAACTAAGTGTTTGTGG	0	0	0	0
tsrna-25858	CTGTTAACCGAAAGGTTGGTGGT	7	37	2	7
tsrna-25857	CTGTTAACCGAAAGGTTGGTGG	0	2	0	0
tsrna-25856	CTGTTAACCGAAAGGTTGGTG	0	3	0	0
tsrna-25850	CTGTTAACCGAAAGATTGGTGGT	0	0	0	0
tsrna-25849	CTGTTAACCGAAAGATTGGTGG	0	0	1	0
tsrna-25837	CTGTGGCGCAATCGGTTAGC	0	1	0	0
tsrna-25834	CTGTGATGGCCGAGTGGTT	0	1	0	0
tsrna-25833	CTGTCTTGTAAACAGGAGATCCTGGGT	22	94	9	4
tsrna-25832	CTGTCACGCGGGAGACTGGGGT	34	122	32	8
tsrna-25831	CTGTCACGCGGGAGACCGGGGTTCGATTCCCCGACGGGGAG	18	65	13	2
tsrna-25830	CTGTCACGCGGGAGACCGGGGTTCGATTCC	15	53	9	2
tsrna-25829	CTGTCACGCGGGAGACCGGGGTTCG	12	36	8	2
tsrna-25828	CTGTCACGCGGGAGACCGGGGTTC	15	45	6	0
tsrna-25827	CTGTCACGCGGGAGACCGGGGTT	18	56	5	2
tsrna-25826	CTGTCACGCGGGAGACCGGGGT	9	47	10	3
tsrna-25825	CTGTCACGCGGGAGACCGGGG	23	49	4	1
tsrna-25824	CTGTCACGCGGGAGACCGGG	15	41	5	1
tsrna-25823	CTGTCACGCGGGAGACCGG	18	38	9	3
tsrna-25822	CTGTCACGCGGGAGACCG	10	47	4	5
tsrna-25821	CTGTCACGCGGGAGACC	18	36	5	3
tsrna-25820	CTGTCACGCGGGAGAC	16	43	5	2
tsrna-25816	CTGTAGTGTAGTGGTTATCACGTTCGC	0	0	1	0
tsrna-25812	CTGTAGTGTAGTGGTTATCACGT	1	0	0	0
tsrna-25809	CTGTAGTGTAGTGGTTATCA	0	1	0	0
tsrna-25804	CTGTAGATCCTTAGGTCGCTGGTTCGATTC	1	0	1	1
tsrna-25803	CTGTAGATCCTTAGGTCGCTGGTTCG	1	0	0	0
tsrna-25802	CTGTAGATCCTTAGGTCGCTGGTTC	0	0	0	0
tsrna-25801	CTGTAGATCCTTAGGTCGCTGGTT	0	0	1	0
tsrna-25789	CTGTAAATCTAAAGACAGGGG	1	0	0	0
tsrna-25782	CTGTAAAGCTAACTTAGCATTAACCT	0	1	0	0
tsrna-25771	CTGGTTTTCACCCAGGTGGCCCGGGTTCG	2	5	4	2
tsrna-25770	CTGGTTTTCACCCAGGTGGCCCGGG	2	4	2	0
tsrna-25769	CTGGTTTTCACCCAGGTGGCCCGG	1	4	4	1
tsrna-25768	CTGGTTTTCACCCAGGTGGCCCG	2	0	1	1
tsrna-25767	CTGGTTTTCACCCAGGTGGCCC	3	3	2	0
tsrna-25766	CTGGTTTTCACCCAGGTGGCC	3	6	4	0
tsrna-25765	CTGGTTTTCACCCAGGCGGCCCGG	2	1	0	0
tsrna-25764	CTGGTTTTCACCCAGGCGGCCCG	2	2	0	0
tsrna-25763	CTGGTTTTCACCCAGGCGGCCC	2	1	0	1
tsrna-25762	CTGGTTCGATTCCGGCTCGAAGGACCA	61	183	25	26
tsrna-25761	CTGGTTCGATTCCGGCTCGAAGGACC	1	12	1	0
tsrna-25758	CTGGTTCGATTCCGGCTCGAAGG	0	0	0	0
tsrna-25755	CTGGTTCGATCCCGGGTTTCGGCACCA	40	108	10	9
tsrna-25754	CTGGTTCGATCCCGGGTTTCGGCA	0	0	0	0
tsrna-25752	CTGGTTCGATCCCGGGTTTCGG	0	0	0	0
tsrna-25751	CTGGTTCGAATCCGGCTCGAAGGACCA	16	51	12	7
tsrna-25750	CTGGTTCGAATCCGGCTCGAAGGACC	2	6	2	0
tsrna-25747	CTGGTTCGAATCCGGCTCGAAGG	0	0	0	1
tsrna-25744	CTGGTTCAAATCCGGGTGCCCCCTCCA	172	160	77	18
tsrna-25742	CTGGTGTAGTGGTATCATGCAAGATTC	3	4	1	0
tsrna-25741	CTGGTGTAGTGGTATCATGCAAGATT	3	4	1	0
tsrna-25740	CTGGTGTAGTGGTATCATGCAAGA	2	1	2	0
tsrna-25739	CTGGTGTAGTGGTATCATGCAAG	1	0	1	0
tsrna-25738	CTGGTGTAGTGGTATCATGCA	0	0	1	1
tsrna-25737	CTGGTGTAGTGGTATCATGC	0	0	1	0
tsrna-25736	CTGGTGTAGTGGTATCATG	1	1	1	0
tsrna-25734	CTGGTGGTTCAGTGGTAGAATTCT	0	1	4	0
tsrna-25733	CTGGTGGTCTAGTGGTTAGGATTCGGCGCTCTC	35	52	19	15
tsrna-25732	CTGGTGGTCTAGTGGTTAGGATTCGGCGCTC	40	53	17	13

tsrna-25731	CTGGTGGTCTAGTGGTTAGGATTCGGCGCT	40	45	20	11
tsrna-25730	CTGGTGGTCTAGTGGTTAGGATTCGGCGC	20	17	15	8
tsrna-25729	CTGGTGGTCTAGTGGTTAGGATTCGGCG	13	10	8	4
tsrna-25728	CTGGTGGTCTAGTGGTTAGGATTCGGC	7	6	8	4
tsrna-25727	CTGGTGGTCTAGTGGTTAGGATTCGG	3	1	1	2
tsrna-25726	CTGGTGGTCTAGTGGTTAGGATTCG	0	3	1	0
tsrna-25725	CTGGTGGTCTAGTGGTTAGGATTC	6	2	1	0
tsrna-25724	CTGGTGGTCTAGTGGTTAGGATT	2	3	0	2
tsrna-25723	CTGGTGGTCTAGTGGTTAGGAT	0	1	0	0
tsrna-25718	CTGGTGGTCTAGTGGCTAGGATTCGGCGC	13	34	7	32
tsrna-25717	CTGGTGGTCTAGTGGCTAGGATTCGGCG	17	41	5	32
tsrna-25716	CTGGTGGTCTAGTGGCTAGGATTCGGC	10	14	1	15
tsrna-25715	CTGGTGGTCTAGTGGCTAGGATTCGG	3	6	3	9
tsrna-25714	CTGGTGGTCTAGTGGCTAGGATTCG	2	4	3	6
tsrna-25713	CTGGTGGTCTAGTGGCTAGGA	0	0	0	2
tsrna-25712	CTGGTGGTCTAGTGGCTAGG	0	0	0	0
tsrna-25710	CTGGTCTCCGGATGGAGGCGTGG	0	7	1	0
tsrna-25709	CTGGTCTCCGGATGGAGGCG	0	0	0	0
tsrna-25705	CTGGTCTCCAATGGAGGCGTGG	0	1	0	0
tsrna-25703	CTGGTCTAGTGGTTAGGATT	0	0	0	0
tsrna-25693	CTGGTAGAGCAGAGGA	0	1	0	0
tsrna-25692	CTGGGTTCGATCCCCAGTACCTCCACCA	28	44	13	19
tsrna-25691	CTGGGTTCGAGCCCCAGTGGAACCACCA	41	27	30	21
tsrna-25690	CTGGGTTCGAGCCCCAGTGGAACC	1	2	0	0
tsrna-25689	CTGGGTTCGAATCCCAGCGGTGCCTCCA	24	51	4	4
tsrna-25688	CTGGGTTCGAATCCCAGCGGGGCCTCCA	1	5	0	0
tsrna-25686	CTGGGGATTGTGGGTTCGAGTCCCATCTGGGTCGCCA	1	13	2	0
tsrna-25685	CTGGGGATTGTGGGTTCGAGTCCCATCTGGGTCGCC	3	4	0	0
tsrna-25684	CTGGGGATTGTGGGTTCGAGTCCCATCTGG	1	3	0	0
tsrna-25683	CTGGGGATTGTGGGTTCG	0	1	0	0
tsrna-25681	CTGGGGATTGTGGGTT	0	0	0	0
tsrna-25680	CTGGGCGGAAACACCA	1	1	0	0
tsrna-25679	CTGGCGGTCTAGTGGTTAGGATTCGGCGC	20	20	17	9
tsrna-25678	CTGGATAGCTCAGTTGGTAGAAC	1	1	0	0
tsrna-25673	CTGGACTTTGAATCCAGCGATCC	1	1	0	0
tsrna-25672	CTGGACTTTGAATCCAGCGA	0	2	0	0
tsrna-25671	CTGGACTTTGAATCCAGCG	0	0	1	0
tsrna-25665	CTGGACTCTGAATCCAGCGATCCGAGTTCG	0	3	0	1
tsrna-25664	CTGGACTCTGAATCCAGCGATCCGAGTTC	0	1	0	0
tsrna-25663	CTGGACTCTGAATCCAGCGATCCGAGTT	1	2	0	0
tsrna-25662	CTGGACTCTGAATCCAGCGATCCGAGT	0	1	0	0
tsrna-25661	CTGGACTCTGAATCCAGCGATCCGAG	0	1	0	1
tsrna-25660	CTGGACTCTGAATCCAGCGATCCGA	0	1	0	0
tsrna-25659	CTGGACTCTGAATCCAGCGATCCG	1	1	0	0
tsrna-25658	CTGGACTCTGAATCCAGCGATCC	0	2	0	1
tsrna-25656	CTGGACTCTGAATCCAGCGAT	0	1	0	0
tsrna-25654	CTGGACTCTGAATCCAGCG	0	0	0	1
tsrna-25653	CTGGACTCTGAATCCAGCA	1	0	0	0
tsrna-25652	CTGGACTCTGAATCCAGC	0	1	0	0
tsrna-25651	CTGGACTCTGAATCCAG	1	0	0	0
tsrna-25648	CTGCTTTACACGCAGAAGGTCCTGG	0	0	0	0
tsrna-25645	CTGCTAATCCATTGTGCTTTGC	1	2	0	0
tsrna-25644	CTGCTAATCCATTGTGCTT	0	1	0	0
tsrna-25643	CTGCTAATCCATTGTGCTCTGC	0	2	1	1
tsrna-25639	CTGCTAACTCATGCCCCCATGTCTAACAACATGGCTTTCTCAC	7	3	21	0
tsrna-25638	CTGCTAACTCATGCCCCCATGTCTAACAAC	1	0	0	0
tsrna-25637	CTGCTAACTCATGCCCCCATGTCTAACAA	0	0	0	0
tsrna-25636	CTGCTAACTCATGCCCCCATGTCTAACA	0	0	1	0
tsrna-25635	CTGCTAACTCATGCCCCCATGTCTAAC	0	0	0	0
tsrna-25633	CTGCTAACTCATGCCCCCATGTCTA	1	0	0	0
tsrna-25631	CTGCTAACTCATGCCCCCATGTC	0	0	0	0
tsrna-25618	CTGCGTGTTCGAATCACGTCGGGGTCACC	0	0	0	1
tsrna-25614	CTGCGTGTTCGAATCACGTCGGGGT	1	0	0	0
tsrna-25604	CTGCCTTCCAAGCAGTTGACCCGGGTTCG	30	37	15	17

tsrna-25603	CTGCCTTCCAAGCAGTTGACCCGGGTTC	26	34	20	11
tsrna-25602	CTGCCTTCCAAGCAGTTGACCCGGGTT	38	31	15	15
tsrna-25601	CTGCCTTCCAAGCAGTTGACCCGGGT	31	38	22	14
tsrna-25600	CTGCCTTCCAAGCAGTTGACCCGGG	1	5	2	0
tsrna-25599	CTGCCTTCCAAGCAGTTGACCCGG	1	2	2	2
tsrna-25598	CTGCCTTCCAAGCAGTTGACCCG	0	0	1	0
tsrna-25595	CTGCCTTCCAAGCAGTTGAC	0	0	0	0
tsrna-25591	CTGCCTGTCACGCGGGAGAC	11	50	5	1
tsrna-25590	CTGCCACGCGGGAGGCCCGGGTTT	7	19	0	0
tsrna-25589	CTGCCACGCGGGAGGCCCGGGTTCG	4	17	2	1
tsrna-25588	CTGCCACGCGGGAGGCCCGGGTTC	6	17	1	0
tsrna-25587	CTGCCACGCGGGAGGCCCGGGTT	4	21	1	2
tsrna-25586	CTGCCACGCGGGAGGCCCGGGT	1	11	2	0
tsrna-25585	CTGCCACGCGGGAGGCCCGGG	0	2	0	0
tsrna-25584	CTGCCACGCGGGAGGCCCGG	0	0	1	0
tsrna-25582	CTGCCACGCGGGAGGCCC	0	0	2	0
tsrna-25579	CTGCAGATCAAGAGGTCCCTGGTTCA	7	7	0	2
tsrna-25578	CTGCAGATCAAGAGGTCCCTGGTTC	3	5	2	2
tsrna-25577	CTGCAGATCAAGAGGTCCCTGGTT	5	4	1	2
tsrna-25576	CTGCAGATCAAGAGGTCCCTGGT	2	3	2	3
tsrna-25575	CTGCAGATCAAGAGGTCCCTGG	0	0	0	0
tsrna-25574	CTGCAGATCAAGAGGTCCCTG	0	0	1	0
tsrna-25572	CTGCAGATCAAGAGGTCCCCGGTTCAA	0	4	1	0
tsrna-25571	CTGCAGATCAAGAGGTCCCCGGTTCA	1	1	0	0
tsrna-25570	CTGCAGATCAAGAGGTCCCCGGTTC	0	1	3	1
tsrna-25569	CTGCAGATCAAGAGGTCCCCGGTT	4	4	1	0
tsrna-25568	CTGCAGATCAAGAGGTCCCCGGT	0	2	0	1
tsrna-25558	CTGCAGATCAAGAAGTCCCCGGT	1	2	0	0
tsrna-25554	CTGCAGATCAAAAGGTCCCTGGT	11	4	2	3
tsrna-25550	CTGATTTGCGTTCAGTTGATGCAGAGTGGG	0	1	1	0
tsrna-25549	CTGATTTGCGTTCAGTTGATGCAGAGTGG	2	0	1	1
tsrna-25548	CTGATTTGCGTTCAGTTGATGCAGAGTG	0	1	0	0
tsrna-25545	CTGATTTGCGTTCAGTTGATGCAGA	0	0	1	0
tsrna-25538	CTGATTCCGGATCAGAAGATTGAGG	0	1	0	0
tsrna-25537	CTGATAACACCAAGGTCGCGGGC	5	26	3	3
tsrna-25536	CTGATAACACCAAGGTCGCGG	0	2	1	1
tsrna-25534	CTGATAAAAGAGTTACTTTGA	0	1	0	0
tsrna-25532	CTGAGTTCGAACCTCAGAGGGGGCACCA	1	2	0	0
tsrna-25530	CTGAGTGAAGCATTGGACTGTAAATCTAA	0	0	1	1
tsrna-25529	CTGAGTGAAGCATTGGACTGTAAATCTA	1	0	0	0
tsrna-25528	CTGAGTGAAGCATTGGACTGTAAATCT	0	1	1	0
tsrna-25527	CTGAGTGAAGCATTGGACTGTAAATC	1	1	2	0
tsrna-25526	CTGAGTGAAGCATTGGACTGTAAAT	0	1	1	0
tsrna-25525	CTGAGTGAAGCATTGGACTGTAAA	0	1	0	1
tsrna-25524	CTGAGTGAAGCATTGGACTGTAA	1	0	0	0
tsrna-25523	CTGAGTGAAGCATTGGACTGTA	1	0	0	0
tsrna-25515	CTGAGGGTCCAGGGTTCAAGTCCCTGTTCGGGCGCCA	7	13	9	2
tsrna-25514	CTGAGGGTCCAGGGTTCAAGTCCCTGTTCGGGCGCC	0	0	1	0
tsrna-25513	CTGAGGGTCCAGGGTTCAAGTCCCTGTTCGGGCGC	0	0	0	0
tsrna-25512	CTGAGGGTCCAGGGTTCAAGTCCCTGTTCGGGC	1	1	0	0
tsrna-25511	CTGAGGGTCCAGGGTTCAAGTCCCTGTTCG	0	1	0	0
tsrna-25510	CTGAGGGTCCAGGGTTCA	0	0	0	0
tsrna-25509	CTGAGGGTCCAGGGTTC	1	0	0	0
tsrna-25503	CTGACTTCGGATCAGAAGATTGAGGGT	6	5	1	2
tsrna-25497	CTGACTCCAGATCAGAAGGTTGCGTGTTC	1	2	0	1
tsrna-25496	CTGACTCCAGATCAGAAGGTTGCGTGTT	0	5	1	0
tsrna-25495	CTGACTCCAGATCAGAAGGTTGCGTGT	0	1	0	0
tsrna-25494	CTGACTCCAGATCAGAAGGTTGCGTG	0	1	0	0
tsrna-25490	CTGACTCCAGATCAGAAGGCTGCGTGTT	0	0	1	0
tsrna-25480	CTGACAACAGAGGCTTACGACCCCTTATTTACCCCA	2	1	0	0
tsrna-25479	CTGACAACAGAGGCTTACGACCCCTTATTT	0	0	0	0
tsrna-25478	CTGACAACAGAGGCTTACGACCCCTTA	0	0	0	0
tsrna-25475	CTGAATCCAGCGATCCGAGTTCAAATCTCGGTGGAACCTCCA	160	216	74	26
tsrna-25474	CTGAATCCAGCGATCCGAGTTCAAATCTCGGTGGAACCT	1	2	1	0

tsrna-25473	CTGAATCCAGCGATCCGAGTTCAA	0	1	0	0
tsrna-25472	CTGAATCCAGCGATCCGAGTTCA	0	1	0	0
tsrna-25471	CTGAATCCAGCGATCCGAGTTC	0	0	0	0
tsrna-25470	CTGAATCCAGCGATCCGAGTT	0	2	0	0
tsrna-25468	CTGAATCCAGCGATCCGAG	0	2	0	0
tsrna-25467	CTGAATCCAGCGATCCGA	0	1	0	0
tsrna-25466	CTGAATCCAGCGATCCG	0	1	0	0
tsrna-25464	CTGAAGGTCGTGAGTTCGATCCTCACACGGGGCACCA	4	29	1	0
tsrna-25463	CTGAAGGTCGTGAGTTCGATCCTCACACGGGGCACC	2	5	2	0
tsrna-25462	CTGAAGGTCGTGAGTTCGATCCTCACACGGGGCAC	1	1	1	0
tsrna-25461	CTGAAGGTCGTGAGTTCGATCCTCACACGGGGC	1	2	0	0
tsrna-25460	CTGAAGGTCGTGAGTTCGATCCTCACACGG	3	4	1	0
tsrna-25459	CTGAAGGTCGTGAGTTCGATCC	1	4	0	1
tsrna-25458	CTGAAGGTCGTGAGTTCGAGCCTCACACGGGGCACCA	0	3	3	1
tsrna-25457	CTGAAGGTCGTGAGTTCGAGCCTCACACGG	0	4	1	2
tsrna-25456	CTGAAGGTCGTGAGTTCG	1	1	2	0
tsrna-25455	CTGAAGGTCGTGAGTTC	0	2	2	0
tsrna-25454	CTGAAGGTCGTGAGTT	1	4	1	1
tsrna-25453	CTGAAGGTCCTGAGTTCGAGCCTCAGAGAGGGCACCA	10	45	12	5
tsrna-25452	CTGAAGGTCCTGAGTTCGAACCTCAGAGGGGGCACCA	12	45	13	1
tsrna-25451	CTGAAGGTCCTGAGTTCGAACCTCAGAGGGGGCACC	10	33	12	2
tsrna-25450	CTGAAGGTCCTGAGTTCGAACCTCAGAGGGGGC	15	29	12	4
tsrna-25449	CTGAAGGTCCTGAGTTCGAACC	14	23	9	4
tsrna-25448	CTGAAGGTCCTGAGTTCGAAC	14	45	10	3
tsrna-25447	CTGAAGGTCCTGAGTTCGAA	14	32	10	4
tsrna-25446	CTGAAGGTCCTGAGTTCGA	14	41	3	2
tsrna-25445	CTGAAGGTCCTGAGTTCG	13	37	3	4
tsrna-25444	CTGAAGGTCCTGAGTTC	9	33	7	2
tsrna-25443	CTGAAGGTCCTGAGTT	17	40	9	5
tsrna-25442	CTGAAGATCTAAAGGTCCCTGGTT	19	23	12	4
tsrna-25441	CTGAAGATCTAAAGGTCCCTGGT	12	16	4	3
tsrna-25440	CTGAAGATCTAAAGGTCCCTGG	0	0	0	0
tsrna-25439	CTGAAGATCTAAAGGTCCCTG	2	0	0	0
tsrna-25438	CTGAAGATCTAAAGGTCCCT	1	0	0	0
tsrna-25436	CTGAAGATCTAAAGGTCC	0	1	0	0
tsrna-25434	CTGAAAATGTTTAGACGGGCTCACATCACC	69	49	25	6
tsrna-25433	CTGAAAATGTTTAGACGGGCTCACATC	31	35	12	6
tsrna-25432	CTGAAAATGTTTAGACGGGCTCACAT	29	36	15	2
tsrna-25431	CTGAAAATGTTTAGACGGGCTCACA	42	33	11	3
tsrna-25430	CTGAAAATGTTTAGACGGGCTCAC	25	15	3	1
tsrna-25429	CTGAAAATGTTTAGACGGGCTCA	11	15	4	1
tsrna-25428	CTGAAAATGTTTAGACGGGCTC	6	11	0	0
tsrna-25427	CTGAAAATGTTTAGACGGGCT	0	4	1	1
tsrna-25426	CTGAAAATGTTTAGACGGGC	1	1	0	0
tsrna-25425	CTGAAAATGTTTAGACGGG	0	2	0	0
tsrna-25421	CTCTTTTAGTATAAATAGTACCGTTAACTT	0	0	1	0
tsrna-25414	CTCTTCGGGGGCGTGGGTTCGAA	0	2	0	0
tsrna-25413	CTCTTCGGGGGCGTGGGTTCG	1	1	0	0
tsrna-25412	CTCTTCGGGGGCGTGGGTTC	1	3	0	0
tsrna-25411	CTCTTCGGGGGCGTGGGTT	1	3	0	0
tsrna-25410	CTCTTCGGGGGCGTGGGT	0	2	1	0
tsrna-25408	CTCTTCGGGGGCGTGG	0	1	0	0
tsrna-25407	CTCTTCGGAGGCGTGG	1	1	0	0
tsrna-25406	CTCTTAGCGCAGCGGGCAGC	0	0	0	0
tsrna-25404	CTCTTAATCTCAGGGTCGTGGGTT	7	9	1	5
tsrna-25403	CTCTTAATCTCAGGGTCGTGGGT	3	12	1	3
tsrna-25402	CTCTTAATCTCAGGGTCGTGGG	3	10	3	1
tsrna-25401	CTCTTAATCTCAGGGTCGTGG	4	9	1	2
tsrna-25400	CTCTTAATCTCAGGGTCGTG	6	5	2	2
tsrna-25399	CTCTTAATCTCAGGGTCGT	1	8	2	1
tsrna-25397	CTCTTAATCCCAGGGTCGTGGG	0	1	0	0
tsrna-25396	CTCTTAATCCCAGGGTCGTGG	0	1	0	0
tsrna-25395	CTCTTAATCCCAGGGTCGTG	1	0	0	0
tsrna-25394	CTCTTAATCCCAGGGTCGT	0	0	1	0

tsrna-25389	CTCTGTGGCGCAATGGATAGCGCATTGGA	4	9	1	0
tsrna-25388	CTCTGTGGCGCAATGGATAGCGCATTGG	4	7	2	0
tsrna-25387	CTCTGTGGCGCAATGGATAGCGCATTG	0	0	0	0
tsrna-25386	CTCTGTGGCGCAATGGATAGCGCATT	0	2	0	0
tsrna-25379	CTCTGTGGCGCAATGGACGAGCGC	0	0	0	0
tsrna-25371	CTCTGTGGCGCAATGG	0	0	0	0
tsrna-25370	CTCTGTGGCGCAATCGGTTAGCGCATTCGG	0	2	0	0
tsrna-25361	CTCTGGACTTTGAATCCAGCGATCCGAG	2	2	1	0
tsrna-25360	CTCTGGACTTTGAATCCAGCGATCCG	1	3	0	0
tsrna-25359	CTCTGGACTTTGAATCCAGCG	1	2	0	0
tsrna-25358	CTCTGGACTTTGAATCCAGC	0	1	0	0
tsrna-25357	CTCTGGACTTTGAATCCAG	0	1	0	0
tsrna-25353	CTCTGGACTCTGAATCCGGTA	0	1	0	0
tsrna-25351	CTCTGGACTCTGAATCCAGCGATCCGAGTT	1	0	0	1
tsrna-25350	CTCTGGACTCTGAATCCAGCGATCCGAGT	0	1	0	0
tsrna-25349	CTCTGGACTCTGAATCCAGCGATCCGAG	0	1	1	1
tsrna-25348	CTCTGGACTCTGAATCCAGCGATCCGA	0	3	0	0
tsrna-25347	CTCTGGACTCTGAATCCAGCGATCCG	1	1	0	0
tsrna-25346	CTCTGGACTCTGAATCCAGCGATCC	1	1	0	0
tsrna-25345	CTCTGGACTCTGAATCCAGCGATC	0	2	0	0
tsrna-25344	CTCTGGACTCTGAATCCAGCGAT	1	2	0	0
tsrna-25343	CTCTGGACTCTGAATCCAGCGA	2	2	0	0
tsrna-25342	CTCTGGACTCTGAATCCAGCG	0	4	0	0
tsrna-25341	CTCTGGACTCTGAATCCAGCC	0	1	0	0
tsrna-25340	CTCTGGACTCTGAATCCAGC	1	3	1	0
tsrna-25339	CTCTGGACTCTGAATCCAG	0	1	1	0
tsrna-25338	CTCTGGACTCTGAATCCA	0	1	0	0
tsrna-25335	CTCTGCGTTGTGGCCGCAGCAACCTCGGTT	1	0	0	0
tsrna-25332	CTCTGCGTTGTGGCCGCAGCAACC	0	0	1	0
tsrna-25324	CTCTGCACGCGTGGGTTCG	1	1	0	0
tsrna-25319	CTCTGAATCCAGCGATCCGAGTTCGAGTCT	0	1	0	0
tsrna-25318	CTCTGAATCCAGCGATCCGAGTTCAAA	0	2	0	0
tsrna-25317	CTCTGAATCCAGCGATCCGAGTTCAA	1	1	0	0
tsrna-25316	CTCTGAATCCAGCGATCCGAGTTCA	1	0	0	0
tsrna-25314	CTCTGAATCCAGCGATCCGAGTT	0	1	0	0
tsrna-25313	CTCTGAATCCAGCGATCCGAGT	0	1	0	0
tsrna-25312	CTCTGAATCCAGCGATCCGAG	0	1	0	0
tsrna-25310	CTCTGAATCCAGCGATCCG	1	0	0	0
tsrna-25309	CTCTGAATCCAGCGATCC	1	1	0	0
tsrna-25308	CTCTGAATCCAGCGATC	0	1	0	0
tsrna-25306	CTCTCACCGCCGCGGCCCGGGTTCGATTCCCGGTCAGGGAA	30	309	23	8
tsrna-25305	CTCTCACCGCCGCGGCCCGGGTTCGATTCC	0	0	0	0
tsrna-25304	CTCTCACCGCCGCGGCCCGGGTTCGA	0	0	0	1
tsrna-25302	CTCTCACCGCCGCGGCCCGGGTTC	0	1	0	1
tsrna-25301	CTCTCACCGCCGCGGCCCGGGTT	0	1	0	1
tsrna-25298	CTCTCACCGCCGCGGCCCGG	0	0	0	1
tsrna-25296	CTCTCACCGCCGCGGCCC	1	0	0	0
tsrna-25292	CTCGTTGGTCTAGGGGTATGATTCTCGGTT	5	2	3	2
tsrna-25291	CTCGTTGGTCTAGGGGTATGATTCTCGG	3	1	3	0
tsrna-25290	CTCGTTGGTCTAGGGGTATGATTCTCGCTT	29	35	18	25
tsrna-25289	CTCGTTGGTCTAGGGGTATGATTCTCGCT	23	19	10	20
tsrna-25288	CTCGTTGGTCTAGGGGTATGATTCTCGC	12	5	8	12
tsrna-25287	CTCGTTGGTCTAGGGGTATGATTCTCG	1	0	0	0
tsrna-25286	CTCGTTGGTCTAGGGGTATGATTCTC	1	1	1	0
tsrna-25285	CTCGTTGGTCTAGGGGTATGATTCT	1	0	0	0
tsrna-25284	CTCGTTGGTCTAGGGGTATGATTC	0	0	0	0
tsrna-25283	CTCGTTGGTCTAGGGGTATGATT	0	0	0	1
tsrna-25273	CTCGTTAGTATAGTGGTGAGTATCCCCGCCTGT	1	6	3	1
tsrna-25272	CTCGTTAGTATAGTGGTGAGTATCCCCGCCT	2	5	1	2
tsrna-25271	CTCGTTAGTATAGTGGTGAGTATCCCCGCC	3	4	1	0
tsrna-25270	CTCGTTAGTATAGTGGTGAGTATCCCCGC	1	4	1	1
tsrna-25269	CTCGTTAGTATAGTGGTGAGTATCCCCG	0	2	0	1
tsrna-25268	CTCGTTAGTATAGTGGTGAGTATCCCC	0	1	0	0
tsrna-25267	CTCGTTAGTATAGTGGTGAGTATCCC	0	1	0	0

tsrna-25243	CTCGGTTCGAATCCGAGTCACGGCACCA	2	14	0	0
tsrna-25242	CTCGGTTCGAATCCGAGTCACGGCAC	0	1	0	0
tsrna-25241	CTCGGTTCGAATCCGAGTCACGGCA	1	1	0	0
tsrna-25235	CTCGGTGGGACCTCCA	3	6	1	3
tsrna-25234	CTCGGTGGGACCCCCA	0	0	1	0
tsrna-25233	CTCGGTGGAACCTCCA	6	17	3	3
tsrna-25232	CTCGCTTTGGGTGCGAGAGGTCCCGG	0	1	0	0
tsrna-25231	CTCGCTTTGGGTGCGAGAGGTCCCG	0	2	0	0
tsrna-25228	CTCGCTGGGGCCTCCA	0	1	0	2
tsrna-25227	CTCGCCTGCCACGCGGGAGGCCCGGGTTCG	5	29	2	3
tsrna-25226	CTCGCCTGCCACGCGGGAGGCCCGGGT	7	24	6	0
tsrna-25225	CTCGCCTGCCACGCGGGAGGCCCGGG	0	1	0	0
tsrna-25224	CTCGCCTGCCACGCGGGAGGCCCGG	0	2	0	1
tsrna-25222	CTCGCCTGCCACGCGGGAGGCCC	0	1	0	0
tsrna-25214	CTCCTGGCTGGCTCGCCA	11	54	12	4
tsrna-25211	CTCCTGGCTGGCTCACCA	3	4	2	0
tsrna-25209	CTCCTCAAAGCAATACACTGAAAATGTTT	2	1	2	1
tsrna-25203	CTCCTAAGCCAGGGATTGTGGGT	136	448	90	47
tsrna-25202	CTCCTAAGCCAGGGATTGTGGG	2	5	1	2
tsrna-25201	CTCCTAAGCCAGGGATTGTGG	1	1	0	0
tsrna-25200	CTCCTAAGCCAGGGATTGTG	1	0	0	0
tsrna-25199	CTCCTAAGCCAGGGATTGT	0	0	1	0
tsrna-25196	CTCCGGATGGAGGCGTGGG	0	7	1	0
tsrna-25195	CTCCGGATGGAGGCGTGG	0	4	1	0
tsrna-25194	CTCCGGATGGAGGCGTG	1	3	0	0
tsrna-25193	CTCCGGAGCTGGGGATTGTGGGTTC	0	3	0	1
tsrna-25192	CTCCGGAGCTGGGGATTGTGGGT	1	1	0	0
tsrna-25190	CTCCGGAGCTGGGGATTGTGG	0	0	0	0
tsrna-25188	CTCCGGAGCTGGGGATTGT	0	0	1	0
tsrna-25187	CTCCGAGGTGATTTTCATATTGAATTGCA	0	0	0	1
tsrna-25186	CTCCGAGGTGATTTTCATATTGAATTGC	1	0	0	0
tsrna-25185	CTCCGAGGTGATTTTCATATTGAATTG	0	0	0	0
tsrna-25178	CTCCGAGGTGATTTTCATAT	0	0	0	0
tsrna-25177	CTCCGAGGTGATTTTCATA	1	0	0	0
tsrna-25173	CTCCCTGGTGGTCTAGTGGTTAGGATTCGGCGCTCTC	640	305	703	107
tsrna-25172	CTCCCTGGTGGTCTAGTGGTTAGGATTCGGCGCTC	149	104	118	45
tsrna-25171	CTCCCTGGTGGTCTAGTGGTTAGGATTCGGCGCT	44	81	44	37
tsrna-25170	CTCCCTGGTGGTCTAGTGGTTAGGATTCGGCGC	37	61	32	35
tsrna-25169	CTCCCTGGTGGTCTAGTGGTTAGGATTCGGCG	42	39	37	30
tsrna-25168	CTCCCTGGTGGTCTAGTGGTTAGGATTCGGC	37	45	31	23
tsrna-25167	CTCCCTGGTGGTCTAGTGGTTAGGATTCGG	14	40	28	25
tsrna-25166	CTCCCTGGTGGTCTAGTGGTTAGG	19	28	22	23
tsrna-25165	CTCCCGGTGTGGGAACCA	0	4	1	1
tsrna-25162	CTCCCGGTATGGGAACCA	0	1	0	0
tsrna-25157	CTCCCAGTGGGGCCTCCA	0	2	0	0
tsrna-25155	CTCCCACGCGGGAGACCCGG	1	0	0	0
tsrna-25153	CTCCCACATGGTCTAGCGGTTAGGATTCCT	9	9	7	17
tsrna-25148	CTCCAGTCTCTTCGGGGGCGTGGGTTCGAATCCCACCGCTG(	7	30	1	1
tsrna-25147	CTCCAGTCTCTTCGGGGGCGTGGGTTCGA	1	1	1	1
tsrna-25146	CTCCAGTCTCTTCGGGGGCGTGGGTTC	0	3	0	0
tsrna-25145	CTCCAGTCTCTTCGGGGGCGTGGGT	0	1	0	0
tsrna-25144	CTCCAGTCTCTTCGGGGGCGTGGG	0	0	0	0
tsrna-25140	CTCCAGTCTCTTCGGGGGCG	0	1	0	0
tsrna-25131	CTCCAGTCATTTCGATGGCGTGGGTTCGAATCCCACCGCTGC	6	18	1	1
tsrna-25129	CTCCAGATCAGAAGGTTGCGTGTTCA	0	4	1	0
tsrna-25128	CTCCAGATCAGAAGGTTGCGTGTTC	1	2	0	0
tsrna-25127	CTCCAGATCAGAAGGTTGCGTGTT	3	3	3	0
tsrna-25125	CTCCAGATCAGAAGGTTGCGTG	1	0	0	0
tsrna-25118	CTCCAGATCAGAAGGCTGCGTGTTCGAA	0	0	0	0
tsrna-25117	CTCCAGATCAGAAGGCTGCGTGTTCGA	1	0	1	0
tsrna-25116	CTCCAGATCAGAAGGCTGCGTGTTCG	0	0	1	0
tsrna-25115	CTCCAGATCAGAAGGCTGCGTGTTC	0	0	1	1
tsrna-25114	CTCCAGATCAGAAGGCTGCGTGTT	1	1	0	0
tsrna-25113	CTCCAGATCAGAAGGCTGCGTGT	0	1	0	0

tsrna-25105	CTCCAATGGAGGCGTGGGT	10	26	10	0
tsrna-25104	CTCCAAATAAAAGTACCA	1	0	0	0
tsrna-25101	CTCATTAAATTATGATAATCATATTTACC	0	0	1	0
tsrna-25100	CTCATTAAATTATGATAATCATATTTA	0	0	1	0
tsrna-25099	CTCATGCCCCCATGTCTAACAACATGGCTTTCTCACCA	12	7	22	0
tsrna-25098	CTCATGCCCCCATGTCTAACAACATGGCTTTCTCACC	2	1	2	1
tsrna-25097	CTCATGCCCCCATGTCTAACAACATGGCTTTCTCAC	2	0	1	0
tsrna-25096	CTCATGCCCCCATGTCTAACAACATGGCTT	0	0	1	0
tsrna-25094	CTCATGCCCCCATGTCTAACAACATGGC	0	0	0	0
tsrna-25093	CTCATGCCCCCATGTCTAACAACATGG	0	0	1	0
tsrna-25082	CTCATAATCTGAAGGTCGTGAGTTCG	31	45	22	6
tsrna-25081	CTCATAATCTGAAGGTCGTGAGTT	21	44	18	8
tsrna-25080	CTCATAATCTGAAGGTCGTGAGT	21	34	21	6
tsrna-25079	CTCATAATCTGAAGGTCGTGAG	3	0	3	1
tsrna-25078	CTCATAATCTGAAGGTCGTGA	3	4	3	1
tsrna-25077	CTCATAATCTGAAGGTCGTG	1	8	1	0
tsrna-25076	CTCATAATCTGAAGGTCGT	1	0	1	0
tsrna-25075	CTCATAATCTGAAGGTCCTGAGTTCGAACCTCAGAGGGGGCA	143	366	88	67
tsrna-25074	CTCATAATCTGAAGGTCCTGAGT	116	301	105	52
tsrna-25073	CTCATAATCTGAAGGTCCTGAG	8	23	5	3
tsrna-25072	CTCATAATCTGAAGGTCCTGA	6	28	7	1
tsrna-25071	CTCATAATCTGAAGGTCCT	0	6	1	0
tsrna-25070	CTCATAATCTGAAGGTCC	2	3	0	0
tsrna-25067	CTCAGTTGGGAGAGCGTTAGACTGA	0	2	0	1
tsrna-25064	CTCAGTGGTAGAGCATTTGACTGCA	6	4	1	1
tsrna-25063	CTCAGTGGTAGAGCATTTGACTGC	4	3	1	2
tsrna-25062	CTCAGTGGTAGAGCATTTGACTG	0	1	1	0
tsrna-25061	CTCAGTGGTAGAGCATTTGACT	2	1	0	0
tsrna-25060	CTCAGTGGTAGAGCATTTGAC	2	0	0	0
tsrna-25059	CTCAGTGGTAGAGCATTTGA	1	0	1	0
tsrna-25058	CTCAGTGGTAGAGCATTTG	1	1	1	0
tsrna-25055	GGTTCAAATCCGGGTGCCCCCTCCA	155	197	66	21
tsrna-25054	GGTTCAAATCCGGGTGCCCCCTCC	1	5	2	0
tsrna-25053	GGTTCAAATCCGGGTGCCCCCTC	0	1	0	0
tsrna-25048	GGTTCAAATCCCGGACGAGCCCCCA	131	204	57	53
tsrna-25046	GGTTCAAATCCCGGACGA	0	1	1	0
tsrna-25043	GGTTATCACGTTCGCCTCACACG	0	0	1	0
tsrna-25034	GGTTATCACGTCTGCTTTACA	0	0	1	0
tsrna-25033	GGTTATCACGTCTGCTTTAC	1	0	0	0
tsrna-25030	GGTTATCACGTCTGCTT	0	0	0	0
tsrna-25021	GGTTATACCCTTCCCGTACTACCA	1	3	0	0
tsrna-25019	GGTTATACCCTTCCCGTACTAC	0	0	0	0
tsrna-25018	GGTTATACCCTTCCCGTACTA	0	0	1	0
tsrna-25017	GGTTATACCCTTCCCGTACT	1	0	0	0
tsrna-25012	GGTTAGTATCCCCGCCTGTCACGCGGGAG	14	58	10	3
tsrna-25011	GGTTAGTATCCCCGCCTGTCAC	0	1	0	0
tsrna-25010	GGTTAGTATCCCCGCCTGTC	0	0	0	1
tsrna-25007	GGTTAGTATCCCCGCCT	0	1	0	0
tsrna-25005	GGTTAGTACTCTGCGTTGTGGCCGCAGCAACCT	4	3	0	0
tsrna-25004	GGTTAGTACTCTGCGTTGTGGCCGCAGC	3	3	0	0
tsrna-25003	GGTTAGTACTCTGCGTTGTGGCCGC	5	0	1	0
tsrna-25002	GGTTAGTACTCTGCGTTGTGGCCG	5	1	0	0
tsrna-25001	GGTTAGTACTCTGCGTTGTGGCC	3	3	1	1
tsrna-25000	GGTTAGTACTCTGCGTTGTGGC	2	0	1	1
tsrna-24999	GGTTAGTACTCTGCGTTGTGG	2	0	2	0
tsrna-24998	GGTTAGTACTCTGCGTTGTG	3	1	1	0
tsrna-24997	GGTTAGTACTCTGCGTTGT	2	0	0	2
tsrna-24996	GGTTAGTACTCTGCGTTG	0	1	1	0
tsrna-24995	GGTTAGTACTCTGCGTT	0	0	2	0
tsrna-24993	GGTTAGTACTCTGCGCTGT	0	1	0	0
tsrna-24992	GGTTAGTACTCTGCGCTG	3	0	0	0
tsrna-24989	GGTTAGGCCTCTTTTTACCACCA	2	2	5	2
tsrna-24988	GGTTAGGCCTCTTTTTACCACC	1	0	1	0
tsrna-24987	GGTTAGGCCTCTTTTTACCAC	0	1	0	0

tsrna-24986	GGTTAGGCCTCTTTTTACCA	0	0	1	0
tsrna-24985	GGTTAGGCCTCTTTTTACC	0	0	1	0
tsrna-24983	GGTTAGGCCTCTTTTTA	0	0	0	0
tsrna-24981	GGTTAGGATTCGGCGCTCTCATC	4	0	0	0
tsrna-24980	GGTTAGGATTCGGCGCTCTCAT	0	5	1	2
tsrna-24979	GGTTAGGATTCGGCGCTCTCACCGCCGCGGCCCGGGTT	6	7	5	3
tsrna-24978	GGTTAGGATTCGGCGCTCTCACCGCCGCGGCCCGGG	6	14	3	2
tsrna-24977	GGTTAGGATTCGGCGCTCTCACCGCCGCGGCCC	9	21	3	4
tsrna-24976	GGTTAGGATTCGGCGCTCTCACCGCCGCGG	4	19	2	2
tsrna-24975	GGTTAGGATTCGGCGCTCTCACCGCC	5	11	6	1
tsrna-24974	GGTTAGGATTCGGCGCTCTCACCGC	0	5	1	0
tsrna-24973	GGTTAGGATTCGGCGCTCTCACCG	3	2	2	1
tsrna-24972	GGTTAGGATTCGGCGCTCTCACC	0	4	2	0
tsrna-24971	GGTTAGGATTCGGCGCTCTCAC	1	4	2	0
tsrna-24970	GGTTAGGATTCGGCGCTCTCA	0	1	0	0
tsrna-24969	GGTTAGGATTCGGCGCTCTC	2	2	0	2
tsrna-24968	GGTTAGGATTCGGCGCTCT	1	1	2	0
tsrna-24967	GGTTAGGATTCGGCGCTC	1	3	0	1
tsrna-24966	GGTTAGGATTCGGCGCT	2	2	0	1
tsrna-24965	GGTTAGGATTCGGCGC	1	0	0	0
tsrna-24964	GGTTAGGATTCCTGGTTTTCACC	4	4	6	9
tsrna-24963	GGTTAGGATTCCTGGTTTTCAC	9	0	0	3
tsrna-24962	GGTTAGGATTCCTGGTTTTC	0	0	0	0
tsrna-24960	GGTTAGGATTCCTGGTTT	0	1	0	0
tsrna-24959	GGTTAGGATTCCTGGTT	0	0	0	0
tsrna-24955	GGTTAGCTCAGTTGGTTAGA	0	0	1	0
tsrna-24946	GGTTAGCGCGTTCGGCTGTTAA	0	1	0	0
tsrna-24941	GGTTAGCGCGCGGTACTTATA	0	1	0	0
tsrna-24940	GGTTAGCGCGCGGTACTTA	3	0	0	0
tsrna-24937	GGTTAGCATAGCTGCCTTCCAAGC	0	1	0	0
tsrna-24936	GGTTAGCATAGCTGCCTTCCAAG	0	0	0	0
tsrna-24930	GGTTAGCACTCTGGACTTTGAATCCAGCGATCCGAGTT	7	18	3	3
tsrna-24929	GGTTAGCACTCTGGACTTTGAATCCAGCGATCCGAGT	1	4	2	1
tsrna-24928	GGTTAGCACTCTGGACTTTGAATCCAGCGATCCGAG	5	4	2	0
tsrna-24927	GGTTAGCACTCTGGACTTTGAATCCAGCAATCCGAG	5	3	3	1
tsrna-24926	GGTTAGCACTCTGGACTTTGAATCCAGC	2	1	2	5
tsrna-24925	GGTTAGCACTCTGGACTTTGAATCC	2	11	3	2
tsrna-24924	GGTTAGCACTCTGGACTTTGAATC	0	5	0	0
tsrna-24922	GGTTAGCACTCTGGACTTTGAA	0	1	0	0
tsrna-24918	GGTTAGCACTCTGGACTT	0	0	0	0
tsrna-24917	GGTTAGCACTCTGGACTCTGAATCCAGCGATCCGAGTTC	5	7	2	0
tsrna-24916	GGTTAGCACTCTGGACTCTGAATCCAGCGATCCGAGTT	0	1	2	1
tsrna-24915	GGTTAGCACTCTGGACTCTGAATCCAGCGATCCGAGT	4	5	1	0
tsrna-24914	GGTTAGCACTCTGGACTCTGAATCCAGCGATCCGAG	5	7	1	2
tsrna-24913	GGTTAGCACTCTGGACTCTGAATCCAGCGATCCG	1	4	3	0
tsrna-24912	GGTTAGCACTCTGGACTCTGAATCCAGCGATCC	2	7	2	2
tsrna-24911	GGTTAGCACTCTGGACTCTGAATCCAGCGATC	2	4	2	0
tsrna-24910	GGTTAGCACTCTGGACTCTGAATCCAGCG	0	5	1	2
tsrna-24909	GGTTAGCACTCTGGACTCTGAATCCAGC	3	3	4	0
tsrna-24908	GGTTAGCACTCTGGACTCTGAATCCAG	1	3	1	0
tsrna-24907	GGTTAGCACTCTGGACTCTGAATCCA	1	1	0	0
tsrna-24906	GGTTAGCACTCTGGACTCTGAATCC	1	3	0	0
tsrna-24905	GGTTAGCACTCTGGACTCTGAATC	0	1	0	0
tsrna-24904	GGTTAGCACTCTGGACTCTGAAT	0	0	0	0
tsrna-24903	GGTTAGCACTCTGGACTCTGAA	0	0	1	0
tsrna-24897	GGTTAGCACTCTGGAC	0	0	0	0
tsrna-24884	GGTTAAGGCGTTGGACT	0	0	0	0
tsrna-24882	GGTTAAGGCGATGGACTAGAAA	0	1	0	0
tsrna-24879	GGTTAAATACAGACCAAGAGCCTTCA	9	5	13	5
tsrna-24878	GGTTAAATACAGACCAAGAGCC	1	0	1	1
tsrna-24876	GGTTAAATACAGACCAAGAG	0	0	1	1
tsrna-24875	GGTTAAATACAGACCAAGA	0	0	1	0
tsrna-24872	GGTGTGAGAGGTCCCGGGT	1	0	0	0
tsrna-24870	GGTGTAGTGGTATCATGCAAGATTCCCA	0	0	0	0

tsrna-24869	GGTGTAGTGGTATCATGCAAGATTCCC	1	0	0	0
tsrna-24867	GGTGTAGTGGTATCATGCAAGATTC	0	0	0	0
tsrna-24865	GGTGTAGTGGTATCATGCAAGAT	0	0	1	0
tsrna-24861	GGTGTAGTGGTATCATGCA	1	0	0	0
tsrna-24859	GGTGTAGTGGTATCATG	1	0	0	0
tsrna-24858	GGTGTAGCTCAGTGGTAGAGCGCGTGC	6	14	2	1
tsrna-24857	GGTGTAGCTCAGTGGTAGAGCGCG	0	0	1	0
tsrna-24855	GGTGTAGCTCAGTGGTAGAGCATTTGACT	3	1	0	0
tsrna-24854	GGTGTAGCTCAGTGGTAGAGC	1	0	0	0
tsrna-24848	GGTGTAATGGTTAGCACTCTGGGCT	0	1	0	0
tsrna-24847	GGTGTAATGGTTAGCACTCTGGACTTTGA	0	0	1	0
tsrna-24846	GGTGTAATGGTTAGCACTCTGGACTTTG	2	1	0	1
tsrna-24845	GGTGTAATGGTTAGCACTCTGGACTTT	1	0	2	1
tsrna-24844	GGTGTAATGGTTAGCACTCTGGACTT	1	0	0	0
tsrna-24843	GGTGTAATGGTTAGCACTCTGGACTCTGAATCCAGC	7	6	9	7
tsrna-24842	GGTGTAATGGTTAGCACTCTGGACTCTGA	2	0	1	2
tsrna-24841	GGTGTAATGGTTAGCACTCTGGACTCTG	1	1	0	1
tsrna-24840	GGTGTAATGGTTAGCACTCTGGACTCT	0	2	1	2
tsrna-24839	GGTGTAATGGTTAGCACTCTGGACTC	1	0	1	0
tsrna-24838	GGTGTAATGGTTAGCACTCTGGACT	0	0	1	0
tsrna-24837	GGTGTAATGGTTAGCACTCTGGAC	0	0	0	1
tsrna-24836	GGTGTAATGGTTAGCACTCTGGA	0	1	0	2
tsrna-24835	GGTGTAATGGTTAGCACTCTGG	0	1	0	0
tsrna-24832	GGTGTAATGGTGAGCACTCTGGACTC	1	0	1	0
tsrna-24830	GGTGTAATGGTGAGCACTCTGGAC	0	1	0	0
tsrna-24829	GGTGTAATGGTGAGCACTCTGGA	0	0	0	0
tsrna-24828	GGTGTAATGGTGAGCACTCTGG	1	0	0	0
tsrna-24826	GGTGTAATGGTCAGCACTCTGGACT	1	0	0	0
tsrna-24824	GGTGTAATGGTAAGCACTCTGGACTC	0	0	0	0
tsrna-24821	GGTGGTTTAGTGGTAGAATTCTCGCCT	1	5	0	0
tsrna-24820	GGTGGTTTAGTGGTAGAATTCTCGCC	5	4	2	1
tsrna-24819	GGTGGTTTAGTGGTAGAATTCTCGC	1	0	0	0
tsrna-24818	GGTGGTTTAGTGGTAGAATTCTCG	1	0	0	0
tsrna-24817	GGTGGTTTAGTGGTAGAATTCTC	0	0	0	1
tsrna-24815	GGTGGTTTAGTGGTAGAAT	0	1	0	0
tsrna-24813	GGTGGTTCAGTGGTAGAATTCTT	2	2	1	1
tsrna-24812	GGTGGTTCAGTGGTAGAATTCTCGCCTTTC	11	8	5	2
tsrna-24811	GGTGGTTCAGTGGTAGAATTCTCGCCTGCC	21	34	9	6
tsrna-24810	GGTGGTTCAGTGGTAGAATTCTCGCCTGC	15	11	4	1
tsrna-24809	GGTGGTTCAGTGGTAGAATTCTCGCCTG	11	11	7	3
tsrna-24808	GGTGGTTCAGTGGTAGAATTCTCGCCTCC	8	10	5	9
tsrna-24807	GGTGGTTCAGTGGTAGAATTCTCGCCTC	6	9	4	4
tsrna-24806	GGTGGTTCAGTGGTAGAATTCTCGCCT	1	10	3	7
tsrna-24805	GGTGGTTCAGTGGTAGAATTCTCGCC	4	6	3	2
tsrna-24804	GGTGGTTCAGTGGTAGAATTCTCGC	2	3	1	2
tsrna-24803	GGTGGTTCAGTGGTAGAATTCTCG	2	0	0	1
tsrna-24802	GGTGGTTCAGTGGTAGAATTCTC	0	5	1	0
tsrna-24801	GGTGGTTCAGTGGTAGAATTCT	2	2	1	0
tsrna-24800	GGTGGTTCAGTGGTAGAATTC	3	1	1	0
tsrna-24799	GGTGGTTCAGTGGTAGAATT	2	3	1	0
tsrna-24798	GGTGGTTCAGTGGTAGAAT	4	2	1	1
tsrna-24797	GGTGGTTCAGTGGTAGAA	2	1	1	1
tsrna-24796	GGTGGTTCAGTGGTAGA	0	1	0	0
tsrna-24794	GGTGGTTCAATGGTAGAATTCTCGCCT	1	0	1	0
tsrna-24793	GGTGGTCTAGTGGTTAGGATTCGGCGCTCT	39	43	20	16
tsrna-24792	GGTGGTCTAGTGGTTAGGATTCGGCGCTC	35	38	22	11
tsrna-24791	GGTGGTCTAGTGGTTAGGATTCGGCGCT	33	37	23	19
tsrna-24790	GGTGGTCTAGTGGTTAGGATTCGGCGC	21	23	8	7
tsrna-24789	GGTGGTCTAGTGGTTAGGATTCGGCG	15	9	7	8
tsrna-24788	GGTGGTCTAGTGGTTAGGATTCGGC	8	4	5	7
tsrna-24787	GGTGGTCTAGTGGTTAGGATTCGG	4	0	1	0
tsrna-24786	GGTGGTCTAGTGGTTAGGATTCG	4	2	0	1
tsrna-24785	GGTGGTCTAGTGGTTAGGATTC	1	0	1	1
tsrna-24784	GGTGGTCTAGTGGTTAGGATT	2	3	2	0

tsrna-24783	GGTGGTCTAGTGGTTAGGAT	1	0	1	0
tsrna-24778	GGTGGTCTAGTGGCTAGGATTCGGCGCT	15	40	6	29
tsrna-24777	GGTGGTCTAGTGGCTAGGATTCGGCGC	12	33	9	39
tsrna-24776	GGTGGTCTAGTGGCTAGGATTCGGCG	7	30	7	31
tsrna-24775	GGTGGTCTAGTGGCTAGGATTCGGC	10	27	3	10
tsrna-24774	GGTGGTCTAGTGGCTAGGATTCGG	1	3	0	1
tsrna-24773	GGTGGTCTAGTGGCTAGGATTCG	3	1	0	0
tsrna-24772	GGTGGTCTAGTGGCTAGGATT	2	3	0	0
tsrna-24771	GGTGGTCTAGTGGCTAGGAT	1	0	1	2
tsrna-24770	GGTGGTCTAGTGGCTAGGA	0	0	1	0
tsrna-24766	GGTGGTATAGTGGTTAGCATAGCTGCCT	0	0	0	1
tsrna-24764	GGTGGTATAGTGGTTAGCATAGCTGC	1	0	0	0
tsrna-24763	GGTGGTATAGTGGTTAGCATAGCTG	0	0	0	0
tsrna-24755	GGTGGTATAGTGGTGAGCATAGCTGCCTTC	1	1	0	0
tsrna-24754	GGTGGTATAGTGGTGAGCATAGCTGCCTT	1	0	0	0
tsrna-24753	GGTGGTATAGTGGTGAGCATAGCTGCCT	1	1	0	0
tsrna-24752	GGTGGTATAGTGGTGAGCATAGCTGCC	1	1	1	0
tsrna-24751	GGTGGTATAGTGGTGAGCATAGCTGC	1	1	0	0
tsrna-24750	GGTGGTATAGTGGTGAGCATAGCTG	2	0	0	0
tsrna-24749	GGTGGTATAGTGGTGAGCATAGCT	3	0	0	0
tsrna-24748	GGTGGTATAGTGGTGAGCATAGC	0	1	0	0
tsrna-24746	GGTGGTATAGTGGTGAGCATA	0	0	0	0
tsrna-24744	GGTGGTATAGTGGTGAGCA	0	1	0	0
tsrna-24741	GGTGGTATAGTGGTAAGCATAGCTGCC	0	1	0	0
tsrna-24740	GGTGGTATAGTGGTAAGCATAGCTG	2	0	0	0
tsrna-24739	GGTGGTATAGTGGTAAGCATAGC	1	0	0	0
tsrna-24733	GGTGGTAGAGCATTTGACTGC	1	1	0	1
tsrna-24732	GGTGGTAGAGCATTTGACTG	0	0	0	1
tsrna-24730	GGTGGTAGAGCATTTGA	0	1	0	0
tsrna-24722	GGTGGCCCGGGTTCGACTCCCGGTATGGG	0	2	0	0
tsrna-24721	GGTGGCCCGGGTTCGACTCCCGGTATGG	0	1	0	0
tsrna-24716	GGTGGCACGGAGAATTTTG	0	0	1	0
tsrna-24715	GGTGGAGTTAAAGACTTTTTCTCTGACCA	26	36	18	17
tsrna-24714	GGTGGAGTTAAAGACTTTTTCTCTGACC	3	11	4	3
tsrna-24713	GGTGGAGTTAAAGACTTTTTCTCTGAC	1	5	0	0
tsrna-24712	GGTGGAGTTAAAGACTTTTTCTCTGA	1	4	2	0
tsrna-24711	GGTGGAGTTAAAGACTTTTTCTCTG	0	2	0	0
tsrna-24710	GGTGGAGTTAAAGACTTTTTCTCT	0	2	0	0
tsrna-24709	GGTGGAGTTAAAGACTTTTTCTC	0	2	0	0
tsrna-24706	GGTGGAGTTAAAGACTTTTT	0	2	0	0
tsrna-24705	GGTGGAGTTAAAGACTTTT	0	1	0	0
tsrna-24704	GGTGGAGTTAAAGACTTT	0	0	1	0
tsrna-24700	GGTGCTTAGCTGTTAACT	0	0	1	0
tsrna-24698	GGTGCTAATGGTGGAGTTAAAGACTTTTTCTCTGACCA	35	50	29	18
tsrna-24697	GGTGCTAATGGTGGAGTTAAAGACTTTTTCTCTGACC	5	32	5	0
tsrna-24696	GGTGCTAATGGTGGAGTTAAAGACTTTTTCTCTGAC	11	21	1	2
tsrna-24695	GGTGCTAATGGTGGAGTTAAAGACTTTTTC	4	8	3	0
tsrna-24694	GGTGCTAATGGTGGAGTTAAAGACT	3	6	2	1
tsrna-24693	GGTGCTAATGGTGGAGTTAAAGAC	0	0	2	1
tsrna-24692	GGTGCTAATGGTGGAGTTAAAGA	2	3	1	0
tsrna-24691	GGTGCTAATGGTGGAGTTAAAG	0	3	1	0
tsrna-24690	GGTGCTAATGGTGGAGTTAAA	2	3	2	0
tsrna-24689	GGTGCTAATGGTGGAGTTAA	2	2	0	0
tsrna-24688	GGTGCTAATGGTGGAGTTA	0	2	1	0
tsrna-24687	GGTGCTAATGGTGGAGTT	0	1	0	0
tsrna-24684	GGTGCGAGAGGTCCCGGGTTCAAATCCCGG	0	3	1	0
tsrna-24683	GGTGCGAGAGGTCCCGGGTTCAAATCCCG	1	4	0	1
tsrna-24682	GGTGCGAGAGGTCCCGGGTTCAAA	0	5	1	0
tsrna-24681	GGTGCGAGAGGTCCCGGGTTCAA	0	5	0	0
tsrna-24680	GGTGCGAGAGGTCCCGGGTTCA	0	2	0	0
tsrna-24679	GGTGCGAGAGGTCCCGGGTTC	1	3	0	0
tsrna-24678	GGTGCGAGAGGTCCCGGGTT	0	4	0	0
tsrna-24677	GGTGCGAGAGGTCCCGGGT	0	2	1	0
tsrna-24673	GGTGCAGTGGTAGAATTCTCGCCT	6	3	6	0

tsrna-24672	GGTGCAGTGGTAGAATTCTCGCC	1	1	1	1
tsrna-24671	GGTGCAGTGGTAGAATTCTCGC	0	4	0	0
tsrna-24665	GGTGCAACTCCAAATAAAAGTAC	0	0	0	0
tsrna-24659	GGTGAGTATCCCCGCCTGTCACGCGGGAG	15	44	10	4
tsrna-24658	GGTGAGTATCCCCGCCTGTCACGCG	2	8	0	0
tsrna-24657	GGTGAGTATCCCCGCCTGTCACGC	3	3	0	1
tsrna-24656	GGTGAGTATCCCCGCCTGTCACG	1	4	1	2
tsrna-24655	GGTGAGTATCCCCGCCTGTCAC	4	4	1	1
tsrna-24654	GGTGAGTATCCCCGCCTGTCA	1	2	1	1
tsrna-24653	GGTGAGTATCCCCGCCTGTC	4	11	0	1
tsrna-24652	GGTGAGTATCCCCGCCTGT	0	3	0	0
tsrna-24651	GGTGAGTATCCCCGCCTG	0	2	0	0
tsrna-24650	GGTGAGTATCCCCGCCT	0	0	0	2
tsrna-24649	GGTGAGTATCCCCGCC	0	2	0	0
tsrna-24647	GGTGAGCATAGCTGCCTTCCAAG	0	1	1	0
tsrna-24645	GGTGAGCATAGCTGCCTTC	0	0	1	0
tsrna-24642	GGTGAGCACTCTGGACTCTGAATCCAGCGATCCGAG	1	2	0	1
tsrna-24641	GGTGAGCACTCTGGACTCTGAATCCAGCG	2	4	0	1
tsrna-24640	GGTGAGCACTCTGGACTCTGAATCCAGC	0	6	3	2
tsrna-24639	GGTGAGCACTCTGGACTCTGAATCCA	0	2	1	0
tsrna-24638	GGTGAGCACTCTGGACTCTGAATCC	0	2	1	1
tsrna-24637	GGTGAGCACTCTGGACTCTGAATC	0	0	0	0
tsrna-24628	GGTCTCTGTGGCGCAATGGAC	0	0	0	0
tsrna-24627	GGTCTCGTAAACCGAAGATCAC	0	1	1	0
tsrna-24626	GGTCTCCGGATGGAGGCGTGG	1	1	2	0
tsrna-24625	GGTCTCCGGATGGAGGCG	0	1	0	0
tsrna-24623	GGTCTCCAATGGAGGCGTGGGT	17	19	6	0
tsrna-24619	GGTCTAGTGGTTAGGATTCGGCGCTCTCAC	33	53	20	18
tsrna-24618	GGTCTAGTGGTTAGGATTCGGCGCTCTCA	41	54	20	13
tsrna-24617	GGTCTAGTGGTTAGGATTCGGCGCTCTC	48	47	27	12
tsrna-24616	GGTCTAGTGGTTAGGATTCGGCGCTCT	31	46	20	16
tsrna-24615	GGTCTAGTGGTTAGGATTCGGCGCTC	33	40	26	12
tsrna-24614	GGTCTAGTGGTTAGGATTCGGCGCT	38	50	19	15
tsrna-24613	GGTCTAGTGGTTAGGATTCGGCGC	17	12	13	13
tsrna-24612	GGTCTAGTGGTTAGGATTCGGCG	9	11	5	7
tsrna-24611	GGTCTAGTGGTTAGGATTCGGC	5	8	3	3
tsrna-24610	GGTCTAGTGGTTAGGATTCGG	4	2	2	0
tsrna-24609	GGTCTAGTGGTTAGGATTCG	0	2	1	0
tsrna-24608	GGTCTAGTGGTTAGGATTC	3	0	0	0
tsrna-24607	GGTCTAGTGGTTAGGATT	0	2	2	0
tsrna-24606	GGTCTAGTGGTTAGGAT	0	0	0	0
tsrna-24604	GGTCTAGTGGCTAGGATTCGGCGCTTT	8	41	7	37
tsrna-24603	GGTCTAGTGGCTAGGATTCGGCGCTT	16	33	8	41
tsrna-24602	GGTCTAGTGGCTAGGATTCGGCGCT	10	64	3	31
tsrna-24601	GGTCTAGTGGCTAGGATTCGGCGC	10	39	4	38
tsrna-24600	GGTCTAGTGGCTAGGATTCGGCG	8	27	2	22
tsrna-24599	GGTCTAGTGGCTAGGATTCGGC	2	12	5	4
tsrna-24598	GGTCTAGTGGCTAGGATTCGG	0	0	1	0
tsrna-24597	GGTCTAGTGGCTAGGATTCG	1	0	0	0
tsrna-24596	GGTCTAGGGGTATGATTCTCGGTTT	6	0	2	0
tsrna-24595	GGTCTAGGGGTATGATTCTCGGTT	7	0	1	2
tsrna-24594	GGTCTAGGGGTATGATTCTCGGT	3	4	0	3
tsrna-24593	GGTCTAGGGGTATGATTCTCGG	1	0	1	0
tsrna-24592	GGTCTAGGGGTATGATTCTCGCTTT	11	13	10	17
tsrna-24591	GGTCTAGGGGTATGATTCTCGCTTC	21	15	12	10
tsrna-24590	GGTCTAGGGGTATGATTCTCGCTT	17	10	11	11
tsrna-24589	GGTCTAGGGGTATGATTCTCGCT	22	21	10	13
tsrna-24588	GGTCTAGGGGTATGATTCTCGC	10	6	6	9
tsrna-24587	GGTCTAGGGGTATGATTCTCG	0	0	0	0
tsrna-24586	GGTCTAGGGGTATGATTCTC	0	0	0	1
tsrna-24585	GGTCTAGGGGTATGATTCT	0	0	1	0
tsrna-24584	GGTCTAGGGGTATGATTC	0	0	0	0
tsrna-24581	GGTCTAGCGGTTAGGATTCCTGGTTTTC	8	7	4	0
tsrna-24580	GGTCTAGCGGTTAGGATTCCTGGTTTT	3	6	3	4

tsrna-24579	GGTCTAGCGGTTAGGATTCCTGGTTT	4	11	1	0
tsrna-24578	GGTCTAGCGGTTAGGATTCCTGGTT	3	4	5	0
tsrna-24577	GGTCTAGCGGTTAGGATTCCTGGT	2	5	0	1
tsrna-24576	GGTCTAGCGGTTAGGATTCCTGG	5	3	1	3
tsrna-24575	GGTCTAGCGGTTAGGATTCCTG	0	1	0	0
tsrna-24567	GGTCTAAGGCGCTGGATTAAGG	0	0	0	1
tsrna-24566	GGTCTAAGGCGCTGGATTAAG	0	1	0	0
tsrna-24565	GGTCTAAGGCGCTGGATT	0	0	1	0
tsrna-24555	GGTCGTGGTTGTAGTCCGTGCGAGAATACCA	10	36	17	9
tsrna-24554	GGTCGTGGTTGTAGTCCGTGCGAGAATACC	3	6	4	2
tsrna-24553	GGTCGTGGTTGTAGTCCGTGCGAGAATAC	1	1	5	1
tsrna-24552	GGTCGTGGTTGTAGTCCGTGCGAGAATA	2	2	2	0
tsrna-24551	GGTCGTGGTTGTAGTCCGTGCGAGAAT	1	2	4	0
tsrna-24550	GGTCGTGGTTGTAGTCCGTGCGAGAA	1	3	2	0
tsrna-24549	GGTCGTGGTTGTAGTCCGTGCGAGA	2	0	1	0
tsrna-24548	GGTCGTGGTTGTAGTCCGTGCGAG	0	1	0	0
tsrna-24547	GGTCGTGGTTGTAGTCCGTGCGA	1	1	0	0
tsrna-24546	GGTCGTGGTTGTAGTCCGTGCG	0	0	0	0
tsrna-24545	GGTCGTGGTTGTAGTCCGTGC	0	0	0	0
tsrna-24531	GGTCGCTGGTTCGAATCCGGCTCGAAGGAC	1	1	1	0
tsrna-24529	GGTCGCTGGTTCGAATCCGG	0	1	0	0
tsrna-24528	GGTCGCTGGTTCGAATCC	0	0	0	0
tsrna-24527	GGTCGCGGGTTCGATCCCCGTACGGG	0	1	0	0
tsrna-24515	GGTCGCGAGTTCAAATCTCGCTGGGGCCTC	0	0	0	0
tsrna-24512	GGTCGCAGTCTCCCCTGGAGGCGTGGGTTC	3	126	3	0
tsrna-24511	GGTCGCAGTCTCCCCTGGAGGC	0	5	1	0
tsrna-24510	GGTCGCAGTCTCCCCTGGAGG	0	1	0	0
tsrna-24508	GGTCGATTCCCCGACGGGGAGCCA	3	17	0	0
tsrna-24507	GGTCGATTCCCCGACGGGGAGCC	0	1	0	0
tsrna-24501	GGTCGATGGATCGAAACCATCCTCTGCTAC	0	0	0	0
tsrna-24495	GGTCGAAACCGAGCGGAAACACCA	0	1	0	0
tsrna-24489	GGTCCTGGGTTCGAGCCCCAGTGGAACCACC	2	5	3	1
tsrna-24488	GGTCCTGGGTTCGAGCCCCAGTGGAACCAC	0	0	4	0
tsrna-24487	GGTCCTGGGTTCGAGCCCCAGTGGAACCA	0	1	0	0
tsrna-24485	GGTCCTGGGTTCGAGCCCCAGTGGA	0	1	0	0
tsrna-24473	GGTCCGAGAGGTCCCGGGTTC	0	0	0	1
tsrna-24472	GGTCCGAGAGGTCCCGGGTT	0	2	0	0
tsrna-24471	GGTCCGAGAGGTCCCGGGT	0	0	0	0
tsrna-24468	GGTCCCTGGTTCGATCCCGG	1	0	0	0
tsrna-24461	GGTCCCGGGTTCAAATCCCGGACGAGCCCC	5	1	0	0
tsrna-24460	GGTCCCGGGTTCAAATCCCGGACGAGCCC	0	1	0	0
tsrna-24459	GGTCCCGGGTTCAAATCCCGGACGAGCC	1	2	0	0
tsrna-24453	GGTCCCCGGTTCGAAACCGGGCGGAAACAC	3	0	0	1
tsrna-24448	GGTCCCATGGTGTAATGGTTAGCACTCTGGACTTTG	0	2	0	1
tsrna-24447	GGTCCCATGGTGTAATGGTTAGCACTCTGGACT	0	2	1	0
tsrna-24446	GGTCCCATGGTGTAATGGTTAGCACTCTGGAC	1	2	0	0
tsrna-24445	GGTCCCATGGTGTAATGGTTAGCACTCTGGA	1	0	1	2
tsrna-24444	GGTCCCATGGTGTAATGGTTAGCACTCTGG	0	3	0	1
tsrna-24443	GGTCCCATGGTGTAATGGTTAGCACTCTG	0	1	0	0
tsrna-24432	GGTCCCATGGTGTAATGG	0	1	0	0
tsrna-24429	GGTCCAGTGGTAGAATTCTC	0	0	0	1
tsrna-24426	GGTCCAGGGTTCAAGTCCCTGTTCGGGCGC	0	1	0	0
tsrna-24425	GGTCCAGGGTTCAAGTCCCTGTTCGGGCG	0	0	0	0
tsrna-24423	GGTCAGCTAAATAAGCTATCGGGCCCATAC	0	1	4	1
tsrna-24422	GGTCAGCTAAATAAGCTATCGGGCCCA	0	0	2	0
tsrna-24420	GGTCAGCTAAATAAGCTATCGGGCC	1	0	0	0
tsrna-24411	GGTCAGCACTCTGGACTCTGAATCCAGC	1	4	1	0
tsrna-24410	GGTCAGCACTCTGGACTCTGAATCC	1	3	0	0
tsrna-24400	GGTATTAGAAAAACCATTTCATAACTTTGT	0	0	1	0
tsrna-24398	GGTATGATTCTCGGTTTGGGTC	0	1	0	0
tsrna-24396	GGTATGATTCTCGGTTTGGG	0	1	0	0
tsrna-24395	GGTATGATTCTCGGTTTGG	0	0	1	0
tsrna-24391	GGTATGATTCTCGCTTTGGGTGC	0	0	0	1
tsrna-24390	GGTATGATTCTCGCTTTGGGTG	2	0	0	0

tsrna-24388	GGTATGATTCTCGCTTTG	0	0	2	1
tsrna-24387	GGTATGATTCTCGCTTT	0	1	0	0
tsrna-24386	GGTATGATTCTCGCTTCGGGTGTG	0	2	0	0
tsrna-24385	GGTATGATTCTCGCTTCGGGTGC	0	0	0	0
tsrna-24384	GGTATGATTCTCGCTTCGGGTG	2	0	1	0
tsrna-24383	GGTATGATTCTCGCTTCGG	1	2	0	0
tsrna-24381	GGTATGATTCTCGCTTC	0	1	0	0
tsrna-24380	GGTATGATTCTCGCTT	0	1	0	0
tsrna-24379	GGTATCATGCAAGATTCCCATTC	0	0	0	0
tsrna-24364	GGTATAGTGGTGAGCATAGCTGCCTTCCA	0	2	0	0
tsrna-24362	GGTATAGTGGTGAGCATAGCTGCC	0	1	0	0
tsrna-24360	GGTATAGTGGTGAGCATAGCTG	0	1	0	0
tsrna-24356	GGTATAGTGGTAAGCATAGCTGC	0	0	0	0
tsrna-24352	GGTATAGCTCAGTGGTAGAGCATTTGACTG	0	0	2	2
tsrna-24351	GGTATAGCTCAGTGGTAGAGCATTTGACT	1	1	2	0
tsrna-24350	GGTATAGCTCAGTGGTAGAGCATTTGAC	2	0	0	0
tsrna-24349	GGTATAGCTCAGTGGTAGAGCATTTG	2	2	1	0
tsrna-24348	GGTATAGCTCAGTGGTAGAGCA	0	0	0	0
tsrna-24346	GGTATAGCTCAGTGGTAGAG	0	1	0	0
tsrna-24344	GGTATAGCTCAGTGGGTAGAGCATTTGACT	1	2	1	1
tsrna-24343	GGTATAGCTCAGTGGGTAGAG	0	0	0	0
tsrna-24342	GGTATAGCTCAGGGGTAGAGCATTTGACTG	1	1	1	1
tsrna-24341	GGTATAGCTCAGGGGTAGAGCATTTGACT	1	0	0	1
tsrna-24337	GGTAGTGTGGCCGAGCGGTCTAAGGCGCTGGATT	0	2	1	0
tsrna-24336	GGTAGTGTGGCCGAGCGGTCTAAGGCGCTG	0	0	0	0
tsrna-24335	GGTAGTGTGGCCGAGCGGTCTAAGGCGCT	0	0	0	1
tsrna-24333	GGTAGTGTGGCCGAGCGGTCTAAGGC	0	1	0	0
tsrna-24332	GGTAGTGTGGCCGAGCGGTCTAAGG	1	0	0	0
tsrna-24331	GGTAGTGTGGCCGAGCGGTCTAAG	0	0	1	0
tsrna-24330	GGTAGTGTGGCCGAGCGGTCTAA	0	0	0	0
tsrna-24329	GGTAGTGTGGCCGAGCGGTCTA	0	0	0	0
tsrna-24328	GGTAGTGTGGCCGAGCGGTCT	0	0	1	0
tsrna-24324	GGTAGTGTGGCCGAGCG	1	0	0	0
tsrna-24321	GGTAGTCGTGGCCGAGTGG	9	8	3	4
tsrna-24310	GGTAGCGTGGCCGAGTGGTCTAAGGCGCTGGATTT	3	4	7	4
tsrna-24309	GGTAGCGTGGCCGAGTGGTCTAAGGCGCTGGATT	2	7	2	2
tsrna-24308	GGTAGCGTGGCCGAGTGGTCTAAGGCGCTG	3	4	3	0
tsrna-24307	GGTAGCGTGGCCGAGTGGTCTAAGGCGCT	3	4	1	1
tsrna-24306	GGTAGCGTGGCCGAGTGGTCTAAGGCGC	2	4	4	1
tsrna-24305	GGTAGCGTGGCCGAGTGGTCTAAGGCG	2	6	0	1
tsrna-24304	GGTAGCGTGGCCGAGTGGTCTAAGGC	1	4	4	0
tsrna-24303	GGTAGCGTGGCCGAGTGGTCTAAGG	1	5	2	1
tsrna-24302	GGTAGCGTGGCCGAGTGGTCTAAGAC	4	7	2	0
tsrna-24301	GGTAGCGTGGCCGAGTGGTCTAAG	1	7	2	0
tsrna-24300	GGTAGCGTGGCCGAGTGGTCTAA	1	2	0	3
tsrna-24299	GGTAGCGTGGCCGAGTGGTCTA	1	7	1	3
tsrna-24298	GGTAGCGTGGCCGAGTGGTCT	1	4	2	4
tsrna-24297	GGTAGCGTGGCCGAGTGGTC	2	6	1	3
tsrna-24296	GGTAGCGTGGCCGAGTGGT	1	5	2	1
tsrna-24295	GGTAGCGTGGCCGAGTGG	2	7	4	1
tsrna-24294	GGTAGCGTGGCCGAGTG	4	4	1	1
tsrna-24293	GGTAGCGTGGCCGAGT	1	1	0	0
tsrna-24292	GGTAGCGTGGCCGAGCGGTCTAAGGCGCTGGATT	6	10	4	5
tsrna-24291	GGTAGCGTGGCCGAGCGGTCTAAGGCGCTGGAT	0	8	3	6
tsrna-24290	GGTAGCGTGGCCGAGCGGTCTAAGGCGCTG	4	2	0	2
tsrna-24289	GGTAGCGTGGCCGAGCGGTCTAAGGCGCT	2	4	0	2
tsrna-24288	GGTAGCGTGGCCGAGCGGTCTAAGGCGC	3	5	2	1
tsrna-24287	GGTAGCGTGGCCGAGCGGTCTAAGGCG	0	0	4	4
tsrna-24286	GGTAGCGTGGCCGAGCGGTCTAAGGC	3	8	0	1
tsrna-24285	GGTAGCGTGGCCGAGCGGTCTAAGG	1	8	0	1
tsrna-24284	GGTAGCGTGGCCGAGCGGTCTAAG	1	5	1	3
tsrna-24283	GGTAGCGTGGCCGAGCGGTCTAA	2	3	0	3
tsrna-24282	GGTAGCGTGGCCGAGCGGTCTA	2	2	2	2
tsrna-24281	GGTAGCGTGGCCGAGCGGTCT	2	3	1	4

tsrna-24280	GGTAGCGTGGCCGAGCGGTC	2	5	0	1
tsrna-24279	GGTAGCGTGGCCGAGCGGT	1	1	2	0
tsrna-24278	GGTAGCGTGGCCGAGCGG	0	0	0	0
tsrna-24277	GGTAGCGTGGCCGAGCG	0	0	1	0
tsrna-24276	GGTAGCGTGGCCGAGC	0	0	0	0
tsrna-24275	GGTAGCGGGATCGATGCCCGCATCCTCCAC	1	0	1	0
tsrna-24274	GGTAGCGGGATCGATGCC	0	0	1	0
tsrna-24272	GGTAGCGCGTCTGACTCC	1	1	0	0
tsrna-24264	GGTAGAGCATTTGACTGCAGATC	0	0	0	0
tsrna-24261	GGTAGAGCATTTGACTG	0	0	0	0
tsrna-24259	GGTAGAGCATGGGACTCTTAATCCC	5	5	5	4
tsrna-24258	GGTAGAGCATGGGACTCTTAATCC	4	8	4	2
tsrna-24257	GGTAGAGCATGGGACTCT	5	1	0	0
tsrna-24256	GGTAGAGCATGGGACTC	1	0	0	0
tsrna-24255	GGTAGAGCATGGGACT	1	4	1	3
tsrna-24254	GGTAGAGCATGAGACTCTTAATCT	2	8	1	2
tsrna-24253	GGTAGAGCATGAGACTCTTA	1	3	2	1
tsrna-24252	GGTAGAGCATGAGACTC	1	5	0	0
tsrna-24251	GGTAGAGCATGAGACT	2	5	0	1
tsrna-24250	GGTAGAGCATCAGACTTTTAATCTGAGGGTCCAGGGT	11	30	6	1
tsrna-24249	GGTAGAGCATCAGACTTTTAATC	1	0	0	0
tsrna-24244	GGTAGAATTCTCGCCTGCCACGCGGGAGGCCCGGGTT	62	77	40	13
tsrna-24243	GGTAGAATTCTCGCCTGCCACGCGGGAGGCCCGGGT	51	78	25	11
tsrna-24242	GGTAGAATTCTCGCCTGCCACGCGGGAGGCCC	40	39	20	10
tsrna-24241	GGTAGAATTCTCGCCTGCCACGCGGGAGGC	36	46	21	13
tsrna-24240	GGTAGAATTCTCGCCTGCCACGCGGGAG	55	32	21	10
tsrna-24239	GGTAGAATTCTCGCCTGCCACGCGGGA	43	43	19	18
tsrna-24238	GGTAGAATTCTCGCCTGCCACGCGG	26	41	23	19
tsrna-24237	GGTAGAATTCTCGCCTGCCACGC	32	41	21	14
tsrna-24236	GGTAGAATTCTCGCCTGCCACG	35	23	18	9
tsrna-24235	GGTAGAATTCTCGCCTGCCAC	9	5	4	2
tsrna-24234	GGTAGAATTCTCGCCTGCCA	4	4	0	0
tsrna-24233	GGTAGAATTCTCGCCTGCC	3	6	3	2
tsrna-24232	GGTAGAATTCTCGCCTGC	1	3	1	0
tsrna-24231	GGTAGAATTCTCGCCTG	1	2	0	0
tsrna-24230	GGTAGAATTCTCGCCTCCCACGCGGGAGACCCGGGTT	2	1	3	0
tsrna-24229	GGTAGAATTCTCGCCTCCCACGCGGGAGACCCGGGT	1	4	0	0
tsrna-24228	GGTAGAATTCTCGCCTCCCACG	2	2	1	0
tsrna-24227	GGTAGAATTCTCGCCTCCCA	0	3	0	3
tsrna-24226	GGTAGAATTCTCGCCTCCC	0	1	1	0
tsrna-24225	GGTAGAATTCTCGCCTCC	4	2	0	2
tsrna-24224	GGTAGAATTCTCGCCTC	0	0	0	0
tsrna-24223	GGTAGAATTCTCGCCT	2	3	1	1
tsrna-24222	GGTAATCGCATAAAACTTAAAACTTTACAG	2	1	6	0
tsrna-24221	GGTAATCGCATAAAACTTAAAACTTTACA	4	2	4	0
tsrna-24220	GGTAATCGCATAAAACTTAAAACTTTA	3	4	7	0
tsrna-24219	GGTAATCGCATAAAACTTAAAACTTT	1	4	5	0
tsrna-24218	GGTAATCGCATAAAACTTAAAACTT	0	0	1	0
tsrna-24216	GGTAATCGCATAAAACTTAAAAC	0	0	0	0
tsrna-24203	GGTAAGCACTCTGGACTCTGAATCCAGC	0	4	1	2
tsrna-24202	GGTAAGCACTCTGGACTCTGAATCCA	0	0	0	0
tsrna-24201	GGTAAGCACTCTGGACTCTGAATCC	2	2	0	0
tsrna-24191	GGTAAATATAGTTTAACCAAAACATCAGATTGTGAATCTGACA	0	4	2	0
tsrna-24190	GGTAAATATAGTTTAACCAAAACATCAGATTGTGA	1	0	3	1
tsrna-24189	GGTAAATATAGTTTAACCAAAACATCAGATTGT	0	0	1	0
tsrna-24188	GGTAAATATAGTTTAACCAAAACATCAGAT	1	0	0	0
tsrna-24187	GGTAAATATAGTTTAACCAAAACATCAG	1	0	0	1
tsrna-24184	GGTAAAATGGCTGAGTGAAGCATTGGACTGTAAATCT	12	9	21	8
tsrna-24183	GGTAAAATGGCTGAGTGAAGCATTGGACTGTAAATC	17	9	21	6
tsrna-24182	GGTAAAATGGCTGAGTGAAGCATTGGACTGTAAA	17	13	16	14
tsrna-24181	GGTAAAATGGCTGAGTGAAGCATTGGACTGTAA	20	7	13	15
tsrna-24180	GGTAAAATGGCTGAGTGAAGCATTGGACTGTA	10	12	8	10
tsrna-24179	GGTAAAATGGCTGAGTGAAGCATTGGACTGT	2	6	2	2
tsrna-24178	GGTAAAATGGCTGAGTGAAGCATTGGACTG	1	1	0	0

tsrna-24177	GGTAAAATGGCTGAGTGAAGCATTGGACT	0	1	0	1
tsrna-24176	GGTAAAATGGCTGAGTGAAGCATTGGAC	1	1	0	1
tsrna-24175	GGTAAAATGGCTGAGTGAAGCATTGGA	0	4	0	0
tsrna-24174	GGTAAAATGGCTGAGTGAAGCATTGG	0	2	0	0
tsrna-24173	GGTAAAATGGCTGAGTGAAGCATTG	0	2	0	0
tsrna-24172	GGTAAAATGGCTGAGTGAAGCATT	0	1	0	0
tsrna-24171	GGTAAAATGGCTGAGTGAAGCAT	2	0	0	0
tsrna-24170	GGTAAAATGGCTGAGTGAAGCA	0	0	0	1
tsrna-24169	GGTAAAATGGCTGAGTGAAGC	0	1	0	0
tsrna-24168	GGTAAAATGGCTGAGTGAAG	0	0	0	0
tsrna-24167	GGTAAAATGGCTGAGTGAA	0	2	0	0
tsrna-24166	GGTAAAATGGCTGAGTGA	0	0	0	0
tsrna-24165	GGTAAAATGGCTGAGTG	0	0	0	0
tsrna-24163	GGGTTTTGCAGTCCTTACCA	0	3	0	0
tsrna-24161	GGGTTTTGCAGTCCTTAC	0	0	1	0
tsrna-24160	GGGTTTTGCAGTCCTTA	0	1	1	0
tsrna-24159	GGGTTTTGCAGTCCTT	0	1	1	0
tsrna-24158	GGGTTTCCCCGCGCAGGTTCG	0	0	0	0
tsrna-24154	GGGTTTCCCCGCACAGGTT	2	0	0	0
tsrna-24153	GGGTTTCCCCGCACAGGT	1	0	0	0
tsrna-24152	GGGTTTAAGTCCCATTGGTCTAGCCA	2	3	0	0
tsrna-24151	GGGTTTAAGTCCCATTGGTCTAGCC	0	1	0	0
tsrna-24150	GGGTTTAAGTCCCATTGGTCTAGC	1	0	0	0
tsrna-24149	GGGTTTAAGTCCCATTGGTCTAG	0	1	0	0
tsrna-24148	GGGTTTAAGTCCCATTGGTCTA	1	1	1	0
tsrna-24147	GGGTTTAAGTCCCATTGGTCT	0	1	0	0
tsrna-24144	GGGTTTAAGTCCCATTG	0	2	0	0
tsrna-24142	GGGTTCGATTCTCATAGTCCTAGCCA	1	2	0	1
tsrna-24141	GGGTTCGATTCTCATAGTCCTAGCC	1	2	0	1
tsrna-24140	GGGTTCGATTCTCATAGTCCTAGC	1	5	0	0
tsrna-24139	GGGTTCGATTCTCATAGTCCTAG	0	2	0	0
tsrna-24138	GGGTTCGATTCTCATAGTCCT	0	3	1	0
tsrna-24137	GGGTTCGATTCTCATAGTCC	1	2	1	0
tsrna-24136	GGGTTCGATTCTCATAGTC	0	2	0	0
tsrna-24134	GGGTTCGATTCCTTCCTTTTTTGCCA	1	2	1	2
tsrna-24133	GGGTTCGATTCCTTCCTTTTTTGCC	0	4	3	0
tsrna-24132	GGGTTCGATTCCTTCCTTTTTTGC	1	1	0	0
tsrna-24130	GGGTTCGATTCCCGGTCAGGGAACCA	7	292	4	3
tsrna-24129	GGGTTCGATTCCCGGTCAGGGAACC	0	11	1	0
tsrna-24128	GGGTTCGATTCCCGGTCAGGGAAC	1	2	1	0
tsrna-24127	GGGTTCGATTCCCGGTCAGGGAA	0	1	0	0
tsrna-24126	GGGTTCGATTCCCGGTCAGGGA	0	1	0	0
tsrna-24124	GGGTTCGATTCCCGGTCAGG	0	1	0	0
tsrna-24121	GGGTTCGATTCCCGGGCGGCGCACCA	9	62	5	3
tsrna-24120	GGGTTCGATTCCCGGCCCATGCACCA	16	32	6	12
tsrna-24119	GGGTTCGATTCCCGGCCAATGCACCA	45	168	17	13
tsrna-24118	GGGTTCGATTCCCCGACGGGGAGCCA	2	21	2	1
tsrna-24110	GGGTTCGATCCCCGGCATCTCCACCA	225	384	140	80
tsrna-24109	GGGTTCGATCCCCGGCATCTCCACC	16	25	5	4
tsrna-24108	GGGTTCGATCCCCGGCACCTCCACCA	139	306	93	54
tsrna-24106	GGGTTCGATCCCCAGTACCTCCACCA	24	70	17	9
tsrna-24105	GGGTTCGATCCCCAGCATCTCCACCA	9	13	0	4
tsrna-24104	GGGTTCGATCCCCAGCATCTCCACC	2	1	0	0
tsrna-24100	GGGTTCGAGTCCCTTCGTGGTCGCCA	0	5	0	0
tsrna-24098	GGGTTCGAGTCCCTTCGTGGTCGC	1	0	0	0
tsrna-24095	GGGTTCGAGTCCCATCTGGGTCGCCA	2	16	4	0
tsrna-24094	GGGTTCGAGTCCCACCAGAGTCGCCA	2	2	0	1
tsrna-24091	GGGTTCGAGCCCCAGTGGAACCACCA	45	19	24	13
tsrna-24090	GGGTTCGAGCCCCAGTGGAACCACC	5	1	0	2
tsrna-24089	GGGTTCGAGCCCCAGTGGAACCAC	0	0	1	0
tsrna-24088	GGGTTCGAGCCCCAGTGGAACCA	0	1	0	0
tsrna-24087	GGGTTCGAGCCCCAGTGGAACC	1	0	1	0
tsrna-24085	GGGTTCGAGCCCCACGTTGGGCGCCA	14	190	7	1
tsrna-24084	GGGTTCGAGCCCCACGTTGGGCGCC	0	7	0	0

tsrna-24081	GGGTTCGACTCCCGGTGTGGGAACCA	63	269	27	20
tsrna-24080	GGGTTCGACTCCCGGTGTGGGAACC	6	19	0	0
tsrna-24079	GGGTTCGACTCCCGGTGTGGGAAC	0	1	0	0
tsrna-24078	GGGTTCGACTCCCGGTGTGGGAA	0	0	1	0
tsrna-24077	GGGTTCGACTCCCGGTGTGGGA	0	0	1	0
tsrna-24076	GGGTTCGACTCCCGGTGTGGG	0	0	1	0
tsrna-24071	GGGTTCGACTCCCGGTATGGGAACCA	8	85	4	3
tsrna-24070	GGGTTCGACTCCCGGTATGGGAAC	0	2	0	0
tsrna-24068	GGGTTCGACTCCCAGCGGGGCCTCCA	0	0	0	1
tsrna-24066	GGGTTCGAATCCCTTCGTGGTTGCCA	0	4	0	0
tsrna-24064	GGGTTCGAATCCCTTCGTGGTTGC	0	1	0	0
tsrna-24062	GGGTTCGAATCCCATCCTCGTCGCCA	5	13	3	0
tsrna-24061	GGGTTCGAATCCCATCCTCGTCGCC	0	1	0	0
tsrna-24059	GGGTTCGAATCCCATCCTCGTCG	0	0	0	0
tsrna-24058	GGGTTCGAATCCCAGCGGTGCCTCCA	29	41	5	3
tsrna-24056	GGGTTCGAATCCCAGCGGGGCCTCCA	2	4	1	1
tsrna-24055	GGGTTCGAATCCCACTTCTGACACCA	16	24	10	9
tsrna-24054	GGGTTCGAATCCCACTTCTGACACC	0	2	1	1
tsrna-24053	GGGTTCGAATCCCACTTCTGACAC	0	1	0	0
tsrna-24052	GGGTTCGAATCCCACTCCTGACACCA	103	103	51	29
tsrna-24051	GGGTTCGAATCCCACTCCTGACAC	1	0	0	0
tsrna-24050	GGGTTCGAATCCCACCGCTGCCACCA	18	36	1	0
tsrna-24048	GGGTTCGAATCCCACCAGAGTCGCCA	3	9	0	0
tsrna-24045	GGGTTCAATTCCCCGACGGGGAGCCA	0	3	0	0
tsrna-24044	GGGTTCAATTCCCCGACGGGGAGCC	1	1	0	0
tsrna-24042	GGGTTCAATCCCCGGCATCTCCACCA	126	161	79	50
tsrna-24041	GGGTTCAATCCCCGGCACCTCCACCA	219	336	114	53
tsrna-24040	GGGTTCAATCCCCGGCACCTCCAC	0	0	0	1
tsrna-24039	GGGTTCAATCCCCGGCACCTCCA	1	0	0	0
tsrna-24038	GGGTTCAAGTCCCTGTTCGGGCGCCA	7	4	4	3
tsrna-24033	GGGTTCAAGTCCCTGTCCAGGCGCCA	0	3	0	0
tsrna-24027	GGGTTAGGCCTCTTTTTACCACCA	2	1	3	1
tsrna-24025	GGGTTAGGCCTCTTTTTACCAC	0	0	0	0
tsrna-24024	GGGTTAGGCCTCTTTTTACCA	0	1	0	1
tsrna-24023	GGGTTAGGCCTCTTTTTACC	0	0	0	0
tsrna-24022	GGGTTAGGCCTCTTTTTAC	0	0	1	0
tsrna-24021	GGGTTAGGCCTCTTTTTA	0	0	0	0
tsrna-24018	GGGTGTGATAGGTGGCACGGAGAATTTTG	1	1	0	0
tsrna-24017	GGGTGTGATAGGTGGCACGGAGAA	0	1	0	0
tsrna-24014	GGGTGTGATAGGTGGCACGGA	0	0	0	0
tsrna-24007	GGGTGTAGCTCAGTGGTAGAGCGCGTGC	7	12	2	0
tsrna-24006	GGGTGTAGCTCAGTGGTAGAGC	0	0	0	0
tsrna-24005	GGGTGTAGCTCAGTGGTAGAG	0	0	0	0
tsrna-24003	GGGTGTAGCTCAGTGGTAG	0	0	0	0
tsrna-23999	GGGTGGTTCAGTGGTAGAATTCTCGCCTGC	8	12	7	2
tsrna-23998	GGGTGGTTCAGTGGTAGAATTCTCGCCT	6	11	3	3
tsrna-23997	GGGTGGTTCAGTGGTAGAATTCTCGCC	6	6	2	2
tsrna-23996	GGGTGGTTCAGTGGTAGAATTCTCGC	4	1	1	1
tsrna-23995	GGGTGGTTCAGTGGTAGAATTCTCG	2	2	2	1
tsrna-23994	GGGTGGTTCAGTGGTAGAATTCTC	3	2	2	0
tsrna-23993	GGGTGGTTCAGTGGTAGAATTCT	0	1	0	0
tsrna-23992	GGGTGGTTCAGTGGTAGAA	1	0	0	0
tsrna-23989	GGGTGCTTAGCTGTTAACTAAG	1	0	0	0
tsrna-23988	GGGTGCTTAGCTGTTAACTAA	0	0	0	0
tsrna-23982	GGGTGCTAATGGTGGAGTTAAAGACTTTTTCTCTGACCA	23	33	20	23
tsrna-23981	GGGTGCTAATGGTGGAGTTAAAGACTTTTT	5	7	5	0
tsrna-23980	GGGTGCTAATGGTGGAGTTAAAGACT	0	3	3	0
tsrna-23979	GGGTGCTAATGGTGGAGTTAAAGAC	0	1	1	2
tsrna-23978	GGGTGCTAATGGTGGAGTTAAAGA	1	4	1	0
tsrna-23977	GGGTGCTAATGGTGGAGTTAAAG	0	7	2	0
tsrna-23976	GGGTGCTAATGGTGGAGTTAAA	1	2	1	0
tsrna-23975	GGGTGCTAATGGTGGAGTTAA	2	3	1	0
tsrna-23974	GGGTGCTAATGGTGGAGTTA	1	3	0	0
tsrna-23973	GGGTGCTAATGGTGGAGTT	1	0	0	0

tsrna-23970	GGGTGCGAGAGGTCCCGGGTTCAAATCCCG	0	0	1	0
tsrna-23969	GGGTGCGAGAGGTCCCGGGTTC	0	1	0	0
tsrna-23968	GGGTGCGAGAGGTCCCGGGTT	1	4	1	0
tsrna-23967	GGGTGCGAGAGGTCCCGGGT	1	1	0	0
tsrna-23963	GGGTGCGAGAGGTCCC	0	0	0	0
tsrna-23962	GGGTCTCCCCGCGCAGGT	0	3	0	0
tsrna-23956	GGGTCGATTCCCCGACGGGGAGCCA	1	9	1	0
tsrna-23954	GGGTCGATTCCCCGACGGGGAGC	0	1	0	0
tsrna-23953	GGGTCGATTCCCCGACGGGGAG	0	1	0	0
tsrna-23946	GGGTCCGAGAGGTCCCGGGTTCAAATCCCG	1	0	1	0
tsrna-23945	GGGTCCGAGAGGTCCCGGGTTC	1	1	1	0
tsrna-23944	GGGTCCGAGAGGTCCCGGGTT	0	1	0	0
tsrna-23943	GGGTCCGAGAGGTCCCGGGT	0	1	0	0
tsrna-23934	GGGTCCAGGGTTCAAGTCCCTGTTCGGGCG	0	0	0	0
tsrna-23929	GGGTATGATTCTCGGTTTGGG	0	0	0	0
tsrna-23927	GGGTATGATTCTCGGTTTG	1	0	0	0
tsrna-23923	GGGTATGATTCTCGCTTTGGGTG	2	0	0	0
tsrna-23922	GGGTATGATTCTCGCTTTGGG	3	1	0	1
tsrna-23921	GGGTATGATTCTCGCTTTGG	1	1	2	0
tsrna-23920	GGGTATGATTCTCGCTTTG	0	2	0	2
tsrna-23919	GGGTATGATTCTCGCTTT	0	3	0	0
tsrna-23918	GGGTATGATTCTCGCTTCGG	1	3	2	0
tsrna-23917	GGGTATGATTCTCGCTTCG	1	0	1	0
tsrna-23916	GGGTATGATTCTCGCTTC	0	3	0	0
tsrna-23914	GGGTATGATTCTCGCT	0	0	0	1
tsrna-23913	GGGTATAGCTCAGTGGTAGAGCGCGTGC	12	26	2	0
tsrna-23912	GGGTATAGCTCAGTGGTAGAGCATTTGACT	4	2	0	0
tsrna-23910	GGGTATAGCTCAGTGGTAGAG	0	0	1	1
tsrna-23909	GGGTATAGCTCAGTGGTAGA	1	2	0	0
tsrna-23907	GGGTATAGCTCAGTGGGTAGAGCATTTGAC	0	0	1	0
tsrna-23906	GGGTATAGCTCAGTGG	0	0	0	0
tsrna-23905	GGGTATAGCTCAGGGGTAGAGCATTTGACT	0	8	1	0
tsrna-23904	GGGTATAGCTCAGGGGTAGAGCA	0	0	0	2
tsrna-23903	GGGTATAGCTCAGGGGTAGAGC	0	0	0	2
tsrna-23902	GGGTATAGCTCAGGGGTAGAG	0	3	1	0
tsrna-23901	GGGTATAGCTCAGGGGTAGA	0	0	0	0
tsrna-23900	GGGTATAGCTCAGGGGTAG	3	1	2	1
tsrna-23899	GGGTATAGCTCAGGGG	0	1	1	3
tsrna-23898	GGGTATAGCTCAGCGGTAGAGCGCGTGC	0	9	0	1
tsrna-23896	GGGTAGCGTGGCCGAGCGGTCT	0	5	0	2
tsrna-23895	GGGTAGCGTGGCCGAGCGGTC	0	5	1	3
tsrna-23894	GGGTAGCGTGGCCGAGCGGT	0	4	1	3
tsrna-23893	GGGTAGCGTGGCCGAGCGG	1	0	2	1
tsrna-23890	GGGTAGAGCATTTGACTGC	1	0	0	0
tsrna-23887	GGGGTTTTGCAGTCCTTACCA	1	1	0	1
tsrna-23886	GGGGTTTTGCAGTCCTTACC	0	1	1	0
tsrna-23885	GGGGTTTTGCAGTCCTTAC	0	0	0	0
tsrna-23883	GGGGTTTTGCAGTCCTT	0	1	0	0
tsrna-23882	GGGGTTCGATTCCTTCCTTTTTTGCCA	3	1	0	1
tsrna-23881	GGGGTTCGATTCCTTCCTTTTTTGCC	0	5	0	0
tsrna-23880	GGGGTTCGATTCCTTCCTTTTTTGC	1	2	0	0
tsrna-23879	GGGGTTCGATTCCTTCCTTT	0	1	0	0
tsrna-23877	GGGGTTCGATTCCCCGACGGGGAGCCA	0	19	0	1
tsrna-23874	GGGGTTCGATTCCCCGACGGGGAG	1	1	0	0
tsrna-23873	GGGGTTCGATTCCCCGACGGGGA	0	1	0	0
tsrna-23869	GGGGTTCAATTCCCCGACGGGGAGCCA	0	3	0	0
tsrna-23867	GGGGTTCAATTCCCCGACGGGGAGC	0	1	0	0
tsrna-23866	GGGGTTAGGCCTCTTTTTACCACCA	3	1	4	1
tsrna-23865	GGGGTTAGGCCTCTTTTTACCACC	0	0	1	0
tsrna-23864	GGGGTTAGGCCTCTTTTTACCAC	0	0	2	0
tsrna-23863	GGGGTTAGGCCTCTTTTTACCA	1	0	0	0
tsrna-23862	GGGGTTAGGCCTCTTTTTACC	1	0	0	0
tsrna-23861	GGGGTTAGGCCTCTTTTTAC	0	0	0	0
tsrna-23859	GGGGTTAGGCCTCTTTTT	0	0	0	0

tsrna-23858	GGGGTTAGGCCTCTTTT	0	0	0	0
tsrna-23857	GGGGTTAGGCCTCTTT	0	1	0	0
tsrna-23856	GGGGTTAGAGCACTGGT	0	0	1	0
tsrna-23852	GGGGTGTGATAGGTGGCACGGA	0	0	0	0
tsrna-23846	GGGGTGTAGCTCAGTGGTAGAGCGCGTGCT	8	27	2	0
tsrna-23845	GGGGTGTAGCTCAGTGGTAGAGCGCGTGC	4	28	1	0
tsrna-23844	GGGGTGTAGCTCAGTGGTAGAGCGCGTG	1	0	0	0
tsrna-23842	GGGGTGTAGCTCAGTGGTAGAGCATTTGAC	1	0	0	0
tsrna-23839	GGGGTGTAGCTCAGTGGTAGA	1	0	0	0
tsrna-23838	GGGGTGTAGCTCAGTGGTAG	0	0	0	0
tsrna-23832	GGGGTCGATTCCCCGACGGGGAGCCA	0	13	0	0
tsrna-23831	GGGGTCGATTCCCCGACGGGGAGCC	0	0	0	0
tsrna-23829	GGGGTATGATTCTCGGTTTGG	1	0	0	0
tsrna-23828	GGGGTATGATTCTCGGTTTG	1	1	0	0
tsrna-23827	GGGGTATGATTCTCGGTTT	1	0	0	0
tsrna-23823	GGGGTATGATTCTCGCTTTGGGTG	1	2	1	1
tsrna-23822	GGGGTATGATTCTCGCTTTGG	0	0	0	0
tsrna-23821	GGGGTATGATTCTCGCTTTG	1	1	2	0
tsrna-23820	GGGGTATGATTCTCGCTTT	1	1	3	0
tsrna-23819	GGGGTATGATTCTCGCTTCGG	2	0	2	0
tsrna-23818	GGGGTATGATTCTCGCTTCG	0	0	0	1
tsrna-23817	GGGGTATGATTCTCGCTTC	0	1	1	0
tsrna-23816	GGGGTATGATTCTCGCTTA	1	1	2	1
tsrna-23815	GGGGTATGATTCTCGCTT	1	1	2	1
tsrna-23814	GGGGTATGATTCTCGCT	2	2	0	1
tsrna-23813	GGGGTATGATTCTCGC	2	2	1	0
tsrna-23812	GGGGTATAGCTCAGTGGTAGAGCATTTGACT	2	5	0	1
tsrna-23811	GGGGTATAGCTCAGTGGTAGAGCATTTGAC	1	0	0	1
tsrna-23810	GGGGTATAGCTCAGTGGTAGAGCATTTGA	2	2	0	0
tsrna-23809	GGGGTATAGCTCAGTGGTAGAGCAT	1	1	0	0
tsrna-23808	GGGGTATAGCTCAGTGGTAGAGCA	0	1	0	1
tsrna-23807	GGGGTATAGCTCAGTGGTAGAGC	1	2	0	0
tsrna-23806	GGGGTATAGCTCAGTGGTAGAG	0	0	0	2
tsrna-23805	GGGGTATAGCTCAGTGGTAGA	0	1	2	0
tsrna-23804	GGGGTATAGCTCAGTGGTAG	1	1	0	1
tsrna-23803	GGGGTATAGCTCAGTGGT	0	1	0	0
tsrna-23802	GGGGTATAGCTCAGTGGGTAGAGCATTTG	0	1	0	0
tsrna-23800	GGGGTATAGCTCAGTGGGTAGAGC	1	0	0	0
tsrna-23797	GGGGTATAGCTCAGGTGGTAGAGCATTTG	0	0	1	1
tsrna-23796	GGGGTATAGCTCAGGGGTAGAGCATTTGAC	0	0	1	1
tsrna-23795	GGGGTATAGCTCAGGGGTAGAGCATT	1	0	0	0
tsrna-23794	GGGGTATAGCTCAGGGGTAGAGCAT	0	2	1	2
tsrna-23793	GGGGTATAGCTCAGGGGTAGAGCA	1	1	1	3
tsrna-23792	GGGGTATAGCTCAGGGGTAGAGC	0	0	1	1
tsrna-23791	GGGGTATAGCTCAGGGGTAGAG	1	0	0	0
tsrna-23790	GGGGTATAGCTCAGGGGTAGA	0	2	2	0
tsrna-23789	GGGGTATAGCTCAGGGGTAG	0	2	0	0
tsrna-23788	GGGGTATAGCTCAGGGG	1	1	0	0
tsrna-23787	GGGGTATAGCTCAGGG	0	0	1	1
tsrna-23786	GGGGTAGAGCATTTGACTGCAGATCAAG	0	3	0	0
tsrna-23784	GGGGTAGAGCATTTGACTGC	1	1	0	0
tsrna-23780	GGGGGTTCGATTCCTTCCTTTTTTGCCA	2	4	0	2
tsrna-23779	GGGGGTTCGATTCCTTCCTTTTTTGCC	2	4	2	0
tsrna-23778	GGGGGTTCGATTCCTTCCTTTTTTGC	0	3	0	1
tsrna-23777	GGGGGTTCGATTCCTTCCTT	0	4	0	0
tsrna-23776	GGGGGTGTAGCTCAGTGGTAGAGCGT	1	6	2	0
tsrna-23775	GGGGGTGTAGCTCAGTGGTAGAGCGCGTGCTTC	13	31	8	6
tsrna-23774	GGGGGTGTAGCTCAGTGGTAGAGCGCGTGCTT	11	23	8	4
tsrna-23773	GGGGGTGTAGCTCAGTGGTAGAGCGCGTGCT	18	34	8	0
tsrna-23772	GGGGGTGTAGCTCAGTGGTAGAGCGCGTGC	9	32	5	2
tsrna-23771	GGGGGTGTAGCTCAGTGGTAGAGCGCGTG	5	6	5	3
tsrna-23770	GGGGGTGTAGCTCAGTGGTAGAGCGCGT	3	5	0	3
tsrna-23769	GGGGGTGTAGCTCAGTGGTAGAGCGCG	5	4	3	3
tsrna-23768	GGGGGTGTAGCTCAGTGGTAGAGCGCATGCTT	5	14	2	9

tsrna-23767	GGGGGTGTAGCTCAGTGGTAGAGCGCATGC	2	7	2	4
tsrna-23766	GGGGGTGTAGCTCAGTGGTAGAGCGCAT	1	2	2	3
tsrna-23765	GGGGGTGTAGCTCAGTGGTAGAGCGCA	2	4	2	3
tsrna-23764	GGGGGTGTAGCTCAGTGGTAGAGCGC	5	6	2	3
tsrna-23763	GGGGGTGTAGCTCAGTGGTAGAGCG	2	4	1	1
tsrna-23762	GGGGGTGTAGCTCAGTGGTAGAGCATTTGACTG	9	14	6	11
tsrna-23761	GGGGGTGTAGCTCAGTGGTAGAGCATTTGACT	7	11	6	6
tsrna-23760	GGGGGTGTAGCTCAGTGGTAGAGCATTTGA	2	5	4	3
tsrna-23759	GGGGGTGTAGCTCAGTGGTAGAGCATTTG	5	1	0	4
tsrna-23758	GGGGGTGTAGCTCAGTGGTAGAGCATT	5	6	2	2
tsrna-23757	GGGGGTGTAGCTCAGTGGTAGAGCAT	3	1	0	3
tsrna-23756	GGGGGTGTAGCTCAGTGGTAGAGCACATGC	3	3	0	6
tsrna-23755	GGGGGTGTAGCTCAGTGGTAGAGCAC	7	1	1	3
tsrna-23754	GGGGGTGTAGCTCAGTGGTAGAGCA	2	6	6	1
tsrna-23753	GGGGGTGTAGCTCAGTGGTAGAGC	3	6	4	0
tsrna-23752	GGGGGTGTAGCTCAGTGGTAGAG	4	5	1	4
tsrna-23751	GGGGGTGTAGCTCAGTGGTAGA	4	5	0	2
tsrna-23750	GGGGGTGTAGCTCAGTGGTAG	4	3	1	2
tsrna-23749	GGGGGTGTAGCTCAGTGGTA	1	4	3	3
tsrna-23748	GGGGGTGTAGCTCAGTGGT	1	9	0	0
tsrna-23747	GGGGGTGTAGCTCAGTGG	3	1	1	0
tsrna-23746	GGGGGTGTAGCTCAGTG	1	3	0	2
tsrna-23745	GGGGGTGTAGCTCAGT	1	0	1	0
tsrna-23740	GGGGGTCGATTCCCCGACGGGGAGCCA	1	13	0	0
tsrna-23739	GGGGGTATAGTTCAGGGGTAG	0	0	0	1
tsrna-23738	GGGGGTATAGCTTAGCGGTAGAGCATTTGACT	4	8	10	7
tsrna-23737	GGGGGTATAGCTTAGCGGTAGAGCATTTG	3	2	3	2
tsrna-23736	GGGGGTATAGCTTAGCGGTAGAGC	1	0	7	0
tsrna-23735	GGGGGTATAGCTTAGCGGTA	2	2	1	2
tsrna-23734	GGGGGTATAGCTTAGCGG	3	4	6	0
tsrna-23733	GGGGGTATAGCTTAGC	3	5	3	4
tsrna-23732	GGGGGTATAGCTCAGTGGTAGAGCGCGTGCTT	12	33	6	4
tsrna-23731	GGGGGTATAGCTCAGTGGTAGAGCGCGTGC	12	30	7	5
tsrna-23730	GGGGGTATAGCTCAGTGGTAGAGCATTTGACTGCA	43	34	21	8
tsrna-23729	GGGGGTATAGCTCAGTGGTAGAGCATTTGACTGC	43	36	22	7
tsrna-23728	GGGGGTATAGCTCAGTGGTAGAGCATTTGACTG	36	20	6	7
tsrna-23727	GGGGGTATAGCTCAGTGGTAGAGCATTTGACT	15	18	10	7
tsrna-23726	GGGGGTATAGCTCAGTGGTAGAGCATTTGAC	3	4	6	4
tsrna-23725	GGGGGTATAGCTCAGTGGTAGAGCATTTGA	4	3	3	4
tsrna-23724	GGGGGTATAGCTCAGTGGTAGAGCATTTG	1	6	1	2
tsrna-23723	GGGGGTATAGCTCAGTGGTAGAGCATTT	7	5	2	3
tsrna-23722	GGGGGTATAGCTCAGTGGTAGAGCATT	8	1	7	6
tsrna-23721	GGGGGTATAGCTCAGTGGTAGAGCAT	2	2	1	0
tsrna-23720	GGGGGTATAGCTCAGTGGTAGAGCA	6	3	3	5
tsrna-23719	GGGGGTATAGCTCAGTGGTAGAGC	4	0	2	1
tsrna-23718	GGGGGTATAGCTCAGTGGTAGAG	2	4	5	2
tsrna-23717	GGGGGTATAGCTCAGTGGTAGA	5	3	2	3
tsrna-23716	GGGGGTATAGCTCAGTGGTAG	2	4	3	2
tsrna-23715	GGGGGTATAGCTCAGTGGTA	3	3	5	0
tsrna-23714	GGGGGTATAGCTCAGTGGT	1	2	5	5
tsrna-23713	GGGGGTATAGCTCAGTGGGTAGAGCATTTGACTGC	9	4	2	2
tsrna-23712	GGGGGTATAGCTCAGTGGGTAGAGCATTTGACTG	6	6	0	5
tsrna-23711	GGGGGTATAGCTCAGTGGGTAGAGCATTTGACT	4	4	1	2
tsrna-23710	GGGGGTATAGCTCAGTGGGTAGAGCATTTG	1	3	1	3
tsrna-23709	GGGGGTATAGCTCAGTGGGTAGAGCATTT	0	4	1	2
tsrna-23708	GGGGGTATAGCTCAGTGGGTAGAGCATT	2	3	4	0
tsrna-23707	GGGGGTATAGCTCAGTGGGTAGAGCAT	2	3	1	1
tsrna-23706	GGGGGTATAGCTCAGTGGGTAGAGCA	0	1	2	2
tsrna-23705	GGGGGTATAGCTCAGTGGGTAGAGC	2	2	0	2
tsrna-23704	GGGGGTATAGCTCAGTGGGTAGAG	3	1	4	0
tsrna-23703	GGGGGTATAGCTCAGTGGGTAGA	1	1	1	0
tsrna-23702	GGGGGTATAGCTCAGTGGGTAG	4	1	0	1
tsrna-23701	GGGGGTATAGCTCAGTGGGTA	0	1	3	2
tsrna-23700	GGGGGTATAGCTCAGTGGGT	0	0	1	2

tsrna-23699	GGGGGTATAGCTCAGTGGG	1	4	1	2
tsrna-23698	GGGGGTATAGCTCAGTGG	3	2	0	0
tsrna-23697	GGGGGTATAGCTCAGTG	1	2	2	0
tsrna-23696	GGGGGTATAGCTCAGT	1	1	2	0
tsrna-23695	GGGGGTATAGCTCAGGTGGTAGAGCATTTGACTGC	2	2	3	0
tsrna-23694	GGGGGTATAGCTCAGGTGGTAGAGCATTTGACTG	0	3	2	2
tsrna-23693	GGGGGTATAGCTCAGGTGGTAGAGCATTTGACT	1	1	0	1
tsrna-23692	GGGGGTATAGCTCAGGTGGTAGAGCATTTG	1	2	0	0
tsrna-23691	GGGGGTATAGCTCAGGTGGTAGAGCATT	1	0	1	2
tsrna-23690	GGGGGTATAGCTCAGGTGGTAGAGCAT	1	0	0	3
tsrna-23689	GGGGGTATAGCTCAGGTGGTAGAGCA	0	2	3	1
tsrna-23688	GGGGGTATAGCTCAGGTGGTAGAGC	0	2	0	2
tsrna-23687	GGGGGTATAGCTCAGGTGGTAGAG	0	3	1	0
tsrna-23686	GGGGGTATAGCTCAGGTGGTAGA	2	2	0	2
tsrna-23685	GGGGGTATAGCTCAGGTGGTAG	2	2	1	1
tsrna-23684	GGGGGTATAGCTCAGGTGGTA	1	2	1	1
tsrna-23683	GGGGGTATAGCTCAGGTGGT	1	0	2	1
tsrna-23682	GGGGGTATAGCTCAGGTGG	3	0	0	1
tsrna-23681	GGGGGTATAGCTCAGGT	0	1	1	0
tsrna-23680	GGGGGTATAGCTCAGGGGTAGAGCATTTGACTGC	9	12	5	4
tsrna-23679	GGGGGTATAGCTCAGGGGTAGAGCATTTGACTG	6	13	2	5
tsrna-23678	GGGGGTATAGCTCAGGGGTAGAGCATTTGACT	5	11	6	2
tsrna-23677	GGGGGTATAGCTCAGGGGTAGAGCATTTGA	0	3	0	6
tsrna-23676	GGGGGTATAGCTCAGGGGTAGAGCATTTG	0	3	2	5
tsrna-23675	GGGGGTATAGCTCAGGGGTAGAGCATTT	2	3	2	3
tsrna-23674	GGGGGTATAGCTCAGGGGTAGAGCATT	2	1	2	4
tsrna-23673	GGGGGTATAGCTCAGGGGTAGAGCAT	1	1	1	5
tsrna-23672	GGGGGTATAGCTCAGGGGTAGAGCACTTG	2	2	0	2
tsrna-23671	GGGGGTATAGCTCAGGGGTAGAGCA	1	1	1	6
tsrna-23670	GGGGGTATAGCTCAGGGGTAGAGC	0	1	2	2
tsrna-23669	GGGGGTATAGCTCAGGGGTAGAG	1	0	0	0
tsrna-23668	GGGGGTATAGCTCAGGGGTAGA	1	2	2	2
tsrna-23667	GGGGGTATAGCTCAGGGGTAG	1	4	0	1
tsrna-23666	GGGGGTATAGCTCAGGGGTA	0	2	1	2
tsrna-23665	GGGGGTATAGCTCAGGGGT	2	3	3	0
tsrna-23664	GGGGGTATAGCTCAGGGG	0	1	1	2
tsrna-23663	GGGGGTATAGCTCAGGG	0	3	0	1
tsrna-23662	GGGGGTATAGCTCAGG	0	1	0	1
tsrna-23661	GGGGGTATAGCTCAGCGGTAGAGCGCGTGCT	6	23	3	6
tsrna-23660	GGGGGTATAGCTCAGCGGTAGAGCGCGTGC	5	13	1	9
tsrna-23659	GGGGGTATAGCTCAGCGGTAGAGCGCG	3	4	1	2
tsrna-23658	GGGGGTATAGCTCAGCGGTAGAGC	3	3	0	1
tsrna-23657	GGGGGTATAGCTCAGCGGTAGAG	1	4	1	2
tsrna-23656	GGGGGTATAGCTCAGCGGTAGA	3	1	1	5
tsrna-23655	GGGGGTATAGCTCAGCGGTAG	0	0	1	5
tsrna-23654	GGGGGTATAGCTCAGCGGTA	0	2	1	4
tsrna-23653	GGGGGTATAGCTCAGCGGT	2	5	2	1
tsrna-23652	GGGGGTATAGCTCAGCGG	0	2	0	1
tsrna-23651	GGGGGTATAGCTCAGCG	1	2	0	4
tsrna-23650	GGGGGTATAGCTCAGC	1	2	1	1
tsrna-23649	GGGGGGTTCGATTCCTTCCTTTTTTGCCA	3	5	2	3
tsrna-23648	GGGGGGTTCGATTCCTTCCTTTTTTGCC	2	6	0	0
tsrna-23647	GGGGGGTTCGATTCCTTCCTTTTTTGC	2	0	0	0
tsrna-23646	GGGGGGTTCGATTCCTTCCTTTTTTG	0	6	0	0
tsrna-23645	GGGGGGTTCGATTCCTTCCTTT	1	2	1	0
tsrna-23644	GGGGGGTTCGATTCCTTCCTT	0	1	0	0
tsrna-23641	GGGGGGTGTAGCTCAGTGG	1	1	0	1
tsrna-23638	GGGGGCATAGCTCAGTGGTAGAGCATTTGACTGC	2	10	7	9
tsrna-23637	GGGGGCATAGCTCAGTGGTAGAGCATTTGACTG	1	5	1	5
tsrna-23636	GGGGGCATAGCTCAGTGGTAGAGCATTTGACT	2	7	1	2
tsrna-23635	GGGGGCATAGCTCAGTGGTAGAGCATTTGA	1	1	0	1
tsrna-23634	GGGGGCATAGCTCAGTGGTAGAGCATTTG	3	4	0	1
tsrna-23633	GGGGGCATAGCTCAGTGGTAGAGCA	0	2	2	0
tsrna-23632	GGGGGCATAGCTCAGTGGTAGAGC	0	1	2	0

tsrna-23631	GGGGGCATAGCTCAGTGGTAGA	0	0	0	4
tsrna-23630	GGGGGCATAGCTCAGTGGT	2	2	1	4
tsrna-23629	GGGGGCATAGCTCAGTGG	0	1	0	1
tsrna-23627	GGGGGCAGAGCATTTGACTG	1	0	0	0
tsrna-23625	GGGGGATTAGCTCAAG	6	9	4	1
tsrna-23624	GGGGGATTAGCTCAAATGGTAGAGCGCTCGCTT	14	27	10	5
tsrna-23623	GGGGGATTAGCTCAAATGGTAGAGCGCTCGCT	22	26	9	5
tsrna-23622	GGGGGATTAGCTCAAATGGTAGAGCGCTCGC	17	19	14	13
tsrna-23621	GGGGGATTAGCTCAAATGGTAGAGCGCTCG	21	21	17	9
tsrna-23620	GGGGGATTAGCTCAAATGGTAGAGCGCTC	12	25	12	1
tsrna-23619	GGGGGATTAGCTCAAATGGTAGAGCGCT	14	14	6	1
tsrna-23618	GGGGGATTAGCTCAAATGGTAGAGCGC	17	21	10	6
tsrna-23617	GGGGGATTAGCTCAAATGGTAGAGCG	9	24	11	8
tsrna-23616	GGGGGATTAGCTCAAATGGTAGAGC	17	14	10	9
tsrna-23615	GGGGGATTAGCTCAAATGGTAGAG	24	23	14	5
tsrna-23614	GGGGGATTAGCTCAAATGGTAGA	17	25	7	4
tsrna-23613	GGGGGATTAGCTCAAATGGTAG	16	15	11	5
tsrna-23612	GGGGGATTAGCTCAAATGGTA	10	16	14	4
tsrna-23611	GGGGGATTAGCTCAAATGGT	17	17	10	4
tsrna-23610	GGGGGATTAGCTCAAATGG	10	18	6	3
tsrna-23609	GGGGGATTAGCTCAAATG	9	21	8	2
tsrna-23608	GGGGGATTAGCTCAAAT	16	21	11	9
tsrna-23607	GGGGGATTAGCTCAAA	16	20	14	4
tsrna-23605	GGGGCGTGGGTTCGAATC	0	1	0	0
tsrna-23598	GGGGATTGTGGGTTCGAGTCCCATCTGGGTCGCC	0	2	1	0
tsrna-23597	GGGGATTGTGGGTTCGAGTCCCATC	0	1	0	0
tsrna-23596	GGGGATTGTGGGTTCGAGTCCCA	0	2	0	0
tsrna-23595	GGGGATTGTGGGTTCGAGTCCC	0	3	0	0
tsrna-23592	GGGGATTGTGGGTTCG	0	0	0	0
tsrna-23591	GGGGATTAGCTCAAATGGTAGAGC	10	15	5	3
tsrna-23590	GGGGATTAGCTCAAATGGTAGAG	12	7	8	2
tsrna-23589	GGGGATTAGCTCAAATGGTAGA	10	14	7	6
tsrna-23588	GGGGATTAGCTCAAATGGTAG	17	14	5	3
tsrna-23587	GGGGATTAGCTCAAATGGT	11	8	2	5
tsrna-23586	GGGGATTAGCTCAAATGG	10	13	7	2
tsrna-23585	GGGGATTAGCTCAAAT	6	16	6	6
tsrna-23584	GGGGATGTAGCTCAGTGGTAGAGCGCGCGCTTC	11	21	8	13
tsrna-23583	GGGGATGTAGCTCAGTGGTAGAGCGCGCGCTT	14	19	1	7
tsrna-23582	GGGGATGTAGCTCAGTGGTAGAGCGCGCGCT	12	18	7	10
tsrna-23581	GGGGATGTAGCTCAGTGGTAGAGCGCGCGC	6	15	7	4
tsrna-23580	GGGGATGTAGCTCAGTGGTAGAGCGCGCG	9	12	0	5
tsrna-23579	GGGGATGTAGCTCAGTGGTAGAGCGCGC	6	13	4	6
tsrna-23578	GGGGATGTAGCTCAGTGGTAGAGCGCG	12	8	6	6
tsrna-23577	GGGGATGTAGCTCAGTGGTAGAGCGCATGCTTTGCA	7	5	7	4
tsrna-23576	GGGGATGTAGCTCAGTGGTAGAGCGCATGCTT	3	11	0	7
tsrna-23575	GGGGATGTAGCTCAGTGGTAGAGCGCATGCT	6	12	5	8
tsrna-23574	GGGGATGTAGCTCAGTGGTAGAGCGCATGC	6	9	4	4
tsrna-23573	GGGGATGTAGCTCAGTGGTAGAGCGCATG	7	13	3	5
tsrna-23572	GGGGATGTAGCTCAGTGGTAGAGCGCAT	5	8	1	4
tsrna-23571	GGGGATGTAGCTCAGTGGTAGAGCGCA	5	13	2	4
tsrna-23570	GGGGATGTAGCTCAGTGGTAGAGCGC	4	14	2	5
tsrna-23569	GGGGATGTAGCTCAGTGGTAGAGCG	7	14	2	6
tsrna-23568	GGGGATGTAGCTCAGTGGTAGAGC	6	12	4	4
tsrna-23567	GGGGATGTAGCTCAGTGGTAGAG	3	9	2	3
tsrna-23566	GGGGATGTAGCTCAGTGGTAGA	1	10	3	6
tsrna-23565	GGGGATGTAGCTCAGTGGTAG	8	4	5	6
tsrna-23564	GGGGATGTAGCTCAGTGGTA	5	7	3	3
tsrna-23563	GGGGATGTAGCTCAGTGGT	3	6	1	3
tsrna-23562	GGGGATGTAGCTCAGTGG	2	4	0	0
tsrna-23561	GGGGATGTAGCTCAGTG	1	3	0	0
tsrna-23560	GGGGATGTAGCTCAGT	4	2	0	0
tsrna-23559	GGGGATATAGCTCAGGGGTAGAGCATTTG	1	0	1	1
tsrna-23558	GGGGAATTAGCTCAGGCGGTAGAGC	0	2	0	1
tsrna-23557	GGGGAATTAGCTCAGGCGGTAGAG	1	0	0	0

tsrna-23556	GGGGAATTAGCTCAGGCGGTAGA	1	0	0	0
tsrna-23555	GGGGAATTAGCTCAGGCGGTA	0	1	0	0
tsrna-23551	GGGGAATTAGCTCAAGTGGTAGAGCGCTTG	2	1	1	1
tsrna-23547	GCAGTTGACCCGGGTTCGATTCCCGGCCA	1	4	0	0
tsrna-23546	GCAGTTGACCCGGGTTCGA	0	0	1	0
tsrna-23545	GCAGTTGACCCGGGTTCG	0	1	0	0
tsrna-23544	GCAGTTGACCCGGGTTC	0	0	0	0
tsrna-23543	GCAGTGGTAGAATTCTCGCCT	3	0	2	1
tsrna-23542	GCAGTGGTAGAATTCTCGCC	1	0	3	0
tsrna-23541	GCAGTGGTAGAATTCTCGC	1	1	1	0
tsrna-23540	GCAGTGGTAGAATTCTCG	2	1	0	0
tsrna-23539	GCAGTCTCCCCTGGAGGCGTGGGT	0	2	1	0
tsrna-23533	GCAGGTTCGAGTCCTGCCGCGGTCGCCA	2	11	0	0
tsrna-23531	GCAGGTTCGAATCCTGCTCACAGCGCCA	3	17	5	1
tsrna-23529	GCAGGTTCGAATCCTGCTCACAG	0	0	1	0
tsrna-23528	GCAGGTTCGAATCCTGCCGACTACGC	0	2	0	1
tsrna-23525	GCAGGTTCGAACCCTGCTCGCTGCGCCA	10	32	5	0
tsrna-23524	GCAGCTTCAAACCTGCCGGGGCTTCC	1	1	0	0
tsrna-23520	GCAGCGGAAGCGTGCTGGGCCC	4	41	9	7
tsrna-23519	GCAGCGGAAGCGTGCTGGGCC	5	24	3	2
tsrna-23518	GCAGCGGAAGCGTGCTGGGC	5	22	5	0
tsrna-23517	GCAGCGATGGCCGAGTGGTTAAGGCGTTGG	1	4	0	0
tsrna-23516	GCAGCGATGGCCGAGTGGTTAAGGCGTTG	2	5	3	1
tsrna-23515	GCAGCGATGGCCGAGTGGTTAAGGC	1	5	0	0
tsrna-23514	GCAGCGATGGCCGAGTGGTTAAGG	1	5	0	0
tsrna-23513	GCAGCGATGGCCGAGTGGTTAAG	4	5	1	1
tsrna-23512	GCAGCGATGGCCGAGTGGTTAA	3	3	1	0
tsrna-23511	GCAGCGATGGCCGAGTGGTTA	0	1	1	1
tsrna-23510	GCAGCGATGGCCGAGTGGTT	3	1	0	0
tsrna-23509	GCAGCGATGGCCGAGTGGT	3	4	1	0
tsrna-23508	GCAGCGATGGCCGAGTGG	1	6	0	0
tsrna-23507	GCAGCGATGGCCGAGT	1	0	1	1
tsrna-23506	GCAGCAACCTCGGTTCGAATCCGAGTCACG	1	0	0	0
tsrna-23502	GCAGCAACCTCGGTTCGAA	0	0	1	0
tsrna-23501	GCAGCAACCTCGGTTCGA	1	0	0	0
tsrna-23500	GCAGCAACCTCGGTTCG	0	0	0	0
tsrna-23498	GCAGATCAAGAGGTCCCTGGTTCA	2	7	1	1
tsrna-23497	GCAGATCAAGAGGTCCCTGGTTC	3	14	3	1
tsrna-23496	GCAGATCAAGAGGTCCCTGGTT	2	8	5	0
tsrna-23495	GCAGATCAAGAGGTCCCTGGT	0	4	6	2
tsrna-23493	GCAGATCAAGAGGTCCCCGGTTCA	0	5	0	1
tsrna-23492	GCAGATCAAGAGGTCCCCGGTTC	0	6	0	0
tsrna-23491	GCAGATCAAGAGGTCCCCGGTT	1	1	0	1
tsrna-23490	GCAGATCAAGAGGTCCCCGGT	0	1	0	1
tsrna-23487	GCAGATCAAGAGGTCCCCAGT	0	1	0	0
tsrna-23483	GCAGATCAAGAAGTCCCCGGTT	1	8	0	0
tsrna-23482	GCAGATCAAGAAGTCCCCGGT	1	7	0	1
tsrna-23480	GCAGATCAAAAGGTCCCTGGT	1	6	1	1
tsrna-23479	GCAGAGTGGGGTTTTGCAGTCCTTACCA	0	1	1	1
tsrna-23478	GCAGAGTGGGGTTTTGCAGTCCTTACC	0	1	0	0
tsrna-23477	GCAGAGTGGGGTTTTGCAGTCCTTAC	0	0	0	0
tsrna-23476	GCAGAGTGGGGTTTTGCAGTCCTTA	0	0	2	0
tsrna-23475	GCAGAGTGGGGTTTTGCAGTCCTT	0	0	1	0
tsrna-23474	GCAGAGTGGGGTTTTGCAGTCCT	2	0	0	0
tsrna-23473	GCAGAGTGGGGTTTTGCAGTCC	0	2	0	0
tsrna-23472	GCAGAGTGGGGTTTTGCAGTC	0	0	0	0
tsrna-23471	GCAGAGTGGGGTTTTGCAGT	1	0	0	0
tsrna-23470	GCAGAGTGGGGTTTTGCAG	0	0	0	0
tsrna-23469	GCAGAGTGGGGTTTTGCA	0	0	1	0
tsrna-23467	GCAGAGTGGGGTTTTG	0	1	0	0
tsrna-23466	GCAGAGTGGCGCAGCGGAAGCGTGCTGGGC	3	28	3	1
tsrna-23465	GCAGAGTGGCGCAGCGGAAGCGTGCTGGG	3	31	1	0
tsrna-23464	GCAGAGTGGCGCAGCGGAAGCGTGCTGG	0	4	0	0
tsrna-23463	GCAGAGTGGCGCAGCGGAAGCGTGCTG	0	2	0	0

tsrna-23451	GCAGAGCCCGGTAATCGCATAAAACTTAA	1	0	2	0
tsrna-23450	GCAGAGCCCGGTAATCGCATAAAACTTA	0	1	2	0
tsrna-23449	GCAGAGCCCGGTAATCGCATAAAACTT	0	0	1	0
tsrna-23448	GCAGAGCCCGGTAATCGCATAAAACT	0	0	0	0
tsrna-23447	GCAGAGCCCGGTAATCGCATAAAAC	0	1	0	0
tsrna-23446	GCAGAGCCCGGTAATCGCATAAAA	0	0	0	1
tsrna-23444	GCAGAGCCCGGTAATCGCATAA	0	0	1	0
tsrna-23442	GCAGAGCCCGGTAATCGCAT	0	1	0	0
tsrna-23437	GCAGAAGGTCCTGGGTTCGAGCCCCAGTGGAACCACCA	55	41	32	27
tsrna-23436	GCAGAAGGTCCTGGGTTCGAGCCCCAGTGGAACCACC	5	10	5	1
tsrna-23435	GCAGAAGGTCCTGGGTTCGAGCCCCAGTGGAACCAC	5	1	2	0
tsrna-23434	GCAGAAGGTCCTGGGTTCGAGCCCCAGTGGAACC	2	2	4	0
tsrna-23433	GCAGAAGGTCCTGGGTTCGAGCCCCAGTGG	1	2	0	0
tsrna-23432	GCAGAAGGTCCTGGGTTCGAGCCCCAGTG	0	3	2	0
tsrna-23431	GCAGAAGGTCCTGGGT	0	3	0	0
tsrna-23430	GCACTGGTGGTTCAGTGGTAGAATTCTCGCCT	137	33	170	8
tsrna-23429	GCACTGGTGGTTCAGTGGTAGAATTCTCGCC	84	30	68	8
tsrna-23428	GCACTGGTGGTTCAGTGGTAGAATTCTCGC	16	7	6	3
tsrna-23427	GCACTGGTGGTTCAGTGGTAGAATTCTCG	4	3	4	5
tsrna-23426	GCACTGGTGGTTCAGTGGTAGAATTCTC	3	1	7	3
tsrna-23425	GCACTGGTGGTTCAGTGGTAGAATTCT	3	3	2	0
tsrna-23424	GCACTGGTGGTTCAGTGGTAGAATTC	2	2	3	0
tsrna-23423	GCACTGGTGGTTCAGTGGTAGAATT	1	4	1	0
tsrna-23422	GCACTGGTGGTTCAGTGGTAGAAT	1	0	3	0
tsrna-23421	GCACTGGTGGTTCAGTGGTAGAA	1	1	0	0
tsrna-23420	GCACTGGTGGTTCAGTGGTAGA	2	1	0	0
tsrna-23416	GCACTCTGGACTTTGAATCCAGC	1	2	4	0
tsrna-23415	GCACTCTGGACTTTGAATCCA	2	3	2	0
tsrna-23414	GCACTCTGGACTTTGAATCC	2	3	2	0
tsrna-23413	GCACTCTGGACTTTGAATC	2	4	0	0
tsrna-23409	GCACTCTGGACTCTGAATCCG	0	2	0	0
tsrna-23408	GCACTCTGGACTCTGAATCCAGCGATCCGAGTTCA	2	2	1	0
tsrna-23407	GCACTCTGGACTCTGAATCCAGCGATCCGAGTTC	1	3	2	0
tsrna-23406	GCACTCTGGACTCTGAATCCAGCGATCCGAGTT	2	3	1	1
tsrna-23405	GCACTCTGGACTCTGAATCCAGCGATCCGAGT	2	3	1	1
tsrna-23404	GCACTCTGGACTCTGAATCCAGCGATCCGAG	1	6	0	1
tsrna-23403	GCACTCTGGACTCTGAATCCAGCGATCCG	1	2	0	1
tsrna-23402	GCACTCTGGACTCTGAATCCAGCGATCC	2	4	2	0
tsrna-23401	GCACTCTGGACTCTGAATCCAGCGATC	0	5	1	1
tsrna-23400	GCACTCTGGACTCTGAATCCAGCGAT	0	2	0	0
tsrna-23399	GCACTCTGGACTCTGAATCCAGCGA	0	4	0	1
tsrna-23398	GCACTCTGGACTCTGAATCCAGCG	0	4	0	0
tsrna-23397	GCACTCTGGACTCTGAATCCAGC	0	3	0	0
tsrna-23396	GCACTCTGGACTCTGAATCCAG	1	3	2	1
tsrna-23395	GCACTCTGGACTCTGAATCCA	0	2	0	0
tsrna-23394	GCACTCTGGACTCTGAATCC	0	1	0	0
tsrna-23392	GCACTCTGGACTCTGAAT	0	1	0	0
tsrna-23389	GCACGTATGAGGCCCCGGGTT	1	4	1	2
tsrna-23388	GCACGTATGAGGCCCCGGGT	1	4	0	1
tsrna-23386	GCACGAGGTCCTGGGTTCGATC	2	9	0	3
tsrna-23385	GCACGAGGTCCTGGGTTCGAT	0	10	1	0
tsrna-23384	GCACGAGGTCCTGGGTT	2	6	4	1
tsrna-23383	GCACGAGGTCCTGGGT	2	8	1	1
tsrna-23382	GCACGAGGCCCCGGGTTCAATCCCCGGCACCT	0	1	0	0
tsrna-23381	GCACGAGGCCCCGGGTTCAATC	1	0	0	0
tsrna-23380	GCACGAGGCCCCGGGTTCAA	1	2	2	0
tsrna-23379	GCACGAGGCCCCGGGTTCA	0	1	0	0
tsrna-23378	GCACGAGGCCCCGGGTTC	0	1	0	0
tsrna-23377	GCACGAGGCCCCGGGTT	0	0	1	0
tsrna-23375	GCACCCTGGACTCTGAATCCAGC	1	1	0	0
tsrna-23373	GCACCCAACTTACACTTAGGAGATTTCAACTTAACTTGACCGC	1	3	11	0
tsrna-23372	GCACCCAACTTACACTTAGGAGATTTCAACTTAACTTGACC	1	0	11	0
tsrna-23371	GCACCCAACTTACACTTAGGAGATTTCAAC	0	0	0	0
tsrna-23369	GCACCCAACTTACACTTAGGAGATTTCA	0	1	0	0

tsrna-23366	GCACCCAACTTACACTTAGGAGATT	1	0	0	0
tsrna-23356	GCAATGGTGGTTCAGTGGTAGAATTCTCGCCTT	48	25	63	6
tsrna-23355	GCAATGGTGGTTCAGTGGTAGAATTCTCGCCT	45	21	35	1
tsrna-23354	GCAATGGTGGTTCAGTGGTAGAATTCTCGCC	28	17	33	2
tsrna-23353	GCAATGGTGGTTCAGTGGTAGAATTCTCGC	11	5	6	3
tsrna-23352	GCAATGGTGGTTCAGTGGTAGAATTCTCG	5	4	5	0
tsrna-23351	GCAATGGTGGTTCAGTGGTAGAATTCTC	0	4	2	0
tsrna-23350	GCAATGGTGGTTCAGTGGTAGAATTCT	0	0	1	0
tsrna-23349	GCAATGGTGGTTCAGTGGTAGAATTC	1	3	2	1
tsrna-23348	GCAATGGTGGTTCAGTGGTAGAATT	3	1	1	0
tsrna-23347	GCAATGGTGGTTCAGTGGTAGAAT	3	2	1	2
tsrna-23346	GCAATGGTGGTTCAGTGGTAGAA	1	1	0	0
tsrna-23342	GCAATGGATAGCGCATTGGACT	9	8	3	3
tsrna-23341	GCAATGGATAGCGCATTGGA	4	12	4	1
tsrna-23340	GCAATGGATAGCGCATTGG	4	4	2	1
tsrna-23339	GCAATGGATAGCGCATTG	0	0	0	0
tsrna-23333	GCAATACTTAATTTCTGCCA	3	0	3	1
tsrna-23332	GCAATACTTAATTTCTGCC	1	0	0	0
tsrna-23329	GCAATACACTGAAAATGTTTAGACGGGCTCAC	29	15	10	0
tsrna-23328	GCAATACACTGAAAATGTTTAGACGGGCTC	4	6	2	4
tsrna-23327	GCAAGATTCCCATTCTTGCGACCCG	0	2	1	1
tsrna-23326	GCAAGATTCCCATTCTTGCGA	1	1	0	0
tsrna-23318	GCAAATTCGAAGAAGCAGCTTCAAACCTGCCGGGGCTT	1	2	2	0
tsrna-23317	GCAAATTCGAAGAAGCAGCTTCAAACCTGC	1	0	0	0
tsrna-23316	GCAAATTCGAAGAAGCAGCTTC	0	0	0	0
tsrna-23314	GCAAATTCGAAGAAGCAGCT	0	0	1	0
tsrna-23308	GATTTGCGTTCAGTTGATGCAGAGTGGGGT	1	0	0	0
tsrna-23307	GATTTGCGTTCAGTTGATGCAGA	0	0	1	0
tsrna-23306	GATTTGCGTTCAGTTGATGCAG	1	1	0	0
tsrna-23305	GATTTGCGTTCAGTTGATGCA	0	0	0	0
tsrna-23300	GATTTCGACTCATTAAATTATGATAATC	3	1	3	0
tsrna-23299	GATTTCGACTCATTAAATTATGATA	5	3	2	0
tsrna-23298	GATTTCGACTCATTAAATTATGA	0	0	1	0
tsrna-23297	GATTTCGACTCATTAAATTATG	0	0	0	0
tsrna-23296	GATTTCGACTCATTAAATTA	0	0	1	0
tsrna-23292	GATTTCAACTTAACTTGACCGCTCTGACCA	25	29	38	10
tsrna-23291	GATTTCAACTTAACTTGACCGCTCTGACC	1	4	5	2
tsrna-23290	GATTTCAACTTAACTTGACCGCTCTGAC	0	2	2	0
tsrna-23289	GATTTCAACTTAACTTGACCGCTCTGA	1	0	0	0
tsrna-23288	GATTTCAACTTAACTTGACCGCTCTG	0	1	2	0
tsrna-23287	GATTTCAACTTAACTTGACCGCTCT	0	0	0	0
tsrna-23286	GATTTCAACTTAACTTGACCGCTC	0	0	1	0
tsrna-23276	GATTTAGGCTCCAGTCTCTTCGGAGGCGTG	1	0	0	0
tsrna-23275	GATTTAGGCTCCAGTCTCTTCGG	0	1	0	0
tsrna-23268	GATTGTGAATCTGACAACAGAGGCTTACGACCCCTTATTTACC	31	13	24	1
tsrna-23267	GATTGTGAATCTGACAACAGAGGCTTACGACCCCTTATTTACC	20	5	27	0
tsrna-23266	GATTGTGAATCTGACAACAGAGGCTTACGACCCCTTATTTACC	30	9	32	0
tsrna-23265	GATTGTGAATCTGACAACAGAGGCTTACGACCCCTTATTTACC	26	16	33	0
tsrna-23264	GATTGTGAATCTGACAACAGAGGCTTACGACCCCTT	2	3	6	0
tsrna-23263	GATTGTGAATCTGACAACAGAGGCTTACGACCCCT	5	3	5	0
tsrna-23262	GATTGTGAATCTGACAACAGAGGCTTACGACCCC	2	4	3	0
tsrna-23261	GATTGTGAATCTGACAACAGAGGCTTACGACCC	0	3	4	0
tsrna-23260	GATTGTGAATCTGACAACAGAGGCTTACGACC	1	0	0	0
tsrna-23259	GATTGTGAATCTGACAACAGAGGCTTACGA	0	4	1	1
tsrna-23258	GATTGTGAATCTGACAACAGAGGCTTACG	0	1	1	0
tsrna-23257	GATTGTGAATCTGACAACAGAGGCTTAC	0	4	2	0
tsrna-23256	GATTGTGAATCTGACAACAGAGGCTTA	1	4	0	0
tsrna-23255	GATTGTGAATCTGACAACAGAGGCTT	0	2	1	0
tsrna-23254	GATTGTGAATCTGACAACAGAGGCT	0	5	2	0
tsrna-23253	GATTGTGAATCTGACAACAGAGGC	0	4	1	0
tsrna-23252	GATTGTGAATCTGACAACAGAGG	2	2	1	0
tsrna-23251	GATTGTGAATCTGACAACAGAG	1	1	1	0
tsrna-23250	GATTGTGAATCTGACAACAGA	0	1	0	0
tsrna-23249	GATTGTGAATCTGACAACAG	1	1	0	0

tsrna-23248	GATTGTGAATCTGACAACA	0	0	1	0
tsrna-23247	GATTGTGAATCTGACAAC	0	2	0	0
tsrna-23246	GATTGTGAATCTGACAA	0	1	0	0
tsrna-23245	GATTGTGAATCTGACA	0	3	1	1
tsrna-23244	GATTGCAGGTTCGAGTCCTGCCGCGGTCGCCA	2	5	3	0
tsrna-23240	GATTGAGGGTTCGAGTCCCTTCGTGGTCGCCA	2	1	0	0
tsrna-23239	GATTGAGGGTTCGAGTCCCTTCGTGGTCGCC	0	1	0	0
tsrna-23234	GATTGAGGGTTCGAATCCCTCCGTGGTTAC	1	1	0	0
tsrna-23233	GATTGAAGCCAGTTGATTAGGGTGCTTAGC	0	0	0	0
tsrna-23231	GATTGAAGCCAGTTGATTAG	0	0	0	0
tsrna-23230	GATTGAAGCCAGTTGATTA	0	0	0	0
tsrna-23225	GATTCTCATAGTCCTAGCCA	0	0	0	0
tsrna-23224	GATTCTCATAGTCCTAGCC	0	0	0	0
tsrna-23223	GATTCTCATAGTCCTAGC	1	1	0	0
tsrna-23221	GATTCTCAGGGATGGGTTCG	0	2	1	0
tsrna-23220	GATTCTAGGTTCGACTCCTGGCTGGCTCGC	0	0	1	0
tsrna-23219	GATTCTAGGTTCGACTCCTGGCTGGCTCG	0	1	0	0
tsrna-23216	GATTCTAGGTTCGACTCCTG	0	0	0	0
tsrna-23215	GATTCGGCGCTTTCACCGCCGCGGCCC	1	5	1	5
tsrna-23214	GATTCGGCGCTCTCACCGCCGCGGCCCGGGTT	1	5	0	1
tsrna-23213	GATTCGGCGCTCTCACCGCCGCGGCCCGGGT	0	1	0	0
tsrna-23212	GATTCGGCGCTCTCACCGCCGCGGCCCGGG	1	2	0	0
tsrna-23211	GATTCGGCGCTCTCACCGCCGCGGCCCGG	0	2	1	0
tsrna-23210	GATTCGGCGCTCTCACCGCCGCGGCCCG	1	0	0	0
tsrna-23209	GATTCGGCGCTCTCACCGCCGCGGCCC	0	0	0	0
tsrna-23208	GATTCGGCGCTCTCACCGCCGCGGC	0	1	0	0
tsrna-23207	GATTCGGCGCTCTCACCGCCGCGG	1	2	1	0
tsrna-23206	GATTCGGCGCTCTCACCGCCGC	1	2	0	0
tsrna-23205	GATTCGGCGCTCTCACCGCCG	0	0	0	1
tsrna-23204	GATTCGGCGCTCTCACCGCC	0	0	0	0
tsrna-23203	GATTCGGCGCTCTCACCGC	1	0	0	0
tsrna-23202	GATTCGGCGCTCTCACCG	0	0	0	0
tsrna-23201	GATTCGGCGCTCTCACC	0	0	0	0
tsrna-23200	GATTCCTTCCTTTTTTGCCA	0	1	0	0
tsrna-23198	GATTCCTTCCTTTTTTGC	0	0	0	0
tsrna-23195	GATTCCTGGTTTTCACCCA	1	1	0	0
tsrna-23193	GATTCCGGCTCGAAGGACCA	58	129	28	9
tsrna-23192	GATTCCGGCTCGAAGGACC	3	8	0	0
tsrna-23191	GATTCCGGCTCGAAGGAC	0	1	0	0
tsrna-23188	GATTCCGGATCAGAAGATTGAGGGT	9	5	4	3
tsrna-23182	GATTCCCGGTCAGGGAACCA	4	2	0	2
tsrna-23178	GATTCCCGGGCGGCGCACCA	7	34	6	0
tsrna-23176	GATTCCCGGCCCATGCACCA	6	10	6	5
tsrna-23175	GATTCCCGGCCCATGCACC	1	2	0	0
tsrna-23173	GATTCCCGGCCAATGCACCA	2	4	2	0
tsrna-23172	GATTCCCGGCCAATGCACC	2	1	0	0
tsrna-23170	GATTCCCGGCCAACGCACCA	0	1	1	2
tsrna-23169	GATTCCCGGCCAACGCACC	1	1	0	1
tsrna-23165	GATTCCCCGACGGGGAGCCA	1	0	0	0
tsrna-23160	GATTCCCATTCTTGCGACCCGGGTTCG	4	9	0	0
tsrna-23159	GATTCCCATTCTTGCGACCCGGGTT	3	3	1	0
tsrna-23158	GATTCCCATTCTTGCGACCCGGGT	1	1	0	0
tsrna-23157	GATTCCCATTCTTGCGACCCGGG	0	2	0	0
tsrna-23156	GATTCCCATTCTTGCGACCCGG	1	0	0	1
tsrna-23155	GATTCCCATTCTTGCGACCCG	1	0	0	0
tsrna-23154	GATTCCCATTCTTGCGACCC	3	2	0	0
tsrna-23153	GATTCCCATTCTTGCGACC	0	2	0	0
tsrna-23152	GATTCCCATTCTTGCGAC	0	1	1	0
tsrna-23151	GATTCCCATTCTTGCGA	0	0	0	0
tsrna-23150	GATTCCCATTCTTGCG	1	0	0	0
tsrna-23148	GATTCCAGGTTCGACTCCTGGCTGGCTCG	0	0	0	0
tsrna-23135	GATTCATATCCGCGTGGGT	1	4	0	1
tsrna-23134	GATTAGGGTGCTTAGCTGTTAACTAAGTGT	8	1	7	0
tsrna-23133	GATTAGGGTGCTTAGCTGTTAACTAAG	3	3	6	0

tsrna-23132	GATTAGGGTGCTTAGCTGTTAACTA	3	1	9	0
tsrna-23131	GATTAGGGTGCTTAGCTGTTAACT	5	1	8	0
tsrna-23130	GATTAGGGTGCTTAGCTGTTAAC	1	0	2	0
tsrna-23129	GATTAGGGTGCTTAGCTGTTAA	0	1	2	0
tsrna-23128	GATTAGGGTGCTTAGCTGTTA	3	1	1	0
tsrna-23127	GATTAGGGTGCTTAGCTGTT	0	0	0	0
tsrna-23126	GATTAGGGTGCTTAGCTGT	0	2	1	0
tsrna-23122	GATTAGCTCAAATGGTAGAGCGCTCGCTT	0	0	0	0
tsrna-23116	GATTAAGGCTCCAGTCTCT	1	0	0	0
tsrna-23115	GATTAAGAGAACCAACACCTCTTTACAGTGACCA	1	1	1	0
tsrna-23112	GATTAAGAGAACCAACACCTCTTTACAGTG	0	0	0	0
tsrna-23110	GATTAAGAGAACCAACACCTCTTTACAG	0	0	0	0
tsrna-23108	GATTAAGAGAACCAACACCTCTTTAC	0	1	0	0
tsrna-23097	GATGTAGCTCAGTGGTAGAGCGCAT	1	0	0	0
tsrna-23093	GATGTAGCTCAGTGGTAGAG	0	0	0	0
tsrna-23089	GATGGTTTTTCATATCATTGGTCGTGGTTGTAGTCCGTGCGAC	10	13	17	16
tsrna-23088	GATGGTTTTTCATATCATTGGTCGTGGTTGTAGTCCGTGCGAC	10	19	18	13
tsrna-23087	GATGGTTTTTCATATCATTGGTCGTGGTTGTAGTCCGTGCGAC	8	14	23	8
tsrna-23086	GATGGTTTTTCATATCATTGGTCGTGGTTGTAGTCC	1	3	6	0
tsrna-23085	GATGGTTTTTCATATCATTGGTCGTGGTTGTAGTC	1	1	0	1
tsrna-23084	GATGGTTTTTCATATCATTGGTCGTGGTTGTAGT	1	1	1	0
tsrna-23083	GATGGTTTTTCATATCATTGGTCGTGGTTG	0	1	1	1
tsrna-23082	GATGGTTTTTCATATCATTGGTCGTGGT	2	0	0	0
tsrna-23081	GATGGTTTTTCATATCATTGGTCGTGG	0	1	0	0
tsrna-23075	GATGGGTTCGATTCTCATAGTCCTAGCCA	1	5	3	1
tsrna-23074	GATGGGTTCGATTCTCATAGTCCTAGC	0	5	1	4
tsrna-23073	GATGGGTTCGATTCTCATAGTCCTAG	0	3	3	1
tsrna-23072	GATGGGTTCGATTCTCATAGTCC	0	2	2	1
tsrna-23071	GATGGGTTCGATTCTCATAGTC	0	1	0	0
tsrna-23070	GATGGGGTGTGATAGGTGGCACGGAGAATTTTG	1	2	1	1
tsrna-23069	GATGGGGTGTGATAGGTGGCACGGAGA	0	0	0	0
tsrna-23068	GATGGGGTGTGATAGGTGGCACGGAG	0	0	0	0
tsrna-23058	GATGGCGTGGGTTCGAATCCCACC	0	0	0	0
tsrna-23054	GATGGCCGAGTGGTCTAAGGCGCCAGAC	1	0	0	0
tsrna-23047	GATGGCCGAGCGGTCTAAGGCGCTGCGTTC	0	1	0	0
tsrna-23046	GATGGCCGAGCGGTCTAAGGCGCTGCGTT	0	1	0	0
tsrna-23033	GATGGCAGAGCCCGGTAATCGCATAAA	1	0	0	0
tsrna-23030	GATGGCAGAGCCCGGTAATCGCA	0	1	0	0
tsrna-23022	GATGGATCGAAACCATCCTCTGCTACCA	16	22	15	13
tsrna-23020	GATGGAAACCATCCTCTGCTACC	0	0	0	0
tsrna-23019	GATGCGAGAGGTCCCGGGT	0	0	1	0
tsrna-23017	GATGCCCGCATTCTCCACCA	1	3	0	0
tsrna-23016	GATGCCCGCATCCTCCACCA	0	2	1	0
tsrna-23015	GATGCCCGCATCCTCCACC	1	0	1	0
tsrna-23012	GATGCAGAGTGGGGTTTTGCAGTCCTTACC	3	0	0	0
tsrna-23011	GATGCAGAGTGGGGTTTTGCAGTCCTTAC	2	2	1	0
tsrna-23010	GATGCAGAGTGGGGTTTTGCAGTCCTTA	1	1	0	0
tsrna-23009	GATGCAGAGTGGGGTTTTGCAGTCCTT	0	1	1	0
tsrna-23008	GATGCAGAGTGGGGTTTTGCAGTCCT	2	0	1	1
tsrna-23007	GATGCAGAGTGGGGTTTTGCAGTCC	0	0	0	0
tsrna-23006	GATGCAGAGTGGGGTTTTGCAGTC	1	2	2	0
tsrna-23005	GATGCAGAGTGGGGTTTTGCAGT	0	0	0	0
tsrna-23004	GATGCAGAGTGGGGTTTTGCAG	0	1	0	0
tsrna-23003	GATGCAGAGTGGGGTTTTGCA	0	0	0	0
tsrna-23002	GATGCAGAGTGGGGTTTTGC	0	0	0	0
tsrna-22997	GATGAAAACCTTTTTCCAAGGACACCA	3	4	3	0
tsrna-22996	GATGAAAACCTTTTTCCAAGGACACC	1	0	0	0
tsrna-22994	GATGAAAACCTTTTTCCAAGGACA	0	0	0	0
tsrna-22987	GATCGTATAGTGGTTAGTACTCTGCGTTGT	29	9	13	9
tsrna-22986	GATCGTATAGTGGTTAGTACTCTGCGTTG	21	13	7	6
tsrna-22985	GATCGTATAGTGGTTAGTACTCTGCGTT	16	9	7	6
tsrna-22984	GATCGTATAGTGGTTAGTACTCTGCGT	11	4	3	1
tsrna-22983	GATCGTATAGTGGTTAGTACTCTGCGCTG	12	4	5	2
tsrna-22982	GATCGTATAGTGGTTAGTACTCTGCG	2	4	0	1

tsrna-22981	GATCGTATAGTGGTTAGTACTCTGC	2	1	2	1
tsrna-22980	GATCGTATAGTGGTTAGTACTCTG	2	2	0	0
tsrna-22979	GATCGTATAGTGGTTAGTACTCT	1	0	3	0
tsrna-22978	GATCGTATAGTGGTTAGTACTC	1	1	0	1
tsrna-22977	GATCGTATAGTGGTTAGTACT	1	0	1	0
tsrna-22976	GATCGTATAGTGGTTAGTAC	0	0	0	0
tsrna-22971	GATCGATGCCCGCATTCTCCACCA	2	3	0	0
tsrna-22970	GATCGATGCCCGCATTCTCCACC	0	1	0	0
tsrna-22968	GATCGATGCCCGCATCCTCCACCA	1	6	1	0
tsrna-22960	GATCGAAACCATCCTCTGCTACCA	19	20	8	11
tsrna-22959	GATCGAAACCATCCTCTGCTACC	3	6	2	2
tsrna-22958	GATCGAAACCATCCTCTGCTAC	1	0	0	0
tsrna-22952	GATCCTTAGGTCGCTGGTTCG	0	0	0	0
tsrna-22946	GATCCTCGCTGGGGCCTCCA	6	8	4	5
tsrna-22945	GATCCTCACCTGGAGCACCA	1	2	0	0
tsrna-22944	GATCCTCACACGGGGCACCA	0	4	0	0
tsrna-22943	GATCCGAGTTCAAATCTCGGTGGAACCTCC	10	11	7	2
tsrna-22942	GATCCCGGGTTTCGGCACCA	44	67	9	13
tsrna-22941	GATCCCGGGTTTCGGCACC	5	3	0	0
tsrna-22937	GATCCCGGGCGGAAACACCA	16	28	11	5
tsrna-22936	GATCCCCGTACTGGCCACCA	1	2	3	0
tsrna-22935	GATCCCCGTACGGGCCACCA	10	58	3	0
tsrna-22934	GATCCCCGGCATCTCCACCA	102	111	43	40
tsrna-22933	GATCCCCGGCATCTCCACC	3	7	2	2
tsrna-22930	GATCCCCGGCACCTCCACCA	140	233	87	20
tsrna-22929	GATCCCCGACACCTCCACCA	3	6	0	1
tsrna-22928	GATCCCCAGTACCTCCACCA	1	2	0	0
tsrna-22927	GATCCCCAGTACCTCCACC	0	0	1	0
tsrna-22926	GATCCCCAGCATCTCCACCA	1	5	1	0
tsrna-22923	GATCCCACCCAGGGACGCCA	1	6	0	1
tsrna-22922	GATCAGAAGGCTGCGTGTTCGAA	0	1	0	0
tsrna-22921	GATCAGAAGGCTGCGTGTTCG	1	0	1	0
tsrna-22920	GATCAGAAGGCTGCGTGTTC	0	1	0	0
tsrna-22918	GATCAGAAGATTCTAGGTTCGACTCCTGGC	2	11	1	0
tsrna-22917	GATCAGAAGATTCCAGGTTC	0	0	1	0
tsrna-22916	GATCAAGAGGTCCCTGGTT	4	2	1	3
tsrna-22915	GATCAAGAGGTCCCCGGTTCA	1	5	2	0
tsrna-22914	GATCAAGAGGTCCCCGGTTC	0	5	2	0
tsrna-22913	GATCAAGAGGTCCCCGGTT	1	2	0	2
tsrna-22912	GATCAAGAGGTCCCCGGT	1	4	1	0
tsrna-22911	GATCAAAAGGTCCCTGGTT	9	9	2	8
tsrna-22910	GATCAAAACCAGGCGGAAACACCA	6	6	3	0
tsrna-22909	GATAGGTGGCACGGAGAATTTTGGATT	1	4	1	0
tsrna-22908	GATAGGTGGCACGGAGAATTTTGGAT	1	2	1	0
tsrna-22907	GATAGGTGGCACGGAGAATTTTGGA	1	1	1	0
tsrna-22906	GATAGGTGGCACGGAGAATTTTGG	0	1	1	0
tsrna-22905	GATAGGTGGCACGGAGAATTTTG	0	2	0	0
tsrna-22904	GATAGGTGGCACGGAGAATTTT	0	0	0	0
tsrna-22903	GATAGGTGGCACGGAGAATTT	0	0	0	0
tsrna-22902	GATAGGTGGCACGGAGAATT	0	0	0	0
tsrna-22898	GATAGCTCAGTTGGTAGAGCGGAGGACTGT	0	1	2	1
tsrna-22896	GATAGCTCAGTTGGTAGAGC	1	2	1	0
tsrna-22895	GATAGCTCAGTTGGTAGAG	0	0	0	1
tsrna-22894	GATAGCTCAGTTGGTAGAACA	0	0	1	0
tsrna-22893	GATAGCTCAGTTGGGAGAGC	0	1	0	0
tsrna-22891	GATAGCTCAGTCGGTAGAGCATCAGACTTTT	28	10	18	18
tsrna-22890	GATAGCTCAGTCGGTAGAGCATCAGACTTT	9	20	6	1
tsrna-22889	GATAGCTCAGTCGGTAGAGCATCAGACT	11	7	12	2
tsrna-22888	GATAGCTCAGTCGGTAGAGCATCAGAC	9	12	6	2
tsrna-22887	GATAGCTCAGTCGGTAGAGCATC	5	4	3	1
tsrna-22881	GATAGCTCAGCTGGTAGAGCGGAGGACTGT	0	0	0	0
tsrna-22878	GATAGAGTAAATAATAGGAGCTTAAACCCC	0	0	1	0
tsrna-22877	GATAGAGTAAATAATAGGAGCTTAAACCC	0	0	0	0
tsrna-22876	GATAGAGTAAATAATAGGAGCTTA	1	0	1	0

tsrna-22875	GATAGAGTAAATAATAGGAGCTT	0	0	0	1
tsrna-22874	GATAGAGTAAATAATAGGAGCT	0	0	1	0
tsrna-22870	GATAATCATATTTACCAACCA	0	0	0	0
tsrna-22863	GATAAGGCGTCTGACTTCG	1	0	0	0
tsrna-22855	GATAACAGCTATCCATTGGTCTTAGGCCCC	1	1	1	1
tsrna-22854	GATAACAGCTATCCATTGGTCTTAGGCCC	0	0	2	0
tsrna-22853	GATAACAGCTATCCATTGGTCTTAGGCC	0	0	0	0
tsrna-22852	GATAACAGCTATCCATTGGTCTTAGGC	0	0	1	0
tsrna-22851	GATAACAGCTATCCATTGGTCTTAGG	0	0	0	0
tsrna-22850	GATAACAGCTATCCATTGGTCTTAG	0	0	0	0
tsrna-22849	GATAACAGCTATCCATTGGTCTTA	0	0	0	0
tsrna-22848	GATAACAGCTATCCATTGGTCTT	0	0	0	0
tsrna-22841	GATAACACCAAGGTCGCGGGCTCG	0	1	0	0
tsrna-22840	GATAACACCAAGGTCGCGGGCT	2	2	0	0
tsrna-22838	GATAAAAGAGTTACTTTGATAGAGTAAATAATAGGAGCT	1	5	5	0
tsrna-22837	GATAAAAGAGTTACTTTGATAGAGTA	1	4	2	0
tsrna-22836	GATAAAAGAGTTACTTTGATAGAG	2	2	1	0
tsrna-22835	GATAAAAGAGTTACTTTGATAGA	0	4	1	0
tsrna-22834	GATAAAAGAGTTACTTTGATAG	1	0	0	1
tsrna-22832	GAGTTCGATCCTCACACGGGGCACCA	0	1	0	0
tsrna-22830	GAGTTCGAGTCTCGGTGGAACCTCCA	65	136	20	37
tsrna-22829	GAGTTCGAGTCTCGGTGGAACCTC	2	1	0	0
tsrna-22828	GAGTTCGAGTCTCGGTGGAAC	0	2	0	0
tsrna-22826	GAGTTCGAGCCTCACCTGGAGCACCA	0	3	0	0
tsrna-22823	GAGTTCGAACCTCAGAGGGGGCACCA	0	7	0	0
tsrna-22821	GAGTTCAAATCTCGGTGGGACCTCCA	78	90	25	33
tsrna-22820	GAGTTCAAATCTCGGTGGAACCTCCA	166	219	48	24
tsrna-22819	GAGTTCAAATCTCGGTGGAACCTC	1	3	1	0
tsrna-22818	GAGTTCAAATCTCGGTGGAACCT	0	1	0	0
tsrna-22815	GAGTTCAAATCTCGCTGGGGCCTC	0	0	0	0
tsrna-22814	GAGTTAAAGACTTTTTCTCTGACCA	20	34	14	11
tsrna-22813	GAGTTAAAGACTTTTTCTCTGACC	3	15	0	3
tsrna-22812	GAGTTAAAGACTTTTTCTCTGAC	1	0	0	0
tsrna-22810	GAGTTAAAGACTTTTTCTCTG	0	0	0	0
tsrna-22792	GAGTGGTCTAAGGCGCTGGATT	0	1	1	1
tsrna-22788	GAGTGGGGTTTTGCAGTCCTTACCA	1	4	2	0
tsrna-22787	GAGTGGGGTTTTGCAGTCCTTACC	2	1	0	0
tsrna-22786	GAGTGGGGTTTTGCAGTCCTTAC	1	2	0	0
tsrna-22785	GAGTGGGGTTTTGCAGTCCTTA	0	0	0	0
tsrna-22784	GAGTGGGGTTTTGCAGTCCTT	0	0	0	0
tsrna-22783	GAGTGGGGTTTTGCAGTCCT	0	1	1	0
tsrna-22782	GAGTGGGGTTTTGCAGTCC	0	0	0	0
tsrna-22778	GAGTGGCGCAGCGGAAGCGTGCTGGGCCC	12	56	4	6
tsrna-22777	GAGTGGCGCAGCGGAAGCGTGCTGGGCC	4	32	0	0
tsrna-22776	GAGTGGCGCAGCGGAAGCGTGCTGGGC	2	26	1	1
tsrna-22775	GAGTGGCGCAGCGGAAGCGTGCTGGG	4	26	1	0
tsrna-22774	GAGTGGCGCAGCGGAAGCGTGCTGG	0	6	0	0
tsrna-22769	GAGTGAAGCATTGGACTGTAAATCTAAAGA	2	1	1	0
tsrna-22768	GAGTGAAGCATTGGACTGTAAATCTAAAG	2	0	1	0
tsrna-22767	GAGTGAAGCATTGGACTGTAAATCTAAA	0	2	0	0
tsrna-22766	GAGTGAAGCATTGGACTGTAAATCTAA	1	2	1	1
tsrna-22765	GAGTGAAGCATTGGACTGTAAATCTA	1	1	1	0
tsrna-22764	GAGTGAAGCATTGGACTGTAAATCT	0	1	0	1
tsrna-22763	GAGTGAAGCATTGGACTGTAAATC	0	2	1	0
tsrna-22762	GAGTGAAGCATTGGACTGTAAAT	1	0	0	0
tsrna-22761	GAGTGAAGCATTGGACTGTAAA	0	1	0	0
tsrna-22760	GAGTGAAGCATTGGACTGTAA	1	1	0	0
tsrna-22759	GAGTGAAGCATTGGACTGTA	0	0	0	1
tsrna-22754	GAGTCTCGGTGGAACCTCCA	30	103	21	10
tsrna-22753	GAGTCTCGGTGGAACCTCC	0	5	3	1
tsrna-22751	GAGTCCTGCCGCGGTCGCCA	5	6	0	0
tsrna-22750	GAGTCCTGCCGCGGTCGCC	0	0	1	0
tsrna-22746	GAGTCCCGGCGGAGTCGCCA	0	3	0	0
tsrna-22744	GAGTCCCATCTGGGTCGCCA	0	4	0	0

tsrna-22743	GAGTCCCATCTGGGGTGCCA	0	1	0	0
tsrna-22742	GAGTCCCACCAGAGTCGCCA	0	4	0	0
tsrna-22739	GAGTATCCCCGCCTGTCACGCGGGAGACCG	14	68	11	1
tsrna-22738	GAGTATCCCCGCCTGTCACGCGGGAGACC	16	54	9	2
tsrna-22737	GAGTATCCCCGCCTGTCACGC	0	1	0	0
tsrna-22736	GAGTATCCCCGCCTGTCACG	1	0	0	0
tsrna-22734	GAGTATCCCCGCCTGTCA	0	0	0	1
tsrna-22733	GAGTATCCCCGCCTGTC	0	0	1	0
tsrna-22731	GAGTAAATAATAGGAGCTTAAACCCCCTT	1	0	0	0
tsrna-22730	GAGTAAATAATAGGAGCTTAAACCC	0	1	0	0
tsrna-22729	GAGTAAATAATAGGAGCTTAAACC	1	0	1	1
tsrna-22726	GAGTAAATAATAGGAGCTTA	0	0	0	0
tsrna-22725	GAGTAAATAATAGGAGCTT	0	0	0	0
tsrna-22721	GAGGTTCCGGGTTCGAGTCCCGGC	0	0	0	0
tsrna-22720	GAGGTTCAATTCCTCTTCT	0	2	0	0
tsrna-22719	GAGGTTCAATTCCTCTT	0	1	0	0
tsrna-22717	GAGGTGGCCGAGTGGTTAAGGC	0	1	0	0
tsrna-22710	GAGGTCGATGGATCGAAACCATCCTCTGCT	0	0	0	0
tsrna-22709	GAGGTCGATGGATCGAAACCATCC	0	1	0	0
tsrna-22707	GAGGTCGATGGATCGAAACCA	0	0	0	0
tsrna-22702	GAGGTCCTGGGTTCGATCCCC	0	1	0	0
tsrna-22701	GAGGTCCCTGGTTCAAATCCGGGTGC	0	1	0	0
tsrna-22696	GAGGTCCCGGGTTCGATCCCCGGCATC	0	0	0	1
tsrna-22695	GAGGTCCCGGGTTCGATCCCCGGCAT	0	1	0	0
tsrna-22689	GAGGTCCCGGGTTCGATCCCCA	0	1	0	0
tsrna-22687	GAGGTCCCGGGTTCGATCCC	0	0	0	1
tsrna-22684	GAGGTCCCGGGTTCAAATCCCGGACGAGCC	2	1	1	0
tsrna-22683	GAGGTCCCGGGTTCAAATCCCGGACGAGC	0	3	0	0
tsrna-22678	GAGGTCCCCGGTTCAAA	0	0	0	0
tsrna-22677	GAGGTATGATTCTCGCTT	0	1	1	0
tsrna-22676	GAGGGTTCGAGTCCCTTCGTGGTCGCCA	0	4	0	0
tsrna-22674	GAGGGTTCGAGTCCCTTCGTGGTCGC	0	1	0	0
tsrna-22673	GAGGGTTCGAATCCCTTCGTGGTTGC	0	0	0	0
tsrna-22669	GAGGGTCCAGGGTTCATGTCCC	0	0	0	0
tsrna-22665	GAGGGTCCAGGGTTCAAGTCCCTGTTCGGGCGCCA	5	12	4	0
tsrna-22660	GAGGGTCCAGGGTTCAAGTCCCT	0	0	0	0
tsrna-22658	GAGGGTCCAGGGTTCAAGTC	0	1	0	0
tsrna-22657	GAGGGTCCAGGGTTCA	1	0	0	0
tsrna-22656	GAGGCTTACGACCCCTTATTTACCCCA	1	3	0	0
tsrna-22645	GAGGCGTGGGTTCGAATCCCACTTCTGAC	0	0	0	0
tsrna-22644	GAGGCGTGGGTTCGAATCCCACT	0	0	1	0
tsrna-22643	GAGGCGTGGGTTCGAATCCCAC	0	0	0	1
tsrna-22636	GAGGCCCCGGGTTCGATCCCCGGCATCTCC	1	2	0	0
tsrna-22635	GAGGCCCCGGGTTCGATCCCCGGCATC	0	1	0	0
tsrna-22626	GAGGCCCCGGGTTCAATCCCC	0	1	0	0
tsrna-22623	GAGGACTGTAGATCCTTAGGTCGCTGGTTC	2	1	0	0
tsrna-22622	GAGCTTAAACCCCCTTATTTCTACCA	2	2	3	0
tsrna-22621	GAGCTTAAACCCCCTTATTTCTACC	2	2	0	0
tsrna-22620	GAGCTTAAACCCCCTTATTTCTAC	0	3	0	0
tsrna-22619	GAGCTTAAACCCCCTTATTTCTA	2	0	1	0
tsrna-22618	GAGCTTAAACCCCCTTATTTCT	0	2	0	0
tsrna-22617	GAGCTTAAACCCCCTTATTTC	1	1	2	0
tsrna-22616	GAGCTTAAACCCCCTTATTT	0	2	0	0
tsrna-22613	GAGCTTAAACCCCCTTA	0	0	0	0
tsrna-22611	GAGCTGGGGATTGTGGGTTCG	0	0	0	0
tsrna-22609	GAGCTGGGGATTGTGGGTT	0	3	0	0
tsrna-22608	GAGCTGGGGATTGTGGGT	0	3	1	0
tsrna-22605	GAGCGTTAGACTGAAGATCTAAAGGTCCCT	0	5	1	2
tsrna-22604	GAGCGTTAGACTGAAGATCTAAAGGTC	0	4	1	1
tsrna-22598	GAGCGGTCTAAGGCGCTGGATT	0	1	0	1
tsrna-22587	GAGCCTCACCTGGAGCACCA	0	0	1	0
tsrna-22585	GAGCCTACCCAGGGACGCCA	0	2	0	0
tsrna-22584	GAGCCCGGTAATCGCATAAAACTTAAAACT	5	1	5	0
tsrna-22583	GAGCCCGGTAATCGCATAAAACTTAAAAC	0	0	1	0

tsrna-22582	GAGCCCGGTAATCGCATAAAACTTAAAA	0	1	2	0
tsrna-22581	GAGCCCGGTAATCGCATAAAACTTAAA	0	0	2	0
tsrna-22580	GAGCCCGGTAATCGCATAAAACTTAA	0	1	0	0
tsrna-22579	GAGCCCGGTAATCGCATAAAACTTA	0	0	1	0
tsrna-22576	GAGCCCGGTAATCGCATAAAAC	0	0	1	0
tsrna-22575	GAGCCCGGTAATCGCATAAAA	0	0	0	0
tsrna-22572	GAGCCCGGTAATCGCATA	0	0	0	0
tsrna-22569	GAGCCCCAGTGGAACCACCA	1	3	5	0
tsrna-22568	GAGCCCCAGTGGAACCACC	0	0	0	0
tsrna-22567	GAGCCCCACGTTGGGCGCCA	9	24	3	2
tsrna-22566	GAGCCCACCCAGGGACGCCA	3	61	0	1
tsrna-22565	GAGCCCACCCAGGGACGCC	0	3	0	0
tsrna-22562	GAGCATGGGACTCTTAATCCCAGGGTCGTG	1	1	1	0
tsrna-22557	GAGCACTCTGGACTCTGAATCCAGCGATCC	0	3	1	0
tsrna-22556	GAGCACTCTGGACTCTGAATCCAGCGATC	1	1	1	0
tsrna-22555	GAGCACTCTGGACTCTGAATCCAGCGAT	1	3	1	2
tsrna-22554	GAGCACTCTGGACTCTGAATCCAGCGA	0	2	2	0
tsrna-22553	GAGCACTCTGGACTCTGAATCCAGCG	1	2	0	0
tsrna-22552	GAGCACTCTGGACTCTGAATCCAGC	1	0	0	0
tsrna-22551	GAGCACTCTGGACTCTGAATCCAG	0	0	0	0
tsrna-22550	GAGCACTCTGGACTCTGAATCCA	0	5	0	0
tsrna-22549	GAGCACTCTGGACTCTGAATCC	0	3	0	0
tsrna-22548	GAGCACTCTGGACTCTGAATC	1	0	0	0
tsrna-22542	GAGATTTCAACTTAACTTGACCGCTCTGACCA	75	49	55	25
tsrna-22541	GAGATTTCAACTTAACTTGACCGCTCTGACC	8	9	7	2
tsrna-22540	GAGATTTCAACTTAACTTGACCGCTCTGAC	5	2	0	0
tsrna-22539	GAGATTTCAACTTAACTTGACCGCTCTGA	1	3	1	0
tsrna-22538	GAGATTTCAACTTAACTTGACCGCTCTG	0	4	0	0
tsrna-22537	GAGATTTCAACTTAACTTGACCGCTCT	0	1	0	0
tsrna-22536	GAGATTTCAACTTAACTTGACCGCTC	0	0	1	0
tsrna-22533	GAGATTTCAACTTAACTTGACCG	0	0	1	0
tsrna-22527	GAGATGAAAACCTTTTTCCAAGGACACCA	2	5	1	0
tsrna-22526	GAGATGAAAACCTTTTTCCAAGGACACC	3	2	0	0
tsrna-22525	GAGATGAAAACCTTTTTCCAAGGACA	0	0	1	0
tsrna-22518	GAGAGGTCCCGGGTTCAAATCCCGGACGAG	1	1	1	0
tsrna-22517	GAGAGGTCCCGGGTTCAAA	0	0	1	0
tsrna-22516	GAGAGGTCCCGGGTTCA	1	0	0	0
tsrna-22506	GAGACCGGGGTTCGATTCCCCGACGGGGAGCCA	1	21	0	0
tsrna-22505	GAGACCGGGGTTCGATTCCCCGACGGGGAGCC	0	2	0	0
tsrna-22504	GAGACCGGGGTTCGATTCCCCGACGGGGAGC	0	1	0	0
tsrna-22501	GAGACCGGGGTTCGATTCCCCGACGGGG	0	1	0	0
tsrna-22500	GAGACCGGGGTTCGATTCCCCGACGGG	0	0	0	0
tsrna-22486	GAGAATTTTGGATTCTCAGGGATG	0	0	0	0
tsrna-22485	GAGAATAGTTTAAATTAGAATCTTAGCTTTGG	11	3	7	2
tsrna-22484	GAGAATAGTTTAAATTAGAATCTTAGCTTT	4	1	4	1
tsrna-22476	GAGAACCAACACCTCTTTACAGTG	0	0	1	0
tsrna-22468	GAGAAAGCTCACAAGAACTGCTAACTCATGCCCCCATGTCTA/	883	2097	731	455
tsrna-22467	GAGAAAGCTCACAAGAACTGCTAACTCATGCCCCCATGTCTA/	902	2143	722	437
tsrna-22466	GAGAAAGCTCACAAGAACTGCTAACTCATGCCCCCATGTCTA/	919	2131	724	457
tsrna-22465	GAGAAAGCTCACAAGAACTGCTAACTCATGCCCCCATGTCT	834	2106	677	433
tsrna-22464	GAGAAAGCTCACAAGAACTGCTAACTCATGCCCCCA	846	2101	630	440
tsrna-22463	GAGAAAGCTCACAAGAACTGCTAACTCATGCCCCC	880	2077	696	476
tsrna-22462	GAGAAAGCTCACAAGAACTGCTAACTCATGCCCC	874	2040	695	460
tsrna-22461	GAGAAAGCTCACAAGAACTGCTAACTCATGCCC	839	2052	629	405
tsrna-22460	GAGAAAGCTCACAAGAACTGCTAACTCATGCC	785	2081	693	474
tsrna-22459	GAGAAAGCTCACAAGAACTGCTAACTCATG	860	2068	657	476
tsrna-22458	GAGAAAGCTCACAAGAACTGCTAACTCAT	795	2084	691	439
tsrna-22457	GAGAAAGCTCACAAGAACTGCTAACTCA	853	2030	678	467
tsrna-22456	GAGAAAGCTCACAAGAACTGCTAACTC	836	2055	656	477
tsrna-22455	GAGAAAGCTCACAAGAACTGCTAACT	818	1986	656	453
tsrna-22454	GAGAAAGCTCACAAGAACTGCTAAC	795	1794	597	432
tsrna-22453	GAGAAAGCTCACAAGAACTGCTAA	305	999	237	139
tsrna-22452	GAGAAAGCTCACAAGAACTGCTA	281	900	207	109
tsrna-22451	GAGAAAGCTCACAAGAACTGCT	218	881	212	130

tsrna-22450	GAGAAAGCTCACAAGAACTGC	81	238	63	23
tsrna-22449	GAGAAAGCTCACAAGAACTG	64	197	57	12
tsrna-22448	GAGAAAGCTCACAAGAACT	65	162	61	10
tsrna-22447	GAGAAAGCTCACAAGAAC	0	7	2	0
tsrna-22446	GAGAAAGCTCACAAGAA	0	0	0	0
tsrna-22445	GAGAAAGCTCACAAGA	0	1	0	0
tsrna-22444	GACTTTTTCTCTGACCA	3	1	4	3
tsrna-22443	GACTTTTTCTCTGACC	0	0	0	0
tsrna-22442	GACTTTTAATCTGAGGGTCCAGG	9	34	4	3
tsrna-22441	GACTTTTAATCTGAGGGTCCAG	6	36	4	0
tsrna-22440	GACTTTTAATCTGAGGGTCC	6	24	3	0
tsrna-22439	GACTTTGAATCCAGCGATCCGAGTTC	2	5	2	0
tsrna-22438	GACTTTGAATCCAGCGATCCGAGT	3	2	2	0
tsrna-22437	GACTTTGAATCCAGCGATCCGAG	1	0	2	0
tsrna-22436	GACTTTGAATCCAGCGATCCGA	1	2	0	0
tsrna-22435	GACTTTGAATCCAGCGATCCG	1	0	1	0
tsrna-22434	GACTTTGAATCCAGCGATCC	3	1	3	1
tsrna-22433	GACTTTGAATCCAGCGATC	1	1	0	0
tsrna-22432	GACTTTGAATCCAGCGA	0	4	1	0
tsrna-22431	GACTTTGAATCCAGCG	1	0	0	0
tsrna-22430	GACTTTGAATCCAGCAATCCGA	0	4	1	0
tsrna-22429	GACTTTGAATCCAGCAATCCG	0	2	1	1
tsrna-22425	GACTTCGGATCAGAAGATTGAGGGT	2	7	5	2
tsrna-22413	GACTGTAGATCCTTAGGTCGCTGG	0	0	0	1
tsrna-22410	GACTGTAAATCTAAAGACAGGGGTTAGGCC	0	1	0	0
tsrna-22409	GACTGTAAATCTAAAGACAGGGGTTAGG	0	1	0	0
tsrna-22408	GACTGTAAATCTAAAGACAGGGGTTAG	1	0	0	0
tsrna-22407	GACTGTAAATCTAAAGACAGGGGTT	0	0	0	0
tsrna-22405	GACTGTAAATCTAAAGACAGGGG	0	0	1	0
tsrna-22404	GACTGTAAATCTAAAGACAGGG	1	0	0	0
tsrna-22398	GACTGCAGATCAAGAGGTCCCTGGT	1	8	1	4
tsrna-22397	GACTGCAGATCAAGAGGTCCCCGGT	2	4	1	1
tsrna-22392	GACTGAAGATCTAAAGGTCCCTGGT	12	12	9	3
tsrna-22391	GACTCTTAATCTCAGGGTC	1	0	1	0
tsrna-22390	GACTCTGAATCCAGCGATCCGAGTTCAAGT	0	1	0	1
tsrna-22389	GACTCTGAATCCAGCGATCCGAGTTCAA	0	3	2	0
tsrna-22388	GACTCTGAATCCAGCGATCCGAGTTCA	0	4	0	0
tsrna-22387	GACTCTGAATCCAGCGATCCGAGTTC	0	1	0	0
tsrna-22386	GACTCTGAATCCAGCGATCCGAGT	0	2	0	0
tsrna-22385	GACTCTGAATCCAGCGATCCGAG	0	0	0	0
tsrna-22384	GACTCTGAATCCAGCGATCCGA	0	1	0	0
tsrna-22383	GACTCTGAATCCAGCGATCCG	0	0	0	1
tsrna-22382	GACTCTGAATCCAGCGATCC	0	1	0	0
tsrna-22380	GACTCTGAATCCAGCGAT	0	0	0	1
tsrna-22377	GACTCGAAATCCAATGGGG	1	13	0	0
tsrna-22376	GACTCCTGGCTGGCTCGCCA	10	61	0	3
tsrna-22375	GACTCCTGGCTGGCTCGCC	0	0	0	0
tsrna-22372	GACTCCCGGTGTGGGAACCA	1	4	1	1
tsrna-22370	GACTCCCGGTGTGGGAAC	0	0	0	0
tsrna-22367	GACTCCCGGTATGGGAACCA	0	0	0	1
tsrna-22365	GACTCCAGATCAGAAGGTTGCGTGTTCA	3	4	0	0
tsrna-22364	GACTCCAGATCAGAAGGTTGCGTGTT	0	4	0	0
tsrna-22363	GACTCCAGATCAGAAGGTTGCGTGT	0	1	0	0
tsrna-22362	GACTCCAGATCAGAAGGTTGCGTG	1	0	0	0
tsrna-22355	GACTCCAGATCAGAAGGCTGCGTGTTCG	1	0	0	0
tsrna-22354	GACTCCAGATCAGAAGGCTGCGTGTT	0	0	2	1
tsrna-22339	GACTCAAGTTCTGGTCTCCGGATGGAGGCG	1	1	1	0
tsrna-22338	GACTCAAGTTCTGGTCTCCGGA	2	2	0	1
tsrna-22337	GACTCAAGTTCTGGTCTCCGG	3	3	0	2
tsrna-22336	GACTCAAGTTCTGGTCTCCG	3	3	0	0
tsrna-22335	GACTCAAGTTCTGGTCTCCAATGGAGGCGT	0	5	1	0
tsrna-22334	GACTCAAGTTCTGGTCTCC	0	0	0	0
tsrna-22333	GACTCAAGTTCTGGTCTC	2	2	1	1
tsrna-22332	GACTCAAGTTCTGGTC	0	4	0	1

tsrna-22331	GACTACGGATCAGAAGATTCTAGGTTCGAC	4	6	0	1
tsrna-22330	GACGGGCTCACATCACCCCATAAACACCA	11	2	7	7
tsrna-22329	GACGGGCTCACATCACCCCATAAACACC	0	1	1	2
tsrna-22317	GACGAGGTGGCCGAGTGGTTAAGGCGATGG	16	55	10	6
tsrna-22316	GACGAGGTGGCCGAGTGGTTAAGGCGATG	17	47	7	8
tsrna-22315	GACGAGGTGGCCGAGTGGTTAAGGCGA	14	56	10	9
tsrna-22314	GACGAGGTGGCCGAGTGGTTAAGGCG	18	43	10	6
tsrna-22313	GACGAGGTGGCCGAGTGGTTAAGGC	18	50	16	7
tsrna-22312	GACGAGGTGGCCGAGTGGTTAAGG	6	63	4	11
tsrna-22311	GACGAGGTGGCCGAGTGGTTAAG	14	66	14	6
tsrna-22310	GACGAGGTGGCCGAGTGGTTAA	11	47	9	8
tsrna-22309	GACGAGGTGGCCGAGTGGTTA	10	41	11	9
tsrna-22308	GACGAGGTGGCCGAGTGGTT	11	57	10	6
tsrna-22307	GACGAGGTGGCCGAGTGGT	16	44	10	5
tsrna-22306	GACGAGGTGGCCGAGTGG	9	46	6	3
tsrna-22305	GACGAGGTGGCCGAGTG	11	60	6	3
tsrna-22304	GACGAGGTGGCCGAGT	17	45	9	4
tsrna-22303	GACCTCGTGGCGCAATGGTAGCGCGTCTGACTCC	1	2	0	0
tsrna-22302	GACCTCGTGGCGCAATGGTAGCGCGTCTGACT	0	1	0	0
tsrna-22300	GACCTCGTGGCGCAATGGTAGCGCGTCTG	0	0	1	0
tsrna-22299	GACCTCGTGGCGCAATGGTAGCGC	0	1	1	0
tsrna-22296	GACCTCGTGGCGCAATGGTAG	0	0	1	0
tsrna-22293	GACCTCGTGGCGCAATGG	0	0	0	1
tsrna-22290	GACCTCGTGGCGCAACGGTAGCGCGTCTGACTCC	2	7	1	2
tsrna-22289	GACCTCGTGGCGCAACGGTAGCGCGTCTGACT	0	3	0	0
tsrna-22285	GACCTCGTGGCGCAACGGTAGCGCG	0	1	0	0
tsrna-22272	GACCGGGGTTCGATTCCCCGACGGGGAGCCA	0	14	1	0
tsrna-22271	GACCGGGGTTCGATTCCCCGACGGGGAGCC	0	0	0	1
tsrna-22270	GACCGGGGTTCGATTCCCCGACGGGGAGC	0	2	0	0
tsrna-22269	GACCGGGGTTCGATTCCCCGACGGGGAG	1	0	0	0
tsrna-22267	GACCGGGGTTCGATTCCCCGACGGGG	0	1	0	0
tsrna-22266	GACCGGGGTTCGATTCCCCGACGGG	0	0	0	0
tsrna-22252	GACCGCGTGGCCTAATGGATAAGGCGTCTGACTT	0	0	1	0
tsrna-22251	GACCGCGTGGCCTAATGGATAAGGCGTCTGACT	0	1	0	0
tsrna-22237	GACCCGGGTTCGATTCCCGGCCAACGCACC	12	21	0	2
tsrna-22230	GACCCCTTATTTACCCCA	0	1	0	0
tsrna-22227	GACCCCCGGCTCCTCCACCA	13	25	4	2
tsrna-22226	GACCCAGTGGCCTAATGGATAAGGCATCAGCCTCC	5	4	1	0
tsrna-22225	GACCCAGTGGCCTAATGGATAAGGCATCAGCCTC	4	3	4	1
tsrna-22224	GACCCAGTGGCCTAATGGATAAGGCATCAGCCT	4	3	5	0
tsrna-22223	GACCCAGTGGCCTAATGGATAAGGCATCAGCC	6	5	2	0
tsrna-22222	GACCCAGTGGCCTAATGGATAAGGCATCAGC	4	2	3	0
tsrna-22221	GACCCAGTGGCCTAATGGATAAGGCATCAG	1	5	1	0
tsrna-22220	GACCCAGTGGCCTAATGGATAAGGCATCA	2	1	5	0
tsrna-22219	GACCCAGTGGCCTAATGGATAAGGCATC	0	2	2	1
tsrna-22218	GACCCAGTGGCCTAATGGATAAGGCAT	0	0	0	0
tsrna-22210	GACCCAGTGGCCTAATGGA	0	0	0	0
tsrna-22204	GACCACGTGGCCTAATGGATAAG	0	0	0	0
tsrna-22198	GACCAAGAGCCTTCAAAGCCCTC	0	1	0	0
tsrna-22195	GACCAAGAGCCTTCAAAGCC	0	1	0	0
tsrna-22193	GACCAAGAGCCTTCAAA	1	0	0	0
tsrna-22192	GACATATGTCCGCGTGGGT	5	6	3	0
tsrna-22191	GACATATGTCCGCGTGGG	1	0	0	0
tsrna-22190	GACAGGGGTTAGGCCTCTTTTTACCACCA	1	2	2	1
tsrna-22189	GACAGGGGTTAGGCCTCTTTTTACCACC	0	1	2	0
tsrna-22188	GACAGGGGTTAGGCCTCTTTTTACCAC	0	1	2	0
tsrna-22187	GACAGGGGTTAGGCCTCTTTTTACCA	0	1	0	0
tsrna-22186	GACAGGGGTTAGGCCTCTTTTTACC	0	0	2	1
tsrna-22185	GACAGGGGTTAGGCCTCTTT	1	0	0	0
tsrna-22184	GACAGGGGTTAGGCCTCTT	0	0	0	0
tsrna-22183	GACAGGGGTTAGGCCTCT	0	1	0	0
tsrna-22180	GACAACATTCAAAAAAGAGTACCA	0	0	0	0
tsrna-22174	GACAACAGAGGCTTACGACCCCTTATTTAC	0	0	0	0
tsrna-22169	GAATTTTGGATTCTCAGGGATG	0	0	0	0

tsrna-22166	GAATTGCAAATTCGAAGAAGCAGCTTCAAACCTGCCGGGGCT	1	3	4	0
tsrna-22165	GAATTGCAAATTCGAAGAAGCAGCTTCAAACCTGCCGGGGCT	1	1	1	0
tsrna-22164	GAATTGCAAATTCGAAGAAGCAGCTTCAAACCTGCCGGGGCT	0	2	1	0
tsrna-22163	TGGTCTAGTGGCTAGGATTCGGCGC	10	31	7	32
tsrna-22162	TGGTCTAGTGGCTAGGATTCGGCG	10	30	6	25
tsrna-22161	TGGTCTAGTGGCTAGGATTCGGC	7	19	4	10
tsrna-22160	TGGTCTAGTGGCTAGGATTCGG	0	2	1	0
tsrna-22159	TGGTCTAGTGGCTAGGATTCG	1	1	0	1
tsrna-22158	TGGTCTAGGGGTATGATTCTCGGTT	2	2	4	1
tsrna-22157	TGGTCTAGGGGTATGATTCTCGGT	8	5	2	0
tsrna-22156	TGGTCTAGGGGTATGATTCTCGG	1	1	1	0
tsrna-22155	TGGTCTAGGGGTATGATTCTCGCTTC	19	16	15	18
tsrna-22154	TGGTCTAGGGGTATGATTCTCGCTT	20	33	14	17
tsrna-22153	TGGTCTAGGGGTATGATTCTCGCT	17	16	9	14
tsrna-22152	TGGTCTAGGGGTATGATTCTCGC	13	9	7	11
tsrna-22151	TGGTCTAGGGGTATGATTCTCG	0	1	0	1
tsrna-22150	TGGTCTAGGGGTATGATTCTC	0	0	0	0
tsrna-22149	TGGTCTAGGGGTATGATTCT	0	0	0	0
tsrna-22144	TGGTCTAGCGGTTAGGATTCCTGGTTTTCACCCAG	14	19	14	5
tsrna-22143	TGGTCTAGCGGTTAGGATTCCTGGTTTTCACCC	13	13	9	2
tsrna-22142	TGGTCTAGCGGTTAGGATTCCTGGTTTTCA	12	8	6	4
tsrna-22141	TGGTCTAGCGGTTAGGATTCCTGGTTTTC	12	4	7	1
tsrna-22140	TGGTCTAGCGGTTAGGATTCCTGGTTTT	5	5	3	3
tsrna-22139	TGGTCTAGCGGTTAGGATTCCTGGTTT	5	9	0	1
tsrna-22138	TGGTCTAGCGGTTAGGATTCCTGGTT	4	9	3	2
tsrna-22137	TGGTCTAGCGGTTAGGATTCCTGGT	7	4	2	1
tsrna-22136	TGGTCTAGCGGTTAGGATTCCTGG	5	5	2	0
tsrna-22135	TGGTCTAGCGGTTAGGATTCCTG	1	2	2	0
tsrna-22134	TGGTCTAGCGGTTAGGATTCCT	1	1	3	0
tsrna-22128	TGGTCTAAGGCGCTGGATTTAG	3	5	1	0
tsrna-22127	TGGTCTAAGGCGCTGGATTT	2	2	2	0
tsrna-22126	TGGTCTAAGGCGCTGGATT	0	2	0	0
tsrna-22125	TGGTCTAAGGCGCTGGAT	0	1	0	0
tsrna-22124	TGGTCTAAGGCGCTGGA	1	1	0	0
tsrna-22122	TGGTCTAAGGCGCCAGACTCAAG	4	1	2	5
tsrna-22121	TGGTCTAAGGCGCCAGACTCA	3	0	2	1
tsrna-22120	TGGTCTAAGGCGCCAGACTC	2	1	2	2
tsrna-22117	TGGTCGTGGTTGTAGTCCGTGCGAGAATACCA	21	50	34	14
tsrna-22116	TGGTCGTGGTTGTAGTCCGTGCGAGAATACC	6	7	6	1
tsrna-22115	TGGTCGTGGTTGTAGTCCGTGCGAGAATAC	4	7	8	3
tsrna-22114	TGGTCGTGGTTGTAGTCCGTGCGAGAATA	0	4	2	1
tsrna-22113	TGGTCGTGGTTGTAGTCCGTGCGAGAAT	0	3	3	1
tsrna-22112	TGGTCGTGGTTGTAGTCCGTGCGAGAA	0	4	2	1
tsrna-22111	TGGTCGTGGTTGTAGTCCGTGCGAGA	1	5	2	4
tsrna-22110	TGGTCGTGGTTGTAGTCCGTGCGAG	0	1	2	0
tsrna-22109	TGGTCGTGGTTGTAGTCCGTGCGA	0	1	0	0
tsrna-22108	TGGTCGTGGTTGTAGTCCGTGCG	0	1	1	0
tsrna-22107	TGGTCGTGGTTGTAGTCCGTGC	1	0	1	0
tsrna-22106	TGGTCGTGGTTGTAGTCCGTG	2	1	0	0
tsrna-22105	TGGTCGTGGTTGTAGTCCGT	0	0	0	0
tsrna-22104	TGGTCGTGGTTGTAGTCCG	0	0	1	0
tsrna-22103	TGGTCGTGGTTGTAGTCC	0	0	0	0
tsrna-22100	TGGTCATCACGTTCGCC	0	0	1	0
tsrna-22098	TGGTCAGCACTCTGGACTCTGAATCCA	1	4	2	0
tsrna-22097	TGGTCAGCACTCTGGACTCTGAATC	0	1	0	0
tsrna-22093	TGGTCAGCACTCTGGACT	0	0	0	0
tsrna-22091	TGGTATGATTCTCGCT	1	1	0	2
tsrna-22088	TGGTATCATGCAAGATTC	0	1	0	0
tsrna-22086	TGGTATATAGTTTAAACAAAACGAATGATTTCGACTCA	1	2	1	0
tsrna-22085	TGGTATATAGTTTAAACAAAACGAATGATTTCGACTC	0	1	0	0
tsrna-22084	TGGTATATAGTTTAAACAAAACGAATGATTTCGACT	0	0	0	0
tsrna-22083	TGGTATATAGTTTAAACAAAACGAATGATT	0	0	1	0
tsrna-22076	TGGTATAGTGGTTAGCATAGCTG	1	0	0	0
tsrna-22074	TGGTATAGTGGTGAGCATAGCTGC	0	2	0	1

tsrna-22073	TGGTATAGTGGTGAGCATAGCTG	0	0	0	0
tsrna-22072	TGGTATAGTGGTGAGCATAGC	0	1	0	0
tsrna-22070	TGGTATAGTGGTAAGCATAGCTGC	0	2	0	0
tsrna-22069	TGGTATAGTGGTAAGCATAGCTG	1	0	0	0
tsrna-22050	TGGTAGAGCGCTCGCTTAGC	1	0	1	1
tsrna-22049	TGGTAGAGCGCTCGCTTAG	0	0	1	0
tsrna-22048	TGGTAGAGCGCGTGCTTCGCA	6	32	4	0
tsrna-22047	TGGTAGAGCGCGTGCTTCGC	8	26	6	0
tsrna-22046	TGGTAGAGCGCGTGCTTAGC	13	38	6	1
tsrna-22045	TGGTAGAGCGCGTGCTTAG	7	21	2	0
tsrna-22044	TGGTAGAGCGCGTGCT	6	16	2	0
tsrna-22042	TGGTAGAGCATTTGACTGCAGATCAAGAGG	0	0	0	0
tsrna-22041	TGGTAGAGCATTTGACTGCAGATCAAG	0	2	0	0
tsrna-22040	TGGTAGAGCATTTGACTGCAGATCA	0	1	0	0
tsrna-22039	TGGTAGAGCATTTGACTGCAGATC	0	1	1	0
tsrna-22037	TGGTAGAGCATTTGACTGCA	0	2	0	0
tsrna-22036	TGGTAGAGCATTTGACTGC	1	0	0	0
tsrna-22035	TGGTAGAGCATTTGACTG	0	1	0	0
tsrna-22034	TGGTAGAGCATTTGACT	1	0	0	0
tsrna-22032	TGGTAGAGCATGGGACTCT	3	5	3	0
tsrna-22028	TGGTAGAATTCTTGCCTGCC	0	0	1	0
tsrna-22025	TGGTAGAATTCTCGCCTGCCACGCGGGAGGCCCGGGTTT	135	186	135	27
tsrna-22024	TGGTAGAATTCTCGCCTGCCACGCGGGAGGCCCGGGTT	132	185	126	27
tsrna-22023	TGGTAGAATTCTCGCCTGCCACGCGGGAGGCCCGGGT	124	158	104	21
tsrna-22022	TGGTAGAATTCTCGCCTGCCACGCGGGAGGCCCGGG	74	146	44	27
tsrna-22021	TGGTAGAATTCTCGCCTGCCACGCGGGAGGCCCGG	80	143	56	18
tsrna-22020	TGGTAGAATTCTCGCCTGCCACGCGGGAGGCCCG	70	131	43	22
tsrna-22019	TGGTAGAATTCTCGCCTGCCACGCGGGAGGCCC	76	125	45	20
tsrna-22018	TGGTAGAATTCTCGCCTGCCACGCGGGAGGCC	61	127	39	18
tsrna-22017	TGGTAGAATTCTCGCCTGCCACGCGGGAGG	75	119	36	21
tsrna-22016	TGGTAGAATTCTCGCCTGCCACGCGGGAG	52	146	34	19
tsrna-22015	TGGTAGAATTCTCGCCTGCCACGCGGGA	53	119	29	20
tsrna-22014	TGGTAGAATTCTCGCCTGCCACGCGGG	69	112	40	22
tsrna-22013	TGGTAGAATTCTCGCCTGCCACGCGG	60	113	42	27
tsrna-22012	TGGTAGAATTCTCGCCTGCCACGCG	61	95	39	28
tsrna-22011	TGGTAGAATTCTCGCCTGCCACGC	60	83	32	22
tsrna-22010	TGGTAGAATTCTCGCCTGCCACG	46	90	20	16
tsrna-22009	TGGTAGAATTCTCGCCTGCCAC	15	30	13	4
tsrna-22008	TGGTAGAATTCTCGCCTGCCA	10	27	7	1
tsrna-22007	TGGTAGAATTCTCGCCTGCC	10	36	11	1
tsrna-22006	TGGTAGAATTCTCGCCTGC	5	3	5	4
tsrna-22005	TGGTAGAATTCTCGCCTG	0	4	1	0
tsrna-22004	TGGTAGAATTCTCGCCTCCCACGCGGGAGACCCGGGT	3	16	3	11
tsrna-22003	TGGTAGAATTCTCGCCTCCCACGCGGGAGACCC	6	13	4	12
tsrna-22002	TGGTAGAATTCTCGCCTCCCACGCGGGAG	6	11	3	8
tsrna-22001	TGGTAGAATTCTCGCCTCCCACG	5	9	2	6
tsrna-22000	TGGTAGAATTCTCGCCTCCCA	3	18	1	1
tsrna-21999	TGGTAGAATTCTCGCCTCCC	3	13	3	9
tsrna-21998	TGGTAGAATTCTCGCCTCC	2	11	1	0
tsrna-21997	TGGTAGAATTCTCGCCTC	2	1	2	2
tsrna-21996	TGGTAGAATTCTCGCCT	2	1	0	0
tsrna-21995	TGGTAGAATTCTCGCC	1	0	0	0
tsrna-21994	TGGTAGAATTCTCACC	1	1	0	0
tsrna-21985	TGGTAAGCACTCTGGACTCTGAATCCA	1	1	2	0
tsrna-21984	TGGTAAGCACTCTGGACTCTGAATCC	2	2	2	2
tsrna-21983	TGGTAAGCACTCTGGACTCTGAATC	0	0	2	0
tsrna-21973	TGGGTTTAAGTCCCATTGGTCTAGCCA	1	0	0	1
tsrna-21972	TGGGTTTAAGTCCCATTGGTCTAGCC	2	1	0	1
tsrna-21971	TGGGTTTAAGTCCCATTGGTCTAGC	0	0	1	0
tsrna-21968	TGGGTTTAAGTCCCATTGGTCT	0	0	0	0
tsrna-21967	TGGGTTTAAGTCCCATTGGTC	1	0	1	0
tsrna-21962	TGGGTTCGATTCTCATAGTCCTAGCCA	2	4	0	2
tsrna-21961	TGGGTTCGATTCTCATAGTCCTAGCC	2	5	0	1
tsrna-21960	TGGGTTCGATTCTCATAGTCCTAGC	2	4	1	1

tsrna-21959	TGGGTTCGATTCTCATAGTCCTAG	1	6	0	0
tsrna-21958	TGGGTTCGATTCTCATAGTCCTA	0	1	0	0
tsrna-21957	TGGGTTCGATTCTCATAGTCC	0	2	1	0
tsrna-21956	TGGGTTCGATTCTCATAGTC	0	0	1	0
tsrna-21955	TGGGTTCGATCCCCAGTACCTCCACCA	36	54	14	13
tsrna-21954	TGGGTTCGAGTCCCATCTGGGTCGCCA	1	4	0	0
tsrna-21953	TGGGTTCGAGTCCCATCTGGGTCGCC	0	0	1	0
tsrna-21950	TGGGTTCGAGTCCCACCAGAGTCGCCA	0	7	0	0
tsrna-21948	TGGGTTCGAGCCCCAGTGGAACCACCA	31	36	12	15
tsrna-21947	TGGGTTCGAGCCCCAGTGGAACCA	1	1	0	0
tsrna-21946	TGGGTTCGAGCCCCAGTGGAACC	1	0	1	0
tsrna-21945	TGGGTTCGAGCCCCACGTTGGGCGCCA	9	199	7	4
tsrna-21944	TGGGTTCGAATCCCATCCTCGTCGCCA	6	18	2	1
tsrna-21943	TGGGTTCGAATCCCATCCTCGTCGCC	1	2	0	0
tsrna-21941	TGGGTTCGAATCCCATCCTCGTCG	1	0	0	0
tsrna-21940	TGGGTTCGAATCCCAGCGGTGCCTCCA	24	60	12	3
tsrna-21939	TGGGTTCGAATCCCAGCGGGGCCTCCA	0	7	0	1
tsrna-21938	TGGGTTCGAATCCCACTTCTGACACCA	8	30	5	5
tsrna-21937	TGGGTTCGAATCCCACTTCTGACAC	0	0	0	0
tsrna-21936	TGGGTTCGAATCCCACCGCTGCCACC	1	5	0	0
tsrna-21935	TGGGTTCGAATCCCACCAGAGTCGCCA	6	2	0	2
tsrna-21934	TGGGTTCGAATCCCACCAGAGTCG	0	1	0	0
tsrna-21933	TGGGTGGTTCAGTGGTAGAATTCTCGCCTGCC	15	37	17	4
tsrna-21932	TGGGTGGTTCAGTGGTAGAATTCTCGCCTG	7	20	2	7
tsrna-21931	TGGGTGGTTCAGTGGTAGAATTCTCGCCT	12	20	4	3
tsrna-21930	TGGGTGGTTCAGTGGTAGAATTCTCGCC	11	8	0	2
tsrna-21929	TGGGTGGTTCAGTGGTAGAATTCTCGC	13	2	1	1
tsrna-21928	TGGGTGGTTCAGTGGTAGAATTCTCG	2	3	3	2
tsrna-21927	TGGGTGGTTCAGTGGTAGAATTCTC	2	5	2	1
tsrna-21926	TGGGTGGTTCAGTGGTAGAATTCT	3	5	1	1
tsrna-21925	TGGGTGGTTCAGTGGTAGAATTC	0	2	2	0
tsrna-21924	TGGGTGGTTCAGTGGTAGAATT	7	2	4	0
tsrna-21923	TGGGTGGTTCAGTGGTAGAAT	0	4	1	1
tsrna-21922	TGGGTGGTTCAGTGGTAGAA	1	1	0	1
tsrna-21921	TGGGTGGTTCAGTGGTAGA	0	0	0	0
tsrna-21920	TGGGTGGTTCAGTGGTAG	2	0	0	0
tsrna-21919	TGGGTGGTTCAGTGGTA	0	0	0	0
tsrna-21917	TGGGTGCTAATGGTGGAGTTAAAGACTTTT	3	7	6	2
tsrna-21916	TGGGTGCTAATGGTGGAGTTAAAGACT	2	6	3	1
tsrna-21915	TGGGTGCTAATGGTGGAGTTAAAGAC	1	4	0	0
tsrna-21914	TGGGTGCTAATGGTGGAGTTAAAGA	1	2	1	2
tsrna-21913	TGGGTGCTAATGGTGGAGTTAAAG	2	8	2	1
tsrna-21912	TGGGTGCTAATGGTGGAGTTAAA	1	2	1	1
tsrna-21911	TGGGTGCTAATGGTGGAGTTAA	2	6	0	0
tsrna-21910	TGGGTGCTAATGGTGGAGTTA	0	2	1	0
tsrna-21909	TGGGTGCTAATGGTGGAGTT	0	1	0	0
tsrna-21906	TGGGTGCGAGAGGTCCCGGGTT	1	2	0	0
tsrna-21905	TGGGTGCGAGAGGTCCCGGGT	0	1	0	0
tsrna-21903	TGGGTCCGAGAGGTCCCGGGT	0	1	0	0
tsrna-21899	TGGGTAGAGCATTTGACTGC	1	0	1	0
tsrna-21894	TGGGGTTTTGCAGTCCTTACCA	0	3	1	0
tsrna-21893	TGGGGTTTTGCAGTCCTTACC	0	0	0	0
tsrna-21891	TGGGGTTTCCCCGCGCAGGTTCG	0	1	0	0
tsrna-21889	TGGGGTTTCCCCGCGCAGGTT	0	0	0	0
tsrna-21880	TGGGGGTTCCCCGCGCAGGT	3	2	1	0
tsrna-21879	TGGGGGGTTCGATTCCTTCCTTTTTTGCC	0	1	0	1
tsrna-21878	TGGGGGGTTCGATTCCTTCCTTTTTTGC	0	1	0	2
tsrna-21877	TGGGGGGTTCGATTCCTTCCTTTTTTG	1	2	1	0
tsrna-21876	TGGGGGGTTCGATTCCTTCCTT	0	1	0	0
tsrna-21874	TGGGGATTGTGGGTTCGAGTCCCATCTGGGTCGCC	0	1	1	0
tsrna-21873	TGGGGATTGTGGGTTCGAGTCCCATC	0	1	0	0
tsrna-21872	TGGGGATTGTGGGTTCGAGTCCC	0	2	0	0
tsrna-21871	TGGGGATTGTGGGTTCG	0	1	0	0
tsrna-21869	TGGGCCCATAACCCAGAGGTCGATGGATCG	5	3	1	1

tsrna-21868	TGGGCCCATAACCCAGAGGTCGATGGA	3	5	0	0
tsrna-21867	TGGGCCCATAACCCAGAGGTCGATG	1	1	0	0
tsrna-21866	TGGGCCCATAACCCAGAGGTCGAT	1	0	0	0
tsrna-21864	TGGGCCCATAACCCAGAGGTCG	0	1	0	0
tsrna-21860	TGGGAGAGCGTTAGACTGAAGA	0	1	0	0
tsrna-21854	TGGGAGAGCATTAGACTGAA	0	0	0	0
tsrna-21853	TGGGACTCTTAATCCCAGGGTCGTGGGTTC	0	3	0	0
tsrna-21848	TGGCTGAGTGAAGCATTGGACTGTAAATC	2	0	0	0
tsrna-21847	TGGCTGAGTGAAGCATTGGACTGTAAAT	0	1	1	0
tsrna-21846	TGGCTGAGTGAAGCATTGGACTGTAAA	0	2	2	0
tsrna-21845	TGGCTGAGTGAAGCATTGGACTGTAA	1	0	0	0
tsrna-21844	TGGCTGAGTGAAGCATTGGACTGTA	1	1	0	0
tsrna-21842	TGGCTGAGTGAAGCATTGGACTG	0	1	1	0
tsrna-21841	TGGCTGAGTGAAGCATTGGACT	0	0	0	0
tsrna-21833	TGGCTAGGATTCGGCGC	1	11	0	5
tsrna-21832	TGGCTAGGATTCGGCG	4	2	1	1
tsrna-21831	TGGCGGTCTAGTGGTTAGGATTCGG	6	11	3	1
tsrna-21830	TGGCGGTCTAGTGGTTAGGATTCG	2	14	7	1
tsrna-21829	TGGCGCAGCGGAAGCGTGCTGGGCC	4	30	1	3
tsrna-21828	TGGCGCAGCGGAAGCGTGCTGGGC	2	26	2	0
tsrna-21827	TGGCGCAGCGGAAGCGTGCTGGG	3	21	2	1
tsrna-21826	TGGCGCAATGGATAGCGCATTG	2	1	0	0
tsrna-21825	TGGCCTCCTAAGCCAGGGATTGTGG	0	1	1	0
tsrna-21824	TGGCCTAATGGATAAGGCGTCTGA	0	2	0	0
tsrna-21823	TGGCCTAATGGATAAGGCATTGGCCTCCT	5	7	4	1
tsrna-21822	TGGCCTAATGGATAAGGCATTGGCC	7	3	1	4
tsrna-21821	TGGCCTAATGGATAAGGCATTG	1	7	4	0
tsrna-21820	TGGCCTAATGGATAAGGCATCAGCCT	4	3	0	2
tsrna-21817	TGGCCGCAGCAACCTCGGTTCGAAT	0	1	0	0
tsrna-21816	TGGCCGCAGCAACCTCGGTTCGAA	1	1	0	0
tsrna-21815	TGGCCGCAGCAACCTCGGTTCGA	0	2	0	0
tsrna-21814	TGGCCGCAGCAACCTCGGTTCG	0	1	0	0
tsrna-21813	TGGCCGCAGCAACCTCGGTTC	0	1	0	0
tsrna-21804	TGGCCGAGTGGTCTAAGGCGCCAGAC	0	1	0	0
tsrna-21801	TGGCCGAGCGGTCTAAGGCGCTGGATT	1	1	1	0
tsrna-21800	TGGCCGAGCGGTCTAAGGCGCTGCGTTC	0	1	0	1
tsrna-21789	TGGATTTAGGCTCCAGTCTCTTCGG	1	0	0	0
tsrna-21787	TGGATCGAAACCATCCTCTGCTACCA	11	24	14	6
tsrna-21786	TGGATCGAAACCATCCTCTGCTACC	5	5	2	1
tsrna-21785	TGGATCGAAACCATCCTCTGCTAC	0	1	0	0
tsrna-21784	TGGATCGAAACCATCCTCTGCTA	0	0	0	0
tsrna-21777	TGGATAGCTCAGTCGGTAGAG	0	1	0	0
tsrna-21774	TGGATAGCGCATTGGAC	2	2	1	0
tsrna-21773	TGGATAGCGCATTGGA	2	0	2	0
tsrna-21772	TGGATAAGGCGTCTGATTCC	1	3	1	1
tsrna-21771	TGGATAAGGCGTCTGATT	1	0	0	0
tsrna-21770	TGGATAAGGCGTCTGACTTCGGATCAGAAG	0	3	1	1
tsrna-21769	TGGATAAGGCGTCTGACTTCGGATCA	1	0	0	0
tsrna-21768	TGGATAAGGCGTCTGACTTCGGATC	1	1	0	0
tsrna-21767	TGGATAAGGCGTCTGACTTCGGAT	0	1	0	0
tsrna-21765	TGGATAAGGCGTCTGACTTCGG	1	1	0	0
tsrna-21764	TGGATAAGGCGTCTGACTTCG	1	0	0	0
tsrna-21763	TGGATAAGGCGTCTGACTTC	0	0	0	0
tsrna-21761	TGGATAAGGCGTCTGACT	0	0	0	1
tsrna-21758	TGGATAAGGCATTGGCCTCCTAAGCCAGGG	3	3	2	0
tsrna-21757	TGGATAAGGCATTGGCCTCCTAAG	2	1	3	0
tsrna-21756	TGGATAAGGCATTGGCCTC	0	0	2	0
tsrna-21754	TGGATAAGGCATTGGCC	0	0	2	0
tsrna-21753	TGGATAAGGCATTGGC	2	0	0	0
tsrna-21752	TGGATAAGGCATCAGCCT	0	0	0	0
tsrna-21751	TGGATAAGGCACTGGC	9	13	10	6
tsrna-21750	TGGATAACGCGTCTGACTACGGATC	2	1	0	1
tsrna-21749	TGGATAACGCGTCTGACTACGGAT	0	0	1	1
tsrna-21748	TGGATAACGCGTCTGACTACGGA	2	1	1	2

tsrna-21747	TGGATAACGCGTCTGACTACGG	0	1	0	0
tsrna-21746	TGGATAACGCGTCTGACTACG	1	0	2	0
tsrna-21745	TGGATAACGCGTCTGACTAC	0	0	0	1
tsrna-21743	TGGAGTTAAAGACTTTTTCTCTGACCA	27	37	16	18
tsrna-21742	TGGAGTTAAAGACTTTTTCTCTGACC	4	4	1	0
tsrna-21741	TGGAGTTAAAGACTTTTTCTCTGAC	0	6	0	0
tsrna-21740	TGGAGTTAAAGACTTTTTCTCTGA	1	2	0	0
tsrna-21739	TGGAGTTAAAGACTTTTTCTCTG	0	3	0	0
tsrna-21738	TGGAGGCGTGGGTTCGAATCCCACT	0	3	0	0
tsrna-21736	TGGAGGCGTGGGTTCGAATCCC	0	0	0	0
tsrna-21731	TGGAGGCGTGGGTTCG	0	1	0	0
tsrna-21730	TGGAGGCCATGGGGTTGGCTTG	0	1	0	0
tsrna-21727	TGGACTTTGAATCCAGCGATCCGAG	0	2	2	0
tsrna-21726	TGGACTTTGAATCCAGCGATCCG	0	2	0	0
tsrna-21725	TGGACTTTGAATCCAGCGATCC	1	3	2	1
tsrna-21724	TGGACTTTGAATCCAGCGA	0	3	0	1
tsrna-21723	TGGACTTTGAATCCAGC	0	1	0	1
tsrna-21722	TGGACTTGAAATCCATTGGGG	0	4	0	0
tsrna-21721	TGGACTTCTAATTCAAAGGTTC	0	1	0	0
tsrna-21714	TGGACTGTAAATCTAAAGACAGGGG	1	0	0	0
tsrna-21707	TGGACTGCTAATCCATTGTGCT	2	0	2	0
tsrna-21706	TGGACTGCTAATCCATTGTGC	0	0	2	0
tsrna-21705	TGGACTGCTAATCCATTGTG	0	1	0	0
tsrna-21700	TGGACTCTGAATCCAGCGATCCGAG	0	2	0	0
tsrna-21698	TGGACTCTGAATCCAGCGATCCG	0	1	0	0
tsrna-21696	TGGACTCTGAATCCAGCGATC	0	1	0	1
tsrna-21692	TGGACTCTGAATCCAGC	0	0	0	1
tsrna-21690	TGGACATATGTCCGCGTGGGTT	34	35	26	4
tsrna-21689	TGGACATATGTCCGCGTGGGT	25	19	17	1
tsrna-21688	TGGACATATGTCCGCGTGGG	1	0	1	0
tsrna-21687	TGGACATATGTCCGCGTGG	2	1	1	0
tsrna-21686	TGCTTTGCACGTATGAGGCCCCGGGT	27	54	12	28
tsrna-21685	TGCTTTGCACGTATGAGGCCCC	1	2	0	0
tsrna-21684	TGCTTTGCACGCGTGGGTT	0	6	0	0
tsrna-21683	TGCTTTGCACGCGTGGGT	2	5	0	0
tsrna-21682	TGCTTTACACGCAGAAGGTCCTGGGT	10	36	8	2
tsrna-21681	TGCTTTACACGCAGAAGGTCCTG	2	0	0	0
tsrna-21680	TGCTTAGCTGTTAACTAAGTGTTTGTGGGT	1	0	0	2
tsrna-21678	TGCTCTGCACGCGTGGGT	2	6	0	0
tsrna-21677	TGCTACTAATGCCAGGGTCGAGGTTT	1	3	2	6
tsrna-21676	TGCTACTAATGCCAGGGTCGAGGTT	0	0	3	3
tsrna-21673	TGCTAATGGTGGAGTTAAAGACTTTTTCTCTGACCA	39	46	20	14
tsrna-21672	TGCTAATGGTGGAGTTAAAGACTTTTTCTCTGACC	12	13	4	1
tsrna-21671	TGCTAATGGTGGAGTTAAAGACTTTTTCTC	6	4	4	1
tsrna-21670	TGCTAATGGTGGAGTTAAAGACT	3	5	1	1
tsrna-21669	TGCTAATGGTGGAGTTAAAGAC	1	2	0	0
tsrna-21668	TGCTAATGGTGGAGTTAAAGA	1	4	1	0
tsrna-21667	TGCTAATGGTGGAGTTAAAG	2	2	0	0
tsrna-21666	TGCTAATGGTGGAGTTAAA	2	3	0	0
tsrna-21665	TGCTAATGGTGGAGTTAA	1	1	1	0
tsrna-21664	TGCTAATGGTGGAGTTA	1	1	1	0
tsrna-21662	TGCTAATCCATTGTGCTTTGCACGCGTGGG	0	1	1	0
tsrna-21661	TGCTAATCCATTGTGCTTTGCACGC	0	2	0	0
tsrna-21659	TGCTAATCCATTGTGCTTTGCA	0	1	0	0
tsrna-21654	TGCTAATCCATTGTGCTCTGC	0	3	1	0
tsrna-21653	TGCTAATCCATTGTGCTCTG	0	1	0	0
tsrna-21652	TGCTAATCCATTGTGCTC	0	1	0	0
tsrna-21651	TGCTAATCCATTGTGCT	1	0	0	0
tsrna-21649	TGCTAACTCATGCCCCCATGTCTAACAACATGGCTTTCTCACC	21	3	34	1
tsrna-21648	TGCTAACTCATGCCCCCATGTCTAACAACATGGCTTTCTCACC	10	3	16	0
tsrna-21647	TGCTAACTCATGCCCCCATGTCTAACAAC	0	0	0	0
tsrna-21646	TGCTAACTCATGCCCCCATGTCTAACA	0	0	0	0
tsrna-21642	TGCTAACTCATGCCCCCATGTCT	0	0	0	0
tsrna-21640	TGCTAACTCATGCCCCCATGT	0	0	0	0

tsrna-21632	TGCGTGTTCAAGTCACGTCGGGGTCA	0	0	0	0
tsrna-21626	TGCGAGAGGTCCCGGGTTCAAATCCCGGAC	0	5	0	0
tsrna-21625	TGCGAGAGGTCCCGGGTTCA	0	1	0	0
tsrna-21624	TGCGAGAGGTCCCGGGTTC	1	2	0	0
tsrna-21623	TGCGAGAGGTCCCGGGTT	0	3	0	0
tsrna-21622	TGCGAGAGGTCCCGGGT	0	1	0	0
tsrna-21621	TGCGAGAGGTAGCGGGA	1	12	2	0
tsrna-21620	TGCGACCCGGGTTCGATTCCCGGGCGGCG	0	3	0	0
tsrna-21619	TGCCTTCCAAGCAGTTGACCCGGGTTCG	30	37	13	12
tsrna-21618	TGCCTTCCAAGCAGTTGACCCGGGTTC	33	27	21	9
tsrna-21617	TGCCTTCCAAGCAGTTGACCCGGGT	29	28	13	14
tsrna-21616	TGCCTTCCAAGCAGTTGACCCGGG	3	3	3	4
tsrna-21615	TGCCTTCCAAGCAGTTGACCCGG	1	0	1	2
tsrna-21614	TGCCTTCCAAGCAGTTGACCCG	1	2	3	3
tsrna-21613	TGCCTTCCAAGCAGTTGACCC	1	1	1	0
tsrna-21612	TGCCTGTCACGCGGGAGACCGG	22	40	9	5
tsrna-21611	TGCCGTGATCGTATAGTGGTTAGTACTCTGCGTTGTGGCCGC	33	16	16	9
tsrna-21610	TGCCGTGATCGTATAGTGGTTAGTACTCTGCGTTGTGGCC	34	17	16	7
tsrna-21609	TGCCGTGATCGTATAGTGGTTAGTACTCTGCGTTGTG	30	12	13	5
tsrna-21608	TGCCGTGATCGTATAGTGGTTAGTACTCTGCGTTGT	38	19	13	8
tsrna-21607	TGCCGTGATCGTATAGTGGTTAGTACTCTGCGTTG	31	16	15	4
tsrna-21606	TGCCGTGATCGTATAGTGGTTAGTACTCTGCGTT	23	7	11	6
tsrna-21605	TGCCGTGATCGTATAGTGGTTAGTACTCTGCGT	3	3	1	1
tsrna-21604	TGCCGTGATCGTATAGTGGTTAGTACTCTGCG	4	5	5	3
tsrna-21603	TGCCGTGATCGTATAGTGGTTAGTACTCTGC	1	1	2	2
tsrna-21602	TGCCGTGATCGTATAGTGGTTAGTACTCTG	0	0	3	2
tsrna-21601	TGCCGTGATCGTATAGTGGTTAGTACTCT	2	1	1	0
tsrna-21600	TGCCGTGATCGTATAGTGGTTAGTACTC	2	2	0	0
tsrna-21597	TGCCGTGATCGTATAGTGGTTAGTA	0	0	1	1
tsrna-21592	TGCCGTGATCGTATAGTGGT	0	0	0	1
tsrna-21591	TGCCGTGATCGTATAGTGG	0	0	0	1
tsrna-21587	TGCCGAGGTTGTGAGTTCG	0	0	0	0
tsrna-21584	TGCCCGCATTCTCCACCA	1	5	0	1
tsrna-21581	TGCCCGCATCCTCCACCA	0	1	1	0
tsrna-21580	TGCCCGCATCCTCCACC	0	1	0	0
tsrna-21578	TGCCCCCATGTCTAACAACATGGCTTTCTCACCA	7	4	7	0
tsrna-21577	TGCCCCCATGTCTAACAACATGGCTTTCTCACC	3	1	1	0
tsrna-21576	TGCCCCCATGTCTAACAACATGGCTTTCTCA	1	0	1	0
tsrna-21575	TGCCCCCATGTCTAACAACATGGCTTTCTC	1	0	0	1
tsrna-21573	TGCCCCCATGTCTAACAACATGGCTTTC	0	1	0	0
tsrna-21566	TGCCCCCATGTCTAACAACA	0	0	1	0
tsrna-21561	TGCCCACATTCTCCACCA	1	1	0	1
tsrna-21560	TGCCATGATCGTATAGTGGTTAGTACTCTG	0	2	0	1
tsrna-21558	TGCCATGATCGTATAGTGGTTAGT	0	1	0	0
tsrna-21553	TGCCACGCGGGAGGCCCGGGTTCGATTCCCGGCCCATGCAC	24	48	11	15
tsrna-21552	TGCCACGCGGGAGGCCCGGGTTCGATTCCCGGCCAATGCAC	48	156	16	12
tsrna-21551	TGCCACGCGGGAGGCCCGGGTTCG	0	6	2	1
tsrna-21550	TGCCACGCGGGAGGCCCGGGTTC	3	7	2	0
tsrna-21549	TGCCACGCGGGAGGCCCGGGTT	1	3	0	0
tsrna-21548	TGCCACGCGGGAGGCCCGGGT	1	5	0	0
tsrna-21547	TGCCACGCGGGAGGCCCGGG	0	0	1	0
tsrna-21546	TGCCACGCGGGAGGCCCGG	0	1	0	0
tsrna-21545	TGCCACGCGGGAGGCCCG	0	1	0	0
tsrna-21543	TGCATTGGTGGTTCAGTGGTAGAATTCTCG	37	17	60	43
tsrna-21542	TGCATGTATGAGGTCCCGGGTT	0	5	0	0
tsrna-21541	TGCATGGGTGGTTCAGTGGTAGAATTCTCG	25	16	22	47
tsrna-21540	TGCATGGGTGGTTCAGTGGTAGA	5	5	4	8
tsrna-21539	TGCATGAGGTCCCGGGTTCGATCCCCAGC	1	11	0	1
tsrna-21538	TGCATGAGGTCCCGGGTTCG	0	4	0	0
tsrna-21537	TGCATGAGGTCCCGGGTTC	1	4	1	1
tsrna-21536	TGCATGAGGTCCCGGGT	2	2	0	0
tsrna-21535	TGCAGTGGTAGAATTCTCGCCT	2	7	2	1
tsrna-21534	TGCAGTGGTAGAATTCTCGCC	3	5	2	0
tsrna-21533	TGCAGTGGTAGAATTCTCGC	0	0	0	1

tsrna-21532	TGCAGTGGTAGAATTCTCG	0	0	0	0
tsrna-21529	TGCAGATCAAGAGGTCCCTGGT	1	4	0	1
tsrna-21528	TGCAGATCAAGAGGTCCCCGGT	0	5	2	0
tsrna-21526	TGCAGAGTGGGGTTTTGCAGTCCTTACCA	1	3	1	0
tsrna-21525	TGCAGAGTGGGGTTTTGCAGTCCT	0	0	1	0
tsrna-21524	TGCAGAGTGGGGTTTTGCAGTCC	0	1	0	0
tsrna-21523	TGCAGAGTGGGGTTTTGCAGTC	0	0	0	0
tsrna-21522	TGCAGAGTGGGGTTTTGCAGT	0	0	0	0
tsrna-21520	TGCAGAGTGGGGTTTTGC	0	0	0	0
tsrna-21517	TGCACGTATGAGGCCCCGGGTTCA	1	6	4	0
tsrna-21516	TGCACGTATGAGGCCCCGGGTT	2	2	1	1
tsrna-21515	TGCACGTATGAGGCCCCGGGT	2	4	1	1
tsrna-21514	TGCACGTATGAGGCCCCGGG	0	0	0	1
tsrna-21512	TGCACGCGTGGGTTCGAATCCCATCCTCGTCGC	0	0	0	1
tsrna-21511	TGCACGCGTGGGTTCGAATCCCATCCTCGT	0	1	0	0
tsrna-21510	TGCACGCGTGGGTTCGAATCCCATC	1	0	0	0
tsrna-21508	TGCACGCGTGGGTTCGAATCCCA	0	0	1	0
tsrna-21506	TGCACGCGTGGGTTCGAATCC	0	1	0	0
tsrna-21504	TGCACGCGTGGGTTCGAAT	0	1	1	0
tsrna-21502	TGCACGAGGTCCTGGGT	1	6	1	0
tsrna-21501	TGCACGAGGCCCCGGGTTCAATCCCCGGCACCTCCACC	17	28	13	3
tsrna-21500	TGCACGAGGCCCCGGGTTCAATCCCCGGCACCT	0	3	0	0
tsrna-21499	TGCACGAGGCCCCGGGTTCAATCCCCGGC	0	1	0	0
tsrna-21498	TGCACGAGGCCCCGGGTTCA	1	3	1	0
tsrna-21497	TGCACGAGGCCCCGGGTTC	0	5	1	0
tsrna-21496	TGCACGAGGCCCCGGGTT	0	1	0	0
tsrna-21493	TGCACACGTGGGTTCGAATCCCATCCTCGT	1	0	0	0
tsrna-21492	TGCACACGTGGGTTCGAATCCC	1	0	0	0
tsrna-21491	TGCAATACTTAATTTCTGCCA	2	0	0	1
tsrna-21490	TGCAATACTTAATTTCTGCC	2	0	0	0
tsrna-21487	TGCAAGATTCCCATTCTTGCGACCCG	0	3	0	1
tsrna-21486	TGCAACTCCAAATAAAAGTACCA	0	0	0	0
tsrna-21479	TGCAAATTCGAAGAAGCAGCTTCAAACCTGCCGGGGCTTCCA	12	23	6	9
tsrna-21478	TGCAAATTCGAAGAAGCAGCTTCAAACCTGCCGGGGCTT	0	4	1	0
tsrna-21475	TGATTTGCGTTCAGTTGATGCAGAGT	1	0	0	0
tsrna-21474	TGATTTGCGTTCAGTTGATGCA	1	0	1	0
tsrna-21472	TGATTTCGACTCATTAAATTATGATAATCATATTTACCAACC	36	6	70	3
tsrna-21471	TGATTTCGACTCATTAAATTATGATAATC	7	4	6	0
tsrna-21466	TGATTCTCGCTTTGGGTGCGAG	0	0	0	0
tsrna-21465	TGATTCTCGCTTTGGGTGCGA	0	0	0	0
tsrna-21456	TGATTCCGGATCAGAAGATTGAGGGT	3	6	0	2
tsrna-21455	TGATTCCGGATCAGAAGATTGAGG	0	1	0	0
tsrna-21451	TGATTAGGGTGCTTAGCTGTTA	3	0	4	1
tsrna-21450	TGATTAGGGTGCTTAGCTGTT	0	0	1	0
tsrna-21448	TGATGCAGAGTGGGGTTTTGCAGTCCTTAC	0	2	0	1
tsrna-21447	TGATGCAGAGTGGGGTTTTGCAGTCC	0	0	0	0
tsrna-21446	TGATGCAGAGTGGGGTTTTGCAGTC	0	0	0	0
tsrna-21445	TGATGCAGAGTGGGGTTTTGCA	0	1	0	0
tsrna-21444	TGATGCAGAGTGGGGTTTTGC	0	0	0	0
tsrna-21443	TGATCGTATAGTGGTTAGTACTCTGCGTTG	22	10	9	10
tsrna-21442	TGATCGTATAGTGGTTAGTACTCTGCGTT	16	9	10	5
tsrna-21441	TGATCGTATAGTGGTTAGTACTCTGCGT	7	6	1	2
tsrna-21440	TGATCGTATAGTGGTTAGTACTCTGCGCTG	6	2	2	4
tsrna-21439	TGATCGTATAGTGGTTAGTACTCTGCGC	3	5	2	2
tsrna-21438	TGATCGTATAGTGGTTAGTACTCTGCG	4	2	1	2
tsrna-21437	TGATCGTATAGTGGTTAGTACTCTGC	2	0	1	2
tsrna-21436	TGATCGTATAGTGGTTAGTACTCTG	2	4	2	0
tsrna-21435	TGATCGTATAGTGGTTAGTACTCT	2	1	0	2
tsrna-21434	TGATCGTATAGTGGTTAGTACTC	0	0	1	0
tsrna-21433	TGATCGTATAGTGGTTAGTACT	0	1	0	0
tsrna-21431	TGATCGTATAGTGGTTAGTA	0	0	0	0
tsrna-21427	TGATAGGTGGCACGGAGAATTTTGGATT	0	1	1	0
tsrna-21426	TGATAGGTGGCACGGAGAATTTTGGA	1	2	0	0
tsrna-21425	TGATAGGTGGCACGGAGAATTTTGG	1	0	1	1

tsrna-21424	TGATAGGTGGCACGGAGAATTTTG	0	1	1	1
tsrna-21422	TGATAGGTGGCACGGAGAATTT	0	0	1	0
tsrna-21421	TGATAGGTGGCACGGAGAATT	0	1	1	0
tsrna-21420	TGATAGGTGGCACGGAGAAT	0	0	0	0
tsrna-21418	TGATAGGTGGCACGGAGA	0	0	0	0
tsrna-21416	TGATAATCATATTTACCAACCA	0	1	0	0
tsrna-21410	TGATAACACCAAGGTCGCGGGC	0	6	4	0
tsrna-21408	TGAGTTCGATCCTCACACGGGGCACCA	0	6	0	0
tsrna-21407	TGAGTTCGAGCCTCACCTGGAGCACCA	0	5	0	0
tsrna-21402	TGAGTGAAGCATTGGACTGTAAATCTAAAG	1	1	1	0
tsrna-21401	TGAGTGAAGCATTGGACTGTAAATCTAAA	1	2	3	1
tsrna-21400	TGAGTGAAGCATTGGACTGTAAATCTAA	2	0	1	0
tsrna-21399	TGAGTGAAGCATTGGACTGTAAATCTA	0	1	2	0
tsrna-21398	TGAGTGAAGCATTGGACTGTAAATCT	0	2	1	0
tsrna-21397	TGAGTGAAGCATTGGACTGTAAATC	0	0	4	1
tsrna-21396	TGAGTGAAGCATTGGACTGTAAAT	0	0	0	0
tsrna-21395	TGAGTGAAGCATTGGACTGTAAA	1	1	1	0
tsrna-21394	TGAGTGAAGCATTGGACTGTAA	0	0	0	0
tsrna-21393	TGAGTGAAGCATTGGACTGTA	0	1	2	0
tsrna-21392	TGAGTGAAGCATTGGACTGT	0	0	0	0
tsrna-21388	TGAGTATCCCCGCCTGTCACGCGGGAGACC	21	47	8	3
tsrna-21387	TGAGTATCCCCGCCTGTCACGCGGG	10	21	3	3
tsrna-21386	TGAGTATCCCCGCCTGTCACGC	0	2	0	0
tsrna-21385	TGAGTATCCCCGCCTGTC	0	1	0	0
tsrna-21384	TGAGTATCCCCGCCTGT	1	0	0	0
tsrna-21380	TGAGGTCCCGGGTTCGATCCCCAGC	0	1	1	1
tsrna-21379	TGAGGTCCCGGGTTCGATCCCC	0	1	0	0
tsrna-21378	TGAGGTCCCGGGTTCGATCCC	0	1	0	0
tsrna-21377	TGAGGTCCCGGGTTCGATCC	0	2	0	1
tsrna-21376	TGAGGTCCCGGGTTCG	0	2	0	0
tsrna-21374	TGAGGGTCCAGGGTTCAAGTCCCTGTTCGGGCGC	0	3	0	1
tsrna-21373	TGAGGGTCCAGGGTTCAAGTCCCTGTTCGGGCG	0	0	2	0
tsrna-21372	TGAGGGTCCAGGGTTCAAGTCCCTGTTCGGGC	0	3	0	0
tsrna-21371	TGAGGGTCCAGGGTTCAAGTCCCT	0	1	0	0
tsrna-21366	TGAGGGTCCAGGGTTC	0	0	0	0
tsrna-21365	TGAGGCCCCGGGTTCGATCCCCGGCATC	0	1	0	0
tsrna-21364	TGAGGCCCCGGGTTCGATCCCCGGC	0	1	0	0
tsrna-21363	TGAGGCCCCGGGTTCGATCCCC	1	4	0	0
tsrna-21362	TGAGCACTCTGGACTCTGAATCCAGCG	1	3	1	1
tsrna-21361	TGAGCACTCTGGACTCTGAATCCAGC	2	3	0	0
tsrna-21360	TGAGCACTCTGGACTCTGAATCCA	0	3	3	0
tsrna-21359	TGAGCACTCTGGACTCTGAATCC	1	2	2	0
tsrna-21358	TGAGCACTCTGGACTCTGAATC	0	1	0	0
tsrna-21352	TGAGACTCTTAATCTC	0	1	0	0
tsrna-21349	TGACTTCGGATCAGAAGATTGAGGGT	1	5	1	2
tsrna-21348	TGACTTCGGATCAGAAGAT	1	0	0	0
tsrna-21347	TGACTGCAGATCAAGAGGTCCCTGGTTC	2	11	0	1
tsrna-21346	TGACTGCAGATCAAGAGGTCCCTGGTT	0	6	2	4
tsrna-21345	TGACTGCAGATCAAGAGGTCCCTGGT	4	6	4	1
tsrna-21341	TGACTGCAGATCAAGAGGTCCCCGGTTC	1	5	1	2
tsrna-21340	TGACTGCAGATCAAGAGGTCCCCGGTT	3	4	0	1
tsrna-21339	TGACTGCAGATCAAGAGGTCCCCGGT	1	7	2	0
tsrna-21329	TGACTCCAGATCAGAAGGTTGCGTGTT	1	4	0	1
tsrna-21328	TGACTCCAGATCAGAAGGTTGCGTGT	0	3	1	0
tsrna-21327	TGACTCCAGATCAGAAGGTTGCGTG	0	1	1	0
tsrna-21322	TGACTCCAGATCAGAAGGCTGCGTGTT	0	1	1	0
tsrna-21315	TGACTACGGATCAGAAGATTCTAGGTTCG	2	8	2	1
tsrna-21314	TGACCCGGGTTCGATTCCCGGCCAACGCACCA	46	77	21	12
tsrna-21310	GTGGGTTCGAATCCCACCCTCGTCGC	0	0	0	0
tsrna-21309	GTGGGTTCGAATCCCACCACTGCCAC	0	1	0	0
tsrna-21307	GTGGGTTCGAACCCCACTCCTGGTACCA	0	1	0	0
tsrna-21306	GTGGGTTCGAACCCCACTCCTGGTACC	0	0	0	0
tsrna-21301	GTGGGTAGAGCATTTGACTGC	1	2	0	1
tsrna-21299	GTGGGTAGAGCATTTGACT	0	2	0	0

tsrna-21298	GTGGGTAGAGCATTTGAC	0	1	0	0
tsrna-21296	GTGGGGTTTTGCAGTCCTTACCA	0	1	0	0
tsrna-21295	GTGGGGTTTTGCAGTCCTTACC	0	0	0	0
tsrna-21294	GTGGGGTTTTGCAGTCCTTA	0	2	0	0
tsrna-21293	GTGGGGTTTTGCAGTCCTT	0	1	0	0
tsrna-21292	GTGGGGTTTTGCAGTCCT	0	0	0	0
tsrna-21284	GTGGCTAGGATTCGGCGCTTTC	5	17	8	10
tsrna-21283	GTGGCTAGGATTCGGCGCTTT	5	17	2	9
tsrna-21282	GTGGCTAGGATTCGGCGCTT	4	22	1	5
tsrna-21281	GTGGCTAGGATTCGGCGCT	5	13	3	10
tsrna-21280	GTGGCTAGGATTCGGCGC	1	11	5	5
tsrna-21279	GTGGCTAGGATTCGGCG	0	15	1	11
tsrna-21278	GTGGCTAGGATTCGGC	3	14	1	1
tsrna-21277	GTGGCGCAGCGGAAGCGTGCTGGGCCC	10	48	5	7
tsrna-21276	GTGGCGCAGCGGAAGCGTGCTGGGCC	10	31	2	1
tsrna-21275	GTGGCGCAGCGGAAGCGTGCTGGGC	2	35	0	1
tsrna-21274	GTGGCGCAGCGGAAGCGTGCTGGG	0	21	4	1
tsrna-21273	GTGGCGCAGCGGAAGCGTGCTG	0	1	0	0
tsrna-21271	GTGGCGCAATGGATAGCGCATTGG	2	7	1	1
tsrna-21270	GTGGCGCAATGGATAGCGCATTG	0	0	0	0
tsrna-21268	GTGGCGCAACGGTAGCGCGTCTGACT	0	2	1	0
tsrna-21267	GTGGCCTAATGGATAAGGCGTCTGATTCCG	1	10	1	2
tsrna-21266	GTGGCCTAATGGATAAGGCATTGGCC	6	5	5	0
tsrna-21265	GTGGCCTAATGGATAAGGCATTG	5	1	1	0
tsrna-21264	GTGGCCTAATGGATAAGGCATCAGCCTC	0	4	0	1
tsrna-21263	GTGGCCTAATGGATAAGGCATCAGCCT	4	5	3	0
tsrna-21262	GTGGCCTAATGGATAAGGCATCAGC	5	2	3	1
tsrna-21261	GTGGCCTAATGGATAAGGCATCAG	2	4	1	0
tsrna-21260	GTGGCCTAATGGATAAGGCACTG	71	36	50	17
tsrna-21258	GTGGCCGCAGCAACCTCGGTTCG	0	2	0	0
tsrna-21257	GTGGCCGCAGCAACCTCGGTT	1	1	0	0
tsrna-21254	GTGGCCGCAGCAACCTCG	0	0	0	0
tsrna-21248	GTGGCCGAGCGGTCTAAGGCGCT	0	0	0	0
tsrna-21244	GTGGCACGGAGAATTTTGGATT	0	1	0	0
tsrna-21240	GTGGAGTTAAAGACTTTTTCTCTGACCA	24	38	11	22
tsrna-21239	GTGGAGTTAAAGACTTTTTCTCTGACC	4	13	1	1
tsrna-21238	GTGGAGTTAAAGACTTTTTCTCTGAC	0	1	0	0
tsrna-21236	GTGGAGTTAAAGACTTTTTCTCTG	0	2	1	0
tsrna-21235	GTGGAGTTAAAGACTTTTTCTCT	0	2	0	0
tsrna-21233	GTGGAGTTAAAGACTTTTTCT	0	0	1	0
tsrna-21231	GTGGAGTTAAAGACTTTTT	0	0	0	0
tsrna-21228	GTGGAGTTAAAGACTT	0	1	0	0
tsrna-21227	GTGGAGTGGTTATCACGTTCGCCT	0	1	0	0
tsrna-21225	GTGCTTTGCACGCGTGGGTT	1	2	1	0
tsrna-21222	GTGCTGGGCCCATAACCCAGAGGTCGATGGATC	5	10	1	1
tsrna-21218	GTGCTAATGGTGGAGTTAAAGACTTTTTCTCTGACC	6	22	5	1
tsrna-21217	GTGCTAATGGTGGAGTTAAAGACTTTTTCT	7	15	4	0
tsrna-21216	GTGCTAATGGTGGAGTTAAAGACT	0	6	4	0
tsrna-21215	GTGCTAATGGTGGAGTTAAAGAC	1	4	0	0
tsrna-21214	GTGCTAATGGTGGAGTTAAAGA	1	2	1	0
tsrna-21213	GTGCTAATGGTGGAGTTAAAG	1	0	0	2
tsrna-21212	GTGCTAATGGTGGAGTTAA	0	5	2	0
tsrna-21211	GTGCTAATGGTGGAGTTA	0	3	0	0
tsrna-21210	GTGCTAATGGTGGAGTT	0	0	0	0
tsrna-21208	GTGCGAGAGGTCCCGGGTTC	0	4	0	0
tsrna-21207	GTGCGAGAGGTCCCGGGTT	0	1	0	0
tsrna-21206	GTGCGAGAGGTCCCGGGT	1	0	0	0
tsrna-21204	GTGCAGTGGTAGAATTCTCGCCT	1	3	0	2
tsrna-21203	GTGCAGTGGTAGAATTCTCGCC	2	7	1	1
tsrna-21202	GTGCAGTGGTAGAATTCTCGC	0	1	0	0
tsrna-21201	GTGCAGTGGTAGAATTCTCG	0	0	0	0
tsrna-21198	GTGCAACTCCAAATAAAAGTACCA	0	0	1	0
tsrna-21197	GTGCAACTCCAAATAAAAGTACC	0	2	0	0
tsrna-21196	GTGCAACTCCAAATAAAAGTAC	0	1	0	0

tsrna-21193	GTGCAACTCCAAATAAAAG	0	0	0	1
tsrna-21192	GTGCAACTCCAAATAAAA	0	1	0	0
tsrna-21190	GTGCAACTCCAAATAA	0	0	1	0
tsrna-21187	GTGATGGCCGAGTGGTTAAGGC	0	1	0	0
tsrna-21185	GTGATCGTATAGTGGTTAGTACTCTGCGTTG	24	16	13	8
tsrna-21184	GTGATCGTATAGTGGTTAGTACTCTGCGTT	23	17	5	2
tsrna-21183	GTGATCGTATAGTGGTTAGTACTCTGCGT	8	3	0	1
tsrna-21182	GTGATCGTATAGTGGTTAGTACTCTGCG	5	2	1	0
tsrna-21181	GTGATCGTATAGTGGTTAGTACTCTGC	3	2	0	0
tsrna-21180	GTGATCGTATAGTGGTTAGTACTCTG	0	1	1	2
tsrna-21179	GTGATCGTATAGTGGTTAGTACTCT	1	1	1	1
tsrna-21178	GTGATCGTATAGTGGTTAGTACTC	0	0	0	1
tsrna-21177	GTGATCGTATAGTGGTTAGTACT	1	0	1	0
tsrna-21169	GTGATAGGTGGCACGGAGAATTTTG	0	4	1	0
tsrna-21165	GTGAGTATCCCCGCCTGTCACGCGGGAGAC	17	77	10	4
tsrna-21164	GTGAGTATCCCCGCCTGTCACGCGGG	4	28	6	0
tsrna-21163	GTGAGTATCCCCGCCTGTCACGC	4	7	0	0
tsrna-21162	GTGAGTATCCCCGCCTGTCACG	2	3	1	0
tsrna-21161	GTGAGTATCCCCGCCTGTCAC	1	3	1	0
tsrna-21160	GTGAGTATCCCCGCCTGTCA	0	3	1	1
tsrna-21159	GTGAGTATCCCCGCCTGTC	3	4	1	1
tsrna-21158	GTGAGTATCCCCGCCTGT	0	0	0	1
tsrna-21157	GTGAGTATCCCCGCCTG	0	0	0	0
tsrna-21156	GTGAGTATCCCCGCCT	0	1	0	1
tsrna-21155	GTGAGCATAGCTGCCTTCCAAGCAG	0	0	0	0
tsrna-21154	GTGAGCATAGCTGCCTTCCAAGCA	1	0	0	0
tsrna-21151	GTGAGCACTCTGGACTCTGAATCCAGCG	2	2	1	0
tsrna-21150	GTGAGCACTCTGGACTCTGAATCCAGC	3	4	1	1
tsrna-21149	GTGAGCACTCTGGACTCTGAATCCA	1	2	0	1
tsrna-21148	GTGAGCACTCTGGACTCTGAATCC	1	0	0	0
tsrna-21147	GTGAGCACTCTGGACTCTGAATC	1	1	0	0
tsrna-21140	GTGAGAGGTCCCGGGTTCAAATCCCGGACG	1	1	0	0
tsrna-21138	GTGAATCTGACAACAGAGGCTTACGACCCCTTATTTACCCCA	24	9	13	0
tsrna-21137	GTGAATCTGACAACAGAGGCTTACGACCCCTTATTTACCCC	17	7	7	0
tsrna-21136	GTGAATCTGACAACAGAGGCTTACGACCCCTTATTTACC	10	8	5	0
tsrna-21135	GTGAATCTGACAACAGAGGCTTACGACCCCTTATTT	14	6	6	0
tsrna-21134	GTGAATCTGACAACAGAGGCTTACGACCCC	1	1	0	0
tsrna-21133	GTGAATCTGACAACAGAGGCTTACGACCC	0	0	0	0
tsrna-21132	GTGAATCTGACAACAGAGGCTTACGA	0	0	1	0
tsrna-21129	GTGAATCTGACAACAGAGGCTTA	0	0	0	0
tsrna-21128	GTGAATCTGACAACAGAGGCTT	0	1	0	0
tsrna-21127	GTGAATCTGACAACAGAGGCT	0	1	0	0
tsrna-21126	GTGAATCTGACAACAGAGGC	0	1	0	0
tsrna-21121	GTGAAGCATTGGACTGTAAATCTAAAGAC	0	2	1	0
tsrna-21120	GTGAAGCATTGGACTGTAAATCTAAA	0	0	1	0
tsrna-21119	GTGAAGCATTGGACTGTAAATCTAA	1	0	0	0
tsrna-21113	GTGAAGCATTGGACTGTAA	0	0	0	0
tsrna-21112	GTGAAGCATTGGACTGTA	0	0	1	0
tsrna-21109	GTCTTGTAAACCAGGGGTCGCGA	1	3	1	1
tsrna-21108	GTCTTGTAAACCAGGGGTCGCG	1	6	1	0
tsrna-21104	GTCTCTTCGGGGGCGTGGGTTCG	1	4	0	0
tsrna-21103	GTCTCTTCGGGGGCGTGGGTTC	1	1	0	1
tsrna-21102	GTCTCTTCGGGGGCGTGGGTT	0	4	0	1
tsrna-21088	GTCTCTGTGGCGCAATGGGT	1	0	1	0
tsrna-21087	GTCTCTGTGGCGCAATGGG	0	1	0	0
tsrna-21086	GTCTCTGTGGCGCAATGGACGAGCGC	0	0	0	0
tsrna-21083	GTCTCTGTGGCGCAATGGACGAG	0	1	0	0
tsrna-21082	GTCTCTGTGGCGCAATGGACGA	0	0	0	0
tsrna-21080	GTCTCTGTGGCGCAATGGAC	0	0	0	0
tsrna-21079	GTCTCTGTGGCGCAATGGA	0	0	0	0
tsrna-21078	GTCTCTGTGGCGCAATGG	0	0	1	0
tsrna-21077	GTCTCTGTGGCGCAATG	0	0	2	0
tsrna-21076	GTCTCTGTGGCGCAATCGGTTAGCGCGTTC	0	2	0	0
tsrna-21075	GTCTCTGTGGCGCAATCGGTTAGCGCATTC	1	0	1	0

tsrna-21074	GTCTCTGTGGCGCAATCGGTTAGCGC	0	2	0	0
tsrna-21073	GTCTCTGTGGCGCAATCGGTTAGCG	0	0	0	0
tsrna-21071	GTCTCTGTGGCGCAATCGGTTAG	2	0	0	0
tsrna-21070	GTCTCTGTGGCGCAATCGGTTA	0	0	0	0
tsrna-21068	GTCTCTGTGGCGCAATCGGTC	0	1	0	0
tsrna-21067	GTCTCTGTGGCGCAATCGGT	0	0	0	0
tsrna-21060	GTCTCTGTGGAACCTCCA	0	2	2	0
tsrna-21059	GTCTCGGTGGAACCTCCA	42	94	7	11
tsrna-21058	GTCTCGGTGGAACCTCC	5	4	1	1
tsrna-21057	GTCTCGGTGGAACCTC	1	1	0	0
tsrna-21056	GTCTCCGGATGGAGGCGTGGG	1	8	1	0
tsrna-21055	GTCTCCGGATGGAGGCGTGG	1	6	0	0
tsrna-21054	GTCTCCGGATGGAGGCGTG	2	5	0	0
tsrna-21050	GTCTCATAATCTGAAGGTCGTGAGT	25	50	25	11
tsrna-21049	GTCTCATAATCTGAAGGTCGTG	3	7	3	2
tsrna-21048	GTCTCATAATCTGAAGGTCCTGAGT	129	326	83	62
tsrna-21047	GTCTCATAATCTGAAGGTCCTGA	2	21	7	3
tsrna-21046	GTCTCATAATCTGAAGGTCCTG	4	15	4	5
tsrna-21045	GTCTCATAATCTGAAGGTCCT	1	3	1	0
tsrna-21044	GTCTCATAATCTGAAGGTCC	0	5	2	0
tsrna-21043	GTCTAGTGGTTAGGATTCGGCGCTCTCACCGCCGCGGCCCG(	51	63	33	25
tsrna-21042	GTCTAGTGGTTAGGATTCGGCGCTCTCACCGCCGCGGCCCG(	69	73	35	17
tsrna-21041	GTCTAGTGGTTAGGATTCGGCGCTCTCACC	46	47	18	17
tsrna-21040	GTCTAGTGGTTAGGATTCGGCGCTCTCA	37	40	28	27
tsrna-21039	GTCTAGTGGTTAGGATTCGGCGCTCTC	42	42	22	22
tsrna-21038	GTCTAGTGGTTAGGATTCGGCGCTCT	22	48	28	12
tsrna-21037	GTCTAGTGGTTAGGATTCGGCGCTC	52	50	22	16
tsrna-21036	GTCTAGTGGTTAGGATTCGGCGCT	32	41	18	14
tsrna-21035	GTCTAGTGGTTAGGATTCGGCGC	17	14	16	8
tsrna-21034	GTCTAGTGGTTAGGATTCGGCG	13	9	9	3
tsrna-21033	GTCTAGTGGTTAGGATTCGGC	6	5	4	1
tsrna-21032	GTCTAGTGGTTAGGATTCGG	4	0	3	0
tsrna-21031	GTCTAGTGGTTAGGATTCG	4	0	1	1
tsrna-21030	GTCTAGTGGTTAGGATTC	1	0	1	0
tsrna-21029	GTCTAGTGGTTAGGATT	2	0	1	0
tsrna-21028	GTCTAGTGGTTAGGAT	1	0	0	0
tsrna-21026	GTCTAGTGGCTAGGATTCGGCGCTTT	11	41	9	27
tsrna-21025	GTCTAGTGGCTAGGATTCGGCGCT	17	46	6	28
tsrna-21024	GTCTAGTGGCTAGGATTCGGCGC	10	31	5	20
tsrna-21023	GTCTAGTGGCTAGGATTCGGC	7	22	3	9
tsrna-21020	GTCTAGGGGTATGATTCTCGGTTTG	5	3	0	1
tsrna-21019	GTCTAGGGGTATGATTCTCGGTT	10	2	3	2
tsrna-21018	GTCTAGGGGTATGATTCTCGGT	0	2	1	1
tsrna-21017	GTCTAGGGGTATGATTCTCGG	1	0	1	2
tsrna-21016	GTCTAGGGGTATGATTCTCGCTTC	17	21	14	15
tsrna-21015	GTCTAGGGGTATGATTCTCGCTT	16	13	7	12
tsrna-21014	GTCTAGGGGTATGATTCTCGCT	14	14	12	18
tsrna-21013	GTCTAGGGGTATGATTCTCGC	14	9	7	12
tsrna-21012	GTCTAGGGGTATGATTCTCG	0	0	0	0
tsrna-21011	GTCTAGGGGTATGATTCTC	0	0	0	1
tsrna-21008	GTCTAGCGGTTAGGATTCCTGGTTTTCACC	9	13	6	4
tsrna-21007	GTCTAGCGGTTAGGATTCCTGGTTTTC	5	9	1	5
tsrna-21006	GTCTAGCGGTTAGGATTCCTGGTTTT	2	4	0	1
tsrna-21005	GTCTAGCGGTTAGGATTCCTGGTTT	3	4	1	0
tsrna-21004	GTCTAGCGGTTAGGATTCCTGGTT	8	6	1	1
tsrna-21003	GTCTAGCGGTTAGGATTCCTGGT	1	3	0	3
tsrna-21002	GTCTAGCGGTTAGGATTCCTGG	0	3	1	0
tsrna-21001	GTCTAGCGGTTAGGATTCCTG	1	0	0	0
tsrna-20993	GTCTAAGGCGCCAGACTCAAG	0	0	0	1
tsrna-20992	GTCTAACAACATGGCTTTCTCACCA	0	3	2	0
tsrna-20991	GTCTAACAACATGGCTTTCTCACC	0	1	0	0
tsrna-20983	GTCGTGGTTGTAGTCCGTGCGAGAATACCA	13	22	12	12
tsrna-20982	GTCGTGGTTGTAGTCCGTGCGAGAATACC	3	4	3	1
tsrna-20981	GTCGTGGTTGTAGTCCGTGCGAGAATAC	2	2	2	1

tsrna-20980	GTCGTGGTTGTAGTCCGTGCGAGAATA	1	3	1	0
tsrna-20979	GTCGTGGTTGTAGTCCGTGCGAGAAT	0	3	0	0
tsrna-20978	GTCGTGGTTGTAGTCCGTGCGAGAA	2	1	3	0
tsrna-20977	GTCGTGGTTGTAGTCCGTGCGAGA	0	3	1	0
tsrna-20976	GTCGTGGTTGTAGTCCGTGCGAG	0	1	1	0
tsrna-20975	GTCGTGGTTGTAGTCCGTGCGA	0	0	2	0
tsrna-20974	GTCGTGGTTGTAGTCCGTGCG	1	0	1	0
tsrna-20968	GTCGTGGGTTCGAGCCCCACGTTGGGCGCCA	15	216	3	2
tsrna-20967	GTCGTGGGTTCGAGCCCCACGTTGGGCGC	0	2	0	0
tsrna-20966	GTCGTGGGTTCGAGCCCCACGTTGGGCG	0	1	0	0
tsrna-20963	GTCGTGGCCGAGTGGTTAAG	0	0	0	0
tsrna-20961	GTCGGTGGAGCATGGGACT	2	0	0	2
tsrna-20960	GTCGGTGGAGCATGGGAC	2	0	0	0
tsrna-20958	GTCGGTAGAGCATGGGACTCTTAATCCC	10	27	15	5
tsrna-20957	GTCGGTAGAGCATGGGACTCTTAATCC	14	27	8	6
tsrna-20956	GTCGGTAGAGCATGGGACTCTTAATC	9	16	4	1
tsrna-20955	GTCGGTAGAGCATGGGACTCTTAAT	3	16	3	1
tsrna-20954	GTCGGTAGAGCATGGGACTCTTA	9	18	4	2
tsrna-20953	GTCGGTAGAGCATGGGACTCTT	8	17	2	2
tsrna-20952	GTCGGTAGAGCATGGGACTCT	4	13	4	0
tsrna-20951	GTCGGTAGAGCATGGGACTC	11	14	3	0
tsrna-20950	GTCGGTAGAGCATGGGACT	6	4	2	2
tsrna-20949	GTCGGTAGAGCATGGGAC	3	11	6	2
tsrna-20948	GTCGGTAGAGCATGGGA	7	8	4	0
tsrna-20947	GTCGGTAGAGCATGGG	1	2	0	0
tsrna-20946	GTCGGTAGAGCATGAGACTCTTAATCTC	14	16	6	8
tsrna-20945	GTCGGTAGAGCATGAGACTCTTAATCT	11	24	11	1
tsrna-20944	GTCGGTAGAGCATGAGACTCTTAA	11	17	3	8
tsrna-20943	GTCGGTAGAGCATGAGACTCTT	10	30	3	3
tsrna-20942	GTCGGTAGAGCATGAGACTCT	18	16	6	4
tsrna-20941	GTCGGTAGAGCATGAGACTC	12	27	4	3
tsrna-20940	GTCGGTAGAGCATGAGACT	7	25	6	0
tsrna-20939	GTCGGTAGAGCATGAGAC	10	19	1	2
tsrna-20938	GTCGGTAGAGCATGAGA	1	6	0	1
tsrna-20937	GTCGGTAGAGCATGAG	2	4	1	0
tsrna-20936	GTCGGTAGAGCATCAGACTTTTAATCT	5	7	2	0
tsrna-20935	GTCGGTAGAGCATCAGACTTTTAATC	2	3	1	0
tsrna-20934	GTCGGTAGAGCATCAGACTTTTAAT	4	5	0	0
tsrna-20933	GTCGGTAGAGCATCAGACTTTTAA	1	2	1	0
tsrna-20932	GTCGGTAGAGCATCAGACTTTTA	4	3	1	0
tsrna-20931	GTCGGTAGAGCATCAGACTTTT	0	3	0	0
tsrna-20930	GTCGGTAGAGCATCAGACTTT	0	1	2	0
tsrna-20929	GTCGGTAGAGCATCAGACTT	2	0	2	0
tsrna-20928	GTCGGTAGAGCATCAGACT	1	6	0	0
tsrna-20927	GTCGGTAGAGCATCAGAC	0	1	3	0
tsrna-20926	GTCGGTAGAGCATCAGA	2	0	0	0
tsrna-20925	GTCGGTAGAGCATCAG	1	0	0	0
tsrna-20924	GTCGCTGGTTCGATTCCGGCTCGAAGGACC	6	27	1	5
tsrna-20917	GTCGCAGTCTCCCCTGGAGG	0	2	0	0
tsrna-20916	GTCGATTCCCCGACGGGGAGCCA	0	13	0	0
tsrna-20914	GTCGATGGATCGAAACCATCCTCTGCTACC	3	2	0	0
tsrna-20912	GTCCTTGTAGTATAAACTAATACACCAGTC	1	1	3	1
tsrna-20911	GTCCTTGTAGTATAAACTAATACACCAGT	6	2	4	2
tsrna-20910	GTCCTTGTAGTATAAACTAATACACCAG	7	2	1	3
tsrna-20909	GTCCTTGTAGTATAAACTAATACACCA	6	1	0	0
tsrna-20908	GTCCTTGTAGTATAAACTAATACACC	10	1	0	3
tsrna-20907	GTCCTTGTAGTATAAACTAATACAC	8	1	1	2
tsrna-20906	GTCCTTGTAGTATAAACTAATACA	6	1	2	3
tsrna-20905	GTCCTTGTAGTATAAACTAATAC	7	1	2	2
tsrna-20904	GTCCTTGTAGTATAAACTAATA	6	1	2	1
tsrna-20903	GTCCTTGTAGTATAAACTAAT	3	2	0	0
tsrna-20902	GTCCTTGTAGTATAAACTAA	3	3	1	0
tsrna-20901	GTCCTTGTAGTATAAACTA	0	0	0	1
tsrna-20900	GTCCTTGTAGTATAAACT	0	0	1	1

tsrna-20899	GTCCTTGTAGTATAAAC	1	0	0	0
tsrna-20891	GTCCTGCCGCGGTCGCCA	1	4	2	0
tsrna-20887	GTCCTCGTTAGTATAGTGGTGAG	1	0	0	0
tsrna-20886	GTCCGTGCGAGAATACCA	1	0	0	0
tsrna-20883	GTCCCTTCGTGGTCGCCA	0	1	0	0
tsrna-20881	GTCCCTGTTCGGGCGCCA	8	9	3	2
tsrna-20878	GTCCCTGTTCAGGCGCCA	0	0	0	0
tsrna-20872	GTCCCTGGTTCGATCCCGG	0	0	0	0
tsrna-20863	GTCCCGGGTTCAAATCCCGGACGAG	0	0	0	0
tsrna-20855	GTCCCGGCGGAGTCGCCA	0	0	0	0
tsrna-20845	GTCCCATTGGTCTAGCCA	0	1	0	0
tsrna-20842	GTCCCATGGTGTAATGGTTAGCACTCTGG	0	1	1	0
tsrna-20837	GTCCCATCTGGGTCGCCA	1	4	1	1
tsrna-20836	GTCCCATCTGGGTCGCC	0	2	0	0
tsrna-20835	GTCCCATCTGGGGTGCCA	0	0	0	0
tsrna-20828	GTCCCACCAGAGTCGCCA	0	1	0	0
tsrna-20825	GTCCAGGGTTCAAGTCCCTGTTCGGGCGCCA	5	14	3	3
tsrna-20824	GTCCAGGGTTCAAGTCCCTGTTCGGGCGCC	1	0	0	0
tsrna-20823	GTCCAGGGTTCAAGTCCCTGTTCGGGCGC	0	0	0	1
tsrna-20822	GTCATTTCGATGGCGTGGGTTCG	3	4	5	0
tsrna-20821	GTCATTTCGATGGCGTGG	0	1	0	0
tsrna-20820	GTCATGGAGGCCATGGGGTTGGCTTGA	0	1	1	1
tsrna-20819	GTCATGGAGGCCATGGGGTTGGC	0	1	0	0
tsrna-20818	GTCATGGAGGCCATGGGGTTGG	0	1	0	0
tsrna-20817	GTCATGGAGGCCATGGGGTTG	0	1	0	0
tsrna-20816	GTCATGGAGGCCATGGGGTT	0	0	1	0
tsrna-20811	GTCAGGATGGCCGAGTGGTCTAAGGCGCCAGACT	2	7	2	0
tsrna-20810	GTCAGGATGGCCGAGTGGTCTAAGGCGCCAGAC	0	6	3	1
tsrna-20809	GTCAGGATGGCCGAGTGGTCTAAGGCGCCAGA	3	5	0	1
tsrna-20808	GTCAGGATGGCCGAGTGGTCTAAGGCGCCA	3	5	2	6
tsrna-20807	GTCAGGATGGCCGAGTGGTCTAAGGCGCC	6	3	5	4
tsrna-20806	GTCAGGATGGCCGAGTGGTCTAAGGCGC	2	7	0	4
tsrna-20805	GTCAGGATGGCCGAGTGGTCTAAGGC	3	12	4	4
tsrna-20804	GTCAGGATGGCCGAGTGGTCTAAGG	0	5	0	3
tsrna-20803	GTCAGGATGGCCGAGTGGTCTAAG	2	7	0	0
tsrna-20802	GTCAGGATGGCCGAGTGGTCTAA	3	7	1	1
tsrna-20801	GTCAGGATGGCCGAGTGGTCTA	0	6	3	1
tsrna-20800	GTCAGGATGGCCGAGTGGTCT	1	10	0	2
tsrna-20799	GTCAGGATGGCCGAGTGGTC	4	6	2	1
tsrna-20798	GTCAGGATGGCCGAGTGGT	5	2	3	2
tsrna-20797	GTCAGGATGGCCGAGTGG	0	3	1	0
tsrna-20796	GTCAGGATGGCCGAGTG	3	2	2	0
tsrna-20795	GTCAGGATGGCCGAGT	1	6	3	0
tsrna-20794	GTCAGGATGGCCGAGCGGTCTAAGGCGCTGCGTTC	5	12	1	3
tsrna-20793	GTCAGGATGGCCGAGCGGTCTAAGGCGCTGCGTT	2	11	3	6
tsrna-20792	GTCAGGATGGCCGAGCGGTCTAAGGCGCTGCGT	5	13	3	0
tsrna-20791	GTCAGGATGGCCGAGCGGTCTAAGGCGCTGCG	6	16	5	2
tsrna-20790	GTCAGGATGGCCGAGCGGTCTAAGGCGCTGC	4	14	4	2
tsrna-20789	GTCAGGATGGCCGAGCGGTCTAAGGCGCTG	6	18	4	4
tsrna-20788	GTCAGGATGGCCGAGCGGTCTAAGGCGCT	3	12	2	6
tsrna-20787	GTCAGGATGGCCGAGCGGTCTAAGGCGC	3	11	1	5
tsrna-20786	GTCAGGATGGCCGAGCGGTCTAAGGCG	8	20	0	5
tsrna-20785	GTCAGGATGGCCGAGCGGTCTAAGGC	4	16	2	3
tsrna-20784	GTCAGGATGGCCGAGCGGTCTAAGG	5	10	4	3
tsrna-20783	GTCAGGATGGCCGAGCGGTCTAAG	3	5	3	3
tsrna-20782	GTCAGGATGGCCGAGCGGTCTAA	3	4	0	3
tsrna-20781	GTCAGGATGGCCGAGCGGTCTA	2	6	4	2
tsrna-20780	GTCAGGATGGCCGAGCGGTCT	1	5	1	3
tsrna-20779	GTCAGGATGGCCGAGCGGTC	2	8	0	2
tsrna-20778	GTCAGGATGGCCGAGCGGT	0	9	0	1
tsrna-20777	GTCAGGATGGCCGAGCGG	0	7	0	1
tsrna-20776	GTCAGGATGGCCGAGCG	1	16	2	1
tsrna-20775	GTCAGGATGGCCGAGC	1	8	0	1
tsrna-20774	GTCAGCTAAATAAGCTATCGGGCCCAT	1	1	0	0

tsrna-20773	GTCAGCTAAATAAGCTATCGGGCCCA	0	1	0	0
tsrna-20772	GTCAGCTAAATAAGCTATCGGGCCC	1	0	0	0
tsrna-20767	GTCAGCACTCTGGACTCTGAATCCAGC	3	4	0	0
tsrna-20766	GTCAGCACTCTGGACTCTGAATCC	0	0	0	0
tsrna-20765	GTCAGCACTCTGGACTCTGAATC	1	0	0	0
tsrna-20758	GTCACGTCGGGGTCACCA	7	7	1	4
tsrna-20757	GTCACGTCGGGGTCACC	0	0	0	0
tsrna-20755	GTCACGGTGGCCGAGTGGTTAAGGCGTTGG	1	5	2	0
tsrna-20754	GTCACGGTGGCCGAGTGGTTAAGGCGTTG	3	6	0	1
tsrna-20753	GTCACGGTGGCCGAGTGGTTAAGGC	1	5	2	1
tsrna-20752	GTCACGGTGGCCGAGTGGTTAAGG	3	9	1	0
tsrna-20751	GTCACGGTGGCCGAGTGGTTAAG	1	12	0	0
tsrna-20750	GTCACGGTGGCCGAGTGGTTAA	1	4	0	1
tsrna-20749	GTCACGGTGGCCGAGTGGTTA	2	7	1	0
tsrna-20748	GTCACGGTGGCCGAGTGGTT	1	6	0	1
tsrna-20747	GTCACGGTGGCCGAGTGGT	1	4	0	0
tsrna-20746	GTCACGGTGGCCGAGTGG	1	11	0	0
tsrna-20745	GTCACGGTGGCCGAGTG	1	3	0	0
tsrna-20744	GTCACGGTGGCCGAGT	0	3	0	0
tsrna-20743	GTCACGCGGGAGACCGGGGTTCGATTCCCCGACGGGGAGC(	0	20	1	0
tsrna-20737	GTCAAAGTTAAATTATAGGCTAAATCCTA	7	0	2	1
tsrna-20736	GTCAAAGTTAAATTATAGGCTAAATCCT	2	1	1	0
tsrna-20735	GTCAAAGTTAAATTATAGGCT	0	0	1	0
tsrna-20734	GTCAAAGTTAAATTATAGGC	0	0	2	0
tsrna-20733	GTCAAAGTTAAATTATAGG	0	1	0	0
tsrna-20730	GTATGATTCTCGGTTTGGGTCCGAGA	2	0	0	0
tsrna-20728	GTATGATTCTCGGTTTGGGTC	0	0	1	0
tsrna-20727	GTATGATTCTCGGTTTGGGT	1	0	0	0
tsrna-20725	GTATGATTCTCGGTTTGG	0	1	0	0
tsrna-20722	GTATGATTCTCGCTTTGGGTGCGAGAGGTCCCGGGT	8	55	7	15
tsrna-20721	GTATGATTCTCGCTTTGGGTG	1	0	0	0
tsrna-20718	GTATGATTCTCGCTTT	0	1	0	0
tsrna-20715	GTATGATTCTCGCTTCGGGTGCGAGAGGTC	1	0	0	0
tsrna-20712	GTATGATTCTCGCTTCGGGTG	1	1	0	0
tsrna-20711	GTATGATTCTCGCTTCGGG	1	1	0	0
tsrna-20710	GTATGATTCTCGCTTCGG	1	0	0	0
tsrna-20707	GTATGAGGTCCCGGGTTCGATCCCCGGCATCTCCACC	16	28	6	3
tsrna-20706	GTATGAGGTCCCGGGTTCGATCCCCGGC	4	1	1	0
tsrna-20705	GTATGAGGTCCCGGGTTCGAT	0	4	0	0
tsrna-20704	GTATGAGGTCCCGGGTTCG	0	4	1	2
tsrna-20703	GTATGAGGTCCCGGGTTC	1	2	0	0
tsrna-20702	GTATGAGGTCCCGGGTT	0	4	0	0
tsrna-20701	GTATGAGGTCCCGGGT	0	5	0	0
tsrna-20700	GTATGAGGCCTCGGGT	1	5	2	0
tsrna-20699	GTATGAGGCCCCGGGTTCGATCCCCGGCATCTCCACC	11	20	11	0
tsrna-20698	GTATGAGGCCCCGGGTTCGATCCCCGGC	0	2	0	1
tsrna-20697	GTATGAGGCCCCGGGTTCGATCC	0	3	0	1
tsrna-20696	GTATGAGGCCCCGGGTTCGAT	0	5	0	0
tsrna-20695	GTATGAGGCCCCGGGTTCG	1	0	1	0
tsrna-20694	GTATGAGGCCCCGGGTTCAATCCCCGGC	3	1	1	0
tsrna-20693	GTATGAGGCCCCGGGTTC	0	4	0	1
tsrna-20692	GTATGAGGCCCCGGGTT	0	2	0	0
tsrna-20691	GTATGAGGCCCCGGGT	0	1	0	0
tsrna-20690	GTATCCCCGCCTGTCACGCGGGAGACCGG	9	41	11	4
tsrna-20689	GTATCCCCGCCTGTCACGCGGG	6	29	2	1
tsrna-20683	GTATCATGCAAGATTCCCATTC	0	0	0	0
tsrna-20678	GTATAGTGGTTAGTATCCCCG	0	0	1	0
tsrna-20673	GTATAGTGGTTAGTACTCTGCGTTGTGGCC	25	11	14	10
tsrna-20672	GTATAGTGGTTAGTACTCTGCGTTGTGGC	35	15	5	6
tsrna-20671	GTATAGTGGTTAGTACTCTGCGTTGTG	24	13	11	7
tsrna-20670	GTATAGTGGTTAGTACTCTGCGTTGT	26	17	15	8
tsrna-20669	GTATAGTGGTTAGTACTCTGCGTTG	19	16	10	10
tsrna-20668	GTATAGTGGTTAGTACTCTGCGTT	12	7	9	6
tsrna-20667	GTATAGTGGTTAGTACTCTGCGT	4	3	0	2

tsrna-20666	GTATAGTGGTTAGTACTCTGCG	2	1	0	1
tsrna-20665	GTATAGTGGTTAGTACTCTGC	1	0	0	1
tsrna-20664	GTATAGTGGTTAGTACTCTG	0	0	1	0
tsrna-20662	GTATAGTGGTTAGTACTC	0	0	0	0
tsrna-20658	GTATAGTGGTTAGCATAGCTGCCTT	0	0	0	0
tsrna-20653	GTATAGTGGTGAGTATCCCCGCCTGTC	2	11	1	1
tsrna-20652	GTATAGTGGTGAGTATCCCCGCCTGT	1	6	0	1
tsrna-20651	GTATAGTGGTGAGTATCCCCGCCTG	2	3	0	1
tsrna-20650	GTATAGTGGTGAGTATCCCCGCCT	1	6	0	0
tsrna-20649	GTATAGTGGTGAGTATCCCCGCC	3	4	2	0
tsrna-20648	GTATAGTGGTGAGTATCCCCGC	1	3	0	0
tsrna-20647	GTATAGTGGTGAGTATCCCCG	0	1	0	0
tsrna-20645	GTATAGTGGTGAGTATCCC	0	1	0	0
tsrna-20644	GTATAGTGGTGAGTATCC	1	1	0	0
tsrna-20640	GTATAGTGGTGAGCATAGCTGCCTTC	0	1	0	0
tsrna-20639	GTATAGTGGTGAGCATAGCTGCCTT	1	1	0	1
tsrna-20638	GTATAGTGGTGAGCATAGCTGCCT	0	1	1	0
tsrna-20636	GTATAGTGGTGAGCATAGCTGC	0	1	0	0
tsrna-20635	GTATAGTGGTGAGCATAGCTG	0	1	0	0
tsrna-20633	GTATAGTGGTGAGCATAGC	1	0	0	0
tsrna-20629	GTATAGCTCAGTGGTAGAGCATTTGACTGC	8	6	4	0
tsrna-20628	GTATAGCTCAGTGGTAGAGCATTTGACTG	6	1	0	1
tsrna-20627	GTATAGCTCAGTGGTAGAGCATTTGACTAC	3	4	0	1
tsrna-20626	GTATAGCTCAGTGGTAGAGCATTTGACT	1	2	1	0
tsrna-20625	GTATAGCTCAGTGGTAGAGCATTTGAC	4	0	0	0
tsrna-20624	GTATAGCTCAGTGGTAGAGCATTTG	1	1	0	0
tsrna-20621	GTATAGCTCAGTGGGTAGAGCATTTGACTG	2	0	0	1
tsrna-20620	GTATAGCTCAGTGGGTAGAGCATTTGACT	0	1	0	0
tsrna-20619	GTATAGCTCAGGTGGTAGAGCATTTGACTG	1	0	0	0
tsrna-20618	GTATAGCTCAGGGGTAGAGCATTTGACTGC	2	3	3	8
tsrna-20617	GTATAGCTCAGGGGTAGAGCATTTGACTG	0	0	0	2
tsrna-20616	GTATAGCTCAGGGGTAGAGCATTTGACT	0	3	0	2
tsrna-20615	GTATAAATAGTACCGTTAACTTCC	1	0	1	0
tsrna-20614	GTATAAATAGTACCGTTAACTTC	0	0	0	0
tsrna-20600	GTAGTGTGGCCGAGCGGTCT	0	0	0	0
tsrna-20599	GTAGTGTAGTGGTTATCACGTTCGCCTC	1	1	0	0
tsrna-20598	GTAGTGTAGTGGTTATCACGTTCGCCT	2	0	0	0
tsrna-20597	GTAGTGTAGTGGTTATCACGTTCGCC	0	1	0	0
tsrna-20596	GTAGTGTAGTGGTTATCACGTTCGC	2	0	1	0
tsrna-20595	GTAGTGTAGTGGTTATCACGTTCG	0	0	0	1
tsrna-20594	GTAGTGTAGTGGTTATCACGTTC	0	0	0	0
tsrna-20592	GTAGTGTAGTGGTTATCACG	0	1	0	0
tsrna-20591	GTAGTGTAGTGGTTATCAC	0	1	0	1
tsrna-20583	GTAGTGTAGTGGTCATC	2	0	0	0
tsrna-20582	GTAGTGTAGTGGTCAT	1	0	0	0
tsrna-20580	GTAGTGTAGCGGTTATCAC	1	0	0	0
tsrna-20574	GTAGTGGTTATCACGTTCGCCTA	1	0	0	0
tsrna-20571	GTAGTGGTTATCACGTTCGC	0	0	0	0
tsrna-20567	GTAGTGGTTATCACGTCTGCTTTAC	3	0	2	0
tsrna-20566	GTAGTGGTTATCACGTCTGCTTTA	1	0	0	0
tsrna-20565	GTAGTGGTTATCACGTCTGCTTT	0	0	0	0
tsrna-20554	GTAGTGGTTATCACATCTGCTT	0	0	0	0
tsrna-20552	GTAGTGGTCATCACGTTCGCC	0	1	0	0
tsrna-20551	GTAGTGGTCATCACGTTCGC	0	1	0	0
tsrna-20550	GTAGTGGTATCATGCAAGATTCCCATTCTT	1	1	3	0
tsrna-20549	GTAGTGGTATCATGCAAGATTCCC	0	0	0	0
tsrna-20548	GTAGTGGTATCATGCAAGATTCC	0	0	0	0
tsrna-20547	GTAGTGGTATCATGCAAGATTC	0	0	0	0
tsrna-20545	GTAGTGGTATCATGCAAGAT	0	0	1	0
tsrna-20542	GTAGTCGTGGCCGAGTGGTTAAGGTGATGG	5	16	5	7
tsrna-20541	GTAGTCGTGGCCGAGTGGTTAAGGTGA	6	24	3	9
tsrna-20540	GTAGTCGTGGCCGAGTGGTTAAGGT	14	20	5	4
tsrna-20539	GTAGTCGTGGCCGAGTGGTTAAGGCGATGG	6	19	2	10
tsrna-20538	GTAGTCGTGGCCGAGTGGTTAAGGCGATG	10	20	9	5

tsrna-20537	GTAGTCGTGGCCGAGTGGTTAAGGCGA	13	14	6	1
tsrna-20536	GTAGTCGTGGCCGAGTGGTTAAGGCG	11	14	3	4
tsrna-20535	GTAGTCGTGGCCGAGTGGTTAAGGC	10	18	9	4
tsrna-20534	GTAGTCGTGGCCGAGTGGTTAAGG	4	17	6	4
tsrna-20533	GTAGTCGTGGCCGAGTGGTTAAG	4	16	6	8
tsrna-20532	GTAGTCGTGGCCGAGTGGTTAA	7	24	3	3
tsrna-20531	GTAGTCGTGGCCGAGTGGTTA	11	16	3	2
tsrna-20530	GTAGTCGTGGCCGAGTGGTT	14	17	7	7
tsrna-20529	GTAGTCGTGGCCGAGTGGT	9	14	5	2
tsrna-20528	GTAGTCGTGGCCGAGTGG	4	18	3	3
tsrna-20527	GTAGTCGTGGCCGAGTG	1	6	1	4
tsrna-20526	GTAGTCGTGGCCGAGT	3	7	2	2
tsrna-20525	GTAGTCCGTGCGAGAATACCA	1	3	0	0
tsrna-20523	GTAGTCCGTGCGAGAATAC	0	0	0	0
tsrna-20521	GTAGTCCGTGCGAGAAT	0	0	0	0
tsrna-20519	GTAGTATAAACTAATACACCAGT	0	0	1	0
tsrna-20517	GTAGTATAAACTAATACACCA	0	0	1	0
tsrna-20514	GTAGCTTACCTCCTCAAAGCAATA	0	0	0	0
tsrna-20508	GTAGCTCAGTGGTAGAGCGCGTGC	12	26	3	0
tsrna-20507	GTAGCTCAGTGGTAGAGCGCATGCTT	0	0	0	0
tsrna-20505	GTAGCTCAGTGGTAGAGCGCATGC	0	0	0	0
tsrna-20500	GTAGCTCAGTGGTAGAGCATTTGACTGC	1	2	1	2
tsrna-20499	GTAGCTCAGTGGTAGAGCATTTGACTG	3	0	0	0
tsrna-20498	GTAGCTCAGTGGTAGAGCATTTGACT	0	2	1	0
tsrna-20497	GTAGCTCAGTGGTAGAGCATTTGAC	0	0	0	0
tsrna-20490	GTAGCGTGGCCGAGTGGTCTAAGGCGCT	0	0	0	0
tsrna-20489	GTAGCGTGGCCGAGTGGTCTAAGGC	0	0	0	1
tsrna-20488	GTAGCGTGGCCGAGTGGTCTAAGG	1	0	0	0
tsrna-20487	GTAGCGTGGCCGAGTGGTCTA	2	0	0	0
tsrna-20483	GTAGCGTGGCCGAGCGGTCTAAGGCGCTG	0	1	0	0
tsrna-20482	GTAGCGTGGCCGAGCGGTCTAAGGCGCT	0	3	0	0
tsrna-20481	GTAGCGTGGCCGAGCGGTCTAAGGC	0	1	0	0
tsrna-20479	GTAGCGTGGCCGAGCGGTCTAAG	0	0	1	0
tsrna-20477	GTAGCGTGGCCGAGCGGTCTA	0	0	0	0
tsrna-20476	GTAGCGTGGCCGAGCGGTCT	0	1	0	0
tsrna-20475	GTAGCGTGGCCGAGCGGTC	0	0	1	0
tsrna-20474	GTAGCGTGGCCGAGCGGT	0	1	0	0
tsrna-20473	GTAGCGTGGCCGAGCGG	0	0	0	1
tsrna-20472	GTAGCGTGGCCGAGCG	1	0	0	1
tsrna-20471	GTAGCGGTTATCACGTCTGCTTT	0	0	0	0
tsrna-20463	GTAGATTGAAGCCAGTTGATTAGGGTGCTT	2	1	2	1
tsrna-20462	GTAGATTGAAGCCAGTTGATT	2	1	0	0
tsrna-20455	GTAGAGCATTTGACTGC	0	1	0	0
tsrna-20454	GTAGAGCATTTGACTG	0	0	0	0
tsrna-20453	GTAGAGCATGGGACTCTTAATCCCAGGGTCGTGGGT	3	7	2	2
tsrna-20452	GTAGAGCATGGGACTCTTAATCC	1	3	0	0
tsrna-20450	GTAGAGCATGAGACTCTTAATCTCAGGGTCGTGGGT	10	14	4	7
tsrna-20449	GTAGAGCATGAGACTC	0	0	0	0
tsrna-20448	GTAGAGCATCAGACTTTTAATCTGAGGGTCCAGGGT	13	45	7	4
tsrna-20447	GTAGAGCATCAGACTTTTAATC	1	0	0	0
tsrna-20442	GTAGAATTCTCGCCTGCCACGCGGGAGGCCCGGGTTT	53	86	38	21
tsrna-20441	GTAGAATTCTCGCCTGCCACGCGGGAGGCCCGGGTTC	46	74	51	17
tsrna-20440	GTAGAATTCTCGCCTGCCACGCGGGAGGCCCGGGTT	52	89	28	11
tsrna-20439	GTAGAATTCTCGCCTGCCACGCGGGAGGCCCGGGT	57	56	22	14
tsrna-20438	GTAGAATTCTCGCCTGCCACGCGGGAGGCCCGGG	49	47	29	16
tsrna-20437	GTAGAATTCTCGCCTGCCACGCGGGAGGCCCGG	40	44	30	11
tsrna-20436	GTAGAATTCTCGCCTGCCACGCGGGAGGCCCG	42	43	27	15
tsrna-20435	GTAGAATTCTCGCCTGCCACGCGGGAGGCCC	50	34	18	12
tsrna-20434	GTAGAATTCTCGCCTGCCACGCGGGAGGCC	36	41	20	17
tsrna-20433	GTAGAATTCTCGCCTGCCACGCGGGAGGC	42	46	21	16
tsrna-20432	GTAGAATTCTCGCCTGCCACGCGGGAGG	45	32	23	11
tsrna-20431	GTAGAATTCTCGCCTGCCACGCGGGAG	27	46	28	22
tsrna-20430	GTAGAATTCTCGCCTGCCACGCGGGA	40	48	22	16
tsrna-20429	GTAGAATTCTCGCCTGCCACGCGGG	33	32	22	11

tsrna-20428	GTAGAATTCTCGCCTGCCACGCGG	37	44	13	13
tsrna-20427	GTAGAATTCTCGCCTGCCACGCG	39	39	25	20
tsrna-20426	GTAGAATTCTCGCCTGCCACGC	37	25	16	17
tsrna-20425	GTAGAATTCTCGCCTGCCACG	30	32	10	14
tsrna-20424	GTAGAATTCTCGCCTGCCAC	7	2	5	4
tsrna-20423	GTAGAATTCTCGCCTGCCA	3	3	0	1
tsrna-20422	GTAGAATTCTCGCCTGCC	2	0	0	1
tsrna-20421	GTAGAATTCTCGCCTGC	2	1	1	1
tsrna-20420	GTAGAATTCTCGCCTG	2	0	0	0
tsrna-20419	GTAGAATTCTCGCCTCCCACGCGGGAGACCCGGGTT	2	2	0	1
tsrna-20418	GTAGAATTCTCGCCTCCCACGCGGGAGACCCGGGT	2	3	0	2
tsrna-20417	GTAGAATTCTCGCCTCCCACG	3	1	0	2
tsrna-20416	GTAGAATTCTCGCCTCCC	1	0	1	0
tsrna-20415	GTAGAATTCTCGCCTCC	2	0	1	1
tsrna-20414	GTAGAATTCTCGCCTC	1	0	0	0
tsrna-20413	GTACTTATAATGCCGAGGTTGTG	2	10	1	1
tsrna-20411	GTACTCTGCGTTGTGGCCGCA	1	0	0	0
tsrna-20405	GTACGAGGCCCCGGGTTCG	0	4	0	0
tsrna-20403	GTACCGTTAACTTCCAATTAACTAGTTTTG	0	0	1	0
tsrna-20399	GTACCGTTAACTTCCAATTA	0	0	1	0
tsrna-20395	GTAATGGTTAGCACTCTGGGCTT	0	0	0	0
tsrna-20393	GTAATGGTTAGCACTCTGGGC	0	0	0	0
tsrna-20392	GTAATGGTTAGCACTCTGGACTTTGAATCC	6	5	3	1
tsrna-20391	GTAATGGTTAGCACTCTGGACTTTGAATC	1	4	2	0
tsrna-20390	GTAATGGTTAGCACTCTGGACTTTGAAT	0	1	1	0
tsrna-20389	GTAATGGTTAGCACTCTGGACTTTGAA	0	0	2	0
tsrna-20388	GTAATGGTTAGCACTCTGGACTTTGA	1	1	0	0
tsrna-20387	GTAATGGTTAGCACTCTGGACTTTG	0	0	1	0
tsrna-20386	GTAATGGTTAGCACTCTGGACTTT	0	0	0	1
tsrna-20385	GTAATGGTTAGCACTCTGGACTT	2	1	0	0
tsrna-20384	GTAATGGTTAGCACTCTGGACTCTGAATCCAGCGAT	5	12	4	2
tsrna-20383	GTAATGGTTAGCACTCTGGACTCTGAATCC	5	3	1	0
tsrna-20382	GTAATGGTTAGCACTCTGGACTCTGAATC	0	0	0	1
tsrna-20381	GTAATGGTTAGCACTCTGGACTCTGAAT	0	1	0	0
tsrna-20380	GTAATGGTTAGCACTCTGGACTCTGAA	2	0	0	0
tsrna-20379	GTAATGGTTAGCACTCTGGACTCTGA	0	0	0	0
tsrna-20378	GTAATGGTTAGCACTCTGGACTCTG	0	0	0	0
tsrna-20377	GTAATGGTTAGCACTCTGGACTCT	1	0	0	0
tsrna-20376	GTAATGGTTAGCACTCTGGACTC	0	1	0	0
tsrna-20375	GTAATGGTTAGCACTCTGGACT	0	0	0	0
tsrna-20374	GTAATGGTTAGCACTCTGGAC	1	1	1	0
tsrna-20369	GTAATGGTTAGCACTC	0	0	0	0
tsrna-20368	GTAATGGTGAGCACTTTGGACTCTGA	0	4	0	0
tsrna-20367	GTAATGGTGAGCACTCTGGACTCTG	0	0	0	0
tsrna-20364	GTAATGGTGAGCACTCTGGACT	0	1	0	0
tsrna-20363	GTAATGGTGAGCACTCTGGAC	0	1	0	1
tsrna-20352	GTAATGGTAAGCACTCTGGACTCTGAATC	0	0	1	0
tsrna-20351	GTAATGGTAAGCACTCTGGACTCTG	0	0	0	1
tsrna-20346	GTAATGGTAAGCACTCTGG	0	1	0	0
tsrna-20345	GTAATGGTAAGCACTCTG	0	0	0	0
tsrna-20344	GTAATCGCATAAAACTTAAAACTTTACAGT	4	0	1	1
tsrna-20343	GTAATCGCATAAAACTTAAAACTTTACAG	2	3	3	0
tsrna-20342	GTAATCGCATAAAACTTAAAACTTTACA	2	0	5	1
tsrna-20341	GTAATCGCATAAAACTTAAAACTT	0	1	0	0
tsrna-20337	GTAATCGCATAAAACTTA	0	1	0	0
tsrna-20336	GTAAGTTGCAATACTTAATTTCTGCCA	2	0	1	0
tsrna-20335	GTAAGTTGCAATACTTAATTTCTGCC	0	0	0	0
tsrna-20330	GTAAGGTCAGCTAAATAAGCTATCGGGCCC	1	0	1	0
tsrna-20325	GTAAGGTCAGCTAAATAAGCT	0	0	0	0
tsrna-20324	GTAAGGTCAGCTAAATAAGC	0	0	1	0
tsrna-20319	GTAAGCACTCTGGACTCTGAATCC	0	1	1	0
tsrna-20318	GTAAGCACTCTGGACTCTGAATC	0	1	0	0
tsrna-20312	GTAAATCTAAAGACAGGGGTTAGGCCT	0	1	0	0
tsrna-20311	GTAAATCTAAAGACAGGGGTTAGGC	1	0	0	0

tsrna-20307	GTAAATATAGTTTAACCAAAACATCAGAT	0	0	2	0
tsrna-20305	GTAAATAATAGGAGCTTAAACCCCCTTATT	2	0	1	0
tsrna-20304	GTAAATAATAGGAGCTTAAACCCCCTTAT	0	0	0	1
tsrna-20303	GTAAATAATAGGAGCTTAAACCCCCTTA	2	0	0	0
tsrna-20302	GTAAATAATAGGAGCTTAAACCCCCT	0	0	0	0
tsrna-20291	GTAAACCGGAGATGAAAACCTTTTTCCAAGGACACCA	43	42	115	3
tsrna-20290	GTAAACCGGAGATGAAAACCTTTTTCCAAGGACACC	11	21	9	0
tsrna-20289	GTAAACCGGAGATGAAAACCTTTTTCCAAG	2	10	1	0
tsrna-20288	GTAAACCGGAGATGAAAACCTT	0	1	0	0
tsrna-20286	GTAAACCGGAGATGAAAACC	0	1	0	0
tsrna-20285	GTAAACCGGAGATGAAAAC	1	0	0	0
tsrna-20284	GTAAACCGAAGATCGCGGGTTCGAACCCCGTCCGTGCCTCCA	14	41	1	3
tsrna-20283	GTAAACCGAAGATCGCGGGT	2	22	1	2
tsrna-20279	GTAAACCGAAGATCACGGGTTCGAACCCCGTCCGTGCCTCCA	4	18	1	3
tsrna-20278	GTAAACCGAAGATCACGGGTTC	0	3	1	0
tsrna-20276	GTAAACCAGGGGTCGCGAGTTCA	11	28	4	6
tsrna-20275	GTAAACCAGGGGTCGCGAGTTC	12	26	8	5
tsrna-20274	GTAAACCAGGGGTCGCGAGTT	7	26	5	6
tsrna-20273	GTAAACCAGGGGTCGCGAGT	11	12	9	3
tsrna-20272	GTAAACCAGGGGTCGCGAG	0	1	0	0
tsrna-20271	GTAAACCAGGGGTCGCGA	2	3	0	0
tsrna-20270	GTAAACCAGGGGTCGCG	0	2	0	0
tsrna-20268	GTAAACAGGAGATCCTGGGT	12	18	3	2
tsrna-20267	GTAAAATGGCTGAGTGAAGCATTGGACTGT	1	1	0	0
tsrna-20266	GTAAAATGGCTGAGTGAAGCATTGGACTG	0	0	1	0
tsrna-20265	GTAAAATGGCTGAGTGAAGCATTGGACT	1	2	0	0
tsrna-20264	GTAAAATGGCTGAGTGAAGCATTGGAC	0	0	0	0
tsrna-20263	GTAAAATGGCTGAGTGAAGCATTGG	0	0	1	0
tsrna-20262	GTAAAATGGCTGAGTGAAGCATTG	0	1	1	0
tsrna-20261	GTAAAATGGCTGAGTGAAGCATT	1	0	1	0
tsrna-20260	GTAAAATGGCTGAGTGAAGCA	1	2	0	0
tsrna-20259	GTAAAATGGCTGAGTGAAGC	2	1	0	0
tsrna-20258	GTAAAATGGCTGAGTGAAG	1	0	0	0
tsrna-20257	GTAAAATGGCTGAGTGAA	1	0	0	0
tsrna-20256	GGTTTTTCATATCATTGGTCGTGGTTGTAGTCCGTGCGAGAAT	156	153	213	57
tsrna-20255	GGTTTTTCATATCATTGGTCGTGGTTGTAGTCCGTGCGAGAAT	22	34	37	18
tsrna-20254	GGTTTTTCATATCATTGGTCGTGGTTGTAGTCCGTGCGAGAAT	14	20	15	11
tsrna-20253	GGTTTTTCATATCATTGGTCGTGGTTGTAGTCCGTGCGAGAAT	11	16	13	14
tsrna-20252	GGTTTTTCATATCATTGGTCGTGGTTGTAGTCCGTGCGAGAA	8	19	24	10
tsrna-20251	GGTTTTTCATATCATTGGTCGTGGTTGTAGTCCGTGCGAGA	7	17	16	10
tsrna-20250	GGTTTTTCATATCATTGGTCGTGGTTGTAGTCCGTGCGAG	11	8	12	20
tsrna-20249	GGTTTTTCATATCATTGGTCGTGGTTGTAGTCCGTGCGA	6	11	14	4
tsrna-20248	GGTTTTTCATATCATTGGTCGTGGTTGTAGTCCGTGCG	4	5	10	7
tsrna-20247	GGTTTTTCATATCATTGGTCGTGGTTGTAGTCCGTGC	8	3	16	0
tsrna-20246	GGTTTTTCATATCATTGGTCGTGGTTGTAGTCCGTG	1	6	2	4
tsrna-20245	GGTTTTTCATATCATTGGTCGTGGTTGTAGTCC	1	3	1	0
tsrna-20244	GGTTTTTCATATCATTGGTCGTGGTTGTAGTC	1	1	0	0
tsrna-20243	GGTTTTTCATATCATTGGTCGTGGTTGTAG	3	0	1	1
tsrna-20242	GGTTTTTCATATCATTGGTCGTGGTTGTA	0	1	1	0
tsrna-20241	GGTTTTTCATATCATTGGTCGTGGTTGT	0	0	0	0
tsrna-20240	GGTTTTTCATATCATTGGTCGTGGTTG	0	1	0	0
tsrna-20239	GGTTTTTCATATCATTGGTCGTGGTT	0	0	0	0
tsrna-20238	GGTTTTTCATATCATTGGTCGTGGT	0	0	0	0
tsrna-20237	GGTTTTTCATATCATTGGTCGTGG	0	0	0	0
tsrna-20236	GGTTTTTCATATCATTGGTCGTG	0	0	0	1
tsrna-20235	GGTTTTTCATATCATTGGTCGT	0	1	0	0
tsrna-20234	GGTTTTTCATATCATTGGTCG	0	1	0	0
tsrna-20228	GGTTTTGCAGTCCTTACCA	1	0	0	0
tsrna-20226	GGTTTTGCAGTCCTTAC	0	0	1	0
tsrna-20224	GGTTTTCACCCAGGTGGCCCGG	3	5	4	0
tsrna-20223	GGTTTTCACCCAGGTGGCCCG	4	1	2	1
tsrna-20222	GGTTTTCACCCAGGTGGCCC	3	0	4	0
tsrna-20221	GGTTTTCACCCAGGCGGCCCGGGT	3	6	0	0
tsrna-20220	GGTTTTCACCCAGGCGGCCCGG	2	0	1	0

tsrna-20219	GGTTTTCACCCAGGCGGCCCG	1	2	0	0
tsrna-20218	GGTTTTCACCCAGGCGGCCC	2	4	0	0
tsrna-20217	GGTTTTCACCCAGGCGGCC	1	0	0	0
tsrna-20212	GGTTTCCGTAGTGTAGTGGTTATCACGTTCGCCT	398	148	427	115
tsrna-20211	GGTTTCCGTAGTGTAGTGGTTATCACGTTCGCC	369	168	378	99
tsrna-20210	GGTTTCCGTAGTGTAGTGGTTATCACGTTC	99	45	67	73
tsrna-20209	GGTTTCCGTAGTGTAGTGGTTATC	97	33	59	67
tsrna-20208	GGTTTCCGTAGTGTAGTGGTTAT	21	13	6	8
tsrna-20207	GGTTTCCGTAGTGTAGTGGTT	16	16	2	7
tsrna-20206	GGTTTCCGTAGTGTAGTGGTC	16	13	7	4
tsrna-20205	GGTTTCCGTAGTGTAGTGGT	14	15	7	7
tsrna-20204	GGTTTCCGTAGTGTAGTGG	16	13	3	0
tsrna-20203	GGTTTCCGTAGTGTAGCGGTT	8	10	7	7
tsrna-20202	GGTTTCCGTAGTGTAG	7	15	3	0
tsrna-20200	GGTTTCCCCGCGCAGGTTC	0	0	0	0
tsrna-20198	GGTTTCCCCGCGCAGGT	0	1	0	0
tsrna-20196	GGTTTCCCCGCACAGGTT	0	1	0	0
tsrna-20194	GGTTTAGTGGTAGAATTCTCGCCT	7	5	4	0
tsrna-20193	GGTTTAGTGGTAGAATTCTCGCC	0	5	0	2
tsrna-20192	GGTTTAGTGGTAGAATTCTCGC	0	0	0	0
tsrna-20191	GGTTTAGTGGTAGAATTCTCG	1	1	0	0
tsrna-20187	GGTTTAAGTCCCATTGGTCTAGCCA	0	2	0	0
tsrna-20185	GGTTTAAGTCCCATTGGTCTAGC	0	0	3	0
tsrna-20183	GGTTTAAGTCCCATTGGTCTA	0	0	0	0
tsrna-20182	GGTTTAAGTCCCATTGGTCT	0	0	0	0
tsrna-20181	GGTTTAAGTCCCATTGGTC	1	0	0	0
tsrna-20180	GGTTTAAGTCCCATTGGT	0	1	0	0
tsrna-20179	GGTTTAAGTCCCATTGG	0	0	0	0
tsrna-20174	GGTTGTGGGTTCGAATCCCACCAGAGTCGC	0	0	0	0
tsrna-20172	GGTTGTAGTCCGTGCGAGAATACCA	2	2	1	1
tsrna-20171	GGTTGTAGTCCGTGCGAGAATACC	2	4	0	0
tsrna-20170	GGTTGTAGTCCGTGCGAGAATAC	0	0	0	0
tsrna-20169	GGTTGTAGTCCGTGCGAGAATA	1	1	1	0
tsrna-20168	GGTTGTAGTCCGTGCGAGAAT	1	0	0	0
tsrna-20167	GGTTGTAGTCCGTGCGAGAA	1	0	0	0
tsrna-20166	GGTTGTAGTCCGTGCGAGA	0	1	1	0
tsrna-20165	GGTTGTAGTCCGTGCGAG	0	0	0	0
tsrna-20160	GGTTGGTGGTTCGATCCCACCCAGGGACGC	0	3	0	0
tsrna-20159	GGTTGGTGGTTCGAGCCCACCCAGGGACGC	1	2	0	0
tsrna-20158	GGTTGGTGGTTCGAGCCCACCCAGGGACG	0	2	0	0
tsrna-20157	GGTTGCGTGTTCAAGTCACGTCGGGGTCAC	0	0	0	0
tsrna-20153	GGTTGCGTGTTCAAATCACGTCGGGGTCAC	0	0	0	0
tsrna-20151	GGTTCTTGTAGTTGAAAT	0	0	0	0
tsrna-20150	GGTTCGATTCTCATAGTCCTAGCCA	1	5	1	0
tsrna-20149	GGTTCGATTCTCATAGTCCTAGCC	0	3	0	1
tsrna-20148	GGTTCGATTCTCATAGTCCTAGC	0	1	0	1
tsrna-20147	GGTTCGATTCTCATAGTCCTAG	0	1	0	0
tsrna-20146	GGTTCGATTCTCATAGTCCTA	1	0	0	1
tsrna-20145	GGTTCGATTCTCATAGTCCT	1	1	0	1
tsrna-20144	GGTTCGATTCTCATAGTCC	0	1	0	0
tsrna-20140	GGTTCGATTCCTTCCTTTTTTGCCA	0	1	1	0
tsrna-20139	GGTTCGATTCCTTCCTTTTTTGCC	0	0	0	0
tsrna-20138	GGTTCGATTCCTTCCTTTTTTGC	0	1	0	2
tsrna-20137	GGTTCGATTCCTTCCTTTTTTG	0	2	1	0
tsrna-20136	GGTTCGATTCCTTCCTTTTTT	0	1	0	0
tsrna-20135	GGTTCGATTCCTTCCTTTTT	0	0	0	0
tsrna-20134	GGTTCGATTCCTTCCTTTT	0	0	0	0
tsrna-20132	GGTTCGATTCCTTCCTT	0	1	0	0
tsrna-20130	GGTTCGATTCCGGCTCGAAGGACCA	72	154	25	25
tsrna-20121	GGTTCGATTCCCGGTCAGGGAACCA	10	286	1	3
tsrna-20120	GGTTCGATTCCCGGTCAGGGAACC	0	11	1	0
tsrna-20119	GGTTCGATTCCCGGTCAGGGAAC	0	3	0	0
tsrna-20118	GGTTCGATTCCCGGTCAGGGAA	0	3	0	0
tsrna-20117	GGTTCGATTCCCGGTCAGGGA	0	3	0	0

tsrna-20116	GGTTCGATTCCCGGTCAGGG	0	1	0	0
tsrna-20111	GGTTCGATTCCCGGGCGGCGCACCA	9	61	5	1
tsrna-20110	GGTTCGATTCCCGGGCGGCGCACC	5	8	0	0
tsrna-20101	GGTTCGATTCCCGGCCCATGCACCA	18	29	11	9
tsrna-20100	GGTTCGATTCCCGGCCCATGCACC	4	2	1	1
tsrna-20099	GGTTCGATTCCCGGCCCATGCAC	0	1	0	0
tsrna-20096	GGTTCGATTCCCGGCCAGGGAACCA	0	6	0	0
tsrna-20095	GGTTCGATTCCCGGCCAGGGAACC	0	1	0	0
tsrna-20090	GGTTCGATTCCCGGCCAATGCACCA	41	126	8	13
tsrna-20089	GGTTCGATTCCCGGCCAATGCACC	11	8	0	1
tsrna-20086	GGTTCGATTCCCGGCCAATGC	0	1	0	0
tsrna-20085	GGTTCGATTCCCGGCCAACGCACCA	47	107	19	11
tsrna-20084	GGTTCGATTCCCGGCCAACGCACC	8	11	3	2
tsrna-20083	GGTTCGATTCCCGGCCAACGCAC	0	2	0	0
tsrna-20082	GGTTCGATTCCCGGCCAACGCA	0	1	0	0
tsrna-20081	GGTTCGATTCCCGGCCAACGC	0	0	0	0
tsrna-20078	GGTTCGATTCCCCGACGGGGAGCCA	2	21	2	0
tsrna-20077	GGTTCGATTCCCCGACGGGGAGCC	0	1	0	0
tsrna-20075	GGTTCGATTCCCCGACGGGGAG	0	0	0	0
tsrna-20060	GGTTCGATCCCGGGTTTCGGCACCA	32	115	7	12
tsrna-20059	GGTTCGATCCCGGGTTTCGGCAC	0	0	1	0
tsrna-20056	GGTTCGATCCCGGGTTTCGG	0	0	0	0
tsrna-20053	GGTTCGATCCCGGGCGGA	0	1	0	0
tsrna-20051	GGTTCGATCCCCGTACTGGCCACCA	6	12	3	1
tsrna-20050	GGTTCGATCCCCGTACGGGCCACCA	5	61	3	4
tsrna-20044	GGTTCGATCCCCGGCATCTCCACCA	246	397	119	88
tsrna-20043	GGTTCGATCCCCGGCATCTCCACC	12	19	4	1
tsrna-20042	GGTTCGATCCCCGGCATCTCCAC	0	1	0	1
tsrna-20041	GGTTCGATCCCCGGCATCTCCA	1	0	0	0
tsrna-20040	GGTTCGATCCCCGGCATCTCC	0	1	0	0
tsrna-20039	GGTTCGATCCCCGGCATCTC	0	0	0	0
tsrna-20035	GGTTCGATCCCCGGCACCTCCACCA	180	266	109	48
tsrna-20034	GGTTCGATCCCCGGCACCTCCACC	6	21	2	2
tsrna-20033	GGTTCGATCCCCGGCACCTCCAC	1	0	0	0
tsrna-20031	GGTTCGATCCCCGGCACCTCC	0	0	1	0
tsrna-20029	GGTTCGATCCCCGACACCTCCACCA	20	25	0	5
tsrna-20028	GGTTCGATCCCCAGTACCTCCACCA	22	63	13	8
tsrna-20027	GGTTCGATCCCCAGTACCTCCACC	8	5	1	0
tsrna-20019	GGTTCGATCCCCAGCATCTCCACCA	11	13	7	1
tsrna-20018	GGTTCGATCCCCAGCATCTCCACC	0	1	0	0
tsrna-20014	GGTTCGATCCCCAGCATCTC	0	1	0	0
tsrna-20009	GGTTCGAGTCCTGCCGCGGTCGCCA	2	5	1	0
tsrna-20007	GGTTCGAGTCCTGCCGCGGTCGC	0	0	0	0
tsrna-20002	GGTTCGAGTCCCTTCGTGGTCGCCA	1	2	0	0
tsrna-20001	GGTTCGAGTCCCTTCGTGGTCGCC	0	1	0	0
tsrna-19996	GGTTCGAGTCCCGGCGGAGTCGCCA	0	5	0	0
tsrna-19988	GGTTCGAGTCCCATCTGGGTCGCCA	1	4	0	1
tsrna-19987	GGTTCGAGTCCCATCTGGGTCGCC	0	2	0	1
tsrna-19984	GGTTCGAGTCCCATCTGGGGTGCCA	0	9	0	0
tsrna-19976	GGTTCGAGTCCCACCAGAGTCGCCA	2	2	0	0
tsrna-19975	GGTTCGAGTCCCACCAGAGTCGCC	0	1	0	0
tsrna-19973	GGTTCGAGTCCCACCAGAGTCG	0	1	0	0
tsrna-19969	GGTTCGAGCCCCAGTGGAACCACCA	44	29	25	18
tsrna-19968	GGTTCGAGCCCCAGTGGAACCAC	2	0	1	0
tsrna-19967	GGTTCGAGCCCCAGTGGAACC	1	0	0	0
tsrna-19961	GGTTCGAGCCCCACGTTGGGCGCCA	14	214	4	3
tsrna-19960	GGTTCGAGCCCCACGTTGGGCGCC	2	7	0	0
tsrna-19952	GGTTCGAGCCCACCCAGGGACGCCA	7	97	0	2
tsrna-19951	GGTTCGAGCCCACCCAGGGACGCC	0	7	0	0
tsrna-19950	GGTTCGAGCCCACCCAGGGACGC	0	1	0	0
tsrna-19943	GGTTCGACTCCTGGCTGGCTCGCCA	43	111	10	5
tsrna-19942	GGTTCGACTCCTGGCTGGCTCGCC	1	6	0	1
tsrna-19941	GGTTCGACTCCTGGCTGGCTCGC	0	0	0	0
tsrna-19939	GGTTCGACTCCTGGCTGGCTC	0	0	0	0

tsrna-19935	GGTTCGACTCCCGGTGTGGGAACCA	72	269	23	14
tsrna-19934	GGTTCGACTCCCGGTGTGGGAACC	1	22	0	2
tsrna-19933	GGTTCGACTCCCGGTGTGGGAAC	0	2	0	0
tsrna-19932	GGTTCGACTCCCGGTGTGGGAA	0	3	0	0
tsrna-19930	GGTTCGACTCCCGGTGTGGG	0	1	0	0
tsrna-19929	GGTTCGACTCCCGGTGTGG	0	1	0	0
tsrna-19925	GGTTCGACTCCCGGTATGGGAACCA	11	55	1	2
tsrna-19924	GGTTCGACTCCCGGTATGGGAACC	4	8	1	0
tsrna-19923	GGTTCGACTCCCGGTATGGGAAC	1	2	0	0
tsrna-19922	GGTTCGACTCCCGGTATGGGAA	1	2	0	0
tsrna-19920	GGTTCGACTCCCGGTATGGG	0	1	0	0
tsrna-19915	GGTTCGACTCCCAGCGGGGCCTCC	0	0	1	0
tsrna-19914	GGTTCGACTCCCAGCGGGGC	0	0	0	0
tsrna-19913	GGTTCGACCCCCGGCTCCTCCACCA	12	24	9	5
tsrna-19910	GGTTCGAATCCTGTTCGTGACGCC	2	0	0	2
tsrna-19909	GGTTCGAATCCTGTTCGTGACGC	0	0	0	0
tsrna-19908	GGTTCGAATCCTGTCG	0	1	0	0
tsrna-19907	GGTTCGAATCCTGCTCACAGCGCCA	13	33	4	0
tsrna-19906	GGTTCGAATCCTGCTCACAGCGCC	1	2	1	0
tsrna-19904	GGTTCGAATCCTGCCGACTACGCCA	90	244	26	26
tsrna-19903	GGTTCGAATCCTGCCGACTACGCC	5	14	3	2
tsrna-19902	GGTTCGAATCCTGCCGACTACGC	0	0	0	0
tsrna-19899	GGTTCGAATCCTGCCGAC	0	0	0	0
tsrna-19889	GGTTCGAATCCGAGTCACGGCACCA	1	1	0	0
tsrna-19883	GGTTCGAATCCCTTCGTGGTTGCCA	0	2	0	0
tsrna-19880	GGTTCGAATCCCATCCTCGTCGCCA	4	12	4	3
tsrna-19879	GGTTCGAATCCCATCCTCGTCGCC	1	2	0	0
tsrna-19872	GGTTCGAATCCCAGCGGTGCCTCCA	24	63	11	6
tsrna-19871	GGTTCGAATCCCAGCGGTGCCTCC	1	2	1	0
tsrna-19865	GGTTCGAATCCCAGCGGGGCCTCCA	1	9	0	0
tsrna-19864	GGTTCGAATCCCAGCGGGGCCTCC	1	1	0	0
tsrna-19857	GGTTCGAATCCCACTTCTGACACCA	10	27	8	8
tsrna-19856	GGTTCGAATCCCACTTCTGACACC	2	4	1	0
tsrna-19855	GGTTCGAATCCCACTTCTGACAC	1	0	1	0
tsrna-19854	GGTTCGAATCCCACTTCTGACA	0	0	0	0
tsrna-19851	GGTTCGAATCCCACTCCTGACACCA	105	118	57	30
tsrna-19850	GGTTCGAATCCCACTCCTGACACC	5	14	2	0
tsrna-19849	GGTTCGAATCCCACTCCTGACAC	1	0	0	0
tsrna-19848	GGTTCGAATCCCACTCCTGACA	0	0	1	0
tsrna-19844	GGTTCGAATCCCACCTTCGTCGCCA	0	6	1	1
tsrna-19840	GGTTCGAATCCCACCGCTGCCACCA	19	27	2	2
tsrna-19839	GGTTCGAATCCCACCGCTGCCACC	1	3	1	0
tsrna-19838	GGTTCGAATCCCACCGCTGCCAC	0	1	0	0
tsrna-19832	GGTTCGAATCCCACCCTCGTCGCCA	3	17	4	5
tsrna-19831	GGTTCGAATCCCACCAGAGTCGCCA	2	3	0	0
tsrna-19830	GGTTCGAATCCCACCAGAGTCGCC	1	2	0	0
tsrna-19824	GGTTCGAACCCTGCTCGCTGCGCCA	11	26	1	1
tsrna-19823	GGTTCGAACCCTGCTCGCTGCGCC	0	2	0	0
tsrna-19817	GGTTCGAACCCCGTCCGTGCCTCCA	3	11	2	0
tsrna-19813	GGTTCGAACCCCGTCCGTGCC	0	0	0	0
tsrna-19810	GGTTCGAACCCCACTCCTGGTACCA	1	0	0	0
tsrna-19805	GGTTCGAAACTGGGCGGAAACACC	0	10	2	1
tsrna-19804	GGTTCGAAACCGGGCGGAAACACCA	261	721	119	44
tsrna-19803	GGTTCGAAACCGGGCGGAAACACC	78	185	29	6
tsrna-19802	GGTTCGAAACCGGGCGGAAACAC	1	3	0	0
tsrna-19801	GGTTCGAAACCGGGCGGAAACA	0	2	0	0
tsrna-19797	GGTTCGAAACCGGGCGGA	0	1	0	0
tsrna-19794	GGTTCGAAACCGGGCAGAAGCACCA	33	132	9	5
tsrna-19793	GGTTCGAAACCGGGCAGAAGCA	0	1	0	0
tsrna-19789	GGTTCCGGGTTCGAGTCCCGGCAGAGATGC	0	0	1	0
tsrna-19786	GGTTCCATGGTGTAATGGTTAGCACTCTGGACTCTGAATCCA(	10	15	9	8
tsrna-19785	GGTTCCATGGTGTAATGGTTAGCACTCTGGACTCTGAATCCA(	17	16	5	5
tsrna-19784	GGTTCCATGGTGTAATGGTTAGCACTCTGGACTCTGAATCCA(	13	15	6	10
tsrna-19783	GGTTCCATGGTGTAATGGTTAGCACTCTGGACTCTGAATCCA(	15	16	8	3

tsrna-19782	GGTTCCATGGTGTAATGGTTAGCACTCTGGACTCTGAATCCA	9	16	1	11
tsrna-19781	GGTTCCATGGTGTAATGGTTAGCACTCTGGACTCTGAATCC	6	12	1	7
tsrna-19780	GGTTCCATGGTGTAATGGTTAGCACTCTGGACTCTGAATC	3	3	1	1
tsrna-19779	GGTTCCATGGTGTAATGGTTAGCACTCTGGACTCTG	2	3	2	1
tsrna-19778	GGTTCCATGGTGTAATGGTTAGCACTCTGGACTCT	0	4	0	0
tsrna-19777	GGTTCCATGGTGTAATGGTTAGCACTCTGGACTC	0	4	0	4
tsrna-19776	GGTTCCATGGTGTAATGGTTAGCACTCTGGACT	2	8	1	0
tsrna-19775	GGTTCCATGGTGTAATGGTTAGCACTCTGGAC	2	7	0	0
tsrna-19774	GGTTCCATGGTGTAATGGTTAGCACTCTGGA	0	7	2	1
tsrna-19773	GGTTCCATGGTGTAATGGTTAGCACTCTGG	2	4	0	0
tsrna-19772	GGTTCCATGGTGTAATGGTTAGCACTCTG	0	4	0	0
tsrna-19771	GGTTCCATGGTGTAATGGTTAGCACTCT	0	1	1	1
tsrna-19770	GGTTCCATGGTGTAATGGTTAGCACTC	0	4	0	1
tsrna-19769	GGTTCCATGGTGTAATGGTTAGCACT	0	2	0	0
tsrna-19768	GGTTCCATGGTGTAATGGTTAGCAC	0	1	0	0
tsrna-19767	GGTTCCATGGTGTAATGGTTAGCA	0	5	0	0
tsrna-19766	GGTTCCATGGTGTAATGGTTAGC	0	3	0	0
tsrna-19765	GGTTCCATGGTGTAATGGTTAG	0	4	2	1
tsrna-19764	GGTTCCATGGTGTAATGGTTA	0	2	0	0
tsrna-19763	GGTTCCATGGTGTAATGGTT	0	5	0	0
tsrna-19762	GGTTCCATGGTGTAATGGTGAGCACTCTGGACTCT	0	2	0	0
tsrna-19761	GGTTCCATGGTGTAATGGTGAGCACTCTGGACTC	1	4	0	0
tsrna-19760	GGTTCCATGGTGTAATGGTGAGCACTCTGGACT	1	5	0	1
tsrna-19759	GGTTCCATGGTGTAATGGTGAGCACTCTGGAC	0	2	0	0
tsrna-19758	GGTTCCATGGTGTAATGGTGAGCACTCTGGA	1	6	0	0
tsrna-19757	GGTTCCATGGTGTAATGGTGAGCACTCTGG	2	4	0	0
tsrna-19756	GGTTCCATGGTGTAATGGTGAGCACTCTG	0	8	0	0
tsrna-19755	GGTTCCATGGTGTAATGGTGAGCACTCT	1	4	0	0
tsrna-19754	GGTTCCATGGTGTAATGGTGAGCACTC	0	6	0	0
tsrna-19753	GGTTCCATGGTGTAATGGTGAGCACT	1	0	0	0
tsrna-19752	GGTTCCATGGTGTAATGGTGAGCA	0	1	0	0
tsrna-19751	GGTTCCATGGTGTAATGGTGAGC	1	3	0	0
tsrna-19750	GGTTCCATGGTGTAATGGTGAG	2	2	0	0
tsrna-19749	GGTTCCATGGTGTAATGGTGA	1	4	1	1
tsrna-19748	GGTTCCATGGTGTAATGGTG	0	3	0	0
tsrna-19747	GGTTCCATGGTGTAATGGTAAGCACTCTGG	0	6	0	1
tsrna-19746	GGTTCCATGGTGTAATGGTAAGCACTCTG	1	3	0	1
tsrna-19745	GGTTCCATGGTGTAATGGTAAGC	0	4	0	0
tsrna-19744	GGTTCCATGGTGTAATGGTA	0	3	0	1
tsrna-19743	GGTTCCATGGTGTAATGGT	0	7	0	0
tsrna-19742	GGTTCCATGGTGTAATGG	1	2	0	0
tsrna-19741	GGTTCCATGGTGTAATG	1	4	1	1
tsrna-19740	GGTTCCATGGTGTAAT	0	8	0	0
tsrna-19739	GGTTCCATAGTGTAGTGGTTATCACGTCTGCTTTACACGCAG/	51	22	73	5
tsrna-19738	GGTTCCATAGTGTAGTGGTTATCACGTCTGCTTTAC	54	24	53	3
tsrna-19737	GGTTCCATAGTGTAGTGGTTATCACGTCTGCTTTA	46	19	42	4
tsrna-19736	GGTTCCATAGTGTAGTGGTTATCACGTCTGCTTT	43	16	41	2
tsrna-19735	GGTTCCATAGTGTAGTGGTTATCACGTCTGCTT	18	13	15	4
tsrna-19734	GGTTCCATAGTGTAGTGGTTATCACGTCTGCT	17	13	19	2
tsrna-19733	GGTTCCATAGTGTAGTGGTTATCACGTCTGC	19	8	17	0
tsrna-19732	GGTTCCATAGTGTAGTGGTTATCACGTCTG	2	4	1	1
tsrna-19731	GGTTCCATAGTGTAGTGGTTATCACGTCT	2	4	2	2
tsrna-19730	GGTTCCATAGTGTAGTGGTTATCACGTC	4	2	3	0
tsrna-19729	GGTTCCATAGTGTAGTGGTTATCACGT	2	1	1	2
tsrna-19728	GGTTCCATAGTGTAGTGGTTATCACG	3	1	3	1
tsrna-19727	GGTTCCATAGTGTAGTGGTTATCACATCTGCTTT	9	3	12	4
tsrna-19726	GGTTCCATAGTGTAGTGGTTATCACATCTGCTT	7	5	1	1
tsrna-19725	GGTTCCATAGTGTAGTGGTTATCACATCTGCT	5	6	2	5
tsrna-19724	GGTTCCATAGTGTAGTGGTTATCACATCTGC	1	0	4	2
tsrna-19723	GGTTCCATAGTGTAGTGGTTATCACATCTG	4	2	0	2
tsrna-19722	GGTTCCATAGTGTAGTGGTTATCACATCT	4	2	1	2
tsrna-19721	GGTTCCATAGTGTAGTGGTTATCACATC	3	1	3	1
tsrna-19720	GGTTCCATAGTGTAGTGGTTATCACAT	4	2	4	0
tsrna-19719	GGTTCCATAGTGTAGTGGTTATCACA	3	3	2	2

tsrna-19718	GGTTCCATAGTGTAGTGGTTATCAC	3	4	1	1
tsrna-19717	GGTTCCATAGTGTAGTGGTTATCA	3	2	3	1
tsrna-19716	GGTTCCATAGTGTAGTGGTTATC	4	1	3	2
tsrna-19715	GGTTCCATAGTGTAGTGGTTAT	3	8	3	0
tsrna-19714	GGTTCCATAGTGTAGTGGTTA	2	5	2	2
tsrna-19713	GGTTCCATAGTGTAGTGGTT	2	5	5	1
tsrna-19712	GGTTCCATAGTGTAGTGGT	3	2	1	0
tsrna-19711	GGTTCCATAGTGTAGTGG	5	5	1	0
tsrna-19710	GGTTCCATAGTGTAGTG	2	2	2	1
tsrna-19709	GGTTCCATAGTGTAGT	0	2	0	1
tsrna-19708	GGTTCCATAGTGTAGCGGTTATCACGTCTGCTTTACACGCAG[illegible]	20	5	16	5
tsrna-19707	GGTTCCATAGTGTAGCGGTTATCACGTCTGCTTTAC	22	9	25	3
tsrna-19706	GGTTCCATAGTGTAGCGGTTATCACGTCTGCTTT	20	6	21	1
tsrna-19705	GGTTCCATAGTGTAGCGGTTATCACGTCTGCTT	7	7	8	1
tsrna-19704	GGTTCCATAGTGTAGCGGTTATCACGTCTGCT	8	5	11	2
tsrna-19703	GGTTCCATAGTGTAGCGGTTATCACGTCTGC	9	7	2	0
tsrna-19702	GGTTCCATAGTGTAGCGGTTATCACGTCTG	3	3	3	2
tsrna-19701	GGTTCCATAGTGTAGCGGTTATCACGTCT	0	2	3	1
tsrna-19700	GGTTCCATAGTGTAGCGGTTATCACGTC	4	2	2	0
tsrna-19699	GGTTCCATAGTGTAGCGGTTATCACGT	3	5	2	0
tsrna-19698	GGTTCCATAGTGTAGCGGTTATCACG	4	1	3	0
tsrna-19697	GGTTCCATAGTGTAGCGGTTATCAC	2	6	4	0
tsrna-19696	GGTTCCATAGTGTAGCGGTTATCA	3	6	7	0
tsrna-19695	GGTTCCATAGTGTAGCGGTTATC	1	3	0	0
tsrna-19694	GGTTCCATAGTGTAGCGGTTAT	4	4	0	1
tsrna-19693	GGTTCCATAGTGTAGCGGTTA	2	3	2	0
tsrna-19692	GGTTCCATAGTGTAGCGGTT	2	3	1	0
tsrna-19691	GGTTCCATAGTGTAGCGGT	4	7	0	0
tsrna-19690	GGTTCCATAGTGTAGCGG	5	2	0	0
tsrna-19689	GGTTCCATAGTGTAGCG	1	5	2	2
tsrna-19688	GGTTCCATAGTGTAGC	1	4	1	0
tsrna-19685	GGTTCAGTGGTAGAATTCTTGCCTGC	0	0	0	1
tsrna-19684	GGTTCAGTGGTAGAATTCTTGCCT	0	0	0	0
tsrna-19683	GGTTCAGTGGTAGAATTCTTGCC	0	1	0	0
tsrna-19682	GGTTCAGTGGTAGAATTCTTGC	1	0	0	0
tsrna-19680	GGTTCAGTGGTAGAATTCTCGCCTT	4	4	2	2
tsrna-19679	GGTTCAGTGGTAGAATTCTCGCCTGCCAT	31	75	23	8
tsrna-19678	GGTTCAGTGGTAGAATTCTCGCCTGCCACG	54	92	26	15
tsrna-19677	GGTTCAGTGGTAGAATTCTCGCCTGCCAC	28	48	9	12
tsrna-19676	GGTTCAGTGGTAGAATTCTCGCCTGCCA	14	36	8	3
tsrna-19675	GGTTCAGTGGTAGAATTCTCGCCTGCC	13	41	10	7
tsrna-19674	GGTTCAGTGGTAGAATTCTCGCCTGC	10	13	7	3
tsrna-19673	GGTTCAGTGGTAGAATTCTCGCCTG	8	9	2	2
tsrna-19672	GGTTCAGTGGTAGAATTCTCGCCTCCC	12	22	2	8
tsrna-19671	GGTTCAGTGGTAGAATTCTCGCCTCC	3	17	3	9
tsrna-19670	GGTTCAGTGGTAGAATTCTCGCCTC	5	9	1	3
tsrna-19669	GGTTCAGTGGTAGAATTCTCGCCT	3	7	2	1
tsrna-19668	GGTTCAGTGGTAGAATTCTCGCC	1	2	0	1
tsrna-19667	GGTTCAGTGGTAGAATTCTCGC	0	1	0	1
tsrna-19666	GGTTCAGTGGTAGAATTCTCG	0	0	0	0
tsrna-19665	GGTTCAGTGGTAGAATTCTC	0	1	0	0
tsrna-19663	GGTTCAGTGGTAGAATTC	0	0	0	1
tsrna-19660	GGTTCAATTCCTCTTCTTAACACCA	4	0	0	1
tsrna-19659	GGTTCAATTCCTCTTCTTAACACC	0	0	0	1
tsrna-19657	GGTTCAATTCCTCTTCTTAAC	1	0	0	0
tsrna-19653	GGTTCAATTCCCGGTCAGGGAACCA	1	16	2	0
tsrna-19652	GGTTCAATTCCCCGACGGGGAGCCA	0	4	0	0
tsrna-19642	GGTTCAATGGTAGAATTCTCGCCT	2	1	0	0
tsrna-19641	GGTTCAATGGTAGAATTCTCGC	1	1	0	0
tsrna-19640	GGTTCAATGGTAGAATTCTCG	0	0	0	1
tsrna-19639	GGTTCAATCCCCGGCATCTCCACCA	121	172	70	42
tsrna-19638	GGTTCAATCCCCGGCACCTCCACCA	195	310	125	68
tsrna-19637	GGTTCAATCCCCGGCACCTCCACC	12	19	6	2
tsrna-19636	GGTTCAATCCCCGGCACCTCCAC	0	0	0	0

tsrna-19632	GGTTCAATCCCCAGCACCTCCACCA	6	12	4	2
tsrna-19631	GGTTCAATCCCCAGCACCTCCACC	0	2	0	0
tsrna-19629	GGTTCAAGTCCCTGTTCGGGCGCCA	8	13	3	3
tsrna-19622	GGTTCAAGTCCCTGTTCG	0	0	0	0
tsrna-19618	TCACGTCTGCTTTACACGC	1	0	0	0
tsrna-19617	TCACGTCTGCTTTACACG	0	1	0	0
tsrna-19616	TCACGTCGGGGTCACCA	3	1	2	1
tsrna-19612	TCACGCGGGAGACCGGGGTTCGATTCCCCGACGGGGAGCCA	2	18	0	1
tsrna-19611	TCACGCGGGAGACCGGGGTTCGATTCCCCGACGGGGAGCC	0	5	1	0
tsrna-19610	TCACGCGGGAGACCGGGGTTCGATTCCCCGACGGGGAGC	0	1	0	0
tsrna-19609	TCACGCGGGAGACCGGGGTTCGATTCCCCGACGGGGAG	0	1	1	0
tsrna-19608	TCACGCGGGAGACCGGGGTTCGATTCCCCGACGGGGA	0	1	0	0
tsrna-19607	TCACGCGGGAGACCGGGGTTCGATTCCCCGACGGGG	0	0	0	0
tsrna-19606	TCACGCGGGAGACCGGGGTTCGATTCCCCGACGGG	0	0	0	0
tsrna-19603	TCACGCGGGAGACCGGGGTTCGA	0	1	0	0
tsrna-19602	TCACGCGGGAGACCGGGGTTCAATTCCCCGACGGGGAGCCA	0	6	0	0
tsrna-19599	TCACGCGGGAGACCGGGGTTCAATTCCCCGACGGGG	1	0	0	0
tsrna-19598	TCACGCGGGAGACCGGGGTTCAATTCCCCG	0	1	0	0
tsrna-19596	TCACCGCCGCGGCCCGGGTTCGATTCCCGGTCAGGGAACCA	45	300	19	12
tsrna-19595	TCACCGCCGCGGCCCGGGTTCGATTCCCGGTCAGGGAACC	5	11	11	0
tsrna-19594	TCACCGCCGCGGCCCGGGTTCGATTCCCGGTCAGGGAAC	1	4	2	1
tsrna-19593	TCACCGCCGCGGCCCGGGTTCGATTCCCGGTCAGGGAA	0	1	0	1
tsrna-19592	TCACCGCCGCGGCCCGGGTTCGATTCCCGGTCAGGGA	0	3	2	0
tsrna-19591	TCACCGCCGCGGCCCGGGTTCGATTCCCGGTCAGGG	0	1	0	0
tsrna-19590	TCACCGCCGCGGCCCGGGTTCGATTCCCGG	0	1	0	0
tsrna-19588	TCACCGCCGCGGCCCGGGTTCG	0	1	0	0
tsrna-19587	TCACCGCCGCGGCCCGGGTTC	0	1	0	0
tsrna-19581	TCACCCCATAAACACCA	0	1	0	0
tsrna-19577	TCACCCAGGCGGCCCGGGTTCGACTCCCGGTGTGGGAACCA	89	297	50	27
tsrna-19576	TCACCCAGGCGGCCCGGGTTCGACTCCCGGTGTGGGAACC	5	12	7	3
tsrna-19575	TCACCCAGGCGGCCCGGGTTCGACTCCCGGTGTGGGAAC	3	2	2	0
tsrna-19573	TCACCCAGGCGGCCCGGGTTCGACTCCCGGTGTG	0	1	0	0
tsrna-19570	TCACCCAGGCGGCCCGGGTTCGACTCCC	0	0	0	0
tsrna-19564	TCACCCAGGCGGCCCGGG	0	0	0	0
tsrna-19561	TCACATCACCCCATAAACACCA	3	1	4	3
tsrna-19554	TCACACGCGAAAGGTCCCCGGTTT	2	12	0	0
tsrna-19553	TCACACGCGAAAGGTCCCCGGTTCGAAACC	3	7	0	1
tsrna-19552	TCACACGCGAAAGGTCCCCGGTT	2	6	0	1
tsrna-19551	TCACACGCGAAAGGTCCCCGGT	2	6	0	0
tsrna-19549	TCACACGCGAAAGGTCCCCG	0	1	0	0
tsrna-19547	TCACAAGAACTGCTAACTCATGCCCCCATGTCTAACAACATGC	0	3	4	2
tsrna-19546	TCACAAGAACTGCTAACTCATGCCCCCATGTCTAACAACATGC	0	2	2	0
tsrna-19545	TCACAAGAACTGCTAACTCATGCCCCCATGTCTAACAACATGC	1	2	2	0
tsrna-19544	TCACAAGAACTGCTAACTCATGCCCCCATGTCTAACAACA	1	1	1	0
tsrna-19543	TCACAAGAACTGCTAACTCATGCCCCCATGTCTAACA	0	0	0	1
tsrna-19542	TCACAAGAACTGCTAACTCATGCCCCCATGTCTAAC	1	1	3	1
tsrna-19541	TCACAAGAACTGCTAACTCATGCCCCCATG	3	1	1	0
tsrna-19540	TCACAAGAACTGCTAACTCATGCCCCC	0	0	0	0
tsrna-19539	TCACAAGAACTGCTAACTCATGCCC	0	0	2	0
tsrna-19538	TCACAAGAACTGCTAACTCATGCC	1	1	0	0
tsrna-19537	TCACAAGAACTGCTAACTCATGC	1	0	0	0
tsrna-19536	TCACAAGAACTGCTAACTCATG	0	1	0	0
tsrna-19534	TCACAAGAACTGCTAACTCA	2	0	0	0
tsrna-19533	TCACAAGAACTGCTAACTC	0	0	1	0
tsrna-19532	TCACAAGAACTGCTAACT	1	0	0	0
tsrna-19531	TCACAAGAACTGCTAAC	0	0	0	1
tsrna-19529	TCAATTCTCGCTGGGGCCTCCA	39	42	17	18
tsrna-19528	TCAATTCCTCTTCTTAACACCA	0	3	0	1
tsrna-19527	TCAATTCCGGCTCGAAGGACCA	48	142	22	16
tsrna-19526	TCAATTCCCGGCCAATGCACCA	11	15	8	16
tsrna-19525	TCAATTCCCGGCCAATGCACC	3	3	0	1
tsrna-19523	TCAATTCCCCGACGGGGAGCC	1	0	0	0
tsrna-19520	TCAATGGTAGAATTCTCGCCT	2	3	3	0
tsrna-19519	TCAATGGTAGAATTCTCGCC	3	2	0	0

tsrna-19518	TCAATGGTAGAATTCTCGC	0	0	0	0
tsrna-19517	TCAATCCCTGGCACCTCCACCA	3	2	1	0
tsrna-19516	TCAATCCCGGGTTTCGGCACCA	35	81	5	9
tsrna-19515	TCAATCCCGGGTTTCGGCACC	3	14	1	1
tsrna-19514	TCAATCCCCGGCATCTCCACCA	140	144	57	43
tsrna-19513	TCAATCCCCGGCATCTCCACC	9	4	5	0
tsrna-19510	TCAATCCCCGGCACCTCCACCA	219	327	126	57
tsrna-19509	TCAATCCCCGGCACCTCCACC	11	17	8	2
tsrna-19508	TCAATCCCCGGCACCTCCAC	0	0	1	0
tsrna-19504	TCAATCCCCAGCACCTCCACCA	4	8	1	3
tsrna-19503	TCAATCCCCAGCACCTCCACC	1	0	0	0
tsrna-19502	TCAATCCCCAATACCTCCACCA	0	1	2	0
tsrna-19501	TCAAGTTCTGGTCTCCGGATGGA	0	1	0	0
tsrna-19498	TCAAGTTCTGGTCTCCGGA	0	0	1	0
tsrna-19494	TCAAGTCTCGGTGGAACCTCCA	56	109	18	13
tsrna-19493	TCAAGTCTCGGTGGAACCTCC	2	5	1	1
tsrna-19492	TCAAGTCCCTGTTCGGGCGCCA	5	11	5	2
tsrna-19488	TCAAGTCCCTGTTCAGGCGCCA	0	2	0	0
tsrna-19487	TCAAGTCCCTGTTCAGGCGCC	0	1	0	1
tsrna-19486	TCAAGTCCCTGTTCAGGCACCA	0	3	2	0
tsrna-19485	TCAAGTCCCTGTCCAGGCGCCA	0	1	0	0
tsrna-19484	TCAAGTCACGTCGGGGTCACCA	4	4	4	4
tsrna-19483	TCAAGTCACGTCGGGGTCACC	0	1	1	0
tsrna-19477	TCAAGCCCCAGTGGAACCACCA	2	5	2	5
tsrna-19476	TCAAGAGGTCCCTGGTTCAAATCCGGGTGCCCCCTCC	8	14	1	2
tsrna-19475	TCAAGAGGTCCCCGGTTCAAATCCGGGTGCCCCCTCC	5	7	3	2
tsrna-19474	TCAAGAGGTCCCCGGTTCAAATCCGGGTGCCCCCTC	0	4	3	0
tsrna-19473	TCAAGAGGTCCCCGGTTCAAATCCGGGTGCCCCCT	1	3	2	1
tsrna-19472	TCAAGAGGTCCCCGGTTCAAATCCGGGTGC	0	3	0	2
tsrna-19471	TCAAGAGGTCCCCGGTTCAAA	4	4	0	1
tsrna-19470	TCAAGAGGTCCCCGGTTCA	2	1	1	0
tsrna-19469	TCAAGAGGTCCCCGGTTC	2	3	1	0
tsrna-19468	TCAAGAGGTCCCCGGTT	1	5	0	2
tsrna-19467	TCAAGAGGTCCCCGGT	1	4	0	1
tsrna-19466	TCAACTTAACTTGACCGCTCTGACCA	14	23	10	7
tsrna-19465	TCAACTTAACTTGACCGCTCTGACC	3	2	3	0
tsrna-19464	TCAACTTAACTTGACCGCTCTGAC	2	0	0	0
tsrna-19463	TCAACTTAACTTGACCGCTCTGA	1	0	0	0
tsrna-19462	TCAACTTAACTTGACCGCTCTG	2	1	1	0
tsrna-19455	TCAAATCTGGGTGCCCCCTCCA	1	1	0	0
tsrna-19454	TCAAATCTCGGTGGGACCTCCA	80	107	36	24
tsrna-19453	TCAAATCTCGGTGGGACCTCC	7	8	0	0
tsrna-19452	TCAAATCTCGGTGGGACCTC	0	0	0	0
tsrna-19451	TCAAATCTCGGTGGGACC	0	1	0	0
tsrna-19450	TCAAATCTCGGTGGAACCTCCA	156	218	47	16
tsrna-19449	TCAAATCTCGGTGGAACCTCC	16	13	1	0
tsrna-19448	TCAAATCTCGGTGGAACCTC	1	4	0	0
tsrna-19447	TCAAATCTCGGTGGAACCT	1	1	0	0
tsrna-19444	TCAAATCTCGCTGGGGCCTCCA	37	56	16	21
tsrna-19443	TCAAATCTCGCTGGGGCCTCC	7	5	1	1
tsrna-19442	TCAAATCTCGCTGGGGCCTC	0	0	0	0
tsrna-19441	TCAAATCCGGGTGCCCCCTCCA	148	165	77	19
tsrna-19440	TCAAATCCGGGTGCCCCCTCC	3	11	0	0
tsrna-19439	TCAAATCCGGGTGCCCCCTC	0	3	0	0
tsrna-19436	TCAAATCCCGGACGAGCCCCCA	117	186	43	59
tsrna-19435	TCAAATCCCGGACGAGCCCCC	5	7	1	3
tsrna-19434	TCAAATCCCGGACGAGCCCC	1	3	1	0
tsrna-19433	TCAAATCCCGGACGAGCCC	0	0	0	0
tsrna-19432	TCAAATCCCGGACGAGCC	0	0	0	0
tsrna-19429	TCAAATCCCACCGCTGCCACCA	0	0	0	0
tsrna-19428	TCAAATCCAGGTGCCCCCTCCA	2	4	0	2
tsrna-19427	TCAAATCACGTCGGGGTCACCA	5	10	7	9
tsrna-19426	TCAAATCACGTCGGGGTCACC	1	0	1	0
tsrna-19420	TCAAAGTTAAATTATAGGCTAAATCCTATATATCTTACCA	7	4	8	0

tsrna-19419	TCAAAGTTAAATTATAGGCTAAATCCTATATATCTTACC	5	0	8	1
tsrna-19418	TCAAAGTTAAATTATAGGCTAAATCCTATATATCTT	9	2	12	0
tsrna-19417	TCAAAGTTAAATTATAGGCTAAATCCTATATATCT	8	1	13	1
tsrna-19416	TCAAAGTTAAATTATAGGCTAAATCCTATATATC	6	3	9	1
tsrna-19415	TCAAAGTTAAATTATAGGCTAAATCCTATATA	9	2	5	0
tsrna-19414	TCAAAGTTAAATTATAGGCTAAATCCTAT	3	1	1	0
tsrna-19409	TCAAAGGTTGTGGGTTCGAGTCCCACCAGAGTCGCCA	4	14	1	2
tsrna-19408	TCAAAGGTTGTGGGTTCGAGTCCCACCAGAGTCGCC	0	2	0	0
tsrna-19407	TCAAAGGTTGTGGGTTCGAGTCCCACCAGA	0	3	0	0
tsrna-19406	TCAAAGGTTGTGGGTTCGAGTCCCACCAG	0	1	1	0
tsrna-19405	TCAAAGGTTGTGGGTTCGAATCCCACCAGAGTCGCCA	6	10	0	0
tsrna-19404	TCAAAGGTTGTGGGTTCGAATCCCACCAGA	1	0	0	0
tsrna-19403	TCAAAGGTTGTGGGTTCGAATCCCACCAG	0	1	0	0
tsrna-19402	TCAAAGGTTGTGGGTTC	1	1	0	2
tsrna-19401	TCAAAGGTTGTGGGTT	0	1	0	0
tsrna-19400	TCAAAGGTTCCGGGTTCGAGTCCCGGCGGAGTCGCCA	1	3	0	0
tsrna-19399	TCAAAGGTTCCGGGTTCGAGTCCCGGCGGAGTCGCC	0	0	0	0
tsrna-19398	TCAAAGGTTCCGGGTTCGAGTCCCGGCGGAGTCGC	0	0	1	0
tsrna-19397	TCAAAGGTTCCGGGTTCGAGTCCCGGCGGAGTC	0	0	0	0
tsrna-19396	TCAAAGGTTCCGGGTTCGAGTCCCGGCGGA	0	1	0	0
tsrna-19393	TCAAAGGTTCCGGGTTC	1	1	0	0
tsrna-19392	TCAAAGGTTCCGGGTT	0	1	0	0
tsrna-19385	TCAAAGCAATACACTGAAAATGTTTAGACGGGCTCA	13	22	7	0
tsrna-19384	TCAAAGCAATACACTGAAAATGTTTAGACG	0	1	0	0
tsrna-19383	TCAAAGCAATACACTGAA	0	0	0	0
tsrna-19380	TCAAACCTGCCGGGGCTTCCA	1	6	2	2
tsrna-19379	TCAAACCTGCCGGGGCTTCC	0	0	0	0
tsrna-19376	TCAAACCTGCCGGGGCT	1	0	0	0
tsrna-19373	TATTGAATTGCAAATTCGAAGAAGCAGCTTCAAACCTGCCGG	1	0	0	0
tsrna-19372	TATTGAATTGCAAATTCGAAGAAGCAGCTTCAAACCTGCCGG	0	1	1	0
tsrna-19370	TATGTCCGCGTGGGTTCGAACC	5	4	1	0
tsrna-19369	TATGTCCGCGTGGGTTCGAAC	5	6	1	0
tsrna-19368	TATGTCCGCGTGGGTTCGAA	3	8	3	0
tsrna-19367	TATGTCCGCGTGGGTTCGA	1	5	1	0
tsrna-19366	TATGTCCGCGTGGGTTCG	3	1	0	0
tsrna-19365	TATGTCCGCGTGGGTT	1	4	2	0
tsrna-19364	TATGTAGCTTACCTCCTCAAAGCA	0	0	0	1
tsrna-19360	TATGTAGCTTACCTCC	1	0	0	0
tsrna-19359	TATGGTCTAGCGGTTAGGATTCCTGGTTTT	5	9	10	2
tsrna-19358	TATGGTCTAGCGGTTAGGATTCCTGGTT	5	5	4	2
tsrna-19357	TATGGTCTAGCGGTTAGGATTCCTGG	4	4	2	0
tsrna-19356	TATGGTCTAGCGGTTAGGATTCCTG	1	1	0	0
tsrna-19355	TATGGTCTAGCGGTTAGGATTCCT	2	1	1	1
tsrna-19354	TATGGTCTAGCGGTTAGGATTCC	0	0	0	0
tsrna-19347	TATGATTCTCGGTTTGGGTC	1	0	0	0
tsrna-19344	TATGATTCTCGGTTTGG	0	1	0	0
tsrna-19342	TATGATTCTCGCTTTGGGTGCGAGAGGTCCCGGGTT	10	75	4	24
tsrna-19341	TATGATTCTCGCTTTGGGTGCGAGAGGTCCCGGGT	9	60	3	10
tsrna-19338	TATGATTCTCGCTTCGGGTGTGAGAGGTCCCGGGT	2	1	0	0
tsrna-19337	TATGATTCTCGCTTCGGGTGTGAGAGGTCC	1	0	0	0
tsrna-19333	TATGATTCTCGCTTCGGGTGCGAGAGGTCCCGGGT	0	10	1	0
tsrna-19329	TATGATTCTCGCTTCGGGTG	2	0	0	0
tsrna-19326	TATGATAATCATATTTACCAACC	0	0	1	0
tsrna-19320	TATGAGGTCCCGGGTTCGATCCCCGGC	0	4	0	0
tsrna-19319	TATGAGGTCCCGGGTTCGA	0	6	0	0
tsrna-19318	TATGAGGCCCCGGGTTCGATCCCCGGC	1	6	0	0
tsrna-19317	TATGAGGCCCCGGGTTCAATCCCCGGC	0	3	0	0
tsrna-19316	TATCGGGCCCATACCCCGAAAATGTTGGTTATACCC	1	2	1	0
tsrna-19315	TATCGGGCCCATACCCCGAAAATG	2	0	0	0
tsrna-19314	TATCGGGCCCATACCCCGAAA	0	1	0	0
tsrna-19313	TATCGGGCCCATACCC	0	1	0	0
tsrna-19312	TATCCCCGCCTGTCACGCGGGAGACCGG	12	52	9	7
tsrna-19307	TATCCATTGGTCTTAGGCCCCA	2	1	0	0
tsrna-19306	TATCCATTGGTCTTAGGCCCC	0	0	0	0

tsrna-19303	TATCCATTGGTCTTAGGC	0	1	0	0
tsrna-19301	TATCCATTGGTCTTAG	0	0	0	0
tsrna-19300	TATCATTGGTCGTGGTTGTAGTCCGTGCGAGAATACCA	127	166	159	54
tsrna-19299	TATCATTGGTCGTGGTTGTAGTCCGTGCGAGAATACC	15	16	30	16
tsrna-19298	TATCATTGGTCGTGGTTGTAGTCCGTGCGAGAATAC	11	19	12	12
tsrna-19297	TATCATTGGTCGTGGTTGTAGTCCGTGCGAGAATA	21	13	11	17
tsrna-19296	TATCATTGGTCGTGGTTGTAGTCCGTGCGAGAAT	10	12	11	10
tsrna-19295	TATCATTGGTCGTGGTTGTAGTCCGTGCGAGAA	13	14	9	9
tsrna-19294	TATCATTGGTCGTGGTTGTAGTCCGTGCGAGA	8	12	6	7
tsrna-19293	TATCATTGGTCGTGGTTGTAGTCCGTGCGAG	8	7	5	5
tsrna-19292	TATCATTGGTCGTGGTTGTAGTCCGTGCGA	5	2	4	5
tsrna-19291	TATCATTGGTCGTGGTTGTAGTCCGTGCG	2	9	10	2
tsrna-19290	TATCATTGGTCGTGGTTGTAGTCCGTGC	1	4	2	6
tsrna-19289	TATCATTGGTCGTGGTTGTAGTCCGTG	3	3	1	1
tsrna-19288	TATCATTGGTCGTGGTTGTAGTCCGT	3	3	3	0
tsrna-19287	TATCATTGGTCGTGGTTGTAGTCCG	2	0	2	0
tsrna-19286	TATCATTGGTCGTGGTTGTAGTCC	2	1	2	0
tsrna-19285	TATCATTGGTCGTGGTTGTAGTC	1	0	0	0
tsrna-19284	TATCATTGGTCGTGGTTGTAGT	0	0	0	0
tsrna-19283	TATCATTGGTCGTGGTTGTAG	0	0	0	0
tsrna-19275	TATCACGTTCGCCTCACACGC	0	0	0	0
tsrna-19270	TATCACGTCTGCTTTACACGC	0	1	0	0
tsrna-19269	TATCACGTCTGCTTTACACG	0	1	0	0
tsrna-19266	TATAGTGGTTAGTACTCTGCGTTGTGGCCG	29	11	15	6
tsrna-19265	TATAGTGGTTAGTACTCTGCGTTGTGGCC	33	17	13	6
tsrna-19264	TATAGTGGTTAGTACTCTGCGTTGTGGC	24	5	11	14
tsrna-19263	TATAGTGGTTAGTACTCTGCGTTGTGG	31	12	15	7
tsrna-19262	TATAGTGGTTAGTACTCTGCGTTGTG	31	16	9	8
tsrna-19261	TATAGTGGTTAGTACTCTGCGTTGT	22	5	10	7
tsrna-19260	TATAGTGGTTAGTACTCTGCGTTG	21	8	9	6
tsrna-19259	TATAGTGGTTAGTACTCTGCGTT	17	4	6	4
tsrna-19258	TATAGTGGTTAGTACTCTGCGT	5	1	3	0
tsrna-19257	TATAGTGGTTAGTACTCTGCGCT	5	3	5	2
tsrna-19256	TATAGTGGTTAGTACTCTGCGC	2	2	2	0
tsrna-19255	TATAGTGGTTAGTACTCTGCG	2	3	1	0
tsrna-19254	TATAGTGGTTAGTACTCTGC	1	2	1	0
tsrna-19253	TATAGTGGTTAGTACTCTG	0	0	0	1
tsrna-19252	TATAGTGGTTAGTACTCT	1	0	0	0
tsrna-19250	TATAGTGGTGAGTATCCCCGCCTGTC	3	8	2	4
tsrna-19249	TATAGTGGTGAGTATCCCCGCCTGT	1	3	0	1
tsrna-19248	TATAGTGGTGAGTATCCCCGCCT	1	4	0	0
tsrna-19247	TATAGTGGTGAGTATCCCCGCC	1	6	0	0
tsrna-19246	TATAGTGGTGAGTATCCCCGC	1	2	0	0
tsrna-19244	TATAGTGGTGAGCATAGCTGCCT	0	1	0	0
tsrna-19243	TATAGTGGTGAGCATAGCTGC	0	1	0	0
tsrna-19241	TATAGCTCAGTGGTAGAGCATTTGACTGC	6	3	1	2
tsrna-19240	TATAGCTCAGTGGTAGAGCATTTGACTG	1	0	1	0
tsrna-19239	TATAGCTCAGTGGTAGAGCATTTGACT	2	1	2	0
tsrna-19237	TATAGCTCAGTGGTAGAGCATTTG	0	0	1	0
tsrna-19236	TATAGCTCAGTGGGTAGAGCATTTGACTG	0	2	0	0
tsrna-19235	TATAGCTCAGTGGGTAGAGCATTTGACT	0	0	1	0
tsrna-19233	TATAGCTCAGGTGGTAGAGCATTTGACTG	1	2	0	0
tsrna-19232	TATAGCTCAGGGGTTAGAGCACTGG	0	1	0	0
tsrna-19231	TATAGCTCAGGGGTAGAGCATTTGACTGC	1	0	1	4
tsrna-19230	TATAGCTCAGGGGTAGAGCATTTGACTG	0	2	0	2
tsrna-19229	TATAGCTCAGGGGTAGAGCATTTGACT	0	0	0	0
tsrna-19228	TATAGCTCAGGGGTAGAGCATTTG	0	1	0	1
tsrna-19227	TATACCCTTCCCGTACTACCA	1	2	1	0
tsrna-19226	TATACCCTTCCCGTACTACC	1	0	0	0
tsrna-19222	TATAATGCCGAGGTTGTGAGTTCG	5	24	8	1
tsrna-19221	TATAATGCCGAGGTTGTGAGTTC	1	7	2	0
tsrna-19220	TATAATGCCGAGGTTGTGAGTT	1	9	1	1
tsrna-19219	TATAATGCCGAGGTTGTGAGT	3	2	0	0
tsrna-19218	TATAATGCCGAGGTTGTGAG	3	6	1	0

tsrna-19217	TATAATGCCGAGGTTGTG	0	2	1	0
tsrna-19216	TATAAATAGTACCGTTAACTTCCA	0	0	1	0
tsrna-19215	TATAAATAGTACCGTTAACTTCC	0	0	0	0
tsrna-19214	TATAAATAGTACCGTTAACTTC	0	0	0	0
tsrna-19212	TAGTTTTGACAACATTCAAAAAAGAGTACC	0	0	1	2
tsrna-19204	TAGTGTAGTGGTTATCACGTTCGCCT	0	1	0	0
tsrna-19201	TAGTGTAGTGGTTATCACGTCTGCTTTACACGCAGA	3	0	2	1
tsrna-19200	TAGTGTAGTGGTTATCACGTCTGCTTT	0	0	0	1
tsrna-19199	TAGTGTAGTGGTTATCACGTCTGCTT	0	0	0	1
tsrna-19192	TAGTGTAGTGGTCATCACGTTCGCCT	0	1	0	0
tsrna-19191	TAGTGTAGTGGTCATCACGTTCGCC	0	0	0	0
tsrna-19188	TAGTGTAGTGGTCATC	0	0	0	0
tsrna-19187	TAGTGTAGCGGTTATCACGTCTGC	0	0	0	0
tsrna-19186	TAGTGTAGCGGTTATCACATTCGCC	1	0	1	0
tsrna-19185	TAGTGTAGCGGTTATC	1	0	0	0
tsrna-19184	TAGTGGTTATCACGTTCGCCTCACACGC	1	0	0	0
tsrna-19182	TAGTGGTTATCACGTTCGCCTC	0	0	0	0
tsrna-19180	TAGTGGTTATCACGTTCGCC	0	1	0	0
tsrna-19177	TAGTGGTTATCACGTCTGCTTTACA	1	0	3	0
tsrna-19175	TAGTGGTTATCACGTCTGCTT	1	0	0	0
tsrna-19172	TAGTGGTTAGTATCCCCGCCTGTCACGCGG	13	5	4	6
tsrna-19171	TAGTGGTTAGTATCCCCGCCTGTC	2	1	4	1
tsrna-19170	TAGTGGTTAGTATCCCCGCCTGT	2	0	0	1
tsrna-19169	TAGTGGTTAGTATCCCCGCCTG	0	1	1	0
tsrna-19168	TAGTGGTTAGTATCCCCGCCT	1	0	0	0
tsrna-19167	TAGTGGTTAGTATCCCCGCC	1	0	0	0
tsrna-19166	TAGTGGTTAGTATCCCCGC	2	0	0	0
tsrna-19165	TAGTGGTTAGTATCCCCG	1	0	0	0
tsrna-19164	TAGTGGTTAGTACTCTGCGTTGTGGCCGCAGCAACCT	53	20	17	6
tsrna-19163	TAGTGGTTAGTACTCTGCGTTGTGGCCGC	19	7	14	11
tsrna-19162	TAGTGGTTAGTACTCTGCGTTGTGGCC	27	11	6	3
tsrna-19161	TAGTGGTTAGTACTCTGCGTTGTGGC	26	10	8	8
tsrna-19160	TAGTGGTTAGTACTCTGCGTTGTGG	27	15	19	5
tsrna-19159	TAGTGGTTAGTACTCTGCGTTGTG	36	11	5	7
tsrna-19158	TAGTGGTTAGTACTCTGCGTTGT	22	9	11	6
tsrna-19157	TAGTGGTTAGTACTCTGCGTTG	26	13	6	4
tsrna-19156	TAGTGGTTAGTACTCTGCGTT	22	5	5	4
tsrna-19155	TAGTGGTTAGTACTCTGCGT	6	3	2	1
tsrna-19154	TAGTGGTTAGTACTCTGCGCTGT	8	0	5	2
tsrna-19153	TAGTGGTTAGTACTCTGCGCTG	4	3	2	1
tsrna-19152	TAGTGGTTAGTACTCTGCGCT	2	1	4	0
tsrna-19151	TAGTGGTTAGTACTCTGCGC	6	1	1	1
tsrna-19150	TAGTGGTTAGTACTCTGCG	1	1	0	0
tsrna-19149	TAGTGGTTAGTACTCTGC	0	0	0	1
tsrna-19147	TAGTGGTTAGTACTCT	0	0	0	0
tsrna-19146	TAGTGGTTAGGATTCGGCGCTCTCACCGCCGCGGCCCGGGT	51	73	40	18
tsrna-19145	TAGTGGTTAGGATTCGGCGCTCTCACCGCCGCGGCCCG	57	85	36	14
tsrna-19144	TAGTGGTTAGGATTCGGCGCTCTCACCGCCGCGGCCC	59	76	34	24
tsrna-19143	TAGTGGTTAGGATTCGGCGCTCTCACCGCCGCGGCC	43	40	25	26
tsrna-19142	TAGTGGTTAGGATTCGGCGCTCTCACCGCC	39	55	32	21
tsrna-19141	TAGTGGTTAGGATTCGGCGCTCTCACCGC	25	48	25	19
tsrna-19140	TAGTGGTTAGGATTCGGCGCTCTCACC	36	39	29	14
tsrna-19139	TAGTGGTTAGGATTCGGCGCTCTCAC	35	41	21	19
tsrna-19138	TAGTGGTTAGGATTCGGCGCTCTCA	39	39	23	15
tsrna-19137	TAGTGGTTAGGATTCGGCGCTCTC	43	38	23	15
tsrna-19136	TAGTGGTTAGGATTCGGCGCTCT	56	53	28	11
tsrna-19135	TAGTGGTTAGGATTCGGCGCTC	33	40	21	9
tsrna-19134	TAGTGGTTAGGATTCGGCGCT	28	43	19	12
tsrna-19133	TAGTGGTTAGGATTCGGCGC	19	17	10	8
tsrna-19132	TAGTGGTTAGGATTCGGCG	15	9	11	3
tsrna-19131	TAGTGGTTAGGATTCGGC	12	4	4	1
tsrna-19130	TAGTGGTTAGGATTCGG	3	0	0	2
tsrna-19129	TAGTGGTTAGGATTCG	0	0	0	0
tsrna-19128	TAGTGGTTAGGATTCAGCGCT	16	21	13	4

tsrna-19127	TAGTGGTTAGCATAGCTGCCTTCC	0	1	0	0
tsrna-19124	TAGTGGTTAGCATAGCTGCCT	0	1	0	0
tsrna-19122	TAGTGGTTAGCATAGCTGC	1	0	0	0
tsrna-19118	TAGTGGTGAGTATCCCCGCCTGTCACGCGG	12	22	9	12
tsrna-19117	TAGTGGTGAGTATCCCCGCCTGTCACGC	8	25	11	9
tsrna-19116	TAGTGGTGAGTATCCCCGCCTGTCAC	5	14	4	6
tsrna-19115	TAGTGGTGAGTATCCCCGCCTGTCA	4	12	0	1
tsrna-19114	TAGTGGTGAGTATCCCCGCCTGTC	5	9	1	1
tsrna-19113	TAGTGGTGAGTATCCCCGCCTGT	1	3	0	0
tsrna-19112	TAGTGGTGAGTATCCCCGCCTG	1	2	0	0
tsrna-19111	TAGTGGTGAGTATCCCCGCCT	0	5	0	0
tsrna-19110	TAGTGGTGAGTATCCCCGCC	2	2	0	1
tsrna-19109	TAGTGGTGAGTATCCCCGC	2	1	0	0
tsrna-19108	TAGTGGTGAGTATCCCCG	0	1	0	0
tsrna-19105	TAGTGGTGAGCATAGCTGCCTTCC	0	0	1	0
tsrna-19104	TAGTGGTGAGCATAGCTGCCTTC	1	0	0	0
tsrna-19103	TAGTGGTGAGCATAGCTGCCTT	3	1	0	0
tsrna-19101	TAGTGGTGAGCATAGCTGCC	1	0	0	0
tsrna-19097	TAGTGGTGAGCATAGC	1	0	0	0
tsrna-19096	TAGTGGTCATCACGTTCGCCT	0	0	1	0
tsrna-19095	TAGTGGTCATCACGTTCGCC	0	1	0	0
tsrna-19094	TAGTGGTATGATTCTCGCTTT	13	3	1	1
tsrna-19093	TAGTGGTATGATTCTCGCTT	6	1	4	6
tsrna-19092	TAGTGGTATGATTCTCGCT	6	2	2	6
tsrna-19091	TAGTGGTATGATTCTCGC	8	1	0	6
tsrna-19083	TAGTGGTAGAATTCTCGCCT	0	5	2	1
tsrna-19082	TAGTGGTAGAATTCTCGCC	1	6	1	0
tsrna-19081	TAGTGGTAGAATTCTCGC	0	1	0	1
tsrna-19080	TAGTGGTAGAATTCTCG	0	1	0	0
tsrna-19074	TAGTGGCTAGGATTCGGCGCTTTCACCGCCGCGGCCC	22	63	18	42
tsrna-19073	TAGTGGCTAGGATTCGGCGCTTTC	17	45	10	32
tsrna-19072	TAGTGGCTAGGATTCGGCGCTTT	12	41	8	31
tsrna-19071	TAGTGGCTAGGATTCGGCGCTT	17	36	5	32
tsrna-19070	TAGTGGCTAGGATTCGGCGCT	18	37	6	36
tsrna-19069	TAGTGGCTAGGATTCGGCGC	6	28	6	23
tsrna-19068	TAGTGGCTAGGATTCGGCG	8	20	6	23
tsrna-19067	TAGTGGCTAGGATTCGGC	6	14	4	7
tsrna-19066	TAGTGGCTAGGATTCGG	1	0	0	0
tsrna-19065	TAGTCGTGGCCGAGTGGTTAAGGT	0	0	1	0
tsrna-19064	TAGTCGTGGCCGAGTGGTTAAGGC	0	0	0	0
tsrna-19063	TAGTCGTGGCCGAGTGGTTAAGG	1	1	0	0
tsrna-19062	TAGTCGTGGCCGAGTGGTTAAG	0	0	0	1
tsrna-19061	TAGTCGTGGCCGAGTGGTTAA	1	0	0	0
tsrna-19060	TAGTCGTGGCCGAGTGGTTA	1	0	0	0
tsrna-19059	TAGTCGTGGCCGAGTGG	1	1	0	0
tsrna-19058	TAGTCCGTGCGAGAATACCA	1	0	0	0
tsrna-19057	TAGTCCGTGCGAGAATACC	0	0	0	0
tsrna-19056	TAGTCCGTGCGAGAATAC	0	0	0	0
tsrna-19054	TAGTATCCCCGCCTGTCACGC	0	1	0	0
tsrna-19052	TAGTATCCCCGCCTGTC	0	1	0	0
tsrna-19049	TAGTACTCTGCGTTGTGGCCGCAGCAACCTCGGT	11	2	4	2
tsrna-19048	TAGTACTCTGCGTTGTGGCCGCAGCAACCT	1	2	0	0
tsrna-19047	TAGTACTCTGCGTTGTGGCCGCAGC	0	2	0	0
tsrna-19045	TAGTACTCTGCGTTGTGGCCGC	1	0	0	0
tsrna-19044	TAGTACTCTGCGTTGTGGCCG	1	2	0	0
tsrna-19043	TAGTACTCTGCGTTGTGGCC	2	0	0	0
tsrna-19042	TAGTACTCTGCGTTGTGGC	1	0	0	0
tsrna-19041	TAGTACTCTGCGTTGTGG	0	0	0	0
tsrna-19040	TAGTACTCTGCGTTGTG	0	0	0	0
tsrna-19039	TAGTACTCTGCGTTGT	0	0	0	0
tsrna-19038	TAGTACCGTTAACTTCCAATTAACTAGTTT	0	2	0	0
tsrna-19037	TAGTACCGTTAACTTCCAATTA	1	0	1	0
tsrna-19036	TAGTACCGTTAACTTCCAATT	0	0	0	0
tsrna-19035	TAGTACCGTTAACTTCCA	1	0	0	0

tsrna-19032	TAGGTTCGACTCCTGGCTGGCTCGCCA	39	138	12	8
tsrna-19031	TAGGTTCGACTCCTGGCTGGCTCGCC	0	4	1	1
tsrna-19030	TAGGTTCGACTCCTGGCTGGCTCG	0	2	0	0
tsrna-19029	TAGGTTCGACTCCTGGCTGGCTC	0	0	0	0
tsrna-19027	TAGGTGGTTCAGTGGTAGAATTCT	5	2	1	3
tsrna-19026	TAGGTGGCACGGAGAATTTTGGATT	0	0	0	0
tsrna-19025	TAGGTGGCACGGAGAATTTTGGAT	0	2	1	0
tsrna-19024	TAGGTGGCACGGAGAATTTTGGA	1	0	0	0
tsrna-19023	TAGGTGGCACGGAGAATTTTG	0	0	0	1
tsrna-19021	TAGGTCGCTGGTTCGATTCCGGC	0	1	1	0
tsrna-19020	TAGGTCGCTGGTTCGAATCCGGCT	1	0	0	0
tsrna-19019	TAGGTCGCTGGTTCGAATCCGGC	0	0	0	1
tsrna-19018	TAGGTCGCTGGTTCGAATCCGG	0	0	2	0
tsrna-19016	TAGGGTGCTTAGCTGTTAACTA	0	0	0	1
tsrna-19013	TAGGGGTATGATTCTCGGTTTGG	4	1	2	0
tsrna-19012	TAGGGGTATGATTCTCGGTTTG	5	1	2	0
tsrna-19011	TAGGGGTATGATTCTCGGTTT	3	2	0	1
tsrna-19010	TAGGGGTATGATTCTCGGTT	2	1	2	2
tsrna-19009	TAGGGGTATGATTCTCGGT	1	0	1	0
tsrna-19008	TAGGGGTATGATTCTCGG	1	0	0	0
tsrna-19007	TAGGGGTATGATTCTCGCTTTG	16	10	5	5
tsrna-19006	TAGGGGTATGATTCTCGCTTT	14	12	5	11
tsrna-19005	TAGGGGTATGATTCTCGCTTCG	13	14	8	11
tsrna-19004	TAGGGGTATGATTCTCGCTTC	10	10	4	21
tsrna-19003	TAGGGGTATGATTCTCGCTT	9	9	4	6
tsrna-19002	TAGGGGTATGATTCTCGCT	13	10	7	1
tsrna-19001	TAGGGGTATGATTCTCGC	11	9	9	6
tsrna-19000	TAGGGGTATGATTCTCG	0	0	1	0
tsrna-18994	TAGGCTAAATCCTATATATCTTACCA	0	0	0	0
tsrna-18993	TAGGCTAAATCCTATATATCTTACC	2	0	0	1
tsrna-18992	TAGGCTAAATCCTATATATCT	0	1	0	0
tsrna-18991	TAGGCTAAATCCTATATATC	1	0	0	0
tsrna-18990	TAGGCCTCTTTTTACCACCA	1	0	0	1
tsrna-18985	TAGGATTCGGTGCTCTCA	0	0	1	0
tsrna-18983	TAGGATTCGGCGCTTTCACCGCCGCGGCCC	1	23	7	9
tsrna-18982	TAGGATTCGGCGCTTT	0	4	2	1
tsrna-18981	TAGGATTCGGCGCTCTCAT	0	0	0	0
tsrna-18980	TAGGATTCGGCGCTCTCACCGCCGCGGCCCGGGTTC	7	14	3	4
tsrna-18979	TAGGATTCGGCGCTCTCACCGCCGCGGCCCGGGTT	5	19	2	2
tsrna-18978	TAGGATTCGGCGCTCTCACCGCCGCGGCCCGGG	3	7	4	2
tsrna-18977	TAGGATTCGGCGCTCTCACCGCCGCGGCCC	4	10	5	0
tsrna-18976	TAGGATTCGGCGCTCTCACCGCCGCGGCC	2	12	6	0
tsrna-18975	TAGGATTCGGCGCTCTCACCGCCGCGGC	9	13	4	5
tsrna-18974	TAGGATTCGGCGCTCTCACCGCC	3	12	2	3
tsrna-18973	TAGGATTCGGCGCTCTCACCGC	2	7	1	0
tsrna-18972	TAGGATTCGGCGCTCTCACCG	1	2	1	1
tsrna-18971	TAGGATTCGGCGCTCTCACC	2	3	1	1
tsrna-18970	TAGGATTCGGCGCTCTCAC	1	2	0	0
tsrna-18969	TAGGATTCGGCGCTCTCA	1	0	1	0
tsrna-18968	TAGGATTCGGCGCTCTC	0	4	0	1
tsrna-18967	TAGGATTCGGCGCTCT	0	2	2	1
tsrna-18966	TAGGATTCCTGGTTTTCACCCAGGCGGCCCGGGTTCGACTCC	8	18	5	3
tsrna-18965	TAGGATTCCTGGTTTTCACCCAGGCGGCCCGGGTTCGACTC	13	21	9	5
tsrna-18964	TAGGATTCCTGGTTTTCACCCA	8	5	3	1
tsrna-18963	TAGGATTCCTGGTTTTCACCC	8	11	5	1
tsrna-18962	TAGGATTCCTGGTTTTC	1	0	3	0
tsrna-18961	TAGGATGTGGTGTGATAGG	0	0	0	1
tsrna-18960	TAGGATGGGGTGTGATAGGTGGCACGGAGAATTTTGGATTC	55	51	74	36
tsrna-18959	TAGGATGGGGTGTGATAGGTGGCACGGAGAATTTTGG	56	45	72	48
tsrna-18958	TAGGATGGGGTGTGATAGGTGGCACGGAGAATTTTG	55	37	66	34
tsrna-18957	TAGGATGGGGTGTGATAGGTGGCACGGAGAATTTT	36	43	46	27
tsrna-18956	TAGGATGGGGTGTGATAGGTGGCACGGAGAATTT	33	49	44	38
tsrna-18955	TAGGATGGGGTGTGATAGGTGGCACGGAGAATT	38	33	40	17
tsrna-18954	TAGGATGGGGTGTGATAGGTGGCACGGAGAAT	25	30	21	18

tsrna-18953	TAGGATGGGGTGTGATAGGTGGCACGGAGAA	21	31	24	15
tsrna-18952	TAGGATGGGGTGTGATAGGTGGCACGGAGA	21	37	27	24
tsrna-18951	TAGGATGGGGTGTGATAGGTGGCACGGAG	7	17	8	17
tsrna-18950	TAGGATGGGGTGTGATAGGTGGCACGGA	10	10	7	13
tsrna-18949	TAGGATGGGGTGTGATAGGTGGCACGG	5	11	9	5
tsrna-18948	TAGGATGGGGTGTGATAGGTGGCACG	9	9	1	7
tsrna-18947	TAGGATGGGGTGTGATAGGTGGCAC	3	2	5	2
tsrna-18946	TAGGATGGGGTGTGATAGGTGGCA	3	5	1	1
tsrna-18945	TAGGATGGGGTGTGATAGGTGGC	0	8	1	1
tsrna-18944	TAGGATGGGGTGTGATAGGTGG	3	4	0	0
tsrna-18943	TAGGATGGGGTGTGATAGGTG	4	5	1	0
tsrna-18942	TAGGATGGGGTGTGATAGGT	1	2	0	0
tsrna-18941	TAGGATGGGGTGTGATAGG	0	3	0	1
tsrna-18940	TAGGATGGGGTGTGATAG	3	2	0	1
tsrna-18939	TAGGATGGGGTGTGATA	1	2	0	1
tsrna-18938	TAGGATGGGGTGTGAT	1	2	0	1
tsrna-18937	TAGGAGCTTAAACCCCCTTATTTCTACCA	2	4	2	0
tsrna-18936	TAGGAGCTTAAACCCCCTTATTTCTACC	2	2	0	0
tsrna-18932	TAGGAGATTTCAACTTAACTTGACCGCTCTGACCA	103	43	87	20
tsrna-18931	TAGGAGATTTCAACTTAACTTGACCGCTCTGACC	17	7	5	3
tsrna-18930	TAGGAGATTTCAACTTAACTTGACCGCTCTGAC	2	1	1	0
tsrna-18929	TAGGAGATTTCAACTTAACTTGACCGCTCT	4	0	0	0
tsrna-18928	TAGGAGATTTCAACTTAACTTGACCGCTC	2	0	2	0
tsrna-18927	TAGGAGATTTCAACTTAACTTGACCGCT	1	0	0	0
tsrna-18926	TAGGAGATTTCAACTTAACTTGACCGC	1	0	0	0
tsrna-18925	TAGGAGATTTCAACTTAACTTGACCG	0	0	0	0
tsrna-18924	TAGGAGATTTCAACTTAACTTGACC	0	0	0	0
tsrna-18923	TAGGAGATTTCAACTTAACTTGAC	1	1	0	0
tsrna-18922	TAGGAGATTTCAACTTAACTTGA	0	0	1	0
tsrna-18917	TAGCTTTGGGTGCTAATGGTGGAGTTAAAGACTTTTTCTCTGA	13	25	8	0
tsrna-18916	TAGCTTTGGGTGCTAATGGTGGAGTTAAAGACTTTT	2	11	2	0
tsrna-18915	TAGCTTTGGGTGCTAATGGTGGAGTTAAAG	2	5	0	0
tsrna-18914	TAGCTTTGGGTGCTAATGGTGGAGTTAAA	0	8	2	0
tsrna-18913	TAGCTTTGGGTGCTAATGGTGGAGTTAA	5	3	0	0
tsrna-18912	TAGCTTTGGGTGCTAATGGTGGAGTTA	3	3	1	1
tsrna-18911	TAGCTTTGGGTGCTAATGGTGGAGTT	1	1	0	0
tsrna-18910	TAGCTTTGGGTGCTAATGGTGGAGT	0	1	0	0
tsrna-18909	TAGCTTTGGGTGCTAATGGTGGAG	0	2	0	0
tsrna-18908	TAGCTTTGGGTGCTAATGGTGGA	0	1	0	0
tsrna-18903	TAGCTTAGCGGTAGAGCATTTGACTG	0	0	0	0
tsrna-18901	TAGCTGTTAACTAAGTGTTTGTGGGTTTAAGTCCCATTGGTCT.	9	1	9	4
tsrna-18900	TAGCTGTTAACTAAGTGTTTGTGGGTTTAAGTCCCATTGGTCT.	5	2	9	2
tsrna-18899	TAGCTGTTAACTAAGTGTTTGTGGGTTTAAGTCCCA	3	1	1	1
tsrna-18898	TAGCTGTTAACTAAGTGTTTGTGGGTTTA	1	2	0	1
tsrna-18897	TAGCTGTTAACTAAGTGTTTGTGGGTTT	1	3	0	0
tsrna-18896	TAGCTGTTAACTAAGTGTTTGTGG	0	0	0	0
tsrna-18893	TAGCTGCCTTCCAAGCAGTTGACCCGG	3	3	1	0
tsrna-18892	TAGCTGCCTTCCAAGCAGTTGACCCG	0	3	1	0
tsrna-18891	TAGCTGCCTTCCAAGCAGTTGACCC	0	1	0	2
tsrna-18884	TAGCTCAGTGGTAGAGCATTTGACTGCA	5	1	3	0
tsrna-18883	TAGCTCAGTGGTAGAGCATTTGACTGC	2	1	1	1
tsrna-18882	TAGCTCAGTGGTAGAGCATTTGACTG	2	2	0	0
tsrna-18881	TAGCTCAGTGGTAGAGCATTTGACTA	0	3	0	0
tsrna-18880	TAGCTCAGTGGTAGAGCATTTGACT	1	4	1	0
tsrna-18879	TAGCTCAGTGGTAGAGCATTTGAC	1	0	0	0
tsrna-18878	TAGCTCAGTGGTAGAGCATTTGA	1	0	0	0
tsrna-18877	TAGCTCAGTGGTAGAGCATTTG	3	1	0	0
tsrna-18876	TAGCTCAGTGGTAGAGCATTT	1	1	0	0
tsrna-18874	TAGCTCAGTGGGTAGAGCATTTGACTGC	3	4	0	1
tsrna-18873	TAGCTCAGTGGGTAGAGCATTTGACTG	0	4	0	0
tsrna-18872	TAGCTCAGTGGGTAGAGCATTTGACT	0	0	0	0
tsrna-18871	TAGCTCAGTCGGTAGAGCATGGGACTCTT	10	20	5	2
tsrna-18870	TAGCTCAGTCGGTAGAGCATGGGACTCT	20	26	8	3
tsrna-18869	TAGCTCAGTCGGTAGAGCATGGGACTC	13	20	6	3

tsrna-18868	TAGCTCAGTCGGTAGAGCATGGGACT	16	15	11	2
tsrna-18867	TAGCTCAGTCGGTAGAGCATGGGAC	6	20	5	3
tsrna-18866	TAGCTCAGTCGGTAGAGCATGGGA	9	16	4	1
tsrna-18865	TAGCTCAGTCGGTAGAGCATGGG	2	1	1	1
tsrna-18864	TAGCTCAGTCGGTAGAGCATGG	1	2	0	0
tsrna-18863	TAGCTCAGTCGGTAGAGCATGAGACTCTT	21	31	6	6
tsrna-18862	TAGCTCAGTCGGTAGAGCATGAGACTCT	16	33	10	1
tsrna-18861	TAGCTCAGTCGGTAGAGCATGAGACT	12	12	3	5
tsrna-18860	TAGCTCAGTCGGTAGAGCATGAGAC	8	24	5	2
tsrna-18859	TAGCTCAGTCGGTAGAGCATGAGA	2	9	2	2
tsrna-18858	TAGCTCAGTCGGTAGAGCATGAG	2	2	3	0
tsrna-18857	TAGCTCAGTCGGTAGAGCATGA	2	0	0	0
tsrna-18856	TAGCTCAGTCGGTAGAGCATG	1	0	2	0
tsrna-18855	TAGCTCAGTCGGTAGAGCATCAGACTTTT	9	12	18	5
tsrna-18854	TAGCTCAGTCGGTAGAGCATCAGACTTT	16	5	12	3
tsrna-18853	TAGCTCAGTCGGTAGAGCATCAGACT	13	9	10	2
tsrna-18852	TAGCTCAGTCGGTAGAGCATCAGAC	16	14	5	3
tsrna-18851	TAGCTCAGTCGGTAGAGCATCAGA	5	6	3	2
tsrna-18850	TAGCTCAGTCGGTAGAGCATCAG	2	1	4	1
tsrna-18849	TAGCTCAGTCGGTAGAGCATC	4	3	3	0
tsrna-18844	TAGCTCAGGTGGTAGAGCATTTGACTG	0	0	0	0
tsrna-18842	TAGCTCAGGGGTTAGAGCACTGGTCT	3	5	4	8
tsrna-18841	TAGCTCAGGGGTAGAGCATTTGACTGC	2	3	1	3
tsrna-18840	TAGCTCAGGGGTAGAGCATTTGACTG	1	2	0	1
tsrna-18839	TAGCTCAGGGGTAGAGCATTTGACT	0	0	0	0
tsrna-18837	TAGCTCAGGGGTAGAGCATTTGA	0	1	0	0
tsrna-18832	TAGCGTGGCCGAGTGGTCTAAGG	0	1	0	0
tsrna-18831	TAGCGTGGCCGAGTGGTCTAAG	0	1	0	0
tsrna-18830	TAGCGTGGCCGAGCGGTCTAAGGCGCTGG	0	1	0	0
tsrna-18827	TAGCGTGGCCGAGCGGTCTAAGG	1	2	0	0
tsrna-18825	TAGCGTGGCCGAGCGGTCTAA	0	1	0	0
tsrna-18824	TAGCGTGGCCGAGCGGTCTA	1	0	0	0
tsrna-18821	TAGCGTGGCCGAGCGG	0	1	0	0
tsrna-18817	TAGCGGTTAGGATTCCTGGTTTTCACCCAG	16	17	8	7
tsrna-18816	TAGCGGTTAGGATTCCTGGTTTTCACC	12	13	9	4
tsrna-18815	TAGCGGTTAGGATTCCTGGTTTTCAC	9	5	5	2
tsrna-18814	TAGCGGTTAGGATTCCTGGTTTTCA	13	8	5	3
tsrna-18813	TAGCGGTTAGGATTCCTGGTTTTC	2	6	4	4
tsrna-18812	TAGCGGTTAGGATTCCTGGTTTT	6	0	2	0
tsrna-18811	TAGCGGTTAGGATTCCTGGTTT	5	5	1	1
tsrna-18810	TAGCGGTTAGGATTCCTGGTT	1	5	1	1
tsrna-18809	TAGCGGTTAGGATTCCTGGT	1	3	0	2
tsrna-18808	TAGCGGTTAGGATTCCTGG	2	2	1	1
tsrna-18807	TAGCGGTTAGGATTCCTG	0	1	1	0
tsrna-18804	TAGCGGTAGAGCATTTGACTGC	0	0	0	0
tsrna-18803	TAGCGGTAGAGCATTTGACTG	1	2	0	0
tsrna-18802	TAGCGGTAGAGCATTTGACT	0	1	0	0
tsrna-18801	TAGCGGGATCGATGCCCGCATTCTCCACC	0	0	0	0
tsrna-18800	TAGCGGGATCGATGCCCGCATCCTCCACC	0	1	0	0
tsrna-18798	TAGCGCGTTCGGCTGTTAACCGA	2	16	3	0
tsrna-18797	TAGCGCGTTCGGCTGTTAACCG	7	16	3	0
tsrna-18796	TAGCGCGTTCGGCTGTTAACCA	9	38	11	0
tsrna-18795	TAGCGCGTTCGGCTGTTAACC	1	2	0	1
tsrna-18793	TAGCGCGTTCGGCTGTTAA	0	0	0	0
tsrna-18791	TAGCGCGTTCGGCTGTT	0	0	0	0
tsrna-18788	TAGCGCGTCTGACTCC	0	0	0	1
tsrna-18787	TAGCGCATTGGACTTCTAATTCAAAGGTTGTGGGT	44	87	28	17
tsrna-18786	TAGCGCATTGGACTTCTAATTCAAAGGTTCCGGG	3	3	2	1
tsrna-18785	TAGCATTAACCTTTTAAGTTAAAGATTAAGAGAACC	0	0	2	0
tsrna-18780	TAGCACTCTGGACTTTGAATCCAGCGATCCGAGTT	3	8	1	0
tsrna-18779	TAGCACTCTGGACTTTGAATCCAGC	3	5	1	1
tsrna-18778	TAGCACTCTGGACTTTGAATCCA	1	5	3	0
tsrna-18777	TAGCACTCTGGACTTTGAATCC	3	6	1	1
tsrna-18776	TAGCACTCTGGACTTTGAATC	2	2	1	1

tsrna-18770	TAGCACTCTGGACTCTGAATCCAGCGATCCGAGTTCA	0	2	3	0
tsrna-18769	TAGCACTCTGGACTCTGAATCCAGCGATCCGAGTTC	1	1	0	0
tsrna-18768	TAGCACTCTGGACTCTGAATCCAGCGATCCGAGTT	2	4	1	0
tsrna-18767	TAGCACTCTGGACTCTGAATCCAGCGATCCGAGT	0	7	0	0
tsrna-18766	TAGCACTCTGGACTCTGAATCCAGCGATCCGAG	2	5	0	0
tsrna-18765	TAGCACTCTGGACTCTGAATCCAGCGATCC	1	4	1	1
tsrna-18764	TAGCACTCTGGACTCTGAATCCAGCGATC	2	2	0	0
tsrna-18763	TAGCACTCTGGACTCTGAATCCAGCGAT	0	7	1	0
tsrna-18762	TAGCACTCTGGACTCTGAATCCAGCGA	3	4	1	0
tsrna-18761	TAGCACTCTGGACTCTGAATCCAGCG	1	0	1	0
tsrna-18760	TAGCACTCTGGACTCTGAATCCAGC	3	3	0	1
tsrna-18759	TAGCACTCTGGACTCTGAATCCAG	0	5	2	0
tsrna-18758	TAGCACTCTGGACTCTGAATCCA	0	1	0	0
tsrna-18757	TAGCACTCTGGACTCTGAATCC	0	2	0	0
tsrna-18756	TAGCACTCTGGACTCTGAATC	0	1	0	0
tsrna-18750	TAGATTGAAGCCAGTTGATTAGGGTGCTTAGCTGTTAACT	42	4	40	1
tsrna-18749	TAGATTGAAGCCAGTTGATTAGGGTGCTTAGCTGTT	18	2	30	2
tsrna-18748	TAGATTGAAGCCAGTTGATTAGGGTGCTTAGCT	5	2	8	0
tsrna-18747	TAGATTGAAGCCAGTTGATTAGGGTGCTTA	1	0	3	0
tsrna-18746	TAGATTGAAGCCAGTTGATTAGGGTGCTT	1	1	2	1
tsrna-18745	TAGATTGAAGCCAGTTGATTAGGGTGCT	1	0	1	0
tsrna-18744	TAGATTGAAGCCAGTTGATTAGGGTGC	0	1	1	0
tsrna-18743	TAGATTGAAGCCAGTTGATTAGGGTG	0	1	0	0
tsrna-18742	TAGATTGAAGCCAGTTGATTAGGGT	0	1	2	0
tsrna-18741	TAGATTGAAGCCAGTTGATTAGGG	0	2	0	0
tsrna-18740	TAGATTGAAGCCAGTTGATTAGG	0	0	0	0
tsrna-18739	TAGATTGAAGCCAGTTGATTAG	1	1	2	0
tsrna-18738	TAGATTGAAGCCAGTTGATTA	0	0	2	0
tsrna-18737	TAGATTGAAGCCAGTTGATT	0	1	0	1
tsrna-18736	TAGATTGAAGCCAGTTGAT	2	1	0	1
tsrna-18735	TAGATTGAAGCCAGTTGA	1	0	0	1
tsrna-18734	TAGATTGAAGCCAGTTG	0	1	0	1
tsrna-18733	TAGATTGAAGCCAGTT	1	0	0	0
tsrna-18721	TAGAGCATGGGACTCTTAATCCCAGGGTCGTGGGTT	2	5	0	1
tsrna-18720	TAGAGCATGGGACTCTTAATCCCAGGGTCGTGGGT	2	0	2	1
tsrna-18719	TAGAGCATGGGACTCTTAATCCCAGGGTCGTGG	0	3	0	0
tsrna-18718	TAGAGCATGGGACTCTTAATCCCAGGGTCG	0	1	1	0
tsrna-18717	TAGAGCATGGGACTCTTAATCCC	1	3	1	0
tsrna-18716	TAGAGCATGGGACTCTTAATCC	2	1	0	1
tsrna-18715	TAGAGCATGGGACTCTTAATC	0	0	0	0
tsrna-18713	TAGAGCATGAGACTCTTAATCTCAGGGTCGTGGGTT	6	22	6	7
tsrna-18712	TAGAGCATGAGACTCTTAATCTCAGGGTCGTGGGT	9	20	1	7
tsrna-18711	TAGAGCATGAGACTCTTAATCT	2	6	0	2
tsrna-18710	TAGAGCATCAGACTTTTAATCTGAGGGTCCAGGGT	15	48	12	5
tsrna-18708	TAGACGGGCTCACATCACCCCATAAACACCA	18	6	21	9
tsrna-18707	TAGACGGGCTCACATCACCCCATAAACACC	7	2	11	1
tsrna-18706	TAGACGGGCTCACATCACCCCATAAACA	1	1	3	0
tsrna-18705	TAGACGGGCTCACATCACCCCATA	1	1	3	1
tsrna-18704	TAGACGGGCTCACATCACCCCAT	1	0	2	0
tsrna-18703	TAGACGGGCTCACATCACCCCA	0	0	1	0
tsrna-18702	TAGACGGGCTCACATCACCCC	2	2	2	0
tsrna-18701	TAGACGGGCTCACATCACCC	0	0	0	1
tsrna-18700	TAGACGGGCTCACATCACC	0	0	5	0
tsrna-18699	TAGACGGGCTCACATCAC	2	2	1	0
tsrna-18698	TAGACGGGCTCACATCA	1	0	2	0
tsrna-18697	TAGACGGGCTCACATC	1	0	1	0
tsrna-18696	TAGAATTCTCGCCTGCCACGCGGGAGGCCC	29	37	27	9
tsrna-18695	TAGAATTCTCGCCTGCCACGCGGG	51	43	15	18
tsrna-18694	TAGAATTCTCGCCTGCCACGCGG	28	40	19	12
tsrna-18693	TAGAATTCTCGCCTGCCACGCG	41	49	21	13
tsrna-18692	TAGAATTCTCGCCTGCCACGC	36	29	14	7
tsrna-18691	TAGAATTCTCGCCTGCCACG	33	32	21	6
tsrna-18690	TAGAATTCTCGCCTGCCAC	3	8	0	3
tsrna-18689	TAGAATTCTCGCCTGCCA	2	2	1	1

tsrna-18688	TAGAATTCTCGCCTGCC	3	1	1	1
tsrna-18687	TAGAATTCTCGCCTGC	1	1	2	0
tsrna-18686	TAGAATTCTCGCCTCCCACGC	2	0	0	1
tsrna-18685	TAGAATTCTCGCCTCCCACG	2	1	1	0
tsrna-18684	TAGAATTCTCGCCTCCC	0	0	1	1
tsrna-18683	TAGAATTCTCGCCTCC	1	1	1	0
tsrna-18682	TAGAATCTTAGCTTTGGGTGCTAATGGTGGAGTTAA	1	2	1	1
tsrna-18681	TAGAATCTTAGCTTTGGGTGCTAATGGTGG	1	1	0	0
tsrna-18679	TAGAATCTTAGCTTTGGGTGC	0	0	0	0
tsrna-18677	TAGAATCTTAGCTTTGGGT	1	0	1	0
tsrna-18676	TAGAATCTTAGCTTTGG	1	0	0	0
tsrna-18675	TAGAATCTTAGCTTTG	0	0	0	0
tsrna-18674	TAGAAATCCATTGGGGTTTC	0	7	0	0
tsrna-18673	TACTTTGATAGAGTAAATAATAGGAGCT	0	0	1	0
tsrna-18672	TACTTATAATGCCGAGGTTGTG	2	4	3	1
tsrna-18669	TACTCTGCGTTGTGGCCGCAGC	0	2	0	0
tsrna-18668	TACTCTGCGTTGTGGCCGCAG	0	0	1	0
tsrna-18661	TACTCGTTAGTATAGTGGT	0	0	0	1
tsrna-18658	TACGACCCCTTATTTACCCCA	0	0	0	0
tsrna-18653	TACCTCCTCAAAGCAATACACTGAAAATGT	6	1	5	2
tsrna-18652	TACCTCCTCAAAGCAATACACTGAA	2	5	5	2
tsrna-18651	TACCTCCTCAAAGCAATACACTGA	1	3	1	0
tsrna-18650	TACCTCCTCAAAGCAATACACTG	0	3	0	1
tsrna-18649	TACCTCCTCAAAGCAATACACT	3	0	1	0
tsrna-18648	TACCTCCTCAAAGCAATACAC	0	0	0	0
tsrna-18647	TACCTCCTCAAAGCAATACA	0	0	0	0
tsrna-18639	TACCGTTAACTTCCAATTA	0	0	1	0
tsrna-18638	TACCCTTCCCGTACTACCA	0	0	0	0
tsrna-18635	TACCCCGAAAATGTTGGTTATACCCTTCCCGTACTA	0	0	3	0
tsrna-18634	TACCCCGAAAATGTTGGTTATACCCTTCCC	1	0	0	0
tsrna-18630	TACAGTCAGAGGTTCA	1	0	0	0
tsrna-18629	TACAGACCAAGAGCCTTCAAAG	6	0	0	1
tsrna-18628	TACAGACCAAGAGCCTTCA	0	2	0	0
tsrna-18627	TACAGACCAAGAGCCTTC	0	1	0	0
tsrna-18626	TACACTTAGGAGATTTCAACTTAACTTGACCGCTCTGACCA	92	51	79	14
tsrna-18625	TACACTTAGGAGATTTCAACTTAACTTGACCGCTCTGACC	17	10	14	1
tsrna-18624	TACACTTAGGAGATTTCAACTTAACTTGACCGCTCTGA	5	4	1	0
tsrna-18623	TACACTTAGGAGATTTCAACTTAACTTGACCGCTCTG	0	0	0	1
tsrna-18622	TACACTTAGGAGATTTCAACTTAACTTGACCGCTCT	1	2	0	0
tsrna-18621	TACACTTAGGAGATTTCAACTTAACTTGACCGCTC	0	0	3	1
tsrna-18620	TACACTTAGGAGATTTCAACTTAACTTGACCGCT	1	0	1	0
tsrna-18619	TACACTTAGGAGATTTCAACTTAACTTGACC	0	0	0	0
tsrna-18618	TACACTTAGGAGATTTCAACTTAACTTGAC	1	0	2	0
tsrna-18617	TACACTTAGGAGATTTCAACTTAACTTGA	0	0	0	0
tsrna-18615	TACACTTAGGAGATTTCAACTTAACT	0	0	2	0
tsrna-18607	TACACTGAAAATGTTTAGACGGGCTCAC	30	17	8	2
tsrna-18606	TACACTGAAAATGTTTAGAC	0	0	0	0
tsrna-18604	TACACCAGTCTTGTAAACCGG	2	1	0	0
tsrna-18603	TACAACGATGGTTTTTCATATCATTGGTCGTGGTTGTAGTC	2	1	3	3
tsrna-18602	TACAACGATGGTTTTTCATATCATTGGTCGTGGTTGTAGT	1	2	1	0
tsrna-18601	TACAACGATGGTTTTTCATATCATTGGTCG	1	0	0	0
tsrna-18597	TAATTCAAAGGTTGTGGGTTCGAGTCCCACCAGAGTCGCCA	2	22	1	1
tsrna-18596	TAATTCAAAGGTTGTGGGTTCGAATCCCACCAGAGTCGCCA	10	19	3	2
tsrna-18594	TAATTCAAAGGTTCCGGGTTCGAGTCCCGGCGGAGTCGCCA	1	4	0	0
tsrna-18593	TAATTCAAAGGTTCCGGGTTCGAGTCCCGGCGGAGTCGCC	1	3	0	0
tsrna-18592	TAATTCAAAGGTTCCGGGTTCGAGTCCCGG	0	1	0	0
tsrna-18591	TAATTCAAAGGTTCCGGGTT	0	1	0	0
tsrna-18590	TAATTAAAGTGGCTGATTTGCGTTCA	0	1	0	0
tsrna-18589	TAATTAAAGTGGCTGATTTGCGTTC	0	0	0	0
tsrna-18588	TAATTAAAGTGGCTGATTTGCG	0	0	0	0
tsrna-18587	TAATTAAAGTGGCTGATTTGC	0	0	0	0
tsrna-18586	TAATTAAAGTGGCTGATTTG	0	1	0	0
tsrna-18584	TAATGGTTAGCACTCTGGGCT	0	0	0	0
tsrna-18583	TAATGGTTAGCACTCTGGGC	1	0	0	0

tsrna-18582	TAATGGTTAGCACTCTGGACTTTGAATCC	3	8	2	1
tsrna-18581	TAATGGTTAGCACTCTGGACTTTGAATC	4	1	1	0
tsrna-18580	TAATGGTTAGCACTCTGGACTTTGAAT	0	0	0	1
tsrna-18579	TAATGGTTAGCACTCTGGACTTTGAA	1	1	0	0
tsrna-18578	TAATGGTTAGCACTCTGGACTTTGA	0	0	0	1
tsrna-18577	TAATGGTTAGCACTCTGGACTTTG	0	0	0	0
tsrna-18576	TAATGGTTAGCACTCTGGACTTT	1	0	0	0
tsrna-18574	TAATGGTTAGCACTCTGGACTCTGAATCCAGCGATCCGAGTT(	4	6	2	2
tsrna-18573	TAATGGTTAGCACTCTGGACTCTGAATCCAGCGATC	8	6	3	2
tsrna-18572	TAATGGTTAGCACTCTGGACTCTGAATCC	0	5	1	3
tsrna-18571	TAATGGTTAGCACTCTGGACTCTGAATC	1	1	3	0
tsrna-18570	TAATGGTTAGCACTCTGGACTCTGAAT	0	1	0	0
tsrna-18569	TAATGGTTAGCACTCTGGACTCTGAA	2	0	0	0
tsrna-18567	TAATGGTTAGCACTCTGGACTCTG	0	0	0	1
tsrna-18566	TAATGGTTAGCACTCTGGACTCT	0	1	0	1
tsrna-18565	TAATGGTTAGCACTCTGGACTC	1	1	0	0
tsrna-18564	TAATGGTTAGCACTCTGGACT	1	0	0	0
tsrna-18562	TAATGGTTAGCACTCTGGA	0	0	0	0
tsrna-18558	TAATGGTGGAGTTAAAGACTTTTTCTCTGACCA	29	63	20	21
tsrna-18557	TAATGGTGGAGTTAAAGACTTTTTCTCTGACC	6	24	4	2
tsrna-18556	TAATGGTGGAGTTAAAGACTTTTTCTCTGAC	5	17	3	1
tsrna-18555	TAATGGTGGAGTTAAAGACTTTTTCTCTGA	10	13	0	0
tsrna-18554	TAATGGTGGAGTTAAAGACTTTTTCTCTG	8	19	3	1
tsrna-18553	TAATGGTGGAGTTAAAGACTTTTTC	1	13	2	2
tsrna-18552	TAATGGTGGAGTTAAAGACTTTTT	0	10	1	0
tsrna-18551	TAATGGTGGAGTTAAAGACTTTT	3	7	4	0
tsrna-18550	TAATGGTGGAGTTAAAGACTTT	2	7	4	0
tsrna-18549	TAATGGTGGAGTTAAAGACTT	2	5	2	0
tsrna-18548	TAATGGTGGAGTTAAAGACT	2	2	1	0
tsrna-18547	TAATGGTGGAGTTAAAGAC	0	3	1	0
tsrna-18546	TAATGGTGGAGTTAAAGA	3	2	1	0
tsrna-18545	TAATGGTGGAGTTAAAG	0	2	1	1
tsrna-18544	TAATGGTGGAGTTAAA	0	1	0	0
tsrna-18543	TAATGGTGAGCACTTTGGACTCTGA	0	2	0	0
tsrna-18542	TAATGGTGAGCACTTTGGACTCTG	1	1	0	0
tsrna-18540	TAATGGTGAGCACTCTGGACTCTGAATCC	2	5	0	1
tsrna-18537	TAATGGTGAGCACTCTGGACTCT	0	0	0	0
tsrna-18534	TAATGGTGAGCACTCTGGAC	0	0	1	0
tsrna-18530	TAATGGTCAGCACTCTGGACTCTGAA	0	0	0	0
tsrna-18522	TAATGGTAAGCACTCTGGACTCTGAATCC	0	1	1	0
tsrna-18512	TAATGGATAAGGCGTCTGATTCC	0	3	0	0
tsrna-18510	TAATGGATAAGGCGTCTGACTTCGGATC	0	2	1	0
tsrna-18509	TAATGGATAAGGCGTCTGACTTCGGAT	1	1	0	1
tsrna-18508	TAATGGATAAGGCGTCTGACTTCGGA	0	0	0	1
tsrna-18507	TAATGGATAAGGCGTCTGACTTCG	0	1	1	0
tsrna-18506	TAATGGATAAGGCGTCTGACTTC	1	0	0	1
tsrna-18504	TAATGGATAAGGCGTCTGACT	0	0	1	0
tsrna-18503	TAATGGATAAGGCGTCTGAC	1	0	0	0
tsrna-18501	TAATGGATAAGGCGTCTG	0	1	0	0
tsrna-18500	TAATGGATAAGGCGTCT	1	0	0	0
tsrna-18499	TAATGGATAAGGCATTGG	3	2	2	0
tsrna-18498	TAATGGATAAGGCATTG	2	1	1	0
tsrna-18497	TAATGGATAAGGCATT	0	0	1	0
tsrna-18496	TAATGGATAAGGCATCAGCCTCC	2	2	3	1
tsrna-18495	TAATGGATAAGGCATCAGCCTC	8	3	5	0
tsrna-18494	TAATGGATAAGGCATCAGCCT	1	2	2	0
tsrna-18493	TAATGGATAAGGCATCAGCC	4	1	2	1
tsrna-18492	TAATGGATAAGGCATCAGC	6	2	1	1
tsrna-18491	TAATGGATAAGGCATCAG	2	2	1	0
tsrna-18490	TAATGGATAAGGCATCA	6	2	2	1
tsrna-18489	TAATGGATAAGGCATC	0	1	3	2
tsrna-18488	TTCACCGCCGCGGCCCGGGTT	0	0	0	0
tsrna-18483	TTCACCCAGGCGGCCCGGGTTCGACTCCCGGTGTGGGAACC.	75	338	37	23
tsrna-18478	TTCAATTCTCGCTGGGGCCTCCA	38	47	23	19

tsrna-18476	TTCAATTCCCCGACGGGGAGCCA	0	2	0	0
tsrna-18474	TTCAATGGTAGAATTCTCGCCT	1	5	1	0
tsrna-18473	TTCAATCCCGGGTTTCGGCACCA	37	93	13	12
tsrna-18472	TTCAATCCCCGGCATCTCCACCA	127	133	59	49
tsrna-18469	TTCAATCCCCGGCACCTCCACCA	251	297	115	37
tsrna-18468	TTCAATCCCCGGCACCTCCACC	7	22	8	5
tsrna-18467	TTCAATCCCCGGCACCTCCAC	0	0	0	1
tsrna-18465	TTCAATCCCCAGCACCTCCACCA	3	13	2	6
tsrna-18464	TTCAAGTCTCGGTGGAACCTCCA	68	106	29	18
tsrna-18463	TTCAAGTCCCTGTTCGGGCGCCA	10	12	3	3
tsrna-18457	TTCAAGTCCCTGTTCAGGCGCCA	1	3	0	0
tsrna-18455	TTCAAGTCCCTGTCCAGGCGCCA	0	0	0	0
tsrna-18454	TTCAAGTCACGTCGGGGTCACCA	5	7	1	1
tsrna-18453	TTCAAGTCACGTCGGGGTCACC	0	0	2	0
tsrna-18452	TTCAAGTCACGTCGGGGTCAC	1	0	0	0
tsrna-18446	TTCAAGCCTCACCTGGAGCACCA	0	1	1	0
tsrna-18445	TTCAACTTAACTTGACCGCTCTGACCA	18	21	33	5
tsrna-18444	TTCAACTTAACTTGACCGCTCTGACC	0	4	1	0
tsrna-18443	TTCAACTTAACTTGACCGCTCTGAC	0	0	0	0
tsrna-18442	TTCAACTTAACTTGACCGCTCTGA	0	0	0	1
tsrna-18441	TTCAACTTAACTTGACCGCTCTG	1	0	0	0
tsrna-18440	TTCAACTTAACTTGACCGCTCT	0	0	0	0
tsrna-18439	TTCAACTTAACTTGACCGCTC	0	0	0	0
tsrna-18437	TTCAACTTAACTTGACCGC	0	0	0	0
tsrna-18433	TTCAAATCTGGGTGCCCCCTCCA	0	0	0	0
tsrna-18432	TTCAAATCTCGGTGGGACCTCCA	71	105	32	19
tsrna-18431	TTCAAATCTCGGTGGGACCTCC	3	9	2	2
tsrna-18429	TTCAAATCTCGGTGGGACCT	0	0	0	0
tsrna-18425	TTCAAATCTCGGTGGAACCTCCA	159	190	57	29
tsrna-18424	TTCAAATCTCGGTGGAACCTCC	4	7	3	0
tsrna-18423	TTCAAATCTCGGTGGAACCTC	0	2	1	0
tsrna-18422	TTCAAATCTCGGTGGAACCT	0	3	1	2
tsrna-18421	TTCAAATCTCGGTGGAACC	0	2	0	0
tsrna-18418	TTCAAATCTCGCTGGGGCCTCCA	31	53	25	15
tsrna-18417	TTCAAATCTCGCTGGGGCCTCC	4	6	2	0
tsrna-18416	TTCAAATCTCGCTGGGGCCTC	0	1	0	0
tsrna-18414	TTCAAATCCGGGTGCCCCCTCCA	170	162	59	24
tsrna-18413	TTCAAATCCGGGTGCCCCCTCC	4	7	1	2
tsrna-18412	TTCAAATCCGGGTGCCCCCTC	2	1	0	0
tsrna-18409	TTCAAATCCCGGACGAGCCCCCA	147	156	59	38
tsrna-18408	TTCAAATCCCGGACGAGCCCCC	7	7	3	0
tsrna-18407	TTCAAATCCCGGACGAGCCCC	1	2	1	0
tsrna-18406	TTCAAATCCCGGACGAGCCC	2	0	1	0
tsrna-18405	TTCAAATCCCGGACGAGCC	0	1	0	0
tsrna-18401	TTCAAATCCAGGTGCCCCCTCCA	1	2	0	1
tsrna-18400	TTCAAATCACGTCGGGGTCACCA	4	8	13	1
tsrna-18399	TTCAAATCACGTCGGGGTCACC	0	1	0	1
tsrna-18398	TTCAAATCACGTCGGGGTCAC	0	0	0	0
tsrna-18393	TTCAAAGGTTGTGGGTTCGAGTCCCACCAGAGTCGCCA	1	9	0	0
tsrna-18392	TTCAAAGGTTGTGGGTTCGAGTCCCACCAGAGTCGCC	1	3	0	1
tsrna-18391	TTCAAAGGTTGTGGGTTCGAGTCCCACCAGAGTCGC	1	2	0	0
tsrna-18390	TTCAAAGGTTGTGGGTTCGAGTCCCACCAG	1	0	0	0
tsrna-18389	TTCAAAGGTTGTGGGTTCGAGTCCCACCA	0	1	0	0
tsrna-18388	TTCAAAGGTTGTGGGTTCGAATCCCACCAGAGTCGCCA	3	11	0	1
tsrna-18387	TTCAAAGGTTGTGGGTTCGAATCCCACCAGAGTCGCC	1	6	1	1
tsrna-18385	TTCAAAGGTTGTGGGTTCGAATCCCACCA	2	3	0	0
tsrna-18384	TTCAAAGGTTCCGGGTTCGAGTCCCGGCGGAGTCGCCA	0	7	0	0
tsrna-18383	TTCAAAGGTTCCGGGTTCGAGTCCCGGCGGAGTCGCC	0	0	0	0
tsrna-18381	TTCAAAGGTTCCGGGTTCGAGTCCCGGCGGAGTC	0	0	0	0
tsrna-18379	TTCAAAGGTTCCGGGTTCGAGTCCCGGCG	1	0	0	0
tsrna-18378	TTCAAAGGTTCCGGGTTCG	0	0	0	0
tsrna-18373	TTCAAACCTGCCGGGGCTTCCA	0	2	1	0
tsrna-18372	TTCAAACCTGCCGGGGCTTCC	0	0	0	1
tsrna-18365	TTATGTAGCTTACCTCC	1	0	0	0

tsrna-18356	TTATCACGTTCGCCTCACACGC	0	1	0	0
tsrna-18352	TTATCACGTCTGCTTTACACG	0	0	1	0
tsrna-18349	TTATACCCTTCCCGTACTACCA	1	3	0	0
tsrna-18348	TTATACCCTTCCCGTACTACC	0	1	0	0
tsrna-18345	TTATAATGCCGAGGTTGTGAGTTC	1	16	8	3
tsrna-18344	TTATAATGCCGAGGTTGTGAGTT	4	8	3	0
tsrna-18343	TTATAATGCCGAGGTTGTGAGT	7	9	1	1
tsrna-18342	TTATAATGCCGAGGTTGTGAG	4	9	0	0
tsrna-18341	TTATAATGCCGAGGTTGTGA	1	5	0	1
tsrna-18340	TTAGTGGTAGAATTCTCGCCT	2	7	1	1
tsrna-18339	TTAGTGGTAGAATTCTCGCC	2	4	0	0
tsrna-18338	TTAGTGGTAGAATTCTCGC	1	1	0	1
tsrna-18337	TTAGTGGTAGAATTCTCG	0	0	0	0
tsrna-18334	TTAGTATAGTGGTGAGTATCCCCG	1	0	0	1
tsrna-18333	TTAGTACTCTGCGTTGTGGCCGCAGCAACCTCGGT	17	6	6	3
tsrna-18332	TTAGTACTCTGCGTTGTGGCCGCAGCAACC	0	2	0	0
tsrna-18331	TTAGTACTCTGCGTTGTGGCCGCAGC	4	2	0	0
tsrna-18330	TTAGTACTCTGCGTTGTGGCCGCA	2	0	0	0
tsrna-18329	TTAGTACTCTGCGTTGTGGCCGC	0	0	2	0
tsrna-18328	TTAGTACTCTGCGTTGTGGCCG	1	5	1	0
tsrna-18327	TTAGTACTCTGCGTTGTGGCC	2	0	0	0
tsrna-18326	TTAGTACTCTGCGTTGTGGC	1	2	0	0
tsrna-18325	TTAGTACTCTGCGTTGTGG	0	1	0	0
tsrna-18324	TTAGTACTCTGCGTTGTG	1	2	2	0
tsrna-18323	TTAGTACTCTGCGTTGT	0	0	0	0
tsrna-18322	TTAGTACTCTGCGTTG	1	0	0	0
tsrna-18320	TTAGGTCGCTGGTTCGATTCCGGCTCGAAGGACC	12	31	6	5
tsrna-18319	TTAGGTCGCTGGTTCGATTCCGGCTCGAAG	0	1	1	0
tsrna-18318	TTAGGTCGCTGGTTCGATTCCGGC	1	0	0	0
tsrna-18316	TTAGGGTGCTTAGCTGTTAACTAAGTGTTTGTGGGT	2	1	0	1
tsrna-18315	TTAGGGTGCTTAGCTGTTAACTA	1	0	2	0
tsrna-18314	TTAGGGTGCTTAGCTGTTAACT	0	1	0	0
tsrna-18312	TTAGGGTGCGAGAGGTCCCGGGTT	0	7	2	3
tsrna-18311	TTAGGGTGCGAGAGGTCCCGGGT	0	7	1	1
tsrna-18310	TTAGGGTGCGAGAGGTCCCGG	0	4	0	0
tsrna-18306	TTAGGCTCCAGTCTCTTCGG	0	1	0	0
tsrna-18303	TTAGGCCTCTTTTTACCACCA	0	2	1	1
tsrna-18298	TTAGGCCCCAAAAATTTTGGTGCAACTCCAAATAAAAGTACC	18	122	9	0
tsrna-18297	TTAGGATTCGGCGCTCTCACCGCCGCGGCCCGGGTTC	7	15	5	2
tsrna-18296	TTAGGATTCGGCGCTCTCACCGCCGCGGCCCGGGTT	3	15	4	1
tsrna-18295	TTAGGATTCGGCGCTCTCACCGCCGCGGCCCGGG	1	7	3	3
tsrna-18294	TTAGGATTCGGCGCTCTCACCGCCGCGGCCC	3	10	3	2
tsrna-18293	TTAGGATTCGGCGCTCTCACCGCCGCGGCC	5	18	2	2
tsrna-18292	TTAGGATTCGGCGCTCTCACCGCCGCGGC	3	13	1	1
tsrna-18291	TTAGGATTCGGCGCTCTCACCGCC	6	13	1	1
tsrna-18290	TTAGGATTCGGCGCTCTCACCGC	2	4	1	0
tsrna-18289	TTAGGATTCGGCGCTCTCACCG	1	7	1	0
tsrna-18288	TTAGGATTCGGCGCTCTCACC	2	3	1	0
tsrna-18287	TTAGGATTCGGCGCTCTCAC	0	2	0	0
tsrna-18286	TTAGGATTCGGCGCTCTCA	0	2	1	0
tsrna-18285	TTAGGATTCGGCGCTCTC	0	3	0	0
tsrna-18284	TTAGGATTCGGCGCTCT	0	1	1	0
tsrna-18283	TTAGGATTCGGCGCTC	0	3	0	0
tsrna-18282	TTAGGAGATTTCAACTTAACTTGACCGCTCTGACCA	73	49	86	16
tsrna-18281	TTAGGAGATTTCAACTTAACTTGACCGCTCTGACC	8	9	12	3
tsrna-18280	TTAGGAGATTTCAACTTAACTTGACCGCTC	1	0	1	1
tsrna-18279	TTAGGAGATTTCAACTTAACTTGACCGCT	0	1	0	0
tsrna-18276	TTAGGAGATTTCAACTTAACTTGACC	0	0	0	1
tsrna-18275	TTAGGAGATTTCAACTTAACTTGAC	1	0	0	0
tsrna-18269	TTAGCTTTGGGTGCTAATGGTGGAGTTAAAGACTTTTTCTCTG	12	19	10	1
tsrna-18268	TTAGCTTTGGGTGCTAATGGTGGAGTTAA	2	3	0	0
tsrna-18267	TTAGCTTTGGGTGCTAATGGTGGAGTTA	1	6	0	0
tsrna-18266	TTAGCTTTGGGTGCTAATGGTGGAGTT	0	1	0	1
tsrna-18264	TTAGCTTTGGGTGCTAATGGTGGA	0	1	0	0

tsrna-18263	TTAGCTTTGGGTGCTAATGGTGG	1	0	0	0
tsrna-18258	TTAGCTGTTAACTAAGTGTTTGTGGGTTTAAGTCCCATTGGTC	8	5	18	0
tsrna-18257	TTAGCTGTTAACTAAGTGTTTGTGGGTTTAAGTCCC	2	2	0	0
tsrna-18254	TTAGCGGTAGAGCATTTGACTGC	1	0	0	1
tsrna-18253	TTAGCGGTAGAGCATTTGACTG	0	0	0	0
tsrna-18252	TTAGCGGTAGAGCATTTGACT	0	1	0	0
tsrna-18251	TTAGCGCGTTCGGCTGTTAACCG	3	11	1	0
tsrna-18250	TTAGCGCGTTCGGCTGTTAACC	0	2	0	1
tsrna-18244	TTAGCATTAACCTTTTAAGTTAAAGATTAAGAGAACC	0	0	0	1
tsrna-18243	TTAGCATTAACCTTTTAAGTTAAAGATTAAGAGAAC	1	1	0	0
tsrna-18238	TTAGCACTCTGGGCTTTGAATC	3	0	0	0
tsrna-18236	TTAGCACTCTGGACTTTGAATCCAGCGATCCGAGTT	3	10	3	2
tsrna-18235	TTAGCACTCTGGACTTTGAATCCAGCG	3	2	3	0
tsrna-18234	TTAGCACTCTGGACTTTGAATCCAGC	2	3	0	1
tsrna-18233	TTAGCACTCTGGACTTTGAATCCA	1	5	1	0
tsrna-18232	TTAGCACTCTGGACTTTGAATCC	2	10	0	3
tsrna-18231	TTAGCACTCTGGACTTTGAATC	1	3	2	0
tsrna-18224	TTAGCACTCTGGACTCTGAATCCAGCGATCCGAGTTCA	5	1	0	1
tsrna-18223	TTAGCACTCTGGACTCTGAATCCAGCGATCCGAGTTC	2	7	1	0
tsrna-18222	TTAGCACTCTGGACTCTGAATCCAGCGATCCGAGTT	1	3	0	0
tsrna-18221	TTAGCACTCTGGACTCTGAATCCAGCGATCCGAGT	2	2	1	0
tsrna-18220	TTAGCACTCTGGACTCTGAATCCAGCGATCCGAG	4	2	0	1
tsrna-18219	TTAGCACTCTGGACTCTGAATCCAGCGAT	2	7	0	0
tsrna-18218	TTAGCACTCTGGACTCTGAATCCAGCGA	1	4	1	0
tsrna-18217	TTAGCACTCTGGACTCTGAATCCAGCG	0	2	1	0
tsrna-18216	TTAGCACTCTGGACTCTGAATCCAGC	1	7	1	0
tsrna-18215	TTAGCACTCTGGACTCTGAATCCAG	1	5	0	0
tsrna-18214	TTAGCACTCTGGACTCTGAATCCA	1	2	1	0
tsrna-18213	TTAGCACTCTGGACTCTGAATCC	0	4	0	0
tsrna-18205	TTAGAGCCAGACTGCCTGGGTTTGA	2	0	1	0
tsrna-18204	TTAGAGCCAGACTGCCTGGGTTTG	1	2	5	1
tsrna-18203	TTAGAGCCAGACTGCCTGGGTTT	3	1	2	1
tsrna-18202	TTAGAGCCAGACTGCCTGGGTT	0	0	1	0
tsrna-18201	TTAGAGCCAGACTGCCTGGGT	1	0	0	0
tsrna-18197	TTAGACGGGCTCACATCACCCCATAAACACCA	31	14	33	20
tsrna-18196	TTAGACGGGCTCACATCACCCCATAAACACC	12	5	7	1
tsrna-18195	TTAGACGGGCTCACATCACCCCATAAACAC	6	1	10	1
tsrna-18194	TTAGACGGGCTCACATCACCCCATAAACA	7	1	6	1
tsrna-18193	TTAGACGGGCTCACATCACCCCATAAAC	2	2	9	1
tsrna-18192	TTAGACGGGCTCACATCACCCCATAAA	1	0	2	0
tsrna-18191	TTAGACGGGCTCACATCACCCCATAA	1	1	4	0
tsrna-18190	TTAGACGGGCTCACATCACCCCATA	1	1	5	0
tsrna-18189	TTAGACGGGCTCACATCACCCCAT	4	0	3	0
tsrna-18188	TTAGACGGGCTCACATCACCCCA	1	0	2	0
tsrna-18187	TTAGACGGGCTCACATCACCCC	3	0	4	0
tsrna-18186	TTAGACGGGCTCACATCACCC	0	1	2	1
tsrna-18185	TTAGACGGGCTCACATCACC	4	1	3	0
tsrna-18184	TTAGACGGGCTCACATCAC	2	0	1	0
tsrna-18183	TTAGACGGGCTCACATCA	0	1	2	0
tsrna-18182	TTAGACGGGCTCACATC	1	2	2	0
tsrna-18181	TTAGACGGGCTCACAT	0	0	3	0
tsrna-18180	TTAGAATCTTAGCTTTGGGTGCT	1	0	0	0
tsrna-18179	TTAGAATCTTAGCTTTGGGTGC	0	0	1	1
tsrna-18178	TTAGAATCTTAGCTTTGGGTG	1	0	0	0
tsrna-18177	TTAGAATCTTAGCTTTGGGT	0	0	0	1
tsrna-18176	TTAGAATCTTAGCTTTGG	0	0	0	0
tsrna-18174	TTACGACCCCTTATTTACCCCA	0	1	1	1
tsrna-18169	TTACCTCCTCAAAGCAATACACTGAAAATG	9	5	5	1
tsrna-18168	TTACCTCCTCAAAGCAATACACT	2	1	2	0
tsrna-18167	TTACCTCCTCAAAGCAATACA	1	0	0	0
tsrna-18166	TTACCTCCTCAAAGCAATAC	0	1	0	0
tsrna-18165	TTACCTCCTCAAAGCAATA	1	0	0	0
tsrna-18163	TTACAGTCAGAGGTTCAATTC	1	2	0	0
tsrna-18161	TTACAGTCAGAGGTTC	0	0	1	0

tsrna-18160	TTACACTTAGGAGATTTCAACTTAACTTGACCGCTCTGACCA	93	56	105	17
tsrna-18159	TTACACTTAGGAGATTTCAACTTAACTTGACCGCTCTGACC	20	12	15	0
tsrna-18158	TTACACTTAGGAGATTTCAACTTAACTTGACCGCTCTGAC	1	8	5	0
tsrna-18157	TTACACTTAGGAGATTTCAACTTAACTTGACCGCTCTG	2	3	3	0
tsrna-18156	TTACACTTAGGAGATTTCAACTTAACTTGACCGCT	1	2	0	1
tsrna-18155	TTACACTTAGGAGATTTCAACTTAACTTGACC	1	0	2	0
tsrna-18154	TTACACTTAGGAGATTTCAACTTAACTTGA	0	0	1	0
tsrna-18153	TTACACTTAGGAGATTTCAACTTAACTTG	0	0	0	0
tsrna-18150	TTACACTTAGGAGATTTCAACT	0	0	1	0
tsrna-18145	TTACACGCAGAAGGTCCTGGGTTCGAGCCCCAGTGGAACCA(	48	49	45	21
tsrna-18144	TTACACGCAGAAGGTCCTGGGT	1	3	1	0
tsrna-18142	TTAATCTGAGGGTCCAGGGTTCATG	2	11	4	1
tsrna-18141	TTAATCTGAGGGTCCAGGGTTCAT	4	16	0	0
tsrna-18140	TTAATCTGAGGGTCCAGGGTTCAAGTCCCTGTTCGGGCGCCA	13	54	8	3
tsrna-18139	TTAATCTGAGGGTCCAGGGTTCAAGT	8	29	3	1
tsrna-18138	TTAATCTGAGGGTCCAGGGTTCAAG	6	22	1	2
tsrna-18137	TTAATCTGAGGGTCCAGGGTTCAA	5	17	0	1
tsrna-18136	TTAATCTGAGGGTCCAGGGTTCA	5	15	2	2
tsrna-18135	TTAATCTGAGGGTCCAGGGTTC	4	9	5	0
tsrna-18134	TTAATCTGAGGGTCCAGGGTT	4	13	1	0
tsrna-18133	TTAATCTGAGGGTCCAGGGT	1	9	0	1
tsrna-18132	TTAATCTGAGGGTCCAGGG	1	8	2	0
tsrna-18131	TTAATCTGAGGGTCCAGG	4	6	1	2
tsrna-18130	TTAATCTGAGGGTCCAG	1	5	0	2
tsrna-18129	TTAATCTGAGGGTCCA	1	12	1	0
tsrna-18128	TTAATCTCAGGGTCGTGGGTTCGAGCCCCACGTTGGGCGCC/	17	279	11	5
tsrna-18127	TTAATCTCAGGGTCGTGGGTTC	4	6	2	0
tsrna-18126	TTAATCTCAGGGTCGTGGGTT	3	11	6	1
tsrna-18125	TTAATCTCAGGGTCGTGGGT	2	8	4	2
tsrna-18124	TTAATCTCAGGGTCGTGGGC	4	8	3	1
tsrna-18123	TTAATCTCAGGGTCGTGGG	5	7	1	1
tsrna-18122	TTAATCTCAGGGTCGTGG	5	5	0	3
tsrna-18121	TTAATCTCAGGGTCGTG	6	8	0	1
tsrna-18120	TTAATCCCAGGGTCGTGGGT	0	0	1	0
tsrna-18119	TTAATCCCAGGGTCGTGGG	1	1	0	0
tsrna-18118	TTAATCCCAGGGTCGTGG	0	1	0	0
tsrna-18116	TTAATCCCAGGGTCGT	0	0	1	0
tsrna-18115	TTAAGTTAAAGATTAAGAGAACCAACACCTCTTTACAGTGACC.	7	6	9	1
tsrna-18114	TTAAGTTAAAGATTAAGAGAACCAACACCTCTTTACAGTGACC	6	1	6	2
tsrna-18113	TTAAGTTAAAGATTAAGAGAACCAACACCTCTTTACAGTGAC	2	0	3	0
tsrna-18112	TTAAGTTAAAGATTAAGAGAACCAACACCTCTTTACAGTGA	1	0	2	1
tsrna-18111	TTAAGTTAAAGATTAAGAGAACCAACACCT	0	0	1	1
tsrna-18110	TTAAGTTAAAGATTAAGAGAACCAACACC	2	0	0	3
tsrna-18109	TTAAGTTAAAGATTAAGAGAACCA	0	0	0	0
tsrna-18108	TTAAGTTAAAGATTAAGAGAACC	0	0	1	0
tsrna-18107	TTAAGTTAAAGATTAAGAGAAC	1	0	0	0
tsrna-18104	TTAAGTCCCATTGGTCTAGCC	1	0	0	0
tsrna-18099	TTAAGGCTCCAGTCTCTTCGGG	0	1	0	0
tsrna-18098	TTAAGGCTCCAGTCTCTTCGG	1	0	0	0
tsrna-18097	TTAAGGCTCCAGTCTCTTCG	0	0	1	0
tsrna-18087	TTAAGAGAACCAACACCTCTTTACAGTGACCA	1	0	1	2
tsrna-18086	TTAAGAGAACCAACACCTCTTTACAGTGACC	0	0	0	1
tsrna-18085	TTAAGAGAACCAACACCTCTTTACAGTGAC	0	1	0	0
tsrna-18080	TTAACTTGACCGCTCTGACCA	6	17	10	2
tsrna-18079	TTAACTTGACCGCTCTGACC	0	6	1	0
tsrna-18078	TTAACTTGACCGCTCTGAC	0	0	0	0
tsrna-18077	TTAACTTGACCGCTCTGA	0	0	1	0
tsrna-18074	TTAACTTCCAATTAACTAGTTTTGAC	0	0	0	0
tsrna-18065	TTAACTAAGTGTTTGTGGGTTTAAGTCCCATTGGTCTAGCCA	7	1	2	1
tsrna-18064	TTAACTAAGTGTTTGTGGGTTTAAGTCCCATTGGTCTAGCC	7	2	3	0
tsrna-18063	TTAACTAAGTGTTTGTGGGTTTAAGTCCCATTGGTCTAGC	2	1	0	0
tsrna-18062	TTAACTAAGTGTTTGTGGGTTTAAGTCCCATTGGTCTAG	5	2	4	1
tsrna-18061	TTAACTAAGTGTTTGTGGGTTTAAGTCCCATTGGTCTA	7	2	1	0
tsrna-18060	TTAACTAAGTGTTTGTGGGTTTAAGTCCCATTGGTCT	3	3	5	1

tsrna-18059	TTAACTAAGTGTTTGTGGGTTTAAGTCCCATTGGTC	1	1	6	0
tsrna-18058	TTAACTAAGTGTTTGTGGGTTTAAGTCCC	1	2	0	0
tsrna-18057	TTAACTAAGTGTTTGTGGGTTTAAGTCC	2	2	0	0
tsrna-18056	TTAACTAAGTGTTTGTGGGTTTAAGTC	1	0	1	0
tsrna-18055	TTAACTAAGTGTTTGTGGGTTTAAGT	2	1	1	0
tsrna-18054	TTAACTAAGTGTTTGTGGGTTTAAG	1	1	1	0
tsrna-18053	TTAACTAAGTGTTTGTGGGTTTA	1	1	0	0
tsrna-18052	TTAACTAAGTGTTTGTGGGTTT	1	0	1	0
tsrna-18051	TTAACTAAGTGTTTGTGGGTT	0	0	0	0
tsrna-18050	TTAACTAAGTGTTTGTGGGT	0	1	0	0
tsrna-18046	TTAACCTTTTAAGTTAAAGATTAAGAGAACC	0	0	0	0
tsrna-18045	TTAACCTTTTAAGTTAAAGATTAAGAGAAC	0	0	0	0
tsrna-18043	TTAACCGAAAGGTTGGTGGTTCGATCCCACCCAGGGACGCCA	4	14	6	2
tsrna-18042	TTAACCGAAAGGTTGGTGGTTCGAGCCCACCCAGGGACGCCA	23	121	5	2
tsrna-18041	TTAACCGAAAGGTTGGTGGTTCGAGCCCACCCAGGGACGCC	11	20	6	0
tsrna-18040	TTAACCGAAAGGTTGGTGGTTCGAGCCCACCCAGGGACG	4	10	3	0
tsrna-18039	TTAACCGAAAGGTTGGTGGTTCGAGCCCACCCAGGGAC	0	6	1	0
tsrna-18038	TTAACCGAAAGGTTGGTGGTTCGAGCCCACCCAGGG	1	5	1	0
tsrna-18037	TTAACCGAAAGGTTGGTGGTTCGAGCCCAC	2	3	1	0
tsrna-18036	TTAACCGAAAGGTTGGTGGTTCGA	3	7	1	0
tsrna-18035	TTAACCGAAAGGTTGGTGGTTCG	0	5	0	0
tsrna-18034	TTAACCGAAAGGTTGGTGGTTC	0	0	1	0
tsrna-18033	TTAACCGAAAGGTTGGTGGTT	0	1	0	0
tsrna-18032	TTAACCGAAAGGTTGGTGGT	0	0	1	0
tsrna-18031	TTAACCGAAAGGTTGGTGG	0	0	0	0
tsrna-18027	TTAACCAAAACATCAGATTGTGAATCT	0	0	0	0
tsrna-18018	TTAACACAAAGCACCCAACTTACACTTAGG	0	0	0	0
tsrna-18013	TTAAATTATGATAATCATATTTACCAAC	0	0	1	0
tsrna-18011	TTAAATACAGACCAAGAGCC	1	0	0	0
tsrna-18010	TTAAATACAGACCAAGA	0	1	0	0
tsrna-18008	TTAAAGTGGCTGATTTGCGTTCA	0	0	0	0
tsrna-18007	TTAAAGTGGCTGATTTGCGTTC	0	1	0	0
tsrna-18006	TTAAAGTGGCTGATTTGC	0	1	0	0
tsrna-18004	TTAAAGATTAAGAGAACCAACACCTCTTTACAGTGACCA	10	2	7	0
tsrna-18003	TTAAAGATTAAGAGAACCAACACCTCTTTACAGTGACC	5	0	2	1
tsrna-18002	TTAAAGATTAAGAGAACCAACACCTCTTTACAGTGAC	6	0	2	0
tsrna-18001	TTAAAGATTAAGAGAACCAACACCTCTTT	0	0	0	0
tsrna-17997	TTAAAGATTAAGAGAACCAAC	0	0	0	0
tsrna-17996	TTAAAGATTAAGAGAACCAA	1	0	0	0
tsrna-17993	TTAAAGACTTTTTCTCTGACCA	16	24	9	12
tsrna-17992	TTAAAGACTTTTTCTCTGACC	3	11	2	3
tsrna-17988	TTAAACCCCCTTATTTCTACCA	0	1	1	0
tsrna-17987	TTAAACCCCCTTATTTCTACC	0	2	0	0
tsrna-17985	TTAAACCCCCTTATTTC	0	1	0	0
tsrna-17983	TGTTTGTGGGTTTAAGTCCCATTGGTCTAGCCA	2	0	2	0
tsrna-17982	TGTTTGTGGGTTTAAGTCCCATTGGTCTAGCC	1	0	1	0
tsrna-17981	TGTTTGTGGGTTTAAGTCCCATTGGTCTAGC	1	0	1	0
tsrna-17980	TGTTTGTGGGTTTAAGTCCCATTGGTCTAG	0	1	0	0
tsrna-17979	TGTTTGTGGGTTTAAGTCCCATTGG	0	1	0	0
tsrna-17973	TGTTTAGACGGGCTCACATCACCCCATAAACACC	33	9	20	5
tsrna-17972	TGTTTAGACGGGCTCACATCACCCCATAAA	17	11	21	5
tsrna-17971	TGTTTAGACGGGCTCACATCACCCCATAA	17	10	20	4
tsrna-17970	TGTTTAGACGGGCTCACATCACCCCATA	25	7	13	6
tsrna-17969	TGTTTAGACGGGCTCACATCACCCCAT	19	8	20	3
tsrna-17968	TGTTTAGACGGGCTCACATCACCCCA	11	8	15	3
tsrna-17967	TGTTTAGACGGGCTCACATCACCCC	21	5	13	1
tsrna-17966	TGTTTAGACGGGCTCACATCACCC	15	6	16	3
tsrna-17965	TGTTTAGACGGGCTCACATCACC	15	8	14	1
tsrna-17964	TGTTTAGACGGGCTCACATCAC	13	9	8	1
tsrna-17963	TGTTTAGACGGGCTCACATCA	12	6	8	3
tsrna-17962	TGTTTAGACGGGCTCACATC	10	5	13	2
tsrna-17961	TGTTTAGACGGGCTCACAT	10	9	7	1
tsrna-17960	TGTTTAGACGGGCTCACA	7	5	7	1
tsrna-17959	TGTTTAGACGGGCTCAC	7	3	4	2

tsrna-17958	TGTTTAGACGGGCTCA	3	0	1	0
tsrna-17957	TGTTGGTTATACCCTTCCCGTACTACCA	4	3	2	0
tsrna-17956	TGTTGGTTATACCCTTCCCGTACTACC	0	1	1	0
tsrna-17954	TGTTGGTTATACCCTTCCCGTACTA	1	0	0	0
tsrna-17953	TGTTGGTTATACCCTTCCCGTACT	1	0	1	0
tsrna-17952	TGTTGGTTATACCCTTCCCGTAC	0	0	0	0
tsrna-17949	TGTTGGTTATACCCTTCCCG	1	0	0	0
tsrna-17946	TGTTCGAATCACGTCGGGGTCACCA	6	8	4	5
tsrna-17945	TGTTCGAATCACGTCGGGGTCACC	0	3	0	0
tsrna-17937	TGTTCAAGTCACGTCGGGGTCACCA	7	5	3	2
tsrna-17936	TGTTCAAGTCACGTCGGGGTCACC	0	3	0	0
tsrna-17934	TGTTCAAGTCACGTCGGGGTCA	0	1	0	0
tsrna-17928	TGTTCAAATCACGTCGGGGTCACCA	3	10	3	5
tsrna-17926	TGTTCAAATCACGTCGGGGTCAC	0	0	0	0
tsrna-17919	TGTTAACTAAGTGTTTGTGGGTTTAAGTCCCATTGGTCTAGC	7	5	2	0
tsrna-17918	TGTTAACTAAGTGTTTGTGGGTTTAAGTCC	0	0	0	0
tsrna-17917	TGTTAACTAAGTGTTTGTGGGTTTAAGTC	1	0	1	0
tsrna-17916	TGTTAACTAAGTGTTTGTGGGTTTA	1	1	1	0
tsrna-17915	TGTTAACTAAGTGTTTGTGGGTTT	0	3	0	1
tsrna-17914	TGTTAACTAAGTGTTTGTGGGTT	0	0	0	0
tsrna-17913	TGTTAACTAAGTGTTTGTGGGT	1	2	0	0
tsrna-17912	TGTTAACTAAGTGTTTGTGGG	0	0	0	1
tsrna-17911	TGTTAACTAAGTGTTTGTGG	1	0	0	0
tsrna-17910	TGTTAACTAAGTGTTTGTG	0	0	1	0
tsrna-17909	TGTTAACCGAAAGGTTGGTGGT	3	3	0	0
tsrna-17907	TGTGTGAGGTCCCGGGTTCA	1	6	0	0
tsrna-17906	TGTGTGAGGTCCCGGGTTC	0	2	2	0
tsrna-17905	TGTGTGAGGTCCCGGGT	2	2	0	0
tsrna-17904	TGTGGTTAGGATTCGGCGC	0	0	4	0
tsrna-17901	TGTGGGTTTAAGTCCCATTGGTCTAGCCA	0	0	1	0
tsrna-17900	TGTGGGTTTAAGTCCCATTGGTCTAGCC	0	1	0	0
tsrna-17899	TGTGGGTTTAAGTCCCATTGGTCTAGC	0	2	3	0
tsrna-17898	TGTGGGTTTAAGTCCCATTGGTCTAG	1	1	1	0
tsrna-17897	TGTGGGTTTAAGTCCCATTGGTCTA	1	1	1	0
tsrna-17896	TGTGGGTTTAAGTCCCATTGGTCT	0	1	0	0
tsrna-17890	TGTGGGTTCGAGTCCCATCTGGGTCGCCA	0	13	0	1
tsrna-17889	TGTGGGTTCGAGTCCCATCTGGGTCGCC	0	1	0	0
tsrna-17881	TGTGGCCGCAGCAACCTCGGTT	0	1	0	0
tsrna-17880	TGTGGCCGCAGCAACCTCGGT	1	1	0	0
tsrna-17876	TGTGCTTTGCACGCGTGGGTTC	2	6	1	0
tsrna-17875	TGTGATGGCCGAGTGGTTAAGG	0	0	0	0
tsrna-17873	TGTGAATCTGACAACAGAGGCTTACGACCCCTTATTTACCCCA	22	10	13	1
tsrna-17872	TGTGAATCTGACAACAGAGGCTTACGACCCCTTATTTACCCC	13	8	12	0
tsrna-17871	TGTGAATCTGACAACAGAGGCTTACGACCCCTTATTTACCC	20	13	14	0
tsrna-17870	TGTGAATCTGACAACAGAGGCTTACGACCCCTTATT	9	10	6	0
tsrna-17869	TGTGAATCTGACAACAGAGGCTTACGACCC	0	0	0	0
tsrna-17868	TGTGAATCTGACAACAGAGGCTTACGACC	1	1	0	0
tsrna-17867	TGTGAATCTGACAACAGAGGCTTACGA	0	0	0	0
tsrna-17866	TGTGAATCTGACAACAGAGGCTTACG	0	0	0	0
tsrna-17865	TGTGAATCTGACAACAGAGGCTTAC	0	0	0	0
tsrna-17864	TGTGAATCTGACAACAGAGGCTTA	0	1	0	0
tsrna-17862	TGTGAATCTGACAACAGAGGCT	0	0	0	0
tsrna-17860	TGTGAATCTGACAACAGAGG	1	0	0	0
tsrna-17859	TGTGAATCTGACAACAGAG	0	0	1	0
tsrna-17858	TGTGAATCTGACAACAGA	0	1	0	0
tsrna-17855	TGTCTAACAACATGGCTTTCTCACCA	3	0	0	0
tsrna-17853	TGTCTAACAACATGGCTTTCTCAC	0	0	0	0
tsrna-17852	TGTCTAACAACATGGCTTTCTCA	1	0	0	0
tsrna-17851	TGTCTAACAACATGGCTTTCTC	1	0	0	0
tsrna-17849	TGTCTAACAACATGGCTTTC	0	0	0	0
tsrna-17846	TGTCCGCGTGGGTTCGAACCCCACTCCTGGTACC	1	1	0	0
tsrna-17844	TGTCACGCGGGAGACCGGGGTTCGATTCCCCGACGGGGAGC	5	18	3	1
tsrna-17843	TGTCACGCGGGAGACCGGGGTTCGATTCCC	0	1	0	0
tsrna-17842	TGTCACGCGGGAGACCGGGGTTCG	0	2	2	0

tsrna-17841	TGTCACGCGGGAGACCGGGGTTCAATTCCCCGACGGGGAGC	0	4	1	0
tsrna-17840	TGTCACGCGGGAGACCGGGGTT	2	2	0	0
tsrna-17839	TGTCACGCGGGAGACCGGGGT	0	1	0	0
tsrna-17838	TGTCACGCGGGAGACCGGGG	2	1	0	0
tsrna-17837	TGTCACGCGGGAGACCGG	2	4	0	0
tsrna-17836	TGTCACGCGGGAGACCG	0	3	0	1
tsrna-17835	TGTCACGCGGGAGACC	0	1	0	0
tsrna-17834	TGTCAAAGTTAAATTATAGGCTAAATCCT	7	1	4	0
tsrna-17833	TGTATGAGGTCCCGGGTTCGAT	3	5	0	0
tsrna-17832	TGTATGAGGTCCCGGGTTCGA	3	6	0	0
tsrna-17831	TGTATGAGGTCCCGGGTTCG	0	3	0	1
tsrna-17830	TGTATGAGGTCCCGGGTTC	1	2	0	0
tsrna-17829	TGTATGAGGTCCCGGGTT	2	5	0	0
tsrna-17828	TGTATGAGGTCCCGGGT	1	3	0	0
tsrna-17827	TGTATGAGGCCCCGGGTTCGATCCCCGGCATCTCCACC	18	26	6	3
tsrna-17826	TGTATGAGGCCCCGGGTTCGATCCCCGGCATCTCCAC	0	5	3	0
tsrna-17825	TGTATGAGGCCCCGGGTTCGATCCCCGGC	2	7	4	0
tsrna-17824	TGTATGAGGCCCCGGGTTCGATC	0	4	0	0
tsrna-17823	TGTATGAGGCCCCGGGTTCGA	3	3	0	0
tsrna-17822	TGTATGAGGCCCCGGGTTCG	1	6	1	0
tsrna-17821	TGTATGAGGCCCCGGGT	0	4	1	0
tsrna-17820	TGTAGTTGAAATACAACGATGGTTTTTC	0	1	1	0
tsrna-17819	TGTAGTGTAGTGGTTATCACGTTCGCCT	0	1	0	0
tsrna-17818	TGTAGTGTAGTGGTTATCACGTTCGCC	1	0	1	0
tsrna-17816	TGTAGTGGTTATCACGTTCGCCTCAC	0	0	0	1
tsrna-17815	TGTAGTGGTTATCACGTTCGCCTCA	0	0	1	0
tsrna-17800	TGTAGTGGTTATCACATTCGCC	0	1	0	0
tsrna-17798	TGTAGTGGTCATCACGTTCGCCT	0	1	0	0
tsrna-17795	TGTAGTGGTATCATGCAAGATTCCC	0	0	0	0
tsrna-17793	TGTAGTGGTATCATGCAAGATTC	0	1	1	0
tsrna-17788	TGTAGTCGTGGCCGAGTGG	7	13	8	5
tsrna-17787	TGTAGTCGTGGCCGAGTG	4	3	0	4
tsrna-17786	TGTAGTCGTGGCCGAG	0	3	0	3
tsrna-17785	TGTAGTCCGTGCGAGAATACCA	0	0	1	0
tsrna-17784	TGTAGTCCGTGCGAGAATACC	0	0	0	0
tsrna-17783	TGTAGTCCGTGCGAGAATAC	0	1	0	0
tsrna-17782	TGTAGTCCGTGCGAGAATA	0	1	0	0
tsrna-17778	TGTAGTATAAACTAATACACCAG	0	0	1	0
tsrna-17777	TGTAGCTTACCTCCTCAAAGCAATA	0	1	0	0
tsrna-17774	TGTAGCTTACCTCCTCAAAG	0	0	0	0
tsrna-17769	TGTAGCTCAGTGGTAGAGCGCGTGCTT	6	30	1	0
tsrna-17768	TGTAGCTCAGTGGTAGAGCGCGTGC	8	18	1	0
tsrna-17767	TGTAGCTCAGTGGTAGAGCGCGTG	0	1	0	0
tsrna-17760	TGTAGCTCAGTGGTAGAGCATTTGACTGC	2	3	1	2
tsrna-17759	TGTAGCTCAGTGGTAGAGCATTTGACTG	1	4	2	0
tsrna-17758	TGTAGCTCAGTGGTAGAGCATTTGACT	1	3	0	1
tsrna-17748	TGTAGCGGTTATCACATTCGCC	0	0	0	0
tsrna-17744	TGTAGATCCTTAGGTCGCTGGT	0	1	0	0
tsrna-17740	TGTACGAGGCCCCGGGTTCG	1	2	0	1
tsrna-17739	TGTACGAGGCCCCGGGTTC	1	3	0	0
tsrna-17738	TGTACGAGGCCCCGGGT	0	2	0	0
tsrna-17735	TGTAATGGTTAGCACTCTGGACTTTGAATCCA	12	5	4	5
tsrna-17734	TGTAATGGTTAGCACTCTGGACTTTGAATC	4	1	2	0
tsrna-17733	TGTAATGGTTAGCACTCTGGACTTTGAA	1	1	0	0
tsrna-17732	TGTAATGGTTAGCACTCTGGACTTTGA	0	0	1	2
tsrna-17731	TGTAATGGTTAGCACTCTGGACTTTG	0	0	0	0
tsrna-17730	TGTAATGGTTAGCACTCTGGACTTT	3	0	1	1
tsrna-17729	TGTAATGGTTAGCACTCTGGACTT	0	0	1	0
tsrna-17728	TGTAATGGTTAGCACTCTGGACTCTGAATCCAGCGA	5	4	4	4
tsrna-17727	TGTAATGGTTAGCACTCTGGACTCTGAATCCA	4	6	3	3
tsrna-17726	TGTAATGGTTAGCACTCTGGACTCTGAATCC	3	7	2	6
tsrna-17725	TGTAATGGTTAGCACTCTGGACTCTGAATC	0	2	1	2
tsrna-17724	TGTAATGGTTAGCACTCTGGACTCTGAAT	2	1	1	0
tsrna-17723	TGTAATGGTTAGCACTCTGGACTCTGAA	0	1	0	0

tsrna-17722	TGTAATGGTTAGCACTCTGGACTCTGA	1	0	0	1
tsrna-17721	TGTAATGGTTAGCACTCTGGACTCTG	0	2	0	0
tsrna-17720	TGTAATGGTTAGCACTCTGGACTCT	1	1	1	0
tsrna-17719	TGTAATGGTTAGCACTCTGGACTC	1	0	0	0
tsrna-17718	TGTAATGGTTAGCACTCTGGACT	0	1	0	0
tsrna-17717	TGTAATGGTTAGCACTCTGGAC	0	0	0	2
tsrna-17716	TGTAATGGTTAGCACTCTGGA	0	0	0	1
tsrna-17714	TGTAATGGTTAGCACTCTG	1	0	0	0
tsrna-17711	TGTAATGGTGAGCACTCTGGACTCTGAATCCA	0	12	1	3
tsrna-17710	TGTAATGGTGAGCACTCTGGACTCTGA	0	0	0	0
tsrna-17709	TGTAATGGTGAGCACTCTGGACTCTG	1	0	0	0
tsrna-17708	TGTAATGGTGAGCACTCTGGACTCT	0	0	0	0
tsrna-17706	TGTAATGGTGAGCACTCTGGACT	0	0	0	0
tsrna-17705	TGTAATGGTGAGCACTCTGGAC	0	0	0	1
tsrna-17703	TGTAATGGTGAGCACTCTGG	0	0	0	0
tsrna-17702	TGTAATGGTGAGCACTCTG	0	1	0	0
tsrna-17696	TGTAATGGTAAGCACTCTGGACTCTGA	0	2	0	0
tsrna-17695	TGTAATGGTAAGCACTCTGGACTCTG	0	0	0	0
tsrna-17692	TGTAATGGTAAGCACTCTGGACT	0	0	0	0
tsrna-17690	TGTAATGGTAAGCACTCTGGA	0	0	0	0
tsrna-17679	TGTAAACCGGAGATGAAAACCTTTTTCCAAGGACACCA	38	34	93	0
tsrna-17678	TGTAAACCGGAGATGAAAACCTTTTTCCAAGGACACC	7	19	16	0
tsrna-17677	TGTAAACCGGAGATGAAAACCTTTTTCCAAGGACAC	2	16	1	0
tsrna-17676	TGTAAACCGGAGATGAAAACCTTTTTCCA	4	7	1	0
tsrna-17675	TGTAAACCGGAGATGAAAACCTT	0	0	1	0
tsrna-17674	TGTAAACCGGAGATGAAAACCT	1	0	0	0
tsrna-17673	TGTAAACCGGAGATGAAAACC	0	1	0	0
tsrna-17672	TGTAAACCAGGGGTCGCGAGTTC	8	27	8	3
tsrna-17671	TGTAAACCAGGGGTCGCGAGT	14	24	6	2
tsrna-17670	TGTAAACCAGGGGTCGCGAG	2	5	1	0
tsrna-17669	TGTAAACCAGGGGTCGCGA	0	3	0	0
tsrna-17668	TGTAAACCAGGGGTCGCG	0	0	1	0
tsrna-17667	TGTAAACCAGGGGTCG	1	0	0	0
tsrna-17666	TGGTTTTTCATATCATTGGTCGTGGTTGTAGTCCGTGCGAGAA	17	19	22	14
tsrna-17665	TGGTTTTTCATATCATTGGTCGTGGTTGTAGTCCGT	4	4	5	0
tsrna-17664	TGGTTTTTCATATCATTGGTCGTGGTTGTA	1	1	2	0
tsrna-17663	TGGTTTTTCATATCATTGGTCGTGGTTGT	0	0	0	0
tsrna-17662	TGGTTTTTCATATCATTGGTCGTGGTTG	0	2	2	0
tsrna-17661	TGGTTTTTCATATCATTGGTCGTGGTT	0	1	0	0
tsrna-17659	TGGTTTTTCATATCATTGGTCGTGG	0	0	0	0
tsrna-17657	TGGTTTTTCATATCATTGGTCGT	0	0	0	0
tsrna-17651	TGGTTTTCACCCAGGTGGCCCGG	2	5	4	0
tsrna-17650	TGGTTTTCACCCAGGTGGCCCG	1	7	2	0
tsrna-17649	TGGTTTTCACCCAGGTGGCC	2	3	3	0
tsrna-17648	TGGTTTTCACCCAGGCGGCCCGGGT	1	2	1	0
tsrna-17647	TGGTTTTCACCCAGGCGGCCCGG	1	4	0	1
tsrna-17646	TGGTTTTCACCCAGGCGGCCCG	0	5	0	0
tsrna-17645	TGGTTTTCACCCAGGCGGCCC	2	1	1	1
tsrna-17644	TGGTTTTCACCCAGGCGGCC	0	3	0	0
tsrna-17643	TGGTTTAGTGGTAGAATTCTCGCCT	3	2	1	1
tsrna-17642	TGGTTTAGTGGTAGAATTCTCGCC	1	6	1	2
tsrna-17641	TGGTTTAGTGGTAGAATTCTCGC	2	1	1	0
tsrna-17640	TGGTTTAGTGGTAGAATTCTCG	0	0	0	0
tsrna-17639	TGGTTTAGTGGTAGAATTCTC	0	0	1	0
tsrna-17637	TGGTTGTAGTCCGTGCGAGAATACCA	0	5	1	2
tsrna-17636	TGGTTGTAGTCCGTGCGAGAATACC	0	0	1	1
tsrna-17635	TGGTTGTAGTCCGTGCGAGAATAC	2	0	0	0
tsrna-17634	TGGTTGTAGTCCGTGCGAGAATA	1	1	0	0
tsrna-17633	TGGTTGTAGTCCGTGCGAGAAT	0	2	0	0
tsrna-17632	TGGTTGTAGTCCGTGCGAGAA	0	1	0	0
tsrna-17631	TGGTTGTAGTCCGTGCGAGA	0	0	0	1
tsrna-17630	TGGTTGTAGTCCGTGCGAG	0	0	0	0
tsrna-17629	TGGTTGTAGTCCGTGCGA	0	0	0	0
tsrna-17626	TGGTTCGATTCCGGCTCGAAGGACCA	74	158	20	24

tsrna-17625	TGGTTCGATTCCGGCTCGAAGGACC	7	12	2	3
tsrna-17623	TGGTTCGATTCCGGCTCGAAGGA	0	0	0	0
tsrna-17621	TGGTTCGATTCCGGCTCGAAG	1	0	0	0
tsrna-17620	TGGTTCGATTCCAGCTCGAAGGACCA	0	0	0	0
tsrna-17619	TGGTTCGATCCCGGGTTTCGGCACCA	38	83	14	10
tsrna-17617	TGGTTCGATCCCGGGTTTCGG	0	1	0	0
tsrna-17615	TGGTTCGAGCCCACCCAGGGACGCCA	2	120	0	3
tsrna-17611	TGGTTCGAGCCCACCCAG	0	1	0	0
tsrna-17608	TGGTTCGAATCCGGCTCGAAGGACCA	23	42	14	9
tsrna-17607	TGGTTCGAATCCGGCTCGAAGGACC	3	5	0	1
tsrna-17606	TGGTTCGAATCCGGCTCGAAGGAC	0	1	0	0
tsrna-17602	TGGTTCAGTGGTAGAATTCTT	2	2	1	0
tsrna-17601	TGGTTCAGTGGTAGAATTCTCGCCTGCCAC	24	66	13	9
tsrna-17600	TGGTTCAGTGGTAGAATTCTCGCCTGCC	16	35	6	9
tsrna-17599	TGGTTCAGTGGTAGAATTCTCGCCTGC	15	16	4	5
tsrna-17598	TGGTTCAGTGGTAGAATTCTCGCCTG	4	8	2	2
tsrna-17597	TGGTTCAGTGGTAGAATTCTCGCCTCC	7	16	1	13
tsrna-17596	TGGTTCAGTGGTAGAATTCTCGCCTC	12	9	2	1
tsrna-17595	TGGTTCAGTGGTAGAATTCTCGCCT	7	8	3	3
tsrna-17594	TGGTTCAGTGGTAGAATTCTCGCC	7	2	2	2
tsrna-17593	TGGTTCAGTGGTAGAATTCTCGC	2	0	0	2
tsrna-17592	TGGTTCAGTGGTAGAATTCTCG	3	0	1	0
tsrna-17591	TGGTTCAGTGGTAGAATTCTC	0	2	1	0
tsrna-17590	TGGTTCAGTGGTAGAATTCT	3	0	0	1
tsrna-17589	TGGTTCAGTGGTAGAATTC	6	3	0	2
tsrna-17588	TGGTTCAGTGGTAGAATT	1	3	1	0
tsrna-17587	TGGTTCAGTGGTAGAAT	1	2	0	1
tsrna-17586	TGGTTCAGTGGTAGAA	0	1	1	1
tsrna-17584	TGGTTCAAATCCGGGTGCCCCCTCCA	125	206	70	15
tsrna-17583	TGGTTATCACGTTCGCCTCACAC	1	0	0	0
tsrna-17582	TGGTTATCACGTTCGCCTCACA	2	0	1	1
tsrna-17576	TGGTTATCACGTTCGC	0	1	0	0
tsrna-17575	TGGTTATCACGTCTGCTTTACACGCAGAAGGTCCTGGGT	14	40	9	6
tsrna-17574	TGGTTATCACGTCTGCTTTACACGC	0	1	1	0
tsrna-17573	TGGTTATCACGTCTGCTTTACA	0	1	3	0
tsrna-17572	TGGTTATCACGTCTGCTTTAC	0	0	0	1
tsrna-17568	TGGTTATCACATTCGCCTCAC	1	0	0	0
tsrna-17567	TGGTTATACCCTTCCCGTACTACCA	1	2	0	0
tsrna-17558	TGGTTAGTATCCCCGCCTGTCACGCGGGAG	23	61	15	3
tsrna-17557	TGGTTAGTATCCCCGCCTGTCACGC	2	2	1	5
tsrna-17556	TGGTTAGTATCCCCGCCTGTCAC	0	3	3	2
tsrna-17555	TGGTTAGTATCCCCGCCTGTC	2	1	1	2
tsrna-17554	TGGTTAGTATCCCCGCCTGT	0	0	0	0
tsrna-17551	TGGTTAGTATCCCCGC	0	0	0	0
tsrna-17550	TGGTTAGTACTCTGCGTTGTGGCCGCAGC	9	8	2	1
tsrna-17549	TGGTTAGTACTCTGCGTTGTGGCCGCA	14	5	5	2
tsrna-17548	TGGTTAGTACTCTGCGTTGTGGCCGC	7	3	1	3
tsrna-17547	TGGTTAGTACTCTGCGTTGTGGCCG	10	5	4	1
tsrna-17546	TGGTTAGTACTCTGCGTTGTGGCC	8	3	5	2
tsrna-17545	TGGTTAGTACTCTGCGTTGTGGC	9	3	5	1
tsrna-17544	TGGTTAGTACTCTGCGTTGTGG	10	3	6	1
tsrna-17543	TGGTTAGTACTCTGCGTTGTG	8	1	4	1
tsrna-17542	TGGTTAGTACTCTGCGTTGT	9	5	9	2
tsrna-17541	TGGTTAGTACTCTGCGTTG	5	3	3	0
tsrna-17540	TGGTTAGTACTCTGCGTT	9	4	5	2
tsrna-17539	TGGTTAGTACTCTGCGT	0	0	1	0
tsrna-17538	TGGTTAGTACTCTGCGCTGTG	0	1	1	0
tsrna-17537	TGGTTAGTACTCTGCGCTGT	1	2	0	0
tsrna-17536	TGGTTAGTACTCTGCGCTG	2	0	2	0
tsrna-17535	TGGTTAGTACTCTGCGCT	1	0	2	0
tsrna-17534	TGGTTAGTACTCTGCGC	1	0	1	0
tsrna-17532	TGGTTAGGATTCGGCGCTCTCAT	10	6	3	3
tsrna-17531	TGGTTAGGATTCGGCGCTCTCACCGCCGCGGCCCGGGT	9	24	11	13
tsrna-17530	TGGTTAGGATTCGGCGCTCTCACCGCCGCGGCCCGGG	10	21	7	2

tsrna-17529	TGGTTAGGATTCGGCGCTCTCACCGCCGCGGCCCGG	14	24	8	6
tsrna-17528	TGGTTAGGATTCGGCGCTCTCACCGCCGCGGCCC	8	24	8	8
tsrna-17527	TGGTTAGGATTCGGCGCTCTCACCGCCGCGGC	12	16	12	6
tsrna-17526	TGGTTAGGATTCGGCGCTCTCACCGCCGCG	13	19	8	4
tsrna-17525	TGGTTAGGATTCGGCGCTCTCACCGCCGC	7	17	11	4
tsrna-17524	TGGTTAGGATTCGGCGCTCTCACCGCC	10	14	7	7
tsrna-17523	TGGTTAGGATTCGGCGCTCTCACCGC	13	12	4	8
tsrna-17522	TGGTTAGGATTCGGCGCTCTCACCG	8	10	5	6
tsrna-17521	TGGTTAGGATTCGGCGCTCTCACC	7	3	5	3
tsrna-17520	TGGTTAGGATTCGGCGCTCTCAC	12	6	3	3
tsrna-17519	TGGTTAGGATTCGGCGCTCTCA	8	12	4	3
tsrna-17518	TGGTTAGGATTCGGCGCTCTC	10	4	6	3
tsrna-17517	TGGTTAGGATTCGGCGCTCT	7	6	3	3
tsrna-17516	TGGTTAGGATTCGGCGCTC	5	8	3	2
tsrna-17515	TGGTTAGGATTCGGCGCT	1	4	2	3
tsrna-17514	TGGTTAGGATTCGGCGC	0	0	0	1
tsrna-17512	TGGTTAGCATAGCTGCCTTCCAAG	0	0	1	0
tsrna-17506	TGGTTAGCACTCTGGACTTTGAATCCAGCG	7	6	2	2
tsrna-17505	TGGTTAGCACTCTGGACTTTGAATCCAGC	4	3	2	3
tsrna-17504	TGGTTAGCACTCTGGACTTTGAATCCA	3	4	4	2
tsrna-17503	TGGTTAGCACTCTGGACTTTGAATCC	2	5	5	0
tsrna-17502	TGGTTAGCACTCTGGACTTTGAATC	0	2	1	1
tsrna-17499	TGGTTAGCACTCTGGACTTTGA	0	0	0	0
tsrna-17495	TGGTTAGCACTCTGGACTCTGAATCCAGCGATCCGAGTTCA	6	3	4	0
tsrna-17494	TGGTTAGCACTCTGGACTCTGAATCCAGCGATCCGAGTTC	2	4	3	0
tsrna-17493	TGGTTAGCACTCTGGACTCTGAATCCAGCGATCCGA	3	2	1	0
tsrna-17492	TGGTTAGCACTCTGGACTCTGAATCCAGCG	2	5	2	0
tsrna-17491	TGGTTAGCACTCTGGACTCTGAATCCAGC	4	3	4	1
tsrna-17490	TGGTTAGCACTCTGGACTCTGAATCCAG	3	3	2	1
tsrna-17489	TGGTTAGCACTCTGGACTCTGAATCCA	4	3	1	0
tsrna-17488	TGGTTAGCACTCTGGACTCTGAATCC	2	1	0	1
tsrna-17487	TGGTTAGCACTCTGGACTCTGAATC	0	0	1	0
tsrna-17486	TGGTTAGCACTCTGGACTCTGAAT	0	0	1	0
tsrna-17483	TGGTTAGCACTCTGGACTCTG	0	1	1	0
tsrna-17481	TGGTTAGCACTCTGGACTC	0	0	0	0
tsrna-17480	TGGTTAGCACTCTGGACT	1	0	0	0
tsrna-17478	TGGTTAGCACTCTGGA	1	0	0	1
tsrna-17477	TGGTTAGAGCGTGGTGCTAATA	2	4	3	1
tsrna-17475	TGGTTAAGGCGTTGGACTTGAAATC	1	0	0	0
tsrna-17474	TGGTTAAGGCGTTGGACTTGAAAT	0	0	0	0
tsrna-17471	TGGTTAAGGCGTTGGACTTAAGATC	0	0	1	0
tsrna-17470	TGGTTAAGGCGTTGGACTTAAGAT	0	0	0	0
tsrna-17469	TGGTTAAGGCGTTGGACTTAAGA	0	0	0	0
tsrna-17467	TGGTTAAGGCGTTGGACTTAA	0	1	0	0
tsrna-17466	TGGTTAAGGCGTTGGACTTA	0	1	0	0
tsrna-17456	TGGTTAAGGCGATGGACTGCTAAT	2	2	0	1
tsrna-17455	TGGTTAAGGCGATGGACTGCTAA	0	0	0	2
tsrna-17454	TGGTTAAGGCGATGGACTGCTA	1	0	0	0
tsrna-17453	TGGTTAAGGCGATGGACTAGAAATC	1	3	0	0
tsrna-17452	TGGTTAAGGCGATGGACTAGAA	0	1	0	0
tsrna-17451	TGGTTAAGGCGATGGACTAGA	0	1	0	0
tsrna-17450	TGGTTAAGGCGATGGACTAG	0	2	0	2
tsrna-17444	TGGTGTAGTGGTATCATGCAAGATTC	2	0	0	0
tsrna-17443	TGGTGTAGTGGTATCATGCAAGATT	1	2	0	0
tsrna-17442	TGGTGTAGTGGTATCATGCAAGA	0	2	3	0
tsrna-17441	TGGTGTAGTGGTATCATGCAAG	1	0	0	0
tsrna-17438	TGGTGTAGTGGTATCATG	1	0	0	0
tsrna-17437	TGGTGTAATGGTTAGCACTCTGGGCT	1	1	0	0
tsrna-17436	TGGTGTAATGGTTAGCACTCTGGACTTTGAATCC	15	12	6	9
tsrna-17435	TGGTGTAATGGTTAGCACTCTGGACTTTGAATC	8	7	3	9
tsrna-17434	TGGTGTAATGGTTAGCACTCTGGACTTTG	0	0	1	1
tsrna-17433	TGGTGTAATGGTTAGCACTCTGGACTTT	1	1	1	0
tsrna-17432	TGGTGTAATGGTTAGCACTCTGGACTT	0	0	3	3
tsrna-17431	TGGTGTAATGGTTAGCACTCTGGACTCTGAATCCAGCGATCC	12	9	6	4

tsrna-17430	TGGTGTAATGGTTAGCACTCTGGACTCTGAATCCAGCGATCC(	6	17	5	3
tsrna-17429	TGGTGTAATGGTTAGCACTCTGGACTCTGAATCCAG	12	9	9	4
tsrna-17428	TGGTGTAATGGTTAGCACTCTGGACTCTGAATCCA	7	8	2	1
tsrna-17427	TGGTGTAATGGTTAGCACTCTGGACTCTGAATC	2	5	0	4
tsrna-17426	TGGTGTAATGGTTAGCACTCTGGACTCTGAAT	3	2	1	2
tsrna-17425	TGGTGTAATGGTTAGCACTCTGGACTCTG	4	2	1	2
tsrna-17424	TGGTGTAATGGTTAGCACTCTGGACTCT	0	1	0	1
tsrna-17423	TGGTGTAATGGTTAGCACTCTGGACTC	2	2	2	0
tsrna-17422	TGGTGTAATGGTTAGCACTCTGGACT	1	1	1	0
tsrna-17421	TGGTGTAATGGTTAGCACTCTGGAC	1	1	0	0
tsrna-17420	TGGTGTAATGGTTAGCACTCTGGA	0	2	1	0
tsrna-17419	TGGTGTAATGGTTAGCACTCTGG	0	0	1	0
tsrna-17414	TGGTGTAATGGTGAGCACTCTGGACTCTG	0	0	0	0
tsrna-17413	TGGTGTAATGGTGAGCACTCTGGACTCT	0	1	0	1
tsrna-17412	TGGTGTAATGGTGAGCACTCTGGACTC	1	0	0	0
tsrna-17411	TGGTGTAATGGTGAGCACTCTGGACT	1	0	0	0
tsrna-17410	TGGTGTAATGGTGAGCACTCTGGAC	0	1	0	0
tsrna-17406	TGGTGTAATGGTCAGCACTCTGGACTC	0	0	0	0
tsrna-17403	TGGTGTAATGGTCAGCACTCTGGA	1	0	0	0
tsrna-17401	TGGTGTAATGGTAAGCACTCTGGACTC	0	0	0	0
tsrna-17400	TGGTGTAATGGTAAGCACTCTGGACT	0	0	0	1
tsrna-17394	TGGTGGTTCAGTGGTAGAATTCTCGCCTGC	11	10	7	2
tsrna-17393	TGGTGGTTCAGTGGTAGAATTCTCGCCTG	3	7	2	4
tsrna-17392	TGGTGGTTCAGTGGTAGAATTCTCGCCT	7	4	8	3
tsrna-17391	TGGTGGTTCAGTGGTAGAATTCTCGCC	4	12	5	0
tsrna-17390	TGGTGGTTCAGTGGTAGAATTCTCGC	4	5	2	0
tsrna-17389	TGGTGGTTCAGTGGTAGAATTCTCG	1	0	1	0
tsrna-17388	TGGTGGTTCAGTGGTAGAATTCTC	5	5	1	0
tsrna-17387	TGGTGGTTCAGTGGTAGAATTCT	2	6	2	2
tsrna-17386	TGGTGGTTCAGTGGTAGAATT	3	2	0	0
tsrna-17385	TGGTGGTTCAGTGGTAGAAT	2	1	1	1
tsrna-17384	TGGTGGTTCAGTGGTAGAA	4	0	0	0
tsrna-17383	TGGTGGTTCAGTGGTAGA	0	1	0	1
tsrna-17380	TGGTGGTCTAGTGGTTAGGATTCGGCGCTCTCACC	37	53	30	16
tsrna-17379	TGGTGGTCTAGTGGTTAGGATTCGGCGCTC	36	54	27	15
tsrna-17378	TGGTGGTCTAGTGGTTAGGATTCGGCGCT	29	33	17	11
tsrna-17377	TGGTGGTCTAGTGGTTAGGATTCGGCGC	13	14	8	8
tsrna-17376	TGGTGGTCTAGTGGTTAGGATTCGGCG	14	14	15	5
tsrna-17375	TGGTGGTCTAGTGGTTAGGATTCGGC	6	12	3	5
tsrna-17374	TGGTGGTCTAGTGGTTAGGATTCGG	6	4	3	0
tsrna-17373	TGGTGGTCTAGTGGTTAGGATTCG	2	0	1	2
tsrna-17372	TGGTGGTCTAGTGGTTAGGATTC	1	1	1	1
tsrna-17371	TGGTGGTCTAGTGGTTAGGATT	3	0	2	1
tsrna-17370	TGGTGGTCTAGTGGTTAGGAT	3	0	0	0
tsrna-17365	TGGTGGTCTAGTGGCTAGGATTCGGCGC	5	38	8	31
tsrna-17364	TGGTGGTCTAGTGGCTAGGATTCGGCG	17	46	3	25
tsrna-17363	TGGTGGTCTAGTGGCTAGGATTCGGC	4	16	4	17
tsrna-17362	TGGTGGTCTAGTGGCTAGGATTCGG	0	4	1	3
tsrna-17361	TGGTGGTCTAGTGGCTAGGATTCG	4	2	0	6
tsrna-17360	TGGTGGTCTAGTGGCTAGGAT	0	1	1	4
tsrna-17358	TGGTGGTCTAGTGGCTAG	0	1	0	0
tsrna-17357	TGGTGGAGTTAAAGACTTTTTCTCTGACCA	23	36	15	15
tsrna-17356	TGGTGGAGTTAAAGACTTTTTCTCTGACC	2	13	2	0
tsrna-17355	TGGTGGAGTTAAAGACTTTTTCTCTGAC	1	5	2	0
tsrna-17354	TGGTGGAGTTAAAGACTTTTTCTCTGA	1	0	3	0
tsrna-17353	TGGTGGAGTTAAAGACTTTTTCTCTG	2	5	1	0
tsrna-17351	TGGTGGAGTTAAAGACTTTTTCT	0	0	0	0
tsrna-17350	TGGTGGAGTTAAAGACTTTTT	0	0	0	0
tsrna-17349	TGGTGGAGTTAAAGACTTTT	0	1	1	0
tsrna-17348	TGGTGGAGTTAAAGACTTT	0	1	0	0
tsrna-17347	TGGTGGAGTTAAAGACTT	1	0	0	0
tsrna-17344	TGGTGCAACTCCAAATAAAAGTACCA	1	0	1	0
tsrna-17343	TGGTGCAACTCCAAATAAAAGTACC	1	0	0	0
tsrna-17340	TGGTGAGTATCCCCGCCTGTCACGCGGGAG	35	57	32	6

tsrna-17339	TGGTGAGTATCCCCGCCTGTCACGC	4	5	0	0
tsrna-17338	TGGTGAGTATCCCCGCCTGTCACG	5	7	1	3
tsrna-17337	TGGTGAGTATCCCCGCCTGTCAC	8	7	3	0
tsrna-17336	TGGTGAGTATCCCCGCCTGTC	5	13	3	0
tsrna-17335	TGGTGAGTATCCCCGCCTGT	2	3	1	0
tsrna-17334	TGGTGAGTATCCCCGCCTG	1	3	1	0
tsrna-17333	TGGTGAGTATCCCCGCCT	1	2	0	1
tsrna-17332	TGGTGAGTATCCCCGCC	0	1	0	0
tsrna-17331	TGGTGAGTATCCCCGC	1	1	0	0
tsrna-17330	TGGTGAGCATAGCTGCCTTCCAAG	0	1	0	0
tsrna-17329	TGGTGAGCATAGCTGCCTTCC	0	1	0	0
tsrna-17324	TGGTGAGCACTCTGGACTCTGAATCCAGC	1	5	0	2
tsrna-17323	TGGTGAGCACTCTGGACTCTGAATCCA	1	4	0	0
tsrna-17322	TGGTGAGCACTCTGGACTCTGAATCC	2	1	0	0
tsrna-17321	TGGTGAGCACTCTGGACTCTGAATC	1	1	0	0
tsrna-17320	TGGTGAGCACTCTGGACTCTGAAT	0	1	0	0
tsrna-17311	TGGTGAGCACCCTGGACTCTGA	0	0	0	0
tsrna-17310	TGGTCTCCGGATGGAGGCGTGGGTT	7	39	4	0
tsrna-17309	TGGTCTCCGGATGGAGGCGTGG	1	3	1	0
tsrna-17306	TGGTCTCCAATGGAGGCGTGGGTT	15	26	10	0
tsrna-17305	TGGTCTCCAATGGAGGCGTGGGT	15	25	16	0
tsrna-17304	TGGTCTAGTGGTTAGGATTCGGCGCTCTCA	39	66	28	16
tsrna-17303	TGGTCTAGTGGTTAGGATTCGGCGCTCTC	40	60	26	17
tsrna-17302	TGGTCTAGTGGTTAGGATTCGGCGCTCT	46	40	23	31
tsrna-17301	TGGTCTAGTGGTTAGGATTCGGCGCTC	37	46	30	24
tsrna-17300	TGGTCTAGTGGTTAGGATTCGGCGCT	32	39	28	11
tsrna-17299	TGGTCTAGTGGTTAGGATTCGGCGC	16	12	9	9
tsrna-17298	TGGTCTAGTGGTTAGGATTCGGCG	15	7	7	8
tsrna-17297	TGGTCTAGTGGTTAGGATTCGGC	9	6	5	1
tsrna-17296	TGGTCTAGTGGTTAGGATTCGG	3	3	2	1
tsrna-17295	TGGTCTAGTGGTTAGGATTCG	0	2	4	0
tsrna-17294	TGGTCTAGTGGTTAGGATTC	0	0	1	1
tsrna-17293	TGGTCTAGTGGTTAGGATT	1	0	0	1
tsrna-17290	TGGTCTAGTGGTATGATTCTCGC	6	0	5	2
tsrna-17289	TTTTTCCAAGGACACCA	1	0	2	0
tsrna-17288	TTTTTCCAAGGACACC	0	0	1	1
tsrna-17287	TTTTTCATATCATTGGTCGTGGTTGTAGTCCGTGCGAGAATAC	138	156	177	52
tsrna-17286	TTTTTCATATCATTGGTCGTGGTTGTAGTCCGTGCGAGAATAC	19	34	23	18
tsrna-17285	TTTTTCATATCATTGGTCGTGGTTGTAGTCCGTGCGAGAATA	17	21	13	16
tsrna-17284	TTTTTCATATCATTGGTCGTGGTTGTAGTCCGTGCGAGAAT	11	16	8	15
tsrna-17283	TTTTTCATATCATTGGTCGTGGTTGTAGTCCGTGCGAGAA	8	17	12	10
tsrna-17282	TTTTTCATATCATTGGTCGTGGTTGTAGTCCGTGCGAGA	5	16	10	7
tsrna-17281	TTTTTCATATCATTGGTCGTGGTTGTAGTCCGTGCG	7	3	5	7
tsrna-17280	TTTTTCATATCATTGGTCGTGGTTGTAGTC	2	1	1	0
tsrna-17279	TTTTTCATATCATTGGTCGTGGTTGTAGT	1	1	0	0
tsrna-17278	TTTTTCATATCATTGGTCGTGGTTGTAG	0	0	1	0
tsrna-17277	TTTTTCATATCATTGGTCGTGGTTGTA	0	0	0	1
tsrna-17275	TTTTTCATATCATTGGTCGTGGTTG	0	0	0	0
tsrna-17263	TTTTGCAGTCCTTACCA	0	2	0	0
tsrna-17261	TTTTCCAAGGACACCA	1	1	1	0
tsrna-17260	TTTTCATATCATTGGTCGTGGTTGTAGTCCGTGCGAGAATACC	154	163	176	60
tsrna-17259	TTTTCATATCATTGGTCGTGGTTGTAGTCCGTGCGAGAATACC	21	27	16	18
tsrna-17258	TTTTCATATCATTGGTCGTGGTTGTAGTCCGTGCGAGAATA	10	24	11	9
tsrna-17257	TTTTCATATCATTGGTCGTGGTTGTAGTCCGTGCGAGAAT	10	21	13	14
tsrna-17256	TTTTCATATCATTGGTCGTGGTTGTAGTCCGTGCGAGAA	11	19	9	14
tsrna-17255	TTTTCATATCATTGGTCGTGGTTGTAGTCCGTGCGAGA	7	4	12	6
tsrna-17254	TTTTCATATCATTGGTCGTGGTTGTAGTCCGTGCGA	7	4	4	6
tsrna-17253	TTTTCATATCATTGGTCGTGGTTGTAGTCC	0	2	1	1
tsrna-17252	TTTTCATATCATTGGTCGTGGTTGTAGTC	0	1	2	0
tsrna-17251	TTTTCATATCATTGGTCGTGGTTGTAGT	0	1	2	1
tsrna-17250	TTTTCATATCATTGGTCGTGGTTGTAG	1	0	0	0
tsrna-17249	TTTTCATATCATTGGTCGTGGTTGTA	0	0	0	0
tsrna-17248	TTTTCATATCATTGGTCGTGGTTGT	0	0	0	1
tsrna-17247	TTTTCATATCATTGGTCGTGGTTG	0	1	0	0

tsrna-17239	TTTTCACCCAGGTGGCCCGGG	2	2	2	0
tsrna-17238	TTTTCACCCAGGTGGCCCGG	4	4	3	0
tsrna-17237	TTTTCACCCAGGTGGCCCG	1	4	1	0
tsrna-17236	TTTTCACCCAGGTGGCCC	5	1	1	0
tsrna-17235	TTTTCACCCAGGCGGCCCGGGTTCGACTCCCGGTGTGGGAA(	87	356	48	28
tsrna-17234	TTTTCACCCAGGCGGCCCGGGTTCGACTCCCGGTGTGG	1	4	0	0
tsrna-17233	TTTTCACCCAGGCGGCCCGGGTTCGACTCCCGGTGTG	0	1	1	0
tsrna-17231	TTTTCACCCAGGCGGCCCGGGTTCGACTCC	2	1	0	0
tsrna-17230	TTTTCACCCAGGCGGCCCGGGT	4	1	0	1
tsrna-17229	TTTTCACCCAGGCGGCCCGGG	1	0	0	0
tsrna-17228	TTTTCACCCAGGCGGCCCGG	2	2	0	0
tsrna-17227	TTTTCACCCAGGCGGCCCG	0	3	0	0
tsrna-17225	TTTTCACCCAGGCGGCC	0	1	0	0
tsrna-17224	TTTTAATCTGAGGGTCCAGGGTTCAAGTCCCTGTTCGGGCGC	16	52	11	5
tsrna-17223	TTTTAATCTGAGGGTCCAGGGT	4	10	1	1
tsrna-17222	TTTTAATCTGAGGGTCCAGGG	1	18	2	0
tsrna-17221	TTTTAATCTGAGGGTCCAGG	2	11	2	0
tsrna-17220	TTTTAATCTGAGGGTCCAG	3	14	4	0
tsrna-17219	TTTTAATCTGAGGGTCCA	5	9	6	1
tsrna-17218	TTTTAATCTGAGGGTCC	2	8	1	1
tsrna-17217	TTTTAAGTTAAAGATTAAGAGAACCAACACCTCTTTACAGTGA(	5	1	9	2
tsrna-17216	TTTTAAGTTAAAGATTAAGAGAACCAACACCTCTTTACAGTGA(	4	1	6	0
tsrna-17215	TTTTAAGTTAAAGATTAAGAGAACCAACACCTCTTTACAGTGA(	4	1	3	0
tsrna-17214	TTTTAAGTTAAAGATTAAGAGAACCAACACC	0	0	0	1
tsrna-17213	TTTTAAGTTAAAGATTAAGAGAACCAACAC	0	0	0	0
tsrna-17212	TTTTAAGTTAAAGATTAAGAGAACC	0	0	0	0
tsrna-17206	TTTGTGGGTTTAAGTCCCATTGGTCTAGCCA	1	1	1	1
tsrna-17205	TTTGTGGGTTTAAGTCCCATTGGTCTAGCC	2	0	1	0
tsrna-17203	TTTGTGGGTTTAAGTCCCATTGGTC	0	0	1	0
tsrna-17200	TTTGTGGGTTTAAGTC	0	0	0	0
tsrna-17199	TTTGTCAAAGTTAAATTATAGGCT	3	0	0	0
tsrna-17198	TTTGTCAAAGTTAAATTATAG	3	0	1	0
tsrna-17197	TTTGGTGCAACTCCAAATAAAAGTACCA	0	6	2	0
tsrna-17195	TTTGGTCTAGGGGTATGAT	1	0	0	0
tsrna-17193	TTTGGGTGCTAATGGTGGAGTTAAAGACTTTTTCTC	6	17	6	0
tsrna-17192	TTTGGGTGCTAATGGTGGAGTTAAAGACTT	3	6	2	2
tsrna-17191	TTTGGGTGCTAATGGTGGAGTTAAAGAC	2	3	0	2
tsrna-17190	TTTGGGTGCTAATGGTGGAGTTAAAGA	1	2	3	0
tsrna-17189	TTTGGGTGCTAATGGTGGAGTTAAAG	1	7	0	1
tsrna-17188	TTTGGGTGCTAATGGTGGAGTTAAA	0	4	1	0
tsrna-17187	TTTGGGTGCTAATGGTGGAGTTAA	0	2	0	0
tsrna-17186	TTTGGGTGCTAATGGTGGAGTTA	1	1	0	1
tsrna-17179	TTTGGGTGCGAGAGGTCCCGGGTT	11	67	6	26
tsrna-17178	TTTGGGTGCGAGAGGTCCCGGGT	8	42	2	7
tsrna-17177	TTTGGGTGCGAGAGGTCCCGGG	2	2	0	0
tsrna-17175	TTTGGGTGCGAGAGGTCCCG	0	1	0	0
tsrna-17173	TTTGGGTGCGAGAGGTCC	0	1	0	0
tsrna-17171	TTTGGGTCCGAGAGGTCCCGGGTTCA	0	2	0	0
tsrna-17170	TTTGGGTCCGAGAGGTCCCGGGTT	0	1	0	0
tsrna-17169	TTTGGGTCCGAGAGGTCCCGGGT	0	1	0	0
tsrna-17163	TTTGGGGGGTTCGATTCCTTCCTTTTTTGC	1	2	0	1
tsrna-17159	TTTGCATGTATGAGGTCCCGGGT	1	4	1	2
tsrna-17158	TTTGCATGTATGAGGCCTC	0	0	0	1
tsrna-17157	TTTGCATGTATGAGGCCCCGGGT	2	2	0	0
tsrna-17154	TTTGCACGTATGAGGCCCCGGGTT	10	27	7	15
tsrna-17153	TTTGCACGTATGAGGCCCCGGGT	8	26	5	10
tsrna-17152	TTTGCACGTATGAGGCCCCGGG	1	0	0	0
tsrna-17151	TTTGCACGTATGAGGCCCCGG	2	0	0	1
tsrna-17150	TTTGCACGTATGAGGCCCCG	1	2	0	0
tsrna-17149	TTTGCACGTATGAGGCCCC	1	0	2	1
tsrna-17148	TTTGCACGTATGAGGCCC	0	0	0	0
tsrna-17147	TTTGATAGAGTAAATAATAGGAGCTTAAACCC	1	0	0	0
tsrna-17146	TTTGATAGAGTAAATAATAGGAGCT	0	0	0	0
tsrna-17142	TTTGACTGCAGATCAAGAGGTCCCTGGT	3	6	2	4

tsrna-17140	TTTGACTGCAGATCAAGAGGTCC	0	0	1	0
tsrna-17138	TTTGACTGCAGATCAAGAG	0	2	0	0
tsrna-17137	TTTGACAACATTCAAAAAAGAGTACCA	0	0	0	0
tsrna-17136	TTTGACAACATTCAAAAAAGAGTA	0	0	0	0
tsrna-17135	TTTGAATCCAGCGATCCGAGTT	0	0	0	1
tsrna-17131	TTTGAATCCAGCGATCCG	0	0	0	1
tsrna-17130	TTTGAATCCAGCAATCCGAGT	1	8	0	1
tsrna-17129	TTTCGACTCATTAAATTATGATAATCATAT	5	1	2	0
tsrna-17128	TTTCGACTCATTAAATTATGATAATCAT	2	2	0	0
tsrna-17127	TTTCCGTAGTGTAGTGGTTATCACGTTCGCCTCACACGCGAA	10	7	16	4
tsrna-17126	TTTCCGTAGTGTAGTGGTTATCACGTTCGCCTCACACG	17	9	14	6
tsrna-17125	TTTCCGTAGTGTAGTGGTTATCACGTTCGCCTCACA	19	5	10	3
tsrna-17124	TTTCCGTAGTGTAGTGGTTATCACGTTCGCCTCAC	17	7	13	2
tsrna-17123	TTTCCGTAGTGTAGTGGTTATCACGTTCGCCTCA	12	8	5	1
tsrna-17122	TTTCCGTAGTGTAGTGGTTATCACGTTCGCCTC	24	4	7	2
tsrna-17121	TTTCCGTAGTGTAGTGGTTATCACGTTCGCCT	14	1	9	2
tsrna-17120	TTTCCGTAGTGTAGTGGTTATCACGTTCGCC	16	7	9	2
tsrna-17119	TTTCCGTAGTGTAGTGGTTATCACGTTCGC	11	2	3	1
tsrna-17118	TTTCCGTAGTGTAGTGGTTATCACGTTCG	6	3	1	2
tsrna-17117	TTTCCGTAGTGTAGTGGTTATCACGTTC	15	5	3	2
tsrna-17116	TTTCCGTAGTGTAGTGGTTATCACGTT	8	0	5	2
tsrna-17115	TTTCCGTAGTGTAGTGGTTATCACG	8	2	4	2
tsrna-17114	TTTCCGTAGTGTAGTGGTTATCA	7	0	5	2
tsrna-17113	TTTCCGTAGTGTAGTGGTTATC	6	1	4	1
tsrna-17112	TTTCCGTAGTGTAGTGGTT	1	2	0	0
tsrna-17111	TTTCCGTAGTGTAGTGGTCATCACGTTCGCCT	4	2	2	1
tsrna-17110	TTTCCGTAGTGTAGTGGTCATCACGTTCGCC	5	1	4	3
tsrna-17109	TTTCCGTAGTGTAGTGGTCATCACGTTCGC	9	1	1	2
tsrna-17108	TTTCCGTAGTGTAGTGGTCATCACGTTCG	8	1	1	2
tsrna-17107	TTTCCGTAGTGTAGTGGTCATCACGTTC	2	0	1	0
tsrna-17106	TTTCCGTAGTGTAGTGGTCATCACGTT	4	1	3	3
tsrna-17105	TTTCCGTAGTGTAGTGGTCATCAC	3	1	2	1
tsrna-17104	TTTCCGTAGTGTAGTGGTCATC	2	0	2	0
tsrna-17102	TTTCCGTAGTGTAGTGGT	1	0	0	0
tsrna-17101	TTTCCGTAGTGTAGTGG	0	0	1	0
tsrna-17100	TTTCCGTAGTGTAGCGGTTATCACATTCGCCT	6	1	5	1
tsrna-17099	TTTCCGTAGTGTAGCGGTTATCACATTCGCC	4	2	5	0
tsrna-17098	TTTCCGTAGTGTAGCGGTTATCACATTC	2	3	2	0
tsrna-17093	TTTCCCCGCACAGGTTCGAATC	3	1	0	0
tsrna-17092	TTTCATATCATTGGTCGTGGTTGTAGTCCGTGCGAGAATACCA	159	157	190	51
tsrna-17091	TTTCATATCATTGGTCGTGGTTGTAGTCCGTGCGAGAATACC	28	30	24	17
tsrna-17090	TTTCATATCATTGGTCGTGGTTGTAGTCCGTGCGAGAATA	14	20	18	14
tsrna-17089	TTTCATATCATTGGTCGTGGTTGTAGTCCGTGCGAGAAT	7	12	11	6
tsrna-17088	TTTCATATCATTGGTCGTGGTTGTAGTCCGTGCGAGAA	6	18	12	13
tsrna-17087	TTTCATATCATTGGTCGTGGTTGTAGTCCGTGCGAGA	1	15	11	18
tsrna-17086	TTTCATATCATTGGTCGTGGTTGTAGTCCGTGCGAG	7	8	12	4
tsrna-17085	TTTCATATCATTGGTCGTGGTTGTAGTCCG	3	0	0	1
tsrna-17084	TTTCATATCATTGGTCGTGGTTGTAGTCC	2	1	1	0
tsrna-17083	TTTCATATCATTGGTCGTGGTTGTAGTC	2	0	1	1
tsrna-17082	TTTCATATCATTGGTCGTGGTTGTAGT	0	0	0	0
tsrna-17081	TTTCATATCATTGGTCGTGGTTGTAG	1	0	0	0
tsrna-17080	TTTCATATCATTGGTCGTGGTTGTA	0	0	0	0
tsrna-17079	TTTCATATCATTGGTCGTGGTTGT	1	0	1	0
tsrna-17075	TTTCATATCATTGGTCGTG	1	0	0	0
tsrna-17073	TTTCATAACTTTGTCAAAGTTAAATTATAGGCT	4	8	10	1
tsrna-17072	TTTCATAACTTTGTCAAAGTTAAA	2	2	1	0
tsrna-17071	TTTCATAACTTTGTCAAAGTTA	2	1	0	0
tsrna-17069	TTTCACCGCCGCGGCCCGGGTTCGATTCCCGGTCAGGGAAC(	37	321	16	3
tsrna-17068	TTTCACCGCCGCGGCCCGGGT	0	1	0	0
tsrna-17066	TTTCACCGCCGCGGCCC	0	0	0	1
tsrna-17064	TTTCACCCAGGCGGCCCGGGTTCGACTCCCGGTGTGGGAAC(	79	353	53	22
tsrna-17063	TTTCACCCAGGCGGCCCGGGTTCGACTCCCGGTGTGGGAAC(	9	17	6	0
tsrna-17062	TTTCACCCAGGCGGCCCGGGTTCGACTCCCGGTGTG	1	0	0	0
tsrna-17056	TTTCAACTTAACTTGACCGCTCTGACCA	19	31	25	12

tsrna-17055	TTTCAACTTAACTTGACCGCTCTGACC	3	5	2	0
tsrna-17054	TTTCAACTTAACTTGACCGCTCTGAC	2	2	0	1
tsrna-17053	TTTCAACTTAACTTGACCGCTCTGA	1	1	0	0
tsrna-17052	TTTCAACTTAACTTGACCGCTCTG	0	2	0	0
tsrna-17051	TTTCAACTTAACTTGACCGCTCT	0	1	1	0
tsrna-17044	TTTATGTAGCTTACCTCCTCAAA	2	0	0	1
tsrna-17043	TTTATGTAGCTTACCTCCTCA	0	0	1	0
tsrna-17042	TTTATGTAGCTTACCTCCTC	1	0	1	0
tsrna-17040	TTTATGTAGCTTACCTCC	0	0	0	1
tsrna-17039	TTTATGTAGCTTACCTC	0	0	0	1
tsrna-17038	TTTAGTGGTAGAATTCTCGCCT	1	6	1	4
tsrna-17037	TTTAGTGGTAGAATTCTCGCC	2	6	2	2
tsrna-17036	TTTAGTGGTAGAATTCTCGC	0	0	0	0
tsrna-17035	TTTAGTGGTAGAATTCTCG	0	0	0	0
tsrna-17032	TTTAGGCTCCAGTCTCTTCGGAGGCGTGGG	1	0	0	0
tsrna-17024	TTTAGGCTCCAGTCATTTCGATGGCGTGGG	0	2	1	0
tsrna-17016	TTTAGACGGGCTCACATCACCCCATAAACACCA	76	8	44	12
tsrna-17015	TTTAGACGGGCTCACATCACCCCATAAACACC	14	2	9	2
tsrna-17014	TTTAGACGGGCTCACATCACCCCATAAACAC	6	2	9	1
tsrna-17013	TTTAGACGGGCTCACATCACCCCATAAACA	6	2	6	0
tsrna-17012	TTTAGACGGGCTCACATCACCCCATAAAC	6	2	6	0
tsrna-17011	TTTAGACGGGCTCACATCACCCCATAAA	7	0	6	0
tsrna-17010	TTTAGACGGGCTCACATCACCCCATAA	5	1	6	0
tsrna-17009	TTTAGACGGGCTCACATCACCCCATA	2	1	9	0
tsrna-17008	TTTAGACGGGCTCACATCACCCCA	0	0	7	1
tsrna-17007	TTTAGACGGGCTCACATCACCCC	2	0	6	0
tsrna-17006	TTTAGACGGGCTCACATCACCC	2	2	4	0
tsrna-17005	TTTAGACGGGCTCACATCACC	3	2	8	0
tsrna-17004	TTTAGACGGGCTCACATCAC	3	0	2	0
tsrna-17003	TTTAGACGGGCTCACATCA	1	0	4	0
tsrna-17002	TTTAGACGGGCTCACATC	3	1	1	0
tsrna-17001	TTTAGACGGGCTCACAT	2	2	1	0
tsrna-17000	TTTAGACGGGCTCACA	3	0	0	1
tsrna-16999	TTTACAGTCAGAGGTTCA	0	0	2	0
tsrna-16996	TTTACACGCAGAAGGTCCTGGGT	6	35	5	2
tsrna-16995	TTTACACGCAGAAGGTCCTG	0	1	0	0
tsrna-16993	TTTACACGCAGAAGGTCC	1	0	0	1
tsrna-16992	TTTAATCTGAGGGTCCAGGGTTCAAGTCCCTGTTCGGGCGCC	19	42	9	1
tsrna-16991	TTTAATCTGAGGGTCCAG	4	4	2	0
tsrna-16990	TTTAAGTTAAAGATTAAGAGAACCAACACCTCTTTACAGTGAC	6	0	14	3
tsrna-16989	TTTAAGTTAAAGATTAAGAGAACCAACACCTCTTTACAGTGAC	4	2	6	0
tsrna-16988	TTTAAGTTAAAGATTAAGAGAACCAACACCTCTTTACAGTGAC	5	1	7	0
tsrna-16987	TTTAAGTTAAAGATTAAGAGAACCAACACCT	0	0	0	2
tsrna-16986	TTTAAGTTAAAGATTAAGAGAACCAACACC	1	1	0	0
tsrna-16985	TTTAAGTTAAAGATTAAGAGAACC	0	0	0	0
tsrna-16968	TTTAAATTAGAATCTTAGCTTTGG	5	0	6	2
tsrna-16967	TTTAAAGGATAACAGCTATCCATTGGTC	1	0	0	0
tsrna-16966	TTGTGGGTTTAAGTCCCATTGGTCTAGCCA	2	0	1	0
tsrna-16965	TTGTGGGTTTAAGTCCCATTGGTCTAGCC	1	0	0	0
tsrna-16964	TTGTGGGTTTAAGTCCCATTGGTCTAGC	1	0	0	0
tsrna-16963	TTGTGGGTTTAAGTCCCATTGGTCTAG	0	1	0	0
tsrna-16957	TTGTGGGTTCGAATCCCACCAGAGTCGCC	1	3	0	0
tsrna-16955	TTGTGGCCGCAGCAACCTCGGTT	0	1	0	0
tsrna-16954	TTGTGGCCGCAGCAACCTCGGT	1	0	0	0
tsrna-16949	TTGTGGCCGCAGCAACC	0	0	0	0
tsrna-16948	TTGTGCTCTGCACGCGTGGGTTCG	3	9	0	0
tsrna-16947	TTGTGAGTTCGATCCTCACCTGGAGCACC	2	7	1	0
tsrna-16946	TTGTGAGTTCGAGCCTCACCTGGAGCACC	0	9	4	1
tsrna-16943	TTGTGAATCTGACAACAGAGGCTTACGACCCCTTATTTACCCC	15	10	25	0
tsrna-16942	TTGTGAATCTGACAACAGAGGCTTACGACCCCTTATTTACCCC	27	10	8	0
tsrna-16941	TTGTGAATCTGACAACAGAGGCTTACGACCCCTTATTTACCC	17	16	15	0
tsrna-16940	TTGTGAATCTGACAACAGAGGCTTACGACCCCTTATTTACC	18	7	22	1
tsrna-16939	TTGTGAATCTGACAACAGAGGCTTACGACCCCTTAT	12	9	8	0
tsrna-16938	TTGTGAATCTGACAACAGAGGCTTACGACCCCT	1	4	3	0

tsrna-16937	TTGTGAATCTGACAACAGAGGCTTACGACCCC	1	1	0	0
tsrna-16936	TTGTGAATCTGACAACAGAGGCTTACGACCC	1	3	0	0
tsrna-16935	TTGTGAATCTGACAACAGAGGCTTACGACC	0	1	0	0
tsrna-16934	TTGTGAATCTGACAACAGAGGCTTACGAC	1	0	0	0
tsrna-16933	TTGTGAATCTGACAACAGAGGCTTACGA	1	0	0	0
tsrna-16931	TTGTGAATCTGACAACAGAGGCTTAC	0	1	0	0
tsrna-16929	TTGTGAATCTGACAACAGAGGCTT	0	1	0	0
tsrna-16928	TTGTGAATCTGACAACAGAGGCT	0	1	0	0
tsrna-16927	TTGTGAATCTGACAACAGAGGC	0	1	0	0
tsrna-16926	TTGTGAATCTGACAACAGAGG	0	0	1	0
tsrna-16925	TTGTGAATCTGACAACAGAG	1	1	1	1
tsrna-16924	TTGTGAATCTGACAACAGA	0	0	1	0
tsrna-16922	TTGTGAATCTGACAACA	1	1	0	0
tsrna-16921	TTGTGAATCTGACAAC	0	0	1	0
tsrna-16920	TTGTCAAAGTTAAATTATAGGCTAAATCCT	3	0	6	0
tsrna-16919	TTGTCAAAGTTAAATTATAGGCT	1	0	5	1
tsrna-16918	TTGTCAAAGTTAAATTATAGG	2	0	0	1
tsrna-16917	TTGTCAAAGTTAAATTATAG	0	0	2	0
tsrna-16916	TTGTAGTTGAAATACAACGATGGTTTTTC	0	0	0	0
tsrna-16915	TTGTAGTTGAAATACAACGATGGTT	1	0	1	0
tsrna-16912	TTGTAGTCCGTGCGAGAATACCA	1	2	0	2
tsrna-16911	TTGTAGTCCGTGCGAGAATACC	1	0	0	0
tsrna-16910	TTGTAGTCCGTGCGAGAATAC	0	0	0	0
tsrna-16909	TTGTAGTCCGTGCGAGAATA	1	0	1	0
tsrna-16906	TTGTAGTCCGTGCGAGA	0	0	0	0
tsrna-16902	TTGTAAACCGGAGATGAAAACCTTTTTCCAAGGACACCA	32	39	111	0
tsrna-16901	TTGTAAACCGGAGATGAAAACCTTTTTCCAAGGACACC	8	14	14	1
tsrna-16900	TTGTAAACCGGAGATGAAAACCTTTTTCCAAGGACA	5	11	2	3
tsrna-16899	TTGTAAACCGGAGATGAAAACCTTTTTCCAAGGAC	3	5	5	0
tsrna-16898	TTGTAAACCGGAGATGAAAACCTTTTTCC	2	10	0	1
tsrna-16897	TTGTAAACCGGAGATGAAAACCTT	0	0	0	0
tsrna-16896	TTGTAAACCGGAGATGAAAACCT	0	1	0	1
tsrna-16894	TTGTAAACCGGAGATGAAAAC	0	1	0	0
tsrna-16893	TTGTAAACCGGAGATGAAAA	1	0	0	0
tsrna-16888	TTGTAAACCAGGGGTCGCGAGTTCAA	42	102	20	13
tsrna-16887	TTGTAAACCAGGGGTCGCGAGTTCA	28	100	10	15
tsrna-16886	TTGTAAACCAGGGGTCGCGAGTT	30	93	17	15
tsrna-16885	TTGTAAACCAGGGGTCGCGAGT	27	56	11	11
tsrna-16884	TTGTAAACCAGGGGTCGCGAG	3	8	2	0
tsrna-16883	TTGTAAACCAGGGGTCGCGA	1	0	1	0
tsrna-16882	TTGTAAACCAGGGGTCGCG	0	1	1	0
tsrna-16881	TTGTAAACCAGGGGTCGC	1	0	1	0
tsrna-16880	TTGTAAACCAGGGGTCG	0	1	0	0
tsrna-16879	TTGGTTATACCCTTCCCGTACTACCA	3	8	0	1
tsrna-16878	TTGGTTATACCCTTCCCGTACTACC	2	1	0	0
tsrna-16877	TTGGTTATACCCTTCCCGTACTAC	0	0	1	0
tsrna-16876	TTGGTTATACCCTTCCCGTACTA	0	0	0	0
tsrna-16868	TTGGTGGTTTAGTGGTAGAATTCTCGC	0	0	0	0
tsrna-16867	TTGGTGGTTTAGTGGTAGAATTCTCG	0	0	0	0
tsrna-16866	TTGGTGGTTTAGTGGTAGAATTCTC	0	1	0	0
tsrna-16861	TTGGTGGTTTAGTGGTAGAA	1	0	0	0
tsrna-16859	TTGGTGGTTCGATCCCACCCAGGGACGCC	0	2	0	0
tsrna-16857	TTGGTGGTTCGAGCCCATCCAGGGACGCC	1	7	0	0
tsrna-16856	TTGGTGGTTCGAGCCCACCCAGGGACGCC	2	8	0	0
tsrna-16855	TTGGTGGTTCGAGCCCACCCAGGGACG	0	2	0	1
tsrna-16854	TTGGTGGTTCGAGCCCACCCAGGGAC	0	0	0	0
tsrna-16848	TTGGTGGTTCAGTGGTAGAATTCTCGCCTGCCACGC	77	112	30	23
tsrna-16847	TTGGTGGTTCAGTGGTAGAATTCTCGCCTGCCA	19	52	13	5
tsrna-16846	TTGGTGGTTCAGTGGTAGAATTCTCGCCTGCC	21	37	16	5
tsrna-16845	TTGGTGGTTCAGTGGTAGAATTCTCGCCTGC	11	13	9	6
tsrna-16844	TTGGTGGTTCAGTGGTAGAATTCTCGCCTG	12	14	7	3
tsrna-16843	TTGGTGGTTCAGTGGTAGAATTCTCGCCTCCC	17	19	7	13
tsrna-16842	TTGGTGGTTCAGTGGTAGAATTCTCGCCTCC	13	21	6	11
tsrna-16841	TTGGTGGTTCAGTGGTAGAATTCTCGCCTC	5	15	5	5

tsrna-16840	TTGGTGGTTCAGTGGTAGAATTCTCGCCT	9	7	5	2
tsrna-16839	TTGGTGGTTCAGTGGTAGAATTCTCGCC	7	10	10	2
tsrna-16838	TTGGTGGTTCAGTGGTAGAATTCTCGC	4	3	3	1
tsrna-16837	TTGGTGGTTCAGTGGTAGAATTCTCG	5	3	3	0
tsrna-16836	TTGGTGGTTCAGTGGTAGAATTCTC	6	8	6	1
tsrna-16835	TTGGTGGTTCAGTGGTAGAATTCT	1	4	0	0
tsrna-16834	TTGGTGGTTCAGTGGTAGAATTC	3	9	4	3
tsrna-16833	TTGGTGGTTCAGTGGTAGAATT	4	2	2	2
tsrna-16832	TTGGTGGTTCAGTGGTAGAAT	1	3	3	0
tsrna-16831	TTGGTGGTTCAGTGGTAGAA	1	1	1	0
tsrna-16830	TTGGTGGTTCAGTGGTAGA	2	1	1	0
tsrna-16826	TTGGTGGTGCAGTGGTAGAATTCTCGC	0	0	0	1
tsrna-16825	TTGGTGGTATAGTGGTTAGCATAGCTGCC	1	1	0	1
tsrna-16824	TTGGTGGTATAGTGGTTAGCATAGCTGC	0	0	0	1
tsrna-16823	TTGGTGGTATAGTGGTTAGCATAGCTG	0	1	0	0
tsrna-16822	TTGGTGGTATAGTGGTTAGCATAGC	0	0	0	0
tsrna-16817	TTGGTGGTATAGTGGTGAGCATAGCTGC	1	3	0	1
tsrna-16816	TTGGTGGTATAGTGGTGAGCATAGCTG	0	1	0	0
tsrna-16815	TTGGTGGTATAGTGGTGAGCATAGCT	0	2	0	0
tsrna-16813	TTGGTGGTATAGTGGTGAGCATAG	0	1	0	0
tsrna-16812	TTGGTGGTATAGTGGTGAGCATA	0	1	0	0
tsrna-16811	TTGGTGGTATAGTGGTGAGCAT	0	0	0	0
tsrna-16810	TTGGTGGTATAGTGGTGAGCA	0	2	0	0
tsrna-16806	TTGGTGGTATAGTGGTAAGCATAG	0	1	0	0
tsrna-16805	TTGGTGGTATAGTGGTAAGCATA	0	0	1	0
tsrna-16803	TTGGTGCAACTCCAAATAAAAGTACCA	0	3	0	0
tsrna-16798	TTGGTCTAGTGGTATGATTCTCGCTT	7	3	1	5
tsrna-16797	TTGGTCTAGTGGTATGATTCTCGC	4	1	2	2
tsrna-16795	TTGGTCTAGGGGTATGATTCTCGGTTTG	1	2	1	1
tsrna-16794	TTGGTCTAGGGGTATGATTCTCGGTTT	5	4	0	1
tsrna-16793	TTGGTCTAGGGGTATGATTCTCGGTT	0	5	2	2
tsrna-16792	TTGGTCTAGGGGTATGATTCTCGGT	3	3	0	0
tsrna-16791	TTGGTCTAGGGGTATGATTCTCGG	1	0	1	0
tsrna-16790	TTGGTCTAGGGGTATGATTCTCGCTTT	37	21	19	22
tsrna-16789	TTGGTCTAGGGGTATGATTCTCGCTTCG	30	27	13	15
tsrna-16788	TTGGTCTAGGGGTATGATTCTCGCTTC	25	28	9	17
tsrna-16787	TTGGTCTAGGGGTATGATTCTCGCTT	16	26	17	22
tsrna-16786	TTGGTCTAGGGGTATGATTCTCGCT	25	22	12	14
tsrna-16785	TTGGTCTAGGGGTATGATTCTCGC	10	17	10	9
tsrna-16784	TTGGTCTAGGGGTATGATTCTCG	0	1	0	0
tsrna-16782	TTGGTCTAGGGGTATGATTCT	0	1	0	0
tsrna-16776	TTGGTCGTGGTTGTAGTCCGTGCGAGAATACCA	70	113	88	26
tsrna-16775	TTGGTCGTGGTTGTAGTCCGTGCGAGAATACC	14	22	11	8
tsrna-16774	TTGGTCGTGGTTGTAGTCCGTGCGAGAATAC	8	10	4	7
tsrna-16773	TTGGTCGTGGTTGTAGTCCGTGCGAGAATA	2	8	3	5
tsrna-16772	TTGGTCGTGGTTGTAGTCCGTGCGAGAAT	2	6	6	6
tsrna-16771	TTGGTCGTGGTTGTAGTCCGTGCGAGAA	3	8	5	1
tsrna-16770	TTGGTCGTGGTTGTAGTCCGTGCGAGA	2	9	5	5
tsrna-16769	TTGGTCGTGGTTGTAGTCCGTGCGAG	1	6	2	1
tsrna-16768	TTGGTCGTGGTTGTAGTCCGTGCGA	1	4	3	1
tsrna-16767	TTGGTCGTGGTTGTAGTCCGTGCG	2	4	1	1
tsrna-16766	TTGGTCGTGGTTGTAGTCCGTGC	1	3	0	0
tsrna-16765	TTGGTCGTGGTTGTAGTCCGTG	0	3	2	2
tsrna-16764	TTGGTCGTGGTTGTAGTCCGT	0	0	0	0
tsrna-16763	TTGGTCGTGGTTGTAGTCCG	0	0	0	1
tsrna-16762	TTGGTCGTGGTTGTAGTCC	0	0	0	0
tsrna-16761	TTGGTCGTGGTTGTAGTC	0	1	0	0
tsrna-16759	TTGGTCGTGGTTGTAG	0	0	0	0
tsrna-16747	TTGGTAGAGCATCAGAC	1	0	0	0
tsrna-16746	TTGGTAGAGCATCAGA	2	0	0	0
tsrna-16740	TTGGGTGCTAATGGTGGAGTTAAAGACTTTTTCTCTGACCA	38	43	20	24
tsrna-16739	TTGGGTGCTAATGGTGGAGTTAAAGACTTTTTCTCTGACC	8	21	6	2
tsrna-16738	TTGGGTGCTAATGGTGGAGTTAAAGACTTTTTCTCT	8	18	3	0
tsrna-16737	TTGGGTGCTAATGGTGGAGTTAAAGACTTT	3	5	3	1

tsrna-16736	TTGGGTGCTAATGGTGGAGTTAAAGACT	3	5	4	1
tsrna-16735	TTGGGTGCTAATGGTGGAGTTAAAGAC	0	0	0	1
tsrna-16734	TTGGGTGCTAATGGTGGAGTTAAAGA	2	7	2	0
tsrna-16733	TTGGGTGCTAATGGTGGAGTTAAAG	2	4	0	0
tsrna-16732	TTGGGTGCTAATGGTGGAGTTAAA	0	4	0	1
tsrna-16731	TTGGGTGCTAATGGTGGAGTTAA	1	1	0	1
tsrna-16730	TTGGGTGCTAATGGTGGAGTTA	3	2	0	1
tsrna-16729	TTGGGTGCTAATGGTGGAGTT	0	0	0	0
tsrna-16723	TTGGGTGCGAGAGGTCCCGGGTT	4	66	1	21
tsrna-16722	TTGGGTGCGAGAGGTCCCGGGT	8	39	5	16
tsrna-16721	TTGGGTGCGAGAGGTCCCGGG	0	1	0	0
tsrna-16719	TTGGGTGCGAGAGGTCCCG	0	1	0	0
tsrna-16718	TTGGGTGCGAGAGGTCCC	0	2	0	0
tsrna-16716	TTGGGTCCGAGAGGTCCCGGGTT	0	2	0	0
tsrna-16709	TTGGGGTTTCCCCGCGCAGGT	0	3	0	0
tsrna-16708	TTGGGGGGTTCGATTCCTTCCTTTTTTGCC	0	6	1	1
tsrna-16707	TTGGGGGGTTCGATTCCTTCCTTTTTTGC	0	4	0	0
tsrna-16706	TTGGGGGGTTCGATTCCTTCCTTTTTTG	1	3	1	0
tsrna-16705	TTGGGGGGTTCGATTCCTTC	1	0	0	0
tsrna-16704	TTGGGGGGTTCGATTCCTT	0	0	0	0
tsrna-16703	TTGGGGGGTTCGATTCCT	0	0	0	0
tsrna-16702	TTGGGGGGTTCGATTCC	0	0	2	0
tsrna-16699	TTGGGAGAGCGTTAGACTGAAGAT	0	0	0	1
tsrna-16698	TTGGGAGAGCGTTAGACTGAAGA	0	1	0	0
tsrna-16696	TTGGGAGAGCGTTAGACTGAA	0	1	0	0
tsrna-16692	TTGGCCTCCTAAGCCAGGGATTGTGG	1	3	4	0
tsrna-16691	TTGGATTCTCAGGGATGGGTTCGATTCTCATAGTCCTAGCCA	11	23	19	22
tsrna-16690	TTGGATTCTCAGGGATGGGTTCG	1	2	1	0
tsrna-16689	TTGGACTTCTAATTCAAAGGTTGTGGGT	13	48	10	4
tsrna-16686	TTGGACTGTAAATCTAAAGACAGGGGTTAGGCCTCT	5	7	3	3
tsrna-16685	TTGGACTGTAAATCTAAAGACAGGGGTTAG	0	1	0	0
tsrna-16684	TTGGACTGTAAATCTAAAGACAGGGGTT	0	0	0	0
tsrna-16682	TTGGACTGTAAATCTAAAGACAGGGG	0	0	1	0
tsrna-16673	TTGCGTTCAGTTGATGCAGAGTGGGGTTTT	2	2	1	2
tsrna-16672	TTGCGTTCAGTTGATGCAGAGTGGG	0	2	1	0
tsrna-16670	TTGCGTTCAGTTGATGCAGAGTG	0	0	3	0
tsrna-16668	TTGCGTTCAGTTGATGCAGAG	0	0	1	0
tsrna-16662	TTGCGTGTTCAAGTCACGTCGGGGTCACC	1	3	0	0
tsrna-16655	TTGCGTGTTCAAATCACGTCGGGGTCACC	0	2	0	0
tsrna-16651	TTGCGACCCGGGTTCGATTCCCGGGCGGCGCACCA	18	66	1	0
tsrna-16650	TTGCGACCCGGGTTCGATTCCCGGGCGGCGCACC	6	13	0	0
tsrna-16649	TTGCGACCCGGGTTCGATTCCCGGGC	1	1	0	0
tsrna-16648	TTGCGACCCGGGTTCGATTCCCGGG	0	6	0	0
tsrna-16646	TTGCATGTATGAGGCCTCGGGT	4	11	2	1
tsrna-16645	TTGCATGTATGAGGCCTC	0	1	0	0
tsrna-16644	TTGCATGTATGAGGCCCCGGGT	3	5	0	0
tsrna-16641	TTGCACGTATGAGGCCCCGGGTTCA	6	13	3	2
tsrna-16640	TTGCACGTATGAGGCCCCGGGTTC	3	9	3	6
tsrna-16639	TTGCACGTATGAGGCCCCGGGTT	6	10	0	1
tsrna-16638	TTGCACGTATGAGGCCCCGGGT	6	8	2	0
tsrna-16637	TTGCACGTATGAGGCCCCGGG	1	0	0	0
tsrna-16636	TTGCACGTATGAGGCCCCGG	0	1	0	0
tsrna-16632	TTGCACGCGTGGGTTCGAATCCCATCCTCGTCGC	0	1	1	0
tsrna-16631	TTGCACGCGTGGGTTCGAAT	0	2	0	0
tsrna-16630	TTGCAATACTTAATTTCTGCCA	3	1	0	0
tsrna-16629	TTGCAATACTTAATTTCTGCC	1	0	1	0
tsrna-16628	TTGCAATACTTAATTTCTGC	1	0	0	0
tsrna-16625	TTGCAAATTCGAAGAAGCAGCTTCAAACCTGCCGGGGCTT	1	1	1	0
tsrna-16622	TTGCAAATTCGAAGAAGCAGCTT	1	0	0	0
tsrna-16617	TTGATTAGGGTGCTTAGCTGTTAACTAAGTGTTTGT	7	0	7	0
tsrna-16616	TTGATTAGGGTGCTTAGCTGTTAAC	1	1	2	0
tsrna-16615	TTGATTAGGGTGCTTAGCTGTTA	2	1	2	0
tsrna-16614	TTGATTAGGGTGCTTAGCTGTT	1	1	1	0
tsrna-16613	TTGATTAGGGTGCTTAGCTGT	1	1	0	0

tsrna-16612	TTGATTAGGGTGCTTAGCTG	0	0	0	0
tsrna-16608	TTGATGCAGAGTGGGGTTTTGCAGTCC	2	1	1	0
tsrna-16607	TTGATGCAGAGTGGGGTTTTGCAGTC	0	0	0	0
tsrna-16606	TTGATGCAGAGTGGGGTTTTGCA	0	0	0	0
tsrna-16605	TTGATGCAGAGTGGGGTTTTGC	0	0	0	0
tsrna-16604	TTGATGCAGAGTGGGGTTTTG	0	0	0	0
tsrna-16603	TTGATAGAGTAAATAATAGGAGCTTAAACCC	1	1	2	1
tsrna-16602	TTGATAGAGTAAATAATAGGAGCT	0	0	0	0
tsrna-16596	TTGACTGCAGATCAAGAGGTCCCTGGTT	4	12	3	7
tsrna-16595	TTGACTGCAGATCAAGAGGTCCCTGGT	1	3	1	4
tsrna-16594	TTGACTGCAGATCAAGAGGTCCCTGG	1	2	1	0
tsrna-16593	TTGACTGCAGATCAAGAGGTCCCT	0	1	0	1
tsrna-16592	TTGACTGCAGATCAAGAGGTCCCCGGT	3	6	2	2
tsrna-16591	TTGACTGCAGATCAAGAGGTCCCCGG	1	2	0	0
tsrna-16590	TTGACTGCAGATCAAGAGGTCCCCG	0	2	0	0
tsrna-16589	TTGACTGCAGATCAAGAGGTCCCCAGT	0	1	1	0
tsrna-16587	TTGACTGCAGATCAAGAGGTCCC	0	0	1	0
tsrna-16585	TTGACTGCAGATCAAGAG	0	1	0	0
tsrna-16582	TTGACTGCAGATCAAAAGGTCCCTGGT	7	10	3	2
tsrna-16581	TTGACCGCTCTGACCA	0	1	0	1
tsrna-16579	TTGACAACATTCAAAAAAGAGTACCA	0	1	0	0
tsrna-16576	TTGAATTGCAAATTCGAAGAAGCAGCTTCAAACCTGCCGGGG	0	3	1	0
tsrna-16575	TTGAATTGCAAATTCGAAGAAGCAGCTTCAAACCTGCCGGGG	0	5	1	0
tsrna-16574	TTGAATCCCACCGCTGCCACCA	0	1	0	0
tsrna-16573	TTGAATCCAGCGATCCGAGTT	0	1	0	0
tsrna-16569	TTGAAGCCAGTTGATTAGGGTGCTTAGCTG	0	0	0	0
tsrna-16567	TTGAAATCCATTGGGGTTTCC	1	64	1	2
tsrna-16566	TTGAAATCCATTGGGGTTTC	0	67	1	1
tsrna-16565	TTGAAATCCATTGGGG	1	6	0	0
tsrna-16564	TTGAAATACAACGATGGTTTTTCATATC	0	2	0	0
tsrna-16563	TTGAAACCAGCTTTGGGGGGTTCGA	1	1	0	0
tsrna-16562	TTGAAACCAGCTTTGGGGGGTTCG	0	2	0	0
tsrna-16561	TTGAAACCAGCTTTGGGGGGTTC	0	1	2	0
tsrna-16560	TTGAAACCAGCTTTGGGGGGTT	0	1	0	0
tsrna-16559	TTGAAACCAGCTTTGGGGGGT	0	1	0	0
tsrna-16557	TTCTTGCGACCCGGGTTCGATTCCCGGGCGGCGCACC	5	11	2	0
tsrna-16556	TTCTTGCGACCCGGGTTCGATTCCCGGGCGGCG	0	4	0	0
tsrna-16555	TTCTTGCGACCCGGGTTCGATTCCCGGGCGGC	0	4	1	0
tsrna-16554	TTCTTGCGACCCGGGTTCGATTCCCGGGCG	1	5	0	0
tsrna-16553	TTCTTGCGACCCGGGTTCGA	0	1	0	0
tsrna-16552	TTCTTGCGACCCGGGTTCG	0	1	0	0
tsrna-16551	TTCTTGCGACCCGGGTTC	0	1	0	0
tsrna-16548	TTCTGTAGTGTAGTGGTTATCACGTTCGCCT	0	1	0	0
tsrna-16547	TTCTGTAGTGTAGTGGTTATCACGTTCGCC	0	0	0	0
tsrna-16545	TTCTGTAGTGTAGTGGTTATCACGTTCG	0	0	0	0
tsrna-16543	TTCTGTAGTGTAGTGGTTATCACGTT	1	0	0	0
tsrna-16541	TTCTGTAGTGTAGTGGTTATCACG	0	0	1	0
tsrna-16539	TTCTGTAGTGTAGTGGTTATC	0	0	0	0
tsrna-16535	TTCTGGTCTCCGGATGGAGGCGTGGGTTCGAATCCC	14	62	6	2
tsrna-16534	TTCTGGTCTCCGGATGGAGGCGTGGGTTCG	10	49	3	3
tsrna-16533	TTCTGGTCTCCGGATGGAGGCGTGGG	1	5	2	0
tsrna-16532	TTCTGGTCTCCGGATGGAGGCGTGG	0	4	1	0
tsrna-16531	TTCTGGTCTCCGGATGGAGGCGT	0	3	3	0
tsrna-16530	TTCTGGTCTCCGGATGGAGGCG	0	1	0	0
tsrna-16525	TTCTGGTCTCCGGATGG	0	0	0	1
tsrna-16523	TTCTGGTCTCCAATGGAGGCGTGGG	2	2	2	1
tsrna-16521	TTCTGGTCTCCAATGGAGGCGTG	0	1	0	0
tsrna-16520	TTCTGGTCTCCAATGGAGGCG	0	1	0	0
tsrna-16510	TTCTCGCTGGGGCCTCCA	27	41	13	10
tsrna-16509	TTCTCGCTGGGGCCTCC	1	7	0	1
tsrna-16508	TTCTCGCCTGCCACGCGGGAGGCCCGGGT	5	33	5	2
tsrna-16507	TTCTCGCCTGCCACGCGGGAGGCCCGG	0	7	0	0
tsrna-16506	TTCTCGCCTGCCACGCGGGAGGCCCG	1	4	2	0
tsrna-16505	TTCTCGCCTGCCACGCGGGAGGCCC	0	6	1	0

tsrna-16504	TTCTCGCCTGCCACGCGGGA	1	4	1	0
tsrna-16503	TTCTCGCCTGCCACGCGG	1	8	0	1
tsrna-16502	TTCTCGCCTGCCACGCG	0	5	0	0
tsrna-16501	TTCTCGCCTGCCACGC	1	4	0	0
tsrna-16500	TTCTCATAGTCCTAGCCA	1	0	0	0
tsrna-16499	TTCTCATAGTCCTAGCC	0	1	0	0
tsrna-16498	TTCTCATAGTCCTAGC	0	0	0	1
tsrna-16497	TTCTCAGGGATGGTTCGATTCTCATAGTCCTAGCCA	15	19	16	19
tsrna-16496	TTCTCAGGGATGGTTCGATTCT	4	5	6	0
tsrna-16495	TTCTCAGGGATGGTTCGATTC	3	3	3	0
tsrna-16494	TTCTCAGGGATGGTTCG	0	1	0	0
tsrna-16493	TTCTAGGTTCGACTCCTGGCTGGCTCGC	1	0	0	0
tsrna-16492	TTCTAGGTTCGACTCCTGGCTGGCTCG	0	0	0	0
tsrna-16491	TTCTAATTCAAAGGTTGTGGGTTCGAGTCC	4	9	0	3
tsrna-16490	TTCTAATTCAAAGGTTGTGGGTTCGAATCC	6	7	3	2
tsrna-16489	TTCTAATTCAAAGGTTGTGGGT	0	3	0	0
tsrna-16486	TTCTAATTCAAAGGTTCCGGGTTCGAGTCCCGGCGGAGTCGC	3	11	0	1
tsrna-16485	TTCTAATTCAAAGGTTCCGGGTTCGAGTCC	0	3	0	1
tsrna-16484	TTCTAATTCAAAGGTTCCGGGTT	1	3	0	2
tsrna-16483	TTCTAATTCAAAGGTTCCGGGT	1	2	0	0
tsrna-16477	TTCGGGTGTGAGAGGTCCCGGGT	0	2	0	0
tsrna-16475	TTCGGGTGCGAGAGGTCCCGGGTT	0	7	2	0
tsrna-16474	TTCGGGTGCGAGAGGTCCCGGGT	1	2	0	0
tsrna-16473	TTCGGGTGCGAGAGGTCCCGG	1	1	0	0
tsrna-16471	TTCGGGTGCGAGAGGTCCC	1	0	0	0
tsrna-16469	TTCGGCTGTTAACCGA	7	9	2	0
tsrna-16468	TTCGGCGCTCTCACCGCCGCGGCCCGGGTTCGATTCC	0	0	0	0
tsrna-16467	TTCGGCGCTCTCACCGCCGCGGCCCGGGTTCGATTC	0	0	0	0
tsrna-16466	TTCGGCGCTCTCACCGCCGCGGCCCGGGTTCGATT	1	3	0	0
tsrna-16465	TTCGGCGCTCTCACCGCCGCGGCCCGGGTTCGAT	0	3	0	2
tsrna-16464	TTCGGCGCTCTCACCGCCGCGGCCCGGGTTCG	0	1	0	0
tsrna-16463	TTCGGCGCTCTCACCGCCGCGGCCCGGGTTC	0	2	1	0
tsrna-16462	TTCGGCGCTCTCACCGCCGCGGCCCGGGTT	0	1	1	0
tsrna-16461	TTCGGCGCTCTCACCGCCGCGGCCCGGGT	0	0	0	0
tsrna-16460	TTCGGCGCTCTCACCGCCGCGGCCCGGG	0	4	0	0
tsrna-16459	TTCGGCGCTCTCACCGCCGCGGCCCGG	0	1	0	0
tsrna-16458	TTCGGCGCTCTCACCGCCGCGGCCCG	0	0	1	1
tsrna-16457	TTCGGCGCTCTCACCGCCGCGGCCC	0	0	0	0
tsrna-16456	TTCGGCGCTCTCACCGCC	0	0	1	0
tsrna-16453	TTCGGATCAGAAGATTGCAGGT	0	0	0	0
tsrna-16450	TTCGGATCAGAAGATTGAGGGT	3	2	2	0
tsrna-16447	TTCGATTCTCATAGTCCTAGCCA	0	0	0	0
tsrna-16446	TTCGATTCTCATAGTCCTAGCC	0	1	0	0
tsrna-16445	TTCGATTCTCATAGTCCTAGC	0	0	1	0
tsrna-16443	TTCGATTCCTTCCTTTTTTGCCA	0	0	0	0
tsrna-16442	TTCGATTCCTTCCTTTTTTGCC	0	0	0	0
tsrna-16440	TTCGATTCCTTCCTTTTTTG	0	0	0	0
tsrna-16439	TTCGATTCCGGCTCGAAGGACCA	77	163	26	22
tsrna-16438	TTCGATTCCGGCTCGAAGGACC	1	11	1	0
tsrna-16437	TTCGATTCCGGCTCGAAGGAC	0	0	0	0
tsrna-16434	TTCGATTCCCGGTCAGGGAACCA	12	301	3	0
tsrna-16433	TTCGATTCCCGGTCAGGGAACC	1	7	0	0
tsrna-16432	TTCGATTCCCGGTCAGGGAAC	0	6	0	0
tsrna-16431	TTCGATTCCCGGTCAGGGAA	0	1	0	0
tsrna-16430	TTCGATTCCCGGTCAGGGA	0	1	0	0
tsrna-16426	TTCGATTCCCGGGCGGCGCACCA	14	58	7	3
tsrna-16425	TTCGATTCCCGGGCGGCGCACC	4	7	1	0
tsrna-16424	TTCGATTCCCGGGCGGCGCAC	1	0	0	0
tsrna-16418	TTCGATTCCCGGCCCATGCACCA	20	39	8	18
tsrna-16417	TTCGATTCCCGGCCCATGCACC	5	3	2	2
tsrna-16415	TTCGATTCCCGGCCAATGCACCA	39	136	4	10
tsrna-16414	TTCGATTCCCGGCCAATGCACC	3	13	2	0
tsrna-16413	TTCGATTCCCGGCCAATGCAC	0	2	0	0
tsrna-16411	TTCGATTCCCGGCCAATGC	0	1	0	0

tsrna-16410	TTCGATTCCCGGCCAACGCACCA	48	98	28	18
tsrna-16409	TTCGATTCCCGGCCAACGCACC	4	14	3	3
tsrna-16408	TTCGATTCCCGGCCAACGCAC	0	2	0	0
tsrna-16407	TTCGATTCCCGGCCAACGCA	0	1	0	0
tsrna-16406	TTCGATTCCCGGCCAACGC	0	1	0	0
tsrna-16405	TTCGATTCCCCGACGGGGAGCCA	1	12	0	0
tsrna-16404	TTCGATTCCCCGACGGGGAGCC	0	1	0	0
tsrna-16403	TTCGATTCCCCGACGGGGAGC	0	1	0	0
tsrna-16397	TTCGATTCCAGCTCGAAGGACCA	0	0	0	0
tsrna-16395	TTCGATCCTCGCTGGGGCCTCCA	10	9	0	2
tsrna-16394	TTCGATCCTCACCTGGAGCACCA	1	4	1	0
tsrna-16393	TTCGATCCTCACCTGGAGCACC	0	1	0	0
tsrna-16392	TTCGATCCTCACACGGGGCACCA	0	5	0	0
tsrna-16390	TTCGATCCCGGGTTTCGGCACCA	29	99	13	10
tsrna-16389	TTCGATCCCGGGTTTCGGCACC	5	11	1	2
tsrna-16383	TTCGATCCCGGGCGGAAACACCA	22	62	14	5
tsrna-16381	TTCGATCCCCGTACTGGCCACCA	4	7	6	0
tsrna-16380	TTCGATCCCCGTACGGGCCACCA	11	64	5	2
tsrna-16379	TTCGATCCCCGTACGGGCCACC	1	2	1	0
tsrna-16377	TTCGATCCCCGGCATCTCCACCA	242	368	125	77
tsrna-16376	TTCGATCCCCGGCATCTCCACC	9	21	5	2
tsrna-16369	TTCGATCCCCGGCACCTCCACCA	132	281	78	36
tsrna-16368	TTCGATCCCCGACACCTCCACCA	11	27	0	7
tsrna-16367	TTCGATCCCCAGTACCTCCACCA	37	47	17	17
tsrna-16366	TTCGATCCCCAGCATCTCCACCA	13	16	3	2
tsrna-16365	TTCGATCCCCAGCATCTCCACC	0	1	0	0
tsrna-16362	TTCGATCCCACCCAGGGACGCCA	3	7	0	0
tsrna-16361	TTCGATAGCTCAGTTGGTAGAGCGGAGGAC	0	2	0	0
tsrna-16360	TTCGATAGCTCAGTTGGTAGA	0	0	1	1
tsrna-16359	TTCGATAGCTCAGCTGGTAGAGCGGAGGACT	2	0	0	0
tsrna-16358	TTCGATAGCTCAGCTGGTAGA	3	2	2	0
tsrna-16357	TTCGAGTCTCGGTGGAACCTCCA	60	144	23	29
tsrna-16356	TTCGAGTCTCGGTGGAACCTCC	2	13	3	0
tsrna-16355	TTCGAGTCTCGGTGGAACCTC	0	0	1	0
tsrna-16351	TTCGAGTCCTGCCGCGGTCGCCA	1	7	2	0
tsrna-16346	TTCGAGTCCCTTCGTGGTCGCCA	1	2	0	0
tsrna-16345	TTCGAGTCCCGGCGGAGTCGCCA	0	4	1	0
tsrna-16340	TTCGAGTCCCATCTGGGTCGCCA	1	9	1	0
tsrna-16339	TTCGAGTCCCATCTGGGGTGCCA	1	3	0	0
tsrna-16337	TTCGAGTCCCACCTGGGGTGCCA	1	6	0	0
tsrna-16336	TTCGAGTCCCACCAGAGTCGCCA	0	7	0	0
tsrna-16335	TTCGAGCCTCAGAGAGGGCACCA	0	1	0	0
tsrna-16334	TTCGAGCCTCACCTGGAGCACCA	1	3	0	1
tsrna-16333	TTCGAGCCTCACCTGGAGCACC	0	2	0	0
tsrna-16330	TTCGAGCCTCACCTGGAGC	0	1	0	0
tsrna-16329	TTCGAGCCTCACCTGGA	0	1	0	0
tsrna-16327	TTCGAGCCCCAGTGGAACCACCA	32	37	25	14
tsrna-16326	TTCGAGCCCCAGTGGAACCACC	4	5	0	0
tsrna-16325	TTCGAGCCCCAGTGGAACCAC	1	0	0	0
tsrna-16324	TTCGAGCCCCAGTGGAACCA	0	0	0	0
tsrna-16323	TTCGAGCCCCAGTGGAACC	0	1	0	0
tsrna-16322	TTCGAGCCCCACGTTGGGCGCCA	11	206	3	1
tsrna-16317	TTCGAGCCCACCCAGGGACGCCA	3	81	2	0
tsrna-16316	TTCGAGCCCACCCAGGGACGCC	0	1	0	1
tsrna-16312	TTCGACTCCTGGCTGGCTCGCCA	41	117	12	4
tsrna-16311	TTCGACTCCTGGCTGGCTCGCC	1	7	0	1
tsrna-16310	TTCGACTCCTGGCTGGCTCGC	1	0	0	0
tsrna-16305	TTCGACTCCCGGTGTGGGAACCA	73	280	24	18
tsrna-16304	TTCGACTCCCGGTGTGGGAACC	5	3	0	0
tsrna-16303	TTCGACTCCCGGTGTGGGAAC	0	3	0	0
tsrna-16297	TTCGACTCCCGGTATGGGAACCA	8	66	7	3
tsrna-16296	TTCGACTCCCGGTATGGGAAC	0	2	0	0
tsrna-16293	TTCGACTCATTAAATTATGATAATCATATT	2	0	1	0
tsrna-16291	TTCGAATCTCGGTGGGACCTCCA	55	93	22	19

tsrna-16290	TTCGAATCCTGTTCGTGACGCCA	4	9	3	2
tsrna-16289	TTCGAATCCTGTCGGCTACGCCA	5	13	7	8
tsrna-16288	TTCGAATCCTGCTCACAGCGCCA	4	17	4	2
tsrna-16287	TTCGAATCCTGCTCACAGCGCC	0	1	0	1
tsrna-16285	TTCGAATCCTGCCGACTACGCCA	111	237	37	16
tsrna-16284	TTCGAATCCTGCCGACTACGCC	4	18	0	0
tsrna-16283	TTCGAATCCTGCCGACTACGC	0	1	0	0
tsrna-16281	TTCGAATCCGGCTCGGAGGACCA	0	1	0	0
tsrna-16280	TTCGAATCCGGCTCGAAGGACCA	22	48	12	6
tsrna-16279	TTCGAATCCGGCTCGAAGGACC	2	5	0	0
tsrna-16270	TTCGAATCCCTTCGTGGTTGCCA	0	8	0	0
tsrna-16269	TTCGAATCCCTCCGTGGTTACCA	2	13	1	0
tsrna-16268	TTCGAATCCCATCCTCGTCGCCA	5	15	2	0
tsrna-16267	TTCGAATCCCATCCTCGTCGCC	0	1	1	0
tsrna-16266	TTCGAATCCCATCCTCGTCGC	0	1	0	0
tsrna-16265	TTCGAATCCCATCCTCGTCG	0	1	0	0
tsrna-16264	TTCGAATCCCAGTAGAGCCTCCA	14	20	5	10
tsrna-16263	TTCGAATCCCAGCGGTGCCTCCA	38	46	8	4
tsrna-16262	TTCGAATCCCAGCGGTGCCTCC	2	1	0	0
tsrna-16256	TTCGAATCCCAGCGGGGCCTCCA	0	4	0	1
tsrna-16253	TTCGAATCCCACTTCTGACACCA	11	28	11	11
tsrna-16252	TTCGAATCCCACTTCTGACACC	6	3	3	0
tsrna-16251	TTCGAATCCCACTTCTGACAC	0	0	0	0
tsrna-16250	TTCGAATCCCACTTCTGACA	0	0	0	0
tsrna-16248	TTCGAATCCCACTCCTGACACCA	90	99	60	29
tsrna-16247	TTCGAATCCCACTCCTGACACC	6	10	2	2
tsrna-16246	TTCGAATCCCACTCCTGACAC	0	2	0	0
tsrna-16242	TTCGAATCCCACCTTCGTCGCCA	2	4	0	1
tsrna-16241	TTCGAATCCCACCGCTGCCACCA	13	30	9	4
tsrna-16240	TTCGAATCCCACCGCTGCCACC	0	5	0	0
tsrna-16235	TTCGAATCCCACCAGAGTCGCCA	4	6	0	0
tsrna-16233	TTCGAATCCCACCACTGCCACCA	2	20	0	0
tsrna-16232	TTCGAATCACGTCGGGGTCACCA	6	9	4	2
tsrna-16231	TTCGAATCACGTCGGGGTCACC	0	1	1	0
tsrna-16230	TTCGAATCACGTCGGGGTCAC	0	0	0	1
tsrna-16224	TTCGAAGAAGCAGCTTCAAACCTGCCGGGGCTT	1	1	0	0
tsrna-16223	TTCGAAGAAGCAGCTTCAAACCTGCCGGGGCT	0	0	1	0
tsrna-16222	TTCGAAGAAGCAGCTTCAAACCTGCCGGGG	0	1	0	0
tsrna-16219	TTCGAACCTCAGAGGGGGCACCA	0	7	0	0
tsrna-16215	TTCGAACCCTGCTCGCTGCGCCA	7	24	6	4
tsrna-16214	TTCGAACCCTGCTCGCTGCGCC	0	5	0	0
tsrna-16211	TTCGAACCCCGTCCGTGCCTCCA	5	11	4	2
tsrna-16206	TTCGAACCCCACTTCTGGTACCA	0	7	0	0
tsrna-16205	TTCGAACCCCACTCCTGGTACCA	0	1	0	0
tsrna-16201	TTCGAAACCGGGCGGAAACACCA	315	719	118	37
tsrna-16200	TTCGAAACCGGGCGGAAACACC	69	218	25	2
tsrna-16199	TTCGAAACCGGGCGGAAACAC	1	4	0	0
tsrna-16198	TTCGAAACCGGGCGGAAACA	1	1	0	0
tsrna-16193	TTCGAAACCGGGCAGAAGCACCA	31	143	9	5
tsrna-16192	TTCGAAACCGGGCAGAAGCACC	0	6	0	0
tsrna-16191	TTCGAAACCGGGCAGAAGCAC	0	1	0	0
tsrna-16190	TTCGAAACCGGGCAGAAGCA	0	0	0	0
tsrna-16186	TTCCTTCCTTTTTTGCCA	1	0	0	0
tsrna-16184	TTCCTGGTTTTCACCCAGGTGGCCCG	3	6	3	0
tsrna-16183	TTCCTGGTTTTCACCCAGGCGGCCCGGGTTCGACTCCCGGTC	0	3	0	0
tsrna-16182	TTCCTGGTTTTCACCCAGGCGGCCCG	2	4	0	1
tsrna-16180	TTCCTGGCCAATGCACCA	0	0	3	0
tsrna-16179	TTCCTCTTCTTAACACCA	1	0	0	0
tsrna-16176	TTCCTCGTTAGTATAGTGGTGAGT	3	8	3	5
tsrna-16175	TTCCTCGTTAGTATAGTGGTGAG	0	1	0	2
tsrna-16174	TTCCTCGTTAGTATAGTGG	1	0	0	0
tsrna-16173	TTCCGTAGTGTAGTGGTTATCACGTTCGCCTCAC	7	4	3	1
tsrna-16172	TTCCGTAGTGTAGTGGTTATCACGTTCGCCTCA	2	0	1	3
tsrna-16171	TTCCGTAGTGTAGTGGTTATCACGTTCGCCTC	5	1	3	0

tsrna-16170	TTCCGTAGTGTAGTGGTTATCACGTTCGCCT	1	0	5	7
tsrna-16169	TTCCGTAGTGTAGTGGTTATCACGTTCGCC	2	3	6	3
tsrna-16168	TTCCGTAGTGTAGTGGTTATCACGTTCGC	3	1	0	0
tsrna-16167	TTCCGTAGTGTAGTGGTTATCACGTTCG	6	1	1	0
tsrna-16166	TTCCGTAGTGTAGTGGTTATCACGTTC	2	0	3	0
tsrna-16165	TTCCGTAGTGTAGTGGTTATCACGTT	1	1	2	1
tsrna-16164	TTCCGTAGTGTAGTGGTTATCACG	1	1	3	1
tsrna-16163	TTCCGTAGTGTAGTGGTTATCA	2	0	0	1
tsrna-16162	TTCCGTAGTGTAGTGGTTATC	3	1	1	0
tsrna-16160	TTCCGTAGTGTAGTGGTCATCACGTTCGCCT	2	1	0	1
tsrna-16159	TTCCGTAGTGTAGTGGTCATCACGTTCGCC	1	1	4	0
tsrna-16158	TTCCGTAGTGTAGTGGTCATCACGTT	1	0	1	3
tsrna-16157	TTCCGTAGTGTAGTGGTCATC	1	0	0	1
tsrna-16155	TTCCGTAGTGTAGTGGT	0	0	0	1
tsrna-16153	TTCCGTAGTGTAGCGGTTATCACATTCGCCT	1	1	1	0
tsrna-16152	TTCCGTAGTGTAGCGGTTATCACATTCGCC	0	0	2	0
tsrna-16151	TTCCGTAGTGTAGCGGTTATCACATT	0	2	1	0
tsrna-16150	TTCCGGGTTCGAGTCCCGGCGGAGTCGCCA	2	1	0	0
tsrna-16146	TTCCGGCTCGAAGGACCA	60	115	16	12
tsrna-16145	TTCCGGCTCGAAGGACC	2	1	1	1
tsrna-16143	TTCCGGATCAGAAGATTGAGGGTTCG	3	11	3	2
tsrna-16142	TTCCGGATCAGAAGATTGAGGGTT	2	10	6	0
tsrna-16141	TTCCGGATCAGAAGATTGAGGGT	2	4	3	1
tsrna-16135	TTCCCTGGTGGTCTAGTGGTTAGGATTCGG	15	34	26	21
tsrna-16134	TTCCCTGGTGGTCTAGTGGTTAGGATTCG	25	30	21	18
tsrna-16133	TTCCCTGGTGGTCTAGTGGTTAGG	19	27	29	18
tsrna-16132	TTCCCTGGTGGTCTAGTGGTTAG	20	35	30	16
tsrna-16131	TTCCCTGGTGGTCTAGTGGTTA	31	25	20	13
tsrna-16130	TTCCCTGGTGGTCTAGTGG	1	13	0	4
tsrna-16129	TTCCCTGGTGGTCTAGTG	3	15	3	4
tsrna-16127	TTCCCGGTCAGGGAACCA	3	6	1	1
tsrna-16124	TTCCCGGGCGGCGCACCA	8	30	6	0
tsrna-16123	TTCCCGGGCGGCGCACC	7	5	1	0
tsrna-16121	TTCCCGGCCCATGCACCA	4	10	2	1
tsrna-16120	TTCCCGGCCCATGCACC	2	1	0	0
tsrna-16118	TTCCCGGCCACTGCACCA	0	3	0	0
tsrna-16117	TTCCCGGCCAATGCACCA	0	0	2	0
tsrna-16116	TTCCCGGCCAATGCACC	1	0	0	0
tsrna-16110	TTCCCCGCACAGGTTCGAATCCT	5	15	2	0
tsrna-16109	TTCCCCGCACAGGTTCGAA	0	0	0	0
tsrna-16105	TTCCCATTCTTGCGACCCGGGTT	1	5	2	0
tsrna-16104	TTCCCATTCTTGCGACCCGGGT	1	2	0	0
tsrna-16102	TTCCCATTCTTGCGACCCGG	1	1	0	0
tsrna-16099	TTCCCATTCTTGCGACC	0	0	0	0
tsrna-16097	TTCCCATATGGTCTAGCGGTTAGGATTCCT	1	1	2	0
tsrna-16096	TTCCCATATGGTCTAGCGGTTAGGATT	4	0	1	1
tsrna-16095	TTCCCATATGGTCTAGCGGTTAGG	0	1	2	3
tsrna-16094	TTCCCATATGGTCTAGCGGTTAG	1	1	2	1
tsrna-16093	TTCCCACATGGTCTAGCGGTTAGGATTCCT	1	8	8	13
tsrna-16092	TTCCCACATGGTCTAGCGGTTAGGATT	5	6	8	16
tsrna-16091	TTCCCACATGGTCTAGCGGTTAGG	3	4	10	15
tsrna-16090	TTCCATGGTGTAATGGTTAGCACTCTGGACTCT	1	1	0	0
tsrna-16089	TTCCATGGTGTAATGGTTAGCACTCTGGACT	0	2	1	1
tsrna-16088	TTCCATGGTGTAATGGTTAGCACTCTGGAC	1	0	0	1
tsrna-16087	TTCCATGGTGTAATGGTTAGCACTCTGGA	0	0	1	0
tsrna-16083	TTCCATGGTGTAATGGTGAGCACTCTGGAC	1	0	0	0
tsrna-16079	TTCCATAGTGTAGTGGTTATCACGTCTGCTTTAC	4	0	7	0
tsrna-16078	TTCCATAGTGTAGTGGTTATCACGTCTGCTTT	1	0	1	0
tsrna-16077	TTCCATAGTGTAGTGGTTATCACGTCTGCTT	1	0	0	0
tsrna-16075	TTCCATAGTGTAGTGGTTATCACGTCTGC	0	0	1	0
tsrna-16074	TTCCATAGTGTAGTGGTTATCACGTCTG	1	0	0	0
tsrna-16072	TTCCATAGTGTAGCGGTTATCACGTCTG	0	0	1	0
tsrna-16071	TTCCAGGTTCGACTCCTGGCTGGCTCGCC	0	1	0	1
tsrna-16070	TTCCAGGTTCGACTCCTGGCTGGCTCGC	0	0	0	0

tsrna-16069	TTCCAGGTTCGACTCCTGGCTGGCTCG	0	1	0	0
tsrna-16061	TTCCAATTAACTAGTTTTGACAACATTCAAAAAAGAGTACCA	0	1	0	0
tsrna-16058	TTCCAAGCAGTTGACCCGGGTTCGATTCCCGGCCAACGCACC	59	133	29	15
tsrna-16057	TTCCAAGCAGTTGACCCGGGT	0	6	2	0
tsrna-16056	TTCCAAGCAGTTGACCCGG	1	1	0	0
tsrna-16053	TTCATATTGAATTGCAAATTCG	0	0	0	0
tsrna-16052	TTCATATTGAATTGCAAATTC	1	0	0	0
tsrna-16049	TTCATATCCGCGTGGGT	0	2	0	0
tsrna-16048	TTCATATCATTGGTCGTGGTTGTAGTCCGTGCGAGAATACCA	137	147	221	46
tsrna-16047	TTCATATCATTGGTCGTGGTTGTAGTCCGTGCGAGAATACC	13	36	23	21
tsrna-16046	TTCATATCATTGGTCGTGGTTGTAGTCCGTGCGAGAATA	16	18	8	12
tsrna-16045	TTCATATCATTGGTCGTGGTTGTAGTCCGTGCGAGAAT	4	16	11	12
tsrna-16044	TTCATATCATTGGTCGTGGTTGTAGTCCGTGCGAGAA	6	16	7	9
tsrna-16043	TTCATATCATTGGTCGTGGTTGTAGTCCGTGCGAGA	7	15	9	11
tsrna-16042	TTCATATCATTGGTCGTGGTTGTAGTCCGTGCGAG	2	10	8	4
tsrna-16041	TTCATATCATTGGTCGTGGTTGTAGTCCGTGCGA	2	5	3	3
tsrna-16040	TTCATATCATTGGTCGTGGTTGTAGTCCGTGCG	6	3	4	3
tsrna-16039	TTCATATCATTGGTCGTGGTTGTAGTCCGT	1	0	3	1
tsrna-16038	TTCATATCATTGGTCGTGGTTGTAGTCCG	1	3	0	0
tsrna-16037	TTCATATCATTGGTCGTGGTTGTAGTCC	1	1	2	0
tsrna-16036	TTCATATCATTGGTCGTGGTTGTAGTC	1	2	0	0
tsrna-16035	TTCATATCATTGGTCGTGGTTGTAGT	1	0	0	1
tsrna-16034	TTCATATCATTGGTCGTGGTTGTAG	0	1	1	0
tsrna-16033	TTCATATCATTGGTCGTGGTTGTA	0	1	0	0
tsrna-16032	TTCATATCATTGGTCGTGGTTGT	0	0	0	0
tsrna-16028	TTCATATCATTGGTCGTGG	0	0	0	0
tsrna-16025	TTCATAACTTTGTCAAAGTTAAATTATAGGCT	5	6	5	1
tsrna-16024	TTCATAACTTTGTCAAAGTTAAATTATAGGC	4	5	10	0
tsrna-16023	TTCAGTTGATGCAGAGTGGGGTTTTGCAGTCCTTACCA	21	8	26	3
tsrna-16022	TTCAGTTGATGCAGAGTGGGGTTTTGCAGTCCTTACC	6	5	5	1
tsrna-16021	TTCAGTTGATGCAGAGTGGGGTTTTGCAGTCCTTAC	3	2	6	1
tsrna-16020	TTCAGTTGATGCAGAGTGGGGTTTTGCAGTCCT	0	8	1	5
tsrna-16019	TTCAGTTGATGCAGAGTGGGGTTTTGCAGTC	1	2	0	0
tsrna-16018	TTCAGTTGATGCAGAGTGGGGTTTTGCAGT	1	2	2	0
tsrna-16017	TTCAGTTGATGCAGAGTGGGGTTTTGCAG	1	1	0	0
tsrna-16016	TTCAGTTGATGCAGAGTGGGGTTTTGCA	1	2	1	0
tsrna-16015	TTCAGTTGATGCAGAGTGGGGTTTTGC	2	0	0	0
tsrna-16014	TTCAGTTGATGCAGAGTGGGGTTTTG	0	0	1	0
tsrna-16013	TTCAGTTGATGCAGAGTGGGGTTTT	0	0	1	0
tsrna-16012	TTCAGTTGATGCAGAGTGGGGTTT	0	0	1	1
tsrna-16011	TTCAGTTGATGCAGAGTGGGGTT	0	0	0	0
tsrna-16010	TTCAGTTGATGCAGAGTGGGGT	0	0	0	0
tsrna-16009	TTCAGTTGATGCAGAGTGGGG	0	0	0	0
tsrna-16007	TTCAGTTGATGCAGAGTGG	0	1	1	0
tsrna-16006	TTCAGTGGTAGAATTTTC	1	0	0	0
tsrna-16005	TTCAGTGGTAGAATTCTTGCCTGC	0	0	0	0
tsrna-16001	TTCAGTGGTAGAATTCTCGCCTT	4	5	3	4
tsrna-16000	TTCAGTGGTAGAATTCTCGCCTGCCACGC	43	102	26	23
tsrna-15999	TTCAGTGGTAGAATTCTCGCCTGCCA	8	32	5	4
tsrna-15998	TTCAGTGGTAGAATTCTCGCCTGCC	14	43	8	5
tsrna-15997	TTCAGTGGTAGAATTCTCGCCTGC	2	12	4	0
tsrna-15996	TTCAGTGGTAGAATTCTCGCCTG	3	6	5	2
tsrna-15995	TTCAGTGGTAGAATTCTCGCCTCC	2	14	3	3
tsrna-15994	TTCAGTGGTAGAATTCTCGCCTC	5	4	3	3
tsrna-15993	TTCAGTGGTAGAATTCTCGCCT	3	11	2	1
tsrna-15992	TTCAGTGGTAGAATTCTCGCC	2	3	0	0
tsrna-15991	TTCAGTGGTAGAATTCTCGC	1	1	1	0
tsrna-15985	TTCAGGTCGCAGTCTCCCCTG	0	1	0	0
tsrna-15979	GCTGTTAACCGAAAGGTTGGTGGT	2	33	1	3
tsrna-15978	GCTGTTAACCGAAAGGTTGGTGG	0	4	0	0
tsrna-15977	GCTGTTAACCGAAAGGTTGGTG	0	0	0	0
tsrna-15976	GCTGTTAACCGAAAGGTTGGT	0	2	0	0
tsrna-15975	GCTGTTAACCGAAAGGTTGG	0	1	0	0
tsrna-15974	GCTGTGATGGCCGAGTGGTTAAGGTGTTGG	1	3	0	1

tsrna-15973	GCTGTGATGGCCGAGTGGTTAAGGTGTTG	1	4	0	0
tsrna-15972	GCTGTGATGGCCGAGTGGTTAAGGT	1	4	1	0
tsrna-15971	GCTGTGATGGCCGAGTGGTTAAGGCGTTGG	1	5	0	0
tsrna-15970	GCTGTGATGGCCGAGTGGTTAAGGCGTTG	1	3	1	0
tsrna-15969	GCTGTGATGGCCGAGTGGTTAAGGCGT	0	2	0	0
tsrna-15968	GCTGTGATGGCCGAGTGGTTAAGGC	0	4	0	0
tsrna-15967	GCTGTGATGGCCGAGTGGTTAAGG	1	6	1	0
tsrna-15966	GCTGTGATGGCCGAGTGGTTAAG	1	5	1	0
tsrna-15965	GCTGTGATGGCCGAGTGGTTAA	1	6	0	0
tsrna-15964	GCTGTGATGGCCGAGTGGTTA	0	3	0	0
tsrna-15963	GCTGTGATGGCCGAGTGGTT	1	5	1	0
tsrna-15962	GCTGTGATGGCCGAGTGGT	1	6	0	0
tsrna-15961	GCTGTGATGGCCGAGTGG	0	5	0	0
tsrna-15960	GCTGTGATGGCCGAGTG	1	0	0	0
tsrna-15959	GCTGTGATGGCCGAGT	0	1	0	0
tsrna-15958	GCTGGTTCGATTCCGGCTCGAAGGACCA	80	151	31	25
tsrna-15955	GCTGGTTCGAATCCGGCTCGAAGGACCA	29	53	8	8
tsrna-15953	GCTGGTGTAGTGGTATCATGCAAGATTCCCATTCTT	5	3	7	0
tsrna-15952	GCTGGTGTAGTGGTATCATGCAAGATTCCC	2	2	2	1
tsrna-15951	GCTGGTGTAGTGGTATCATGCAAGATTCC	3	1	4	1
tsrna-15950	GCTGGTGTAGTGGTATCATGCAAGATTC	3	1	0	1
tsrna-15949	GCTGGTGTAGTGGTATCATGCAAGATT	1	2	2	0
tsrna-15948	GCTGGTGTAGTGGTATCATGCAAGAT	0	2	0	0
tsrna-15947	GCTGGTGTAGTGGTATCATGCAAGA	3	3	2	0
tsrna-15946	GCTGGTGTAGTGGTATCATGCAAG	1	2	0	0
tsrna-15945	GCTGGTGTAGTGGTATCATGCAA	0	0	0	0
tsrna-15944	GCTGGTGTAGTGGTATCATGCA	0	0	0	0
tsrna-15943	GCTGGTGTAGTGGTATCATGC	1	0	0	0
tsrna-15942	GCTGGTGTAGTGGTATCATG	1	0	0	0
tsrna-15941	GCTGGTGTAGTGGTATCAT	1	0	0	0
tsrna-15940	GCTGGTGTAGTGGTATCA	0	0	1	0
tsrna-15939	GCTGGTGTAGTGGTATC	0	0	0	1
tsrna-15935	GCTGGGGATTGTGGGTTCGAGTCCCATCTGGGTCGCCA	5	16	0	1
tsrna-15934	GCTGGGGATTGTGGGTTCGAGTCCCATCTGGGTCGCC	2	2	2	0
tsrna-15933	GCTGGGGATTGTGGGTTCGAGTCCCATCTG	1	4	1	1
tsrna-15932	GCTGGGGATTGTGGGTTCGAGTC	0	1	0	1
tsrna-15931	GCTGGGGATTGTGGGTTCGAG	1	1	0	0
tsrna-15930	GCTGGGGATTGTGGGTTCG	0	0	0	0
tsrna-15929	GCTGGGGATTGTGGGTTC	1	1	0	0
tsrna-15928	GCTGGGGATTGTGGGTT	0	1	1	0
tsrna-15925	GCTGCCTTCCAAGCAGTTGACCCGGGT	25	37	15	12
tsrna-15924	GCTGCCTTCCAAGCAGTTGACCCGGG	2	0	1	2
tsrna-15923	GCTGCCTTCCAAGCAGTTGACCCGG	3	1	0	1
tsrna-15922	GCTGCCTTCCAAGCAGTTGACCCG	2	2	1	0
tsrna-15921	GCTGCCTTCCAAGCAGTTGACCC	1	0	0	1
tsrna-15920	GCTGATTTGCGTTCAGTTGATGCAG	0	0	0	1
tsrna-15919	GCTGATTTGCGTTCAGTTGATG	1	0	0	0
tsrna-15917	GCTGAGTGAAGCATTGGACTGTAAATCTA	1	1	0	0
tsrna-15916	GCTGAGTGAAGCATTGGACTGTAAATCT	0	3	0	0
tsrna-15915	GCTGAGTGAAGCATTGGACTGTAAATC	0	0	1	0
tsrna-15914	GCTGAGTGAAGCATTGGACTGTAAAT	0	1	0	2
tsrna-15913	GCTGAGTGAAGCATTGGACTGTAAA	0	1	0	0
tsrna-15912	GCTGAGTGAAGCATTGGACTGTAA	1	3	1	0
tsrna-15911	GCTGAGTGAAGCATTGGACTGTA	0	1	0	0
tsrna-15909	GCTGAGTGAAGCATTGGACTG	0	1	0	0
tsrna-15907	GCTGAGTGAAGCATTGGAC	0	1	0	0
tsrna-15902	GCTCTGTGGCGCAATGGATAGCGCATTG	1	0	1	0
tsrna-15897	GCTCTGCACGCGTGGGT	0	1	0	0
tsrna-15895	GCTCTCACCGCCGCGGCCCGGGTTCGATTCCCGGTCAGGGA	33	305	21	6
tsrna-15894	GCTCTCACCGCCGCGGCCCGGGTTCGATTCCCGGTCAGG	0	2	0	0
tsrna-15890	GCTCTCACCGCCGCGGCCCGGGTT	1	3	0	1
tsrna-15889	GCTCTCACCGCCGCGGCCCGGGT	0	1	0	0
tsrna-15887	GCTCTCACCGCCGCGGCCCGG	0	1	0	0
tsrna-15880	GCTCGTTGGTCTAGGGGTATGATTCTCGGT	7	2	0	0

tsrna-15879	GCTCGTTGGTCTAGGGGTATGATTCTCGG	0	2	0	0
tsrna-15878	GCTCGTTGGTCTAGGGGTATGATTCTCGCTT	23	24	15	21
tsrna-15877	GCTCGTTGGTCTAGGGGTATGATTCTCGCT	22	28	17	21
tsrna-15876	GCTCGTTGGTCTAGGGGTATGATTCTCGC	12	9	4	16
tsrna-15875	GCTCGTTGGTCTAGGGGTATGATTCTCG	0	2	1	0
tsrna-15874	GCTCGTTGGTCTAGGGGTATGATTCTC	1	0	1	0
tsrna-15873	GCTCGTTGGTCTAGGGGTATGATTCT	0	0	0	0
tsrna-15871	GCTCGTTGGTCTAGGGGTATGATT	0	0	0	0
tsrna-15858	GCTCCGAGGTGATTTTCATATTGAATTGC	1	3	0	0
tsrna-15857	GCTCCGAGGTGATTTTCATATTGAATTG	2	0	0	0
tsrna-15856	GCTCCGAGGTGATTTTCATATTGAATT	1	0	0	0
tsrna-15852	GCTCCGAGGTGATTTTCATATTG	1	0	0	0
tsrna-15844	GCTCCATAGCTCAGTGGT	2	0	0	0
tsrna-15842	GCTCCATAGCTCAGGGGT	0	1	0	0
tsrna-15826	GCTCCAGTCTCTTCGGGGGCGTGGGTTCG	1	0	1	0
tsrna-15824	GCTCCAGTCTCTTCGGGGGCGTGGG	0	0	0	0
tsrna-15819	GCTCCAGTCTCTTCGGGGGC	0	1	0	0
tsrna-15815	GCTCCAGTCTCTTCGGAGGCGTGGGTTCG	0	0	0	0
tsrna-15814	GCTCCAGTCTCTTCGGAGGCGTGGGTTC	1	2	1	0
tsrna-15813	GCTCCAGTCTCTTCGGAGGCGTGG	0	0	0	0
tsrna-15812	GCTCCAGTCTCTTCGGAGGCGTG	0	1	0	0
tsrna-15804	GCTCCAGTCATTTCGATGGCGTGGGTTCG	3	6	3	0
tsrna-15803	GCTCAGTTGGTAGAGCGGAGGACTGTAG	0	0	0	1
tsrna-15800	GCTCAGTTGGTAGAGCATCAG	1	0	0	0
tsrna-15797	GCTCAGTGGTAGAGCGCGTGCTT	4	18	2	0
tsrna-15796	GCTCAGTGGTAGAGCGCGTGC	2	20	1	0
tsrna-15795	GCTCAGTGGTAGAGCATTTGACTGC	0	1	1	0
tsrna-15794	GCTCAGTGGTAGAGCATTTGACTG	5	2	0	0
tsrna-15793	GCTCAGTGGTAGAGCATTTGACTAC	1	0	0	1
tsrna-15792	GCTCAGTGGTAGAGCATTTGACTA	0	3	1	1
tsrna-15791	GCTCAGTGGTAGAGCATTTGACT	1	0	1	0
tsrna-15790	GCTCAGTGGTAGAGCATTTGAC	3	1	0	0
tsrna-15789	GCTCAGTGGTAGAGCATTTGA	1	2	0	0
tsrna-15788	GCTCAGTGGTAGAGCATTTG	0	0	1	0
tsrna-15787	GCTCAGTGGTAGAGCATTTAACT	0	1	0	2
tsrna-15786	GCTCAGTGGTAGAGCATTT	0	2	0	0
tsrna-15784	GCTCAGTGGGTAGAGCATTTGACTGC	0	3	0	0
tsrna-15783	GCTCAGTGGGTAGAGCATTTGACTG	3	0	0	0
tsrna-15782	GCTCAGTGGGTAGAGCATTTGACT	0	2	1	0
tsrna-15781	GCTCAGTCGGTAGAGCATGGGACTCTTAATCC	23	22	10	6
tsrna-15780	GCTCAGTCGGTAGAGCATGGGACTCTTA	9	18	5	2
tsrna-15779	GCTCAGTCGGTAGAGCATGGGACTCTT	11	20	7	2
tsrna-15778	GCTCAGTCGGTAGAGCATGGGACTCT	15	24	7	1
tsrna-15777	GCTCAGTCGGTAGAGCATGGGACTC	16	26	8	2
tsrna-15776	GCTCAGTCGGTAGAGCATGGGACT	12	13	9	4
tsrna-15775	GCTCAGTCGGTAGAGCATGGGAC	13	19	3	3
tsrna-15774	GCTCAGTCGGTAGAGCATGGGA	7	15	5	1
tsrna-15773	GCTCAGTCGGTAGAGCATGGG	3	3	0	0
tsrna-15772	GCTCAGTCGGTAGAGCATGG	1	1	0	0
tsrna-15771	GCTCAGTCGGTAGAGCATGAGACTC	15	32	12	9
tsrna-15770	GCTCAGTCGGTAGAGCATGAGACT	8	26	8	3
tsrna-15769	GCTCAGTCGGTAGAGCATGAGAC	19	32	6	7
tsrna-15768	GCTCAGTCGGTAGAGCATGAGA	2	6	3	2
tsrna-15767	GCTCAGTCGGTAGAGCATGAG	2	3	0	0
tsrna-15765	GCTCAGTCGGTAGAGCATG	0	2	0	0
tsrna-15764	GCTCAGTCGGTAGAGCATCAGACTTTTA	20	7	8	6
tsrna-15763	GCTCAGTCGGTAGAGCATCAGACTTTT	11	15	14	7
tsrna-15762	GCTCAGTCGGTAGAGCATCAGACTTT	7	16	3	2
tsrna-15761	GCTCAGTCGGTAGAGCATCAGACTT	9	8	6	1
tsrna-15760	GCTCAGTCGGTAGAGCATCAGACT	8	10	3	0
tsrna-15759	GCTCAGTCGGTAGAGCATCAGAC	9	13	5	2
tsrna-15758	GCTCAGTCGGTAGAGCATCAGA	13	11	3	0
tsrna-15757	GCTCAGTCGGTAGAGCATCAG	1	4	2	1
tsrna-15756	GCTCAGTCGGTAGAGCATCA	3	5	1	0

tsrna-15755	GCTCAGTCGGTAGAGCATC	3	1	2	0
tsrna-15754	GCTCAGTCGGTAGAGCAT	0	1	0	0
tsrna-15751	GCTCAGGTGGTAGAGCATTTGACTGC	1	0	1	0
tsrna-15750	GCTCAGGTGGTAGAGCATTTGACTG	1	0	0	0
tsrna-15749	GCTCAGGTGGTAGAGCATTTGACT	1	0	0	1
tsrna-15748	GCTCAGGGGTTAGAGCACTGGT	1	1	0	0
tsrna-15746	GCTCAGGGGTAGAGCATTTGACTGC	0	1	0	0
tsrna-15745	GCTCAGGGGTAGAGCATTTGACTG	1	0	0	0
tsrna-15744	GCTCAGGGGTAGAGCATTTGACT	0	1	0	0
tsrna-15739	GCTCACATCACCCCATAAACACCA	5	2	1	0
tsrna-15738	GCTCACATCACCCCATAAACACC	1	0	0	0
tsrna-15730	GCTCACAAGAACTGCTAACTCATGCCCCCATGTCTAACAACAT	5	1	2	1
tsrna-15729	GCTCACAAGAACTGCTAACTCATGCCCCCATGTCTA	1	2	2	2
tsrna-15728	GCTCACAAGAACTGCTAACTCATGCCCCC	0	0	1	0
tsrna-15727	GCTCACAAGAACTGCTAACTCATGCCCC	0	0	1	0
tsrna-15726	GCTCACAAGAACTGCTAACTCATGCC	1	3	0	0
tsrna-15725	GCTCACAAGAACTGCTAACTCATGC	3	0	2	0
tsrna-15724	GCTCACAAGAACTGCTAACTCATG	3	2	0	0
tsrna-15723	GCTCACAAGAACTGCTAACTCAT	0	0	1	1
tsrna-15722	GCTCACAAGAACTGCTAACTCA	0	0	0	0
tsrna-15721	GCTCACAAGAACTGCTAACTC	0	1	1	0
tsrna-15720	GCTCACAAGAACTGCTAACT	1	1	0	1
tsrna-15719	GCTCACAAGAACTGCTAAC	0	0	1	0
tsrna-15714	GCTATCGGGCCCATACCCCGAAAATGTTGG	4	1	2	0
tsrna-15713	GCTATCGGGCCCATACCCCGAAAATGTTG	2	0	3	0
tsrna-15712	GCTATCGGGCCCATACCCCGAAAATG	4	1	1	0
tsrna-15711	GCTATCGGGCCCATACCCCGAAA	0	0	0	0
tsrna-15710	GCTATCGGGCCCATACCCCGAA	0	1	0	0
tsrna-15709	GCTATCGGGCCCATACCCCGA	0	1	0	1
tsrna-15708	GCTATCGGGCCCATACCCCG	0	0	0	0
tsrna-15707	GCTATCGGGCCCATACCCC	0	0	3	0
tsrna-15706	GCTATCGGGCCCATACCC	0	0	1	0
tsrna-15704	GCTATCGGGCCCATAC	0	1	0	1
tsrna-15703	GCTATCCATTGGTCTTAGGCCCCA	0	0	1	0
tsrna-15702	GCTATCCATTGGTCTTAGGCCCC	0	0	0	0
tsrna-15694	GCTAGGATTCGGCGCTTTCACCGCCGCGGCCC	7	21	7	14
tsrna-15693	GCTAGGATTCGGCGCTTTCA	2	8	5	2
tsrna-15692	GCTAGGATTCGGCGCTTTC	1	2	0	1
tsrna-15691	GCTAGGATTCGGCGCTTT	0	3	0	1
tsrna-15690	GCTAGGATTCGGCGCTT	0	3	0	1
tsrna-15689	GCTAGGATTCGGCGCT	1	0	2	0
tsrna-15688	GCTAGCTCAGTCGGTAGAGCATGGGACTCT	18	16	6	2
tsrna-15687	GCTAGCTCAGTCGGTAGAGCATGGGACTC	9	14	10	3
tsrna-15686	GCTAGCTCAGTCGGTAGAGCATGGGACT	18	13	8	5
tsrna-15685	GCTAGCTCAGTCGGTAGAGCATGGGAC	8	16	11	3
tsrna-15684	GCTAGCTCAGTCGGTAGAGCATGGGA	4	14	1	1
tsrna-15683	GCTAGCTCAGTCGGTAGAGCATGGG	4	2	2	0
tsrna-15682	GCTAGCTCAGTCGGTAGAGCATGG	0	2	1	0
tsrna-15681	GCTAGCTCAGTCGGTAGAGCATGAGACTCT	17	21	5	7
tsrna-15680	GCTAGCTCAGTCGGTAGAGCATGAGACTC	16	29	2	6
tsrna-15679	GCTAGCTCAGTCGGTAGAGCATGAGACT	7	14	3	3
tsrna-15678	GCTAGCTCAGTCGGTAGAGCATGAGAC	18	19	7	3
tsrna-15677	GCTAGCTCAGTCGGTAGAGCATGAGA	0	5	1	1
tsrna-15676	GCTAGCTCAGTCGGTAGAGCATGAG	1	5	0	0
tsrna-15675	GCTAGCTCAGTCGGTAGAGCATGA	1	2	1	1
tsrna-15674	GCTAGCTCAGTCGGTAGAGCATG	0	1	0	0
tsrna-15673	GCTAGCTCAGTCGGTAGAGCAT	0	0	1	0
tsrna-15672	GCTAGCTCAGTCGGTAGAGCA	0	1	0	0
tsrna-15666	GCTAATGGTGGAGTTAAAGACTTTTTCTCTGACCA	25	52	19	12
tsrna-15665	GCTAATGGTGGAGTTAAAGACTTTTTCTCTGACC	17	23	7	1
tsrna-15664	GCTAATGGTGGAGTTAAAGACTTTTTCTCTGAC	5	16	3	0
tsrna-15663	GCTAATGGTGGAGTTAAAGACTTTTTCTCT	11	13	3	1
tsrna-15662	GCTAATGGTGGAGTTAAAGACTTTTTCTC	3	20	5	1
tsrna-15661	GCTAATGGTGGAGTTAAAGACTTTTTCT	3	10	1	1

tsrna-15660	GCTAATGGTGGAGTTAAAGACTTTTTC	9	9	5	0
tsrna-15659	GCTAATGGTGGAGTTAAAGACTTTTT	3	13	2	1
tsrna-15658	GCTAATGGTGGAGTTAAAGACTTTT	2	5	2	0
tsrna-15657	GCTAATGGTGGAGTTAAAGACTTT	3	1	2	1
tsrna-15656	GCTAATGGTGGAGTTAAAGACTT	2	3	2	0
tsrna-15655	GCTAATGGTGGAGTTAAAGACT	3	7	2	0
tsrna-15654	GCTAATGGTGGAGTTAAAGAC	0	4	0	0
tsrna-15653	GCTAATGGTGGAGTTAAAGA	2	1	1	0
tsrna-15652	GCTAATGGTGGAGTTAAAG	5	7	0	0
tsrna-15651	GCTAATGGTGGAGTTAAA	1	2	0	0
tsrna-15650	GCTAATGGTGGAGTTAA	1	3	0	1
tsrna-15649	GCTAATGGTGGAGTTA	0	3	0	0
tsrna-15648	GCTAATCCATTGTGCTTTGCACGCGTGGGT	1	9	2	0
tsrna-15639	GCTAACTCATGCCCCCATGTCTAACAACATGGCTTTCTCACCA	18	6	39	1
tsrna-15638	GCTAACTCATGCCCCCATGTCTAACAACATGGCTTTCTCACC	9	3	9	1
tsrna-15637	GCTAACTCATGCCCCCATGTCTAACAACATGGCTTTCTCA	3	0	3	0
tsrna-15636	GCTAACTCATGCCCCCATGTCTAACAACATGGCT	2	1	1	0
tsrna-15635	GCTAACTCATGCCCCCATGTCTAACAACAT	0	1	0	0
tsrna-15634	GCTAACTCATGCCCCCATGTCTAACAACA	0	0	0	0
tsrna-15633	GCTAACTCATGCCCCCATGTCTAACAAC	0	0	0	0
tsrna-15632	GCTAACTCATGCCCCCATGTCTAACA	0	0	0	0
tsrna-15631	GCTAACTCATGCCCCCATGTCTAAC	0	0	0	0
tsrna-15629	GCTAACTCATGCCCCCATGTCTA	0	1	0	0
tsrna-15628	GCTAACTCATGCCCCCATGTCT	1	0	0	0
tsrna-15621	GCTAAATCCTATATATCTTACCA	0	1	0	0
tsrna-15615	GCTAAATAAGCTATCGGGCCCATACCCCG	1	3	2	0
tsrna-15614	GCTAAATAAGCTATCGGGCCCATACC	0	0	1	1
tsrna-15613	GCTAAATAAGCTATCGGGCCCATA	1	0	1	0
tsrna-15612	GCTAAATAAGCTATCGGGCCCAT	0	0	0	0
tsrna-15611	GCTAAATAAGCTATCGGGCCCA	0	2	0	0
tsrna-15608	GCTAAATAAGCTATCGGGC	1	0	0	0
tsrna-15604	GCGTTTGGCTGTTAACTAAA	0	0	0	1
tsrna-15598	GCGTTGGTGGTTTAGTGGTAGAATTCTCGC	2	0	2	0
tsrna-15597	GCGTTGGTGGTTTAGTGGTAGAATTCTCG	1	0	1	0
tsrna-15591	GCGTTGGTGGTGTAGTGGTGAGCA	3	1	0	0
tsrna-15590	GCGTTGGTGGTGTAGTGGTGAGC	0	1	0	0
tsrna-15582	GCGTTGGTGGTATAGTGGTTAGCATAGCTGCCTTCC	7	2	9	1
tsrna-15581	GCGTTGGTGGTATAGTGGTTAGCATAGCTGCCTTC	3	6	6	1
tsrna-15580	GCGTTGGTGGTATAGTGGTTAGCATAGCTGCCTT	5	1	5	1
tsrna-15579	GCGTTGGTGGTATAGTGGTTAGCATAGCTGCCT	7	6	6	0
tsrna-15578	GCGTTGGTGGTATAGTGGTTAGCATAGCTGCC	4	4	3	1
tsrna-15577	GCGTTGGTGGTATAGTGGTTAGCATAGCTGC	3	4	0	0
tsrna-15576	GCGTTGGTGGTATAGTGGTTAGCATAGCTG	1	2	1	0
tsrna-15575	GCGTTGGTGGTATAGTGGTTAGCATAGCT	0	2	0	0
tsrna-15574	GCGTTGGTGGTATAGTGGTTAGCATAGC	0	0	0	0
tsrna-15573	GCGTTGGTGGTATAGTGGTTAGCATAG	0	1	0	1
tsrna-15572	GCGTTGGTGGTATAGTGGTTAGCATA	0	1	1	0
tsrna-15571	GCGTTGGTGGTATAGTGGTTAGCAT	0	1	0	0
tsrna-15570	GCGTTGGTGGTATAGTGGTTAGCA	0	1	0	0
tsrna-15569	GCGTTGGTGGTATAGTGGTTAGC	0	1	0	0
tsrna-15568	GCGTTGGTGGTATAGTGGTTAG	0	1	0	0
tsrna-15567	GCGTTGGTGGTATAGTGGTTA	0	2	0	0
tsrna-15566	GCGTTGGTGGTATAGTGGTT	0	0	1	0
tsrna-15565	GCGTTGGTGGTATAGTGGTGAGCATAGT	3	15	1	0
tsrna-15564	GCGTTGGTGGTATAGTGGTGAGCATAGCTGCCTTCCAAGCAG	10	24	10	2
tsrna-15563	GCGTTGGTGGTATAGTGGTGAGCATAGCTGCCTTCC	4	14	5	2
tsrna-15562	GCGTTGGTGGTATAGTGGTGAGCATAGCTGCCTTC	2	12	0	0
tsrna-15561	GCGTTGGTGGTATAGTGGTGAGCATAGCTGCCTT	3	12	2	1
tsrna-15560	GCGTTGGTGGTATAGTGGTGAGCATAGCTGCCT	0	8	1	0
tsrna-15559	GCGTTGGTGGTATAGTGGTGAGCATAGCTGCC	4	6	0	0
tsrna-15558	GCGTTGGTGGTATAGTGGTGAGCATAGCTGC	2	6	0	1
tsrna-15557	GCGTTGGTGGTATAGTGGTGAGCATAGCTG	1	5	0	2
tsrna-15556	GCGTTGGTGGTATAGTGGTGAGCATAGCT	1	11	1	1
tsrna-15555	GCGTTGGTGGTATAGTGGTGAGCATAGC	0	10	0	2

tsrna-15554	GCGTTGGTGGTATAGTGGTGAGCATAG	0	8	1	0
tsrna-15553	GCGTTGGTGGTATAGTGGTGAGCATA	2	5	1	0
tsrna-15552	GCGTTGGTGGTATAGTGGTGAGCAT	1	11	1	1
tsrna-15551	GCGTTGGTGGTATAGTGGTGAGCA	0	8	1	1
tsrna-15550	GCGTTGGTGGTATAGTGGTGAGC	1	5	0	0
tsrna-15549	GCGTTGGTGGTATAGTGGTGAG	0	3	0	0
tsrna-15548	GCGTTGGTGGTATAGTGGTGA	0	1	1	1
tsrna-15547	GCGTTGGTGGTATAGTGGTG	1	0	0	0
tsrna-15546	GCGTTGGTGGTATAGTGGTAAGCATAGCTGCCTTCC	2	2	3	2
tsrna-15545	GCGTTGGTGGTATAGTGGTAAGCATAGCTGCCTTC	4	4	3	1
tsrna-15544	GCGTTGGTGGTATAGTGGTAAGCATAGCTGCCT	4	3	3	1
tsrna-15543	GCGTTGGTGGTATAGTGGTAAGCATAGCTGCC	2	2	1	2
tsrna-15542	GCGTTGGTGGTATAGTGGTAAGCATAGCTGC	2	2	1	4
tsrna-15541	GCGTTGGTGGTATAGTGGTAAGCATAGCTG	1	1	0	1
tsrna-15540	GCGTTGGTGGTATAGTGGTAAGCATAGCT	1	0	0	3
tsrna-15539	GCGTTGGTGGTATAGTGGTAAGCATAGC	0	2	0	0
tsrna-15538	GCGTTGGTGGTATAGTGGTAAGCATAG	0	1	0	1
tsrna-15537	GCGTTGGTGGTATAGTGGTAAGCATA	0	0	0	1
tsrna-15535	GCGTTGGTGGTATAGTGGTAAGCA	0	1	0	0
tsrna-15534	GCGTTGGTGGTATAGTGGTAAGC	2	0	0	0
tsrna-15533	GCGTTGGTGGTATAGTGGTAAG	0	1	0	1
tsrna-15532	GCGTTGGTGGTATAGTGGTAA	0	3	0	0
tsrna-15531	GCGTTGGTGGTATAGTGGTA	0	1	0	0
tsrna-15529	GCGTTGGTGGTATAGTGG	1	3	0	0
tsrna-15528	GCGTTGGTGGTATAGTG	1	1	0	0
tsrna-15527	GCGTTGGTGGTATAGT	1	0	0	0
tsrna-15526	GCGTTCGGCTGTTAACC	1	4	0	0
tsrna-15524	GCGTTCGATTCCCGGTCAGGGAACCA	17	238	5	2
tsrna-15523	GCGTTCAGTTGATGCAGAGTGGG	0	1	0	0
tsrna-15521	GCGTTCAGTTGATGCAG	1	0	0	0
tsrna-15516	GCGTTAGACTGAAGATCT	0	1	0	0
tsrna-15515	GCGTGGGTTCGAATCCCATCCTCGTCGCCA	7	11	4	0
tsrna-15514	GCGTGGGTTCGAATCCCACTTCTGACACC	1	3	1	1
tsrna-15513	GCGTGGGTTCGAATCCCACTTCTGACAC	0	1	1	0
tsrna-15512	GCGTGGCCTAATGGATAAGGCGTCTGACTT	3	0	0	1
tsrna-15511	GCGTGGCCTAATGGATAAGGCGTCTGA	1	0	0	0
tsrna-15510	GCGTGGCCTAATGGATAAGGCGTC	1	0	0	0
tsrna-15499	GCGTGGCCGAGCGGTCTAAGGC	0	1	0	0
tsrna-15494	GCGTGCTGGGCCCATAACCCAGA	0	1	0	1
tsrna-15493	GCGTGCTGGGCCCATAACCCAG	0	1	0	0
tsrna-15492	GCGTGCTGGGCCCATAACCCA	0	1	0	1
tsrna-15491	GCGTGCTGGGCCCATAACCC	2	0	0	0
tsrna-15490	GCGTGCTGGGCCCATAACC	1	0	0	1
tsrna-15485	GCGGTTATCACGTCTGCTTTAC	2	0	1	0
tsrna-15475	GCGGTTAGGATTCCTGGTTTTCACCCAGGC	14	26	6	6
tsrna-15474	GCGGTTAGGATTCCTGGTTTTCACCC	11	13	6	6
tsrna-15473	GCGGTTAGGATTCCTGGTTTTCACC	10	6	5	2
tsrna-15472	GCGGTTAGGATTCCTGGTTTTCAC	6	7	1	6
tsrna-15471	GCGGTTAGGATTCCTGGTTTTCA	6	2	2	2
tsrna-15470	GCGGTTAGGATTCCTGGTTTTC	2	4	2	1
tsrna-15469	GCGGTTAGGATTCCTGGTTTT	5	4	0	1
tsrna-15468	GCGGTTAGGATTCCTGGTTT	2	2	0	1
tsrna-15467	GCGGTTAGGATTCCTGGTT	1	3	1	0
tsrna-15466	GCGGTTAGGATTCCTGGT	2	3	2	0
tsrna-15465	GCGGTTAGGATTCCTGG	1	0	2	0
tsrna-15464	GCGGTTAGGATTCCTG	0	0	0	0
tsrna-15461	GCGGTCTAAGGCGCTGGATT	0	1	0	0
tsrna-15457	GCGGTCTAAGGCGCTGCGTTCA	1	0	0	1
tsrna-15450	GCGGTAGAGCATTTGACTG	1	1	0	0
tsrna-15448	GCGGGTTCGAACCCCGTACGGGCCACC	0	0	0	0
tsrna-15447	GCGGGAGGCCCGGGTTT	5	5	0	0
tsrna-15446	GCGGGAGGCCCGGGTTCGATTCCCGGCCAATGCACC	5	10	2	2
tsrna-15445	GCGGGAGGCCCGGGTTCGATTCCCGGCCAATGC	0	6	1	0
tsrna-15444	GCGGGAGGCCCGGGTTCGATTCCCGGCCAATG	1	0	0	0

tsrna-15443	GCGGGAGGCCCGGGTTCGATTCCCGGCCA	1	1	1	0
tsrna-15442	GCGGGAGGCCCGGGTTCG	1	4	1	0
tsrna-15441	GCGGGAGGCCCGGGTTC	1	7	1	0
tsrna-15440	GCGGGAGGCCCGGGTT	2	3	1	0
tsrna-15439	GCGGGAGACCGGGGTTCGATTCCCCGACGGGGAGCCA	1	12	0	0
tsrna-15438	GCGGGAGACCGGGGTTCGATTCCCCGACGGGGAGCC	0	5	0	0
tsrna-15437	GCGGGAGACCGGGGTTCGATTCCCCGACGGGGAGC	0	1	0	0
tsrna-15436	GCGGGAGACCGGGGTTCGATTCCCCGACGGGGAG	0	0	0	0
tsrna-15435	GCGGGAGACCGGGGTTCGATTCCCCGACGGGGA	1	0	0	0
tsrna-15428	GCGGGAGACCGGGGTTCAATTCCCCGACGGGGAGCCA	0	3	0	0
tsrna-15425	GCGGGAGACCGGGGTTCAATTCCCCGACGG	0	0	1	0
tsrna-15422	GCGGCCCGGGTTCGATTCCCGGTCAGGGAACCA	13	286	0	9
tsrna-15421	GCGGCCCGGGTTCGATTCCCGGTCAGGGAACC	0	9	1	1
tsrna-15420	GCGGCCCGGGTTCGATTCCCGGTCAGGGAA	0	0	1	0
tsrna-15418	GCGGCCCGGGTTCGATTCCCGGTCAGGG	0	2	1	0
tsrna-15415	GCGGCCCGGGTTCGATTCCCGGTCA	0	1	0	0
tsrna-15409	GCGGCCCGGGTTCGATTCC	0	1	0	0
tsrna-15408	GCGGCCCGGGTTCGATTC	1	1	0	0
tsrna-15406	GCGGCCCGGGTTCGACTCCCGGTGTGGGAACCA	70	321	31	31
tsrna-15405	GCGGCCCGGGTTCGACTCCCGGTGTGGGAACC	3	11	3	1
tsrna-15404	GCGGCCCGGGTTCGACTCCCGGTGTGGGAA	0	1	0	0
tsrna-15403	GCGGCCCGGGTTCGACTCCCGGTGTGGGA	2	0	2	0
tsrna-15402	GCGGCCCGGGTTCGACTCCCGGTGTGGG	0	1	0	0
tsrna-15401	GCGGCCCGGGTTCGACTCCCGGTGTGG	1	0	0	0
tsrna-15397	GCGGCCCGGGTTCGACTCCCGGT	0	0	0	0
tsrna-15396	GCGGCCCGGGTTCGACTCCCGG	0	0	1	0
tsrna-15390	GCGGAAGCGTGCTGGGCCCATAACCCAGAG	16	51	8	2
tsrna-15389	GCGGAAGCGTGCTGGGCCCATAACC	9	38	11	10
tsrna-15388	GCGGAAGCGTGCTGGGCCCATA	14	41	8	8
tsrna-15387	GCGGAAGCGTGCTGGGCCCAT	10	39	0	3
tsrna-15386	GCGGAAGCGTGCTGGGCCCA	4	40	7	7
tsrna-15385	GCGGAAGCGTGCTGGGCCC	8	47	2	4
tsrna-15384	GCGGAAGCGTGCTGGGCC	6	21	1	0
tsrna-15383	GCGGAAGCGTGCTGGGC	2	21	1	3
tsrna-15382	GCGGAAGCGTGCTGGG	3	20	2	1
tsrna-15381	GCGCTTTCACCGCCGCGGCCCGGGT	1	7	2	3
tsrna-15380	GCGCTTTCACCGCCGCGGCCCGG	2	3	0	2
tsrna-15379	GCGCTTTCACCGCCGCGGCCCG	1	4	1	0
tsrna-15378	GCGCTTTCACCGCCGCGGCCC	1	3	0	0
tsrna-15377	GCGCTTTCACCGCCGCGGCC	1	2	1	3
tsrna-15376	GCGCTCTCACCGCCGCGGCCCGGGTTCGATTCCCGGTCAGG	45	300	22	11
tsrna-15375	GCGCTCTCACCGCCGCGGCCCGGGTTCGATTCCCGGTCAGG	7	11	6	2
tsrna-15374	GCGCTCTCACCGCCGCGGCCCGGGTTCGATTCCCGGTCAGG	4	4	6	0
tsrna-15373	GCGCTCTCACCGCCGCGGCCCGGGTTCGATTCCCGGTCAGG	1	3	2	0
tsrna-15372	GCGCTCTCACCGCCGCGGCCCGGGTTCGATTCCCGGTCAGG	0	4	1	0
tsrna-15370	GCGCTCTCACCGCCGCGGCCCGGGTTCG	1	3	0	0
tsrna-15369	GCGCTCTCACCGCCGCGGCCCGGGTTC	0	1	0	0
tsrna-15368	GCGCTCTCACCGCCGCGGCCCGGGTT	0	2	0	0
tsrna-15365	GCGCTCTCACCGCCGCGGCCCGG	0	0	0	0
tsrna-15363	GCGCTCTCACCGCCGCGGCCC	0	1	0	0
tsrna-15362	GCGCTCTCACCGCCGCGGCC	0	1	0	0
tsrna-15357	GCGCGTTTGGCTGTTAACT	1	0	0	0
tsrna-15356	GCGCGTTCGGCTGTTAACCGAAAGGTTGGT	6	29	9	5
tsrna-15355	GCGCGTTCGGCTGTTAACCGAAAGGT	7	16	8	1
tsrna-15354	GCGCGTTCGGCTGTTAACCGAAAGG	7	17	5	1
tsrna-15353	GCGCGTTCGGCTGTTAACCGAAAG	4	18	3	1
tsrna-15352	GCGCGTTCGGCTGTTAACCGAAA	3	20	7	1
tsrna-15351	GCGCGTTCGGCTGTTAACC	2	1	0	0
tsrna-15350	GCGCGTTCGGCTGTTAAC	0	1	0	0
tsrna-15346	GCGCCGTGGCTTAGTTGGTTAAAGC	0	0	0	1
tsrna-15344	GCGCCGCTGGTGTAGTGGTATCATGCAAGATTCCCATTCTTG	172	107	228	9
tsrna-15343	GCGCCGCTGGTGTAGTGGTATCATGCAAGATTCCCA	124	79	90	7
tsrna-15342	GCGCCGCTGGTGTAGTGGTATCATGCAAGATTCCC	109	81	103	9
tsrna-15341	GCGCCGCTGGTGTAGTGGTATCATGCAAGATTCC	89	75	78	6

tsrna-15340	GCGCCGCTGGTGTAGTGGTATCATGCAAGATTC	71	70	55	12
tsrna-15339	GCGCCGCTGGTGTAGTGGTATCATGCAAGATT	48	59	55	8
tsrna-15338	GCGCCGCTGGTGTAGTGGTATCATGCAAGAT	24	46	24	6
tsrna-15337	GCGCCGCTGGTGTAGTGGTATCATGCAAGA	18	46	20	7
tsrna-15336	GCGCCGCTGGTGTAGTGGTATCATGCAAG	12	25	20	3
tsrna-15335	GCGCCGCTGGTGTAGTGGTATCATGCAA	11	12	10	0
tsrna-15334	GCGCCGCTGGTGTAGTGGTATCATGCA	9	15	10	0
tsrna-15333	GCGCCGCTGGTGTAGTGGTATCATGC	4	5	1	0
tsrna-15332	GCGCCGCTGGTGTAGTGGTATCATG	1	1	3	0
tsrna-15331	GCGCCGCTGGTGTAGTGGTATCAT	1	5	2	1
tsrna-15330	GCGCCGCTGGTGTAGTGGTATCA	0	8	1	0
tsrna-15329	GCGCCGCTGGTGTAGTGGTATC	0	5	1	0
tsrna-15328	GCGCCGCTGGTGTAGTGGTAT	0	3	0	0
tsrna-15327	GCGCCGCTGGTGTAGTGGTA	1	6	2	1
tsrna-15326	GCGCCGCTGGTGTAGTGGT	0	3	0	0
tsrna-15325	GCGCCGCTGGTGTAGTGG	1	3	0	0
tsrna-15323	GCGCCGCTGGTGTAGT	0	0	1	0
tsrna-15320	GCGCAGCGGAAGCGTGCTGGGCCCA	15	51	8	3
tsrna-15319	GCGCAGCGGAAGCGTGCTGGGCCC	10	56	5	7
tsrna-15318	GCGCAGCGGAAGCGTGCTGGGCC	1	32	1	1
tsrna-15317	GCGCAGCGGAAGCGTGCTGGGC	2	31	1	1
tsrna-15316	GCGCAGCGGAAGCGTGCTGGG	3	29	0	2
tsrna-15315	GCGCAGCGGAAGCGTGCTGG	0	5	0	0
tsrna-15314	GCGCAGCGGAAGCGTGCTG	0	2	0	0
tsrna-15313	GCGCAGCGGAAGCGTGCT	0	1	0	0
tsrna-15311	GCGCAATGGATAGCGCATTGGACT	9	15	2	4
tsrna-15310	GCGCAATGGATAGCGCATTGG	7	5	3	0
tsrna-15309	GCGCAATGGATAGCGCATTG	1	0	0	0
tsrna-15308	GCGCAATGGATAGCGCATT	0	0	0	1
tsrna-15307	GCGCAATGGATAACGCGTCTGACTACGG	2	4	0	3
tsrna-15306	GCGCAATGGATAACGCGTCTGACT	1	1	0	0
tsrna-15305	GCGCAATGGATAACGCGTCTGAC	2	1	0	0
tsrna-15300	GCGATCCGAGTTCAAATCTCGGTGGAACCTC	0	3	0	0
tsrna-15299	GCGATCCGAGTTCAAATCTCGGTGGAACCT	0	1	0	0
tsrna-15296	GCGAGTTCAATTCTCGCTGGGGCCTC	1	0	0	0
tsrna-15294	GCGAGTTCAAATCTCGCTGGGGCCTCC	1	8	0	3
tsrna-15293	GCGAGTTCAAATCTCGCTGGGGCCTC	1	1	1	0
tsrna-15290	GCGAGAGGTCCCGGGTTCAAATCCCGGACGAGCCCC	5	7	3	0
tsrna-15289	GCGAGAGGTCCCGGGTTCAAATCCCGGACG	0	2	0	0
tsrna-15288	GCGAGAGGTCCCGGGTTCAAA	0	2	0	0
tsrna-15287	GCGAGAGGTCCCGGGTTCA	0	3	0	0
tsrna-15286	GCGAGAGGTCCCGGGTTC	0	1	0	2
tsrna-15285	GCGAGAGGTCCCGGGTT	0	1	0	0
tsrna-15283	GCGAGAGGTAGCGGGATCG	7	13	9	0
tsrna-15282	GCGAGAGGTAGCGGGATC	11	10	3	0
tsrna-15281	GCGAGAGGTAGCGGGAT	1	3	0	1
tsrna-15280	GCGAGAGGTAGCGGGA	1	11	0	0
tsrna-15279	GCGACCCGGGTTCGATTCCCGGGCGGCGC	0	2	0	0
tsrna-15273	GCGACCCGGGTTCGATTCCCGG	0	1	0	0
tsrna-15271	GCGAAAGGTCCCTGGA	0	3	0	0
tsrna-15270	GCGAAAGGTCCCCGGTTT	0	0	0	0
tsrna-15269	GCGAAAGGTCCCCGGTTCGATCCCGGGCGGAAACACC	2	7	4	1
tsrna-15268	GCGAAAGGTCCCCGGTTCGATCCCGGGCGG	0	2	0	0
tsrna-15267	GCGAAAGGTCCCCGGTTCGAAACCGGGCGGAAACACCA	285	707	129	41
tsrna-15266	GCGAAAGGTCCCCGGTTCGAAACCGGGCGGAAACACC	78	191	25	6
tsrna-15265	GCGAAAGGTCCCCGGTTCGAAACCGGGCGGAAACA	0	5	0	0
tsrna-15263	GCGAAAGGTCCCCGGTTCGAAACCGGGCG	0	3	1	0
tsrna-15262	GCGAAAGGTCCCCGGTTCGAAACCGGGCAG	0	3	1	0
tsrna-15261	GCGAAAGGTCCCCGGTTCGAAACCGGGC	1	0	0	0
tsrna-15260	GCGAAAGGTCCCCGGTTCGAAACCGG	0	2	0	0
tsrna-15259	GCGAAAGGTCCCCGGTTCGAAACC	0	2	0	0
tsrna-15258	GCGAAAGGTCCCCGGTTCGAAAC	1	2	0	0
tsrna-15257	GCGAAAGGTCCCCGGTTCGAAA	1	3	0	0
tsrna-15256	GCGAAAGGTCCCCGGTTCG	2	1	0	0

tsrna-15255	GCGAAAGGTCCCCGGTTC	1	0	1	0
tsrna-15254	GCGAAAGGTCCCCGGTT	0	1	0	0
tsrna-15251	GCCTTGGTGGTGCAGTGGTAGAATTCTCGC	3	1	0	0
tsrna-15250	GCCTTGGTGGTGCAGTGGTAGAATTCTCG	3	1	0	0
tsrna-15249	GCCTTCGATAGCTCAGTTGGTAGAGCGGAG	7	21	7	4
tsrna-15248	GCCTTCCAAGCAGTTGACCCGGGT	30	36	14	17
tsrna-15247	GCCTTCCAAGCAGTTGACCCGGG	0	0	3	1
tsrna-15246	GCCTTCCAAGCAGTTGACCCGG	0	0	3	1
tsrna-15245	GCCTTCCAAGCAGTTGACCCG	2	2	0	1
tsrna-15244	GCCTTCAAAGCCCTCAGTAAGTTGCAATACTTAATTTCTGCCA	9	3	6	3
tsrna-15242	GCCTGTCACGCGGGAGACCGGGGTTCGATTCCCCGACGGG	21	67	11	2
tsrna-15241	GCCTGTCACGCGGGAGACCGGGGTTCGATTCCCCGACGGG	16	43	6	3
tsrna-15240	GCCTGTCACGCGGGAGACCGGGGTTCGATTCCCCGACGGG	15	63	5	5
tsrna-15239	GCCTGTCACGCGGGAGACCGGGGTTCGATTCCCCGACGGG	17	61	9	2
tsrna-15238	GCCTGTCACGCGGGAGACCGGGGTTCGATTCCCCGACGGG	10	44	7	6
tsrna-15237	GCCTGTCACGCGGGAGACCGGGGTTCGATT	16	53	8	4
tsrna-15236	GCCTGTCACGCGGGAGACCGGGGTTCG	13	46	8	1
tsrna-15235	GCCTGTCACGCGGGAGACCGGGGTTCAATT	8	59	7	3
tsrna-15234	GCCTGTCACGCGGGAGACCGGGGTTC	12	33	9	3
tsrna-15233	GCCTGTCACGCGGGAGACCGGGGTT	15	56	10	2
tsrna-15232	GCCTGTCACGCGGGAGACCGGGGT	16	50	5	2
tsrna-15231	GCCTGTCACGCGGGAGACCGGGG	11	51	6	1
tsrna-15230	GCCTGTCACGCGGGAGACCGGG	9	41	12	4
tsrna-15229	GCCTGTCACGCGGGAGACCGG	12	44	4	3
tsrna-15228	GCCTGTCACGCGGGAGACCG	12	47	9	2
tsrna-15227	GCCTGTCACGCGGGAGACC	14	43	4	0
tsrna-15226	GCCTGTCACGCGGGAGAC	17	51	8	5
tsrna-15225	GCCTGTCACGCGGGAGA	17	53	11	4
tsrna-15224	GCCTGTCACGCGGGAG	8	53	13	5
tsrna-15223	GCCTGGGTAGCTCAGTCGGTAGAGCATCAGAC	15	13	7	1
tsrna-15222	GCCTGGGTAGCTCAGTCGGTAGAGCATCAG	4	5	5	0
tsrna-15221	GCCTGGGTAGCTCAGTCGGTAGAGCATCA	5	2	1	0
tsrna-15220	GCCTGGGTAGCTCAGTCGGTAGAGCATC	2	2	1	0
tsrna-15219	GCCTGGGTAGCTCAGTCGGTAGAGCAT	0	1	0	0
tsrna-15218	GCCTGGGTAGCTCAGTCGGTAGAGCA	0	0	1	0
tsrna-15216	GCCTGGGTAGCTCAGTCGGTAGAG	0	0	0	0
tsrna-15211	GCCTGGGTAGCTCAGTCG	1	0	0	0
tsrna-15209	GCCTGGATAGCTCAGTTGGTAGAGCATCAGAC	3	0	3	0
tsrna-15208	GCCTGGATAGCTCAGTTGGTAGAGCATCAG	1	0	0	0
tsrna-15207	GCCTGGATAGCTCAGTTGGTAGAGCATC	0	0	0	0
tsrna-15206	GCCTGGATAGCTCAGTTGGTAGAGCAT	0	0	1	1
tsrna-15205	GCCTGGATAGCTCAGTTGGTAGAGCA	1	1	0	0
tsrna-15204	GCCTGGATAGCTCAGTTGGTAGAGC	0	0	0	0
tsrna-15203	GCCTGGATAGCTCAGTTGGTAGAG	0	3	2	0
tsrna-15202	GCCTGGATAGCTCAGTTGGTAG	1	1	1	1
tsrna-15201	GCCTGGATAGCTCAGTTGGT	0	0	0	0
tsrna-15199	GCCTGGATAGCTCAGTCGGTAGAGCATCAGACT	17	21	23	4
tsrna-15198	GCCTGGATAGCTCAGTCGGTAGAGCATCAGAC	11	8	7	3
tsrna-15197	GCCTGGATAGCTCAGTCGGTAGAGCATCAG	3	8	6	3
tsrna-15196	GCCTGGATAGCTCAGTCGGTAGAGCATCA	5	4	5	4
tsrna-15195	GCCTGGATAGCTCAGTCGGTAGAGCATC	5	4	3	0
tsrna-15194	GCCTGGATAGCTCAGTCGGTAGAGCAT	1	1	2	3
tsrna-15193	GCCTGGATAGCTCAGTCGGTAGAGCA	2	1	1	1
tsrna-15192	GCCTGGATAGCTCAGTCGGTAGAGC	0	0	1	0
tsrna-15191	GCCTGGATAGCTCAGTCGGTAGAG	0	1	1	0
tsrna-15190	GCCTGGATAGCTCAGTCGGTAGA	0	0	0	1
tsrna-15189	GCCTGGATAGCTCAGTCGGTAG	0	0	1	0
tsrna-15188	GCCTGGATAGCTCAGTCGGTA	0	1	0	0
tsrna-15185	GCCTGGATAGCTCAGTCG	0	0	0	0
tsrna-15183	GCCTGCCACGCGGGAGGCCCGGGTTCG	5	19	3	1
tsrna-15182	GCCTGCCACGCGGGAGGCCCGGGTT	3	15	1	1
tsrna-15181	GCCTGCCACGCGGGAGGCCCGGGT	4	14	0	2
tsrna-15179	GCCTGCCACGCGGGAGGCCCGG	0	1	0	0
tsrna-15178	GCCTGCCACGCGGGAGGCCCG	0	1	0	0

tsrna-15177	GCCTGCCACGCGGGAGGCCC	0	1	0	0
tsrna-15170	GCCTCGTTAGCGCAGTAGGTAGCGCGTCAGTC	1	1	0	0
tsrna-15169	GCCTCGTTAGCGCAGTAGGTAGCGCGTCAG	0	1	0	0
tsrna-15163	GCCTCGTTAGCGCAGTAGGTA	0	1	0	0
tsrna-15156	GCCTCGTGGCGCAACGGTAGCGCGTCTGAC	1	1	0	0
tsrna-15154	GCCTCCTTAGCGCAGTAGGCAGCG	1	0	0	0
tsrna-15151	GCCTCCTTAGCGCAGTAGGCA	0	0	0	0
tsrna-15150	GCCTCCTTAGCGCAGTAGG	0	2	0	0
tsrna-15148	GCCTCCTTAGCGCAGTA	0	0	0	0
tsrna-15147	GCCTCCTTAGCGCAGT	0	0	0	0
tsrna-15146	GCCTCCTAAGCCAGGGATTGTGGGT	137	367	89	44
tsrna-15145	GCCTCCTAAGCCAGGGATTGTGGG	1	4	2	0
tsrna-15144	GCCTCCTAAGCCAGGGATTGTGG	0	0	0	0
tsrna-15143	GCCTCCTAAGCCAGGGATTGTG	0	1	0	1
tsrna-15142	GCCTCCGGAGCTGGGGATTGTGGGT	2	1	0	1
tsrna-15141	GCCTCCGGAGCTGGGGATTGTGGG	0	1	0	0
tsrna-15132	GCCTCACACGCGAAAGGTCCCCG	0	0	0	0
tsrna-15130	GCCTAATGGATAAGGCGTCTGACT	0	0	1	0
tsrna-15129	GCCTAATGGATAAGGCATTGGCCTCCTAAG	6	7	6	1
tsrna-15128	GCCTAATGGATAAGGCATTGGCCTCC	3	2	4	1
tsrna-15127	GCCTAATGGATAAGGCATTGGCCTC	5	1	2	3
tsrna-15126	GCCTAATGGATAAGGCATTGGCCT	6	3	2	0
tsrna-15125	GCCTAATGGATAAGGCATTGGCC	3	1	3	0
tsrna-15124	GCCTAATGGATAAGGCATTGGC	4	4	3	2
tsrna-15123	GCCTAATGGATAAGGCATTGG	6	3	4	1
tsrna-15122	GCCTAATGGATAAGGCATTG	4	2	2	0
tsrna-15121	GCCTAATGGATAAGGCATCAGCCTCCGGAG	4	1	3	0
tsrna-15120	GCCTAATGGATAAGGCATCAGCCTCC	4	3	6	0
tsrna-15119	GCCTAATGGATAAGGCATCAGCCTC	3	1	2	1
tsrna-15118	GCCTAATGGATAAGGCATCAGCCT	4	0	1	2
tsrna-15117	GCCTAATGGATAAGGCATCAGCC	5	3	1	0
tsrna-15116	GCCTAATGGATAAGGCATCAGC	2	2	4	0
tsrna-15115	GCCTAATGGATAAGGCATCAG	1	6	3	0
tsrna-15114	GCCTAATGGATAAGGCATC	2	1	1	0
tsrna-15111	GCCGTGATCGTATAGTGGTTAGTACTCTGCGTTGTGGCCGCA	25	19	13	2
tsrna-15110	GCCGTGATCGTATAGTGGTTAGTACTCTGCGTTGTGGCCG	23	19	20	8
tsrna-15109	GCCGTGATCGTATAGTGGTTAGTACTCTGCGTTGTGGCC	31	12	12	7
tsrna-15108	GCCGTGATCGTATAGTGGTTAGTACTCTGCGTTGTG	33	9	20	8
tsrna-15107	GCCGTGATCGTATAGTGGTTAGTACTCTGCGTTGT	29	14	14	9
tsrna-15106	GCCGTGATCGTATAGTGGTTAGTACTCTGCGTTG	23	15	16	6
tsrna-15105	GCCGTGATCGTATAGTGGTTAGTACTCTGCGTT	21	10	9	9
tsrna-15104	GCCGTGATCGTATAGTGGTTAGTACTCTGCGT	8	3	3	2
tsrna-15103	GCCGTGATCGTATAGTGGTTAGTACTCTGCG	8	2	2	3
tsrna-15102	GCCGTGATCGTATAGTGGTTAGTACTCTGC	2	2	1	2
tsrna-15101	GCCGTGATCGTATAGTGGTTAGTACTCTG	2	1	1	2
tsrna-15100	GCCGTGATCGTATAGTGGTTAGTACTCT	0	0	1	1
tsrna-15099	GCCGTGATCGTATAGTGGTTAGTACTC	0	0	0	1
tsrna-15098	GCCGTGATCGTATAGTGGTTAGTACT	1	1	1	0
tsrna-15094	GCCGTGATCGTATAGTGGTTAG	0	0	0	0
tsrna-15087	GCCGGTTAGCTCAGTTGGTTAGA	0	0	0	0
tsrna-15086	GCCGGTTAGCTCAGTTGGTTAG	1	0	0	0
tsrna-15085	GCCGGTTAGCTCAGTTGGTT	0	1	0	0
tsrna-15084	GCCGGTTAGCTCAGTTGGTCAGAGC	0	0	0	1
tsrna-15083	GCCGGTTAGCTCAGTTGGTCAGA	2	1	0	0
tsrna-15082	GCCGGTTAGCTCAGTTGGT	0	0	1	0
tsrna-15081	GCCGGTTAGCTCAGTTGG	1	0	0	0
tsrna-15080	GCCGGTTAGCTCAGTTG	0	0	1	0
tsrna-15079	GCCGGTTAGCTCAGTT	0	0	1	0
tsrna-15078	GCCGGTTAGCTCAGTCGGC	0	0	1	1
tsrna-15077	GCCGGTTAGCTCAGTCGG	0	0	0	1
tsrna-15076	GCCGCTGGTGTAGTGGTATCATGCAAGATTCCC	10	6	13	1
tsrna-15075	GCCGCTGGTGTAGTGGTATCATGCAAGATT	5	3	5	0
tsrna-15074	GCCGCTGGTGTAGTGGTATCATGCAAGAT	2	1	2	0
tsrna-15073	GCCGCTGGTGTAGTGGTATCATGCAAGA	4	0	1	0

tsrna-15072	GCCGCTGGTGTAGTGGTATCATGCAAG	0	1	2	1
tsrna-15071	GCCGCTGGTGTAGTGGTATCATGCAA	1	1	1	0
tsrna-15070	GCCGCTGGTGTAGTGGTATCATGCA	2	0	0	0
tsrna-15068	GCCGCTGGTGTAGTGGTATCATG	0	0	0	0
tsrna-15066	GCCGCGTGGCCTAATGGATAAGGCGTCTG	0	2	0	1
tsrna-15058	GCCGCGGCCCGGGTTCGATTCCCGGT	0	0	0	0
tsrna-15057	GCCGCGGCCCGGGTTCGATTCCC	0	1	0	0
tsrna-15055	GCCGCGGCCCGGGTTCGATTC	0	0	0	0
tsrna-15051	GCCGCGGCCCGGGTTCG	0	1	0	0
tsrna-15049	GCCGCAGCAACCTCGGTTCGAATCCGAGTCACGGCACCA	8	34	2	0
tsrna-15048	GCCGCAGCAACCTCGG	0	0	1	0
tsrna-15040	GCCGAGGTTGTGAGTTCG	0	1	0	0
tsrna-15037	GCCGAGCGGTCTAAGGCGCTGCGTTC	0	1	0	0
tsrna-15032	GCCGAGATAGCTCAGTTGGGA	0	0	2	0
tsrna-15031	GCCGAGATAGCTCAGTTGGG	0	1	0	0
tsrna-15030	GCCGAGATAGCTCAGTTGG	0	1	0	1
tsrna-15029	GCCGAAATAGCTCAGTTGGGAGAGCGTTAGACT	7	8	6	3
tsrna-15028	GCCGAAATAGCTCAGTTGGGAGAGCGTTAG	4	8	6	4
tsrna-15027	GCCGAAATAGCTCAGTTGGGAGAGC	10	4	1	4
tsrna-15026	GCCGAAATAGCTCAGTTGGGAGAG	2	3	5	5
tsrna-15025	GCCGAAATAGCTCAGTTGGGAGA	6	6	9	5
tsrna-15024	GCCGAAATAGCTCAGTTGGGAG	10	4	6	3
tsrna-15023	GCCGAAATAGCTCAGTTGGGA	11	4	3	6
tsrna-15022	GCCGAAATAGCTCAGTTGGG	5	17	5	7
tsrna-15021	GCCGAAATAGCTCAGTTGG	8	9	5	7
tsrna-15020	GCCGAAATAGCTCAGTTG	7	6	4	4
tsrna-15019	GCCGAAATAGCTCAGTT	2	4	4	4
tsrna-15018	GCCGAAATAGCTCAGT	8	3	6	4
tsrna-15017	GCCCTGTGGTCTAGTGGTTAGG	4	5	7	3
tsrna-15014	GCCCTCTTAGCGCAGTGGG	0	0	1	0
tsrna-15013	GCCCTCTTAGCGCAGCGGGCAGC	1	2	0	0
tsrna-15012	GCCCTCTTAGCGCAGCGGG	1	0	0	0
tsrna-15011	GCCCTCTTAGCGCAGCGG	0	0	0	0
tsrna-15000	GCCCGGTAATCGCATAAAACTTAAAACTTT	3	8	19	1
tsrna-14993	GCCCGGGTTCGATTCCCGGTCAGGGAACC	0	7	0	0
tsrna-14992	GCCCGGGTTCGATTCCCGGTCAGGGAAC	0	3	0	0
tsrna-14991	GCCCGGGTTCGATTCCCGGTCAGGGAA	1	2	0	0
tsrna-14990	GCCCGGGTTCGATTCCCGGTCAGG	0	1	0	0
tsrna-14989	GCCCGGGTTCGACTCCCGGTGTGGGAACC	2	8	0	1
tsrna-14988	GCCCGGGTTCGACTCCCGGTGTGGGAAC	0	1	0	0
tsrna-14987	GCCCGGGTTCGACTCCCGGTGTGGGAA	0	2	0	0
tsrna-14985	GCCCGGGTTCGACTCCCGGTGTGGG	2	0	0	0
tsrna-14984	GCCCGGGTTCGACTCCCGGTGTGG	0	2	0	0
tsrna-14983	GCCCGGCTAGCTCAGTCGGTAGAGCATGGGACTCTTAATCTC	361	137	458	145
tsrna-14982	GCCCGGCTAGCTCAGTCGGTAGAGCATGGGACTCTTAATCCC	435	132	464	139
tsrna-14981	GCCCGGCTAGCTCAGTCGGTAGAGCATGGGACTCTTAATCC	329	146	388	139
tsrna-14980	GCCCGGCTAGCTCAGTCGGTAGAGCATGGGACTCTTAATC	363	118	401	127
tsrna-14979	GCCCGGCTAGCTCAGTCGGTAGAGCATGGGACTCTTAAT	299	131	343	137
tsrna-14978	GCCCGGCTAGCTCAGTCGGTAGAGCATGGGACTCTTAA	326	116	358	131
tsrna-14977	GCCCGGCTAGCTCAGTCGGTAGAGCATGGGACTCTT	327	123	326	142
tsrna-14976	GCCCGGCTAGCTCAGTCGGTAGAGCATGGGACTCT	195	109	205	89
tsrna-14975	GCCCGGCTAGCTCAGTCGGTAGAGCATGGGACTC	59	53	55	33
tsrna-14974	GCCCGGCTAGCTCAGTCGGTAGAGCATGGGACT	23	27	33	13
tsrna-14973	GCCCGGCTAGCTCAGTCGGTAGAGCATGGGAC	23	25	15	7
tsrna-14972	GCCCGGCTAGCTCAGTCGGTAGAGCATGGGA	10	20	9	6
tsrna-14971	GCCCGGCTAGCTCAGTCGGTAGAGCATGGG	6	3	2	4
tsrna-14970	GCCCGGCTAGCTCAGTCGGTAGAGCATGG	2	9	6	2
tsrna-14969	GCCCGGCTAGCTCAGTCGGTAGAGCATGAGACTCTTAATCT	407	302	412	135
tsrna-14968	GCCCGGCTAGCTCAGTCGGTAGAGCATGAGACTCTTAATC	363	339	324	136
tsrna-14967	GCCCGGCTAGCTCAGTCGGTAGAGCATGAGACTCTTAAT	381	284	304	117
tsrna-14966	GCCCGGCTAGCTCAGTCGGTAGAGCATGAGACTCTTAA	347	269	249	119
tsrna-14965	GCCCGGCTAGCTCAGTCGGTAGAGCATGAGACTCTT	352	307	306	120
tsrna-14964	GCCCGGCTAGCTCAGTCGGTAGAGCATGAGACTCT	222	217	175	87
tsrna-14963	GCCCGGCTAGCTCAGTCGGTAGAGCATGAGACTC	104	111	75	58

tsrna-14962	GCCCGGCTAGCTCAGTCGGTAGAGCATGAGACT	16	27	11	4
tsrna-14961	GCCCGGCTAGCTCAGTCGGTAGAGCATGAGACCCTT	17	28	9	6
tsrna-14960	GCCCGGCTAGCTCAGTCGGTAGAGCATGAGACCC	15	24	8	5
tsrna-14959	GCCCGGCTAGCTCAGTCGGTAGAGCATGAGACC	20	26	9	11
tsrna-14958	GCCCGGCTAGCTCAGTCGGTAGAGCATGAGAC	12	25	2	9
tsrna-14957	GCCCGGCTAGCTCAGTCGGTAGAGCATGAGA	9	15	10	2
tsrna-14956	GCCCGGCTAGCTCAGTCGGTAGAGCATGAG	5	4	6	2
tsrna-14955	GCCCGGCTAGCTCAGTCGGTAGAGCATGA	7	4	2	1
tsrna-14954	GCCCGGCTAGCTCAGTCGGTAGAGCATG	6	10	4	1
tsrna-14953	GCCCGGCTAGCTCAGTCGGTAGAGCAT	4	3	2	2
tsrna-14952	GCCCGGCTAGCTCAGTCGGTAGAGCA	1	7	4	4
tsrna-14951	GCCCGGCTAGCTCAGTCGGTAGAGC	1	6	0	4
tsrna-14950	GCCCGGCTAGCTCAGTCGGTAGAG	4	7	1	3
tsrna-14949	GCCCGGCTAGCTCAGTCGGTAGA	3	5	4	4
tsrna-14948	GCCCGGCTAGCTCAGTCGGTAG	2	4	3	2
tsrna-14947	GCCCGGCTAGCTCAGTCGGTA	2	4	1	3
tsrna-14946	GCCCGGCTAGCTCAGTCGGT	2	9	2	3
tsrna-14945	GCCCGGCTAGCTCAGTCGG	1	2	2	1
tsrna-14944	GCCCGGCTAGCTCAGTCGATAGAGCATGAGACTC	5	6	2	4
tsrna-14943	GCCCGGCTAGCTCAGTCGATAGAGCATGAGACT	5	6	4	1
tsrna-14942	GCCCGGCTAGCTCAGTCGA	4	5	0	0
tsrna-14941	GCCCGGCTAGCTCAGTCG	0	3	0	0
tsrna-14940	GCCCGGCTAGCTCAGTC	4	1	3	0
tsrna-14939	GCCCGGCTAGCTCAGT	0	0	0	2
tsrna-14938	GCCCGGATAGCTCAGTCGGTAGAGCATCAGACTTTT	1308	762	1479	217
tsrna-14937	GCCCGGATAGCTCAGTCGGTAGAGCATCAGACTTT	1218	715	1367	207
tsrna-14936	GCCCGGATAGCTCAGTCGGTAGAGCATCAGACTT	1176	724	1409	179
tsrna-14935	GCCCGGATAGCTCAGTCGGTAGAGCATCAGACT	1133	702	1289	207
tsrna-14934	GCCCGGATAGCTCAGTCGGTAGAGCATCAGAC	35	35	28	8
tsrna-14933	GCCCGGATAGCTCAGTCGGTAGAGCATCAGA	21	21	20	4
tsrna-14932	GCCCGGATAGCTCAGTCGGTAGAGCATCAG	16	4	8	8
tsrna-14931	GCCCGGATAGCTCAGTCGGTAGAGCATCA	17	10	17	4
tsrna-14930	GCCCGGATAGCTCAGTCGGTAGAGCATC	11	15	9	5
tsrna-14929	GCCCGGATAGCTCAGTCGGTAGAGCAT	7	1	4	9
tsrna-14928	GCCCGGATAGCTCAGTCGGTAGAGCA	4	0	3	4
tsrna-14927	GCCCGGATAGCTCAGTCGGTAGAGC	3	4	4	4
tsrna-14926	GCCCGGATAGCTCAGTCGGTAGAG	4	2	3	2
tsrna-14925	GCCCGGATAGCTCAGTCGGTAGA	2	4	1	3
tsrna-14924	GCCCGGATAGCTCAGTCGGTAG	1	0	3	4
tsrna-14923	GCCCGGATAGCTCAGTCGGTA	1	1	0	0
tsrna-14922	GCCCGGATAGCTCAGTCGGT	1	4	2	1
tsrna-14921	GCCCGGATAGCTCAGTCGG	0	3	1	0
tsrna-14920	GCCCGGATAGCTCAGTCG	0	0	0	0
tsrna-14919	GCCCGGATAGCTCAGTC	0	0	0	1
tsrna-14918	GCCCGGATAGCTCAGT	1	1	0	0
tsrna-14917	GCCCGCATTCTCCACCA	0	0	1	1
tsrna-14915	GCCCGCATCCTCCACCA	0	1	1	0
tsrna-14913	GCCCCGGTGGCCTAATGGATAAGGCATTGG	5	1	4	1
tsrna-14912	GCCCCGGTGGCCTAATGGATAAGGCATTG	2	6	5	2
tsrna-14901	GCCCCGGGTTCGATCCCCGGCATCTCCAC	0	0	1	0
tsrna-14893	GCCCCCATGTCTAACAACATGGCTTTCTCACCA	0	2	6	0
tsrna-14892	GCCCCCATGTCTAACAACATGGCTTTCTCACC	4	0	2	0
tsrna-14884	GCCCCCATGTCTAACAACATGGC	0	1	0	0
tsrna-14879	GCCCCATGGTGTAATGGTTAGCACTCTGGA	0	2	0	0
tsrna-14878	GCCCCATGGTGTAATGGTTAGCACTCTGG	1	1	0	0
tsrna-14872	GCCCCAGTGGCCTAATGGATAAGGCATTGG	2	2	6	0
tsrna-14871	GCCCCAGTGGCCTAATGGATAAGGCATTG	1	1	2	0
tsrna-14868	GCCCCAGTGGCCTAATGGATAAGGCACTGG	68	37	54	27
tsrna-14867	GCCCCAGTGGCCTAATGGATAAGGCACTG	75	32	51	15
tsrna-14866	GCCCCAGTGGCCTAATGGATAAGGCACT	4	3	2	2
tsrna-14865	GCCCCAGTGGCCTAATGGATAAGGCAC	0	0	0	0
tsrna-14853	GCCCCAGTGGAACCACCA	2	1	0	0
tsrna-14852	GCCCCAGTGGAACCACC	0	0	0	0
tsrna-14850	GCCCCACGTTGGGCGCCA	5	24	4	4

tsrna-14849	GCCCCACGTTGGGCGCC	0	2	0	0
tsrna-14848	GCCCATACCCCGAAAATGTTGGTT	1	0	0	0
tsrna-14843	GCCCATACCCCGAAAA	1	0	0	0
tsrna-14842	GCCCATAACCCAGAGGTCGATGGATC	3	8	2	1
tsrna-14841	GCCCATAACCCAGAGGTCGATGGA	1	7	0	1
tsrna-14840	GCCCATAACCCAGAGGTCGATGG	2	1	0	1
tsrna-14839	GCCCATAACCCAGAGGTCGATG	0	1	1	0
tsrna-14838	GCCCATAACCCAGAGGTCGAT	1	1	0	0
tsrna-14837	GCCCATAACCCAGAGGTCGA	0	0	0	0
tsrna-14836	GCCCATAACCCAGAGGTCG	0	1	0	0
tsrna-14834	GCCCAGCTAGCTCAGTCGGTAGAGCATGAG	2	6	4	2
tsrna-14833	GCCCAGCTAGCTCAGTCGGTAGAGC	0	0	0	0
tsrna-14832	GCCCAGCTAGCTCAGTCGGTAGAG	1	0	0	0
tsrna-14829	GCCCACCCAGGGACGCCA	2	63	1	0
tsrna-14828	GCCCACCCAGGGACGCC	0	1	0	0
tsrna-14826	GCCCACATTCTCCACCA	1	0	0	0
tsrna-14825	GCCATGGGGTTGGCTTGAAACC	0	2	0	0
tsrna-14823	GCCATGATCGTATAGTGGTTAGTACTCTGC	6	2	3	1
tsrna-14822	GCCATGATCGTATAGTGGTTAGTACTCTG	2	1	0	0
tsrna-14821	GCCATGATCGTATAGTGGTTAGTACTC	0	0	0	1
tsrna-14820	GCCAGTTGATTAGGGTGCTTAGCTGTTAAC	0	3	1	0
tsrna-14819	GCCAGTTGATTAGGGTGCTTAGCTG	0	0	0	0
tsrna-14818	GCCAGTTGATTAGGGTGCTTAGCT	0	1	0	0
tsrna-14815	GCCAGTTGATTAGGGTGCTTA	1	0	0	0
tsrna-14812	GCCAGGGATTGTGGGTTCGAGTCCCATCTGGGGTGCCA	1	17	1	0
tsrna-14811	GCCAGGGATTGTGGGTTCGAGTCCCATCTGGGGTGCC	3	6	3	1
tsrna-14810	GCCAGGGATTGTGGGTTCGAGTCCC	2	7	1	1
tsrna-14809	GCCAGGGATTGTGGGTTCGAGTCC	1	4	2	0
tsrna-14808	GCCAGGGATTGTGGGTTCG	1	4	3	2
tsrna-14807	GCCAGGGATTGTGGGTTC	0	10	3	1
tsrna-14806	GCCAGGGATTGTGGGT	3	10	4	1
tsrna-14805	GCCACGCGGGAGGCCCGGGTTT	2	3	0	0
tsrna-14804	GCCACGCGGGAGGCCCGGGTTCGATTCCCGGCCAATGCACC	33	162	11	11
tsrna-14803	GCCACGCGGGAGGCCCGGGTTCG	3	3	0	1
tsrna-14802	GCCACGCGGGAGGCCCGGGTTC	0	4	1	0
tsrna-14801	GCCACGCGGGAGGCCCGGGTT	2	8	0	0
tsrna-14800	GCCACGCGGGAGGCCCGGGT	3	9	0	0
tsrna-14797	GCCACGCGGGAGGCCCG	0	0	1	0
tsrna-14796	GCCACGCGGGAGGCCC	0	1	0	0
tsrna-14795	GCCAAGGTCGCGGGTTCGATCCCCGTA	0	3	0	0
tsrna-14794	GCCAAGGTCGCGGGTTCGAT	0	0	1	0
tsrna-14793	GCCAAGGTCGCGGGTTCG	1	1	0	0
tsrna-14789	GCATTGGTGGTTCAGTGGTAGAATTCTCGCCTGCCACGCGGG	5230	908	5971	376
tsrna-14788	GCATTGGTGGTTCAGTGGTAGAATTCTCGCCTGCCACGCGGG	5266	884	6047	367
tsrna-14787	GCATTGGTGGTTCAGTGGTAGAATTCTCGCCTGCCACGCGGG	5164	913	5896	386
tsrna-14786	GCATTGGTGGTTCAGTGGTAGAATTCTCGCCTGCCACGCGGG	5278	928	5984	424
tsrna-14785	GCATTGGTGGTTCAGTGGTAGAATTCTCGCCTGCCACGCGGG	5239	964	6017	373
tsrna-14784	GCATTGGTGGTTCAGTGGTAGAATTCTCGCCTGCCACGCGGG	5184	1079	5895	455
tsrna-14783	GCATTGGTGGTTCAGTGGTAGAATTCTCGCCTGCCACGCGG	5252	864	6032	374
tsrna-14782	GCATTGGTGGTTCAGTGGTAGAATTCTCGCCTGCCACGCG	5098	888	5899	381
tsrna-14781	GCATTGGTGGTTCAGTGGTAGAATTCTCGCCTGCCACGC	5231	900	5952	410
tsrna-14780	GCATTGGTGGTTCAGTGGTAGAATTCTCGCCTGCCACG	5208	895	5832	378
tsrna-14779	GCATTGGTGGTTCAGTGGTAGAATTCTCGCCTGCCAC	5116	893	5927	366
tsrna-14778	GCATTGGTGGTTCAGTGGTAGAATTCTCGCCTGCCA	5072	853	5865	370
tsrna-14777	GCATTGGTGGTTCAGTGGTAGAATTCTCGCCTGCC	5101	863	5873	384
tsrna-14776	GCATTGGTGGTTCAGTGGTAGAATTCTCGCCTGC	4941	795	5424	381
tsrna-14775	GCATTGGTGGTTCAGTGGTAGAATTCTCGCCTG	4915	788	5384	371
tsrna-14774	GCATTGGTGGTTCAGTGGTAGAATTCTCGCCTCCCACGCGGG	4307	646	5250	373
tsrna-14773	GCATTGGTGGTTCAGTGGTAGAATTCTCGCCTCCCACGCGGG	4344	713	5196	389
tsrna-14772	GCATTGGTGGTTCAGTGGTAGAATTCTCGCCTCCCACGC	4507	638	5178	375
tsrna-14771	GCATTGGTGGTTCAGTGGTAGAATTCTCGCCTCCCAC	4416	702	5133	389
tsrna-14770	GCATTGGTGGTTCAGTGGTAGAATTCTCGCCTCCCA	4380	647	5116	396
tsrna-14769	GCATTGGTGGTTCAGTGGTAGAATTCTCGCCTCCC	4397	694	5156	349
tsrna-14768	GCATTGGTGGTTCAGTGGTAGAATTCTCGCCTCC	4316	713	5240	357

tsrna-14767	GCATTGGTGGTTCAGTGGTAGAATTCTCGCCTC	4256	648	5114	367
tsrna-14766	GCATTGGTGGTTCAGTGGTAGAATTCTCGCCT	4299	661	5017	332
tsrna-14765	GCATTGGTGGTTCAGTGGTAGAATTCTCGCC	2723	397	2966	222
tsrna-14764	GCATTGGTGGTTCAGTGGTAGAATTCTCGC	198	106	203	64
tsrna-14763	GCATTGGTGGTTCAGTGGTAGAATTCTCG	51	30	33	49
tsrna-14762	GCATTGGTGGTTCAGTGGTAGAATTCTC	38	18	34	49
tsrna-14761	GCATTGGTGGTTCAGTGGTAGAATTCT	27	17	23	27
tsrna-14760	GCATTGGTGGTTCAGTGGTAGAATTC	20	21	23	23
tsrna-14759	GCATTGGTGGTTCAGTGGTAGAATT	24	12	21	24
tsrna-14758	GCATTGGTGGTTCAGTGGTAGAAT	15	8	4	8
tsrna-14757	GCATTGGTGGTTCAGTGGTAGAA	7	13	5	4
tsrna-14756	GCATTGGTGGTTCAGTGGTAGA	8	6	5	6
tsrna-14755	GCATTGGTGGTTCAGTGGTAG	5	10	2	1
tsrna-14754	GCATTGGTGGTTCAGTGGTA	2	5	1	1
tsrna-14753	GCATTGGTGGTTCAGTGGT	1	5	1	2
tsrna-14752	GCATTGGTGGTTCAGTGG	2	9	0	1
tsrna-14751	GCATTGGTGGTTCAGTG	0	1	0	0
tsrna-14749	GCATTGGTGGTTCAATGGTAGAATTCTCGCCT	44	10	67	5
tsrna-14748	GCATTGGTGGTTCAATGGTAGAATTCTCGCC	22	13	20	3
tsrna-14747	GCATTGGTGGTTCAATGGTAGAATTCTCGC	8	10	0	0
tsrna-14746	GCATTGGTGGTTCAATGGTAGAATTCTCG	6	5	3	0
tsrna-14745	GCATTGGTGGTTCAATGGTAGAATTCTC	4	8	0	0
tsrna-14744	GCATTGGTGGTTCAATGGTAGAATTCT	3	4	4	0
tsrna-14743	GCATTGGTGGTTCAATGGTAGAA	2	6	2	0
tsrna-14742	GCATTGGTGGTTCAATGGTAGA	3	6	2	0
tsrna-14741	GCATTGGTGGTTCAATG	2	5	1	0
tsrna-14740	GCATTGGCCTCCTAAGCCAGGGATTGTGG	2	5	1	0
tsrna-14738	GCATTGGACTGTAAATCTAAAGACAGGGGTTAGGCC	0	0	3	0
tsrna-14736	GCATTGGACTGTAAATCTAAAGACAGGGG	0	2	0	0
tsrna-14735	GCATTGGACTGTAAATCTAAAGACAGGG	0	2	2	0
tsrna-14733	GCATTGGACTGTAAATCTAAAGACAG	0	1	0	0
tsrna-14732	GCATTGGACTGTAAATCTAAAGACA	0	0	0	0
tsrna-14731	GCATTGGACTGTAAATCTAAAGAC	0	0	1	0
tsrna-14726	GCATTGGACTGTAAATCTA	0	0	0	0
tsrna-14722	GCATTCGGCTGTTAACC	4	5	1	0
tsrna-14721	GCATTAACCTTTTAAGTTAAAGATTAAGAGAACC	1	0	2	0
tsrna-14712	GCATGTGTGAGGTCCCGGGTT	0	2	1	0
tsrna-14711	GCATGTGTGAGGTCCCGGGT	0	3	1	0
tsrna-14709	GCATGTATGAGGTCCCGGGT	7	5	0	0
tsrna-14708	GCATGTATGAGGCCCCGGGTT	1	10	1	1
tsrna-14707	GCATGTATGAGGCCCCGGGT	0	0	1	0
tsrna-14706	GCATGTACGAGGCCCCGGGT	0	3	0	1
tsrna-14705	GCATGGGTGGTTCAGTGGTAGAATTCTCGCCTGCCACGCGG(	1446	543	2055	390
tsrna-14704	GCATGGGTGGTTCAGTGGTAGAATTCTCGCCTGCCACGCG	1510	614	1978	328
tsrna-14703	GCATGGGTGGTTCAGTGGTAGAATTCTCGCCTGCCACG	1461	506	2023	349
tsrna-14702	GCATGGGTGGTTCAGTGGTAGAATTCTCGCCTGCCA	1447	445	2010	326
tsrna-14701	GCATGGGTGGTTCAGTGGTAGAATTCTCGCCTGCC	1412	462	1994	311
tsrna-14700	GCATGGGTGGTTCAGTGGTAGAATTCTCGCCTGC	1410	407	1921	285
tsrna-14699	GCATGGGTGGTTCAGTGGTAGAATTCTCGCCTG	1360	384	1967	302
tsrna-14698	GCATGGGTGGTTCAGTGGTAGAATTCTCGCCT	1343	382	1863	292
tsrna-14697	GCATGGGTGGTTCAGTGGTAGAATTCTCGCC	862	244	1074	213
tsrna-14696	GCATGGGTGGTTCAGTGGTAGAATTCTCGC	72	55	66	60
tsrna-14695	GCATGGGTGGTTCAGTGGTAGAATTCTCG	22	20	24	36
tsrna-14694	GCATGGGTGGTTCAGTGGTAGAATTCTC	12	16	9	35
tsrna-14693	GCATGGGTGGTTCAGTGGTAGAATTCT	12	15	9	29
tsrna-14692	GCATGGGTGGTTCAGTGGTAGAATTC	8	12	4	24
tsrna-14691	GCATGGGTGGTTCAGTGGTAGAATT	9	9	8	18
tsrna-14690	GCATGGGTGGTTCAGTGGTAGAAT	6	6	10	7
tsrna-14689	GCATGGGTGGTTCAGTGGTAGAA	7	3	3	7
tsrna-14688	GCATGGGTGGTTCAGTGGTAGA	5	5	4	4
tsrna-14687	GCATGGGTGGTTCAGTGGTAG	5	4	1	6
tsrna-14686	GCATGGGTGGTTCAGTGGTA	0	4	2	2
tsrna-14685	GCATGGGTGGTTCAGTGGT	0	3	3	2
tsrna-14684	GCATGGGTGGTTCAGTGG	1	4	0	1

tsrna-14683	GCATGGGTGGTTCAGTG	0	0	1	2
tsrna-14682	GCATGGGTGGTTCAGT	1	3	1	1
tsrna-14681	GCATGGGTGATTCAGTGGTAGAA	0	0	0	1
tsrna-14680	GCATGGGTGATTCAGTGGTAGA	1	0	0	0
tsrna-14679	GCATGGGACTCTTAATCCCAGGGTCGTGGG	1	1	0	0
tsrna-14678	GCATGGGACTCTTAATCCC	1	0	0	0
tsrna-14677	GCATGGGACTCTTAATCC	1	0	0	1
tsrna-14675	GCATGGGACTCTTAAT	0	0	1	0
tsrna-14674	GCATGCGAGAGGTAGCGGG	0	0	0	0
tsrna-14673	GCATGCATGAGGTCCCGGGT	2	3	0	0
tsrna-14672	GCATGCACGAGGTCCTGGGTT	5	15	1	10
tsrna-14671	GCATGCACGAGGTCCTGGGT	3	23	1	3
tsrna-14670	GCATGCACGAGGTCCTGGG	0	1	0	0
tsrna-14669	GCATGCACGAGGCCCTGGGT	2	9	1	0
tsrna-14668	GCATGCACGAGGCCCCGGGTT	1	1	0	2
tsrna-14667	GCATGCACGAGGCCCCGGGT	0	4	0	0
tsrna-14663	GCATGAGGTCCCGGGTTCGATCCCCAGC	2	6	0	0
tsrna-14662	GCATGAGGTCCCGGGTTCG	0	3	0	1
tsrna-14661	GCATGAGGTCCCGGGTTC	0	8	1	0
tsrna-14660	GCATGAGGTCCCGGGTT	0	3	0	1
tsrna-14659	GCATGAGGTCCCGGGT	0	1	0	0
tsrna-14658	GCATGAGACTCTTAATCTC	2	5	1	0
tsrna-14657	GCATGAGACTCTTAATCT	1	1	1	0
tsrna-14656	GCATGAGACTCTTAATC	0	1	0	1
tsrna-14651	GCATCAGACTTTTAATCTGAGGGTCCAG	3	33	9	2
tsrna-14650	GCATAGGTGGTTCAGTGGTAGAATTCT	3	3	3	0
tsrna-14649	GCATAGGTGGTTCAGTGGTAGAATTC	3	3	2	1
tsrna-14648	GCATAGGTGGTTCAGTGGTAGAATT	2	1	1	1
tsrna-14647	GCATAGGTGGTTCAGTGGTAGAA	2	2	0	0
tsrna-14646	GCATAGGTGGTTCAGTGGTAGA	0	0	1	0
tsrna-14645	GCATAGCTGCCTTCCAAGCAGTTGACCCGG	0	2	2	2
tsrna-14644	GCATAGCTGCCTTCCAAGCAGTTGACCCG	3	2	2	1
tsrna-14643	GCATAGCTGCCTTCCAAGCAGTT	0	3	0	0
tsrna-14642	GCATAGCTCAGTGGTAGAGCATTTGACTG	4	1	2	1
tsrna-14641	GCATAGCTCAGTGGTAGAGCATTTGACT	0	3	1	0
tsrna-14640	TGACAACATTCAAAAAAGAGTACCA	0	0	0	1
tsrna-14639	TGACAACATTCAAAAAAGAGTACC	1	0	0	0
tsrna-14638	TGACAACAGAGGCTTACGACCCCTTATTT	1	0	0	0
tsrna-14637	TGAATTGCAAATTCGAAGAAGCAGCTTCAAACCTGCCGGGGC	1	3	0	0
tsrna-14636	TGAATCTGACAACAGAGGCTTACGACCCCTTATTTACCCCA	17	11	6	0
tsrna-14635	TGAATCTGACAACAGAGGCTTACGACCCCTTATTTACCCC	15	4	9	1
tsrna-14634	TGAATCTGACAACAGAGGCTTACGACCCCTTATTTACCC	10	7	7	0
tsrna-14633	TGAATCTGACAACAGAGGCTTACGACCCCTTATTTACC	13	7	5	1
tsrna-14632	TGAATCTGACAACAGAGGCTTACGACCCCTTATTTAC	7	4	7	0
tsrna-14631	TGAATCTGACAACAGAGGCTTACGACCCCTTATTTA	20	8	4	0
tsrna-14630	TGAATCTGACAACAGAGGCTTACGACCCCTTATTT	18	7	4	0
tsrna-14629	TGAATCTGACAACAGAGGCTTACGACCCCTTATT	14	3	1	0
tsrna-14628	TGAATCTGACAACAGAGGCTTACGACCCCTTAT	9	12	3	0
tsrna-14627	TGAATCTGACAACAGAGGCTTACGACCCCTTA	6	7	3	0
tsrna-14626	TGAATCTGACAACAGAGGCTTACGACCCCTT	2	1	0	0
tsrna-14625	TGAATCTGACAACAGAGGCTTACGACCCCT	1	1	1	0
tsrna-14624	TGAATCTGACAACAGAGGCTTACGACCCC	0	0	0	0
tsrna-14623	TGAATCTGACAACAGAGGCTTACGACCC	0	0	0	0
tsrna-14622	TGAATCTGACAACAGAGGCTTACGACC	0	1	0	0
tsrna-14621	TGAATCTGACAACAGAGGCTTACGAC	0	0	0	0
tsrna-14620	TGAATCTGACAACAGAGGCTTACGA	0	0	0	0
tsrna-14607	TGAATCCAGCGATCCGAGTTCAAATCTCGGTGGAACCTCCA	153	221	61	27
tsrna-14599	TGAATCCAGCGATCCGAGT	0	1	0	0
tsrna-14593	TGAAGGTCCTGAGTTCGAA	4	7	1	1
tsrna-14592	TGAAGGTCCTGAGTTCG	2	11	2	1
tsrna-14591	TGAAGCCAGTTGATTAG	1	0	0	0
tsrna-14590	TGAAGCATTGGACTGTAAATCTAAAGACAG	1	0	0	0
tsrna-14589	TGAAGCATTGGACTGTAAATCTAAA	0	0	1	0
tsrna-14588	TGAAGCATTGGACTGTAAATCTAA	0	1	1	0

tsrna-14582	TGAAGCATTGGACTGTAA	0	0	0	0
tsrna-14581	TGAAGCATTGGACTGTA	0	0	1	0
tsrna-14579	TGAAGATCTAAAGGTCCCTGGT	10	20	10	2
tsrna-14578	TGAAATACAACGATGGTTTTTCATATCATT	1	0	0	1
tsrna-14576	TGAAACCAGCTTTGGGGGGTTCG	0	0	0	1
tsrna-14574	TGAAAATGTTTAGACGGGCTCACA	52	34	12	2
tsrna-14573	TGAAAATGTTTAGACGGGCTCAC	20	27	3	0
tsrna-14572	TGAAAATGTTTAGACGGGCTCA	14	13	2	2
tsrna-14571	TGAAAATGTTTAGACGGGCTC	7	4	4	1
tsrna-14570	TGAAAATGTTTAGACGGGCT	0	4	1	0
tsrna-14569	TGAAAATGTTTAGACGGGC	0	1	0	0
tsrna-14568	TGAAAACCTTTTTCCAAGGACACCA	2	1	2	0
tsrna-14567	TGAAAACCTTTTTCCAAGGACACC	1	0	0	0
tsrna-14566	TGAAAACCTTTTTCCAAGGACAC	0	0	0	0
tsrna-14565	TGAAAACCTTTTTCCAAGGACA	0	0	0	0
tsrna-14560	TCTTGTAAACCAGGGGTCGCG	0	5	0	0
tsrna-14559	TCTTGGTGGGACCTCCA	1	5	0	0
tsrna-14558	TCTTGCGACCCGGGTTCGATTCCCGGGCGGCGCACCA	15	66	6	2
tsrna-14557	TCTTGCGACCCGGGTTCGATTCCCGGGCGGCGCACC	3	12	4	1
tsrna-14556	TCTTGCGACCCGGGTTCGATTCCCGGGCGGCGC	0	6	0	0
tsrna-14555	TCTTGCGACCCGGGTTCGATTCCCGGGCGGCG	5	2	0	0
tsrna-14554	TCTTGCGACCCGGGTTCGATTCCCGGGCGGC	0	5	0	0
tsrna-14553	TCTTGCGACCCGGGTTCGATTCCCGGGCGG	1	2	0	0
tsrna-14552	TCTTGCGACCCGGGTTCGATTCCCG	0	6	1	0
tsrna-14551	TCTTGCGACCCGGGTTCGATTCCC	1	2	0	0
tsrna-14550	TCTTGCGACCCGGGTTCGATTCC	0	1	0	0
tsrna-14548	TCTTGCGACCCGGGTTCGATT	1	1	0	0
tsrna-14547	TCTTGCGACCCGGGTTCGAT	1	1	0	0
tsrna-14546	TCTTGCGACCCGGGTTCGA	1	0	0	0
tsrna-14545	TCTTGCGACCCGGGTTCG	0	1	0	0
tsrna-14543	TCTTGCGACCCGGGTT	0	1	0	0
tsrna-14542	TCTTCGGGGGCGTGGGTTC	0	4	0	0
tsrna-14541	TCTTCGGGGGCGTGGGTT	1	1	0	0
tsrna-14540	TCTTCGGGGGCGTGGGT	0	4	0	0
tsrna-14538	TCTTCGGAGGCGTGGGTTCG	0	0	0	0
tsrna-14537	TCTTCGGAGGCGTGGGT	0	1	0	0
tsrna-14536	TCTTCGGAGGCGTGGG	0	2	0	0
tsrna-14535	TCTTAGCTTTGGGTGCTAATGGTGGAGTTAAAGACT	2	6	1	3
tsrna-14534	TCTTAGCTTTGGGTGCTAATGGTGGAGTT	0	3	1	0
tsrna-14532	TCTTAATCTCAGGGTCGTGGGT	7	9	1	6
tsrna-14531	TCTTAATCTCAGGGTCGTGGG	7	9	3	3
tsrna-14530	TCTTAATCTCAGGGTCGTGG	7	3	5	1
tsrna-14529	TCTTAATCTCAGGGTCGTG	7	9	0	2
tsrna-14528	TCTTAATCCCAGGGTCGTGGGT	1	0	0	1
tsrna-14527	TCTTAATCCCAGGGTCGTGG	1	0	0	0
tsrna-14526	TCTTAATCCCAGGGTCGTG	1	0	0	0
tsrna-14525	TCTTAATCCCAGGGTCGT	1	0	0	0
tsrna-14524	TCTTAATCCCAGGGTCG	0	0	0	1
tsrna-14514	TCTGTAGTGTAGTGGTTATCACG	0	0	0	0
tsrna-14509	TCTGGTCTCCGGATGGAGGCGTGGGTTCGAATCCCACTTCTG	5	59	10	1
tsrna-14508	TCTGGTCTCCGGATGGAGGCGTGG	1	9	0	1
tsrna-14504	TCTGGTCTCCAATGGAGGCGTGG	0	1	0	0
tsrna-14503	TCTGGGTGCCCCCTCCA	0	2	0	0
tsrna-14499	TCTGGACTCTGAATCCGGTAAT	0	0	0	1
tsrna-14497	TCTGGACTCTGAATCCGGT	0	1	0	0
tsrna-14495	TCTGGACTCTGAATCCAGCGATCCGAGTTC	1	2	2	0
tsrna-14494	TCTGGACTCTGAATCCAGCGATCCGAGTT	1	1	0	0
tsrna-14493	TCTGGACTCTGAATCCAGCGATCCGAGT	0	0	1	0
tsrna-14492	TCTGGACTCTGAATCCAGCGATCCGAG	0	3	0	0
tsrna-14491	TCTGGACTCTGAATCCAGCGATCCGA	0	2	0	0
tsrna-14490	TCTGGACTCTGAATCCAGCGATCCG	1	1	0	0
tsrna-14489	TCTGGACTCTGAATCCAGCGATCC	1	1	1	0
tsrna-14488	TCTGGACTCTGAATCCAGCGATC	0	0	1	0
tsrna-14487	TCTGGACTCTGAATCCAGCGAT	0	2	0	0

tsrna-14486	TCTGGACTCTGAATCCAGCGA	1	3	0	0
tsrna-14485	TCTGGACTCTGAATCCAGCG	0	1	0	0
tsrna-14484	TCTGGACTCTGAATCCAGC	1	0	0	0
tsrna-14483	TCTGGACTCTGAATCCAG	0	2	0	0
tsrna-14480	TCTGCTTTACACGCAGAAGGTCCTGGGTTC	14	31	9	2
tsrna-14479	TCTGATTCCGGATCAGAAGATTGAGGGTTC	4	16	2	1
tsrna-14475	TCTGAGGGTCCAGGGTTCATGTCCCTGTTC	0	1	0	0
tsrna-14473	TCTGAGGGTCCAGGGTTCATGTCCCTG	0	1	0	0
tsrna-14472	TCTGAGGGTCCAGGGTTCATGTCCCT	0	1	0	0
tsrna-14471	TCTGAGGGTCCAGGGTTCATGTCCC	0	1	0	0
tsrna-14470	TCTGAGGGTCCAGGGTTCATG	0	1	0	0
tsrna-14469	TCTGAGGGTCCAGGGTTCAAGTCCCTGTTCGGGCGCCA	8	14	5	4
tsrna-14468	TCTGAGGGTCCAGGGTTCAAGTCCCTGTTCGGGCGCC	1	3	1	0
tsrna-14467	TCTGAGGGTCCAGGGTTCAAGTCCCTGTTCGGGCGC	0	3	1	0
tsrna-14466	TCTGAGGGTCCAGGGTTCAAGTCCCTGTTCAGGCGCCA	0	7	0	0
tsrna-14465	TCTGAGGGTCCAGGGTTCAAGTCCCTGTTC	0	0	0	0
tsrna-14464	TCTGAGGGTCCAGGGTTCAAGTCCCTGTCCAGGCGCCA	1	2	0	0
tsrna-14463	TCTGAGGGTCCAGGGTTCAAGT	0	1	0	0
tsrna-14462	TCTGAGGGTCCAGGGTTCAAG	0	1	1	0
tsrna-14461	TCTGAGGGTCCAGGGTTCAA	0	2	0	0
tsrna-14460	TCTGAGGGTCCAGGGTTCA	0	1	0	0
tsrna-14459	TCTGAGGGTCCAGGGTTC	1	0	0	0
tsrna-14454	TCTGACTTCGGATCAGAAGATTGC	1	0	0	0
tsrna-14453	TCTGACTTCGGATCAGAAGATTGAGGGTTC	7	7	5	1
tsrna-14452	TCTGACTTCGGATCAGAAGATTGAGGGT	1	8	1	1
tsrna-14443	TCTGACTCCAGATCAGAAGGTTGCGTGTT	2	4	2	0
tsrna-14432	TCTGACAACAGAGGCTTACGACCCCTTATTTACCCCA	2	1	0	0
tsrna-14431	TCTGACAACAGAGGCTTACGACCCCTTATTTACCCC	0	0	0	0
tsrna-14428	TCTGACAACAGAGGCTTACGACCCCTTAT	0	0	0	0
tsrna-14427	TCTGACAACAGAGGCTTACGACCCCTTA	0	0	0	0
tsrna-14426	TCTGACAACAGAGGCTTACGACCCCTT	0	0	1	0
tsrna-14425	TCTGACAACAGAGGCTTACGACCCCT	0	0	0	0
tsrna-14416	TCTGAATCCAGCGATCCGAGTTCGAGTCTCGGTGGAACCT	1	2	1	1
tsrna-14415	TCTGAATCCAGCGATCCGAGTTCA	0	2	0	0
tsrna-14414	TCTGAATCCAGCGATCCGAGTTC	0	0	0	1
tsrna-14412	TCTGAATCCAGCGATCCGAGT	0	0	1	0
tsrna-14410	TCTGAATCCAGCGATCCGA	0	1	0	0
tsrna-14406	TCTGAAGGTCGTGAGTTCGATCCTCACACGGGGCACCA	3	23	2	0
tsrna-14405	TCTGAAGGTCGTGAGTTCGATCCTCACACGGGGCACC	5	11	4	0
tsrna-14404	TCTGAAGGTCGTGAGTTCGATCCTCACACGGGGCAC	2	4	2	0
tsrna-14403	TCTGAAGGTCGTGAGTTCGATCCTCACACG	0	6	0	0
tsrna-14402	TCTGAAGGTCGTGAGTTCGAGCCTCACACGGGGCACCA	1	8	0	1
tsrna-14401	TCTGAAGGTCGTGAGTTCGAGCCTCACACGGGGCACC	2	7	2	0
tsrna-14400	TCTGAAGGTCGTGAGTTCGAGCCTCACACGGGGCAC	2	2	2	0
tsrna-14399	TCTGAAGGTCGTGAGTTCGAGCCTCACACG	1	3	1	0
tsrna-14398	TCTGAAGGTCGTGAGTTCG	2	3	3	1
tsrna-14397	TCTGAAGGTCGTGAGTTC	0	2	0	0
tsrna-14396	TCTGAAGGTCGTGAGTT	3	5	1	0
tsrna-14395	TCTGAAGGTCGTGAGT	2	5	3	0
tsrna-14394	TCTGAAGGTCCTGAGTTCGAGCCTCAGAGAGGGCACCA	10	36	10	5
tsrna-14393	TCTGAAGGTCCTGAGTTCGAACCTCAGAGGGGGCACCA	11	46	8	1
tsrna-14392	TCTGAAGGTCCTGAGTTCGAACCTCAGAGGGGGCACC	16	41	13	1
tsrna-14391	TCTGAAGGTCCTGAGTTCGAACCTCAGAGGGGGCAC	14	36	4	1
tsrna-14390	TCTGAAGGTCCTGAGTTCGAACCTCAGAGG	8	33	10	3
tsrna-14389	TCTGAAGGTCCTGAGTTCGAACC	18	24	4	5
tsrna-14388	TCTGAAGGTCCTGAGTTCGAAC	8	28	13	1
tsrna-14387	TCTGAAGGTCCTGAGTTCGA	18	34	7	3
tsrna-14386	TCTGAAGGTCCTGAGTTCG	20	34	6	3
tsrna-14385	TCTGAAGGTCCTGAGTTC	6	49	5	6
tsrna-14384	TCTGAAGGTCCTGAGT	5	33	8	4
tsrna-14383	TCTCTTCGGGGGCGTGGGT	0	3	1	0
tsrna-14379	TCTCTTCGGAGGCGTGG	1	1	0	0
tsrna-14371	TCTCTGTGGAACCTCCA	0	1	0	0
tsrna-14369	TCTCGGTGGGACCTCCA	24	49	9	4

tsrna-14368	TCTCGGTGGGACCTCC	2	5	0	1
tsrna-14367	TCTCGGTGGGACCCCCA	0	3	0	1
tsrna-14366	TCTCGGTGGAACCTCCA	37	96	20	10
tsrna-14365	TCTCGGTGGAACCTCC	2	4	2	0
tsrna-14360	TCTCGCTGGGGCCTCCA	26	27	17	10
tsrna-14359	TCTCGCTGGGGCCTCC	3	4	0	0
tsrna-14358	TCTCGCCTGCCACGCGGGAGGCCCGGGT	10	26	3	3
tsrna-14357	TCTCGCCTGCCACGCGGGAGGCCCGGG	2	1	0	0
tsrna-14356	TCTCGCCTGCCACGCGGGAGGCCCG	2	2	0	0
tsrna-14355	TCTCGCCTGCCACGCGGGAGGCCC	0	3	0	0
tsrna-14354	TCTCGCCTGCCACGCGGGAGGCC	1	1	1	1
tsrna-14353	TCTCGCCTGCCACGCGGGAGG	0	1	1	0
tsrna-14352	TCTCGCCTGCCACGCGGGA	0	1	0	0
tsrna-14351	TCTCGCCTGCCACGCGGG	0	1	0	0
tsrna-14349	TCTCGCCTGCCACGCG	0	1	0	0
tsrna-14347	TCTCCGGATGGAGGCGTGGGTTCGAA	5	38	7	1
tsrna-14346	TCTCCGGATGGAGGCGTGGGTTCG	12	46	3	0
tsrna-14345	TCTCCGGATGGAGGCGTGGGT	5	37	1	1
tsrna-14344	TCTCCGGATGGAGGCGTGGG	1	4	0	1
tsrna-14343	TCTCCGGATGGAGGCGTGG	3	2	1	0
tsrna-14342	TCTCCGGATGGAGGCGTG	1	3	0	1
tsrna-14341	TCTCCGGATGGAGGCGT	1	0	0	0
tsrna-14340	TCTCCCCTGGAGGCGTGGGT	0	2	1	0
tsrna-14339	TCTCCCCTGGAGGCGTGG	0	1	0	0
tsrna-14338	TCTCCAATGGAGGCGTGGGTTCGAATCCCACTTCTG	18	24	13	0
tsrna-14337	TCTCCAATGGAGGCGTGGGT	11	11	9	1
tsrna-14336	TCTCATAGTCCTAGCCA	0	0	1	0
tsrna-14334	TCTCATAATCTGAAGGTCGT	0	0	1	0
tsrna-14333	TCTCATAATCTGAAGGTCCTGAGT	134	358	112	66
tsrna-14332	TCTCATAATCTGAAGGTCCTGAG	9	27	8	4
tsrna-14331	TCTCATAATCTGAAGGTCCTGA	2	14	8	1
tsrna-14330	TCTCATAATCTGAAGGTCCT	3	5	3	0
tsrna-14329	TCTCAGGGTCGTGGGTTCGAGCCCCACGTTGGGCGCCA	5	248	3	5
tsrna-14328	TCTCAGGGTCGTGGGTTCGAGCCCCACGTTGGGCGC	0	0	1	1
tsrna-14327	TCTCAGGGTCGTGGGTTCGAGCCCCACGTT	0	1	0	1
tsrna-14326	TCTCAGGGTCGTGGGTTCG	0	1	1	0
tsrna-14325	TCTCAGGGATGGGTTCGATTCTCATAGTCCT	9	15	9	10
tsrna-14324	TCTCAGGGATGGGTTCGATTCTC	0	9	6	0
tsrna-14323	TCTCAGGGATGGGTTCGATTCT	5	4	2	1
tsrna-14322	TCTCAGGGATGGGTTCGATTC	3	4	0	0
tsrna-14321	TCTCACCGCCGCGGCCCGGGTTCGATTCCCGGTCAGGGAAC	32	316	19	3
tsrna-14320	TCTCACCGCCGCGGCCCGGGTTCGATTCCCGGTCAGGGAAC	8	13	5	1
tsrna-14319	TCTCACCGCCGCGGCCCGGGTTCGATTCCCGGTCA	0	1	0	0
tsrna-14318	TCTCACCGCCGCGGCCCGGGTTCGATTCCC	0	1	0	0
tsrna-14315	TCTCACCGCCGCGGCCCGGGT	0	1	1	0
tsrna-14312	TCTCACCGCCGCGGCCCG	0	1	0	0
tsrna-14309	TCTAGTGGTTAGGATTCGGCGCTCTCACCGCCGCGGCCC	63	84	39	20
tsrna-14308	TCTAGTGGTTAGGATTCGGCGCTCTCACCGCCGCGG	44	58	21	22
tsrna-14307	TCTAGTGGTTAGGATTCGGCGCTCTCACCGCC	36	49	25	15
tsrna-14306	TCTAGTGGTTAGGATTCGGCGCTCTCACCG	37	41	24	15
tsrna-14305	TCTAGTGGTTAGGATTCGGCGCTCTCACC	38	50	23	9
tsrna-14304	TCTAGTGGTTAGGATTCGGCGCTCTCAC	41	54	16	16
tsrna-14303	TCTAGTGGTTAGGATTCGGCGCTCTCA	50	35	28	17
tsrna-14302	TCTAGTGGTTAGGATTCGGCGCTCTC	32	52	22	22
tsrna-14301	TCTAGTGGTTAGGATTCGGCGCTCT	32	38	19	14
tsrna-14300	TCTAGTGGTTAGGATTCGGCGCTC	30	51	21	15
tsrna-14299	TCTAGTGGTTAGGATTCGGCGCT	31	34	22	8
tsrna-14298	TCTAGTGGTTAGGATTCGGCGC	14	17	8	6
tsrna-14297	TCTAGTGGTTAGGATTCGGCG	8	11	2	6
tsrna-14296	TCTAGTGGTTAGGATTCGGC	13	2	6	2
tsrna-14295	TCTAGTGGTTAGGATTCGG	1	0	2	2
tsrna-14294	TCTAGTGGTTAGGATTCG	3	1	2	0
tsrna-14293	TCTAGTGGTTAGGATTC	3	3	1	0
tsrna-14292	TCTAGTGGCTAGGATTCGGCGCT	13	40	7	24

tsrna-14291	TCTAGTGGCTAGGATTCGGCGC	13	51	8	32
tsrna-14290	TCTAGTGGCTAGGATTCGG	0	0	0	0
tsrna-14289	TCTAGGTTCGACTCCTGGCTGGCTCGCC	3	4	1	0
tsrna-14288	TCTAGGTTCGACTCCTGGCTGGCTCGC	0	1	0	0
tsrna-14287	TCTAGGTTCGACTCCTGGCTGGCTCG	0	0	0	0
tsrna-14286	TCTAGGTTCGACTCCTGGCTGGCTC	0	0	0	0
tsrna-14285	TCTAGGTTCGACTCCTGGCTGGCT	0	1	0	0
tsrna-14282	TCTAGGGGTATGATTCTCGGTT	3	1	1	3
tsrna-14281	TCTAGGGGTATGATTCTCGGT	3	2	2	0
tsrna-14280	TCTAGGGGTATGATTCTCGG	2	1	1	0
tsrna-14279	TCTAGGGGTATGATTCTCGCTT	18	24	12	11
tsrna-14278	TCTAGGGGTATGATTCTCGCT	19	29	7	16
tsrna-14277	TCTAGGGGTATGATTCTCGC	6	7	6	12
tsrna-14276	TCTAGGGGTATGATTCTCG	0	0	0	0
tsrna-14274	TCTAGCGGTTAGGATTCCTGGTTTT	7	8	2	3
tsrna-14273	TCTAGCGGTTAGGATTCCTGGTT	3	8	1	1
tsrna-14272	TCTAGCGGTTAGGATTCCTGGT	10	5	0	0
tsrna-14271	TCTAGCGGTTAGGATTCCTGG	2	0	4	0
tsrna-14270	TCTAGCGGTTAGGATTCCTG	2	1	1	0
tsrna-14268	TCTAATTCAAAGGTTGTGGGTTCGAGTCCCACCAGAGTCGCC	3	23	2	4
tsrna-14265	TCTAACAACATGGCTTTCTCACCA	2	0	1	0
tsrna-14264	TCTAACAACATGGCTTTCTCACC	0	1	0	0
tsrna-14263	TCTAACAACATGGCTTTCTCAC	1	0	0	0
tsrna-14262	TCTAACAACATGGCTTTCTCA	1	0	0	0
tsrna-14259	TCTAAAGGTCCCTGGTTCGATCCCGGGTTTCGGCACCA	54	131	22	23
tsrna-14258	TCTAAAGGTCCCTGGTTCGATCCCGGGTTTCGGCACC	23	27	3	6
tsrna-14257	TCTAAAGGTCCCTGGTTCGATCCCGGGTTTCGGCAC	9	16	8	6
tsrna-14256	TCTAAAGGTCCCTGGTTCGATCCCGGGTTT	10	17	15	2
tsrna-14255	TCTAAAGGTCCCTGGTTCGATC	13	23	9	6
tsrna-14254	TCTAAAGGTCCCTGGTTCGAT	18	14	12	1
tsrna-14253	TCTAAAGGTCCCTGGTTCGA	18	15	5	5
tsrna-14252	TCTAAAGGTCCCTGGTTCG	18	14	9	1
tsrna-14251	TCTAAAGGTCCCTGGTT	11	22	11	4
tsrna-14250	TCTAAAGGTCCCTGGT	6	15	4	1
tsrna-14249	TCTAAAGACAGGGGTTAGGCCTCTTTTTAC	1	2	0	0
tsrna-14248	TCTAAAGACAGGGGTTAGGCCTCT	0	1	2	0
tsrna-14247	TCTAAAGACAGGGGTTAGGCCTC	1	1	1	0
tsrna-14246	TCTAAAGACAGGGGTTAGGCCT	1	0	1	0
tsrna-14244	TCGTTGGTCTAGTGGTATGATTCTCGC	9	5	5	1
tsrna-14242	TCGTTGGTCTAGTGGTATGAT	0	0	1	0
tsrna-14240	TCGTTGGTCTAGGGGTATGATTCTCGGTT	9	4	3	1
tsrna-14239	TCGTTGGTCTAGGGGTATGATTCTCGGT	3	2	0	3
tsrna-14238	TCGTTGGTCTAGGGGTATGATTCTCGG	0	2	3	0
tsrna-14237	TCGTTGGTCTAGGGGTATGATTCTCGCTTC	26	27	15	15
tsrna-14236	TCGTTGGTCTAGGGGTATGATTCTCGCTT	23	22	19	23
tsrna-14235	TCGTTGGTCTAGGGGTATGATTCTCGCT	25	22	13	16
tsrna-14234	TCGTTGGTCTAGGGGTATGATTCTCGC	7	6	13	21
tsrna-14233	TCGTTGGTCTAGGGGTATGATTCTCG	1	1	0	0
tsrna-14232	TCGTTGGTCTAGGGGTATGATTCTC	1	0	1	0
tsrna-14231	TCGTTGGTCTAGGGGTATGATTCT	0	0	0	0
tsrna-14230	TCGTTGGTCTAGGGGTATGATTC	0	0	0	0
tsrna-14222	TCGTTAGTATAGTGGTTAGTATCCCCGCC	1	1	0	0
tsrna-14221	TCGTTAGTATAGTGGTTAGTATCCCCG	0	1	0	0
tsrna-14220	TCGTTAGTATAGTGGTTAGTATCCCC	0	1	0	0
tsrna-14217	TCGTTAGTATAGTGGTTAGTATC	0	1	0	0
tsrna-14216	TCGTTAGTATAGTGGTTAGT	0	1	0	0
tsrna-14215	TCGTTAGTATAGTGGTGAGTATCCCCGCCT	1	5	2	0
tsrna-14214	TCGTTAGTATAGTGGTGAGTATCCCCGCC	2	6	0	2
tsrna-14213	TCGTTAGTATAGTGGTGAGTATCCCCGC	1	3	0	0
tsrna-14212	TCGTTAGTATAGTGGTGAGTATCCCCG	1	3	0	0
tsrna-14211	TCGTTAGTATAGTGGTGAGTATCCCC	0	2	0	0
tsrna-14210	TCGTTAGTATAGTGGTGAGTATCCC	0	1	1	0
tsrna-14208	TCGTTAGTATAGTGGTGAGTATC	0	0	1	0
tsrna-14206	TCGTTAGTATAGTGGTGAGTA	0	1	0	0

tsrna-14205	TCGTTAGTATAGTGGTGAGT	0	1	0	0
tsrna-14198	TCGTGGTTGTAGTCCGTGCGAGAATACCA	10	23	10	7
tsrna-14197	TCGTGGTTGTAGTCCGTGCGAGAATACC	2	9	6	0
tsrna-14196	TCGTGGTTGTAGTCCGTGCGAGAATAC	2	1	1	0
tsrna-14195	TCGTGGTTGTAGTCCGTGCGAGAATA	3	1	2	0
tsrna-14194	TCGTGGTTGTAGTCCGTGCGAGAAT	0	3	2	1
tsrna-14193	TCGTGGTTGTAGTCCGTGCGAGAA	1	1	0	0
tsrna-14192	TCGTGGTTGTAGTCCGTGCGAGA	0	0	0	0
tsrna-14191	TCGTGGTTGTAGTCCGTGCGAG	0	2	0	0
tsrna-14190	TCGTGGTTGTAGTCCGTGCGA	0	0	0	0
tsrna-14189	TCGTGGTTGTAGTCCGTGCG	0	0	1	1
tsrna-14187	TCGTGGTTGTAGTCCGTG	0	0	1	0
tsrna-14184	TCGTGGGTTCGAGCCCCACGTTGGGCGCCA	11	237	10	5
tsrna-14183	TCGTGGGTTCGAGCCCCACGTTGGGCGCC	0	12	2	0
tsrna-14182	TCGTGGGTTCGAGCCCCACGTTGGGCGC	0	2	0	0
tsrna-14175	TCGTGAGTTCGATCCTCACACGGGGCACC	0	1	0	0
tsrna-14173	TCGTATAGTGGTTAGTACTCTGCGTTGTGGCCGCAGCAACCT	44	22	32	12
tsrna-14172	TCGTATAGTGGTTAGTACTCTGCGTTGTGG	34	15	16	9
tsrna-14171	TCGTATAGTGGTTAGTACTCTGCGTTGTG	23	15	17	5
tsrna-14170	TCGTATAGTGGTTAGTACTCTGCGTTGT	23	21	12	9
tsrna-14169	TCGTATAGTGGTTAGTACTCTGCGTTG	32	13	12	4
tsrna-14168	TCGTATAGTGGTTAGTACTCTGCGTT	27	11	16	9
tsrna-14167	TCGTATAGTGGTTAGTACTCTGCGT	8	3	4	1
tsrna-14166	TCGTATAGTGGTTAGTACTCTGCGCTG	7	3	4	3
tsrna-14165	TCGTATAGTGGTTAGTACTCTGCGC	7	3	4	2
tsrna-14164	TCGTATAGTGGTTAGTACTCTGCG	2	4	3	1
tsrna-14163	TCGTATAGTGGTTAGTACTCTGC	2	1	0	0
tsrna-14162	TCGTATAGTGGTTAGTACTCTG	1	1	1	2
tsrna-14161	TCGTATAGTGGTTAGTACTCT	2	1	2	0
tsrna-14160	TCGTATAGTGGTTAGTACTC	2	0	0	2
tsrna-14159	TCGTATAGTGGTTAGTACT	1	0	0	0
tsrna-14155	TCGTAAACCGAAGATCGCGGGTTCGAACCCCGTCCGTGCCTC	66	177	27	12
tsrna-14154	TCGTAAACCGAAGATCGCGGGTTCGAACCCCGTCCGTGCCTC	39	200	25	9
tsrna-14153	TCGTAAACCGAAGATCGCGGGTTCGAACCC	46	168	33	8
tsrna-14152	TCGTAAACCGAAGATCGCGGGTT	42	163	27	10
tsrna-14151	TCGTAAACCGAAGATCGCGGGT	44	159	26	4
tsrna-14150	TCGTAAACCGAAGATCGCGGG	1	3	1	0
tsrna-14148	TCGTAAACCGAAGATCACGGGTTCGAACCCCGTCCGTGCCTC	24	96	14	5
tsrna-14147	TCGTAAACCGAAGATCACGGGTTCGAACCC	9	84	1	2
tsrna-14146	TCGTAAACCGAAGATCACGGGT	8	73	4	4
tsrna-14145	TCGTAAACAGGAGATCCTGGGT	17	158	14	7
tsrna-14144	TCGTAAACAGGAGATCC	5	5	0	2
tsrna-14141	TCGGTTAGCGCGTTCGGCTGTT	0	0	1	0
tsrna-14139	TCGGTAGAGCTATCAGACTTTTA	0	1	0	1
tsrna-14136	TCGGTAGAGCTATCAGAC	0	0	0	2
tsrna-14135	TCGGTAGAGCTATCAGA	0	0	0	1
tsrna-14134	TCGGTAGAGCATGGGACTCTTAATCTC	10	26	10	1
tsrna-14133	TCGGTAGAGCATGGGACTCTTAATCCCAGGGTCGTGGGTTC	24	29	15	4
tsrna-14132	TCGGTAGAGCATGGGACTCTTAATCCCAGGGTCGTGGGT	16	23	12	4
tsrna-14131	TCGGTAGAGCATGGGACTCTTAATCCCAGGGTCGTGGG	22	18	15	6
tsrna-14130	TCGGTAGAGCATGGGACTCTTAATCCCAGGGTCGTGG	12	23	8	5
tsrna-14129	TCGGTAGAGCATGGGACTCTTAATCCC	13	25	9	4
tsrna-14128	TCGGTAGAGCATGGGACTCTTAATCC	15	16	4	5
tsrna-14127	TCGGTAGAGCATGGGACTCTTAATC	6	16	3	3
tsrna-14126	TCGGTAGAGCATGGGACTCTTAAT	5	5	3	0
tsrna-14125	TCGGTAGAGCATGGGACTCT	7	9	2	2
tsrna-14124	TCGGTAGAGCATGGGACTC	9	12	4	2
tsrna-14123	TCGGTAGAGCATGGGACT	4	9	3	0
tsrna-14122	TCGGTAGAGCATGGGAC	7	11	6	1
tsrna-14121	TCGGTAGAGCATGGGA	5	7	1	1
tsrna-14120	TCGGTAGAGCATGAGACTCTTAATCTCAGGGTCGTGGGT	19	38	9	13
tsrna-14119	TCGGTAGAGCATGAGACTCTTAATCTC	8	22	2	4
tsrna-14118	TCGGTAGAGCATGAGACTCTTAATCT	11	27	3	4
tsrna-14117	TCGGTAGAGCATGAGACTCTTAATC	7	19	4	6

tsrna-14116	TCGGTAGAGCATGAGACTCTTA	13	23	8	4
tsrna-14115	TCGGTAGAGCATGAGACTCTT	9	23	7	4
tsrna-14114	TCGGTAGAGCATGAGACTC	6	16	4	3
tsrna-14113	TCGGTAGAGCATGAGACT	7	13	3	4
tsrna-14112	TCGGTAGAGCATGAGACCC	5	17	2	3
tsrna-14111	TCGGTAGAGCATGAGAC	6	12	2	2
tsrna-14110	TCGGTAGAGCATGAGA	2	4	1	0
tsrna-14109	TCGGTAGAGCATCAGACTTTTAATCTG	3	5	2	1
tsrna-14108	TCGGTAGAGCATCAGACTTTTAATCT	0	6	0	1
tsrna-14107	TCGGTAGAGCATCAGACTTTTAATC	5	2	1	1
tsrna-14106	TCGGTAGAGCATCAGACTTTTAAT	3	2	0	0
tsrna-14105	TCGGTAGAGCATCAGACTTTTA	2	4	1	0
tsrna-14104	TCGGTAGAGCATCAGACTTTT	1	2	1	2
tsrna-14103	TCGGTAGAGCATCAGACTTT	0	2	1	0
tsrna-14102	TCGGTAGAGCATCAGACTT	2	4	0	1
tsrna-14101	TCGGTAGAGCATCAGACT	1	1	0	0
tsrna-14100	TCGGTAGAGCATCAGAC	0	5	1	0
tsrna-14099	TCGGTAGAGCATCAGA	1	4	0	0
tsrna-14098	TCGGTAGAGCATAAGAC	11	17	6	2
tsrna-14097	TCGGGTGTGAGAGGTCCCGGGTT	0	7	0	0
tsrna-14096	TCGGGTGTGAGAGGTCCCGGGT	0	0	0	0
tsrna-14095	TCGGGTGCGAGAGGTCCCGGGTT	3	2	0	1
tsrna-14094	TCGGGTGCGAGAGGTCCCGGGT	0	3	0	0
tsrna-14092	TCGGGTGCGAGAGGTCCCGG	0	1	0	0
tsrna-14085	TCGGCTGTTAACCGAAAGGTTGGTGGT	27	116	21	39
tsrna-14084	TCGGCTGTTAACCGAAAGGTTGGTGG	4	21	7	6
tsrna-14083	TCGGCTGTTAACCGAAAGGTTGGTG	2	33	5	10
tsrna-14082	TCGGCTGTTAACCGAAAGGTTGG	5	10	0	4
tsrna-14081	TCGGCTGTTAACCGAAAGGTTG	4	14	6	3
tsrna-14080	TCGGCGCTTTCACCGCCGCGGCCC	0	3	1	5
tsrna-14079	TCGGCGCTCTCACCGCCGCGGCCCGGGTTC	0	0	0	0
tsrna-14077	TCGGCGCTCTCACCGCCGCGGCCCGGGT	1	0	0	0
tsrna-14074	TCGGCGCTCTCACCGCCGCGG	0	1	0	0
tsrna-14073	TCGGCGCTCTCACCGCCG	0	1	0	0
tsrna-14071	TCGGCGCTCTCACCGC	0	0	0	1
tsrna-14070	TCGGATCAGAAGATTGCAGGTTCGAGTCCTGCCGCGGTCGCC	2	5	1	0
tsrna-14068	TCGGATCAGAAGATTGCAGGTT	0	0	0	0
tsrna-14064	TCGGATCAGAAGATTGAGGGTTCGAGTCCCTTCGTGGTCGCC	7	11	4	5
tsrna-14063	TCGGATCAGAAGATTGAGGGTTCGAATCCCTTCGTGGTTGCCA	5	16	5	2
tsrna-14062	TCGGATCAGAAGATTGAGGGTTC	5	15	1	2
tsrna-14061	TCGGATCAGAAGATTGAGGGT	4	6	2	0
tsrna-14055	TCGCTTCGGGTGTGAGAGGTCCC	0	1	0	0
tsrna-14049	TCGCTGGTTCGATTCCGGCTCGAAGGACCA	77	656	28	31
tsrna-14048	TCGCTGGTTCGATTCCGGCTCGAAGGACC	2	18	3	3
tsrna-14047	TCGCTGGTTCGATTCCGGCTCGAAGGAC	0	0	0	1
tsrna-14045	TCGCTGGTTCGATTCCGGCTCGAAGG	0	1	0	0
tsrna-14043	TCGCTGGTTCGATTCCGGC	0	1	0	0
tsrna-14041	TCGCTGGTTCGATTCCAGCTCGAAGGACC	0	1	0	0
tsrna-14039	TCGCTGGTTCGAATCCGGCTCGAAGGACC	0	7	2	0
tsrna-14037	TCGCTGGTTCGAATCCGGCTCGAAGGA	0	2	0	0
tsrna-14033	TCGCGGGTTCGATCCCCGTACTGGCCACC	0	1	1	0
tsrna-14032	TCGCGGGTTCGATCCCCGTACTGGCCAC	0	0	0	0
tsrna-14031	TCGCGGGTTCGATCCCCGTACTGGCCA	0	1	1	0
tsrna-14030	TCGCGGGTTCGATCCCCGTACGGGCCACCA	8	124	5	5
tsrna-14029	TCGCGGGTTCGATCCCCGTACGGGCCACC	2	9	1	1
tsrna-14023	TCGCGGGTTCGAACCCCGTACGGGCCACC	0	0	0	0
tsrna-14022	TCGCGAGTTCAAATCTCGCTGGGGCCTCC	1	4	2	2
tsrna-14021	TCGCGAGTTCAAATCTCGCTGGGGCCTC	1	0	0	0
tsrna-14020	TCGCCTGCCACGCGGGAGGCCCGGGTT	2	29	5	3
tsrna-14019	TCGCCTGCCACGCGGGAGGCCCGGGT	3	22	4	1
tsrna-14018	TCGCCTGCCACGCGGGAGGCCCGGG	2	0	0	0
tsrna-14017	TCGCCTGCCACGCGGGAGGCCCGG	0	1	0	0
tsrna-14013	TCGCATGTGTGAGGTCCCGGGT	0	4	1	0
tsrna-14011	TCGCATGTATGAGGTCCCGGGTT	1	5	0	1

tsrna-14010	TCGCATGTATGAGGTCCCGGGT	0	6	0	0
tsrna-14009	TCGCATGTATGAGGTCCC	0	0	1	0
tsrna-14008	TCGCATGTACGAGGCCCCGGGT	0	1	0	0
tsrna-14007	TCGCATAAAACTTAAAACTT	1	0	0	0
tsrna-14006	TCGATTCTCATAGTCCTAGCCA	0	2	0	1
tsrna-14005	TCGATTCTCATAGTCCTAGCC	0	0	0	0
tsrna-14004	TCGATTCTCATAGTCCTAGC	1	0	0	0
tsrna-14003	TCGATTCTCATAGTCCTAG	0	0	0	0
tsrna-14002	TCGATTCTCATAGTCCTA	0	2	0	1
tsrna-14001	TCGATTCCTTCCTTTTTTGCCA	0	2	0	1
tsrna-13998	TCGATTCCTTCCTTTTTTG	0	1	0	0
tsrna-13997	TCGATTCCGGCTCGAAGGACCA	64	159	32	18
tsrna-13996	TCGATTCCGGCTCGAAGGACC	4	9	1	0
tsrna-13994	TCGATTCCGGCTCGAAGGA	0	1	0	0
tsrna-13991	TCGATTCCCTGACGGGGAGCCA	0	2	0	0
tsrna-13990	TCGATTCCCGGTCAGGGAACCA	10	283	5	4
tsrna-13989	TCGATTCCCGGTCAGGGAACC	1	11	0	0
tsrna-13988	TCGATTCCCGGTCAGGGAAC	0	5	0	0
tsrna-13987	TCGATTCCCGGTCAGGGAA	0	2	0	0
tsrna-13985	TCGATTCCCGGGCGGCGCACCA	22	75	8	3
tsrna-13984	TCGATTCCCGGGCGGCGCACC	7	7	1	0
tsrna-13983	TCGATTCCCGGGCGGCGCAC	0	1	0	0
tsrna-13979	TCGATTCCCGGCCCATGCACCA	32	37	10	13
tsrna-13978	TCGATTCCCGGCCCATGCACC	2	2	0	1
tsrna-13975	TCGATTCCCGGCCAATGCACCA	31	140	10	10
tsrna-13974	TCGATTCCCGGCCAATGCACC	1	8	0	0
tsrna-13973	TCGATTCCCGGCCAATGCAC	0	1	0	0
tsrna-13972	TCGATTCCCGGCCAATGCA	0	2	0	0
tsrna-13971	TCGATTCCCGGCCAACGCACCA	44	104	22	16
tsrna-13970	TCGATTCCCGGCCAACGCACC	15	11	1	1
tsrna-13968	TCGATTCCCGGCCAACGCA	0	0	0	0
tsrna-13963	TCGATTCCCCGACGGGGAGCCA	0	20	1	1
tsrna-13962	TCGATTCCCCGACGGGGAGCC	0	0	0	0
tsrna-13959	TCGATTCCCCGACGGGGA	0	1	0	0
tsrna-13956	TCGATTCCCAGCCAATGCACCA	2	6	0	0
tsrna-13952	TCGATGGATCGAAACCATCCTCTGCTACC	0	3	0	1
tsrna-13951	TCGATGGATCGAAACCATCCTCTGCTAC	0	0	1	0
tsrna-13945	TCGATGCCCGCATTCTCCACCA	2	5	0	0
tsrna-13944	TCGATGCCCGCATTCTCCACC	0	2	0	0
tsrna-13942	TCGATGCCCGCATCCTCCACCA	2	4	1	0
tsrna-13941	TCGATGCCCGCATCCTCCACC	0	1	0	0
tsrna-13936	TCGATCCTCGCTGGGGCCTCCA	10	9	1	3
tsrna-13935	TCGATCCTCGCTGGGGCCTCC	1	0	0	1
tsrna-13934	TCGATCCTCACCTGGAGCACCA	0	3	0	1
tsrna-13932	TCGATCCTCACACGGGGCACCA	0	3	0	0
tsrna-13930	TCGATCCCGGGTTTCGGCACCA	40	104	14	8
tsrna-13929	TCGATCCCGGGTTTCGGCACC	5	4	3	1
tsrna-13924	TCGATCCCGGGCGGAAACACCA	18	37	8	7
tsrna-13923	TCGATCCCGGGCGGAAACACC	4	6	1	0
tsrna-13921	TCGATCCCCGTACTGGCCACCA	4	2	5	0
tsrna-13919	TCGATCCCCGTACGGGCCACCA	12	73	6	7
tsrna-13918	TCGATCCCCGTACGGGCCACC	2	1	0	0
tsrna-13917	TCGATCCCCGGCATCTCCACCA	238	389	135	81
tsrna-13916	TCGATCCCCGGCATCTCCACC	6	24	2	3
tsrna-13915	TCGATCCCCGGCATCTCCAC	0	1	1	0
tsrna-13911	TCGATCCCCGGCACCTCCACCA	186	286	95	42
tsrna-13910	TCGATCCCCGGCACCTCCACC	9	20	6	1
tsrna-13909	TCGATCCCCGACACCTCCACCA	20	24	3	3
tsrna-13908	TCGATCCCCAGTACCTCCACCA	41	63	19	11
tsrna-13907	TCGATCCCCAGTACCTCCACC	2	5	3	2
tsrna-13904	TCGATCCCCAGCATCTCCACCA	11	15	7	2
tsrna-13902	TCGATCCCCAGCATCTCCAC	0	0	0	0
tsrna-13900	TCGATCCCACCCAGGGACGCCA	2	4	1	0
tsrna-13899	TCGATAGCTCAGTTGGTAGAGCGGAGGACT	1	0	0	3

tsrna-13898	TCGATAGCTCAGTTGGTAGAGC	1	0	1	0
tsrna-13897	TCGATAGCTCAGTTGGTAGAG	0	0	1	2
tsrna-13896	TCGATAGCTCAGTTGGTAGA	1	1	0	1
tsrna-13895	TCGATAGCTCAGTTGGTAG	0	0	2	0
tsrna-13894	TCGATAGCTCAGTTGG	0	0	2	0
tsrna-13893	TCGATAGCTCAGCTGGTAGAGCGGAGGACT	0	0	0	0
tsrna-13892	TCGATAGCTCAGCTGGTAGAGCGG	1	0	0	0
tsrna-13891	TCGATAGCTCAGCTGGTAGAGC	1	1	0	0
tsrna-13890	TCGATAGCTCAGCTGGTAGA	0	0	1	2
tsrna-13889	TCGATAGCTCAGCTGGTAG	1	0	1	0
tsrna-13888	TCGAGTCTCGGTGGAACCTCCA	61	119	19	24
tsrna-13887	TCGAGTCTCGGTGGAACCTCC	7	3	0	1
tsrna-13886	TCGAGTCTCGGTGGAACCTC	0	1	0	0
tsrna-13885	TCGAGTCCTGCCGCGGTCGCCA	3	6	1	1
tsrna-13884	TCGAGTCCTGCCGCGGTCGCC	0	0	1	0
tsrna-13881	TCGAGTCCCTTCGTGGTCGCCA	1	0	0	0
tsrna-13880	TCGAGTCCCTTCGTGGTCGCC	0	1	0	0
tsrna-13879	TCGAGTCCCGGCGGAGTCGCCA	0	3	0	0
tsrna-13873	TCGAGTCCCATCTGGGTCGCCA	3	8	0	0
tsrna-13872	TCGAGTCCCATCTGGGTCGCC	1	0	0	0
tsrna-13870	TCGAGTCCCATCTGGGGTGCCA	0	4	0	0
tsrna-13868	TCGAGTCCCATCTGGGGTGC	0	1	0	0
tsrna-13867	TCGAGTCCCACCTGGGGTGCCA	1	5	1	0
tsrna-13866	TCGAGTCCCACCTGGGGTACCA	0	6	0	0
tsrna-13864	TCGAGTCCCACCCGGGGTACCA	0	1	0	0
tsrna-13863	TCGAGTCCCACCAGAGTCGCCA	0	2	0	0
tsrna-13862	TCGAGTCCCACCAGAGTCGCC	0	0	0	0
tsrna-13860	TCGAGCCTCAGAGAGGGCACCA	0	1	0	0
tsrna-13859	TCGAGCCTCACCTGGAGCACCA	0	4	2	0
tsrna-13858	TCGAGCCTCACCTGGAGCACC	0	1	0	0
tsrna-13854	TCGAGCCCCAGTGGAACCACCA	38	28	28	17
tsrna-13853	TCGAGCCCCAGTGGAACCACC	2	6	1	2
tsrna-13852	TCGAGCCCCAGTGGAACCAC	0	0	0	0
tsrna-13850	TCGAGCCCCAGTGGAACC	0	2	0	0
tsrna-13847	TCGAGCCCCACGTTGGGCGCCA	9	193	8	4
tsrna-13846	TCGAGCCCACCCAGGGACGCCA	5	104	0	0
tsrna-13845	TCGAGCCCACCCAGGGACGCC	0	6	0	0
tsrna-13842	TCGACTCCTGGCTGGCTCGCCA	30	118	20	3
tsrna-13841	TCGACTCCTGGCTGGCTCGCC	0	3	0	0
tsrna-13840	TCGACTCCTGGCTGGCTCGC	0	2	0	0
tsrna-13836	TCGACTCCCGGTGTGGGAACCA	54	260	26	19
tsrna-13835	TCGACTCCCGGTGTGGGAACC	1	11	1	0
tsrna-13832	TCGACTCCCGGTGTGGGA	0	1	0	0
tsrna-13829	TCGACTCCCGGTATGGGAACCA	9	80	1	6
tsrna-13827	TCGACTCCCAGCGGGGCCTCCA	1	0	0	0
tsrna-13826	TCGACTCATTAAATTATGATAATCATATTT	0	0	3	0
tsrna-13825	TCGACTCATTAAATTATGATAATCAT	1	0	0	0
tsrna-13824	TCGACCCCCGGCTCCTCCACCA	18	23	12	2
tsrna-13822	TCGAATCTCGGTGGGACCTCCA	59	133	24	25
tsrna-13821	TCGAATCCTGTTCGTGACGCCA	15	6	3	1
tsrna-13820	TCGAATCCTGTTCGTGACGCC	2	3	1	0
tsrna-13819	TCGAATCCTGTCGGCTACGCCA	4	17	6	8
tsrna-13818	TCGAATCCTGCTCACAGCGCCA	8	26	3	2
tsrna-13817	TCGAATCCTGCTCACAGCGCC	1	3	0	0
tsrna-13814	TCGAATCCTGCCGACTACGCCA	103	244	27	15
tsrna-13813	TCGAATCCTGCCGACTACGCC	9	13	1	0
tsrna-13812	TCGAATCCTGCCGACTACGC	0	2	0	0
tsrna-13811	TCGAATCCGGCTCGGAGGACCA	2	0	1	0
tsrna-13808	TCGAATCCGGCTCGAAGGACCA	21	57	9	4
tsrna-13807	TCGAATCCGGCTCGAAGGACC	1	5	0	1
tsrna-13802	TCGAATCCGAGTCACGGCACCA	1	3	0	0
tsrna-13801	TCGAATCCGAGTCACGGCACC	0	0	0	0
tsrna-13797	TCGAATCCCTTCGTGGTTGCCA	0	7	0	0
tsrna-13795	TCGAATCCCTCCGTGGTTACCA	1	4	2	0

tsrna-13794	TCGAATCCCATCCTCGTCGCCA	8	13	2	1
tsrna-13793	TCGAATCCCATCCTCGTCGCC	1	1	0	0
tsrna-13789	TCGAATCCCAGTAGAGCCTCCA	11	18	10	10
tsrna-13788	TCGAATCCCAGTAGAGCCTCC	3	7	0	0
tsrna-13787	TCGAATCCCAGCGGTGCCTCCA	24	49	12	4
tsrna-13786	TCGAATCCCAGCGGTGCCTCC	0	1	0	0
tsrna-13782	TCGAATCCCAGCGGGGCCTCCA	0	3	0	1
tsrna-13781	TCGAATCCCAGCGGGGCCTCC	0	2	0	0
tsrna-13779	TCGAATCCCAGCGAGGCCTCCA	0	0	0	0
tsrna-13778	TCGAATCCCACTTTTGACACCA	0	0	0	1
tsrna-13777	TCGAATCCCACTTCTGACACCA	9	21	8	9
tsrna-13776	TCGAATCCCACTTCTGACACC	1	2	1	0
tsrna-13775	TCGAATCCCACTTCTGACAC	0	0	0	0
tsrna-13772	TCGAATCCCACTCCTGACACCA	114	110	64	25
tsrna-13771	TCGAATCCCACTCCTGACACC	3	8	1	4
tsrna-13770	TCGAATCCCACTCCTGACAC	0	0	1	0
tsrna-13769	TCGAATCCCACTCCTGACA	1	0	0	0
tsrna-13767	TCGAATCCCACCTTCGTCGCCA	0	4	0	0
tsrna-13766	TCGAATCCCACCTTCGTCGCC	2	1	0	0
tsrna-13764	TCGAATCCCACCGCTGCCACCA	15	36	4	2
tsrna-13763	TCGAATCCCACCGCTGCCACC	1	2	0	0
tsrna-13759	TCGAATCCCACCCTCGTCGCCA	3	23	3	5
tsrna-13758	TCGAATCCCACCCTCGTCGCC	0	1	0	1
tsrna-13757	TCGAATCCCACCAGAGTCGCCA	4	4	1	0
tsrna-13756	TCGAATCCCACCAGAGTCGCC	2	0	0	0
tsrna-13754	TCGAATCCCACCACTGCCACCA	1	21	0	2
tsrna-13753	TCGAATCCCACCACTGCCACC	1	1	0	0
tsrna-13752	TCGAATCACGTCGGGGTCACCA	4	9	5	2
tsrna-13751	TCGAATCACGTCGGGGTCACC	0	1	1	0
tsrna-13745	TCGAAGAAGCAGCTTCAAACCTGCCGGGGCTT	0	0	0	0
tsrna-13744	TCGAAGAAGCAGCTTCAAACCTGCCGGGGCT	2	0	0	0
tsrna-13742	TCGAACCTCAGAGGGGGCACCA	0	1	0	0
tsrna-13738	TCGAACCCTGCTCGCTGCGCCA	8	37	4	1
tsrna-13737	TCGAACCCTGCTCGCTGCGCC	1	3	0	0
tsrna-13734	TCGAACCCCGTCCGTGCCTCCA	8	8	2	1
tsrna-13733	TCGAACCCCGTCCGTGCCTCC	0	1	1	0
tsrna-13729	TCGAACCCCACTTCTGGTACCA	2	11	0	0
tsrna-13727	TCGAACCCCACTCCTGGTACC	0	1	0	0
tsrna-13724	TCGAAATCCAATGGGGTTTCCCC	9	386	2	2
tsrna-13723	TCGAAACCGGGCGGAAACACCA	298	716	125	39
tsrna-13722	TCGAAACCGGGCGGAAACACC	68	183	24	3
tsrna-13721	TCGAAACCGGGCGGAAACAC	0	1	1	0
tsrna-13720	TCGAAACCGGGCGGAAACA	0	2	0	0
tsrna-13716	TCGAAACCGGGCAGAAGCACCA	33	127	16	5
tsrna-13715	TCGAAACCGGGCAGAAGCACC	1	9	1	0
tsrna-13710	TCGAAACCATCCTCTGCTACCA	12	14	13	11
tsrna-13709	TCGAAACCATCCTCTGCTACC	5	6	2	1
tsrna-13707	TCGAAACCATCCTCTGCTA	0	0	0	0
tsrna-13706	TCGAAACCATCCTCTGCT	0	1	0	0
tsrna-13702	TCCTTGTAGTATAAACTAATACACCAG	1	0	0	0
tsrna-13701	TCCTTGGTGGTCTAGTGGCTAGGATTCGG	3	7	0	5
tsrna-13700	TCCTTGGTGGTCTAGTGGC	0	1	2	0
tsrna-13699	TCCTTGGTGGTCTAGTGG	0	0	0	0
tsrna-13696	TCCTTAGGTCGCTGGTTCGATTCCGGCTCG	1	0	4	0
tsrna-13695	TCCTTAGGTCGCTGGTTCGATT	1	1	0	0
tsrna-13693	TCCTTAGGTCGCTGGTTCGAATCCGGCTCG	0	1	0	0
tsrna-13691	TCCTTAGGTCGCTGGTTCG	0	0	0	0
tsrna-13690	TCCTTAGGTCGCTGGTTC	0	1	0	0
tsrna-13689	TCCTTAGGTCGCTGGTT	0	0	1	0
tsrna-13688	TCCTTAGGTCGCTGGT	0	0	1	0
tsrna-13687	TCCTGTTCGTGACGCCA	1	3	1	0
tsrna-13685	TCCTGTCGGCTACGCCA	1	0	0	0
tsrna-13684	TCCTGGTTTTCACCCAGGTGGCCCG	5	1	1	0
tsrna-13683	TCCTGGGTTCGATCCCCAGTACCTCCACC	2	4	1	0

tsrna-13682	TCCTGGGTTCGAGCCCCAGTGGAACCACC	1	3	1	1
tsrna-13681	TCCTGGGTTCGAGCCCCAGTGGAACCAC	0	0	0	0
tsrna-13680	TCCTGGGTTCGAGCCCCAGTGGAACCA	2	2	0	1
tsrna-13679	TCCTGGGTTCGAATCCCAGCGGTGCCTCC	2	0	0	0
tsrna-13678	TCCTGGGTTCAATCCCCAATACCTCCACC	0	0	0	0
tsrna-13677	TCCTGGCTGGCTCGCCA	4	39	0	0
tsrna-13675	TCCTGCTCACAGCGCCA	2	5	1	0
tsrna-13673	TCCTGCCGCGGTCGCCA	0	1	0	0
tsrna-13671	TCCTGCCGACTACGCCA	18	22	1	0
tsrna-13670	TCCTGCCGACTACGCC	1	3	0	0
tsrna-13665	TCCTGAGTTCGAACCTCAGAGGGGGCACC	0	1	0	0
tsrna-13659	TCCTCTTCTTAACACCA	0	1	1	0
tsrna-13657	TCCTCGTTAGTATGGTGG	1	0	0	0
tsrna-13656	TCCTCGTTAGTATAGTGGTTAGTATCCCCGCCTGTC	8	3	4	3
tsrna-13655	TCCTCGTTAGTATAGTGGTTAGTATCCCCGCCTGT	8	3	2	3
tsrna-13654	TCCTCGTTAGTATAGTGGTTAGTATCCCCGCCTG	2	2	4	5
tsrna-13653	TCCTCGTTAGTATAGTGGTTAGTATCCCCGCCT	1	3	2	1
tsrna-13652	TCCTCGTTAGTATAGTGGTTAGTATCCCCGCC	1	0	2	3
tsrna-13651	TCCTCGTTAGTATAGTGGTTAGTATCCCCG	0	4	3	1
tsrna-13650	TCCTCGTTAGTATAGTGGTTAGTATCCCC	1	1	0	0
tsrna-13649	TCCTCGTTAGTATAGTGGTTAGTATCCC	2	0	0	2
tsrna-13648	TCCTCGTTAGTATAGTGGTTAGTATCC	0	0	1	2
tsrna-13647	TCCTCGTTAGTATAGTGGTTAGTATC	0	3	1	3
tsrna-13646	TCCTCGTTAGTATAGTGGTTAGT	2	0	3	1
tsrna-13645	TCCTCGTTAGTATAGTGGTTAG	0	0	0	2
tsrna-13644	TCCTCGTTAGTATAGTGGTT	1	0	1	0
tsrna-13643	TCCTCGTTAGTATAGTGGTGAGTATCCCCGCCTGTC	33	28	22	16
tsrna-13642	TCCTCGTTAGTATAGTGGTGAGTATCCCCGCCTGT	16	21	19	14
tsrna-13641	TCCTCGTTAGTATAGTGGTGAGTATCCCCGCCTG	14	17	11	8
tsrna-13640	TCCTCGTTAGTATAGTGGTGAGTATCCCCGCCT	18	16	9	13
tsrna-13639	TCCTCGTTAGTATAGTGGTGAGTATCCCCGCC	20	13	4	12
tsrna-13638	TCCTCGTTAGTATAGTGGTGAGTATCCCCGC	9	11	4	8
tsrna-13637	TCCTCGTTAGTATAGTGGTGAGTATCCCCG	5	18	2	4
tsrna-13636	TCCTCGTTAGTATAGTGGTGAGTATCCCC	7	10	2	4
tsrna-13635	TCCTCGTTAGTATAGTGGTGAGTATCCC	5	8	1	4
tsrna-13634	TCCTCGTTAGTATAGTGGTGAGTATCC	3	12	2	5
tsrna-13633	TCCTCGTTAGTATAGTGGTGAGTATC	2	4	0	6
tsrna-13632	TCCTCGTTAGTATAGTGGTGAGTAT	7	7	2	3
tsrna-13631	TCCTCGTTAGTATAGTGGTGAGTA	6	7	1	2
tsrna-13630	TCCTCGTTAGTATAGTGGTGAGT	7	12	3	5
tsrna-13629	TCCTCGTTAGTATAGTGGTGAG	2	1	2	4
tsrna-13628	TCCTCGTTAGTATAGTGGTGA	1	0	1	2
tsrna-13627	TCCTCGTTAGTATAGTGGTG	0	0	1	0
tsrna-13626	TCCTCGTTAGTATAGTGGT	0	0	1	0
tsrna-13625	TCCTCGTTAGTATAGTGG	1	0	0	0
tsrna-13624	TCCTCGTTAGTATAGTG	0	0	0	2
tsrna-13623	TCCTCGTTAGTATAGT	1	0	0	0
tsrna-13622	TCCTCGCTGGGGCCTCCA	8	6	4	3
tsrna-13621	TCCTCACCTGGAGCACCA	1	1	0	0
tsrna-13619	TCCTCACACGGGGCACCA	0	2	0	0
tsrna-13612	TCCTAAGCCAGGGATTGTGGGTTCGAGTCCCATCTGGGGTGC	172	486	103	44
tsrna-13611	TCCTAAGCCAGGGATTGTGGGTTCGAGTCCCATCTGGGGTGC	152	486	111	51
tsrna-13610	TCCTAAGCCAGGGATTGTGGGTTCGAGTCCCACCCGGGGTAC	143	477	95	62
tsrna-13609	TCCTAAGCCAGGGATTGTGGGTTCGAGTCC	151	454	108	51
tsrna-13608	TCCTAAGCCAGGGATTGTGGGTTCG	189	461	114	56
tsrna-13607	TCCTAAGCCAGGGATTGTGGGTTC	174	481	108	51
tsrna-13606	TCCTAAGCCAGGGATTGTGGGTT	151	464	99	39
tsrna-13605	TCCTAAGCCAGGGATTGTGGGT	146	435	81	43
tsrna-13604	TCCTAAGCCAGGGATTGTGGG	1	6	4	2
tsrna-13603	TCCTAAGCCAGGGATTGTGG	1	1	1	0
tsrna-13602	TCCTAAGCCAGGGATTGTG	0	1	0	1
tsrna-13599	TCCGTGGTGGTCTAGTGGCTAGGATTCGGC	9	22	6	12
tsrna-13598	TCCGTGGTGGTCTAGTGGCTAGGATTCGG	1	4	0	4
tsrna-13597	TCCGTGGTGGTCTAGTGGC	0	0	1	0

tsrna-13593	TCCGTGCGAGAATACCA	1	0	0	0
tsrna-13591	TCCGTAGTGTAGTGGTTATCACGTTCGCCTCAC	2	1	1	1
tsrna-13590	TCCGTAGTGTAGTGGTTATCACGTTCGCCTC	2	0	1	0
tsrna-13589	TCCGTAGTGTAGTGGTTATCACGTTCGCCT	2	1	2	0
tsrna-13588	TCCGTAGTGTAGTGGTTATCACGTTCGCC	2	0	0	1
tsrna-13587	TCCGTAGTGTAGTGGTTATCACGTTCGC	1	3	1	1
tsrna-13586	TCCGTAGTGTAGTGGTTATCACGTTCG	1	0	1	1
tsrna-13585	TCCGTAGTGTAGTGGTTATCACGTTC	3	0	0	0
tsrna-13584	TCCGTAGTGTAGTGGTTATCACGTT	3	1	1	1
tsrna-13583	TCCGTAGTGTAGTGGTTATCACG	4	0	0	1
tsrna-13582	TCCGTAGTGTAGTGGTTATCA	0	0	1	1
tsrna-13581	TCCGTAGTGTAGTGGTTATC	2	1	1	0
tsrna-13578	TCCGTAGTGTAGTGGTCATCACGTTCGCCT	1	0	0	1
tsrna-13577	TCCGTAGTGTAGTGGTCATCACGTTCGCC	1	1	0	0
tsrna-13576	TCCGTAGTGTAGTGGTCATCACGTTC	0	0	1	0
tsrna-13575	TCCGTAGTGTAGTGGTCATCACG	3	0	3	0
tsrna-13574	TCCGTAGTGTAGTGGTCATC	1	0	0	0
tsrna-13571	TCCGTAGTGTAGCGGTTATCACATT	1	0	0	0
tsrna-13570	TCCGGGTTCGAGTCCCGGCGGAGTCGCCA	0	4	0	0
tsrna-13569	TCCGGGTTCGAGTCCCGGCGGAGTCGCC	0	1	0	0
tsrna-13566	TCCGGGTGCCCCCTCCA	101	126	49	12
tsrna-13565	TCCGGGTGCCCCCTCC	0	1	0	0
tsrna-13564	TCCGGGTGCCCCCCCCA	10	13	4	0
tsrna-13563	TCCGGGGTTCAAGTCCCTGTTCGGGCGCC	0	0	0	0
tsrna-13560	TCCGGCTCGAAGGACCA	1	3	0	1
tsrna-13559	TCCGGCTCGAAGGACC	1	3	0	0
tsrna-13558	TCCGGATGGAGGCGTGGGTTCGAATCCCACTTCTG	1	30	4	2
tsrna-13557	TCCGGATGGAGGCGTGGGTTCGAATC	6	34	4	2
tsrna-13556	TCCGGATGGAGGCGTGGGTTCGAA	6	42	2	1
tsrna-13555	TCCGGATGGAGGCGTGGGTTCG	6	35	4	0
tsrna-13554	TCCGGATGGAGGCGTGGGTT	9	37	3	2
tsrna-13553	TCCGGATGGAGGCGTGGGT	3	34	3	1
tsrna-13552	TCCGGATGGAGGCGTGGG	1	10	1	0
tsrna-13551	TCCGGATGGAGGCGTGG	1	3	0	0
tsrna-13550	TCCGGATGGAGGCGTG	2	7	1	1
tsrna-13549	TCCGGATCAGAAGATTGAGGGT	3	8	2	0
tsrna-13547	TCCGGAGCTGGGGATTGTGGGT	0	2	0	0
tsrna-13545	TCCGGAGCTGGGGATTGTGG	0	0	0	0
tsrna-13542	TCCGCGTGGGTTCGAACCCCACTCCTGG	0	1	0	0
tsrna-13534	TCCCTGTTCGGGCGCCA	2	6	6	4
tsrna-13531	TCCCTGTGGTCTAGTGGTTAGGA	202	338	243	291
tsrna-13530	TCCCTGTGGTCTAGTGGTTAGG	184	307	258	165
tsrna-13529	TCCCTGTGGTCTAGTGGTTAG	140	294	232	163
tsrna-13528	TCCCTGTGGTCTAGTGGTTA	153	283	270	148
tsrna-13527	TCCCTGTGGTCTAGTGGTT	67	177	71	29
tsrna-13526	TCCCTGTGGTCTAGTGGT	50	198	55	37
tsrna-13525	TCCCTGTGGTCTAGTGG	48	188	64	44
tsrna-13524	TCCCTGTGGTCTAGTG	57	206	62	50
tsrna-13523	TCCCTGTCCAGGCGCCA	0	0	0	0
tsrna-13521	TCCCTGGTTCGATCCCGGGTTTCGGCACC	6	6	1	1
tsrna-13519	TCCCTGGTTCGATCCCGGGTTTCGGCA	0	1	0	0
tsrna-13518	TCCCTGGTTCGATCCCGGGTTTCGGC	0	0	0	0
tsrna-13516	TCCCTGGTTCGATCCCGGGTT	0	1	0	0
tsrna-13512	TCCCTGGTTCAATCCCGGGTTTCGGCACC	8	5	3	0
tsrna-13511	TCCCTGGTTCAAATCCGGGTGCCCCCTCC	4	5	2	0
tsrna-13510	TCCCTGGTGGTCTAGTGGTTAGGATTCGGCGCTCTCACCGCC	833	327	1235	177
tsrna-13509	TCCCTGGTGGTCTAGTGGTTAGGATTCGGCGCTCTCACCGCC	835	355	1190	148
tsrna-13508	TCCCTGGTGGTCTAGTGGTTAGGATTCGGCGCTCTCACCGCC	828	351	1215	144
tsrna-13507	TCCCTGGTGGTCTAGTGGTTAGGATTCGGCGCTCTCACCGCC	818	343	1116	170
tsrna-13506	TCCCTGGTGGTCTAGTGGTTAGGATTCGGCGCTCTCACCGC	722	292	944	137
tsrna-13505	TCCCTGGTGGTCTAGTGGTTAGGATTCGGCGCTCTCACCG	750	309	947	124
tsrna-13504	TCCCTGGTGGTCTAGTGGTTAGGATTCGGCGCTCTCACC	674	326	831	132
tsrna-13503	TCCCTGGTGGTCTAGTGGTTAGGATTCGGCGCTCTCAC	616	283	689	106
tsrna-13502	TCCCTGGTGGTCTAGTGGTTAGGATTCGGCGCTCTCA	644	279	689	86

tsrna-13501	TCCCTGGTGGTCTAGTGGTTAGGATTCGGCGCTCTC	658	297	700	116
tsrna-13500	TCCCTGGTGGTCTAGTGGTTAGGATTCGGCGCTCT	633	267	652	110
tsrna-13499	TCCCTGGTGGTCTAGTGGTTAGGATTCGGCGCTC	161	116	111	54
tsrna-13498	TCCCTGGTGGTCTAGTGGTTAGGATTCGGCGCT	60	82	42	32
tsrna-13497	TCCCTGGTGGTCTAGTGGTTAGGATTCGGCGC	57	44	32	32
tsrna-13496	TCCCTGGTGGTCTAGTGGTTAGGATTCGGCG	35	54	30	32
tsrna-13495	TCCCTGGTGGTCTAGTGGTTAGGATTCGGC	35	43	35	27
tsrna-13494	TCCCTGGTGGTCTAGTGGTTAGGATTCGG	22	36	23	19
tsrna-13493	TCCCTGGTGGTCTAGTGGTTAGGATTCG	21	37	32	25
tsrna-13492	TCCCTGGTGGTCTAGTGGTTAGGATTC	22	41	22	23
tsrna-13491	TCCCTGGTGGTCTAGTGGTTAGGATT	23	37	21	18
tsrna-13490	TCCCTGGTGGTCTAGTGGTTAGGAT	27	29	24	15
tsrna-13489	TCCCTGGTGGTCTAGTGGTTAGGA	19	33	23	14
tsrna-13488	TCCCTGGTGGTCTAGTGGTTAGG	23	41	25	18
tsrna-13487	TCCCTGGTGGTCTAGTGGTTAG	24	36	27	15
tsrna-13486	TCCCTGGTGGTCTAGTGGTTA	23	27	30	10
tsrna-13485	TCCCTGGTGGTCTAGTGGTT	2	14	2	6
tsrna-13484	TCCCTGGTGGTCTAGTGGT	8	18	3	3
tsrna-13483	TCCCTGGTGGTCTAGTGGCTAGGATTCGGCGCTTTCACC	72	93	112	91
tsrna-13482	TCCCTGGTGGTCTAGTGGCTAGGATTCGGCGCTTTC	77	77	81	56
tsrna-13481	TCCCTGGTGGTCTAGTGGCTAGGATTCGGCGCTTT	76	75	73	40
tsrna-13480	TCCCTGGTGGTCTAGTGGCTAGGATTCGGCGCTT	39	66	47	43
tsrna-13479	TCCCTGGTGGTCTAGTGGCTAGGATTCGGCGCT	25	49	13	45
tsrna-13478	TCCCTGGTGGTCTAGTGGCTAGGATTCGGCGC	14	55	10	40
tsrna-13477	TCCCTGGTGGTCTAGTGGCTAGGATTCGGCG	16	51	10	35
tsrna-13476	TCCCTGGTGGTCTAGTGGCTAGGATTCGGC	16	30	6	18
tsrna-13475	TCCCTGGTGGTCTAGTGGCTAGGATTCGG	7	23	4	4
tsrna-13474	TCCCTGGTGGTCTAGTGGCTAGGATTCG	6	10	1	15
tsrna-13473	TCCCTGGTGGTCTAGTGGCTAGGATTC	4	13	3	6
tsrna-13472	TCCCTGGTGGTCTAGTGGCTAGGATT	6	26	5	12
tsrna-13471	TCCCTGGTGGTCTAGTGGCTAGGAT	5	19	2	5
tsrna-13470	TCCCTGGTGGTCTAGTGGCTAGGA	5	17	7	8
tsrna-13469	TCCCTGGTGGTCTAGTGGCTAGG	3	23	2	2
tsrna-13468	TCCCTGGTGGTCTAGTGGCTAG	4	17	2	4
tsrna-13467	TCCCTGGTGGTCTAGTGGCTA	3	15	2	4
tsrna-13466	TCCCTGGTGGTCTAGTGGCT	1	23	3	1
tsrna-13465	TCCCTGGTGGTCTAGTGGC	1	20	0	1
tsrna-13464	TCCCTGGTGGTCTAGTGG	2	17	3	2
tsrna-13463	TCCCTGGTGGTCTAGTG	4	21	7	3
tsrna-13462	TCCCTGGTGGTCTAGT	4	12	2	2
tsrna-13461	TCCCTGGTGGTCTAATGGTTAGG	8	29	7	2
tsrna-13460	TCCCTGGTGGTCTAATGGTTAG	2	32	6	5
tsrna-13459	TCCCTGGTCTAGTGGTTAGGATT	4	7	5	5
tsrna-13458	TCCCTGGTCTAGTGGTTAGGAT	0	1	0	1
tsrna-13457	TCCCTGGTCTAGTGGTTAGGA	0	2	0	0
tsrna-13456	TCCCTGGTCTAGTGGTTAGG	0	0	0	0
tsrna-13455	TCCCTGGTCTAGTGGTTAG	0	0	2	0
tsrna-13454	TCCCTGGTCTAGTGGTTA	0	0	0	0
tsrna-13453	TCCCTGGTAGTCTAGTGGC	0	1	0	0
tsrna-13452	TCCCTGGTAGTCTAGTGG	0	0	0	0
tsrna-13451	TCCCTGGTAGTCTAGTG	0	3	0	0
tsrna-13450	TCCCTGGTAGTCTAGT	0	0	0	0
tsrna-13449	TCCCTGGCACCTCCACCA	0	0	0	0
tsrna-13448	TCCCTCCGTGGTTACCA	0	1	0	0
tsrna-13447	TCCCGGTGTGGGAACCA	0	2	0	0
tsrna-13445	TCCCGGTCAGGGAACCA	1	1	0	0
tsrna-13442	TCCCGGGTTTCGGCACCA	38	109	13	9
tsrna-13441	TCCCGGGTTTCGGCACC	2	6	2	0
tsrna-13440	TCCCGGGTTTCGGCAC	0	1	0	0
tsrna-13439	TCCCGGGTTCGATCCCCGGCATCTCCACC	6	15	4	3
tsrna-13438	TCCCGGGTTCGATCCCCGGCACCTCCACC	8	22	5	2
tsrna-13436	TCCCGGGTTCGATCCCCAGCATCTCCACC	1	3	0	2
tsrna-13432	TCCCGGGTTCAATCCCCGGCATCTCCACC	2	6	1	1
tsrna-13431	TCCCGGGTTCAATCCCCGGCACCTCCACC	10	20	3	1

tsrna-13430	TCCCGGGTTCAAATCCCGGACGAGCCCCC	6	11	1	0
tsrna-13429	TCCCGGGTTCAAATCCCGGACGAG	0	0	0	0
tsrna-13428	TCCCGGGCGGCGCACCA	3	2	0	1
tsrna-13427	TCCCGGGCGGCGCACC	0	2	1	0
tsrna-13426	TCCCGGGCGGAAACACCA	16	25	8	4
tsrna-13425	TCCCGGGCGGAAACACC	4	7	0	1
tsrna-13424	TCCCGGCGGAGTCGCCA	0	0	0	0
tsrna-13423	TCCCGGCGGAGTCGCC	0	0	0	0
tsrna-13422	TCCCGGCCCATGCACCA	1	2	2	1
tsrna-13420	TCCCGGCCAATGCACCA	1	0	0	2
tsrna-13419	TCCCGGCCAATGCACC	1	1	1	0
tsrna-13416	TCCCGGACGAGCCCCCA	24	43	11	16
tsrna-13415	TCCCGGACGAGCCCCC	1	1	0	0
tsrna-13412	TCCCCGTACTGGCCACCA	1	8	2	0
tsrna-13411	TCCCCGTACTGGCCACC	1	0	0	0
tsrna-13410	TCCCCGTACGGGCCACCA	10	44	4	3
tsrna-13409	TCCCCGTACGGGCCACC	0	2	0	0
tsrna-13408	TCCCCGGTTCGATCCCGGGCGGAAACACC	3	5	2	0
tsrna-13407	TCCCCGGTTCGATCCCGGGCGGA	0	0	1	0
tsrna-13405	TCCCCGGTTCGAAACCGGGCGGAAACACC	60	194	30	2
tsrna-13401	TCCCCGGTTCAAATCCGGGTGCCCCCTCC	5	2	1	2
tsrna-13400	TCCCCGGCATCTCCACCA	100	125	55	32
tsrna-13399	TCCCCGGCATCTCCACC	3	2	3	0
tsrna-13398	TCCCCGGCATCTCCAC	0	1	0	0
tsrna-13397	TCCCCGGCACCTCCACCA	138	238	78	47
tsrna-13396	TCCCCGGCACCTCCACC	9	9	4	0
tsrna-13395	TCCCCGGCACCTCCAC	0	0	1	0
tsrna-13394	TCCCCGCGCAGGTTCGAATCCTGCCGACT	0	2	0	1
tsrna-13393	TCCCCGCGCAGGTTCGAATCCTG	0	3	0	0
tsrna-13392	TCCCCGCGCAGGTTCGAATCCT	1	2	0	0
tsrna-13389	TCCCCGCCTGTCACGCGGGAGACCGGGGTTCGATTC	15	63	6	8
tsrna-13388	TCCCCGCCTGTCACGCGGGAGACCGGGGTT	11	56	5	2
tsrna-13387	TCCCCGCCTGTCACGCGGGAGACCGG	13	44	7	2
tsrna-13386	TCCCCGCCTGTCACGCGGGAGACCG	10	46	11	5
tsrna-13382	TCCCCGACACCTCCACCA	7	1	2	1
tsrna-13381	TCCCCGACACCTCCACC	1	0	0	0
tsrna-13378	TCCCCAGTACCTCCACCA	1	3	1	1
tsrna-13377	TCCCCAGTACCTCCACC	0	1	0	0
tsrna-13375	TCCCCAGCATCTCCACCA	1	3	1	0
tsrna-13372	TCCCCAGCACTTCCACCA	0	0	0	0
tsrna-13371	TCCCCAGCACCTCCACCA	0	1	0	0
tsrna-13362	TCCCATGGTGTAATGGTTAGCACTCTGGACT	1	1	1	0
tsrna-13361	TCCCATGGTGTAATGGTTAGCACTCTGGAC	0	2	0	3
tsrna-13360	TCCCATGGTGTAATGGTTAGCACTCTGG	0	0	0	1
tsrna-13359	TCCCATGGTGTAATGGTTAGCACTCTG	1	0	0	0
tsrna-13358	TCCCATCTGGGTCGCCA	2	2	1	0
tsrna-13357	TCCCATCTGGGGTGCCA	0	1	0	0
tsrna-13356	TCCCATCCTCGTCGCCA	4	0	4	0
tsrna-13355	TCCCATCCTCGTCGCC	1	1	0	0
tsrna-13354	TCCCATATGGTCTAGCGGTTAGGATTCCTGGTTTTCACCCA	44	19	87	9
tsrna-13353	TCCCATATGGTCTAGCGGTTAGGATTCCTGGTTTTCACCC	42	11	78	9
tsrna-13352	TCCCATATGGTCTAGCGGTTAGGATTCCTGGTTTTCACC	34	17	65	10
tsrna-13351	TCCCATATGGTCTAGCGGTTAGGATTCCTGGTTTTCA	37	6	65	5
tsrna-13350	TCCCATATGGTCTAGCGGTTAGGATTCCTGGTTTTC	27	5	69	6
tsrna-13349	TCCCATATGGTCTAGCGGTTAGGATTCCTGGTTTT	26	9	47	3
tsrna-13348	TCCCATATGGTCTAGCGGTTAGGATTCCTGGTTT	24	8	46	3
tsrna-13347	TCCCATATGGTCTAGCGGTTAGGATTCCTGGTT	2	4	7	2
tsrna-13346	TCCCATATGGTCTAGCGGTTAGGATTCCTGGT	7	11	6	4
tsrna-13345	TCCCATATGGTCTAGCGGTTAGGATTCCTGG	4	6	1	1
tsrna-13344	TCCCATATGGTCTAGCGGTTAGGATTCCTG	5	2	0	3
tsrna-13343	TCCCATATGGTCTAGCGGTTAGGATTCCT	2	1	1	1
tsrna-13342	TCCCATATGGTCTAGCGGTTAGGATTCC	1	1	2	1
tsrna-13341	TCCCATATGGTCTAGCGGTTAGGATTC	1	0	1	4
tsrna-13340	TCCCATATGGTCTAGCGGTTAGGATT	2	0	1	3

tsrna-13339	TCCCATATGGTCTAGCGGTTAGGAT	0	0	1	2
tsrna-13338	TCCCATATGGTCTAGCGGTTAGGA	0	0	4	2
tsrna-13337	TCCCATATGGTCTAGCGGTTAGG	1	1	2	3
tsrna-13336	TCCCATATGGTCTAGCGGTTAG	0	1	0	0
tsrna-13335	TCCCATATGGTCTAGCGGTTA	0	0	0	0
tsrna-13334	TCCCATATGGTCTAGCGGTT	0	0	0	0
tsrna-13333	TCCCATATGGTCTAGCGGT	0	0	0	0
tsrna-13332	TCCCATATGGTCTAGCGG	0	0	0	0
tsrna-13331	TCCCATATGGTCTAGCG	0	0	0	0
tsrna-13329	TCCCAGTAGAGCCTCCA	1	2	0	0
tsrna-13328	TCCCAGTAGAGCCTCC	1	0	0	0
tsrna-13327	TCCCAGCGGTGCCTCCA	0	0	1	0
tsrna-13322	TCCCACTTCTGACACCA	0	0	0	2
tsrna-13321	TCCCACTTCTGACACC	1	1	0	0
tsrna-13320	TCCCACTCCTGACACCA	12	11	10	5
tsrna-13319	TCCCACTCCTGACACC	1	0	1	0
tsrna-13318	TCCCACCTTCGTCGCCA	0	1	0	0
tsrna-13315	TCCCACCTGGGGTACCA	0	1	0	0
tsrna-13314	TCCCACCGCTGCTACCA	1	0	0	0
tsrna-13311	TCCCACCCTCGTCGCCA	0	3	0	3
tsrna-13310	TCCCACCCTCGTCGCC	0	1	0	0
tsrna-13309	TCCCACCCGGGGTACCA	1	0	0	0
tsrna-13308	TCCCACCCAGGGACGCCA	4	4	0	2
tsrna-13306	TCCCACCAGAGTCGCCA	0	0	0	0
tsrna-13303	TCCCACATGGTCTAGCGGTTAGGATTCCTGGTTTTCACCCAG(	119	43	294	29
tsrna-13302	TCCCACATGGTCTAGCGGTTAGGATTCCTGGTTTTCACCCAG	109	29	253	20
tsrna-13301	TCCCACATGGTCTAGCGGTTAGGATTCCTGGTTTTCACCCA	109	28	276	29
tsrna-13300	TCCCACATGGTCTAGCGGTTAGGATTCCTGGTTTTCACCC	119	26	233	24
tsrna-13299	TCCCACATGGTCTAGCGGTTAGGATTCCTGGTTTTCACC	121	25	249	22
tsrna-13298	TCCCACATGGTCTAGCGGTTAGGATTCCTGGTTTTCA	125	37	248	32
tsrna-13297	TCCCACATGGTCTAGCGGTTAGGATTCCTGGTTTTC	93	26	237	22
tsrna-13296	TCCCACATGGTCTAGCGGTTAGGATTCCTGGTTTT	109	20	195	22
tsrna-13295	TCCCACATGGTCTAGCGGTTAGGATTCCTGGTTT	98	19	146	15
tsrna-13294	TCCCACATGGTCTAGCGGTTAGGATTCCTGGTT	3	10	5	11
tsrna-13293	TCCCACATGGTCTAGCGGTTAGGATTCCTGGT	4	16	11	15
tsrna-13292	TCCCACATGGTCTAGCGGTTAGGATTCCTGG	10	6	16	7
tsrna-13291	TCCCACATGGTCTAGCGGTTAGGATTCCTG	8	5	10	18
tsrna-13290	TCCCACATGGTCTAGCGGTTAGGATTCCT	5	11	9	16
tsrna-13289	TCCCACATGGTCTAGCGGTTAGGATTCC	5	5	13	12
tsrna-13288	TCCCACATGGTCTAGCGGTTAGGATTC	3	13	9	6
tsrna-13287	TCCCACATGGTCTAGCGGTTAGGATT	6	4	7	18
tsrna-13286	TCCCACATGGTCTAGCGGTTAGGAT	8	8	12	14
tsrna-13285	TCCCACATGGTCTAGCGGTTAGGA	5	7	5	11
tsrna-13284	TCCCACATGGTCTAGCGGTTAGG	3	14	8	14
tsrna-13283	TCCCACATGGTCTAGCGGTTAG	4	6	10	10
tsrna-13282	TCCCACATGGTCTAGCGGTTA	4	7	9	12
tsrna-13281	TCCCACATGGTCTAGCGGTT	0	4	1	9
tsrna-13280	TCCCACATGGTCTAGCGGT	4	2	1	2
tsrna-13279	TCCCACATGGTCTAGCGG	1	5	5	5
tsrna-13278	TCCCACATGGTCTAGCG	3	1	3	5
tsrna-13277	TCCCACATGGTCTAGC	0	3	6	3
tsrna-13276	TCCATTGTGCTTTGCACGCGTGGGTTCGAATCCCATCCTCGT(	15	34	8	0
tsrna-13275	TCCATTGTGCTTTGCACGCGTGGGTTCGAATCCCATCCTCGT(	6	11	2	0
tsrna-13274	TCCATTGTGCTTTGCACGCGTGGGTTCGA	1	11	0	0
tsrna-13272	TCCATTGGTCTTAGGCCCCA	0	0	0	0
tsrna-13271	TCCATTGGTCTTAGGCCCC	0	0	1	0
tsrna-13267	TCCATTGGGGTTTCCCCGCGCAGGTTCGAATCCTGCCGACTA	131	276	40	20
tsrna-13266	TCCATTGGGGTTTCCCCGCGCAGGTTCGAATCCTGCCGACTA	11	16	2	1
tsrna-13265	TCCATTGGGGTTTCCCCGCGCAGGTTCGA	0	1	0	0
tsrna-13264	TCCATTGGGGTTTCCCCGCGCAGGTT	1	0	0	0
tsrna-13263	TCCATTGGGGTTTCCCCGCGCAGGT	0	1	0	0
tsrna-13254	TCCATGGTGTAATGGTTAGCACTCTGGACT	2	3	0	0
tsrna-13253	TCCATGGTGTAATGGTTAGCACTCTGGAC	3	1	0	1
tsrna-13252	TCCATGGTGTAATGGTTAGCACTCTGGA	1	1	1	2

tsrna-13251	TCCATGGTGTAATGGTTAGCACTCTGG	0	1	0	0
tsrna-13247	TCCATGGTGTAATGGTGAGCACTCTG	0	0	0	0
tsrna-13246	TCCATAGTGTAGTGGTTATCACGTCTGCTT	1	0	0	0
tsrna-13245	TCCATAGTGTAGTGGTTATCACGTCTGCT	0	1	2	0
tsrna-13243	TCCATAGTGTAGTGGTTATCACG	0	0	0	0
tsrna-13242	TCCATAGTGTAGCGGTTATCACGTCTG	0	0	1	0
tsrna-13240	TCCAGTCTCTTCGGGGGCGTG	0	1	0	0
tsrna-13238	TCCAGTCTCTTCGGGGG	0	1	0	0
tsrna-13236	TCCAGGTTCGACTCCTGGCTGGCTCGCC	0	1	0	0
tsrna-13235	TCCAGGTTCGACTCCTGGCTGGCTCG	0	0	0	0
tsrna-13231	TCCAGGTGCCCCCTCCA	0	2	0	0
tsrna-13229	TCCAGGGTTCAAGTCCCTGTTCGGGCGCCA	8	17	5	0
tsrna-13228	TCCAGGGTTCAAGTCCCTGTTCGGGCGCC	0	1	0	0
tsrna-13227	TCCAGGGTTCAAGTCCCTGTTCGGGCGC	0	1	0	0
tsrna-13226	TCCAGGGTTCAAGTCCCTGTTCGGGCG	0	0	0	0
tsrna-13222	TCCAGGGTTCAAGTCCCTGTTCAGGCGCC	0	0	1	1
tsrna-13221	TCCAGGGTTCAAGTCCCTGTCCAGGCGCC	0	0	0	0
tsrna-13216	TCCAGCGATCCGAGTTCAAATCTCGGTGG	0	0	0	0
tsrna-13210	TCCAGATCAGAAGGTTGCGTGTT	2	4	0	2
tsrna-13209	TCCAGATCAGAAGGTTGCGTGT	0	4	1	0
tsrna-13204	TCCAGAGGTTCCGGGTTCGAGTCCCGGCAG	0	0	0	1
tsrna-13203	TCCAATTAACTAGTTTTGACAACATTCAAAAAAGAGTACCA	2	0	1	0
tsrna-13195	TCCAATGGGGTCTCCCCGCGCAGGT	1	1	0	0
tsrna-13193	TCCAATGGAGGCGTGGGTTCGAATCCCACTTCTGACACCA	31	91	19	10
tsrna-13192	TCCAATGGAGGCGTGGGTTCGAATCCCACTTCTG	12	28	7	1
tsrna-13191	TCCAATGGAGGCGTGGGTTCGAA	14	24	7	1
tsrna-13190	TCCAATGGAGGCGTGGGTTCG	18	26	9	1
tsrna-13189	TCCAATGGAGGCGTGGGTTC	10	20	7	1
tsrna-13188	TCCAATGGAGGCGTGGGTT	13	19	10	2
tsrna-13187	TCCAATGGAGGCGTGGGT	6	17	6	0
tsrna-13186	TCCAATGGAGGCGTGGG	0	2	0	0
tsrna-13185	TCCAATGGACATATGTCCGCGTGG	2	2	2	0
tsrna-13181	TCATTTCGATGGCGTGGGT	3	2	3	0
tsrna-13180	TCATTTCGATGGCGTGGG	1	1	0	0
tsrna-13179	TCATTTCGATGGCGTGG	0	2	0	0
tsrna-13178	TCATTTCGATGGCGTG	0	0	1	0
tsrna-13177	TCATTGGTCGTGGTTGTAGTCCGTGCGAGAATACCA	114	131	140	49
tsrna-13176	TCATTGGTCGTGGTTGTAGTCCGTGCGAGAATACC	10	25	20	12
tsrna-13175	TCATTGGTCGTGGTTGTAGTCCGTGCGAGAATA	9	17	15	9
tsrna-13174	TCATTGGTCGTGGTTGTAGTCCGTGCGAGAAT	3	11	7	10
tsrna-13173	TCATTGGTCGTGGTTGTAGTCCGTGCGAGAA	6	11	9	10
tsrna-13172	TCATTGGTCGTGGTTGTAGTCCGTGCGAGA	4	14	6	9
tsrna-13171	TCATTGGTCGTGGTTGTAGTCCGTGCGAG	4	6	3	9
tsrna-13170	TCATTGGTCGTGGTTGTAGTCCGTGCGA	0	6	2	3
tsrna-13169	TCATTGGTCGTGGTTGTAGTCCGTGCG	4	4	3	3
tsrna-13168	TCATTGGTCGTGGTTGTAGTCCGTGC	2	0	0	1
tsrna-13167	TCATTGGTCGTGGTTGTAGTCCGTG	1	3	1	2
tsrna-13166	TCATTGGTCGTGGTTGTAGTCCGT	0	0	1	0
tsrna-13165	TCATTGGTCGTGGTTGTAGTCCG	1	2	0	0
tsrna-13164	TCATTGGTCGTGGTTGTAGTCC	0	0	1	1
tsrna-13163	TCATTGGTCGTGGTTGTAGTC	0	2	0	0
tsrna-13162	TCATTGGTCGTGGTTGTAGT	0	0	0	0
tsrna-13160	TCATTGGTCGTGGTTGTA	0	0	1	0
tsrna-13157	TCATGGAGGCCATGGGGTTGGCTTGA	0	0	0	0
tsrna-13156	TCATGGAGGCCATGGGGTTGG	0	0	0	0
tsrna-13155	TCATGGAGGCCATGGGGTT	0	1	0	0
tsrna-13154	TCATGCCCCCATGTCTAACAACATGGCTTTCTCACCA	11	8	12	0
tsrna-13153	TCATGCCCCCATGTCTAACAACATGGCTTTCTCACC	2	2	5	0
tsrna-13152	TCATGCCCCCATGTCTAACAACATGGCTTT	0	0	1	0
tsrna-13151	TCATGCCCCCATGTCTAACAACATGGCT	0	1	0	0
tsrna-13149	TCATGCCCCCATGTCTAACAACATG	0	1	0	0
tsrna-13148	TCATGCCCCCATGTCTAACAACA	0	1	0	0
tsrna-13144	TCATGCAAGATTCCCATTCTTGCGACCCGG	1	0	1	0
tsrna-13142	TCATATCATTGGTCGTGGTTGTAGTCCGTGCGAGAATACCA	169	135	184	48

tsrna-13141	TCATATCATTGGTCGTGGTTGTAGTCCGTGCGAGAATACC	18	24	24	20
tsrna-13140	TCATATCATTGGTCGTGGTTGTAGTCCGTGCGAGAATA	20	13	11	9
tsrna-13139	TCATATCATTGGTCGTGGTTGTAGTCCGTGCGAGAAT	6	13	6	14
tsrna-13138	TCATATCATTGGTCGTGGTTGTAGTCCGTGCGAGAA	3	10	11	7
tsrna-13137	TCATATCATTGGTCGTGGTTGTAGTCCGTGCGAGA	6	15	15	11
tsrna-13136	TCATATCATTGGTCGTGGTTGTAGTCCGTG	2	6	2	1
tsrna-13135	TCATATCATTGGTCGTGGTTGTAGTCCGT	3	0	1	1
tsrna-13134	TCATATCATTGGTCGTGGTTGTAGTCCG	1	2	2	1
tsrna-13133	TCATATCATTGGTCGTGGTTGTAGTCC	1	5	2	0
tsrna-13132	TCATATCATTGGTCGTGGTTGTAGTC	1	1	2	0
tsrna-13131	TCATATCATTGGTCGTGGTTGTAGT	1	0	0	0
tsrna-13130	TCATATCATTGGTCGTGGTTGTAG	0	0	0	0
tsrna-13129	TCATATCATTGGTCGTGGTTGTA	0	0	0	0
tsrna-13128	TCATATCATTGGTCGTGGTTGT	0	0	0	0
tsrna-13123	TCATAATCTGAAGGTCGTGAGTTCGATCCTCACACGGGGCAC	27	67	17	9
tsrna-13122	TCATAATCTGAAGGTCGTGAGTTCGAGCCTCACACGGGGCAC	32	54	28	7
tsrna-13121	TCATAATCTGAAGGTCGTGAGTTC	22	33	14	5
tsrna-13120	TCATAATCTGAAGGTCGTGAGT	24	43	22	6
tsrna-13119	TCATAATCTGAAGGTCGTGAG	2	7	1	1
tsrna-13118	TCATAATCTGAAGGTCGTG	0	6	0	1
tsrna-13117	TCATAATCTGAAGGTCCTGAGTTCGAACCTCAGAGGGGGCAC	116	298	88	57
tsrna-13116	TCATAATCTGAAGGTCCTGAGT	95	276	91	50
tsrna-13115	TCATAATCTGAAGGTCCTGAG	7	29	2	2
tsrna-13114	TCATAATCTGAAGGTCCTG	4	18	2	3
tsrna-13113	TCATAACTTTGTCAAAGTTAAATT	0	3	0	0
tsrna-13112	TCAGTTGGTTAGAGCGTGGTGC	0	0	0	3
tsrna-13110	TCAGTTGGTAGAGCATCAG	2	0	0	0
tsrna-13108	TCAGTTGATGCAGAGTGGGGTTTTGCAGTC	1	1	0	0
tsrna-13107	TCAGTTGATGCAGAGTGGGGTTTT	1	0	0	1
tsrna-13106	TCAGTTGATGCAGAGTGGGGTT	0	0	0	0
tsrna-13105	TCAGTGGTCTAGTGGTTAGGATTC	1	0	1	0
tsrna-13102	TCAGTGGTAGAGCATTTGACTGCA	2	6	2	0
tsrna-13101	TCAGTGGTAGAGCATTTGACTGC	4	3	0	1
tsrna-13100	TCAGTGGTAGAGCATTTGACTG	0	3	0	0
tsrna-13099	TCAGTGGTAGAGCATTTGACT	2	2	1	0
tsrna-13098	TCAGTGGTAGAGCATTTGAC	1	0	0	0
tsrna-13097	TCAGTGGTAGAGCATTTGA	2	0	0	0
tsrna-13096	TCAGTGGTAGAGCATTTG	2	2	1	0
tsrna-13095	TCAGTGGTAGAATTTTC	1	0	0	0
tsrna-13088	TCAGTGGTAGAATTCTCGCCTTT	4	7	4	1
tsrna-13087	TCAGTGGTAGAATTCTCGCCTT	5	8	2	0
tsrna-13086	TCAGTGGTAGAATTCTCGCCTGCCACG	49	97	21	23
tsrna-13085	TCAGTGGTAGAATTCTCGCCTGCCA	17	40	9	4
tsrna-13084	TCAGTGGTAGAATTCTCGCCTGCC	9	37	10	2
tsrna-13083	TCAGTGGTAGAATTCTCGCCTGC	7	7	4	4
tsrna-13082	TCAGTGGTAGAATTCTCGCCTG	3	4	3	1
tsrna-13081	TCAGTGGTAGAATTCTCGCCTCCC	4	9	1	14
tsrna-13080	TCAGTGGTAGAATTCTCGCCTCC	3	9	6	4
tsrna-13079	TCAGTGGTAGAATTCTCGCCTC	4	8	4	3
tsrna-13078	TCAGTGGTAGAATTCTCGCCT	3	5	1	0
tsrna-13077	TCAGTGGTAGAATTCTCGCC	2	1	1	1
tsrna-13076	TCAGTGGTAGAATTCTCGC	0	0	0	0
tsrna-13072	TCAGTGGGTAGAGCATTTGACTG	0	0	0	0
tsrna-13071	TCAGTGGGTAGAGCATTTGACT	1	2	1	0
tsrna-13070	TCAGTGGGTAGAGCATTTGA	0	0	1	0
tsrna-13069	TCAGTGGGTAGAGCATCAGAC	0	1	0	0
tsrna-13068	TCAGTCTCATAATCTGAAGGTCG	0	4	1	1
tsrna-13067	TCAGTCTCATAATCTGAAGGTC	0	5	1	0
tsrna-13066	TCAGTCGGTAGAGCTATC	2	0	0	0
tsrna-13065	TCAGTCGGTAGAGCATGGGACTCTTAATCC	15	26	18	2
tsrna-13064	TCAGTCGGTAGAGCATGGGACTCTTAATC	15	13	6	2
tsrna-13063	TCAGTCGGTAGAGCATGGGACTCTTAAT	13	18	8	2
tsrna-13062	TCAGTCGGTAGAGCATGGGACTCTTAA	14	23	9	2
tsrna-13061	TCAGTCGGTAGAGCATGGGACTCTTA	12	21	12	1

tsrna-13060	TCAGTCGGTAGAGCATGGGACTCTT	13	23	5	4
tsrna-13059	TCAGTCGGTAGAGCATGGGACTCT	9	25	8	3
tsrna-13058	TCAGTCGGTAGAGCATGGGACTC	14	17	4	0
tsrna-13057	TCAGTCGGTAGAGCATGGGACT	17	17	5	2
tsrna-13056	TCAGTCGGTAGAGCATGGGAC	11	16	4	1
tsrna-13055	TCAGTCGGTAGAGCATGGGA	7	10	5	0
tsrna-13054	TCAGTCGGTAGAGCATGGG	3	4	1	1
tsrna-13053	TCAGTCGGTAGAGCATGG	2	1	1	0
tsrna-13052	TCAGTCGGTAGAGCATGAGACTCTTA	13	31	4	9
tsrna-13051	TCAGTCGGTAGAGCATGAGACTCTT	17	35	8	8
tsrna-13050	TCAGTCGGTAGAGCATGAGACTCT	21	31	6	3
tsrna-13049	TCAGTCGGTAGAGCATGAGACTC	14	31	4	3
tsrna-13048	TCAGTCGGTAGAGCATGAGACT	6	24	1	6
tsrna-13047	TCAGTCGGTAGAGCATGAGAC	12	15	4	2
tsrna-13046	TCAGTCGGTAGAGCATGAGA	6	10	2	1
tsrna-13045	TCAGTCGGTAGAGCATGAG	3	8	1	0
tsrna-13044	TCAGTCGGTAGAGCATGA	0	4	0	0
tsrna-13043	TCAGTCGGTAGAGCATG	2	1	2	0
tsrna-13042	TCAGTCGGTAGAGCATCAGACTTTTAATC	11	14	10	3
tsrna-13041	TCAGTCGGTAGAGCATCAGACTTTTAA	9	15	9	2
tsrna-13040	TCAGTCGGTAGAGCATCAGACTTTTA	9	9	5	1
tsrna-13039	TCAGTCGGTAGAGCATCAGACTTTT	10	10	9	2
tsrna-13038	TCAGTCGGTAGAGCATCAGACTTT	15	13	4	2
tsrna-13037	TCAGTCGGTAGAGCATCAGACTT	11	6	5	1
tsrna-13036	TCAGTCGGTAGAGCATCAGACT	7	9	8	2
tsrna-13035	TCAGTCGGTAGAGCATCAGAC	14	12	8	1
tsrna-13034	TCAGTCGGTAGAGCATCAGA	7	17	3	0
tsrna-13033	TCAGTCGGTAGAGCATCAG	6	3	3	1
tsrna-13032	TCAGTCGGTAGAGCATCA	5	5	3	2
tsrna-13031	TCAGTCGGTAGAGCATC	1	2	2	1
tsrna-13030	TCAGTCGGTAGAGCAT	0	0	0	0
tsrna-13029	TCAGTCAGTAGAGCATCAGAC	2	0	0	1
tsrna-13024	TCAGGTGGTAGAGCATTTGACTG	1	0	0	0
tsrna-13023	TCAGGTGGTAGAGCATTTGACT	0	1	0	0
tsrna-13019	TCAGGGTCGTGGGTTCGAGCC	0	1	0	0
tsrna-13018	TCAGGGTCGTGGGTTCGAGC	0	0	0	0
tsrna-13017	TCAGGGTCGTGGGTTCGAG	0	0	0	0
tsrna-13015	TCAGGGTCGTGGGTTCG	0	0	0	0
tsrna-13014	TCAGGGTCGTGGGTTC	0	0	0	0
tsrna-13013	TCAGGGGTTAGAGCACTGG	0	1	0	0
tsrna-13012	TCAGGGGTAGAGCATTTGACTGC	0	0	0	0
tsrna-13009	TCAGGGATGGGTTCGATTCTCA	3	6	2	1
tsrna-13008	TCAGGGATGGGTTCGATTCTC	5	3	1	0
tsrna-13007	TCAGGGATGGGTTCGATTCT	1	2	4	1
tsrna-13006	TCAGGGATGGGTTCGA	2	0	1	0
tsrna-13005	TCAGGATGGCCGAGTGGTCTAAG	0	2	0	0
tsrna-13004	TCAGGATGGCCGAGCGGTCTAAGGCGCTGCGTTC	1	5	2	2
tsrna-13003	TCAGGATGGCCGAGCGGTCTAAGGCGCTGCGTT	2	6	1	0
tsrna-13002	TCAGGATGGCCGAGCGGTCTAAGGCGCTGCG	1	2	1	2
tsrna-13001	TCAGGATGGCCGAGCGGTCTAAGGCGCTGC	0	9	1	0
tsrna-13000	TCAGGATGGCCGAGCGGTCTAAGGCGCTG	1	3	1	0
tsrna-12999	TCAGGATGGCCGAGCGGTCTAAGGC	3	0	3	0
tsrna-12998	TCAGGATGGCCGAGCGGTCTAAGG	1	4	0	0
tsrna-12997	TCAGGATGGCCGAGCGGTCTAAG	0	2	1	0
tsrna-12996	TCAGGATGGCCGAGCGGTCTA	0	1	0	0
tsrna-12994	TCAGGATGGCCGAGCGGTC	1	0	0	1
tsrna-12993	TCAGGATGGCCGAGCGG	1	0	0	0
tsrna-12992	TCAGGATGGCCGAGCG	0	1	0	0
tsrna-12989	TCAGCTAAATAAGCTATCGGGCCCATACCCCGAAA	5	3	4	1
tsrna-12988	TCAGCTAAATAAGCTATCGGGCCCA	1	3	2	0
tsrna-12987	TCAGCTAAATAAGCTATCGGGCCC	0	0	0	0
tsrna-12986	TCAGCTAAATAAGCTATCGGGCC	0	0	0	0
tsrna-12984	TCAGCCTCCGGAGCTGGGGATTGTGGGT	1	2	2	1
tsrna-12983	TCAGCCTCCGGAGCTGGGGATTGTGGG	0	1	0	1

tsrna-12978	TCAGCACTCTGGACTCTGAATCC	0	1	0	0
tsrna-12977	TCAGCACTCTGGACTCTGAATC	0	1	0	0
tsrna-12972	TCAGATTGTGAATCTGACAACAGAGGCTTACGACCC	1	8	9	0
tsrna-12971	TCAGATTGTGAATCTGACAACAGAGGCTT	1	3	2	0
tsrna-12970	TCAGATTGTGAATCTGACAACAGAGGCT	0	4	1	1
tsrna-12969	TCAGATTGTGAATCTGACAACAG	0	4	2	0
tsrna-12968	TCAGATTGTGAATCTGACAACA	1	3	1	0
tsrna-12967	TCAGATTGTGAATCTGACAAC	0	1	0	1
tsrna-12966	TCAGATTGTGAATCTGACA	0	0	1	0
tsrna-12962	TCAGAGTTCAAGTCTCACTGGGA	0	1	0	0
tsrna-12960	TCAGACTTTTAATCTGAGGGTCCAGGGT	21	43	6	9
tsrna-12959	TCAGACTTTTAATCTGAGGGTCCAG	12	39	9	3
tsrna-12958	TCAGAAGGTTGCGTGTTCAAGTCACGTCGGGGTCACCA	9	15	3	1
tsrna-12957	TCAGAAGGTTGCGTGTTCAAGTCACGTCGGGGTCACC	2	6	4	0
tsrna-12956	TCAGAAGGTTGCGTGTTCAAGTCACGTCGG	2	6	1	0
tsrna-12955	TCAGAAGGTTGCGTGTTCAAG	4	4	0	0
tsrna-12954	TCAGAAGGTTGCGTGTTCAAATCACGTCGGGGTCACCA	8	29	8	6
tsrna-12953	TCAGAAGGTTGCGTGTTCAAATCACGTCGGGGTCACC	5	10	0	0
tsrna-12952	TCAGAAGGTTGCGTGTTCAAATCACGTCGG	1	2	0	0
tsrna-12951	TCAGAAGGTTGCGTGTTCAAA	1	2	0	0
tsrna-12950	TCAGAAGGTTGCGTGTTCAA	1	5	1	0
tsrna-12949	TCAGAAGGTTGCGTGTTCA	4	2	0	2
tsrna-12948	TCAGAAGGTTGCGTGTTC	4	5	1	0
tsrna-12947	TCAGAAGGTTGCGTGTT	1	4	1	0
tsrna-12946	TCAGAAGGTTGCGTGT	1	3	0	0
tsrna-12945	TCAGAAGGCTGCGTGTTCGAATCACGTCGGGGTCACCA	7	17	6	5
tsrna-12944	TCAGAAGGCTGCGTGTTCGAATCACGTCGGGGTCACC	1	1	1	0
tsrna-12943	TCAGAAGGCTGCGTGTTCGAATCACGTCGGGGTCAC	0	2	2	0
tsrna-12942	TCAGAAGGCTGCGTGTTCGAATCACGTCGGGGTC	2	1	1	0
tsrna-12941	TCAGAAGGCTGCGTGTTCGAATCACGTCGGGG	0	2	0	0
tsrna-12940	TCAGAAGGCTGCGTGTTCGAATCACGTCGGG	0	4	0	1
tsrna-12939	TCAGAAGGCTGCGTGTTCGAATCACGTCGG	2	1	1	0
tsrna-12938	TCAGAAGGCTGCGTGTTCGAATCACGTCG	2	1	0	0
tsrna-12937	TCAGAAGGCTGCGTGTTCGAATCA	0	1	0	0
tsrna-12936	TCAGAAGGCTGCGTGTTCGAATC	0	2	0	0
tsrna-12935	TCAGAAGGCTGCGTGTTCGAAT	0	2	1	1
tsrna-12933	TCAGAAGGCTGCGTGTTCGA	0	1	1	0
tsrna-12932	TCAGAAGGCTGCGTGTTCG	0	0	0	0
tsrna-12931	TCAGAAGGCTGCGTGTTC	1	0	0	0
tsrna-12928	TCAGAAGATTGCAGGTTCGAGTCCTGCCGCGGTCGCCA	3	7	2	0
tsrna-12927	TCAGAAGATTGCAGGTTCGAGTCCTGCCGCGGTCGCC	0	1	0	0
tsrna-12925	TCAGAAGATTGAGGGTTCGAGTCCCTTCGTGGTCGCCA	4	16	5	4
tsrna-12924	TCAGAAGATTGAGGGTTCGAGTCCCTTCGTGGTCGCC	3	7	6	3
tsrna-12923	TCAGAAGATTGAGGGTTCGAGTCCCTTCGT	7	9	2	0
tsrna-12922	TCAGAAGATTGAGGGTTCGAATCCCTTCGTGGTTGCCA	1	15	1	2
tsrna-12921	TCAGAAGATTGAGGGTTCGAATCCCTTCGTGGTTGCC	9	8	0	2
tsrna-12920	TCAGAAGATTGAGGGTTCGAATCCCTTCGT	3	13	2	2
tsrna-12919	TCAGAAGATTGAGGGTTCGAATCCCTCCGT	5	5	1	2
tsrna-12918	TCAGAAGATTCTAGGTTCGACTCCTGGCTGGCTCGCCA	39	144	20	10
tsrna-12917	TCAGAAGATTCTAGGTTCGACTCCTGGCTGGCTCGCC	8	15	2	0
tsrna-12916	TCAGAAGATTCTAGGTTCGACTCCTGGCTG	0	6	1	1
tsrna-12915	TCAGAAGATTCCAGGTTCGACTCCTGGCTGGCTCGCCA	30	134	21	13
tsrna-12914	TCAGAAGATTCCAGGTTCGACTCCTGGCTGGCTCGCC	2	5	4	0
tsrna-12913	TCAGAAGATTCCAGGTTCGACTCCTGGCTGGCTCGC	0	2	0	1
tsrna-12912	TCAGAAGATTCCAGGTTCG	4	3	0	0
tsrna-12911	TCAGAAGATTCCAGGTTC	2	1	0	0
tsrna-12910	TAATGGATAAGGCACTGGC	23	24	14	7
tsrna-12909	TAATGGATAAGGCACTGG	11	13	8	3
tsrna-12908	TAATGGATAAGGCACTG	17	16	6	4
tsrna-12907	TAATGGATAAGGCACT	6	1	1	1
tsrna-12906	TAATGCCGAGGTTGTGAGTTCGAG	3	21	2	0
tsrna-12905	TAATGCCGAGGTTGTGAGTTCGA	1	9	1	0
tsrna-12904	TAATGCCGAGGTTGTGAGTTCG	1	12	0	0
tsrna-12903	TAATGCCGAGGTTGTGAGTTCA	3	11	2	0

tsrna-12902	TAATGCCGAGGTTGTGAGTTC	1	3	2	0
tsrna-12901	TAATGCCGAGGTTGTGAGTT	0	3	1	0
tsrna-12900	TAATGCCGAGGTTGTGAGT	0	0	0	0
tsrna-12899	TAATGCCGAGGTTGTGAG	0	0	0	0
tsrna-12896	TAATCTGAGGGTCCAGGGTTCAAGTCCCTGTTCGGGCGCCA	6	23	4	2
tsrna-12895	TAATCTGAGGGTCCAGGGTTCA	0	4	0	0
tsrna-12894	TAATCTGAGGGTCCAGGGTTC	1	1	1	0
tsrna-12893	TAATCTGAGGGTCCAGGGTT	0	1	0	0
tsrna-12892	TAATCTGAAGGTCGTGAGTTCGATCCTCACACGGGGCACCA	4	40	2	3
tsrna-12891	TAATCTGAAGGTCGTGAGTTCGATCCTCACACGGGGCACC	8	9	5	2
tsrna-12890	TAATCTGAAGGTCGTGAGTTCGATCCTCACACGGGGCAC	7	14	4	1
tsrna-12889	TAATCTGAAGGTCGTGAGTTCGATCCTCACACGGGGC	11	16	5	0
tsrna-12888	TAATCTGAAGGTCGTGAGTTCGAGCCTCACACGGGGCACCA	9	18	10	3
tsrna-12887	TAATCTGAAGGTCGTGAGTTCGAGCCTCACACGGGGCACC	3	16	6	2
tsrna-12886	TAATCTGAAGGTCGTGAGTTCGAGCCTCACACGGGGCAC	7	12	3	1
tsrna-12885	TAATCTGAAGGTCGTGAGTTCGAGCCTCACACGGGGC	10	15	9	0
tsrna-12884	TAATCTGAAGGTCGTGAGTTCGAGCCTCACACGGGG	3	16	0	1
tsrna-12883	TAATCTGAAGGTCGTGAGTTCGAGC	5	19	5	0
tsrna-12882	TAATCTGAAGGTCGTGAGTTCG	5	14	5	1
tsrna-12881	TAATCTGAAGGTCGTGAGTTC	4	9	3	4
tsrna-12880	TAATCTGAAGGTCGTGAGT	3	7	3	0
tsrna-12879	TAATCTGAAGGTCGTGAG	1	3	0	0
tsrna-12878	TAATCTGAAGGTCGTGA	1	4	0	0
tsrna-12877	TAATCTGAAGGTCGTG	1	1	0	0
tsrna-12876	TAATCTGAAGGTCCTGAGTTCGAGCCTCAGAGAGGGC	73	171	37	22
tsrna-12875	TAATCTGAAGGTCCTGAGTTCGAACCTCAGAGGGGGCACCA	65	169	39	24
tsrna-12874	TAATCTGAAGGTCCTGAGTTCGAACCTCAGAGGGGGCACC	51	157	36	25
tsrna-12873	TAATCTGAAGGTCCTGAGTTCGAACCTCAGAGGGGGCAC	46	142	40	20
tsrna-12872	TAATCTGAAGGTCCTGAGTTCGAACCTCAGAGGGGGC	61	168	47	16
tsrna-12871	TAATCTGAAGGTCCTGAGTTCGAACCTCAG	63	149	46	15
tsrna-12870	TAATCTGAAGGTCCTGAGTTCGAACC	61	175	32	27
tsrna-12869	TAATCTGAAGGTCCTGAGTTCG	65	142	46	15
tsrna-12868	TAATCTGAAGGTCCTGAGT	63	156	37	20
tsrna-12867	TAATCTGAAGGTCCTGAG	2	13	1	0
tsrna-12866	TAATCTGAAGGTCCTGA	2	8	3	1
tsrna-12865	TAATCTGAAGGTCCTG	3	6	0	0
tsrna-12864	TAATCTCAGGGTCGTGGGTTCG	0	1	0	0
tsrna-12863	TAATCTCAGGGTCGTGGGTTC	1	1	0	0
tsrna-12862	TAATCTCAGGGTCGTGGGTT	0	1	0	0
tsrna-12861	TAATCTCAGGGTCGTGGGT	1	0	0	0
tsrna-12860	TAATCTCAGGGTCGTGG	0	0	0	0
tsrna-12856	TAATCCATTGTGCTTTGCACGCGTGGGTTC	0	9	2	0
tsrna-12855	TAATCCAGAGGTTCCGGGTTCGAG	0	0	0	0
tsrna-12854	TAATCCAGAGGTTCCGGGTTCGA	2	0	0	1
tsrna-12847	TAATAGGAGCTTAAACCCCCTTATTTCTAC	0	0	1	0
tsrna-12846	TAATAGGAGCTTAAACCCCCT	0	0	1	0
tsrna-12840	TAATAACGCCAAGGTCGCGGGT	8	19	3	3
tsrna-12839	TAAGTTGCAATACTTAATTTCTGCCA	5	0	1	3
tsrna-12838	TAAGTTGCAATACTTAATTTCTGCC	0	0	1	0
tsrna-12837	TAAGTTAAAGATTAAGAGAACCAACACCTCTTTACAGTGACCA	4	1	16	1
tsrna-12836	TAAGTTAAAGATTAAGAGAACCAACACCTCTTTACAGTGACC	6	2	1	0
tsrna-12835	TAAGTTAAAGATTAAGAGAACCAACACC	0	0	0	0
tsrna-12834	TAAGTTAAAGATTAAGAGAACC	1	0	0	0
tsrna-12833	TAAGTTAAAGATTAAGAGAAC	0	0	0	0
tsrna-12832	TAAGTGTTTGTGGGTTTAAGTCCCATTGGTCTAGCCA	0	1	1	1
tsrna-12831	TAAGTGTTTGTGGGTTTAAGTCCCATTGGTCTAGC	1	3	0	0
tsrna-12830	TAAGTGTTTGTGGGTTTAAGTCCCATTGGT	1	0	0	0
tsrna-12829	TAAGTGTTTGTGGGTTTAAGTCCCATT	0	0	3	1
tsrna-12828	TAAGTGTTTGTGGGTTTAAGTCC	0	0	0	0
tsrna-12827	TAAGTGTTTGTGGGTTTAAGTC	1	0	1	0
tsrna-12825	TAAGTGTTTGTGGGTTTAAG	1	0	0	0
tsrna-12821	TAAGTCCCATTGGTCTAGCCA	0	1	0	0
tsrna-12818	TAAGGTCAGCTAAATAAGCTATCGGGCCCA	1	0	0	0
tsrna-12817	TAAGGTCAGCTAAATAAGCTATCGGGCCC	0	0	0	0

tsrna-12795	TAAGGCGTCTGACTTCGGATC	0	0	0	0
tsrna-12791	TAAGGCGTCTGACTTCG	0	1	0	0
tsrna-12789	TAAGGCGCCAGACTCAAG	0	0	0	0
tsrna-12788	TAAGCTATCGGGCCCATACCCCGAAAATGTTGGTTA	3	3	7	0
tsrna-12787	TAAGCTATCGGGCCCATACCCCGAAAATGTTGGTT	3	0	5	1
tsrna-12786	TAAGCTATCGGGCCCATACCCCGAAAATGTT	0	2	1	0
tsrna-12785	TAAGCTATCGGGCCCATACCCCGAAAATGT	0	3	0	1
tsrna-12784	TAAGCTATCGGGCCCATACCCCGAAA	0	1	1	0
tsrna-12783	TAAGCTATCGGGCCCATACCCCGAA	1	0	0	0
tsrna-12782	TAAGCTATCGGGCCCATACCCCGA	0	2	1	0
tsrna-12781	TAAGCTATCGGGCCCATACCCC	1	1	0	1
tsrna-12780	TAAGCTATCGGGCCCATACC	0	1	1	1
tsrna-12779	TAAGCTATCGGGCCCATA	0	1	1	0
tsrna-12778	TAAGCTATCGGGCCCA	1	0	0	0
tsrna-12777	TAAGCCAGGGATTGTGGGTTCGAGTCCCATCTGGGGTGCCA	24	71	16	10
tsrna-12776	TAAGCCAGGGATTGTGGGTTCGAGTCCCATCTGGGGTGCC	29	46	10	9
tsrna-12775	TAAGCCAGGGATTGTGGGTTCGAGTCCCATCTGGGGTGC	30	54	10	6
tsrna-12774	TAAGCCAGGGATTGTGGGTTCGAGTCCCACCTGGGGTACC	26	53	12	5
tsrna-12773	TAAGCCAGGGATTGTGGGTTCGAGTCCCACCCGGGGTACC	22	62	15	5
tsrna-12772	TAAGCCAGGGATTGTGGGTTCGAGTCCC	18	56	12	7
tsrna-12771	TAAGCCAGGGATTGTGGGTTCGAGT	27	59	13	8
tsrna-12770	TAAGCCAGGGATTGTGGGTTCGAG	15	47	10	7
tsrna-12769	TAAGCCAGGGATTGTGGGTTCGA	18	53	19	3
tsrna-12768	TAAGCCAGGGATTGTGGGTTCG	19	54	12	7
tsrna-12767	TAAGCCAGGGATTGTGGGTTC	25	48	9	7
tsrna-12766	TAAGCCAGGGATTGTGGGTT	18	60	14	9
tsrna-12765	TAAGCCAGGGATTGTGGGT	19	50	11	10
tsrna-12764	TAAGCCAGGGATTGTGGG	0	0	0	0
tsrna-12761	TAAGCACTCTGGACTCTGAATCCAGCG	2	4	1	1
tsrna-12760	TAAGCACTCTGGACTCTGAATCCAGC	0	4	0	2
tsrna-12759	TAAGCACTCTGGACTCTGAATCCA	0	4	1	0
tsrna-12758	TAAGCACTCTGGACTCTGAATCC	0	1	1	0
tsrna-12757	TAAGCACTCTGGACTCTGAATC	1	0	1	0
tsrna-12751	TAAGATGGCAGAGCCCGGTAATCGCATAAAACTTAA	0	0	0	1
tsrna-12746	TAAGAGAACCAACACCTCTTTACAGTGACCA	1	0	1	0
tsrna-12745	TAAGAGAACCAACACCTCTTTACAGTGACC	0	0	0	0
tsrna-12738	TAACTTTGTCAAAGTTAAATTATAGGCT	11	3	5	1
tsrna-12737	TAACTTTGTCAAAGTTAAATT	0	2	1	0
tsrna-12735	TAACTTGACCGCTCTGACCA	10	14	8	0
tsrna-12734	TAACTTGACCGCTCTGACC	1	6	1	0
tsrna-12733	TAACTTGACCGCTCTGAC	0	1	0	0
tsrna-12725	TAACTCATGCCCCCATGTCTAACAACATGGCTTTCTCACCA	10	10	31	0
tsrna-12724	TAACTCATGCCCCCATGTCTAACAACATGGCTTTCTCACC	14	4	11	0
tsrna-12723	TAACTCATGCCCCCATGTCTAACAACATGGCT	0	1	1	0
tsrna-12719	TAACTAAGTGTTTGTGGGTTT	0	2	0	0
tsrna-12718	TAACTAAGTGTTTGTGGGTT	1	1	0	0
tsrna-12717	TAACGCTAAGGTCGCGGGTTCGA	66	123	83	19
tsrna-12715	TAACGCGTCTGACTACGGATCAGAAGA	0	1	0	0
tsrna-12713	TAACGCGTCTGACTACGGAT	0	0	0	0
tsrna-12711	TAACGCCAAGGTCGCGGGTTCGATCCCCGTACGGGCCACCA	19	141	6	4
tsrna-12710	TAACGCCAAGGTCGCGGGTTCGATCCCCGTACGGGCCACC	7	13	3	0
tsrna-12709	TAACGCCAAGGTCGCGGGTTCGATCCCCGTACGGGC	1	2	1	0
tsrna-12708	TAACGCCAAGGTCGCGGGTTCGATC	4	5	0	0
tsrna-12707	TAACGCCAAGGTCGCGGGTTCGAT	2	12	0	0
tsrna-12706	TAACGCCAAGGTCGCGGGTTCGAA	5	5	1	0
tsrna-12705	TAACGCCAAGGTCGCGGGTTCGA	3	12	1	0
tsrna-12704	TAACGCCAAGGTCGCGGGTTCG	0	7	2	0
tsrna-12703	TAACGCCAAGGTCGCGGGTTC	2	2	1	0
tsrna-12702	TAACGCCAAGGTCGCGGGTT	0	1	0	0
tsrna-12701	TAACGCCAAGGTCGCGGGT	0	3	0	0
tsrna-12698	TAACCTTTTAAGTTAAAGATTAAGAGAACC	1	0	0	0
tsrna-12697	TAACCGAAAGGTTGGTGGTTCGAGCCCACCCAGGGACGCCA	11	111	2	3
tsrna-12696	TAACCGAAAGGTTGGTGGTTCGAGCCCACCCAGGGACGCC	4	13	1	1
tsrna-12695	TAACCGAAAGGTTGGTGGTTCGAGCCCACCCAGGGAC	1	4	1	1

tsrna-12694	TAACCGAAAGGTTGGTGGTTCGA	0	1	0	0
tsrna-12693	TAACCGAAAGGTTGGTGGTTCG	1	0	0	0
tsrna-12692	TAACCGAAAGGTTGGTGGTT	0	3	0	0
tsrna-12691	TAACCCAGAGGTCGATGGATCT	13	25	3	2
tsrna-12690	TAACCCAGAGGTCGATGGATCGAAACCATC	5	3	0	0
tsrna-12689	TAACCCAGAGGTCGATGGATCGAAACCA	0	3	0	0
tsrna-12688	TAACCCAGAGGTCGATGGATCGAAACC	2	1	0	0
tsrna-12687	TAACCCAGAGGTCGATGGATCGAAAC	1	4	2	0
tsrna-12686	TAACCCAGAGGTCGATGGATCGAAA	0	5	0	0
tsrna-12685	TAACCCAGAGGTCGATGGATCGAA	3	4	0	0
tsrna-12684	TAACCCAGAGGTCGATGGATCGA	0	4	1	1
tsrna-12683	TAACCCAGAGGTCGATGGATCG	0	1	1	0
tsrna-12682	TAACCCAGAGGTCGATGGATC	1	3	1	0
tsrna-12681	TAACCCAGAGGTCGATGGAT	0	4	0	1
tsrna-12680	TAACCCAGAGGTCGATGGA	3	1	1	0
tsrna-12679	TAACCCAGAGGTCGATGG	0	3	0	1
tsrna-12678	TAACCCAGAGGTCGATG	1	0	0	0
tsrna-12677	TAACCCAGAGGTCGAT	0	1	0	0
tsrna-12676	TAACCCAGAGGTCAATGGATCGA	0	1	0	0
tsrna-12675	TAACCAAAACATCAGATTGTGAATCTGACAACAGAGGCTTACC	5	10	9	1
tsrna-12674	TAACCAAAACATCAGATTGTGAATCTGACAACAGAGGCT	0	5	5	0
tsrna-12673	TAACCAAAACATCAGATTGTGAATCTGACAACAGAGG	1	2	1	0
tsrna-12672	TAACCAAAACATCAGATTGTGAATCTGAC	0	0	1	0
tsrna-12662	TAACAGCTATCCATTGGTCTTAGGCCCC	1	1	0	0
tsrna-12661	TAACAGCTATCCATTGGTCTTAGGCCC	0	0	0	0
tsrna-12660	TAACAGCTATCCATTGGTCTTAGGCC	0	0	1	0
tsrna-12659	TAACAGCTATCCATTGGTCTTAGGC	0	0	0	0
tsrna-12658	TAACAGCTATCCATTGGTCTTAGG	0	0	0	0
tsrna-12657	TAACAGCTATCCATTGGTCTTAG	0	0	1	0
tsrna-12656	TAACAGCTATCCATTGGTCTTA	0	1	0	0
tsrna-12655	TAACAGCTATCCATTGGTCTT	0	0	0	0
tsrna-12650	TAACACCAAGGTCGCGGGCTCGAC	0	0	0	0
tsrna-12648	TAACACCAAGGTCGCGGGCTCG	0	1	0	0
tsrna-12647	TAACACCAAGGTCGCGGGCT	0	0	2	0
tsrna-12644	TAACACAAAGCACCCAACTTACACTTAGGA	0	0	1	0
tsrna-12643	TAACACAAAGCACCCAACTTACACTTAGG	0	0	0	0
tsrna-12640	TAACACAAAGCACCCAACTTACACTT	0	0	0	0
tsrna-12634	TAACACAAAGCACCCAACTT	0	0	0	0
tsrna-12633	TAACACAAAGCACCCAACT	0	0	0	0
tsrna-12629	TAACAACATGGCTTTCTCACCA	0	0	1	0
tsrna-12628	TAACAACATGGCTTTCTCACC	0	0	0	0
tsrna-12627	TAACAACATGGCTTTCTCAC	0	0	0	0
tsrna-12626	TAACAACATGGCTTTCTCA	1	0	0	0
tsrna-12621	TAAATTATAGGCTAAATCCTATATATCTT	1	0	1	0
tsrna-12619	TAAATTAGAATCTTAGCTTTGGGTGCTAATGGTGGAGTTAAAG	6	6	7	0
tsrna-12618	TAAATTAGAATCTTAGCTTTGGGTGCTAATGG	4	2	3	1
tsrna-12617	TAAATTAGAATCTTAGCTTTGGGTGCTA	1	2	4	0
tsrna-12616	TAAATTAGAATCTTAGCTTTGGGTGCT	6	1	1	1
tsrna-12615	TAAATTAGAATCTTAGCTTTGGGTG	3	4	1	2
tsrna-12614	TAAATTAGAATCTTAGCTTTGGGT	7	1	3	0
tsrna-12613	TAAATTAGAATCTTAGCTTTGG	2	0	3	1
tsrna-12612	TAAATTAGAATCTTAGCTTTG	1	0	4	2
tsrna-12611	TAAATTAGAATCTTAGCTTT	1	2	4	1
tsrna-12609	TAAATCTAAAGACAGGGGTTAGGCCTCTTTTTACCACCA	74	45	65	6
tsrna-12608	TAAATCTAAAGACAGGGGTTAGGCCTCTTTTTACCACC	43	48	27	2
tsrna-12607	TAAATCTAAAGACAGGGGTTAGGCCTCTTT	7	18	2	0
tsrna-12606	TAAATCTAAAGACAGGGGTTAGGCCTCTT	5	6	2	0
tsrna-12605	TAAATCTAAAGACAGGGGTTAGGCCTC	1	1	0	0
tsrna-12604	TAAATCTAAAGACAGGGGTTAGGCCT	0	0	0	0
tsrna-12602	TAAATCTAAAGACAGGGG	1	0	0	0
tsrna-12598	TAAATAGTACCGTTAACTTCCA	1	2	3	0
tsrna-12596	TAAATACAGACCAAGAGCCTTCAAAGCCCT	22	6	15	2
tsrna-12595	TAAATACAGACCAAGAGCCTTCAA	18	1	7	6
tsrna-12594	TAAATACAGACCAAGAGCCTTCA	12	3	9	3

tsrna-12593	TAAATACAGACCAAGAGCCTTC	10	0	8	0
tsrna-12592	TAAATACAGACCAAGAGCCTT	0	0	1	0
tsrna-12591	TAAATACAGACCAAGAGCCT	1	0	1	0
tsrna-12590	TAAATACAGACCAAGAGCC	0	0	0	0
tsrna-12586	TAAATAATAGGAGCTTAAACCC	0	1	0	0
tsrna-12585	TAAATAATAGGAGCTTAAACC	0	0	0	0
tsrna-12583	TAAATAATAGGAGCTT	0	0	0	0
tsrna-12582	TAAATAAGCTATCGGGCCCATA	1	1	1	0
tsrna-12581	TAAATAAGCTATCGGGCCCA	1	1	1	0
tsrna-12580	TAAATAAGCTATCGGGCCC	0	0	0	0
tsrna-12577	TAAAGTGGCTGATTTGCGTTCA	0	0	0	0
tsrna-12576	TAAAGTGGCTGATTTGCGTTC	0	1	0	0
tsrna-12575	TAAAGGTCCCTGGTTCGATCCC	12	11	7	2
tsrna-12574	TAAAGGTCCCTGGTTCG	11	17	7	6
tsrna-12572	TAAAGATTAAGAGAACCAACACCTCTTTACAGTGACCA	10	1	9	0
tsrna-12571	TAAAGATTAAGAGAACCAACACCTCTTTACAGTGACC	3	2	3	1
tsrna-12570	TAAAGATTAAGAGAACCAACACCTCTTTACAGTGAC	2	0	1	0
tsrna-12569	TAAAGATTAAGAGAACCAACACCTCTTTACAGTGA	1	1	2	0
tsrna-12568	TAAAGATTAAGAGAACCAACACCTCTTTAC	1	0	0	0
tsrna-12567	TAAAGATTAAGAGAACCAACACCTCTTTA	1	1	0	0
tsrna-12566	TAAAGATTAAGAGAACCAACACC	0	0	1	0
tsrna-12564	TAAAGATTAAGAGAACCAACA	0	0	0	0
tsrna-12562	TAAAGATTAAGAGAACCAA	0	0	0	0
tsrna-12561	TAAAGATTAAGAGAACCA	0	0	0	0
tsrna-12558	TAAAGACTTTTTCTCTGACCA	8	15	14	11
tsrna-12557	TAAAGACTTTTTCTCTGACC	0	1	3	0
tsrna-12556	TAAAGACTTTTTCTCTGAC	0	2	0	0
tsrna-12554	TAAAGACTTTTTCTCTG	0	1	0	0
tsrna-12553	TAAAGACAGGGGTTAGGCCTCTTTTTACCACCA	6	6	5	1
tsrna-12552	TAAAGACAGGGGTTAGGCCTCTTTTTACCACC	2	4	1	2
tsrna-12551	TAAAGACAGGGGTTAGGCCTCTTTTTACC	1	5	1	0
tsrna-12550	TAAAGACAGGGGTTAGGCCT	0	0	0	1
tsrna-12549	TAAAGACAGGGGTTAGGCC	0	0	0	0
tsrna-12547	TAAACCGGAGATGAAAACCTTTTTCCAAGGACACCA	43	32	94	0
tsrna-12546	TAAACCGGAGATGAAAACCTTTTTCCAAGGACACC	8	12	6	0
tsrna-12545	TAAACCGGAGATGAAAACCTTTTTCCAAGG	2	11	1	0
tsrna-12544	TAAACCGGAGATGAAAACCTTTTTCCAAG	3	9	0	0
tsrna-12543	TAAACCGGAGATGAAAACCT	0	0	0	0
tsrna-12540	TAAACCGAAGATCGCGGGTTCGAACCCCGTCCGTGCCTCCA	14	33	2	0
tsrna-12539	TAAACCGAAGATCGCGGGTTCGAACCCCGT	3	18	1	0
tsrna-12538	TAAACCGAAGATCGCGGGTTCGAAC	3	8	0	0
tsrna-12537	TAAACCGAAGATCGCGGGTTCGAA	1	8	1	0
tsrna-12536	TAAACCGAAGATCGCGGGTTCGA	2	6	3	0
tsrna-12535	TAAACCGAAGATCGCGGGTTCG	3	4	2	0
tsrna-12534	TAAACCGAAGATCGCGGGT	2	6	0	2
tsrna-12532	TAAACCGAAGATCACGGGTTCGAACCCCGTCCGTGCCTCCA	4	16	3	0
tsrna-12531	TAAACCGAAGATCACGGGTTCGA	0	2	2	0
tsrna-12530	TAAACCCCCTTATTTCTACCA	1	3	0	0
tsrna-12529	TAAACCCCCTTATTTCTACC	1	1	1	0
tsrna-12528	TAAACCCCCTTATTTCTAC	0	1	0	0
tsrna-12527	TAAACCAGGGGTCGCGAGTTCGA	0	1	1	1
tsrna-12526	TAAACCAGGGGTCGCGAGTTCG	0	5	0	1
tsrna-12525	TAAACCAGGGGTCGCGAGTTCAAATCTCGCTGGGGCCTCCA	41	133	23	22
tsrna-12524	TAAACCAGGGGTCGCGAGTTCAAATCTCGCTGGGGCCT	1	3	1	0
tsrna-12523	TAAACCAGGGGTCGCGAGTTCAAA	1	0	2	0
tsrna-12522	TAAACCAGGGGTCGCGAGTTCAA	0	2	1	0
tsrna-12521	TAAACCAGGGGTCGCGAGTTCA	1	1	0	0
tsrna-12520	TAAACCAGGGGTCGCGAGTTC	1	3	0	0
tsrna-12519	TAAACCAGGGGTCGCGAGTT	2	1	0	1
tsrna-12518	TAAACCAGGGGTCGCGAGT	0	2	0	0
tsrna-12516	TAAACCAGGGGTCGCGA	0	1	0	0
tsrna-12514	TAAACAGGAGATCCTGGGTTCGAATCCCAGCGGTGCCTC	11	30	6	2
tsrna-12513	TAAACAGGAGATCCTGGGTTCGAATCCCAGCGGTGCCT	5	22	8	0
tsrna-12512	TAAACAGGAGATCCTGGGTTCGAATCCCAGCGGTGCC	10	31	3	3

tsrna-12511	TAAACAGGAGATCCTGGGTTCGAATCCCAGCGGTGC	17	27	4	1
tsrna-12510	TAAACAGGAGATCCTGGGTTCGAATCCCAGCGGGGCCTC	10	21	6	2
tsrna-12509	TAAACAGGAGATCCTGGGTTCGAATCCCAGCGGGGCCT	5	19	8	1
tsrna-12508	TAAACAGGAGATCCTGGGTTCGAATCCCAGCGGGGCC	11	22	7	2
tsrna-12507	TAAACAGGAGATCCTGGGTTCGAATCCCAGCGGGGC	5	27	2	1
tsrna-12506	TAAACAGGAGATCCTGGGTTCGA	9	20	4	2
tsrna-12505	TAAACAGGAGATCCTGGGTTCG	8	23	12	0
tsrna-12504	TAAACAAAACGAATGATTTCGACTCATTAAATTATG	3	10	9	1
tsrna-12503	TAAACAAAACGAATGATTTCGACTC	1	3	2	0
tsrna-12501	TAAAATGGCTGAGTGAAGCATTGGACTGT	0	0	1	0
tsrna-12500	TAAAATGGCTGAGTGAAGCATTGGACTG	0	0	1	0
tsrna-12499	TAAAATGGCTGAGTGAAGCATTGGACT	0	1	0	0
tsrna-12498	TAAAATGGCTGAGTGAAGCATTGGAC	0	0	0	0
tsrna-12497	TAAAATGGCTGAGTGAAGCATTGG	1	0	0	0
tsrna-12496	TAAAATGGCTGAGTGAAGCATTG	1	1	0	0
tsrna-12492	TAAAACTTAAAACTTTACAGTCAG	0	0	0	1
tsrna-12490	GTTTTTCATATCATTGGTCGTGGTTGTAGTCCGTGCGAGAATA	145	163	182	62
tsrna-12489	GTTTTTCATATCATTGGTCGTGGTTGTAGTCCGTGCGAGAATA	18	37	21	22
tsrna-12488	GTTTTTCATATCATTGGTCGTGGTTGTAGTCCGTGCGAGAATA	11	33	11	15
tsrna-12487	GTTTTTCATATCATTGGTCGTGGTTGTAGTCCGTGCGAGAAT	7	17	11	13
tsrna-12486	GTTTTTCATATCATTGGTCGTGGTTGTAGTCCGTGCGAGAA	9	12	8	12
tsrna-12485	GTTTTTCATATCATTGGTCGTGGTTGTAGTCCGTGCGAGA	9	15	9	15
tsrna-12484	GTTTTTCATATCATTGGTCGTGGTTGTAGTCCGTGCGA	5	10	5	7
tsrna-12483	GTTTTTCATATCATTGGTCGTGGTTGTAGTCCGTGCG	8	6	9	7
tsrna-12482	GTTTTTCATATCATTGGTCGTGGTTGTAGTCCGTGC	1	4	3	2
tsrna-12481	GTTTTTCATATCATTGGTCGTGGTTGTAGTCC	2	1	1	1
tsrna-12480	GTTTTTCATATCATTGGTCGTGGTTGTAGT	0	0	1	0
tsrna-12479	GTTTTTCATATCATTGGTCGTGGTTGTAG	0	0	0	0
tsrna-12478	GTTTTTCATATCATTGGTCGTGGTTGTA	0	1	0	0
tsrna-12477	GTTTTTCATATCATTGGTCGTGGTTGT	0	1	0	0
tsrna-12476	GTTTTTCATATCATTGGTCGTGGTTG	1	0	0	0
tsrna-12475	GTTTTTCATATCATTGGTCGTGGTT	1	0	0	0
tsrna-12474	GTTTTTCATATCATTGGTCGTGGT	0	0	0	0
tsrna-12473	GTTTTTCATATCATTGGTCGTGG	0	1	0	1
tsrna-12465	GTTTTGCAGTCCTTACCA	0	0	1	0
tsrna-12463	GTTTTGACAACATTCAAAAAAGAGTACCA	0	1	1	0
tsrna-12462	GTTTTCACCCAGGTGGCCCGGGTT	3	7	1	1
tsrna-12461	GTTTTCACCCAGGTGGCCCGGGT	5	5	2	0
tsrna-12460	GTTTTCACCCAGGTGGCCCGG	3	1	1	0
tsrna-12459	GTTTTCACCCAGGTGGCCCG	5	8	1	1
tsrna-12458	GTTTTCACCCAGGTGGCCC	3	6	2	0
tsrna-12457	GTTTTCACCCAGGTGGCC	6	3	2	0
tsrna-12456	GTTTTCACCCAGGCGGCCCGGGTTCGACTCCCGGTGTGGGA	83	332	48	20
tsrna-12455	GTTTTCACCCAGGCGGCCCGGGT	1	0	1	0
tsrna-12454	GTTTTCACCCAGGCGGCCCGGG	1	2	1	0
tsrna-12453	GTTTTCACCCAGGCGGCCCGG	0	4	0	0
tsrna-12452	GTTTTCACCCAGGCGGCCCG	2	5	0	0
tsrna-12451	GTTTTCACCCAGGCGGCCC	3	2	1	0
tsrna-12450	GTTTTCACCCAGGCGGCC	3	0	0	0
tsrna-12449	GTTTTCACCCAGGCGGC	0	1	1	0
tsrna-12448	GTTTTCACCCAGGCGG	1	3	1	1
tsrna-12447	GTTTGTGGGTTTAAGTCCCATTGGTCTAGCCA	0	1	1	0
tsrna-12445	GTTTGTGGGTTTAAGTCCCATTGGTCTAGC	0	0	0	0
tsrna-12444	GTTTGTGGGTTTAAGTCCCATTGGTCTA	1	0	1	0
tsrna-12443	GTTTGTGGGTTTAAGTCCCATTGGTCT	0	0	1	0
tsrna-12442	GTTTGTGGGTTTAAGTCCCATTGGTC	0	1	0	0
tsrna-12440	GTTTGTGGGTTTAAGTCCCATTGG	0	1	0	0
tsrna-12436	GTTTGTGGGTTTAAGTCCCA	0	0	0	0
tsrna-12433	GTTTGTGGGTTTAAGTC	0	0	0	0
tsrna-12432	GTTTGTGGGTTTAAGT	0	0	0	0
tsrna-12431	GTTTGGGTCCGAGAGGTCCCGGGT	0	2	0	0
tsrna-12426	GTTTCTGTGGTGTAGTGGTTATC	3	1	1	1
tsrna-12425	GTTTCCGTGGTGTAGTGGTTATCACATTCGCCT	13	2	11	7
tsrna-12424	GTTTCCGTGGTGTAGTGGTTATCACATTCG	10	1	1	11

tsrna-12423	GTTTCCGTGGTGTAGTGGTTATCACA	5	4	5	8
tsrna-12422	GTTTCCGTGGTGTAGTGGTTATC	3	6	4	4
tsrna-12421	GTTTCCGTGGTGTAGTGGTTAT	1	1	1	1
tsrna-12420	GTTTCCGTGGTGTAGTGGTT	1	2	0	0
tsrna-12419	GTTTCCGTGGTGTAGTGGT	0	1	1	3
tsrna-12418	GTTTCCGTGGTGTAGTGG	0	1	0	1
tsrna-12417	GTTTCCGTGGTGTAGT	0	0	0	0
tsrna-12416	GTTTCCGTAGTGTAGTGGTTATT	188	76	185	65
tsrna-12415	GTTTCCGTAGTGTAGTGGTTATCACGTTTGCCT	112	46	76	48
tsrna-12414	GTTTCCGTAGTGTAGTGGTTATCACGTTTGCC	115	48	68	76
tsrna-12413	GTTTCCGTAGTGTAGTGGTTATCACGTTTG	115	45	62	72
tsrna-12412	GTTTCCGTAGTGTAGTGGTTATCACGTTCGCCTCACACGCGA/	444	144	425	108
tsrna-12411	GTTTCCGTAGTGTAGTGGTTATCACGTTCGCCTCACACGCGA/	460	186	448	119
tsrna-12410	GTTTCCGTAGTGTAGTGGTTATCACGTTCGCCTCACACGCGA	450	147	450	108
tsrna-12409	GTTTCCGTAGTGTAGTGGTTATCACGTTCGCCTCACACGCG	406	165	426	88
tsrna-12408	GTTTCCGTAGTGTAGTGGTTATCACGTTCGCCTCACACGC	445	152	444	114
tsrna-12407	GTTTCCGTAGTGTAGTGGTTATCACGTTCGCCTCACACG	414	171	446	97
tsrna-12406	GTTTCCGTAGTGTAGTGGTTATCACGTTCGCCTCACAC	485	159	429	101
tsrna-12405	GTTTCCGTAGTGTAGTGGTTATCACGTTCGCCTCACA	419	169	469	120
tsrna-12404	GTTTCCGTAGTGTAGTGGTTATCACGTTCGCCTCAC	448	187	459	98
tsrna-12403	GTTTCCGTAGTGTAGTGGTTATCACGTTCGCCTCA	424	182	449	104
tsrna-12402	GTTTCCGTAGTGTAGTGGTTATCACGTTCGCCTC	421	163	406	99
tsrna-12401	GTTTCCGTAGTGTAGTGGTTATCACGTTCGCCTAAC	438	169	418	106
tsrna-12400	GTTTCCGTAGTGTAGTGGTTATCACGTTCGCCTAA	465	163	464	93
tsrna-12399	GTTTCCGTAGTGTAGTGGTTATCACGTTCGCCTA	426	163	465	105
tsrna-12398	GTTTCCGTAGTGTAGTGGTTATCACGTTCGCCT	426	155	451	97
tsrna-12397	GTTTCCGTAGTGTAGTGGTTATCACGTTCGCC	400	163	375	100
tsrna-12396	GTTTCCGTAGTGTAGTGGTTATCACGTTCGC	141	73	114	61
tsrna-12395	GTTTCCGTAGTGTAGTGGTTATCACGTTCG	116	43	76	77
tsrna-12394	GTTTCCGTAGTGTAGTGGTTATCACGTTC	101	36	80	74
tsrna-12393	GTTTCCGTAGTGTAGTGGTTATCACGTT	113	43	69	82
tsrna-12392	GTTTCCGTAGTGTAGTGGTTATCACGT	96	35	69	74
tsrna-12391	GTTTCCGTAGTGTAGTGGTTATCACG	107	41	75	57
tsrna-12390	GTTTCCGTAGTGTAGTGGTTATCAC	99	38	55	70
tsrna-12389	GTTTCCGTAGTGTAGTGGTTATCA	100	44	83	76
tsrna-12388	GTTTCCGTAGTGTAGTGGTTATC	106	25	66	57
tsrna-12387	GTTTCCGTAGTGTAGTGGTTAT	18	24	11	4
tsrna-12386	GTTTCCGTAGTGTAGTGGTTA	11	17	12	5
tsrna-12385	GTTTCCGTAGTGTAGTGGTT	20	15	8	8
tsrna-12384	GTTTCCGTAGTGTAGTGGTCATCACGTTCGCCTA	131	55	109	44
tsrna-12383	GTTTCCGTAGTGTAGTGGTCATCACGTTCGCCT	132	71	101	53
tsrna-12382	GTTTCCGTAGTGTAGTGGTCATCACGTTCGCC	93	40	69	44
tsrna-12381	GTTTCCGTAGTGTAGTGGTCATCACGTTCGC	101	34	62	38
tsrna-12380	GTTTCCGTAGTGTAGTGGTCATCACGTTCG	87	32	49	39
tsrna-12379	GTTTCCGTAGTGTAGTGGTCATCACGTTC	100	49	59	36
tsrna-12378	GTTTCCGTAGTGTAGTGGTCATCACGTT	76	43	51	42
tsrna-12377	GTTTCCGTAGTGTAGTGGTCATCACGT	75	40	55	50
tsrna-12376	GTTTCCGTAGTGTAGTGGTCATCACG	73	45	54	39
tsrna-12375	GTTTCCGTAGTGTAGTGGTCATCAC	68	25	61	46
tsrna-12374	GTTTCCGTAGTGTAGTGGTCATCA	76	26	55	29
tsrna-12373	GTTTCCGTAGTGTAGTGGTCATC	86	21	42	33
tsrna-12372	GTTTCCGTAGTGTAGTGGTCAT	22	17	7	9
tsrna-12371	GTTTCCGTAGTGTAGTGGTCA	23	22	8	8
tsrna-12370	GTTTCCGTAGTGTAGTGGTC	11	10	11	4
tsrna-12369	GTTTCCGTAGTGTAGTGGT	21	20	5	7
tsrna-12368	GTTTCCGTAGTGTAGTGG	8	18	7	2
tsrna-12367	GTTTCCGTAGTGTAGTG	11	15	9	9
tsrna-12366	GTTTCCGTAGTGTAGT	5	12	5	3
tsrna-12365	GTTTCCGTAGTGTAGCGGTTATCACATTCGCCTCAC	218	94	188	18
tsrna-12364	GTTTCCGTAGTGTAGCGGTTATCACATTCGCCTCA	201	67	188	27
tsrna-12363	GTTTCCGTAGTGTAGCGGTTATCACATTCGCCTC	181	73	181	16
tsrna-12362	GTTTCCGTAGTGTAGCGGTTATCACATTCGCCT	125	62	126	19
tsrna-12361	GTTTCCGTAGTGTAGCGGTTATCACATTCGCC	98	47	69	20
tsrna-12360	GTTTCCGTAGTGTAGCGGTTATCACATTCGC	28	37	31	11

tsrna-12359	GTTTCCGTAGTGTAGCGGTTATCACATTCG	13	29	10	10
tsrna-12358	GTTTCCGTAGTGTAGCGGTTATCACATTC	12	23	16	5
tsrna-12357	GTTTCCGTAGTGTAGCGGTTATCACATT	21	19	15	11
tsrna-12356	GTTTCCGTAGTGTAGCGGTTATCACAT	16	16	7	7
tsrna-12355	GTTTCCGTAGTGTAGCGGTTATCACA	21	22	11	3
tsrna-12354	GTTTCCGTAGTGTAGCGGTTATCAC	17	15	14	7
tsrna-12353	GTTTCCGTAGTGTAGCGGTTATCA	14	18	13	8
tsrna-12352	GTTTCCGTAGTGTAGCGGTTATC	14	14	8	6
tsrna-12351	GTTTCCGTAGTGTAGCGGTTAT	10	17	3	4
tsrna-12350	GTTTCCGTAGTGTAGCGGTTA	9	17	11	4
tsrna-12349	GTTTCCGTAGTGTAGCGGTT	11	11	7	6
tsrna-12348	GTTTCCGTAGTGTAGCGGT	6	11	2	3
tsrna-12347	GTTTCCGTAGTGTAGCGG	7	13	5	4
tsrna-12346	GTTTCCGTAGTGTAGCG	4	12	3	5
tsrna-12345	GTTTCCGTAGTGTAGC	5	14	2	0
tsrna-12344	GTTTCCGTAGTGGAGTGGTTATCACGTTCGCCTC	0	1	2	1
tsrna-12343	GTTTCCGTAGTGGAGTGGTTATCACGTTCGCCT	0	1	1	4
tsrna-12342	GTTTCCGTAGTGGAGTGGTTATCACGTTCGCC	0	1	1	0
tsrna-12341	GTTTCCGTAGTGGAGTGGTTATCACGTTCGC	2	0	0	0
tsrna-12340	GTTTCCGTAGTGGAGTGGTTATCACGTTCG	0	0	0	0
tsrna-12339	GTTTCCGTAGTGGAGTGGTTATCACGTT	1	0	0	1
tsrna-12338	GTTTCCGTAGTGGAGTGGTTATCACG	1	0	0	0
tsrna-12337	GTTTCCGTAGTGGAGTGGTTATCAC	0	1	2	1
tsrna-12336	GTTTCCGTAGTGGAGTGGTTATCA	2	0	0	0
tsrna-12335	GTTTCCGTAGTGGAGTGGTTATC	0	0	2	2
tsrna-12333	GTTTCCGTAGTGGAGTGGTT	0	0	1	0
tsrna-12322	GTTTATGTAGCTTACCTCCTCAAAGCAATACACTGAA	69	38	59	40
tsrna-12321	GTTTATGTAGCTTACCTCCTCAAAGCAATACACTGA	71	38	40	35
tsrna-12320	GTTTATGTAGCTTACCTCCTCAAAGCAATACACTG	60	40	40	34
tsrna-12319	GTTTATGTAGCTTACCTCCTCAAAGCAATACACT	51	34	30	46
tsrna-12318	GTTTATGTAGCTTACCTCCTCAAAGCAATACA	35	20	23	20
tsrna-12317	GTTTATGTAGCTTACCTCCTCAAAGCAATA	25	15	23	29
tsrna-12316	GTTTATGTAGCTTACCTCCTCAAAGCAAT	17	11	11	20
tsrna-12315	GTTTATGTAGCTTACCTCCTCAAAGCAA	13	5	10	15
tsrna-12314	GTTTATGTAGCTTACCTCCTCAAAGCA	8	3	14	17
tsrna-12313	GTTTATGTAGCTTACCTCCTCAAAGC	10	3	10	19
tsrna-12312	GTTTATGTAGCTTACCTCCTCAAAG	13	1	12	21
tsrna-12311	GTTTATGTAGCTTACCTCCTCAAA	8	3	16	15
tsrna-12310	GTTTATGTAGCTTACCTCCTCAA	5	1	6	13
tsrna-12309	GTTTATGTAGCTTACCTCCTCA	12	0	7	12
tsrna-12308	GTTTATGTAGCTTACCTCCTC	9	0	5	6
tsrna-12307	GTTTATGTAGCTTACCTCCT	4	0	2	10
tsrna-12306	GTTTATGTAGCTTACCTCC	1	1	1	1
tsrna-12305	GTTTATGTAGCTTACCTC	4	0	4	3
tsrna-12304	GTTTATGTAGCTTACCT	3	0	1	1
tsrna-12302	GTTTAGTGGTAGAATTCTCGCCT	3	4	1	1
tsrna-12301	GTTTAGTGGTAGAATTCTCGCC	0	5	0	0
tsrna-12300	GTTTAGTGGTAGAATTCTCGC	0	0	0	0
tsrna-12297	GTTTAGTGGTAGAATTCT	0	0	0	0
tsrna-12295	GTTTAGACGGGCTCACATCACCCCATAAACACCA	76	11	57	15
tsrna-12294	GTTTAGACGGGCTCACATCACCCCATAAACACC	16	5	17	6
tsrna-12293	GTTTAGACGGGCTCACATCACCCCATAAACA	9	4	18	2
tsrna-12292	GTTTAGACGGGCTCACATCACCCCATAAAC	10	4	18	1
tsrna-12291	GTTTAGACGGGCTCACATCACCCCATAAA	14	7	19	0
tsrna-12290	GTTTAGACGGGCTCACATCACCCCATAA	8	5	11	0
tsrna-12289	GTTTAGACGGGCTCACATCACCCCATA	8	4	17	1
tsrna-12288	GTTTAGACGGGCTCACATCACCCCAT	8	4	16	2
tsrna-12287	GTTTAGACGGGCTCACATCACCCCA	9	2	14	2
tsrna-12286	GTTTAGACGGGCTCACATCACCCC	13	1	8	2
tsrna-12285	GTTTAGACGGGCTCACATCACCC	8	3	8	0
tsrna-12284	GTTTAGACGGGCTCACATCACC	8	4	8	0
tsrna-12283	GTTTAGACGGGCTCACATCAC	8	2	7	1
tsrna-12282	GTTTAGACGGGCTCACATCA	9	3	6	1
tsrna-12281	GTTTAGACGGGCTCACATC	4	1	6	0

tsrna-12280	GTTTAGACGGGCTCACAT	4	1	8	0
tsrna-12279	GTTTAGACGGGCTCACA	7	3	4	0
tsrna-12278	GTTTAGACGGGCTCAC	1	4	3	0
tsrna-12277	GTTTAAGTCCCATTGGTCTAGCCA	0	2	3	0
tsrna-12276	GTTTAAGTCCCATTGGTCTAGCC	0	1	0	0
tsrna-12275	GTTTAAGTCCCATTGGTCTAGC	0	0	0	0
tsrna-12272	GTTTAAGTCCCATTGGTCT	0	0	0	0
tsrna-12268	GTTTAACCAAAACATCAGATTGTGAATCTGACAACAGAGGCT	3	8	1	0
tsrna-12260	GTTTAAATTAGAATCTTAGCTTTGGGTGCT	13	5	9	2
tsrna-12259	GTTTAAATTAGAATCTTAGCTTTGGGTGC	9	1	5	0
tsrna-12258	GTTTAAATTAGAATCTTAGCTTTGGGTG	6	2	6	3
tsrna-12257	GTTTAAATTAGAATCTTAGCTTTGG	5	3	9	3
tsrna-12256	GTTTAAATTAGAATCTTAGCTTTG	7	0	2	0
tsrna-12255	GTTTAAATTAGAATCTTAGCTTT	2	0	4	1
tsrna-12254	GTTTAAATTAGAATCTTAGCT	3	3	1	0
tsrna-12253	GTTTAAATTAGAATCTTAGC	2	0	0	0
tsrna-12249	GTTGTGGCCGCAGCAACCTCGGT	0	2	0	1
tsrna-12246	GTTGTGGCCGCAGCAACCTC	0	0	0	1
tsrna-12244	GTTGTCTAGTGGCTAGGATTCGG	0	1	0	0
tsrna-12243	GTTGTAGTCCGTGCGAGAATACCA	1	2	0	1
tsrna-12242	GTTGTAGTCCGTGCGAGAATACC	1	1	0	0
tsrna-12241	GTTGTAGTCCGTGCGAGAATAC	0	0	0	0
tsrna-12240	GTTGTAGTCCGTGCGAGAATA	0	0	1	1
tsrna-12239	GTTGTAGTCCGTGCGAGAAT	0	0	0	0
tsrna-12238	GTTGTAGTCCGTGCGAGAA	0	0	0	0
tsrna-12234	GTTGGTTATACCCTTCCCGTACTACCA	4	0	0	1
tsrna-12233	GTTGGTTATACCCTTCCCGTACTACC	0	2	0	1
tsrna-12231	GTTGGTTATACCCTTCCCGTACTA	0	0	0	0
tsrna-12230	GTTGGTTATACCCTTCCCGTACT	1	0	0	0
tsrna-12220	GTTGGTGGTTCGAGCCCACCCAGGGACGCC	1	6	1	0
tsrna-12219	GTTGGTGGTATAGTGGTTAGCATAGCTGCC	0	2	0	0
tsrna-12218	GTTGGTGGTATAGTGGTTAGCATAGCTGC	2	0	0	0
tsrna-12217	GTTGGTGGTATAGTGGTTAGCATAGCTG	1	1	0	0
tsrna-12212	GTTGGTGGTATAGTGGTTAGCAT	0	0	0	0
tsrna-12208	GTTGGTGGTATAGTGGTGAGCATAGCTGCC	2	0	0	0
tsrna-12207	GTTGGTGGTATAGTGGTGAGCATAGCTGC	1	0	2	0
tsrna-12206	GTTGGTGGTATAGTGGTGAGCATAGCTG	0	2	0	1
tsrna-12205	GTTGGTGGTATAGTGGTGAGCATAGCT	0	2	0	0
tsrna-12204	GTTGGTGGTATAGTGGTGAGCATAGC	0	1	0	0
tsrna-12203	GTTGGTGGTATAGTGGTGAGCATAG	0	0	1	0
tsrna-12202	GTTGGTGGTATAGTGGTGAGCATA	0	0	0	0
tsrna-12201	GTTGGTGGTATAGTGGTGAGCAT	0	1	0	0
tsrna-12200	GTTGGTGGTATAGTGGTGAGCA	0	2	0	0
tsrna-12199	GTTGGTGGTATAGTGGTGAGC	0	0	1	0
tsrna-12197	GTTGGTGGTATAGTGGTGA	0	0	0	0
tsrna-12195	GTTGGTGGTATAGTGGTAAGCATAGCTGC	0	2	0	1
tsrna-12187	GTTGGTCTAGGGGTATGATTCTCGGTT	4	1	2	1
tsrna-12186	GTTGGTCTAGGGGTATGATTCTCGGT	7	5	2	3
tsrna-12185	GTTGGTCTAGGGGTATGATTCTCGCTTT	25	17	14	13
tsrna-12184	GTTGGTCTAGGGGTATGATTCTCGCTT	25	13	12	11
tsrna-12183	GTTGGTCTAGGGGTATGATTCTCGCT	18	20	10	30
tsrna-12182	GTTGGTCTAGGGGTATGATTCTCGC	13	9	5	12
tsrna-12181	GTTGGTCTAGGGGTATGATTCT	1	0	1	0
tsrna-12167	GTTGGGAGAGCGTTAGACTGAAGATC	0	2	0	0
tsrna-12166	GTTGGGAGAGCGTTAGACTGAAGAT	0	1	0	0
tsrna-12164	GTTGGGAGAGCGTTAGACTGAAG	0	0	0	1
tsrna-12159	GTTGGCTTGAAACCAGCTTTGGGG	0	0	0	0
tsrna-12157	GTTGCAATACTTAATTTCTGCCA	0	0	0	1
tsrna-12156	GTTGCAATACTTAATTTCTGCC	0	0	0	0
tsrna-12154	GTTGCAATACTTAATTTCTG	1	0	0	0
tsrna-12150	GTTGATTAGGGTGCTTAGCTGTTAACTAAGTGTTTGTGGGTTT	3	8	12	1
tsrna-12149	GTTGATTAGGGTGCTTAGCTGTTAACTAAGTGTTTG	5	3	5	0
tsrna-12148	GTTGATTAGGGTGCTTAGCTGTTAACTAAG	6	1	9	1
tsrna-12147	GTTGATTAGGGTGCTTAGCTGTTAACTA	3	1	10	0

tsrna-12146	GTTGATTAGGGTGCTTAGCTGTTAACT	5	1	8	2
tsrna-12145	GTTGATTAGGGTGCTTAGCTGTTAAC	0	1	2	0
tsrna-12144	GTTGATTAGGGTGCTTAGCTGTTAA	1	2	2	0
tsrna-12143	GTTGATTAGGGTGCTTAGCTGTTA	1	0	2	0
tsrna-12142	GTTGATTAGGGTGCTTAGCTGTT	0	0	0	0
tsrna-12141	GTTGATTAGGGTGCTTAGCTGT	0	2	1	0
tsrna-12140	GTTGATTAGGGTGCTTAGCTG	0	0	1	0
tsrna-12139	GTTGATTAGGGTGCTTAGCT	0	0	0	0
tsrna-12138	GTTGATTAGGGTGCTTAGC	0	1	1	0
tsrna-12134	GTTGATGCAGAGTGGGGTTTTGCAGTCCTT	1	2	0	1
tsrna-12133	GTTGATGCAGAGTGGGGTTTTGCAGTCCT	0	0	1	1
tsrna-12132	GTTGATGCAGAGTGGGGTTTTGCAGTCC	1	1	1	1
tsrna-12131	GTTGATGCAGAGTGGGGTTTTGCAGTC	0	0	3	0
tsrna-12130	GTTGATGCAGAGTGGGGTTTTGCAGT	0	0	0	0
tsrna-12129	GTTGATGCAGAGTGGGGTTTTGCAG	0	1	0	0
tsrna-12128	GTTGATGCAGAGTGGGGTTTTGCA	0	1	0	0
tsrna-12127	GTTGATGCAGAGTGGGGTTTTGC	0	0	1	0
tsrna-12126	GTTGATGCAGAGTGGGGTTTTG	1	0	0	0
tsrna-12125	GTTGATGCAGAGTGGGGTTTT	0	0	0	0
tsrna-12124	GTTGATGCAGAGTGGGGTTT	0	0	0	0
tsrna-12123	GTTGATGCAGAGTGGGGTT	0	0	0	0
tsrna-12122	GTTGATGCAGAGTGGGGT	0	0	0	0
tsrna-12119	GTTGACCCGGGTTCGATTCCCGGC	0	0	0	0
tsrna-12117	GTTGAAATACAACGATGGTTTTTCATATCATTGGTCGTGGTTG	0	2	1	1
tsrna-12116	GTTGAAATACAACGATGGTTTTTCATATCATTGGTC	0	1	1	1
tsrna-12115	GTTGAAATACAACGATGGTTTTTCATATC	0	0	1	0
tsrna-12113	GTTGAAATACAACGATGGTTTTTCA	1	0	0	0
tsrna-12110	GTTCTTGTAGTTGAAATACAACGATGGTTTTTCATATCATTGGT	4	28	3	3
tsrna-12109	GTTCTTGTAGTTGAAATACAACGATGGTTTTTCATATCA	11	16	6	7
tsrna-12108	GTTCTTGTAGTTGAAATACAACGATGGTTTTTCATATC	8	23	3	2
tsrna-12107	GTTCTTGTAGTTGAAATACAACGATGGTTTTTCATA	5	23	2	2
tsrna-12106	GTTCTTGTAGTTGAAATACAACGATGGTTTTTCAT	9	18	4	2
tsrna-12105	GTTCTTGTAGTTGAAATACAACGATGGTTTTTCA	6	17	6	4
tsrna-12104	GTTCTTGTAGTTGAAATACAACGATGGTTTTTC	4	22	3	0
tsrna-12103	GTTCTTGTAGTTGAAATACAACGATGGTTT	6	19	4	0
tsrna-12102	GTTCTTGTAGTTGAAATACAACGATGGTT	1	18	2	0
tsrna-12101	GTTCTTGTAGTTGAAATACAACGATGGT	5	21	4	0
tsrna-12100	GTTCTTGTAGTTGAAATACAACGATGG	1	10	1	1
tsrna-12099	GTTCTTGTAGTTGAAATACAACGATG	2	16	3	2
tsrna-12098	GTTCTTGTAGTTGAAATACAACGAT	2	15	0	3
tsrna-12097	GTTCTTGTAGTTGAAATACAACGA	3	14	1	2
tsrna-12096	GTTCTTGTAGTTGAAATACAACG	5	8	1	2
tsrna-12095	GTTCTTGTAGTTGAAATACAAC	5	3	0	2
tsrna-12094	GTTCTTGTAGTTGAAATACAA	2	1	1	0
tsrna-12093	GTTCTTGTAGTTGAAATACA	5	4	1	2
tsrna-12092	GTTCTTGTAGTTGAAATAC	2	1	0	1
tsrna-12091	GTTCTTGTAGTTGAAATA	3	1	1	0
tsrna-12090	GTTCTTGTAGTTGAAAT	0	0	0	0
tsrna-12088	GTTCTGGTCTCCGGATGGAGGCGTGGGTTC	10	34	6	0
tsrna-12087	GTTCTGGTCTCCGGATGGAGGCGT	1	1	0	0
tsrna-12082	GTTCTGGTCTCCAATGGAGGCGTG	1	0	0	0
tsrna-12081	GTTCGATTCTCATAGTCCTAGCCA	0	2	0	1
tsrna-12080	GTTCGATTCTCATAGTCCTAGCC	1	1	0	1
tsrna-12079	GTTCGATTCTCATAGTCCTAGC	0	1	1	0
tsrna-12078	GTTCGATTCTCATAGTCCTAG	1	1	0	0
tsrna-12077	GTTCGATTCTCATAGTCCTA	0	1	1	0
tsrna-12076	GTTCGATTCTCATAGTCCT	1	1	0	0
tsrna-12075	GTTCGATTCTCATAGTCC	0	0	0	1
tsrna-12072	GTTCGATTCCTTCCTTTTTTGCCA	0	0	1	1
tsrna-12071	GTTCGATTCCTTCCTTTTTTGCC	0	0	1	0
tsrna-12069	GTTCGATTCCTTCCTTTTTTG	0	1	0	0
tsrna-12068	GTTCGATTCCTTCCTTTTTT	0	1	0	0
tsrna-12063	GTTCGATTCCTGGTCAGGGAACCA	1	31	0	1
tsrna-12062	GTTCGATTCCTGGTCAGGG	1	0	0	0

tsrna-12061	GTTCGATTCCGGCTCGAAGGACCA	74	183	25	28
tsrna-12060	GTTCGATTCCGGCTCGAAGGACC	1	8	1	0
tsrna-12057	GTTCGATTCCGGCTCGAAGG	1	0	0	0
tsrna-12054	GTTCGATTCCCGGTCATGGAACCA	0	2	0	0
tsrna-12053	GTTCGATTCCCGGTCATGGAAC	0	1	0	0
tsrna-12051	GTTCGATTCCCGGTCAGGGAACCA	13	275	4	2
tsrna-12050	GTTCGATTCCCGGTCAGGGAACC	0	5	0	0
tsrna-12049	GTTCGATTCCCGGTCAGGGAAC	0	4	0	0
tsrna-12048	GTTCGATTCCCGGTCAGGGAA	0	3	0	0
tsrna-12047	GTTCGATTCCCGGTCAGGGA	0	3	0	0
tsrna-12045	GTTCGATTCCCGGTCAGGAAACCA	1	11	0	0
tsrna-12044	GTTCGATTCCCGGTCAGGA	0	2	0	0
tsrna-12040	GTTCGATTCCCGGGCGGCGCACCA	21	60	2	2
tsrna-12039	GTTCGATTCCCGGGCGGCGCACC	3	12	1	1
tsrna-12037	GTTCGATTCCCGGGCGGCGCA	0	0	0	0
tsrna-12031	GTTCGATTCCCGGCCCATGCACCA	22	37	9	14
tsrna-12030	GTTCGATTCCCGGCCCATGCACC	2	5	1	2
tsrna-12023	GTTCGATTCCCGGCCAGGGAACCA	0	11	0	0
tsrna-12022	GTTCGATTCCCGGCCAGGGAACC	0	1	0	0
tsrna-12017	GTTCGATTCCCGGCCAATGCACCA	37	124	13	7
tsrna-12016	GTTCGATTCCCGGCCAATGCACC	8	12	0	0
tsrna-12013	GTTCGATTCCCGGCCAATGC	0	0	0	0
tsrna-12010	GTTCGATTCCCGGCCAACGCACCA	41	98	18	13
tsrna-12009	GTTCGATTCCCGGCCAACGCACC	8	26	2	0
tsrna-12008	GTTCGATTCCCGGCCAACGCAC	0	1	0	0
tsrna-12007	GTTCGATTCCCGGCCAACGCA	0	1	0	0
tsrna-12005	GTTCGATTCCCGGCCAACG	0	1	0	0
tsrna-12001	GTTCGATTCCCCGACGGGGAGCCA	2	17	0	0
tsrna-12000	GTTCGATTCCCCGACGGGGAGCC	0	1	0	0
tsrna-11997	GTTCGATTCCCCGACGGGGA	0	1	0	0
tsrna-11984	GTTCGATCCTCGCTGGGGCCTCCA	6	12	0	4
tsrna-11983	GTTCGATCCTCGCTGGGGCCTC	0	0	0	0
tsrna-11978	GTTCGATCCTCACCTGGAGCACCA	1	3	0	0
tsrna-11977	GTTCGATCCTCACCTGGAGCACC	2	0	0	0
tsrna-11974	GTTCGATCCTCACCTGGAGC	0	2	0	0
tsrna-11971	GTTCGATCCTCACACGGGGCACCA	0	4	0	0
tsrna-11964	GTTCGATCCCGGGTTTCGGCACCA	26	97	14	16
tsrna-11963	GTTCGATCCCGGGTTTCGGCACC	5	5	1	1
tsrna-11962	GTTCGATCCCGGGTTTCGGCAC	0	1	0	0
tsrna-11955	GTTCGATCCCGGGCGGAAACACCA	28	40	7	10
tsrna-11953	GTTCGATCCCGGGCGGA	0	0	1	0
tsrna-11952	GTTCGATCCCCGTACTGGCCACCA	3	4	7	1
tsrna-11951	GTTCGATCCCCGTACGGGCCACCA	5	58	4	3
tsrna-11950	GTTCGATCCCCGTACGGGCCACC	0	4	0	0
tsrna-11944	GTTCGATCCCCGGCATCTCCACCA	236	381	134	73
tsrna-11943	GTTCGATCCCCGGCATCTCCACC	11	17	6	2
tsrna-11942	GTTCGATCCCCGGCATCTCCAC	0	0	0	0
tsrna-11940	GTTCGATCCCCGGCATCTCC	0	1	0	0
tsrna-11935	GTTCGATCCCCGGCACCTCCACCA	172	331	84	39
tsrna-11934	GTTCGATCCCCGGCACCTCCACC	13	12	4	0
tsrna-11933	GTTCGATCCCCGGCACCTCCAC	0	1	0	0
tsrna-11932	GTTCGATCCCCGGCACCTCCA	0	0	0	2
tsrna-11931	GTTCGATCCCCGGCACCTCC	0	1	0	0
tsrna-11930	GTTCGATCCCCGGCACCTC	1	1	0	0
tsrna-11929	GTTCGATCCCCGACACCTCCACCA	14	29	0	2
tsrna-11928	GTTCGATCCCCGACACCTCCACC	0	4	1	0
tsrna-11927	GTTCGATCCCCAGTACCTCCACCA	38	74	12	10
tsrna-11926	GTTCGATCCCCAGTACCTCCACC	2	5	0	0
tsrna-11925	GTTCGATCCCCAGTACCTCCAC	0	1	0	0
tsrna-11918	GTTCGATCCCCAGCATCTCCACCA	8	20	5	4
tsrna-11917	GTTCGATCCCCAGCATCTCCACC	0	1	0	0
tsrna-11910	GTTCGATCCCACCCAGGGACGCCA	1	6	0	0
tsrna-11908	GTTCGAGTCTCGGTGGAACCTCCA	40	136	11	24
tsrna-11907	GTTCGAGTCTCGGTGGAACCTCC	5	12	2	4

tsrna-11906	GTTCGAGTCTCGGTGGAACCTC	1	3	0	1
tsrna-11905	GTTCGAGTCTCGGTGGAACCT	0	1	0	0
tsrna-11902	GTTCGAGTCCTGCCGCGGTCGCCA	3	4	0	0
tsrna-11895	GTTCGAGTCCCTTCGTGGTCGCCA	0	2	0	0
tsrna-11887	GTTCGAGTCCCGGCGGAGTCGCCA	1	1	0	0
tsrna-11880	GTTCGAGTCCCATCTGGGTCGCCA	2	8	0	1
tsrna-11879	GTTCGAGTCCCATCTGGGTCGCC	0	2	0	0
tsrna-11875	GTTCGAGTCCCATCTGGGGTGCCA	1	8	0	0
tsrna-11874	GTTCGAGTCCCATCTGGGGTGCC	0	1	0	0
tsrna-11867	GTTCGAGTCCCACCTGGGGTGCCA	1	5	0	0
tsrna-11865	GTTCGAGTCCCACCTGGGGTACCA	0	4	0	0
tsrna-11859	GTTCGAGTCCCACCCGGGGTACCA	0	1	0	0
tsrna-11855	GTTCGAGTCCCACCAGAGTCGCCA	0	3	1	0
tsrna-11851	GTTCGAGCCTCAGAGAGGGCACCA	0	0	0	0
tsrna-11849	GTTCGAGCCTCACCTGGAGCACC	1	0	0	0
tsrna-11846	GTTCGAGCCTCACCTGGAGC	0	1	0	0
tsrna-11835	GTTCGAGCCCCAGTGGAACCACCA	30	25	30	19
tsrna-11834	GTTCGAGCCCCAGTGGAACCACC	1	2	0	0
tsrna-11833	GTTCGAGCCCCAGTGGAACCAC	0	0	1	0
tsrna-11832	GTTCGAGCCCCAGTGGAACCA	1	0	0	1
tsrna-11831	GTTCGAGCCCCAGTGGAACC	0	1	0	0
tsrna-11829	GTTCGAGCCCCAGTGGAA	0	0	1	0
tsrna-11826	GTTCGAGCCCCACGTTGGGCGCCA	10	191	3	5
tsrna-11825	GTTCGAGCCCCACGTTGGGCGCC	2	10	0	0
tsrna-11818	GTTCGAGCCCACCCAGGGACGCCA	6	94	1	0
tsrna-11817	GTTCGAGCCCACCCAGGGACGCC	2	10	0	0
tsrna-11816	GTTCGAGCCCACCCAGGGACGC	0	1	0	0
tsrna-11809	GTTCGACTCCTGGCTGGCTCGCCA	46	96	13	6
tsrna-11808	GTTCGACTCCTGGCTGGCTCGCC	2	7	1	0
tsrna-11807	GTTCGACTCCTGGCTGGCTCGC	0	1	0	0
tsrna-11804	GTTCGACTCCTGGCTGGCT	0	0	0	0
tsrna-11801	GTTCGACTCCCGGTGTGGGAACCA	66	292	33	16
tsrna-11800	GTTCGACTCCCGGTGTGGGAACC	2	8	0	1
tsrna-11799	GTTCGACTCCCGGTGTGGGAAC	0	2	0	0
tsrna-11795	GTTCGACTCCCGGTGTGG	0	1	0	0
tsrna-11794	GTTCGACTCCCGGTGTG	1	0	0	0
tsrna-11792	GTTCGACTCCCGGTATGGGAACCA	8	67	4	4
tsrna-11791	GTTCGACTCCCGGTATGGGAACC	1	4	0	0
tsrna-11790	GTTCGACTCCCGGTATGGGAAC	1	4	0	0
tsrna-11788	GTTCGACTCCCGGTATGGGA	0	1	0	0
tsrna-11783	GTTCGACTCCCAGCGGGGCCTCCA	3	0	0	0
tsrna-11782	GTTCGACTCCCAGCGGGGCCTCC	0	1	0	0
tsrna-11781	GTTCGACTCCCAGCGGGGC	0	0	1	0
tsrna-11780	GTTCGACTCCCAGCGGGG	0	2	0	0
tsrna-11779	GTTCGACTCCCAGCGG	0	2	1	0
tsrna-11778	GTTCGACCCCCGGCTCCTCCACCA	21	15	5	4
tsrna-11774	GTTCGAATCTCGGTGGGACCTCCA	60	107	31	16
tsrna-11772	GTTCGAATCCTGTTCGTGACGCCA	6	9	3	5
tsrna-11771	GTTCGAATCCTGTTCGTGACG	0	1	0	0
tsrna-11770	GTTCGAATCCTGTCGGCTACGCCA	7	16	5	10
tsrna-11767	GTTCGAATCCTGCTCACAGCGCCA	11	19	3	1
tsrna-11766	GTTCGAATCCTGCTCACAGCGCC	2	1	0	0
tsrna-11763	GTTCGAATCCTGCTCACAGC	0	0	0	0
tsrna-11762	GTTCGAATCCTGCCGACTACGCCA	100	232	27	22
tsrna-11761	GTTCGAATCCTGCCGACTACGCC	7	13	3	3
tsrna-11754	GTTCGAATCCGGCTCGGAGGACCA	2	2	0	0
tsrna-11749	GTTCGAATCCGGCTCGAAGGACCA	23	36	13	5
tsrna-11748	GTTCGAATCCGGCTCGAAGGACC	2	7	1	1
tsrna-11742	GTTCGAATCCGAGTCACGGCACCA	0	1	0	0
tsrna-11733	GTTCGAATCCCTTCGTGGTTGCCA	0	11	1	0
tsrna-11730	GTTCGAATCCCTTCGTGGTTG	0	1	0	0
tsrna-11728	GTTCGAATCCCTCCGTGGTTACCA	3	4	2	0
tsrna-11725	GTTCGAATCCCATCCTCGTCGCCA	7	13	6	1
tsrna-11724	GTTCGAATCCCATCCTCGTCGCC	1	0	0	0

tsrna-11717	GTTCGAATCCCAGTAGAGCCTCCA	21	41	14	10
tsrna-11716	GTTCGAATCCCAGCGGTGCCTCCA	25	60	13	3
tsrna-11715	GTTCGAATCCCAGCGGTGCCTCC	0	2	0	0
tsrna-11709	GTTCGAATCCCAGCGGGGCCTCCA	0	4	0	0
tsrna-11700	GTTCGAATCCCAGCGAGGCCTCCA	0	2	0	0
tsrna-11699	GTTCGAATCCCACTTCTGACACCA	15	36	12	8
tsrna-11698	GTTCGAATCCCACTTCTGACACC	1	6	0	1
tsrna-11697	GTTCGAATCCCACTTCTGACAC	0	0	0	0
tsrna-11691	GTTCGAATCCCACTCCTGACACCA	106	108	53	25
tsrna-11690	GTTCGAATCCCACTCCTGACACC	8	10	3	0
tsrna-11689	GTTCGAATCCCACTCCTGACAC	1	0	2	1
tsrna-11688	GTTCGAATCCCACTCCTGACA	0	0	0	0
tsrna-11687	GTTCGAATCCCACTCCTGAC	1	0	0	0
tsrna-11682	GTTCGAATCCCACCTTCGTCGCCA	1	5	0	0
tsrna-11681	GTTCGAATCCCACCGCTGCCACCA	9	37	5	3
tsrna-11680	GTTCGAATCCCACCGCTGCCACC	0	2	0	0
tsrna-11679	GTTCGAATCCCACCGCTGCCAC	0	0	0	0
tsrna-11677	GTTCGAATCCCACCGCTGCC	0	0	0	2
tsrna-11672	GTTCGAATCCCACCCTCGTCGCCA	6	13	1	0
tsrna-11670	GTTCGAATCCCACCAGAGTCGCCA	1	6	0	1
tsrna-11668	GTTCGAATCCCACCACTGCCACCA	4	25	1	0
tsrna-11667	GTTCGAATCACGTCGGGGTCACCA	2	7	2	1
tsrna-11666	GTTCGAATCACGTCGGGGTCACC	0	0	0	0
tsrna-11658	GTTCGAACCTCAGAGGGGGCACCA	2	7	0	0
tsrna-11653	GTTCGAACCCTGCTCGCTGCGCCA	11	27	3	4
tsrna-11652	GTTCGAACCCTGCTCGCTGCGCC	0	3	1	0
tsrna-11646	GTTCGAACCCCGTCCGTGCCTCCA	4	9	2	1
tsrna-11645	GTTCGAACCCCGTCCGTGCCTCC	0	1	0	0
tsrna-11644	GTTCGAACCCCGTCCGTGCCTC	0	0	0	0
tsrna-11638	GTTCGAACCCCGTACGGGCCACCA	1	3	3	0
tsrna-11637	GTTCGAACCCCACTCCTGGTACCA	0	1	0	0
tsrna-11630	GTTCGAAACCGGGCGGAAACACCA	308	709	112	45
tsrna-11629	GTTCGAAACCGGGCGGAAACACC	66	188	29	4
tsrna-11628	GTTCGAAACCGGGCGGAAACAC	2	0	1	0
tsrna-11627	GTTCGAAACCGGGCGGAAACA	0	1	0	0
tsrna-11626	GTTCGAAACCGGGCGGAAAC	0	1	0	0
tsrna-11622	GTTCGAAACCGGGCAGAAGCACCA	34	136	18	3
tsrna-11617	GTTCCATGGTGTAATGGTTAGCACTCTGGACTCTGA	0	2	0	2
tsrna-11616	GTTCCATGGTGTAATGGTTAGCACTCTGGACT	1	2	0	4
tsrna-11615	GTTCCATGGTGTAATGGTTAGCACTCTGGA	1	1	0	1
tsrna-11614	GTTCCATGGTGTAATGGTTAGCACTCTGG	0	1	1	0
tsrna-11610	GTTCCATGGTGTAATGGTTAGC	0	0	0	0
tsrna-11607	GTTCCATGGTGTAATGGTGAGCACTCTG	1	0	0	0
tsrna-11605	GTTCCATGGTGTAATGGT	0	1	0	0
tsrna-11603	GTTCCATAGTGTAGTGGTTATCACGTCTGCTTT	3	0	3	0
tsrna-11602	GTTCCATAGTGTAGTGGTTATCACGTCTGCTT	2	0	2	1
tsrna-11601	GTTCCATAGTGTAGTGGTTATCACGTCTGCT	1	1	0	0
tsrna-11600	GTTCCATAGTGTAGTGGTTATCACGTCTGC	2	1	1	0
tsrna-11599	GTTCCATAGTGTAGTGGTTATCACGTCTG	1	0	0	0
tsrna-11597	GTTCCATAGTGTAGTGGTTATCACGTC	1	0	0	0
tsrna-11594	GTTCCATAGTGTAGTGGTTATCACATCTGC	1	0	0	1
tsrna-11592	GTTCCATAGTGTAGTGGTTATCAC	3	0	1	0
tsrna-11587	GTTCCATAGTGTAGTGG	0	0	0	0
tsrna-11585	GTTCCATAGTGTAGCGGTTATCACGTCTG	0	1	0	0
tsrna-11583	GTTCCATAGTGTAGCGGTTATCACGTC	0	0	1	0
tsrna-11582	GTTCCATAGTGTAGCGGTTATC	0	1	1	0
tsrna-11581	GTTCCATAGTGTAGCGGTT	0	1	0	0
tsrna-11579	GTTCCATAGTGTAGCGG	0	0	0	0
tsrna-11578	GTTCCATAGTGTAGCG	0	0	0	0
tsrna-11576	GTTCAGTTGATGCAGAGTGGGGTTTTG	0	0	0	0
tsrna-11575	GTTCAGTTGATGCAGAGTGGGGTTTT	1	0	0	0
tsrna-11572	GTTCAGTGGTAGAATTCTTGCCTG	0	1	0	0
tsrna-11570	GTTCAGTGGTAGAATTCTTGCC	0	1	0	0
tsrna-11569	GTTCAGTGGTAGAATTCTTGC	1	0	0	0

tsrna-11568	GTTCAGTGGTAGAATTCTT	0	0	0	0
tsrna-11567	GTTCAGTGGTAGAATTCTCGCCTT	6	7	2	0
tsrna-11566	GTTCAGTGGTAGAATTCTCGCCTGCCACGC	54	95	35	24
tsrna-11565	GTTCAGTGGTAGAATTCTCGCCTGCCA	20	44	13	2
tsrna-11564	GTTCAGTGGTAGAATTCTCGCCTGCC	18	39	5	3
tsrna-11563	GTTCAGTGGTAGAATTCTCGCCTGC	3	8	9	2
tsrna-11562	GTTCAGTGGTAGAATTCTCGCCTG	5	7	2	1
tsrna-11561	GTTCAGTGGTAGAATTCTCGCCTCCCACGCGGGAGACCCGG(	5	15	10	10
tsrna-11560	GTTCAGTGGTAGAATTCTCGCCTCCC	5	14	3	9
tsrna-11559	GTTCAGTGGTAGAATTCTCGCCTCC	3	15	1	10
tsrna-11558	GTTCAGTGGTAGAATTCTCGCCTC	3	5	0	2
tsrna-11557	GTTCAGTGGTAGAATTCTCGCCT	4	4	0	2
tsrna-11556	GTTCAGTGGTAGAATTCTCGCC	1	5	2	0
tsrna-11555	GTTCAGTGGTAGAATTCTCGC	1	2	0	0
tsrna-11554	GTTCAGTGGTAGAATTCTCG	2	0	0	0
tsrna-11553	GTTCAGTGGTAGAATTCTC	0	0	0	0
tsrna-11552	GTTCAGTGGTAGAATTCT	0	1	0	0
tsrna-11549	GTTCAGGTCGCAGTCTCCCCTGGAGGCGTG	0	13	0	0
tsrna-11547	GTTCAGGTCGCAGTCTCCCCTGG	0	1	0	0
tsrna-11539	GTTCAATTCTCGCTGGGGCCTC	0	0	0	0
tsrna-11538	GTTCAATTCCTCTTCTTAACACCA	0	1	1	0
tsrna-11537	GTTCAATTCCTCTTCTTAACACC	1	1	0	0
tsrna-11534	GTTCAATTCCCGGTCAGGGAACCA	1	18	1	0
tsrna-11533	GTTCAATTCCCGGTCAGGGAACC	1	1	0	0
tsrna-11532	GTTCAATTCCCGGTCAGGGAAC	0	1	0	0
tsrna-11531	GTTCAATTCCCCGACGGGGAGCCA	0	4	0	0
tsrna-11522	GTTCAATGGTAGAATTCTCGCCT	0	3	0	1
tsrna-11521	GTTCAATGGTAGAATTCTCGCC	0	2	0	0
tsrna-11520	GTTCAATCCCGGGTTTCGGCACCA	35	103	7	10
tsrna-11518	GTTCAATCCCCGGCATCTCCACCA	106	172	90	49
tsrna-11517	GTTCAATCCCCGGCATCTCCACC	3	10	2	3
tsrna-11516	GTTCAATCCCCGGCATCTCCAC	0	2	0	0
tsrna-11515	GTTCAATCCCCGGCATCTCCA	0	0	0	1
tsrna-11511	GTTCAATCCCCGGCACCTCCACCA	223	286	127	54
tsrna-11510	GTTCAATCCCCGGCACCTCCACC	9	12	4	1
tsrna-11509	GTTCAATCCCCGGCACCTCCAC	0	1	0	0
tsrna-11507	GTTCAATCCCCGGCACCTCC	0	0	0	0
tsrna-11503	GTTCAATCCCCAGCACCTCCACCA	7	9	1	2
tsrna-11502	GTTCAAGTCTCGGTGGAACCTCCA	55	125	22	19
tsrna-11501	GTTCAAGTCTCGGTGGAACCTCC	7	10	0	0
tsrna-11500	GTTCAAGTCTCGGTGGAACCTC	0	1	0	0
tsrna-11499	GTTCAAGTCTCGGTGGAACCT	0	1	0	0
tsrna-11495	GTTCAAGTCCCTGTTCGGGCGCCA	8	8	3	3
tsrna-11494	GTTCAAGTCCCTGTTCGGGCGCC	0	1	0	0
tsrna-11487	GTTCAAGTCCCTGTTCAGGCGCCA	2	1	1	0
tsrna-11486	GTTCAAGTCACGTCGGGGTCACCA	4	4	0	3
tsrna-11485	GTTCAAGTCACGTCGGGGTCACC	0	0	2	0
tsrna-11477	GTTCAAATCTCGGTGGGACCTCCA	54	108	33	22
tsrna-11476	GTTCAAATCTCGGTGGGACCTCC	6	3	3	2
tsrna-11475	GTTCAAATCTCGGTGGGACCTC	0	1	0	0
tsrna-11474	GTTCAAATCTCGGTGGGACC	0	0	0	0
tsrna-11473	GTTCAAATCTCGGTGGGAC	0	0	0	0
tsrna-11472	GTTCAAATCTCGGTGGGA	0	1	0	0
tsrna-11470	GTTCAAATCTCGGTGGAACCTCCA	163	229	46	26
tsrna-11469	GTTCAAATCTCGGTGGAACCTCC	3	10	1	0
tsrna-11468	GTTCAAATCTCGGTGGAACCTC	0	6	0	0
tsrna-11467	GTTCAAATCTCGGTGGAACCT	1	1	1	0
tsrna-11466	GTTCAAATCTCGGTGGAACC	0	0	0	0
tsrna-11461	GTTCAAATCTCGCTGGGGCCTCCA	40	53	15	15
tsrna-11460	GTTCAAATCTCGCTGGGGCCTCC	4	3	1	2
tsrna-11459	GTTCAAATCTCGCTGGGGCCTC	0	0	0	0
tsrna-11458	GTTCAAATCTCGCTGGGGCCT	0	1	0	0
tsrna-11456	GTTCAAATCTCGCTGGGGC	0	0	0	0
tsrna-11454	GTTCAAATCCGGGTGCCCCCTCCA	136	167	77	21

tsrna-11453	GTTCAAATCCGGGTGCCCCCTCC	7	11	1	1
tsrna-11452	GTTCAAATCCGGGTGCCCCCTC	0	2	0	0
tsrna-11451	GTTCAAATCCGGGTGCCCCCT	0	1	0	0
tsrna-11445	GTTCAAATCCCGGACGAGCCCCCA	118	186	46	50
tsrna-11444	GTTCAAATCCCGGACGAGCCCCC	6	17	4	2
tsrna-11443	GTTCAAATCCCGGACGAGCCCC	2	1	0	0
tsrna-11442	GTTCAAATCCCGGACGAGCCC	1	0	0	0
tsrna-11441	GTTCAAATCCCGGACGAGCC	2	0	0	0
tsrna-11436	GTTCAAATCCAGGTGCCCCCTCCA	1	1	2	2
tsrna-11434	GTTCAAATCACGTCGGGGTCACCA	11	18	3	3
tsrna-11433	GTTCAAATCACGTCGGGGTCACC	0	0	2	0
tsrna-11425	GTTATCACGTTCGCCTCACACG	0	1	0	0
tsrna-11421	GTTATCACGTCTGCTTTACACGC	0	0	1	0
tsrna-11413	GTTATACCCTTCCCGTACTACCA	1	3	1	0
tsrna-11407	GTTATACCCTTCCCGTA	0	0	0	0
tsrna-11405	GTTAGTATCCCCGCCTGTC	1	0	0	0
tsrna-11404	GTTAGTATCCCCGCCTGT	2	0	0	0
tsrna-11402	GTTAGTATAGTGGTTAGTATCCCCGCC	2	2	0	0
tsrna-11396	GTTAGTATAGTGGTGAGTATCCCCGCC	1	4	1	2
tsrna-11395	GTTAGTATAGTGGTGAGTATCCCCGC	2	3	0	0
tsrna-11394	GTTAGTATAGTGGTGAGTATCCCCG	2	0	1	0
tsrna-11393	GTTAGTATAGTGGTGAGTATCCCC	0	0	0	0
tsrna-11385	GTTAGTACTCTGCGTTGTGGCCGCAGCAACCT	2	1	1	1
tsrna-11384	GTTAGTACTCTGCGTTGTGGCCGCAGC	4	2	2	0
tsrna-11383	GTTAGTACTCTGCGTTGTGGCCGCA	3	0	2	0
tsrna-11382	GTTAGTACTCTGCGTTGTGGCCGC	1	0	0	1
tsrna-11381	GTTAGTACTCTGCGTTGTGGCCG	2	0	1	0
tsrna-11380	GTTAGTACTCTGCGTTGTGGCC	5	1	0	0
tsrna-11379	GTTAGTACTCTGCGTTGTGGC	0	1	4	0
tsrna-11378	GTTAGTACTCTGCGTTGTGG	1	0	0	0
tsrna-11377	GTTAGTACTCTGCGTTGTG	2	2	0	0
tsrna-11376	GTTAGTACTCTGCGTTGT	0	0	0	0
tsrna-11373	GTTAGGCCTCTTTTTACCACCA	2	3	2	1
tsrna-11372	GTTAGGCCTCTTTTTACCACC	0	1	1	0
tsrna-11371	GTTAGGCCTCTTTTTACCAC	0	0	0	0
tsrna-11370	GTTAGGCCTCTTTTTACCA	0	0	0	1
tsrna-11369	GTTAGGCCTCTTTTTACC	0	0	1	0
tsrna-11368	GTTAGGCCTCTTTTTAC	1	0	0	0
tsrna-11367	GTTAGGCCTCTTTTTA	0	0	0	0
tsrna-11366	GTTAGGATTCGGCGCTCTCAT	2	0	0	0
tsrna-11365	GTTAGGATTCGGCGCTCTCACCGCCGCGGCCCGGGTTC	5	21	4	5
tsrna-11364	GTTAGGATTCGGCGCTCTCACCGCCGCGGCCCGGGTT	6	11	2	4
tsrna-11363	GTTAGGATTCGGCGCTCTCACCGCCGCGGCCCGGGT	3	14	2	4
tsrna-11362	GTTAGGATTCGGCGCTCTCACCGCCGCGGCCCGGG	4	20	2	5
tsrna-11361	GTTAGGATTCGGCGCTCTCACCGCCGCGGCCC	4	25	2	3
tsrna-11360	GTTAGGATTCGGCGCTCTCACCGCCGCGGCC	4	12	6	5
tsrna-11359	GTTAGGATTCGGCGCTCTCACCGCCGCGGC	7	18	2	3
tsrna-11358	GTTAGGATTCGGCGCTCTCACCGCC	6	17	3	1
tsrna-11357	GTTAGGATTCGGCGCTCTCACCGC	1	6	3	1
tsrna-11356	GTTAGGATTCGGCGCTCTCACCG	1	5	2	1
tsrna-11355	GTTAGGATTCGGCGCTCTCACC	3	2	1	0
tsrna-11354	GTTAGGATTCGGCGCTCTCAC	1	2	1	0
tsrna-11353	GTTAGGATTCGGCGCTCTCA	0	0	1	0
tsrna-11352	GTTAGGATTCGGCGCTCTC	0	1	0	0
tsrna-11351	GTTAGGATTCGGCGCTCT	0	1	0	0
tsrna-11350	GTTAGGATTCGGCGCTC	1	3	0	1
tsrna-11349	GTTAGGATTCGGCGCT	0	0	0	0
tsrna-11348	GTTAGGATTCCTGGTTTTCACC	6	6	0	1
tsrna-11347	GTTAGGATTCCTGGTTTTCA	1	1	1	0
tsrna-11346	GTTAGGATTCCTGGTTTTC	1	1	4	0
tsrna-11339	GTTAGCGCGTTCGGCTGTTAACC	0	7	1	0
tsrna-11336	GTTAGCGCGTTCGGCTGTTA	0	0	1	0
tsrna-11327	GTTAGCACTCTGGACTTTGAATCCAGCGATCCGAGT	5	4	2	4
tsrna-11326	GTTAGCACTCTGGACTTTGAATCCAGCG	10	7	1	0

tsrna-11325	GTTAGCACTCTGGACTTTGAATCCAGC	0	5	2	1
tsrna-11324	GTTAGCACTCTGGACTTTGAATCCA	5	8	3	0
tsrna-11323	GTTAGCACTCTGGACTTTGAATCC	1	5	0	0
tsrna-11322	GTTAGCACTCTGGACTTTGAATC	2	4	1	1
tsrna-11315	GTTAGCACTCTGGACTCTGAATCCAGCGATCCGAGTTCA	1	3	0	2
tsrna-11314	GTTAGCACTCTGGACTCTGAATCCAGCGATCCGAGTTC	4	5	2	1
tsrna-11313	GTTAGCACTCTGGACTCTGAATCCAGCGATCCGAGT	1	3	1	0
tsrna-11312	GTTAGCACTCTGGACTCTGAATCCAGCGATCCGAG	5	3	4	0
tsrna-11311	GTTAGCACTCTGGACTCTGAATCCAGCGA	1	4	1	0
tsrna-11310	GTTAGCACTCTGGACTCTGAATCCAGCG	0	5	1	2
tsrna-11309	GTTAGCACTCTGGACTCTGAATCCAGC	0	3	1	0
tsrna-11308	GTTAGCACTCTGGACTCTGAATCCAG	0	5	1	0
tsrna-11307	GTTAGCACTCTGGACTCTGAATCCA	0	0	1	1
tsrna-11306	GTTAGCACTCTGGACTCTGAATCC	0	3	2	0
tsrna-11305	GTTAGCACTCTGGACTCTGAATC	1	0	0	0
tsrna-11303	GTTAGCACTCTGGACTCTGAA	0	0	0	0
tsrna-11288	GTTAAGATGGCAGAGCCTGGTAAT	1	1	0	0
tsrna-11281	GTTAAGATGGCAGAGCCCGGTAATCGCATAAAACTTAAAACTT	34	5	56	5
tsrna-11280	GTTAAGATGGCAGAGCCCGGTAATCGCATAAAACTTAAAACTT	12	3	44	4
tsrna-11279	GTTAAGATGGCAGAGCCCGGTAATCGCATAAAACTTAAAACTT	13	3	40	2
tsrna-11278	GTTAAGATGGCAGAGCCCGGTAATCGCATAAAACTTAAAACTT	17	6	45	1
tsrna-11277	GTTAAGATGGCAGAGCCCGGTAATCGCATAAAACTTAAAACTT	7	3	9	0
tsrna-11276	GTTAAGATGGCAGAGCCCGGTAATCGCATAAAACTTAAAACT	3	1	4	1
tsrna-11275	GTTAAGATGGCAGAGCCCGGTAATCGCATAAAACTTAAAAC	0	0	0	0
tsrna-11274	GTTAAGATGGCAGAGCCCGGTAATCGCATAAAACTTAAAA	0	1	0	0
tsrna-11273	GTTAAGATGGCAGAGCCCGGTAATCGCATAAAACTTAAA	1	0	3	1
tsrna-11272	GTTAAGATGGCAGAGCCCGGTAATCGCATAAAACTTAA	1	0	1	0
tsrna-11271	GTTAAGATGGCAGAGCCCGGTAATCGCATAAAACTTA	0	2	0	0
tsrna-11270	GTTAAGATGGCAGAGCCCGGTAATCGCATAAAACTT	0	2	1	0
tsrna-11269	GTTAAGATGGCAGAGCCCGGTAATCGCATAAAACT	1	2	0	1
tsrna-11268	GTTAAGATGGCAGAGCCCGGTAATCGCATAAAAC	0	0	2	0
tsrna-11267	GTTAAGATGGCAGAGCCCGGTAATCGCATAAAA	0	0	1	0
tsrna-11266	GTTAAGATGGCAGAGCCCGGTAATCGCATAAA	0	1	3	0
tsrna-11265	GTTAAGATGGCAGAGCCCGGTAATCGCATAA	0	0	0	0
tsrna-11264	GTTAAGATGGCAGAGCCCGGTAATCGCATA	0	0	0	0
tsrna-11263	GTTAAGATGGCAGAGCCCGGTAATCGCAT	0	1	0	0
tsrna-11261	GTTAAGATGGCAGAGCCCGGTAATCGC	1	0	0	1
tsrna-11258	GTTAAGATGGCAGAGCCCGGTAAT	0	1	0	0
tsrna-11257	GTTAAGATGGCAGAGCCCGGTAA	0	1	0	0
tsrna-11251	GTTAAGATGGCAGAGCCCGGC	0	1	0	0
tsrna-11236	GTTAACTTCCAATTAACTAGT	1	0	0	0
tsrna-11230	GTTAACTAAGTGTTTGTGGGTTTAAGTCCCATTGGTCTAGCCA	5	0	3	1
tsrna-11229	GTTAACTAAGTGTTTGTGGGTTTAAGTCCCATTGGTCTAGCC	2	1	3	1
tsrna-11228	GTTAACTAAGTGTTTGTGGGTTTAAGTCCCATTGGT	2	1	5	0
tsrna-11227	GTTAACTAAGTGTTTGTGGGTTTAAGTCCC	1	0	0	1
tsrna-11226	GTTAACTAAGTGTTTGTGGGTTTAAGTCC	4	0	0	0
tsrna-11225	GTTAACTAAGTGTTTGTGGGTTTAAGTC	1	1	1	1
tsrna-11224	GTTAACTAAGTGTTTGTGGGTTTAAGT	2	0	0	0
tsrna-11223	GTTAACTAAGTGTTTGTGGGTTTAAG	0	0	1	0
tsrna-11222	GTTAACTAAGTGTTTGTGGGTTTAA	0	0	1	0
tsrna-11221	GTTAACTAAGTGTTTGTGGGTTTA	0	0	3	0
tsrna-11220	GTTAACTAAGTGTTTGTGGGTTT	0	0	1	0
tsrna-11219	GTTAACTAAGTGTTTGTGGGTT	1	0	0	0
tsrna-11218	GTTAACTAAGTGTTTGTGGGT	0	0	1	0
tsrna-11212	GTTAACCGAAAGGTTGGTGGTTCGAGCCCA	1	7	0	0
tsrna-11211	GTTAACCGAAAGGTTGGTGGTTCGAGCCC	1	3	0	0
tsrna-11210	GTTAACCGAAAGGTTGGTGGTTC	1	2	0	0
tsrna-11209	GTTAACCGAAAGGTTGGTGGTT	1	2	0	0
tsrna-11208	GTTAACCGAAAGGTTGGTGGT	1	1	0	0
tsrna-11207	GTTAACCGAAAGGTTGGTGG	0	1	0	0
tsrna-11203	GTTAAATTATAGGCTAAATCCTATATATC	1	1	0	0
tsrna-11200	GTTAAATTATAGGCTAAATCCTATA	0	1	0	0
tsrna-11199	GTTAAATTATAGGCTAAATCCTAT	0	2	0	0
tsrna-11191	GTTAAATTATAGGCTA	0	0	1	0

tsrna-11190	GTTAAATACAGACCAAGAGCCTTCAAAGCC	17	11	12	7
tsrna-11189	GTTAAATACAGACCAAGAGCCTTCA	11	0	11	1
tsrna-11188	GTTAAATACAGACCAAGAGCCTTC	12	1	11	3
tsrna-11187	GTTAAATACAGACCAAGAGCC	0	1	1	0
tsrna-11184	GTTAAATACAGACCAAGA	1	0	0	0
tsrna-11182	GTTAAATACAGACCAA	0	0	0	0
tsrna-11181	GTTAAAGATTAAGAGAACCAACACCTCTTTACAGTGACCA	7	1	4	1
tsrna-11180	GTTAAAGATTAAGAGAACCAACACCTCTTTACAGTGACC	4	0	3	1
tsrna-11179	GTTAAAGATTAAGAGAACCAACACCTCTTTACAGTGAC	1	0	2	1
tsrna-11178	GTTAAAGATTAAGAGAACCAACACCTCTTTACAGTGA	1	0	4	0
tsrna-11177	GTTAAAGATTAAGAGAACCAACACCTCTTT	1	0	1	2
tsrna-11176	GTTAAAGATTAAGAGAACCAACACCTCTT	0	0	0	0
tsrna-11175	GTTAAAGATTAAGAGAACCAACACCTCT	2	1	0	2
tsrna-11174	GTTAAAGATTAAGAGAACCAACACCT	0	0	0	1
tsrna-11173	GTTAAAGATTAAGAGAACCAACACC	0	0	0	0
tsrna-11172	GTTAAAGATTAAGAGAACCAACAC	0	0	0	0
tsrna-11171	GTTAAAGATTAAGAGAACCAACA	0	0	1	0
tsrna-11170	GTTAAAGATTAAGAGAACCAAC	1	0	0	0
tsrna-11169	GTTAAAGATTAAGAGAACCAA	0	0	0	0
tsrna-11167	GTTAAAGATTAAGAGAACC	0	0	0	0
tsrna-11163	GTTAAAGACTTTTTCTCTGACCA	23	35	12	15
tsrna-11162	GTTAAAGACTTTTTCTCTGACC	4	9	0	0
tsrna-11161	GTTAAAGACTTTTTCTCTGAC	1	1	0	0
tsrna-11160	GTTAAAGACTTTTTCTCTGA	0	0	1	0
tsrna-11158	GTTAAAGACTTTTTCTCT	1	0	0	0
tsrna-11155	GTGTTTGTGGGTTTAAGTCCCATTGGTCTAGCCA	0	1	0	1
tsrna-11154	GTGTTTGTGGGTTTAAGTCCCATTGGTCTAGCC	2	1	1	0
tsrna-11153	GTGTTTGTGGGTTTAAGTCCCATTGGTCTAGC	1	0	0	0
tsrna-11152	GTGTTTGTGGGTTTAAGTCCCATTGGTCT	0	0	1	0
tsrna-11150	GTGTTTGTGGGTTTAAGTCCCATTG	0	3	1	0
tsrna-11149	GTGTTTGTGGGTTTAAGTCCCATT	0	1	0	1
tsrna-11140	GTGTTCGAATCACGTCGGGGTCACCA	2	8	4	2
tsrna-11133	GTGTTCAAGTCACGTCGGGGTCACCA	6	6	1	2
tsrna-11129	GTGTGATAGGTGGCACGGAGAATTTTG	2	3	2	0
tsrna-11128	GTGTGATAGGTGGCACGGAGA	0	0	0	0
tsrna-11124	GTGTGAGGTCCCGGGTTC	0	3	0	0
tsrna-11123	GTGTGAGGTCCCGGGTT	3	0	0	1
tsrna-11122	GTGTGAGGTCCCGGGT	1	3	2	0
tsrna-11121	GTGTGAGGCCCCGGGT	0	3	0	0
tsrna-11120	GTGTAGTGGTTATCACGTTCGCCTCA	1	0	0	0
tsrna-11118	GTGTAGTGGTTATCACGTTCGCCT	0	1	0	0
tsrna-11117	GTGTAGTGGTTATCACGTTCGCC	0	0	2	0
tsrna-11103	GTGTAGTGGTCATCACGTTCGCCT	0	1	0	0
tsrna-11097	GTGTAGTGGTATCATGCAAGATTCC	1	0	0	0
tsrna-11091	GTGTAGCTTAACACAAAGCACCCAACTTAC	0	0	0	0
tsrna-11089	GTGTAGCTCAGTGGTAGAGCGCGTGC	2	15	4	0
tsrna-11088	GTGTAGCTCAGTGGTAGAGCGC	0	0	0	0
tsrna-11087	GTGTAGCTCAGTGGTAGAGCATTTGACTGC	5	1	0	1
tsrna-11086	GTGTAGCTCAGTGGTAGAGCATTTGACT	0	2	1	0
tsrna-11084	GTGTAGCTCAGTGGTAGAG	0	0	0	0
tsrna-11079	GTGTAGCGGTTATCACATTCGCCTC	0	0	0	0
tsrna-11077	GTGTAGCGGTTATCACATTCGCC	0	0	0	0
tsrna-11073	GTGTAATGGTTAGCACTCTGGGCT	1	1	1	0
tsrna-11071	GTGTAATGGTTAGCACTCTGGACTTTGA	1	1	1	0
tsrna-11070	GTGTAATGGTTAGCACTCTGGACTTTG	0	2	0	0
tsrna-11069	GTGTAATGGTTAGCACTCTGGACTTT	3	4	0	0
tsrna-11068	GTGTAATGGTTAGCACTCTGGACTT	0	1	0	3
tsrna-11067	GTGTAATGGTTAGCACTCTGGACTCTGAATCCAGCG	10	10	4	3
tsrna-11066	GTGTAATGGTTAGCACTCTGGACTCTGAA	1	0	1	0
tsrna-11065	GTGTAATGGTTAGCACTCTGGACTCTGA	5	0	0	1
tsrna-11064	GTGTAATGGTTAGCACTCTGGACTCTG	4	1	1	1
tsrna-11063	GTGTAATGGTTAGCACTCTGGACTCT	1	2	1	0
tsrna-11062	GTGTAATGGTTAGCACTCTGGACTC	0	3	0	1
tsrna-11061	GTGTAATGGTTAGCACTCTGGACT	0	1	0	2

tsrna-11060	GTGTAATGGTTAGCACTCTGGAC	1	0	0	1
tsrna-11059	GTGTAATGGTTAGCACTCTGGA	1	0	0	0
tsrna-11052	GTGTAATGGTGAGCACTCTGGACTCTGA	1	0	2	0
tsrna-11051	GTGTAATGGTGAGCACTCTGGACTCTG	0	0	0	1
tsrna-11050	GTGTAATGGTGAGCACTCTGGACTCT	2	0	0	0
tsrna-11049	GTGTAATGGTGAGCACTCTGGACTC	3	0	0	0
tsrna-11048	GTGTAATGGTGAGCACTCTGGACT	1	0	0	1
tsrna-11047	GTGTAATGGTGAGCACTCTGGAC	0	0	0	0
tsrna-11046	GTGTAATGGTGAGCACTCTGGA	0	0	0	0
tsrna-11045	GTGTAATGGTGAGCACTCTGG	0	1	0	0
tsrna-11043	GTGTAATGGTCAGCACTCTGGACTCTGA	0	1	0	0
tsrna-11041	GTGTAATGGTCAGCACTCTGGACTC	0	0	0	0
tsrna-11034	GTGTAATGGTAAGCACTCTGGACTC	0	0	0	0
tsrna-11031	GTGGTTTAGTGGTAGAATTCTCGCCT	1	2	0	2
tsrna-11030	GTGGTTTAGTGGTAGAATTCTCGCC	2	2	1	1
tsrna-11029	GTGGTTTAGTGGTAGAATTCTCGC	1	1	0	0
tsrna-11028	GTGGTTTAGTGGTAGAATTCTCG	0	2	0	0
tsrna-11027	GTGGTTTAGTGGTAGAATTCTC	0	0	0	0
tsrna-11025	GTGGTTTAGTGGTAGAA	0	1	0	0
tsrna-11024	GTGGTTGTAGTCCGTGCGAGAATACCA	8	17	9	6
tsrna-11023	GTGGTTGTAGTCCGTGCGAGAATACC	1	5	2	1
tsrna-11022	GTGGTTGTAGTCCGTGCGAGAATAC	2	1	3	0
tsrna-11021	GTGGTTGTAGTCCGTGCGAGAATA	2	2	1	1
tsrna-11020	GTGGTTGTAGTCCGTGCGAGAAT	0	1	1	0
tsrna-11019	GTGGTTGTAGTCCGTGCGAGAA	0	2	2	0
tsrna-11018	GTGGTTGTAGTCCGTGCGAGA	0	0	0	0
tsrna-11017	GTGGTTGTAGTCCGTGCGAG	0	0	0	0
tsrna-11016	GTGGTTGTAGTCCGTGCGA	0	0	0	0
tsrna-11015	GTGGTTGTAGTCCGTGCG	0	0	0	0
tsrna-11012	GTGGTTCGAGCCCACCCAGGGACGCCA	5	92	4	0
tsrna-11010	GTGGTTCAGTGGTAGAATTCTTGCCT	1	0	2	0
tsrna-11009	GTGGTTCAGTGGTAGAATTCTT	2	2	3	0
tsrna-11008	GTGGTTCAGTGGTAGAATTCTCGCCTGCC	18	48	9	3
tsrna-11007	GTGGTTCAGTGGTAGAATTCTCGCCTGC	14	6	3	2
tsrna-11006	GTGGTTCAGTGGTAGAATTCTCGCCTG	6	10	3	4
tsrna-11005	GTGGTTCAGTGGTAGAATTCTCGCCTCC	9	12	1	13
tsrna-11004	GTGGTTCAGTGGTAGAATTCTCGCCT	9	7	0	2
tsrna-11003	GTGGTTCAGTGGTAGAATTCTCGCC	5	4	1	0
tsrna-11002	GTGGTTCAGTGGTAGAATTCTCGC	3	2	1	1
tsrna-11001	GTGGTTCAGTGGTAGAATTCTCG	2	1	2	0
tsrna-11000	GTGGTTCAGTGGTAGAATTCTC	2	3	2	1
tsrna-10999	GTGGTTCAGTGGTAGAATTCT	2	1	1	0
tsrna-10998	GTGGTTCAGTGGTAGAATTC	1	1	0	1
tsrna-10997	GTGGTTCAGTGGTAGAATT	3	1	2	0
tsrna-10996	GTGGTTCAGTGGTAGAAT	2	2	2	0
tsrna-10995	GTGGTTCAGTGGTAGAA	0	1	1	0
tsrna-10993	GTGGTTATCACGTTCGCCTCACACGC	4	2	0	1
tsrna-10992	GTGGTTATCACGTTCGCCTCACACG	0	0	0	0
tsrna-10991	GTGGTTATCACGTTCGCCTCACAC	0	3	0	0
tsrna-10990	GTGGTTATCACGTTCGCCTCACA	0	0	0	2
tsrna-10989	GTGGTTATCACGTTCGCCTCAC	0	0	1	0
tsrna-10988	GTGGTTATCACGTTCGCCTCA	0	1	0	0
tsrna-10986	GTGGTTATCACGTTCGCCTA	0	1	0	0
tsrna-10984	GTGGTTATCACGTTCGCC	1	0	0	0
tsrna-10982	GTGGTTATCACGTCTGCTTTACACGCAGAAGGTCCTGGGT	12	32	6	0
tsrna-10981	GTGGTTATCACGTCTGCTTTACACG	4	0	3	1
tsrna-10980	GTGGTTATCACGTCTGCTTTAC	2	0	1	0
tsrna-10977	GTGGTTATCACGTCTGCTT	1	0	0	0
tsrna-10970	GTGGTTAGTATCCCCGCCTGTCACGCGGG	10	40	9	6
tsrna-10969	GTGGTTAGTATCCCCGCCTGTCA	1	1	1	0
tsrna-10968	GTGGTTAGTATCCCCGCCTGTC	3	0	0	1
tsrna-10967	GTGGTTAGTATCCCCGCCTGT	2	0	0	0
tsrna-10966	GTGGTTAGTATCCCCGCCTG	1	0	0	0
tsrna-10965	GTGGTTAGTATCCCCGCCT	2	1	0	0

tsrna-10964	GTGGTTAGTATCCCCGCC	0	0	0	0
tsrna-10963	GTGGTTAGTATCCCCGC	1	0	1	0
tsrna-10961	GTGGTTAGTACTCTGCGTTGTGGCCGCAGC	14	3	1	1
tsrna-10960	GTGGTTAGTACTCTGCGTTGTGGCCGC	11	4	6	3
tsrna-10959	GTGGTTAGTACTCTGCGTTGTGGCCG	11	5	6	3
tsrna-10958	GTGGTTAGTACTCTGCGTTGTGGCC	10	7	4	2
tsrna-10957	GTGGTTAGTACTCTGCGTTGTGGC	12	4	3	2
tsrna-10956	GTGGTTAGTACTCTGCGTTGTGG	10	0	4	4
tsrna-10955	GTGGTTAGTACTCTGCGTTGTG	10	2	7	4
tsrna-10954	GTGGTTAGTACTCTGCGTTGT	12	2	3	2
tsrna-10953	GTGGTTAGTACTCTGCGTTG	8	2	5	0
tsrna-10952	GTGGTTAGTACTCTGCGTT	7	5	3	1
tsrna-10951	GTGGTTAGTACTCTGCGT	0	1	0	0
tsrna-10950	GTGGTTAGTACTCTGCGCTGTGGCC	0	2	1	0
tsrna-10949	GTGGTTAGTACTCTGCGCTGTGG	1	0	0	0
tsrna-10948	GTGGTTAGTACTCTGCGCTGTG	0	1	0	2
tsrna-10947	GTGGTTAGTACTCTGCGCTGT	2	1	0	0
tsrna-10946	GTGGTTAGTACTCTGCGCTG	2	1	1	0
tsrna-10945	GTGGTTAGTACTCTGCGCT	1	2	0	0
tsrna-10944	GTGGTTAGTACTCTGCGC	3	1	0	1
tsrna-10943	GTGGTTAGTACTCTGCG	0	0	0	0
tsrna-10941	GTGGTTAGGATTCGGCGCTCTCAT	9	8	5	6
tsrna-10940	GTGGTTAGGATTCGGCGCTCTCACCGCCGCGGCCCGGGTT	16	21	11	7
tsrna-10939	GTGGTTAGGATTCGGCGCTCTCACCGCCGCGGCCCGGGT	17	28	14	7
tsrna-10938	GTGGTTAGGATTCGGCGCTCTCACCGCCGCGGCCCGGG	13	23	9	4
tsrna-10937	GTGGTTAGGATTCGGCGCTCTCACCGCCGCGGCCCG	7	25	9	6
tsrna-10936	GTGGTTAGGATTCGGCGCTCTCACCGCCGCGGCCC	11	32	5	10
tsrna-10935	GTGGTTAGGATTCGGCGCTCTCACCGCCGC	14	21	8	6
tsrna-10934	GTGGTTAGGATTCGGCGCTCTCACCGCCG	15	18	9	11
tsrna-10933	GTGGTTAGGATTCGGCGCTCTCACCGCC	15	26	6	12
tsrna-10932	GTGGTTAGGATTCGGCGCTCTCACCGC	8	14	8	10
tsrna-10931	GTGGTTAGGATTCGGCGCTCTCACCG	14	14	6	5
tsrna-10930	GTGGTTAGGATTCGGCGCTCTCACC	9	10	4	6
tsrna-10929	GTGGTTAGGATTCGGCGCTCTCAC	8	12	9	9
tsrna-10928	GTGGTTAGGATTCGGCGCTCTCA	10	15	7	12
tsrna-10927	GTGGTTAGGATTCGGCGCTCTC	2	9	4	2
tsrna-10926	GTGGTTAGGATTCGGCGCTCT	9	16	6	3
tsrna-10925	GTGGTTAGGATTCGGCGCTC	6	11	4	0
tsrna-10924	GTGGTTAGGATTCGGCGCT	4	10	5	4
tsrna-10923	GTGGTTAGGATTCGGCGC	2	5	2	1
tsrna-10922	GTGGTTAGGATTCGGCG	2	0	0	0
tsrna-10921	GTGGTTAGGATTCGGC	2	0	1	0
tsrna-10909	GTGGTTAAGGCGTTGGACTTG	0	0	1	0
tsrna-10896	GTGGTTAAGGCGATGGA	0	1	0	0
tsrna-10892	GTGGTGAGTATCCCTGCCTGT	0	1	0	0
tsrna-10891	GTGGTGAGTATCCCCGCCTGTCACGCGGGA	30	48	14	5
tsrna-10890	GTGGTGAGTATCCCCGCCTGTCACGCGGG	32	53	17	4
tsrna-10889	GTGGTGAGTATCCCCGCCTGTCACGCG	4	10	3	0
tsrna-10888	GTGGTGAGTATCCCCGCCTGTCACGC	3	9	9	2
tsrna-10887	GTGGTGAGTATCCCCGCCTGTCACG	2	10	0	0
tsrna-10886	GTGGTGAGTATCCCCGCCTGTCAC	2	8	0	2
tsrna-10885	GTGGTGAGTATCCCCGCCTGTCA	2	10	1	1
tsrna-10884	GTGGTGAGTATCCCCGCCTGTC	1	8	1	3
tsrna-10883	GTGGTGAGTATCCCCGCCTGT	3	2	0	0
tsrna-10882	GTGGTGAGTATCCCCGCCTG	0	8	2	1
tsrna-10881	GTGGTGAGTATCCCCGCCT	1	3	0	2
tsrna-10880	GTGGTGAGTATCCCCGCC	1	1	0	0
tsrna-10879	GTGGTGAGTATCCCCGC	0	0	0	0
tsrna-10878	GTGGTGAGTATCCCCG	0	1	0	0
tsrna-10874	GTGGTGAGCATAGCTGCCT	0	0	1	0
tsrna-10873	GTGGTGAGCATAGCTGCC	0	0	0	0
tsrna-10872	GTGGTGAGCATAGCTGC	0	1	0	0
tsrna-10871	GTGGTGAGCATAGCTG	0	0	0	0
tsrna-10870	GTGGTCTAGTGGTTAGGATTCGGCGCTCTC	32	37	22	20

tsrna-10869	GTGGTCTAGTGGTTAGGATTCGGCGCTCT	39	43	24	26
tsrna-10868	GTGGTCTAGTGGTTAGGATTCGGCGCTC	34	47	23	15
tsrna-10867	GTGGTCTAGTGGTTAGGATTCGGCGCT	49	49	23	14
tsrna-10866	GTGGTCTAGTGGTTAGGATTCGGCGC	17	12	7	6
tsrna-10865	GTGGTCTAGTGGTTAGGATTCGGCG	13	10	7	6
tsrna-10864	GTGGTCTAGTGGTTAGGATTCGGC	5	7	5	4
tsrna-10863	GTGGTCTAGTGGTTAGGATTCGG	4	2	1	1
tsrna-10862	GTGGTCTAGTGGTTAGGATTCG	2	2	1	0
tsrna-10861	GTGGTCTAGTGGTTAGGATTC	2	2	2	0
tsrna-10860	GTGGTCTAGTGGTTAGGATT	1	1	0	0
tsrna-10859	GTGGTCTAGTGGTTAGGAT	0	0	0	1
tsrna-10855	GTGGTCTAGTGGCTAGGATTCGGCGC	9	27	5	32
tsrna-10854	GTGGTCTAGTGGCTAGGATTCGGCG	10	21	5	24
tsrna-10853	GTGGTCTAGTGGCTAGGATTCGG	3	2	0	1
tsrna-10852	GTGGTCTAGTGGCTAGGATTCG	2	0	0	1
tsrna-10851	GTGGTCTAGTGGCTAGGATT	0	2	0	0
tsrna-10849	GTGGTCTAAGGCGCTGGA	0	0	0	0
tsrna-10844	GTGGTATGATTCTCGCTTTG	2	1	2	0
tsrna-10843	GTGGTATGATTCTCGCTTT	1	2	0	0
tsrna-10842	GTGGTATGATTCTCGCTT	3	1	2	0
tsrna-10841	GTGGTATGATTCTCGC	0	2	3	0
tsrna-10832	GTGGTATAGTGGTTAGCATAGCTGCC	0	1	1	0
tsrna-10828	GTGGTATAGTGGTGAGCATAGCTGCC	0	1	0	0
tsrna-10827	GTGGTATAGTGGTGAGCATAGCTGC	0	4	0	0
tsrna-10826	GTGGTATAGTGGTGAGCATAGCTG	1	1	0	2
tsrna-10825	GTGGTATAGTGGTGAGCATAGCT	2	0	0	0
tsrna-10824	GTGGTATAGTGGTGAGCATAGC	0	0	1	0
tsrna-10823	GTGGTATAGTGGTGAGCATAG	0	0	0	0
tsrna-10822	GTGGTATAGTGGTGAGCATA	1	1	1	0
tsrna-10821	GTGGTATAGTGGTGAGCA	0	1	0	0
tsrna-10818	GTGGTATAGTGGTAAGCATAGCTG	0	1	0	0
tsrna-10815	GTGGTAGAGCGCTCGC	0	1	0	0
tsrna-10814	GTGGTAGAGCGCGTGCT	9	21	2	0
tsrna-10813	GTGGTAGAGCGCGTGC	4	17	5	0
tsrna-10810	GTGGTAGAGCATTTGACTGCAGATCAAG	0	3	0	0
tsrna-10809	GTGGTAGAGCATTTGACTGCAGATCA	0	1	0	0
tsrna-10808	GTGGTAGAGCATTTGACTGCAGATC	0	1	0	0
tsrna-10807	GTGGTAGAGCATTTGACTGCA	0	3	0	0
tsrna-10806	GTGGTAGAGCATTTGACTGC	1	1	0	0
tsrna-10805	GTGGTAGAGCATTTGACTG	0	0	0	1
tsrna-10804	GTGGTAGAGCATTTGACT	0	1	0	0
tsrna-10801	GTGGTAGAATTCTTGCCTGC	0	0	0	0
tsrna-10800	GTGGTAGAATTCTCGCCTT	0	3	1	0
tsrna-10799	GTGGTAGAATTCTCGCCTGCCACG	43	82	29	18
tsrna-10798	GTGGTAGAATTCTCGCCTGCCAC	20	39	7	5
tsrna-10797	GTGGTAGAATTCTCGCCTGCCA	13	41	5	1
tsrna-10796	GTGGTAGAATTCTCGCCTGCC	17	31	6	3
tsrna-10795	GTGGTAGAATTCTCGCCTGC	6	9	1	3
tsrna-10794	GTGGTAGAATTCTCGCCTG	1	9	1	3
tsrna-10793	GTGGTAGAATTCTCGCCTCCCACGCGGGAGACCCGGGT	9	18	2	11
tsrna-10792	GTGGTAGAATTCTCGCCTCCCACG	5	6	1	9
tsrna-10791	GTGGTAGAATTCTCGCCTCCC	2	11	1	7
tsrna-10790	GTGGTAGAATTCTCGCCTCC	1	9	1	5
tsrna-10789	GTGGTAGAATTCTCGCCTC	2	3	1	1
tsrna-10788	GTGGTAGAATTCTCGCCT	1	4	0	2
tsrna-10787	GTGGTAGAATTCTCGCC	1	3	0	0
tsrna-10786	GTGGTAGAATTCTCGC	0	2	0	0
tsrna-10783	GTGGTAAGCATAGCTGCCTTCC	1	0	0	0
tsrna-10782	GTGGTAAGCATAGCTGCCTTC	0	1	0	0
tsrna-10778	GTGGGTTTAAGTCCCATTGGTCTAGCCA	1	2	1	1
tsrna-10777	GTGGGTTTAAGTCCCATTGGTCTAGCC	0	0	0	0
tsrna-10776	GTGGGTTTAAGTCCCATTGGTCTAGC	0	0	1	0
tsrna-10775	GTGGGTTTAAGTCCCATTGGTCTAG	1	2	1	0
tsrna-10774	GTGGGTTTAAGTCCCATTGGTCTA	0	0	0	0

tsrna-10772	GTGGGTTCGAGTCCCATCTGGGTCGCCA	2	12	0	1
tsrna-10771	GTGGGTTCGAGTCCCATCTGGGTCGCC	1	0	1	0
tsrna-10770	GTGGGTTCGAATCCCATCCTCGTCGCCA	6	11	4	2
tsrna-10769	GTGGGTTCGAATCCCATCCTCGTCGCC	0	2	0	0
tsrna-10768	GTGGGTTCGAATCCCATCCTCGTCGC	0	1	0	0
tsrna-10767	GTGGGTTCGAATCCCATCCTCGTCG	2	0	0	0
tsrna-10766	GTGGGTTCGAATCCCATCCTCGTC	0	0	0	0
tsrna-10763	GTGGGTTCGAATCCCACTTCTGACACCA	28	28	11	10
tsrna-10762	GTGGGTTCGAATCCCACTTCTGACACC	2	2	1	2
tsrna-10756	GTGGGTTCGAATCCCACTCCTGACACCA	105	118	57	37
tsrna-10755	GTGGGTTCGAATCCCACTCCTGACACC	3	11	3	1
tsrna-10754	GTGGGTTCGAATCCCACTCCTGACAC	0	1	1	0
tsrna-10752	GTGGGTTCGAATCCCACTCCTGAC	1	1	0	0
tsrna-10748	GTGGGTTCGAATCCCACCGCTGCCACCA	12	43	2	5
tsrna-10746	GGGGAATTAGCTCAAGTGGTAGAGC	0	2	0	2
tsrna-10745	GGGGAATTAGCTCAAGTGGTAGA	0	4	0	2
tsrna-10744	GGGGAATTAGCTCAAGTGGTAG	0	0	0	4
tsrna-10743	GGGGAATTAGCTCAAGTGGTA	0	1	1	1
tsrna-10742	GGGGAATTAGCTCAAGTGGT	1	3	0	0
tsrna-10741	GGGGAATTAGCTCAAGTGG	1	2	0	1
tsrna-10740	GGGGAATTAGCTCAAGT	0	0	1	1
tsrna-10739	GGGGAATTAGCTCAAGCGGTAGAGCGCTTG	0	0	0	2
tsrna-10738	GGGGAATTAGCTCAAGCGGTAGAGC	0	1	0	1
tsrna-10737	GGGGAATTAGCTCAAGCGGTAGAG	4	5	0	4
tsrna-10736	GGGGAATTAGCTCAAGCGGTAGA	1	2	0	2
tsrna-10735	GGGGAATTAGCTCAAGCGGTA	0	0	0	2
tsrna-10734	GGGGAATTAGCTCAAGCGGT	0	2	0	1
tsrna-10733	GGGGAATTAGCTCAAGCGG	1	1	0	1
tsrna-10732	GGGGAATTAGCTCAAGCG	0	0	0	1
tsrna-10731	GGGGAATTAGCTCAAGC	0	1	0	2
tsrna-10730	GGGGAATTAGCTCAAATGGTAGAGCGCTCGCTT	7	7	3	3
tsrna-10729	GGGGAATTAGCTCAAATGGTAGAGCGCTCGCT	7	9	2	7
tsrna-10728	GGGGAATTAGCTCAAATGGTAGAGCGCTCG	8	9	4	7
tsrna-10727	GGGGAATTAGCTCAAATGGTAGAGCGC	7	5	1	6
tsrna-10726	GGGGAATTAGCTCAAATGGTAGAGC	3	14	2	1
tsrna-10725	GGGGAATTAGCTCAAATGGTAGAG	9	9	1	4
tsrna-10724	GGGGAATTAGCTCAAATGGTAGA	3	10	3	6
tsrna-10723	GGGGAATTAGCTCAAATGGTAG	8	2	2	10
tsrna-10722	GGGGAATTAGCTCAAATGGTA	5	10	3	5
tsrna-10721	GGGGAATTAGCTCAAATGGT	3	5	2	4
tsrna-10720	GGGGAATTAGCTCAAATGG	3	10	1	2
tsrna-10719	GGGGAATTAGCTCAAATG	5	3	2	4
tsrna-10718	GGGGAATTAGCTCAAAT	5	11	2	1
tsrna-10717	GGGGAATTAGCTCAAA	5	9	1	1
tsrna-10716	GGGCTTAGCTTAATTAAAGTGGCTGATTTG	2	0	1	0
tsrna-10713	GGGCTCCGTGGCGCAATGGA	0	0	0	1
tsrna-10712	GGGCTCACATCACCCCATAAACACCA	6	3	2	1
tsrna-10703	GGGCGTATAGCTCAGGGGTAGAGCATTTG	0	1	0	0
tsrna-10701	GGGCGTATAGCTCAGGGGTAGAGC	0	0	0	0
tsrna-10699	GGGCCCATACCCCGAAAATGTTGGTTATACCCTTCC	1	0	0	0
tsrna-10698	GGGCCCATACCCCGAAAATGTTGGTTATACC	1	0	0	0
tsrna-10697	GGGCCCATACCCCGAAAATGTTGGTTATAC	0	0	0	0
tsrna-10696	GGGCCCATACCCCGAAAATGTTGGTTA	0	1	0	0
tsrna-10692	GGGCCCATACCCCGAAAATG	0	1	0	0
tsrna-10691	GGGCCCATAACCCAGAGGTCGATGGA	11	6	0	2
tsrna-10690	GGGCCCATAACCCAGAGGTCGATG	2	1	0	1
tsrna-10689	GGGCCCATAACCCAGAGGTCGAT	1	1	0	0
tsrna-10688	GGGCCCATAACCCAGAGGTCGA	0	0	0	0
tsrna-10683	GGGCCAGTGGCGCAATGGATAACGCGTCTGACT	1	0	1	2
tsrna-10682	GGGCCAGTGGCGCAATGGATAACGCGTCTGAC	0	0	1	0
tsrna-10681	GGGCCAGTGGCGCAATGGATAACGCGTCTG	0	1	0	0
tsrna-10679	GGGCCAGTGGCGCAATGGATAACGCGTC	0	1	0	0
tsrna-10677	GGGCCAGTGGCGCAATGGATAACG	1	0	0	0
tsrna-10676	GGGCCAGTGGCGCAATGGATAAC	0	0	0	0

tsrna-10673	GGGCCAGTGGCGCAATGGAT	0	1	0	0
tsrna-10671	GGGCCAGTGGCGCAATGG	0	0	0	0
tsrna-10668	GGGCAGAGCATTTGACTGC	0	0	0	0
tsrna-10666	GGGATTGTGGGTTCGAGTCCCATCTGGGTCGCC	1	2	1	0
tsrna-10665	GGGATTGTGGGTTCGAGTCCCATCTGGGGTGCCA	0	4	0	0
tsrna-10664	GGGATTGTGGGTTCGAGTCCCATCTGGGGTGCC	0	3	0	0
tsrna-10663	GGGATTGTGGGTTCGAGTCCCATC	0	1	0	0
tsrna-10662	GGGATTGTGGGTTCGAGTCCCACC	0	1	0	0
tsrna-10660	GGGATTGTGGGTTCGAGTCCC	0	0	0	0
tsrna-10659	GGGATTGTGGGTTCGAGTCC	0	0	0	0
tsrna-10657	GGGATTAGCTCAAATGGTAGAGCGCTCGC	6	15	6	1
tsrna-10656	GGGATTAGCTCAAATGGTAGAGC	7	13	4	4
tsrna-10655	GGGATTAGCTCAAATGGTAGA	11	12	5	3
tsrna-10654	GGGATTAGCTCAAATGGTAG	6	6	3	4
tsrna-10653	GGGATGTAGCTCAGTGGTAGAGC	0	1	0	0
tsrna-10651	GGGATGTAGCTCAGTGGTAGA	0	0	0	0
tsrna-10648	GGGATGTAGCTCAGTGGT	1	1	0	0
tsrna-10645	GGGATGGGTTCGATTCTCATAGTC	1	1	0	0
tsrna-10644	GGGATGGGTTCGATTCTCATAGT	0	0	2	0
tsrna-10643	GGGATGGGTTCGATTCTCATAG	0	0	0	0
tsrna-10642	GGGATGGGTTCGATTCTCATA	0	1	0	0
tsrna-10641	GGGATGGGTTCGATTCTCA	0	2	1	0
tsrna-10640	GGGATGGGTTCGATTCT	0	1	0	0
tsrna-10639	GGGATGGGTTCGATTC	0	0	1	0
tsrna-10638	GGGATCGATGCCCGCATCCTCCACCA	3	7	2	0
tsrna-10637	GGGATCGATGCCCGCATCCTCCACC	0	1	0	0
tsrna-10635	GGGAGGCCCGGGTTCGATTCCCGG	1	0	1	0
tsrna-10626	GGGAGACCGGGGTTCGATTCCCCGACGGGGAGCCA	1	13	0	0
tsrna-10619	GGGAGACCGGGGTTCGATTCCCCGAC	0	0	0	0
tsrna-10611	GGGACTCTTAATCCCAGGGTCGTGGGTT	1	0	0	0
tsrna-10610	GGGAATTAGCTCAAATGGTAGAGC	0	4	1	1
tsrna-10609	GGGAATTAGCTCAAATGGTAGAG	1	1	0	0
tsrna-10608	GGGAATTAGCTCAAATGGTAGA	0	3	0	0
tsrna-10607	GGGAATTAGCTCAAATGGT	0	0	1	1
tsrna-10606	GGCTTGAAACCAGCTTTGGGGG	0	0	0	0
tsrna-10600	GGCTTACGACCCCTTATTTACCCCA	4	1	2	0
tsrna-10597	GGCTTACGACCCCTTATTTACC	0	0	1	0
tsrna-10591	GGCTGTTAACCGAAAGGTTGGTGGT	4	32	2	5
tsrna-10590	GGCTGTTAACCGAAAGGTTGGTG	1	1	0	0
tsrna-10589	GGCTGTTAACCGAAAGGTTGGT	0	1	0	0
tsrna-10588	GGCTGTTAACCGAAAGGTTGG	1	1	0	0
tsrna-10586	GGCTGTTAACCGAAAGGTT	0	0	0	1
tsrna-10584	GGCTGTATAGCTCAGTGGTAGAGCATTTG	2	2	0	0
tsrna-10581	GGCTGGTTAGCTCAGTTGGTTAGAGC	0	2	0	0
tsrna-10575	GGCTGAGTGAAGCATTGGACTGTAAATCT	0	0	2	1
tsrna-10574	GGCTGAGTGAAGCATTGGACTGTAAATC	1	1	1	0
tsrna-10573	GGCTGAGTGAAGCATTGGACTGTAAAT	2	1	1	1
tsrna-10572	GGCTGAGTGAAGCATTGGACTGTAAA	1	2	1	0
tsrna-10571	GGCTGAGTGAAGCATTGGACTGTAA	0	1	0	2
tsrna-10570	GGCTGAGTGAAGCATTGGACTGTA	0	1	0	0
tsrna-10569	GGCTGAGTGAAGCATTGGACTGT	0	1	0	0
tsrna-10568	GGCTGAGTGAAGCATTGGACTG	0	0	0	0
tsrna-10558	GGCTCTGTGGCGCAATGGATAGCGCATTGGACT	7	10	2	3
tsrna-10557	GGCTCTGTGGCGCAATGGATAGCGCATTGG	7	6	1	0
tsrna-10556	GGCTCTGTGGCGCAATGGATAGCGCATTG	1	2	0	0
tsrna-10548	GGCTCTGTGGCGCAATGGAT	0	0	1	0
tsrna-10544	GGCTCTATGGCTTAGTTGGTTAAAGC	0	2	0	0
tsrna-10542	GGCTCTATGGCTTAGTTGG	0	1	0	0
tsrna-10541	GGCTCGTTGGTCTAGTGGTATGATTCTCGC	6	1	1	5
tsrna-10540	GGCTCGTTGGTCTAGTGGTATGATTCTCG	0	0	1	0
tsrna-10539	GGCTCGTTGGTCTAGTGGTATGATTCTC	0	1	0	0
tsrna-10537	GGCTCGTTGGTCTAGTGGTATGATTC	0	0	1	0
tsrna-10526	GGCTCGTTGGTCTAGGGGTATGATTCTCGGTTT	6	6	2	4
tsrna-10525	GGCTCGTTGGTCTAGGGGTATGATTCTCGGTT	1	3	2	1

tsrna-10524	GGCTCGTTGGTCTAGGGGTATGATTCTCGGT	1	1	2	2
tsrna-10523	GGCTCGTTGGTCTAGGGGTATGATTCTCGG	3	0	1	2
tsrna-10522	GGCTCGTTGGTCTAGGGGTATGATTCTCGCTTT	51	62	82	59
tsrna-10521	GGCTCGTTGGTCTAGGGGTATGATTCTCGCTTC	76	63	83	51
tsrna-10520	GGCTCGTTGGTCTAGGGGTATGATTCTCGCTT	52	60	64	51
tsrna-10519	GGCTCGTTGGTCTAGGGGTATGATTCTCGCT	55	58	45	57
tsrna-10518	GGCTCGTTGGTCTAGGGGTATGATTCTCGC	8	12	4	7
tsrna-10517	GGCTCGTTGGTCTAGGGGTATGATTCTCG	0	0	0	0
tsrna-10516	GGCTCGTTGGTCTAGGGGTATGATTCTC	2	0	0	0
tsrna-10515	GGCTCGTTGGTCTAGGGGTATGATTCT	0	0	0	0
tsrna-10514	GGCTCGTTGGTCTAGGGGTATGATTC	1	0	0	0
tsrna-10513	GGCTCGTTGGTCTAGGGGTATGATT	0	1	0	0
tsrna-10500	GGCTCCGTGGCTTAGCTGGTT	1	0	0	0
tsrna-10494	GGCTCCGTGGCGCAATGGATAGCGCATTGGACT	8	14	3	2
tsrna-10493	GGCTCCGTGGCGCAATGGATAGCGCATTGGAC	13	11	4	0
tsrna-10492	GGCTCCGTGGCGCAATGGATAGCGCATTGG	2	6	1	0
tsrna-10491	GGCTCCGTGGCGCAATGGATAGCGCATTG	2	0	0	0
tsrna-10487	GGCTCCGTGGCGCAATGGATAGCGC	1	0	0	0
tsrna-10485	GGCTCCGTGGCGCAATGGATAGC	0	2	0	0
tsrna-10483	GGCTCCGTGGCGCAATGGATA	1	0	0	0
tsrna-10482	GGCTCCGTGGCGCAATGGAT	0	1	0	0
tsrna-10477	GGCTCCGTAGCTTAGTTGGTTAAAGC	0	0	1	0
tsrna-10475	GGCTCCATAGCTCAGTGGTTAGAGCACTGG	4	4	3	0
tsrna-10474	GGCTCCATAGCTCAGTGGTTAGAGCA	6	1	1	0
tsrna-10473	GGCTCCATAGCTCAGTGGTTAGAGC	1	1	0	1
tsrna-10472	GGCTCCATAGCTCAGTGGTTAGAG	3	0	1	0
tsrna-10471	GGCTCCATAGCTCAGTGGTTAGA	2	0	0	0
tsrna-10470	GGCTCCATAGCTCAGTGGT	2	1	1	0
tsrna-10469	GGCTCCATAGCTCAGTGG	2	1	0	0
tsrna-10468	GGCTCCATAGCTCAGGGGTTAGAGCACTGGTCTT	3	9	9	10
tsrna-10467	GGCTCCATAGCTCAGGGGTTAGAGCACTGGTCT	5	4	2	6
tsrna-10466	GGCTCCATAGCTCAGGGGTTAGAGCACTGGTC	3	3	2	6
tsrna-10465	GGCTCCATAGCTCAGGGGTTAGAGCACTGG	3	0	0	0
tsrna-10464	GGCTCCATAGCTCAGGGGTTAGAGCACTG	0	1	0	0
tsrna-10463	GGCTCCATAGCTCAGGGGTTAGAGCACT	0	0	0	0
tsrna-10461	GGCTCCATAGCTCAGGGGTTAGAGCA	1	0	0	0
tsrna-10460	GGCTCCATAGCTCAGGGGTTAGAGC	1	0	1	0
tsrna-10459	GGCTCCATAGCTCAGGGGTTAGAG	0	1	0	0
tsrna-10457	GGCTCCATAGCTCAGGGGTTAG	0	0	0	0
tsrna-10455	GGCTCCATAGCTCAGGGGT	1	0	0	0
tsrna-10453	GGCTCCATAGCTCAGGG	0	0	0	1
tsrna-10449	GGCTCCAGTCTCTTCGGGGGCGTGGGTTCG	0	6	0	0
tsrna-10448	GGCTCCAGTCTCTTCGGGGGCGTGGGTTC	2	3	0	1
tsrna-10447	GGCTCCAGTCTCTTCGGGGGCGTGGGT	1	2	0	0
tsrna-10446	GGCTCCAGTCTCTTCGGGGGCGTGGG	0	0	0	0
tsrna-10444	GGCTCCAGTCTCTTCGGGGGCGTG	1	0	0	0
tsrna-10437	GGCTCCAGTCTCTTCGGAGGCGTGGGTTCG	0	0	0	1
tsrna-10436	GGCTCCAGTCTCTTCGGAGGCGTGGGTTC	0	6	0	0
tsrna-10435	GGCTCCAGTCTCTTCGGAGGCGTGG	0	2	0	0
tsrna-10427	GGCTCCAGTCATTTCGATGGCGTGGGTTCG	4	5	3	0
tsrna-10426	GGCTCCAGTCATTTCGATGGCGTGG	0	1	0	0
tsrna-10423	GGCTCACATCACCCCATAAACACCA	5	3	2	2
tsrna-10422	GGCTCACATCACCCCATAAACACC	0	1	0	0
tsrna-10413	GGCTAGGATTCGGCGCTTTC	1	3	0	0
tsrna-10412	GGCTAGGATTCGGCGCTTT	2	4	3	0
tsrna-10411	GGCTAGGATTCGGCGCTT	0	1	0	1
tsrna-10410	GGCTAGGATTCGGCGCT	0	2	0	2
tsrna-10409	GGCTAGGATTCGGCGC	0	2	0	0
tsrna-10408	GGCTAGCTCAGTCGGTAGAGCATGGGACTCT	20	21	12	5
tsrna-10407	GGCTAGCTCAGTCGGTAGAGCATGGGACTC	15	11	8	1
tsrna-10406	GGCTAGCTCAGTCGGTAGAGCATGGGACT	15	16	2	2
tsrna-10405	GGCTAGCTCAGTCGGTAGAGCATGGGAC	12	14	2	0
tsrna-10404	GGCTAGCTCAGTCGGTAGAGCATGGGA	13	7	8	1
tsrna-10403	GGCTAGCTCAGTCGGTAGAGCATGGG	3	5	4	0

tsrna-10402	GGCTAGCTCAGTCGGTAGAGCATGG	4	2	0	0
tsrna-10401	GGCTAGCTCAGTCGGTAGAGCATGAGACTC	12	29	4	7
tsrna-10400	GGCTAGCTCAGTCGGTAGAGCATGAGACT	11	18	6	2
tsrna-10399	GGCTAGCTCAGTCGGTAGAGCATGAGAC	9	28	6	7
tsrna-10398	GGCTAGCTCAGTCGGTAGAGCATGAGA	6	8	2	0
tsrna-10397	GGCTAGCTCAGTCGGTAGAGCATGAG	0	1	0	0
tsrna-10396	GGCTAGCTCAGTCGGTAGAGCATGA	1	3	0	0
tsrna-10395	GGCTAGCTCAGTCGGTAGAGCATG	0	1	0	0
tsrna-10394	GGCTAGCTCAGTCGGTAGAGCAT	0	0	1	0
tsrna-10391	GGCTAGCTCAGTCGGTAGAG	0	0	1	0
tsrna-10382	GGCGTGGGTTCGAATCCCACTTCTGACAC	1	0	0	1
tsrna-10376	GGCGGTCTAGTGGTTAGGATTCGGCGCTCT	52	59	24	12
tsrna-10375	GGCGGTCTAGTGGTTAGGATTCGGCGCT	34	58	23	19
tsrna-10374	GGCGGTCTAGTGGTTAGGATTCGGCGC	20	27	16	6
tsrna-10373	GGCGGTCTAGTGGTTAGGATTCGGCG	14	14	7	7
tsrna-10372	GGCGGTCTAGTGGTTAGGATTCGGC	12	16	4	3
tsrna-10371	GGCGGTCTAGTGGTTAGGATT	4	17	5	1
tsrna-10370	GGCGGTCTAGTGGTTAGG	5	9	3	1
tsrna-10369	GGCGGCCCGGGTTCGACTCCCGGTGTGGGAACC	7	14	1	1
tsrna-10368	GGCGGCCCGGGTTCGACTCCCGGTGTGGG	1	3	0	0
tsrna-10367	GGCGGCCCGGGTTCGACTCCCGGTGTGG	0	1	0	0
tsrna-10364	GGCGGCCCGGGTTCGACTCCCGGTG	0	0	0	0
tsrna-10357	GGCGCTTTCACCGCCGCGGCCCG	0	2	0	1
tsrna-10356	GGCGCTCTCACCGCCGCGGCCCGGGTT	0	0	1	0
tsrna-10355	GGCGCTCTCACCGCCGCGGCCCGGG	1	0	0	0
tsrna-10354	GGCGCTCTCACCGCCGCGGCCCGG	0	1	0	0
tsrna-10353	GGCGCTCTCACCGCCGCGGCCCG	0	1	0	0
tsrna-10352	GGCGCTCTCACCGCCGCGGCCC	0	1	0	0
tsrna-10350	GGCGCGGTGGCCAAGTGGTAAGGCGTCGGTCT	0	1	0	0
tsrna-10349	GGCGCGGTGGCCAAGTGGTAAGGCGTCGGT	0	0	0	0
tsrna-10348	GGCGCGGTGGCCAAGTGGTAAGGCGTCGG	0	0	0	0
tsrna-10347	GGCGCGGTGGCCAAGTGGTAAGGCGTCG	0	1	0	0
tsrna-10346	GGCGCGGTGGCCAAGTGGTAAGGCGTC	0	0	0	0
tsrna-10344	GGCGCGGTGGCCAAGTGGTAAGGCG	0	0	0	0
tsrna-10342	GGCGCGGTGGCCAAGTGGTAAGG	0	0	0	0
tsrna-10341	GGCGCGGTGGCCAAGTGGTAAG	0	0	0	0
tsrna-10331	GGCGCCGTGGCTTAGTTGGTTAA	0	0	0	1
tsrna-10330	GGCGCCGTGGCTTAGTTGGTTA	0	0	0	1
tsrna-10329	GGCGCCGTGGCTTAGTTGGTT	0	0	1	0
tsrna-10322	GGCGCCGTGGCTTAGCTGGT	1	0	0	0
tsrna-10321	GGCGCCGTGGCTTAGCTGG	0	0	1	0
tsrna-10318	GGCGCAGCGGAAGCGTGCTGGGCCC	5	50	8	10
tsrna-10317	GGCGCAGCGGAAGCGTGCTGGGCC	5	31	0	1
tsrna-10316	GGCGCAGCGGAAGCGTGCTGGGC	3	31	2	1
tsrna-10315	GGCGCAGCGGAAGCGTGCTGGG	2	24	0	1
tsrna-10314	GGCGCAATGGATAGCGCATTGGA	4	15	2	0
tsrna-10313	GGCGCAATGGATAGCGCATTGG	3	12	0	0
tsrna-10312	GGCGCAATGGATAGCGCATTG	1	1	0	0
tsrna-10311	GGCGCAATGGATAACGCGTCTGACTACGG	1	6	0	0
tsrna-10310	GGCGCAACGGTAGCGCGTCTGACTCCAG	1	2	1	1
tsrna-10306	GGCCTCGTGGCGCAACGGTAGCGCGTCTGACTCC	1	10	1	3
tsrna-10305	GGCCTCGTGGCGCAACGGTAGCGCGTCTGACT	0	1	0	1
tsrna-10304	GGCCTCGTGGCGCAACGGTAGCGCGTCTG	0	0	1	0
tsrna-10303	GGCCTCGTGGCGCAACGGTAGCGC	0	0	0	0
tsrna-10298	GGCCTCGTGGCGCAACGGT	0	0	0	0
tsrna-10294	GGCCTCCTAAGCCAGGGATTGTGGGT	147	373	78	43
tsrna-10293	GGCCTCCTAAGCCAGGGATTGTGG	1	0	0	0
tsrna-10291	GGCCTAATGGATAAGGCATTG	3	1	1	0
tsrna-10290	GGCCTAATGGATAAGGCATCAGCCTC	4	1	1	0
tsrna-10289	GGCCTAATGGATAAGGCATCAGCCT	3	7	1	0
tsrna-10288	GGCCTAATGGATAAGGCATCAG	2	1	5	2
tsrna-10283	GGCCGTGATCGTATAGTGGTTAGTACTCTGCGTTGTG	37	15	22	15
tsrna-10282	GGCCGTGATCGTATAGTGGTTAGTACTCTGCGTTGT	42	13	27	9
tsrna-10281	GGCCGTGATCGTATAGTGGTTAGTACTCTGCGTTG	26	9	17	2

tsrna-10280	GGCCGTGATCGTATAGTGGTTAGTACTCTGCGTT	29	16	18	8
tsrna-10279	GGCCGTGATCGTATAGTGGTTAGTACTCTGCGT	2	4	6	3
tsrna-10278	GGCCGTGATCGTATAGTGGTTAGTACTCTGCG	6	1	4	0
tsrna-10277	GGCCGTGATCGTATAGTGGTTAGTACTCTGC	0	4	2	1
tsrna-10276	GGCCGTGATCGTATAGTGGTTAGTACTCTG	0	3	1	0
tsrna-10275	GGCCGTGATCGTATAGTGGTTAGTACTCT	1	0	2	1
tsrna-10274	GGCCGTGATCGTATAGTGGTTAGTACTC	0	2	1	0
tsrna-10273	GGCCGTGATCGTATAGTGGTTAGTACT	0	1	1	0
tsrna-10271	GGCCGTGATCGTATAGTGGTTAGTA	0	0	0	0
tsrna-10269	GGCCGTGATCGTATAGTGGTTAG	0	0	0	0
tsrna-10264	GGCCGTGATCGTATAGTG	0	0	0	0
tsrna-10263	GGCCGTGATCGTATAGT	0	1	0	0
tsrna-10261	GGCCGGTTAGCTCAGTTGGTTAGAGCGTGGTGCT	2	2	2	1
tsrna-10260	GGCCGGTTAGCTCAGTTGGTTAGAGCGTGG	1	2	0	1
tsrna-10259	GGCCGGTTAGCTCAGTTGGTTAGAGCGTG	1	0	0	1
tsrna-10258	GGCCGGTTAGCTCAGTTGGTTAGAGC	1	3	0	0
tsrna-10257	GGCCGGTTAGCTCAGTTGGTTAGAG	2	0	0	1
tsrna-10256	GGCCGGTTAGCTCAGTTGGTTAGA	0	1	1	1
tsrna-10255	GGCCGGTTAGCTCAGTTGGTTAG	1	1	0	1
tsrna-10254	GGCCGGTTAGCTCAGTTGGTTA	0	1	0	0
tsrna-10253	GGCCGGTTAGCTCAGTTGGTT	1	0	1	0
tsrna-10252	GGCCGGTTAGCTCAGTTGGTCAGAGCGTGG	0	0	0	1
tsrna-10251	GGCCGGTTAGCTCAGTTGGTCAGAGC	1	0	0	0
tsrna-10250	GGCCGGTTAGCTCAGTTGGTCAGAG	1	0	0	0
tsrna-10249	GGCCGGTTAGCTCAGTTGGTCAGA	1	2	0	0
tsrna-10248	GGCCGGTTAGCTCAGTTGGTC	1	1	1	0
tsrna-10247	GGCCGGTTAGCTCAGTTGGTAAGAGC	1	0	1	0
tsrna-10246	GGCCGGTTAGCTCAGTTGGTAAGA	2	3	0	0
tsrna-10245	GGCCGGTTAGCTCAGTTGGTA	2	0	1	0
tsrna-10244	GGCCGGTTAGCTCAGTTGGT	1	0	0	0
tsrna-10243	GGCCGGTTAGCTCAGTTGG	2	2	1	0
tsrna-10242	GGCCGGTTAGCTCAGTTG	0	0	1	0
tsrna-10241	GGCCGGTTAGCTCAGTT	1	1	0	0
tsrna-10240	GGCCGGTTAGCTCAGTCGGCTAGAGCGTGG	5	3	2	0
tsrna-10239	GGCCGGTTAGCTCAGTCGGCTAGAGC	2	1	1	0
tsrna-10238	GGCCGGTTAGCTCAGTCGGCTAGA	3	1	1	1
tsrna-10237	GGCCGGTTAGCTCAGTCGGC	0	0	0	0
tsrna-10236	GGCCGGTTAGCTCAGTCGG	1	0	0	1
tsrna-10234	GGCCGGTTAGCTCAGT	1	0	0	0
tsrna-10233	GGCCGCGTGGCCTAATGGATAAGGCGTCTGATT	1	2	0	1
tsrna-10232	GGCCGCGTGGCCTAATGGATAAGGCGTCTG	0	0	1	0
tsrna-10217	GGCCGCAGCAACCTCGGTTCG	0	1	0	0
tsrna-10208	GGCCGAGCGGTCTAAGGCGCTGGATT	0	1	1	2
tsrna-10206	GGCCGAGCGGTCTAAGGCGCTGCGTTCAG	0	2	0	1
tsrna-10201	GGCCGAGCGGTCTAAGGCGCTG	0	1	0	0
tsrna-10200	GGCCGAGCGGTCTAAGGCGCT	0	0	0	0
tsrna-10193	GGCCCTGTGGCTTAGC	0	0	0	1
tsrna-10192	GGCCCTATAGCTCAGGGGTTAGAGCACTGG	1	1	0	0
tsrna-10191	GGCCCTATAGCTCAGGGGTTAGAGCACTG	0	1	0	3
tsrna-10190	GGCCCTATAGCTCAGGGGTTAGAGCACT	1	1	0	0
tsrna-10187	GGCCCTATAGCTCAGGGGTTAGAGC	0	0	0	0
tsrna-10183	GGCCCTATAGCTCAGGGGTT	0	0	0	1
tsrna-10178	GGCCCGGGTTCGATTCCCGGTCAGGGAACC	1	10	0	0
tsrna-10177	GGCCCGGGTTCGATTCCCGGTCAGGGAAC	0	5	0	0
tsrna-10176	GGCCCGGGTTCGATTCCCGGTCAGGGAA	0	2	0	0
tsrna-10175	GGCCCGGGTTCGATTCCCGGTCAGGGA	0	3	0	0
tsrna-10174	GGCCCGGGTTCGATTCCCGGTCAGGG	0	1	0	0
tsrna-10171	GGCCCGGGTTCGATTCCCGGCCAATGCACC	2	14	1	0
tsrna-10164	GGCCCGGGTTCGACTCCCGGTGTGGGAACC	3	11	0	2
tsrna-10163	GGCCCGGGTTCGACTCCCGGTGTGGGAAC	0	2	2	0
tsrna-10162	GGCCCGGGTTCGACTCCCGGTGTGGGAA	0	1	0	0
tsrna-10161	GGCCCGGGTTCGACTCCCGGTGTGGG	0	0	2	0
tsrna-10160	GGCCCGGGTTCGACTCCCGGTGTGG	1	0	0	0
tsrna-10159	GGCCCGGGTTCGACTCCCGGTATGGGAAC	0	3	0	0

tsrna-10158	GGCCCGGGTTCGACTCCCGGTATGGGA	0	2	0	0
tsrna-10157	GGCCCGGGTTCGACTCCCGGTATGG	0	1	0	0
tsrna-10154	GGCCCCGGGTTCGATCCCCGGCATCTCCAC	1	1	0	1
tsrna-10149	GGCCCCATGGTGTAATGGTTAGCACTCTGGACTTTG	2	3	1	0
tsrna-10148	GGCCCCATGGTGTAATGGTTAGCACTCTGGACT	1	2	2	0
tsrna-10147	GGCCCCATGGTGTAATGGTTAGCACTCTGGAC	1	2	1	0
tsrna-10144	GGCCCCATGGTGTAATGGTTAGCACTCTG	0	1	0	0
tsrna-10141	GGCCCCATGGTGTAATGGTTAGCACT	0	0	0	1
tsrna-10133	GGCCCCATGGTGTAATGGTCAGC	0	1	0	0
tsrna-10127	GGCCCATACCCCGAAAATGTTGGTTATACC	1	0	0	0
tsrna-10116	GGCCCATAACCCAGAGGTCGATGGAT	1	3	0	1
tsrna-10115	GGCCCATAACCCAGAGGTCGATGG	1	1	0	0
tsrna-10114	GGCCCATAACCCAGAGGTCGATG	1	2	0	0
tsrna-10113	GGCCCATAACCCAGAGGTCGAT	0	1	0	0
tsrna-10106	GGCATTGGTGGTTCAGTGGTAGAATTCTCGCCT	4289	621	4999	318
tsrna-10105	GGCATTGGTGGTTCAGTGGTAGAATTCTCG	52	24	54	48
tsrna-10103	GGCATCAGCCTCCGGAGCTGGGGATTGTGG	0	1	0	0
tsrna-10097	GGCATAGCTCAGTGGTAGAGCATTTGACTG	5	2	1	0
tsrna-10096	GGCATAGCTCAGTGGTAGAGCATTTGACT	1	2	2	0
tsrna-10095	GGCAGCGATGGCCGAGTGG	1	3	0	1
tsrna-10092	GGCAGAGCCCGGTAATCGCATA	0	0	1	0
tsrna-10091	GGCAGAGCCCGGTAATCGCA	0	1	0	0
tsrna-10087	GGCAGAGCATTTGACTG	0	0	1	0
tsrna-10085	GGCACGGAGAATTTTGGATTCTCAGGGATG	0	6	1	0
tsrna-10084	GGATTTAGGCTCCAGTCTCTTCGGA	1	0	0	0
tsrna-10078	GGATTCTCAGGGATGGGTTCG	0	1	1	0
tsrna-10077	GGATTCTCAGGGATGGGTTC	1	0	1	0
tsrna-10076	GGATTCGGCGCTCTCACCGCCGCGGCCCGGGT	0	2	1	0
tsrna-10075	GGATTCGGCGCTCTCACCGCCGCGGCCCGG	0	7	0	0
tsrna-10074	GGATTCGGCGCTCTCACCGCCGCGGCCC	0	4	0	0
tsrna-10073	GGATTCGGCGCTCTCACCGCCGCGGCC	1	6	1	1
tsrna-10072	GGATTCGGCGCTCTCACCGCCGCGG	0	0	0	0
tsrna-10071	GGATTCGGCGCTCTCACCGCCGCG	0	1	0	0
tsrna-10070	GGATTCGGCGCTCTCACCGCCGC	0	3	2	0
tsrna-10069	GGATTCGGCGCTCTCACCGCCG	0	1	0	0
tsrna-10068	GGATTCGGCGCTCTCACCGCC	0	3	2	1
tsrna-10067	GGATTCGGCGCTCTCACCGC	0	1	0	0
tsrna-10066	GGATTCGGCGCTCTCACCG	1	3	0	0
tsrna-10065	GGATTCGGCGCTCTCACC	0	1	0	0
tsrna-10062	GGATTCCTGGTTTTCACCCAGGC	0	3	0	0
tsrna-10061	GGATTCCTGGTTTTCACCCAG	2	2	1	0
tsrna-10060	GGATTCCTGGTTTTCACCC	1	1	0	0
tsrna-10059	GGATTCCTGGTTTTCACC	0	1	0	0
tsrna-10056	GGATTAGCTCAAATGGTAGAGCGCTCGCTT	0	1	0	0
tsrna-10051	GGATGTAGCTCAGTGGTAGAGCGCATGCTT	0	1	0	0
tsrna-10048	GGATGTAGCTCAGTGGTAGA	0	0	0	1
tsrna-10047	GGATGTAGCTCAGTGGTAG	0	0	1	0
tsrna-10041	GGATGGGGTGTGATAGGTGGCACGGAGA	0	1	0	0
tsrna-10038	GGATGGGGTGTGATAGGTGGCACGG	0	0	0	0
tsrna-10037	GGATGGGGTGTGATAGGTGGCACG	0	1	0	0
tsrna-10027	GGATGGCCGAGTGGTCTAAGGCGCCAGAC	1	0	0	0
tsrna-10021	GGATGGCCGAGCGGTCTAAGGCGCTGCG	0	2	0	0
tsrna-10020	GGATGGCCGAGCGGTCTAAGGCGCTGC	1	0	0	1
tsrna-10017	GGATGGCCGAGCGGTCTAAGGCG	0	1	0	0
tsrna-10011	GGATGGAGGCGTGGGTTCGAA	0	1	0	0
tsrna-10009	GGATGGAGGCGTGGGTTC	1	0	0	0
tsrna-10006	GGATGCGAGAGGTCCCGGGTT	2	2	0	0
tsrna-10005	GGATGCGAGAGGTCCCGGGT	0	2	0	1
tsrna-10004	GGATCGATGCCCGCATTCTCCACCA	4	3	1	0
tsrna-10003	GGATCGATGCCCGCATCCTCCACCA	1	3	1	0
tsrna-10002	GGATCGATGCCCGCATCCTCCACC	0	1	0	0
tsrna-10001	GGATCGATGCCCGCATCCTCCAC	0	1	0	0
tsrna-10000	GGATCGAAACCATCCTCTGCTACCA	14	22	9	6
tsrna-09999	GGATCGAAACCATCCTCTGCTACC	3	5	2	0

tsrna-09996	GGATCGAAACCATCCTCTGCT	0	0	0	0
tsrna-09993	GGATCAGAAGATTGCAGGTTCGAGTCCTGCCGCGGTCGCCA	1	5	1	1
tsrna-09988	GGATCAGAAGATTGAGGGTTCGAGTCCCTT	3	13	3	1
tsrna-09987	GGATCAGAAGATTGAGGGTTCGAG	6	10	5	0
tsrna-09986	GGATCAGAAGATTGAGGGTTCG	6	7	4	3
tsrna-09985	GGATCAGAAGATTGAGGGTTC	3	10	1	1
tsrna-09984	GGATCAGAAGATTGAGGGTT	4	5	3	2
tsrna-09983	GGATCAGAAGATTGAGGGT	4	2	2	0
tsrna-09981	GGATCAGAAGATTCTAGGTTC	1	8	0	1
tsrna-09979	GGATCAGAAGATTCCAGGTTCG	2	1	1	0
tsrna-09978	GGATCAGAAGATTCCAGGTTC	0	5	2	0
tsrna-09977	GGATAGCTCAGTTGGTAGAGCATCAGAC	0	2	2	2
tsrna-09976	GGATAGCTCAGTCGGTAGAGCATCAGACTTTT	28	18	20	25
tsrna-09975	GGATAGCTCAGTCGGTAGAGCATCAGACTTT	16	10	9	5
tsrna-09974	GGATAGCTCAGTCGGTAGAGCATCAGACTT	8	13	10	1
tsrna-09973	GGATAGCTCAGTCGGTAGAGCATCAGACT	7	10	6	2
tsrna-09972	GGATAGCTCAGTCGGTAGAGCATCAGAC	11	12	6	1
tsrna-09971	GGATAGCTCAGTCGGTAGAGCATCAGA	8	13	4	1
tsrna-09970	GGATAGCTCAGTCGGTAGAGCATCAG	7	6	1	0
tsrna-09969	GGATAGCTCAGTCGGTAGAGCATC	1	5	2	0
tsrna-09967	GGATAGCTCAGTCGGTAGAGCA	1	1	0	0
tsrna-09965	GGATAGCTCAGTCGGTAGAG	0	0	1	0
tsrna-09962	GGATAGCGCATTGGACT	1	2	0	0
tsrna-09961	GGATAGCGCATTGGAC	0	2	0	0
tsrna-09954	GGATAAGGCGTCTGACTTC	0	1	0	0
tsrna-09953	GGATAAGGCGTCTGACTT	0	0	0	0
tsrna-09950	GGATAAGGCATCAGCCTCCGGAGCTGGGGATTGT	0	0	0	2
tsrna-09947	GGATAAGGCATCAGCCTC	0	0	0	0
tsrna-09946	GGATAAGGCATCAGCCT	0	1	0	0
tsrna-09943	GGATAACGCGTCTGACT	0	0	1	0
tsrna-09942	GGATAACAGCTATCCATTGGTCTTAGGCCC	0	1	0	0
tsrna-09941	GGATAACAGCTATCCATTGGTCTTAGGCC	0	0	0	0
tsrna-09940	GGATAACAGCTATCCATTGGTCTTAGGC	1	0	0	0
tsrna-09939	GGATAACAGCTATCCATTGGTCTTAGG	0	2	0	0
tsrna-09938	GGATAACAGCTATCCATTGGTCTTAG	0	0	1	0
tsrna-09937	GGATAACAGCTATCCATTGGTCTTA	1	1	0	0
tsrna-09936	GGATAACAGCTATCCATTGGTCTT	0	1	0	0
tsrna-09935	GGATAACAGCTATCCATTGGTCT	0	0	0	0
tsrna-09929	GGAGTTAAAGACTTTTTCTCTGACCA	30	27	14	15
tsrna-09928	GGAGTTAAAGACTTTTTCTCTGACC	5	8	1	2
tsrna-09927	GGAGTTAAAGACTTTTTCTCTGAC	0	2	0	0
tsrna-09926	GGAGTTAAAGACTTTTTCTCTGA	0	0	0	0
tsrna-09925	GGAGTTAAAGACTTTTTCTCTG	0	0	0	0
tsrna-09920	GGAGGCGTGGGTTCGAATC	1	0	0	0
tsrna-09917	GGAGCTTAAACCCCCTTATTTCTACCA	2	4	0	1
tsrna-09916	GGAGCTTAAACCCCCTTATTTCTACC	0	4	1	0
tsrna-09915	GGAGCTTAAACCCCCTTATTTCTAC	1	0	1	0
tsrna-09914	GGAGCTTAAACCCCCTTATTTCTA	1	0	0	0
tsrna-09913	GGAGCTTAAACCCCCTTATTTCT	1	2	0	0
tsrna-09912	GGAGCTTAAACCCCCTTATTTC	0	2	0	1
tsrna-09911	GGAGCTTAAACCCCCTTATTT	0	1	1	0
tsrna-09905	GGAGCTGGGGATTGTGGGTTCGAGTCCCATCTGGGTCGCCA	3	25	3	0
tsrna-09904	GGAGCTGGGGATTGTGGGTTCGAGTCCC	2	2	0	0
tsrna-09903	GGAGCTGGGGATTGTGGGTTCG	0	2	0	0
tsrna-09902	GGAGCTGGGGATTGTGGGTTC	1	1	0	0
tsrna-09901	GGAGCTGGGGATTGTGGGTT	0	2	0	0
tsrna-09900	GGAGCTGGGGATTGTGGGT	0	1	2	0
tsrna-09896	GGAGATTTCAACTTAACTTGACCGCTCTGACCA	90	60	74	12
tsrna-09895	GGAGATTTCAACTTAACTTGACCGCTCTGACC	10	3	14	1
tsrna-09894	GGAGATTTCAACTTAACTTGACCGCTCTGAC	2	2	0	0
tsrna-09893	GGAGATTTCAACTTAACTTGACCGCTCTGA	5	1	3	0
tsrna-09892	GGAGATTTCAACTTAACTTGACCGCTCTG	0	1	1	0
tsrna-09891	GGAGATTTCAACTTAACTTGACCGCTCT	1	0	0	0
tsrna-09887	GGAGATTTCAACTTAACTTGACCG	0	1	0	0

tsrna-09886	GGAGATTTCAACTTAACTTGACC	0	0	2	0
tsrna-09883	GGAGATTTCAACTTAACTTG	0	1	0	0
tsrna-09879	GGAGATGAAAACCTTTTTCCAAGGACACCA	1	3	2	1
tsrna-09878	GGAGATGAAAACCTTTTTCCAAGGACACC	0	1	1	0
tsrna-09877	GGAGATGAAAACCTTTTTCCAAGGACAC	0	0	0	0
tsrna-09864	GGAGATCCTGGGTTCGAAT	2	7	1	2
tsrna-09863	GGAGATCCTGGGTTCG	4	4	2	2
tsrna-09856	GGAGACCGGGGTTCGATTCCCCGACGGGGAGCCA	3	20	1	1
tsrna-09855	GGAGACCGGGGTTCGATTCCCCGACGGGGAGCC	0	2	0	0
tsrna-09854	GGAGACCGGGGTTCGATTCCCCGACGGGGAGC	0	3	0	0
tsrna-09853	GGAGACCGGGGTTCGATTCCCCGACGGGG	0	0	0	0
tsrna-09839	GGAGAATTTTGGATTCTCAGGGA	0	1	0	0
tsrna-09837	GGACTTTGAATCCAGCGATCCGAGT	0	1	2	0
tsrna-09836	GGACTTTGAATCCAGCGATCCGAG	1	1	3	1
tsrna-09835	GGACTTTGAATCCAGCGATCCGA	2	1	0	0
tsrna-09834	GGACTTTGAATCCAGCGATCCG	0	2	0	0
tsrna-09833	GGACTTTGAATCCAGCGATCC	4	2	0	1
tsrna-09832	GGACTTTGAATCCAGCGATC	1	5	0	0
tsrna-09828	GGACTGTAGATCCTTAGGTCGCTGGT	0	1	0	0
tsrna-09822	GGACTGTAAATCTAAAGACAGGGGTTAGGC	0	0	1	0
tsrna-09814	GGACTGCTAATCCATTGTGCTCTG	0	0	2	0
tsrna-09813	GGACTCTTAATCCCAGGGTCGTGGGTTC	1	1	0	0
tsrna-09812	GGACTCTTAATCCCAGGGTCGTGGGTT	0	3	1	0
tsrna-09811	GGACTCTTAATCCCAGGGTCGTGGG	0	1	1	1
tsrna-09810	GGACTCTTAATCCCAGGGTCGTGG	0	1	0	0
tsrna-09809	GGACTCTTAATCCCAGGG	1	0	0	0
tsrna-09805	GGACTCTGAATCCAGCGATCCGAGTTCAAG	0	2	0	0
tsrna-09804	GGACTCTGAATCCAGCGATCCGAGTTCAA	0	1	0	0
tsrna-09803	GGACTCTGAATCCAGCGATCCGAGTTC	1	0	0	0
tsrna-09802	GGACTCTGAATCCAGCGATCCGAGTT	0	0	1	0
tsrna-09800	GGACTCTGAATCCAGCGATCCGAG	0	1	1	0
tsrna-09799	GGACTCTGAATCCAGCGATCCGA	0	1	0	0
tsrna-09798	GGACTCTGAATCCAGCGATCCG	0	1	0	0
tsrna-09797	GGACTCTGAATCCAGCGATCC	0	2	0	0
tsrna-09789	GGAAGCGTGCTGGGCCCATAACCCAGA	0	1	0	3
tsrna-09788	GGAAGCGTGCTGGGCCCATAACCCAG	1	5	0	2
tsrna-09787	GGAAGCGTGCTGGGCCCATAACCC	1	2	0	1
tsrna-09786	GGAAGCGTGCTGGGCCCATAAC	0	2	0	0
tsrna-09785	GGAAGCGTGCTGGGCCCATA	0	2	0	0
tsrna-09784	GGAAGCGTGCTGGGCCCAT	0	3	1	0
tsrna-09783	GGAAGCGTGCTGGGCCCA	0	2	0	0
tsrna-09782	GGAAGCGTGCTGGGCCC	2	3	0	0
tsrna-09781	GGAAGCGTGCTGGGCC	0	4	1	1
tsrna-09780	GGAAAAAGTCATGGAGGCCATGGGGTT	84	326	55	21
tsrna-09779	GGAAAAAGTCATGGAGGCCATGGGG	26	100	17	8
tsrna-09778	GCTTTGGGTGCTAATGGTGGAGTTAAAGACTTTTTCTCTGACC	25	64	21	18
tsrna-09777	GCTTTGGGTGCTAATGGTGGAGTTAAAGACTTTTTCTCTGACC	16	22	11	0
tsrna-09776	GCTTTGGGTGCTAATGGTGGAGTTAAAGACTTTTTCTCTGAC	12	16	5	1
tsrna-09775	GCTTTGGGTGCTAATGGTGGAGTTAAAGACTTTTTC	5	13	4	2
tsrna-09774	GCTTTGGGTGCTAATGGTGGAGTTAAAGAC	1	8	0	0
tsrna-09773	GCTTTGGGTGCTAATGGTGGAGTTAAAGA	4	5	1	0
tsrna-09772	GCTTTGGGTGCTAATGGTGGAGTTAAAG	1	4	1	1
tsrna-09771	GCTTTGGGTGCTAATGGTGGAGTTAAA	3	0	1	0
tsrna-09770	GCTTTGGGTGCTAATGGTGGAGTTAA	2	3	2	0
tsrna-09769	GCTTTGGGTGCTAATGGTGGAGTTA	1	3	2	0
tsrna-09768	GCTTTGGGTGCTAATGGTGGAGTT	1	1	1	0
tsrna-09760	GCTTTGGGTGCGAGAGGTCCCGG	0	1	1	0
tsrna-09759	GCTTTGGGTGCGAGAGGTCCCG	0	1	0	0
tsrna-09758	GCTTTGGGTGCGAGAGGTCCC	0	1	0	1
tsrna-09757	GCTTTGGGTGCGAGAGGTCC	0	2	0	0
tsrna-09754	GCTTTGGGGGGTTCGATTCCTTCCTTTTTTGCCA	0	10	4	3
tsrna-09753	GCTTTGGGGGGTTCGATTCCTTCCTTTTTT	3	4	1	1
tsrna-09752	GCTTTGGGGGGTTCGATTCCTTCC	1	0	0	0
tsrna-09751	GCTTTGGGGGGTTCGATTCCTTC	0	0	1	0

tsrna-09750	GCTTTGGGGGGTTCGATTCCTT	0	0	0	1
tsrna-09749	GCTTTGGGGGGTTCGATTCCT	0	1	0	1
tsrna-09748	GCTTTGGGGGGTTCGATTCC	0	0	1	1
tsrna-09747	GCTTTGGGGGGTTCGATTC	0	0	1	0
tsrna-09746	GCTTTGGGGGGTTCGATT	0	1	0	0
tsrna-09743	GCTTTGCATGTATGAGGCCCCGGGT	2	7	1	0
tsrna-09740	GCTTTGCACGTATGAGGCCCCGGGTT	40	45	22	30
tsrna-09739	GCTTTGCACGTATGAGGCCCCGGGT	24	34	14	26
tsrna-09738	GCTTTGCACGTATGAGGCCCCGGG	1	2	0	0
tsrna-09737	GCTTTGCACGTATGAGGCCCCGG	1	2	0	0
tsrna-09736	GCTTTGCACGTATGAGGCCCCG	1	1	0	0
tsrna-09735	GCTTTGCACGTATGAGGCCCC	1	0	0	1
tsrna-09732	GCTTTGCACGCGTGGGTT	0	6	1	0
tsrna-09731	GCTTTGCACGCGTGGGT	0	2	0	0
tsrna-09730	GCTTTCACCGCCGCGGCCCGGGT	1	8	0	1
tsrna-09729	GCTTTCACCGCCGCGGCCCGG	3	0	0	5
tsrna-09728	GCTTTCACCGCCGCGGCCCG	1	3	0	3
tsrna-09727	GCTTTCACCGCCGCGGCCC	1	3	0	2
tsrna-09726	GCTTTCACCGCCGCGGCC	2	0	0	0
tsrna-09725	GCTTTCACCGCCGCGGC	2	4	1	1
tsrna-09724	GCTTTCACCGCCGCGG	2	3	0	0
tsrna-09723	GCTTTACACGCAGAAGGTCCTGGGT	9	44	3	0
tsrna-09722	GCTTTACACGCAGAAGGTCCTGG	1	0	0	0
tsrna-09721	GCTTTACACGCAGAAGGTCCTG	2	0	0	0
tsrna-09718	GCTTCTGTAGTGTAGTGGTTATCACGTTCGCCTCACACGCGA/	337	147	335	68
tsrna-09717	GCTTCTGTAGTGTAGTGGTTATCACGTTCGCCTCACACG	356	107	273	68
tsrna-09716	GCTTCTGTAGTGTAGTGGTTATCACGTTCGCCTCACA	358	123	277	62
tsrna-09715	GCTTCTGTAGTGTAGTGGTTATCACGTTCGCCTCAC	374	127	315	65
tsrna-09714	GCTTCTGTAGTGTAGTGGTTATCACGTTCGCCTCA	336	141	265	68
tsrna-09713	GCTTCTGTAGTGTAGTGGTTATCACGTTCGCCTC	345	135	292	61
tsrna-09712	GCTTCTGTAGTGTAGTGGTTATCACGTTCGCCT	298	106	263	72
tsrna-09711	GCTTCTGTAGTGTAGTGGTTATCACGTTCGCC	254	103	217	58
tsrna-09710	GCTTCTGTAGTGTAGTGGTTATCACGTTCGC	80	58	61	46
tsrna-09709	GCTTCTGTAGTGTAGTGGTTATCACGTTCG	31	7	29	31
tsrna-09708	GCTTCTGTAGTGTAGTGGTTATCACGTTC	38	8	19	35
tsrna-09707	GCTTCTGTAGTGTAGTGGTTATCACGTT	27	12	28	26
tsrna-09706	GCTTCTGTAGTGTAGTGGTTATCACGT	38	7	23	27
tsrna-09705	GCTTCTGTAGTGTAGTGGTTATCACG	24	4	23	29
tsrna-09704	GCTTCTGTAGTGTAGTGGTTATCAC	33	8	27	20
tsrna-09703	GCTTCTGTAGTGTAGTGGTTATCA	29	5	12	16
tsrna-09702	GCTTCTGTAGTGTAGTGGTTATC	22	6	16	21
tsrna-09701	GCTTCTGTAGTGTAGTGGTTAT	1	0	0	0
tsrna-09700	GCTTCTGTAGTGTAGTGGTTA	2	0	0	0
tsrna-09699	GCTTCTGTAGTGTAGTGGTT	0	0	0	1
tsrna-09698	GCTTCTGTAGTGTAGTGGT	0	1	0	0
tsrna-09692	GCTTCGCATGTATGAGGTCCCGGGT	4	3	1	0
tsrna-09691	GCTTCGCATGTATGAGGCCCCGGGT	4	5	1	0
tsrna-09690	GCTTCAAACCTGCCGGGGCTTCCA	5	2	3	0
tsrna-09689	GCTTCAAACCTGCCGGGGCTTCC	1	0	0	0
tsrna-09688	GCTTCAAACCTGCCGGGGCTTC	0	0	1	0
tsrna-09681	GCTTAGTTGGTTAAAGCGCCTGTCT	4	1	1	5
tsrna-09680	GCTTAGTTGGTTAAAGCGCCTGT	3	0	1	2
tsrna-09675	GCTTACGACCCCTTATTTACCCCA	2	2	0	0
tsrna-09674	GCTTACGACCCCTTATTTACCCC	0	0	1	0
tsrna-09666	GCTTACCTCCTCAAAGCAATACACTG	2	1	3	0
tsrna-09665	GCTTACCTCCTCAAAGCAATACACT	3	2	1	1
tsrna-09664	GCTTACCTCCTCAAAGCAATACAC	1	0	0	0
tsrna-09663	GCTTACCTCCTCAAAGCAATACA	0	0	3	0
tsrna-09661	GCTTACCTCCTCAAAGCAATA	1	0	0	0
tsrna-09659	GCTTACCTCCTCAAAGC	1	0	0	0
tsrna-09657	GCTTAATTAAAGTGGCTGATTTGC	0	0	2	0
tsrna-09656	GCTTAATTAAAGTGGCTGA	0	0	0	0
tsrna-09654	GCTTAACACAAAGCACCCAACTTACACTT	0	0	0	1
tsrna-09646	GCTTAAACCCCCTTATTTCTACCA	1	0	0	0

tsrna-09645	GCTTAAACCCCCTTATTTCTACC	1	4	0	0
tsrna-09644	GCTTAAACCCCCTTATTTCTAC	1	2	0	0
tsrna-09643	GCTTAAACCCCCTTATTTCTA	1	0	0	0
tsrna-09642	GCTTAAACCCCCTTATTTCT	1	2	1	0
tsrna-09641	GCTTAAACCCCCTTATTTC	0	1	0	0
tsrna-09640	GCTTAAACCCCCTTATTT	1	0	0	0
tsrna-09637	GCTGTTAACTGAAAGGTTGGTGGT	1	1	0	0
tsrna-09636	GCTGTTAACTAAGTGTTTGTGGGTTTAAGTCCCATTGGTCTAG	8	3	3	2
tsrna-09635	GCTGTTAACTAAGTGTTTGTGGGTTTAAGTCCCATTGGTCTAG	1	3	1	1
tsrna-09634	GCTGTTAACTAAGTGTTTGTGGGTTTAAGTCCCATTGGTCTAG	7	1	5	0
tsrna-09633	GCTGTTAACTAAGTGTTTGTGGGTTTAAGT	0	0	0	0
tsrna-09632	GCTGTTAACTAAGTGTTTGTGGGTTT	0	1	2	0
tsrna-09631	GCTGTTAACTAAGTGTTTGTGGGTT	0	1	1	0
tsrna-09630	GCTGTTAACTAAGTGTTTGTGGGT	0	0	1	0
tsrna-09624	ACCTTTTTCCAAGGACACCA	2	0	5	0
tsrna-09623	ACCTTTTTCCAAGGACACC	1	0	1	0
tsrna-09622	ACCTTTTAAGTTAAAGATTAAGAGAACCAACACCTCTTTACAG	0	0	7	0
tsrna-09621	ACCTTTTAAGTTAAAGATTAAGAGAACCA	0	0	1	0
tsrna-09620	ACCTTTTAAGTTAAAGATTAAGAGAACC	0	2	0	0
tsrna-09619	ACCTTCGATAGCTCAGTTGGTAGAGC	14	11	8	2
tsrna-09618	ACCTTCGATAGCTCAGCTGGTAGAGC	55	32	26	21
tsrna-09617	ACCTGCCGGGGCTTCCA	1	0	1	0
tsrna-09616	ACCTGCCGGGGCTTCC	0	0	0	0
tsrna-09614	ACCTCTTTACAGTGACC	0	0	0	0
tsrna-09608	ACCTCGTGGCGCAACGGTAGCGCGTCTGAC	0	1	0	0
tsrna-09602	ACCTCCTCAAAGCAATACACT	1	0	0	0
tsrna-09598	ACCTCCTCAAAGCAATA	0	0	0	1
tsrna-09597	ACCTCAGAGGGGGCACCA	1	2	0	0
tsrna-09596	ACCGTTAACTTCCAATTAACTAGTTTTGAC	0	0	0	0
tsrna-09595	ACCGGGGTTCGATTCCCCGACGGGGAGCCA	2	17	0	0
tsrna-09594	ACCGGGGTTCGATTCCCCGACGGGGAGCC	0	1	0	0
tsrna-09593	ACCGGGGTTCGATTCCCCGACGGGGAGC	0	0	0	0
tsrna-09592	ACCGGGGTTCGATTCCCCGACGGGGAG	1	0	0	0
tsrna-09591	ACCGGGGTTCGATTCCCCGACGGGGA	0	1	1	0
tsrna-09589	ACCGGGGTTCGATTCCCCGACGGG	0	1	0	0
tsrna-09586	ACCGGGCGGAAACACCA	167	386	49	17
tsrna-09585	ACCGGGCGGAAACACC	33	95	19	3
tsrna-09584	ACCGGGCAGAAGCACCA	12	55	7	1
tsrna-09583	ACCGGGCAGAAGCACC	0	5	0	0
tsrna-09582	ACCGGGATGGCCGAGTGGTTAAGGC	1	20	1	15
tsrna-09581	ACCGGAGATGAAAACCTTTTTCCAAGGACACCA	28	32	60	2
tsrna-09580	ACCGGAGATGAAAACCTTTTTCCAAGGACACC	3	11	9	0
tsrna-09579	ACCGGAGATGAAAACCTTTTTCCAAGGACA	3	9	4	0
tsrna-09578	ACCGGAGATGAAAACCTTTTTCCAAGGAC	1	5	2	0
tsrna-09577	ACCGGAGATGAAAACCTTTTTCCAAGG	2	6	1	0
tsrna-09576	ACCGGAGATGAAAACCTTTTTCCAAG	0	5	0	0
tsrna-09575	ACCGGAGATGAAAACCTTTTTCCAA	1	3	3	0
tsrna-09574	ACCGGAGATGAAAACCTTTTTCCA	3	5	0	0
tsrna-09573	ACCGGAGATGAAAACCTTTTTCC	2	3	0	0
tsrna-09572	ACCGGAGATGAAAACCTT	0	0	0	0
tsrna-09571	ACCGGAGATGAAAACCT	0	0	0	0
tsrna-09569	ACCGCGTGGCCTAATGGATAAGGCGTCTGA	0	1	1	0
tsrna-09564	ACCGCCGCGGCCCGGGTTCGATTCCCGGTCAGGGAACCA	33	309	17	4
tsrna-09563	ACCGCCGCGGCCCGGGTTCGATTCCCGGTCAGGGAACC	4	14	3	1
tsrna-09562	ACCGCCGCGGCCCGGGTTCGATTCCCGGTCAGGGAAC	2	8	2	0
tsrna-09561	ACCGCCGCGGCCCGGGTTCGATTCCCGGTCAGGGAA	1	0	0	0
tsrna-09560	ACCGCCGCGGCCCGGGTTCGATTCCCGGTCAGGGA	0	1	0	0
tsrna-09559	ACCGCCGCGGCCCGGGTTCGATTCCCGGTCAGGG	0	1	0	0
tsrna-09558	ACCGCCGCGGCCCGGGTTCGATTCCCGGTCAGG	0	1	0	0
tsrna-09556	ACCGCCGCGGCCCGGGTTCGATTCCCGGTC	2	2	0	0
tsrna-09555	ACCGCCGCGGCCCGGGTTCGATTCCCGGT	0	0	0	1
tsrna-09550	ACCGCCGCGGCCCGGGTTCG	0	0	0	0
tsrna-09545	ACCGAGCGGAAACACCA	1	0	0	0
tsrna-09544	ACCGAAGATCGCGGGTTCGAACCCCGTCCGTGCCTCCA	5	18	3	1

tsrna-09543	ACCGAAGATCGCGGGTTCGAACCCCGTCCGTGCCTCC	3	14	0	0
tsrna-09542	ACCGAAGATCGCGGGTTCGAACCCCGTCCGTGCCTC	2	8	0	1
tsrna-09541	ACCGAAGATCGCGGGTTCGAACCCCGTCCG	2	7	1	0
tsrna-09540	ACCGAAGATCGCGGGTTCGA	0	5	2	0
tsrna-09539	ACCGAAGATCGCGGGTTCG	3	6	3	1
tsrna-09538	ACCGAAGATCGCGGGT	3	3	1	0
tsrna-09537	ACCGAAGATCACGGGTTCGAACCCCGTCCGTGCCTCCA	6	8	6	2
tsrna-09536	ACCGAAGATCACGGGTTCGAACCCCGTCCGTGCCTC	0	0	0	0
tsrna-09535	ACCGAAGATCACGGGTTCGAACCCCGTCCGTGCCT	0	3	3	0
tsrna-09534	ACCGAAGATCACGGGTTCGAACCCCGTCCG	2	3	0	0
tsrna-09533	ACCGAAAGGTTGGTGGTTCG	0	0	0	0
tsrna-09532	ACCGAAAGGTTGGTGGTT	1	1	0	0
tsrna-09528	ACCCTGGACTCTGAATCCAGC	0	1	0	0
tsrna-09525	ACCCTGCTCGCTGCGCCA	16	32	2	0
tsrna-09524	ACCCTGCTCGCTGCGCC	2	4	0	0
tsrna-09522	ACCCCTTATTTACCCCA	0	0	1	0
tsrna-09520	ACCCCGTCCGTGCCTCCA	4	5	2	2
tsrna-09517	ACCCCGTACGGCCACCA	2	3	0	1
tsrna-09516	ACCCCGAAAATGTTGGTTATACCCTTCCCGTACTAC	0	0	0	0
tsrna-09515	ACCCCGAAAATGTTGGTTATACCCTTCCCG	0	0	1	0
tsrna-09513	ACCCCCTTATTTCTACCA	0	0	0	1
tsrna-09511	ACCCCCTTATTTCTAC	0	1	0	0
tsrna-09509	ACCCCACTCTCGGTACCA	0	3	0	2
tsrna-09505	ACCCAGTGGCCTAATGGATAAGGCATCAGC	3	1	0	1
tsrna-09504	ACCCAGTGGCCTAATGGATAAGGCATC	2	1	1	0
tsrna-09500	ACCCAGGTGGCCCGGGTTCGACTCCCGGTATGGGAACCA	17	73	12	3
tsrna-09499	ACCCAGGTGGCCCGGGTTCGACTCCCGGTATGGGAACC	2	8	1	0
tsrna-09498	ACCCAGGTGGCCCGGGTTCGACTCCCGGTATGGGAAC	2	5	0	0
tsrna-09497	ACCCAGGTGGCCCGGGTTCGACTCCCGGTATGGGAA	0	2	0	0
tsrna-09495	ACCCAGGTGGCCCGGGTTCGACTCCCGGT	0	1	0	0
tsrna-09491	ACCCAGGCGGCCCGGGTTCGACTCCCGGTGTGGGAACCA	88	330	44	33
tsrna-09490	ACCCAGGCGGCCCGGGTTCGACTCCCGGTGTGGGAACC	3	19	8	2
tsrna-09489	ACCCAGGCGGCCCGGGTTCGACTCCCGGTGTGGGAAC	1	3	5	0
tsrna-09488	ACCCAGGCGGCCCGGGTTCGACTCCCGGTGTGGGAA	0	2	0	0
tsrna-09487	ACCCAGGCGGCCCGGGTTCGACTCCCGGTGTGGGA	0	2	0	0
tsrna-09486	ACCCAGGCGGCCCGGGTTCGACTCCCGGTGTGGG	0	2	0	0
tsrna-09478	ACCCAGGCGGCCCGGGTTCGA	0	1	0	0
tsrna-09475	ACCCAGAGGTCGATGGATCGAAACC	0	2	0	0
tsrna-09474	ACCCAGAGGTCGATGGATCGAAAC	0	0	0	1
tsrna-09473	ACCCAGAGGTCGATGGATCGAAA	1	2	0	1
tsrna-09472	ACCCAGAGGTCGATGGATCGAA	0	1	0	1
tsrna-09471	ACCCAGAGGTCGATGGATCGA	2	4	1	0
tsrna-09470	ACCCAGAGGTCGATGGATCG	1	3	0	0
tsrna-09469	ACCCAGAGGTCGATGGATC	0	3	0	0
tsrna-09468	ACCCAGAGGTCGATGGAT	0	3	0	0
tsrna-09467	ACCCAGAGGTCGATGGA	0	4	0	0
tsrna-09466	ACCCAGAGGTCGATGG	0	0	1	0
tsrna-09465	ACCCACCCAGAGGCGCCA	1	0	0	0
tsrna-09464	ACCCAACTTACACTTAGGAGATTTCAACTTAACTTGACCGCTC	3	3	19	1
tsrna-09463	ACCCAACTTACACTTAGGAGATTTCAACTTAACTTGACCGCTC	9	2	13	0
tsrna-09462	ACCCAACTTACACTTAGGAGATTTCAACTTAACTTGACCGCTC	3	2	12	0
tsrna-09461	ACCCAACTTACACTTAGGAGATTTCAACTT	1	0	0	0
tsrna-09460	ACCCAACTTACACTTAGGAGATTTCAACT	0	0	0	0
tsrna-09456	ACCCAACTTACACTTAG	0	0	0	0
tsrna-09455	ACCATTTCATAACTTTGTCAAAGTT	4	3	4	2
tsrna-09454	ACCATTTCATAACTTTGTC	0	0	3	0
tsrna-09453	ACCATCCTCTGCTACCA	4	1	1	1
tsrna-09452	ACCATCCTCTGCTACC	0	1	0	0
tsrna-09451	ACCAGGGGTCGCGAGTTCAA	0	1	0	0
tsrna-09450	ACCAGGGGTCGCGAGTTCA	3	1	0	0
tsrna-09449	ACCAGGGGTCGCGAGTTC	0	2	0	0
tsrna-09448	ACCAGGGGTCGCGAGTT	1	1	0	0
tsrna-09447	ACCAGGGGTCGCGAGT	0	0	0	0
tsrna-09446	ACCAGGCGGAAACACCA	3	1	1	0

tsrna-09445	ACCAGGATGGCCGAGTGGTTAAGGCGTTGG	1	9	3	0
tsrna-09444	ACCAGGATGGCCGAGTGGTTAAGGCG	6	6	5	1
tsrna-09443	ACCAGGATGGCCGAGTGGTTAAGGC	3	13	5	1
tsrna-09442	ACCAGGATGGCCGAGTGGTTAAGG	2	10	3	2
tsrna-09441	ACCAGGATGGCCGAGTGGTTAAG	2	5	2	1
tsrna-09440	ACCAGGATGGCCGAGTGGTTAA	2	9	0	1
tsrna-09439	ACCAGGATGGCCGAGTGGTTA	3	4	1	0
tsrna-09438	ACCAGGATGGCCGAGTGGTT	0	8	1	0
tsrna-09437	ACCAGGATGGCCGAGTGGT	0	4	1	0
tsrna-09436	ACCAGGATGGCCGAGTGG	1	4	0	0
tsrna-09435	ACCAGGATGGCCGAGTG	3	3	0	1
tsrna-09434	ACCAGGATGGCCGAGT	1	2	1	0
tsrna-09433	ACCAGCTTTGGGGGGTTCG	0	0	0	0
tsrna-09432	ACCAGCTTTGGGGGGTTC	0	0	0	0
tsrna-09431	ACCAGCTTTGGGGGGTT	1	0	0	0
tsrna-09430	ACCAGAATGGCCGAGTGGTTAAGGC	0	5	0	1
tsrna-09429	ACCAGAATGGCCGAGTGGTTAAGG	2	1	0	0
tsrna-09428	ACCAGAATGGCCGAGTGGTTAAG	1	4	0	0
tsrna-09427	ACCAGAATGGCCGAGTGGTTAA	3	3	0	0
tsrna-09426	ACCAGAATGGCCGAGTGGTTA	0	0	3	0
tsrna-09425	ACCAGAATGGCCGAGTGGTT	1	0	1	0
tsrna-09424	ACCAGAATGGCCGAGTGG	1	1	1	0
tsrna-09423	ACCAGAATGGCCGAGTG	0	2	1	0
tsrna-09422	ACCAGAATGGCCGAGT	1	1	1	0
tsrna-09407	ACCAAAACATCAGATTGTGAATCTGACAAC	1	3	2	0
tsrna-09406	ACCAAAACATCAGATTGTGAATCTGACA	0	1	1	0
tsrna-09399	ACATGGTCTAGCGGTTAGGATTCCTGGTTT	6	7	5	2
tsrna-09398	ACATGGTCTAGCGGTTAGGATTCCTGGTT	2	7	2	1
tsrna-09397	ACATGGTCTAGCGGTTAGGATTCCTGGT	4	6	3	4
tsrna-09396	ACATGGTCTAGCGGTTAGGATTCCTGG	3	3	3	2
tsrna-09395	ACATGGTCTAGCGGTTAGGATTCCTG	3	0	2	0
tsrna-09394	ACATGGTCTAGCGGTTAGGATTCCT	1	0	2	0
tsrna-09393	ACATGGTCTAGCGGTTAGGATTCC	0	0	0	0
tsrna-09390	ACATGGTCTAGCGGTTAGGAT	1	0	0	0
tsrna-09386	ACATGGCTTTCTCACC	0	0	0	0
tsrna-09385	ACATCAGATTGTGAATCTGACAACAGAGGCTTACGA	3	8	6	0
tsrna-09384	ACATCAGATTGTGAATCTGACAACAGAGGC	2	6	1	0
tsrna-09383	ACATCAGATTGTGAATCTGACAACAGAGG	1	3	4	0
tsrna-09382	ACATCAGATTGTGAATCTGACAACAGAG	2	4	2	0
tsrna-09381	ACATCAGATTGTGAATCTGACAACAGA	2	1	4	0
tsrna-09380	ACATCAGATTGTGAATCTGACAACAG	2	3	2	0
tsrna-09379	ACATCAGATTGTGAATCTGACAACA	0	1	2	0
tsrna-09378	ACATCAGATTGTGAATCTGACAAC	2	0	1	0
tsrna-09377	ACATCAGATTGTGAATCTGACAA	1	0	1	0
tsrna-09376	ACATCAGATTGTGAATCTGACA	0	2	0	0
tsrna-09371	ACATCACCCCATAAACACCA	0	2	0	1
tsrna-09366	ACATATGTCCGCGTGGGTT	14	15	4	2
tsrna-09365	ACATATGTCCGCGTGGGT	8	8	1	0
tsrna-09364	ACATATGTCCGCGTGGG	2	0	0	0
tsrna-09363	ACAGGTTCGAATCCTGTTCGTGACGC	0	0	1	0
tsrna-09362	ACAGGGGTTAGGCCTCTTTTTACCACCA	1	1	1	1
tsrna-09361	ACAGGGGTTAGGCCTCTTTTTACCACC	1	2	1	0
tsrna-09360	ACAGGGGTTAGGCCTCTTTTTACCAC	2	0	1	0
tsrna-09359	ACAGGGGTTAGGCCTCTTTTTACCA	0	0	0	0
tsrna-09357	ACAGGAGATCCTGGGTTCGACTCCCAGCGG	10	16	4	3
tsrna-09356	ACAGGAGATCCTGGGTTCGAATCCCAGCGGTGCCTCCA	40	76	14	3
tsrna-09355	ACAGGAGATCCTGGGTTCGAATCCCAGCGGTGCCTCC	8	26	8	1
tsrna-09354	ACAGGAGATCCTGGGTTCGAATCCCAGCGGTGCCTC	7	21	8	2
tsrna-09353	ACAGGAGATCCTGGGTTCGAATCCCAGCGGTGCCT	10	23	3	1
tsrna-09352	ACAGGAGATCCTGGGTTCGAATCCCAGCGGTGCC	7	29	6	3
tsrna-09351	ACAGGAGATCCTGGGTTCGAATCCCAGCGGTGC	7	20	10	0
tsrna-09350	ACAGGAGATCCTGGGTTCGAATCCCAGCGGGGCCTCCA	7	31	4	1
tsrna-09349	ACAGGAGATCCTGGGTTCGAATCCCAGCGGGGCCTCC	8	28	4	6
tsrna-09348	ACAGGAGATCCTGGGTTCGAATCCCAGCGGGGCCTC	7	24	2	1

tsrna-09347	ACAGGAGATCCTGGGTTCGAATCCCAGCGGGGCCT	5	19	3	1
tsrna-09346	ACAGGAGATCCTGGGTTCGAATCCCAGCGGGGCC	6	16	3	3
tsrna-09345	ACAGGAGATCCTGGGTTCGAATCCCAGCGG	8	26	3	4
tsrna-09344	ACAGCTATCCATTGGTCTTAGGCCCCA	0	0	4	0
tsrna-09343	ACAGCTATCCATTGGTCTTAGGCCCC	0	1	0	0
tsrna-09340	ACAGCTATCCATTGGTCTTAGGC	0	0	1	0
tsrna-09339	ACAGCTATCCATTGGTCTTAGG	0	0	0	0
tsrna-09332	ACAGAGGCTTACGACCCCTTATTTACCCC	1	0	1	0
tsrna-09331	ACAGAGGCTTACGACCCCTTATTTACCC	0	0	1	0
tsrna-09323	ACAGAGAATAGTTTAAATTAGAATCTTAGC	93	7	20	11
tsrna-09322	ACAGACCAAGAGCCTTCAAAGCCC	7	3	1	0
tsrna-09321	ACAGACCAAGAGCCTTCAAAGCC	6	6	4	0
tsrna-09320	ACAGACCAAGAGCCTTCAAAGC	3	6	2	0
tsrna-09319	ACAGACCAAGAGCCTTCAAAG	6	4	1	0
tsrna-09318	ACAGACCAAGAGCCTTCA	1	1	0	0
tsrna-09317	ACAGACCAAGAGCCTTC	0	1	0	0
tsrna-09316	ACACTTAGGAGATTTCAACTTAACTTGACCGCTCTGACCA	99	42	100	16
tsrna-09315	ACACTTAGGAGATTTCAACTTAACTTGACCGCTCTGACC	10	12	7	3
tsrna-09314	ACACTTAGGAGATTTCAACTTAACTTGACCGCTCTGAC	3	5	4	0
tsrna-09313	ACACTTAGGAGATTTCAACTTAACTTGACCGCTCTG	1	1	2	1
tsrna-09312	ACACTTAGGAGATTTCAACTTAACTTGACCGCT	0	1	3	0
tsrna-09311	ACACTTAGGAGATTTCAACTTAACTTGACC	0	0	1	0
tsrna-09310	ACACTTAGGAGATTTCAACTTAACTTGAC	1	1	2	0
tsrna-09309	ACACTTAGGAGATTTCAACTTAACT	0	0	0	0
tsrna-09308	ACACTTAGGAGATTTCAACTTA	0	0	0	0
tsrna-09307	ACACTTAGGAGATTTCAACTT	0	0	0	0
tsrna-09302	ACACTGAAAATGTTTAGACGGGCTCACATCACCCCATAAACA(	185	56	100	30
tsrna-09301	ACACTGAAAATGTTTAGACGGGCTCACATCACCCCATAAACA	114	58	62	9
tsrna-09300	ACACTGAAAATGTTTAGACGGGCTCACATCACC	88	40	48	6
tsrna-09299	ACACTGAAAATGTTTAGACGGGCTCACAT	43	30	13	3
tsrna-09298	ACACTGAAAATGTTTAGACGGGCTCACA	42	30	20	3
tsrna-09297	ACACTGAAAATGTTTAGACGGGCTCAC	16	14	4	3
tsrna-09296	ACACTGAAAATGTTTAGACGGGCTCA	14	12	3	5
tsrna-09295	ACACTGAAAATGTTTAGACGGGCTC	9	4	0	0
tsrna-09294	ACACTGAAAATGTTTAGACGGGCT	0	3	0	0
tsrna-09293	ACACTGAAAATGTTTAGACGGGC	0	3	0	0
tsrna-09292	ACACTGAAAATGTTTAGACGGG	0	2	0	0
tsrna-09291	ACACTGAAAATGTTTAGACGG	0	0	2	0
tsrna-09288	ACACGCGAAAGGTCCCCGGTTT	0	4	1	0
tsrna-09287	ACACGCGAAAGGTCCCCGGTTCGAAACCGGGCGGAAACACC	72	185	28	6
tsrna-09286	ACACGCGAAAGGTCCCCGGTTCGAAACCGGGCGGAAAC	0	2	0	0
tsrna-09285	ACACGCGAAAGGTCCCCGGTTC	1	3	0	0
tsrna-09284	ACACGCGAAAGGTCCCCGGTT	0	5	0	0
tsrna-09283	ACACGCGAAAGGTCCCCGGT	0	1	0	0
tsrna-09279	ACACGCAGAAGGTCCTGGGTTCGAGCCCCAGTGGAACCACC/	35	35	50	23
tsrna-09278	ACACGCAGAAGGTCCTGGGTTCGAGCCCCAGTGGAACCACC	16	16	14	2
tsrna-09277	ACACGCAGAAGGTCCTGGGTTCGAGCCCCAGTGGAACCAC	9	8	14	0
tsrna-09276	ACACGCAGAAGGTCCTGGGTTCGAGCCCCAGTGGAACCA	7	8	9	1
tsrna-09275	ACACGCAGAAGGTCCTGGGTTCGAGCCCCAGTGGAACC	8	5	8	2
tsrna-09274	ACACGCAGAAGGTCCTGGGTTCGAGCCCCAGTGGAAC	1	3	0	0
tsrna-09273	ACACGCAGAAGGTCCTGGGTTCGAGCCCC	1	1	1	0
tsrna-09271	ACACCTCTTTACAGTGACCA	1	0	0	0
tsrna-09270	ACACCTCTTTACAGTGACC	0	0	0	0
tsrna-09267	ACACCAGTCTTGTAAACC	0	1	0	0
tsrna-09266	ACACAAAGCACCCAACTTACACTTAGGAG	0	0	0	0
tsrna-09265	ACACAAAGCACCCAACTTACACTTAG	0	0	0	0
tsrna-09258	ACAAGAACTGCTAACTCATGCCCCCATGTCTAACAACATGGCT	6	3	3	0
tsrna-09257	ACAAGAACTGCTAACTCATGCCCCCATGTCTAACAACATGGCT	1	1	6	0
tsrna-09256	ACAAGAACTGCTAACTCATGCCCCCATGTCTAACAACATGGCT	5	1	2	0
tsrna-09255	ACAAGAACTGCTAACTCATGCCCCCATGTCTAACAACATGGCT	1	0	4	1
tsrna-09254	ACAAGAACTGCTAACTCATGCCCCCATGTCTAACAACATGGC	0	2	2	1
tsrna-09253	ACAAGAACTGCTAACTCATGCCCCCATGTCTAACAACATGG	4	0	3	3
tsrna-09252	ACAAGAACTGCTAACTCATGCCCCCATGTCTAACAACATG	2	3	6	0
tsrna-09251	ACAAGAACTGCTAACTCATGCCCCCATGTCTAACAACA	3	1	1	1

tsrna-09250	ACAAGAACTGCTAACTCATGCCCCCATGTCTAACAA	1	3	2	0
tsrna-09249	ACAAGAACTGCTAACTCATGCCCCCATGTCTAACA	1	0	1	0
tsrna-09248	ACAAGAACTGCTAACTCATGCCCCCATGTCT	0	2	0	0
tsrna-09247	ACAAGAACTGCTAACTCATGCCCCCATGTC	2	0	3	0
tsrna-09246	ACAAGAACTGCTAACTCATGCCCCCATGT	0	0	1	0
tsrna-09245	ACAAGAACTGCTAACTCATGCCCCCATG	2	1	0	0
tsrna-09244	ACAAGAACTGCTAACTCATGCCCCCA	0	1	1	0
tsrna-09243	ACAAGAACTGCTAACTCATGCCCCC	1	0	0	0
tsrna-09242	ACAAGAACTGCTAACTCATGCCCC	0	0	0	0
tsrna-09241	ACAAGAACTGCTAACTCATGCCC	0	1	1	0
tsrna-09240	ACAAGAACTGCTAACTCATGCC	1	0	0	0
tsrna-09239	ACAAGAACTGCTAACTCATGC	1	0	0	0
tsrna-09237	ACAAGAACTGCTAACTCAT	1	0	0	0
tsrna-09236	ACAAGAACTGCTAACTCA	1	0	0	0
tsrna-09235	ACAAGAACTGCTAACTC	1	0	0	0
tsrna-09233	ACAACGATGGTTTTTCATATCATTGGTCGT	0	1	1	0
tsrna-09232	ACAACGATGGTTTTTCATATC	0	1	0	0
tsrna-09229	ACAACATGGCTTTCTCACCA	1	0	0	0
tsrna-09224	ACAAAGCACCCAACTTACACTTAGGAGATT	0	0	0	0
tsrna-09223	ACAAAGCACCCAACTTACACTTAGGAG	0	1	1	0
tsrna-09221	ACAAAGCACCCAACTTACAC	0	0	0	0
tsrna-09220	ACAAAACGAATGATTTCGACTCA	0	2	3	0
tsrna-09219	ACAAAACGAATGATTTCGACTC	0	1	1	0
tsrna-09218	AATTTTGGTGCAACTCCAAATAAAAGTACC	0	0	0	0
tsrna-09213	AATTTTGGATTCTCAGGGATGGGTTCGATT	1	1	3	0
tsrna-09212	AATTTTGGATTCTCAGGGATGGGTTCG	1	1	0	1
tsrna-09211	AATTTTGGATTCTCAGGGATG	0	0	0	0
tsrna-09209	AATTTGACTAGAGATCAAGA	24	73	15	0
tsrna-09208	AATTGCAAATTCGAAGAAGCAGCTTCAAACCTGCCGGGGCTT	1	2	0	0
tsrna-09207	AATTGCAAATTCGAAGAAGCAGCTTCAAACCTGCCGGGGCT	0	1	0	0
tsrna-09206	AATTGCAAATTCGAAGAAGCAGCT	1	1	0	0
tsrna-09204	AATTCTCGCTGGGGCCTCCA	40	37	12	16
tsrna-09203	AATTCTCGCCTGCCACGCGGGAGGCCCGG	4	6	0	1
tsrna-09202	AATTCTCGCCTGCCACGCGGGAGGCCCG	3	8	2	1
tsrna-09201	AATTCTCGCCTGCCACGCGGGAGGCCC	5	8	1	0
tsrna-09200	AATTCTCGCCTGCCACGCGGG	4	10	0	0
tsrna-09199	AATTCTCGCCTGCCACGCGG	1	5	0	2
tsrna-09198	AATTCTCGCCTGCCACGCG	0	10	1	1
tsrna-09197	AATTCTCGCCTGCCACGC	1	6	2	0
tsrna-09196	AATTCTCGCCTGCCACG	2	10	1	0
tsrna-09195	AATTCTCGCCTGCCAC	0	1	1	0
tsrna-09194	AATTCGAAGAAGCAGCTTCAAACCTGCCGGGGCTTCC	0	5	0	0
tsrna-09193	AATTCGAAGAAGCAGCTTCAAACCTGCCGGGGCTT	0	2	0	0
tsrna-09192	AATTCGAAGAAGCAGCTTCAAACCTGCCGGGGCT	2	0	1	0
tsrna-09191	AATTCGAAGAAGCAGCTTCAAACCTGCCGG	0	2	0	0
tsrna-09190	AATTCCTCTTCTTAACACCA	0	4	0	1
tsrna-09189	AATTCCGGCTCGAAGGACCA	69	159	30	13
tsrna-09187	AATTCCCGGCCAATGCACCA	4	7	0	1
tsrna-09186	AATTCCCCGACGGGGAGCCA	0	0	0	0
tsrna-09185	AATTCAAAGGTTCCGGGTTCGAGTCCCGGCGGAGTCGCCA	0	5	1	0
tsrna-09184	AATTCAAAGGTTCCGGGTTC	0	0	2	0
tsrna-09180	AATTATGATAATCATATTTACCAA	0	0	1	0
tsrna-09172	AATTAGAATCTTAGCTTTGGGTGCTAATGGTGGAGTTAAAGAC	4	6	6	2
tsrna-09171	AATTAGAATCTTAGCTTTGGGTGCTAATGGTGGAGT	1	1	0	1
tsrna-09170	AATTAGAATCTTAGCTTTGGGTGCTAATGG	1	0	0	0
tsrna-09169	AATTAGAATCTTAGCTTTGGGTGCT	2	2	1	0
tsrna-09168	AATTAGAATCTTAGCTTTGGGTGC	0	1	0	0
tsrna-09167	AATTAGAATCTTAGCTTTGGGTG	3	1	3	1
tsrna-09166	AATTAGAATCTTAGCTTTGGGT	1	1	1	0
tsrna-09165	AATTAGAATCTTAGCTTTGGG	0	0	0	0
tsrna-09164	AATTAGAATCTTAGCTTTGG	0	1	0	0
tsrna-09163	AATTAGAATCTTAGCTTTG	0	2	0	2
tsrna-09162	AATTAACTAGTTTTGACAACATTCAAAAAA	0	0	0	0
tsrna-09161	AATTAACTAGTTTTGACAACATTCAAAAA	0	0	0	0

tsrna-09158	AATTAACTAGTTTTGACAACATTCA	2	0	0	0
tsrna-09157	AATTAACTAGTTTTGACAACATTC	0	0	1	0
tsrna-09156	AATTAACTAGTTTTGACAACATT	0	0	0	0
tsrna-09155	AATTAACTAGTTTTGACAACAT	0	1	0	0
tsrna-09151	AATTAACTAGTTTTGACA	1	0	0	0
tsrna-09148	AATTAAAGTGGCTGATTTGCGTTCA	1	0	0	0
tsrna-09147	AATTAAAGTGGCTGATTTGCGTTC	0	0	0	0
tsrna-09146	AATTAAAGTGGCTGATTTGCGTT	0	0	0	0
tsrna-09145	AATTAAAGTGGCTGATTTGC	0	0	1	0
tsrna-09144	AATTAAAGTGGCTGATTTG	0	0	1	0
tsrna-09143	AATGTTTAGACGGGCTCACATCACCCCATAAACACCA	100	12	67	20
tsrna-09142	AATGTTTAGACGGGCTCACATCACCCCATAAACACC	38	19	34	11
tsrna-09141	AATGTTTAGACGGGCTCACATCACCCCATAAACA	37	11	33	10
tsrna-09140	AATGTTTAGACGGGCTCACATCACCCCATAAA	31	13	27	7
tsrna-09139	AATGTTTAGACGGGCTCACATCACCCCATAA	24	7	26	6
tsrna-09138	AATGTTTAGACGGGCTCACATCACCCCATA	30	11	25	4
tsrna-09137	AATGTTTAGACGGGCTCACATCACCCCA	30	14	21	8
tsrna-09136	AATGTTTAGACGGGCTCACATCACCCC	19	5	17	3
tsrna-09135	AATGTTTAGACGGGCTCACATCACCC	32	5	18	5
tsrna-09134	AATGTTTAGACGGGCTCACATCACC	18	10	13	3
tsrna-09133	AATGTTTAGACGGGCTCACATCA	13	8	10	5
tsrna-09132	AATGTTTAGACGGGCTCACATC	15	10	12	2
tsrna-09131	AATGTTTAGACGGGCTCACAT	16	9	20	2
tsrna-09130	AATGTTTAGACGGGCTCACA	12	11	11	2
tsrna-09129	AATGTTTAGACGGGCTCAC	8	4	8	0
tsrna-09128	AATGTTTAGACGGGCTCA	7	2	0	1
tsrna-09127	AATGTTTAGACGGGCTC	2	2	1	0
tsrna-09126	AATGTTTAGACGGGCT	0	0	1	0
tsrna-09125	AATGTTGGTTATACCCTTCCCGTACTACCA	7	13	0	5
tsrna-09124	AATGTTGGTTATACCCTTCCCGTACTACC	0	1	1	0
tsrna-09119	AATGTTGGTTATACCC	0	1	0	0
tsrna-09118	AATGGTTAGCACTCTGGGCT	1	0	0	0
tsrna-09116	AATGGTTAGCACTCTGGACTTTGAATCCAG	11	3	4	4
tsrna-09115	AATGGTTAGCACTCTGGACTTTGAATCCA	4	11	4	5
tsrna-09114	AATGGTTAGCACTCTGGACTTTGAATCC	7	6	3	3
tsrna-09113	AATGGTTAGCACTCTGGACTTTGAATC	2	6	1	1
tsrna-09112	AATGGTTAGCACTCTGGACTTTGAAT	1	0	0	0
tsrna-09111	AATGGTTAGCACTCTGGACTTTGAA	1	0	0	1
tsrna-09110	AATGGTTAGCACTCTGGACTTTGA	0	1	2	0
tsrna-09106	AATGGTTAGCACTCTGGACTCTGAATCCAGCGATCCGAGTTC/	4	5	5	2
tsrna-09105	AATGGTTAGCACTCTGGACTCTGAATCCAGCGATCCGAGT	3	7	3	2
tsrna-09104	AATGGTTAGCACTCTGGACTCTGAATCCAGCGATCCGAG	3	5	0	2
tsrna-09103	AATGGTTAGCACTCTGGACTCTGAATCCAGCGATCC	2	5	3	2
tsrna-09102	AATGGTTAGCACTCTGGACTCTGAATCCAGCG	5	4	2	1
tsrna-09101	AATGGTTAGCACTCTGGACTCTGAATCCAGC	3	1	4	0
tsrna-09100	AATGGTTAGCACTCTGGACTCTGAATCCAG	4	8	5	1
tsrna-09099	AATGGTTAGCACTCTGGACTCTGAATCCA	3	4	1	1
tsrna-09098	AATGGTTAGCACTCTGGACTCTGAATCC	3	5	4	0
tsrna-09097	AATGGTTAGCACTCTGGACTCTGAATC	0	2	2	0
tsrna-09096	AATGGTTAGCACTCTGGACTCTGAAT	0	0	0	1
tsrna-09095	AATGGTTAGCACTCTGGACTCTGAA	2	0	0	0
tsrna-09094	AATGGTTAGCACTCTGGACTCTGA	1	0	2	0
tsrna-09093	AATGGTTAGCACTCTGGACTCTG	1	1	0	2
tsrna-09091	AATGGTTAGCACTCTGGACTC	1	0	0	0
tsrna-09090	AATGGTTAGCACTCTGGACT	0	1	0	0
tsrna-09088	AATGGTTAGCACTCTGGA	0	0	1	0
tsrna-09085	AATGGTGGTTCAGTGGTAGAATTCTCGCCT	28	21	17	3
tsrna-09084	AATGGTGGTTCAGTGGTAGAATTCTCGC	3	8	0	2
tsrna-09083	AATGGTGGTTCAGTGGTAGAATTCTCG	3	4	3	2
tsrna-09082	AATGGTGGTTCAGTGGTAGAATTCTC	1	4	1	0
tsrna-09081	AATGGTGGTTCAGTGGTAGAATTCT	3	0	2	1
tsrna-09080	AATGGTGGAGTTAAAGACTTTTTCTCTGACCA	27	52	18	13
tsrna-09079	AATGGTGGAGTTAAAGACTTTTTCTCTGACC	8	24	6	2
tsrna-09078	AATGGTGGAGTTAAAGACTTTTTCTCTGAC	2	10	3	0

tsrna-09077	AATGGTGGAGTTAAAGACTTTTTCTCTGA	4	9	1	1
tsrna-09076	AATGGTGGAGTTAAAGACTTTTTCTCTG	5	10	2	1
tsrna-09075	AATGGTGGAGTTAAAGACTTTTTCTCT	2	14	5	0
tsrna-09074	AATGGTGGAGTTAAAGACTTTTTCTC	3	10	0	0
tsrna-09073	AATGGTGGAGTTAAAGACTTTTTCT	6	14	3	0
tsrna-09072	AATGGTGGAGTTAAAGACTTTTTC	9	7	3	0
tsrna-09071	AATGGTGGAGTTAAAGACTTTTT	2	8	3	0
tsrna-09070	AATGGTGGAGTTAAAGACTTTT	2	3	2	0
tsrna-09069	AATGGTGGAGTTAAAGACTTT	0	2	0	0
tsrna-09068	AATGGTGGAGTTAAAGACTT	3	3	1	0
tsrna-09067	AATGGTGGAGTTAAAGACT	2	2	2	1
tsrna-09066	AATGGTGGAGTTAAAGAC	1	1	1	0
tsrna-09065	AATGGTGGAGTTAAAGA	1	2	0	0
tsrna-09064	AATGGTGGAGTTAAAG	0	2	0	0
tsrna-09063	AATGGTGAGCACTTTGGACTCTGA	0	0	0	0
tsrna-09062	AATGGTGAGCACTTTGGACTCTG	0	0	0	0
tsrna-09061	AATGGTGAGCACTTTGGACTCT	0	1	0	0
tsrna-09058	AATGGTGAGCACTCTGGACTCTGAATCCAG	4	6	0	0
tsrna-09057	AATGGTGAGCACTCTGGACTCTGAATCCA	0	4	0	0
tsrna-09056	AATGGTGAGCACTCTGGACTCTGAATCC	1	4	0	3
tsrna-09055	AATGGTGAGCACTCTGGACTCTGAATC	0	1	0	0
tsrna-09054	AATGGTGAGCACTCTGGACTCTGA	0	2	0	0
tsrna-09048	AATGGTGAGCACTCTGGA	0	0	0	0
tsrna-09038	AATGGTAGAATTCTCG	0	0	1	0
tsrna-09037	AATGGTAAGCACTCTGGACTCTGAATCC	2	4	0	0
tsrna-09034	AATGGTAAGCACTCTGGACTCTG	0	0	0	0
tsrna-09026	AATGGCTGAGTGAAGCATTGGACTGTAAA	4	2	0	0
tsrna-09025	AATGGCTGAGTGAAGCATTGGACTGTAA	0	2	1	0
tsrna-09024	AATGGCTGAGTGAAGCATTGGACTGTA	1	1	1	0
tsrna-09023	AATGGCTGAGTGAAGCATTGGACTGT	1	1	0	0
tsrna-09022	AATGGCTGAGTGAAGCATTGGACTG	0	0	0	0
tsrna-09021	AATGGCTGAGTGAAGCATTGGACT	1	1	0	0
tsrna-09020	AATGGCTGAGTGAAGCATTGGAC	0	3	1	0
tsrna-09019	AATGGCTGAGTGAAGCATTGGA	0	1	1	1
tsrna-09018	AATGGCTGAGTGAAGCATTGG	2	0	2	0
tsrna-09017	AATGGCTGAGTGAAGCATTG	0	0	1	0
tsrna-09016	AATGGCTGAGTGAAGCATT	0	1	0	0
tsrna-09015	AATGGCTGAGTGAAGCA	0	0	0	0
tsrna-09014	AATGGCTGAGTGAAGC	0	0	0	0
tsrna-09012	AATGGATAGCGCATTGGACTTC	7	20	1	0
tsrna-09011	AATGGATAGCGCATTGGACT	5	14	1	3
tsrna-09010	AATGGATAGCGCATTGGAC	5	16	1	1
tsrna-09009	AATGGATAGCGCATTGGA	5	6	0	3
tsrna-09008	AATGGATAGCGCATTGG	5	4	1	1
tsrna-09007	AATGGATAAGGCGTCTGATTCC	2	6	1	0
tsrna-09006	AATGGATAAGGCGTCTGACTTCGGATC	1	1	1	0
tsrna-09005	AATGGATAAGGCGTCTGACTTCGGAT	0	1	0	0
tsrna-09004	AATGGATAAGGCGTCTGACTTCG	1	0	1	1
tsrna-09003	AATGGATAAGGCGTCTGACTTC	1	1	0	0
tsrna-09002	AATGGATAAGGCGTCTGACTT	0	1	0	1
tsrna-09001	AATGGATAAGGCGTCTGACT	0	2	0	0
tsrna-09000	AATGGATAAGGCGTCTGA	0	1	0	0
tsrna-08999	AATGGATAAGGCATTGGC	1	1	1	0
tsrna-08998	AATGGATAAGGCATTGG	1	1	1	0
tsrna-08997	AATGGATAAGGCATTG	1	2	0	0
tsrna-08996	AATGGATAAGGCATCAGCCTCC	1	0	1	1
tsrna-08995	AATGGATAAGGCATCAGCCTC	2	2	2	1
tsrna-08994	AATGGATAAGGCATCAGCCT	6	1	5	1
tsrna-08993	AATGGATAAGGCACTGGC	19	21	10	4
tsrna-08992	AATGGATAAGGCACTGG	11	18	5	4
tsrna-08991	AATGGATAAGGCACTG	7	12	10	3
tsrna-08990	AATGGATAACGCGTCTGACTACGGA	2	0	3	1
tsrna-08989	AATGGATAACGCGTCTGACTACGG	1	0	1	0
tsrna-08988	AATGGATAACGCGTCTGACTACG	1	2	1	3

tsrna-08986	AATGGAGGCGTGGGTTCGAATCCCACTTCTGACACCA	24	56	9	12
tsrna-08985	AATGGAGGCGTGGGTTCGAATCCCACTTCTGACACC	2	8	6	0
tsrna-08984	AATGGAGGCGTGGGTTCGAATCCCACTTCTGACA	0	3	0	0
tsrna-08983	AATGGAGGCGTGGGTT	1	4	0	0
tsrna-08982	AATGCCGAGGTTGTGAGTTCGATC	1	10	0	0
tsrna-08981	AATGCCGAGGTTGTGAGTTCGAT	0	5	0	0
tsrna-08980	AATGCCGAGGTTGTGAGTTCGAGC	7	74	3	0
tsrna-08979	AATGCCGAGGTTGTGAGTTCGAG	1	13	1	0
tsrna-08978	AATGCCGAGGTTGTGAGTTCGA	2	6	0	0
tsrna-08977	AATGCCGAGGTTGTGAGTTCG	2	2	1	0
tsrna-08976	AATGCCGAGGTTGTGAGTTCAAGC	0	3	0	0
tsrna-08975	AATGCCGAGGTTGTGAGTTC	0	3	0	0
tsrna-08974	AATGCCGAGGTTGTGAGTT	2	2	0	0
tsrna-08970	AATGATTTCGACTCATTA	0	1	0	0
tsrna-08969	AATGATTTCGACTCATT	0	1	0	0
tsrna-08967	AATCTTAGCTTTGGGTGCTAATGGTGGAGTTAAAGA	3	1	1	0
tsrna-08966	AATCTTAGCTTTGGGTGCTAATGGTGGAGT	1	0	0	0
tsrna-08962	AATCTGAGGGTCCAGGGTTCAAGT	2	2	0	0
tsrna-08961	AATCTGAGGGTCCAGGGTTCAAG	1	4	0	0
tsrna-08960	AATCTGACAACAGAGGCTTACGACCCCTTATTTACCCCA	4	3	4	1
tsrna-08959	AATCTGACAACAGAGGCTTACGACCCCTTATTTACCCC	2	0	3	0
tsrna-08958	AATCTGACAACAGAGGCTTACGACCCCTTATTTACCC	2	1	1	0
tsrna-08957	AATCTGACAACAGAGGCTTACGACCCCTTATTTACC	3	0	0	0
tsrna-08956	AATCTGACAACAGAGGCTTACGACCCCTT	0	0	0	0
tsrna-08955	AATCTGACAACAGAGGCTTACGACCCCT	0	0	0	0
tsrna-08949	AATCTGAAGGTCGTGAGTTCGATCCTCACACGGGGCACCA	7	39	2	0
tsrna-08948	AATCTGAAGGTCGTGAGTTCGATCCTCACACGGGGCACC	4	12	7	0
tsrna-08947	AATCTGAAGGTCGTGAGTTCGATCCTCACACGGGGCAC	4	8	3	0
tsrna-08946	AATCTGAAGGTCGTGAGTTCGAGCCTCACACGGGGCACCA	11	18	1	1
tsrna-08945	AATCTGAAGGTCGTGAGTTCGAG	3	8	2	1
tsrna-08944	AATCTGAAGGTCGTGAGT	0	6	2	0
tsrna-08943	AATCTGAAGGTCCTGAGTTCGAACCTCAGAGGGGGCACCA	18	42	12	5
tsrna-08942	AATCTGAAGGTCCTGAGTTCGA	12	42	11	2
tsrna-08941	AATCTGAAGGTCCTGAGT	9	27	17	3
tsrna-08940	AATCTGAAGGTCCTGA	0	3	0	0
tsrna-08939	AATCTCGGTGGGACCTCCA	52	93	25	9
tsrna-08938	AATCTCGGTGGGACCTCC	1	5	1	0
tsrna-08937	AATCTCGGTGGAACCTCCA	101	182	49	18
tsrna-08936	AATCTCGGTGGAACCTCC	3	13	2	1
tsrna-08935	AATCTCGGTGGAACCTC	0	2	0	0
tsrna-08934	AATCTCGGTGGAACCT	1	2	1	0
tsrna-08933	AATCTCGCTGGGGCCTCCA	53	59	22	17
tsrna-08932	AATCTCAGGGTCGTGGGTTCGAG	0	2	0	0
tsrna-08931	AATCTCAGGGTCGTGGGTTCG	0	0	0	0
tsrna-08929	AATCTAAAGACAGGGGTTAGGCCTCTTTTTACCACC	34	20	16	1
tsrna-08928	AATCTAAAGACAGGGGTTAGGCCTCTTTTTACC	24	19	11	0
tsrna-08927	AATCTAAAGACAGGGGTTAGGCCTCTTTTT	10	20	9	1
tsrna-08926	AATCTAAAGACAGGGGTTAGGCCTCT	5	2	0	0
tsrna-08925	AATCTAAAGACAGGGGTTAGG	0	0	0	0
tsrna-08924	AATCTAAAGACAGGGGTTAG	0	1	0	0
tsrna-08923	AATCGGTTAGCGCGTTCGGCT	0	1	0	0
tsrna-08922	AATCCTGTTCGTGACGCCA	2	7	1	1
tsrna-08921	AATCCTGTTCGTGACGCC	1	0	0	0
tsrna-08919	AATCCTGTTCGTGACG	0	0	0	0
tsrna-08918	AATCCTGCTCACAGCGCCA	4	13	2	2
tsrna-08917	AATCCTGCTCACAGCGCC	1	1	2	0
tsrna-08916	AATCCTGCCGACTACGCCA	95	248	36	15
tsrna-08915	AATCCTGCCGACTACGCC	5	11	3	2
tsrna-08914	AATCCTGCCGACTACGC	0	2	0	0
tsrna-08910	AATCCGGGTGCCCCCTCCA	140	203	54	27
tsrna-08909	AATCCGGGTGCCCCCTCC	3	5	1	0
tsrna-08908	AATCCGGGTGCCCCCTC	0	1	0	0
tsrna-08907	AATCCGGCTCGGAGGACCA	1	4	0	0
tsrna-08904	AATCCGGCTCGAAGGACCA	16	23	12	2

tsrna-08903	AATCCGGCTCGAAGGACC	1	2	0	0
tsrna-08898	AATCCCGGGTTTCGGCACCA	51	71	17	14
tsrna-08896	AATCCCGGACGAGCCCCCA	123	157	38	33
tsrna-08895	AATCCCGGACGAGCCCCC	5	10	1	3
tsrna-08894	AATCCCGGACGAGCCCC	1	1	0	1
tsrna-08893	AATCCCGGACGAGCCC	0	0	0	0
tsrna-08892	AATCCCCGGCATCTCCACCA	113	124	59	18
tsrna-08891	AATCCCCGGCATCTCCACC	6	5	1	2
tsrna-08888	AATCCCCGGCACCTCCACCA	168	249	86	25
tsrna-08887	AATCCCCGGCACCTCCACC	7	17	4	0
tsrna-08884	AATCCCCAGCACCTCCACCA	0	0	0	0
tsrna-08882	AATCCCATCCTCGTCGCCA	1	6	1	0
tsrna-08878	AATCCCAGTAGAGCCTCCA	2	4	2	6
tsrna-08876	AATCCCAGCGGTGCCTCCA	0	0	0	0
tsrna-08873	AATCCCAGCGGGGCCTCCA	0	1	0	0
tsrna-08872	AATCCCACTTCTGACACCA	3	6	2	4
tsrna-08871	AATCCCACTTCTGACACC	0	1	0	0
tsrna-08868	AATCCCACTCCTGACACCA	75	58	39	19
tsrna-08867	AATCCCACTCCTGACACC	2	3	1	0
tsrna-08866	AATCCCACTCCTGACAC	0	0	1	0
tsrna-08864	AATCCCACCTTCGTCGCCA	0	6	2	2
tsrna-08861	AATCCCACCGCTGCCACCA	0	1	0	0
tsrna-08859	AATCCCACCGCTGCCAC	0	0	0	0
tsrna-08858	AATCCCACCCTCGTCGCCA	1	17	1	2
tsrna-08857	AATCCCACCCTCGTCGCC	0	1	0	1
tsrna-08854	AATCCCACCAGAGTCGCCA	2	6	1	0
tsrna-08853	AATCCCACCAGAGTCGCC	0	1	0	0
tsrna-08849	AATCCATTGGGGTTTCCCCGCGCAGGTTCGAATCCTGCCGAC	2	5	0	0
tsrna-08848	AATCCATTGGGGTTTCCCCGCGCAGGTTCGAATCCTGCCGAC	1	4	0	0
tsrna-08847	AATCCATTGGGGTTTCCCCGCGCAGGTTCGAATCCTGCCGA	3	5	0	0
tsrna-08842	AATCCAGGTGCCCCCTCCA	2	1	0	1
tsrna-08840	AATCCAGCGATCCGAGTTCGAG	0	1	0	0
tsrna-08837	AATCCAGCGATCCGAGTTCAAGTC	0	1	0	0
tsrna-08836	AATCCAGCGATCCGAGTTCAAGT	0	0	1	0
tsrna-08835	AATCCAGCGATCCGAGTTCAAG	0	1	0	0
tsrna-08834	AATCCAGCGATCCGAGTTCAAATCTCGGTGGAACCTC	0	0	1	0
tsrna-08832	AATCCAGCGATCCGAGTTCAAATCT	0	0	1	0
tsrna-08822	AATCCAGCAATCCGAGTTCGAAT	0	3	0	0
tsrna-08821	AATCCAGCAATCCGAGTTCGAA	0	2	0	0
tsrna-08820	AATCCAGCAATCCGAGTTCGA	0	2	0	0
tsrna-08819	AATCCAGCAATCCGAGTTCG	1	2	0	0
tsrna-08818	AATCATATTTACCAACCA	0	0	1	0
tsrna-08815	AATCACGTCGGGGTCACCA	4	8	2	2
tsrna-08814	AATCACGTCGGGGTCACC	0	0	1	0
tsrna-08810	AATATAGTTTAACCAAAACATCAGATTGTG	0	0	0	0
tsrna-08809	AATAGTACCGTTAACTTCCAATTAACTAGTTTTGAC	6	3	4	0
tsrna-08808	AATAGTACCGTTAACTTCCAATTAACTAGT	3	2	3	0
tsrna-08807	AATAGTACCGTTAACTTCCAATTA	1	2	6	0
tsrna-08805	AATAGTACCGTTAACTTCC	0	1	0	0
tsrna-08803	AATAGTACCGTTAACTT	0	0	0	0
tsrna-08801	AATAGGAGCTTAAACCCCCTTATTTCTACC	1	6	1	0
tsrna-08800	AATAGGAGCTTAAACCCCC	0	0	1	0
tsrna-08798	AATAGGAGCTTAAACCC	0	0	0	0
tsrna-08792	AATACTTAATTTCTGCCA	2	0	0	0
tsrna-08790	AATACAGACCAAGAGCCTTCA	0	0	0	0
tsrna-08789	AATACAGACCAAGAGCCTTC	0	0	0	0
tsrna-08788	AATACAGACCAAGAGCC	0	0	0	0
tsrna-08786	AATACACTGAAAATGTTTAGACGGGCTCACATCACCCCATAAA	128	52	79	16
tsrna-08785	AATACACTGAAAATGTTTAGACGGGCTCAC	24	20	14	1
tsrna-08784	AATACACTGAAAATGTTTAGACGGGCTC	6	13	4	0
tsrna-08783	AATACACCAGTCTTGTAAACC	9	3	4	1
tsrna-08782	AATACAACGATGGTTTTTCATATCATTGGTCGTGGTTGTAGTC	5	4	8	1
tsrna-08781	AATACAACGATGGTTTTTCATATCATTGGTCGTGGTTGTA	2	2	2	0
tsrna-08780	AATACAACGATGGTTTTTCATATCATTGGT	0	0	0	0

tsrna-08778	AATACAACGATGGTTTTTC	1	0	0	0
tsrna-08777	AATAATAGGAGCTTAAACCCCCTTATTTCT	2	1	0	0
tsrna-08776	AATAATAGGAGCTTAAACCCCCTT	1	0	0	0
tsrna-08773	AATAATAGGAGCTTAAACCCC	0	0	0	0
tsrna-08772	AATAATAGGAGCTTAAACCC	0	0	0	1
tsrna-08771	AATAATAGGAGCTTAAACC	0	0	0	0
tsrna-08770	AATAAGCTATCGGGCCCATACCCCGAAA	0	1	1	0
tsrna-08769	AATAAGCTATCGGGCCCATACC	0	1	1	1
tsrna-08768	AATAAGCTATCGGGCCCATA	0	0	0	0
tsrna-08767	AATAAGCTATCGGGCCCAT	0	0	0	0
tsrna-08766	AATAAGCTATCGGGCCCA	0	2	0	0
tsrna-08765	AATAAGCTATCGGGCCC	0	0	0	0
tsrna-08763	AATAACGCCAAGGTCGCGGGTTCGA	9	9	3	0
tsrna-08762	AATAACGCCAAGGTCGCGGGTT	3	6	0	0
tsrna-08761	AATAACGCCAAGGTCGCGGGT	4	6	0	0
tsrna-08760	AATAACGCCAAGGTCGCGG	0	1	0	0
tsrna-08759	AAGTTGCAATACTTAATTTCTGCCA	2	0	0	1
tsrna-08758	AAGTTGCAATACTTAATTTCTGCC	0	0	0	0
tsrna-08757	AAGTTGCAATACTTAATTTCTGC	1	0	0	0
tsrna-08756	AAGTTCTGGTCTCCGGATGGAGGCGTGGGT	5	37	5	1
tsrna-08754	AAGTTCTGGTCTCCGGATGGAGG	0	1	0	0
tsrna-08753	AAGTTCTGGTCTCCGGATGGAG	0	0	0	1
tsrna-08745	AAGTTAAAGATTAAGAGAACCAACACCTCTTTACAGTGACCA	8	4	9	1
tsrna-08744	AAGTTAAAGATTAAGAGAACCAACACCTCTTTACAGTGACC	5	1	6	1
tsrna-08743	AAGTTAAAGATTAAGAGAACCAACACCTCTTTACAGTGAC	3	1	8	3
tsrna-08742	AAGTTAAAGATTAAGAGAACCAACACC	0	0	0	0
tsrna-08741	AAGTTAAAGATTAAGAGAACC	0	0	0	0
tsrna-08739	AAGTGTTTGTGGGTTTAAGTCCCATTGGTCTAGCCA	2	3	1	0
tsrna-08738	AAGTGTTTGTGGGTTTAAGTCCCATTGGTCTAGCC	1	0	0	0
tsrna-08737	AAGTGTTTGTGGGTTTAAGTCCCATTGGTCTAGC	0	0	2	0
tsrna-08736	AAGTGTTTGTGGGTTTAAGTCCCATTGGTCTA	0	2	0	0
tsrna-08735	AAGTGTTTGTGGGTTTAAGTCCCATTGGTC	0	1	0	0
tsrna-08734	AAGTGTTTGTGGGTTTAAGTCCCATTG	0	0	0	0
tsrna-08733	AAGTGTTTGTGGGTTTAAGTCCCATT	0	3	0	0
tsrna-08732	AAGTGTTTGTGGGTTTAAGTCCCA	0	0	0	0
tsrna-08731	AAGTGTTTGTGGGTTTAAGTCCC	0	0	1	0
tsrna-08730	AAGTGTTTGTGGGTTTAAGTCC	0	1	0	0
tsrna-08729	AAGTGTTTGTGGGTTTAAGTC	0	0	0	0
tsrna-08724	AAGTGTTTGTGGGTTT	0	0	1	0
tsrna-08721	AAGTGGCTGATTTGCGTTCAGTTGATGC	0	1	0	0
tsrna-08720	AAGTGGCTGATTTGCGTTCAGTTGATG	0	2	2	0
tsrna-08719	AAGTGGCTGATTTGCGTTCAGT	0	1	0	0
tsrna-08718	AAGTGGCTGATTTGCGTTCAG	0	0	0	0
tsrna-08717	AAGTGGCTGATTTGCGTTCA	0	0	0	0
tsrna-08715	AAGTGGCTGATTTGCGTT	0	0	0	0
tsrna-08712	AAGTCTCGGTGGAACCTCCA	50	107	15	11
tsrna-08711	AAGTCCCTGTTCGGGCGCCA	6	9	3	0
tsrna-08709	AAGTCCCTGTTCAGGCGCCA	0	0	0	0
tsrna-08706	AAGTCATGGAGGCCATGGGGTTGG	0	3	1	0
tsrna-08705	AAGTCATGGAGGCCATGGGGTTG	0	3	0	0
tsrna-08704	AAGTCATGGAGGCCATGGGGTT	0	1	0	0
tsrna-08703	AAGTCATGGAGGCCATGGGGT	0	1	0	0
tsrna-08702	AAGTCATGGAGGCCATGGGG	0	0	1	0
tsrna-08698	AAGTCACGTCGGGGTCACCA	2	3	3	0
tsrna-08697	AAGTCACGTCGGGGTCACC	0	0	0	1
tsrna-08696	AAGTCACGTCGGGGTCAC	0	0	0	0
tsrna-08695	AAGTCACGTCGGGGTCA	0	1	0	0
tsrna-08694	AAGTCACGTCGGGGTC	0	1	0	0
tsrna-08693	AAGGTTGTGGGTTCGAGTCCCACC	0	0	1	0
tsrna-08692	AAGGTTGGTGGTTCGAGCCCACCCAGGGAC	1	1	0	0
tsrna-08688	AAGGTTGCGTGTTCAAATCACGTCGGGGTC	0	2	0	1
tsrna-08687	AAGGTTGCGTGTTCAAATCA	0	1	0	0
tsrna-08684	AAGGTTCCGGGTTCGAGTCCCGGCG	0	0	0	0
tsrna-08681	AAGGTTCCGGGTTCGAGTCCCG	0	0	1	0

tsrna-08676	AAGGTCGTGAGTTCGAGCCTCACACGGGGC	0	0	0	0
tsrna-08673	AAGGTCGCGGGTTCGATCCCCGTACGGGCC	1	0	1	0
tsrna-08671	AAGGTCGCGGGTTCGATCCCCG	0	2	0	0
tsrna-08670	AAGGTCGCGGGTTCGATCCCC	0	1	1	0
tsrna-08668	AAGGTCGCGGGTTCGATCC	0	1	0	0
tsrna-08667	AAGGTCGCGGGTTCGATC	1	0	1	0
tsrna-08666	AAGGTCGCGGGTTCGA	0	0	0	0
tsrna-08665	AAGGTCCTGGGTTCGAGCCCCAGTGGAACCACCA	47	26	18	13
tsrna-08664	AAGGTCCTGGGTTCGAGCCCCAGTGGAACC	1	1	1	0
tsrna-08663	AAGGTCCTGGGTTCGAGCCCCAGTGGAAC	0	1	0	0
tsrna-08661	AAGGTCCTGGGTTCGAGCCCCAGTGGA	1	0	0	0
tsrna-08658	AAGGTCCTGGGTTCGAGCCCCAGT	0	0	0	0
tsrna-08657	AAGGTCCTGGGTTCGAGCCCCAG	0	0	0	0
tsrna-08656	AAGGTCCTGGGTTCGAGCCCCA	0	2	0	0
tsrna-08654	AAGGTCCTGGGTTCGAGCCC	1	0	1	0
tsrna-08653	AAGGTCCTGGGTTCGAGCC	1	0	0	0
tsrna-08652	AAGGTCCTGGGTTCGAGC	1	0	0	0
tsrna-08651	AAGGTCCTGGGTTCGAG	1	1	0	0
tsrna-08650	AAGGTCCTGAGTTCGAACCTC	0	0	0	1
tsrna-08647	AAGGTCCTGAGTTCGAA	1	0	0	0
tsrna-08646	AAGGTCCCTGGTTCGATCCCGGGTTTCGGC	4	9	4	1
tsrna-08645	AAGGTCCCTGGTTCGATCCCGGGTTTCGG	6	11	4	1
tsrna-08644	AAGGTCCCTGGTTCGATCCCGGG	5	8	5	3
tsrna-08643	AAGGTCCCTGGTTCGATCCCGG	5	8	3	1
tsrna-08642	AAGGTCCCTGGTTCGATCCC	6	14	1	1
tsrna-08641	AAGGTCCCTGGTTCGAT	4	12	1	2
tsrna-08638	AAGGTCCCCGGTTCGAAACC	1	2	0	0
tsrna-08637	AAGGTCCCCGGTTCGAAAC	2	0	0	0
tsrna-08634	AAGGTCAGCTAAATAAGCTATCGGGCCCA	0	1	1	0
tsrna-08633	AAGGTCAGCTAAATAAGCTATCGGGCCC	0	0	0	0
tsrna-08626	AAGGTCAGCTAAATAAGC	0	0	1	0
tsrna-08623	AAGGTATTAGAAAAACCATTTCATAACTTTGTCAAAGTTA	15	15	18	1
tsrna-08622	AAGGTATTAGAAAAACCATTTCATAACTTTGTCAAA	17	10	9	2
tsrna-08621	AAGGTATTAGAAAAACCATTTCATAACTTT	1	1	2	1
tsrna-08620	AAGGTATTAGAAAAACCATTTCATA	0	0	1	1
tsrna-08619	AAGGTATTAGAAAAACCATTTCA	0	0	1	0
tsrna-08616	AAGGTATTAGAAAAACC	0	1	0	0
tsrna-08614	AAGGGCTTAGCTTAATTAAAGTGGCTGATT	9	22	5	31
tsrna-08613	AAGGGCTTAGCTTAATTAAAGTGGCTGA	5	12	5	20
tsrna-08612	AAGGGCTTAGCTTAATTAAAGTGGCTG	3	14	5	17
tsrna-08611	AAGGGCTTAGCTTAATTAAAGTGGCT	3	8	2	7
tsrna-08610	AAGGGCTTAGCTTAATTAAAGTGGC	5	8	1	8
tsrna-08609	AAGGGCTTAGCTTAATTAAAGTGG	5	6	4	7
tsrna-08608	AAGGGCTTAGCTTAATTAAAGTG	4	5	1	12
tsrna-08607	AAGGGCTTAGCTTAATTAAAGT	2	3	1	2
tsrna-08606	AAGGGCTTAGCTTAATTAAAG	3	6	1	3
tsrna-08605	AAGGGCTTAGCTTAATTA	1	3	0	5
tsrna-08604	AAGGGCTTAGCTTAATT	2	1	0	0
tsrna-08587	AAGGATAACAGCTATCCATTGGTCTTAG	0	0	0	0
tsrna-08585	AAGGATAACAGCTATCCATTGGTCT	0	1	0	0
tsrna-08581	AAGCTCCGAGGTGATTTTCATA	1	0	0	0
tsrna-08580	AAGCTCACAAGAACTGCTAACTCATGCCCCCATGTCTAACAA(	0	1	3	2
tsrna-08579	AAGCTCACAAGAACTGCTAACTCATGCCCCCATGTC	2	2	0	1
tsrna-08578	AAGCTCACAAGAACTGCTAACTCATGCCCC	4	1	1	0
tsrna-08577	AAGCTCACAAGAACTGCTAACTCATGCC	2	0	0	0
tsrna-08576	AAGCTCACAAGAACTGCTAACTCATGC	0	1	0	1
tsrna-08575	AAGCTCACAAGAACTGCTAACTCATG	0	1	0	2
tsrna-08574	AAGCTCACAAGAACTGCTAACTCA	1	0	0	0
tsrna-08573	AAGCTCACAAGAACTGCTAACTC	1	1	0	0
tsrna-08572	AAGCTCACAAGAACTGCTAACT	0	0	0	1
tsrna-08571	AAGCTCACAAGAACTGCTAAC	1	0	0	1
tsrna-08570	AAGCTCACAAGAACTGCT	1	0	0	0
tsrna-08569	AAGCTATCGGGCCCATACCCCGAAAATGTT	1	3	1	0
tsrna-08568	AAGCTATCGGGCCCATACCCCGAAA	0	0	1	0

tsrna-08567	AAGCTATCGGGCCCATACCCCGAA	0	2	0	0
tsrna-08566	AAGCTATCGGGCCCATACCCC	0	1	1	0
tsrna-08565	AAGCTATCGGGCCCATACC	0	0	0	2
tsrna-08563	AAGCGTGCTGGGCCCATAAC	0	0	0	0
tsrna-08562	AAGCGTGCTGGGCCCA	0	0	0	0
tsrna-08560	AAGCCAGGGATTGTGGGTTCGAGTCCCATCTGGGGTGCCA	8	23	5	2
tsrna-08559	AAGCCAGGGATTGTGGGTTCGAGTCCCATCTGGGGTGCC	5	14	3	1
tsrna-08558	AAGCCAGGGATTGTGGGTTCGAGTCCCATCTGGGGTGC	5	20	2	4
tsrna-08557	AAGCCAGGGATTGTGGGTTCGAGTCCCATCTGGGGTG	4	11	6	0
tsrna-08556	AAGCCAGGGATTGTGGGTTCGAGTCCCACCTGGGGTGCCA	8	19	1	0
tsrna-08555	AAGCCAGGGATTGTGGGTTCGAGTCCCACCTGGGGTACC	4	18	4	3
tsrna-08554	AAGCCAGGGATTGTGGGTTCGAGTCCCACCCGGGGTACC	5	14	9	3
tsrna-08553	AAGCCAGGGATTGTGGGTTCGAGTCCCACCCGGGGTA	6	23	3	1
tsrna-08552	AAGCCAGGGATTGTGGGTTCGAGTCCC	6	13	6	1
tsrna-08551	AAGCCAGGGATTGTGGGTTCGAGTCC	2	11	6	3
tsrna-08550	AAGCCAGGGATTGTGGGTTCGAGTC	2	12	4	0
tsrna-08549	AAGCCAGGGATTGTGGGTTCG	6	13	3	1
tsrna-08548	AAGCCAGGGATTGTGGGTTC	2	12	3	0
tsrna-08547	AAGCCAGGGATTGTGGGTT	7	15	2	2
tsrna-08546	AAGCCAGGGATTGTGGGT	4	9	7	2
tsrna-08543	AAGCATTGGACTGTAAATCTAAAGACAGGGGTTAGG	0	0	2	1
tsrna-08542	AAGCATTGGACTGTAAATCTAAAGACAGGG	0	0	0	0
tsrna-08541	AAGCATTGGACTGTAAATCTAAAGACAGG	2	0	1	0
tsrna-08540	AAGCATTGGACTGTAAATCTAAAGACA	0	2	1	0
tsrna-08538	AAGCATTGGACTGTAAATCTAAAGA	0	0	0	0
tsrna-08537	AAGCATTGGACTGTAAATCTAAAG	0	0	1	0
tsrna-08536	AAGCATTGGACTGTAAATCTAAA	0	0	0	0
tsrna-08533	AAGCATTGGACTGTAAATCT	0	0	1	0
tsrna-08528	AAGCATAGCTGCCTTCCAAGC	0	0	1	0
tsrna-08527	AAGCAGTTGACCCGGGTTCGATTCCCGGCCAACGCACC	12	29	2	1
tsrna-08526	AAGCAGTTGACCCGGGTTCG	0	1	1	0
tsrna-08525	AAGCAGTTGACCCGGGT	1	1	1	0
tsrna-08524	AAGCAGCTTCAAACCTGCCGGGGCTTCC	1	0	2	0
tsrna-08523	AAGCAGCTTCAAACCTGCCGGGGCTT	0	0	0	0
tsrna-08522	AAGCAGCTTCAAACCTGCCGGGGCT	0	1	0	0
tsrna-08521	AAGCAGCTTCAAACCTGCCGGGGC	0	1	0	0
tsrna-08518	AAGCAGAGTGGCGCAGCGGAAGCGTGCTGG	0	9	0	1
tsrna-08517	AAGCACTCTGGACTCTGAATCCAGCGATCC	2	4	1	1
tsrna-08516	AAGCACTCTGGACTCTGAATCCAGCGA	1	4	0	1
tsrna-08515	AAGCACTCTGGACTCTGAATCCAGCG	2	1	0	0
tsrna-08514	AAGCACTCTGGACTCTGAATCCAGC	2	2	1	1
tsrna-08512	AAGCACTCTGGACTCTGAATCC	2	3	0	0
tsrna-08505	AAGCACCCAACTTACACTTAGGAGATTTC	0	1	2	0
tsrna-08504	AAGCACCCAACTTACACTTAGGAGATTT	0	0	0	0
tsrna-08503	AAGCACCCAACTTACACTTAGGAGATT	0	0	0	0
tsrna-08502	AAGCACCCAACTTACACTTAGGAGAT	0	0	0	0
tsrna-08501	AAGCACCCAACTTACACTTAGGAGA	0	2	0	0
tsrna-08500	AAGCACCCAACTTACACTTAGGAG	0	1	0	0
tsrna-08499	AAGCACCCAACTTACACTTAGGA	1	0	0	0
tsrna-08491	AAGCAATACACTGAAAATGTTTAGACGGGCTC	13	14	4	2
tsrna-08490	AAGCAATACACTGAAAATGTTTAGACGGGCT	0	2	0	0
tsrna-08489	AAGCAATACACTGAAAATGTTTAGACGGGC	4	3	0	1
tsrna-08487	AAGATTGAGGGTTCGAGTCCCTTCGTGGTCGCCA	5	18	4	2
tsrna-08486	AAGATTGAGGGTTCGAGTCCCTTCGTGGTCGCC	3	5	4	2
tsrna-08485	AAGATTGAGGGTTCGAGTCCCT	4	6	1	1
tsrna-08484	AAGATTCTAGGTTCGACTCCTGGCTGGCTC	1	4	1	1
tsrna-08483	AAGATTCTAGGTTCGACTCCTG	1	4	0	1
tsrna-08482	AAGATTCTAGGTTCGACTCC	0	4	2	1
tsrna-08481	AAGATTCCCATTCTTGCGACCCGG	1	2	3	0
tsrna-08480	AAGATTCCCATTCTTGCGACCCG	3	4	0	0
tsrna-08478	AAGATTCCCATTCTTGCGAC	2	2	0	0
tsrna-08477	AAGATTCCAGGTTCGACTCCTGGCTGGCTC	1	1	0	0
tsrna-08476	AAGATTCCAGGTTCGACTCCTG	1	1	0	0
tsrna-08475	AAGATTCCAGGTTCGACTCC	1	0	0	0

tsrna-08474	AAGATTCCAGGTTCGACTC	0	0	0	0
tsrna-08473	AAGATTCCAGGTTCGACT	0	0	0	0
tsrna-08472	AAGATTCCAGGTTCGAC	1	0	0	1
tsrna-08471	AAGATTAAGAGAACCAACACCTCTTTACAGTGACCA	2	1	3	1
tsrna-08470	AAGATTAAGAGAACCAACACCTCTTTACAGTGACC	1	2	2	0
tsrna-08469	AAGATTAAGAGAACCAACACCTCTTTACAGTGAC	0	0	0	0
tsrna-08468	AAGATTAAGAGAACCAACACCTCTTTACAG	0	0	0	0
tsrna-08467	AAGATTAAGAGAACCAACACCTCTTTACA	0	0	0	0
tsrna-08466	AAGATTAAGAGAACCAACACCTCT	0	0	0	0
tsrna-08465	AAGATTAAGAGAACCAACACCTC	1	0	0	0
tsrna-08464	AAGATTAAGAGAACCAACACCT	0	0	0	0
tsrna-08463	AAGATTAAGAGAACCAACACC	0	0	0	0
tsrna-08448	AAGATCACGGGTTCGAACCCCG	0	0	1	0
tsrna-08447	AAGAGGTCCCTGGTTCA	1	8	2	1
tsrna-08446	AAGAGGTCCCTGGTTC	2	3	3	0
tsrna-08445	AAGAGGTCCCGGGTTCAAATCC	0	2	0	1
tsrna-08444	AAGAGGTCCCCGGTTCAAATCCGGGTGCCCCCTCC	7	7	2	1
tsrna-08443	AAGAGGTCCCCGGTTCAAA	3	4	0	0
tsrna-08442	AAGAGGTCCCCGGTTCA	1	3	0	0
tsrna-08441	AAGAGGTCCCCGGTTC	3	1	0	0
tsrna-08436	AAGAGAACCAACACCTCTTTACAGTGACCA	0	2	0	0
tsrna-08435	AAGAGAACCAACACCTCTTTACAGTGACC	1	2	1	0
tsrna-08433	AAGAGAACCAACACCTCTTTACAGTG	0	1	0	0
tsrna-08427	AAGACTTTTTCTCTGACCA	11	7	4	8
tsrna-08426	AAGACTTTTTCTCTGACC	0	0	3	0
tsrna-08425	AAGACTTTTTCTCTGAC	1	0	0	1
tsrna-08423	AAGACAGGGGTTAGGCCTCTTTTTACCACCA	4	5	8	1
tsrna-08422	AAGACAGGGGTTAGGCCTCTTTTTACCACC	0	1	2	0
tsrna-08421	AAGACAGGGGTTAGGCCTCTTTTTACCAC	4	0	0	0
tsrna-08420	AAGACAGGGGTTAGGCCTCTTTTTACCA	1	2	1	1
tsrna-08419	AAGACAGGGGTTAGGCCTCTTTTTACC	0	1	2	0
tsrna-08418	AAGACAGGGGTTAGGCCTCTTTTTAC	0	1	0	0
tsrna-08417	AAGACAGGGGTTAGGCCTCTT	0	0	1	0
tsrna-08416	AAGACAGGGGTTAGGCCTCT	1	1	0	0
tsrna-08415	AAGACAGGGGTTAGGCCTC	0	1	0	0
tsrna-08413	AAGACAGGGGTTAGGCC	0	1	0	0
tsrna-08412	AAGAAGCAGCTTCAAACCTGCCGGGGCTTCC	0	6	0	0
tsrna-08411	AAGAAGCAGCTTCAAACCTGCCGGGGCTTC	0	1	0	0
tsrna-08410	AAGAAGCAGCTTCAAACCTGCCGGGGCTT	1	0	0	0
tsrna-08409	AAGAAGCAGCTTCAAACCTGCCGGGGCT	0	0	0	0
tsrna-08408	AAGAAGCAGCTTCAAACCTGCCGGGGC	1	0	1	0
tsrna-08405	AAGAAGCAGCTTCAAACC	0	1	0	0
tsrna-08404	AAGAACTGCTAACTCATGCCCCCATGTCTAACAACATGGCTTT	7	3	8	0
tsrna-08403	AAGAACTGCTAACTCATGCCCCCATGTCTAACAACATGGCTTT	4	0	5	1
tsrna-08402	AAGAACTGCTAACTCATGCCCCCATGTCTAACAACATGGCTTT	3	1	2	1
tsrna-08401	AAGAACTGCTAACTCATGCCCCCATGTCTAACAACATGGCTTT	1	0	1	0
tsrna-08400	AAGAACTGCTAACTCATGCCCCCATGTCTAACAACATGGCTT	2	4	2	0
tsrna-08399	AAGAACTGCTAACTCATGCCCCCATGTCTAACAACATGGCT	2	1	1	0
tsrna-08398	AAGAACTGCTAACTCATGCCCCCATGTCTAACAACATGGC	1	2	2	1
tsrna-08397	AAGAACTGCTAACTCATGCCCCCATGTCTAACAACATGG	0	4	3	0
tsrna-08396	AAGAACTGCTAACTCATGCCCCCATGTCTAACAACATG	2	3	4	0
tsrna-08395	AAGAACTGCTAACTCATGCCCCCATGTCTAACAACA	1	0	2	0
tsrna-08394	AAGAACTGCTAACTCATGCCCCCATGTCTAACA	2	1	1	0
tsrna-08393	AAGAACTGCTAACTCATGCCCCCATGTCTAAC	1	0	0	1
tsrna-08392	AAGAACTGCTAACTCATGCCCCCATGTCT	0	3	1	0
tsrna-08391	AAGAACTGCTAACTCATGCCCCCATGTC	1	1	0	1
tsrna-08390	AAGAACTGCTAACTCATGCCCCCATGT	0	1	0	0
tsrna-08389	AAGAACTGCTAACTCATGCCCCCATG	0	0	0	0
tsrna-08388	AAGAACTGCTAACTCATGCCCCCAT	0	0	1	0
tsrna-08387	AAGAACTGCTAACTCATGCCCCCA	0	0	1	1
tsrna-08386	AAGAACTGCTAACTCATGCCCCC	0	0	0	0
tsrna-08385	AAGAACTGCTAACTCATGCCCC	0	0	0	0
tsrna-08384	AAGAACTGCTAACTCATGCCC	0	1	0	0
tsrna-08382	AAGAACTGCTAACTCATGC	0	0	0	0

tsrna-08381	AAGAACTGCTAACTCATG	1	0	0	0
tsrna-08379	AAGAACTGCTAACTCA	0	0	0	0
tsrna-08378	AACTTTGTCAAAGTTAAATTATAGGCTAA	5	1	11	0
tsrna-08377	AACTTTGTCAAAGTTAAATTATAGGCT	5	1	6	1
tsrna-08376	AACTTTGTCAAAGTTAAATTATAGGC	6	1	8	1
tsrna-08375	AACTTTACAGTCAGAGGTTCA	1	0	1	0
tsrna-08374	AACTTTACAGTCAGAGGTTC	2	1	0	0
tsrna-08370	AACTTGACCGCTCTGACCA	7	15	4	0
tsrna-08369	AACTTGACCGCTCTGACC	1	2	1	0
tsrna-08368	AACTTGACCGCTCTGAC	0	0	1	0
tsrna-08366	AACTTCCAATTAACTAGTTTTGACAACATT	1	1	0	0
tsrna-08363	AACTTCCAATTAACTAGTTTTGACAAC	0	0	0	0
tsrna-08362	AACTTCCAATTAACTAGTTTTGACAA	0	0	1	0
tsrna-08361	AACTTCCAATTAACTAGTTTTGACA	0	0	1	0
tsrna-08355	AACTTAGCATTAACCTTTTAAGTTAAAGATTAAGAGAACC	0	1	0	0
tsrna-08354	AACTTAGCATTAACCTTTTAAGTTAAAGATTAAGAG	1	0	0	0
tsrna-08353	AACTTAGCATTAACCTTTTAAGTTA	1	0	0	0
tsrna-08347	AACTTACACTTAGGAGATTTCAACTTAACTTGACCGCTCTGAC	91	47	87	10
tsrna-08346	AACTTACACTTAGGAGATTTCAACTTAACTTGACCGCTCTGAC	13	16	18	1
tsrna-08345	AACTTACACTTAGGAGATTTCAACTTAACTTGACCGCTCTGAC	4	0	8	0
tsrna-08344	AACTTACACTTAGGAGATTTCAACTTAACTTGACCGCTCTGA	4	4	7	0
tsrna-08343	AACTTACACTTAGGAGATTTCAACTTAACTTGACCGCTCTG	5	1	6	0
tsrna-08342	AACTTACACTTAGGAGATTTCAACTTAACTTGACCGCTCT	0	2	5	0
tsrna-08341	AACTTACACTTAGGAGATTTCAACTTAACTTGACCGCTC	0	4	9	1
tsrna-08340	AACTTACACTTAGGAGATTTCAACTTAACTTGACCGCT	3	1	7	0
tsrna-08339	AACTTACACTTAGGAGATTTCAACTTAACTTGACCGC	0	1	7	0
tsrna-08338	AACTTACACTTAGGAGATTTCAACTTAACTTGACCG	3	0	15	0
tsrna-08337	AACTTACACTTAGGAGATTTCAACTTAACTTGACC	6	1	6	0
tsrna-08336	AACTTACACTTAGGAGATTTCAACTTAACT	0	0	2	0
tsrna-08334	AACTTACACTTAGGAGATTTCAACTTA	0	0	4	0
tsrna-08333	AACTTACACTTAGGAGATTTCAACTT	0	0	1	0
tsrna-08332	AACTTACACTTAGGAGATTTCAACT	2	0	1	0
tsrna-08331	AACTTACACTTAGGAGATTTCAAC	0	0	0	0
tsrna-08329	AACTTACACTTAGGAGATTTCA	0	0	1	0
tsrna-08328	AACTTACACTTAGGAGATTTC	0	1	0	0
tsrna-08322	AACTTAACTTGACCGCTCTGACCA	21	17	16	1
tsrna-08321	AACTTAACTTGACCGCTCTGACC	1	1	3	0
tsrna-08320	AACTTAACTTGACCGCTCTGAC	0	0	0	0
tsrna-08319	AACTTAACTTGACCGCTCTGA	3	0	0	0
tsrna-08318	AACTTAACTTGACCGCTCTG	0	0	0	1
tsrna-08317	AACTTAACTTGACCGCTCT	0	0	0	0
tsrna-08308	AACTTAAAACTTTACAATCAG	0	0	0	0
tsrna-08307	AACTGCTAACTCATGCCCCCATGTCTAACAACATGGCTTTCTC	11	5	42	0
tsrna-08306	AACTGCTAACTCATGCCCCCATGTCTAACAACATGGCTTTCTC	8	1	21	0
tsrna-08305	AACTGCTAACTCATGCCCCCATGTCTAACAACATGGCTTTCTC	1	5	3	0
tsrna-08304	AACTGCTAACTCATGCCCCCATGTCTAACAACATGGCT	1	1	0	1
tsrna-08303	AACTGCTAACTCATGCCCCCATGTCTAACAACATGGC	0	1	0	1
tsrna-08302	AACTGCTAACTCATGCCCCCATGTCTAACAACATGG	1	1	0	0
tsrna-08301	AACTGCTAACTCATGCCCCCATGTCTAACAACA	1	0	0	0
tsrna-08300	AACTGCTAACTCATGCCCCCATGTCTAACA	1	0	0	0
tsrna-08299	AACTGCTAACTCATGCCCCCATGTCTAAC	0	1	0	0
tsrna-08298	AACTGCTAACTCATGCCCCCATGTCT	0	0	0	0
tsrna-08296	AACTGCTAACTCATGCCCCCATGT	0	1	0	0
tsrna-08295	AACTGCTAACTCATGCCCCCATG	0	0	0	1
tsrna-08294	AACTGCTAACTCATGCCCCCAT	0	0	0	0
tsrna-08284	AACTCATGCCCCCATGTCTAACAACATGGCTTTCTCACCA	16	10	33	1
tsrna-08283	AACTCATGCCCCCATGTCTAACAACATGGCTTTCTCACC	3	1	10	1
tsrna-08282	AACTCATGCCCCCATGTCTAACAACATGGCT	0	0	2	0
tsrna-08281	AACTCATGCCCCCATGTCTAACAACATGGC	0	0	0	0
tsrna-08277	AACTCATGCCCCCATGTCTAACA	0	0	0	0
tsrna-08266	AACTAATACACCAGTCTTG	1	2	4	0
tsrna-08265	AACTAAGTGTTTGTGGGTTTAAGTCCCATTGGTCTAGCCA	2	2	4	1
tsrna-08264	AACTAAGTGTTTGTGGGTTTAAGTCCCATTGGTCTA	4	0	3	0
tsrna-08263	AACTAAGTGTTTGTGGGTTTA	0	1	0	0

tsrna-08262	AACTAAGTGTTTGTGGGTTT	1	0	1	1
tsrna-08261	AACTAAGTGTTTGTGGGTT	0	0	0	0
tsrna-08260	AACTAAGTGTTTGTGGG	0	1	0	0
tsrna-08258	AACGGTAGCGCGTCTGACTCC	3	5	0	6
tsrna-08257	AACGGTAGCGCGTCTGACT	0	2	0	0
tsrna-08255	AACGCCAAGGTCGCGGGTTCG	0	1	2	0
tsrna-08253	AACGATGGTTTTTCATATCATTGGTCGTGGTTGTAGTCCGTGC	19	19	18	11
tsrna-08252	AACGATGGTTTTTCATATCATTGGTCGTGGTTGTAGTCC	4	1	6	0
tsrna-08251	AACGATGGTTTTTCATATCATTGGTCGTGGTTGTAGTC	0	5	2	0
tsrna-08250	AACGATGGTTTTTCATATCATTGGTCGTGGTTGTAGT	4	1	5	0
tsrna-08249	AACGATGGTTTTTCATATCATTGGTCGTGG	1	0	0	0
tsrna-08248	AACGATGGTTTTTCATATCATTGGTCG	0	0	0	0
tsrna-08247	AACGATGGTTTTTCATATCA	0	1	0	0
tsrna-08246	AACGATGGTTTTTCATATC	0	0	1	0
tsrna-08245	AACGAATGATTTCGACTCATTAAATTATG	1	2	3	0
tsrna-08244	AACGAATGATTTCGACTCATT	0	3	0	1
tsrna-08243	AACGAATGATTTCGACTCA	0	2	1	0
tsrna-08240	AACCTTTTTCCAAGGACACCA	4	2	3	0
tsrna-08239	AACCTTTTTCCAAGGACACC	1	0	0	0
tsrna-08238	AACCTTTTAAGTTAAAGATTAAGAGAACC	0	1	0	0
tsrna-08237	AACCTGCCGGGGCTTCCA	0	1	0	0
tsrna-08233	AACCGGGCGGAAACACCA	257	703	103	40
tsrna-08232	AACCGGGCGGAAACACC	63	191	41	9
tsrna-08231	AACCGGGCGGAAACAC	0	1	0	0
tsrna-08230	AACCGGGCAGAAGCACCA	33	124	13	2
tsrna-08229	AACCGGGCAGAAGCACC	0	5	2	1
tsrna-08228	AACCGGGCAGAAGCAC	0	0	0	0
tsrna-08227	AACCGGAGATGAAAACCTTTTTCCAAGGACACCA	34	37	103	0
tsrna-08226	AACCGGAGATGAAAACCTTTTTCCAAGGACACC	6	19	12	1
tsrna-08225	AACCGGAGATGAAAACCTTTTTCCAAGGAC	1	13	4	0
tsrna-08224	AACCGGAGATGAAAACCTTTTTCCAAGG	2	10	0	0
tsrna-08223	AACCGGAGATGAAAACCTTTTTCCAAG	3	10	2	0
tsrna-08222	AACCGGAGATGAAAACCTTTTTCCAA	0	5	1	0
tsrna-08221	AACCGGAGATGAAAACCTT	1	0	0	0
tsrna-08220	AACCGGAGATGAAAACCT	0	0	0	0
tsrna-08219	AACCGGAGATGAAAACC	0	0	0	0
tsrna-08217	AACCGAAGATCGCGGGTTCGAACC	2	11	3	0
tsrna-08216	AACCGAAGATCGCGGGTTCGAAC	4	3	0	0
tsrna-08215	AACCGAAGATCGCGGGTTCGAA	2	14	1	1
tsrna-08214	AACCGAAGATCGCGGGTTCGA	7	6	0	0
tsrna-08213	AACCGAAAGGTTGGTGGTTCG	0	1	0	0
tsrna-08211	AACCCTGCTCGCTGCGCCA	15	28	5	1
tsrna-08210	AACCCCGTCCGTGCCTCCA	4	11	0	1
tsrna-08209	AACCCCCTTATTTCTACCA	0	1	1	0
tsrna-08208	AACCCCCTTATTTCTACC	0	2	0	0
tsrna-08205	AACCCAGAGGTCGATGGATCGAAACC	1	1	1	1
tsrna-08203	AACCCAGAGGTCGATGGATCGAA	1	2	1	1
tsrna-08202	AACCCAGAGGTCGATGGATCGA	0	1	1	0
tsrna-08201	AACCCAGAGGTCGATGGATCG	0	1	0	0
tsrna-08200	AACCCAGAGGTCGATGGATC	0	2	1	1
tsrna-08199	AACCCAGAGGTCGATGGAT	0	3	0	0
tsrna-08198	AACCCAGAGGTCGATGGA	0	3	0	0
tsrna-08197	AACCCAGAGGTCGATGG	1	0	0	0
tsrna-08196	AACCCAGAGGTCGATG	1	0	0	0
tsrna-08195	AACCCAGAGGTCAATGGATCGAAG	0	0	0	1
tsrna-08194	AACCCAGAGGTCAATGGATCGA	0	0	0	1
tsrna-08193	AACCATTTCATAACTTTGTCA	0	1	0	0
tsrna-08192	AACCATTTCATAACTTTGTC	0	1	1	0
tsrna-08191	AACCATCCTCTGCTACCA	2	6	1	0
tsrna-08190	AACCATCCTCTGCTACC	1	0	0	0
tsrna-08189	AACCATCCTCTGCTAC	0	0	0	0
tsrna-08188	AACCAGGGGTCGCGAGTTCA	0	0	0	0
tsrna-08187	AACCAGGGGTCGCGAGTTC	0	2	0	0
tsrna-08186	AACCAGGGGTCGCGAGTT	0	1	0	0

tsrna-08185	AACCAGGGGTCGCGAGT	0	1	2	0
tsrna-08184	AACCAGGGGTCGCGAG	0	0	0	0
tsrna-08183	AACCAGGCGGAAACACCA	4	14	3	0
tsrna-08182	AACCAGCTTTGGGGGGTTC	0	1	0	0
tsrna-08180	AACCAACACCTCTTTACAGTGACCA	0	0	1	0
tsrna-08173	AACCAAAACATCAGATTGTGAATCTGACAACAGAGGCTTACGA	2	9	7	1
tsrna-08172	AACCAAAACATCAGATTGTGAATCTGACAACAGAGGCT	3	3	3	0
tsrna-08171	AACCAAAACATCAGATTGTGAATCTGACAACAGAGG	1	3	1	0
tsrna-08170	AACCAAAACATCAGATTGTGAATCTGACAA	1	2	1	0
tsrna-08169	AACCAAAACATCAGATTGTGAATCTGACA	1	1	2	0
tsrna-08168	AACCAAAACATCAGATTGTGAATCTGAC	0	1	0	0
tsrna-08167	AACCAAAACATCAGATTGTGAATCT	0	1	0	0
tsrna-08157	AACATGGCTTTCTCACCA	0	1	0	0
tsrna-08154	AACATCAGATTGTGAATCTGACAACAGAGGCTTACGACCCCTT	25	16	40	0
tsrna-08153	AACATCAGATTGTGAATCTGACAACAGAGGCTTACGACCCCT	10	15	8	0
tsrna-08152	AACATCAGATTGTGAATCTGACAACAGAGGCTTACGACCCC	5	12	8	0
tsrna-08151	AACATCAGATTGTGAATCTGACAACAGAGGCTTACGACCC	2	7	5	0
tsrna-08150	AACATCAGATTGTGAATCTGACAACAGAGGCTTACGACC	4	5	8	0
tsrna-08149	AACATCAGATTGTGAATCTGACAACAGAGGCTTACGAC	4	7	5	1
tsrna-08148	AACATCAGATTGTGAATCTGACAACAGAGGCTTACGA	2	7	3	1
tsrna-08147	AACATCAGATTGTGAATCTGACAACAGAGGCTTACG	2	7	4	1
tsrna-08146	AACATCAGATTGTGAATCTGACAACAGAGGCTTAC	2	7	6	0
tsrna-08145	AACATCAGATTGTGAATCTGACAACAGAGGCTT	1	11	3	1
tsrna-08144	AACATCAGATTGTGAATCTGACAACAGAGGCT	1	8	4	1
tsrna-08143	AACATCAGATTGTGAATCTGACAACAGAGGC	2	8	1	0
tsrna-08142	AACATCAGATTGTGAATCTGACAACAGAGG	3	5	2	0
tsrna-08141	AACATCAGATTGTGAATCTGACAACAGAG	2	3	6	0
tsrna-08140	AACATCAGATTGTGAATCTGACAACAGA	1	2	3	0
tsrna-08139	AACATCAGATTGTGAATCTGACAACAG	0	3	4	0
tsrna-08138	AACATCAGATTGTGAATCTGACAACA	1	2	1	0
tsrna-08137	AACATCAGATTGTGAATCTGACAAC	1	3	1	0
tsrna-08136	AACATCAGATTGTGAATCTGACAA	0	2	2	0
tsrna-08135	AACATCAGATTGTGAATCTGACA	0	0	1	0
tsrna-08128	AACAGGAGATCCTGGGTTCGAATCCCAGCGGTGCCT	6	21	3	2
tsrna-08127	AACAGGAGATCCTGGGTTCGAATCCCAGCGGTGCC	10	17	4	1
tsrna-08126	AACAGGAGATCCTGGGTTCGAATCCCAGCGGTGC	6	24	4	0
tsrna-08125	AACAGGAGATCCTGGGTTCGAATCCCAGCGGGGCCT	6	22	6	2
tsrna-08124	AACAGGAGATCCTGGGTTCGAATCCCAGCGGGGCC	7	13	6	1
tsrna-08123	AACAGCTATCCATTGGTCTTAGGCCCC	1	0	0	0
tsrna-08122	AACAGCTATCCATTGGTCTTAGGCCC	0	0	0	0
tsrna-08121	AACAGCTATCCATTGGTCTTAGGCC	0	0	0	0
tsrna-08120	AACAGCTATCCATTGGTCTTAGGC	0	0	0	0
tsrna-08119	AACAGCTATCCATTGGTCTTAGG	0	0	0	0
tsrna-08118	AACAGCTATCCATTGGTCTTAG	0	1	0	0
tsrna-08117	AACAGCTATCCATTGGTCTTA	0	0	0	0
tsrna-08115	AACAGCTATCCATTGGTCT	0	0	1	0
tsrna-08101	AACACCAAGGTCGCGGGCTCGA	0	1	1	0
tsrna-08100	AACACAAAGCACCCAACTTACACTTAGGAG	0	0	0	0
tsrna-08099	AACACAAAGCACCCAACTTACACTTAGGA	0	0	0	0
tsrna-08098	AACACAAAGCACCCAACTTACACTTAG	0	0	1	0
tsrna-08097	AACACAAAGCACCCAACTTACACTTA	0	0	0	0
tsrna-08095	AACACAAAGCACCCAACTTACA	0	0	1	0
tsrna-08089	AACAACATGGCTTTCTCACCA	0	1	1	0
tsrna-08088	AACAACATGGCTTTCTCACC	0	0	0	0
tsrna-08087	AACAACATGGCTTTCTCAC	0	0	0	0
tsrna-08084	AACAAAACGAATGATTTCGACTCA	2	1	1	0
tsrna-08083	AACAAAACGAATGATTTCGACTC	0	4	2	0
tsrna-08080	AAATTTTGGTGCAACTCCAAATA	0	2	0	0
tsrna-08079	AAATTTTGGTGCAACTCCA	0	1	0	0
tsrna-08076	AAATTCGAAGAAGCAGCTTCAAACCTGCCGGGGCTT	0	2	2	0
tsrna-08075	AAATTCGAAGAAGCAGCTTCAAACCTGCCGGGGCT	0	1	1	0
tsrna-08073	AAATTATGATAATCATATTTACCAACC	0	0	0	0
tsrna-08069	AAATTAGAATCTTAGCTTTGGGTGCTAATGGTGGAGTTAAAGA	6	2	2	1
tsrna-08068	AAATTAGAATCTTAGCTTTGGGTGCTAATGGTGGAG	1	4	3	1

tsrna-08067	AAATTAGAATCTTAGCTTTGGGTGCTAATG	7	0	4	0
tsrna-08066	AAATTAGAATCTTAGCTTTGGGTGC	3	3	1	0
tsrna-08065	AAATTAGAATCTTAGCTTTGGGTG	0	1	2	1
tsrna-08064	AAATTAGAATCTTAGCTTTGG	2	0	1	2
tsrna-08063	AAATGTTTAGACGGGCTCACATCACCCCATAAA	46	15	30	2
tsrna-08062	AAATGTTTAGACGGGCTCACATCACCCCATA	51	15	24	7
tsrna-08061	AAATGTTTAGACGGGCTCACATCACC	21	14	25	4
tsrna-08060	AAATGTTTAGACGGGCTCACAT	24	5	12	6
tsrna-08059	AAATGTTTAGACGGGCTCACA	17	7	9	2
tsrna-08058	AAATGTTTAGACGGGCTCAC	13	10	4	1
tsrna-08057	AAATGTTTAGACGGGCTCA	3	1	2	0
tsrna-08056	AAATGTTTAGACGGGCTC	3	3	1	1
tsrna-08055	AAATGTTTAGACGGGCT	0	0	0	1
tsrna-08054	AAATGTTTAGACGGGC	0	1	0	0
tsrna-08053	AAATGTTGGTTATACCCTTCCCGTACTACCA	3	20	2	3
tsrna-08052	AAATGTTGGTTATACCCTTCCCGTACTACC	1	0	0	0
tsrna-08046	AAATGGTAGAGCGCTCGC	1	1	0	0
tsrna-08045	AAATGGCTGAGTGAAGCATTGGACTGTAA	2	4	1	0
tsrna-08044	AAATGGCTGAGTGAAGCATTGGACTGTA	1	2	0	0
tsrna-08043	AAATGGCTGAGTGAAGCATTGGACTGT	0	3	1	0
tsrna-08042	AAATGGCTGAGTGAAGCATTGGACTG	2	0	0	0
tsrna-08041	AAATGGCTGAGTGAAGCATTGGACT	0	0	1	0
tsrna-08040	AAATGGCTGAGTGAAGCATTGGAC	0	1	1	0
tsrna-08039	AAATGGCTGAGTGAAGCATTGGA	1	1	0	0
tsrna-08037	AAATGGCTGAGTGAAGCATTG	0	0	1	0
tsrna-08036	AAATGGCTGAGTGAAGC	0	2	1	0
tsrna-08034	AAATCTGGGTGCCCCCTCCA	1	0	1	0
tsrna-08033	AAATCTCGGTGGGACCTCCA	57	95	25	20
tsrna-08032	AAATCTCGGTGGGACCTCC	5	8	3	1
tsrna-08031	AAATCTCGGTGGGACCTC	0	0	0	0
tsrna-08030	AAATCTCGGTGGGACCCCCA	5	8	2	1
tsrna-08029	AAATCTCGGTGGAACCTCCA	105	174	23	21
tsrna-08028	AAATCTCGGTGGAACCTCC	8	9	6	0
tsrna-08027	AAATCTCGGTGGAACCTC	0	2	0	0
tsrna-08026	AAATCTCGGTGGAACCT	0	1	0	0
tsrna-08025	AAATCTCGGTGGAACC	0	1	0	0
tsrna-08024	AAATCTCGCTGGGGCCTCCA	32	58	22	9
tsrna-08023	AAATCTAAAGACAGGGGTTAGGCCTCTTTTTACC	36	32	18	0
tsrna-08022	AAATCTAAAGACAGGGGTTAGGCCTC	1	2	0	0
tsrna-08021	AAATCCTATATATCTTACCA	0	1	0	0
tsrna-08019	AAATCCGGGTGCCCCCTCCA	137	180	62	18
tsrna-08018	AAATCCGGGTGCCCCCTCC	3	8	0	0
tsrna-08017	AAATCCGGGTGCCCCCTC	1	0	0	0
tsrna-08016	AAATCCCGGACGAGCCCCCA	110	154	48	39
tsrna-08015	AAATCCCGGACGAGCCCCC	7	6	0	2
tsrna-08014	AAATCCCGGACGAGCCCC	3	3	1	0
tsrna-08013	AAATCCCGGACGAGCCC	2	3	0	0
tsrna-08011	AAATCCAGGTGCCCCCTCCA	0	2	1	2
tsrna-08010	AAATCACGTCGGGGTCACCA	1	7	1	7
tsrna-08009	AAATCACGTCGGGGTCACC	0	1	1	0
tsrna-08004	AAATAGTACCGTTAACTTCCAATTAACTAG	3	1	0	0
tsrna-08003	AAATAGTACCGTTAACTTCCA	0	0	1	0
tsrna-08002	AAATAGTACCGTTAACTTCC	0	0	0	0
tsrna-08000	AAATAGTACCGTTAACT	0	1	0	0
tsrna-07997	AAATACAGACCAAGAGCCTTCA	2	1	3	2
tsrna-07996	AAATACAGACCAAGAGCCTTC	1	0	0	0
tsrna-07995	AAATACAGACCAAGAGCCT	0	0	0	0
tsrna-07994	AAATACAGACCAAGAGCC	0	0	0	0
tsrna-07991	AAATACAACGATGGTTTTTCATATCATTGGTCGTGGTTGTAGT[illegible]	5	1	4	1
tsrna-07990	AAATACAACGATGGTTTTTCATATCATTGGTCGTGGTTGTAGT[illegible]	3	1	4	0
tsrna-07989	AAATACAACGATGGTTTTTCATATCATTGGTCGTGGTTGTA	1	0	7	0
tsrna-07988	AAATACAACGATGGTTTTTCATATCATTGGTCGT	1	0	0	0
tsrna-07986	AAATACAACGATGGTTTTTC	0	0	1	0
tsrna-07985	AAATAATAGGAGCTTAAACCCCCTTATTTCTACC	2	0	0	0

tsrna-07984	AAATAATAGGAGCTTAAACCCCCTTATTTC	1	0	0	1
tsrna-07983	AAATAATAGGAGCTTAAACCCCCTT	0	0	0	0
tsrna-07982	AAATAATAGGAGCTTAAACCCCCT	0	1	0	0
tsrna-07980	AAATAATAGGAGCTTAAACCCCC	0	0	0	1
tsrna-07977	AAATAAGCTATCGGGCCCATACCCCGAAA	0	3	1	0
tsrna-07976	AAATAAGCTATCGGGCCCATACCCCGAA	0	2	3	1
tsrna-07975	AAATAAGCTATCGGGCCCATACCCCGA	1	3	2	0
tsrna-07974	AAATAAGCTATCGGGCCCATACCCCG	0	0	0	0
tsrna-07973	AAATAAGCTATCGGGCCCATACC	1	0	3	0
tsrna-07972	AAATAAGCTATCGGGCCCATA	1	2	0	0
tsrna-07971	AAATAAGCTATCGGGCCCAT	0	0	0	0
tsrna-07970	AAATAAGCTATCGGGCCCA	0	1	0	0
tsrna-07967	AAAGTTAAATTATAGGCT	1	0	0	0
tsrna-07966	AAAGTGGCTGATTTGCGTTCA	0	2	0	0
tsrna-07962	AAAGTGGCTGATTTGCG	0	0	0	0
tsrna-07961	AAAGTGGCTGATTTGC	0	1	0	0
tsrna-07960	AAAGTCATGGAGGCCATGGGGTTGG	2	3	1	0
tsrna-07959	AAAGTCATGGAGGCCATGGGGTTG	1	2	2	0
tsrna-07958	AAAGTCATGGAGGCCATGGGGTT	0	2	1	0
tsrna-07957	AAAGTCATGGAGGCCATGGGGT	0	0	0	0
tsrna-07956	AAAGTCATGGAGGCCATGGGG	0	0	0	0
tsrna-07954	AAAGTCATGGAGGCCATGG	0	0	0	0
tsrna-07952	AAAGTCATGGAGGCCA	0	0	0	0
tsrna-07951	AAAGGTTGGTGGTTCGAGCCCACCCAGGGACGCCA	5	112	2	1
tsrna-07949	AAAGGTTCCGGGTTCGAGTCCCGGCGGAGTCGC	0	0	0	0
tsrna-07948	AAAGGTTCCGGGTTCG	1	0	0	0
tsrna-07947	AAAGGTCCCTGGTTCGATCCC	9	15	1	2
tsrna-07946	AAAGGTCCCTGGTTCGATCC	5	15	2	4
tsrna-07945	AAAGGTCCCTGGTTCG	4	5	3	2
tsrna-07944	AAAGGTCCCTGGTTCAATCCC	7	9	7	1
tsrna-07943	AAAGGTCCCCGGTTCGAAACC	0	2	0	0
tsrna-07942	AAAGGTCCCCGGTTCGAAAC	0	3	0	1
tsrna-07940	AAAGGATAACAGCTATCCATTGGTCTTAG	0	0	0	0
tsrna-07939	AAAGGATAACAGCTATCCATTGGTCTTA	0	1	0	0
tsrna-07938	AAAGGATAACAGCTATCCATTGGTCTT	0	0	0	0
tsrna-07937	AAAGGATAACAGCTATCCATTGGTCT	1	0	0	0
tsrna-07935	AAAGGATAACAGCTATCCATTGG	0	0	0	0
tsrna-07932	AAAGCTCACAAGAACTGCTAACTCATGCCCCCATGT	0	3	0	1
tsrna-07931	AAAGCTCACAAGAACTGCTAACTCATGCCC	0	2	0	0
tsrna-07930	AAAGCTCACAAGAACTGCTAACTCATGCC	1	0	0	0
tsrna-07929	AAAGCTCACAAGAACTGCTAACTCATGC	1	1	0	0
tsrna-07928	AAAGCTCACAAGAACTGCTAACTCATG	0	0	0	0
tsrna-07927	AAAGCTCACAAGAACTGCTAACTCAT	1	1	0	0
tsrna-07926	AAAGCTCACAAGAACTGCTAACTCA	1	2	2	0
tsrna-07925	AAAGCTCACAAGAACTGCTAACTC	1	1	0	0
tsrna-07924	AAAGCTCACAAGAACTGCTAACT	0	1	0	0
tsrna-07923	AAAGCTCACAAGAACTGCT	1	0	0	0
tsrna-07919	AAAGCACCCAACTTACACTTAGGAGATTT	0	0	0	0
tsrna-07918	AAAGCACCCAACTTACACTTAGGAG	0	0	0	0
tsrna-07917	AAAGCACCCAACTTACACTTAGG	0	0	0	0
tsrna-07912	AAAGCAATACACTGAAAATGTTTAGACGGGCTCACATCACC	116	47	89	8
tsrna-07911	AAAGCAATACACTGAAAATGTTTAGACGGGCTC	7	9	4	1
tsrna-07910	AAAGCAATACACTGAAAATGTTTAGACGGGCT	0	4	2	0
tsrna-07909	AAAGCAATACACTGAAAATGTTTAGACGGG	2	1	1	0
tsrna-07907	AAAGATTAAGAGAACCAACACCTCTTTACAGTGACCA	3	6	9	0
tsrna-07906	AAAGATTAAGAGAACCAACACCTCTTTACAGTGACC	0	2	1	0
tsrna-07905	AAAGATTAAGAGAACCAACACCTCTTTACAGTGAC	0	0	1	0
tsrna-07904	AAAGATTAAGAGAACCAACACCTCTTTACA	1	2	0	0
tsrna-07903	AAAGATTAAGAGAACCAACACCTCTTTAC	2	0	1	0
tsrna-07902	AAAGATTAAGAGAACCAACACCTCT	0	0	3	1
tsrna-07901	AAAGATTAAGAGAACCAACACCTC	0	0	0	0
tsrna-07900	AAAGATTAAGAGAACCAACACCT	0	0	1	0
tsrna-07899	AAAGATTAAGAGAACCAACACC	0	0	0	0
tsrna-07895	AAAGATTAAGAGAACCAA	0	0	0	0

tsrna-07894	AAAGATTAAGAGAACCA	0	0	0	0
tsrna-07892	AAAGACTTTTTCTCTGACCA	13	13	9	5
tsrna-07891	AAAGACTTTTTCTCTGACC	2	2	3	0
tsrna-07890	AAAGACTTTTTCTCTGAC	0	0	0	0
tsrna-07889	AAAGACTTTTTCTCTGA	0	0	1	0
tsrna-07887	AAAGACAGGGGTTAGGCCTCTTTTTACCACCA	10	4	9	4
tsrna-07886	AAAGACAGGGGTTAGGCCTCTTTTTACCACC	2	2	1	0
tsrna-07885	AAAGACAGGGGTTAGGCCTCTTTTTACCAC	1	4	0	0
tsrna-07884	AAAGACAGGGGTTAGGCCTCTTTTTACCA	0	1	0	0
tsrna-07883	AAAGACAGGGGTTAGGCCTCTTTTTACC	0	1	2	0
tsrna-07882	AAAGACAGGGGTTAGGCCTCT	0	0	0	0
tsrna-07881	AAAGACAGGGGTTAGGCCTC	0	0	0	0
tsrna-07880	AAAGACAGGGGTTAGGCCT	0	0	1	0
tsrna-07879	AAAGACAGGGGTTAGGCC	0	0	0	0
tsrna-07877	AAACTTTACAGTCAGAGGTTCA	0	0	0	0
tsrna-07876	AAACTTTACAGTCAGAGGTTC	0	0	0	0
tsrna-07874	AAACTTAAAACTTTACAGTCAGAGGTTC	0	2	0	0
tsrna-07871	AAACGAATGATTTCGACTCATTAAATTATG	4	2	6	1
tsrna-07870	AAACGAATGATTTCGACTCATT	0	1	1	0
tsrna-07869	AAACGAATGATTTCGACTCA	0	0	1	0
tsrna-07868	AAACGAATGATTTCGACTC	1	3	2	0
tsrna-07866	AAACGAATGATTTCGAC	0	0	0	0
tsrna-07865	AAACCTTTTTCCAAGGACACCA	4	2	4	0
tsrna-07864	AAACCTTTTTCCAAGGACACC	1	0	0	0
tsrna-07863	AAACCTGCCGGGGCTTCCA	2	1	2	0
tsrna-07859	AAACCGGGCGGAAACACCA	289	728	96	36
tsrna-07858	AAACCGGGCGGAAACACC	70	222	19	5
tsrna-07856	AAACCCCCTTATTTCTACC	1	1	0	0
tsrna-07854	AAACCATTTCATAACTTTGTCAAAGTTA	6	8	8	1
tsrna-07853	AAACCATTTCATAACTTTGTCA	4	1	3	1
tsrna-07852	AAACCATTTCATAACTTTGTC	1	0	3	0
tsrna-07850	AAACCATCCTCTGCTACCA	3	6	1	3
tsrna-07849	AAACCATCCTCTGCTACC	0	0	0	0
tsrna-07847	AAACCATCCTCTGCTA	0	0	0	0
tsrna-07846	AAACCAGGGGTCGCGAGTTC	0	0	0	0
tsrna-07845	AAACCAGGGGTCGCGAGTT	0	1	1	0
tsrna-07844	AAACCAGGGGTCGCGAGT	1	1	0	0
tsrna-07843	AAACCAGCTTTGGGGGGTTC	0	0	0	0
tsrna-07842	AAACATCAGATTGTGAATCTGACAACAGAGGCTTACGACCCC[illegible]	10	9	11	1
tsrna-07841	AAACATCAGATTGTGAATCTGACAACAGAGGCTTACGACCCC[illegible]	4	11	10	0
tsrna-07840	AAACATCAGATTGTGAATCTGACAACAGAGGCTTACGACCCC	5	6	7	1
tsrna-07839	AAACATCAGATTGTGAATCTGACAACAGAGGCTTACGACCC	5	11	6	0
tsrna-07838	AAACATCAGATTGTGAATCTGACAACAGAGGCTTACGACC	2	11	6	2
tsrna-07837	AAACATCAGATTGTGAATCTGACAACAGAGGCTTACGAC	7	9	7	3
tsrna-07836	AAACATCAGATTGTGAATCTGACAACAGAGGCTTACGA	4	10	5	1
tsrna-07835	AAACATCAGATTGTGAATCTGACAACAGAGGCTTACG	0	3	7	0
tsrna-07834	AAACATCAGATTGTGAATCTGACAACAGAGGCTTAC	2	4	6	0
tsrna-07833	AAACATCAGATTGTGAATCTGACAACAGAGGCTTA	4	2	8	0
tsrna-07832	AAACATCAGATTGTGAATCTGACAACAGAGGCTT	0	5	4	0
tsrna-07831	AAACATCAGATTGTGAATCTGACAACAGAGGCT	1	5	5	0
tsrna-07830	AAACATCAGATTGTGAATCTGACAACAGAGGC	1	2	5	0
tsrna-07829	AAACATCAGATTGTGAATCTGACAACAGAGG	0	1	3	0
tsrna-07828	AAACATCAGATTGTGAATCTGACAACAGAG	1	1	1	0
tsrna-07827	AAACATCAGATTGTGAATCTGACAACAGA	2	1	1	0
tsrna-07826	AAACATCAGATTGTGAATCTGACAACAG	1	3	1	0
tsrna-07825	AAACATCAGATTGTGAATCTGACAACA	1	1	1	1
tsrna-07824	AAACATCAGATTGTGAATCTGACAAC	0	3	0	0
tsrna-07823	AAACATCAGATTGTGAATCTGACAA	0	1	1	0
tsrna-07822	AAACATCAGATTGTGAATCTGACA	0	2	1	0
tsrna-07821	AAACATCAGATTGTGAATCTGAC	0	0	0	0
tsrna-07818	AAACATCAGATTGTGAATCT	0	0	0	0
tsrna-07814	AAACAGGAGATCCTGGGTTCGAATCCCAGCGGTGCCT	5	19	10	2
tsrna-07813	AAACAAAACGAATGATTTCGACTCA	0	3	0	0
tsrna-07810	AAAATTTTGGTGCAACTCCAAATA	0	1	0	0

tsrna-07809	AAAATTTTGGTGCAACTCCA	1	0	1	0
tsrna-07805	AAAATGTTTAGACGGGCTCACATCACCCCATAAACACCA	103	29	83	18
tsrna-07804	AAAATGTTTAGACGGGCTCACATCACCCCATAAA	52	24	49	6
tsrna-07803	AAAATGTTTAGACGGGCTCACATCACCCCATA	50	20	25	9
tsrna-07802	AAAATGTTTAGACGGGCTCACATCACCCC	34	19	19	5
tsrna-07801	AAAATGTTTAGACGGGCTCACATCACCC	34	15	28	6
tsrna-07800	AAAATGTTTAGACGGGCTCACATCACC	30	11	14	5
tsrna-07799	AAAATGTTTAGACGGGCTCACATCA	25	7	17	2
tsrna-07798	AAAATGTTTAGACGGGCTCACAT	25	18	14	0
tsrna-07797	AAAATGTTTAGACGGGCTCACA	23	13	10	1
tsrna-07796	AAAATGTTTAGACGGGCTCAC	15	6	5	0
tsrna-07795	AAAATGTTTAGACGGGCTCA	7	3	3	1
tsrna-07794	AAAATGTTTAGACGGGCTC	4	4	0	0
tsrna-07793	AAAATGTTTAGACGGGCT	1	1	0	0
tsrna-07791	AAAATGTTGGTTATACCCTTCCCGTACTACCA	7	21	8	2
tsrna-07790	AAAATGTTGGTTATACCCTTCCCGTACTAC	0	1	1	0
tsrna-07782	AAAATGGCTGAGTGAAGCATTGGACTGTA	0	1	0	0
tsrna-07781	AAAATGGCTGAGTGAAGCATTGGACTGT	0	1	0	0
tsrna-07780	AAAATGGCTGAGTGAAGCATTGGACTG	0	3	1	0
tsrna-07779	AAAATGGCTGAGTGAAGCATTGGACT	2	1	1	0
tsrna-07778	AAAATGGCTGAGTGAAGCATTGGAC	0	2	0	0
tsrna-07777	AAAATGGCTGAGTGAAGC	1	1	0	0
tsrna-07776	AAAATGGCTGAGTGAAG	0	0	0	0
tsrna-07775	AAAAGTCATGGAGGCCATGGGGTTGG	1	6	2	0
tsrna-07774	AAAAGTCATGGAGGCCATGGGGTT	2	8	2	0
tsrna-07773	AAAAGTCATGGAGGCCATGGGG	1	0	0	0
tsrna-07772	AAAAGTCATGGAGGCCATGGG	0	2	0	0
tsrna-07771	AAAAGTCATGGAGGCCATG	0	1	0	0
tsrna-07768	AAAACTTAAAACTTTACAGTCAGAGGTTCAATTCCTCTTC	15	19	9	4
tsrna-07765	AAAACGAATGATTTCGACTCATTAAATT	0	6	1	0
tsrna-07764	AAAACGAATGATTTCGACTCATT	2	5	1	0
tsrna-07763	AAAACGAATGATTTCGACTCA	0	1	0	0
tsrna-07762	AAAACGAATGATTTCGACTC	0	2	2	0
tsrna-07759	AAAACCTTTTTCCAAGGACACCA	1	1	0	0
tsrna-07758	AAAACCTTTTTCCAAGGACACC	1	0	0	0
tsrna-07756	AAAACCTTTTTCCAAGGACA	0	0	0	0
tsrna-07754	AAAACCATTTCATAACTTTGTC	1	1	3	0
tsrna-07753	AAAACATCAGATTGTGAATCTGACAACAGAGGCTTACGACCC(	1	9	9	0
tsrna-07752	AAAACATCAGATTGTGAATCTGACAACAGAGGCTTACGACCC(	6	9	6	0
tsrna-07751	AAAACATCAGATTGTGAATCTGACAACAGAGGCTTACGACCC	1	11	12	1
tsrna-07750	AAAACATCAGATTGTGAATCTGACAACAGAGGCTTACGACC	3	9	5	1
tsrna-07749	AAAACATCAGATTGTGAATCTGACAACAGAGGCTTACGA	2	2	11	0
tsrna-07748	AAAACATCAGATTGTGAATCTGACAACAGAGGCTTACG	2	10	7	1
tsrna-07747	AAAACATCAGATTGTGAATCTGACAACAGAGGCTTA	4	5	1	0
tsrna-07746	AAAACATCAGATTGTGAATCTGACAACAGAGGCTT	3	6	2	0
tsrna-07745	AAAACATCAGATTGTGAATCTGACAACAGAGGCT	4	6	6	0
tsrna-07744	AAAACATCAGATTGTGAATCTGACAACAGAGGC	0	5	0	0
tsrna-07743	AAAACATCAGATTGTGAATCTGACAACAGAGG	0	2	7	0
tsrna-07742	AAAACATCAGATTGTGAATCTGACAACAGA	1	6	3	0
tsrna-07741	AAAACATCAGATTGTGAATCTGACAACAG	1	3	1	0
tsrna-07740	AAAACATCAGATTGTGAATCTGACAACA	3	3	0	1
tsrna-07739	AAAACATCAGATTGTGAATCTGACAAC	1	4	0	0
tsrna-07738	AAAACATCAGATTGTGAATCTGACAA	2	1	1	0
tsrna-07737	AAAACATCAGATTGTGAATCTGACA	0	1	2	0
tsrna-07736	AAAACATCAGATTGTGAATCTGAC	0	1	0	0
tsrna-07734	AAAACATCAGATTGTGAATCTG	0	0	0	0
tsrna-07727	AAAAATTTTGGTGCAACTCCAAATAAAAGTACC	0	3	1	0
tsrna-07726	AAAAATTTTGGTGCAACTCCAAATAAAAGTAC	0	0	1	1
tsrna-07725	AAAAATTTTGGTGCAACTCCAAATAAAAGT	0	0	1	0
tsrna-07724	AAAAATTTTGGTGCAACTCCAAATA	0	0	2	0
tsrna-07723	AAAAATTTTGGTGCAACTCCAAAT	0	0	0	0
tsrna-07722	AAAAATTTTGGTGCAACTCCAAA	0	0	0	0
tsrna-07720	AAAAATTTTGGTGCAACTCC	0	2	1	0
tsrna-07719	AAAAATTTTGGTGCAACTC	0	1	0	0

tsrna-07717	AAAAAGTCATGGAGGCCATGGGGTTGG	1	22	2	1
tsrna-07716	AAAAAGTCATGGAGGCCATGGGGTT	1	12	1	1
tsrna-07715	AAAAAGTCATGGAGGCCATGGGG	0	1	0	0
tsrna-07714	AAAAAGTCATGGAGGCCATGGG	0	5	0	0
tsrna-07713	AAAAAGTCATGGAGGCCATG	0	2	0	0
tsrna-07712	AAAAAGTCATGGAGGCC	1	2	0	0
tsrna-07711	AAAAACCATTTCATAACTTTGTCA	6	1	3	1
tsrna-07710	ATTTGATTCCCGGTCAGGGAACCA	4	18	0	3
tsrna-07709	ATTTGATTCCCGGTCAGGGAACC	0	2	0	0
tsrna-07708	ATTTCGATGGCGTGGGT	2	1	1	0
tsrna-07707	ATTTCGACTCATTAAATTATGATAATCAT	2	0	1	1
tsrna-07706	ATTTCATAACTTTGTCAAAGTTAAATTATAGGCT	8	6	10	1
tsrna-07705	ATTTCATAACTTTGTCAAAGTTAAATTAT	4	7	0	0
tsrna-07704	ATTTCATAACTTTGTCAAAGTT	2	2	1	0
tsrna-07700	ATTTCAACTTAACTTGACCGCTCTGACCA	20	27	27	10
tsrna-07699	ATTTCAACTTAACTTGACCGCTCTGACC	2	4	5	2
tsrna-07698	ATTTCAACTTAACTTGACCGCTCTGAC	1	0	2	0
tsrna-07697	ATTTCAACTTAACTTGACCGCTCTGA	0	0	1	1
tsrna-07696	ATTTCAACTTAACTTGACCGCTCTG	0	0	1	0
tsrna-07695	ATTTCAACTTAACTTGACCGCTCT	0	0	2	0
tsrna-07688	ATTTAGGTTAAATACAGACCAAG	0	0	0	0
tsrna-07682	ATTGTGAATCTGACAACAGAGGCTTACGACCCCTTATTTACCC	22	18	26	1
tsrna-07681	ATTGTGAATCTGACAACAGAGGCTTACGACCCCTTATTTACCC	18	12	27	0
tsrna-07680	ATTGTGAATCTGACAACAGAGGCTTACGACCCCTTATTTACCC	21	16	19	0
tsrna-07679	ATTGTGAATCTGACAACAGAGGCTTACGACCCCTTATTTACC	16	4	24	1
tsrna-07678	ATTGTGAATCTGACAACAGAGGCTTACGACCCCTTA	18	7	12	0
tsrna-07677	ATTGTGAATCTGACAACAGAGGCTTACGACCCCT	5	2	4	0
tsrna-07676	ATTGTGAATCTGACAACAGAGGCTTACGACCCC	5	1	2	0
tsrna-07675	ATTGTGAATCTGACAACAGAGGCTTACGACCC	1	2	3	0
tsrna-07674	ATTGTGAATCTGACAACAGAGGCTTACGACC	0	1	0	0
tsrna-07673	ATTGTGAATCTGACAACAGAGGCTTACGAC	0	4	1	0
tsrna-07672	ATTGTGAATCTGACAACAGAGGCTTACGA	2	0	0	0
tsrna-07671	ATTGTGAATCTGACAACAGAGGCTTACG	0	2	0	0
tsrna-07670	ATTGTGAATCTGACAACAGAGGCTTAC	0	4	1	0
tsrna-07669	ATTGTGAATCTGACAACAGAGGCTTA	0	2	1	0
tsrna-07668	ATTGTGAATCTGACAACAGAGGCTT	0	1	1	0
tsrna-07667	ATTGTGAATCTGACAACAGAGGCT	0	2	1	0
tsrna-07666	ATTGTGAATCTGACAACAGAGGC	1	2	0	0
tsrna-07665	ATTGTGAATCTGACAACAGAGG	0	0	0	0
tsrna-07664	ATTGTGAATCTGACAACAGAG	1	0	0	0
tsrna-07663	ATTGTGAATCTGACAACAGA	0	2	0	0
tsrna-07662	ATTGTGAATCTGACAACAG	0	1	0	0
tsrna-07659	ATTGTGAATCTGACAA	0	0	0	0
tsrna-07658	ATTGGTGGTTCAGTGGTAGAATTCTCGCCTGCCATG	68	73	67	10
tsrna-07657	ATTGGTGGTTCAGTGGTAGAATTCTCGCCTGCCACG	105	108	70	19
tsrna-07656	ATTGGTGGTTCAGTGGTAGAATTCTCGCCTGCCA	47	63	58	16
tsrna-07655	ATTGGTGGTTCAGTGGTAGAATTCTCGCCTGCC	52	43	44	11
tsrna-07654	ATTGGTGGTTCAGTGGTAGAATTCTCGCCTGC	21	19	18	2
tsrna-07653	ATTGGTGGTTCAGTGGTAGAATTCTCGCCTG	18	17	21	1
tsrna-07652	ATTGGTGGTTCAGTGGTAGAATTCTCGCCT	23	16	18	5
tsrna-07651	ATTGGTGGTTCAGTGGTAGAATTCTCGCC	15	16	15	3
tsrna-07650	ATTGGTGGTTCAGTGGTAGAATTCTCGC	5	6	3	3
tsrna-07649	ATTGGTGGTTCAGTGGTAGAATTCTCG	3	4	4	3
tsrna-07648	ATTGGTGGTTCAGTGGTAGAATTCTC	5	5	5	0
tsrna-07647	ATTGGTGGTTCAGTGGTAGAATTCT	7	5	4	2
tsrna-07646	ATTGGTGGTTCAGTGGTAGAATTC	2	5	3	2
tsrna-07645	ATTGGTGGTTCAGTGGTAGAATT	4	8	4	2
tsrna-07644	ATTGGTGGTTCAGTGGTAGAAT	4	2	0	2
tsrna-07643	ATTGGTGGTTCAGTGGTAGAA	1	1	5	0
tsrna-07642	ATTGGTGGTTCAGTGGTAGA	0	2	1	0
tsrna-07641	ATTGGTGGTTCAGTGGTAG	0	0	0	1
tsrna-07640	ATTGGTGGTTCAGTGGTA	0	1	0	0
tsrna-07638	ATTGGTGGTTCAGTGG	0	0	0	0
tsrna-07636	ATTGGTCGTGGTTGTAGTCCGTGCGAGAATACCA	91	141	112	43

tsrna-07635	ATTGGTCGTGGTTGTAGTCCGTGCGAGAATACC	7	23	8	12
tsrna-07634	ATTGGTCGTGGTTGTAGTCCGTGCGAGAATAC	8	11	7	9
tsrna-07633	ATTGGTCGTGGTTGTAGTCCGTGCGAGAATA	5	16	8	9
tsrna-07632	ATTGGTCGTGGTTGTAGTCCGTGCGAGAAT	3	10	4	8
tsrna-07631	ATTGGTCGTGGTTGTAGTCCGTGCGAGAA	9	4	5	7
tsrna-07630	ATTGGTCGTGGTTGTAGTCCGTGCGAGA	5	6	6	8
tsrna-07629	ATTGGTCGTGGTTGTAGTCCGTGCGAG	3	3	9	2
tsrna-07628	ATTGGTCGTGGTTGTAGTCCGTGCGA	2	7	2	1
tsrna-07627	ATTGGTCGTGGTTGTAGTCCGTGCG	2	1	4	1
tsrna-07626	ATTGGTCGTGGTTGTAGTCCGTGC	1	5	0	1
tsrna-07625	ATTGGTCGTGGTTGTAGTCCGTG	1	1	0	0
tsrna-07624	ATTGGTCGTGGTTGTAGTCCGT	1	2	1	0
tsrna-07623	ATTGGTCGTGGTTGTAGTCCG	0	1	0	0
tsrna-07622	ATTGGTCGTGGTTGTAGTCC	2	1	0	0
tsrna-07621	ATTGGTCGTGGTTGTAGTC	0	0	0	0
tsrna-07620	ATTGGTCGTGGTTGTAGT	0	1	0	0
tsrna-07613	ATTGGACTGTAAATCTAAAGACAGGGGTTAGGCCTC	2	2	0	0
tsrna-07602	ATTGCAGGTTCGAGTCCTGCCGCGGTCGCCA	2	7	1	0
tsrna-07598	ATTGCAAATTCGAAGAAGCAGCTTCAAACCTGCCGGGGCTT	1	1	1	0
tsrna-07597	ATTGCAAATTCGAAGAAGCAGCTTCAAACCTGCCGGGGCT	0	3	1	0
tsrna-07596	ATTGAGGGTTCGAGTCCCTTCGTGGTCGCCA	1	4	0	0
tsrna-07594	ATTGAAGCCAGTTGATTAGGGTGCTTAGC	0	1	1	0
tsrna-07593	ATTGAAGCCAGTTGATTAGGGTGCTTAG	0	0	0	0
tsrna-07592	ATTGAAGCCAGTTGATTAGGG	1	0	0	0
tsrna-07590	ATTCTTGCGACCCGGGTTCGATTCCCGGGCGGCGCACCA	15	85	10	0
tsrna-07589	ATTCTTGCGACCCGGGTTCGATTCCCGGGCGGCGCACC	4	15	1	2
tsrna-07588	ATTCTTGCGACCCGGGTTCGATTCCCGGGCGGCGCAC	1	7	3	2
tsrna-07587	ATTCTTGCGACCCGGGTTCGATTCCCGGGCGGCGCA	2	10	0	0
tsrna-07586	ATTCTTGCGACCCGGGTTCGATTCCCGGGCGGCGC	2	7	0	0
tsrna-07585	ATTCTTGCGACCCGGGTTCGATTCCCGGGCGGCG	2	5	0	0
tsrna-07584	ATTCTTGCGACCCGGGTTCGATTCCCGGGCGGC	1	3	1	0
tsrna-07583	ATTCTTGCGACCCGGGTTCGATTCCCGGGCGG	1	4	0	0
tsrna-07582	ATTCTTGCGACCCGGGTTCGATTCCCGGGC	0	12	1	0
tsrna-07581	ATTCTTGCGACCCGGGTTCGATT	0	4	0	0
tsrna-07580	ATTCTTGCGACCCGGGTTCGAT	0	3	0	0
tsrna-07579	ATTCTTGCGACCCGGGTTCGA	1	0	1	0
tsrna-07578	ATTCTTGCGACCCGGGTTCG	0	1	0	0
tsrna-07577	ATTCTTGCGACCCGGGTTC	0	3	0	0
tsrna-07576	ATTCTTGCGACCCGGGTT	0	1	0	0
tsrna-07566	ATTCTCGCTGGGGCCTCCA	29	42	22	18
tsrna-07565	ATTCTCGCCTGCCACGCGGGAGGCCCGGGT	10	44	8	2
tsrna-07564	ATTCTCGCCTGCCACGCGGGAGGCCCGG	2	7	0	0
tsrna-07563	ATTCTCGCCTGCCACGCGGGAGGCCCG	1	6	0	1
tsrna-07562	ATTCTCGCCTGCCACGCGG	2	6	1	0
tsrna-07561	ATTCTCGCCTGCCACGC	1	6	0	0
tsrna-07560	ATTCTCGCCTGCCACG	0	6	1	0
tsrna-07556	ATTCTCATAGTCCTAG	0	0	0	0
tsrna-07555	ATTCTCAGGGATGGGTTCGATTCTCATAGTCCT	10	23	21	14
tsrna-07554	ATTCTAGGTTCGACTCCTGGCTGGCTCGCC	3	5	0	0
tsrna-07553	ATTCTAGGTTCGACTCCTGGCTGGCTCGC	0	0	0	0
tsrna-07552	ATTCTAGGTTCGACTCCTGGCTGGCTCG	0	2	0	0
tsrna-07549	ATTCGGTGCTGAAAGA	1	12	0	0
tsrna-07548	ATTCGGCGCTTTCACCGCCGCGGCCC	0	4	2	0
tsrna-07547	ATTCGGCGCTCTCACCGCCGCGGCCCGGGTTCGATT	0	1	0	0
tsrna-07546	ATTCGGCGCTCTCACCGCCGCGGCCCGGGTTC	1	1	0	0
tsrna-07545	ATTCGGCGCTCTCACCGCCGCGGCCCGGGTT	0	4	0	1
tsrna-07544	ATTCGGCGCTCTCACCGCCGCGGCCCGGGT	0	0	0	0
tsrna-07543	ATTCGGCGCTCTCACCGCCGCGGCCCGGG	1	1	0	0
tsrna-07542	ATTCGGCGCTCTCACCGCCGCGGCCCGG	0	1	0	0
tsrna-07541	ATTCGGCGCTCTCACCGCCGCGGCCCG	1	1	0	0
tsrna-07540	ATTCGGCGCTCTCACCGCCGCGGCCC	1	1	0	0
tsrna-07538	ATTCGGCGCTCTCACCGC	1	0	0	0
tsrna-07537	ATTCGGCGCTCTCACC	0	1	0	0
tsrna-07536	ATTCGAAGAAGCAGCTTCAAACCTGCCGGGGCTTCC	0	7	1	0

tsrna-07535	ATTCGAAGAAGCAGCTTCAAACCTGCCGGGGCTT	0	1	1	0
tsrna-07534	ATTCGAAGAAGCAGCTTCAAACCTGCCGGGGCT	0	1	0	0
tsrna-07531	ATTCCTTCCTTTTTTGCCA	0	0	0	0
tsrna-07528	ATTCCTTCCTTTTTTG	0	0	0	0
tsrna-07527	ATTCCTGGTTTTCACCCAGGTGGCCCG	2	2	0	0
tsrna-07526	ATTCCTGGTTTTCACCCAGGCGGCCCGGGTTCGACTCCCGGT	2	7	1	1
tsrna-07524	ATTCCTCTTCTTAACACCA	0	5	2	0
tsrna-07523	ATTCCGGCTCGAAGGACCA	52	124	26	16
tsrna-07522	ATTCCGGCTCGAAGGACC	1	4	0	0
tsrna-07520	ATTCCGGCTCGAAGGA	0	1	0	0
tsrna-07516	ATTCCCGGTCAGGGAACCA	2	6	2	1
tsrna-07515	ATTCCCGGTCAGGGAACC	0	1	0	0
tsrna-07513	ATTCCCGGGCGGCGCACCA	9	34	5	1
tsrna-07512	ATTCCCGGCCCATGCACCA	3	14	2	5
tsrna-07511	ATTCCCGGCCCATGCACC	0	3	0	0
tsrna-07510	ATTCCCGGCCAATGCACCA	0	11	2	0
tsrna-07509	ATTCCCGGCCAATGCACC	0	1	1	0
tsrna-07507	ATTCCCGGCCAACGCACCA	2	3	1	0
tsrna-07506	ATTCCCGGCCAACGCACC	2	0	0	0
tsrna-07505	ATTCCCCGACGGGGAGCCA	0	2	0	0
tsrna-07503	ATTCCCATTCTTGCGACCCGGG	3	0	1	0
tsrna-07502	ATTCCCATTCTTGCGACCCGG	2	1	0	0
tsrna-07501	ATTCCCATTCTTGCGACCCG	0	0	0	0
tsrna-07500	ATTCCCATTCTTGCGACC	0	1	0	0
tsrna-07499	ATTCCAGGTTCGACTCCTGGCTGGCTCGCC	2	3	1	0
tsrna-07498	ATTCCAGGTTCGACTCCTGGCTGGCTCGC	0	0	0	1
tsrna-07497	ATTCCAGGTTCGACTCCTGGCTGGCTCG	0	0	0	0
tsrna-07495	ATTCCAGGTTCGACTCCTGGC	0	1	0	0
tsrna-07487	ATTCAAAGGTTGTGGGTTCGAGTCCCACCAGAGTCGCCA	1	16	0	1
tsrna-07486	ATTCAAAGGTTGTGGGTTCGAGTCCCACCAGAGTCG	0	2	0	0
tsrna-07485	ATTCAAAGGTTGTGGGTTCGAATCCCACCAGAGTCGCCA	4	12	1	3
tsrna-07484	ATTCAAAGGTTGTGGGTTCGAATCCCACCAGAGTCG	0	6	0	0
tsrna-07483	ATTCAAAGGTTGTGGGTT	1	1	0	0
tsrna-07482	ATTCAAAGGTTGTGGGT	0	1	0	0
tsrna-07480	ATTCAAAGGTTCCGGGTTCGAGTCCCGGCGGAGTCGCCA	2	5	0	1
tsrna-07479	ATTCAAAGGTTCCGGGTTCGAGTCCCGGCGGAGTCGCC	1	2	0	0
tsrna-07478	ATTCAAAGGTTCCGGGTTCGAGTCCCGGCGGAGTCG	0	1	2	0
tsrna-07477	ATTCAAAGGTTCCGGGTTCGAGTCCCGGCG	0	6	0	0
tsrna-07476	ATTCAAAGGTTCCGGGTTCGAG	0	1	0	0
tsrna-07474	ATTCAAAGGTTCCGGGT	0	1	0	0
tsrna-07464	ATTAGGGTGCTTAGCTGTTAACTAAGTGTTTGTGGG	2	1	5	1
tsrna-07463	ATTAGGGTGCTTAGCTGTTAACTAAGTGTT	0	2	2	0
tsrna-07462	ATTAGGGTGCTTAGCTGTTAACTA	1	5	3	0
tsrna-07461	ATTAGGGTGCTTAGCTGTTAACT	2	2	3	0
tsrna-07460	ATTAGGGTGCTTAGCTGTTAAC	1	2	1	0
tsrna-07459	ATTAGGGTGCTTAGCTGTTAA	0	1	0	0
tsrna-07458	ATTAGGGTGCTTAGCTGTTA	1	0	0	0
tsrna-07457	ATTAGGGTGCTTAGCTGTT	1	0	0	0
tsrna-07451	ATTAGAATCTTAGCTTTGGGTGCTAATGGTGGAGTTAAAGACT	2	3	2	1
tsrna-07450	ATTAGAATCTTAGCTTTGGGTGCTAATGGTGGAGTT	2	3	1	0
tsrna-07449	ATTAGAATCTTAGCTTTGGGTGCTAATGGT	1	1	2	0
tsrna-07448	ATTAGAATCTTAGCTTTGGGTGCT	0	0	0	0
tsrna-07447	ATTAGAATCTTAGCTTTGGGTGC	1	0	2	0
tsrna-07446	ATTAGAATCTTAGCTTTGGGTG	0	1	1	0
tsrna-07445	ATTAGAATCTTAGCTTTGGGT	1	0	0	0
tsrna-07444	ATTAGAATCTTAGCTTTGGG	0	1	0	0
tsrna-07443	ATTAGAATCTTAGCTTTGG	0	0	0	1
tsrna-07442	ATTAGAATCTTAGCTTTG	1	0	0	0
tsrna-07441	ATTAAGAGAACCAACACCTCTTTACAGTGACCA	2	1	3	0
tsrna-07440	ATTAAGAGAACCAACACCTCTTTACAGTGACC	0	1	0	0
tsrna-07439	ATTAAGAGAACCAACACCTCTTTACAGTGAC	0	0	1	0
tsrna-07438	ATTAAGAGAACCAACACCTCTTTACAGTGA	1	0	0	0
tsrna-07433	ATTAAGAGAACCAACACC	0	0	0	0
tsrna-07424	ATTAAATTATGATAATCATATTTACCAACC	0	0	0	0

tsrna-07423	ATTAAAGTGGCTGATTTGCGTTCA	0	1	0	1
tsrna-07422	ATTAAAGTGGCTGATTTGCGTTC	0	2	0	0
tsrna-07421	ATTAAAGTGGCTGATTTGCGTT	0	0	0	0
tsrna-07420	ATTAAAGTGGCTGATTTGC	0	0	0	0
tsrna-07418	ATGTTTAGACGGGCTCACATCACCCCATAAACACCA	96	21	64	15
tsrna-07417	ATGTTTAGACGGGCTCACATCACCCCATAAACACC	36	13	24	8
tsrna-07416	ATGTTTAGACGGGCTCACATCACCCCATAAACA	33	10	28	7
tsrna-07415	ATGTTTAGACGGGCTCACATCACCCCATAAA	27	8	22	6
tsrna-07414	ATGTTTAGACGGGCTCACATCACCCCATAA	26	18	21	5
tsrna-07413	ATGTTTAGACGGGCTCACATCACCCCATA	29	10	26	5
tsrna-07412	ATGTTTAGACGGGCTCACATCACCCCAT	25	6	33	4
tsrna-07411	ATGTTTAGACGGGCTCACATCACCCCA	23	14	31	9
tsrna-07410	ATGTTTAGACGGGCTCACATCACCCC	16	9	15	3
tsrna-07409	ATGTTTAGACGGGCTCACATCACCC	26	8	21	4
tsrna-07408	ATGTTTAGACGGGCTCACATCACC	18	7	15	1
tsrna-07407	ATGTTTAGACGGGCTCACATCAC	20	7	15	2
tsrna-07406	ATGTTTAGACGGGCTCACATCA	19	9	16	0
tsrna-07405	ATGTTTAGACGGGCTCACATC	16	10	7	0
tsrna-07404	ATGTTTAGACGGGCTCACAT	15	15	10	1
tsrna-07403	ATGTTTAGACGGGCTCACA	8	8	8	0
tsrna-07402	ATGTTTAGACGGGCTCAC	13	6	3	1
tsrna-07401	ATGTTTAGACGGGCTCA	1	1	1	3
tsrna-07400	ATGTTTAGACGGGCTC	1	0	0	0
tsrna-07399	ATGTTGGTTATACCCTTCCCGTACTACCA	2	9	3	3
tsrna-07398	ATGTTGGTTATACCCTTCCCGTACTACC	1	2	0	0
tsrna-07395	ATGTTGGTTATACCCTTCCCGTACT	0	1	0	0
tsrna-07392	ATGTTGGTTATACCCTTCCCGT	0	0	0	0
tsrna-07388	ATGTGTGAGGTCCCGGGT	1	3	0	0
tsrna-07387	ATGTCTAACAACATGGCTTTCTCACCA	0	2	3	0
tsrna-07386	ATGTCTAACAACATGGCTTTCTCACC	0	2	2	0
tsrna-07385	ATGTCTAACAACATGGCTTTCTCAC	0	0	0	0
tsrna-07383	ATGTCTAACAACATGGCTTTCTC	0	0	1	0
tsrna-07377	ATGTCCGCGTGGGTTCGAACCCCACTCCTGGTACCA	2	6	1	0
tsrna-07376	ATGTCCGCGTGGGTTCGAACCCCACTCCTGGTACC	5	6	1	0
tsrna-07375	ATGTCCGCGTGGGTTCGAACCCCACTCCTGGTA	3	1	0	0
tsrna-07374	ATGTCCGCGTGGGTTCGAACCCCACTCCTGGT	2	5	1	0
tsrna-07373	ATGTCCGCGTGGGTTCGAACCCCACTCCTGG	0	2	1	0
tsrna-07372	ATGTCCGCGTGGGTTCGAACCCCACTCCTG	1	2	0	0
tsrna-07371	ATGTATGAGGTCCCGGGT	0	3	1	0
tsrna-07365	ATGTAGCTCAGTGGTAGAGCGCATGCTT	0	0	0	0
tsrna-07364	ATGTAGCTCAGTGGTAGAGCGCA	0	0	0	0
tsrna-07362	ATGTAGCTCAGTGGTAGAG	0	0	1	0
tsrna-07359	ATGGTTTTTCATATCATTGGTCGTGGTTGTAGTCCGTGCGAGA	16	25	16	18
tsrna-07358	ATGGTTTTTCATATCATTGGTCGTGGTTGTAGTCCGTGCGAGA	12	14	14	10
tsrna-07357	ATGGTTTTTCATATCATTGGTCGTGGTTGTAGTCCGTGCGAGA	12	23	13	12
tsrna-07356	ATGGTTTTTCATATCATTGGTCGTGGTTGTAGTCCGTGCGAGA	4	22	18	12
tsrna-07355	ATGGTTTTTCATATCATTGGTCGTGGTTGTAGTCCGTGCGAG	10	9	14	4
tsrna-07354	ATGGTTTTTCATATCATTGGTCGTGGTTGTAGTCCGTGCGA	5	12	7	7
tsrna-07353	ATGGTTTTTCATATCATTGGTCGTGGTTGTAGTCCGTGCG	10	10	12	4
tsrna-07352	ATGGTTTTTCATATCATTGGTCGTGGTTGTAGTCCGTGC	5	6	6	0
tsrna-07351	ATGGTTTTTCATATCATTGGTCGTGGTTGTAGTCCG	3	3	3	2
tsrna-07350	ATGGTTTTTCATATCATTGGTCGTGGTTGTAGTCC	5	0	2	0
tsrna-07349	ATGGTTTTTCATATCATTGGTCGTGGTTGTAGTC	6	2	5	0
tsrna-07348	ATGGTTTTTCATATCATTGGTCGTGGTTGTAGT	2	2	0	1
tsrna-07347	ATGGTTTTTCATATCATTGGTCGTGGTTGTA	0	1	1	1
tsrna-07346	ATGGTTTTTCATATCATTGGTCGTGGTTGT	1	1	1	0
tsrna-07345	ATGGTTTTTCATATCATTGGTCGTGGTTG	0	0	0	0
tsrna-07344	ATGGTTTTTCATATCATTGGTCGTGG	1	0	0	1
tsrna-07337	ATGGTTAGCACTCTGGGCT	0	0	0	0
tsrna-07335	ATGGTTAGCACTCTGGG	0	1	0	0
tsrna-07334	ATGGTTAGCACTCTGGACTTTGAATCCAGC	10	9	3	4
tsrna-07333	ATGGTTAGCACTCTGGACTTTGAATCCA	2	4	2	1
tsrna-07332	ATGGTTAGCACTCTGGACTTTGAATCC	2	5	3	0
tsrna-07331	ATGGTTAGCACTCTGGACTTTGAATC	4	6	1	0

tsrna-07329	ATGGTTAGCACTCTGGACTTTGAA	0	0	0	1
tsrna-07327	ATGGTTAGCACTCTGGACTTTG	2	0	0	0
tsrna-07325	ATGGTTAGCACTCTGGACTT	0	0	0	0
tsrna-07324	ATGGTTAGCACTCTGGACTCTGAATCCAGCGATCCGAGTTCA	7	4	1	0
tsrna-07323	ATGGTTAGCACTCTGGACTCTGAATCCAGCGATCCGAGTTC	6	5	3	1
tsrna-07322	ATGGTTAGCACTCTGGACTCTGAATCCAGCGATCCGAGTT	3	9	3	2
tsrna-07321	ATGGTTAGCACTCTGGACTCTGAATCCAGCGATCCGAGT	6	1	0	4
tsrna-07320	ATGGTTAGCACTCTGGACTCTGAATCCAGCGATCCGAG	1	4	1	0
tsrna-07319	ATGGTTAGCACTCTGGACTCTGAATCCAGCGATCCG	2	0	2	2
tsrna-07318	ATGGTTAGCACTCTGGACTCTGAATCCAGCG	6	6	1	1
tsrna-07317	ATGGTTAGCACTCTGGACTCTGAATCCAGC	2	5	1	2
tsrna-07316	ATGGTTAGCACTCTGGACTCTGAATCCAG	2	5	3	2
tsrna-07315	ATGGTTAGCACTCTGGACTCTGAATCCA	2	4	1	1
tsrna-07314	ATGGTTAGCACTCTGGACTCTGAATCC	0	1	2	0
tsrna-07313	ATGGTTAGCACTCTGGACTCTGAATC	4	0	0	0
tsrna-07312	ATGGTTAGCACTCTGGACTCTGAAT	0	0	0	0
tsrna-07310	ATGGTTAGCACTCTGGACTCTGA	0	1	1	0
tsrna-07309	ATGGTTAGCACTCTGGACTCTG	0	0	1	0
tsrna-07308	ATGGTTAGCACTCTGGACTCT	0	0	1	1
tsrna-07307	ATGGTTAGCACTCTGGACTC	0	0	0	0
tsrna-07306	ATGGTTAGCACTCTGGACT	0	1	0	0
tsrna-07305	ATGGTTAGCACTCTGGAC	0	0	0	0
tsrna-07301	ATGGTGTAATGGTTAGCACTCTGGACTTTG	3	0	0	0
tsrna-07300	ATGGTGTAATGGTTAGCACTCTGGACTTT	0	0	0	0
tsrna-07299	ATGGTGTAATGGTTAGCACTCTGGACTT	3	0	1	1
tsrna-07298	ATGGTGTAATGGTTAGCACTCTGGACTCTGAATCCA	6	6	6	3
tsrna-07297	ATGGTGTAATGGTTAGCACTCTGGACTCTG	0	0	0	2
tsrna-07296	ATGGTGTAATGGTTAGCACTCTGGACTCT	0	0	0	1
tsrna-07295	ATGGTGTAATGGTTAGCACTCTGGACTC	2	5	0	0
tsrna-07294	ATGGTGTAATGGTTAGCACTCTGGACT	0	5	2	1
tsrna-07293	ATGGTGTAATGGTTAGCACTCTGGAC	2	2	0	1
tsrna-07292	ATGGTGTAATGGTTAGCACTCTGGA	0	1	0	0
tsrna-07290	ATGGTGTAATGGTTAGCACTCTG	0	0	0	0
tsrna-07289	ATGGTGTAATGGTTAGCACTCT	1	0	0	0
tsrna-07288	ATGGTGTAATGGTGAGCACTCTGGACTC	1	0	0	0
tsrna-07287	ATGGTGTAATGGTGAGCACTCTGGACT	0	2	0	0
tsrna-07286	ATGGTGTAATGGTGAGCACTCTGGAC	0	1	0	1
tsrna-07282	ATGGTGTAATGGTCAGCACTCTGGACT	1	0	0	0
tsrna-07278	ATGGTGGTTCAGTGGTAGAATTCTCGCCT	29	15	21	2
tsrna-07277	ATGGTGGTTCAGTGGTAGAATTCTCGCC	25	14	11	2
tsrna-07276	ATGGTGGTTCAGTGGTAGAATTCTCGC	2	4	4	1
tsrna-07275	ATGGTGGTTCAGTGGTAGAATTCTCG	1	1	3	0
tsrna-07274	ATGGTGGTTCAGTGGTAGAATTCTC	2	1	1	0
tsrna-07273	ATGGTGGTTCAGTGGTAGAATTCT	2	5	1	1
tsrna-07272	ATGGTGGTTCAGTGGTAGAATTC	2	3	1	0
tsrna-07271	ATGGTGGTTCAGTGGTAGAA	0	0	0	1
tsrna-07270	ATGGTGGAGTTAAAGACTTTTTCTCTGACCA	30	46	12	15
tsrna-07269	ATGGTGGAGTTAAAGACTTTTTCTCTGACC	3	18	1	1
tsrna-07268	ATGGTGGAGTTAAAGACTTTTTCTCTGAC	1	7	1	0
tsrna-07267	ATGGTGGAGTTAAAGACTTTTTCTCTGA	0	5	3	0
tsrna-07266	ATGGTGGAGTTAAAGACTTTTTCTCTG	1	4	0	0
tsrna-07265	ATGGTGGAGTTAAAGACTTTTTCTC	0	4	0	0
tsrna-07264	ATGGTGGAGTTAAAGACTTTTTCT	1	2	2	0
tsrna-07263	ATGGTGGAGTTAAAGACTTTTTC	0	2	0	0
tsrna-07262	ATGGTGGAGTTAAAGACTTTTT	0	4	2	0
tsrna-07261	ATGGTGGAGTTAAAGACTTTT	1	1	3	0
tsrna-07258	ATGGTGGAGTTAAAGACT	0	0	0	0
tsrna-07255	ATGGTGAGCACTTTGGACTCTGA	0	1	0	0
tsrna-07252	ATGGTGAGCACTCTGGACTCTGAATCCAGC	0	4	3	1
tsrna-07251	ATGGTGAGCACTCTGGACTCTGAATCCA	1	1	1	0
tsrna-07250	ATGGTGAGCACTCTGGACTCTGAATCC	1	5	1	1
tsrna-07249	ATGGTGAGCACTCTGGACTCTGAATC	1	3	0	0
tsrna-07246	ATGGTGAGCACTCTGGACTCTGA	0	1	0	0
tsrna-07245	ATGGTGAGCACTCTGGACTCTG	0	1	0	0

tsrna-07243	ATGGTGAGCACTCTGGACTC	0	0	0	0
tsrna-07242	ATGGTGAGCACTCTGGACT	0	0	0	0
tsrna-07238	ATGGTGAGCACCCTGGACT	0	0	0	0
tsrna-07237	ATGGTCTAGCGGTTAGGATTCCTGGTTTTC	6	8	6	6
tsrna-07236	ATGGTCTAGCGGTTAGGATTCCTGGTTTT	6	5	4	2
tsrna-07235	ATGGTCTAGCGGTTAGGATTCCTGGTTT	6	9	0	2
tsrna-07234	ATGGTCTAGCGGTTAGGATTCCTGGTT	1	8	3	1
tsrna-07233	ATGGTCTAGCGGTTAGGATTCCTGGT	5	6	5	0
tsrna-07232	ATGGTCTAGCGGTTAGGATTCCTGG	3	6	1	1
tsrna-07231	ATGGTCTAGCGGTTAGGATTCCTG	2	1	0	0
tsrna-07230	ATGGTCTAGCGGTTAGGATTCCT	0	1	1	0
tsrna-07224	ATGGTCAGCACTCTGGACTCTGAATCCAGC	1	3	3	0
tsrna-07223	ATGGTCAGCACTCTGGACTCTGAAT	0	0	0	0
tsrna-07215	ATGGTAGCGCGTCTGACTCC	0	1	0	0
tsrna-07214	ATGGTAGCGCGTCTGACT	0	0	0	1
tsrna-07211	ATGGTAGAGCGCTCGCTT	1	2	0	0
tsrna-07210	ATGGTAGAGCGCTCGC	1	2	0	0
tsrna-07209	ATGGTAGAATTCTCGCCT	1	3	1	1
tsrna-07208	ATGGTAAGCACTCTGGACTCTGAATCCA	0	4	2	1
tsrna-07207	ATGGTAAGCACTCTGGACTCTGAATCC	3	1	2	0
tsrna-07200	ATGGTAAGCACTCTGGACTC	0	0	0	0
tsrna-07195	ATGGGTTCGATTCTCATAGTCCTAGC	0	4	0	1
tsrna-07194	ATGGGTTCGATTCTCATAGTCCTAG	0	5	1	1
tsrna-07193	ATGGGTGGTTCAGTGGTAGAATTCTCGCCTGCCA	37	54	34	9
tsrna-07192	ATGGGTGGTTCAGTGGTAGAATTCTCGCCTGCC	39	46	28	6
tsrna-07191	ATGGGTGGTTCAGTGGTAGAATTCTCGCCTGC	25	31	26	3
tsrna-07190	ATGGGTGGTTCAGTGGTAGAATTCTCGCCTG	20	17	16	3
tsrna-07189	ATGGGTGGTTCAGTGGTAGAATTCTCGCCT	19	22	25	6
tsrna-07188	ATGGGTGGTTCAGTGGTAGAATTCTCGCC	12	19	20	2
tsrna-07187	ATGGGTGGTTCAGTGGTAGAATTCTCGC	5	2	3	1
tsrna-07186	ATGGGTGGTTCAGTGGTAGAATTCTCG	3	3	3	1
tsrna-07185	ATGGGTGGTTCAGTGGTAGAATTCTC	2	3	2	1
tsrna-07184	ATGGGTGGTTCAGTGGTAGAATTCT	4	3	1	1
tsrna-07183	ATGGGTGGTTCAGTGGTAGAATTC	2	3	0	1
tsrna-07182	ATGGGTGGTTCAGTGGTAGAATT	3	2	0	0
tsrna-07181	ATGGGTGGTTCAGTGGTAGAAT	2	1	3	0
tsrna-07180	ATGGGTGGTTCAGTGGTAGAA	3	1	0	0
tsrna-07179	ATGGGTGGTTCAGTGGTAGA	0	1	0	1
tsrna-07174	ATGGGGTGTGATAGGTGGCACGGAG	0	0	0	0
tsrna-07167	ATGGGACTCTTAATCCCAGGGTCGTGGGTT	1	2	1	0
tsrna-07166	ATGGGACTCTTAATCC	0	1	1	0
tsrna-07165	ATGGCTGAGTGAAGCATTGGACTGTAAAT	0	2	2	0
tsrna-07164	ATGGCTGAGTGAAGCATTGGACTGTAAA	4	0	1	0
tsrna-07163	ATGGCTGAGTGAAGCATTGGACTGTAA	1	3	2	0
tsrna-07162	ATGGCTGAGTGAAGCATTGGACTGTA	0	2	1	0
tsrna-07161	ATGGCTGAGTGAAGCATTGGACTGT	0	0	1	0
tsrna-07160	ATGGCTGAGTGAAGCATTGGACTG	0	0	1	0
tsrna-07159	ATGGCTGAGTGAAGCATTGGACT	0	2	0	0
tsrna-07158	ATGGCTGAGTGAAGCATTGGAC	1	1	0	0
tsrna-07157	ATGGCTGAGTGAAGCATTGGA	0	3	2	1
tsrna-07156	ATGGCTGAGTGAAGCATTGG	0	1	1	0
tsrna-07154	ATGGCTGAGTGAAGCATT	0	1	0	0
tsrna-07149	ATGGCCGAGCGGTCTAAGGCGCTGCGTTC	0	0	1	0
tsrna-07136	ATGGATCGAAACCATCCTCTGCTACCA	13	24	12	8
tsrna-07135	ATGGATCGAAACCATCCTCTGCTACC	2	2	2	1
tsrna-07134	ATGGATCGAAACCATCCTCTGCTA	0	0	0	0
tsrna-07133	ATGGATCGAAACCATCCTCTGC	0	0	0	0
tsrna-07131	ATGGATAGCGCATTGGACTTCTAATTCAAAGGTTCCGGGT	35	35	7	9
tsrna-07130	ATGGATAGCGCATTGGACTTC	2	10	2	2
tsrna-07129	ATGGATAGCGCATTGGAC	3	7	1	1
tsrna-07128	ATGGATAGCGCATTGGA	3	7	2	0
tsrna-07127	ATGGATAGCGCATTGG	5	4	0	0
tsrna-07126	ATGGATAAGGCGTCTGATTCC	0	8	0	1
tsrna-07125	ATGGATAAGGCGTCTGATTC	1	2	0	1

tsrna-07124	ATGGATAAGGCGTCTGATT	0	1	0	0
tsrna-07123	ATGGATAAGGCGTCTGACTTCGGATCAGA	0	0	1	1
tsrna-07122	ATGGATAAGGCGTCTGACTTCGGATC	2	0	0	1
tsrna-07121	ATGGATAAGGCGTCTGACTTCGGAT	0	0	1	0
tsrna-07120	ATGGATAAGGCGTCTGACTTCGGA	1	2	1	0
tsrna-07119	ATGGATAAGGCGTCTGACTTCGG	1	0	0	0
tsrna-07118	ATGGATAAGGCGTCTGACTTCG	0	1	0	0
tsrna-07117	ATGGATAAGGCGTCTGACTTC	1	1	1	0
tsrna-07115	ATGGATAAGGCGTCTGACT	0	0	0	0
tsrna-07114	ATGGATAAGGCGTCTGAC	1	1	1	0
tsrna-07112	ATGGATAAGGCGTCTG	1	0	0	0
tsrna-07111	ATGGATAAGGCATTGGC	1	1	0	0
tsrna-07110	ATGGATAAGGCATTGG	2	2	0	0
tsrna-07109	ATGGATAAGGCATCAGCCTCCGGAGCTGGG	6	3	0	0
tsrna-07108	ATGGATAAGGCATCAGCCTCCGGAGCTGG	3	2	3	0
tsrna-07107	ATGGATAAGGCATCAGCCTCCGGAGCTG	2	2	3	1
tsrna-07106	ATGGATAAGGCATCAGCCTCC	3	2	1	0
tsrna-07105	ATGGATAAGGCATCAGCCTC	3	3	1	1
tsrna-07104	ATGGATAAGGCATCAGCCT	4	4	2	0
tsrna-07103	ATGGATAAGGCATCAGCC	3	4	1	0
tsrna-07102	ATGGATAAGGCATCAGC	1	2	2	2
tsrna-07101	ATGGATAAGGCATCAG	1	4	3	0
tsrna-07100	ATGGATAAGGCACTGGC	16	17	6	4
tsrna-07099	ATGGATAAGGCACTGG	14	6	7	2
tsrna-07098	ATGGATAACGCGTCTGACTACGGA	1	1	1	0
tsrna-07097	ATGGATAACGCGTCTGACTACGG	2	1	1	3
tsrna-07096	ATGGATAACGCGTCTGACTACG	0	1	0	0
tsrna-07095	ATGGATAACGCGTCTGACTAC	0	0	0	1
tsrna-07093	ATGGATAACGCGTCTGACT	0	0	0	0
tsrna-07090	ATGGAGGCGTGGGTTCGAATCCCACTTCTGACACCA	20	48	8	6
tsrna-07089	ATGGAGGCGTGGGTTCGAATCCCACTTCTGACACC	5	10	0	1
tsrna-07088	ATGGAGGCGTGGGTTCGAATCCCACTTCTGACA	0	0	0	0
tsrna-07087	ATGGAGGCCATGGGGTTGGCTTG	0	2	0	0
tsrna-07086	ATGGAGGCCATGGGGTTGGCTT	0	2	0	0
tsrna-07085	ATGGAGGCCATGGGGTTGGCT	1	0	0	0
tsrna-07084	ATGGAGGCCATGGGGTTGGC	0	0	0	0
tsrna-07083	ATGGAGGCCATGGGGTTGG	2	0	1	0
tsrna-07082	ATGGAGGCCATGGGGTTG	0	0	0	0
tsrna-07081	ATGGAGGCCATGGGGTT	0	1	0	0
tsrna-07080	ATGGAGGCCATGGGGT	0	0	0	0
tsrna-07079	ATGCTTTGCACGTATGAGGCCCCGGGT	29	79	22	31
tsrna-07078	ATGCGAGAGGTAGCGGGA	3	15	0	0
tsrna-07077	ATGCCGAGGTTGTGAGTTCGATC	2	2	1	0
tsrna-07076	ATGCCGAGGTTGTGAGTTCG	0	2	0	0
tsrna-07074	ATGCCGAGGTTGTGAGTT	0	1	0	0
tsrna-07073	ATGCCGAGGTTGTGAGT	0	2	0	0
tsrna-07072	ATGCCCGCATTCTCCACCA	1	5	1	0
tsrna-07071	ATGCCCGCATCCTCCACCA	2	3	0	0
tsrna-07070	ATGCCCGCATCCTCCACC	0	2	0	0
tsrna-07069	ATGCCCGCATCCTCCAC	0	1	0	0
tsrna-07067	ATGCCCCCATGTCTAACAACATGGCTTTCTCACCA	5	5	10	1
tsrna-07066	ATGCCCCCATGTCTAACAACATGGCTTTCTCACC	3	0	3	0
tsrna-07065	ATGCCCCCATGTCTAACAACATGGCTTTCT	0	1	1	0
tsrna-07064	ATGCCCCCATGTCTAACAACATGGCTTTC	0	2	0	0
tsrna-07062	ATGCCCCCATGTCTAACAACATGGC	0	1	0	0
tsrna-07059	ATGCCCCCATGTCTAACAACA	0	1	0	0
tsrna-07054	ATGCAGAGTGGGGTTTTGCAGTCCTTACC	1	1	0	1
tsrna-07053	ATGCAGAGTGGGGTTTTGCAGTCCT	0	1	0	1
tsrna-07052	ATGCAGAGTGGGGTTTTGCAGTCC	0	1	1	0
tsrna-07051	ATGCAGAGTGGGGTTTTGCAGTC	0	0	0	0
tsrna-07050	ATGCAGAGTGGGGTTTTGCAG	0	0	0	0
tsrna-07049	ATGCAGAGTGGGGTTTTGCA	0	0	0	0
tsrna-07046	ATGCACGAGGTCCTGGGT	3	7	1	1
tsrna-07045	ATGCACGAGGCCCCGGGT	0	1	0	0

tsrna-07044	ATGCAAGATTCCCATTCTTGCGACCCGG	2	3	0	1
tsrna-07043	ATGCAAGATTCCCATTCTTGCGACCCG	0	1	2	0
tsrna-07042	ATGATTTCGACTCATTAAATTATGATA	6	0	2	0
tsrna-07036	ATGATTCTCGGTTTGGGT	0	1	0	0
tsrna-07031	ATGATTCTCGCTTTGGGT	0	0	1	0
tsrna-07028	ATGATTCTCGCTTCGGGTGTGA	1	0	0	0
tsrna-07025	ATGATTCTCGCTTCGGGTGCGAG	0	1	0	0
tsrna-07021	ATGATCGTATAGTGGTTAGTACTCTGCG	8	7	3	0
tsrna-07020	ATGATCGTATAGTGGTTAGTACTCTGC	2	5	0	2
tsrna-07019	ATGATCGTATAGTGGTTAGTACTCTG	0	1	0	0
tsrna-07018	ATGATCGTATAGTGGTTAGTACTCT	2	0	1	0
tsrna-07017	ATGATCGTATAGTGGTTAGTACTC	0	1	0	0
tsrna-07009	ATGAGGTCCCGGGTTCGATCCCCGGC	1	4	0	0
tsrna-07008	ATGAGGTCCCGGGTTCGATCCCCAGC	1	5	1	0
tsrna-07007	ATGAGGTCCCGGGTTCGATCCCC	0	1	0	0
tsrna-07006	ATGAGGTCCCGGGTTCGATCCC	1	2	1	0
tsrna-07005	ATGAGGTCCCGGGTTCGATCC	1	2	0	0
tsrna-07004	ATGAGGTCCCGGGTTCGATC	0	4	0	0
tsrna-07003	ATGAGGTCCCGGGTTCG	2	2	1	0
tsrna-07002	ATGAGGCCCCGGGTTCGATCCCCGGC	0	2	0	0
tsrna-07001	ATGAGGCCCCGGGTTCGATCCC	2	3	1	0
tsrna-07000	ATGAGGCCCCGGGTTCGATCC	0	1	0	1
tsrna-06999	ATGAGGCCCCGGGTTCGATC	0	2	0	0
tsrna-06998	ATGAGGCCCCGGGTTCG	1	10	0	1
tsrna-06997	ATGAAAACCTTTTTCCAAGGACACCA	2	1	4	0
tsrna-06996	ATGAAAACCTTTTTCCAAGGACACC	0	2	0	0
tsrna-06995	ATGAAAACCTTTTTCCAAGGACA	0	2	0	0
tsrna-06990	ATCTTAGCTTTGGGTGCTAATGGTGGAGTT	1	1	0	0
tsrna-06986	ATCTGGGTGCCCCCTCCA	0	1	0	0
tsrna-06985	ATCTGAGGGTCCAGGGTTT	1	9	0	0
tsrna-06984	ATCTGAGGGTCCAGGGTTCATGTCCCTGTT	0	1	0	0
tsrna-06983	ATCTGAGGGTCCAGGGTTCAAGTCCCTGTTCGGGCGCCA	7	24	5	5
tsrna-06982	ATCTGAGGGTCCAGGGTTCAAGTCCCTGTTCGGGCGCC	1	4	0	1
tsrna-06981	ATCTGAGGGTCCAGGGTTCAAGTCCCTGTTCGGGCG	1	2	0	0
tsrna-06980	ATCTGAGGGTCCAGGGTTCAAGT	0	4	1	1
tsrna-06979	ATCTGACAACAGAGGCTTACGACCCCTTATTTACCC	0	0	1	1
tsrna-06976	ATCTGAAGGTCGTGAGTTCGATCCTCACACGGGGCACCA	3	28	2	0
tsrna-06975	ATCTGAAGGTCGTGAGTTCGATCCTCACACGGGGCACC	2	6	2	1
tsrna-06974	ATCTGAAGGTCGTGAGTTCGATCCTCACACGGGGCAC	0	4	2	0
tsrna-06973	ATCTGAAGGTCGTGAGTTCGAGCCTCACACGGGGCACCA	5	6	2	0
tsrna-06972	ATCTGAAGGTCGTGAGTTCGAGCCTCACACGGGGCACC	1	4	2	0
tsrna-06971	ATCTGAAGGTCCTGAGTTCGAACCTCAGAGGGGGCACCA	11	35	7	3
tsrna-06970	ATCTGAAGGTCCTGAGTTCGAACCTCAGAGGGGGCACC	15	48	6	2
tsrna-06969	ATCTGAAGGTCCTGAGT	17	30	13	7
tsrna-06968	ATCTCGGTGGTACCTCCA	0	3	1	0
tsrna-06967	ATCTCGGTGGGACCTCCA	53	86	13	19
tsrna-06966	ATCTCGGTGGGACCTCC	4	8	2	1
tsrna-06965	ATCTCGGTGGGACCTC	0	1	0	0
tsrna-06964	ATCTCGGTGGAACCTCCA	105	155	38	16
tsrna-06963	ATCTCGGTGGAACCTCC	3	11	3	1
tsrna-06962	ATCTCGGTGGAACCTC	0	2	0	0
tsrna-06961	ATCTCGCTGGGGCCTCCA	35	63	22	11
tsrna-06960	ATCTCGCTGGGGCCTCC	2	7	0	0
tsrna-06958	ATCTAAAGGTCCCTGGTTCGATCCCGGGTTTCGGCACCA	52	155	21	17
tsrna-06957	ATCTAAAGGTCCCTGGTTCGATCCCGGGTTTCGGCACC	27	48	9	9
tsrna-06956	ATCTAAAGGTCCCTGGTTCGATCCCGGGTT	9	40	9	2
tsrna-06955	ATCTAAAGGTCCCTGGTTCGATC	25	27	8	3
tsrna-06954	ATCTAAAGGTCCCTGGTTCGAT	16	31	5	4
tsrna-06953	ATCTAAAGGTCCCTGGTTCGA	18	26	5	5
tsrna-06952	ATCTAAAGGTCCCTGGTTCG	11	28	3	4
tsrna-06951	ATCTAAAGGTCCCTGGTTC	19	25	6	5
tsrna-06950	ATCTAAAGGTCCCTGGTT	16	25	7	2
tsrna-06949	ATCTAAAGGTCCCTGGT	8	14	7	5
tsrna-06948	ATCTAAAGGTCCCTGG	0	0	0	1

tsrna-06947	ATCTAAAGACAGGGGTTAGGCCTCTTTTTACCACC	5	10	9	0
tsrna-06946	ATCTAAAGACAGGGGTTAGGCCTCTTTTT	4	8	1	0
tsrna-06945	ATCTAAACCATCCTCTGCTACCA	1	6	0	1
tsrna-06944	ATCGTATAGTGGTTAGTACTCTGCGTTGTG	34	11	9	10
tsrna-06943	ATCGTATAGTGGTTAGTACTCTGCGTTGT	27	11	18	12
tsrna-06942	ATCGTATAGTGGTTAGTACTCTGCGTTG	28	12	17	5
tsrna-06941	ATCGTATAGTGGTTAGTACTCTGCGTT	24	10	13	6
tsrna-06940	ATCGTATAGTGGTTAGTACTCTGCGT	6	4	7	1
tsrna-06939	ATCGTATAGTGGTTAGTACTCTGCG	6	5	2	3
tsrna-06938	ATCGTATAGTGGTTAGTACTCTGC	5	1	3	1
tsrna-06937	ATCGTATAGTGGTTAGTACTCTG	2	0	2	1
tsrna-06936	ATCGTATAGTGGTTAGTACTCT	1	0	1	0
tsrna-06935	ATCGTATAGTGGTTAGTACTC	1	1	0	1
tsrna-06931	ATCGGGCCCATACCCCGAAAATGTTGGTT	0	1	0	0
tsrna-06927	ATCGATGCCCGCATTCTCCACCA	1	4	0	0
tsrna-06925	ATCGATGCCCGCATCCTCCACCA	1	3	0	1
tsrna-06921	ATCGAAACCATCCTCTGCTACCA	20	26	7	8
tsrna-06920	ATCGAAACCATCCTCTGCTACC	6	3	1	0
tsrna-06918	ATCGAAACCATCCTCTGC	0	0	0	0
tsrna-06916	ATCCTTAGGTCGCTGGTTCGATTCCGGCTCGAAGGA	2	5	2	2
tsrna-06915	ATCCTTAGGTCGCTGGTTCGATTCCGGCTC	3	2	1	1
tsrna-06914	ATCCTTAGGTCGCTGGTTCGATT	2	2	2	0
tsrna-06913	ATCCTTAGGTCGCTGGTTCGAAT	0	0	3	0
tsrna-06912	ATCCTTAGGTCGCTGGTTCGA	0	2	0	0
tsrna-06911	ATCCTTAGGTCGCTGGTTCG	1	0	2	0
tsrna-06910	ATCCTTAGGTCGCTGGTTC	1	1	1	1
tsrna-06909	ATCCTTAGGTCGCTGGTT	0	1	0	0
tsrna-06906	ATCCTGTTCGTGACGCCA	4	6	5	2
tsrna-06903	ATCCTGTCGGCTACGCCA	4	7	2	7
tsrna-06902	ATCCTGTCGGCTACGCC	1	1	0	0
tsrna-06901	ATCCTGGGTTCGAATCCCAGCGGTGCCTCC	4	3	0	0
tsrna-06900	ATCCTGGGTTCGAATCCCAGCGGGGCCTCC	0	1	0	0
tsrna-06899	ATCCTGCTCACAGCGCCA	7	13	2	1
tsrna-06898	ATCCTGCTCACAGCGCC	3	1	0	0
tsrna-06897	ATCCTGCTCACAGCGC	0	0	1	0
tsrna-06896	ATCCTGCCGACTACGCCA	90	229	32	20
tsrna-06895	ATCCTGCCGACTACGCC	5	12	2	0
tsrna-06894	ATCCTGCCGACTACGC	0	1	0	0
tsrna-06893	ATCCTCGCTGGGGCCTCCA	13	8	1	4
tsrna-06892	ATCCTCACCTGGAGCACCA	1	2	0	1
tsrna-06889	ATCCGGGTGCCCCCTCCA	137	189	59	15
tsrna-06888	ATCCGGGTGCCCCCTCC	5	4	1	2
tsrna-06887	ATCCGGGTGCCCCCTC	0	3	0	0
tsrna-06886	ATCCGGCTCGGAGGACCA	1	3	0	0
tsrna-06885	ATCCGGCTCGGAGGACC	0	1	0	0
tsrna-06883	ATCCGGCTCGAAGGACCA	24	29	11	3
tsrna-06882	ATCCGGCTCGAAGGACC	0	2	0	0
tsrna-06881	ATCCGGCTCGAAGGAC	0	0	0	0
tsrna-06876	ATCCCTGGTGGTCTAGTGGTTAGGATTCGG	20	41	22	19
tsrna-06875	ATCCCTGGTGGTCTAGTGGTTAGGATTCG	20	39	21	19
tsrna-06874	ATCCCTGGTGGTCTAGTGGTTAGGATTC	26	34	28	22
tsrna-06873	ATCCCTCCGTGGTTACCA	3	10	0	1
tsrna-06872	ATCCCTCCGTGGTTACC	0	4	0	0
tsrna-06871	ATCCCGGGTTTCGGCACCA	39	99	11	7
tsrna-06870	ATCCCGGGTTTCGGCACC	0	6	1	3
tsrna-06869	ATCCCGGGTTTCGGCAC	0	1	0	0
tsrna-06867	ATCCCGGGCGGAAACACCA	16	30	5	6
tsrna-06866	ATCCCGGACGAGCCCCCA	123	154	46	55
tsrna-06865	ATCCCGGACGAGCCCCC	8	11	0	0
tsrna-06864	ATCCCGGACGAGCCCC	1	2	0	0
tsrna-06863	ATCCCCGTACTGGCCACCA	1	3	2	2
tsrna-06862	ATCCCCGTACGGGCCACCA	7	51	4	4
tsrna-06861	ATCCCCGGCATCTCCACCA	102	126	41	29
tsrna-06860	ATCCCCGGCATCTCCACC	3	4	1	2

tsrna-06857	ATCCCCGGCACCTCCACCA	159	258	85	27
tsrna-06856	ATCCCCGGCACCTCCACC	6	11	3	3
tsrna-06855	ATCCCCGGCACCTCCAC	1	1	0	0
tsrna-06854	ATCCCCGGCACCTCCA	0	0	0	0
tsrna-06853	ATCCCCGCCTGTCACGCGGGAGACCGGGGTTCGATTC	13	46	8	5
tsrna-06852	ATCCCCGCCTGTCACGCGGGAGACCGG	20	58	9	1
tsrna-06851	ATCCCCGCCTGTCACGCGGGAGACCG	12	48	4	4
tsrna-06849	ATCCCCGACACCTCCACCA	5	2	1	1
tsrna-06848	ATCCCCAGTACCTCCACCA	0	2	1	0
tsrna-06844	ATCCCCAGCATCTCCACCA	1	5	0	1
tsrna-06842	ATCCCCAGCACCTCCACCA	0	1	0	0
tsrna-06839	ATCCCATCCTCGTCGCCA	5	1	0	1
tsrna-06838	ATCCCATCCTCGTCGCC	0	1	0	0
tsrna-06836	ATCCCATATGGTCTAGCGGTTAGGATTCCT	2	1	0	0
tsrna-06835	ATCCCAGTAGAGCCTCCA	3	9	3	3
tsrna-06834	ATCCCAGTAGAGCCTCC	0	1	0	0
tsrna-06826	ATCCCAGCGAGGCCTCCA	0	0	0	1
tsrna-06825	ATCCCACTTCTGACACCA	4	2	2	0
tsrna-06823	ATCCCACTTCTGACAC	0	0	0	0
tsrna-06822	ATCCCACTGCTGCCACCA	0	3	0	0
tsrna-06821	ATCCCACTCCTGACACCA	55	65	46	27
tsrna-06820	ATCCCACTCCTGACACC	0	0	0	0
tsrna-06819	ATCCCACTCCTGACAC	0	0	0	1
tsrna-06818	ATCCCACCTTCGTCGCCA	1	0	0	0
tsrna-06817	ATCCCACCTTCGTCGCC	0	0	0	0
tsrna-06815	ATCCCACCGCTGCTACCA	1	5	1	0
tsrna-06814	ATCCCACCGCTGCCACCA	0	0	0	0
tsrna-06813	ATCCCACCGCTGCCACC	0	1	0	0
tsrna-06812	ATCCCACCGCTGCCAC	0	0	1	0
tsrna-06811	ATCCCACCCTCGTCGCCA	4	15	2	2
tsrna-06810	ATCCCACCCTCGTCGCC	0	1	0	1
tsrna-06808	ATCCCACCCAGGGACGCCA	2	3	0	0
tsrna-06807	ATCCCACCAGAGTCGCCA	2	7	1	1
tsrna-06806	ATCCCACCAGAGTCGCC	1	3	0	0
tsrna-06802	ATCCCACATGGTCTAGCGGTTAGGATTCCT	10	9	5	10
tsrna-06801	ATCCCACATGGTCTAGCGGTTAGGATT	1	12	12	11
tsrna-06800	ATCCCACATGGTCTAGCGGT	3	3	1	2
tsrna-06799	ATCCATTGTGCTTTGCACGCGTGGGTTCG	1	4	0	0
tsrna-06798	ATCCATTGGTCTTAGGCCCCAAAAATTTTGGTGCAACTCCAAA	26	55	32	2
tsrna-06797	ATCCATTGGTCTTAGGCCCCAAAAATTTTGGTGCAACTCC	3	2	22	0
tsrna-06796	ATCCATTGGTCTTAGGCCCCAAAAATTTTGGTGCAA	1	2	4	0
tsrna-06795	ATCCATTGGTCTTAGGCCCCAA	0	0	4	0
tsrna-06794	ATCCATTGGTCTTAGGCCCCA	0	0	2	0
tsrna-06792	ATCCATTGGTCTTAGGCCC	0	0	1	0
tsrna-06788	ATCCAGGTGCCCCTTCCA	0	2	1	0
tsrna-06787	ATCCAGGTGCCCCCTCCA	0	3	0	4
tsrna-06784	ATCCAGCGATCCGAGTTCAAATC	1	0	0	0
tsrna-06776	ATCATTGGTCGTGGTTGTAGTCCGTGCGAGAATACCA	122	131	155	39
tsrna-06775	ATCATTGGTCGTGGTTGTAGTCCGTGCGAGAATACC	21	33	21	14
tsrna-06774	ATCATTGGTCGTGGTTGTAGTCCGTGCGAGAATA	15	7	11	6
tsrna-06773	ATCATTGGTCGTGGTTGTAGTCCGTGCGAGAAT	11	11	5	12
tsrna-06772	ATCATTGGTCGTGGTTGTAGTCCGTGCGAGAA	10	18	12	8
tsrna-06771	ATCATTGGTCGTGGTTGTAGTCCGTGCGAGA	5	8	8	6
tsrna-06770	ATCATTGGTCGTGGTTGTAGTCCGTGCGAG	4	12	3	6
tsrna-06769	ATCATTGGTCGTGGTTGTAGTCCGTGCGA	2	8	8	3
tsrna-06768	ATCATTGGTCGTGGTTGTAGTCCGTGCG	3	3	2	4
tsrna-06767	ATCATTGGTCGTGGTTGTAGTCCGTGC	3	4	2	1
tsrna-06766	ATCATTGGTCGTGGTTGTAGTCCGTG	1	6	1	0
tsrna-06765	ATCATTGGTCGTGGTTGTAGTCCGT	0	1	1	0
tsrna-06764	ATCATTGGTCGTGGTTGTAGTCCG	0	2	2	0
tsrna-06763	ATCATTGGTCGTGGTTGTAGTCC	0	0	0	0
tsrna-06762	ATCATTGGTCGTGGTTGTAGTC	0	0	0	0
tsrna-06761	ATCATTGGTCGTGGTTGTAGT	0	2	1	0
tsrna-06760	ATCATTGGTCGTGGTTGTAG	0	0	0	0

tsrna-06758	ATCATTGGTCGTGGTTGT	0	0	0	0
tsrna-06755	ATCATGCAAGATTCCCATTCTT	0	0	0	1
tsrna-06751	ATCAGATTGTGAATCTGACAACAG	1	0	2	0
tsrna-06750	ATCAGATTGTGAATCTGACAACA	0	1	2	0
tsrna-06749	ATCAGATTGTGAATCTGACA	0	0	2	0
tsrna-06747	ATCAGATTGTGAATCTGA	0	0	0	0
tsrna-06744	ATCAGACTTTTAATCTGAGGGTCCAG	11	33	4	5
tsrna-06743	ATCAGAAGGTTGCGTGTTCA	1	4	1	0
tsrna-06742	ATCAGAAGGTTGCGTGTTC	2	2	2	0
tsrna-06741	ATCAGAAGGTTGCGTGTT	1	4	0	1
tsrna-06740	ATCAGAAGGCTGCGTGTTCGAA	0	2	1	0
tsrna-06739	ATCAGAAGGCTGCGTGTTC	0	1	0	0
tsrna-06736	ATCAGAAGATTGAGGGTTCGAGTCCCTTCGTGGTCGCCA	2	15	5	4
tsrna-06735	ATCAGAAGATTGAGGGTTCGAGTCCCTTCGTGGTCGCC	5	10	3	1
tsrna-06734	ATCAGAAGATTGAGGGTTCGAGTCCCTTCGTGGTCGC	8	13	0	1
tsrna-06733	ATCAGAAGATTCTAGGTTCGACTCCTGGCTGGCTCGCC	13	17	11	3
tsrna-06732	ATCAGAAGATTCTAGGTTCGACTCCTGGCTGGCTC	5	9	1	0
tsrna-06731	ATCAGAAGATTCCAGGTTCGACTCCTGGCTGGCTCGCC	7	2	3	1
tsrna-06730	ATCAGAAGATTCCAGGTTCGACTCCTGGCT	0	2	0	0
tsrna-06729	ATCACGTTCGCCTCACACGCGA	1	1	0	0
tsrna-06728	ATCACGTTCGCCTCACACGCG	0	0	0	0
tsrna-06723	ATCACGTTCGCCTCAC	0	0	0	0
tsrna-06719	ATCACGTCTGCTTTACAC	1	0	0	0
tsrna-06716	ATCACGTCGGGGTCACCA	3	8	3	4
tsrna-06715	ATCACGTCGGGGTCACC	1	0	1	0
tsrna-06713	ATCACCCCATAAACACCA	1	1	0	0
tsrna-06710	ATCAAGAGGTCCCCGGTTC	1	3	0	0
tsrna-06709	ATCAAGAGGTCCCCGGTT	0	4	0	1
tsrna-06708	ATCAAGAGGTCCCCGGT	1	3	2	0
tsrna-06706	ATATTGAATTGCAAATTCGAAGAAGCAGCTTCAAACCTGCCGC	1	1	1	0
tsrna-06705	ATATTGAATTGCAAATTCGAAGAAGCAGCTTCAAACCTGCCGC	1	2	0	0
tsrna-06702	ATATGTCCGCGTGGGTT	4	5	0	0
tsrna-06701	ATATGTCCGCGTGGGT	1	2	1	0
tsrna-06700	ATATGGTCTAGCGGTTAGGATTCCTGGTTT	5	3	6	3
tsrna-06699	ATATGGTCTAGCGGTTAGGATTCCTGGTT	5	6	3	2
tsrna-06698	ATATGGTCTAGCGGTTAGGATTCCTGGT	6	5	5	3
tsrna-06697	ATATGGTCTAGCGGTTAGGATTCCTGG	3	4	0	0
tsrna-06696	ATATGGTCTAGCGGTTAGGATTCCTG	3	0	2	0
tsrna-06695	ATATGGTCTAGCGGTTAGGATTCCT	0	0	0	1
tsrna-06694	ATATGGTCTAGCGGTTAGGATTCC	0	0	0	0
tsrna-06693	ATATGGTCTAGCGGTTAGGATTC	0	0	0	0
tsrna-06689	ATATCATTGGTCGTGGTTGTAGTCCGTGCGAGAATACCA	150	132	195	51
tsrna-06688	ATATCATTGGTCGTGGTTGTAGTCCGTGCGAGAATACC	22	33	19	14
tsrna-06687	ATATCATTGGTCGTGGTTGTAGTCCGTGCGAGAATA	9	21	9	9
tsrna-06686	ATATCATTGGTCGTGGTTGTAGTCCGTGCGAGAAT	12	13	11	2
tsrna-06685	ATATCATTGGTCGTGGTTGTAGTCCGTGCGAGAA	7	10	10	5
tsrna-06684	ATATCATTGGTCGTGGTTGTAGTCCGTGCGAGA	6	22	7	11
tsrna-06683	ATATCATTGGTCGTGGTTGTAGTCCGTGCGAG	4	11	2	4
tsrna-06682	ATATCATTGGTCGTGGTTGTAGTCCGTGCGA	5	4	9	8
tsrna-06681	ATATCATTGGTCGTGGTTGTAGTCCGTGCG	3	4	0	5
tsrna-06680	ATATCATTGGTCGTGGTTGTAGTCCGTGC	2	3	0	0
tsrna-06679	ATATCATTGGTCGTGGTTGTAGTCCGTG	5	3	2	0
tsrna-06678	ATATCATTGGTCGTGGTTGTAGTCCGT	2	1	1	0
tsrna-06677	ATATCATTGGTCGTGGTTGTAGTCCG	0	2	0	1
tsrna-06676	ATATCATTGGTCGTGGTTGTAGTCC	3	1	0	1
tsrna-06675	ATATCATTGGTCGTGGTTGTAGTC	1	0	1	0
tsrna-06674	ATATCATTGGTCGTGGTTGTAGT	0	0	0	0
tsrna-06673	ATATCATTGGTCGTGGTTGTAG	0	0	0	0
tsrna-06663	ATAGTGTAGTGGTTATCACGTCTGCTTT	0	0	1	0
tsrna-06659	ATAGTGTAGTGGTTATC	1	0	1	0
tsrna-06658	ATAGTGGTTAGTATCCCCGCCTGT	0	1	1	1
tsrna-06657	ATAGTGGTTAGTATCCCCGCCT	1	1	1	0
tsrna-06656	ATAGTGGTTAGTACTCTGCGTTGTGGCCG	21	13	15	8
tsrna-06655	ATAGTGGTTAGTACTCTGCGTTGTGGCC	24	14	12	9

tsrna-06654	ATAGTGGTTAGTACTCTGCGTTGTGGC	22	13	5	7
tsrna-06653	ATAGTGGTTAGTACTCTGCGTTGTGG	22	11	6	7
tsrna-06652	ATAGTGGTTAGTACTCTGCGTTGTG	25	5	10	12
tsrna-06651	ATAGTGGTTAGTACTCTGCGTTGT	24	9	11	9
tsrna-06650	ATAGTGGTTAGTACTCTGCGTTG	15	7	13	7
tsrna-06649	ATAGTGGTTAGTACTCTGCGTT	20	9	6	5
tsrna-06648	ATAGTGGTTAGTACTCTGCGT	7	1	3	1
tsrna-06647	ATAGTGGTTAGTACTCTGCGC	4	3	1	1
tsrna-06646	ATAGTGGTTAGTACTCTGCG	2	1	1	1
tsrna-06645	ATAGTGGTTAGTACTCTGC	1	3	0	0
tsrna-06641	ATAGTGGTGAGTATCCCCGCCTGTC	7	7	1	2
tsrna-06640	ATAGTGGTGAGTATCCCCGCCTGT	2	3	0	1
tsrna-06639	ATAGTGGTGAGTATCCCCGCCTG	1	5	0	0
tsrna-06638	ATAGTGGTGAGTATCCCCGCCT	2	5	1	0
tsrna-06637	ATAGTGGTGAGTATCCCCGCC	0	8	1	0
tsrna-06636	ATAGTGGTGAGTATCCCCGC	3	4	0	0
tsrna-06635	ATAGTGGTGAGTATCCCCG	1	3	0	0
tsrna-06633	ATAGTGGTGAGCATAGCTGCC	1	0	0	0
tsrna-06631	ATAGTACCGTTAACTTCCAATTAACTAGTT	2	0	3	0
tsrna-06630	ATAGTACCGTTAACTTCCAATTA	0	0	1	0
tsrna-06629	ATAGTACCGTTAACTTCCA	1	0	0	0
tsrna-06626	ATAGGTGGTTCAGTGGTAGAATTCT	2	1	1	0
tsrna-06625	ATAGGTGGTTCAGTGGTAGAATTC	1	4	2	1
tsrna-06624	ATAGGTGGTTCAGTGGTAGAA	0	2	1	0
tsrna-06623	ATAGGTGGTTCAGTGGTAGA	0	1	1	0
tsrna-06621	ATAGGTGGCACGGAGAATTTTGGATT	2	1	0	0
tsrna-06620	ATAGGTGGCACGGAGAATTTTGGAT	0	1	2	0
tsrna-06619	ATAGGTGGCACGGAGAATTTTGGA	1	2	2	0
tsrna-06618	ATAGGTGGCACGGAGAATTTTGG	0	2	0	0
tsrna-06617	ATAGGTGGCACGGAGAATTTTG	0	2	2	0
tsrna-06616	ATAGGTGGCACGGAGAATTTT	1	0	0	0
tsrna-06614	ATAGGTGGCACGGAGAATT	0	0	0	0
tsrna-06613	ATAGGTGGCACGGAGAAT	1	0	0	0
tsrna-06611	ATAGGTGGCACGGAGA	0	0	0	0
tsrna-06610	ATAGGAGCTTAAACCCCCTTATTTCTACC	4	2	1	0
tsrna-06605	ATAGCTTAGGGGTAGAGCATTTGACTGC	2	0	0	0
tsrna-06604	ATAGCTTAGCGGTAGAGCATTTGACTGC	0	0	0	5
tsrna-06603	ATAGCTTAGCGGTAGAGCATTTGACTG	1	0	0	1
tsrna-06601	ATAGCTGCCTTCCAAGCAGTTGACCCGG	1	4	0	0
tsrna-06600	ATAGCTGCCTTCCAAGCAGTTGACCCG	0	2	1	1
tsrna-06598	ATAGCTCAGTTGGTAGAGCGGAGGA	0	1	0	0
tsrna-06597	ATAGCTCAGTTGGTAGAACATCAGA	1	0	0	0
tsrna-06593	ATAGCTCAGTGGTAGAGCATTTGACTGCAG	3	3	2	0
tsrna-06592	ATAGCTCAGTGGTAGAGCATTTGACTGCA	4	0	0	0
tsrna-06591	ATAGCTCAGTGGTAGAGCATTTGACTGC	0	2	2	2
tsrna-06590	ATAGCTCAGTGGTAGAGCATTTGACTG	4	1	1	0
tsrna-06589	ATAGCTCAGTGGTAGAGCATTTGACTAC	2	2	1	1
tsrna-06588	ATAGCTCAGTGGTAGAGCATTTGACTA	5	2	1	0
tsrna-06587	ATAGCTCAGTGGTAGAGCATTTGACT	5	0	0	0
tsrna-06586	ATAGCTCAGTGGTAGAGCATTTGAC	2	1	0	0
tsrna-06585	ATAGCTCAGTGGTAGAGCATTTGA	1	0	0	0
tsrna-06584	ATAGCTCAGTGGTAGAGCATTTG	0	1	0	0
tsrna-06583	ATAGCTCAGTGGTAGAGCATTT	2	1	0	0
tsrna-06582	ATAGCTCAGTGGTAGAGCATT	1	2	0	0
tsrna-06580	ATAGCTCAGTGGGTAGAGCATTTGACTGC	0	1	1	0
tsrna-06579	ATAGCTCAGTGGGTAGAGCATTTGACTG	1	2	1	1
tsrna-06578	ATAGCTCAGTGGGTAGAGCATTTGACT	0	2	0	0
tsrna-06577	ATAGCTCAGTGGGTAGAGCATTTGAC	1	2	0	0
tsrna-06575	ATAGCTCAGTCGGTAGAGCATCAGACTTTT	20	11	13	11
tsrna-06574	ATAGCTCAGTCGGTAGAGCATCAGACTTT	17	6	4	4
tsrna-06573	ATAGCTCAGTCGGTAGAGCATCAGACT	17	12	4	1
tsrna-06572	ATAGCTCAGTCGGTAGAGCATCAGAC	11	7	13	0
tsrna-06571	ATAGCTCAGTCGGTAGAGCATCAGA	8	7	6	1
tsrna-06570	ATAGCTCAGTCGGTAGAGCATCAG	5	4	4	0

tsrna-06569	ATAGCTCAGTCGGTAGAGCATC	1	2	1	1
tsrna-06564	ATAGCTCAGGTGGTAGAGCATTTGACTGC	2	0	0	0
tsrna-06563	ATAGCTCAGGTGGTAGAGCATTTGACTG	0	1	0	0
tsrna-06562	ATAGCTCAGGTGGTAGAGCATTTGACT	0	0	0	0
tsrna-06559	ATAGCTCAGGGGTAGAGCATTTGACTGC	2	2	1	8
tsrna-06558	ATAGCTCAGGGGTAGAGCATTTGACTG	1	0	0	2
tsrna-06557	ATAGCTCAGGGGTAGAGCATTTGACT	1	1	0	0
tsrna-06556	ATAGCTCAGGGGTAGAGCATTTGAC	0	0	0	1
tsrna-06555	ATAGCTCAGGGGTAGAGCATTTGA	1	0	0	0
tsrna-06554	ATAGCTCAGGGGTAGAGCATTTG	0	1	0	0
tsrna-06553	ATAGAGTAAATAATAGGAGCTTAAACCCCCTTATTTCTACC	6	2	6	0
tsrna-06552	ATAGAGTAAATAATAGGAGCTTAAACCCCCTTATTTCT	4	2	7	0
tsrna-06551	ATAGAGTAAATAATAGGAGCTTAAACCCCC	2	0	0	0
tsrna-06550	ATAGAGTAAATAATAGGAGCTTAAACCC	0	0	0	0
tsrna-06549	ATAGAGTAAATAATAGGAGCTT	2	0	0	0
tsrna-06548	ATAGAGTAAATAATAGGAGCT	0	0	0	0
tsrna-06544	ATACTTAATTTCTGCCA	1	0	0	0
tsrna-06543	ATACTTAATTTCTGCC	0	0	0	0
tsrna-06542	ATACCCTTCCCGTACTACCA	1	1	0	0
tsrna-06541	ATACCCTTCCCGTACTACC	2	0	0	0
tsrna-06538	ATACCCCGAAAATGTTGGTTATACCCTTCC	1	0	0	0
tsrna-06535	ATACAGACCAAGAGCCTTCAAAG	7	5	3	1
tsrna-06533	ATACAGACCAAGAGCCTTC	1	0	0	0
tsrna-06531	ATACACTGAAAATGTTTAGACGGGCTC	8	5	2	2
tsrna-06530	ATACACTGAAAATGTTTAGACGGGCT	2	2	0	1
tsrna-06526	ATACACCAGTCTTGTAAACCGG	2	4	2	0
tsrna-06525	ATACACCAGTCTTGTAAACCG	1	3	2	0
tsrna-06524	ATACACCAGTCTTGTAAACC	0	1	0	0
tsrna-06523	ATACAACGATGGTTTTTCATATCATTGGTCGTGGTTGTAGTCC	0	3	5	0
tsrna-06522	ATACAACGATGGTTTTTCATATCATTGGTCGTGGTTGTAGTC	3	0	4	1
tsrna-06521	ATACAACGATGGTTTTTCATATCATTGGTCGTGGTTGTAGT	3	3	5	0
tsrna-06520	ATACAACGATGGTTTTTCATATCATTGGTCGTGGTTGTA	1	1	5	0
tsrna-06519	ATACAACGATGGTTTTTCATATCATTGGTCGTGGTTGT	0	1	0	0
tsrna-06518	ATACAACGATGGTTTTTCATATCATTGGTC	0	1	0	0
tsrna-06512	ATAATGCCGAGGTTGTGAGTTCGA	4	21	1	2
tsrna-06511	ATAATGCCGAGGTTGTGAGTTCG	3	24	1	3
tsrna-06510	ATAATGCCGAGGTTGTGAGTTCA	5	20	0	0
tsrna-06509	ATAATGCCGAGGTTGTGAGTTC	2	7	0	0
tsrna-06508	ATAATGCCGAGGTTGTGAGTT	1	9	2	1
tsrna-06507	ATAATGCCGAGGTTGTGAGT	2	10	1	0
tsrna-06506	ATAATGCCGAGGTTGTGAG	1	3	1	0
tsrna-06505	ATAATGCCGAGGTTGTGA	0	1	1	0
tsrna-06504	ATAATGCCGAGGTTGTG	1	1	0	0
tsrna-06502	ATAATCTGAAGGTCGTGAGTTCGATCCTCACACGGGGCACCA	4	49	5	2
tsrna-06501	ATAATCTGAAGGTCGTGAGTTCGAGCCTCACACGGGGCACCA	12	21	11	2
tsrna-06500	ATAATCTGAAGGTCGTGAGTTCGAGCCTCACACGGG	11	21	7	3
tsrna-06499	ATAATCTGAAGGTCGTGAGTTCGAGCCTCA	7	24	8	2
tsrna-06498	ATAATCTGAAGGTCGTGAGTTCGAGCCTC	4	24	5	3
tsrna-06497	ATAATCTGAAGGTCGTGAGTTCG	6	12	11	5
tsrna-06496	ATAATCTGAAGGTCGTGAGTTC	10	6	4	2
tsrna-06495	ATAATCTGAAGGTCGTGAGTT	6	14	10	3
tsrna-06494	ATAATCTGAAGGTCGTGAGT	9	11	5	4
tsrna-06493	ATAATCTGAAGGTCGTGAG	1	1	0	1
tsrna-06492	ATAATCTGAAGGTCGTG	0	3	2	1
tsrna-06491	ATAATCTGAAGGTCCTGAGTTCG	68	171	61	20
tsrna-06490	ATAATCTGAAGGTCCTGAGTT	60	149	43	23
tsrna-06489	ATAATCTGAAGGTCCTGAGT	59	159	41	32
tsrna-06488	ATAATCTGAAGGTCCTGAG	2	12	4	2
tsrna-06487	ATAATCTGAAGGTCCTG	3	5	1	0
tsrna-06481	ATAATAGGAGCTTAAACCCCCTTATTTCT	0	3	0	1
tsrna-06476	ATAATAGGAGCTTAAACCCC	0	0	0	0
tsrna-06475	ATAATAGGAGCTTAAACCC	0	0	1	0
tsrna-06470	ATAAGGCGTCTGACTTCGG	0	1	0	0
tsrna-06469	ATAAGGCGTCTGACTTCG	0	0	0	0

tsrna-06468	ATAAGCTATCGGGCCCATACC	0	0	1	0
tsrna-06467	ATAAGCTATCGGGCCCATA	0	0	0	0
tsrna-06466	ATAAGCTATCGGGCCCA	0	1	0	0
tsrna-06465	ATAAGCTATCGGGCCC	0	1	0	0
tsrna-06464	ATAACTTTGTCAAAGTTAAATTATAGGCT	6	2	8	2
tsrna-06461	ATAACGCCAAGGTCGCGGGTTCGATCCCCGTACGGGCCACC	6	18	2	0
tsrna-06460	ATAACGCCAAGGTCGCGGGTTCGA	4	14	5	2
tsrna-06459	ATAACGCCAAGGTCGCGGGTTCG	7	8	1	2
tsrna-06458	ATAACGCCAAGGTCGCGGGTT	5	6	0	1
tsrna-06457	ATAACGCCAAGGTCGCGGGT	1	8	4	0
tsrna-06455	ATAACGCCAAGGTCGCGG	1	0	0	0
tsrna-06454	ATAACCCAGAGGTCGATGGATCTA	9	34	7	12
tsrna-06453	ATAACCCAGAGGTCGATGGATCT	13	29	8	6
tsrna-06452	ATAACCCAGAGGTCGATGGATCGAAACCA	0	5	0	2
tsrna-06451	ATAACCCAGAGGTCGATGGATCGAAACC	0	10	0	2
tsrna-06450	ATAACCCAGAGGTCGATGGATCGAAAC	4	6	1	0
tsrna-06449	ATAACCCAGAGGTCGATGGATCGAAA	0	4	2	0
tsrna-06448	ATAACCCAGAGGTCGATGGATCGAA	2	10	0	1
tsrna-06447	ATAACCCAGAGGTCGATGGATCGA	5	2	1	1
tsrna-06446	ATAACCCAGAGGTCGATGGATCG	1	3	1	1
tsrna-06445	ATAACCCAGAGGTCGATGGATC	1	4	2	2
tsrna-06444	ATAACCCAGAGGTCGATGGAT	1	6	2	1
tsrna-06443	ATAACCCAGAGGTCGATGGA	2	4	0	1
tsrna-06442	ATAACCCAGAGGTCGATGG	0	2	0	0
tsrna-06441	ATAACCCAGAGGTCGATG	2	3	0	0
tsrna-06440	ATAACCCAGAGGTCGAT	2	1	1	0
tsrna-06438	ATAACAGCTATCCATTGGTCTTAGGCCCC	0	0	0	0
tsrna-06437	ATAACAGCTATCCATTGGTCTTAGGCCC	0	0	1	0
tsrna-06436	ATAACAGCTATCCATTGGTCTTAGGCC	1	0	1	0
tsrna-06435	ATAACAGCTATCCATTGGTCTTAGGC	0	0	1	0
tsrna-06434	ATAACAGCTATCCATTGGTCTTAGG	0	0	1	0
tsrna-06433	ATAACAGCTATCCATTGGTCTTAG	0	0	0	0
tsrna-06432	ATAACAGCTATCCATTGGTCTTA	1	1	0	0
tsrna-06431	ATAACAGCTATCCATTGGTCTT	0	0	0	0
tsrna-06424	ATAACACCAAGGTCGCGGGCTCG	0	1	0	0
tsrna-06423	ATAACACCAAGGTCGCGGGCT	0	2	1	0
tsrna-06422	ATAACACCAAGGTCGCGGGC	0	2	1	0
tsrna-06420	ATAAATAGTACCGTTAACTTCC	2	1	2	0
tsrna-06417	AGTTTTGACAACATTCAAAAAAGAGTACC	0	0	1	0
tsrna-06415	AGTTTTGACAACATTCAAAAAAGAGT	0	1	0	0
tsrna-06414	AGTTTCTGTGGTGTAG	1	0	0	0
tsrna-06413	AGTTTCCGTAGTGTAGTGGTTATCACGTTC	111	42	77	79
tsrna-06412	AGTTTCCGTAGTGTAGTGGTTATC	107	37	59	62
tsrna-06411	AGTTTCCGTAGTGTAGTGGTTA	19	23	6	8
tsrna-06410	AGTTTCCGTAGTGTAGTGGTT	9	17	6	7
tsrna-06409	AGTTTCCGTAGTGTAGTGGTC	16	18	5	5
tsrna-06408	AGTTTCCGTAGTGTAGTGG	14	23	3	2
tsrna-06407	AGTTTCCGTAGTGTAG	6	16	3	3
tsrna-06406	AGTTTATGTAGCTTACCTCCTCA	7	3	5	7
tsrna-06405	AGTTTATGTAGCTTACCTCCTC	4	0	2	8
tsrna-06404	AGTTTATGTAGCTTACCTCCT	9	0	5	10
tsrna-06403	AGTTTATGTAGCTTACCTCC	2	0	3	3
tsrna-06402	AGTTTATGTAGCTTACCTC	2	0	3	3
tsrna-06401	AGTTTAACCAAAACATCAGATTGTGAATCTGACAACAGAGGCT	2	6	3	0
tsrna-06396	AGTTTAAATTAGAATCTTAGCTTTG	6	3	2	0
tsrna-06395	AGTTGGTTAGAGCGTGGT	0	0	0	0
tsrna-06386	AGTTGGTAGAGCATCAGAC	0	1	0	0
tsrna-06385	AGTTGGTAGAGCATCAGA	1	1	0	0
tsrna-06381	AGTTGGGAGAGCGTTAGACTGAAGAT	1	0	0	0
tsrna-06379	AGTTGGGAGAGCGTTAGACTGAAG	0	1	0	0
tsrna-06375	AGTTGCAATACTTAATTTCTGCCA	0	0	2	1
tsrna-06374	AGTTGCAATACTTAATTTCTGCC	1	0	0	0
tsrna-06369	AGTTGATTAGGGTGCTTAGCTGTTAACTAAGTGTTTGTGGGTT	12	9	19	2
tsrna-06368	AGTTGATTAGGGTGCTTAGCTGTTAACTAAGTGTTT	9	6	7	2

tsrna-06367	AGTTGATTAGGGTGCTTAGCTGTTAACTAA	7	5	14	3
tsrna-06366	AGTTGATTAGGGTGCTTAGCTGTTAACTA	5	5	9	1
tsrna-06365	AGTTGATTAGGGTGCTTAGCTGTTAACT	1	3	6	1
tsrna-06364	AGTTGATTAGGGTGCTTAGCTGTTAAC	1	2	3	1
tsrna-06363	AGTTGATTAGGGTGCTTAGCTGTTA	0	2	0	0
tsrna-06362	AGTTGATTAGGGTGCTTAGCTGTT	0	1	2	1
tsrna-06361	AGTTGATTAGGGTGCTTAGCTGT	0	0	2	0
tsrna-06360	AGTTGATTAGGGTGCTTAGCTG	1	0	0	0
tsrna-06359	AGTTGATTAGGGTGCTTAGCT	0	0	0	0
tsrna-06358	AGTTGATTAGGGTGCTTAGC	1	0	0	0
tsrna-06353	AGTTGATGCAGAGTGGGGTTTTGCAGTCCT	2	1	0	0
tsrna-06352	AGTTGATGCAGAGTGGGGTTTTGCAGTCC	0	2	1	0
tsrna-06351	AGTTGATGCAGAGTGGGGTTTTGCAGTC	0	0	3	0
tsrna-06350	AGTTGATGCAGAGTGGGGTTTTGCA	0	0	0	1
tsrna-06349	AGTTGATGCAGAGTGGGGTTTTGC	0	1	1	0
tsrna-06348	AGTTGATGCAGAGTGGGGTTTTG	0	3	0	0
tsrna-06347	AGTTGATGCAGAGTGGGGTTTT	0	0	0	0
tsrna-06346	AGTTGATGCAGAGTGGGGTTT	0	1	0	0
tsrna-06345	AGTTGATGCAGAGTGGGGTT	0	0	0	0
tsrna-06336	AGTTGAAATACAACGATGGTTTTTCAT	1	0	0	0
tsrna-06335	AGTTCTTGTAGTTGAAATACAACG	4	7	2	4
tsrna-06334	AGTTCTGGTCTCCGGATGGAGGCGT	2	4	1	0
tsrna-06333	AGTTCTGGTCTCCGGATGGAGGCG	0	2	0	0
tsrna-06331	AGTTCTGGTCTCCGGATGGAGG	0	1	0	0
tsrna-06325	AGTTCGATCCTCGCTGGGGCCTCCA	5	13	5	3
tsrna-06324	AGTTCGATCCTCGCTGGGGCCTC	0	0	0	0
tsrna-06323	AGTTCGATCCTCGCTGGGGCCT	0	0	0	0
tsrna-06320	AGTTCGATCCTCACCTGGAGCACC	1	0	0	0
tsrna-06319	AGTTCGATCCTCACCTGGAGCA	0	1	0	0
tsrna-06316	AGTTCGATCCTCACACGGGGCACCA	0	3	1	0
tsrna-06311	AGTTCGAGTCTCGGTGGAACCTCCA	62	149	26	20
tsrna-06310	AGTTCGAGTCTCGGTGGAACC	0	0	0	0
tsrna-06309	AGTTCGAGTCTCGGTGGAAC	0	1	0	0
tsrna-06307	AGTTCGAGCCTCACCTGGAGCACCA	0	1	0	0
tsrna-06306	AGTTCGAGCCTCACCTGGAGCACC	0	1	0	0
tsrna-06296	AGTTCGAATCTCGGTGGGACCTC	0	0	0	0
tsrna-06294	AGTTCGAACCTCAGAGGGGGCACCA	2	7	0	0
tsrna-06290	AGTTCAATTCTCGCTGGGGCCTC	1	0	0	0
tsrna-06289	AGTTCAAGTCTCGGTGGAACCTCCA	62	102	18	12
tsrna-06288	AGTTCAAGTCTCGGTGGAACCTC	1	3	0	0
tsrna-06285	AGTTCAAATCTCGGTGGGACCTCCA	71	117	28	21
tsrna-06284	AGTTCAAATCTCGGTGGGACCTC	0	1	0	0
tsrna-06282	AGTTCAAATCTCGGTGGGACC	0	0	0	0
tsrna-06281	AGTTCAAATCTCGGTGGGAC	0	1	0	0
tsrna-06280	AGTTCAAATCTCGGTGGGA	0	0	1	0
tsrna-06278	AGTTCAAATCTCGGTGGAACCTCCA	158	175	59	31
tsrna-06277	AGTTCAAATCTCGGTGGAACCTCC	10	13	5	2
tsrna-06276	AGTTCAAATCTCGGTGGAACCTC	0	4	1	0
tsrna-06275	AGTTCAAATCTCGGTGGAACCT	0	0	0	0
tsrna-06274	AGTTCAAATCTCGGTGGAACC	1	2	0	0
tsrna-06268	AGTTCAAATCTCGCTGGGGCCTCCA	43	55	18	19
tsrna-06267	AGTTCAAATCTCGCTGGGGCCTCC	5	14	1	1
tsrna-06266	AGTTCAAATCTCGCTGGGGCCTC	0	0	0	0
tsrna-06262	AGTTCAAATCTCGCTGGG	0	0	0	0
tsrna-06261	AGCGGTTAGGATTCCTGGTTTTCAC	9	4	7	3
tsrna-06260	AGCGGTTAGGATTCCTGGTTTTCA	8	4	4	5
tsrna-06259	AGCGGTTAGGATTCCTGGTTTTC	13	3	0	0
tsrna-06258	AGCGGTTAGGATTCCTGGTTTT	1	3	1	1
tsrna-06257	AGCGGTTAGGATTCCTGGTTT	4	3	1	2
tsrna-06256	AGCGGTTAGGATTCCTGGTT	2	5	0	0
tsrna-06255	AGCGGTTAGGATTCCTGGT	2	7	1	0
tsrna-06254	AGCGGTTAGGATTCCTGG	4	2	1	1
tsrna-06253	AGCGGTTAGGATTCCTG	0	0	1	0
tsrna-06252	AGCGGTCTAAGGCGCTGGATT	2	1	1	1

tsrna-06250	AGCGGTCTAAGGCGCTGCGTTC	0	0	0	0
tsrna-06248	AGCGGTAGAGCATTTGACTGC	0	2	1	1
tsrna-06247	AGCGGTAGAGCATTTGACTG	0	0	1	0
tsrna-06242	AGCGGAAGCGTGCTGGGCCC	8	36	5	3
tsrna-06241	AGCGGAAGCGTGCTGGGCC	4	20	1	0
tsrna-06240	AGCGCGTTCGGCTGTTAACCGAAAGGTTGG	9	39	13	11
tsrna-06239	AGCGCGTTCGGCTGTTAACCGAAAGGT	9	17	7	0
tsrna-06238	AGCGCGTTCGGCTGTTAACCGAAAGG	9	16	5	2
tsrna-06237	AGCGCGTTCGGCTGTTAACCGAAAG	9	18	3	2
tsrna-06236	AGCGCGTTCGGCTGTTAACCGAAA	12	14	7	4
tsrna-06235	AGCGCGTTCGGCTGTTAACCGA	3	5	4	0
tsrna-06234	AGCGCGTTCGGCTGTTAACCG	5	15	2	0
tsrna-06233	AGCGCGTTCGGCTGTTAACC	1	0	1	0
tsrna-06227	AGCGCCGCTGGTGTAGTGGTATCATGCAAG	7	17	16	3
tsrna-06222	AGCGATCCGAGTTCAAATCTCGGTGGAACCTC	0	3	0	0
tsrna-06218	AGCCTCCGGAGCTGGGGATTGTGGGT	1	3	1	0
tsrna-06217	AGCCTCCGGAGCTGGGGATTGTGGG	0	1	0	0
tsrna-06215	AGCCTCACCTGGAGCACCA	1	1	1	0
tsrna-06214	AGCCGTGATCGTATAGTGGTTAGTACTCTGCGTTGT	33	14	16	5
tsrna-06213	AGCCGTGATCGTATAGTGGTTAGTACTCTGCGTTG	35	19	13	4
tsrna-06212	AGCCGTGATCGTATAGTGGTTAGTACTCTGCGTT	23	6	10	3
tsrna-06211	AGCCGTGATCGTATAGTGGTTAGTACTCTGCGT	2	3	3	1
tsrna-06210	AGCCGTGATCGTATAGTGGTTAGTACTCTGCG	5	3	2	1
tsrna-06209	AGCCGTGATCGTATAGTGGTTAGTACTCTGC	2	3	3	2
tsrna-06208	AGCCGTGATCGTATAGTGGTTAGTACTCTG	2	1	0	0
tsrna-06207	AGCCGTGATCGTATAGTGGTTAGTACTCT	5	0	0	0
tsrna-06205	AGCCGTGATCGTATAGTGGTTAGTACT	1	1	0	0
tsrna-06196	AGCCGTGATCGTATAGTG	0	0	0	0
tsrna-06194	AGCCCTCAGTAAGTTGCAATACTTAATTTCTGCCA	1	1	2	1
tsrna-06192	AGCCCTCAGTAAGTTGCAATACT	0	0	1	0
tsrna-06186	AGCCCGGTAATCGCATAAAACTTAAAACTT	3	1	3	0
tsrna-06181	AGCCCGGCTAGCTCAGTCGGTAGAGCATG	2	4	2	4
tsrna-06180	AGCCCGGCTAGCTCAGTCGGTAGAG	2	7	1	0
tsrna-06179	AGCCCGGCTAGCTCAGTCGG	1	1	0	3
tsrna-06178	AGCCCGGCTAGCTCAG	1	0	0	0
tsrna-06177	AGCCCGGATAGCTCAGTCGGTAGAGCATCAGACTTTT	1364	734	1469	185
tsrna-06176	AGCCCGGATAGCTCAGTCGGTAGAGCATCAGACTTT	1214	726	1400	196
tsrna-06175	AGCCCGGATAGCTCAGTCGGTAGAGCATC	17	5	15	3
tsrna-06174	AGCCCGGATAGCTCAGTCGGTAGAGCA	4	2	2	4
tsrna-06173	AGCCCGGATAGCTCAGTCGGTAGAG	4	2	3	4
tsrna-06172	AGCCCGGATAGCTCAGTCGGTAGA	3	6	5	5
tsrna-06171	AGCCCGGATAGCTCAGTCGGTAG	1	3	2	2
tsrna-06170	AGCCCGGATAGCTCAGTCGGTA	5	2	0	0
tsrna-06169	AGCCCGGATAGCTCAGTCGG	1	5	0	0
tsrna-06166	AGCCCCAGTGGAACCACCA	2	2	1	0
tsrna-06164	AGCCCCACGTTGGGCGCCA	7	33	5	5
tsrna-06163	AGCCCACCCAGGGACGCCA	3	50	1	1
tsrna-06161	AGCCAGGGATTGTGGGTTCGAGTCCCATCTGGGGTGCCA	7	22	4	0
tsrna-06160	AGCCAGGGATTGTGGGTTCGAGTCCCATCTGGGGTGCC	6	10	6	1
tsrna-06159	AGCCAGGGATTGTGGGTTCGAGTCCCACCCGGGGTA	6	9	4	2
tsrna-06158	AGCCAGGGATTGTGGGTTCGAGTCCC	5	10	1	0
tsrna-06157	AGCCAGGGATTGTGGGTTCG	0	8	3	1
tsrna-06156	AGCCAGGGATTGTGGGTTC	1	8	1	1
tsrna-06155	AGCCAGGGATTGTGGGTT	4	9	1	0
tsrna-06154	AGCCAGGGATTGTGGGT	1	9	1	0
tsrna-06150	AGCATTGGTGGTTCAGTGGTAGAATTCTCGCCT	4299	617	5159	344
tsrna-06149	AGCATTGGTGGTTCAGTGGTAGAATTCTCG	51	11	54	55
tsrna-06148	AGCATTGGTGGTTCAGTGGTAGAATTCTC	44	15	36	42
tsrna-06147	AGCATTGGTGGTTCAGTGGTAGAATTCT	15	21	17	30
tsrna-06146	AGCATTGGTGGTTCAGTGGTAGA	8	10	3	5
tsrna-06145	AGCATTGGTGGTTCAGTGGT	2	5	1	0
tsrna-06144	AGCATTGGACTGTAAATCTAAAGACAGGGGTTAGGC	0	0	0	0
tsrna-06143	AGCATTGGACTGTAAATCTAAAGACAGGGG	0	1	0	0
tsrna-06142	AGCATTGGACTGTAAATCTAAAGACAGG	1	0	0	0

tsrna-06141	AGCATTGGACTGTAAATCTAAAGACA	1	0	0	0
tsrna-06138	AGCATTGGACTGTAAATCTAAAG	0	1	0	0
tsrna-06136	AGCATTGGACTGTAAATCTAA	0	0	0	0
tsrna-06135	AGCATTGGACTGTAAATCTA	1	0	0	0
tsrna-06133	AGCATTGGACTGTAAATC	0	1	0	0
tsrna-06130	AGCATTAACCTTTTAAGTTAAAGATTAAGAGAACC	1	1	3	1
tsrna-06126	AGCATGGGTGGTTCAGTGGTAGAATTCTCG	22	20	25	35
tsrna-06125	AGCATGGGTGGTTCAGTGGTAGAATTCT	18	14	9	23
tsrna-06124	AGCATGGGTGGTTCAGTGGTAGA	5	6	2	3
tsrna-06123	AGCATGGGACTCTTAATCCC	1	1	0	0
tsrna-06122	AGCATGGGACTCTTAATCC	1	1	0	0
tsrna-06120	AGCATGGGACTCTTAAT	0	1	0	0
tsrna-06118	AGCATGCACGAGGCCCCGGGT	0	2	0	0
tsrna-06117	AGCATGAGACTCTTAATCT	3	2	0	3
tsrna-06116	AGCATAGCTGCCTTCCAAGCAGTTGACCCG	1	2	3	0
tsrna-06115	AGCATAGCTGCCTTCCAAG	0	0	1	0
tsrna-06114	AGCATAGCTGCCTTCCAA	0	0	0	0
tsrna-06112	AGCAGTTGACCCGGGTTCGATTCCCGGCCAACG	1	1	0	0
tsrna-06111	AGCAGTTGACCCGGGTTCGATTCCCGGCC	0	3	0	0
tsrna-06110	AGCAGTTGACCCGGGTTCGA	0	0	1	1
tsrna-06109	AGCAGTTGACCCGGGTTCG	1	1	1	0
tsrna-06108	AGCAGTTGACCCGGGTTC	0	3	1	0
tsrna-06107	AGCAGTTGACCCGGGTT	1	0	0	0
tsrna-06104	AGCAGAGTGGCGCAGCGGAAGCGTGCTGGGCCCATA	10	65	8	5
tsrna-06103	AGCAGAGTGGCGCAGCGGAAGCGTGCTGGGCCCAT	14	66	5	7
tsrna-06102	AGCAGAGTGGCGCAGCGGAAGCGTGCTGGGCCCA	6	70	7	7
tsrna-06101	AGCAGAGTGGCGCAGCGGAAGCGTGCTGGGCCC	8	50	4	7
tsrna-06100	AGCAGAGTGGCGCAGCGGAAGCGTGCTGGGCC	2	34	4	1
tsrna-06099	AGCAGAGTGGCGCAGCGGAAGCGTGCTGGGC	3	22	3	1
tsrna-06098	AGCAGAGTGGCGCAGCGGAAGCGTGCTGGG	3	18	1	1
tsrna-06097	AGCAGAGTGGCGCAGCGGAAGCGTGCTGG	1	3	0	1
tsrna-06095	AGCAGAGTGGCGCAGCGGAAGCGTGCT	0	1	0	0
tsrna-06082	AGCACTCTGGACTTTGAATCCAGC	2	2	3	0
tsrna-06081	AGCACTCTGGACTTTGAATCCAG	0	1	1	0
tsrna-06080	AGCACTCTGGACTTTGAATCCA	4	5	1	1
tsrna-06079	AGCACTCTGGACTTTGAATCC	3	2	3	0
tsrna-06078	AGCACTCTGGACTTTGAATC	2	4	1	1
tsrna-06073	AGCACTCTGGACTCTGAATCCAGCGATCCGAGTTCA	2	3	0	0
tsrna-06072	AGCACTCTGGACTCTGAATCCAGCGATCCGAGTTC	1	3	3	0
tsrna-06071	AGCACTCTGGACTCTGAATCCAGCGATCCGAGTT	0	6	0	0
tsrna-06070	AGCACTCTGGACTCTGAATCCAGCGATCCGAGT	2	4	1	0
tsrna-06069	AGCACTCTGGACTCTGAATCCAGCGATCCGAG	3	5	0	0
tsrna-06068	AGCACTCTGGACTCTGAATCCAGCGATCCG	1	1	1	0
tsrna-06067	AGCACTCTGGACTCTGAATCCAGCGATCC	3	4	0	1
tsrna-06066	AGCACTCTGGACTCTGAATCCAGCGATC	1	3	1	0
tsrna-06065	AGCACTCTGGACTCTGAATCCAGCGAT	1	1	0	0
tsrna-06064	AGCACTCTGGACTCTGAATCCAGCGA	1	3	2	1
tsrna-06063	AGCACTCTGGACTCTGAATCCAGCG	2	1	0	0
tsrna-06062	AGCACTCTGGACTCTGAATCCAGC	0	1	0	0
tsrna-06061	AGCACTCTGGACTCTGAATCCAG	2	5	2	0
tsrna-06060	AGCACTCTGGACTCTGAATCCA	1	4	1	0
tsrna-06059	AGCACTCTGGACTCTGAATCC	0	2	1	0
tsrna-06058	AGCACTCTGGACTCTGAATC	0	2	0	1
tsrna-06052	AGCACCCAACTTACACTTAGGAGATTTCAA	0	0	0	0
tsrna-06051	AGCACCCAACTTACACTTAGGAGATTTCA	0	0	1	0
tsrna-06049	AGCACCCAACTTACACTTAGGAGATTT	0	0	0	0
tsrna-06047	AGCACCCAACTTACACTTAGGAGAT	0	0	0	0
tsrna-06046	AGCACCCAACTTACACTTAGGAGA	0	0	0	0
tsrna-06037	AGCAATACACTGAAAATGTTTAGACGGGCTC	6	11	2	0
tsrna-06036	AGCAATACACTGAAAATGTTTAGACGGGCT	2	4	2	0
tsrna-06035	AGATTTCAACTTAACTTGACCGCTCTGACCA	70	46	54	17
tsrna-06034	AGATTTCAACTTAACTTGACCGCTCTGACC	15	7	7	0
tsrna-06033	AGATTTCAACTTAACTTGACCGCTCTGAC	2	5	1	0
tsrna-06032	AGATTTCAACTTAACTTGACCGCTCTGA	2	1	0	0

tsrna-06031	AGATTTCAACTTAACTTGACCGCTCTG	0	2	0	0
tsrna-06030	AGATTTCAACTTAACTTGACCGCTCT	0	0	0	0
tsrna-06029	AGATTTCAACTTAACTTGACCGCTC	1	0	0	0
tsrna-06028	AGATTTCAACTTAACTTGACCGCT	0	0	0	0
tsrna-06024	AGATTTCAACTTAACTTGAC	0	1	0	0
tsrna-06020	AGATTGTGAATCTGACAACAGAGGCTTACGACCCCTTATTTAC	27	11	62	1
tsrna-06019	AGATTGTGAATCTGACAACAGAGGCTTACGACCCCTTATTTAC	32	18	72	1
tsrna-06018	AGATTGTGAATCTGACAACAGAGGCTTACGACCCCTTATTTAC	42	22	80	0
tsrna-06017	AGATTGTGAATCTGACAACAGAGGCTTACGACCCCTTATTTAC	30	15	61	1
tsrna-06016	AGATTGTGAATCTGACAACAGAGGCTTACGACCCCTT	8	11	12	1
tsrna-06015	AGATTGTGAATCTGACAACAGAGGCTTACGACCCCT	4	8	8	1
tsrna-06014	AGATTGTGAATCTGACAACAGAGGCTTACGACCCC	1	2	5	0
tsrna-06013	AGATTGTGAATCTGACAACAGAGGCTTACGACCC	2	6	3	0
tsrna-06012	AGATTGTGAATCTGACAACAGAGGCTTACGACC	3	4	4	0
tsrna-06011	AGATTGTGAATCTGACAACAGAGGCTTACGA	3	5	3	1
tsrna-06010	AGATTGTGAATCTGACAACAGAGGCTTACG	4	3	4	1
tsrna-06009	AGATTGTGAATCTGACAACAGAGGCTTAC	1	4	6	1
tsrna-06008	AGATTGTGAATCTGACAACAGAGGCTT	0	6	3	0
tsrna-06007	AGATTGTGAATCTGACAACAGAGGCT	3	4	7	0
tsrna-06006	AGATTGTGAATCTGACAACAGAGGC	2	6	3	0
tsrna-06005	AGATTGTGAATCTGACAACAGAGG	1	0	3	0
tsrna-06004	AGATTGTGAATCTGACAACAGAG	2	4	2	0
tsrna-06003	AGATTGTGAATCTGACAACAGA	2	3	1	0
tsrna-06002	AGATTGTGAATCTGACAACAG	0	0	1	2
tsrna-06001	AGATTGTGAATCTGACAACA	1	2	1	0
tsrna-06000	AGATTGTGAATCTGACAAC	1	3	3	0
tsrna-05999	AGATTGTGAATCTGACAA	0	0	1	0
tsrna-05998	AGATTGTGAATCTGACA	0	0	1	0
tsrna-05996	AGATTGCAGGTTCGAGTCCTGCCGCGGTCGCC	0	1	0	0
tsrna-05994	AGATTGAGGGTTCGAGTCCCTTCGTGGTCGCCA	0	3	1	0
tsrna-05993	AGATTGAGGGTTCGAGTCCCTTCGTGGTCGCC	0	3	1	0
tsrna-05992	AGATTGAGGGTTCGAGTCCCTTCGTGGTCGC	0	0	0	0
tsrna-05991	AGATTGAGGGTTCGAGTCCCTTCGTGGTCG	0	1	0	0
tsrna-05990	AGATTGAGGGTTCGAGTC	1	0	1	0
tsrna-05988	AGATTGAAGCCAGTTGATTAGGGTGCTTAG	0	0	1	0
tsrna-05987	AGATTGAAGCCAGTTGATTAGGG	2	0	1	0
tsrna-05985	AGATTCTAGGTTCGACTCCTGGCTGGCTCG	0	1	0	0
tsrna-05984	AGATTCTAGGTTCGACTCCTG	0	3	0	0
tsrna-05983	AGATTCTAGGTTCGACTCC	0	3	0	0
tsrna-05982	AGATTCCCATTCTTGCGACCCGGGTTCG	3	12	1	1
tsrna-05981	AGATTCCCATTCTTGCGACCCGGGT	0	5	1	0
tsrna-05980	AGATTCCCATTCTTGCGACCCGGG	0	4	0	0
tsrna-05979	AGATTCCCATTCTTGCGACCCGG	0	0	0	0
tsrna-05978	AGATTCCCATTCTTGCGACCCG	0	1	0	0
tsrna-05977	AGATTCCCATTCTTGCGACCC	1	1	0	0
tsrna-05976	AGATTCCCATTCTTGCGACC	1	1	0	0
tsrna-05975	AGATTCCCATTCTTGCGAC	0	3	0	0
tsrna-05973	AGATTCCAGGTTCGACTCCTGGC	1	0	0	0
tsrna-05967	AGATTAAGAGAACCAACACCTCTTTACAGTGACCA	0	1	1	1
tsrna-05966	AGATTAAGAGAACCAACACCTCTTTACAGTGACC	1	1	0	0
tsrna-05965	AGATTAAGAGAACCAACACCTCTTTACAGTGAC	0	1	0	0
tsrna-05964	AGATTAAGAGAACCAACACCTCTTTACAGT	0	1	0	0
tsrna-05963	AGATTAAGAGAACCAACACCTCTTTACAG	0	1	0	0
tsrna-05960	AGATTAAGAGAACCAACACCTCTTTA	0	0	0	0
tsrna-05959	AGATTAAGAGAACCAACACCTCT	0	0	0	0
tsrna-05958	AGATTAAGAGAACCAACACCTC	0	0	0	0
tsrna-05956	AGATTAAGAGAACCAACACC	0	0	1	0
tsrna-05949	AGATGGCAGAGCCCGGTAATCG	0	0	0	0
tsrna-05945	AGATGAAAACCTTTTTCCAAGGACACCA	6	1	2	0
tsrna-05944	AGATGAAAACCTTTTTCCAAGGACACC	0	0	0	0
tsrna-05939	AGATCTAAAGGTCCCTGGT	11	14	7	1
tsrna-05937	AGATCCTTAGGTCGCTGGT	0	2	0	0
tsrna-05933	AGATCAGAAGGTTGCGTGTTCAAGTCACGTCGGGGTCACC	5	3	1	1
tsrna-05932	AGATCAGAAGGCTGCGTGTTCGAA	0	0	1	0

tsrna-05931	AGATCAGAAGGCTGCGTGTTCGA	0	1	0	0
tsrna-05930	AGATCAGAAGGCTGCGTGTTCG	0	1	0	0
tsrna-05929	AGATCAGAAGGCTGCGTGTTC	1	0	1	0
tsrna-05928	AGATCAGAAGGCTGCGTGTT	0	0	1	0
tsrna-05927	AGATCAGAAGGCTGCGTGT	1	0	0	0
tsrna-05925	AGATCAAGAGGTCCCTGGTT	3	6	0	4
tsrna-05924	AGATCAAGAGGTCCCTGGT	2	7	1	1
tsrna-05923	AGATCAAGAGGTCCCCGGTTCA	2	4	1	0
tsrna-05922	AGATCAAGAGGTCCCCGGTTC	2	3	0	0
tsrna-05921	AGATCAAGAGGTCCCCGGTT	2	6	0	1
tsrna-05920	AGATCAAGAGGTCCCCGGT	2	6	2	0
tsrna-05914	AGATCAAAAGGTCCCTGGT	5	8	3	2
tsrna-05912	AGAGTTCAAGTCTCACTGGG	0	1	0	0
tsrna-05909	AGAGTGTAGCTTAACACAAAGCACCCAACT	0	1	0	0
tsrna-05908	AGAGTGTAGCTTAACACA	0	0	0	0
tsrna-05907	AGAGTGGGGTTTTGCAGTCCTTACCA	1	1	1	0
tsrna-05906	AGAGTGGGGTTTTGCAGTCCTTACC	0	0	0	1
tsrna-05905	AGAGTGGGGTTTTGCAGTCCT	0	0	0	0
tsrna-05904	AGAGTGGGGTTTTGCAGTCC	0	0	0	0
tsrna-05902	AGAGTGGGGTTTTGCAGT	0	0	1	0
tsrna-05899	AGAGTGGCGCAGCGGAAGCGTGCTGGGCCC	8	45	6	6
tsrna-05898	AGAGTGGCGCAGCGGAAGCGTGCTGGGCC	2	31	2	1
tsrna-05897	AGAGTGGCGCAGCGGAAGCGTGCTGGGC	3	19	1	1
tsrna-05896	AGAGTGGCGCAGCGGAAGCGTGCTGGG	2	21	0	1
tsrna-05895	AGAGTGGCGCAGCGGAAGCGTGCTGG	1	4	0	0
tsrna-05887	AGAGTAAATAATAGGAGCTT	0	0	0	0
tsrna-05886	AGAGTAAATAATAGGAGCT	0	0	0	0
tsrna-05880	AGAGGTCGATGGATCGAAAC	0	1	0	0
tsrna-05876	AGAGGTCCCGGGTTCAAATCCC	0	1	0	1
tsrna-05875	AGAGGTCCCGGGTTCAAATC	0	1	0	0
tsrna-05874	AGAGGTCCCGGGTTCAAA	0	0	0	0
tsrna-05872	AGAGGCTTACGACCCCTTATTTACCCCA	2	2	1	0
tsrna-05871	AGAGGCTTACGACCCCTTATTTACCCC	0	0	0	0
tsrna-05863	AGAGCCTTCAAAGCCCTCAGT	0	1	0	0
tsrna-05860	AGAGCCCGGTAATCGCATAAAACTTAAAAC	1	0	1	0
tsrna-05859	AGAGCCCGGTAATCGCATAAAACTTA	1	0	0	0
tsrna-05856	AGAGCCCGGTAATCGCATAA	0	0	1	0
tsrna-05851	AGAGCATGGGACTCTTAATCCCAG	2	1	1	1
tsrna-05850	AGAGCATGGGACTCTTAATC	0	0	1	0
tsrna-05849	AGAGCATGGGACTCTT	0	0	0	1
tsrna-05848	AGAGAATAGTTTAAATTAGAATCTTAGCTT	1	2	2	1
tsrna-05846	AGAGAACCAACACCTCTTTACAGTGACCA	1	1	0	0
tsrna-05836	AGACTTTTTCTCTGACCA	2	8	7	2
tsrna-05835	AGACTTTTTCTCTGACC	0	2	0	0
tsrna-05834	AGACTTTTTCTCTGAC	1	0	0	0
tsrna-05833	AGACTTTTAATCTGAGGGTCCAGGGT	17	45	9	4
tsrna-05832	AGACTTTTAATCTGAGGGTCCAG	10	35	9	4
tsrna-05831	AGACTTTTAATCTGAGGGTCC	5	16	5	3
tsrna-05830	AGACTGAAGATCTAAAGGTCCCTGGT	13	16	6	3
tsrna-05829	AGACTCAAGTTCTGGTCTCCGGA	2	5	1	0
tsrna-05828	AGACTCAAGTTCTGGTCTCCGG	2	4	1	0
tsrna-05827	AGACTCAAGTTCTGGTCTCC	0	3	0	0
tsrna-05826	AGACGGGCTCACATCACCCCATAAACACCA	18	7	5	15
tsrna-05825	AGACGGGCTCACATCACCCCATAAACACC	6	0	2	0
tsrna-05824	AGACGGGCTCACATCACCCCATAAACA	1	0	1	0
tsrna-05823	AGACGGGCTCACATCACCCCATA	0	0	1	0
tsrna-05822	AGACGGGCTCACATCACCCC	0	0	1	0
tsrna-05819	AGACGGGCTCACATCAC	1	0	0	0
tsrna-05817	AGACCTCGTGGCGCAATGG	1	0	0	0
tsrna-05815	AGACCGGGGTTCGATTCCCCGACGGGGAGCCA	0	14	0	0
tsrna-05814	AGACCGGGGTTCGATTCCCCGACGGGGAGCC	0	3	0	0
tsrna-05813	AGACCGGGGTTCGATTCCCCGACGGGGAGC	0	1	0	0
tsrna-05812	AGACCGGGGTTCGATTCCCCGACGGGGAG	0	0	0	1
tsrna-05809	AGACCGGGGTTCGATTCCCCGACGGG	0	0	0	0

tsrna-05798	AGACCAAGAGCCTTCAAAGC	0	0	1	0
tsrna-05797	AGACCAAGAGCCTTCAAAG	1	1	0	0
tsrna-05795	AGACAGGGGTTAGGCCTCTTTTTACCACC	1	0	0	0
tsrna-05794	AGACAGGGGTTAGGCCTCTTTTTACCAC	0	0	1	0
tsrna-05793	AGACAGGGGTTAGGCCTCTTTTTACCA	0	1	1	0
tsrna-05792	AGACAGGGGTTAGGCCTCTTTTTACC	0	0	1	0
tsrna-05791	AGACAGGGGTTAGGCCTCTTT	1	0	0	0
tsrna-05790	AGACAGGGGTTAGGCCTCTT	0	0	0	0
tsrna-05789	AGACAGGGGTTAGGCCTCT	1	0	0	0
tsrna-05788	AGAATTCTCGCCTGCCACGCGGGAGGCCCG	12	13	1	1
tsrna-05787	AGAATTCTCGCCTGCCACGCGGG	8	14	0	1
tsrna-05786	AGAATTCTCGCCTGCCACGCGG	5	12	3	2
tsrna-05785	AGAATTCTCGCCTGCCACGCG	4	14	3	1
tsrna-05784	AGAATTCTCGCCTGCCACGC	1	12	2	1
tsrna-05783	AGAATTCTCGCCTGCCACG	6	6	6	1
tsrna-05782	AGAATTCTCGCCTGCCAC	1	1	0	0
tsrna-05781	AGAATTCTCGCCTGCCA	4	0	1	0
tsrna-05779	AGAATTCTCGCCTCCCACG	0	0	0	1
tsrna-05778	AGAATGGCCGAGTGGTTAAGGCGTTGGACTTAA	1	0	0	0
tsrna-05775	AGAATCTTAGCTTTGGGTGCTAATGGTGGAGTTAAAGACT	5	5	2	2
tsrna-05774	AGAATCTTAGCTTTGGGTGCTAATGGTGGAGTTAAA	5	0	2	1
tsrna-05773	AGAATCTTAGCTTTGGGTGCTAATGGTGG	0	1	0	0
tsrna-05770	AGAATCTTAGCTTTGGGTGC	0	0	0	0
tsrna-05766	AGAATAGTTTAAATTAGAATCTTAGCTTTG	5	3	2	2
tsrna-05765	AGAAGGTTGCGTGTTCA	0	2	0	1
tsrna-05764	AGAAGGTCCTGGGTTCGAGCCCCAGTGGAACCACCA	38	31	17	16
tsrna-05763	AGAAGGTCCTGGGTTCGAGCCCCAGTGGA	2	0	0	0
tsrna-05761	AGAAGGCTGCGTGTTCG	1	0	0	0
tsrna-05759	AGAAGCAGCTTCAAACCTGCCGGGGCTTCC	0	0	1	0
tsrna-05758	AGAAGCAGCTTCAAACCTGCCGGGGCTT	0	0	0	0
tsrna-05757	AGAAGCAGCTTCAAACCTGCCGGGGCT	0	0	0	0
tsrna-05755	AGAAGCAGCTTCAAACCTGCCGGGG	0	1	0	0
tsrna-05752	AGAAGATTGCAGGTTCGAGTCCTGCCGCGGTCGCCA	7	7	1	0
tsrna-05749	AGAAGATTGAGGGTTCGAGTCCCTTCGTGGTCGCCA	4	17	3	4
tsrna-05748	AGAAGATTGAGGGTTCGAGTCCCTTCGTGGTCGCC	3	7	5	1
tsrna-05747	AGAAGATTGAGGGTTCGAGTCCCTTCGT	5	5	1	1
tsrna-05746	AGAAGATTGAGGGTTCG	1	3	2	2
tsrna-05745	AGAAGATTGAGGGTTC	4	7	0	0
tsrna-05744	AGAAGATTCTAGGTTCGACTCCTG	1	7	1	1
tsrna-05743	AGAAGATTCTAGGTTC	1	1	1	0
tsrna-05742	AGAAGATTCCAGGTTCGACTCCTGG	3	2	0	0
tsrna-05741	AGAAGATTCCAGGTTCG	0	3	0	0
tsrna-05740	AGAAGATTCCAGGTTC	1	1	0	0
tsrna-05739	AGAACTGCTAACTCATGCCCCCATGTCTAACAACATGGCTTTC	3	1	8	1
tsrna-05738	AGAACTGCTAACTCATGCCCCCATGTCTAACAACATGGCTTTC	2	1	7	0
tsrna-05737	AGAACTGCTAACTCATGCCCCCATGTCTAACAACATGGCT	1	1	2	0
tsrna-05736	AGAACTGCTAACTCATGCCCCCATGTCTAACAACATGGC	1	0	2	0
tsrna-05735	AGAACTGCTAACTCATGCCCCCATGTCTAACAACATGG	0	1	3	0
tsrna-05734	AGAACTGCTAACTCATGCCCCCATGTCTAACAACAT	0	1	0	0
tsrna-05733	AGAACTGCTAACTCATGCCCCCATGTCTAACAACA	0	0	1	0
tsrna-05732	AGAACTGCTAACTCATGCCCCCATGTCTAACAA	0	0	1	0
tsrna-05731	AGAACTGCTAACTCATGCCCCCATGTCTAACA	1	0	0	1
tsrna-05730	AGAACTGCTAACTCATGCCCCCATGTCTAAC	0	0	0	0
tsrna-05729	AGAACTGCTAACTCATGCCCCCATGTCTAA	1	1	0	0
tsrna-05728	AGAACTGCTAACTCATGCCCCCATGTCTA	0	0	0	1
tsrna-05727	AGAACTGCTAACTCATGCCCCCATGTCT	0	0	1	0
tsrna-05726	AGAACTGCTAACTCATGCCCCCATGTC	1	1	1	0
tsrna-05725	AGAACTGCTAACTCATGCCCCCATGT	0	1	0	0
tsrna-05724	AGAACTGCTAACTCATGCCCCCATG	0	1	0	0
tsrna-05723	AGAACTGCTAACTCATGCCCCCAT	0	0	0	0
tsrna-05721	AGAACTGCTAACTCATGCCCCC	0	0	0	0
tsrna-05720	AGAACTGCTAACTCATGCCCC	0	0	0	0
tsrna-05717	AGAACTGCTAACTCATGC	0	1	0	0
tsrna-05710	AGAACCAACACCTCTTTACAGTG	0	0	0	0

tsrna-05705	AGAAATTTAGGTTAAATACAGACCAAGAGC	1	8	2	2
tsrna-05704	AGAAATTTAGGTTAAATACAGACCAAGA	0	9	0	1
tsrna-05703	AGAAATTTAGGTTAAATACAGACCAAG	1	7	0	0
tsrna-05702	AGAAATTTAGGTTAAATACAGACC	1	4	0	0
tsrna-05701	AGAAATTTAGGTTAAATACAGAC	3	7	0	0
tsrna-05700	AGAAATTTAGGTTAAATACAG	0	6	0	2
tsrna-05699	AGAAATTTAGGTTAAATACA	1	8	0	0
tsrna-05698	AGAAATTTAGGTTAAATAC	0	12	0	2
tsrna-05695	AGAAATATGTCTGATAAAAGAGTTACTTTGATAGAGTAAATAAT	14	20	18	1
tsrna-05694	AGAAATATGTCTGATAAAAGAGTTACTTTGATAGAG	5	8	8	0
tsrna-05693	AGAAATATGTCTGATAAAAGAGTTACTTTGAT	2	2	2	0
tsrna-05692	AGAAATATGTCTGATAAAAGAGTTACTTTG	0	2	3	0
tsrna-05690	AGAAATATGTCTGATAAAAGAGTTA	0	3	2	0
tsrna-05689	AGAAATATGTCTGATAAAAGAGT	0	0	0	0
tsrna-05681	AGAAAGCTCACAAGAACTGCTAACTCATGCCCCCATGTCTAAC	18	41	12	11
tsrna-05680	AGAAAGCTCACAAGAACTGCTAACTCATGCCCCCATGTCTAAC	17	38	15	10
tsrna-05679	AGAAAGCTCACAAGAACTGCTAACTCATGCCCCCAT	16	35	15	9
tsrna-05678	AGAAAGCTCACAAGAACTGCTAACTCATGCC	11	22	9	5
tsrna-05677	AGAAAGCTCACAAGAACTGCTAACTCATGC	19	23	10	8
tsrna-05676	AGAAAGCTCACAAGAACTGCTAACTCATG	7	35	15	10
tsrna-05675	AGAAAGCTCACAAGAACTGCTAACTCA	10	24	9	7
tsrna-05674	AGAAAGCTCACAAGAACTGCTAACT	10	28	11	7
tsrna-05673	AGAAAGCTCACAAGAACTGCTAAC	11	29	6	8
tsrna-05672	AGAAAGCTCACAAGAACTGCTAA	4	4	6	1
tsrna-05671	AGAAAGCTCACAAGAACTGCTA	5	14	4	5
tsrna-05670	AGAAAGCTCACAAGAACTGCT	5	8	4	0
tsrna-05669	AGAAAGCTCACAAGAACTGC	1	5	0	0
tsrna-05668	AGAAAGCTCACAAGAACTG	0	0	2	0
tsrna-05667	AGAAAGCTCACAAGAACT	0	3	0	1
tsrna-05665	AGAAAAAGTCATGGAGGCCA	19	89	7	8
tsrna-05664	ACTTTTTCTCTGACCA	1	1	2	1
tsrna-05663	ACTTTTAATCTGAGGGTCCAGGGTTCAAGTCCCTGTTCGGGC	22	90	19	8
tsrna-05662	ACTTTTAATCTGAGGGTCCAGGGT	14	48	7	4
tsrna-05661	ACTTTTAATCTGAGGGTCCAG	8	30	4	3
tsrna-05660	ACTTTTAATCTGAGGGTCCA	15	27	8	3
tsrna-05659	ACTTTTAATCTGAGGGTCC	9	16	4	0
tsrna-05658	ACTTTTAAAGGATAACAGCTATCCATTGGTCTTAGGCCCCA	7	1	9	1
tsrna-05657	ACTTTTAAAGGATAACAGCTATCCATTGGTCTTAGGC	0	0	1	1
tsrna-05656	ACTTTTAAAGGATAACAGCTATCCATTGGTCTTAGG	2	1	1	2
tsrna-05655	ACTTTTAAAGGATAACAGCTATCCATTGGTCTT	0	1	2	3
tsrna-05654	ACTTTTAAAGGATAACAGCTATCCATTGGTCT	0	0	2	2
tsrna-05653	ACTTTTAAAGGATAACAGCTATCCATTGGTC	1	0	0	0
tsrna-05652	ACTTTTAAAGGATAACAGCTATCCATTGGT	2	0	0	3
tsrna-05651	ACTTTTAAAGGATAACAGCTATCCATTGG	0	0	1	1
tsrna-05650	ACTTTTAAAGGATAACAGCTATCCATTG	0	2	0	0
tsrna-05649	ACTTTTAAAGGATAACAGCTATCCATT	0	1	0	2
tsrna-05648	ACTTTTAAAGGATAACAGCTATCCA	1	1	0	1
tsrna-05647	ACTTTTAAAGGATAACAGCTATCC	2	0	1	1
tsrna-05646	ACTTTTAAAGGATAACAGCTAT	0	0	1	0
tsrna-05645	ACTTTTAAAGGATAACAGCTA	0	0	2	1
tsrna-05644	ACTTTTAAAGGATAACAGCT	0	0	2	2
tsrna-05639	ACTTTGTCAAAGTTAAATTATAGGCTAAATCCTATATATCTTAC	18	2	27	0
tsrna-05638	ACTTTGTCAAAGTTAAATTATAGGCTAA	4	1	4	1
tsrna-05637	ACTTTGTCAAAGTTAAATTATAGGCT	3	1	8	0
tsrna-05636	ACTTTGTCAAAGTTAAATTATAGGC	3	1	3	0
tsrna-05635	ACTTTGTCAAAGTTAAATTATAGG	0	2	2	0
tsrna-05634	ACTTTGTCAAAGTTAAATTATAG	1	0	1	0
tsrna-05633	ACTTTGTCAAAGTTAAATT	0	0	1	0
tsrna-05630	ACTTTGATAGAGTAAATAATAGGAGCTTAAACCCCCT	7	2	4	0
tsrna-05629	ACTTTGATAGAGTAAATAATAGGAGCTTAAACCCCC	7	0	2	0
tsrna-05628	ACTTTGATAGAGTAAATAATAGGAGCTTAAACCCC	4	0	1	1
tsrna-05627	ACTTTGATAGAGTAAATAATAGGAGCTTAAACCC	1	0	4	1
tsrna-05626	ACTTTGATAGAGTAAATAATAGGAGCTTAAACC	1	0	2	1
tsrna-05625	ACTTTGATAGAGTAAATAATAGGAGCTTA	1	1	0	0

tsrna-05624	ACTTTGATAGAGTAAATAATAGGAGCTT	0	1	0	0
tsrna-05623	ACTTTGATAGAGTAAATAATAGGAGCT	0	0	2	1
tsrna-05622	ACTTTGATAGAGTAAATAATAGGAGC	2	0	0	0
tsrna-05618	ACTTTGAATCCAGCGATCCGAGTTCAA	4	0	3	0
tsrna-05617	ACTTTGAATCCAGCGATCCGAGTTC	0	2	3	0
tsrna-05616	ACTTTGAATCCAGCGATCCGAGTT	3	6	0	0
tsrna-05615	ACTTTGAATCCAGCGATCCGAGT	0	1	1	1
tsrna-05614	ACTTTGAATCCAGCGATCCGAG	1	0	0	0
tsrna-05613	ACTTTGAATCCAGCGATCCGA	5	5	0	0
tsrna-05612	ACTTTGAATCCAGCGATCCG	0	0	1	0
tsrna-05611	ACTTTGAATCCAGCGATCC	0	1	1	0
tsrna-05610	ACTTTGAATCCAGCGATC	2	0	2	2
tsrna-05609	ACTTTGAATCCAGCGA	1	0	2	0
tsrna-05608	ACTTTGAATCCAGCAATCCGAGT	3	14	6	8
tsrna-05607	ACTTTGAATCCAGCAATCCGAG	2	0	2	1
tsrna-05606	ACTTTGAATCCAGCAATCCGA	0	2	1	1
tsrna-05605	ACTTTGAATCCAGCAATCCG	3	4	2	1
tsrna-05603	ACTTTACAGTCAGAGGTTC	1	0	0	0
tsrna-05602	ACTTTACAGTCAGAGGTT	0	1	0	0
tsrna-05600	ACTTGACCGCTCTGACCA	6	3	0	1
tsrna-05597	ACTTCTAATTCAAAGGTTCCGGGT	0	2	2	0
tsrna-05593	ACTTCGGATCAGAAGATTGAGGGT	2	5	1	2
tsrna-05587	ACTTATAATGCCGAGGTTGTGAGT	16	17	10	10
tsrna-05586	ACTTAGGAGATTTCAACTTAACTTGACCGCTCTGACCA	85	61	76	17
tsrna-05585	ACTTAGGAGATTTCAACTTAACTTGACCGCTCTGACC	16	9	11	2
tsrna-05584	ACTTAGGAGATTTCAACTTAACTTGACCGCTCTGAC	4	6	0	1
tsrna-05583	ACTTAGGAGATTTCAACTTAACTTGACCGCTCTGA	1	1	3	3
tsrna-05582	ACTTAGGAGATTTCAACTTAACTTGACCGCTCTG	2	2	1	0
tsrna-05581	ACTTAGGAGATTTCAACTTAACTTGACCGCT	1	0	0	0
tsrna-05580	ACTTAGGAGATTTCAACTTAACTTGACCGC	0	0	1	0
tsrna-05579	ACTTAGGAGATTTCAACTTAACTTGACCG	1	3	0	0
tsrna-05578	ACTTAGGAGATTTCAACTTAACTTGACC	0	2	0	0
tsrna-05576	ACTTAGGAGATTTCAACTTAACT	0	0	0	0
tsrna-05569	ACTTACACTTAGGAGATTTCAACTTAACTTGACCGCTCTGACC	76	55	86	15
tsrna-05568	ACTTACACTTAGGAGATTTCAACTTAACTTGACCGCTCTGACC	14	12	16	1
tsrna-05567	ACTTACACTTAGGAGATTTCAACTTAACTTGACCGCTCTGAC	6	3	12	3
tsrna-05566	ACTTACACTTAGGAGATTTCAACTTAACTTGACCGCTCTGA	2	3	5	0
tsrna-05565	ACTTACACTTAGGAGATTTCAACTTAACTTGACCGCTCTG	2	1	4	0
tsrna-05564	ACTTACACTTAGGAGATTTCAACTTAACTTGACCGCTCT	4	1	3	0
tsrna-05563	ACTTACACTTAGGAGATTTCAACTTAACTTGACCGCTC	0	0	5	0
tsrna-05562	ACTTACACTTAGGAGATTTCAACTTAACTTGACCGCT	3	0	7	0
tsrna-05561	ACTTACACTTAGGAGATTTCAACTTAACTTGACCGC	3	0	2	0
tsrna-05560	ACTTACACTTAGGAGATTTCAACTTAACTTGACCG	1	1	4	0
tsrna-05559	ACTTACACTTAGGAGATTTCAACTTAACTTGACC	2	0	2	0
tsrna-05558	ACTTACACTTAGGAGATTTCAACTTAACTT	2	0	1	0
tsrna-05557	ACTTACACTTAGGAGATTTCAACTTAACT	0	0	0	0
tsrna-05556	ACTTACACTTAGGAGATTTCAACTTA	0	0	0	0
tsrna-05555	ACTTACACTTAGGAGATTTCAACTT	0	0	0	0
tsrna-05554	ACTTACACTTAGGAGATTTCAACT	0	0	0	0
tsrna-05545	ACTTAAGATCCAATGGACATATGT	0	0	0	0
tsrna-05539	ACTTAACTTGACCGCTCTGACCA	9	18	19	3
tsrna-05538	ACTTAACTTGACCGCTCTGACC	2	3	1	0
tsrna-05537	ACTTAACTTGACCGCTCTGAC	0	1	1	0
tsrna-05536	ACTTAACTTGACCGCTCTGA	0	1	0	0
tsrna-05535	ACTTAACTTGACCGCTCTG	0	0	0	0
tsrna-05534	ACTTAACTTGACCGCTCT	0	0	0	0
tsrna-05531	ACTTAAAACTTTACAGTCAGAGGTTC	0	1	1	0
tsrna-05528	ACTTAAAACTTTACAGTCAGAG	0	1	0	0
tsrna-05522	ACTGTAGATCCTTAGGTCGCTGGTTCGATT	2	1	1	1
tsrna-05521	ACTGTAGATCCTTAGGTCGCTGGTT	0	0	0	1
tsrna-05520	ACTGTAGATCCTTAGGTCGCTGGT	0	1	0	0
tsrna-05514	ACTGTAAATCTAAAGACAGGGGTTAGGCCT	2	0	1	0
tsrna-05513	ACTGTAAATCTAAAGACAGGGGTTAGGC	0	0	0	0
tsrna-05502	ACTGTAAAGCTAACTTAGCATTAACC	0	0	0	0

tsrna-05501	ACTGTAAAGCTAACTTAGCATTAAC	0	0	0	1
tsrna-05499	ACTGTAAAGCTAACTTAGCATT	0	1	0	0
tsrna-05498	ACTGTAAAGCTAACTTAGCA	0	0	0	0
tsrna-05493	ACTGGTGGTTCAGTGGTAGAATTCTCGCCT	7	8	1	3
tsrna-05492	ACTGGTGGTTCAGTGGTAGAATTCTCGC	4	4	2	0
tsrna-05491	ACTGGTGGTTCAGTGGTAGAATTCTCG	4	5	0	0
tsrna-05490	ACTGGTGGTTCAGTGGTAGAATTCTC	3	3	2	0
tsrna-05489	ACTGGTGGTTCAGTGGTAGAATTCT	0	4	1	1
tsrna-05488	ACTGGGCGGAAACACCA	1	3	1	0
tsrna-05486	ACTGCTAACTCATGCCCCCATGTCTAACAACATGGCTTTCTCA	13	5	43	0
tsrna-05485	ACTGCTAACTCATGCCCCCATGTCTAACAACATGGCTTTCTCA	11	6	25	1
tsrna-05484	ACTGCTAACTCATGCCCCCATGTCTAACAACATGGCT	0	1	0	0
tsrna-05483	ACTGCTAACTCATGCCCCCATGTCTAACAA	0	0	1	0
tsrna-05481	ACTGCTAACTCATGCCCCCATGTCTAAC	0	1	0	0
tsrna-05478	ACTGCTAACTCATGCCCCCATGT	0	1	0	0
tsrna-05475	ACTGCTAACTCATGCCCCCA	0	0	0	0
tsrna-05470	ACTGCAGATCAAGAGGTCCCCGGT	0	3	0	1
tsrna-05466	ACTGAAGATCTAAAGGTCCCTGGT	9	21	8	0
tsrna-05464	ACTGAAAATGTTTAGACGGGCTCACATCAC	72	32	37	3
tsrna-05463	ACTGAAAATGTTTAGACGGGCTCACATCA	52	40	29	11
tsrna-05462	ACTGAAAATGTTTAGACGGGCTCACA	37	23	14	5
tsrna-05461	ACTGAAAATGTTTAGACGGGCTCAC	30	21	5	3
tsrna-05460	ACTGAAAATGTTTAGACGGGCTCA	11	12	0	2
tsrna-05459	ACTGAAAATGTTTAGACGGGCTC	10	10	5	0
tsrna-05458	ACTGAAAATGTTTAGACGGGCT	1	3	0	1
tsrna-05457	ACTGAAAATGTTTAGACGGGC	1	0	0	0
tsrna-05456	ACTGAAAATGTTTAGACGGG	1	1	0	0
tsrna-05455	ACTGAAAATGTTTAGACGG	0	2	0	0
tsrna-05453	ACTCTTTTAGTATAAATAGTACCGTTAACTTCC	3	0	7	0
tsrna-05452	ACTCTTTTAGTATAAATAGTACCGTTAACT	0	0	2	0
tsrna-05440	ACTCTTAATCTCAGGGTCGTGGGT	6	11	2	4
tsrna-05439	ACTCTTAATCTCAGGGTCGTGG	1	9	2	2
tsrna-05438	ACTCTTAATCTCAGGGTCGTG	3	7	2	3
tsrna-05436	ACTCTTAATCCCAGGGTCGTGG	0	0	0	0
tsrna-05435	ACTCTTAATCCCAGGGTCGTG	0	1	0	0
tsrna-05434	ACTCTTAATCCCAGGG	0	2	0	0
tsrna-05432	ACTCTGGACTTTGAATCCA	1	1	0	0
tsrna-05431	ACTCTGGACTTTGAATCC	1	1	0	0
tsrna-05430	ACTCTGGACTTTGAATC	0	0	0	0
tsrna-05428	ACTCTGGACTCTGAATCCGGTA	0	2	0	0
tsrna-05427	ACTCTGGACTCTGAATCCGGT	0	3	0	0
tsrna-05426	ACTCTGGACTCTGAATCCGG	0	2	0	0
tsrna-05425	ACTCTGGACTCTGAATCCAGCGATCCGAGTTCA	1	3	0	0
tsrna-05424	ACTCTGGACTCTGAATCCAGCGATCCGAGTTC	4	6	1	3
tsrna-05423	ACTCTGGACTCTGAATCCAGCGATCCGAGT	4	2	0	0
tsrna-05422	ACTCTGGACTCTGAATCCAGCGATCCGAG	4	2	0	2
tsrna-05421	ACTCTGGACTCTGAATCCAGCGATCCGA	0	1	0	1
tsrna-05420	ACTCTGGACTCTGAATCCAGCGATCCG	3	1	1	1
tsrna-05419	ACTCTGGACTCTGAATCCAGCGATCC	0	3	0	0
tsrna-05418	ACTCTGGACTCTGAATCCAGCGATC	0	3	0	0
tsrna-05417	ACTCTGGACTCTGAATCCAGCGAT	1	4	0	0
tsrna-05416	ACTCTGGACTCTGAATCCAGCGA	1	3	1	0
tsrna-05415	ACTCTGGACTCTGAATCCAGCG	0	0	0	0
tsrna-05414	ACTCTGGACTCTGAATCCAGC	1	3	0	1
tsrna-05413	ACTCTGGACTCTGAATCCAG	1	2	1	1
tsrna-05412	ACTCTGGACTCTGAATCCA	1	1	0	0
tsrna-05411	ACTCTGGACTCTGAATCC	0	0	0	0
tsrna-05410	ACTCTGGACTCTGAATC	0	1	0	0
tsrna-05402	ACTCTGAATCCAGCGATCCGAGTTCAA	0	1	1	0
tsrna-05401	ACTCTGAATCCAGCGATCCGAGTTC	0	3	0	0
tsrna-05400	ACTCTGAATCCAGCGATCCGAGTT	0	0	0	0
tsrna-05397	ACTCTGAATCCAGCGATCCGA	1	1	0	0
tsrna-05396	ACTCTGAATCCAGCGATCCG	0	0	0	0
tsrna-05390	ACTCCTGGCTGGCTCGCCA	16	67	8	2

tsrna-05389	ACTCCTGGCTGGCTCGCC	0	0	1	0
tsrna-05386	ACTCCCGGTGTGGGAACCA	2	2	1	0
tsrna-05385	ACTCCCGGTGTGGGAACC	0	0	0	0
tsrna-05384	ACTCCCGGTGTGGGAAC	0	1	0	0
tsrna-05383	ACTCCAGATCAGAAGGTTGCGTGTTCAAGT	0	8	0	0
tsrna-05382	ACTCCAGATCAGAAGGTTGCGTGTT	0	1	0	0
tsrna-05381	ACTCCAGATCAGAAGGTTGCGTGT	0	1	0	0
tsrna-05380	ACTCCAGATCAGAAGGTTGCGTG	0	0	0	0
tsrna-05373	ACTCCAGATCAGAAGGCTGCGTGT	1	0	0	0
tsrna-05359	ACTCATGCCCCCATGTCTAACAACATGGCTTTCTCACCA	10	7	13	2
tsrna-05358	ACTCATGCCCCCATGTCTAACAACATGGCTTTCTCACC	8	3	10	0
tsrna-05357	ACTCATGCCCCCATGTCTAACAACATGGCT	1	0	0	0
tsrna-05356	ACTCATGCCCCCATGTCTAACAACATGGC	0	0	0	0
tsrna-05354	ACTCATGCCCCCATGTCTAACAACATG	0	1	0	0
tsrna-05353	ACTCATGCCCCCATGTCTAACAACA	0	0	1	0
tsrna-05352	ACTCATGCCCCCATGTCTAACA	0	0	0	0
tsrna-05345	ACTCAAGTTCTGGTCTCCGGATGG	1	5	0	1
tsrna-05344	ACTCAAGTTCTGGTCTCCGGA	1	1	0	0
tsrna-05343	ACTCAAGTTCTGGTCTCCGG	2	2	0	1
tsrna-05342	ACTCAAGTTCTGGTCTCCG	3	3	2	1
tsrna-05341	ACTCAAGTTCTGGTCTCCAAT	1	4	2	0
tsrna-05340	ACTCAAGTTCTGGTCTCCAA	1	4	0	2
tsrna-05339	ACTCAAGTTCTGGTCTCCA	1	2	2	1
tsrna-05338	ACTCAAGTTCTGGTCTCC	2	3	0	3
tsrna-05337	ACTCAAGTTCTGGTCTC	1	5	0	0
tsrna-05336	ACTCAAGTTCTGGTCT	1	1	0	0
tsrna-05330	ACTAGTTTTGACAACATT	0	0	0	1
tsrna-05326	ACTAAGTGTTTGTGGGTTTAAGTCCCATTGGTCTAGCCA	3	4	3	1
tsrna-05325	ACTAAGTGTTTGTGGGTTTAAGTCCCATTGGTCTAGCC	4	2	1	0
tsrna-05324	ACTAAGTGTTTGTGGGTTTAAGTCCCATTGGTCTAGC	4	3	0	0
tsrna-05323	ACTAAGTGTTTGTGGGTTTAAGTCCCATTGGTCTAG	2	1	3	0
tsrna-05322	ACTAAGTGTTTGTGGGTTTAAGTCCCATTGGTCTA	0	2	3	1
tsrna-05321	ACTAAGTGTTTGTGGGTTTAAGTCCCATTGGTCT	2	1	2	2
tsrna-05320	ACTAAGTGTTTGTGGGTTTAAGTCCCATTGGTC	0	2	3	0
tsrna-05319	ACTAAGTGTTTGTGGGTTTAAGTCCCATTGGT	0	1	1	0
tsrna-05318	ACTAAGTGTTTGTGGGTTTAAGTCCCATTGG	2	2	2	0
tsrna-05317	ACTAAGTGTTTGTGGGTTTAAGTCCCATTG	4	2	3	0
tsrna-05316	ACTAAGTGTTTGTGGGTTTAAGTCCCATT	3	1	3	0
tsrna-05315	ACTAAGTGTTTGTGGGTTTAAGTCCCA	0	0	1	0
tsrna-05312	ACTAAGTGTTTGTGGGTTTAAG	0	0	0	0
tsrna-05311	ACTAAGTGTTTGTGGGTTTAA	0	1	0	0
tsrna-05310	ACTAAGTGTTTGTGGGTTTA	0	0	1	0
tsrna-05307	ACTAAGTGTTTGTGGGT	0	1	0	0
tsrna-05303	ACGTATGAGGCCCCGGGTTCAATCCCCGGCATCTCCACCA	137	146	48	42
tsrna-05302	ACGTATGAGGCCCCGGGTTCAATCCCCGGCATCTCCACC	4	16	1	2
tsrna-05301	ACGTATGAGGCCCCGGGTTCAATCCCCGGCATCTCCAC	2	8	1	0
tsrna-05300	ACGTATGAGGCCCCGGGT	1	1	0	0
tsrna-05297	ACGGTAGCGCGTCTGACTCC	3	5	1	1
tsrna-05296	ACGGTAGCGCGTCTGACTC	1	1	0	2
tsrna-05295	ACGGTAGCGCGTCTGACT	0	1	0	2
tsrna-05294	ACGGTAGCGCGTCTGA	0	1	0	0
tsrna-05293	ACGGGCTCACATCACCCCATAAACACCA	8	4	3	4
tsrna-05292	ACGGGCTCACATCACCCCATAAACACC	0	0	0	1
tsrna-05287	ACGCGTGGGTTCGAATCCCATCCTCGTCGCCA	11	10	2	3
tsrna-05284	ACGCGGGAGGCCCGGGTTCGATTCCCGGCCCATGCACC	2	10	0	1
tsrna-05283	ACGCGGGAGGCCCGGGTTCGATTCCCGGCCCATG	2	4	0	0
tsrna-05282	ACGCGGGAGGCCCGGGTTCGATTCCCGGCCAATGCACCA	41	152	11	13
tsrna-05281	ACGCGGGAGGCCCGGGTTCGATTCCCGGCCAATGCACC	5	6	3	0
tsrna-05280	ACGCGGGAGGCCCGGGTTCGATTCCCGGCCAATGCAC	1	5	1	0
tsrna-05279	ACGCGGGAGGCCCGGGTTCGATTCCCGGCCAATGCA	1	5	0	0
tsrna-05278	ACGCGGGAGGCCCGGGTTCGATTCCCGGCCAATGC	0	5	2	0
tsrna-05277	ACGCGGGAGGCCCGGGTTCGATTCCCGGCCAATG	3	3	1	0
tsrna-05276	ACGCGGGAGGCCCGGGTTC	0	1	1	1
tsrna-05275	ACGCGGGAGGCCCGGGTT	0	7	0	0

tsrna-05274	ACGCGGGAGGCCCGGGT	0	5	0	0
tsrna-05272	ACGCGGGAGACCGGGGTTCGATTCCCCGACGGGGAGCCA	2	9	3	1
tsrna-05271	ACGCGGGAGACCGGGGTTCGATTCCCCGACGGGGAGCC	0	3	0	0
tsrna-05270	ACGCGGGAGACCGGGGTTCGATTCCCCGACGGGGAGC	1	3	1	0
tsrna-05269	ACGCGGGAGACCGGGGTTCGATTCCCCGACGGGGAG	0	2	0	0
tsrna-05268	ACGCGGGAGACCGGGGTTCGATTCCCCGACGGGGA	2	0	0	0
tsrna-05267	ACGCGGGAGACCGGGGTTCGATTCCCCGACGGGG	0	0	0	0
tsrna-05258	ACGCGGGAGACCGGGGTTCAATTCCCCGACGGGGAGCCA	0	4	0	0
tsrna-05257	ACGCGGGAGACCGGGGTTCAATTCCCCGACGGGGAGCC	1	0	0	0
tsrna-05256	ACGCGGGAGACCGGGGTTCAATTCCCCGACGGGGAGC	1	2	0	0
tsrna-05253	ACGCGGGAGACCGGGGTTCAATTCCCCGACGGGG	1	0	1	0
tsrna-05249	ACGCGAAAGGTCCCCGGTTT	0	5	0	0
tsrna-05248	ACGCGAAAGGTCCCCGGTTCGATCCCGGGCGGAAACACCA	17	50	8	7
tsrna-05247	ACGCGAAAGGTCCCCGGTTCGATCCCGGGCGGAAACACC	4	9	1	0
tsrna-05246	ACGCGAAAGGTCCCCGGTTCGATCCCGGGCGGAAACA	0	1	0	0
tsrna-05245	ACGCGAAAGGTCCCCGGTTCGAAACCGGGCGGAAACACCA	306	700	109	49
tsrna-05244	ACGCGAAAGGTCCCCGGTTCGAAACCGGGCGGAAACACC	70	198	28	3
tsrna-05243	ACGCGAAAGGTCCCCGGTTCGAAACCGGGCGGAAACAC	4	4	1	0
tsrna-05242	ACGCGAAAGGTCCCCGGTTCGAAACCGGGCGGAAACA	0	4	0	1
tsrna-05241	ACGCGAAAGGTCCCCGGTTCGAAACCGGGCGGAAAC	0	1	0	1
tsrna-05240	ACGCGAAAGGTCCCCGGTTCGAAACCGGGCAGAAGCACC	1	8	0	0
tsrna-05239	ACGCGAAAGGTCCCCGGTTCGAAACCGGGC	0	5	1	0
tsrna-05238	ACGCGAAAGGTCCCCGGTTCG	0	2	0	0
tsrna-05237	ACGCGAAAGGTCCCCGGTTC	0	5	0	0
tsrna-05236	ACGCGAAAGGTCCCCGGTT	0	4	1	0
tsrna-05235	ACGCGAAAGGTCCCCGGT	0	2	0	1
tsrna-05232	ACGCCAAGGTCGCGGGTTCGAT	0	2	0	0
tsrna-05231	ACGCCAAGGTCGCGGGTTCGA	1	3	0	0
tsrna-05230	ACGCCAAGGTCGCGGGTTCG	1	1	0	0
tsrna-05229	ACGCCAAGGTCGCGGGTT	0	1	2	1
tsrna-05228	ACGCCAAGGTCGCGGGT	0	1	1	0
tsrna-05226	ACGCAGAAGGTCCTGGGTTCGAGCCCCAGTGGAACCACCA	48	35	37	23
tsrna-05225	ACGCAGAAGGTCCTGGGTTCGAGCCCCAGTGGAACCACC	10	14	8	2
tsrna-05224	ACGCAGAAGGTCCTGGGTTCGAGCCCCAGTGGAACCAC	5	4	8	0
tsrna-05223	ACGCAGAAGGTCCTGGGTTCGAGCCCCAGTGGAACCA	8	7	4	0
tsrna-05222	ACGCAGAAGGTCCTGGGTTCGAGCCCCAGTGGAACC	9	5	8	0
tsrna-05221	ACGCAGAAGGTCCTGGGTTCGAGCCCCAGTGGAAC	2	9	0	1
tsrna-05220	ACGCAGAAGGTCCTGGGTTCGAGCCCCAGTGGAA	2	5	0	0
tsrna-05219	ACGCAGAAGGTCCTGGGTTCGAGCCCCAGT	3	6	1	0
tsrna-05218	ACGCAGAAGGTCCTGGGTTCGAGCCCCAG	2	3	3	0
tsrna-05217	ACGCAGAAGGTCCTGGGTTCAAGCCCCAGTGGAACCACC	1	4	2	3
tsrna-05216	ACGCAGAAGGTCCTGGGT	1	1	0	0
tsrna-05215	ACGATGGTTTTTCATATCATTGGTCGTGGTTGTAGTCCGTGCG	15	24	28	17
tsrna-05214	ACGATGGTTTTTCATATCATTGGTCGTGGTTGTAGTCCGTGCG	12	22	17	10
tsrna-05213	ACGATGGTTTTTCATATCATTGGTCGTGGTTGTAGTCCGTGCG	11	21	18	5
tsrna-05212	ACGATGGTTTTTCATATCATTGGTCGTGGTTGTAGTCC	2	1	3	3
tsrna-05211	ACGATGGTTTTTCATATCATTGGTCGTGGTTGTAGTC	2	0	1	0
tsrna-05210	ACGATGGTTTTTCATATCATTGGTCGTGGTTGTAGT	2	0	0	0
tsrna-05209	ACGATGGTTTTTCATATCATTGGTCGTGGTTGTAG	2	2	1	1
tsrna-05208	ACGATGGTTTTTCATATCATTGGTCGTGGTTGTA	0	1	2	1
tsrna-05207	ACGATGGTTTTTCATATCATTGGTCGTGGT	0	1	0	0
tsrna-05206	ACGATGGTTTTTCATATCATTGGTCGTGG	0	1	0	0
tsrna-05201	ACGAGGTGGCCGAGTGGTTAAGGCGATGG	0	3	0	0
tsrna-05200	ACGAGGTGGCCGAGTGGTTAAGGC	1	0	0	0
tsrna-05199	ACGAGGTGGCCGAGTGGTTAAGG	0	0	1	1
tsrna-05198	ACGAGGTGGCCGAGTGGTTAAG	0	0	0	1
tsrna-05197	ACGAGGTGGCCGAGTGGTTAA	0	0	0	0
tsrna-05195	ACGAGGTGGCCGAGTGGTT	0	1	0	0
tsrna-05194	ACGAGGTGGCCGAGTGGT	0	0	0	0
tsrna-05193	ACGAGGTGGCCGAGTGG	2	3	0	0
tsrna-05192	ACGAGGTCCTGGGTTC	2	6	0	0
tsrna-05191	ACGAGGCCCCGGGTTC	0	2	0	0
tsrna-05190	ACGACCCCTTATTTACCCCA	0	2	1	0
tsrna-05185	ACGAATGATTTCGACTCATTAAATTATG	2	3	6	0

tsrna-05184	ACGAATGATTTCGACTCAT	0	2	2	0
tsrna-05183	ACGAATGATTTCGACTCA	0	2	0	0
tsrna-05182	ACGAATGATTTCGACTC	0	3	0	0
tsrna-05179	CGCGGGTTCGATCCCCGTACTGGCCACCA	4	5	3	1
tsrna-05178	CGCGGGTTCGATCCCCGTACTGGCCACC	0	2	2	0
tsrna-05177	CGCGGGTTCGATCCCCGTACTGGCCAC	0	1	0	0
tsrna-05176	CGCGGGTTCGATCCCCGTACTGGCCA	0	0	0	0
tsrna-05175	CGCGGGTTCGATCCCCGTACTGGCC	0	0	0	0
tsrna-05173	CGCGGGTTCGATCCCCGTACGGGCCACCA	11	50	6	3
tsrna-05172	CGCGGGTTCGATCCCCGTACGGGCCACC	0	1	0	0
tsrna-05168	CGCGGGTTCGATCCCCGTACGGGC	0	1	0	0
tsrna-05166	CGCGGGTTCGAACCCCGTCCGTGCCTCCA	7	7	3	2
tsrna-05165	CGCGGGTTCGAACCCCGTCCGTGCCTC	0	0	0	0
tsrna-05163	CGCGGGAGGCCCGGGTTCGATTCCCGGCCCATGC	0	5	0	0
tsrna-05162	CGCGGGAGGCCCGGGTTCGATTCCCGGCCCATG	0	5	1	0
tsrna-05161	CGCGGGAGGCCCGGGTTCGATTCCCGGCCAATGCACCA	50	142	17	6
tsrna-05160	CGCGGGAGGCCCGGGTTCGATTCCCGGCCAATGCACC	6	13	1	0
tsrna-05159	CGCGGGAGGCCCGGGTTCGATTCCCGGCCAATGCAC	1	5	0	0
tsrna-05158	CGCGGGAGGCCCGGGTTCGATTCCCGGCCAATGCA	0	6	1	0
tsrna-05157	CGCGGGAGGCCCGGGTTCGATTCCCGGCCAATGC	1	3	0	0
tsrna-05156	CGCGGGAGGCCCGGGTTCGATTCCCGGCCAATG	0	5	0	0
tsrna-05155	CGCGGGAGGCCCGGGTTCG	2	1	0	0
tsrna-05154	CGCGGGAGGCCCGGGTTC	0	7	0	0
tsrna-05153	CGCGGGAGGCCCGGGTT	1	1	0	0
tsrna-05152	CGCGGGAGGCCCGGGT	0	4	0	0
tsrna-05151	CGCGGGAGACCGGGGTTCGATTCCCCGACGGGGAGCCA	0	14	1	0
tsrna-05150	CGCGGGAGACCGGGGTTCGATTCCCCGACGGGGAGCC	1	2	0	0
tsrna-05149	CGCGGGAGACCGGGGTTCGATTCCCCGACGGGGAGC	0	2	1	0
tsrna-05147	CGCGGGAGACCGGGGTTCGATTCCCCGACGGGGA	0	0	0	0
tsrna-05146	CGCGGGAGACCGGGGTTCGATTCCCCGACGGGG	0	1	0	0
tsrna-05145	CGCGGGAGACCGGGGTTCGATTCCCCGACGGG	0	0	1	0
tsrna-05136	CGCGGGAGACCGGGGTTCAATTCCCCGACGGGGAGCCA	0	2	0	0
tsrna-05134	CGCGGGAGACCGGGGTTCAATTCCCCGACGGGGAG	0	1	0	0
tsrna-05132	CGCGGCCCGGGTTCGATTCCCGGTCAGGGAACCA	21	315	6	6
tsrna-05131	CGCGGCCCGGGTTCGATTCCCGGTCAGGGAACC	0	13	1	0
tsrna-05130	CGCGGCCCGGGTTCGATTCCCGGTCAGGG	0	0	1	0
tsrna-05128	CGCGGCCCGGGTTCGATTCCCGGTCAG	0	1	1	0
tsrna-05122	CGCGAGTTCAAATCTCGCTGGGGCCT	0	0	0	0
tsrna-05120	CGCGAAAGGTCCCCGGTTT	0	2	0	0
tsrna-05119	CGCGAAAGGTCCCCGGTTCGAAACCGGGCGGAAACACC	56	187	29	7
tsrna-05118	CGCGAAAGGTCCCCGGTTCGAAACCGGGCG	0	0	0	0
tsrna-05117	CGCGAAAGGTCCCCGGTTCG	0	6	1	0
tsrna-05116	CGCGAAAGGTCCCCGGTTC	0	2	1	0
tsrna-05115	CGCGAAAGGTCCCCGGTT	1	2	0	0
tsrna-05114	CGCGAAAGGTCCCCGGT	0	1	0	0
tsrna-05112	CGCCTGTCACGCGGGAGACTGG	54	141	32	7
tsrna-05111	CGCCTGTCACGCGGGAGACCGGGGTTCGATTCCCCGACGGC	20	65	8	3
tsrna-05110	CGCCTGTCACGCGGGAGACCGGGGTTCGATTCCCCGACGGC	17	61	7	4
tsrna-05109	CGCCTGTCACGCGGGAGACCGGGGTTCGATTCCCCGACGGC	20	57	9	1
tsrna-05108	CGCCTGTCACGCGGGAGACCGGGGTTCGATTCCCCGACGGC	11	56	3	2
tsrna-05107	CGCCTGTCACGCGGGAGACCGGGGTTCGATTCCCCGACGGC	14	62	13	4
tsrna-05106	CGCCTGTCACGCGGGAGACCGGGGTTCGATTCCCCGACGGC	14	58	4	2
tsrna-05105	CGCCTGTCACGCGGGAGACCGGGGTTCGATTCCCCGACGGC	10	44	8	4
tsrna-05104	CGCCTGTCACGCGGGAGACCGGGGTTCGATTCCCCGACGG	13	63	3	5
tsrna-05103	CGCCTGTCACGCGGGAGACCGGGGTTCGATTCCCCGACG	14	59	7	4
tsrna-05102	CGCCTGTCACGCGGGAGACCGGGGTTCGATTCCCCG	12	49	7	7
tsrna-05101	CGCCTGTCACGCGGGAGACCGGGGTTCG	10	46	10	1
tsrna-05100	CGCCTGTCACGCGGGAGACCGGGGTTCAATTCCCCGACGGG	19	50	6	0
tsrna-05099	CGCCTGTCACGCGGGAGACCGGGGTTCAATTCCCCGACGGG	20	52	8	1
tsrna-05098	CGCCTGTCACGCGGGAGACCGGGGTTCA	13	53	11	6
tsrna-05097	CGCCTGTCACGCGGGAGACCGGGGTTC	11	49	12	1
tsrna-05096	CGCCTGTCACGCGGGAGACCGGGGTT	19	55	9	1
tsrna-05095	CGCCTGTCACGCGGGAGACCGGGGT	19	52	4	3
tsrna-05094	CGCCTGTCACGCGGGAGACCGGGG	13	59	5	2

tsrna-05093	CGCCTGTCACGCGGGAGACCGGG	10	60	8	2
tsrna-05092	CGCCTGTCACGCGGGAGACCGG	5	37	7	2
tsrna-05091	CGCCTGTCACGCGGGAGACCG	23	45	11	2
tsrna-05090	CGCCTGTCACGCGGGAGACC	17	57	4	3
tsrna-05089	CGCCTGTCACGCGGGAGAC	14	35	9	3
tsrna-05088	CGCCTGTCACGCGGGAGA	20	58	4	3
tsrna-05087	CGCCTGTCACGCGGGAG	8	44	10	4
tsrna-05086	CGCCTGTCACGCGGGA	10	34	3	2
tsrna-05085	CGCCTGCCACGCGGGAGGCCCGGGTTCG	5	21	2	1
tsrna-05084	CGCCTGCCACGCGGGAGGCCCGGGT	3	15	3	0
tsrna-05083	CGCCTGCCACGCGGGAGGCCCGGG	0	2	0	0
tsrna-05082	CGCCTGCCACGCGGGAGGCCCGG	0	1	0	0
tsrna-05081	CGCCTGCCACGCGGGAGGCCCG	0	1	0	0
tsrna-05080	CGCCTGCCACGCGGGAGGCCC	1	0	0	0
tsrna-05074	CGCCTCCCACGCGGGAGACCCG	0	0	0	0
tsrna-05073	CGCCTCACACGCGAAAGGTCCCCGG	0	5	0	0
tsrna-05071	CGCCGTGATCGTATAGTGGTTAGTACTCTGCGTTGT	26	9	17	10
tsrna-05070	CGCCGTGATCGTATAGTGGTTAGTACTCTGCGTTG	28	10	20	4
tsrna-05069	CGCCGTGATCGTATAGTGGTTAGTACTCTGCGTT	21	12	9	2
tsrna-05068	CGCCGTGATCGTATAGTGGTTAGTACTCTGCG	6	2	2	2
tsrna-05067	CGCCGTGATCGTATAGTGGTTAGTACTCTGC	2	2	0	2
tsrna-05066	CGCCGTGATCGTATAGTGGTTAGTACTCTG	2	1	1	0
tsrna-05065	CGCCGTGATCGTATAGTGGTTAGTACTCT	2	1	1	0
tsrna-05064	CGCCGTGATCGTATAGTGGTTAGTACTC	0	0	1	0
tsrna-05063	CGCCGTGATCGTATAGTGGTTAGTACT	0	0	0	0
tsrna-05055	CGCCGCTGGTGTAGTGGTATCATGCAAGATTCCC	14	5	25	0
tsrna-05054	CGCCGCTGGTGTAGTGGTATCATGCAAGATTCC	17	6	21	2
tsrna-05053	CGCCGCTGGTGTAGTGGTATCATGCAAGATTC	14	3	17	1
tsrna-05052	CGCCGCTGGTGTAGTGGTATCATGCAAGATT	9	4	9	1
tsrna-05051	CGCCGCTGGTGTAGTGGTATCATGCAAGAT	9	2	4	1
tsrna-05050	CGCCGCTGGTGTAGTGGTATCATGCAAGA	9	2	9	2
tsrna-05049	CGCCGCTGGTGTAGTGGTATCATGCAAG	3	0	6	0
tsrna-05048	CGCCGCTGGTGTAGTGGTATCATGCAA	1	1	0	0
tsrna-05047	CGCCGCTGGTGTAGTGGTATCATGCA	4	2	2	0
tsrna-05046	CGCCGCTGGTGTAGTGGTATCATGC	2	1	1	0
tsrna-05045	CGCCGCTGGTGTAGTGGTATCATG	1	1	1	0
tsrna-05044	CGCCGCTGGTGTAGTGGTATC	0	1	1	0
tsrna-05043	CGCCGCTGGTGTAGTGGTA	0	0	1	0
tsrna-05039	CGCCGCGGCCCGGGTTCGATTCCCGGTCAGGGAACCA	16	339	10	6
tsrna-05038	CGCCGCGGCCCGGGTTCGATTCCCGGTCAGGGAACC	3	9	0	0
tsrna-05037	CGCCGCGGCCCGGGTTCGATTCCCGGTCAGGGAAC	0	3	0	0
tsrna-05036	CGCCGCGGCCCGGGTTCGATTCCCGGTCAGGGAA	1	2	0	0
tsrna-05035	CGCCGCGGCCCGGGTTCGATTCCCGGTCAGGGA	0	1	0	0
tsrna-05033	CGCCGCGGCCCGGGTTCGATTCCCGGTCAGG	0	0	0	0
tsrna-05031	CGCCGCGGCCCGGGTTCGATTCCCGGTCA	0	0	1	0
tsrna-05021	CGCCCGGCTAGCTCAGTCGGTAGAGCATGGGACTCT	212	109	194	94
tsrna-05020	CGCCCGGCTAGCTCAGTCGGTAGAGCATGGGACTC	78	47	73	30
tsrna-05019	CGCCCGGCTAGCTCAGTCGGTAGAGCATGGGACT	35	48	32	16
tsrna-05018	CGCCCGGCTAGCTCAGTCGGTAGAGCATGAGACTC	95	102	84	57
tsrna-05017	CGCCCGGATAGCTCAGTCGGTAGAGCATC	13	15	16	9
tsrna-05016	CGCCCGGATAGCTCAGTCGGTAGAGCA	6	3	3	5
tsrna-05015	CGCCCGGATAGCTCAGTCGGTAGAG	0	3	7	5
tsrna-05014	CGCCCGGATAGCTCAGTCGGTAGA	1	5	6	4
tsrna-05013	CGCCCGGATAGCTCAGTCGGTAG	0	1	3	2
tsrna-05012	CGCCCGGATAGCTCAGTCGGTA	4	2	3	1
tsrna-05011	CGCCCGGATAGCTCAGTCGGT	5	6	0	0
tsrna-05010	CGCCCGGATAGCTCAGTCGG	0	1	1	1
tsrna-05009	CGCCCGGATAGCTCAGTCG	1	0	0	1
tsrna-05008	CGCCCGCATTCTCCACCA	0	3	0	0
tsrna-05006	CGCCAAGGTCGCGGGTTCGATCCCCGTACGGGCCACC	2	8	2	1
tsrna-05005	CGCCAAGGTCGCGGGTTCGAT	1	4	0	0
tsrna-05004	CGCCAAGGTCGCGGGTTCGA	0	1	0	0
tsrna-05003	CGCCAAGGTCGCGGGTTCG	0	0	0	0
tsrna-05002	CGCCAAGGTCGCGGGTTC	0	3	1	0

tsrna-05001	CGCATTGGTGGTTCAGTGGTAGAATTCTCGCCTGCC	5194	904	5837	363
tsrna-05000	CGCATTGGTGGTTCAGTGGTAGAATTCTCGCCTGC	5033	807	5592	350
tsrna-04999	CGCATTGGTGGTTCAGTGGTAGAATTCTCGCCTG	4862	774	5459	329
tsrna-04998	CGCATTGGTGGTTCAGTGGTAGAATTCTCGCCT	4273	671	5135	309
tsrna-04997	CGCATTGGTGGTTCAGTGGTAGAATTCTCGCC	2704	409	2914	199
tsrna-04996	CGCATTGGTGGTTCAGTGGTAGAATTCTCGC	201	121	178	76
tsrna-04995	CGCATTGGTGGTTCAGTGGTAGAATTCTCG	62	17	43	50
tsrna-04994	CGCATTGGACTTCTAAT	0	1	0	0
tsrna-04991	CGCATGTATGAGGTCCCGGGT	3	8	1	0
tsrna-04990	CGCATGGGTGGTTCAGTGGTAGAATTCTCGCCT	1305	371	1914	285
tsrna-04987	CGCAGCGGAAGCGTGCTGGGC	5	19	4	1
tsrna-04986	CGCAGCAACCTCGGTTCGAATCCGAGTCACGGCACCA	3	23	0	1
tsrna-04985	CGCAGCAACCTCGGTTCGAATCCGAGTCACGGCACC	0	6	0	1
tsrna-04984	CGCAGCAACCTCGGTTCGAATCCGAGTCACGGCAC	1	2	0	0
tsrna-04983	CGCAGCAACCTCGGTTCGAATCCGAGTCACGGC	0	0	0	0
tsrna-04982	CGCAGCAACCTCGGTTCGAATCCGAGTCACGG	0	1	0	0
tsrna-04981	CGCAGCAACCTCGGTTCGAATCCGAGTCAC	0	0	1	0
tsrna-04978	CGCAGAAGGTCCTGGGTTCGAGCCCCAGTGGAACCACCA	62	49	36	27
tsrna-04977	CGCAGAAGGTCCTGGGTTCGAGCCCCAGTGGAACCACC	13	11	9	1
tsrna-04976	CGCAGAAGGTCCTGGGTTCGAGCCCCAGTGGAACC	9	10	6	0
tsrna-04975	CGCAGAAGGTCCTGGGTTCGAGCCCCAGTGGAAC	3	4	3	0
tsrna-04974	CGCAGAAGGTCCTGGGTTCGAGCCCCAGTG	1	5	0	0
tsrna-04973	CGCACGTTGGGCGCCA	0	2	1	1
tsrna-04972	CGCAATGGATAGCGCATTGGACT	6	14	1	5
tsrna-04971	CGCAATGGATAACGCGTCTGACT	1	0	0	0
tsrna-04970	CGATTCTCATAGTCCTAGCCA	0	1	1	0
tsrna-04969	CGATTCTCATAGTCCTAGCC	0	1	1	0
tsrna-04967	CGATTCTCATAGTCCTAG	0	1	1	1
tsrna-04966	CGATTCCTTCCTTTTTTGCCA	0	0	0	1
tsrna-04965	CGATTCCTTCCTTTTTTGCC	0	0	0	0
tsrna-04964	CGATTCCTTCCTTTTTTGC	0	0	0	0
tsrna-04963	CGATTCCTTCCTTTTTTG	1	0	0	0
tsrna-04962	CGATTCCGGCTCGAAGGACCA	66	144	28	10
tsrna-04961	CGATTCCGGCTCGAAGGACC	2	6	1	0
tsrna-04960	CGATTCCGGCTCGAAGGAC	0	1	0	0
tsrna-04956	CGATTCCCGGTCAGGGAACCA	1	11	2	0
tsrna-04955	CGATTCCCGGTCAGGGAACC	0	1	0	0
tsrna-04950	CGATTCCCGGGCGGCGCACCA	13	24	2	0
tsrna-04949	CGATTCCCGGGCGGCGCAC	1	0	0	0
tsrna-04947	CGATTCCCGGCCCATGCACCA	9	13	2	2
tsrna-04946	CGATTCCCGGCCAATGCACCA	8	6	5	0
tsrna-04945	CGATTCCCGGCCAATGCACC	1	0	0	2
tsrna-04944	CGATTCCCGGCCAATGCAC	0	0	0	0
tsrna-04942	CGATTCCCGGCCAACGCACCA	2	11	3	3
tsrna-04941	CGATTCCCGGCCAACGCACC	0	2	0	1
tsrna-04940	CGATTCCCGGCCAACGCA	0	1	0	0
tsrna-04938	CGATTCCCCGACGGGGAGCCA	1	1	0	0
tsrna-04929	CGATGGTTTTTCATATCATTGGTCGTGGTTGTAGTC	0	0	0	1
tsrna-04928	CGATGGTTTTTCATATCATTGGTCGTGGTT	0	1	0	0
tsrna-04923	CGATGGATCGAAACCATCCTCTGCTACCA	14	17	5	16
tsrna-04922	CGATGGATCGAAACCATCCTCTGCTACC	2	3	2	0
tsrna-04921	CGATGGATCGAAACCATCCTCTGCT	1	0	0	0
tsrna-04919	CGATGCCCGCATTCTCCACCA	1	3	0	0
tsrna-04918	CGATGCCCGCATCCTCCACCA	3	3	2	0
tsrna-04917	CGATGCCCGCATCCTCCACC	0	1	0	0
tsrna-04914	CGATCCTCGCTGGGGCCTCCA	6	9	2	5
tsrna-04913	CGATCCTCACCTGGAGCACCA	2	1	0	1
tsrna-04910	CGATCCTCACACGGGGCACCA	0	5	0	0
tsrna-04908	CGATCCGAGTTCAAATCTCGGTGGAACCTC	1	1	0	0
tsrna-04906	CGATCCCGGGTTTCGGCACCA	36	104	10	9
tsrna-04905	CGATCCCGGGTTTCGGCACC	4	7	3	1
tsrna-04900	CGATCCCGGGCGGAAACACCA	12	39	6	6
tsrna-04899	CGATCCCCGTACTGGCCACCA	1	7	4	2
tsrna-04897	CGATCCCCGTACGGGCCACCA	3	48	3	2

tsrna-04896	CGATCCCCGGCATCTCCACCA	110	139	52	33
tsrna-04895	CGATCCCCGGCATCTCCACC	1	4	3	1
tsrna-04894	CGATCCCCGGCATCTCCAC	1	0	0	0
tsrna-04893	CGATCCCCGGCATCTCCA	0	1	0	0
tsrna-04891	CGATCCCCGGCACCTCCACCA	149	249	72	32
tsrna-04890	CGATCCCCGGCACCTCCACC	7	12	3	2
tsrna-04889	CGATCCCCGACACCTCCACCA	2	5	1	1
tsrna-04888	CGATCCCCGACACCTCCACC	1	0	0	0
tsrna-04887	CGATCCCCAGTACCTCCACCA	2	4	0	0
tsrna-04886	CGATCCCCAGTACCTCCACC	1	0	0	0
tsrna-04884	CGATCCCCAGTACCTC	0	1	0	0
tsrna-04883	CGATCCCCAGCATCTCCACCA	4	3	0	0
tsrna-04882	CGATCCCCAGCATCTCCACC	0	1	0	0
tsrna-04879	CGATCCCACCCAGGGACGCCA	1	3	0	0
tsrna-04878	CGATAGCTCAGTTGGTAGAGCGGAGGACT	0	0	0	1
tsrna-04876	CGATAGCTCAGTTGGTAGA	0	0	0	0
tsrna-04875	CGATAGCTCAGCTGGTAGAGCGGAGGACTG	0	0	0	0
tsrna-04873	CGATAGCTCAGCTGGTAGA	0	0	0	0
tsrna-04872	CGAGTTCGAGTCTCGGTGGAACC	0	1	0	0
tsrna-04871	CGAGTTCAAGTCTCGGTGGAACCTCCA	64	113	19	13
tsrna-04870	CGAGTTCAAATCTCGGTGGGACCTCCA	87	121	30	22
tsrna-04869	CGAGTTCAAATCTCGGTGGGACCTC	0	3	0	0
tsrna-04868	CGAGTTCAAATCTCGGTGGGACCT	0	2	0	0
tsrna-04866	CGAGTTCAAATCTCGGTGGAACCTCCA	150	210	67	21
tsrna-04865	CGAGTTCAAATCTCGGTGGAACCTCC	12	15	5	0
tsrna-04864	CGAGTTCAAATCTCGGTGGAACCTC	0	0	0	1
tsrna-04863	CGAGTTCAAATCTCGGTGGAACCT	0	1	0	0
tsrna-04862	CGAGTTCAAATCTCGGTGGAACC	1	0	1	0
tsrna-04861	CGAGTTCAAATCTCGCTGGGGCCTCCA	42	59	15	23
tsrna-04860	CGAGTTCAAATCTCGCTGGGGCCTCC	4	2	3	1
tsrna-04859	CGAGTTCAAATCTCGCTGGGGCCTC	0	0	0	0
tsrna-04858	CGAGTGGTTAAGGTGTTGGACT	1	0	0	0
tsrna-04854	CGAGTCTCGGTGGAACCTCCA	49	105	15	13
tsrna-04853	CGAGTCCTGCCGCGGTCGCCA	1	4	1	0
tsrna-04849	CGAGTCCCTTCGTGGTCGCCA	0	3	0	0
tsrna-04848	CGAGTCCCGGCGGAGTCGCCA	0	2	1	0
tsrna-04845	CGAGTCCCATCTGGGTCGCCA	0	2	0	1
tsrna-04842	CGAGTCCCACCTGGGGTGCCA	0	1	0	0
tsrna-04839	CGAGTCCCACCCGGGGTACCA	0	0	0	0
tsrna-04838	CGAGTCCCACCAGAGTCGCCA	1	0	0	0
tsrna-04836	CGAGGTGGCCGAGTGGTTAAGGCGATGGAC	0	4	0	0
tsrna-04826	CGAGGTCCTGGGTTCGATCCCCAGTAC	0	0	1	0
tsrna-04825	CGAGGTCCTGGGTTCGATCCCCAGTA	0	1	0	0
tsrna-04824	CGAGGTCCTGGGTTCGATCCCCAGT	1	1	0	0
tsrna-04823	CGAGGTCCTGGGTTCGATCCCC	0	0	1	1
tsrna-04822	CGAGGTCCTGGGTTCGATCCC	0	0	0	0
tsrna-04820	CGAGGCCCCGGGTTCAATCCCCGGCACCT	1	3	0	0
tsrna-04819	CGAGGCCCCGGGTTCAATCCCCGGCACC	0	0	1	0
tsrna-04818	CGAGGCCCCGGGTTCAATCCCCGGCA	0	1	0	0
tsrna-04816	CGAGGCCCCGGGTTCAATCCCCGG	0	0	1	0
tsrna-04814	CGAGGCCCCGGGTTCAATCCC	0	1	0	0
tsrna-04807	CGAGCCTCACCTGGAGCACCA	1	0	0	0
tsrna-04806	CGAGCCTCACCTGGAGCACC	0	1	0	0
tsrna-04804	CGAGCCCCAGTGGAACCACCA	5	8	3	5
tsrna-04803	CGAGCCCCAGTGGAACCACC	0	0	0	0
tsrna-04802	CGAGCCCCAGTGGAACCAC	0	0	0	0
tsrna-04801	CGAGCCCCACGTTGGGCGCCA	3	40	2	1
tsrna-04800	CGAGCCCCACGTTGGGCGCC	0	5	0	0
tsrna-04799	CGAGCCCCACGTTGGGCGC	0	1	0	0
tsrna-04798	CGAGCCCACCCAGGGACGCCA	4	61	1	1
tsrna-04797	CGAGCCCACCCAGGGACGCC	0	6	0	0
tsrna-04793	CGAGAGGTCCCGGGTTCAAATCCCGGACGAGCCCC	2	8	2	0
tsrna-04792	CGAGAGGTCCCGGGTTCAAATCCCGGACG	0	1	0	0
tsrna-04791	CGAGAGGTCCCGGGTTCAAATCCCG	0	0	0	0

tsrna-04790	CGAGAGGTCCCGGGTTCAAA	0	3	0	0
tsrna-04788	CGAGAGGTCCCGGGTTC	0	0	1	0
tsrna-04787	CGAGAGGTCCCGGGTT	1	0	1	0
tsrna-04786	CGACTCCTGGCTGGCTCGCCA	20	59	5	2
tsrna-04785	CGACTCCTGGCTGGCTCGCC	0	0	2	0
tsrna-04782	CGACTCCCGGTGTGGGAACCA	8	7	2	2
tsrna-04781	CGACTCCCGGTGTGGGAACC	0	1	0	0
tsrna-04779	CGACTCCCGGTATGGGAACCA	1	2	0	1
tsrna-04777	CGACTCATTAAATTATGATAATCATATTT	0	0	1	0
tsrna-04771	CGACCCCCGGCTCCTCCACCA	8	21	5	1
tsrna-04770	CGAATGATTTCGACTCATTAAATTATGAT	1	4	0	0
tsrna-04769	CGAATGATTTCGACTCATT	0	1	0	0
tsrna-04768	CGAATGATTTCGACTCA	0	0	0	0
tsrna-04767	CGAATGATTTCGACTC	0	2	1	0
tsrna-04766	CGAATCTCGGTGGGACCTCCA	54	91	22	25
tsrna-04765	CGAATCTCGGTGGGACCTC	0	1	0	0
tsrna-04764	CGAATCCTGTTCGTGACGCCA	4	4	2	4
tsrna-04763	CGAATCCTGTTCGTGACGCC	4	2	0	0
tsrna-04762	CGAATCCTGTCGGCTACGCCA	5	12	2	8
tsrna-04761	CGAATCCTGCTCACAGCGCCA	7	19	3	4
tsrna-04760	CGAATCCTGCTCACAGCGCC	0	2	1	0
tsrna-04758	CGAATCCTGCCGACTACGCCA	91	245	43	20
tsrna-04757	CGAATCCTGCCGACTACGCC	6	8	3	2
tsrna-04756	CGAATCCTGCCGACTACGC	0	0	0	0
tsrna-04754	CGAATCCGGCTCGGAGGACCA	0	2	1	1
tsrna-04751	CGAATCCGGCTCGAAGGACCA	15	42	4	7
tsrna-04750	CGAATCCGGCTCGAAGGACC	0	1	0	0
tsrna-04749	CGAATCCGGCTCGAAGGAC	0	0	0	0
tsrna-04741	CGAATCCCATCCTCGTCGCCA	2	6	2	0
tsrna-04740	CGAATCCCATCCTCGTCGCC	1	0	0	1
tsrna-04737	CGAATCCCAGTAGAGCCTCCA	1	4	1	5
tsrna-04736	CGAATCCCAGCGGTGCCTCCA	3	6	0	0
tsrna-04730	CGAATCCCAGCGAGGCCTCCA	0	0	0	2
tsrna-04729	CGAATCCCACTTCTGACACCA	1	4	5	0
tsrna-04728	CGAATCCCACTTCTGACACC	0	1	0	0
tsrna-04727	CGAATCCCACTTCTGACAC	0	0	0	0
tsrna-04726	CGAATCCCACTTCTGACA	0	0	1	0
tsrna-04725	CGAATCCCACTCCTGACACCA	80	56	45	26
tsrna-04724	CGAATCCCACTCCTGACACC	5	0	1	0
tsrna-04722	CGAATCCCACTCCTGACA	0	0	1	0
tsrna-04720	CGAATCCCACCTTCGTCGCCA	1	2	0	1
tsrna-04719	CGAATCCCACCGCTGCCACCA	3	1	0	0
tsrna-04718	CGAATCCCACCGCTGCCACC	0	1	0	1
tsrna-04714	CGAATCCCACCCTCGTCGCCA	6	26	1	2
tsrna-04713	CGAATCCCACCAGAGTCGCCA	5	2	0	1
tsrna-04712	CGAATCCCACCAGAGTCGCC	0	3	0	0
tsrna-04710	CGAATCCCACCACTGCCACCA	0	1	0	0
tsrna-04709	CGAATCACGTCGGGGTCACCA	1	8	3	3
tsrna-04708	CGAATCACGTCGGGGTCACC	0	0	0	0
tsrna-04703	CGAAGATCGCGGGTTCG	0	1	0	0
tsrna-04702	CGAAGATCGCGGGTTC	0	3	0	0
tsrna-04701	CGAAGAAGCAGCTTCAAACCTGCCGGGGCTTCC	1	6	2	0
tsrna-04700	CGAAGAAGCAGCTTCAAACCTGCCGGGGCTT	0	2	0	0
tsrna-04699	CGAAGAAGCAGCTTCAAACCTGCCGGGGCT	0	1	2	0
tsrna-04698	CGAAGAAGCAGCTTCAAACCTGCCGGGGC	0	1	0	0
tsrna-04696	CGAACCTCAGAGGGGGCACCA	0	2	0	0
tsrna-04695	CGAACCCTGCTCGCTGCGCCA	11	28	3	0
tsrna-04694	CGAACCCTGCTCGCTGCGCC	2	1	0	0
tsrna-04690	CGAACCCCGTCCGTGCCTCCA	5	12	2	0
tsrna-04688	CGAACCCCGTACGGGCCACCA	1	1	0	0
tsrna-04685	CGAAATCCAATGGGGTTTCCCC	0	8	0	0
tsrna-04680	CGAAAGGTTGGTGGTTCGAGCCCACCCAGGGACGCCA	7	109	3	0
tsrna-04679	CGAAAGGTTGGTGGTTCGAGCCCACCCAGGGACGCC	2	13	1	2
tsrna-04678	CGAAAGGTTGGTGGTTCGAGCCCACCCAGG	1	2	0	0

tsrna-04675	CGAAAGGTTGGTGGTT	0	0	1	0
tsrna-04674	CGAAAGGTCCCCGGTTCGAAACCGGGCGG	0	1	0	0
tsrna-04673	CGAAAGGTCCCCGGTTCGAAACCGGGC	0	0	1	0
tsrna-04672	CGAAAGGTCCCCGGTTCGAA	1	0	0	0
tsrna-04671	CGAAAGGTCCCCGGTTCG	0	1	0	0
tsrna-04670	CGAAAGGTCCCCGGTTC	0	2	0	0
tsrna-04669	CGAAAGGTCCCCGGTT	1	3	0	0
tsrna-04668	CGAAACTGGGCGGAAACACCA	4	8	1	2
tsrna-04667	CGAAACCGGGCGGAAACACCA	296	718	124	31
tsrna-04666	CGAAACCGGGCGGAAACACC	58	172	31	7
tsrna-04665	CGAAACCGGGCGGAAACAC	0	1	0	1
tsrna-04664	CGAAACCGGGCAGAAGCACCA	40	120	12	2
tsrna-04663	CGAAACCATCCTCTGCTACCA	2	6	2	1
tsrna-04662	CGAAACCATCCTCTGCTACC	1	0	0	1
tsrna-04660	CGAAACCATCCTCTGCTA	0	0	0	0
tsrna-04657	CGAAAATGTTGGTTATACCCTTCCCGTACTACCA	6	31	16	1
tsrna-04656	CGAAAATGTTGGTTATACCCTTCCCGTACTACC	2	2	2	0
tsrna-04655	CGAAAATGTTGGTTATACCCTTCCCGTACT	0	0	0	0
tsrna-04654	CGAAAATGTTGGTTATACCCTTCCCGTAC	0	0	0	0
tsrna-04653	CGAAAATGTTGGTTATACCCTTCCCGTA	0	0	0	0
tsrna-04652	CGAAAATGTTGGTTATACCCTTCCCG	0	0	0	0
tsrna-04644	CGAAAATGTTGGTTATA	1	0	0	0
tsrna-04642	CCTTTTTCCAAGGACACCA	0	2	4	2
tsrna-04641	CCTTTTTCCAAGGACACC	0	0	0	0
tsrna-04639	CCTTTTAAGTTAAAGATTAAGAGAACCAACACCTCTTTACAGT(	3	1	5	1
tsrna-04638	CCTTTTAAGTTAAAGATTAAGAGAACCAACACCTCTTTACAGT(	3	2	3	0
tsrna-04637	CCTTTTAAGTTAAAGATTAAGAGAACC	0	0	0	0
tsrna-04636	CCTTTTAAGTTAAAGATTAAGAG	0	0	0	0
tsrna-04635	CCTTCGATAGCTCAGTTGGTAGAGCGGAGGACT	22	16	15	3
tsrna-04634	CCTTCGATAGCTCAGTTGGTAGAGCGGAGG	14	13	8	8
tsrna-04633	CCTTCGATAGCTCAGTTGGTAGAGCG	11	12	15	7
tsrna-04632	CCTTCGATAGCTCAGTTGGTAGAGC	12	6	10	2
tsrna-04631	CCTTCGATAGCTCAGTTGGTAGAG	12	7	8	6
tsrna-04630	CCTTCGATAGCTCAGTTGGTAGA	9	16	9	2
tsrna-04629	CCTTCGATAGCTCAGTTGGTAG	14	15	15	3
tsrna-04628	CCTTCGATAGCTCAGTTGGTA	8	6	10	4
tsrna-04627	CCTTCGATAGCTCAGTTGGT	10	8	9	4
tsrna-04626	CCTTCGATAGCTCAGTTGG	14	10	2	1
tsrna-04625	CCTTCGATAGCTCAGTTG	5	12	9	1
tsrna-04624	CCTTCGATAGCTCAGTT	5	8	12	5
tsrna-04623	CCTTCGATAGCTCAGT	13	10	6	3
tsrna-04622	CCTTCGATAGCTCAGCTGGTAGAGCGGAGG	33	38	21	22
tsrna-04621	CCTTCGATAGCTCAGCTGGTAGAGC	42	37	28	25
tsrna-04620	CCTTCGATAGCTCAGCTGGTAGAG	38	30	21	17
tsrna-04619	CCTTCGATAGCTCAGCTGGTAGA	47	29	19	24
tsrna-04618	CCTTCGATAGCTCAGCTGGTAG	42	29	21	16
tsrna-04617	CCTTCGATAGCTCAGCTGGTA	39	30	12	20
tsrna-04616	CCTTCGATAGCTCAGCTGGT	45	27	25	27
tsrna-04615	CCTTCGATAGCTCAGCTGG	47	22	17	14
tsrna-04614	CCTTCGATAGCTCAGCTG	24	20	11	19
tsrna-04613	CCTTCGATAGCTCAGCT	21	24	20	13
tsrna-04612	CCTTCGATAGCTCAGC	31	16	27	22
tsrna-04609	CCTTCCAAGCAGTTGACCCGGGTTCG	37	43	11	9
tsrna-04608	CCTTCCAAGCAGTTGACCCGGGTTC	29	36	24	7
tsrna-04607	CCTTCCAAGCAGTTGACCCGGGTT	41	23	24	14
tsrna-04606	CCTTCCAAGCAGTTGACCCGGGT	32	43	23	6
tsrna-04605	CCTTCCAAGCAGTTGACCCGGGC	8	22	12	4
tsrna-04604	CCTTCCAAGCAGTTGACCCGGG	1	2	0	3
tsrna-04603	CCTTCCAAGCAGTTGACCCGG	2	1	3	0
tsrna-04602	CCTTCCAAGCAGTTGACCCG	0	2	0	1
tsrna-04601	CCTTCCAAGCAGTTGACCC	2	2	1	0
tsrna-04600	CCTTCCAAGCAGTTGACC	0	0	0	0
tsrna-04599	CCTTCCAAGCAGTTGAC	2	0	0	0
tsrna-04597	CCTTCAAAGCCCTCAGTAAGTTGC	0	1	0	0

tsrna-04594	CCTTAGGTCGCTGGTTCG	0	1	1	0
tsrna-04593	CCTGTTCGTGACGCCA	0	2	0	0
tsrna-04592	CCTGTGGTCTAGTGGTTAGG	2	1	0	0
tsrna-04591	CCTGTCGGCTACGCCA	1	0	0	1
tsrna-04590	CCTGTCACGCGGGAGACCGGGGTTCGATTCCCCGACGGGGA	20	74	7	2
tsrna-04589	CCTGTCACGCGGGAGACCGGGGTTCGATTCCCCGACGGGGA	19	48	12	3
tsrna-04588	CCTGTCACGCGGGAGACCGGGGTTCGATTCCCCGACGGG	18	48	11	2
tsrna-04587	CCTGTCACGCGGGAGACCGGGGTTCGATTC	15	63	6	0
tsrna-04586	CCTGTCACGCGGGAGACCGGGGTTCGA	14	50	8	5
tsrna-04585	CCTGTCACGCGGGAGACCGGGGTTC	18	47	7	2
tsrna-04584	CCTGTCACGCGGGAGACCGGGGTT	14	72	9	2
tsrna-04583	CCTGTCACGCGGGAGACCGGGGT	17	53	6	6
tsrna-04582	CCTGTCACGCGGGAGACCGGGG	13	55	7	0
tsrna-04581	CCTGTCACGCGGGAGACCGGG	15	58	3	4
tsrna-04580	CCTGTCACGCGGGAGACCGG	20	63	8	4
tsrna-04579	CCTGTCACGCGGGAGACCG	14	49	6	1
tsrna-04578	CCTGTCACGCGGGAGACC	13	45	5	3
tsrna-04577	CCTGTCACGCGGGAGAC	13	58	11	4
tsrna-04576	CCTGTCACGCGGGAGA	12	51	5	3
tsrna-04575	CCTGGTTTTCACCCAGGTGGCCCGG	2	1	1	0
tsrna-04574	CCTGGTTTTCACCCAGGTGGCCCG	1	3	4	0
tsrna-04573	CCTGGTTTTCACCCAGGCGGCCCGG	3	3	0	0
tsrna-04572	CCTGGTTCGATCCCGGGTTTCGGCACCA	37	86	12	14
tsrna-04571	CCTGGTTCGATCCCGGGTTTCGGCACC	6	4	3	3
tsrna-04570	CCTGGTTCGATCCCGGGTTTCGGCAC	0	1	0	0
tsrna-04568	CCTGGTTCGATCCCGGGTTTCGGC	1	0	0	0
tsrna-04566	CCTGGTTCAATCCCGGGTTTCGGCACCA	42	99	9	9
tsrna-04565	CCTGGTTCAATCCCGGGTTTCGGCA	0	0	0	0
tsrna-04561	CCTGGTGGTCTAGTGGTTAGGATTCGGCGCTCTC	34	40	27	18
tsrna-04560	CCTGGTGGTCTAGTGGTTAGGATTCGGCGCTCT	41	44	21	22
tsrna-04559	CCTGGTGGTCTAGTGGTTAGGATTCGGCGCTC	40	38	29	15
tsrna-04558	CCTGGTGGTCTAGTGGTTAGGATTCGGCGCT	37	41	19	14
tsrna-04557	CCTGGTGGTCTAGTGGTTAGGATTCGGCGC	17	22	18	13
tsrna-04556	CCTGGTGGTCTAGTGGTTAGGATTCGGCG	14	17	19	4
tsrna-04555	CCTGGTGGTCTAGTGGTTAGGATTCGGC	12	8	6	9
tsrna-04554	CCTGGTGGTCTAGTGGTTAGGATTCGG	3	6	2	3
tsrna-04553	CCTGGTGGTCTAGTGGTTAGGATTCG	3	2	0	2
tsrna-04552	CCTGGTGGTCTAGTGGTTAGGATTC	2	3	1	0
tsrna-04551	CCTGGTGGTCTAGTGGTTAGGATT	1	0	3	0
tsrna-04550	CCTGGTGGTCTAGTGGTTAGGAT	0	1	0	2
tsrna-04549	CCTGGTGGTCTAGTGGTTAGGA	0	1	0	0
tsrna-04545	CCTGGTGGTCTAGTGGCTAGGATTCGGCGC	8	34	8	37
tsrna-04544	CCTGGTGGTCTAGTGGCTAGGATTCGG	1	8	1	3
tsrna-04543	CCTGGTGGTCTAGTGGCTAGGATTCG	1	6	1	5
tsrna-04542	CCTGGTGGTCTAGTGGC	0	0	1	0
tsrna-04541	CCTGGTGGTCTAGTGG	0	1	0	0
tsrna-04539	CCTGGGTTCGAGCCCCAGTGGAACCACCA	39	31	13	24
tsrna-04538	CCTGGGTTCGAGCCCCAGTGGAACCACC	5	2	1	1
tsrna-04537	CCTGGGTTCGAGCCCCAGTGGAACCAC	1	0	0	0
tsrna-04536	CCTGGGTTCGAGCCCCAGTGGAACCA	0	2	0	0
tsrna-04535	CCTGGGTTCGAGCCCCAGTGGAACC	0	0	1	1
tsrna-04534	CCTGGGTTCGAATCCCAGCGGTGCCTCCA	28	46	7	4
tsrna-04533	CCTGGGTTCGAATCCCAGCGGTGCCTCC	1	1	0	1
tsrna-04530	CCTGGGTTCGAATCCCAGCGGTGCC	0	1	0	0
tsrna-04529	CCTGGGTTCGAATCCCAGCGGGGCCTCCA	0	2	0	2
tsrna-04526	CCTGGCTGGCTCGCCA	0	0	1	0
tsrna-04522	CCTGGATAGCTCAGTCGGTAG	0	0	0	0
tsrna-04520	CCTGGAGGCGTGGGTT	0	0	1	0
tsrna-04516	CCTGCCGGGGCTTCCA	0	1	0	0
tsrna-04514	CCTGCCGACTACGCCA	1	2	1	0
tsrna-04513	CCTGCCACGCGGGAGGCCCGGGTTC	3	14	4	0
tsrna-04512	CCTGCCACGCGGGAGGCCCGGGTT	5	23	2	0
tsrna-04511	CCTGCCACGCGGGAGGCCCGGGT	6	15	3	1
tsrna-04507	CCTGCCACGCGGGAGGCCC	0	0	0	0

tsrna-04503	CCTGAGTTCGAGCCTCAGAGAGGGCACCA	0	0	0	0
tsrna-04502	CCTGAGTTCGAGCCTCAGAGAGGGCACC	0	1	0	0
tsrna-04499	CCTGAGTTCGAACCTCAGAGGGGGCACCA	0	6	0	0
tsrna-04493	CCTCTTTTTACCACCA	0	1	0	1
tsrna-04490	CCTCTTCTTAACACCA	1	0	0	0
tsrna-04489	CCTCGTTAGTATAGTGGTGAGTATCCCCGCCT	3	3	0	0
tsrna-04488	CCTCGTTAGTATAGTGGTGAGTATCCCCGC	3	3	1	1
tsrna-04483	CCTCGTGGCGCAATGGTAGCGC	0	0	1	0
tsrna-04478	CCTCGGGTTCGATCCCCGACACCTCCACC	1	8	1	1
tsrna-04477	CCTCGCTGGGGCCTCCA	3	5	0	3
tsrna-04476	CCTCGCTGGGGCCTCC	0	4	0	0
tsrna-04469	CCTCCTAAGCCAGGGATTGTGGGT	120	431	91	39
tsrna-04468	CCTCCTAAGCCAGGGATTGTGGG	2	11	3	2
tsrna-04467	CCTCCTAAGCCAGGGATTGTGG	0	1	0	0
tsrna-04466	CCTCCTAAGCCAGGGATTGTG	1	0	1	0
tsrna-04464	CCTCCGGAGCTGGGGATTGTGGGT	1	1	0	0
tsrna-04462	CCTCCGGAGCTGGGGATTGTGG	0	1	0	0
tsrna-04461	CCTCCGGAGCTGGGGATTGTG	1	1	0	0
tsrna-04460	CCTCAGTAAGTTGCAATACTTAATTTCTGCCA	2	1	4	1
tsrna-04459	CCTCAGTAAGTTGCAATACTTAATTTCTGCC	0	0	0	0
tsrna-04457	CCTCAGTAAGTTGCAATACTTAATTTCTG	0	0	0	0
tsrna-04455	CCTCAGAGGGGGCACCA	0	1	0	0
tsrna-04454	CCTCAGAGAGGGCACCA	1	0	0	0
tsrna-04453	CCTCACCTGGAGCACCA	0	0	2	0
tsrna-04449	CCTCACACGCGAAAGGTCCCCGGT	6	26	2	1
tsrna-04448	CCTCACACGCGAAAGGTCCCCGG	0	1	0	0
tsrna-04447	CCTCACACGCGAAAGGTCCCCG	0	0	0	0
tsrna-04445	CCTCAAAGCAATACACTGAAAATGTTTAG	1	2	0	0
tsrna-04444	CCTCAAAGCAATACACTGAA	0	0	0	0
tsrna-04440	CCTACGTTGGGCGCCA	0	0	0	0
tsrna-04439	CCTAATGGATAAGGCATCAGCCT	4	0	2	0
tsrna-04438	CCTAATGGATAAGGCACTGG	76	42	55	22
tsrna-04437	CCTAAGCCAGGGATTGTGGGTTCGAGTCCCATCTGGGGTGC(	141	393	85	23
tsrna-04436	CCTAAGCCAGGGATTGTGGGTTCGAGTCCCATCTGGGGTGC(	132	423	98	40
tsrna-04435	CCTAAGCCAGGGATTGTGGGTTCGAGTCCCACCTGGGGTAC(	137	412	88	43
tsrna-04434	CCTAAGCCAGGGATTGTGGGTTCGAGTCCCACCCGGGGTAC(	132	424	90	49
tsrna-04433	CCTAAGCCAGGGATTGTGGGTTCGAG	135	420	97	44
tsrna-04432	CCTAAGCCAGGGATTGTGGGTTCG	146	416	106	37
tsrna-04431	CCTAAGCCAGGGATTGTGGGTTC	121	404	74	38
tsrna-04430	CCTAAGCCAGGGATTGTGGGTT	149	391	94	42
tsrna-04429	CCTAAGCCAGGGATTGTGGGT	122	399	82	29
tsrna-04428	CCTAAGCCAGGGATTGTGGG	4	8	1	0
tsrna-04427	CCTAAGCCAGGGATTGTGG	0	2	0	0
tsrna-04426	CCTAAGCCAGGGATTGTG	0	0	0	0
tsrna-04425	CCTAAGCCAGGGATTGT	1	1	0	0
tsrna-04414	CCGTGCGAGAATACCA	0	1	0	0
tsrna-04413	CCGTGATCGTATAGTGGTTAGTACTCTGCGTTGTG	23	18	10	9
tsrna-04412	CCGTGATCGTATAGTGGTTAGTACTCTGCGTTGT	42	14	7	5
tsrna-04411	CCGTGATCGTATAGTGGTTAGTACTCTGCGTTG	22	3	8	2
tsrna-04410	CCGTGATCGTATAGTGGTTAGTACTCTGCGTT	23	10	9	5
tsrna-04409	CCGTGATCGTATAGTGGTTAGTACTCTGCGT	4	6	6	1
tsrna-04408	CCGTGATCGTATAGTGGTTAGTACTCTGCG	6	1	2	2
tsrna-04407	CCGTGATCGTATAGTGGTTAGTACTCTGC	2	1	4	2
tsrna-04406	CCGTGATCGTATAGTGGTTAGTACTCTG	3	1	2	2
tsrna-04405	CCGTGATCGTATAGTGGTTAGTACTCT	1	1	0	0
tsrna-04404	CCGTGATCGTATAGTGGTTAGTACTC	1	0	0	0
tsrna-04403	CCGTGATCGTATAGTGGTTAGTACT	1	0	0	0
tsrna-04401	CCGTGATCGTATAGTGGTTAGTA	0	0	0	0
tsrna-04393	CCGTCTGTCACGCGGGAGACCGG	15	41	7	4
tsrna-04392	CCGTATGGAGGCGTGGGT	12	48	9	2
tsrna-04391	CCGTAGTGTAGTGGTTATCACGTTCGCCTC	2	1	0	0
tsrna-04390	CCGTAGTGTAGTGGTTATCACGTTCGCCT	2	2	1	0
tsrna-04389	CCGTAGTGTAGTGGTTATCACGTTCGCC	2	0	0	1
tsrna-04388	CCGTAGTGTAGTGGTTATCACGTT	1	0	1	0

tsrna-04387	CCGTAGTGTAGTGGTTATCACG	1	1	1	0
tsrna-04386	CCGTAGTGTAGTGGTTATCA	3	1	0	0
tsrna-04385	CCGTAGTGTAGTGGTTATC	2	0	1	0
tsrna-04384	CCGTAGTGTAGTGGTTAT	0	0	0	0
tsrna-04382	CCGTAGTGTAGTGGTCATCACGTTCGCCT	2	0	1	0
tsrna-04381	CCGTAGTGTAGTGGTCATCACGTTCGCC	0	6	0	1
tsrna-04380	CCGTAGTGTAGTGGTCATC	1	0	1	0
tsrna-04378	CCGTAGTGTAGCGGTTATCACATTCGCCTC	2	2	0	0
tsrna-04377	CCGTAGTGTAGCGGTTATCACATT	0	0	1	0
tsrna-04376	CCGTAGTGTAGCGGTTATC	0	0	0	0
tsrna-04375	CCGGTTCAAATCCGGGTGCCCCCTCCA	115	191	77	26
tsrna-04373	CCGGTTCAAATCCGGGTGCCCCC	0	0	0	0
tsrna-04372	CCGGTTAGCTCAGTTGGT	0	0	0	0
tsrna-04367	CCGGGTTTCGGCACCA	5	13	1	0
tsrna-04366	CCGGGTTCGATTCCCGGTCAGGGAACCA	10	314	5	2
tsrna-04365	CCGGGTTCGATTCCCGGTCAGGGAACC	2	15	0	0
tsrna-04364	CCGGGTTCGATTCCCGGTCAGGGAAC	0	5	0	0
tsrna-04363	CCGGGTTCGATTCCCGGTCAGGGAA	0	2	0	0
tsrna-04362	CCGGGTTCGATTCCCGGTCAGGGA	0	1	0	0
tsrna-04355	CCGGGTTCGATTCCCGGCCAGGGAACCA	0	16	0	2
tsrna-04354	CCGGGTTCGATTCCCGGCCAATGCACCA	47	152	18	11
tsrna-04353	CCGGGTTCGATTCCCGGCCAATGCACC	4	15	2	2
tsrna-04352	CCGGGTTCGATTCCCGGCCAACGCACCA	46	96	21	18
tsrna-04351	CCGGGTTCGATCCCCGGCATCTCCACCA	263	349	120	73
tsrna-04350	CCGGGTTCGATCCCCGGCATCTCCAC	1	1	3	0
tsrna-04349	CCGGGTTCGATCCCCAGCATCTCCACCA	20	19	3	2
tsrna-04348	CCGGGTTCGATCCCCAGCATCTCCACC	1	2	0	0
tsrna-04346	CCGGGTTCGACTCCCGGTGTGGGAACCA	61	263	21	21
tsrna-04345	CCGGGTTCGACTCCCGGTGTGGGAACC	4	7	1	0
tsrna-04344	CCGGGTTCGACTCCCGGTGTGGGAAC	0	3	0	0
tsrna-04342	CCGGGTTCGACTCCCGGTGTGGGA	1	0	0	0
tsrna-04337	CCGGGTTCGACTCCCGGTATGGGAACCA	10	79	2	1
tsrna-04336	CCGGGTTCGACTCCCGGTATGGGAAC	0	3	0	0
tsrna-04331	CCGGGTTCAATCCCCGGCACCTCCACCA	220	310	123	50
tsrna-04329	CCGGGTGCCCCCTCCA	1	1	0	0
tsrna-04328	CCGGGGTTCGATTCCCCGACGGGGAGCCA	2	6	1	0
tsrna-04326	CCGGGGTTCGATTCCCCGACGGGGAGC	0	0	0	0
tsrna-04325	CCGGGGTTCGATTCCCCGACGGGGAG	0	0	0	0
tsrna-04324	CCGGGGTTCGATTCCCCGACGGGGA	0	1	0	0
tsrna-04322	CCGGGGTTCGATTCCCCGACGGG	0	0	0	0
tsrna-04317	CCGGGCGGAAACACCA	16	18	2	1
tsrna-04316	CCGGGCAGAAGCACCA	1	5	1	0
tsrna-04315	CCGGCTCGGAGGACCA	0	0	1	0
tsrna-04314	CCGGCTCGAAGGACCA	0	2	0	0
tsrna-04313	CCGGCTAGCTCAGTCGGTAGAGCATGGGACTCTTAATCCC	59	40	75	12
tsrna-04312	CCGGCTAGCTCAGTCGGTAGAGCATGGGACTCTTAA	26	31	16	4
tsrna-04311	CCGGCTAGCTCAGTCGGTAGAGCATGGGACTCT	31	27	14	8
tsrna-04310	CCGGCTAGCTCAGTCGGTAGAGCATGGGACTC	28	29	23	6
tsrna-04309	CCGGCTAGCTCAGTCGGTAGAGCATGGGACT	23	33	18	9
tsrna-04308	CCGGCTAGCTCAGTCGGTAGAGCATGGGAC	16	16	10	4
tsrna-04307	CCGGCTAGCTCAGTCGGTAGAGCATGGGA	13	17	7	3
tsrna-04306	CCGGCTAGCTCAGTCGGTAGAGCATGGG	2	9	2	2
tsrna-04305	CCGGCTAGCTCAGTCGGTAGAGCATGG	3	7	3	2
tsrna-04304	CCGGCTAGCTCAGTCGGTAGAGCATGAGACTCTTAATCTC	99	50	96	19
tsrna-04303	CCGGCTAGCTCAGTCGGTAGAGCATGAGACTCTTAA	16	43	15	6
tsrna-04302	CCGGCTAGCTCAGTCGGTAGAGCATGAGACTCT	13	27	2	6
tsrna-04301	CCGGCTAGCTCAGTCGGTAGAGCATGAGACTC	19	35	10	5
tsrna-04300	CCGGCTAGCTCAGTCGGTAGAGCATGAGACT	15	21	9	7
tsrna-04299	CCGGCTAGCTCAGTCGGTAGAGCATGAGAC	10	21	8	4
tsrna-04298	CCGGCTAGCTCAGTCGGTAGAGCATGAGA	5	15	6	6
tsrna-04297	CCGGCTAGCTCAGTCGGTAGAGCATGAG	4	4	2	3
tsrna-04296	CCGGCTAGCTCAGTCGGTAGAGCATGA	3	3	3	1
tsrna-04295	CCGGCTAGCTCAGTCGGTAGAGCATG	5	1	0	1
tsrna-04294	CCGGCTAGCTCAGTCGGTAGAGCAT	3	5	0	2

tsrna-04293	CCGGCTAGCTCAGTCGGTAGAGCA	3	3	1	1
tsrna-04292	CCGGCTAGCTCAGTCGGTAGAGC	3	0	0	0
tsrna-04291	CCGGCTAGCTCAGTCGGTAGAG	2	3	0	0
tsrna-04290	CCGGCTAGCTCAGTCGGTAGA	3	2	0	0
tsrna-04289	CCGGCTAGCTCAGTCGGTAG	2	4	1	0
tsrna-04288	CCGGCTAGCTCAGTCGGTA	0	2	0	1
tsrna-04287	CCGGCTAGCTCAGTCGG	1	0	0	0
tsrna-04285	CCGGATGGAGGCGTGGGTTC	3	34	4	2
tsrna-04284	CCGGATGGAGGCGTGGGTT	6	27	5	1
tsrna-04283	CCGGATGGAGGCGTGGGT	5	37	0	0
tsrna-04282	CCGGATGGAGGCGTGGG	3	4	0	0
tsrna-04281	CCGGATGGAGGCGTGG	0	5	1	0
tsrna-04280	CCGGATCAGAAGATTGAGGGTTCGAG	1	8	0	1
tsrna-04279	CCGGATCAGAAGATTGAGGGTTCGA	5	5	2	0
tsrna-04278	CCGGATCAGAAGATTGAGGGTTCG	4	5	3	1
tsrna-04277	CCGGATCAGAAGATTGAGGGTT	2	8	8	1
tsrna-04276	CCGGATCAGAAGATTGAGGGT	3	0	3	0
tsrna-04270	CCGGATAGCTCAGTCGGTAGAGCATCAGACTTTTAATCTGA	75	45	51	28
tsrna-04269	CCGGATAGCTCAGTCGGTAGAGCATCAGACTTTT	38	33	30	17
tsrna-04268	CCGGATAGCTCAGTCGGTAGAGCATCAGACT	17	17	8	4
tsrna-04267	CCGGATAGCTCAGTCGGTAGAGCATCAGAC	11	12	7	4
tsrna-04266	CCGGATAGCTCAGTCGGTAGAGCATCAGA	8	4	4	1
tsrna-04265	CCGGATAGCTCAGTCGGTAGAGCATCAG	5	7	4	2
tsrna-04264	CCGGATAGCTCAGTCGGTAGAGCATCA	3	2	1	0
tsrna-04263	CCGGATAGCTCAGTCGGTAGAGCATC	2	3	3	0
tsrna-04262	CCGGATAGCTCAGTCGGTAGAGCAT	2	0	1	1
tsrna-04261	CCGGATAGCTCAGTCGGTAGAGCA	1	0	1	0
tsrna-04260	CCGGATAGCTCAGTCGGTAGAGC	1	4	0	0
tsrna-04257	CCGGATAGCTCAGTCGGTAG	1	0	0	0
tsrna-04256	CCGGATAGCTCAGTCGGT	0	0	1	0
tsrna-04253	CCGGAGCTGGGGATTGTGGGTTCG	0	2	0	1
tsrna-04252	CCGGAGCTGGGGATTGTGGGTT	0	2	1	0
tsrna-04251	CCGGAGCTGGGGATTGTGGGT	0	3	0	0
tsrna-04245	CCGGAGATGAAAACCTTTTTCCAAGGACACCA	13	10	24	1
tsrna-04244	CCGGAGATGAAAACCTTTTTCCAAGGACACC	1	6	6	0
tsrna-04243	CCGGAGATGAAAACCTTTTTCCAAGGACAC	0	2	1	0
tsrna-04242	CCGGAGATGAAAACCTTTTTCCAAGGACA	0	4	1	0
tsrna-04241	CCGGAGATGAAAACCTTTTTCCAAGGAC	0	1	0	1
tsrna-04240	CCGGAGATGAAAACCTTTTTCCAAGG	1	3	0	0
tsrna-04239	CCGGAGATGAAAACCTTTTTCCAAG	0	1	0	0
tsrna-04238	CCGGAGATGAAAACCTTTTTCCAA	1	3	0	0
tsrna-04237	CCGGAGATGAAAACCTTTTTCCA	1	2	0	0
tsrna-04236	CCGGAGATGAAAACCTTTTTCC	0	0	0	0
tsrna-04235	CCGGAGATGAAAACCTT	0	1	0	0
tsrna-04233	CCGCTGGTGTAGTGGTATCATGCAAGATTCCCATTC	44	8	54	1
tsrna-04232	CCGCTGGTGTAGTGGTATCATGCAAGATTCCC	14	2	15	3
tsrna-04231	CCGCTGGTGTAGTGGTATCATGCAAGATTC	7	2	5	0
tsrna-04230	CCGCTGGTGTAGTGGTATCATGCAAGATT	3	3	5	1
tsrna-04229	CCGCTGGTGTAGTGGTATCATGCAAGA	2	2	2	1
tsrna-04228	CCGCTGGTGTAGTGGTATCATGCAAG	3	1	0	1
tsrna-04227	CCGCTGGTGTAGTGGTATCATGCA	1	0	0	0
tsrna-04226	CCGCTGGTGTAGTGGTATCATGC	3	0	0	0
tsrna-04225	CCGCTGGTGTAGTGGTATCATG	1	1	1	0
tsrna-04223	CCGCGTGGCCTAATGGATAAGGCGTCTGA	0	0	0	0
tsrna-04222	CCGCGTGGCCTAATGGATAAGGCGTC	1	0	0	0
tsrna-04218	CCGCGGCCCGGGTTCGATTCCCGGTCAGGG	1	0	0	0
tsrna-04216	CCGCGGCCCGGGTTCGATTCCCGGTCAG	0	0	0	0
tsrna-04214	CCGCGGCCCGGGTTCGATTCCCG	0	0	0	0
tsrna-04206	CCGCCTGTCACGCGGGAGACCGGGGTTCG	17	51	2	3
tsrna-04205	CCGCCTGTCACGCGGGAGACCGGGGTT	13	47	6	2
tsrna-04204	CCGCCTGTCACGCGGGAGACCGGGGT	11	45	7	3
tsrna-04203	CCGCCTGTCACGCGGGAGACCGGGG	16	47	8	1
tsrna-04202	CCGCCTGTCACGCGGGAGACCGGG	18	42	3	4
tsrna-04201	CCGCCTGTCACGCGGGAGACCGG	17	56	3	8

tsrna-04200	CCGCCTGTCACGCGGGAGACCG	14	51	12	5
tsrna-04199	CCGCCTGTCACGCGGGAGACC	17	53	5	0
tsrna-04198	CCGCCTGTCACGCGGGAGAC	14	48	8	3
tsrna-04197	CCGCCTGTCACGCGGGA	11	37	5	5
tsrna-04196	CCGCCGCGGCCCGGGTTCGATTCCCGGTCAGGGAACCA	34	316	15	4
tsrna-04195	CCGCCGCGGCCCGGGTTCGATTCCCGGTCAGGGAACC	2	9	3	1
tsrna-04194	CCGCCGCGGCCCGGGTTCGATTCCCGGTCAGGGAAC	1	4	1	0
tsrna-04193	CCGCCGCGGCCCGGGTTCGATTCCCGGTCAGGGAA	0	2	0	0
tsrna-04192	CCGCCGCGGCCCGGGTTCGATTCCCGGTCAGGGA	0	2	0	0
tsrna-04191	CCGCCGCGGCCCGGGTTCGATTCCCGGTCAGGG	0	1	0	1
tsrna-04190	CCGCCGCGGCCCGGGTTCGATTCCCGGTCAGG	0	0	0	0
tsrna-04187	CCGCCGCGGCCCGGGTTCGATTCCCGGTC	0	0	1	0
tsrna-04185	CCGCCGCGGCCCGGGTTCGATTCCCG	0	0	0	0
tsrna-04184	CCGCCGCGGCCCGGGTTCGATTCC	0	1	0	0
tsrna-04182	CCGCCGCGGCCCGGGTTCGAT	0	0	0	0
tsrna-04176	CCGCAGCAACCTCGGTTCGAATCCGAGTCACGGCACCA	8	44	0	0
tsrna-04175	CCGCAGCAACCTCGGTTCGAATCCGAGTCACGGCACC	0	5	1	0
tsrna-04174	CCGCAGCAACCTCGGTTCGAATCCGAGTCACGGCAC	1	3	0	0
tsrna-04173	CCGCAGCAACCTCGGTTCGAATCCGAGTCACGGC	2	1	0	0
tsrna-04172	CCGCAGCAACCTCGGTTCGAATCCGAGTC	1	1	0	0
tsrna-04171	CCGCAGCAACCTCGGTTCGAATCCG	2	1	0	0
tsrna-04170	CCGCAGCAACCTCGGTTCG	1	1	0	0
tsrna-04168	CCGCAGCAACCTCGGT	0	1	0	0
tsrna-04167	CCGAGTTCGAATCTCGGTGGGACCTC	1	0	0	0
tsrna-04166	CCGAGTTCGAATCTCGGTGGGACC	0	0	0	0
tsrna-04165	CCGAGTTCAAATCTCGGTGGGACCTCCA	58	112	27	26
tsrna-04164	CCGAGTTCAAATCTCGGTGGGACCTCC	0	6	1	0
tsrna-04163	CCGAGTTCAAATCTCGGTGGGACCTC	1	2	0	0
tsrna-04162	CCGAGTTCAAATCTCGGTGGGACCT	0	0	0	0
tsrna-04161	CCGAGTTCAAATCTCGGTGGGACC	0	0	0	0
tsrna-04160	CCGAGTTCAAATCTCGGTGGAACCTCCA	160	229	68	29
tsrna-04159	CCGAGTTCAAATCTCGGTGGAACCTC	1	0	0	0
tsrna-04158	CCGAGTTCAAATCTCGGTGGAACCT	0	1	0	0
tsrna-04157	CCGAGTTCAAATCTCGGTGGAACC	0	1	0	0
tsrna-04155	CCGAGTGGTTAAGGCGTTGGACTT	0	1	0	0
tsrna-04150	CCGAGGTTGTGAGTTCG	0	0	0	0
tsrna-04140	CCGAGAGGTCCCGGGTTCAAATCCCGGACGAGCCCCCA	138	190	55	46
tsrna-04139	CCGAGAGGTCCCGGGTTCAAATCCCGGACGAGCCCCC	4	12	6	3
tsrna-04138	CCGAGAGGTCCCGGGTTCAAATCCCGGACGAGCCCC	3	6	1	0
tsrna-04137	CCGAGAGGTCCCGGGTTCAAATCCCGGACG	1	1	0	0
tsrna-04136	CCGAAGATCGCGGGTTCGAA	2	8	1	0
tsrna-04135	CCGAAGATCGCGGGTTC	2	6	2	0
tsrna-04134	CCGAAGATCGCGGGTT	2	5	0	0
tsrna-04133	CCGAAATAGCTCAGTTGGGAGAGC	6	1	3	3
tsrna-04132	CCGAAATAGCTCAGTTGGGAGA	3	3	1	1
tsrna-04131	CCGAAATAGCTCAGTTGG	2	4	0	2
tsrna-04130	CCGAAAGGTTGGTGGTTCGAGCCCACCCAGGGACGCCA	8	137	6	0
tsrna-04129	CCGAAAGGTTGGTGGTTCGAGCCCACCCAGGGACGCC	1	17	1	0
tsrna-04128	CCGAAAGGTTGGTGGTTCGAGCCCACCCAG	0	1	0	0
tsrna-04127	CCGAAAGGTTGGTGGTTCGAGC	0	2	0	0
tsrna-04126	CCGAAAGGTTGGTGGTTCGAG	0	0	1	0
tsrna-04125	CCGAAAGGTTGGTGGTTCGA	0	1	0	0
tsrna-04124	CCGAAAGGTTGGTGGTTCG	0	1	0	1
tsrna-04123	CCGAAAGGTTGGTGGTTC	0	0	0	0
tsrna-04120	CCGAAAATGTTGGTTATACCCTTCCCGTACTACCA	13	28	16	1
tsrna-04118	CCGAAAATGTTGGTTATACCCTTCCCGTA	0	0	0	0
tsrna-04117	CCGAAAATGTTGGTTATACCCTTCCCG	0	0	1	0
tsrna-04115	CCGAAAATGTTGGTTATACCCTTCC	0	1	0	0
tsrna-04112	CCGAAAATGTTGGTTATACCCT	0	0	0	0
tsrna-04110	CCGAAAATGTTGGTTATACC	0	2	0	0
tsrna-04108	CCGAAAATGTTGGTTATA	0	0	0	0
tsrna-04104	CCCTTCGTGGTTACCA	0	2	0	0
tsrna-04103	CCCTTCGTGGTCGCCA	0	1	0	0
tsrna-04102	CCCTTCGATAGCTCAGTTGGTAGAGC	14	10	7	3

tsrna-04101	CCCTTCGATAGCTCAGCTGGTAGAGC	41	44	20	23
tsrna-04100	CCCTTCCCGTACTACCA	1	1	0	0
tsrna-04098	CCCTGTTCGGGCGCCA	3	8	1	2
tsrna-04096	CCCTGTTCAGGCACCA	0	1	0	0
tsrna-04095	CCCTGTGGTCTAGTGGTTAGG	4	5	3	4
tsrna-04094	CCCTGTGGTCTAGTGGTTAG	4	3	6	1
tsrna-04093	CCCTGTGGTCTAGTGGC	1	6	0	1
tsrna-04091	CCCTGGTTCGATCCCGGGTTTCGGCACCA	43	128	5	6
tsrna-04090	CCCTGGTTCGATCCCGGGTTTCGGCACC	3	11	1	3
tsrna-04089	CCCTGGTTCGATCCCGGGTTTCGGCAC	0	1	0	0
tsrna-04087	CCCTGGTTCGATCCCGGGTTTCGGC	0	1	0	0
tsrna-04084	CCCTGGTTCGATCCCGGGTTTC	0	0	0	0
tsrna-04078	CCCTGGTTCAATCCCGGGTTTCGGCACCA	33	86	8	6
tsrna-04077	CCCTGGTTCAATCCCGGGTTTCGGCACC	7	7	1	0
tsrna-04076	CCCTGGTTCAATCCCGGGTTTCGGCAC	0	1	0	0
tsrna-04072	CCCTGGTTCAAATCCGGGTGCCCCCTCCA	157	201	64	17
tsrna-04071	CCCTGGTTCAAATCCGGGTGCCCCCTCC	4	6	1	1
tsrna-04070	CCCTGGTTCAAATCCGGGTGCCCCCTC	1	1	0	0
tsrna-04067	CCCTGGTGGTCTAGTGGTTAGGATTCGGCGCTCTCACC	54	49	41	24
tsrna-04066	CCCTGGTGGTCTAGTGGTTAGGATTCGGCGCTCTC	49	45	36	16
tsrna-04065	CCCTGGTGGTCTAGTGGTTAGGATTCGGCGCTCT	49	46	32	14
tsrna-04064	CCCTGGTGGTCTAGTGGTTAGGATTCGGCGCTC	41	52	29	14
tsrna-04063	CCCTGGTGGTCTAGTGGTTAGGATTCGGCGCT	38	45	33	17
tsrna-04062	CCCTGGTGGTCTAGTGGTTAGGATTCGGCGC	30	21	7	11
tsrna-04061	CCCTGGTGGTCTAGTGGTTAGGATTCGGCG	14	14	15	7
tsrna-04060	CCCTGGTGGTCTAGTGGTTAGGATTCGGC	14	10	6	6
tsrna-04059	CCCTGGTGGTCTAGTGGTTAGGATTCGG	4	8	1	5
tsrna-04058	CCCTGGTGGTCTAGTGGTTAGGATTCG	3	3	5	3
tsrna-04057	CCCTGGTGGTCTAGTGGTTAGGATTC	1	4	4	0
tsrna-04056	CCCTGGTGGTCTAGTGGTTAGGATT	4	3	1	1
tsrna-04055	CCCTGGTGGTCTAGTGGTTAGGAT	4	2	3	0
tsrna-04054	CCCTGGTGGTCTAGTGGTTAGGA	4	0	1	1
tsrna-04053	CCCTGGTGGTCTAGTGGTTAGG	1	0	0	0
tsrna-04052	CCCTGGTGGTCTAGTGGTTAG	1	2	2	0
tsrna-04051	CCCTGGTGGTCTAGTGGT	0	2	0	0
tsrna-04050	CCCTGGTGGTCTAGTGGCTAGGATTCGGCG	13	34	4	32
tsrna-04049	CCCTGGTGGTCTAGTGGCTAGGATTCGG	4	5	1	6
tsrna-04048	CCCTGGTGGTCTAGTGGCTAGGATTCG	5	7	2	6
tsrna-04047	CCCTGGTGGTCTAGTGGCTAGGATTC	0	7	2	4
tsrna-04046	CCCTGGTGGTCTAGTGGCTAGG	1	4	0	0
tsrna-04045	CCCTGGTGGTCTAGTGGC	2	1	1	1
tsrna-04044	CCCTGGTGGTCTAGTGG	0	1	0	0
tsrna-04043	CCCTGGTGGTCTAGTG	0	3	1	0
tsrna-04042	CCCTGGTCTAGTGGTTAGGATT	1	0	2	0
tsrna-04040	CCCTGGGTTCAATCCCCAGCACCTCCACC	0	2	0	0
tsrna-04039	CCCTGGCACCTCCACCA	0	0	0	0
tsrna-04038	CCCTGGAGGCGTGGGTTCGAAT	0	1	0	0
tsrna-04037	CCCTGGACTCTGAATCCAGC	1	0	0	1
tsrna-04036	CCCTGGACTCTGAATCC	1	0	0	0
tsrna-04034	CCCTGCTCGCTGCGCCA	2	6	2	0
tsrna-04032	CCCTCCGTGGTTACCA	0	0	0	0
tsrna-04030	CCCTCAGTAAGTTGCAATACTTAATTTCTGCCA	10	0	2	0
tsrna-04026	CCCGTACTGGCCACCA	0	1	0	1
tsrna-04025	CCCGTACGGGCCACCA	0	1	1	0
tsrna-04023	CCCGGTTCAAATCCGGGTGCCCCCT	0	0	0	0
tsrna-04021	CCCGGTGTGGGAACCA	0	2	0	1
tsrna-04020	CCCGGTCAGGGAACCA	1	7	0	0
tsrna-04019	CCCGGTATGGGAACCA	0	0	0	1
tsrna-04018	CCCGGTAATCGCATAAAACTTAAAACTTT	4	7	13	1
tsrna-04017	CCCGGTAATCGCATAAAACTTAAAACT	1	1	1	0
tsrna-04013	CCCGGGTTTCGGCACCA	22	41	9	8
tsrna-04012	CCCGGGTTTCGGCACC	2	2	0	1
tsrna-04011	CCCGGGTTCGATTCCCGGTCAGGGAACCA	5	304	6	7
tsrna-04010	CCCGGGTTCGATTCCCGGTCAGGGAACC	0	11	0	0

tsrna-04009	CCCGGGTTCGATTCCCGGTCAGGGAAC	0	2	0	0
tsrna-04008	CCCGGGTTCGATTCCCGGTCAGGGAA	0	1	0	0
tsrna-04007	CCCGGGTTCGATTCCCGGTCAGGG	0	2	0	0
tsrna-04005	CCCGGGTTCGATTCCCGGCCAATGCACCA	38	162	12	7
tsrna-04002	CCCGGGTTCGATCCCCGGCATCTCCACCA	245	343	148	70
tsrna-04001	CCCGGGTTCGATCCCCGGCATCTCCACC	14	14	7	2
tsrna-04000	CCCGGGTTCGATCCCCGGCATCTCCAC	0	1	1	0
tsrna-03999	CCCGGGTTCGATCCCCAGCATCTCCACCA	16	21	2	4
tsrna-03998	CCCGGGTTCGATCCCCAGCATCTCCACC	0	1	0	0
tsrna-03997	CCCGGGTTCGATCCCCAGCATCTCCAC	0	0	0	0
tsrna-03996	CCCGGGTTCGATCCCCAGCATCTCCA	0	1	0	0
tsrna-03992	CCCGGGTTCGACTCCCGGTGTGGGAACCA	63	299	29	21
tsrna-03991	CCCGGGTTCGACTCCCGGTGTGGGAACC	1	12	1	0
tsrna-03990	CCCGGGTTCGACTCCCGGTGTGGGAAC	0	2	0	0
tsrna-03989	CCCGGGTTCGACTCCCGGTGTGGGAA	0	5	0	0
tsrna-03988	CCCGGGTTCGACTCCCGGTGTGGGA	1	3	0	0
tsrna-03987	CCCGGGTTCGACTCCCGGTGTGGG	0	2	0	0
tsrna-03986	CCCGGGTTCGACTCCCGGTGTGG	0	1	0	0
tsrna-03984	CCCGGGTTCGACTCCCGGTATGGGAAC	0	1	0	0
tsrna-03983	CCCGGGTTCAATCCCCGGCATCTCCACCA	132	169	62	57
tsrna-03982	CCCGGGTTCAATCCCCGGCACCTCCACCA	232	361	123	55
tsrna-03980	CCCGGGTTCAAATCCCGGACGAGCCCCCA	116	186	43	49
tsrna-03979	CCCGGGTTCAAATCCCGGACGAGCCCCC	6	18	3	0
tsrna-03978	CCCGGGTTCAAATCCCGGACGAGCCCC	3	1	1	0
tsrna-03977	CCCGGGTTCAAATCCCGGACGAGCCC	0	1	1	2
tsrna-03975	CCCGGGCGGAAACACCA	12	24	5	1
tsrna-03974	CCCGGGCGGAAACACC	1	4	1	1
tsrna-03973	CCCGGCTCCTCCACCA	0	0	1	0
tsrna-03972	CCCGGCTAGCTCAGTCGGTAGAGCATGGGACTCTTAATCCC	50	49	72	16
tsrna-03971	CCCGGCTAGCTCAGTCGGTAGAGCATGGGACTCT	29	30	31	9
tsrna-03970	CCCGGCTAGCTCAGTCGGTAGAGCATGGGACTC	26	24	16	9
tsrna-03969	CCCGGCTAGCTCAGTCGGTAGAGCATGGGACT	20	36	14	8
tsrna-03968	CCCGGCTAGCTCAGTCGGTAGAGCATGGGAC	14	27	12	8
tsrna-03967	CCCGGCTAGCTCAGTCGGTAGAGCATGGGA	10	14	9	4
tsrna-03966	CCCGGCTAGCTCAGTCGGTAGAGCATGGG	1	8	4	3
tsrna-03965	CCCGGCTAGCTCAGTCGGTAGAGCATGG	3	2	2	6
tsrna-03964	CCCGGCTAGCTCAGTCGGTAGAGCATGAGACTC	22	31	9	6
tsrna-03963	CCCGGCTAGCTCAGTCGGTAGAGCATGAGACT	24	38	4	3
tsrna-03962	CCCGGCTAGCTCAGTCGGTAGAGCATGAGAC	16	26	4	6
tsrna-03961	CCCGGCTAGCTCAGTCGGTAGAGCATGAGA	6	15	6	4
tsrna-03960	CCCGGCTAGCTCAGTCGGTAGAGCATGAG	8	6	1	1
tsrna-03959	CCCGGCTAGCTCAGTCGGTAGAGCATGA	2	7	7	1
tsrna-03958	CCCGGCTAGCTCAGTCGGTAGAGCATG	2	4	0	0
tsrna-03957	CCCGGCTAGCTCAGTCGGTAGAGCAT	1	0	0	1
tsrna-03956	CCCGGCTAGCTCAGTCGGTAGAGCA	1	1	0	3
tsrna-03955	CCCGGCTAGCTCAGTCGGTAGAGC	4	3	1	0
tsrna-03954	CCCGGCTAGCTCAGTCGGTAGAG	0	0	1	0
tsrna-03953	CCCGGCTAGCTCAGTCGGTAGA	1	1	3	0
tsrna-03952	CCCGGCTAGCTCAGTCGGTAG	5	0	2	0
tsrna-03951	CCCGGCTAGCTCAGTCGGT	3	3	3	1
tsrna-03950	CCCGGCTAGCTCAGTCGG	2	0	0	0
tsrna-03948	CCCGGCTAGCTCAGTC	0	0	1	0
tsrna-03946	CCCGGCCCATGCACCA	1	1	0	0
tsrna-03944	CCCGGCCAATGCACCA	2	1	0	0
tsrna-03942	CCCGGCATCTCCACCA	0	3	0	0
tsrna-03941	CCCGGCACCTCCACCA	0	2	2	0
tsrna-03940	CCCGGATAGCTCAGTCGGTAGAGCATCAGACTTTT	57	26	55	23
tsrna-03939	CCCGGATAGCTCAGTCGGTAGAGCATCAGACTTT	54	31	37	4
tsrna-03938	CCCGGATAGCTCAGTCGGTAGAGCATCAGACT	29	31	29	6
tsrna-03937	CCCGGATAGCTCAGTCGGTAGAGCATCAGAC	14	9	7	1
tsrna-03936	CCCGGATAGCTCAGTCGGTAGAGCATCAGA	8	6	3	2
tsrna-03935	CCCGGATAGCTCAGTCGGTAGAGCATCAG	4	6	2	0
tsrna-03934	CCCGGATAGCTCAGTCGGTAGAGCATCA	6	8	4	1
tsrna-03933	CCCGGATAGCTCAGTCGGTAGAGCATC	6	4	1	1

tsrna-03932	CCCGGATAGCTCAGTCGGTAGAGCAT	3	0	0	0
tsrna-03931	CCCGGATAGCTCAGTCGGTAGAGCA	1	1	1	0
tsrna-03930	CCCGGATAGCTCAGTCGGTAGAGC	0	2	1	0
tsrna-03929	CCCGGATAGCTCAGTCGGTAGAG	0	0	0	0
tsrna-03928	CCCGGATAGCTCAGTCGGTAGA	1	0	1	1
tsrna-03927	CCCGGATAGCTCAGTCGGTAG	1	0	2	0
tsrna-03926	CCCGGATAGCTCAGTCGGTA	1	0	2	1
tsrna-03925	CCCGGATAGCTCAGTCGGT	0	0	1	0
tsrna-03923	CCCGGACGAGCCCCCA	6	11	1	5
tsrna-03920	CCCGCCTGTCACGCGGGAGACCGGGGTTCGATTC	12	52	6	2
tsrna-03919	CCCGCCTGTCACGCGGGAGACCGG	13	58	5	1
tsrna-03918	CCCGCCTGTCACGCGGGAGACCG	9	49	9	5
tsrna-03917	CCCGCATTCTCCACCA	0	2	1	0
tsrna-03914	CCCGACACCTCCACCA	0	1	0	0
tsrna-03913	CCCGAAAATGTTGGTTATACCCTTCCCGTACTACCA	11	20	16	0
tsrna-03912	CCCGAAAATGTTGGTTATACCCTTCCCGT	0	1	0	0
tsrna-03911	CCCGAAAATGTTGGTTATACCCTTCCC	0	0	0	0
tsrna-03910	CCCGAAAATGTTGGTTATACCCT	1	0	1	0
tsrna-03909	CCCGAAAATGTTGGTTATACCC	0	1	0	0
tsrna-03908	CCCGAAAATGTTGGTTATACC	0	0	0	0
tsrna-03902	CCCCTGTGGTCTAGTGGTTAGG	11	31	23	25
tsrna-03901	CCCCTGTGGTCTAGTGGTTAG	9	26	24	9
tsrna-03900	CCCCTGGCGGTCTAGTGGTTAGGATTCGGC	20	17	16	11
tsrna-03897	CCCCGTACTGGCCACCA	0	1	0	2
tsrna-03896	CCCCGTACGGGCCACCA	0	2	2	0
tsrna-03895	CCCCGTACGGGCCACC	0	0	1	0
tsrna-03893	CCCCGGTTCAAATCCGGGTGCCCCCTCCA	158	175	64	21
tsrna-03892	CCCCGGTTCAAATCCGGGTGCCCCCTCC	0	12	4	1
tsrna-03891	CCCCGGTTCAAATCCGGGTGCCCCCTC	0	1	0	0
tsrna-03888	CCCCGGGTTCGATCCCCGGCATCTCCACC	15	19	7	3
tsrna-03887	CCCCGGGTTCGATCCCCGGCATCTCCAC	1	0	2	0
tsrna-03886	CCCCGGGTTCGATCCCCGGCATCTCCA	0	1	0	0
tsrna-03885	CCCCGGGTTCGATCCCCGGCACCTCCACC	7	27	7	0
tsrna-03882	CCCCGGGTTCGACCCCCGGCTCCTCCACC	0	1	0	0
tsrna-03881	CCCCGGGTTCGACCCCCGGCTCCTCCA	0	0	0	0
tsrna-03880	CCCCGGGTTCAATCCCCGGCATCTCCACC	3	4	2	0
tsrna-03878	CCCCGGGTTCAATCCCCGGCACCTCCACC	6	18	6	2
tsrna-03877	CCCCGGGTTCAATCCCCGGCACCTCCAC	0	0	1	0
tsrna-03876	CCCCGGGTTCAATCCCCGGCACCTCCA	0	0	1	0
tsrna-03873	CCCCGGCTCCTCCACCA	0	1	0	0
tsrna-03871	CCCCGGCATCTCCACCA	1	3	0	0
tsrna-03870	CCCCGGCATCTCCACC	0	1	0	0
tsrna-03869	CCCCGGCACCTCCACCA	2	1	1	1
tsrna-03867	CCCCGCGCAGGTTCGAATCCTGCTCACAGC	1	4	0	0
tsrna-03866	CCCCGCGCAGGTTCGAATCCTGCC	0	0	0	0
tsrna-03865	CCCCGCGCAGGTTCGAATCCTGC	0	2	0	0
tsrna-03862	CCCCGCCTGTCACGCGGGAGACCGGGGTTCGATTC	19	62	4	7
tsrna-03861	CCCCGCCTGTCACGCGGGAGACCGG	18	50	9	3
tsrna-03860	CCCCGCCTGTCACGCGGGAGACCG	19	52	9	0
tsrna-03857	CCCCGAAAATGTTGGTTATACCCTTCCCGTACTACCA	18	21	13	1
tsrna-03856	CCCCGAAAATGTTGGTTATACCCTTCCCGTACTACC	1	5	3	0
tsrna-03855	CCCCGAAAATGTTGGTTATACCCTTCCCGT	0	0	0	0
tsrna-03854	CCCCGAAAATGTTGGTTATACCC	0	0	0	0
tsrna-03848	CCCCCGGCTCCTCCACCA	15	26	5	3
tsrna-03845	CCCCCATGTCTAACAACATGGCTTTCTCACC	1	0	0	0
tsrna-03844	CCCCCATGTCTAACAACATGGCTTTCTCAC	2	0	0	0
tsrna-03843	CCCCCATGTCTAACAACATGGCT	0	0	1	0
tsrna-03842	CCCCCATGTCTAACAACATGGC	0	1	0	0
tsrna-03834	CCCCATGGTGTAATGGTTAGCACTCTGGAC	3	1	0	0
tsrna-03833	CCCCATGGTGTAATGGTTAGCACTCTGG	0	1	0	0
tsrna-03828	CCCCAGTGGAACCACCA	0	1	3	0
tsrna-03825	CCCCAGTACCTCCACC	0	0	1	0
tsrna-03824	CCCCAGCATCTCCACCA	0	0	0	0
tsrna-03822	CCCCAGCACCTCCACCA	0	1	0	0

tsrna-03821	CCCCACTTCTGGTACCA	1	0	0	0
tsrna-03818	CCCCACGTTGGGCGCCA	6	11	6	2
tsrna-03817	CCCCACGTTGGGCGCC	0	2	0	1
tsrna-03815	CCCCAAAAATTTTGGTGCAACTCCAAATAAAAGTACCA	24	94	36	2
tsrna-03814	CCCCAAAAATTTTGGTGCAACTCCAAATAAAAGTAC	21	76	11	4
tsrna-03813	CCCCAAAAATTTTGGTGCAACTCCAAATA	7	25	5	2
tsrna-03812	CCCCAAAAATTTTGGTGCAACTCCAAA	1	2	3	1
tsrna-03811	CCCCAAAAATTTTGGTGCAACTCCAA	1	1	3	0
tsrna-03809	CCCATTCTTGCGACCCGGGTTCGATTCCCGGGCGGCGCACCA	26	66	14	4
tsrna-03808	CCCATTCTTGCGACCCGGGTTCGATTCCCGGGCGGCG	6	5	1	0
tsrna-03807	CCCATTCTTGCGACCCGGGTTCGATTCCCGGGCGGC	1	5	2	1
tsrna-03806	CCCATTCTTGCGACCCGGGTTCGATTCCCGGGCGG	2	12	0	0
tsrna-03805	CCCATTCTTGCGACCCGGGTTCGATTCCCG	0	11	0	0
tsrna-03803	CCCATTCTTGCGACCCGGGTTC	0	0	2	0
tsrna-03802	CCCATTCTTGCGACCCGGGTT	2	1	0	0
tsrna-03801	CCCATTCTTGCGACCCGGGT	0	1	0	0
tsrna-03799	CCCATTCTTGCGACCCGG	0	0	1	0
tsrna-03797	CCCATGTCTAACAACATGGCTTTCTCACC	0	1	0	0
tsrna-03792	CCCATGGTGTAATGGTTAGCACTCTGGACT	2	2	0	0
tsrna-03791	CCCATGGTGTAATGGTTAGCACTCTGGAC	1	1	0	1
tsrna-03790	CCCATGGTGTAATGGTTAGCACTCTGGA	0	1	0	1
tsrna-03789	CCCATGGTGTAATGGTTAGCACTCTGG	1	0	1	0
tsrna-03787	CCCATGGTGTAATGGTTAGCACTCT	1	0	0	0
tsrna-03783	CCCATCTGGGTCGCCA	0	2	0	0
tsrna-03782	CCCATCTGGGGTGCCA	0	0	0	0
tsrna-03781	CCCATCCTCGTCGCCA	1	0	0	0
tsrna-03780	CCCATATGGTCTAGCGGTTAGGATTCCTGG	4	4	2	0
tsrna-03779	CCCATATGGTCTAGCGGTTAGGATTCCTG	0	0	1	0
tsrna-03778	CCCATATGGTCTAGCGGTTAGGATTCCT	0	0	1	1
tsrna-03777	CCCATATGGTCTAGCGGTTAGGATTCC	0	0	0	0
tsrna-03776	CCCATATGGTCTAGCGGTTAGGATTC	0	0	1	0
tsrna-03775	CCCATATGGTCTAGCGGTTAGGATT	0	0	0	1
tsrna-03771	CCCATATGGTCTAGCG	0	0	1	0
tsrna-03770	CCCATACCCCGAAAATGTTGGTTATACCCTTCCCGTACTACCA	15	13	22	2
tsrna-03769	CCCATACCCCGAAAATGTTGGTTA	0	0	0	0
tsrna-03766	CCCATAACCCAGAGGTCGATGGATCG	8	12	2	1
tsrna-03765	CCCATAACCCAGAGGTCGATGGATC	1	11	1	2
tsrna-03764	CCCATAACCCAGAGGTCGATGGAT	4	14	0	1
tsrna-03763	CCCATAACCCAGAGGTCGATGGA	3	6	1	1
tsrna-03762	CCCATAACCCAGAGGTCGATGG	0	2	3	1
tsrna-03761	CCCATAACCCAGAGGTCGATG	0	1	0	0
tsrna-03760	CCCATAACCCAGAGGTCGAT	0	1	1	0
tsrna-03757	CCCAGTGGCCTAATGGATAAGGCATCAGCC	5	3	3	0
tsrna-03753	CCCAGTAGAGCCTCCA	2	1	0	0
tsrna-03751	CCCAGGTGGCCCGGGTTCGACTCCCGGT	0	1	0	0
tsrna-03747	CCCAGGTGGCCCGGGT	0	1	0	0
tsrna-03743	CCCAGGCGGCCCGGGTTCGACTCCCGGTGTGGGAACCA	83	300	38	20
tsrna-03742	CCCAGGCGGCCCGGGTTCGACTCCCGGTGTGGGAACC	9	21	4	0
tsrna-03741	CCCAGGCGGCCCGGGTTCGACTCCCGGTGTGGGAAC	1	2	3	0
tsrna-03740	CCCAGGCGGCCCGGGTTCGACTCCCGGTGTGGG	0	1	0	0
tsrna-03739	CCCAGGCGGCCCGGGTTCGACTCCCGGTGTGG	0	1	0	0
tsrna-03738	CCCAGGCGGCCCGGGTTCGACTCCCGGTGTG	0	0	0	0
tsrna-03735	CCCAGGCGGCCCGGGTTCGACTCC	0	0	0	0
tsrna-03725	CCCAGCGAGGCCTCCA	1	1	0	0
tsrna-03724	CCCAGCATCTCCACCA	0	1	1	0
tsrna-03723	CCCAGCACCTCCACCA	0	0	0	0
tsrna-03722	CCCAGAGGTCGATGGATCGAAACCATCCTC	1	1	0	0
tsrna-03721	CCCAGAGGTCGATGGATCGAAACCAT	0	3	0	1
tsrna-03720	CCCAGAGGTCGATGGATCGAAACCA	1	0	1	0
tsrna-03719	CCCAGAGGTCGATGGATCGAAACC	0	2	0	0
tsrna-03718	CCCAGAGGTCGATGGATCGAAAC	3	2	0	0
tsrna-03717	CCCAGAGGTCGATGGATCGAAA	1	1	0	0
tsrna-03716	CCCAGAGGTCGATGGATCGAA	0	2	1	0
tsrna-03715	CCCAGAGGTCGATGGATCGA	1	2	1	0

tsrna-03714	CCCAGAGGTCGATGGATCG	1	3	1	0
tsrna-03713	CCCAGAGGTCGATGGATC	1	3	2	0
tsrna-03712	CCCAGAGGTCGATGGAT	1	2	0	0
tsrna-03711	CCCAGAGGTCGATGGA	1	2	1	0
tsrna-03709	CCCACTTCTGACACCA	1	1	0	1
tsrna-03707	CCCACTCCTGACACCA	1	1	0	1
tsrna-03706	CCCACGTTGGGCGCCA	5	5	2	3
tsrna-03705	CCCACCTTCGTCGCCA	0	1	0	0
tsrna-03703	CCCACCTGGGGTACCA	0	1	0	0
tsrna-03698	CCCACCCAGGGACGCCA	2	2	0	0
tsrna-03697	CCCACCCAGGGACGCC	0	0	0	1
tsrna-03693	CCCACATGGTCTAGCGGTTAGGATTCCTGGTTTTC	7	9	8	5
tsrna-03692	CCCACATGGTCTAGCGGTTAGGATTCCTGGTTTT	6	7	3	1
tsrna-03691	CCCACATGGTCTAGCGGTTAGGATTCCTGGTTT	5	3	6	6
tsrna-03690	CCCACATGGTCTAGCGGTTAGGATTCCTGGTT	5	5	1	3
tsrna-03689	CCCACATGGTCTAGCGGTTAGGATTCCTGGT	6	5	4	2
tsrna-03688	CCCACATGGTCTAGCGGTTAGGATTCCTGG	1	4	2	0
tsrna-03687	CCCACATGGTCTAGCGGTTAGGATTCCTG	1	1	2	0
tsrna-03686	CCCACATGGTCTAGCGGTTAGGATTCCT	4	0	0	2
tsrna-03685	CCCACATGGTCTAGCGGTTAGGATTCC	1	0	0	0
tsrna-03684	CCCACATGGTCTAGCGGTTAGGATTC	2	0	1	0
tsrna-03683	CCCACATGGTCTAGCGGTTAGGATT	1	0	1	0
tsrna-03682	CCCACATGGTCTAGCGGTTAGGAT	0	0	0	0
tsrna-03681	CCCACATGGTCTAGCGGTTAGGA	0	0	2	0
tsrna-03680	CCCACATGGTCTAGCGGTTAGG	0	0	0	1
tsrna-03679	CCCACATGGTCTAGCGGTTAG	0	0	0	0
tsrna-03678	CCCACATGGTCTAGCGGTTA	0	0	0	1
tsrna-03675	CCCACATGGTCTAGCG	0	0	0	0
tsrna-03674	CCCAATACCTCCACCA	0	0	0	1
tsrna-03673	CCCAACTTACACTTAGGAGATTTCAACTTAACTTGACCGCTCT	16	9	18	0
tsrna-03672	CCCAACTTACACTTAGGAGATTTCAACTTAACTTGACCGCTCT	7	6	15	0
tsrna-03671	CCCAACTTACACTTAGGAGATTTCAACTTAACTTGACCGCTCT	4	3	13	0
tsrna-03670	CCCAACTTACACTTAGGAGATTTCAACTTAACTTGACCGCTCT	1	2	11	0
tsrna-03669	CCCAACTTACACTTAGGAGATTTCAACTT	0	0	0	0
tsrna-03668	CCCAACTTACACTTAGGAGATTTCAACT	0	0	0	0
tsrna-03658	CCCAAAAATTTTGGTGCAACTCCAAATAA	1	2	4	0
tsrna-03657	CCCAAAAATTTTGGTGCAACT	0	1	0	0
tsrna-03656	CCATTTCATAACTTTGTCAAAGTTAAATT	1	3	2	0
tsrna-03655	CCATTTCATAACTTTGTCAAAG	1	0	0	0
tsrna-03654	CCATTTCATAACTTTGTCA	2	1	1	0
tsrna-03653	CCATTGTGCTTTGCACGCGTGGGTTCGAATCCCATCCTCGTC	2	12	3	0
tsrna-03652	CCATTGTGCTCTGCACGCGTGGGT	1	6	2	0
tsrna-03647	CCATTGGGGTTTCCCCGCGCAGGT	0	1	0	0
tsrna-03644	CCATTCTTGCGACCCGGGTTCGATTCCCGGGCGGCGCACCA	32	81	11	1
tsrna-03643	CCATTCTTGCGACCCGGGTTCGATTCCCGGGCGGCGC	3	9	4	0
tsrna-03642	CCATTCTTGCGACCCGGGTTCG	2	7	1	0
tsrna-03641	CCATTCTTGCGACCCGGGTTC	1	1	0	0
tsrna-03640	CCATTCTTGCGACCCGGGTT	0	3	0	0
tsrna-03639	CCATTCTTGCGACCCGGGT	0	3	0	0
tsrna-03637	CCATTCTTGCGACCCGG	0	0	0	0
tsrna-03635	CCATGTCTAACAACATGGCTTTCTCACCA	2	2	1	0
tsrna-03634	CCATGTCTAACAACATGGCTTTCTCACC	0	0	0	0
tsrna-03630	CCATGGTGTAATGGTTAGCACTCTGGACTT	1	1	1	0
tsrna-03629	CCATGGTGTAATGGTTAGCACTCTGGACTC	0	0	0	1
tsrna-03628	CCATGGTGTAATGGTTAGCACTCTGGACT	0	3	0	0
tsrna-03627	CCATGGTGTAATGGTTAGCACTCTGGAC	1	1	0	3
tsrna-03626	CCATGGTGTAATGGTTAGCACTCTGGA	1	0	2	0
tsrna-03616	CCATGGTGTAATGGTGAGCACTCTGG	0	0	0	0
tsrna-03611	CCATGATCGTATAGTGGTTAGTACTCTGCG	2	3	2	5
tsrna-03610	CCATGATCGTATAGTGGTTAGTACTCTGC	2	3	0	0
tsrna-03609	CCATGATCGTATAGTGGTTAGTACTCT	0	2	0	0
tsrna-03608	CCATCCTCTGCTACCA	0	0	0	1
tsrna-03607	CCATCCAGGGACGCCA	0	0	0	1
tsrna-03606	CCATATGGTCTAGCGGTTAGGATTCCTGGT	6	13	0	1

tsrna-03605	CCATATGGTCTAGCGGTTAGGATTCCTGG	5	0	4	1
tsrna-03604	CCATATGGTCTAGCGGTTAGGATTCCTG	0	2	2	1
tsrna-03603	CCATATGGTCTAGCGGTTAGGATTCCT	2	0	2	0
tsrna-03602	CCATATGGTCTAGCGGTTAGGATTCC	1	0	0	0
tsrna-03601	CCATATGGTCTAGCGGTTAGGATTC	0	0	1	1
tsrna-03598	CCATAGTGTAGTGGTTATCACGTCTGCTT	1	0	0	0
tsrna-03596	CCATAGTGTAGTGGTTATCACGTC	0	1	0	0
tsrna-03595	CCATAGTGTAGTGGTTATCACGT	0	0	0	0
tsrna-03592	CCATAGTGTAGCGGTTATCACGTCTGCTTT	0	0	1	0
tsrna-03591	CCATAGTGTAGCGGTTATCACGTCTGCTT	0	0	1	0
tsrna-03590	CCATACCCCGAAAATGTTGGTTATACCCTT	0	0	1	0
tsrna-03587	CCATAACCCAGAGGTCGATGGATCG	4	8	1	0
tsrna-03586	CCATAACCCAGAGGTCGATGGATC	2	8	2	2
tsrna-03585	CCATAACCCAGAGGTCGATGGAT	2	4	1	2
tsrna-03584	CCATAACCCAGAGGTCGATGGA	1	5	2	3
tsrna-03583	CCATAACCCAGAGGTCGATGG	0	1	0	3
tsrna-03582	CCATAACCCAGAGGTCGATG	2	0	0	1
tsrna-03581	CCATAACCCAGAGGTCGAT	1	0	0	0
tsrna-03580	CCATAACCCAGAGGTCGA	1	0	0	0
tsrna-03578	CCAGTTGATTAGGGTGCTTAGCT	0	0	0	0
tsrna-03571	CCAGTCTCTTCGGGGGCGTGGGTTCGA	2	2	0	1
tsrna-03570	CCAGTCTCTTCGGGGGCGTGGGT	0	4	0	0
tsrna-03567	CCAGTCTCTTCGGGGGCGTG	1	0	0	0
tsrna-03562	CCAGGTTCGACTCCTGGCTGGCTCGCCA	33	136	15	5
tsrna-03561	CCAGGTTCGACTCCTGGCTGGCTCGCC	3	2	0	0
tsrna-03559	CCAGGTTCGACTCCTGGCTGGCTCG	0	1	0	0
tsrna-03555	CCAGGGTTCAAGTCCCTGTTCGGGCGCCA	5	15	5	5
tsrna-03554	CCAGGGTTCAAGTCCCTGTTCGGGCGCC	1	0	0	0
tsrna-03553	CCAGGGTTCAAGTCCCTGTTCGGGCGC	0	1	0	0
tsrna-03548	CCAGGGTCGTGGGTTCGAGCCCCACGTTGGGC	0	0	0	0
tsrna-03545	CCAGGGGTCGCGAGTTCGATCCTCGCTGGG	0	1	0	0
tsrna-03544	CCAGGGGTCGCGAGTTCAAATCTCGCTGGGGCCTCC	5	5	0	1
tsrna-03543	CCAGGGGTCGCGAGTTCAAATCTCGCTGGGGCCT	0	3	0	0
tsrna-03542	CCAGGGGTCGCGAGTTCAAATCTCGCTGGG	0	1	1	0
tsrna-03541	CCAGGGGTCGCGAGTTCAAA	0	1	0	0
tsrna-03539	CCAGGGGTCGCGAGTTCA	1	0	0	0
tsrna-03538	CCAGGGGTCGCGAGTTC	0	1	0	0
tsrna-03537	CCAGGGGTCGCGAGTT	0	1	0	0
tsrna-03536	CCAGGGATTGTGGGTTCGAGTCCCATCTGGGGTGCCA	5	15	2	0
tsrna-03535	CCAGGGATTGTGGGTTCGAGTCCCATCTGGGGTGCC	4	8	1	1
tsrna-03534	CCAGGGATTGTGGGTTCGAGTCCCATCTGG	6	8	4	1
tsrna-03533	CCAGGGATTGTGGGTTCGAGTCCC	6	9	1	1
tsrna-03532	CCAGGGATTGTGGGTTCGAGTCC	1	6	1	0
tsrna-03531	CCAGGGATTGTGGGTTCGA	0	8	2	1
tsrna-03530	CCAGGGATTGTGGGTTCG	3	8	1	0
tsrna-03529	CCAGGGATTGTGGGTTC	2	6	4	1
tsrna-03528	CCAGGCGGCCCGGGTTCGACTCCCGGTGTGGGAACCA	70	325	41	21
tsrna-03527	CCAGGCGGCCCGGGTTCGACTCCCGGTGTGGGAACC	10	19	6	0
tsrna-03526	CCAGGCGGCCCGGGTTCGACTCCCGGTGTGGGAAC	4	3	0	0
tsrna-03525	CCAGGCGGCCCGGGTTCGACTCCCGGTGTGGGAA	0	0	0	0
tsrna-03524	CCAGGCGGCCCGGGTTCGACTCCCGGTGTGGGA	0	2	1	0
tsrna-03523	CCAGGCGGCCCGGGTTCGACTCCCGGTGTGGG	0	0	0	0
tsrna-03521	CCAGGCGGCCCGGGTTCGACTCCCGGTGTG	0	1	0	0
tsrna-03520	CCAGGCGGCCCGGGTTCGACTCCCGGTGT	0	1	0	0
tsrna-03508	CCAGCTTTGGGGGGTTCG	1	0	1	0
tsrna-03501	CCAGATCAGAAGGTTGCGTGTTCA	0	2	1	1
tsrna-03500	CCAGATCAGAAGGTTGCGTGTTC	2	1	1	1
tsrna-03499	CCAGATCAGAAGGTTGCGTGTT	3	6	0	0
tsrna-03498	CCAGATCAGAAGGTTGCGTGT	1	0	0	0
tsrna-03497	CCAGATCAGAAGGTTGCGTG	0	1	0	0
tsrna-03494	CCAGATCAGAAGGCTGCGTGTTCGAA	0	0	0	0
tsrna-03493	CCAGATCAGAAGGCTGCGTGTTCG	0	1	0	0
tsrna-03492	CCAGATCAGAAGGCTGCGTGTTC	1	0	0	0
tsrna-03491	CCAGATCAGAAGGCTGCGTGTT	0	0	1	0

tsrna-03490	CCAGATCAGAAGGCTGCGTGT	0	1	0	0
tsrna-03485	CCAGAGGTCGATGGATCGAAACCA	2	1	0	0
tsrna-03484	CCAGAGGTCGATGGATCGAAACC	0	3	0	0
tsrna-03483	CCAGAGGTCGATGGATCGAAAC	0	1	1	0
tsrna-03482	CCAGAGGTCGATGGATCGAAA	0	2	0	0
tsrna-03481	CCAGAGGTCGATGGATCGAA	0	1	0	0
tsrna-03480	CCAGAGGTCGATGGATCGA	1	2	0	1
tsrna-03479	CCAGAGGTCGATGGATCG	0	1	2	0
tsrna-03477	CCAGAGGTCGATGGAT	1	1	0	1
tsrna-03476	CCAGACTCAAGTTCTGGTCTCCGGATGGAG	0	3	0	1
tsrna-03475	CCAGACTCAAGTTCTGGTCTCCGG	1	3	0	0
tsrna-03474	CCAGACTCAAGTTCTGGTCTCC	1	4	3	0
tsrna-03473	CCAGAATGGCCGAGTGGTTAAGGC	0	1	0	0
tsrna-03471	CCACTGTAAAGCTAACTTAGCATTAACCTT	8	13	6	10
tsrna-03470	CCACTGTAAAGCTAACTTAGCATTAACC	4	9	8	7
tsrna-03469	CCACTGTAAAGCTAACTTAGCATTAAC	8	5	6	9
tsrna-03468	CCACTGTAAAGCTAACTTAGCATT	6	3	0	7
tsrna-03467	CCACTGTAAAGCTAACTTAGCA	3	2	4	6
tsrna-03466	CCACTGTAAAGCTAACTTAGC	5	2	3	4
tsrna-03465	CCACTGTAAAGCTAACT	1	0	0	0
tsrna-03464	CCACTGTAAAGCTAAC	0	0	0	0
tsrna-03462	CCACGCGGGAGGCCCGGGTTCGATTCCCGGCCCATGCACCA	18	38	15	11
tsrna-03461	CCACGCGGGAGGCCCGGGTTCGATTCCCGGCCCATGCACC	6	11	0	0
tsrna-03460	CCACGCGGGAGGCCCGGGTTCGATTCCCGGCCAATGCACCA	39	168	11	14
tsrna-03459	CCACGCGGGAGGCCCGGGTTCGATTCCCGGCCAATGCACC	6	8	1	0
tsrna-03458	CCACGCGGGAGGCCCGGGTTCGATTCCCGGCCAATGC	1	5	1	0
tsrna-03457	CCACGCGGGAGGCCCGGGTTCGATTCCCGGCCAATG	3	4	0	0
tsrna-03456	CCACGCGGGAGGCCCGGGTT	1	1	0	0
tsrna-03455	CCACGCGGGAGGCCCGGGT	0	6	1	0
tsrna-03453	CCACCCAGGGACGCCA	1	0	0	0
tsrna-03452	CCACATGGTCTAGCGGTTAGGATTCCTGGTTTTC	11	9	3	4
tsrna-03451	CCACATGGTCTAGCGGTTAGGATTCCTGGTTTT	4	4	5	1
tsrna-03450	CCACATGGTCTAGCGGTTAGGATTCCTGGTTT	4	10	4	3
tsrna-03449	CCACATGGTCTAGCGGTTAGGATTCCTGGTT	4	6	2	0
tsrna-03448	CCACATGGTCTAGCGGTTAGGATTCCTGGT	5	7	3	0
tsrna-03447	CCACATGGTCTAGCGGTTAGGATTCCTGG	4	3	3	2
tsrna-03446	CCACATGGTCTAGCGGTTAGGATTCCTG	3	3	1	0
tsrna-03445	CCACATGGTCTAGCGGTTAGGATTCCT	0	2	0	1
tsrna-03443	CCACATGGTCTAGCGGTTAGGATTC	0	0	1	0
tsrna-03442	CCACATGGTCTAGCGGTTAGGATT	1	0	0	0
tsrna-03441	CCACATGGTCTAGCGGTTAGGAT	0	0	1	0
tsrna-03435	CCAATGGAGGCGTGGGTTCGAATCCCACTTCTGACACCA	26	66	20	6
tsrna-03434	CCAATGGAGGCGTGGGTT	8	21	7	1
tsrna-03433	CCAATGGAGGCGTGGGT	8	14	3	1
tsrna-03432	CCAATGGAGGCGTGGG	2	0	0	0
tsrna-03430	CCAAGGTCGCGGGTTCGAT	2	0	0	0
tsrna-03429	CCAAGGTCGCGGGTTCG	0	2	0	1
tsrna-03428	CCAAGGTCGCGGGTTC	0	1	0	0
tsrna-03427	CCAAGCAGTTGACCCGGGTTCGATTCCCGGCCAACGCACCA	65	112	23	15
tsrna-03426	CCAAGCAGTTGACCCGGGTTCGATTCCCGGCCAACGCACC	10	24	4	3
tsrna-03425	CCAAGCAGTTGACCCGGGTTCG	0	4	0	0
tsrna-03424	CCAAGCAGTTGACCCGGGTTC	2	1	2	0
tsrna-03423	CCAAGCAGTTGACCCGGGTT	0	1	1	0
tsrna-03422	CCAAGCAGTTGACCCGGGT	1	2	0	0
tsrna-03419	CCAACTTACACTTAGGAGATTTCAACTTAACTTGACCGCTCTG	94	47	122	20
tsrna-03418	CCAACTTACACTTAGGAGATTTCAACTTAACTTGACCGCTCTG	16	8	26	1
tsrna-03417	CCAACTTACACTTAGGAGATTTCAACTTAACTTGACCGCTCTG	6	2	12	1
tsrna-03416	CCAACTTACACTTAGGAGATTTCAACTTAACTTGACCGCTCTG	5	4	18	1
tsrna-03415	CCAACTTACACTTAGGAGATTTCAACTTAACTTGACCGCTCTG	2	4	7	0
tsrna-03414	CCAACTTACACTTAGGAGATTTCAACTTAA	0	0	1	0
tsrna-03413	CCAACTTACACTTAGGAGATTTCAACTTA	0	0	3	0
tsrna-03412	CCAACTTACACTTAGGAGATTTCAA	0	0	0	0
tsrna-03411	CCAACTTACACTTAGGAGATTTCA	0	0	0	0
tsrna-03409	CCAACTTACACTTAGGAGATTT	0	0	0	0

tsrna-03403	CCAACACCTCTTTACAGTGACCA	0	1	0	0
tsrna-03402	CCAACACCTCTTTACAGTGACC	0	0	0	0
tsrna-03394	CCAAAACATCAGATTGTGAATCTGACAAC	0	2	0	1
tsrna-03393	CCAAAACATCAGATTGTGAATCTGACA	1	1	1	0
tsrna-03391	CCAAAACATCAGATTGTGAATCTGA	0	0	1	0
tsrna-03385	CCAAAAATTTTGGTGCAACTCCAAATAAA	2	2	5	0
tsrna-03384	CCAAAAATTTTGGTGCAACTCCAAATA	0	5	4	0
tsrna-03383	CCAAAAATTTTGGTGCAACTCCAA	1	0	2	0
tsrna-03382	CCAAAAATTTTGGTGCAACTCCA	0	0	0	0
tsrna-03381	CCAAAAATTTTGGTGCAACTCC	0	0	1	0
tsrna-03380	CCAAAAATTTTGGTGCAACTC	0	1	0	0
tsrna-03377	CATTTGACTGCAGATCAAGAGGTCCCTGGT	1	6	4	10
tsrna-03376	CATTTGACTGCAGATCAAGAGGTCCCCGGT	1	19	3	7
tsrna-03375	CATTTGACTGCAGATCAAGAGGTCCC	0	0	1	0
tsrna-03372	CATTTGACTGCAGATCA	0	1	0	0
tsrna-03370	CATTTCGATGGCGTGGGT	0	6	1	0
tsrna-03369	CATTTCGATGGCGTGGG	2	2	2	0
tsrna-03368	CATTTCGATGGCGTGG	0	3	0	0
tsrna-03367	CATTTCATAACTTTGTCAAAGTTAAATTATAGGCT	10	5	23	0
tsrna-03366	CATTTCATAACTTTGTCAAAGTT	3	1	3	0
tsrna-03364	CATTTCATAACTTTGTC	1	0	0	0
tsrna-03363	CATTGGTGGTTCAGTGGTAGAATTCTCGCCTGCCA	130	53	147	14
tsrna-03362	CATTGGTGGTTCAGTGGTAGAATTCTCGCCTGCC	141	61	166	8
tsrna-03361	CATTGGTGGTTCAGTGGTAGAATTCTCGCCTGC	101	29	93	13
tsrna-03360	CATTGGTGGTTCAGTGGTAGAATTCTCGCCTG	107	25	86	3
tsrna-03359	CATTGGTGGTTCAGTGGTAGAATTCTCGCCT	85	23	89	12
tsrna-03358	CATTGGTGGTTCAGTGGTAGAATTCTCGCC	74	16	66	7
tsrna-03357	CATTGGTGGTTCAGTGGTAGAATTCTCGC	7	5	7	3
tsrna-03356	CATTGGTGGTTCAGTGGTAGAATTCTCG	5	5	3	1
tsrna-03355	CATTGGTGGTTCAGTGGTAGAATTCTC	4	7	5	3
tsrna-03354	CATTGGTGGTTCAGTGGTAGAATTCT	7	5	6	1
tsrna-03353	CATTGGTGGTTCAGTGGTAGAATTC	4	2	2	3
tsrna-03352	CATTGGTGGTTCAGTGGTAGAATT	5	3	4	1
tsrna-03351	CATTGGTGGTTCAGTGGTAGAAT	4	2	1	2
tsrna-03350	CATTGGTGGTTCAGTGGTAGAA	4	1	0	0
tsrna-03349	CATTGGTGGTTCAGTGGTAGA	1	1	0	0
tsrna-03348	CATTGGTGGTTCAGTGGTAG	0	0	0	0
tsrna-03347	CATTGGTGGTTCAGTGGTA	1	1	0	0
tsrna-03346	CATTGGTGGTTCAGTGGT	1	0	0	0
tsrna-03345	CATTGGTGGTTCAGTGG	0	0	0	0
tsrna-03344	CATTGGTGGTTCAGTG	0	0	0	0
tsrna-03340	CATTGGTCGTGGTTGTAGTCCGTGCGAGAATACCA	111	139	145	38
tsrna-03339	CATTGGTCGTGGTTGTAGTCCGTGCGAGAATACC	13	27	11	13
tsrna-03338	CATTGGTCGTGGTTGTAGTCCGTGCGAGAATAC	6	16	12	12
tsrna-03337	CATTGGTCGTGGTTGTAGTCCGTGCGAGAATA	6	17	7	7
tsrna-03336	CATTGGTCGTGGTTGTAGTCCGTGCGAGAAT	7	17	1	7
tsrna-03335	CATTGGTCGTGGTTGTAGTCCGTGCGAGAA	7	15	14	4
tsrna-03334	CATTGGTCGTGGTTGTAGTCCGTGCGAGA	10	7	6	12
tsrna-03333	CATTGGTCGTGGTTGTAGTCCGTGCGAG	5	7	3	4
tsrna-03332	CATTGGTCGTGGTTGTAGTCCGTGCGA	3	4	3	5
tsrna-03331	CATTGGTCGTGGTTGTAGTCCGTGCG	1	7	0	3
tsrna-03330	CATTGGTCGTGGTTGTAGTCCGTGC	0	7	1	0
tsrna-03329	CATTGGTCGTGGTTGTAGTCCGTG	0	2	0	0
tsrna-03328	CATTGGTCGTGGTTGTAGTCCGT	0	2	1	0
tsrna-03327	CATTGGTCGTGGTTGTAGTCCG	0	2	0	0
tsrna-03326	CATTGGTCGTGGTTGTAGTCC	1	1	0	0
tsrna-03325	CATTGGTCGTGGTTGTAGTC	0	0	1	0
tsrna-03323	CATTGGTCGTGGTTGTAG	0	0	0	0
tsrna-03322	CATTGGTCGTGGTTGTA	1	0	0	0
tsrna-03320	CATTGGGGTTTCCCCGCGCAGGTT	1	0	0	0
tsrna-03319	CATTGGGGTTTCCCCGCGCAGGT	0	2	0	0
tsrna-03314	CATTGGGGTCTCCCCGCGCAGGTT	1	0	0	0
tsrna-03313	CATTGGGGTCTCCCCGCGCAGGT	0	2	0	0
tsrna-03311	CATTGGACTGTAAATCTAAAGACAGGGGTTAGGCCT	0	0	1	0

tsrna-03310	CATTGGACTGTAAATCTAAAGACAGGGGTT	1	1	0	1
tsrna-03309	CATTGGACTGTAAATCTAAAGACAGGGGT	1	0	0	0
tsrna-03308	CATTGGACTGTAAATCTAAAGACAGGGG	1	0	0	0
tsrna-03307	CATTGGACTGTAAATCTAAAGACAGGG	0	1	0	0
tsrna-03306	CATTGGACTGTAAATCTAAAGACAGG	1	1	0	0
tsrna-03304	CATTGGACTGTAAATCTAAAGACA	0	2	0	0
tsrna-03301	CATTGGACTGTAAATCTAAAG	1	0	0	0
tsrna-03295	CATTCTTGCGACCCGGGTTCGATTCCCGGGCGGCGCACCA	26	75	15	1
tsrna-03294	CATTCTTGCGACCCGGGTTCGATTCCCGGGCGGCGCACC	9	16	3	1
tsrna-03293	CATTCTTGCGACCCGGGTTCGATTCCCGGG	1	7	0	0
tsrna-03292	CATTCTTGCGACCCGGGTTCGATT	1	1	0	1
tsrna-03291	CATTCTTGCGACCCGGGTTCGAT	1	4	0	0
tsrna-03290	CATTCTTGCGACCCGGGTTCGA	2	3	0	0
tsrna-03289	CATTCTTGCGACCCGGGTTCG	0	2	1	0
tsrna-03288	CATTCTTGCGACCCGGGTTC	0	3	0	0
tsrna-03287	CATTCTTGCGACCCGGGTT	0	1	1	0
tsrna-03286	CATTCTTGCGACCCGGGT	0	2	0	0
tsrna-03284	CATTCTTGCGACCCGG	0	0	0	0
tsrna-03283	CATTCGGCTGTTAACC	2	4	1	0
tsrna-03282	CATTCAAAAAAGAGTACCA	0	1	0	0
tsrna-03278	CATTAAATTATGATAATCATATTTACC	0	2	0	0
tsrna-03276	CATGTGTGAGGTCCCGGGT	0	0	2	0
tsrna-03275	CATGTCTAACAACATGGCTTTCTCACCA	1	1	0	0
tsrna-03274	CATGTCTAACAACATGGCTTTCTCACC	0	2	0	0
tsrna-03272	CATGTCTAACAACATGGCTTTCTCA	0	1	0	0
tsrna-03270	CATGTATGAGGTCCCGGGT	0	2	0	0
tsrna-03269	CATGTATGAGGCCCCGGGT	0	5	0	0
tsrna-03268	CATGTACGAGGCCCCGGGT	0	2	0	0
tsrna-03267	CATGGTGTAATGGTTAGCACTCTGGACTTT	1	4	0	2
tsrna-03266	CATGGTGTAATGGTTAGCACTCTGGACTT	1	1	0	1
tsrna-03265	CATGGTGTAATGGTTAGCACTCTGGACTCT	0	2	1	0
tsrna-03264	CATGGTGTAATGGTTAGCACTCTGGACTC	0	2	1	0
tsrna-03263	CATGGTGTAATGGTTAGCACTCTGGACT	0	0	0	1
tsrna-03262	CATGGTGTAATGGTTAGCACTCTGGAC	1	0	1	0
tsrna-03261	CATGGTGTAATGGTTAGCACTCTGGA	0	0	0	0
tsrna-03260	CATGGTGTAATGGTTAGCACTCTGG	1	0	0	0
tsrna-03258	CATGGTGTAATGGTTAGCACTCT	0	0	1	0
tsrna-03256	CATGGTGTAATGGTGAGCACTCTGGACTC	0	0	0	2
tsrna-03255	CATGGTGTAATGGTGAGCACTCTGGACT	1	0	0	0
tsrna-03254	CATGGTGTAATGGTGAGCACTCTGG	1	0	0	0
tsrna-03252	CATGGTCTAGCGGTTAGGATTCCTGGTTTT	6	4	3	4
tsrna-03251	CATGGTCTAGCGGTTAGGATTCCTGGTTT	5	3	4	0
tsrna-03250	CATGGTCTAGCGGTTAGGATTCCTGGTT	4	5	0	1
tsrna-03249	CATGGTCTAGCGGTTAGGATTCCTGGT	5	3	2	2
tsrna-03248	CATGGTCTAGCGGTTAGGATTCCTGG	6	5	3	1
tsrna-03247	CATGGTCTAGCGGTTAGGATTCCTG	0	1	0	0
tsrna-03246	CATGGTCTAGCGGTTAGGATTCCT	1	1	3	0
tsrna-03239	CATGGGTGGTTCAGTGGTAGAATTCTCGCCTGCC	57	53	70	16
tsrna-03238	CATGGGTGGTTCAGTGGTAGAATTCTCGCCTGC	42	24	45	9
tsrna-03237	CATGGGTGGTTCAGTGGTAGAATTCTCGCCTG	45	29	41	3
tsrna-03236	CATGGGTGGTTCAGTGGTAGAATTCTCGCCT	35	22	48	10
tsrna-03235	CATGGGTGGTTCAGTGGTAGAATTCTCGCC	36	21	30	12
tsrna-03234	CATGGGTGGTTCAGTGGTAGAATTCTCGC	10	7	9	3
tsrna-03233	CATGGGTGGTTCAGTGGTAGAATTCTCG	5	2	3	3
tsrna-03232	CATGGGTGGTTCAGTGGTAGAATTCTC	3	4	2	1
tsrna-03231	CATGGGTGGTTCAGTGGTAGAATTCT	7	3	0	2
tsrna-03230	CATGGGTGGTTCAGTGGTAGAATTC	2	1	0	2
tsrna-03229	CATGGGTGGTTCAGTGGTAGAATT	6	2	1	0
tsrna-03228	CATGGGTGGTTCAGTGGTAGAAT	4	2	2	1
tsrna-03227	CATGGGTGGTTCAGTGGTAGAA	2	1	0	1
tsrna-03226	CATGGGTGGTTCAGTGGTAGA	2	0	1	0
tsrna-03224	CATGGGTGGTTCAGTGGTA	0	0	0	0
tsrna-03223	CATGGGTGGTTCAGTGGT	0	0	0	1
tsrna-03221	CATGGGACTCTTAATCC	2	0	1	0

tsrna-03218	CATGGAGGCGTGGGTT	0	1	0	0
tsrna-03217	CATGGAGGCCATGGGGTTGGCTTGA	0	0	0	0
tsrna-03216	CATGGAGGCCATGGGGTTGGCTT	0	1	1	0
tsrna-03215	CATGGAGGCCATGGGGTTGG	0	1	0	0
tsrna-03214	CATGGAGGCCATGGGGTT	0	0	0	0
tsrna-03213	CATGCGAGAGGTAGCGGGA	0	15	0	0
tsrna-03212	CATGCCCCCATGTCTAACAACATGGCTTTCTCACCA	7	3	11	0
tsrna-03211	CATGCCCCCATGTCTAACAACATGGCTTTCTCACC	11	1	8	0
tsrna-03210	CATGCCCCCATGTCTAACAACATGGCTTTCTCA	0	0	1	0
tsrna-03209	CATGCCCCCATGTCTAACAACATGGCTTTC	1	0	1	0
tsrna-03208	CATGCCCCCATGTCTAACAACATGGCT	0	0	1	0
tsrna-03207	CATGCCCCCATGTCTAACAACATGGC	0	0	1	0
tsrna-03197	CATGCACGAGGTCCTGGGT	2	9	1	0
tsrna-03196	CATGCACGAGGCCCTGGGT	1	15	2	5
tsrna-03195	CATGCACGAGGCCCCGGGTT	0	3	0	1
tsrna-03194	CATGCACGAGGCCCCGGGT	0	1	0	0
tsrna-03193	CATGCAAGATTCCCATTCTTGCGACCCG	0	0	0	0
tsrna-03189	CATGATCGTATAGTGGTTAGTACTCTG	0	4	1	1
tsrna-03188	CATGATCGTATAGTGGTTAGTACTCT	2	1	1	2
tsrna-03186	CATGAGACTCTTAATCT	1	1	1	0
tsrna-03185	CATGAGACTCTTAATC	0	3	1	0
tsrna-03184	CATCAGCCTCCGGAGCTGGGGATTGT	0	0	0	0
tsrna-03181	CATCAGATTGTGAATCTGACAACAGAGGCTTACGAC	3	8	4	1
tsrna-03180	CATCAGATTGTGAATCTGACAACAGAGGCT	1	4	9	0
tsrna-03179	CATCAGATTGTGAATCTGACAACAGAGG	0	2	0	0
tsrna-03178	CATCAGATTGTGAATCTGACAACAGAG	1	3	2	1
tsrna-03177	CATCAGATTGTGAATCTGACAACAGA	2	3	1	0
tsrna-03176	CATCAGATTGTGAATCTGACAACAG	1	5	2	0
tsrna-03175	CATCAGATTGTGAATCTGACAACA	0	5	1	0
tsrna-03174	CATCAGATTGTGAATCTGACAAC	2	2	2	0
tsrna-03173	CATCAGATTGTGAATCTGACA	1	3	1	0
tsrna-03172	CATCAGATTGTGAATCTGAC	0	0	0	0
tsrna-03168	CATCAGACTTTTAATCTGAGGGTCCAG	8	29	9	3
tsrna-03165	CATCACCCCATAAACACCA	1	0	0	0
tsrna-03161	CATATTGAATTGCAAATTCGAAGAAGCAGCTTCAAACCTGCCC	0	2	3	0
tsrna-03159	CATATGTCCGCGTGGGTT	10	5	4	1
tsrna-03158	CATATGTCCGCGTGGGT	1	5	0	1
tsrna-03156	CATATGGTCTAGCGGTTAGGATTCCTGGTT	2	1	4	2
tsrna-03155	CATATGGTCTAGCGGTTAGGATTCCTGG	4	3	0	1
tsrna-03154	CATATGGTCTAGCGGTTAGGATTCCTG	0	2	2	1
tsrna-03153	CATATGGTCTAGCGGTTAGGATTCCT	0	2	3	1
tsrna-03152	CATATGGTCTAGCGGTTAGGATTCC	1	0	0	0
tsrna-03145	CATATCATTGGTCGTGGTTGTAGTCCGTGCGAGAATACCA	148	172	190	54
tsrna-03144	CATATCATTGGTCGTGGTTGTAGTCCGTGCGAGAATACC	22	29	15	15
tsrna-03143	CATATCATTGGTCGTGGTTGTAGTCCGTGCGAGAATA	11	17	11	16
tsrna-03142	CATATCATTGGTCGTGGTTGTAGTCCGTGCGAGAAT	6	14	5	6
tsrna-03141	CATATCATTGGTCGTGGTTGTAGTCCGTGCGAGAA	9	8	11	13
tsrna-03140	CATATCATTGGTCGTGGTTGTAGTCCGTGCGAGA	11	19	3	5
tsrna-03139	CATATCATTGGTCGTGGTTGTAGTCCGTGC	5	4	2	0
tsrna-03138	CATATCATTGGTCGTGGTTGTAGTCCGTG	3	0	3	0
tsrna-03137	CATATCATTGGTCGTGGTTGTAGTCCGT	5	0	1	0
tsrna-03136	CATATCATTGGTCGTGGTTGTAGTCCG	1	1	1	1
tsrna-03135	CATATCATTGGTCGTGGTTGTAGTCC	0	1	2	1
tsrna-03134	CATATCATTGGTCGTGGTTGTAGTC	0	0	2	0
tsrna-03133	CATATCATTGGTCGTGGTTGTAGT	0	0	0	0
tsrna-03132	CATATCATTGGTCGTGGTTGTAG	1	0	0	0
tsrna-03131	CATATCATTGGTCGTGGTTGTA	0	0	0	0
tsrna-03125	CATAGTGTAGTGGTTATCACGTCTGCTTT	1	0	0	0
tsrna-03123	CATAGGTGGTTCAGTGGTAGAATTCT	0	1	0	1
tsrna-03122	CATAGGTGGTTCAGTGGTAGA	1	0	0	0
tsrna-03121	CATAGCTGCCTTCCAAGCAGTTGACCCGGG	2	4	4	2
tsrna-03120	CATAGCTGCCTTCCAAGCAGTTGACCCGG	0	3	0	1
tsrna-03119	CATAGCTGCCTTCCAAGCAGTTGACCCG	1	0	0	0
tsrna-03118	CATAGCTGCCTTCCAAGCAGTTGACC	0	2	0	0

tsrna-03117	CATAGCTGCCTTCCAAGC	0	0	0	1
tsrna-03115	CATAGCTCAGTGGTAGAGCATTTGACTG	0	1	1	1
tsrna-03114	CATAGCTCAGTGGTAGAGCATTTGACT	2	4	1	0
tsrna-03113	CATAGCTCAGGGGTTAGAGCACTGG	0	1	1	0
tsrna-03112	CATACCCCGAAAATGTTGGTTATACCCTTCCCGTACTACCA	13	28	26	2
tsrna-03111	CATACCCCGAAAATGTTGGTTATACCCTTCCCGTAC	1	0	1	0
tsrna-03110	CATACCCCGAAAATGTTGGTT	0	0	1	0
tsrna-03106	CATAATCTGAAGGTCGTGAGTTCGATCCTCACACGGGGCACC	22	65	23	9
tsrna-03105	CATAATCTGAAGGTCGTGAGTTCGATCCTCACACGGGGCACC	23	43	20	6
tsrna-03104	CATAATCTGAAGGTCGTGAGTTCGATCCTCACACGGGGCAC	23	49	12	8
tsrna-03103	CATAATCTGAAGGTCGTGAGTTCGAGCCTCACACGGGGCACC	28	38	22	17
tsrna-03102	CATAATCTGAAGGTCGTGAGTTCGAGCCTCACACGGGGCACC	38	42	16	8
tsrna-03101	CATAATCTGAAGGTCGTGAGTTCG	30	36	23	6
tsrna-03100	CATAATCTGAAGGTCGTGAGTTC	19	37	19	7
tsrna-03099	CATAATCTGAAGGTCGTGAGT	16	51	16	5
tsrna-03098	CATAATCTGAAGGTCGTGAG	1	3	2	0
tsrna-03097	CATAATCTGAAGGTCGTGA	2	2	1	0
tsrna-03096	CATAATCTGAAGGTCGTG	4	6	0	1
tsrna-03095	CATAATCTGAAGGTCCTGAGTTCGAGCCTCAGAGAGGGCACC	124	289	95	56
tsrna-03094	CATAATCTGAAGGTCCTGAGTTCGAACCTCAGAGGGGGCACC	106	286	81	41
tsrna-03093	CATAATCTGAAGGTCCTGAGTTCGAACCTCAGAGGGGGCACC	101	279	91	48
tsrna-03092	CATAATCTGAAGGTCCTGAGTTCGAACCTCAGAGGGGGCAC	117	279	76	56
tsrna-03091	CATAATCTGAAGGTCCTGAGTTCG	117	327	100	51
tsrna-03090	CATAATCTGAAGGTCCTGAGTT	114	253	69	60
tsrna-03089	CATAATCTGAAGGTCCTGAGT	80	280	70	47
tsrna-03088	CATAATCTGAAGGTCCTGAG	7	29	7	3
tsrna-03087	CATAATCTGAAGGTCCTGA	3	10	4	4
tsrna-03086	CATAATCTGAAGGTCCTG	5	8	5	3
tsrna-03085	CATAATCTGAAGGTCCT	1	6	0	0
tsrna-03084	CATAATCTGAAGGTCC	1	2	1	0
tsrna-03083	CATAACTTTGTCAAAGTTAAATTATAGGCT	5	2	6	1
tsrna-03080	CATAACCCAGAGGTCGATGGATCGAAACC	3	9	3	0
tsrna-03079	CATAACCCAGAGGTCGATGGATCGAAAC	3	9	0	2
tsrna-03078	CATAACCCAGAGGTCGATGGATCGAAA	3	12	0	1
tsrna-03077	CATAACCCAGAGGTCGATGGATCGAA	2	7	3	2
tsrna-03076	CATAACCCAGAGGTCGATGGATCGA	3	9	1	1
tsrna-03075	CATAACCCAGAGGTCGATGGATCG	2	11	1	3
tsrna-03074	CATAACCCAGAGGTCGATGGATC	5	4	0	2
tsrna-03073	CATAACCCAGAGGTCGATGGAT	2	4	2	3
tsrna-03072	CATAACCCAGAGGTCGATGGA	0	6	0	0
tsrna-03071	CATAACCCAGAGGTCGATGG	0	1	0	0
tsrna-03070	CATAACCCAGAGGTCGATG	0	0	0	1
tsrna-03069	CATAACCCAGAGGTCGAT	0	4	0	0
tsrna-03068	CATAACCCAGAGGTCGA	0	1	0	0
tsrna-03064	CAGTTGGTTAGAGCGTGG	0	0	0	1
tsrna-03058	CAGTTGGTAGAGCATCAGACTT	1	1	3	0
tsrna-03057	CAGTTGGTAGAGCATCAGACT	0	3	1	0
tsrna-03056	CAGTTGGTAGAGCATCAGAC	0	1	2	0
tsrna-03055	CAGTTGGTAGAGCATCAGA	0	0	0	0
tsrna-03054	CAGTTGGTAGAGCATCAG	1	0	0	0
tsrna-03050	CAGTTGGGAGAGCGTTAGACTGA	0	1	0	0
tsrna-03048	CAGTTGATTAGGGTGCTTAGCTGTTAACTAAGTGTT	8	4	12	2
tsrna-03047	CAGTTGATTAGGGTGCTTAGCTGTTAACT	5	0	10	0
tsrna-03046	CAGTTGATTAGGGTGCTTAGCTGT	1	0	2	0
tsrna-03045	CAGTTGATTAGGGTGCTTAGCTG	0	0	1	0
tsrna-03044	CAGTTGATTAGGGTGCTTAGCT	0	2	0	0
tsrna-03041	CAGTTGATGCAGAGTGGGGTTTTGCAGTCCTTACCA	6	3	7	3
tsrna-03040	CAGTTGATGCAGAGTGGGGTTTTGCAGTCC	0	3	0	0
tsrna-03039	CAGTTGATGCAGAGTGGGGTTTTGCAGTC	0	0	0	0
tsrna-03038	CAGTTGATGCAGAGTGGGGTTTTGCA	0	0	0	0
tsrna-03037	CAGTTGATGCAGAGTGGGGTTTTGC	0	0	0	1
tsrna-03036	CAGTTGATGCAGAGTGGGGTTTTG	0	1	0	0
tsrna-03035	CAGTTGATGCAGAGTGGGGTTTT	1	0	0	0
tsrna-03034	CAGTTGATGCAGAGTGGGGTTT	0	0	0	0

tsrna-03032	CAGTTGATGCAGAGTGGGGT	0	0	0	0
tsrna-03030	CAGTTGACCCGGGTTCGATTCCCGGCCAAC	0	1	0	0
tsrna-03028	CAGTTGACCCGGGTTCGATTC	0	1	0	0
tsrna-03026	CAGTTGACCCGGGTTCGAT	2	1	0	0
tsrna-03025	CAGTTGACCCGGGTTCGA	1	0	0	0
tsrna-03024	CAGTTGACCCGGGTTCG	0	1	1	0
tsrna-03023	CAGTTGACCCGGGTTC	0	1	0	0
tsrna-03022	CAGTGGTAGAGCGCGTGCTT	6	21	1	0
tsrna-03021	CAGTGGTAGAGCGCGTGCT	8	21	3	0
tsrna-03020	CAGTGGTAGAGCGCGTGC	7	12	1	0
tsrna-03019	CAGTGGTAGAGCGCGTG	0	1	0	0
tsrna-03018	CAGTGGTAGAGCGCGCGCTTCG	0	0	0	0
tsrna-03013	CAGTGGTAGAGCATTTGACTGCAGATCAAG	2	2	1	0
tsrna-03012	CAGTGGTAGAGCATTTGACTGCAGATCA	6	2	2	0
tsrna-03011	CAGTGGTAGAGCATTTGACTGCAGATC	1	0	2	0
tsrna-03010	CAGTGGTAGAGCATTTGACTGCAGA	3	3	2	1
tsrna-03009	CAGTGGTAGAGCATTTGACTGCAG	1	4	3	0
tsrna-03008	CAGTGGTAGAGCATTTGACTGCA	3	1	2	0
tsrna-03007	CAGTGGTAGAGCATTTGACTGC	3	3	1	0
tsrna-03006	CAGTGGTAGAGCATTTGACTG	2	0	1	0
tsrna-03005	CAGTGGTAGAGCATTTGACT	2	0	1	1
tsrna-03003	CAGTGGTAGAGCATTTGA	1	0	0	0
tsrna-03001	CAGTGGTAGAGCATTT	1	2	0	0
tsrna-02998	CAGTGGTAGAATTTTC	0	0	1	0
tsrna-02996	CAGTGGTAGAATTCTTGCCTG	0	1	0	0
tsrna-02995	CAGTGGTAGAATTCTTGCCT	0	0	0	0
tsrna-02994	CAGTGGTAGAATTCTTGCC	0	1	0	0
tsrna-02992	CAGTGGTAGAATTCTTG	1	0	0	0
tsrna-02990	CAGTGGTAGAATTCTCGCCTTT	7	6	2	0
tsrna-02989	CAGTGGTAGAATTCTCGCCTT	2	2	2	1
tsrna-02988	CAGTGGTAGAATTCTCGCCTGCCACGC	58	94	24	12
tsrna-02987	CAGTGGTAGAATTCTCGCCTGCCACG	43	88	26	15
tsrna-02986	CAGTGGTAGAATTCTCGCCTGCCAC	22	40	17	11
tsrna-02985	CAGTGGTAGAATTCTCGCCTGCCA	18	35	4	4
tsrna-02984	CAGTGGTAGAATTCTCGCCTGCC	12	29	7	4
tsrna-02983	CAGTGGTAGAATTCTCGCCTGC	6	10	7	4
tsrna-02982	CAGTGGTAGAATTCTCGCCTG	2	6	2	0
tsrna-02981	CAGTGGTAGAATTCTCGCCTCCCACGCGGGAGACCCGGGT	3	11	8	13
tsrna-02980	CAGTGGTAGAATTCTCGCCTCCCACG	5	17	3	7
tsrna-02979	CAGTGGTAGAATTCTCGCCTCCC	5	9	2	9
tsrna-02978	CAGTGGTAGAATTCTCGCCTCC	2	12	1	7
tsrna-02977	CAGTGGTAGAATTCTCGCCTC	1	9	2	1
tsrna-02976	CAGTGGTAGAATTCTCGCCT	4	7	0	1
tsrna-02975	CAGTGGTAGAATTCTCGCC	2	4	3	2
tsrna-02974	CAGTGGTAGAATTCTCGC	1	1	0	0
tsrna-02973	CAGTGGTAGAATTCTCG	0	1	0	0
tsrna-02972	CAGTGGTAGAATTCTCACC	1	1	0	0
tsrna-02971	CAGTGGTAGAATTCTCAC	1	2	1	0
tsrna-02970	CAGTGGTAGAATTCTCA	1	0	1	0
tsrna-02969	CAGTGGTAGAATTCTC	0	0	0	0
tsrna-02968	CAGTGGGTAGAGCATTTGACTGC	1	1	1	0
tsrna-02967	CAGTGGGTAGAGCATTTGACTG	0	2	1	0
tsrna-02966	CAGTGGGTAGAGCATTTGACT	1	0	1	0
tsrna-02965	CAGTGGGTAGAGCATTTGA	0	0	0	0
tsrna-02964	CAGTGGGTAGAGCATTTG	0	0	0	0
tsrna-02963	CAGTGGGTAGAGCATTT	0	0	0	0
tsrna-02962	CAGTGGGTAGAGCATT	0	0	0	0
tsrna-02960	CAGTGGCCTAATGGATAAGGCATCAGCCTC	4	1	4	1
tsrna-02959	CAGTGGCCTAATGGATAAGGCATCAGCCT	3	2	1	2
tsrna-02958	CAGTCTCTTCGGGGGCGTGGGTT	1	1	1	0
tsrna-02957	CAGTCTCTTCGGGGGCGTGGG	0	1	1	0
tsrna-02953	CAGTCTCATAATCTGAAGGTCCTGAGT	175	453	156	84
tsrna-02952	CAGTCTCATAATCTGAAGGTC	1	1	0	0
tsrna-02951	CAGTCGGTAGAGCTATCAGAC	0	1	0	1

tsrna-02950	CAGTCGGTAGAGCTATCAGA	1	1	0	0
tsrna-02949	CAGTCGGTAGAGCTATC	2	0	0	0
tsrna-02948	CAGTCGGTAGAGCATGGGACTCTTAATCT	23	24	10	2
tsrna-02947	CAGTCGGTAGAGCATGGGACTCTTAATCCC	30	29	14	8
tsrna-02946	CAGTCGGTAGAGCATGGGACTCTTAATCC	19	25	12	7
tsrna-02945	CAGTCGGTAGAGCATGGGACTCTTAATC	16	19	10	2
tsrna-02944	CAGTCGGTAGAGCATGGGACTCTTAAT	7	18	8	3
tsrna-02943	CAGTCGGTAGAGCATGGGACTCTTAA	9	15	12	5
tsrna-02942	CAGTCGGTAGAGCATGGGACTCTTA	15	28	5	6
tsrna-02941	CAGTCGGTAGAGCATGGGACTCTT	19	18	6	2
tsrna-02940	CAGTCGGTAGAGCATGGGACTCT	13	17	7	3
tsrna-02939	CAGTCGGTAGAGCATGGGACTC	11	22	9	3
tsrna-02938	CAGTCGGTAGAGCATGGGACT	23	14	9	5
tsrna-02937	CAGTCGGTAGAGCATGGGAC	9	12	2	2
tsrna-02936	CAGTCGGTAGAGCATGGGA	9	10	7	1
tsrna-02935	CAGTCGGTAGAGCATGGG	1	1	1	0
tsrna-02934	CAGTCGGTAGAGCATGG	1	2	0	0
tsrna-02933	CAGTCGGTAGAGCATGAGACTCTTAATCTC	19	35	6	8
tsrna-02932	CAGTCGGTAGAGCATGAGACTCTTAATC	22	34	5	4
tsrna-02931	CAGTCGGTAGAGCATGAGACTCTTAAT	18	19	6	6
tsrna-02930	CAGTCGGTAGAGCATGAGACTCTTAA	13	24	3	7
tsrna-02929	CAGTCGGTAGAGCATGAGACTCTTA	20	36	3	7
tsrna-02928	CAGTCGGTAGAGCATGAGACTCTT	14	34	10	3
tsrna-02927	CAGTCGGTAGAGCATGAGACTCT	24	32	6	9
tsrna-02926	CAGTCGGTAGAGCATGAGACTC	16	26	11	8
tsrna-02925	CAGTCGGTAGAGCATGAGACT	15	26	10	8
tsrna-02924	CAGTCGGTAGAGCATGAGACCC	11	15	2	1
tsrna-02923	CAGTCGGTAGAGCATGAGACC	8	18	2	5
tsrna-02922	CAGTCGGTAGAGCATGAGAC	5	17	3	1
tsrna-02921	CAGTCGGTAGAGCATGAGA	1	7	2	0
tsrna-02920	CAGTCGGTAGAGCATGAG	1	3	0	0
tsrna-02919	CAGTCGGTAGAGCATGA	0	3	1	0
tsrna-02918	CAGTCGGTAGAGCATG	1	1	2	0
tsrna-02917	CAGTCGGTAGAGCATCAGACTTTTAATCTGAGGGTCCAGGGT	42	69	25	4
tsrna-02916	CAGTCGGTAGAGCATCAGACTTTTAATCTGAGGGTCC	28	42	34	2
tsrna-02915	CAGTCGGTAGAGCATCAGACTTTTAATCTGA	22	20	10	5
tsrna-02914	CAGTCGGTAGAGCATCAGACTTTTAATCTG	17	11	8	5
tsrna-02913	CAGTCGGTAGAGCATCAGACTTTTAATCT	7	8	6	4
tsrna-02912	CAGTCGGTAGAGCATCAGACTTTTAATC	14	8	9	4
tsrna-02911	CAGTCGGTAGAGCATCAGACTTTTAAT	17	18	7	1
tsrna-02910	CAGTCGGTAGAGCATCAGACTTTTA	12	15	8	1
tsrna-02909	CAGTCGGTAGAGCATCAGACTTTT	8	9	6	3
tsrna-02908	CAGTCGGTAGAGCATCAGACTTT	9	11	5	1
tsrna-02907	CAGTCGGTAGAGCATCAGACTT	12	5	3	2
tsrna-02906	CAGTCGGTAGAGCATCAGACT	8	12	4	2
tsrna-02905	CAGTCGGTAGAGCATCAGAC	7	6	4	1
tsrna-02904	CAGTCGGTAGAGCATCAGA	4	10	5	1
tsrna-02903	CAGTCGGTAGAGCATCAG	3	1	2	0
tsrna-02902	CAGTCGGTAGAGCATCA	1	2	3	1
tsrna-02901	CAGTCGGTAGAGCATC	1	6	3	0
tsrna-02899	CAGTCAGAGGTTCAATTCCTCT	1	5	0	0
tsrna-02898	CAGTCAGAGGTTCAATTCC	0	2	0	0
tsrna-02896	CAGTAAGTTGCAATACTTAATTTCTGCCA	6	0	3	0
tsrna-02895	CAGTAAGTTGCAATACTTAATTTCTGCC	0	0	0	0
tsrna-02888	CAGGTTCGAGTCCTGCCGCGGTCGCCA	6	3	1	1
tsrna-02887	CAGGTTCGAGTCCTGCCGCGGTCGCC	1	0	0	0
tsrna-02884	CAGGTTCGACTCCTGGCTGGCTCGCCA	42	112	8	3
tsrna-02883	CAGGTTCGACTCCTGGCTGGCTCGCC	2	2	0	0
tsrna-02881	CAGGTTCGAATCCTGTTCGTGACGCCA	6	9	5	6
tsrna-02880	CAGGTTCGAATCCTGTTCGTGACGC	0	1	0	0
tsrna-02878	CAGGTTCGAATCCTGCCGACTACGCCA	105	256	26	18
tsrna-02877	CAGGTTCGAATCCTGCCGACTACGCC	11	20	1	2
tsrna-02875	CAGGTTCGAATCCTGCCGACTACG	0	0	0	0
tsrna-02871	CAGGTTCGAATCCTGCCGAC	0	0	0	0

tsrna-02870	CAGGTTCGAACCCTGCTCGCTGCGCCA	12	32	3	2
tsrna-02869	CAGGTTCGAACCCTGCTCGCTGCGCC	0	5	1	0
tsrna-02868	CAGGTTCGAACCCTGCTCGCTGCGC	0	0	0	0
tsrna-02867	CAGGTGGTAGAGCATTTGACTGC	1	2	1	0
tsrna-02866	CAGGTGGTAGAGCATTTGACTG	1	1	0	0
tsrna-02865	CAGGTGGTAGAGCATTTGACT	0	0	0	1
tsrna-02863	CAGGTGGTAGAGCATCAGA	0	0	0	1
tsrna-02862	CAGGTGGCCCGGGTTCGACTCCCGGTATGGGAACC	2	6	1	1
tsrna-02858	CAGGGTTCAAGTCCCTGTTCGGGCGCCA	5	15	2	0
tsrna-02857	CAGGGTTCAAGTCCCTGTTCGGGCGCC	0	1	0	0
tsrna-02849	CAGGGTTCAAGTCCCTGTCCAGGCGCCA	0	1	0	0
tsrna-02848	CAGGGTCGTGGGTTCGAGCCCCACGTTGGG	0	1	0	0
tsrna-02846	CAGGGGTTAGGCCTCTTTTTACCACCA	2	2	4	2
tsrna-02845	CAGGGGTTAGGCCTCTTTTTACCACC	0	0	2	0
tsrna-02844	CAGGGGTTAGGCCTCTTTTTACCAC	0	0	1	0
tsrna-02843	CAGGGGTTAGGCCTCTTTTTACCA	0	0	1	0
tsrna-02841	CAGGGGTTAGGCCTCTTTTTAC	0	0	0	0
tsrna-02836	CAGGGGTCGCGAGTTCAAA	0	1	0	0
tsrna-02835	CAGGGGTCGCGAGTTCA	1	1	0	0
tsrna-02834	CAGGGGTCGCGAGTTC	1	0	1	0
tsrna-02833	CAGGGGTAGAGCATTTGACTGCAGA	0	1	0	0
tsrna-02832	CAGGGGTAGAGCATTTGACTGCA	0	0	0	0
tsrna-02831	CAGGGGTAGAGCATTTGACTGC	0	0	0	0
tsrna-02829	CAGGGGTAGAGCATTTGACT	0	1	0	0
tsrna-02825	CAGGGGTAGAGCATTT	0	0	0	0
tsrna-02824	CAGGGATTGTGGGTTCGAGTCCCATCTGGGGTGCCA	1	8	2	0
tsrna-02823	CAGGGATTGTGGGTTCGAGTCCCATCTGGGGTGCC	4	5	1	0
tsrna-02822	CAGGGATTGTGGGTTCGAGTCCC	1	8	2	1
tsrna-02821	CAGGGATTGTGGGTTCG	2	4	0	0
tsrna-02820	CAGGGATTGTGGGTTC	1	5	2	0
tsrna-02819	CAGGGATGGGTTCGATTCTCATAGTC	1	2	3	3
tsrna-02818	CAGGGATGGGTTCGATTCTCA	0	1	2	0
tsrna-02817	CAGGGATGGGTTCGATTCTC	1	0	1	0
tsrna-02816	CAGGCGGCCCGGGTTCGACTCCCGGTGTGGGAACCA	86	303	38	21
tsrna-02815	CAGGCGGCCCGGGTTCGACTCCCGGTGTGGGAACC	3	5	5	1
tsrna-02814	CAGGCGGCCCGGGTTCGACTCCCGGTGTGGGAAC	1	1	1	0
tsrna-02813	CAGGCGGCCCGGGTTCGACTCCCGGTGTGGGAA	1	2	0	0
tsrna-02812	CAGGCGGCCCGGGTTCGACTCCCGGTGTGGGA	0	3	0	0
tsrna-02811	CAGGCGGCCCGGGTTCGACTCCCGGTGTGGG	0	1	0	0
tsrna-02809	CAGGCGGCCCGGGTTCGACTCCCGGTGTG	0	1	0	0
tsrna-02807	CAGGCGGCCCGGGTTCGACTCCCGGTG	0	1	0	0
tsrna-02806	CAGGCGGCCCGGGTTCGACTCCCGGT	0	1	0	0
tsrna-02805	CAGGATGGCCGAGTGGTTAAGGCGTTGGAC	0	0	0	0
tsrna-02804	CAGGATGGCCGAGTGGTTAAGGC	0	1	0	0
tsrna-02803	CAGGATGGCCGAGTGGTTAAG	0	0	1	0
tsrna-02800	CAGGATGGCCGAGTGGTCTAAG	0	2	1	0
tsrna-02799	CAGGATGGCCGAGTGGTCTA	0	0	1	0
tsrna-02798	CAGGATGGCCGAGTGGTCT	1	0	0	0
tsrna-02797	CAGGATGGCCGAGCGGTCTAAGGCGCTGCGTTC	0	7	3	0
tsrna-02796	CAGGATGGCCGAGCGGTCTAAGGCGCTGCGTT	0	5	1	1
tsrna-02795	CAGGATGGCCGAGCGGTCTAAGGCGCTGCG	1	5	0	1
tsrna-02794	CAGGATGGCCGAGCGGTCTAAGGCGCTGC	2	7	0	0
tsrna-02793	CAGGATGGCCGAGCGGTCTAAGGCGCTG	2	3	0	1
tsrna-02792	CAGGATGGCCGAGCGGTCTAAGGCGCT	0	4	0	0
tsrna-02791	CAGGATGGCCGAGCGGTCTAAGGC	1	3	0	0
tsrna-02790	CAGGATGGCCGAGCGGTCTAAGG	1	1	1	1
tsrna-02789	CAGGATGGCCGAGCGGTCTAAG	1	3	0	0
tsrna-02788	CAGGATGGCCGAGCGGTCTAA	1	0	0	0
tsrna-02784	CAGGAGATCCTGGGTTCGACTCCCAGCGGG	6	23	6	1
tsrna-02783	CAGGAGATCCTGGGTTCGAATCCCAGCGGTGCCTCC	6	29	2	1
tsrna-02782	CAGGAGATCCTGGGTTCGAATCCCAGCGGTGCCTC	4	21	5	3
tsrna-02781	CAGGAGATCCTGGGTTCGAATCCCAGCGGTGCCT	2	14	5	2
tsrna-02780	CAGGAGATCCTGGGTTCGAATCCCAGCGGTGCC	15	16	7	3
tsrna-02779	CAGGAGATCCTGGGTTCGAATCCCAGCGGTGC	4	23	7	2

tsrna-02778	CAGGAGATCCTGGGTTCGAATCCCAGCGGT	10	24	9	4
tsrna-02777	CAGGAGATCCTGGGTTCGAATCCCAGCGGGGCCTCC	8	14	4	0
tsrna-02776	CAGGAGATCCTGGGTTCGAATCCCAGCGGGGCCTC	13	17	9	1
tsrna-02775	CAGGAGATCCTGGGTTCGAATCCCAGCGGGGCCT	6	10	7	1
tsrna-02774	CAGGAGATCCTGGGTTCGAATCCCAGCGGGGCC	9	23	6	3
tsrna-02773	CAGGAGATCCTGGGTTCGAATCCCAGCGGG	8	18	2	2
tsrna-02772	CAGGAGATCCTGGGTTCG	7	25	1	1
tsrna-02771	CAGCTTTGGGGGGTTCG	0	0	0	0
tsrna-02770	CAGCTTTGGGGGGTTC	0	0	0	0
tsrna-02769	CAGCTTCAAACCTGCCGGGGCTTCCA	1	12	5	1
tsrna-02768	CAGCTTCAAACCTGCCGGGGCTTCC	0	2	0	0
tsrna-02767	CAGCTTCAAACCTGCCGGGGCTTC	0	1	0	0
tsrna-02765	CAGCTTCAAACCTGCCGGGGCT	0	1	0	0
tsrna-02764	CAGCTGGTAGAGCGGAGGACTGTAGATCCT	0	1	0	0
tsrna-02759	CAGCTATCCATTGGTCTTAGGCCCC	0	0	0	0
tsrna-02756	CAGCTATCCATTGGTCTTAGGC	0	0	1	0
tsrna-02754	CAGCTATCCATTGGTCTTAG	0	1	0	0
tsrna-02751	CAGCTATCCATTGGTCT	0	0	0	0
tsrna-02749	CAGCTAAATAAGCTATCGGGCCCA	0	2	0	0
tsrna-02748	CAGCTAAATAAGCTATCGGGCCC	0	0	0	1
tsrna-02747	CAGCTAAATAAGCTATCGGGCC	0	0	0	0
tsrna-02746	CAGCTAAATAAGCTATCGGGC	0	0	0	0
tsrna-02743	CAGCGGAAGCGTGCTGGGCCC	6	37	6	2
tsrna-02742	CAGCGGAAGCGTGCTGGGCC	3	30	1	2
tsrna-02741	CAGCGGAAGCGTGCTGGGC	3	31	3	2
tsrna-02740	CAGCGATGGCCGAGTGGTTAAGGC	0	1	0	0
tsrna-02739	CAGCGATCCGAGTTCAAATCTCGGTGGAAC	0	0	0	0
tsrna-02736	CAGCCTCCGGAGCTGGGGATTGTGGGT	1	2	2	0
tsrna-02734	CAGCCTCCGGAGCTGGGGATTGTGG	0	0	0	0
tsrna-02731	CAGCAGAGTGGCGCAGCGGAAGCGTGCTGGGC	2	34	2	2
tsrna-02730	CAGCACTCTGGACTCTGAATCCAGC	1	3	1	0
tsrna-02729	CAGCACTCTGGACTCTGAATCCAG	2	3	0	1
tsrna-02727	CAGCACTCTGGACTCTGAATCC	1	1	1	0
tsrna-02721	CAGCAACCTCGGTTCGAATC	1	0	0	0
tsrna-02719	CAGATTGTGAATCTGACAACAGAGGCTTACGACCCCTTATTTA	31	20	51	0
tsrna-02718	CAGATTGTGAATCTGACAACAGAGGCTTACGACCCCTTATTTA	34	13	81	0
tsrna-02717	CAGATTGTGAATCTGACAACAGAGGCTTACGACCCC	1	9	5	2
tsrna-02716	CAGATTGTGAATCTGACAACAGAGGCTTACGACCC	3	10	4	0
tsrna-02715	CAGATTGTGAATCTGACAACAGAGGCTTACGAC	0	10	10	0
tsrna-02714	CAGATTGTGAATCTGACAACAGAGGCTTACG	0	6	2	1
tsrna-02713	CAGATTGTGAATCTGACAACAGAGGCTTAC	2	4	2	1
tsrna-02712	CAGATTGTGAATCTGACAACAGAGGCTT	1	6	3	0
tsrna-02711	CAGATTGTGAATCTGACAACAGAGGC	0	4	1	0
tsrna-02710	CAGATTGTGAATCTGACAACAGAG	4	2	2	0
tsrna-02709	CAGATTGTGAATCTGACAACAGA	1	3	2	0
tsrna-02708	CAGATTGTGAATCTGACAACAG	2	1	3	0
tsrna-02707	CAGATTGTGAATCTGACAACA	1	2	0	0
tsrna-02706	CAGATTGTGAATCTGACAAC	0	0	3	1
tsrna-02705	CAGATTGTGAATCTGACAA	0	1	1	0
tsrna-02704	CAGATTGTGAATCTGACA	1	2	1	0
tsrna-02701	CAGATCAGAAGGTTGCGTGT	0	2	0	0
tsrna-02700	CAGATCAGAAGGTTGCGTG	0	2	0	0
tsrna-02698	CAGATCAGAAGGCTGCGTGTTCGAA	0	0	0	0
tsrna-02696	CAGATCAGAAGGCTGCGTGTT	0	0	0	0
tsrna-02695	CAGATCAGAAGGCTGCGTGT	1	0	0	0
tsrna-02691	CAGATCAAGAGGTCCCTGGTT	3	3	2	3
tsrna-02690	CAGATCAAGAGGTCCCTGGT	2	3	0	0
tsrna-02688	CAGATCAAGAGGTCCCCGGTTCA	1	4	1	0
tsrna-02687	CAGATCAAGAGGTCCCCGGTTC	1	3	1	1
tsrna-02686	CAGATCAAGAGGTCCCCGGTT	2	1	1	0
tsrna-02685	CAGATCAAGAGGTCCCCGGT	0	2	0	0
tsrna-02679	CAGATCAAAAGGTCCCTGGTT	1	9	2	2
tsrna-02678	CAGAGTGTAGCTTAACACAAAGCACCCAACTTACACTTAGGA(	11	4	3	5
tsrna-02677	CAGAGTGTAGCTTAACACAAAGCACCCAACTTACACTTAGGA(	8	8	3	2

tsrna-02676	CAGAGTGTAGCTTAACACAAAGCACCCAACTTACACTTAGGAC	10	5	7	0
tsrna-02675	CAGAGTGTAGCTTAACACAAAGCACCCAACTTACACTTAGGA	12	1	4	3
tsrna-02674	CAGAGTGTAGCTTAACACAAAGCACCCAACTTACACTTAGG	8	4	9	4
tsrna-02673	CAGAGTGTAGCTTAACACAAAGCACCCAACTTACACTTA	9	4	9	2
tsrna-02672	CAGAGTGTAGCTTAACACAAAGCACCCAACTTACACTT	14	4	10	2
tsrna-02671	CAGAGTGTAGCTTAACACAAAGCACCCAACTTACACT	11	6	8	2
tsrna-02670	CAGAGTGTAGCTTAACACAAAGCACCCAACTTACAC	9	7	5	7
tsrna-02669	CAGAGTGTAGCTTAACACAAAGCACCCAACTTACA	6	3	5	4
tsrna-02668	CAGAGTGTAGCTTAACACAAAGCACCCAACTTAC	9	5	4	3
tsrna-02667	CAGAGTGTAGCTTAACACAAAGCACCCAACTTA	9	2	6	2
tsrna-02666	CAGAGTGTAGCTTAACACAAAGCACCCAACTT	8	3	2	6
tsrna-02665	CAGAGTGTAGCTTAACACAAAGCACCCAACT	10	4	4	2
tsrna-02664	CAGAGTGTAGCTTAACACAAAGCACCCAAC	8	2	2	4
tsrna-02663	CAGAGTGTAGCTTAACACAAAGCACCCAA	8	5	5	2
tsrna-02662	CAGAGTGTAGCTTAACACAAAGCACCCA	8	2	6	5
tsrna-02661	CAGAGTGTAGCTTAACACAAAGCACCC	10	2	3	4
tsrna-02660	CAGAGTGTAGCTTAACACAAAGCACC	12	5	7	1
tsrna-02659	CAGAGTGTAGCTTAACACAAAGCAC	4	4	6	5
tsrna-02658	CAGAGTGTAGCTTAACACAAAGCA	6	4	6	2
tsrna-02657	CAGAGTGTAGCTTAACACAAAGC	4	1	4	1
tsrna-02656	CAGAGTGTAGCTTAACACAAAG	5	1	2	1
tsrna-02655	CAGAGTGTAGCTTAACACAAA	7	3	2	1
tsrna-02654	CAGAGTGTAGCTTAACACAA	3	2	5	3
tsrna-02653	CAGAGTGTAGCTTAACACA	2	1	1	6
tsrna-02652	CAGAGTGTAGCTTAACAC	6	4	4	0
tsrna-02651	CAGAGTGTAGCTTAACA	6	1	2	2
tsrna-02650	CAGAGTGTAGCTTAAC	3	1	0	0
tsrna-02649	CAGAGTGGGGTTTTGCAGTCCTTACCA	0	0	0	0
tsrna-02648	CAGAGTGGGGTTTTGCAGTCCTTACC	0	1	2	0
tsrna-02647	CAGAGTGGGGTTTTGCAGTCCTTAC	0	0	1	0
tsrna-02646	CAGAGTGGGGTTTTGCAGTCCTTA	0	0	3	0
tsrna-02645	CAGAGTGGGGTTTTGCAGTCCTT	0	1	1	0
tsrna-02644	CAGAGTGGGGTTTTGCAGTCCT	1	1	0	0
tsrna-02643	CAGAGTGGGGTTTTGCAGTCC	0	0	1	0
tsrna-02642	CAGAGTGGGGTTTTGCAGTC	0	0	0	0
tsrna-02639	CAGAGTGGGGTTTTGCA	1	0	0	0
tsrna-02637	CAGAGTGGCGCAGCGGAAGCGTGCTGGGCCC	13	39	2	9
tsrna-02636	CAGAGTGGCGCAGCGGAAGCGTGCTGGGCC	5	31	2	2
tsrna-02635	CAGAGTGGCGCAGCGGAAGCGTGCTGGGC	0	33	2	2
tsrna-02634	CAGAGTGGCGCAGCGGAAGCGTGCTGGG	4	18	0	1
tsrna-02633	CAGAGTGGCGCAGCGGAAGCGTGCTGG	0	3	0	0
tsrna-02632	CAGAGTGGCGCAGCGGAAGCGTGCTG	0	1	0	0
tsrna-02631	CAGAGTGGCGCAGCGGAAGCGTGCT	0	1	0	0
tsrna-02624	CAGAGGTCGATGGATCGAAACCATCCTCTG	1	1	0	0
tsrna-02622	CAGAGGTCGATGGATCGAAACC	0	4	0	0
tsrna-02621	CAGAGGTCGATGGATCGAAAC	0	1	0	0
tsrna-02620	CAGAGGTCGATGGATCGAAA	1	0	1	0
tsrna-02619	CAGAGGTCGATGGATCGAA	0	1	0	0
tsrna-02618	CAGAGGTCGATGGATCG	1	1	0	0
tsrna-02617	CAGAGGTCGATGGATC	0	3	0	1
tsrna-02616	CAGAGGCTTACGACCCCTTATTTACCCCA	3	3	2	1
tsrna-02615	CAGAGGCTTACGACCCCTTATTTACCCC	0	0	0	0
tsrna-02612	CAGAGGCTTACGACCCCTT	0	0	0	0
tsrna-02607	CAGAGCCCGGTAATCGCATA	0	1	0	0
tsrna-02602	CAGAGCATTTGACTGCAGATC	1	1	0	0
tsrna-02601	CAGAGAATAGTTTAAATTAGAATCTTAGCTTTGGGTGCTAATG	27	9	23	13
tsrna-02600	CAGAGAATAGTTTAAATTAGAATCTTAGCTTTGGGT	21	3	10	10
tsrna-02599	CAGAGAATAGTTTAAATTAGAATCTTAGCTTTGG	23	9	16	11
tsrna-02598	CAGAGAATAGTTTAAATTAGAATCTTAGCTTT	17	6	12	14
tsrna-02597	CAGAGAATAGTTTAAATTAGAATCTTAGCTT	17	4	13	8
tsrna-02596	CAGAGAATAGTTTAAATTAGAATCTTAGCT	13	6	8	6
tsrna-02595	CAGAGAATAGTTTAAATTAGAATCTTAGC	17	6	9	6
tsrna-02594	CAGAGAATAGTTTAAATTAGAATCTTAG	2	3	2	2
tsrna-02593	CAGAGAATAGTTTAAATTAGAATCTT	7	4	1	0

tsrna-02592	CAGAGAATAGTTTAAATTAGAATCT	6	2	1	6
tsrna-02591	CAGAGAATAGTTTAAATTAGAATC	3	2	0	6
tsrna-02590	CAGAGAATAGTTTAAATTAGAAT	4	0	0	2
tsrna-02589	CAGAGAATAGTTTAAATTAGAA	1	2	0	1
tsrna-02588	CAGAGAATAGTTTAAATTAGA	1	1	2	0
tsrna-02587	CAGAGAATAGTTTAAATTAG	1	0	0	0
tsrna-02586	CAGAGAATAGTTTAAATTA	1	1	0	1
tsrna-02585	CAGAGAATAGTTTAAATT	3	1	0	1
tsrna-02582	CAGACTTTTAATCTGAGGGTCCAGGGT	19	37	13	3
tsrna-02581	CAGACTTTTAATCTGAGGGTCCAG	9	36	6	1
tsrna-02580	CAGACTCAAGTTCTGGTCTCCGGATGGAGG	1	4	1	0
tsrna-02579	CAGACTCAAGTTCTGGTCTCCGG	2	5	1	2
tsrna-02578	CAGACTCAAGTTCTGGTCTCCG	3	3	0	1
tsrna-02577	CAGACTCAAGTTCTGGTCTCCAATG	1	3	1	1
tsrna-02576	CAGACTCAAGTTCTGGTCTCC	2	3	0	0
tsrna-02575	CAGACCAAGAGCCTTCAAAGCCCTCAGTA	7	9	3	0
tsrna-02574	CAGACCAAGAGCCTTCAAAGCCCTC	3	8	3	0
tsrna-02573	CAGACCAAGAGCCTTCAAAGCCCT	12	6	1	0
tsrna-02572	CAGACCAAGAGCCTTCAAAGCCC	3	4	1	0
tsrna-02571	CAGACCAAGAGCCTTCAAAGCC	6	2	2	0
tsrna-02570	CAGACCAAGAGCCTTCAAAGC	3	9	1	0
tsrna-02569	CAGACCAAGAGCCTTCAAAG	4	5	0	0
tsrna-02568	CAGACCAAGAGCCTTCAAA	2	5	1	0
tsrna-02567	CAGACCAAGAGCCTTCAA	2	1	2	0
tsrna-02566	CAGACCAAGAGCCTTCA	0	3	0	0
tsrna-02563	CAGAAGGTTGCGTGTTC	1	3	0	0
tsrna-02562	CAGAAGGTTGCGTGTT	0	0	0	0
tsrna-02561	CAGAAGGTCCTGGGTTCGAGCCCCAGTGGAACCACCA	39	34	26	22
tsrna-02560	CAGAAGGTCCTGGGTTCGAGCCCCAGTGGAACCACC	4	6	3	0
tsrna-02559	CAGAAGGTCCTGGGTTCGAGCCCCAGTGGAACCAC	4	4	2	1
tsrna-02558	CAGAAGGTCCTGGGTTCGAGCCCCAGTGG	2	0	2	0
tsrna-02557	CAGAAGGTCCTGGGTTCGAGCCCCAGT	1	1	1	0
tsrna-02556	CAGAAGGTCCTGGGTTCAAGCCCCAGTGG	1	2	0	0
tsrna-02554	CAGAAGGCTGCGTGTTCG	0	1	0	0
tsrna-02553	CAGAAGGCTGCGTGTTC	0	0	0	1
tsrna-02552	CAGAAGGCTGCGTGTT	1	2	0	0
tsrna-02549	CAGAAGATTGCAGGTTC	0	0	0	1
tsrna-02547	CAGAAGATTGAGGGTTCGAGTCCCTTCGTGGTCGCCA	5	8	4	1
tsrna-02546	CAGAAGATTGAGGGTTCGAGTCCCTTCGTGGTCGCC	2	13	3	5
tsrna-02545	CAGAAGATTGAGGGTTCGAGTCCCTTCGTG	1	10	4	2
tsrna-02544	CAGAAGATTGAGGGTTCGAGTCCCTTCGT	2	10	1	2
tsrna-02543	CAGAAGATTGAGGGTTCGAATCCCTTCGTG	3	6	2	2
tsrna-02542	CAGAAGATTGAGGGTTC	3	11	4	1
tsrna-02541	CAGAAGATTCTAGGTTCGACTCCTGGCTGGCTCGCC	6	6	6	2
tsrna-02540	CAGAAGATTCTAGGTTCGACTCCTGGCTGG	3	6	3	0
tsrna-02539	CAGAAGATTCTAGGTTC	0	9	1	0
tsrna-02538	CAGAAGATTCCAGGTTCGACTCCTGGCTGGCTCGCC	2	3	0	1
tsrna-02537	CAGAAGATTCCAGGTTCGACTCCTGGCTGG	0	2	0	0
tsrna-02536	CAGAAGATTCCAGGTTC	0	0	0	0
tsrna-02535	CAGAAGATTCCAGGTT	2	2	0	1
tsrna-02534	CACTTAGGAGATTTCAACTTAACTTGACCGCTCTGACCA	106	50	74	15
tsrna-02533	CACTTAGGAGATTTCAACTTAACTTGACCGCTCTGACC	12	7	10	0
tsrna-02532	CACTTAGGAGATTTCAACTTAACTTGACCGCTCTGAC	0	2	4	0
tsrna-02531	CACTTAGGAGATTTCAACTTAACTTGACCGCTCTGA	0	5	5	1
tsrna-02530	CACTTAGGAGATTTCAACTTAACTTGACCGCTCTG	1	0	1	1
tsrna-02529	CACTTAGGAGATTTCAACTTAACTTGACCGCT	2	2	1	1
tsrna-02528	CACTTAGGAGATTTCAACTTAACTTGACCG	2	0	1	0
tsrna-02527	CACTTAGGAGATTTCAACTTAACTTGACC	0	1	1	0
tsrna-02525	CACTTAGGAGATTTCAACTTAACTT	0	0	0	0
tsrna-02524	CACTTAGGAGATTTCAACTTAACT	0	1	0	0
tsrna-02515	CACTGTAAAGCTAACTTAGCATTAACCTTTTAAGTTAAAGATTA	31	15	32	14
tsrna-02514	CACTGTAAAGCTAACTTAGCATTAACCTTTTAAGTTAAAGATTA	26	7	31	11
tsrna-02513	CACTGTAAAGCTAACTTAGCATTAACCTTTTAAGTTAAAGATTA	17	16	26	7
tsrna-02512	CACTGTAAAGCTAACTTAGCATTAACCTTTTAAGTTAAAGATTA	30	16	29	12

tsrna-02511	CACTGTAAAGCTAACTTAGCATTAACCTTTTAAGTTAAAGATTA	35	12	23	16
tsrna-02510	CACTGTAAAGCTAACTTAGCATTAACCTTTTAAGTTAAAGA	39	9	25	12
tsrna-02509	CACTGTAAAGCTAACTTAGCATTAACCTTTTAAGTTAAAG	29	15	22	10
tsrna-02508	CACTGTAAAGCTAACTTAGCATTAACCTTTTAAGTTAA	28	22	19	8
tsrna-02507	CACTGTAAAGCTAACTTAGCATTAACCTTTTAAGTTA	28	17	25	13
tsrna-02506	CACTGTAAAGCTAACTTAGCATTAACCTTTTAAGTT	34	14	19	14
tsrna-02505	CACTGTAAAGCTAACTTAGCATTAACCTTTTAAGT	19	9	9	16
tsrna-02504	CACTGTAAAGCTAACTTAGCATTAACCTTTTAAG	13	14	13	12
tsrna-02503	CACTGTAAAGCTAACTTAGCATTAACCTTTTA	15	11	14	14
tsrna-02502	CACTGTAAAGCTAACTTAGCATTAACCTTTT	15	8	6	8
tsrna-02501	CACTGTAAAGCTAACTTAGCATTAACCTTT	9	7	8	5
tsrna-02500	CACTGTAAAGCTAACTTAGCATTAACCTT	7	11	1	13
tsrna-02499	CACTGTAAAGCTAACTTAGCATTAACCT	13	12	2	10
tsrna-02498	CACTGTAAAGCTAACTTAGCATTAACC	8	10	8	11
tsrna-02497	CACTGTAAAGCTAACTTAGCATTAAC	5	8	4	9
tsrna-02496	CACTGTAAAGCTAACTTAGCATTAA	5	4	5	8
tsrna-02495	CACTGTAAAGCTAACTTAGCATTA	5	7	4	5
tsrna-02494	CACTGTAAAGCTAACTTAGCATT	3	2	3	4
tsrna-02493	CACTGTAAAGCTAACTTAGCAT	3	3	4	5
tsrna-02492	CACTGTAAAGCTAACTTAGCA	5	4	3	5
tsrna-02491	CACTGTAAAGCTAACTTAGC	0	1	2	6
tsrna-02490	CACTGTAAAGCTAACTTAG	0	0	1	2
tsrna-02489	CACTGTAAAGCTAACTTA	0	0	0	0
tsrna-02488	CACTGTAAAGCTAACTT	2	0	0	1
tsrna-02487	CACTGTAAAGCTAACT	0	0	2	0
tsrna-02486	CACTGGTGGTTCAGTGGTAGAATTCTCGCCT	11	8	5	5
tsrna-02485	CACTGGTGGTTCAGTGGTAGAATTCTCGCC	14	10	5	2
tsrna-02484	CACTGGTGGTTCAGTGGTAGAATTCTCG	5	2	2	4
tsrna-02483	CACTGGTGGTTCAGTGGTAGAATTCT	2	2	1	0
tsrna-02482	CACTGGTGGTTCAGTGGTAGAATT	0	3	0	0
tsrna-02481	CACTGAAAATGTTTAGACGGGCTCACATCACCCCATAAACACC	101	51	61	10
tsrna-02480	CACTGAAAATGTTTAGACGGGCTCACATCACCCCATAAACA	106	48	59	16
tsrna-02479	CACTGAAAATGTTTAGACGGGCTCACATC	48	29	18	3
tsrna-02478	CACTGAAAATGTTTAGACGGGCTCACAT	44	28	26	3
tsrna-02477	CACTGAAAATGTTTAGACGGGCTCACA	37	30	14	7
tsrna-02476	CACTGAAAATGTTTAGACGGGCTCAC	31	22	7	1
tsrna-02475	CACTGAAAATGTTTAGACGGGCTCA	13	14	6	1
tsrna-02474	CACTGAAAATGTTTAGACGGGCTC	4	8	3	2
tsrna-02473	CACTGAAAATGTTTAGACGGGCT	0	3	0	0
tsrna-02472	CACTGAAAATGTTTAGACGGGC	0	2	0	1
tsrna-02471	CACTGAAAATGTTTAGACGGG	0	0	0	1
tsrna-02467	CACTCTGGACTTTGAATCCAGC	1	4	2	1
tsrna-02466	CACTCTGGACTTTGAATCCAG	3	3	1	0
tsrna-02465	CACTCTGGACTTTGAATCCA	3	5	3	0
tsrna-02464	CACTCTGGACTTTGAATCC	6	2	1	0
tsrna-02463	CACTCTGGACTTTGAATC	0	3	0	1
tsrna-02460	CACTCTGGACTCTGAATCCGGT	0	3	1	0
tsrna-02459	CACTCTGGACTCTGAATCCGG	0	1	0	2
tsrna-02458	CACTCTGGACTCTGAATCCG	0	2	1	0
tsrna-02457	CACTCTGGACTCTGAATCCAGCGATCCGAGTTCA	2	6	0	1
tsrna-02456	CACTCTGGACTCTGAATCCAGCGATCCGAGTT	2	2	2	0
tsrna-02455	CACTCTGGACTCTGAATCCAGCGATCCGAGT	1	4	3	0
tsrna-02454	CACTCTGGACTCTGAATCCAGCGATCCGAG	2	2	0	0
tsrna-02453	CACTCTGGACTCTGAATCCAGCGATCCGA	1	6	1	1
tsrna-02452	CACTCTGGACTCTGAATCCAGCGATCCG	3	4	0	0
tsrna-02451	CACTCTGGACTCTGAATCCAGCGATCC	2	5	1	0
tsrna-02450	CACTCTGGACTCTGAATCCAGCGATC	3	5	2	0
tsrna-02449	CACTCTGGACTCTGAATCCAGCGAT	1	1	3	2
tsrna-02448	CACTCTGGACTCTGAATCCAGCGA	1	2	0	0
tsrna-02447	CACTCTGGACTCTGAATCCAGCG	1	2	0	0
tsrna-02446	CACTCTGGACTCTGAATCCAGC	0	1	0	0
tsrna-02445	CACTCTGGACTCTGAATCCAG	1	4	0	2
tsrna-02444	CACTCTGGACTCTGAATCCA	1	6	0	0
tsrna-02443	CACTCTGGACTCTGAATCC	0	0	0	0

tsrna-02435	CACGTCTGCTTTACACGCAGA	0	1	0	0
tsrna-02431	CACGTCTGCTTTACACG	1	0	0	0
tsrna-02430	CACGTCGGGGTCACCA	1	2	0	0
tsrna-02429	CACGGGTTCGAACCCCGTCCGTGCCTCCA	4	8	1	3
tsrna-02428	CACGGGTTCGAACCCCGTCCGTGCC	0	1	0	0
tsrna-02427	CACGCGTGGGTTCGAATCCCATCCTCGTCGCCA	12	10	4	2
tsrna-02426	CACGCGTGGGTTCGAATCCCATCCTCGTCGCC	0	3	1	0
tsrna-02425	CACGCGTGGGTTCGAATCCCATCCTCGTCG	0	1	0	0
tsrna-02424	CACGCGGGAGGCCCGGGTTCGATTCCCGGCCAATGCACCA	56	177	23	19
tsrna-02423	CACGCGGGAGGCCCGGGTTCGATTCCCGGCCAATGCACC	9	15	6	0
tsrna-02422	CACGCGGGAGGCCCGGGTTCGATTCCCGGCCAATGC	1	6	1	0
tsrna-02421	CACGCGGGAGGCCCGGGTTCGATTCCCGGCCAATG	1	4	0	0
tsrna-02420	CACGCGGGAGGCCCGGGTTCG	2	1	1	0
tsrna-02419	CACGCGGGAGGCCCGGGTT	1	4	0	0
tsrna-02418	CACGCGGGAGGCCCGGGT	1	2	0	0
tsrna-02417	CACGCGGGAGGCCCGGG	0	1	0	0
tsrna-02416	CACGCGGGAGACCGGGGTTCGATTCCCCGACGGGGAGCCA	0	14	0	0
tsrna-02415	CACGCGGGAGACCGGGGTTCGATTCCCCGACGGGGAGCC	0	1	0	0
tsrna-02414	CACGCGGGAGACCGGGGTTCGATTCCCCGACGGGGAGC	1	0	0	0
tsrna-02413	CACGCGGGAGACCGGGGTTCGATTCCCCGACGGGGAG	0	0	0	0
tsrna-02412	CACGCGGGAGACCGGGGTTCGATTCCCCGACGGGGA	0	1	0	0
tsrna-02411	CACGCGGGAGACCGGGGTTCGATTCCCCGACGGGG	0	0	0	0
tsrna-02405	CACGCGGGAGACCGGGGTTCAATTCCCCGACGGGGAGCCA	1	2	0	0
tsrna-02402	CACGCGGGAGACCCGGGT	0	1	0	0
tsrna-02401	CACGCGAAAGGTCCCCGGTTCGAAACCGGGCGGAAAC	1	7	0	1
tsrna-02400	CACGCGAAAGGTCCCCGGTTCGAAACCGGG	0	2	2	0
tsrna-02399	CACGCGAAAGGTCCCCGGTTCGAA	0	3	0	0
tsrna-02398	CACGCGAAAGGTCCCCGGTTCGA	0	6	1	0
tsrna-02397	CACGCGAAAGGTCCCCGGTTCG	1	9	0	0
tsrna-02396	CACGCGAAAGGTCCCCGGTTC	0	5	0	0
tsrna-02395	CACGCGAAAGGTCCCCGGTT	2	4	0	0
tsrna-02394	CACGCGAAAGGTCCCCGGT	0	3	1	0
tsrna-02390	CACGCAGAAGGTCCTGGGTTCGAGCCCCAGTGGAACCACCA	58	36	48	18
tsrna-02389	CACGCAGAAGGTCCTGGGTTCGAGCCCCAGTGGAACCACC	17	15	18	3
tsrna-02388	CACGCAGAAGGTCCTGGGTTCGAGCCCCAGTGGAACCAC	12	13	6	1
tsrna-02387	CACGCAGAAGGTCCTGGGTTCGAGCCCCAGTGGAACC	9	6	7	1
tsrna-02386	CACGCAGAAGGTCCTGGGTTCGAGCCCCAGTGGAAC	2	6	2	0
tsrna-02385	CACGCAGAAGGTCCTGGGT	1	2	0	0
tsrna-02383	CACCTCTTTACAGTGACCA	0	0	0	0
tsrna-02382	CACCTCTTTACAGTGACC	0	0	0	0
tsrna-02379	CACCGCCGCGGCCCGGGTTCGATTCCCGGTCAGGGAACCA	42	337	19	9
tsrna-02378	CACCGCCGCGGCCCGGGTTCGATTCCCGGTCAGGGAACC	5	14	7	1
tsrna-02377	CACCGCCGCGGCCCGGGTTCGATTCCCGGTCAGGGAAC	1	5	1	0
tsrna-02376	CACCGCCGCGGCCCGGGTTCGATTCCCGGTCAGGGAA	1	3	0	0
tsrna-02375	CACCGCCGCGGCCCGGGTTCGATTCCCGGTCAGGGA	0	1	0	0
tsrna-02374	CACCGCCGCGGCCCGGGTTCGATTCCCGGTCAGGG	0	1	0	1
tsrna-02373	CACCGCCGCGGCCCGGGTTCGATTCCCGGTCAGG	0	1	0	0
tsrna-02372	CACCGCCGCGGCCCGGGTTCGATTCCCGGTCA	0	2	0	0
tsrna-02370	CACCGCCGCGGCCCGGGTTCGATTCCCGGT	0	1	0	1
tsrna-02369	CACCGCCGCGGCCCGGGTTCGATTCCCGG	0	0	0	0
tsrna-02368	CACCGCCGCGGCCCGGGTTCGATTCCCG	0	0	0	1
tsrna-02367	CACCGCCGCGGCCCGGGTTCGATTCCC	0	1	0	0
tsrna-02366	CACCGCCGCGGCCCGGGTTCGATTCC	0	1	0	0
tsrna-02363	CACCGCCGCGGCCCGGGTTCGAT	0	1	0	0
tsrna-02354	CACCCTGGACTCTGAATCCAG	1	0	0	0
tsrna-02349	CACCCAGGTGGCCCGGGTTCGACTCCCGGTATGGG	0	0	0	0
tsrna-02345	CACCCAGGCGGCCCGGGTTCGACTCCCGGTGTGGGAACCA	80	334	50	28
tsrna-02344	CACCCAGGCGGCCCGGGTTCGACTCCCGGTGTGGGAACC	5	13	5	2
tsrna-02343	CACCCAGGCGGCCCGGGTTCGACTCCCGGTGTGGGAAC	2	2	0	0
tsrna-02342	CACCCAGGCGGCCCGGGTTCGACTCCCGGTGTGGGA	0	0	2	0
tsrna-02341	CACCCAGGCGGCCCGGGTTCGACTCCCGGTGTGGG	0	0	1	0
tsrna-02340	CACCCAGGCGGCCCGGGTTCGACTCCCGGTGTGG	0	1	0	0
tsrna-02339	CACCCAGGCGGCCCGGGTTCGACTCCCGGTGTG	0	0	0	0
tsrna-02338	CACCCAGGCGGCCCGGGTTCGACTCCCGGT	0	0	0	0

tsrna-02329	CACCCAACTTACACTTAGGAGATTTCAACTTAACTTGACCGCT	2	5	13	0
tsrna-02328	CACCCAACTTACACTTAGGAGATTTCAACTTAACTTGACCGCT	5	3	16	0
tsrna-02327	CACCCAACTTACACTTAGGAGATTTCAACT	1	0	0	0
tsrna-02314	CACATGGTCTAGCGGTTAGGATTCCTGGTTTTC	6	5	4	4
tsrna-02313	CACATGGTCTAGCGGTTAGGATTCCTGGTTT	2	6	4	4
tsrna-02312	CACATGGTCTAGCGGTTAGGATTCCTGGTT	6	4	3	3
tsrna-02311	CACATGGTCTAGCGGTTAGGATTCCTGGT	7	2	1	1
tsrna-02310	CACATGGTCTAGCGGTTAGGATTCCTGG	7	5	1	3
tsrna-02309	CACATGGTCTAGCGGTTAGGATTCCTG	0	1	3	0
tsrna-02308	CACATGGTCTAGCGGTTAGGATTCCT	2	0	1	0
tsrna-02300	CACATCACCCCATAAACACCA	5	0	0	0
tsrna-02294	CACACGCGAAAGGTCCCCGGT	1	3	1	0
tsrna-02290	CACAAGAACTGCTAACTCATGCCCCCATGTCTAACAACATGG(	5	1	5	0
tsrna-02289	CACAAGAACTGCTAACTCATGCCCCCATGTCTAACAACATGG(	3	0	3	0
tsrna-02288	CACAAGAACTGCTAACTCATGCCCCCATGTCTAACAACATGG	1	3	3	0
tsrna-02287	CACAAGAACTGCTAACTCATGCCCCCATGTCTAACAACA	3	1	1	0
tsrna-02286	CACAAGAACTGCTAACTCATGCCCCCATGTCTAACA	1	2	3	0
tsrna-02285	CACAAGAACTGCTAACTCATGCCCCCATGTCT	2	1	0	1
tsrna-02284	CACAAGAACTGCTAACTCATGCCCCCATGTC	1	1	0	2
tsrna-02283	CACAAGAACTGCTAACTCATGCCCCCATGT	3	0	0	0
tsrna-02282	CACAAGAACTGCTAACTCATGCCCCCATG	0	3	1	0
tsrna-02281	CACAAGAACTGCTAACTCATGCCCCC	0	1	0	0
tsrna-02280	CACAAGAACTGCTAACTCATGCCCC	0	0	1	0
tsrna-02279	CACAAGAACTGCTAACTCATGCCC	0	0	1	0
tsrna-02278	CACAAGAACTGCTAACTCATGCC	1	0	0	0
tsrna-02277	CACAAGAACTGCTAACTCATGC	0	0	2	0
tsrna-02276	CACAAGAACTGCTAACTCATG	1	0	2	0
tsrna-02275	CACAAGAACTGCTAACTCAT	0	0	1	0
tsrna-02274	CACAAGAACTGCTAACTCA	0	0	0	0
tsrna-02273	CACAAGAACTGCTAACTC	1	0	0	0
tsrna-02272	CACAAGAACTGCTAACT	0	0	0	0
tsrna-02271	CACAAGAACTGCTAAC	1	0	0	0
tsrna-02270	CACAAAGCACCCAACTTACACTTAGGAG	0	0	0	0
tsrna-02268	CACAAAGCACCCAACTTACACTTAG	0	0	0	0
tsrna-02259	CAATTCTCGCTGGGGCCTCCA	44	43	21	14
tsrna-02257	CAATTCCTCTTCTTAACACCA	0	2	0	1
tsrna-02256	CAATTCCGGCTCGAAGGACCA	55	134	17	10
tsrna-02255	CAATTCCCGGCCAATGCACCA	0	7	1	1
tsrna-02251	CAATTAACTAGTTTTGACAACA	0	1	0	0
tsrna-02249	CAATGGTGGTTCAGTGGTAGAATTCTCGCCT	27	30	20	4
tsrna-02248	CAATGGTGGTTCAGTGGTAGAATTCTCGC	6	1	1	3
tsrna-02247	CAATGGTAGAATTCTCGCCT	2	4	0	0
tsrna-02246	CAATGGTAGAATTCTCGCC	1	3	0	2
tsrna-02245	CAATGGTAGAATTCTCGC	0	2	0	0
tsrna-02243	CAATGGATAGCGCATTGGA	9	5	2	0
tsrna-02242	CAATGGATAGCGCATTGG	2	2	1	1
tsrna-02240	CAATGGATAGCGCATT	1	0	0	0
tsrna-02239	CAATGGATAACGCGTCTGACT	0	0	1	0
tsrna-02236	CAATGGAGGCGTGGGTT	1	10	2	0
tsrna-02235	CAATGGAGGCGTGGGT	2	7	2	0
tsrna-02233	CAATCCCTGGCACCTCCACCA	0	1	0	0
tsrna-02232	CAATCCCGGGTTTCGGCACCA	34	92	10	15
tsrna-02230	CAATCCCCGGCATCTCCACCA	105	112	48	28
tsrna-02229	CAATCCCCGGCATCTCCACC	2	1	2	0
tsrna-02226	CAATCCCCGGCACCTCCACCA	166	249	79	40
tsrna-02225	CAATCCCCGGCACCTCCACC	12	16	6	1
tsrna-02224	CAATCCCCGGCACCTCCAC	0	0	0	0
tsrna-02220	CAATCCCCAGCACCTCCACCA	0	1	1	0
tsrna-02218	CAATACTTAATTTCTGCCA	2	0	0	0
tsrna-02217	CAATACTTAATTTCTGCC	0	0	0	0
tsrna-02215	CAATACACTGAAAATGTTTAGACGGGCTC	6	13	4	1
tsrna-02209	CAAGTTCTGGTCTCCGG	1	0	0	0
tsrna-02203	CAAGTCTCGGTGGAACCTCCA	44	98	10	10
tsrna-02202	CAAGTCCCTGTTCGGGCGCCA	6	6	3	3

tsrna-02197	CAAGTCCCTGTTCAGGCGCCA	0	1	0	0
tsrna-02195	CAAGTCCCTGTCCAGGCGCCA	0	1	0	0
tsrna-02194	CAAGTCACGTCGGGGTCACCA	5	4	0	1
tsrna-02193	CAAGTCACGTCGGGGTCACC	0	0	0	0
tsrna-02192	CAAGTCACGTCGGGGTCAC	0	1	0	0
tsrna-02188	CAAGGTCGCGGGTTCG	0	1	2	0
tsrna-02187	CAAGCAGTTGACCCGGGTTCGATTCCCGGCC	2	4	0	0
tsrna-02186	CAAGCAGTTGACCCGGGTTCGAT	1	2	2	0
tsrna-02185	CAAGCAGTTGACCCGGGTTCGA	2	5	0	1
tsrna-02184	CAAGCAGTTGACCCGGGTTCG	3	2	1	0
tsrna-02183	CAAGCAGTTGACCCGGGTTC	0	2	0	0
tsrna-02182	CAAGCAGTTGACCCGGGTT	0	4	1	0
tsrna-02181	CAAGCAGTTGACCCGGGT	1	1	0	0
tsrna-02178	CAAGATTCCCATTCTTGCGACCCGG	2	0	0	0
tsrna-02177	CAAGATTCCCATTCTTGCGACCCG	1	4	2	0
tsrna-02176	CAAGAGGTCCCCGGTTC	0	7	0	0
tsrna-02175	CAAGAGCCTTCAAAGCCCTCAGTAAGTTGC	0	1	0	0
tsrna-02173	CAAGAACTGCTAACTCATGCCCCCATGTCTAACAACATGGCTT	0	3	3	0
tsrna-02172	CAAGAACTGCTAACTCATGCCCCCATGTCTAACAACATGGCT	4	1	4	0
tsrna-02171	CAAGAACTGCTAACTCATGCCCCCATGTCTAACAACATGGC	1	1	3	1
tsrna-02170	CAAGAACTGCTAACTCATGCCCCCATGTCTAACAACATGG	0	1	0	0
tsrna-02169	CAAGAACTGCTAACTCATGCCCCCATGTCTAACAACA	1	0	2	0
tsrna-02168	CAAGAACTGCTAACTCATGCCCCCATGTCTAACAAC	0	2	2	0
tsrna-02167	CAAGAACTGCTAACTCATGCCCCCATGTCTAACA	2	1	0	0
tsrna-02166	CAAGAACTGCTAACTCATGCCCCCATGTCTAAC	0	2	1	0
tsrna-02165	CAAGAACTGCTAACTCATGCCCCCATGTCT	1	0	0	0
tsrna-02164	CAAGAACTGCTAACTCATGCCCCCATGTC	2	0	0	0
tsrna-02163	CAAGAACTGCTAACTCATGCCCCCATGT	0	0	1	1
tsrna-02162	CAAGAACTGCTAACTCATGCCCCCATG	1	2	0	0
tsrna-02161	CAAGAACTGCTAACTCATGCCCCCAT	0	0	2	0
tsrna-02160	CAAGAACTGCTAACTCATGCCCCCA	1	1	1	0
tsrna-02159	CAAGAACTGCTAACTCATGCCCCC	1	2	1	0
tsrna-02157	CAAGAACTGCTAACTCATGCCC	0	0	1	0
tsrna-02155	CAAGAACTGCTAACTCATGC	0	1	0	0
tsrna-02154	CAAGAACTGCTAACTCATG	0	1	0	1
tsrna-02150	CAACTTACACTTAGGAGATTTCAACTTAACTTGACCGCTCTGA	88	41	99	17
tsrna-02149	CAACTTACACTTAGGAGATTTCAACTTAACTTGACCGCTCTGA	15	7	23	3
tsrna-02148	CAACTTACACTTAGGAGATTTCAACTTAACTTGACCGCTCTGA	3	3	6	0
tsrna-02147	CAACTTACACTTAGGAGATTTCAACTTAACTTGACCGCTCTGA	2	5	5	1
tsrna-02146	CAACTTACACTTAGGAGATTTCAACTTAACTTGACCGCTCTG	4	1	12	0
tsrna-02145	CAACTTACACTTAGGAGATTTCAACTTAACTTGACCGCTCT	2	0	8	0
tsrna-02144	CAACTTACACTTAGGAGATTTCAACTTAACTTGACCGCT	3	1	4	0
tsrna-02143	CAACTTACACTTAGGAGATTTCAACTTAACTTGACCG	1	1	13	0
tsrna-02142	CAACTTACACTTAGGAGATTTCAACTTAACTTGACC	1	0	9	0
tsrna-02141	CAACTTACACTTAGGAGATTTCAACTTAACTT	1	0	2	0
tsrna-02140	CAACTTACACTTAGGAGATTTCAACTTAACT	1	0	0	0
tsrna-02139	CAACTTACACTTAGGAGATTTCAACTTAAC	0	0	2	0
tsrna-02138	CAACTTACACTTAGGAGATTTCAACTTAA	0	1	0	1
tsrna-02137	CAACTTACACTTAGGAGATTTCAACTTA	0	1	2	0
tsrna-02135	CAACTTACACTTAGGAGATTTCAACT	0	0	1	1
tsrna-02134	CAACTTACACTTAGGAGATTTCAAC	0	0	0	0
tsrna-02133	CAACTTACACTTAGGAGATTTCAA	0	0	2	0
tsrna-02132	CAACTTACACTTAGGAGATTTCA	0	0	0	0
tsrna-02124	CAACTTAACTTGACCGCTCTGACCA	21	20	21	4
tsrna-02123	CAACTTAACTTGACCGCTCTGACC	3	1	1	0
tsrna-02122	CAACTTAACTTGACCGCTCTGAC	1	0	2	0
tsrna-02121	CAACTTAACTTGACCGCTCTGA	2	0	1	0
tsrna-02120	CAACTTAACTTGACCGCTCTG	1	0	0	0
tsrna-02112	CAACGGTAGCGCGTCTGACTCC	3	6	0	1
tsrna-02111	CAACGGTAGCGCGTCTGACT	0	0	1	1
tsrna-02110	CAACGATGGTTTTTCATATCATTGGTCGTGGTTGTAGTCC	4	0	7	0
tsrna-02109	CAACGATGGTTTTTCATATCATTGGTCGTG	1	0	1	0
tsrna-02097	CAACATGGCTTTCTCACCA	0	1	0	0
tsrna-02092	CAACAGAGGCTTACGACCCCTTATTTACCC	0	0	0	0

tsrna-02083	CAAATTCGAAGAAGCAGCTTCAAACCTGCCGGGGCTT	0	0	1	0
tsrna-02082	CAAATTCGAAGAAGCAGCTTCAAACCTGCCGGGGCT	2	1	0	1
tsrna-02081	CAAATCTGGGTGCCCCCTCCA	1	0	0	1
tsrna-02080	CAAATCTCGGTGGGACCTCCA	57	87	28	24
tsrna-02079	CAAATCTCGGTGGGACCTCC	4	5	3	0
tsrna-02078	CAAATCTCGGTGGGACCTC	0	1	0	0
tsrna-02077	CAAATCTCGGTGGAACCTCCA	102	191	46	16
tsrna-02076	CAAATCTCGGTGGAACCTCC	7	5	0	2
tsrna-02075	CAAATCTCGGTGGAACCTC	0	2	0	0
tsrna-02074	CAAATCTCGGTGGAACCT	0	1	0	0
tsrna-02072	CAAATCTCGGTGGAAC	1	0	0	0
tsrna-02071	CAAATCTCGCTGGGGCCTCCA	43	45	17	16
tsrna-02070	CAAATCTCGCTGGGGCCTCC	1	10	2	2
tsrna-02068	CAAATCTCGCTGGGGCCT	0	1	0	0
tsrna-02067	CAAATCCGGGTGCCCCCTCCA	143	184	63	20
tsrna-02066	CAAATCCGGGTGCCCCCTCC	3	12	1	1
tsrna-02065	CAAATCCGGGTGCCCCCTC	1	1	0	0
tsrna-02064	CAAATCCGGGTGCCCCCT	0	1	0	0
tsrna-02063	CAAATCCCGGACGAGCCCCCA	115	158	47	52
tsrna-02062	CAAATCCCGGACGAGCCCCC	4	11	3	2
tsrna-02061	CAAATCCCGGACGAGCCCC	1	1	3	0
tsrna-02060	CAAATCCCGGACGAGCCC	1	0	0	0
tsrna-02059	CAAATCCCGGACGAGCC	0	0	0	0
tsrna-02057	CAAATCCCACCGCTGCCACCA	0	3	0	0
tsrna-02056	CAAATCCAGGTGCCCCCTCCA	0	1	0	0
tsrna-02055	CAAATCACGTCGGGGTCACCA	2	7	4	2
tsrna-02053	CAAATCACGTCGGGGTCAC	0	0	0	0
tsrna-02051	CAAAGTTAAATTATAGGCTAAATCCTATATATCTT	1	1	1	0
tsrna-02050	CAAAGTTAAATTATAGGCTAAATCCTATA	1	0	2	0
tsrna-02047	CAAAGCACCCAACTTACACTTAGGAGATTTCAACTT	1	0	9	0
tsrna-02046	CAAAGCACCCAACTTACACTTAGGAGATTT	1	2	0	1
tsrna-02045	CAAAGCACCCAACTTACACTTAGGAGATT	0	1	0	0
tsrna-02044	CAAAGCACCCAACTTACACTTAGGAGAT	1	0	0	0
tsrna-02043	CAAAGCACCCAACTTACACTTAGGAGA	0	0	0	0
tsrna-02041	CAAAGCACCCAACTTACACTTAGGA	0	1	0	0
tsrna-02031	CAAAGCAATACACTGAAAATGTTTAGACGGGCT	0	11	0	0
tsrna-02030	CAAAGCAATACACTGAAAATGTTTAGACGG	1	1	0	0
tsrna-02029	CAAAGCAATACACTGAA	0	0	1	0
tsrna-02027	CAAACCTGCCGGGGCTTCCA	0	2	1	0
tsrna-02026	CAAACCTGCCGGGGCTTCC	0	0	0	0
tsrna-02024	CAAACCTGCCGGGGCTT	0	0	0	0
tsrna-02021	CAAAACGAATGATTTCGACTC	0	4	1	0
tsrna-02020	CAAAACATCAGATTGTGAATCTGACAACAGAGGCTTACGACC(	20	22	55	1
tsrna-02019	CAAAACATCAGATTGTGAATCTGACAACAGAGGCTTACGACC(	5	11	2	1
tsrna-02018	CAAAACATCAGATTGTGAATCTGACAACAGAGGCTT	2	4	3	0
tsrna-02017	CAAAACATCAGATTGTGAATCTGACAACAGAGGCT	2	6	5	0
tsrna-02016	CAAAACATCAGATTGTGAATCTGACAACAGAGGC	3	4	8	1
tsrna-02015	CAAAACATCAGATTGTGAATCTGACAACAG	0	6	0	0
tsrna-02014	CAAAACATCAGATTGTGAATCTGACAACA	0	1	2	0
tsrna-02013	CAAAACATCAGATTGTGAATCTGACA	0	1	1	0
tsrna-02012	CAAAACATCAGATTGTGAATCTGAC	0	0	1	0
tsrna-02010	CAAAACATCAGATTGTGAATCTG	0	1	0	0
tsrna-02009	CAAAACATCAGATTGTGAATCT	0	0	0	0
tsrna-02003	CAAAAATTTTGGTGCAACTCCAAATAAAAG	1	3	2	0
tsrna-02002	CAAAAATTTTGGTGCAACTCCAAATA	2	3	4	0
tsrna-02001	CAAAAATTTTGGTGCAACTCCA	0	1	1	0
tsrna-02000	CAAAAATTTTGGTGCAACTCC	0	0	1	0
tsrna-01999	CAAAAATTTTGGTGCAACTC	0	1	0	0
tsrna-01997	ATTTTGGTGCAACTCCAAATAAAAGTACC	0	0	0	0
tsrna-01993	ATTTTGGATTCTCAGGGATGGGTTCGATTC	2	1	1	1
tsrna-01992	ATTTTGGATTCTCAGGGATGGGTTCG	0	1	1	0
tsrna-01991	ATTTTGGATTCTCAGGGATGGGTTC	0	3	0	0
tsrna-01987	AGTTAAATTATAGGCTAAATCCTATAT	0	1	1	1
tsrna-01983	AGTTAAAGATTAAGAGAACCAACACCTCTTTACAGTGACC	2	1	2	1

tsrna-01982	AGTTAAAGATTAAGAGAACCAACACCTCTTTACAGTGAC	1	1	1	1
tsrna-01981	AGTTAAAGATTAAGAGAACCAACACCTCTT	1	1	0	1
tsrna-01979	AGTTAAAGATTAAGAGAACCAACA	0	0	0	0
tsrna-01978	AGTTAAAGATTAAGAGAACCA	0	0	0	1
tsrna-01977	AGTTAAAGATTAAGAGAACC	0	0	0	0
tsrna-01974	AGTTAAAGACTTTTTCTCTGACCA	22	26	16	11
tsrna-01973	AGTTAAAGACTTTTTCTCTGACC	3	7	0	1
tsrna-01972	AGTTAAAGACTTTTTCTCTGAC	0	2	1	0
tsrna-01971	AGTTAAAGACTTTTTCTCTGA	0	0	0	0
tsrna-01970	AGTTAAAGACTTTTTCTCTG	0	0	0	0
tsrna-01969	AGTTAAAGACTTTTTCTCT	0	1	0	0
tsrna-01968	AGTGTTTGTGGGTTTAAGTCCCATTGGTCTAGCCA	3	3	0	0
tsrna-01967	AGTGTTTGTGGGTTTAAGTCCCATTGGTCTAGCC	0	2	1	0
tsrna-01966	AGTGTTTGTGGGTTTAAGTCCCATTGGTCTAGC	0	0	0	0
tsrna-01965	AGTGTTTGTGGGTTTAAGTCCCATTGGTCT	0	0	1	0
tsrna-01964	AGTGTTTGTGGGTTTAAGTCCCATTGGTC	0	1	0	0
tsrna-01963	AGTGTTTGTGGGTTTAAGTCCCA	0	0	0	0
tsrna-01945	AGTGTAGTGGTTATCACG	0	0	0	0
tsrna-01941	AGTGTAGCTTAACACAAAGCACCCAACTT	0	0	1	0
tsrna-01937	AGTGTAGCGGTTATCACGTCTGCTTT	1	0	0	0
tsrna-01935	AGTGGTTATCACGTTCGCCTCACACGC	1	0	2	0
tsrna-01934	AGTGGTTATCACGTTCGCCTCACAC	0	0	1	0
tsrna-01933	AGTGGTTATCACGTTCGCCTCAC	1	0	0	0
tsrna-01927	AGTGGTTATCACGTCTGCTTTACA	2	0	0	0
tsrna-01922	AGTGGTTAGTATCCCCGCCTGTC	7	1	0	0
tsrna-01920	AGTGGTTAGTATCCCCGC	2	0	0	0
tsrna-01919	AGTGGTTAGTACTCTGCGTTGTGGCCGCAGCAACCT	20	12	13	2
tsrna-01918	AGTGGTTAGTACTCTGCGTTGTGGCCGCAG	14	9	11	6
tsrna-01917	AGTGGTTAGTACTCTGCGTTGTGGCCGC	16	8	8	3
tsrna-01916	AGTGGTTAGTACTCTGCGTTGTGGCCG	18	8	10	2
tsrna-01915	AGTGGTTAGTACTCTGCGTTGTGGCC	22	8	3	6
tsrna-01914	AGTGGTTAGTACTCTGCGTTGTGGC	11	5	8	3
tsrna-01913	AGTGGTTAGTACTCTGCGTTGTGG	16	5	3	0
tsrna-01912	AGTGGTTAGTACTCTGCGTTGTG	11	5	11	3
tsrna-01911	AGTGGTTAGTACTCTGCGTTGT	17	10	2	8
tsrna-01910	AGTGGTTAGTACTCTGCGTTG	12	4	4	1
tsrna-01909	AGTGGTTAGTACTCTGCGTT	12	4	6	2
tsrna-01908	AGTGGTTAGTACTCTGCGT	1	0	0	0
tsrna-01907	AGTGGTTAGTACTCTGCGC	1	1	0	0
tsrna-01906	AGTGGTTAGTACTCTGCG	0	3	0	1
tsrna-01904	AGTGGTTAGTACTCTG	0	1	0	0
tsrna-01903	AGTGGTTAGGATTCGGCGCTCTCACCGCCGCGGCCCGGGTT	32	48	18	8
tsrna-01902	AGTGGTTAGGATTCGGCGCTCTCACCGCCGCGGCCCGGGT	38	49	14	13
tsrna-01901	AGTGGTTAGGATTCGGCGCTCTCACCGCCGCGGCCCGGG	32	41	14	18
tsrna-01900	AGTGGTTAGGATTCGGCGCTCTCACCGCCGCGGCCCG	30	44	12	19
tsrna-01899	AGTGGTTAGGATTCGGCGCTCTCACCGCCGCGGCCC	28	51	16	19
tsrna-01898	AGTGGTTAGGATTCGGCGCTCTCACCGCCGCGGCC	21	37	19	9
tsrna-01897	AGTGGTTAGGATTCGGCGCTCTCACCGCCG	27	37	19	15
tsrna-01896	AGTGGTTAGGATTCGGCGCTCTCACCGCC	27	41	12	15
tsrna-01895	AGTGGTTAGGATTCGGCGCTCTCACCGC	22	41	11	7
tsrna-01894	AGTGGTTAGGATTCGGCGCTCTCACC	22	34	12	4
tsrna-01893	AGTGGTTAGGATTCGGCGCTCTCAC	19	31	12	9
tsrna-01892	AGTGGTTAGGATTCGGCGCTCTCA	21	27	9	11
tsrna-01891	AGTGGTTAGGATTCGGCGCTCTC	17	40	14	5
tsrna-01890	AGTGGTTAGGATTCGGCGCTCT	30	24	3	8
tsrna-01889	AGTGGTTAGGATTCGGCGCTC	22	20	12	9
tsrna-01888	AGTGGTTAGGATTCGGCGCT	18	17	3	6
tsrna-01887	AGTGGTTAGGATTCGGCGC	5	6	5	2
tsrna-01886	AGTGGTTAGGATTCGGCG	4	6	4	0
tsrna-01885	AGTGGTTAGGATTCGGC	1	3	2	1
tsrna-01884	AGTGGTTAGGATTCGG	1	0	0	0
tsrna-01881	AGTGGTTAGAGCACTGG	0	1	0	0
tsrna-01879	AGTGGTTAAGGCGTTGGACTTAAG	0	0	0	0
tsrna-01878	AGTGGTTAAGGCGTTGGACTTAA	1	1	0	0

tsrna-01874	AGTGGTTAAGGCGATGGACTGCT	0	1	0	1
tsrna-01873	AGTGGTGAGTATCCCCGCCTGTCAC	4	15	1	5
tsrna-01872	AGTGGTGAGTATCCCCGCCTGTC	3	2	1	0
tsrna-01871	AGTGGTGAGTATCCCCGCCTGT	1	1	1	0
tsrna-01870	AGTGGTGAGTATCCCCGCCTG	1	3	2	0
tsrna-01869	AGTGGTGAGTATCCCCGCCT	2	5	0	1
tsrna-01868	AGTGGTGAGTATCCCCGCC	0	5	0	1
tsrna-01867	AGTGGTGAGTATCCCCGC	1	3	0	0
tsrna-01866	AGTGGTGAGTATCCCCG	0	1	0	0
tsrna-01864	AGTGGTGAGCATAGCTGCCTTCC	0	2	0	0
tsrna-01863	AGTGGTGAGCATAGCTGCCTTC	0	0	1	0
tsrna-01862	AGTGGTGAGCATAGCTGCCTT	0	1	1	0
tsrna-01861	AGTGGTGAGCATAGCTGCC	1	0	0	0
tsrna-01860	AGTGGTGAGCATAGCTGC	0	2	0	0
tsrna-01858	AGTGGTCTAGTGGTTAGGATTC	0	0	3	0
tsrna-01857	AGTGGTCTAGTGGTTAGGATT	0	1	0	1
tsrna-01856	AGTGGTCTAGTGGTTAGGAT	1	1	1	0
tsrna-01853	AGTGGTCTAAGGCGCTGGATT	0	3	0	0
tsrna-01852	AGTGGTCTAAGGCGCTGGA	0	0	1	0
tsrna-01851	AGTGGTCATCACGTTCGCC	1	0	0	0
tsrna-01849	AGTGGTATGATTCTCGCTTTG	3	2	3	0
tsrna-01848	AGTGGTATGATTCTCGCTTT	4	1	1	2
tsrna-01847	AGTGGTATGATTCTCGCT	4	2	1	1
tsrna-01846	AGTGGTATCATGCAAGATTC	0	0	1	0
tsrna-01842	AGTGGTAGAGCATTTGACTGCAGATCAAG	0	3	0	1
tsrna-01841	AGTGGTAGAGCATTTGACTGCAGATCA	2	7	1	0
tsrna-01840	AGTGGTAGAGCATTTGACTGCAGATC	1	2	3	1
tsrna-01839	AGTGGTAGAGCATTTGACTGCAGA	4	1	1	0
tsrna-01838	AGTGGTAGAGCATTTGACTGCAG	2	0	0	1
tsrna-01837	AGTGGTAGAGCATTTGACTGCA	1	2	1	0
tsrna-01836	AGTGGTAGAGCATTTGACTGC	3	2	1	1
tsrna-01835	AGTGGTAGAGCATTTGACTG	1	4	1	0
tsrna-01834	AGTGGTAGAGCATTTGACT	2	0	0	0
tsrna-01833	AGTGGTAGAGCATTTGAC	2	0	0	0
tsrna-01832	AGTGGTAGAGCATTTGA	0	1	0	0
tsrna-01830	AGTGGTAGAATTCTTGCCTGC	0	0	0	0
tsrna-01829	AGTGGTAGAATTCTCGCCTTT	3	1	2	0
tsrna-01828	AGTGGTAGAATTCTCGCCTGCCACGCGGGA	60	142	43	15
tsrna-01827	AGTGGTAGAATTCTCGCCTGCCACG	41	102	25	16
tsrna-01826	AGTGGTAGAATTCTCGCCTGCCA	14	44	7	2
tsrna-01825	AGTGGTAGAATTCTCGCCTGCC	14	25	11	5
tsrna-01824	AGTGGTAGAATTCTCGCCTGC	2	11	3	2
tsrna-01823	AGTGGTAGAATTCTCGCCTG	3	4	4	1
tsrna-01822	AGTGGTAGAATTCTCGCCTCCCACGCGGGAGACCCGGGT	4	18	2	10
tsrna-01821	AGTGGTAGAATTCTCGCCTCCCACGCGGGAGACCC	6	10	3	7
tsrna-01820	AGTGGTAGAATTCTCGCCTCCC	4	16	2	3
tsrna-01819	AGTGGTAGAATTCTCGCCTCC	5	16	0	6
tsrna-01818	AGTGGTAGAATTCTCGCCTC	4	6	1	0
tsrna-01817	AGTGGTAGAATTCTCGCCT	8	3	0	0
tsrna-01816	AGTGGTAGAATTCTCGCC	1	2	1	0
tsrna-01815	AGTGGTAGAATTCTCGC	1	0	0	1
tsrna-01810	AGTGGGTAGAGCATTTGACTGC	1	4	0	1
tsrna-01809	AGTGGGTAGAGCATTTGACTG	1	0	0	0
tsrna-01808	AGTGGGTAGAGCATTTGACT	1	1	0	0
tsrna-01807	AGTGGGTAGAGCATTTGAC	1	1	0	0
tsrna-01806	AGTGGGTAGAGCATTTGA	0	0	0	0
tsrna-01805	AGTGGGTAGAGCATCAGAC	1	0	0	0
tsrna-01804	AGTGGGTAGAGCATCAGA	0	1	0	0
tsrna-01803	AGTGGGTAGAGCATCAG	0	2	0	0
tsrna-01801	AGTGGGGTTTTGCAGTCCTTACC	0	1	0	0
tsrna-01800	AGTGGGGTTTTGCAGTCCTTA	0	3	0	0
tsrna-01798	AGTGGGGTTTTGCAGTCCT	0	1	0	0
tsrna-01797	AGTGGGGTTTTGCAGTCC	0	0	0	0
tsrna-01794	AGTGGCTGATTTGCGTTCAGTTGATG	0	1	0	0

tsrna-01790	AGTGGCTAGGATTCGGCGCT	11	25	0	20
tsrna-01789	AGTGGCTAGGATTCGGCGC	13	34	5	16
tsrna-01788	AGTGGCTAGGATTCGGCG	5	19	9	14
tsrna-01787	AGTGGCTAGGATTCGGC	5	16	1	6
tsrna-01786	AGTGGCGCAGCGGAAGCGTGCTGGGCCC	6	59	7	5
tsrna-01785	AGTGGCGCAGCGGAAGCGTGCTGGGCC	7	39	2	1
tsrna-01784	AGTGGCGCAGCGGAAGCGTGCTGGGC	2	22	0	1
tsrna-01783	AGTGGCGCAGCGGAAGCGTGCTGGG	1	15	1	1
tsrna-01782	AGTGGCGCAGCGGAAGCGTGCTG	0	0	1	0
tsrna-01781	AGTGGCGCAGCGGAAGCGTGCT	0	2	0	0
tsrna-01778	AGTGGCCTAATGGATAAGGCATCAGCCTC	1	1	4	2
tsrna-01777	AGTGGCCTAATGGATAAGGCATCA	5	1	1	0
tsrna-01776	AGTGGCCTAATGGATAAGGCATC	3	1	2	0
tsrna-01774	AGTGAAGCATTGGACTGTAAATCTAAAGA	0	1	0	0
tsrna-01773	AGTGAAGCATTGGACTGTAAATCTAAAG	0	0	0	0
tsrna-01772	AGTGAAGCATTGGACTGTAAATCTAAA	0	0	1	0
tsrna-01771	AGTGAAGCATTGGACTGTAAATCTAA	0	0	1	0
tsrna-01769	AGTGAAGCATTGGACTGTAAATCT	1	0	0	0
tsrna-01767	AGTGAAGCATTGGACTGTAAAT	0	0	0	1
tsrna-01765	AGTGAAGCATTGGACTGTAA	0	1	0	0
tsrna-01764	AGTGAAGCATTGGACTGTA	0	2	0	0
tsrna-01760	AGTCTCTTCGGGGGCGTGGGTTC	1	1	0	0
tsrna-01759	AGTCTCTTCGGGGGCGTGGGTT	0	1	3	0
tsrna-01758	AGTCTCTTCGGGGGCGTGGGT	0	1	1	0
tsrna-01757	AGTCTCTTCGGGGGCGTGGG	0	1	0	0
tsrna-01754	AGTCTCTTCGGAGGCGTGG	0	0	2	0
tsrna-01751	AGTCTCGGTGGAACCTCCA	45	90	13	7
tsrna-01750	AGTCTCATAATCTGAAGGTCGTGAGT	37	72	22	14
tsrna-01749	AGTCTCATAATCTGAAGGTCGT	0	2	0	0
tsrna-01748	AGTCTCATAATCTGAAGGTCG	0	2	0	0
tsrna-01747	AGTCTCATAATCTGAAGGTCCTG	4	20	3	4
tsrna-01746	AGTCTCATAATCTGAAGGTCCT	1	7	1	0
tsrna-01739	AGTCGGTAGAGCATGGGACTCTTAATCCC	15	22	8	7
tsrna-01738	AGTCGGTAGAGCATGGGACTCTTAATCC	17	24	13	6
tsrna-01737	AGTCGGTAGAGCATGGGACTCTTAATC	12	19	3	2
tsrna-01736	AGTCGGTAGAGCATGGGACTCTTAAT	5	13	5	4
tsrna-01735	AGTCGGTAGAGCATGGGACTCTTAA	8	13	5	2
tsrna-01734	AGTCGGTAGAGCATGGGACTCTTA	5	19	3	2
tsrna-01733	AGTCGGTAGAGCATGGGACTCTT	7	14	7	2
tsrna-01732	AGTCGGTAGAGCATGGGACTCT	9	15	5	1
tsrna-01731	AGTCGGTAGAGCATGGGACTC	10	17	5	3
tsrna-01730	AGTCGGTAGAGCATGGGACT	5	12	13	0
tsrna-01729	AGTCGGTAGAGCATGGGAC	4	16	5	1
tsrna-01728	AGTCGGTAGAGCATGGGA	5	8	1	0
tsrna-01727	AGTCGGTAGAGCATGGG	2	0	2	0
tsrna-01726	AGTCGGTAGAGCATGG	2	3	2	0
tsrna-01725	AGTCGGTAGAGCATGAGACTCTTAATCTC	17	30	6	10
tsrna-01724	AGTCGGTAGAGCATGAGACTCTTAATCT	12	30	7	8
tsrna-01723	AGTCGGTAGAGCATGAGACTCTTAATC	13	33	8	7
tsrna-01722	AGTCGGTAGAGCATGAGACTCTTAAT	21	32	8	2
tsrna-01721	AGTCGGTAGAGCATGAGACTCTTAA	13	24	8	5
tsrna-01720	AGTCGGTAGAGCATGAGACTCTTA	11	26	7	1
tsrna-01719	AGTCGGTAGAGCATGAGACTCTT	9	23	15	3
tsrna-01718	AGTCGGTAGAGCATGAGACTC	13	31	6	2
tsrna-01717	AGTCGGTAGAGCATGAGACT	9	22	2	3
tsrna-01716	AGTCGGTAGAGCATGAGAC	6	20	7	2
tsrna-01715	AGTCGGTAGAGCATGAGA	3	12	1	3
tsrna-01714	AGTCGGTAGAGCATGAG	1	3	0	0
tsrna-01713	AGTCGGTAGAGCATGA	3	1	0	0
tsrna-01712	AGTCGGTAGAGCATCAGACTTTTAATCTGAGGGTCCAGGGT	23	55	19	3
tsrna-01711	AGTCGGTAGAGCATCAGACTTTTAATCT	6	7	4	0
tsrna-01710	AGTCGGTAGAGCATCAGACTTTTAATC	5	8	3	0
tsrna-01709	AGTCGGTAGAGCATCAGACTTTTAAT	1	5	2	0
tsrna-01708	AGTCGGTAGAGCATCAGACTTTT	7	5	3	1

tsrna-01707	AGTCGGTAGAGCATCAGACTTT	1	5	2	0
tsrna-01706	AGTCGGTAGAGCATCAGACTT	5	12	1	0
tsrna-01705	AGTCGGTAGAGCATCAGACT	2	7	6	0
tsrna-01704	AGTCGGTAGAGCATCAGAC	3	9	1	0
tsrna-01703	AGTCGGTAGAGCATCAGA	1	3	0	0
tsrna-01702	AGTCGGTAGAGCATCAG	0	0	1	0
tsrna-01701	AGTCGGTAGAGCATCA	0	0	1	0
tsrna-01700	AGTCCTTGTAGTATAAACTAATACACCAG	8	2	1	1
tsrna-01699	AGTCCTGCCGCGGTCGCCA	2	3	2	1
tsrna-01696	AGTCCGTGCGAGAATACCA	1	0	0	0
tsrna-01691	AGTCCCTGTTCGGGCGCCA	12	13	3	3
tsrna-01690	AGTCCCTGTTCAGGCGCC	0	1	0	0
tsrna-01688	AGTCCCGGCGGAGTCGCCA	0	3	0	0
tsrna-01686	AGTCCCATTGGTCTAGCC	0	0	0	0
tsrna-01684	AGTCCCATCTGGGTCGCCA	1	1	2	0
tsrna-01682	AGTCCCACCAGAGTCGCCA	1	0	0	0
tsrna-01679	AGTCATTTCGATGGCGTGG	0	2	0	0
tsrna-01678	AGTCATGGAGGCCATGGGGTTGG	0	2	0	0
tsrna-01677	AGTCATGGAGGCCATGGGGTTG	0	0	0	0
tsrna-01676	AGTCATGGAGGCCATGGGGTT	1	0	0	0
tsrna-01674	AGTCATGGAGGCCATGGG	0	0	0	0
tsrna-01672	AGTCACGTCGGGGTCACCA	2	7	1	0
tsrna-01671	AGTCACGTCGGGGTCACC	0	1	0	0
tsrna-01670	AGTCACGTCGGGGTCAC	0	1	0	0
tsrna-01668	AGTCACGGTGGCCGAGTGG	0	8	0	0
tsrna-01667	AGTATCCCCGCCTGTCACGCGGGAGACCGG	14	41	14	6
tsrna-01666	AGTATCCCCGCCTGTCACGCG	1	1	0	0
tsrna-01665	AGTATCCCCGCCTGTCACGC	2	0	0	0
tsrna-01661	AGTATAGTGGTTAGTATCCCCG	0	0	0	0
tsrna-01659	AGTATAGTGGTGAGTATCCCCGC	2	7	1	1
tsrna-01658	AGTATAGTGGTGAGTATCCCCG	0	1	0	0
tsrna-01656	AGTAGTCGTGGCCGAGTGGTTAAGGC	13	6	7	8
tsrna-01655	AGTAGTCGTGGCCGAGTGGTT	7	17	4	6
tsrna-01654	AGTAGTCGTGGCCGAGTGG	7	16	1	1
tsrna-01653	AGTAGTCGTGGCCGAG	0	3	1	0
tsrna-01651	AGTAGGTAGCGCATCAGTCT	1	0	0	0
tsrna-01649	AGTACTCTGCGTTGTGGCCGCAGCAACCTCGGT	3	4	1	0
tsrna-01648	AGTACTCTGCGTTGTGGCCGCAGCAACCTC	0	1	1	0
tsrna-01647	AGTACTCTGCGTTGTGGCCGCAGC	0	2	0	0
tsrna-01645	AGTACTCTGCGTTGTGGCCGC	0	0	0	0
tsrna-01644	AGTACTCTGCGTTGTGGCCG	1	0	0	0
tsrna-01643	AGTACTCTGCGTTGTGGCC	1	1	0	0
tsrna-01642	AGTACTCTGCGTTGTGGC	0	1	0	0
tsrna-01640	AGTACTCTGCGTTGTG	1	0	0	0
tsrna-01639	AGTACCGTTAACTTCCAATTAACTAGTTTTGAC	0	1	0	0
tsrna-01638	AGTACCGTTAACTTCCAATTAACTAGTTTT	1	0	0	0
tsrna-01637	AGTACCGTTAACTTCCAATTAAC	0	0	3	0
tsrna-01636	AGTACCGTTAACTTCCAATTA	0	1	0	0
tsrna-01635	AGTACCGTTAACTTCCAATT	0	0	0	0
tsrna-01632	AGTAAGTTGCAATACTTAATTTCTGCCA	5	0	2	4
tsrna-01630	AGTAAGGTCAGCTAAATAAGCTATCGGGCCCATACCCCGAAA	15	22	21	2
tsrna-01629	AGTAAGGTCAGCTAAATAAGCTATCGGGCCCATACCCCGAAA	19	17	20	6
tsrna-01628	AGTAAGGTCAGCTAAATAAGCTATCGGGCCCATACCCCGAAA	19	22	10	5
tsrna-01627	AGTAAGGTCAGCTAAATAAGCTATCGGGCCCATACCCCGAAA	13	15	18	4
tsrna-01626	AGTAAGGTCAGCTAAATAAGCTATCGGGCCCATACCCCGAAA	15	24	26	2
tsrna-01625	AGTAAGGTCAGCTAAATAAGCTATCGGGCCCATACCCCGAAA	12	17	16	4
tsrna-01624	AGTAAGGTCAGCTAAATAAGCTATCGGGCCCATACCCCGAA	15	10	14	4
tsrna-01623	AGTAAGGTCAGCTAAATAAGCTATCGGGCCCATACCCCGA	17	19	19	3
tsrna-01622	AGTAAGGTCAGCTAAATAAGCTATCGGGCCCATACCCCG	15	28	11	2
tsrna-01621	AGTAAGGTCAGCTAAATAAGCTATCGGGCCCATACCCC	23	15	21	3
tsrna-01620	AGTAAGGTCAGCTAAATAAGCTATCGGGCCCATACCC	13	17	13	5
tsrna-01619	AGTAAGGTCAGCTAAATAAGCTATCGGGCCCATACC	6	14	17	2
tsrna-01618	AGTAAGGTCAGCTAAATAAGCTATCGGGCCCATAC	7	8	14	2
tsrna-01617	AGTAAGGTCAGCTAAATAAGCTATCGGGCCCATA	12	21	18	5

tsrna-01616	AGTAAGGTCAGCTAAATAAGCTATCGGGCCCAT	6	7	8	8
tsrna-01615	AGTAAGGTCAGCTAAATAAGCTATCGGGCCCA	9	15	6	2
tsrna-01614	AGTAAGGTCAGCTAAATAAGCTATCGGGCCC	5	10	10	3
tsrna-01613	AGTAAGGTCAGCTAAATAAGCTATCGGGCC	1	9	6	3
tsrna-01612	AGTAAGGTCAGCTAAATAAGCTATCGGGC	6	9	2	1
tsrna-01611	AGTAAGGTCAGCTAAATAAGCTATCGGG	6	2	4	2
tsrna-01610	AGTAAGGTCAGCTAAATAAGCTATCGG	1	2	2	2
tsrna-01609	AGTAAGGTCAGCTAAATAAGCTATCG	3	1	2	2
tsrna-01608	AGTAAGGTCAGCTAAATAAGCTATC	0	1	3	0
tsrna-01607	AGTAAGGTCAGCTAAATAAGCTAT	4	2	1	0
tsrna-01606	AGTAAGGTCAGCTAAATAAGCTA	1	1	0	1
tsrna-01605	AGTAAGGTCAGCTAAATAAGCT	0	3	4	1
tsrna-01604	AGTAAGGTCAGCTAAATAAGC	2	1	0	2
tsrna-01603	AGTAAGGTCAGCTAAATAAG	2	0	1	0
tsrna-01602	AGTAAGGTCAGCTAAATAA	1	0	0	0
tsrna-01601	AGTAAGGTCAGCTAAATA	1	0	0	0
tsrna-01600	AGTAAGGTCAGCTAAAT	0	2	0	0
tsrna-01599	AGTAAGGTCAGCTAAA	0	0	0	0
tsrna-01598	AGTAAATAATAGGAGCTTAAACCCCCTT	2	0	0	0
tsrna-01597	AGTAAATAATAGGAGCTTAAACCC	0	0	0	0
tsrna-01596	AGTAAATAATAGGAGCTTAAACC	0	0	1	0
tsrna-01587	AGGTTGGTGGTTCGAGCCCACCCAGGGACG	0	2	0	0
tsrna-01584	AGGTTGCGTGTTCAAGTCACGTCGGGGTC	0	1	0	0
tsrna-01583	AGGTTGCGTGTTCAAGTCACGTCGG	0	0	0	0
tsrna-01581	AGGTTGCGTGTTCAAATCACGTCGGGGTC	0	0	2	0
tsrna-01580	AGGTTGCGTGTTCAAA	1	0	0	0
tsrna-01579	AGGTTCGAGTCCTGCCGCGGTCGCCA	5	4	0	1
tsrna-01576	AGGTTCGAGTCCTGCCGCGGTCG	0	0	0	0
tsrna-01574	AGGTTCGACTCCTGGCTGGCTCGCCA	41	97	13	6
tsrna-01573	AGGTTCGACTCCTGGCTGGCTCGC	0	2	0	0
tsrna-01572	AGGTTCGACTCCTGGCTGGCTCG	0	1	0	0
tsrna-01570	AGGTTCGACTCCTGGCTGGCT	0	0	0	1
tsrna-01569	AGGTTCGAATCCTGTTCGTGACGCCA	8	10	3	2
tsrna-01568	AGGTTCGAATCCTGCCGACTACGCCA	110	261	38	16
tsrna-01567	AGGTTCGAATCCTGCCGACTACGCC	7	18	2	1
tsrna-01566	AGGTTCGAATCCTGCCGACTACGC	0	0	1	0
tsrna-01562	AGGTTCGAACCCTGCTCGCTGCGCCA	12	28	6	1
tsrna-01561	AGGTTCGAACCCTGCTCGCTGCGCC	1	3	1	0
tsrna-01560	AGGTTCGAACCCTGCTCGCTGCG	0	1	0	0
tsrna-01559	AGGTTCCGGGTTCGAGTCCCGGCGGAGTCGCC	0	1	0	0
tsrna-01555	AGGTTCCATGGTGTAATGGTTAGCACTCTG	0	4	2	0
tsrna-01554	AGGTGGTAGAGCATTTGACTGC	0	2	1	0
tsrna-01553	AGGTGGTAGAGCATTTGACTG	1	0	0	0
tsrna-01552	AGGTGGTAGAGCATTTGACT	1	1	0	0
tsrna-01550	AGGTGGTAGAGCATTTGA	0	0	0	0
tsrna-01544	AGGTGGCCCGGGTTCGACTCCCGGTATGGG	0	1	0	0
tsrna-01540	AGGTGGCACGGAGAATTTTGGATT	0	2	2	0
tsrna-01539	AGGTGGCACGGAGAATTTTGGA	0	2	0	0
tsrna-01538	AGGTGGCACGGAGAATTTTG	1	0	0	0
tsrna-01537	AGGTGGCACGGAGAATTTT	0	1	0	0
tsrna-01533	AGGTCGCTGGTTCGATTCCGGCTCGAAGG	0	1	1	0
tsrna-01532	AGGTCGCTGGTTCGATTCCGGCT	0	1	0	0
tsrna-01531	AGGTCGCTGGTTCGATTCCGGC	1	1	0	0
tsrna-01530	AGGTCGCTGGTTCGAATCCGGC	0	1	1	0
tsrna-01529	AGGTCGCTGGTTCGAATCC	0	0	0	0
tsrna-01528	AGGTCGCGGGTTCGATCCCCGTACGGGCCA	0	1	0	0
tsrna-01527	AGGTCGCGGGTTCGATCCCCGTACGGGCC	1	0	0	0
tsrna-01525	AGGTCGCGGGTTCGATCCCCGTA	0	2	0	0
tsrna-01524	AGGTCGCGGGTTCGATCCCC	0	3	0	0
tsrna-01522	AGGTCGCGGGTTCGATCC	0	1	0	0
tsrna-01520	AGGTCGCAGTCTCCCCTGGAGGCGTGGGTT	9	108	3	1
tsrna-01519	AGGTCGCAGTCTCCCCTGGAGGC	0	8	0	1
tsrna-01514	AGGTCGATGGATCGAAACC	0	1	0	0
tsrna-01508	AGGTCCTGGGTTCGAGCCCCAGTGGAACCACCA	32	38	20	20

tsrna-01507	AGGTCCTGGGTTCGAGCCCCAGTGGAACCACC	1	1	1	0
tsrna-01506	AGGTCCTGGGTTCGAGCCCCAGTGGAACCA	0	1	0	0
tsrna-01505	AGGTCCTGGGTTCGAGCCCCAGTGGAACC	0	0	0	0
tsrna-01503	AGGTCCTGGGTTCGAGCCCCA	0	0	0	0
tsrna-01501	AGGTCCTGAGTTCGAACCTCAGAGGGGGC	0	0	0	0
tsrna-01500	AGGTCCTGAGTTCGAACCTCA	0	0	1	0
tsrna-01496	AGGTCCCTGGTTCGATCCCGGGTTTCGGC	1	2	0	0
tsrna-01484	AGGTCAGCTAAATAAGCTATCGGGCCCAT	0	2	0	0
tsrna-01483	AGGTCAGCTAAATAAGCTATCGGGCCCA	0	0	0	0
tsrna-01482	AGGTCAGCTAAATAAGCTATCGGGCC	0	0	0	1
tsrna-01473	AGGTAGCGTGGCCGAGTGGTCTAAG	1	9	2	1
tsrna-01472	AGGTAGCGTGGCCGAGCGGTCTAAG	3	3	1	3
tsrna-01471	AGGTAGCGTGGCCGAGCGGTCT	2	4	1	2
tsrna-01470	AGGTAGCGTGGCCGAGCGGTC	2	2	0	1
tsrna-01469	AGGTAGCGTGGCCGAGCGGT	0	2	0	0
tsrna-01468	AGGTAGCGTGGCCGAGCGG	1	2	0	1
tsrna-01467	AGGTAGCGTGGCCGAGC	0	2	0	1
tsrna-01466	AGGTAGCGTGGCCGAG	1	0	0	0
tsrna-01464	AGGTAAAATGGCTGAGTGAAGCATTGGACT	3	2	1	0
tsrna-01463	AGGGTTCGAGTCCCTTCGTGGTCGCCA	0	5	1	0
tsrna-01458	AGGGTTCAAGTCCCTGTTCGGGCGCCA	8	12	4	1
tsrna-01457	AGGGTGGCCGAGCGGTCTAAG	0	0	0	0
tsrna-01456	AGGGTGCTTAGCTGTTAACTAAG	1	0	1	0
tsrna-01455	AGGGTGCTTAGCTGTTAACTA	1	0	0	0
tsrna-01454	AGGGTGCTTAGCTGTTAACT	0	0	2	0
tsrna-01450	AGGGTGCGAGAGGTCCCGGGTT	1	2	0	0
tsrna-01448	AGGGTCGTGGGTTCGAGCCCCACGTTGGGCGCCA	10	219	6	6
tsrna-01447	AGGGTCGTGGGTTCGAGCCCCACGTTGGGCGC	1	0	0	0
tsrna-01431	AGGGTCCAGGGTTCAAGTCCCTGTTCGGGCGC	1	0	0	0
tsrna-01425	AGGGGTTAGGCCTCTTTTTACCACCA	1	1	2	1
tsrna-01424	AGGGGTTAGGCCTCTTTTTACCACC	0	0	1	0
tsrna-01423	AGGGGTTAGGCCTCTTTTTACCAC	1	0	0	0
tsrna-01422	AGGGGTTAGGCCTCTTTTTACCA	0	0	0	1
tsrna-01421	AGGGGTTAGGCCTCTTTTTACC	0	0	1	0
tsrna-01419	AGGGGTTAGGCCTCTTTTT	0	0	0	0
tsrna-01418	AGGGGTTAGGCCTCTTTT	0	0	0	0
tsrna-01416	AGGGGTTAGAGCACTGGT	1	0	0	0
tsrna-01415	AGGGGTTAGAGCACTGG	0	2	0	0
tsrna-01410	AGGGGTATGATTCTCGGTTTGG	2	2	0	0
tsrna-01409	AGGGGTATGATTCTCGGTTTG	1	3	2	0
tsrna-01408	AGGGGTATGATTCTCGGTT	1	4	0	0
tsrna-01407	AGGGGTATGATTCTCGGT	1	1	1	1
tsrna-01406	AGGGGTATGATTCTCGG	0	1	0	0
tsrna-01405	AGGGGTATGATTCTCGCTTTGGGTG	7	5	4	7
tsrna-01404	AGGGGTATGATTCTCGCTTTG	4	6	3	6
tsrna-01403	AGGGGTATGATTCTCGCTTT	6	3	4	3
tsrna-01402	AGGGGTATGATTCTCGCTTC	9	8	7	5
tsrna-01401	AGGGGTATGATTCTCGCTT	6	4	4	4
tsrna-01400	AGGGGTATGATTCTCGCT	2	5	3	3
tsrna-01399	AGGGGTATGATTCTCGC	9	3	0	4
tsrna-01398	AGGGGTATGATTCTCG	0	0	0	1
tsrna-01397	AGGGGTAGAGCATTTGACTGCAGATCAAG	0	3	0	0
tsrna-01396	AGGGGTAGAGCATTTGACTGC	0	0	0	0
tsrna-01391	AGGGGTAGAGCATTTG	0	1	0	0
tsrna-01388	AGGGGGTATAGCTCAGTGGTAGAGCATTTG	4	2	6	2
tsrna-01387	AGGGGGTATAGCTCAGTGGT	4	6	1	2
tsrna-01386	AGGGGGTATAGCTCAGTGGGTAGAGCATTT	4	4	0	2
tsrna-01385	AGGGGGTATAGCTCAGTGGG	0	3	2	1
tsrna-01384	AGGGGGATTAGCTCAAA	8	18	15	4
tsrna-01383	AGGGGATGTAGCTCAGTGG	1	2	0	2
tsrna-01382	AGGGCCAGTGGCGCAATGGA	0	0	0	0
tsrna-01381	AGGGATTGTGGGTTCGAGTCCCATCTGGGGTGCCA	2	15	1	0
tsrna-01380	AGGGATTGTGGGTTCGAGTCCCATCTGGGGTGCC	1	3	1	0
tsrna-01379	AGGGATTGTGGGTTCGAGTCCCATCTGGGG	1	4	0	0

tsrna-01378	AGGGATTGTGGGTTCGAGTCCCATC	0	5	1	0
tsrna-01377	AGGGATTGTGGGTTCGAGTCCCACCCGGGGTACCA	1	4	1	0
tsrna-01376	AGGGATTGTGGGTTCGAGTCCCACC	3	5	2	0
tsrna-01375	AGGGATTGTGGGTTCGAGTCCCA	0	2	0	0
tsrna-01374	AGGGATTGTGGGTTCGAGTCCC	1	1	2	1
tsrna-01373	AGGGATTGTGGGTTCGAGTCC	2	4	1	0
tsrna-01372	AGGGATTGTGGGTTCGAGTC	1	3	1	0
tsrna-01371	AGGGATTGTGGGTTCGAG	0	3	2	0
tsrna-01370	AGGGATTGTGGGTTCG	2	3	1	0
tsrna-01369	AGGGATGGGTTCGATTCTCATAGTCCT	2	8	4	6
tsrna-01368	AGGGATGGGTTCGATTCTCATAGTCC	1	3	4	2
tsrna-01367	AGGGATGGGTTCGATTCTCATAGTC	0	3	3	1
tsrna-01366	AGGGATGGGTTCGATTCTCATA	0	2	2	0
tsrna-01365	AGGGATGGGTTCGATTCTCA	0	1	0	0
tsrna-01364	AGGGATGGGTTCGATTCT	0	0	1	0
tsrna-01363	AGGGATGGGTTCGATTC	0	0	1	0
tsrna-01361	AGGCTTACGACCCCTTATTTACCCCA	0	2	2	1
tsrna-01360	AGGCTTACGACCCCTTATTTACCCC	0	0	0	0
tsrna-01359	AGGCTTACGACCCCTTATTTACCC	0	0	0	0
tsrna-01357	AGGCTTACGACCCCTTATTTAC	0	0	0	0
tsrna-01352	AGGCTGCGTGTTCGAA	0	0	0	0
tsrna-01350	AGGCTCGTTGGTCTAGGGGTATGATTCTCG	0	2	0	0
tsrna-01349	AGGCTCCGTGGCGCAATGGA	0	0	0	0
tsrna-01345	AGGCTCCAGTCTCTTCGGGGGCGTGGGTTC	1	3	0	1
tsrna-01344	AGGCTCCAGTCTCTTCGGGGGCGTGGGT	1	1	0	0
tsrna-01338	AGGCTCCAGTCTCTTCGGG	0	1	0	0
tsrna-01337	AGGCTCCAGTCTCTTCGGAGGCGTGGGTTC	0	2	1	0
tsrna-01331	AGGCTCCAGTCATTTCGATGGCGTGGGTTC	0	3	6	0
tsrna-01330	AGGCTCCAGTCATTTCGATGGCGTGGGTT	1	6	1	0
tsrna-01327	AGGCTAAATCCTATATATCTTACCA	1	0	0	0
tsrna-01318	AGGCGGCCCGGGTTCGACTCCCGGTGTGGGAACCA	73	305	41	29
tsrna-01317	AGGCGGCCCGGGTTCGACTCCCGGTGTGGGAACC	5	19	6	0
tsrna-01316	AGGCGGCCCGGGTTCGACTCCCGGTGTGGGAAC	0	6	1	0
tsrna-01315	AGGCGGCCCGGGTTCGACTCCCGGTGTGGG	0	3	0	0
tsrna-01314	AGGCGGCCCGGGTTCGACTCCCGGTGTGG	0	1	0	0
tsrna-01313	AGGCGGCCCGGGTTCGACTCCCGGTGTG	0	0	0	0
tsrna-01312	AGGCGGCCCGGGTTCGACTCCCGGTG	0	0	0	0
tsrna-01310	AGGCGCTGGATTTAGGCTC	0	0	0	0
tsrna-01309	AGGCCTCTTTTTACCACCA	1	0	0	0
tsrna-01305	AGGCCTCGTGGCGCAACGGTAGCGCGTCTG	0	0	0	2
tsrna-01303	AGGCCGGTTAGCTCAGTTGGT	1	0	0	0
tsrna-01298	AGGCCCCGGGTTCGATCCCCGGCATCTCC	0	1	1	0
tsrna-01297	AGGCCCCGGGTTCGATCCCCGGCATCTC	0	0	0	0
tsrna-01289	AGGCATTGGCCTCCTAAGCCAGGGATTGTG	1	2	0	1
tsrna-01288	AGGCATTGGCCTCCTAAGCCAG	0	0	1	0
tsrna-01287	AGGCATCAGCCTCCGGAGCTGGGGATTGTG	0	1	0	0
tsrna-01286	AGGCATCAGCCTCCGGAGCTGGGGATTGT	0	0	0	0
tsrna-01283	AGGATTCGGCGCTTTCACCGCCGCGGCCC	2	19	5	8
tsrna-01282	AGGATTCGGCGCTCTCACCGCCGCGGCCCGGGTT	4	15	2	3
tsrna-01281	AGGATTCGGCGCTCTCACCGCCGCGGCCCGGGT	5	12	1	2
tsrna-01280	AGGATTCGGCGCTCTCACCGCCGCGGCCCGGG	2	14	6	3
tsrna-01279	AGGATTCGGCGCTCTCACCGCCGCGGCCCGG	7	11	4	1
tsrna-01278	AGGATTCGGCGCTCTCACCGCCGCGGCCCG	2	19	2	1
tsrna-01277	AGGATTCGGCGCTCTCACCGCCGCGGCCC	10	15	0	2
tsrna-01276	AGGATTCGGCGCTCTCACCGCCGCGGCC	5	10	4	1
tsrna-01275	AGGATTCGGCGCTCTCACCGCCGCGGC	2	12	2	2
tsrna-01274	AGGATTCGGCGCTCTCACCGCCGC	8	15	4	3
tsrna-01273	AGGATTCGGCGCTCTCACCGCC	5	5	5	4
tsrna-01272	AGGATTCGGCGCTCTCACCGC	0	5	0	0
tsrna-01271	AGGATTCGGCGCTCTCACCG	1	3	0	0
tsrna-01270	AGGATTCGGCGCTCTCACC	2	4	0	0
tsrna-01269	AGGATTCGGCGCTCTCAC	0	0	1	0
tsrna-01268	AGGATTCGGCGCTCTCA	0	0	1	0
tsrna-01267	AGGATTCGGCGCTCTC	0	2	0	0

tsrna-01266	AGGATTCCTGGTTTTCACCCAG	13	8	5	3
tsrna-01265	AGGATTCCTGGTTTTCACCCA	4	8	4	1
tsrna-01264	AGGATTCCTGGTTTTCACCC	5	5	3	6
tsrna-01263	AGGATTCCTGGTTTTCAC	2	1	2	3
tsrna-01262	AGGATTCCTGGTTTTCA	1	1	2	0
tsrna-01261	AGGATTCCTGGTTTTC	3	1	0	1
tsrna-01260	AGGATGGGGTGTGATAGGTGGCACGGAG	0	2	1	0
tsrna-01257	AGGATGGCCGAGTGGTTAAGGCGTTGGACT	0	0	0	1
tsrna-01253	AGGATGGCCGAGTGGTCTAAGGCGC	0	1	0	0
tsrna-01252	AGGATGGCCGAGTGGTCTAAGGC	1	1	0	0
tsrna-01251	AGGATGGCCGAGTGGTCTAAGG	0	3	0	0
tsrna-01250	AGGATGGCCGAGTGGTCTAAG	1	0	0	0
tsrna-01249	AGGATGGCCGAGTGGTCTAA	0	1	0	0
tsrna-01246	AGGATGGCCGAGCGGTCTAAGGCGCTGCGT	0	7	0	0
tsrna-01245	AGGATGGCCGAGCGGTCTAAGGCGCTGCG	0	3	1	0
tsrna-01244	AGGATGGCCGAGCGGTCTAAGGCGCTGC	1	6	0	1
tsrna-01243	AGGATGGCCGAGCGGTCTAAGGCGCTG	3	4	0	0
tsrna-01242	AGGATGGCCGAGCGGTCTAAGGCGCT	1	5	1	0
tsrna-01241	AGGATGGCCGAGCGGTCTAAGGC	0	2	1	0
tsrna-01240	AGGATGGCCGAGCGGTCTAAGG	0	4	1	0
tsrna-01239	AGGATGGCCGAGCGGTCTAAG	0	2	0	0
tsrna-01236	AGGATGGCCGAGCGGTCT	0	0	1	0
tsrna-01233	AGGATAACAGCTATCCATTGGTCTTAG	0	0	0	0
tsrna-01231	AGGATAACAGCTATCCATTGGTCT	0	0	0	0
tsrna-01230	AGGATAACAGCTATCCATTGGTC	1	0	0	0
tsrna-01226	AGGAGCTTAAACCCCCTTATTTCTACCA	2	2	1	0
tsrna-01225	AGGAGCTTAAACCCCCTTATTTCTACC	1	0	2	0
tsrna-01221	AGGAGATTTCAACTTAACTTGACCGCTCTGACCA	83	59	64	21
tsrna-01220	AGGAGATTTCAACTTAACTTGACCGCTCTGACC	18	8	11	0
tsrna-01219	AGGAGATTTCAACTTAACTTGACCGCTCTGAC	4	5	1	1
tsrna-01218	AGGAGATTTCAACTTAACTTGACCGCTCTGA	1	6	2	0
tsrna-01217	AGGAGATTTCAACTTAACTTGACCGCTCTG	0	2	1	0
tsrna-01214	AGGAGATTTCAACTTAACTTGACCGCT	0	0	0	0
tsrna-01213	AGGAGATTTCAACTTAACTTGACCGC	1	0	1	0
tsrna-01212	AGGAGATTTCAACTTAACTTGACCG	0	1	0	0
tsrna-01211	AGGAGATTTCAACTTAACTTGACC	0	0	0	0
tsrna-01210	AGGAGATTTCAACTTAACTTGAC	0	0	0	0
tsrna-01206	AGGAGATCCTGGGTTCGAATCCCAGCGGGGCCT	2	24	2	1
tsrna-01205	AGGAGATCCTGGGTTCGAATCCCAGCG	5	21	4	2
tsrna-01204	AGGAGATCCTGGGTTCG	9	14	10	0
tsrna-01203	AGGAGATCCTGGGTTC	6	11	1	2
tsrna-01202	AGGACTGTAGATCCTTAGGTCGCTGGTTCG	0	1	0	1
tsrna-01201	AGGACTGTAGATCCTTAGGTCGCTGGT	0	1	0	0
tsrna-01198	AGCTTTGGGTGCTAATGGTGGAGTTAAAGACTTTTTCTCTGAC	11	32	5	2
tsrna-01197	AGCTTTGGGTGCTAATGGTGGAGTTAAAGACTTTTT	5	11	7	0
tsrna-01196	AGCTTTGGGTGCTAATGGTGGAGTTAAAG	2	2	3	0
tsrna-01195	AGCTTTGGGTGCTAATGGTGGAGTTAAA	2	4	1	0
tsrna-01194	AGCTTTGGGTGCTAATGGTGGAGTTA	3	3	0	0
tsrna-01193	AGCTTTGGGTGCTAATGGTGGAGTT	1	2	0	0
tsrna-01188	AGCTTTGGGGGGTTCGATTCCTTCCTTTTTTG	5	8	3	1
tsrna-01187	AGCTTTGGGGGGTTCGATTCCTTCC	0	2	2	1
tsrna-01186	AGCTTTGGGGGGTTCGATTCCT	0	1	0	0
tsrna-01185	AGCTTTGGGGGGTTCGATTCC	0	3	0	1
tsrna-01184	AGCTTTGGGGGGTTCGATTC	0	2	0	0
tsrna-01183	AGCTTTGGGGGGTTCGATT	0	1	0	0
tsrna-01182	AGCTTTGGGGGGTTCGA	0	0	0	0
tsrna-01181	AGCTTTGGGGGGTTCG	0	0	0	0
tsrna-01180	AGCTTCTGTAGTGTAGTGGTT	2	0	0	0
tsrna-01179	AGCTTCAAACCTGCCGGGGCTTCCA	6	7	5	1
tsrna-01178	AGCTTCAAACCTGCCGGGGCTTCC	0	0	1	0
tsrna-01177	AGCTTCAAACCTGCCGGGGCTTC	0	1	0	0
tsrna-01176	AGCTTCAAACCTGCCGGGGCTT	0	0	0	0
tsrna-01174	AGCTTCAAACCTGCCGGGGC	0	2	0	0
tsrna-01171	AGCTTAGCGGTAGAGCATTTGACTG	0	2	0	0

tsrna-01170	AGCTTAAACCCCCTTATTTCTACCA	4	1	1	0
tsrna-01169	AGCTTAAACCCCCTTATTTCTACC	2	1	0	0
tsrna-01168	AGCTTAAACCCCCTTATTTCTAC	1	2	0	0
tsrna-01167	AGCTTAAACCCCCTTATTTCTA	0	3	0	0
tsrna-01165	AGCTTAAACCCCCTTATTTC	1	1	1	0
tsrna-01164	AGCTTAAACCCCCTTATTT	1	1	0	0
tsrna-01163	AGCTTAAACCCCCTTATT	0	1	0	0
tsrna-01161	AGCTGTTAACTAAGTGTTTGTGGGTTTAAGTCCCATTGGTCTA	9	3	5	1
tsrna-01160	AGCTGTTAACTAAGTGTTTGTGGGTTTAAGTCCCATTGGTCTA	7	3	3	2
tsrna-01159	AGCTGTTAACTAAGTGTTTGTGG	0	0	0	0
tsrna-01158	AGCTGTTAACTAAGTGTTTGTG	0	0	0	0
tsrna-01157	AGCTGTTAACTAAGTGTTTG	0	0	0	0
tsrna-01153	AGCTGGGGATTGTGGGTTCGAGTCCCATCTGGGTCGCC	2	7	2	0
tsrna-01152	AGCTGGGGATTGTGGGTTCG	0	2	0	0
tsrna-01151	AGCTGGGGATTGTGGGTTC	0	1	0	0
tsrna-01150	AGCTGGGGATTGTGGGTT	0	2	0	0
tsrna-01149	AGCTGGGGATTGTGGGT	0	1	0	0
tsrna-01147	AGCTGCCTTCCAAGCAGTTGACCCGGG	3	4	2	1
tsrna-01146	AGCTGCCTTCCAAGCAGTTGACCCGG	0	1	0	2
tsrna-01145	AGCTGCCTTCCAAGCAGTTGACCCG	2	0	0	1
tsrna-01144	AGCTGCCTTCCAAGCAGTTGACCC	2	1	1	0
tsrna-01143	AGCTCCGAGGTGATTTTCATATTGAATTGCA	0	0	1	2
tsrna-01142	AGCTCCGAGGTGATTTTCATATTGAATTGC	3	2	1	0
tsrna-01141	AGCTCCGAGGTGATTTTCATATTGAATTG	0	0	0	0
tsrna-01140	AGCTCCGAGGTGATTTTCATATTGAATT	0	1	3	0
tsrna-01139	AGCTCCGAGGTGATTTTCATATTGAAT	2	0	0	0
tsrna-01137	AGCTCCGAGGTGATTTTCATATTGA	0	0	1	0
tsrna-01135	AGCTCCGAGGTGATTTTCATATT	2	0	0	0
tsrna-01134	AGCTCCGAGGTGATTTTCATAT	1	0	1	0
tsrna-01133	AGCTCCGAGGTGATTTTCATA	0	1	1	0
tsrna-01132	AGCTCCGAGGTGATTTTCAT	0	0	0	1
tsrna-01131	AGCTCCGAGGTGATTTTCA	0	1	0	0
tsrna-01122	AGCTCAGTGGTAGAGCATTTGACTGCAG	2	4	0	2
tsrna-01121	AGCTCAGTGGTAGAGCATTTGACTGCA	2	2	1	0
tsrna-01120	AGCTCAGTGGTAGAGCATTTGACTGC	1	3	1	0
tsrna-01119	AGCTCAGTGGTAGAGCATTTGACTG	1	1	0	0
tsrna-01118	AGCTCAGTGGTAGAGCATTTGACTAC	2	4	0	0
tsrna-01117	AGCTCAGTGGTAGAGCATTTGACTA	2	5	1	0
tsrna-01116	AGCTCAGTGGTAGAGCATTTGACT	2	4	0	0
tsrna-01115	AGCTCAGTGGTAGAGCATTTGAC	1	1	1	0
tsrna-01113	AGCTCAGTGGTAGAGCATTTG	0	0	0	0
tsrna-01112	AGCTCAGTGGTAGAGCATTTAACT	2	0	0	0
tsrna-01111	AGCTCAGTGGTAGAGCATTT	0	2	0	0
tsrna-01110	AGCTCAGTGGGTAGAGCATTTGACTGC	2	2	0	1
tsrna-01109	AGCTCAGTGGGTAGAGCATTTGACTG	0	1	0	0
tsrna-01108	AGCTCAGTGGGTAGAGCATTTGACT	0	1	0	0
tsrna-01107	AGCTCAGTGGGTAGAGCATTTGAC	0	1	0	0
tsrna-01106	AGCTCAGTCGGTAGAGCATGGGACTCTTA	13	23	8	5
tsrna-01105	AGCTCAGTCGGTAGAGCATGGGACTCT	12	18	12	2
tsrna-01104	AGCTCAGTCGGTAGAGCATGGGACTC	9	20	11	2
tsrna-01103	AGCTCAGTCGGTAGAGCATGGGACT	7	7	3	4
tsrna-01102	AGCTCAGTCGGTAGAGCATGGGAC	7	13	8	2
tsrna-01101	AGCTCAGTCGGTAGAGCATGGGA	9	16	1	0
tsrna-01100	AGCTCAGTCGGTAGAGCATGGG	1	2	1	0
tsrna-01099	AGCTCAGTCGGTAGAGCATGG	1	4	4	0
tsrna-01098	AGCTCAGTCGGTAGAGCATGAGACTCTT	18	30	5	7
tsrna-01097	AGCTCAGTCGGTAGAGCATGAGACTCT	16	28	11	7
tsrna-01096	AGCTCAGTCGGTAGAGCATGAGACTC	17	24	8	2
tsrna-01095	AGCTCAGTCGGTAGAGCATGAGACT	11	21	8	2
tsrna-01094	AGCTCAGTCGGTAGAGCATGAGAC	12	16	2	0
tsrna-01093	AGCTCAGTCGGTAGAGCATGAGA	7	3	2	1
tsrna-01092	AGCTCAGTCGGTAGAGCATGAG	2	4	1	1
tsrna-01091	AGCTCAGTCGGTAGAGCATGA	2	0	1	0
tsrna-01090	AGCTCAGTCGGTAGAGCATG	4	1	2	0

tsrna-01089	AGCTCAGTCGGTAGAGCATCAGACTTTT	16	9	11	10
tsrna-01088	AGCTCAGTCGGTAGAGCATCAGACTTT	20	9	8	4
tsrna-01087	AGCTCAGTCGGTAGAGCATCAGACTT	9	18	5	0
tsrna-01086	AGCTCAGTCGGTAGAGCATCAGACT	11	15	6	1
tsrna-01085	AGCTCAGTCGGTAGAGCATCAGAC	10	12	2	1
tsrna-01084	AGCTCAGTCGGTAGAGCATCAGA	9	10	4	0
tsrna-01083	AGCTCAGTCGGTAGAGCATCAG	7	2	4	2
tsrna-01082	AGCTCAGTCGGTAGAGCATCA	8	1	5	1
tsrna-01081	AGCTCAGTCGGTAGAGCATC	4	1	4	0
tsrna-01080	AGCTCAGTCGGTAGAGCA	1	0	0	0
tsrna-01077	AGCTCAGGTGGTAGAGCATTTGACTGC	2	1	0	1
tsrna-01076	AGCTCAGGTGGTAGAGCATTTGACTG	1	1	0	0
tsrna-01075	AGCTCAGGTGGTAGAGCATTTGACT	1	0	0	0
tsrna-01073	AGCTCAGGGGTAGAGCATTTGACTGCAG	0	0	0	4
tsrna-01072	AGCTCAGGGGTAGAGCATTTGACTGC	1	0	0	3
tsrna-01071	AGCTCAGGGGTAGAGCATTTGACTG	1	1	1	1
tsrna-01069	AGCTCAGGGGTAGAGCATTTGAC	0	0	0	0
tsrna-01064	AGCTCACAAGAACTGCTAACTCATGCCCCCATGTCT	4	2	0	0
tsrna-01063	AGCTCACAAGAACTGCTAACTCATGCCCCC	2	0	1	1
tsrna-01062	AGCTCACAAGAACTGCTAACTCATG	2	0	0	0
tsrna-01061	AGCTCACAAGAACTGCTAACTCA	1	0	0	0
tsrna-01060	AGCTCACAAGAACTGCTAACTC	1	0	0	0
tsrna-01059	AGCTCACAAGAACTGCTAACT	0	0	1	0
tsrna-01058	AGCTCACAAGAACTGCTAAC	0	0	1	0
tsrna-01052	AGCTATCGGGCCCATACCCCGAAAATGTTGGTT	3	0	1	2
tsrna-01051	AGCTATCGGGCCCATACCCCGAAAATGTTG	2	1	0	0
tsrna-01050	AGCTATCGGGCCCATACCCCGAAAATGTT	0	0	0	0
tsrna-01049	AGCTATCGGGCCCATACCCCGAAA	0	1	1	0
tsrna-01048	AGCTATCGGGCCCATACCCCGAA	0	0	0	0
tsrna-01047	AGCTATCGGGCCCATACCCCGA	0	1	4	0
tsrna-01046	AGCTATCGGGCCCATACCCCG	0	1	0	0
tsrna-01045	AGCTATCGGGCCCATACCCC	0	0	0	0
tsrna-01044	AGCTATCGGGCCCATACCC	0	0	0	0
tsrna-01043	AGCTATCGGGCCCATACC	0	0	0	2
tsrna-01042	AGCTATCGGGCCCATA	0	0	1	0
tsrna-01041	AGCTATCCATTGGTCTTAGGCCCCA	1	1	1	0
tsrna-01040	AGCTATCCATTGGTCTTAGGCCCC	0	1	2	0
tsrna-01037	AGCTATCCATTGGTCTTAGGC	0	0	0	0
tsrna-01035	AGCTATCCATTGGTCTTAG	0	0	0	0
tsrna-01028	AGCTAAATAAGCTATCGGGCCCATACC	1	1	2	2
tsrna-01027	AGCTAAATAAGCTATCGGGCCCA	0	0	1	0
tsrna-01026	AGCTAAATAAGCTATCGGGCCC	0	0	0	0
tsrna-01020	AGCGTTGGTGGTATAGTGGTAAG	0	1	0	0
tsrna-01017	AGCGTGGCCGAGTGGTCTAAGGCGCTGG	0	0	0	0
tsrna-01009	AGCGTGGCCGAGCGGTCTAAGGCGCTGGA	0	0	0	2
tsrna-01006	AGCGTGGCCGAGCGGTCTAAGG	0	1	0	0
tsrna-01001	AGCGTGCTGGGCCCATAAC	0	1	0	0
tsrna-00999	CTCAGTCGGTAGAGCATGGGACTCTTA	9	20	4	3
tsrna-00998	CTCAGTCGGTAGAGCATGGGACTCTT	7	15	4	2
tsrna-00997	CTCAGTCGGTAGAGCATGGGACTCT	11	18	8	4
tsrna-00996	CTCAGTCGGTAGAGCATGGGACTC	8	16	8	3
tsrna-00995	CTCAGTCGGTAGAGCATGGGACT	11	14	6	0
tsrna-00994	CTCAGTCGGTAGAGCATGGGAC	12	19	8	3
tsrna-00993	CTCAGTCGGTAGAGCATGGGA	9	10	0	1
tsrna-00992	CTCAGTCGGTAGAGCATGGG	3	1	1	0
tsrna-00991	CTCAGTCGGTAGAGCATGG	1	3	0	0
tsrna-00990	CTCAGTCGGTAGAGCATGAGACTCTTA	13	31	11	5
tsrna-00989	CTCAGTCGGTAGAGCATGAGACTCT	17	34	4	10
tsrna-00988	CTCAGTCGGTAGAGCATGAGACTC	17	22	7	6
tsrna-00987	CTCAGTCGGTAGAGCATGAGACT	9	23	6	5
tsrna-00986	CTCAGTCGGTAGAGCATGAGAC	13	14	4	1
tsrna-00985	CTCAGTCGGTAGAGCATGAGA	6	4	4	0
tsrna-00984	CTCAGTCGGTAGAGCATGAG	1	6	1	0
tsrna-00983	CTCAGTCGGTAGAGCATGA	0	1	1	0

tsrna-00982	CTCAGTCGGTAGAGCATG	3	1	2	1
tsrna-00981	CTCAGTCGGTAGAGCATCAGACTTTT	16	11	5	1
tsrna-00980	CTCAGTCGGTAGAGCATCAGACTTT	17	10	4	2
tsrna-00979	CTCAGTCGGTAGAGCATCAGACT	18	16	6	1
tsrna-00978	CTCAGTCGGTAGAGCATCAGAC	10	5	6	0
tsrna-00977	CTCAGTCGGTAGAGCATCAGA	13	7	0	0
tsrna-00976	CTCAGTCGGTAGAGCATCAG	6	5	1	0
tsrna-00975	CTCAGTCGGTAGAGCATCA	6	5	5	0
tsrna-00974	CTCAGTCGGTAGAGCATC	4	6	0	1
tsrna-00972	CTCAGTAAGTTGCAATACTTAATTTCTGCCA	2	1	2	1
tsrna-00971	CTCAGTAAGTTGCAATACTTAATTTCTGCC	0	0	0	0
tsrna-00970	CTCAGTAAGTTGCAATACTTAATTTCTGC	0	0	0	0
tsrna-00969	CTCAGTAAGTTGCAATACTTAATTTCTG	0	0	0	0
tsrna-00968	CTCAGTAAGTTGCAATACTTAATTTCT	0	0	1	0
tsrna-00961	CTCAGTAAGTTGCAATACT	0	0	0	0
tsrna-00957	CTCAGGGTCGTGGGTTCGAGCCCCACGTTG	1	2	0	0
tsrna-00956	CTCAGGGTCGTGGGTTCG	0	2	0	0
tsrna-00952	CTCAGGGGTAGAGCATTTGACTGC	0	0	0	0
tsrna-00951	CTCAGGGGTAGAGCATTTGACTG	0	1	0	0
tsrna-00950	CTCAGGGGTAGAGCATTTGACT	0	0	0	0
tsrna-00949	CTCAGGGGTAGAGCATTTGAC	0	0	0	0
tsrna-00948	CTCAGGGGTAGAGCATTTGA	0	0	0	0
tsrna-00947	CTCAGGGATGGGTTCGATTCTCATAGTCCT	11	15	12	10
tsrna-00946	CTCAGGGATGGGTTCGATTCTCA	5	7	2	0
tsrna-00945	CTCAGGGATGGGTTCGATTCTC	3	7	6	2
tsrna-00944	CTCAGGGATGGGTTCGATTCT	1	2	3	1
tsrna-00943	CTCAGGGATGGGTTCGATTC	1	1	1	1
tsrna-00942	CTCAGGGATGGGTTCGATT	3	3	1	0
tsrna-00941	CTCAGGGATGGGTTCGA	0	3	2	0
tsrna-00940	CTCAGGGATGGGTTCG	1	1	0	0
tsrna-00938	CTCAGAGGGGGCACCA	0	0	0	0
tsrna-00935	CTCACCGCCGCGGCCCGGGTTCGATTCCCGGTCAGGGAACC	38	307	15	8
tsrna-00934	CTCACCGCCGCGGCCCGGGTTCGATTCCCGGTCAGGGAACC	4	14	8	1
tsrna-00933	CTCACCGCCGCGGCCCGGGTTCGATTCCCGGTCAGGGAAC	2	7	2	0
tsrna-00932	CTCACCGCCGCGGCCCGGGTTCGATTCCCGGTCAGGGAA	1	2	0	0
tsrna-00931	CTCACCGCCGCGGCCCGGGTTCGATTCCCGGTCAGGGA	0	2	0	0
tsrna-00930	CTCACCGCCGCGGCCCGGGTTCGATTCCCGGTCAGGG	0	0	0	0
tsrna-00929	CTCACCGCCGCGGCCCGGGTTCGATTCCCGGTCAGG	0	1	1	0
tsrna-00928	CTCACCGCCGCGGCCCGGGTTCGATTCCCG	0	1	0	0
tsrna-00925	CTCACCGCCGCGGCCCGGGTTC	0	1	0	0
tsrna-00923	CTCACCGCCGCGGCCCGGGT	1	0	0	0
tsrna-00918	CTCACATCACCCCATAAACACCA	7	3	1	3
tsrna-00916	CTCACATCACCCCATAAACAC	1	0	0	0
tsrna-00909	CTCACACGCGAAAGGTCCCCGGTTT	6	28	5	3
tsrna-00908	CTCACACGCGAAAGGTCCCCGGTTCGAAAC	9	30	3	3
tsrna-00907	CTCACACGCGAAAGGTCCCCGGTTC	5	31	1	2
tsrna-00906	CTCACACGCGAAAGGTCCCCGGTT	6	27	1	0
tsrna-00905	CTCACACGCGAAAGGTCCCCGGT	4	23	4	1
tsrna-00904	CTCACACGCGAAAGGTCCCCGG	0	1	0	0
tsrna-00903	CTCACACGCGAAAGGTCCCCG	1	0	0	0
tsrna-00900	CTCACACGCGAAAGGTCC	0	0	0	0
tsrna-00898	CTCACACGCGAAAAGTCCCCGGT	0	1	0	0
tsrna-00896	CTCACAAGAACTGCTAACTCATGCCCCCATGTCTAACAACATG	4	1	5	0
tsrna-00895	CTCACAAGAACTGCTAACTCATGCCCCCATGTCTAACAACATG	1	1	4	1
tsrna-00894	CTCACAAGAACTGCTAACTCATGCCCCCATGTCTAACAACATG	4	2	4	2
tsrna-00893	CTCACAAGAACTGCTAACTCATGCCCCCATGTCTAA	3	4	1	2
tsrna-00892	CTCACAAGAACTGCTAACTCATGCCCCCAT	1	1	0	0
tsrna-00891	CTCACAAGAACTGCTAACTCATGCCCCC	1	0	3	0
tsrna-00890	CTCACAAGAACTGCTAACTCATGCCCC	0	0	1	0
tsrna-00889	CTCACAAGAACTGCTAACTCATGCCC	2	0	1	0
tsrna-00888	CTCACAAGAACTGCTAACTCATGCC	0	2	1	0
tsrna-00887	CTCACAAGAACTGCTAACTCATGC	1	0	0	0
tsrna-00886	CTCACAAGAACTGCTAACTCATG	1	1	0	0
tsrna-00885	CTCACAAGAACTGCTAACTCAT	0	0	1	0

tsrna-00884	CTCACAAGAACTGCTAACTCA	2	0	0	0
tsrna-00883	CTCACAAGAACTGCTAACTC	0	0	0	0
tsrna-00882	CTCACAAGAACTGCTAACT	0	0	0	0
tsrna-00881	CTCACAAGAACTGCTAAC	1	0	0	0
tsrna-00878	CTCAAGTTCTGGTCTCCGGATGGAG	1	1	0	0
tsrna-00876	CTCAAGTTCTGGTCTCCGGATGG	0	0	0	0
tsrna-00875	CTCAAGTTCTGGTCTCCGGATG	0	1	0	0
tsrna-00874	CTCAAGTTCTGGTCTCCGGA	0	3	0	0
tsrna-00872	CTCAAGTTCTGGTCTCCGC	0	1	0	0
tsrna-00871	CTCAAGTTCTGGTCTCCG	0	1	0	0
tsrna-00870	CTCAAGTTCTGGTCTCCAATGGAGGCGTGG	2	1	1	0
tsrna-00869	CTCAAGTTCTGGTCTCCAATGGAG	0	2	0	0
tsrna-00868	CTCAAGTTCTGGTCTCCAATGG	0	0	0	0
tsrna-00867	CTCAAGTTCTGGTCTCCAATG	0	0	1	0
tsrna-00865	CTCAAGTTCTGGTCTCCAA	0	2	0	0
tsrna-00864	CTCAAGTTCTGGTCTCCA	0	2	0	1
tsrna-00862	CTCAAGTTCTGGTCTC	0	3	0	0
tsrna-00859	CTCAAAGCAATACACTGAAAAT	0	0	0	0
tsrna-00858	CTCAAAGCAATACACTGAAAA	1	0	0	0
tsrna-00856	CTCAAAGCAATACACTGAA	0	1	1	0
tsrna-00852	CTATCGGGCCCATACCCCGAAAATGTTGGTTATACC	0	0	4	0
tsrna-00851	CTATCGGGCCCATACCCCGAAAATGTTGGTT	1	0	4	0
tsrna-00850	CTATCGGGCCCATACCCCGAAAATGTTGGT	1	0	0	0
tsrna-00849	CTATCGGGCCCATACCCCGAAAATGTTGG	5	0	0	1
tsrna-00848	CTATCGGGCCCATACCCCGAAAATGTTG	2	0	1	0
tsrna-00847	CTATCGGGCCCATACCCCGAAA	0	1	0	0
tsrna-00846	CTATCGGGCCCATACCCCGAA	0	0	1	0
tsrna-00844	CTATCGGGCCCATACCC	0	0	1	0
tsrna-00843	CTATCGGGCCCATACC	0	0	1	0
tsrna-00842	CTATCCATTGGTCTTAGGCCCCA	0	0	1	0
tsrna-00841	CTATCCATTGGTCTTAGGCCCC	0	0	0	0
tsrna-00834	CTATAGCTCAGGGGTTAGAGCACTGG	0	0	1	0
tsrna-00833	CTAGTTTTGACAACATTCAAAAAAGAGTAC	0	0	0	0
tsrna-00832	CTAGTGGTTAGGATTCGGCGCTCTCACCGCCGCGGCCCGGG	60	71	28	33
tsrna-00831	CTAGTGGTTAGGATTCGGCGCTCTCACCGCCGCGGCCC	59	70	39	21
tsrna-00830	CTAGTGGTTAGGATTCGGCGCTCTCACCGCCGCGGC	38	53	24	21
tsrna-00829	CTAGTGGTTAGGATTCGGCGCTCTCACCGC	50	37	34	16
tsrna-00828	CTAGTGGTTAGGATTCGGCGCTCTCACC	39	49	21	17
tsrna-00827	CTAGTGGTTAGGATTCGGCGCTCTCAC	41	49	14	19
tsrna-00826	CTAGTGGTTAGGATTCGGCGCTCTCA	36	58	33	22
tsrna-00825	CTAGTGGTTAGGATTCGGCGCTCTC	41	54	20	17
tsrna-00824	CTAGTGGTTAGGATTCGGCGCTCT	38	34	23	14
tsrna-00823	CTAGTGGTTAGGATTCGGCGCTC	33	43	19	17
tsrna-00822	CTAGTGGTTAGGATTCGGCGCT	28	39	23	14
tsrna-00821	CTAGTGGTTAGGATTCGGCGC	21	16	12	4
tsrna-00820	CTAGTGGTTAGGATTCGGCG	17	7	7	7
tsrna-00819	CTAGTGGTTAGGATTCGGC	8	6	3	3
tsrna-00818	CTAGTGGTTAGGATTCGG	2	0	2	0
tsrna-00817	CTAGTGGTTAGGATTCG	0	1	4	2
tsrna-00816	CTAGTGGTTAGGATTC	2	1	1	0
tsrna-00815	CTAGTGGTATGATTCTCGCTTT	12	2	2	4
tsrna-00814	CTAGTGGTATGATTCTCGCTT	7	2	2	7
tsrna-00813	CTAGTGGTATGATTCTCGC	3	5	1	2
tsrna-00810	CTAGTGGCTAGGATTCGGCGCT	11	38	9	27
tsrna-00809	CTAGTGGCTAGGATTCGGCGC	9	28	8	23
tsrna-00808	CTAGTGGCTAGGATTCGGCG	11	36	5	25
tsrna-00806	CTAGTGGCTAGGATTCG	0	0	0	0
tsrna-00805	CTAGGTTCGACTCCTGGCTGGCTCGCCA	45	106	16	8
tsrna-00804	CTAGGTTCGACTCCTGGCTGGCTCGCC	4	2	0	0
tsrna-00803	CTAGGTTCGACTCCTGGCTGGCTCGC	0	0	0	1
tsrna-00802	CTAGGTTCGACTCCTGGCTGGCTCG	1	0	0	0
tsrna-00798	CTAGGGGTATGATTCTCGGTTTGG	6	3	1	3
tsrna-00797	CTAGGGGTATGATTCTCGGTTTG	6	0	0	2
tsrna-00796	CTAGGGGTATGATTCTCGGTTT	6	0	1	1

tsrna-00795	CTAGGGGTATGATTCTCGGTT	3	4	1	1
tsrna-00794	CTAGGGGTATGATTCTCGGT	1	1	1	2
tsrna-00793	CTAGGGGTATGATTCTCGG	1	0	0	0
tsrna-00792	CTAGGGGTATGATTCTCGCTTTG	11	9	7	8
tsrna-00791	CTAGGGGTATGATTCTCGCTTT	7	12	5	14
tsrna-00790	CTAGGGGTATGATTCTCGCTTCGG	9	11	3	12
tsrna-00789	CTAGGGGTATGATTCTCGCTTCG	16	12	7	15
tsrna-00788	CTAGGGGTATGATTCTCGCTTC	15	11	5	7
tsrna-00787	CTAGGGGTATGATTCTCGCTT	10	14	1	5
tsrna-00786	CTAGGGGTATGATTCTCGCT	11	9	3	6
tsrna-00785	CTAGGGGTATGATTCTCGC	5	6	3	5
tsrna-00784	CTAGGGGTATGATTCTCG	1	0	0	0
tsrna-00781	CTAGCTCAGTCGGTAGAGCATGGGACTCT	12	19	9	3
tsrna-00780	CTAGCTCAGTCGGTAGAGCATGGGACTC	11	15	5	3
tsrna-00779	CTAGCTCAGTCGGTAGAGCATGGGACT	15	14	10	4
tsrna-00778	CTAGCTCAGTCGGTAGAGCATGGGAC	19	17	6	0
tsrna-00777	CTAGCTCAGTCGGTAGAGCATGGGA	9	8	4	0
tsrna-00776	CTAGCTCAGTCGGTAGAGCATGGG	1	6	0	0
tsrna-00775	CTAGCTCAGTCGGTAGAGCATGG	1	1	1	0
tsrna-00774	CTAGCTCAGTCGGTAGAGCATGAGACTCTT	14	31	6	4
tsrna-00773	CTAGCTCAGTCGGTAGAGCATGAGACTC	21	27	10	3
tsrna-00772	CTAGCTCAGTCGGTAGAGCATGAGACT	13	24	8	0
tsrna-00771	CTAGCTCAGTCGGTAGAGCATGAGAC	11	18	2	3
tsrna-00770	CTAGCTCAGTCGGTAGAGCATGAGA	1	7	1	3
tsrna-00769	CTAGCTCAGTCGGTAGAGCATGAG	4	3	1	0
tsrna-00768	CTAGCTCAGTCGGTAGAGCATGA	0	5	1	0
tsrna-00767	CTAGCTCAGTCGGTAGAGCATG	0	1	1	0
tsrna-00762	CTAGCGGTTAGGATTCCTGGTTTTCACCC	19	14	10	7
tsrna-00761	CTAGCGGTTAGGATTCCTGGTTTTC	4	8	0	3
tsrna-00760	CTAGCGGTTAGGATTCCTGGTTTT	2	4	3	1
tsrna-00759	CTAGCGGTTAGGATTCCTGGTTT	3	7	1	1
tsrna-00758	CTAGCGGTTAGGATTCCTGGTT	2	4	4	1
tsrna-00757	CTAGCGGTTAGGATTCCTGGT	4	6	2	0
tsrna-00756	CTAGCGGTTAGGATTCCTGG	2	1	2	1
tsrna-00754	CTAGCGGTTAGGATTCCT	1	0	0	0
tsrna-00750	CTAATTCAAAGGTTGTGGGT	2	2	0	0
tsrna-00748	CTAATTCAAAGGTTCCGGGTTCGAGTCCCGGCGGAGTCGCC[illegible]	2	8	0	1
tsrna-00747	CTAATTCAAAGGTTCCGGGTTCG	2	8	0	0
tsrna-00746	CTAATTCAAAGGTTCCGGGTT	2	4	0	0
tsrna-00745	CTAATTCAAAGGTTCCGGGT	0	2	0	0
tsrna-00744	CTAATTCAAAGGTTCCGG	0	1	0	0
tsrna-00743	CTAATGGTGGAGTTAAAGACTTTTTCTCTGACCA	39	51	23	18
tsrna-00742	CTAATGGTGGAGTTAAAGACTTTTTCTCTGACC	14	34	6	2
tsrna-00741	CTAATGGTGGAGTTAAAGACTTTTTCTCTG	13	15	3	0
tsrna-00740	CTAATGGTGGAGTTAAAGACTTTTTCTCT	6	9	1	0
tsrna-00739	CTAATGGTGGAGTTAAAGACTTTTT	6	9	3	1
tsrna-00738	CTAATGGTGGAGTTAAAGACTTTT	3	6	5	0
tsrna-00737	CTAATGGTGGAGTTAAAGACTTT	1	5	4	0
tsrna-00736	CTAATGGTGGAGTTAAAGACTT	1	3	6	0
tsrna-00735	CTAATGGTGGAGTTAAAGACT	0	2	1	0
tsrna-00734	CTAATGGTGGAGTTAAAGAC	2	2	3	0
tsrna-00733	CTAATGGTGGAGTTAAAGA	0	3	0	0
tsrna-00732	CTAATGGTGGAGTTAAAG	0	1	0	0
tsrna-00731	CTAATGGTGGAGTTAAA	0	2	1	1
tsrna-00730	CTAATGGTGGAGTTAA	2	3	2	1
tsrna-00729	CTAATGGATAAGGCGTCTGATTC	0	1	0	0
tsrna-00728	CTAATGGATAAGGCGTCTGATT	1	1	0	0
tsrna-00727	CTAATGGATAAGGCGTCTGACTTCG	0	2	1	0
tsrna-00726	CTAATGGATAAGGCGTCTGACTTC	0	2	0	1
tsrna-00724	CTAATGGATAAGGCGTCTGACT	1	0	0	1
tsrna-00722	CTAATGGATAAGGCGTCTG	1	0	0	0
tsrna-00721	CTAATGGATAAGGCATTGGCCTCCTAAGCC	6	2	7	0
tsrna-00720	CTAATGGATAAGGCATTG	3	4	0	0
tsrna-00719	CTAATGGATAAGGCATT	0	0	1	0

tsrna-00718	CTAATGGATAAGGCATCAGCCTCC	2	1	7	2
tsrna-00717	CTAATGGATAAGGCATCAGCCTC	1	2	3	0
tsrna-00716	CTAATGGATAAGGCATCAGCCT	3	2	4	0
tsrna-00715	CTAATGGATAAGGCATCAGCC	5	0	3	0
tsrna-00714	CTAATGGATAAGGCATCA	0	6	4	0
tsrna-00713	CTAATGGATAAGGCATC	1	1	0	0
tsrna-00711	CTAATGGATAAGGCACTGG	83	41	52	19
tsrna-00710	CTAATGGATAAGGCACTG	76	41	66	16
tsrna-00709	CTAATGGATAAGGCACT	6	3	4	1
tsrna-00708	CTAATGGATAAGGCAC	1	2	0	0
tsrna-00706	CTAATCCATTGTGCTTTGCACGCGTGGGTT	0	7	0	0
tsrna-00698	CTAATAACGCCAAGGTCGCGGGT	41	166	25	19
tsrna-00697	CTAATAACGCCAAGGTCGCGG	1	2	0	0
tsrna-00696	CTAAGTGTTTGTGGGTTTAAGTCCCATTGG	0	0	1	0
tsrna-00695	CTAAGTGTTTGTGGGTTTAAGTC	0	0	0	0
tsrna-00694	CTAAGTGTTTGTGGGTTTAAGT	0	1	0	0
tsrna-00693	CTAAGTGTTTGTGGGTTTAA	0	0	1	0
tsrna-00692	CTAAGTGTTTGTGGGTTTA	0	1	0	0
tsrna-00684	CTAAGCCAGGGATTGTGGGTTCGAGTCCCATCTGGGGTGCCA	129	416	93	37
tsrna-00683	CTAAGCCAGGGATTGTGGGTTCGAGTCCCATCTGGGGTGCC	130	386	90	43
tsrna-00682	CTAAGCCAGGGATTGTGGGTTCGAGTCCCACCTGGGGTGCCA	140	399	84	46
tsrna-00681	CTAAGCCAGGGATTGTGGGTTCGAGTCCCACCTGGGGTGCC	161	439	86	44
tsrna-00680	CTAAGCCAGGGATTGTGGGTTCGAGTCCCACCTGGGGTACCA	129	430	86	46
tsrna-00679	CTAAGCCAGGGATTGTGGGTTCGAGTCCCACCTGGGGTACC	132	415	76	41
tsrna-00678	CTAAGCCAGGGATTGTGGGTTCGAGTCCCACCCGGGGTACCA	145	373	99	42
tsrna-00677	CTAAGCCAGGGATTGTGGGTTCGAGTCCCACCCGGGGTACC	126	412	94	47
tsrna-00676	CTAAGCCAGGGATTGTGGGTTCGAGTCCC	133	430	77	36
tsrna-00675	CTAAGCCAGGGATTGTGGGTTCGAGTC	133	401	91	38
tsrna-00674	CTAAGCCAGGGATTGTGGGTTCGAG	128	389	74	39
tsrna-00673	CTAAGCCAGGGATTGTGGGTTCGA	126	411	73	33
tsrna-00672	CTAAGCCAGGGATTGTGGGTTCG	135	371	87	51
tsrna-00671	CTAAGCCAGGGATTGTGGGTTC	104	424	93	37
tsrna-00670	CTAAGCCAGGGATTGTGGGTT	123	404	77	48
tsrna-00669	CTAAGCCAGGGATTGTGGGT	105	345	64	33
tsrna-00668	CTAAGCCAGGGATTGTGGG	1	10	0	2
tsrna-00667	CTAAGCCAGGGATTGTGG	1	2	0	0
tsrna-00666	CTAAGCCAGGGATTGTG	2	1	0	0
tsrna-00665	CTAAGCCAGGGATTGT	1	0	0	0
tsrna-00662	CTAACTCATGCCCCCATGTCTAACAACATGGCTTTCTCACCA	17	3	37	1
tsrna-00661	CTAACTCATGCCCCCATGTCTAACAACATGGCTTTCTCACC	9	1	19	1
tsrna-00660	CTAACTCATGCCCCCATGTCTAACAACATGGCT	0	2	1	0
tsrna-00659	CTAACTCATGCCCCCATGTCTAACAACATG	0	1	0	0
tsrna-00658	CTAACTCATGCCCCCATGTCTAACA	1	0	0	0
tsrna-00650	CTAACAACATGGCTTTCTCACCA	0	1	1	0
tsrna-00649	CTAACAACATGGCTTTCTCACC	0	0	0	0
tsrna-00647	CTAACAACATGGCTTTCTCA	0	0	1	0
tsrna-00646	CTAACAACATGGCTTTCTC	0	0	0	0
tsrna-00640	CTAAATAAGCTATCGGGCCCATA	0	5	1	0
tsrna-00639	CTAAATAAGCTATCGGGCCCAT	0	0	1	0
tsrna-00638	CTAAATAAGCTATCGGGCCCA	0	0	0	0
tsrna-00637	CTAAATAAGCTATCGGGCCC	0	0	1	0
tsrna-00634	CTAAAGGTCCCTGGTTCGATCCCGGGTTTC	10	22	16	4
tsrna-00633	CTAAAGGTCCCTGGTTCGATC	10	14	9	6
tsrna-00632	CTAAAGGTCCCTGGTTCG	13	13	7	2
tsrna-00631	CTAAAGGTCCCTGGTTC	13	12	9	3
tsrna-00630	CTAAAGACAGGGGTTAGGCCTCTTTTTACCACCA	7	3	11	1
tsrna-00629	CTAAAGACAGGGGTTAGGCCTCTTTTTACCACC	2	2	1	1
tsrna-00628	CTAAAGACAGGGGTTAGGCCTCTTTTTACC	0	2	2	0
tsrna-00627	CTAAAGACAGGGGTTAGGCCTCT	1	1	1	0
tsrna-00626	CTAAAGACAGGGGTTAGGCCT	0	1	0	0
tsrna-00625	CTAAACCATCCTCTGCTACCA	1	4	3	3
tsrna-00624	CGTTTCCGTAGTGTAGTGGTTATCACGTTCGCCTC	404	152	428	125
tsrna-00623	CGTTTCCGTAGTGTAGTGGTTATCACGTTCGCCT	458	178	446	104
tsrna-00622	CGTTTCCGTAGTGTAGTGGTTATCACGTTCGCC	363	151	391	118

tsrna-00621	CGTTTCCGTAGTGTAGTGGTCATCACGTTCGCCT	98	50	104	39
tsrna-00620	CGTTGTGGCCGCAGCAACCTCGGT	0	1	0	0
tsrna-00618	CGTTGGTGGTATAGTGGTTAGCATAGCTG	1	1	0	0
tsrna-00612	CGTTGGTGGTATAGTGGTGAGCATAGCTGC	0	3	0	0
tsrna-00611	CGTTGGTGGTATAGTGGTGAGCATAGCTG	1	1	1	0
tsrna-00610	CGTTGGTGGTATAGTGGTGAGCATAGCT	0	3	0	0
tsrna-00609	CGTTGGTGGTATAGTGGTGAGCATAGC	0	1	0	0
tsrna-00608	CGTTGGTGGTATAGTGGTGAGCATAG	0	1	0	0
tsrna-00607	CGTTGGTGGTATAGTGGTGAGCATA	0	3	0	0
tsrna-00606	CGTTGGTGGTATAGTGGTGAGCA	0	1	0	0
tsrna-00605	CGTTGGTGGTATAGTGGTGAGC	0	0	0	0
tsrna-00602	CGTTGGTGGTATAGTGGTAAGCATAGCTGC	2	0	0	1
tsrna-00597	CGTTGGTCTAGGGGTATGATTCTCGC	14	12	4	12
tsrna-00596	CGTTGGTCTAGGGGTATGATTCTCG	1	1	2	0
tsrna-00595	CGTTGGTCTAGGGGTATGATTCT	1	0	0	0
tsrna-00594	CGTTGGTCTAGGGGTATGATTC	0	0	0	1
tsrna-00588	CGTTCGATTCCCGGTCAGGGAACCA	8	261	5	3
tsrna-00587	CGTTCGATTCCCGGTCAGGGAACC	2	13	0	0
tsrna-00586	CGTTCGATTCCCGGTCAGGGAAC	0	4	0	0
tsrna-00584	CGTTCAGTTGATGCAGAGTGGGGTTTTGCA	1	2	2	1
tsrna-00583	CGTTCAGTTGATGCAGAGTGGGGTTTTGC	0	0	1	1
tsrna-00582	CGTTCAGTTGATGCAGAGTGGGGTTTTG	0	2	0	1
tsrna-00581	CGTTCAGTTGATGCAGAGTGGGGTTTT	0	0	0	1
tsrna-00580	CGTTCAGTTGATGCAGAGTGGGGTTT	0	2	0	0
tsrna-00579	CGTTCAGTTGATGCAGAGTGGGGTT	0	0	0	1
tsrna-00578	CGTTCAGTTGATGCAGAGTGGGGT	1	0	0	0
tsrna-00577	CGTTCAGTTGATGCAGAGTGGGG	1	0	1	0
tsrna-00576	CGTTCAGTTGATGCAGAGTGGG	0	0	0	0
tsrna-00575	CGTTCAGTTGATGCAGAGTGG	1	0	0	0
tsrna-00574	CGTTCAGTTGATGCAGAGTG	0	0	1	0
tsrna-00563	CGTTAGTATAGTGGTTAGTATCCCCGCC	0	1	0	0
tsrna-00557	CGTTAGTATAGTGGTGAGTATCCCCGCCTG	1	3	0	0
tsrna-00556	CGTTAGTATAGTGGTGAGTATCCCCGCC	1	9	0	0
tsrna-00555	CGTTAGTATAGTGGTGAGTATCCCCGC	2	3	2	0
tsrna-00554	CGTTAGTATAGTGGTGAGTATCCCCG	0	0	0	0
tsrna-00552	CGTTAGTATAGTGGTGAGTATCCC	0	1	0	0
tsrna-00551	CGTTAGTATAGTGGTGAGTATCC	0	1	0	0
tsrna-00543	CGTTAACTTCCAATTAACTAG	1	0	0	0
tsrna-00541	CGTGTTCGAATCACGTCGGGGTCACC	1	0	0	0
tsrna-00534	CGTGGTTGTAGTCCGTGCGAGAATACCA	7	20	9	5
tsrna-00533	CGTGGTTGTAGTCCGTGCGAGAATACC	2	6	2	0
tsrna-00532	CGTGGTTGTAGTCCGTGCGAGAATAC	4	4	0	0
tsrna-00531	CGTGGTTGTAGTCCGTGCGAGAATA	2	3	1	1
tsrna-00530	CGTGGTTGTAGTCCGTGCGAGAAT	0	1	2	0
tsrna-00529	CGTGGTTGTAGTCCGTGCGAGAA	1	0	2	0
tsrna-00528	CGTGGTTGTAGTCCGTGCGAGA	2	0	0	0
tsrna-00527	CGTGGTTGTAGTCCGTGCGAG	2	0	1	0
tsrna-00526	CGTGGTTGTAGTCCGTGCGA	0	0	0	0
tsrna-00525	CGTGGTTGTAGTCCGTGCG	1	0	1	0
tsrna-00521	CGTGGGTTCGAGCCCCACGTTGGGCGCCA	14	204	6	9
tsrna-00520	CGTGGGTTCGAGCCCCACGTTGGGCGCC	0	5	0	0
tsrna-00518	CGTGGGTTCGAGCCCCACGTTGGGCG	0	1	0	0
tsrna-00513	CGTGGGTTCGAATCCCATCCTCGTCGCCA	7	24	6	2
tsrna-00510	CGTGGGTTCGAATCCCACTTCTGACAC	0	0	1	0
tsrna-00509	CGTGGGTTCGAATCCCACTCCTGACAC	0	0	0	0
tsrna-00508	CGTGGCGCAATGGATAGCGCATTG	0	0	1	0
tsrna-00496	CGTGCTGGGCCCATAACCCAGA	0	2	0	0
tsrna-00495	CGTGCTGGGCCCATAACCCAG	0	1	1	0
tsrna-00494	CGTGCTGGGCCCATAACCC	2	1	0	0
tsrna-00493	CGTGCTGGGCCCATAACC	0	0	0	0
tsrna-00491	CGTGATCGTATAGTGGTTAGTACTCTGCGTTGTG	29	9	22	12
tsrna-00490	CGTGATCGTATAGTGGTTAGTACTCTGCGTTGT	26	10	10	8
tsrna-00489	CGTGATCGTATAGTGGTTAGTACTCTGCGTTG	27	11	11	2
tsrna-00488	CGTGATCGTATAGTGGTTAGTACTCTGCGTT	23	7	10	5

tsrna-00487	CGTGATCGTATAGTGGTTAGTACTCTGCGT	6	4	2	2
tsrna-00486	CGTGATCGTATAGTGGTTAGTACTCTGCG	8	6	4	2
tsrna-00485	CGTGATCGTATAGTGGTTAGTACTCTGC	3	2	1	1
tsrna-00484	CGTGATCGTATAGTGGTTAGTACTCTG	4	1	2	0
tsrna-00483	CGTGATCGTATAGTGGTTAGTACTCT	0	1	1	0
tsrna-00482	CGTGATCGTATAGTGGTTAGTACTC	0	0	1	0
tsrna-00481	CGTGATCGTATAGTGGTTAGTACT	1	0	0	0
tsrna-00471	CGTCTGTCACGCGGGAGACCGG	10	43	6	2
tsrna-00470	CGTCTGCTTTACACGCAG	0	1	0	0
tsrna-00466	CGTCAGTCTCATAATCTGAAG	1	4	1	0
tsrna-00464	CGTATGGAGGCGTGGGT	1	1	0	1
tsrna-00463	CGTATGAGGCCCCGGGTTCA	0	4	1	0
tsrna-00462	CGTATGAGGCCCCGGGTTC	1	2	0	0
tsrna-00461	CGTATGAGGCCCCGGGTT	1	3	1	0
tsrna-00460	CGTATGAGGCCCCGGGT	0	4	0	0
tsrna-00458	CGTATAGTGGTTAGTACTCTGCGTTGTGGCCGCAGCAACCT	63	26	22	7
tsrna-00457	CGTATAGTGGTTAGTACTCTGCGTTGTGGC	30	12	16	6
tsrna-00456	CGTATAGTGGTTAGTACTCTGCGTTGTG	25	6	8	6
tsrna-00455	CGTATAGTGGTTAGTACTCTGCGTTGT	25	12	8	7
tsrna-00454	CGTATAGTGGTTAGTACTCTGCGTTG	27	9	11	8
tsrna-00453	CGTATAGTGGTTAGTACTCTGCGTT	19	4	4	3
tsrna-00452	CGTATAGTGGTTAGTACTCTGCGT	9	2	3	2
tsrna-00451	CGTATAGTGGTTAGTACTCTGCGCTG	6	4	3	1
tsrna-00450	CGTATAGTGGTTAGTACTCTGCGCT	7	4	4	3
tsrna-00449	CGTATAGTGGTTAGTACTCTGCGC	5	1	4	0
tsrna-00448	CGTATAGTGGTTAGTACTCTGCG	4	0	5	2
tsrna-00447	CGTATAGTGGTTAGTACTCTGC	1	0	1	0
tsrna-00446	CGTATAGTGGTTAGTACTCTG	1	0	0	0
tsrna-00445	CGTATAGTGGTTAGTACTCT	0	2	0	0
tsrna-00441	CGTAGTGTAGTGGTTATCACGTTCGCCTC	0	1	0	0
tsrna-00440	CGTAGTGTAGTGGTTATCACGTTCGCCT	0	0	0	0
tsrna-00438	CGTAGTGTAGTGGTTATCACGTTCGC	1	1	0	0
tsrna-00436	CGTAGTGTAGTGGTTATCACGTT	0	0	1	0
tsrna-00434	CGTAGTGTAGTGGTTATCAC	2	0	0	0
tsrna-00433	CGTAGTGTAGTGGTTATCA	0	0	1	0
tsrna-00432	CGTAGTGTAGTGGTTATC	1	0	0	0
tsrna-00430	CGTAGTGTAGTGGTCATCACGTTCGCC	0	1	0	0
tsrna-00427	CGTAGTGTAGCGGTTATCACATTCGCCT	0	0	0	0
tsrna-00426	CGTAGTCGTGGCCGAGTGGTT	9	21	8	4
tsrna-00425	CGTAGTCGTGGCCGAGTGGT	17	22	5	5
tsrna-00424	CGTAGTCGTGGCCGAGTGG	4	18	3	5
tsrna-00423	CGTAGTCGTGGCCGAGTG	5	5	5	2
tsrna-00422	CGTAGTCGTGGCCGAGT	1	10	4	5
tsrna-00421	CGTAGTCGTGGCCGAG	4	9	0	3
tsrna-00420	CGTAAACCGAAGATCGCGGGTTCGAACCCCGTCCGTGCCTCC	13	46	4	1
tsrna-00419	CGTAAACCGAAGATCGCGGGT	8	25	2	1
tsrna-00418	CGTAAACCGAAGATCGCGGG	0	1	0	0
tsrna-00416	CGTAAACCGAAGATCACGGGTTCGAACCCCGTCCGTGCCTCC	4	23	5	2
tsrna-00414	CGGTTCGATCCCGGGCGGAAACACCA	14	44	10	3
tsrna-00413	CGGTTCGAATCCGAGTCACGGCACCA	1	1	0	0
tsrna-00411	CGGTTCGAATCCGAGTCACGGCA	0	1	0	0
tsrna-00409	CGGTTCGAAACCGGGCGGAAACACCA	289	712	144	35
tsrna-00408	CGGTTCAAATCCGGGTGCCCCCTCCA	144	156	60	23
tsrna-00407	CGGTTATCACGTCTGCTTTACACGCAGAAGGTCCTGGGT	11	34	9	1
tsrna-00406	CGGTTATCACGTCTGCTTTACACGC	2	3	0	1
tsrna-00405	CGGTTATCACGTCTGCTTTACACG	1	1	1	0
tsrna-00404	CGGTTATCACGTCTGCTTTACAC	1	2	1	1
tsrna-00403	CGGTTATCACGTCTGCTTTACA	1	0	1	1
tsrna-00402	CGGTTATCACGTCTGCTTTAC	1	0	1	1
tsrna-00396	CGGTTATCACATTCGCCTCACA	0	0	0	1
tsrna-00395	CGGTTATCACATTCGCCTCAC	0	0	1	0
tsrna-00391	CGGTTAGGATTCCTGGTTTTCACCCAGGCG	20	14	5	2
tsrna-00390	CGGTTAGGATTCCTGGTTTTCACCCAGGC	18	13	12	5
tsrna-00389	CGGTTAGGATTCCTGGTTTTCACCCAG	21	17	7	9

tsrna-00388	CGGTTAGGATTCCTGGTTTTCACCCA	12	21	7	5
tsrna-00387	CGGTTAGGATTCCTGGTTTTCACCC	9	8	5	7
tsrna-00386	CGGTTAGGATTCCTGGTTTTCACC	12	7	8	5
tsrna-00385	CGGTTAGGATTCCTGGTTTTCAC	6	6	3	1
tsrna-00384	CGGTTAGGATTCCTGGTTTTCA	5	4	2	2
tsrna-00383	CGGTTAGGATTCCTGGTTTTC	3	5	2	1
tsrna-00382	CGGTTAGGATTCCTGGTTTT	2	6	1	1
tsrna-00381	CGGTTAGGATTCCTGGTTT	2	2	0	0
tsrna-00380	CGGTTAGGATTCCTGGTT	2	1	1	0
tsrna-00379	CGGTTAGGATTCCTGGT	0	3	0	1
tsrna-00378	CGGTTAGGATTCCTGG	0	4	0	0
tsrna-00377	CGGTTAGCTCAGTTGGTTAGAGCGTGG	0	1	1	1
tsrna-00376	CGGTTAGCTCAGTTGGTTAGAGC	0	0	2	0
tsrna-00369	CGGTTAGCGCGTTCGGCTGTTAA	0	0	1	0
tsrna-00366	CGGTTAGCGCGCGGTACTTAT	1	0	0	0
tsrna-00362	CGGTCTAAGGCGCTGGATTTAG	1	2	0	0
tsrna-00360	CGGTCTAAGGCGCTGGAT	0	1	0	0
tsrna-00359	CGGTCTAAGGCGCTGGA	1	0	0	0
tsrna-00356	CGGTCTAAGGCGCTGCGTTCA	0	0	1	0
tsrna-00353	CGGTCTAAGGCGCTGCGT	1	0	0	0
tsrna-00350	CGGTAGCGTGGCCGAG	0	0	1	0
tsrna-00349	CGGTAGCGCGTCTGACTCC	2	2	1	2
tsrna-00348	CGGTAGCGCGTCTGACTC	0	2	0	0
tsrna-00347	CGGTAGCGCGTCTGACT	0	1	0	1
tsrna-00340	CGGTAGAGCGCGTGCTTA	2	15	0	0
tsrna-00339	CGGTAGAGCGCGTGCT	0	11	1	0
tsrna-00338	CGGTAGAGCATTTGACTGCAGATC	0	0	1	0
tsrna-00337	CGGTAGAGCATGGGACTCTTAATCC	3	5	3	2
tsrna-00336	CGGTAGAGCATGGGACTCTTA	2	5	1	1
tsrna-00335	CGGTAGAGCATGGGACTCTT	3	7	3	1
tsrna-00334	CGGTAGAGCATGGGACTCT	1	2	2	1
tsrna-00333	CGGTAGAGCATGGGACTC	0	5	0	0
tsrna-00332	CGGTAGAGCATGGGACT	3	5	3	0
tsrna-00331	CGGTAGAGCATGGGAC	3	2	1	1
tsrna-00330	CGGTAGAGCATGAGACTCTTA	2	4	1	2
tsrna-00329	CGGTAGAGCATGAGACTCTT	1	6	0	3
tsrna-00328	CGGTAGAGCATGAGACTC	2	2	3	1
tsrna-00327	CGGTAGAGCATGAGACT	3	6	1	0
tsrna-00326	CGGTAGAGCATGAGAC	4	6	1	2
tsrna-00325	CGGTAGAGCATCAGACTTTTAATC	1	1	0	0
tsrna-00322	CGGTAGAGCATCAGACTTTT	0	3	0	0
tsrna-00321	CGGTAGAGCATCAGACTTT	0	1	0	0
tsrna-00320	CGGTAGAGCATCAGACTT	1	1	1	0
tsrna-00317	CGGTAATCGCATAAAACTTAAAACTTTACAGTCAGA	6	5	32	0
tsrna-00316	CGGTAATCGCATAAAACTTAAAACTTTACA	3	2	18	0
tsrna-00315	CGGTAATCGCATAAAACTTAAAACTTTAC	6	4	15	0
tsrna-00314	CGGTAATCGCATAAAACTTAAAACTTT	6	7	14	0
tsrna-00313	CGGTAATCGCATAAAACTTAAAACTT	2	1	1	1
tsrna-00312	CGGTAATCGCATAAAACTTAAAACT	1	2	1	0
tsrna-00309	CGGTAATCGCATAAAACTTAAA	0	0	0	0
tsrna-00302	CGGGTTCGATTCCCGGTCATGGAACCA	0	6	0	0
tsrna-00301	CGGGTTCGATTCCCGGTCAGGGAACCA	9	283	1	4
tsrna-00300	CGGGTTCGATTCCCGGTCAGGGAACC	0	6	0	0
tsrna-00299	CGGGTTCGATTCCCGGTCAGGGAAC	0	7	0	0
tsrna-00298	CGGGTTCGATTCCCGGTCAGGGAA	0	2	0	0
tsrna-00295	CGGGTTCGATTCCCGGGCGGCGCACCA	15	54	6	1
tsrna-00294	CGGGTTCGATTCCCGGCCAGGGAACCA	1	15	0	1
tsrna-00293	CGGGTTCGATTCCCGGCCAATGCACCA	42	151	11	8
tsrna-00292	CGGGTTCGATTCCCGGCCAACGCACCA	45	107	19	13
tsrna-00291	CGGGTTCGATCCCCGTACTGGCCACCA	5	9	3	3
tsrna-00290	CGGGTTCGATCCCCGTACTGGCCACC	1	0	0	0
tsrna-00289	CGGGTTCGATCCCCGTACTGGCCAC	0	1	0	0
tsrna-00285	CGGGTTCGATCCCCGTACGGGCCACCA	8	64	3	3
tsrna-00284	CGGGTTCGATCCCCGTACGGGCCACC	1	2	0	0

tsrna-00283	CGGGTTCGATCCCCGTACGGGCCAC	1	1	0	0
tsrna-00280	CGGGTTCGATCCCCGGCATCTCCACCA	239	376	137	96
tsrna-00279	CGGGTTCGATCCCCGGCACCTCCACCA	177	287	85	38
tsrna-00278	CGGGTTCGATCCCCAGCATCTCCACCA	8	20	4	3
tsrna-00277	CGGGTTCGAGTCCCGGCGGAGTCGCCA	0	6	0	0
tsrna-00276	CGGGTTCGACTCCCGGTGTGGGAACCA	68	280	35	19
tsrna-00275	CGGGTTCGACTCCCGGTGTGGGAACC	2	10	0	0
tsrna-00274	CGGGTTCGACTCCCGGTGTGGGAAC	0	2	0	0
tsrna-00271	CGGGTTCGACTCCCGGTGTGGG	0	1	0	0
tsrna-00270	CGGGTTCGACTCCCGGTGTGG	0	1	0	0
tsrna-00268	CGGGTTCGACTCCCGGTATGGGAACCA	9	78	3	1
tsrna-00267	CGGGTTCGACTCCCGGTATGGGAAC	1	5	0	0
tsrna-00266	CGGGTTCGAACCCCGTCCGTGCCTCCA	4	8	0	0
tsrna-00263	CGGGTTCAATCCCCGGCACCTCCACCA	204	332	116	56
tsrna-00262	CGGGTTCAATCCCCGGCACCTCCA	0	1	0	0
tsrna-00260	CGGGTTCAAATCCCGGACGAGCCCCCA	126	150	49	50
tsrna-00258	CGGGTGCGAGAGGTCCCGGGTT	1	0	0	0
tsrna-00257	CGGGTGCGAGAGGTCCCGGGT	0	1	0	0
tsrna-00255	CGGGGTTCGATTCCCCGACGGGGAGCCA	1	20	0	0
tsrna-00254	CGGGGTTCGATTCCCCGACGGGGAGCC	0	3	0	0
tsrna-00253	CGGGGTTCGATTCCCCGACGGGGAGC	1	0	0	0
tsrna-00248	CGGGGTTCAATTCCCCGACGGGGAGCCA	0	2	0	0
tsrna-00247	CGGGCTCACATCACCCCATAAACACCA	4	0	1	3
tsrna-00241	CGGGCCCATACCCCGAAAATGTTGGTTAT	0	1	0	0
tsrna-00237	CGGGATCGATGCCCGCATCCTCCACCA	0	4	0	0
tsrna-00235	CGGGAGGCCCGGGTTCGATTCCCGGCCAATGCACC	6	12	0	1
tsrna-00234	CGGGAGGCCCGGGTTCGATTCCCGGCCA	0	1	1	0
tsrna-00233	CGGGAGGCCCGGGTTCGATTCCCGGCC	1	0	0	0
tsrna-00231	CGGGAGGCCCGGGTTCGAT	2	0	1	0
tsrna-00230	CGGGAGGCCCGGGTTCG	2	0	1	0
tsrna-00229	CGGGAGACCGGGGTTCGATTCCCCGACGGGGAGCCA	3	19	1	1
tsrna-00228	CGGGAGACCGGGGTTCGATTCCCCGACGGGGAGCC	0	2	0	0
tsrna-00227	CGGGAGACCGGGGTTCGATTCCCCGACGGGGAGC	0	2	0	0
tsrna-00226	CGGGAGACCGGGGTTCGATTCCCCGACGGGGAG	0	3	0	0
tsrna-00223	CGGGAGACCGGGGTTCGATTCCCCGACGGG	0	1	0	0
tsrna-00217	CGGGAGACCGGGGTTCAATTCCCCGACGGGGAGCCA	1	4	0	0
tsrna-00212	CGGCTGTTAACCGAAAGGTTGGTGGTTCGAGCCCAC	7	53	10	6
tsrna-00211	CGGCTGTTAACCGAAAGGTTGGTGGT	8	26	6	9
tsrna-00210	CGGCTGTTAACCGAAAGGTTGGTGG	1	14	1	2
tsrna-00209	CGGCTGTTAACCGAAAGGTTGGTG	3	17	0	3
tsrna-00208	CGGCTGTTAACCGAAAGGTTGGT	4	11	3	1
tsrna-00207	CGGCTGTTAACCGAAAGGTTGG	1	4	0	2
tsrna-00206	CGGCTGTTAACCGAAAGGTT	0	2	0	3
tsrna-00205	CGGCTGTTAACCGAAAGG	2	3	1	3
tsrna-00204	CGGCTGTTAACCGAAAG	2	3	1	0
tsrna-00203	CGGCTCTGTGGCGCAATGGA	0	1	0	0
tsrna-00197	CGGCTCGTTGGTCTAGGGG	0	0	0	0
tsrna-00196	CGGCTAGCTCAGTCGGTAGAGCATGGGACTCTTAAT	16	32	19	2
tsrna-00195	CGGCTAGCTCAGTCGGTAGAGCATGGGACTCTT	22	19	14	2
tsrna-00194	CGGCTAGCTCAGTCGGTAGAGCATGGGACTCT	15	23	17	4
tsrna-00193	CGGCTAGCTCAGTCGGTAGAGCATGGGACTC	16	27	13	6
tsrna-00192	CGGCTAGCTCAGTCGGTAGAGCATGGGACT	13	39	10	5
tsrna-00191	CGGCTAGCTCAGTCGGTAGAGCATGGGAC	13	14	12	4
tsrna-00190	CGGCTAGCTCAGTCGGTAGAGCATGGGA	3	6	4	2
tsrna-00189	CGGCTAGCTCAGTCGGTAGAGCATGGG	3	3	1	0
tsrna-00188	CGGCTAGCTCAGTCGGTAGAGCATGG	1	2	0	2
tsrna-00187	CGGCTAGCTCAGTCGGTAGAGCATGAGACTCTTAATC	22	32	9	5
tsrna-00186	CGGCTAGCTCAGTCGGTAGAGCATGAGACTCTTAAT	21	35	10	4
tsrna-00185	CGGCTAGCTCAGTCGGTAGAGCATGAGACTCT	20	23	18	7
tsrna-00184	CGGCTAGCTCAGTCGGTAGAGCATGAGACTC	15	32	13	15
tsrna-00183	CGGCTAGCTCAGTCGGTAGAGCATGAGACT	8	27	12	3
tsrna-00182	CGGCTAGCTCAGTCGGTAGAGCATGAGAC	6	22	8	2
tsrna-00181	CGGCTAGCTCAGTCGGTAGAGCATGAGA	6	8	2	1
tsrna-00180	CGGCTAGCTCAGTCGGTAGAGCATGAG	2	8	1	2

tsrna-00179	CGGCTAGCTCAGTCGGTAGAGCATGA	2	3	3	1
tsrna-00178	CGGCTAGCTCAGTCGGTAGAGCATG	2	3	1	0
tsrna-00177	CGGCTAGCTCAGTCGGTAGAGCAT	1	1	0	0
tsrna-00176	CGGCTAGCTCAGTCGGTAGAGCA	2	1	1	0
tsrna-00173	CGGCTAGCTCAGTCGGTAGA	0	1	0	0
tsrna-00171	CGGCTAGCTCAGTCGG	0	1	0	0
tsrna-00170	CGGCGCTCTCACCGCCGCGGCCCGGGTTCG	1	2	0	0
tsrna-00169	CGGCGCTCTCACCGCCGCGGCCCGGGTT	0	3	0	0
tsrna-00168	CGGCGCTCTCACCGCCGCGGCCCGG	2	0	0	0
tsrna-00165	CGGCGCTCTCACCGCCGCG	0	1	0	0
tsrna-00163	CGGCCCGGGTTCGATTCCCGGTCAGGGAAC	0	3	0	0
tsrna-00162	CGGCCCGGGTTCGATTCCCGGTCAGGGAA	0	4	0	0
tsrna-00156	CGGCCCGGGTTCGACTCCCGGTGTGGGAACC	4	20	3	1
tsrna-00155	CGGCCCGGGTTCGACTCCCGGTGTGGGAAC	1	2	0	0
tsrna-00154	CGGCCCGGGTTCGACTCCCGGTGTGGGAA	0	1	1	0
tsrna-00145	CGGATGGAGGCGTGGGTTCGAATCCCACT	3	11	0	0
tsrna-00144	CGGATGGAGGCGTGGGTTCG	1	4	0	0
tsrna-00143	CGGATGGAGGCGTGGGTTC	0	12	0	1
tsrna-00142	CGGATGGAGGCGTGGGTT	1	5	1	0
tsrna-00141	CGGATGGAGGCGTGGGT	2	2	0	1
tsrna-00140	CGGATGGAGGCGTGGG	0	4	0	0
tsrna-00139	CGGATCAGAAGATTGCAGGTTCGAGTCCTGCCGCGGTCGCC/	3	8	0	1
tsrna-00134	CGGATCAGAAGATTGAGGGTTCG	6	10	5	2
tsrna-00133	CGGATCAGAAGATTGAGGGTTC	4	7	3	2
tsrna-00132	CGGATCAGAAGATTGAGGGTT	6	9	1	1
tsrna-00131	CGGATCAGAAGATTGAGGGT	2	6	3	1
tsrna-00126	CGGATCAGAAGATTCTAGGTTC	3	5	0	0
tsrna-00122	CGGATCAGAAGATTCCAGGTTCGACTCCTG	2	2	1	0
tsrna-00121	CGGATCAGAAGATTCCAGGTTCG	0	1	0	0
tsrna-00120	CGGATCAGAAGATTCCAGGTT	0	0	1	0
tsrna-00119	CGGATCAGAAGATTCCAGGT	1	0	0	0
tsrna-00116	CGGATAGCTCAGTCGGTAGAGCATCAGACTTTTAATCTGA	48	32	48	23
tsrna-00115	CGGATAGCTCAGTCGGTAGAGCATCAGACTTTTA	40	34	29	28
tsrna-00114	CGGATAGCTCAGTCGGTAGAGCATCAGACTTTT	30	17	33	14
tsrna-00113	CGGATAGCTCAGTCGGTAGAGCATCAGACTTT	17	18	10	8
tsrna-00112	CGGATAGCTCAGTCGGTAGAGCATCAGACTT	18	14	10	1
tsrna-00111	CGGATAGCTCAGTCGGTAGAGCATCAGACT	13	14	8	2
tsrna-00110	CGGATAGCTCAGTCGGTAGAGCATCAGAC	17	11	6	1
tsrna-00109	CGGATAGCTCAGTCGGTAGAGCATCAGA	7	5	7	2
tsrna-00108	CGGATAGCTCAGTCGGTAGAGCATCAG	3	6	2	0
tsrna-00107	CGGATAGCTCAGTCGGTAGAGCATC	4	2	3	0
tsrna-00106	CGGATAGCTCAGTCGGTAGAGCAT	2	0	1	1
tsrna-00105	CGGATAGCTCAGTCGGTAGAGCA	1	0	2	0
tsrna-00104	CGGATAGCTCAGTCGGTAGAGC	1	0	0	0
tsrna-00102	CGGATAGCTCAGTCGGTAGA	0	0	0	1
tsrna-00101	CGGATAGCTCAGTCGGTAG	1	0	0	0
tsrna-00099	CGGATAGCTCAGTCGG	1	1	0	0
tsrna-00096	CGGAGCTGGGGATTGTGGGTTCGAGTCCCATCTGGGTCGCC/	7	14	4	1
tsrna-00095	CGGAGCTGGGGATTGTGGGTTCGA	0	2	0	0
tsrna-00094	CGGAGCTGGGGATTGTGGGTTCG	0	1	0	0
tsrna-00093	CGGAGCTGGGGATTGTGGGTTC	0	2	0	0
tsrna-00092	CGGAGCTGGGGATTGTGGGTT	0	1	0	0
tsrna-00091	CGGAGCTGGGGATTGTGGGT	0	1	0	0
tsrna-00088	CGGAGCTGGGGATTGTG	0	0	0	0
tsrna-00087	CGGAGCTGGGGATTGT	0	0	1	0
tsrna-00086	CGGAGATGAAAACCTTTTTCCAAGGACACCA	4	6	11	0
tsrna-00085	CGGAGATGAAAACCTTTTTCCAAGGACACC	0	2	1	0
tsrna-00084	CGGAGATGAAAACCTTTTTCCAAGGACAC	2	2	1	0
tsrna-00083	CGGAGATGAAAACCTTTTTCCAAGGACA	1	2	3	0
tsrna-00082	CGGAGATGAAAACCTTTTTCCAAGGAC	0	0	0	0
tsrna-00080	CGGAGATGAAAACCTTTTTCCAAGG	0	1	1	0
tsrna-00076	CGGAGATGAAAACCTTTTTCC	0	0	1	0
tsrna-00073	CGGAAGCGTGCTGGGCCCATAACCCAGAGGTCGA	9	51	6	11
tsrna-00072	CGGAAGCGTGCTGGGCCCATAACCCAGA	11	44	10	9

tsrna-00071	CGGAAGCGTGCTGGGCCCATAACCC	8	37	6	5
tsrna-00070	CGGAAGCGTGCTGGGCCCATAACC	14	44	4	6
tsrna-00069	CGGAAGCGTGCTGGGCCCATAAC	12	46	3	2
tsrna-00068	CGGAAGCGTGCTGGGCCCATAA	17	37	5	8
tsrna-00067	CGGAAGCGTGCTGGGCCCATA	10	38	0	6
tsrna-00066	CGGAAGCGTGCTGGGCCCAT	8	32	2	4
tsrna-00065	CGGAAGCGTGCTGGGCCCA	7	46	4	6
tsrna-00064	CGGAAGCGTGCTGGGCCC	7	38	4	8
tsrna-00063	CGGAAGCGTGCTGGGCC	5	16	1	2
tsrna-00062	CGGAAGCGTGCTGGGC	0	20	2	0
tsrna-00061	CGCTTTGGGTGCGAGAGGTCCCGG	0	2	0	0
tsrna-00060	CGCTTTGGGTGCGAGAGGTCCCG	0	1	0	0
tsrna-00059	CGCTTTCACCGCCGCGGCCCGGGTTC	2	7	0	3
tsrna-00058	CGCTTTCACCGCCGCGGCCCGGGTT	2	11	0	4
tsrna-00057	CGCTTTCACCGCCGCGGCCCG	2	5	0	1
tsrna-00056	CGCTTTCACCGCCGCGGCCC	1	2	0	3
tsrna-00055	CGCTTTCACCGCCGCGGCC	0	4	0	2
tsrna-00054	CGCTTTCACCGCCGCGG	0	1	0	1
tsrna-00053	CGCTTCTGTAGTGTAGTGGTTATCACGTTCGCCT	308	121	249	67
tsrna-00051	CGCTGGTTCGATTCCGGCTCGAAGGACCA	73	182	43	29
tsrna-00050	CGCTGGTTCGATTCCGGCTCGAAGGACC	2	11	1	2
tsrna-00049	CGCTGGTTCGATTCCGGCTCGAAGGAC	0	3	0	0
tsrna-00044	CGCTGGTTCGATTCCGGC	0	0	0	1
tsrna-00041	CGCTGGTTCGAATCCGGCTCGAAGGACCA	18	66	12	8
tsrna-00040	CGCTGGTTCGAATCCGGCTCGAAGGACC	3	5	3	0
tsrna-00039	CGCTGGTTCGAATCCGGCTCGAAGGAC	0	1	0	0
tsrna-00033	CGCTGGTGTAGTGGTATCATGCAAGATTCC	3	2	1	1
tsrna-00032	CGCTGGTGTAGTGGTATCATGCAAGATTC	2	3	3	1
tsrna-00031	CGCTGGTGTAGTGGTATCATGCAAGATT	1	4	2	1
tsrna-00030	CGCTGGTGTAGTGGTATCATGCA	2	0	1	0
tsrna-00027	CGCTGGTGTAGTGGTATCAT	1	0	1	0
tsrna-00026	CGCTCTCACCGCCGCGGCCCGGGTTCGATTCCCGGTCAGGG	35	305	14	9
tsrna-00025	CGCTCTCACCGCCGCGGCCCGGGTTCGATTCCCGGTCAGGG	7	2	0	1
tsrna-00024	CGCTCTCACCGCCGCGGCCCGGGTTCGATTCCCGGTCAGGG	1	4	1	1
tsrna-00023	CGCTCTCACCGCCGCGGCCCGGGTTCGATTCCCGGT	0	1	0	0
tsrna-00022	CGCTCTCACCGCCGCGGCCCGGGTTCGATT	0	2	0	0
tsrna-00020	CGCTCTCACCGCCGCGGCCCGGGTTC	0	2	0	0
tsrna-00019	CGCTCTCACCGCCGCGGCCCGGGTT	1	0	0	0
tsrna-00018	CGCTCTCACCGCCGCGGCCCGGGT	0	0	2	0
tsrna-00017	CGCTCTCACCGCCGCGGCCCGGG	0	1	0	1
tsrna-00016	CGCTCTCACCGCCGCGGCCCGG	0	1	0	0
tsrna-00015	CGCTCTCACCGCCGCGGCCCG	0	1	0	0
tsrna-00014	CGCTCTCACCGCCGCGGCCC	0	1	1	0
tsrna-00012	CGCTCTCACCGCCGCGGC	0	0	0	1
tsrna-00009	CGCGTTTGGCTGTTAACT	0	0	0	1
tsrna-00007	CGCGTTCGGCTGTTAACC	0	2	0	0
tsrna-00006	CGCGTGGGTTCGAATCCCATCCTCGTCGCCA	4	24	1	0
tsrna-00005	CGCGTGGCCTAATGGATAAGGCGTCTGA	0	0	0	0

smallRNAName	smallRNASequence	AD D14	AD D21	AD D7	CY1(AD D0
tsrna-26579	AACCGAGCGTCCAAGCTCTTTCCATTTT	21.4248649	5.699848	12.57243	42.52339
tsrna-26578	TGCGGTACCACTTTT	203.464801	70.11594	226.9323	162.3217
tsrna-26577	GTGGGTGGCTTTTTT	5.57046488	8.666892	4.749583	2.33157
tsrna-26576	TCGAGAGGGGCTGTGCTCGCAAGGTTTCTTT	1235.64338	1281.451	789.2689	1596.57
tsrna-26575	AGGTGAAAGTTCCTTT	3.14231352	34.12101	0.558774	1.776434
tsrna-26574	ACCTCAGAAGGTCTCACTTT	91.0556759	39.58662	53.50265	94.81718
tsrna-26572	GATATCCAACCTTCGGCTATAGGGTGGAGACTTTTT	58.061384	33.57445	43.93364	62.28622
tsrna-26571	AGGGAGGTTATGATTAACTTTT	32.5657947	60.66825	20.46511	29.97733
tsrna-26570	GTGGGGTGCCTCACAGCTTCGCTGCGTGAGCATTTT	3.9278919	6.558729	3.212953	2.886706
tsrna-26569	AAGAGGAGTTGTTTT	31.6373839	66.83657	17.4617	25.53624
tsrna-26568	ACAAGTGCGGTTTTTT	48.9915245	26.7034	20.88419	29.97733
tsrna-26567	GGAATGTCAGCTTTT	20.7107028	25.37604	12.15334	11.65785
tsrna-26566	ATAGGTATTAAGGTTTT	3.85647569	0.546561	1.466783	3.774923
tsrna-26565	GAGGCTTAAACTTTT	49.9199353	119.7749	33.52647	49.62913
tsrna-26564	ATGGCCGCATATATTT	2.99948109	0	1.327089	2.775678
tsrna-26563	TTCCGTGGGTTTGTTTT	235.316433	194.966	145.3512	225.3851
tsrna-26562	GAGGGTTCTCACCTTCTCTCTCCGATTT	10.9266811	44.89606	3.352647	20.42899
tsrna-26561	GCAACTGGTCGTTTT	230.103049	253.6823	129.2864	172.2031
tsrna-26560	GCGGGCGGACCTTTT	13.5690811	39.04006	6.914834	8.54909
tsrna-26559	GAGAGCGCTCGGTTTTT	18.7824649	44.66182	11.52472	4.108004
tsrna-26558	TGTGCTCCGGAGTTACCTCGTTT	164.757211	96.89742	177.9697	273.9039
tsrna-26557	GGCGATCACGTAGATTTT	114.980108	117.3544	60.1381	103.2552
tsrna-26556	GCACGAAAATGTGTTTT	0	0	0	0
tsrna-26555	GGTGTGGTCTGTTGTTT	0	0	0.069847	0
tsrna-26554	TAGGGTGTGCGTGTTTTT	4.42780542	7.33953	4.190808	3.10876
tsrna-26553	TTCAAAGGTGAACGTTT	8.92702705	19.20771	3.49234	25.20316
tsrna-26552	GTGTAAGCAGGGTCGTTTT	18.6396325	13.7421	18.09032	44.1888
tsrna-26551	ATGTGGTGGCTTACTTT	517.053407	183.6444	504.3638	471.6433
tsrna-26550	GTGTGTAGCTGCACTTTT	5.35621623	5.075207	3.701881	5.329303
tsrna-26548	GAAGCGGGTGCTCTTATTTT	16844.8001	3796.958	14515.98	9398.891
tsrna-26547	TCGCTGGTTCGAATCCGGCTCGGAGGACCA	0	3.982086	0.069847	0
tsrna-26544	GTAGTCGTGGCCGA	0.07141622	0.07808	0	0.111027
tsrna-26543	GGTTCCATGGTGTA	0	0.07808	0	0.111027
tsrna-26542	GGTTCCATAGTGTA	0	0.15616	0.069847	0
tsrna-26540	GGGGGTATAGCTCAG	0.07141622	0	0	0
tsrna-26539	GGGGGTATAGCTC	0	0.15616	0	0.111027
tsrna-26534	GCCCGGCTAGCTCAG	0	0	0	0
tsrna-26532	CCTTCGATAGCTCAG	0	0.07808	0	0
tsrna-26531	GAATTCTCGCCTGCCACGCGGGAGGCCCGG	0.07141622	0.546561	0	0
tsrna-26530	GAATTCTCGCCTGCCACGCGGGAGGCCC	0.14283243	0.702721	0.20954	0.111027
tsrna-26529	GAATTCTCGCCTGCCACGCGGGAGGCC	0.07141622	0.702721	0.069847	0.111027
tsrna-26528	GAATTCTCGCCTGCCACGCGGG	0.07141622	0.624641	0.069847	0
tsrna-26527	GAATTCTCGCCTGCCACGCGG	0.14283243	0.624641	0	0.111027
tsrna-26526	GAATTCTCGCCTGCCACGCG	0.21424865	0.390401	0	0
tsrna-26525	GAATTCTCGCCTGCCACGC	0.28566487	0.936961	0.069847	0
tsrna-26524	GAATTCTCGCCTGCCACG	0	0.546561	0	0
tsrna-26523	GAATTCTCGCCTGCCAC	0	0	0	0
tsrna-26522	GAATTCTCGCCTGCCA	0	0.07808	0	0
tsrna-26517	GAATGATTTCGACTCATTAAATTATGATA	0.57132973	0.31232	0.628621	0
tsrna-26516	GAATGATTTCGACTCATTAAA	0	0.07808	0	0
tsrna-26515	GAATGATTTCGACTCA	0.07141622	0.07808	0	0
tsrna-26514	GAATCTTAGCTTTGGGTGCTAATGGTGGAGTTAAAG	0.14283243	0.07808	0.069847	0.111027
tsrna-26513	GAATCTTAGCTTTGGGTGCTAATGGTGGAG	0.07141622	0.07808	0	0
tsrna-26508	GAATCTTAGCTTTGGGTGC	0	0	0	0.111027
tsrna-26497	GAATCTCGGTGGGACCTCCA	3.64222704	6.714889	1.74617	1.332326
tsrna-26496	GAATCCTGTTCGTGACGCCA	0.4284973	0	0.20954	0.111027
tsrna-26494	GAATCCTGCTCACAGCGCCA	0.35708108	1.327362	0.139694	0.111027
tsrna-26493	GAATCCTGCTCACAGCGCC	0.14283243	0.31232	0	0
tsrna-26491	GAATCCTGCCGACTACGCCA	7.85578381	17.80227	2.654179	1.887461
tsrna-26490	GAATCCTGCCGACTACGCC	0.21424865	1.093122	0.139694	0
tsrna-26489	GAATCCTGCCGACTACGC	0.07141622	0.07808	0	0
tsrna-26488	GAATCCTGCCGACTACG	0.07141622	0	0	0
tsrna-26487	GAATCCGGCTCGGAGGACCA	0.07141622	0.15616	0	0

tsrna-26483	GAATCCGGCTCGAAGGACCA	1.07124325	3.513605	0.698468	0.222054
tsrna-26482	GAATCCGGCTCGAAGGACC	0	0.23424	0.139694	0
tsrna-26473	GAATCCCATCCTCGTCGCCA	0.28566487	0.07808	0.20954	0
tsrna-26472	GAATCCCATCCTCGTCGCC	0	0.07808	0	0.111027
tsrna-26471	GAATCCCATCCTCGTCGC	0	0	0	0
tsrna-26469	GAATCCCAGTAGAGCCTCCA	0.07141622	0.546561	0.279387	0.999244
tsrna-26468	GAATCCCAGCGGTGCCTCCA	0	0.15616	0.069847	0
tsrna-26464	GAATCCCAGCGAGGCCTCCA	0.07141622	0	0.069847	0.111027
tsrna-26463	GAATCCCACTTCTGACACCA	0.35708108	0.390401	0.20954	0.111027
tsrna-26462	GAATCCCACTTCTGACACC	0	0.31232	0	0
tsrna-26461	GAATCCCACTTCTGACAC	0.14283243	0	0	0
tsrna-26460	GAATCCCACTTCTGACA	0	0	0	0
tsrna-26459	GAATCCCACTCCTGACACCA	5.14196758	4.840967	3.352647	2.997733
tsrna-26458	GAATCCCACTCCTGACACC	0.07141622	0	0.069847	0
tsrna-26457	GAATCCCACTCCTGACAC	0.07141622	0	0	0
tsrna-26455	GAATCCCACCTTCGTCGCCA	0.21424865	0.15616	0	0
tsrna-26454	GAATCCCACCGCTGCCACCA	0.07141622	0	0	0
tsrna-26453	GAATCCCACCGCTGCCACC	0	0	0.069847	0
tsrna-26450	GAATCCCACCCTCGTCGCCA	0.49991351	1.171202	0.069847	0.111027
tsrna-26448	GAATCCCACCAGAGTCGCCA	0.07141622	0.546561	0	0.111027
tsrna-26438	GAATCACGTCGGGGTCACCA	0.35708108	0.468481	0.419081	0.555136
tsrna-26437	GAATCACGTCGGGGTCACC	0	0	0	0
tsrna-26433	GAATAGTTTAAATTAGAATCTTAGCTTTGG	0.71416216	0.390401	0.558774	0.333081
tsrna-26431	GAAGGTTGCGTGTTCAAGTCACGTCGGGGT	0	0.07808	0	0
tsrna-26430	GAAGGTTGCGTGTTCAAGTCACGTC	0	0.07808	0.069847	0
tsrna-26429	GAAGGTTGCGTGTTCAAGTCACGT	0	0.07808	0	0
tsrna-26428	GAAGGTTGCGTGTTCAAATCACGTCGGGGT	0	0.07808	0.069847	0
tsrna-26427	GAAGGTTGCGTGTTCAAATCACGTC	0.07141622	0.07808	0	0.111027
tsrna-26426	GAAGGTCGTGAGTTCGATCC	0	0	0.069847	0
tsrna-26423	GAAGGTCCTGGGTTCGAGCCCCAGTGGAACCACCA	2.49956757	2.420483	2.095404	1.55438
tsrna-26422	GAAGGTCCTGGGTTCGAGCCCCAGTGGAAC	0.14283243	0	0.069847	0
tsrna-26421	GAAGGTCCTGGGTTCGAGCCCCAGTGGAA	0	0.15616	0.069847	0
tsrna-26419	GAAGGTCCTGGGTTCGAGCCCCA	0.07141622	0	0	0
tsrna-26418	GAAGGTCCTGGGTTCGAGCCCC	0.07141622	0.07808	0	0
tsrna-26415	GAAGGTCCTGAGTTCGAACC	0	0	0	0
tsrna-26414	GAAGGTCCTGAGTTCGAA	0	0	0	0
tsrna-26413	GAAGGTCCTGAGTTCG	0	0	0	0
tsrna-26412	GAAGGCTGCGTGTTCGAATCACGTCGGGGT	0	0	0	0
tsrna-26410	GAAGCGTGCTGGGCCCATAACC	0	0	0	0
tsrna-26408	GAAGCCAGTTGATTAGGGTGCTTAGCTGTT	0.21424865	0	0.20954	0.222054
tsrna-26407	GAAGCCAGTTGATTAGGGTGCTTAGCTGT	0.07141622	0	0.20954	0.111027
tsrna-26402	GAAGCATTGGACTGTAAATCTAAAGACAGG	0.14283243	0.15616	0	0
tsrna-26401	GAAGCATTGGACTGTAAATCTAAAGACA	0.07141622	0	0.069847	0
tsrna-26400	GAAGCATTGGACTGTAAATCTAAAGA	0	0	0.069847	0
tsrna-26398	GAAGCATTGGACTGTAAATCTAAA	0	0	0	0
tsrna-26392	GAAGCATTGGACTGTAAA	0	0	0.069847	0
tsrna-26391	GAAGCATTGGACTGTAA	0	0	0	0
tsrna-26390	GAAGCATTGGACTGTA	0	0	0	0
tsrna-26389	GAAGCAGCTTCAAACCTGCCGGGGCTTCC	0.07141622	0.15616	0	0
tsrna-26388	GAAGCAGCTTCAAACCTGCCGGGGCTT	0	0	0	0
tsrna-26387	GAAGCAGCTTCAAACCTGCCGGGGCT	0	0	0	0
tsrna-26382	GAAGATTGCAGGTTCGAGTCCTGCCGCGGTCGCCA	0.14283243	0.390401	0.20954	0
tsrna-26380	GAAGATTGAGGGTTCGAGTCCCTTCGTGGTCGCCA	0.35708108	1.327362	0.139694	0.111027
tsrna-26379	GAAGATTGAGGGTTCGAGTCCCTTCGTGGTCGCC	0.14283243	0.31232	0.20954	0.111027
tsrna-26378	GAAGATTGAGGGTTCGAGTCCCTTCGTGGT	0.07141622	0.23424	0.069847	0.222054
tsrna-26377	GAAGATTCTAGGTTCGACTCCTGGCTGGCT	0	0.702721	0	0.111027
tsrna-26376	GAAGATTCTAGGTTCGACTCCTG	0	0.546561	0	0
tsrna-26375	GAAGATTCCAGGTTCGACTCCTGGC	0	0	0.069847	0.111027
tsrna-26374	GAAGATTCCAGGTTCGACTCC	0	0.07808	0	0
tsrna-26372	GAAGATCGCGGGTTCGAACCCCGTC	0	0	0	0
tsrna-26370	GAAGATCGCGGGTTCGAA	0	0.07808	0	0
tsrna-26369	GAAGATCGCGGGTTCGA	0	0.07808	0	0
tsrna-26368	GAAGATCGCGGGTTCG	0	0.07808	0	0
tsrna-26367	GAAGAAGCAGCTTCAAACCTGCCGGGGCTTCC	0	0.31232	0.139694	0

tsrna-26366	GAAGAAGCAGCTTCAAACCTGCCGGGGCTT	0	0.15616	0.069847	0
tsrna-26365	GAAGAAGCAGCTTCAAACCTGCCGGGGCT	0	0	0	0
tsrna-26364	GAAGAAGCAGCTTCAAACCTGCCGGGGC	0	0.07808	0	0
tsrna-26363	GAAGAAGCAGCTTCAAACCTGCCGGGG	0	0.15616	0	0
tsrna-26358	GAAGAAGCAGCTTCAAACCTG	0.07141622	0	0	0
tsrna-26355	GAACTGCTAACTCATGCCCCCATGTCTAACAACATGGCTTTCT	0.57132973	0.23424	1.117549	0
tsrna-26354	GAACTGCTAACTCATGCCCCCATGTCTAACAACATGGCTTTCT	0.28566487	0.15616	0.279387	0
tsrna-26353	GAACTGCTAACTCATGCCCCCATGTCTAACAACATGGCT	0	0	0	0
tsrna-26352	GAACTGCTAACTCATGCCCCCATGTCTAACAACATGGC	0.07141622	0	0.139694	0
tsrna-26351	GAACTGCTAACTCATGCCCCCATGTCTAACAACA	0	0	0	0
tsrna-26350	GAACTGCTAACTCATGCCCCCATGTCTAACA	0	0	0	0
tsrna-26349	GAACTGCTAACTCATGCCCCCATGTCTAAC	0	0	0.069847	0
tsrna-26348	GAACTGCTAACTCATGCCCCCATGTCTAA	0	0	0	0
tsrna-26347	GAACTGCTAACTCATGCCCCCATGTCT	0.07141622	0.07808	0	0
tsrna-26346	GAACTGCTAACTCATGCCCCCATGTC	0	0	0	0
tsrna-26345	GAACTGCTAACTCATGCCCCCATGT	0	0	0	0
tsrna-26344	GAACTGCTAACTCATGCCCCCATG	0.14283243	0	0	0
tsrna-26343	GAACTGCTAACTCATGCCCCCAT	0	0.07808	0	0
tsrna-26342	GAACTGCTAACTCATGCCCCCA	0	0	0.069847	0
tsrna-26335	GAACCTCAGAGGGGGCACCA	0	0.23424	0	0
tsrna-26333	GAACCCTGCTCGCTGCGCCA	0.57132973	2.967044	0.069847	0.333081
tsrna-26332	GAACCCTGCTCGCTGCGC	0	0.07808	0	0
tsrna-26330	GAACCCCGTCCGTGCCTCCA	0.4284973	0.624641	0.20954	0.333081
tsrna-26328	GAACCCCGTACGGGCCACCA	0	0.15616	0	0.111027
tsrna-26315	GAAATTTAGGTTAAATACAGACCAAGAGCC	0	0.07808	0.069847	0
tsrna-26313	GAAATCCAATGGGGTTTCCCC	0	0.07808	0	0
tsrna-26312	GAAATATGTCTGATAAAAGAGTTACTTTG	0	0.15616	0	0
tsrna-26311	GAAATATGTCTGATAAAAGAGTTACTTT	0	0.07808	0	0.111027
tsrna-26306	GAAATATGTCTGATAAAA	0.07141622	0	0	0
tsrna-26304	GAAATATGTCTGATAA	0	0.07808	0	0
tsrna-26299	GAAATACAACGATGGTTTTTCATATCATTGGTCGTGGTTGTAG	0.07141622	0.390401	0.279387	0.111027
tsrna-26298	GAAATACAACGATGGTTTTTCATATCATTGGTCGTGGTTGTAG	0.07141622	0.07808	0.349234	0.111027
tsrna-26297	GAAATACAACGATGGTTTTTCATATCATTGGTCGTGGTTGTAG	0.07141622	0.07808	0.349234	0.111027
tsrna-26296	GAAATACAACGATGGTTTTTCATATCATTGGTCGTGGTTGTAG	0.07141622	0	0.139694	0.111027
tsrna-26295	GAAATACAACGATGGTTTTTCATATCATTGGTCGTGGTTGTA	0.35708108	0.31232	0.069847	0.111027
tsrna-26294	GAAATACAACGATGGTTTTTCATATCATTG	0.07141622	0	0	0
tsrna-26293	GAAATACAACGATGGTTTTTCATATCA	0	0	0	0.111027
tsrna-26289	GAAATACAACGATGGTTTTT	0	0	0.069847	0
tsrna-26285	GAAAGGTTGGTGGTTCG	0	0	0	0
tsrna-26283	GAAAGGTCCCCGGTTCGAAACCGGGCGGA	0	0	0	0
tsrna-26282	GAAAGGTCCCCGGTTCGAAACCGGGC	0	0.07808	0	0
tsrna-26281	GAAAGGTCCCCGGTTCGAAAC	0	0.23424	0	0
tsrna-26280	GAAAGGTCCCCGGTTCG	0	0.15616	0	0
tsrna-26279	GAAAGGTCCCCGGTTC	0	0.07808	0	0
tsrna-26278	GAAAGCTCACAAGAACTGCTAACTCATGCCCCCATG	0.4284973	0.858881	0	0.111027
tsrna-26277	GAAAGCTCACAAGAACTGCTAACTCATGCC	0.35708108	0.390401	0.139694	0.111027
tsrna-26276	GAAAGCTCACAAGAACTGCTAACTCATGC	0.4284973	0.546561	0.20954	0.999244
tsrna-26275	GAAAGCTCACAAGAACTGCTAACTCATG	0.4284973	0.31232	0.20954	0.222054
tsrna-26274	GAAAGCTCACAAGAACTGCTAACTCA	0.35708108	0.546561	0.488928	0.555136
tsrna-26273	GAAAGCTCACAAGAACTGCTAACTC	0.28566487	0.702721	0.139694	0.111027
tsrna-26272	GAAAGCTCACAAGAACTGCTAACT	0.28566487	0.468481	0	0.444109
tsrna-26271	GAAAGCTCACAAGAACTGCTAAC	0.07141622	0.468481	0.139694	0
tsrna-26270	GAAAGCTCACAAGAACTGCTAA	0.07141622	0.23424	0	0
tsrna-26269	GAAAGCTCACAAGAACTGCTA	0	0.07808	0	0
tsrna-26268	GAAAGCTCACAAGAACTGCT	0	0.15616	0	0
tsrna-26266	GAAAGCTCACAAGAACTG	0	0	0	0.111027
tsrna-26265	GAAAGCTCACAAGAACT	0	0	0.069847	0
tsrna-26263	GAAACCGGGCGGAAACACCA	19.4252109	54.65608	7.264068	3.441841
tsrna-26262	GAAACCGGGCGGAAACACC	4.99913515	15.22562	1.466783	0.333081
tsrna-26261	GAAACCGGGCAGAAGCACCA	2.14248649	10.15041	0.977855	0.666163
tsrna-26260	GAAACCATCCTCTGCTACCA	0	0.23424	0.139694	0.111027
tsrna-26258	GAAACCAGCTTTGGGGGGTTCG	0.14283243	0.07808	0.069847	0
tsrna-26257	GAAACCAGCTTTGGGGGGTTC	0	0.07808	0	0
tsrna-26256	GAAAATGTTTAGACGGGCTCACATCACCCCATAAACACCA	11.8550919	3.435525	6.635447	2.664651

tsrna-26255	GAAAATGTTTAGACGGGCTCACATCACCCCATAAACACC	6.35604326	5.465608	3.841574	1.55438
tsrna-26254	GAAAATGTTTAGACGGGCTCACATCACCCCATAAA	6.78454056	3.669765	2.304945	1.665407
tsrna-26253	GAAAATGTTTAGACGGGCTCACATCACCCCATAA	5.71329731	3.825925	3.073259	1.443353
tsrna-26252	GAAAATGTTTAGACGGGCTCACATCACCCCATA	7.42728651	3.279365	2.095404	1.332326
tsrna-26251	GAAAATGTTTAGACGGGCTCACATCACCCCA	5.2133838	2.732804	2.025557	0.888217
tsrna-26250	GAAAATGTTTAGACGGGCTCACATCACCCC	5.85612975	3.279365	2.235098	0.888217
tsrna-26249	GAAAATGTTTAGACGGGCTCACATCACCC	4.92771893	3.279365	1.955711	0.77719
tsrna-26248	GAAAATGTTTAGACGGGCTCACATCACC	4.71347028	3.825925	1.606477	0.555136
tsrna-26247	GAAAATGTTTAGACGGGCTCACATCA	3.9278919	2.888964	1.466783	0.333081
tsrna-26246	GAAAATGTTTAGACGGGCTCACATC	2.85664866	2.498564	1.047702	0.333081
tsrna-26245	GAAAATGTTTAGACGGGCTCACAT	3.21372974	2.030083	1.676323	0.555136
tsrna-26244	GAAAATGTTTAGACGGGCTCACA	1.92823784	1.717762	0.977855	0.222054
tsrna-26243	GAAAATGTTTAGACGGGCTCAC	1.92823784	1.717762	0.908008	0.333081
tsrna-26242	GAAAATGTTTAGACGGGCTCA	0.99982703	1.093122	0.139694	0.222054
tsrna-26241	GAAAATGTTTAGACGGGCTC	0.28566487	1.171202	0.069847	0.222054
tsrna-26240	GAAAATGTTTAGACGGGCT	0	0.07808	0	0
tsrna-26239	GAAAATGTTTAGACGGGC	0.07141622	0	0	0
tsrna-26236	GAAAATGTTGGTTATACCCTTCCCGTACTACCA	0.78557838	1.561602	0.768315	0.333081
tsrna-26235	GAAAATGTTGGTTATACCCTTCCCGTACTA	0.07141622	0	0.069847	0
tsrna-26234	GAAAATGTTGGTTATACCCTTCCCGTACT	0	0	0	0
tsrna-26233	GAAAATGTTGGTTATACCCTTCCCGTA	0	0	0	0
tsrna-26226	GAAAATGTTGGTTATACCC	0	0	0	0
tsrna-26223	GAAAACCTTTTTCCAAGGACACCA	0.07141622	0.15616	0.069847	0
tsrna-26222	GAAAACCTTTTTCCAAGGACACC	0	0	0	0
tsrna-26221	GAAAACCTTTTTCCAAGGACA	0	0	0	0
tsrna-26217	GAAAAAGTCATGGAGGCCATGGGGTTGGCTTGAAAC	6.49887569	27.40612	5.587744	3.552868
tsrna-26216	GAAAAAGTCATGGAGGCCATGGGGTTGGCT	7.21303786	28.96772	4.051115	2.775678
tsrna-26215	GAAAAAGTCATGGAGGCCATGGGGTTGGC	6.42745948	28.81156	4.81943	3.219787
tsrna-26214	GAAAAAGTCATGGAGGCCATGGGGTTGG	6.85595678	28.88964	4.889276	4.441086
tsrna-26213	GAAAAAGTCATGGAGGCCATGGGGTTG	4.57063785	27.71844	4.260655	3.552868
tsrna-26212	GAAAAAGTCATGGAGGCCATGGGGTT	4.64205407	24.51715	5.448051	2.553624
tsrna-26211	GAAAAAGTCATGGAGGCCATGGGGT	2.71381622	8.823052	0.349234	0.888217
tsrna-26210	GAAAAAGTCATGGAGGCCATGGGG	1.57115676	7.02721	1.327089	1.332326
tsrna-26209	GAAAAAGTCATGGAGGCCATGGG	1.07124325	5.777928	1.187396	0.999244
tsrna-26208	GAAAAAGTCATGGAGGCCATGG	1.64257298	6.558729	0.419081	1.443353
tsrna-26207	GAAAAAGTCATGGAGGCCATG	1.49974054	6.94913	0.488928	0.555136
tsrna-26206	GAAAAAGTCATGGAGGCCAT	1.49974054	5.777928	1.187396	0.888217
tsrna-26205	GAAAAAGTCATGGAGGCCA	1.57115676	5.543688	0.908008	0.555136
tsrna-26204	GAAAAAGTCATGGAGGCC	1.42832433	4.997127	0.838162	1.221299
tsrna-26203	GAAAAAGTCATGGAGGC	0.64274595	2.030083	0.698468	0.555136
tsrna-26202	GAAAAAGTCATGGAGG	0.64274595	1.015041	0.349234	0.333081
tsrna-26201	GAAAAACCATTTCATAACTTTGTCAAAGTT	0.71416216	0.31232	0.419081	0.222054
tsrna-26198	CTTTTTCCAAGGACACCA	0.4284973	0.31232	0.279387	0
tsrna-26197	CTTTTTCCAAGGACACC	0	0	0	0
tsrna-26195	CTTTTCATCTGAGGGTCCAG	0	0.07808	0	0
tsrna-26193	CTTTTAATCTGAGGGTCCGG	0.64274595	2.420483	0.20954	0
tsrna-26192	CTTTTAATCTGAGGGTCCAGGGTTCAAGTCCCTGTTCGGGCG	2.42815136	5.934088	1.047702	0.666163
tsrna-26191	CTTTTAATCTGAGGGTCCAGGGT	0.57132973	3.513605	0.768315	0.333081
tsrna-26190	CTTTTAATCTGAGGGTCCAGGG	0.35708108	1.483522	0.488928	0.111027
tsrna-26189	CTTTTAATCTGAGGGTCCAGG	0.64274595	1.873923	0.419081	0.222054
tsrna-26188	CTTTTAATCTGAGGGTCCAG	0.4284973	3.123204	0.419081	0.444109
tsrna-26187	CTTTTAATCTGAGGGTCCA	0.35708108	1.795843	0.419081	0.444109
tsrna-26186	CTTTTAATCTGAGGGTCC	0.4284973	1.717762	0.139694	0.111027
tsrna-26185	CTTTTAAGTTAAAGATTAAGAGAACCAACACCTCTTTACAGTG	0.71416216	0	1.396936	0.333081
tsrna-26184	CTTTTAAGTTAAAGATTAAGAGAACCAACACCTCTTTACAGTG	0.57132973	0.15616	0.20954	0
tsrna-26183	CTTTTAAGTTAAAGATTAAGAGAACCAACACCTCTTTACAGTG	0.64274595	0	0.139694	0
tsrna-26182	CTTTTAAGTTAAAGATTAAGAGAACCAACACC	0	0.07808	0.139694	0
tsrna-26181	CTTTTAAGTTAAAGATTAAGAGAACCAACAC	0.07141622	0.07808	0	0
tsrna-26180	CTTTTAAGTTAAAGATTAAGAGAACCAAC	0	0	0	0
tsrna-26179	CTTTTAAGTTAAAGATTAAGAGAACCA	0	0	0	0
tsrna-26178	CTTTTAAGTTAAAGATTAAGAGAACC	0	0	0	0
tsrna-26177	CTTTTAAGTTAAAGATTAAGAGAAC	0	0	0.069847	0
tsrna-26174	CTTTTAAGTTAAAGATTAAGA	0	0	0	0
tsrna-26172	CTTTTAAAGGATAACAGCTATCCATTGGTC	0	0	0	0

tsrna-26169	CTTTTAAAGGATAACAGCTAT	0	0	0	0.111027
tsrna-26165	CTTTGTCAAAGTTAAATTATAGGCTAA	0.35708108	0.15616	0.069847	0
tsrna-26164	CTTTGTCAAAGTTAAATTATAGGCT	0.07141622	0	0.139694	0
tsrna-26163	CTTTGTCAAAGTTAAATTATAGGC	0.07141622	0	0.069847	0.111027
tsrna-26162	CTTTGTCAAAGTTAAATTATAGG	0	0	0.139694	0
tsrna-26159	CTTTGGGTGCTAATGGTGGAGTTAAAGACTTTTTCTCTGACC/	1.71398919	4.762887	1.53663	1.776434
tsrna-26158	CTTTGGGTGCTAATGGTGGAGTTAAAGACTTTTTCTCTGACC	0.64274595	2.030083	0.628621	0.555136
tsrna-26157	CTTTGGGTGCTAATGGTGGAGTTAAAGACTTTTTCT	0.35708108	0.936961	0.279387	0.111027
tsrna-26156	CTTTGGGTGCTAATGGTGGAGTTAAAGACT	0	0.624641	0.20954	0
tsrna-26155	CTTTGGGTGCTAATGGTGGAGTTAAAGAC	0	0.23424	0.069847	0.111027
tsrna-26154	CTTTGGGTGCTAATGGTGGAGTTAAAGA	0.07141622	0.23424	0.069847	0.111027
tsrna-26153	CTTTGGGTGCTAATGGTGGAGTTAAAG	0.35708108	0.07808	0.069847	0
tsrna-26152	CTTTGGGTGCTAATGGTGGAGTTAAA	0	0.390401	0.069847	0
tsrna-26151	CTTTGGGTGCTAATGGTGGAGTTAA	0	0.23424	0.069847	0
tsrna-26150	CTTTGGGTGCTAATGGTGGAGTTA	0	0	0.139694	0
tsrna-26149	CTTTGGGTGCTAATGGTGGAGTT	0	0.07808	0	0
tsrna-26141	CTTTGGGTGCGAGAGGTCCCGGGT	0.57132973	3.982086	0.20954	0.999244
tsrna-26140	CTTTGGGTGCGAGAGGTCCCGG	0	0.15616	0	0
tsrna-26139	CTTTGGGTGCGAGAGGTCCCG	0.07141622	0.15616	0	0
tsrna-26138	CTTTGGGGGGTTCGATTCCTTCCTTTTTTG	0	0.15616	0	0
tsrna-26137	CTTTGGGGGGTTCGATTCCTTCC	0.07141622	0	0	0
tsrna-26134	CTTTGCACGTATGAGGCCCCGGGT	0.92841081	1.717762	0.20954	1.332326
tsrna-26133	CTTTGCACGTATGAGGCCCCGG	0.07141622	0.07808	0	0
tsrna-26132	CTTTGCACGTATGAGGCCCCG	0	0.23424	0	0
tsrna-26131	CTTTGCACGTATGAGGCCCC	0	0	0.069847	0
tsrna-26130	CTTTGCACGTATGAGGCCC	0	0	0	0
tsrna-26129	CTTTGCACGCGTGGGTTCGAA	0	0.23424	0.069847	0
tsrna-26128	CTTTGCACGCGTGGGTTCG	0	0.468481	0.139694	0
tsrna-26127	CTTTGCACGCGTGGGTTC	0	0.31232	0	0
tsrna-26126	CTTTGCACGCGTGGGTT	0.07141622	0.07808	0	0
tsrna-26125	CTTTGCACGCGTGGGT	0.14283243	0.390401	0.069847	0
tsrna-26124	CTTTGATAGAGTAAATAATAGGAGCTTAAA	0	0	0	0.111027
tsrna-26123	CTTTGATAGAGTAAATAATAGGAGCTTAA	0	0	0	0
tsrna-26122	CTTTGATAGAGTAAATAATAGGAGCTT	0	0	0.069847	0.111027
tsrna-26121	CTTTGATAGAGTAAATAATAGGAGCT	0	0	0	0
tsrna-26120	CTTTGATAGAGTAAATAATAGGAGC	0	0	0	0
tsrna-26110	CTTTGAATCCAGCGATCCGAGTTCA	0.07141622	0.15616	0	0.111027
tsrna-26109	CTTTGAATCCAGCGATCCGAGTTC	0.07141622	0.15616	0.20954	0.111027
tsrna-26108	CTTTGAATCCAGCGATCCGAGTT	0.14283243	0	0.069847	0
tsrna-26107	CTTTGAATCCAGCGATCCGAGT	0.07141622	0.07808	0.069847	0.111027
tsrna-26106	CTTTGAATCCAGCGATCCGAG	0.21424865	0	0.069847	0
tsrna-26105	CTTTGAATCCAGCGATCCGA	0.07141622	0.23424	0	0.111027
tsrna-26104	CTTTGAATCCAGCGATCCG	0.21424865	0.07808	0.139694	0
tsrna-26103	CTTTGAATCCAGCGATCC	0.28566487	0.07808	0.069847	0
tsrna-26102	CTTTGAATCCAGCGATC	0	0	0.139694	0.111027
tsrna-26101	CTTTGAATCCAGCGAT	0.07141622	0.23424	0	0
tsrna-26100	CTTTGAATCCAGCAATCCGAGTT	0.35708108	1.327362	0.349234	1.110271
tsrna-26099	CTTTGAATCCAGCAATCCGAGT	0.14283243	1.717762	0.20954	0.333081
tsrna-26098	CTTTGAATCCAGCAATCCGAG	0.21424865	0.15616	0.069847	0.111027
tsrna-26097	CTTTGAATCCAGCAATCCGA	0.07141622	0.15616	0.139694	0
tsrna-26096	CTTTCGATAGCTCAGTTGGTAGAGC	0.07141622	0	0.069847	0
tsrna-26095	CTTTCACCGCCGCGGCCCGGGTTC	0	0.31232	0.069847	0.888217
tsrna-26094	CTTTCACCGCCGCGGCCCGGGT	0.07141622	0.31232	0.069847	0.666163
tsrna-26093	CTTTCACCGCCGCGGCCCG	0.14283243	0.23424	0.069847	0.555136
tsrna-26092	CTTTCACCGCCGCGGCCC	0	0.07808	0.069847	0.333081
tsrna-26091	CTTTCACCGCCGCGGCC	0.14283243	0.23424	0	0
tsrna-26090	CTTTCACCGCCGCGGC	0.14283243	0	0.20954	0.111027
tsrna-26089	CTTTACAGTCAGAGGTTCA	0	0	0	0
tsrna-26087	CTTTACAGTCAGAGGTT	0.07141622	0	0	0
tsrna-26085	CTTTACACGCAGAAGGTCCTGG	0.07141622	0	0	0
tsrna-26084	CTTTACACGCAGAAGGTCCTG	0	0.07808	0	0
tsrna-26083	CTTGTAGTTGAAATACAACGATGGTTTTTC	0.07141622	0	0.139694	0
tsrna-26082	CTTGTAGTTGAAATACAACGATGGTTTTT	0.07141622	0.07808	0.069847	0
tsrna-26078	CTTGTAGTATAAACTAATACACCAG	0	0	0.069847	0

tsrna-26077	CTTGTAAACCGGAGATGAAAACCTTTTTCC	0.14283243	0.546561	0.279387	0
tsrna-26074	CTTGTAAACCAGGGGTCGCGAGT	2.28531893	4.606726	1.257243	0.555136
tsrna-26073	CTTGTAAACCAGGGGTCGCGA	0.07141622	0.23424	0.139694	0.222054
tsrna-26072	CTTGTAAACCAGGGGTCGCG	0	0.31232	0.069847	0
tsrna-26071	CTTGTAAACAGGAGATCCTGGGT	0.71416216	6.402569	0.698468	0.77719
tsrna-26068	CTTGACCGCTCTGACCA	0	0.07808	0	0
tsrna-26066	CTTGAAACCAGCTTTGGGGGGTTC	0	0	0.069847	0
tsrna-26065	CTTCTGTAGTGTAGTGGTTATCACGTTCGCCT	0.21424865	0.23424	0.768315	0.222054
tsrna-26064	CTTCTGTAGTGTAGTGGTTATCACGTTCGCC	0.21424865	0	0.069847	0.111027
tsrna-26063	CTTCTGTAGTGTAGTGGTTATCACGTTCGC	0.14283243	0	0	0.111027
tsrna-26062	CTTCTGTAGTGTAGTGGTTATCACGTTCG	0.14283243	0	0.069847	0
tsrna-26061	CTTCTGTAGTGTAGTGGTTATCACGTTC	0.07141622	0	0	0.111027
tsrna-26060	CTTCTGTAGTGTAGTGGTTATCACGTT	0.07141622	0	0	0.111027
tsrna-26059	CTTCTGTAGTGTAGTGGTTATCACGT	0	0	0	0
tsrna-26058	CTTCTGTAGTGTAGTGGTTATCACG	0	0.07808	0	0.111027
tsrna-26057	CTTCTGTAGTGTAGTGGTTATCAC	0.07141622	0	0	0.111027
tsrna-26056	CTTCTGTAGTGTAGTGGTTATCA	0.07141622	0	0	0
tsrna-26055	CTTCTGTAGTGTAGTGGTTATC	0	0	0.139694	0
tsrna-26049	CTTCTAATTCAAAGGTTCCGGGTT	0	0.15616	0.139694	0.111027
tsrna-26048	CTTCTAATTCAAAGGTTCCGGGT	0	0	0.069847	0
tsrna-26047	CTTCTAATTCAAAGGTTCCGGG	0	0.07808	0	0
tsrna-26045	CTTCGGGGGCGTGGGTTCG	0	0.07808	0	0
tsrna-26042	CTTCGGATCAGAAGATTGCAGGTT	0	0	0	0
tsrna-26041	CTTCGGATCAGAAGATTGCAGGT	0.07141622	0	0	0
tsrna-26037	CTTCGGATCAGAAGATTGAGGGTT	0.28566487	0.702721	0.139694	0
tsrna-26036	CTTCGGATCAGAAGATTGAGGGT	0.35708108	0.780801	0	0
tsrna-26030	CTTCGGAGGCGTGGGTTC	0	0.15616	0	0
tsrna-26029	CTTCGGAGGCGTGGGTT	0	0.07808	0	0
tsrna-26028	CTTCGGAGGCGTGGGT	0	0.07808	0	0
tsrna-26027	CTTCGATAGCTCAGTTGGTAGAGCG	0.07141622	0.07808	0	0.111027
tsrna-26026	CTTCGATAGCTCAGTTGGTAGAGC	0.14283243	0.07808	0.139694	0
tsrna-26025	CTTCGATAGCTCAGTTGGTAGAG	0.21424865	0	0.279387	0.111027
tsrna-26024	CTTCGATAGCTCAGTTGGTAGA	0	0	0.069847	0
tsrna-26023	CTTCGATAGCTCAGTTGG	0.07141622	0	0.069847	0
tsrna-26022	CTTCGATAGCTCAGCTGGTAGAGC	0.14283243	0	0.139694	0
tsrna-26021	CTTCGATAGCTCAGCTGGTAGAG	0.21424865	0.15616	0	0.111027
tsrna-26020	CTTCGATAGCTCAGCTGGTAGA	0.21424865	0.15616	0.139694	0
tsrna-26019	CTTCGATAGCTCAGCTGGTAG	0.14283243	0.15616	0	0.222054
tsrna-26018	CTTCGATAGCTCAGCTGG	0	0	0	0.222054
tsrna-26017	CTTCGATAGCTCAGCT	0.14283243	0	0.069847	0.111027
tsrna-26014	CTTCCAAGCAGTTGACCCGGGTTCGATTCC	0.92841081	2.108163	0.488928	0.555136
tsrna-26013	CTTCCAAGCAGTTGACCCGGGTTCG	1.21407568	1.639682	0.977855	0.222054
tsrna-26012	CTTCCAAGCAGTTGACCCGGGTTC	1.49974054	1.249282	0.419081	0.888217
tsrna-26011	CTTCCAAGCAGTTGACCCGGGTT	1.42832433	1.873923	0.628621	0
tsrna-26010	CTTCCAAGCAGTTGACCCGGGT	1.49974054	1.015041	0.558774	0.666163
tsrna-26009	CTTCCAAGCAGTTGACCCGGG	0.07141622	0.15616	0	0
tsrna-26008	CTTCCAAGCAGTTGACCCGG	0	0	0	0
tsrna-26007	CTTCCAAGCAGTTGACCCG	0	0.07808	0	0
tsrna-26006	CTTCCAAGCAGTTGACCC	0.07141622	0	0	0
tsrna-26005	CTTCCAAGCAGTTGAC	0.07141622	0	0	0
tsrna-26004	CTTCAATAGCTCAGCTGGTAGAGC	0.07141622	0	0	0
tsrna-26002	CTTCAAAGCCCTCAGTAAGTTG	0	0	0	0
tsrna-26001	CTTCAAACCTGCCGGGGCTTCCA	0.4284973	0.390401	0.279387	0.222054
tsrna-26000	CTTCAAACCTGCCGGGGCTTCC	0	0.07808	0.069847	0
tsrna-25999	CTTCAAACCTGCCGGGGCTTC	0.07141622	0	0	0
tsrna-25994	CTTATAATGCCGAGGTTGTGAGTTC	0.64274595	1.015041	0.419081	0.77719
tsrna-25993	CTTATAATGCCGAGGTTGTGAGTT	1.2854919	1.952003	0.349234	0.888217
tsrna-25992	CTTATAATGCCGAGGTTGTGAGT	0.99982703	1.249282	0.838162	0.333081
tsrna-25991	CTTATAATGCCGAGGTTGTGAG	0	0.23424	0	0.222054
tsrna-25990	CTTATAATGCCGAGGTTGTGA	0.21424865	0.23424	0.069847	0.111027
tsrna-25989	CTTATAATGCCGAGGTTGTG	0.21424865	0.23424	0.069847	0
tsrna-25988	CTTATAATGCCGAGGTTG	0	0.07808	0	0
tsrna-25987	CTTAGGTCGCTGGTTCGATTCCGGCTCGA	0.07141622	0	0	0
tsrna-25986	CTTAGGTCGCTGGTTCGAATCCGGCTCGA	0.07141622	0.15616	0.069847	0

tsrna-25985	CTTAGGTCGCTGGTTCG	0.14283243	0	0	0
tsrna-25984	CTTAGGTCGCTGGTTC	0	0.07808	0	0
tsrna-25983	CTTAGGAGATTTCAACTTAACTTGACCGCTCTGACCA	6.0703784	4.450566	5.378204	0.999244
tsrna-25982	CTTAGGAGATTTCAACTTAACTTGACCGCTCTGACC	0.64274595	1.171202	0.558774	0.555136
tsrna-25981	CTTAGGAGATTTCAACTTAACTTGACCGCTCTGAC	0.28566487	0.390401	0.279387	0
tsrna-25980	CTTAGGAGATTTCAACTTAACTTGACCGCTCTGA	0.07141622	0.390401	0.20954	0.111027
tsrna-25979	CTTAGGAGATTTCAACTTAACTTGACCGCTCTG	0.07141622	0.15616	0.069847	0
tsrna-25978	CTTAGGAGATTTCAACTTAACTTGACCGCT	0.07141622	0	0.20954	0
tsrna-25977	CTTAGGAGATTTCAACTTAACTTGACCGC	0	0.23424	0	0
tsrna-25976	CTTAGGAGATTTCAACTTAACTTGACCG	0	0.07808	0	0
tsrna-25975	CTTAGGAGATTTCAACTTAACTTGACC	0	0.07808	0.069847	0
tsrna-25974	CTTAGGAGATTTCAACTTAACTTGAC	0.07141622	0	0	0
tsrna-25973	CTTAGGAGATTTCAACTTAACTTG	0.07141622	0	0	0
tsrna-25972	CTTAGGAGATTTCAACTTAACTT	0	0.07808	0	0
tsrna-25967	CTTAGGAGATTTCAACT	0	0	0	0
tsrna-25965	CTTAGCTTTGGGTGCTAATGGTGGAGTTA	0.14283243	0.23424	0	0.111027
tsrna-25962	CTTAGCTGTTAACTAAGTGTTTGTGG	0	0	0	0
tsrna-25961	CTTAGCATTAACCTTTTAAGTTAAAGATTAAGAGAA	0	0	0	0
tsrna-25960	CTTAGCATTAACCTTTTAAGTTAAAGATT	0	0	0.069847	0
tsrna-25953	CTTACGACCCCTTATTTACCCCA	0.14283243	0	0.069847	0
tsrna-25952	CTTACGACCCCTTATTTACCCC	0.07141622	0	0	0
tsrna-25951	CTTACGACCCCTTATTTACCC	0	0	0	0
tsrna-25945	CTTACCTCCTCAAAGCAATACACTGA	0.21424865	0.07808	0	0
tsrna-25944	CTTACCTCCTCAAAGCAATACACT	0.07141622	0.31232	0	0.111027
tsrna-25943	CTTACCTCCTCAAAGCAATACA	0.07141622	0	0	0
tsrna-25942	CTTACCTCCTCAAAGCAATAC	0.21424865	0	0.069847	0
tsrna-25941	CTTACCTCCTCAAAGCAATA	0.14283243	0.07808	0.069847	0
tsrna-25937	CTTACACTTAGGAGATTTCAACTTAACTTGACCGCTCTGACCA	7.42728651	3.669765	6.705293	1.55438
tsrna-25936	CTTACACTTAGGAGATTTCAACTTAACTTGACCGCTCTGACC	0.99982703	0.780801	1.117549	0.111027
tsrna-25935	CTTACACTTAGGAGATTTCAACTTAACTTGACCGCTCTGAC	0.4284973	0.15616	0.419081	0
tsrna-25934	CTTACACTTAGGAGATTTCAACTTAACTTG	0	0	0	0
tsrna-25933	CTTACACTTAGGAGATTTCAACTTAACTT	0	0	0	0
tsrna-25932	CTTACACTTAGGAGATTTCAACTTAACT	0	0	0.069847	0.111027
tsrna-25931	CTTACACTTAGGAGATTTCAACTTA	0	0	0.069847	0
tsrna-25930	CTTACACTTAGGAGATTTCAACTT	0	0	0	0
tsrna-25929	CTTACACTTAGGAGATTTCAACT	0	0.07808	0	0
tsrna-25921	CTTAATCTCAGGGTCGTGGGTTCGAGCCCCACGTTGGGCGC	1.42832433	19.52003	0.628621	0.888217
tsrna-25920	CTTAATCTCAGGGTCGTGGGT	0.35708108	0.390401	0.279387	0.333081
tsrna-25919	CTTAATCTCAGGGTCGTGGG	0.07141622	0.390401	0.069847	0
tsrna-25918	CTTAATCTCAGGGTCGTGG	0.28566487	0.31232	0.279387	0.333081
tsrna-25917	CTTAATCTCAGGGTCGTG	0.35708108	0.31232	0.20954	0
tsrna-25916	CTTAATCCCAGGGTCGTGGGTTCGAGCCCCACGTTGGGCGC	1.21407568	19.12963	0.558774	0.333081
tsrna-25915	CTTAATCCCAGGGTCGTGGGTT	0.07141622	0	0	0
tsrna-25913	CTTAATCCCAGGGTCGTG	0.07141622	0	0	0
tsrna-25912	CTTAATCCCAGGGTCGT	0.14283243	0	0.069847	0
tsrna-25904	CTTAACTTGACCGCTCTGACCA	1.07124325	1.327362	0.628621	0.222054
tsrna-25903	CTTAACTTGACCGCTCTGACC	0.07141622	0.07808	0	0
tsrna-25902	CTTAACTTGACCGCTCTGAC	0	0.07808	0	0
tsrna-25901	CTTAACTTGACCGCTCTGA	0	0.07808	0	0
tsrna-25900	CTTAACTTGACCGCTCTG	0	0	0	0
tsrna-25897	CTTAACACAAAGCACCCAACTTACACTTAG	0	0	0	0
tsrna-25892	CTTAACACAAAGCACCCAACT	0	0	0.069847	0
tsrna-25886	CTTAAACCCCCTTATTTCTACCA	0.14283243	0.07808	0	0
tsrna-25885	CTTAAACCCCCTTATTTCTACC	0	0.15616	0	0
tsrna-25883	CTTAAACCCCCTTATTTCTA	0	0.07808	0	0
tsrna-25882	CTTAAACCCCCTTATTTCT	0	0.07808	0.069847	0
tsrna-25881	CTTAAACCCCCTTATTT	0	0	0.069847	0
tsrna-25880	CTTAAAACTTTACAGTCAGAGGTTCA	0	0	0.069847	0
tsrna-25879	CTTAAAACTTTACAGTCAGAGGTTC	0.07141622	0	0	0
tsrna-25878	CTTAAAACTTTACAGTCAGAGGTT	0	0.07808	0	0
tsrna-25877	CTTAAAACTTTACAGTCAGAGGT	0	0.23424	0	0
tsrna-25876	CTTAAAACTTTACAGTCAGAGG	0	0.15616	0	0
tsrna-25872	CTGTTAACTGAAAGGTTGGTGGT	0	0.07808	0	0
tsrna-25869	CTGTTAACTAAGTGTTTGTGGGTTTAAGTC	0.07141622	0.07808	0	0.111027

tsrna-25868	CTGTTAACTAAGTGTTTGTGGGTTTA	0.07141622	0	0	0
tsrna-25867	CTGTTAACTAAGTGTTTGTGGGTTT	0	0.07808	0	0.111027
tsrna-25866	CTGTTAACTAAGTGTTTGTGGGTT	0	0.07808	0	0
tsrna-25865	CTGTTAACTAAGTGTTTGTGGGT	0.07141622	0	0	0
tsrna-25863	CTGTTAACTAAGTGTTTGTGG	0	0	0	0
tsrna-25858	CTGTTAACCGAAAGGTTGGTGGT	0.49991351	2.888964	0.139694	0.77719
tsrna-25857	CTGTTAACCGAAAGGTTGGTGG	0	0.15616	0	0
tsrna-25856	CTGTTAACCGAAAGGTTGGTG	0	0.23424	0	0
tsrna-25850	CTGTTAACCGAAAGATTGGTGGT	0	0	0	0
tsrna-25849	CTGTTAACCGAAAGATTGGTGG	0	0	0.069847	0
tsrna-25837	CTGTGGCGCAATCGGTTAGC	0	0.07808	0	0
tsrna-25834	CTGTGATGGCCGAGTGGTT	0	0.07808	0	0
tsrna-25833	CTGTCTTGTAAACAGGAGATCCTGGGT	1.57115676	7.33953	0.628621	0.444109
tsrna-25832	CTGTCACGCGGGAGACTGGGGT	2.42815136	9.525773	2.235098	0.888217
tsrna-25831	CTGTCACGCGGGAGACCGGGGTTCGATTCCCCGACGGGGA(	1.2854919	5.075207	0.908008	0.222054
tsrna-25830	CTGTCACGCGGGAGACCGGGGTTCGATTCC	1.07124325	4.138246	0.628621	0.222054
tsrna-25829	CTGTCACGCGGGAGACCGGGGTTCG	0.8569946	2.810884	0.558774	0.222054
tsrna-25828	CTGTCACGCGGGAGACCGGGGTTC	1.07124325	3.513605	0.419081	0
tsrna-25827	CTGTCACGCGGGAGACCGGGGTT	1.2854919	4.372486	0.349234	0.222054
tsrna-25826	CTGTCACGCGGGAGACCGGGGT	0.64274595	3.669765	0.698468	0.333081
tsrna-25825	CTGTCACGCGGGAGACCGGGG	1.64257298	3.825925	0.279387	0.111027
tsrna-25824	CTGTCACGCGGGAGACCGGG	1.07124325	3.201285	0.349234	0.111027
tsrna-25823	CTGTCACGCGGGAGACCGG	1.2854919	2.967044	0.628621	0.333081
tsrna-25822	CTGTCACGCGGGAGACCG	0.71416216	3.669765	0.279387	0.555136
tsrna-25821	CTGTCACGCGGGAGACC	1.2854919	2.810884	0.349234	0.333081
tsrna-25820	CTGTCACGCGGGAGAC	1.14265946	3.357445	0.349234	0.222054
tsrna-25816	CTGTAGTGTAGTGGTTATCACGTTCGC	0	0	0.069847	0
tsrna-25812	CTGTAGTGTAGTGGTTATCACGT	0.07141622	0	0	0
tsrna-25809	CTGTAGTGTAGTGGTTATCA	0	0.07808	0	0
tsrna-25804	CTGTAGATCCTTAGGTCGCTGGTTCGATTC	0.07141622	0	0.069847	0.111027
tsrna-25803	CTGTAGATCCTTAGGTCGCTGGTTCG	0.07141622	0	0	0
tsrna-25802	CTGTAGATCCTTAGGTCGCTGGTTC	0	0	0	0
tsrna-25801	CTGTAGATCCTTAGGTCGCTGGTT	0	0	0.069847	0
tsrna-25789	CTGTAAATCTAAAGACAGGGG	0.07141622	0	0	0
tsrna-25782	CTGTAAAGCTAACTTAGCATTAACCT	0	0.07808	0	0
tsrna-25771	CTGGTTTTCACCCAGGTGGCCCGGGTTCG	0.14283243	0.390401	0.279387	0.222054
tsrna-25770	CTGGTTTTCACCCAGGTGGCCCGGG	0.14283243	0.31232	0.139694	0
tsrna-25769	CTGGTTTTCACCCAGGTGGCCCGG	0.07141622	0.31232	0.279387	0.111027
tsrna-25768	CTGGTTTTCACCCAGGTGGCCCG	0.14283243	0	0.069847	0.111027
tsrna-25767	CTGGTTTTCACCCAGGTGGCCC	0.21424865	0.23424	0.139694	0
tsrna-25766	CTGGTTTTCACCCAGGTGGCC	0.21424865	0.468481	0.279387	0
tsrna-25765	CTGGTTTTCACCCAGGCGGCCCGG	0.14283243	0.07808	0	0
tsrna-25764	CTGGTTTTCACCCAGGCGGCCCG	0.14283243	0.15616	0	0
tsrna-25763	CTGGTTTTCACCCAGGCGGCCC	0.14283243	0.07808	0	0.111027
tsrna-25762	CTGGTTCGATTCCGGCTCGAAGGACCA	4.3563892	14.28866	1.74617	2.886706
tsrna-25761	CTGGTTCGATTCCGGCTCGAAGGACC	0.07141622	0.936961	0.069847	0
tsrna-25758	CTGGTTCGATTCCGGCTCGAAGG	0	0	0	0
tsrna-25755	CTGGTTCGATCCCGGGTTTCGGCACCA	2.85664866	8.432652	0.698468	0.999244
tsrna-25754	CTGGTTCGATCCCGGGTTTCGGCA	0	0	0	0
tsrna-25752	CTGGTTCGATCCCGGGTTTCGG	0	0	0	0
tsrna-25751	CTGGTTCGAATCCGGCTCGAAGGACCA	1.14265946	3.982086	0.838162	0.77719
tsrna-25750	CTGGTTCGAATCCGGCTCGAAGGACC	0.14283243	0.468481	0.139694	0
tsrna-25747	CTGGTTCGAATCCGGCTCGAAGG	0	0	0	0.111027
tsrna-25744	CTGGTTCAAATCCGGGTGCCCCCTCCA	12.2835892	12.49282	5.378204	1.998488
tsrna-25742	CTGGTGTAGTGGTATCATGCAAGATTC	0.21424865	0.31232	0.069847	0
tsrna-25741	CTGGTGTAGTGGTATCATGCAAGATT	0.21424865	0.31232	0.069847	0
tsrna-25740	CTGGTGTAGTGGTATCATGCAAGA	0.14283243	0.07808	0.139694	0
tsrna-25739	CTGGTGTAGTGGTATCATGCAAG	0.07141622	0	0.069847	0
tsrna-25738	CTGGTGTAGTGGTATCATGCA	0	0	0.069847	0.111027
tsrna-25737	CTGGTGTAGTGGTATCATGC	0	0	0.069847	0
tsrna-25736	CTGGTGTAGTGGTATCATG	0.07141622	0.07808	0.069847	0
tsrna-25734	CTGGTGGTTCAGTGGTAGAATTCT	0	0.07808	0.279387	0
tsrna-25733	CTGGTGGTCTAGTGGTTAGGATTCGGCGCTCTC	2.49956757	4.060166	1.327089	1.665407
tsrna-25732	CTGGTGGTCTAGTGGTTAGGATTCGGCGCTC	2.85664866	4.138246	1.187396	1.443353

tsrna-25731	CTGGTGGTCTAGTGGTTAGGATTCGGCGCT	2.85664866	3.513605	1.396936	1.221299
tsrna-25730	CTGGTGGTCTAGTGGTTAGGATTCGGCGC	1.42832433	1.327362	1.047702	0.888217
tsrna-25729	CTGGTGGTCTAGTGGTTAGGATTCGGCG	0.92841081	0.780801	0.558774	0.444109
tsrna-25728	CTGGTGGTCTAGTGGTTAGGATTCGGC	0.49991351	0.468481	0.558774	0.444109
tsrna-25727	CTGGTGGTCTAGTGGTTAGGATTCGG	0.21424865	0.07808	0.069847	0.222054
tsrna-25726	CTGGTGGTCTAGTGGTTAGGATTCG	0	0.23424	0.069847	0
tsrna-25725	CTGGTGGTCTAGTGGTTAGGATTC	0.4284973	0.15616	0.069847	0
tsrna-25724	CTGGTGGTCTAGTGGTTAGGATT	0.14283243	0.23424	0	0.222054
tsrna-25723	CTGGTGGTCTAGTGGTTAGGAT	0	0.07808	0	0
tsrna-25718	CTGGTGGTCTAGTGGCTAGGATTCGGCGC	0.92841081	2.654724	0.488928	3.552868
tsrna-25717	CTGGTGGTCTAGTGGCTAGGATTCGGCG	1.21407568	3.201285	0.349234	3.552868
tsrna-25716	CTGGTGGTCTAGTGGCTAGGATTCGGC	0.71416216	1.093122	0.069847	1.665407
tsrna-25715	CTGGTGGTCTAGTGGCTAGGATTCGG	0.21424865	0.468481	0.20954	0.999244
tsrna-25714	CTGGTGGTCTAGTGGCTAGGATTCG	0.14283243	0.31232	0.20954	0.666163
tsrna-25713	CTGGTGGTCTAGTGGCTAGGA	0	0	0	0.222054
tsrna-25712	CTGGTGGTCTAGTGGCTAGG	0	0	0	0
tsrna-25710	CTGGTCTCCGGATGGAGGCGTGG	0	0.546561	0.069847	0
tsrna-25709	CTGGTCTCCGGATGGAGGCG	0	0	0	0
tsrna-25705	CTGGTCTCCAATGGAGGCGTGG	0	0.07808	0	0
tsrna-25703	CTGGTCTAGTGGTTAGGATT	0	0	0	0
tsrna-25693	CTGGTAGAGCAGAGGA	0	0.07808	0	0
tsrna-25692	CTGGGTTCGATCCCCAGTACCTCCACCA	1.99965406	3.435525	0.908008	2.109516
tsrna-25691	CTGGGTTCGAGCCCCAGTGGAACCACCA	2.92806487	2.108163	2.095404	2.33157
tsrna-25690	CTGGGTTCGAGCCCCAGTGGAACC	0.07141622	0.15616	0	0
tsrna-25689	CTGGGTTCGAATCCCAGCGGTGCCTCCA	1.71398919	3.982086	0.279387	0.444109
tsrna-25688	CTGGGTTCGAATCCCAGCGGGGCCTCCA	0.07141622	0.390401	0	0
tsrna-25686	CTGGGGATTGTGGGTTCGAGTCCCATCTGGGTCGCCA	0.07141622	1.015041	0.139694	0
tsrna-25685	CTGGGGATTGTGGGTTCGAGTCCCATCTGGGTCGCC	0.21424865	0.31232	0	0
tsrna-25684	CTGGGGATTGTGGGTTCGAGTCCCATCTGG	0.07141622	0.23424	0	0
tsrna-25683	CTGGGGATTGTGGGTTCG	0	0.07808	0	0
tsrna-25681	CTGGGGATTGTGGGTT	0	0	0	0
tsrna-25680	CTGGGCGGAAACACCA	0.07141622	0.07808	0	0
tsrna-25679	CTGGCGGTCTAGTGGTTAGGATTCGGCGC	1.42832433	1.561602	1.187396	0.999244
tsrna-25678	CTGGATAGCTCAGTTGGTAGAAC	0.07141622	0.07808	0	0
tsrna-25673	CTGGACTTTGAATCCAGCGATCC	0.07141622	0.07808	0	0
tsrna-25672	CTGGACTTTGAATCCAGCGA	0	0.15616	0	0
tsrna-25671	CTGGACTTTGAATCCAGCG	0	0	0.069847	0
tsrna-25665	CTGGACTCTGAATCCAGCGATCCGAGTTCG	0	0.23424	0	0.111027
tsrna-25664	CTGGACTCTGAATCCAGCGATCCGAGTTC	0	0.07808	0	0
tsrna-25663	CTGGACTCTGAATCCAGCGATCCGAGTT	0.07141622	0.15616	0	0
tsrna-25662	CTGGACTCTGAATCCAGCGATCCGAGT	0	0.07808	0	0
tsrna-25661	CTGGACTCTGAATCCAGCGATCCGAG	0	0.07808	0	0.111027
tsrna-25660	CTGGACTCTGAATCCAGCGATCCGA	0	0.07808	0	0
tsrna-25659	CTGGACTCTGAATCCAGCGATCCG	0.07141622	0.07808	0	0
tsrna-25658	CTGGACTCTGAATCCAGCGATCC	0	0.15616	0	0.111027
tsrna-25656	CTGGACTCTGAATCCAGCGAT	0	0.07808	0	0
tsrna-25654	CTGGACTCTGAATCCAGCG	0	0	0	0.111027
tsrna-25653	CTGGACTCTGAATCCAGCA	0.07141622	0	0	0
tsrna-25652	CTGGACTCTGAATCCAGC	0	0.07808	0	0
tsrna-25651	CTGGACTCTGAATCCAG	0.07141622	0	0	0
tsrna-25648	CTGCTTTACACGCAGAAGGTCCTGG	0	0	0	0
tsrna-25645	CTGCTAATCCATTGTGCTTTGC	0.07141622	0.15616	0	0
tsrna-25644	CTGCTAATCCATTGTGCTT	0	0.07808	0	0
tsrna-25643	CTGCTAATCCATTGTGCTCTGC	0	0.15616	0.069847	0.111027
tsrna-25639	CTGCTAACTCATGCCCCCATGTCTAACAACATGGCTTTCTCA(	0.49991351	0.23424	1.466783	0
tsrna-25638	CTGCTAACTCATGCCCCCATGTCTAACAAC	0.07141622	0	0	0
tsrna-25637	CTGCTAACTCATGCCCCCATGTCTAACAA	0	0	0	0
tsrna-25636	CTGCTAACTCATGCCCCCATGTCTAACA	0	0	0.069847	0
tsrna-25635	CTGCTAACTCATGCCCCCATGTCTAAC	0	0	0	0
tsrna-25633	CTGCTAACTCATGCCCCCATGTCTA	0.07141622	0	0	0
tsrna-25631	CTGCTAACTCATGCCCCCATGTC	0	0	0	0
tsrna-25618	CTGCGTGTTCGAATCACGTCGGGGTCACC	0	0	0	0.111027
tsrna-25614	CTGCGTGTTCGAATCACGTCGGGGT	0.07141622	0	0	0
tsrna-25604	CTGCCTTCCAAGCAGTTGACCCGGGTTCG	2.14248649	2.888964	1.047702	1.887461

tsrna-25603	CTGCCTTCCAAGCAGTTGACCCGGGTTC	1.85682163	2.654724	1.396936	1.221299
tsrna-25602	CTGCCTTCCAAGCAGTTGACCCGGGTT	2.71381622	2.420483	1.047702	1.665407
tsrna-25601	CTGCCTTCCAAGCAGTTGACCCGGGT	2.21390271	2.967044	1.53663	1.55438
tsrna-25600	CTGCCTTCCAAGCAGTTGACCCGGG	0.07141622	0.390401	0.139694	0
tsrna-25599	CTGCCTTCCAAGCAGTTGACCCGG	0.07141622	0.15616	0.139694	0.222054
tsrna-25598	CTGCCTTCCAAGCAGTTGACCCG	0	0	0.069847	0
tsrna-25595	CTGCCTTCCAAGCAGTTGAC	0	0	0	0
tsrna-25591	CTGCCTGTCACGCGGGAGAC	0.78557838	3.904006	0.349234	0.111027
tsrna-25590	CTGCCACGCGGGAGGCCCGGGTTT	0.49991351	1.483522	0	0
tsrna-25589	CTGCCACGCGGGAGGCCCGGGTTCG	0.28566487	1.327362	0.139694	0.111027
tsrna-25588	CTGCCACGCGGGAGGCCCGGGTTC	0.4284973	1.327362	0.069847	0
tsrna-25587	CTGCCACGCGGGAGGCCCGGGTT	0.28566487	1.639682	0.069847	0.222054
tsrna-25586	CTGCCACGCGGGAGGCCCGGGT	0.07141622	0.858881	0.139694	0
tsrna-25585	CTGCCACGCGGGAGGCCCGGG	0	0.15616	0	0
tsrna-25584	CTGCCACGCGGGAGGCCCGG	0	0	0.069847	0
tsrna-25582	CTGCCACGCGGGAGGCCC	0	0	0.139694	0
tsrna-25579	CTGCAGATCAAGAGGTCCCTGGTTCA	0.49991351	0.546561	0	0.222054
tsrna-25578	CTGCAGATCAAGAGGTCCCTGGTTC	0.21424865	0.390401	0.139694	0.222054
tsrna-25577	CTGCAGATCAAGAGGTCCCTGGTT	0.35708108	0.31232	0.069847	0.222054
tsrna-25576	CTGCAGATCAAGAGGTCCCTGGT	0.14283243	0.23424	0.139694	0.333081
tsrna-25575	CTGCAGATCAAGAGGTCCCTGG	0	0	0	0
tsrna-25574	CTGCAGATCAAGAGGTCCCTG	0	0	0.069847	0
tsrna-25572	CTGCAGATCAAGAGGTCCCCGGTTCAA	0	0.31232	0.069847	0
tsrna-25571	CTGCAGATCAAGAGGTCCCCGGTTCA	0.07141622	0.07808	0	0
tsrna-25570	CTGCAGATCAAGAGGTCCCCGGTTC	0	0.07808	0.20954	0.111027
tsrna-25569	CTGCAGATCAAGAGGTCCCCGGTT	0.28566487	0.31232	0.069847	0
tsrna-25568	CTGCAGATCAAGAGGTCCCCGGT	0	0.15616	0	0.111027
tsrna-25558	CTGCAGATCAAGAAGTCCCCGGT	0.07141622	0.15616	0	0
tsrna-25554	CTGCAGATCAAAAGGTCCCTGGT	0.78557838	0.31232	0.139694	0.333081
tsrna-25550	CTGATTTGCGTTCAGTTGATGCAGAGTGGG	0	0.07808	0.069847	0
tsrna-25549	CTGATTTGCGTTCAGTTGATGCAGAGTGG	0.14283243	0	0.069847	0.111027
tsrna-25548	CTGATTTGCGTTCAGTTGATGCAGAGTG	0	0.07808	0	0
tsrna-25545	CTGATTTGCGTTCAGTTGATGCAGA	0	0	0.069847	0
tsrna-25538	CTGATTCCGGATCAGAAGATTGAGG	0	0.07808	0	0
tsrna-25537	CTGATAACACCAAGGTCGCGGGC	0.35708108	2.030083	0.20954	0.333081
tsrna-25536	CTGATAACACCAAGGTCGCGG	0	0.15616	0.069847	0.111027
tsrna-25534	CTGATAAAAGAGTTACTTTGA	0	0.07808	0	0
tsrna-25532	CTGAGTTCGAACCTCAGAGGGGGCACCA	0.07141622	0.15616	0	0
tsrna-25530	CTGAGTGAAGCATTGGACTGTAAATCTAA	0	0	0.069847	0.111027
tsrna-25529	CTGAGTGAAGCATTGGACTGTAAATCTA	0.07141622	0	0	0
tsrna-25528	CTGAGTGAAGCATTGGACTGTAAATCT	0	0.07808	0.069847	0
tsrna-25527	CTGAGTGAAGCATTGGACTGTAAATC	0.07141622	0.07808	0.139694	0
tsrna-25526	CTGAGTGAAGCATTGGACTGTAAAT	0	0.07808	0.069847	0
tsrna-25525	CTGAGTGAAGCATTGGACTGTAAA	0	0.07808	0	0.111027
tsrna-25524	CTGAGTGAAGCATTGGACTGTAA	0.07141622	0	0	0
tsrna-25523	CTGAGTGAAGCATTGGACTGTA	0.07141622	0	0	0
tsrna-25515	CTGAGGGTCCAGGGTTCAAGTCCCTGTTCGGGCGCCA	0.49991351	1.015041	0.628621	0.222054
tsrna-25514	CTGAGGGTCCAGGGTTCAAGTCCCTGTTCGGGCGCC	0	0	0.069847	0
tsrna-25513	CTGAGGGTCCAGGGTTCAAGTCCCTGTTCGGGCGC	0	0	0	0
tsrna-25512	CTGAGGGTCCAGGGTTCAAGTCCCTGTTCGGGC	0.07141622	0.07808	0	0
tsrna-25511	CTGAGGGTCCAGGGTTCAAGTCCCTGTTCG	0	0.07808	0	0
tsrna-25510	CTGAGGGTCCAGGGTTCA	0	0	0	0
tsrna-25509	CTGAGGGTCCAGGGTTC	0.07141622	0	0	0
tsrna-25503	CTGACTTCGGATCAGAAGATTGAGGGT	0.4284973	0.390401	0.069847	0.222054
tsrna-25497	CTGACTCCAGATCAGAAGGTTGCGTGTTC	0.07141622	0.15616	0	0.111027
tsrna-25496	CTGACTCCAGATCAGAAGGTTGCGTGTT	0	0.390401	0.069847	0
tsrna-25495	CTGACTCCAGATCAGAAGGTTGCGTGT	0	0.07808	0	0
tsrna-25494	CTGACTCCAGATCAGAAGGTTGCGTG	0	0.07808	0	0
tsrna-25490	CTGACTCCAGATCAGAAGGCTGCGTGTT	0	0	0.069847	0
tsrna-25480	CTGACAACAGAGGCTTACGACCCCTTATTTACCCCA	0.14283243	0.07808	0	0
tsrna-25479	CTGACAACAGAGGCTTACGACCCCTTATTT	0	0	0	0
tsrna-25478	CTGACAACAGAGGCTTACGACCCCTTA	0	0	0	0
tsrna-25475	CTGAATCCAGCGATCCGAGTTCAAATCTCGGTGGAACCTCCA	11.4265946	16.8653	5.168664	2.886706
tsrna-25474	CTGAATCCAGCGATCCGAGTTCAAATCTCGGTGGAACCT	0.07141622	0.15616	0.069847	0

tsrna-25473	CTGAATCCAGCGATCCGAGTTCAA	0	0.07808	0	0
tsrna-25472	CTGAATCCAGCGATCCGAGTTCA	0	0.07808	0	0
tsrna-25471	CTGAATCCAGCGATCCGAGTTC	0	0	0	0
tsrna-25470	CTGAATCCAGCGATCCGAGTT	0	0.15616	0	0
tsrna-25468	CTGAATCCAGCGATCCGAG	0	0.15616	0	0
tsrna-25467	CTGAATCCAGCGATCCGA	0	0.07808	0	0
tsrna-25466	CTGAATCCAGCGATCCG	0	0.07808	0	0
tsrna-25464	CTGAAGGTCGTGAGTTCGATCCTCACACGGGGCACCA	0.28566487	2.264323	0.069847	0
tsrna-25463	CTGAAGGTCGTGAGTTCGATCCTCACACGGGGCACC	0.14283243	0.390401	0.139694	0
tsrna-25462	CTGAAGGTCGTGAGTTCGATCCTCACACGGGGCAC	0.07141622	0.07808	0.069847	0
tsrna-25461	CTGAAGGTCGTGAGTTCGATCCTCACACGGGGC	0.07141622	0.15616	0	0
tsrna-25460	CTGAAGGTCGTGAGTTCGATCCTCACACGG	0.21424865	0.31232	0.069847	0
tsrna-25459	CTGAAGGTCGTGAGTTCGATCC	0.07141622	0.31232	0	0.111027
tsrna-25458	CTGAAGGTCGTGAGTTCGAGCCTCACACGGGGCACCA	0	0.23424	0.20954	0.111027
tsrna-25457	CTGAAGGTCGTGAGTTCGAGCCTCACACGG	0	0.31232	0.069847	0.222054
tsrna-25456	CTGAAGGTCGTGAGTTCG	0.07141622	0.07808	0.139694	0
tsrna-25455	CTGAAGGTCGTGAGTTC	0	0.15616	0.139694	0
tsrna-25454	CTGAAGGTCGTGAGTT	0.07141622	0.31232	0.069847	0.111027
tsrna-25453	CTGAAGGTCCTGAGTTCGAGCCTCAGAGAGGGCACCA	0.71416216	3.513605	0.838162	0.555136
tsrna-25452	CTGAAGGTCCTGAGTTCGAACCTCAGAGGGGGCACCA	0.8569946	3.513605	0.908008	0.111027
tsrna-25451	CTGAAGGTCCTGAGTTCGAACCTCAGAGGGGGCACC	0.71416216	2.576644	0.838162	0.222054
tsrna-25450	CTGAAGGTCCTGAGTTCGAACCTCAGAGGGGGC	1.07124325	2.264323	0.838162	0.444109
tsrna-25449	CTGAAGGTCCTGAGTTCGAACC	0.99982703	1.795843	0.628621	0.444109
tsrna-25448	CTGAAGGTCCTGAGTTCGAAC	0.99982703	3.513605	0.698468	0.333081
tsrna-25447	CTGAAGGTCCTGAGTTCGAA	0.99982703	2.498564	0.698468	0.444109
tsrna-25446	CTGAAGGTCCTGAGTTCGA	0.99982703	3.201285	0.20954	0.222054
tsrna-25445	CTGAAGGTCCTGAGTTCG	0.92841081	2.888964	0.20954	0.444109
tsrna-25444	CTGAAGGTCCTGAGTTC	0.64274595	2.576644	0.488928	0.222054
tsrna-25443	CTGAAGGTCCTGAGTT	1.21407568	3.123204	0.628621	0.555136
tsrna-25442	CTGAAGATCTAAAGGTCCCTGGTT	1.35690811	1.795843	0.838162	0.444109
tsrna-25441	CTGAAGATCTAAAGGTCCCTGGT	0.8569946	1.249282	0.279387	0.333081
tsrna-25440	CTGAAGATCTAAAGGTCCCTGG	0	0	0	0
tsrna-25439	CTGAAGATCTAAAGGTCCCTG	0.14283243	0	0	0
tsrna-25438	CTGAAGATCTAAAGGTCCCT	0.07141622	0	0	0
tsrna-25436	CTGAAGATCTAAAGGTCC	0	0.07808	0	0
tsrna-25434	CTGAAAATGTTTAGACGGGCTCACATCACC	4.92771893	3.825925	1.74617	0.666163
tsrna-25433	CTGAAAATGTTTAGACGGGCTCACATC	2.21390271	2.732804	0.838162	0.666163
tsrna-25432	CTGAAAATGTTTAGACGGGCTCACAT	2.07107028	2.810884	1.047702	0.222054
tsrna-25431	CTGAAAATGTTTAGACGGGCTCACA	2.99948109	2.576644	0.768315	0.333081
tsrna-25430	CTGAAAATGTTTAGACGGGCTCAC	1.78540541	1.171202	0.20954	0.111027
tsrna-25429	CTGAAAATGTTTAGACGGGCTCA	0.78557838	1.171202	0.279387	0.111027
tsrna-25428	CTGAAAATGTTTAGACGGGCTC	0.4284973	0.858881	0	0
tsrna-25427	CTGAAAATGTTTAGACGGGCT	0	0.31232	0.069847	0.111027
tsrna-25426	CTGAAAATGTTTAGACGGGC	0.07141622	0.07808	0	0
tsrna-25425	CTGAAAATGTTTAGACGGG	0	0.15616	0	0
tsrna-25421	CTCTTTTAGTATAAATAGTACCGTTAACTT	0	0	0.069847	0
tsrna-25414	CTCTTCGGGGGCGTGGGTTCGAA	0	0.15616	0	0
tsrna-25413	CTCTTCGGGGGCGTGGGTTCG	0.07141622	0.07808	0	0
tsrna-25412	CTCTTCGGGGGCGTGGGTTC	0.07141622	0.23424	0	0
tsrna-25411	CTCTTCGGGGGCGTGGGTT	0.07141622	0.23424	0	0
tsrna-25410	CTCTTCGGGGGCGTGGGT	0	0.15616	0.069847	0
tsrna-25408	CTCTTCGGGGGCGTGG	0	0.07808	0	0
tsrna-25407	CTCTTCGGAGGCGTGG	0.07141622	0.07808	0	0
tsrna-25406	CTCTTAGCGCAGCGGGCAGC	0	0	0	0
tsrna-25404	CTCTTAATCTCAGGGTCGTGGGTT	0.49991351	0.702721	0.069847	0.555136
tsrna-25403	CTCTTAATCTCAGGGTCGTGGGT	0.21424865	0.936961	0.069847	0.333081
tsrna-25402	CTCTTAATCTCAGGGTCGTGGG	0.21424865	0.780801	0.20954	0.111027
tsrna-25401	CTCTTAATCTCAGGGTCGTGG	0.28566487	0.702721	0.069847	0.222054
tsrna-25400	CTCTTAATCTCAGGGTCGTG	0.4284973	0.390401	0.139694	0.222054
tsrna-25399	CTCTTAATCTCAGGGTCGT	0.07141622	0.624641	0.139694	0.111027
tsrna-25397	CTCTTAATCCCAGGGTCGTGGG	0	0.07808	0	0
tsrna-25396	CTCTTAATCCCAGGGTCGTGG	0	0.07808	0	0
tsrna-25395	CTCTTAATCCCAGGGTCGTG	0.07141622	0	0	0
tsrna-25394	CTCTTAATCCCAGGGTCGT	0	0	0.069847	0

tsrna-25389	CTCTGTGGCGCAATGGATAGCGCATTGGA	0.28566487	0.702721	0.069847	0
tsrna-25388	CTCTGTGGCGCAATGGATAGCGCATTGG	0.28566487	0.546561	0.139694	0
tsrna-25387	CTCTGTGGCGCAATGGATAGCGCATTG	0	0	0	0
tsrna-25386	CTCTGTGGCGCAATGGATAGCGCATT	0	0.15616	0	0
tsrna-25379	CTCTGTGGCGCAATGGACGAGCGC	0	0	0	0
tsrna-25371	CTCTGTGGCGCAATGG	0	0	0	0
tsrna-25370	CTCTGTGGCGCAATCGGTTAGCGCATTCGG	0	0.15616	0	0
tsrna-25361	CTCTGGACTTTGAATCCAGCGATCCGAG	0.14283243	0.15616	0.069847	0
tsrna-25360	CTCTGGACTTTGAATCCAGCGATCCG	0.07141622	0.23424	0	0
tsrna-25359	CTCTGGACTTTGAATCCAGCG	0.07141622	0.15616	0	0
tsrna-25358	CTCTGGACTTTGAATCCAGC	0	0.07808	0	0
tsrna-25357	CTCTGGACTTTGAATCCAG	0	0.07808	0	0
tsrna-25353	CTCTGGACTCTGAATCCGGTA	0	0.07808	0	0
tsrna-25351	CTCTGGACTCTGAATCCAGCGATCCGAGTT	0.07141622	0	0	0.111027
tsrna-25350	CTCTGGACTCTGAATCCAGCGATCCGAGT	0	0.07808	0	0
tsrna-25349	CTCTGGACTCTGAATCCAGCGATCCGAG	0	0.07808	0.069847	0.111027
tsrna-25348	CTCTGGACTCTGAATCCAGCGATCCGA	0	0.23424	0	0
tsrna-25347	CTCTGGACTCTGAATCCAGCGATCCG	0.07141622	0.07808	0	0
tsrna-25346	CTCTGGACTCTGAATCCAGCGATCC	0.07141622	0.07808	0	0
tsrna-25345	CTCTGGACTCTGAATCCAGCGATC	0	0.15616	0	0
tsrna-25344	CTCTGGACTCTGAATCCAGCGAT	0.07141622	0.15616	0	0
tsrna-25343	CTCTGGACTCTGAATCCAGCGA	0.14283243	0.15616	0	0
tsrna-25342	CTCTGGACTCTGAATCCAGCG	0	0.31232	0	0
tsrna-25341	CTCTGGACTCTGAATCCAGCC	0	0.07808	0	0
tsrna-25340	CTCTGGACTCTGAATCCAGC	0.07141622	0.23424	0.069847	0
tsrna-25339	CTCTGGACTCTGAATCCAG	0	0.07808	0.069847	0
tsrna-25338	CTCTGGACTCTGAATCCA	0	0.07808	0	0
tsrna-25335	CTCTGCGTTGTGGCCGCAGCAACCTCGGTT	0.07141622	0	0	0
tsrna-25332	CTCTGCGTTGTGGCCGCAGCAACC	0	0	0.069847	0
tsrna-25324	CTCTGCACGCGTGGGTTCG	0.07141622	0.07808	0	0
tsrna-25319	CTCTGAATCCAGCGATCCGAGTTCGAGTCT	0	0.07808	0	0
tsrna-25318	CTCTGAATCCAGCGATCCGAGTTCAAA	0	0.15616	0	0
tsrna-25317	CTCTGAATCCAGCGATCCGAGTTCAA	0.07141622	0.07808	0	0
tsrna-25316	CTCTGAATCCAGCGATCCGAGTTCA	0.07141622	0	0	0
tsrna-25314	CTCTGAATCCAGCGATCCGAGTT	0	0.07808	0	0
tsrna-25313	CTCTGAATCCAGCGATCCGAGT	0	0.07808	0	0
tsrna-25312	CTCTGAATCCAGCGATCCGAG	0	0.07808	0	0
tsrna-25310	CTCTGAATCCAGCGATCCG	0.07141622	0	0	0
tsrna-25309	CTCTGAATCCAGCGATCC	0.07141622	0.07808	0	0
tsrna-25308	CTCTGAATCCAGCGATC	0	0.07808	0	0
tsrna-25306	CTCTCACCGCCGCGGCCCGGGTTCGATTCCCGGTCAGGGAA	2.14248649	24.12675	1.606477	0.888217
tsrna-25305	CTCTCACCGCCGCGGCCCGGGTTCGATTCC	0	0	0	0
tsrna-25304	CTCTCACCGCCGCGGCCCGGGTTCGA	0	0	0	0.111027
tsrna-25302	CTCTCACCGCCGCGGCCCGGGTTC	0	0.07808	0	0.111027
tsrna-25301	CTCTCACCGCCGCGGCCCGGGTT	0	0.07808	0	0.111027
tsrna-25298	CTCTCACCGCCGCGGCCCGG	0	0	0	0.111027
tsrna-25296	CTCTCACCGCCGCGGCCC	0.07141622	0	0	0
tsrna-25292	CTCGTTGGTCTAGGGGTATGATTCTCGGTT	0.35708108	0.15616	0.20954	0.222054
tsrna-25291	CTCGTTGGTCTAGGGGTATGATTCTCGG	0.21424865	0.07808	0.20954	0
tsrna-25290	CTCGTTGGTCTAGGGGTATGATTCTCGCTT	2.07107028	2.732804	1.257243	2.775678
tsrna-25289	CTCGTTGGTCTAGGGGTATGATTCTCGCT	1.64257298	1.483522	0.698468	2.220543
tsrna-25288	CTCGTTGGTCTAGGGGTATGATTCTCGC	0.8569946	0.390401	0.558774	1.332326
tsrna-25287	CTCGTTGGTCTAGGGGTATGATTCTCG	0.07141622	0	0	0
tsrna-25286	CTCGTTGGTCTAGGGGTATGATTCTC	0.07141622	0.07808	0.069847	0
tsrna-25285	CTCGTTGGTCTAGGGGTATGATTCT	0.07141622	0	0	0
tsrna-25284	CTCGTTGGTCTAGGGGTATGATTC	0	0	0	0
tsrna-25283	CTCGTTGGTCTAGGGGTATGATT	0	0	0	0.111027
tsrna-25273	CTCGTTAGTATAGTGGTGAGTATCCCCGCCTGT	0.07141622	0.468481	0.20954	0.111027
tsrna-25272	CTCGTTAGTATAGTGGTGAGTATCCCCGCCT	0.14283243	0.390401	0.069847	0.222054
tsrna-25271	CTCGTTAGTATAGTGGTGAGTATCCCCGCC	0.21424865	0.31232	0.069847	0
tsrna-25270	CTCGTTAGTATAGTGGTGAGTATCCCCGC	0.07141622	0.31232	0.069847	0.111027
tsrna-25269	CTCGTTAGTATAGTGGTGAGTATCCCCG	0	0.15616	0	0.111027
tsrna-25268	CTCGTTAGTATAGTGGTGAGTATCCCC	0	0.07808	0	0
tsrna-25267	CTCGTTAGTATAGTGGTGAGTATCCC	0	0.07808	0	0

tsrna-25243	CTCGGTTCGAATCCGAGTCACGGCACCA	0.14283243	1.093122	0	0
tsrna-25242	CTCGGTTCGAATCCGAGTCACGGCAC	0	0.07808	0	0
tsrna-25241	CTCGGTTCGAATCCGAGTCACGGCA	0.07141622	0.07808	0	0
tsrna-25235	CTCGGTGGGACCTCCA	0.21424865	0.468481	0.069847	0.333081
tsrna-25234	CTCGGTGGGACCCCCA	0	0	0.069847	0
tsrna-25233	CTCGGTGGAACCTCCA	0.4284973	1.327362	0.20954	0.333081
tsrna-25232	CTCGCTTTGGGTGCGAGAGGTCCCGG	0	0.07808	0	0
tsrna-25231	CTCGCTTTGGGTGCGAGAGGTCCCG	0	0.15616	0	0
tsrna-25228	CTCGCTGGGCCTCCA	0	0.07808	0	0.222054
tsrna-25227	CTCGCCTGCCACGCGGGAGGCCCGGGTTCG	0.35708108	2.264323	0.139694	0.333081
tsrna-25226	CTCGCCTGCCACGCGGGAGGCCCGGGT	0.49991351	1.873923	0.419081	0
tsrna-25225	CTCGCCTGCCACGCGGGAGGCCCGGG	0	0.07808	0	0
tsrna-25224	CTCGCCTGCCACGCGGGAGGCCCGG	0	0.15616	0	0.111027
tsrna-25222	CTCGCCTGCCACGCGGGAGGCCC	0	0.07808	0	0
tsrna-25214	CTCCTGGCTGGCTCGCCA	0.78557838	4.216326	0.838162	0.444109
tsrna-25211	CTCCTGGCTGGCTCACCA	0.21424865	0.31232	0.139694	0
tsrna-25209	CTCCTCAAAGCAATACACTGAAAATGTTT	0.14283243	0.07808	0.139694	0.111027
tsrna-25203	CTCCTAAGCCAGGGATTGTGGGT	9.71260543	34.97989	6.286213	5.218275
tsrna-25202	CTCCTAAGCCAGGGATTGTGGG	0.14283243	0.390401	0.069847	0.222054
tsrna-25201	CTCCTAAGCCAGGGATTGTGG	0.07141622	0.07808	0	0
tsrna-25200	CTCCTAAGCCAGGGATTGTG	0.07141622	0	0	0
tsrna-25199	CTCCTAAGCCAGGGATTGT	0	0	0.069847	0
tsrna-25196	CTCCGGATGGAGGCGTGGG	0	0.546561	0.069847	0
tsrna-25195	CTCCGGATGGAGGCGTGG	0	0.31232	0.069847	0
tsrna-25194	CTCCGGATGGAGGCGTG	0.07141622	0.23424	0	0
tsrna-25193	CTCCGGAGCTGGGGATTGTGGGTTC	0	0.23424	0	0.111027
tsrna-25192	CTCCGGAGCTGGGGATTGTGGGT	0.07141622	0.07808	0	0
tsrna-25190	CTCCGGAGCTGGGGATTGTGG	0	0	0	0
tsrna-25188	CTCCGGAGCTGGGGATTGT	0	0	0.069847	0
tsrna-25187	CTCCGAGGTGATTTTCATATTGAATTGCA	0	0	0	0.111027
tsrna-25186	CTCCGAGGTGATTTTCATATTGAATTGC	0.07141622	0	0	0
tsrna-25185	CTCCGAGGTGATTTTCATATTGAATTG	0	0	0	0
tsrna-25178	CTCCGAGGTGATTTTCATAT	0	0	0	0
tsrna-25177	CTCCGAGGTGATTTTCATA	0.07141622	0	0	0
tsrna-25173	CTCCCTGGTGGTCTAGTGGTTAGGATTCGGCGCTCTC	45.7063785	23.81443	49.1023	11.8799
tsrna-25172	CTCCCTGGTGGTCTAGTGGTTAGGATTCGGCGCTC	10.6410162	8.120331	8.241923	4.996221
tsrna-25171	CTCCCTGGTGGTCTAGTGGTTAGGATTCGGCGCT	3.14231352	6.324489	3.073259	4.108004
tsrna-25170	CTCCCTGGTGGTCTAGTGGTTAGGATTCGGCGC	2.64240001	4.762887	2.235098	3.88595
tsrna-25169	CTCCCTGGTGGTCTAGTGGTTAGGATTCGGCG	2.99948109	3.045124	2.584332	3.330814
tsrna-25168	CTCCCTGGTGGTCTAGTGGTTAGGATTCGGC	2.64240001	3.513605	2.165251	2.553624
tsrna-25167	CTCCCTGGTGGTCTAGTGGTTAGGATTCGG	0.99982703	3.123204	1.955711	2.775678
tsrna-25166	CTCCCTGGTGGTCTAGTGGTTAGG	1.35690811	2.186243	1.53663	2.553624
tsrna-25165	CTCCCGGTGTGGGAACCA	0	0.31232	0.069847	0.111027
tsrna-25162	CTCCCGGTATGGGAACCA	0	0.07808	0	0
tsrna-25157	CTCCCAGTGGGGCCTCCA	0	0.15616	0	0
tsrna-25155	CTCCCACGCGGGAGACCCGG	0.07141622	0	0	0
tsrna-25153	CTCCCACATGGTCTAGCGGTTAGGATTCCT	0.64274595	0.702721	0.488928	1.887461
tsrna-25148	CTCCAGTCTCTTCGGGGGCGTGGGTTCGAATCCCACCGCTG(	0.49991351	2.342403	0.069847	0.111027
tsrna-25147	CTCCAGTCTCTTCGGGGGCGTGGGTTCGA	0.07141622	0.07808	0.069847	0.111027
tsrna-25146	CTCCAGTCTCTTCGGGGGCGTGGGTTC	0	0.23424	0	0
tsrna-25145	CTCCAGTCTCTTCGGGGGCGTGGGT	0	0.07808	0	0
tsrna-25144	CTCCAGTCTCTTCGGGGGCGTGGG	0	0	0	0
tsrna-25140	CTCCAGTCTCTTCGGGGGCG	0	0.07808	0	0
tsrna-25131	CTCCAGTCATTTCGATGGCGTGGGTTCGAATCCCACCGCTGC	0.4284973	1.405442	0.069847	0.111027
tsrna-25129	CTCCAGATCAGAAGGTTGCGTGTTCA	0	0.31232	0.069847	0
tsrna-25128	CTCCAGATCAGAAGGTTGCGTGTTC	0.07141622	0.15616	0	0
tsrna-25127	CTCCAGATCAGAAGGTTGCGTGTT	0.21424865	0.23424	0.20954	0
tsrna-25125	CTCCAGATCAGAAGGTTGCGTG	0.07141622	0	0	0
tsrna-25118	CTCCAGATCAGAAGGCTGCGTGTTCGAA	0	0	0	0
tsrna-25117	CTCCAGATCAGAAGGCTGCGTGTTCGA	0.07141622	0	0.069847	0
tsrna-25116	CTCCAGATCAGAAGGCTGCGTGTTCG	0	0	0.069847	0
tsrna-25115	CTCCAGATCAGAAGGCTGCGTGTTC	0	0	0.069847	0.111027
tsrna-25114	CTCCAGATCAGAAGGCTGCGTGTT	0.07141622	0.07808	0	0
tsrna-25113	CTCCAGATCAGAAGGCTGCGTGT	0	0.07808	0	0

tsrna-25105	CTCCAATGGAGGCGTGGGT	0.71416216	2.030083	0.698468	0
tsrna-25104	CTCCAAATAAAAGTACCA	0.07141622	0	0	0
tsrna-25101	CTCATTAAATTATGATAATCATATTTACC	0	0	0.069847	0
tsrna-25100	CTCATTAAATTATGATAATCATATTTA	0	0	0.069847	0
tsrna-25099	CTCATGCCCCCATGTCTAACAACATGGCTTTCTCACCA	0.8569946	0.546561	1.53663	0
tsrna-25098	CTCATGCCCCCATGTCTAACAACATGGCTTTCTCACC	0.14283243	0.07808	0.139694	0.111027
tsrna-25097	CTCATGCCCCCATGTCTAACAACATGGCTTTCTCAC	0.14283243	0	0.069847	0
tsrna-25096	CTCATGCCCCCATGTCTAACAACATGGCTT	0	0	0.069847	0
tsrna-25094	CTCATGCCCCCATGTCTAACAACATGGC	0	0	0	0
tsrna-25093	CTCATGCCCCCATGTCTAACAACATGG	0	0	0.069847	0
tsrna-25082	CTCATAATCTGAAGGTCGTGAGTTCG	2.21390271	3.513605	1.53663	0.666163
tsrna-25081	CTCATAATCTGAAGGTCGTGAGTT	1.49974054	3.435525	1.257243	0.888217
tsrna-25080	CTCATAATCTGAAGGTCGTGAGT	1.49974054	2.654724	1.466783	0.666163
tsrna-25079	CTCATAATCTGAAGGTCGTGAG	0.21424865	0	0.20954	0.111027
tsrna-25078	CTCATAATCTGAAGGTCGTGA	0.21424865	0.31232	0.20954	0.111027
tsrna-25077	CTCATAATCTGAAGGTCGTG	0.07141622	0.624641	0.069847	0
tsrna-25076	CTCATAATCTGAAGGTCGT	0.07141622	0	0.069847	0
tsrna-25075	CTCATAATCTGAAGGTCCTGAGTTCGAACCTCAGAGGGGGC/	10.2125189	28.57732	6.146519	7.438818
tsrna-25074	CTCATAATCTGAAGGTCCTGAGT	8.28428111	23.50211	7.333915	5.773411
tsrna-25073	CTCATAATCTGAAGGTCCTGAG	0.57132973	1.795843	0.349234	0.333081
tsrna-25072	CTCATAATCTGAAGGTCCTGA	0.4284973	2.186243	0.488928	0.111027
tsrna-25071	CTCATAATCTGAAGGTCCT	0	0.468481	0.069847	0
tsrna-25070	CTCATAATCTGAAGGTCC	0.14283243	0.23424	0	0
tsrna-25067	CTCAGTTGGGAGAGCGTTAGACTGA	0	0.15616	0	0.111027
tsrna-25064	CTCAGTGGTAGAGCATTTGACTGCA	0.4284973	0.31232	0.069847	0.111027
tsrna-25063	CTCAGTGGTAGAGCATTTGACTGC	0.28566487	0.23424	0.069847	0.222054
tsrna-25062	CTCAGTGGTAGAGCATTTGACTG	0	0.07808	0.069847	0
tsrna-25061	CTCAGTGGTAGAGCATTTGACT	0.14283243	0.07808	0	0
tsrna-25060	CTCAGTGGTAGAGCATTTGAC	0.14283243	0	0	0
tsrna-25059	CTCAGTGGTAGAGCATTTGA	0.07141622	0	0.069847	0
tsrna-25058	CTCAGTGGTAGAGCATTTG	0.07141622	0.07808	0.069847	0
tsrna-25055	GGTTCAAATCCGGGTGCCCCCTCCA	11.0695135	15.38178	4.609889	2.33157
tsrna-25054	GGTTCAAATCCGGGTGCCCCCTCC	0.07141622	0.390401	0.139694	0
tsrna-25053	GGTTCAAATCCGGGTGCCCCCTC	0	0.07808	0	0
tsrna-25048	GGTTCAAATCCCGGACGAGCCCCCA	9.35552435	15.92834	3.981268	5.884438
tsrna-25046	GGTTCAAATCCCGGACGA	0	0.07808	0.069847	0
tsrna-25043	GGTTATCACGTTCGCCTCACACG	0	0	0.069847	0
tsrna-25034	GGTTATCACGTCTGCTTTACA	0	0	0.069847	0
tsrna-25033	GGTTATCACGTCTGCTTTAC	0.07141622	0	0	0
tsrna-25030	GGTTATCACGTCTGCTT	0	0	0	0
tsrna-25021	GGTTATACCCTTCCCGTACTACCA	0.07141622	0.23424	0	0
tsrna-25019	GGTTATACCCTTCCCGTACTAC	0	0	0	0
tsrna-25018	GGTTATACCCTTCCCGTACTA	0	0	0.069847	0
tsrna-25017	GGTTATACCCTTCCCGTACT	0.07141622	0	0	0
tsrna-25012	GGTTAGTATCCCCGCCTGTCACGCGGGAG	0.99982703	4.528646	0.698468	0.333081
tsrna-25011	GGTTAGTATCCCCGCCTGTCAC	0	0.07808	0	0
tsrna-25010	GGTTAGTATCCCCGCCTGTC	0	0	0	0.111027
tsrna-25007	GGTTAGTATCCCCGCCT	0	0.07808	0	0
tsrna-25005	GGTTAGTACTCTGCGTTGTGGCCGCAGCAACCT	0.28566487	0.23424	0	0
tsrna-25004	GGTTAGTACTCTGCGTTGTGGCCGCAGC	0.21424865	0.23424	0	0
tsrna-25003	GGTTAGTACTCTGCGTTGTGGCCGC	0.35708108	0	0.069847	0
tsrna-25002	GGTTAGTACTCTGCGTTGTGGCCG	0.35708108	0.07808	0	0
tsrna-25001	GGTTAGTACTCTGCGTTGTGGCC	0.21424865	0.23424	0.069847	0.111027
tsrna-25000	GGTTAGTACTCTGCGTTGTGGC	0.14283243	0	0.069847	0.111027
tsrna-24999	GGTTAGTACTCTGCGTTGTGG	0.14283243	0	0.139694	0
tsrna-24998	GGTTAGTACTCTGCGTTGTG	0.21424865	0.07808	0.069847	0
tsrna-24997	GGTTAGTACTCTGCGTTGT	0.14283243	0	0	0.222054
tsrna-24996	GGTTAGTACTCTGCGTTG	0	0.07808	0.069847	0
tsrna-24995	GGTTAGTACTCTGCGTT	0	0	0.139694	0
tsrna-24993	GGTTAGTACTCTGCGCTGT	0	0.07808	0	0
tsrna-24992	GGTTAGTACTCTGCGCTG	0.21424865	0	0	0
tsrna-24989	GGTTAGGCCTCTTTTTACCACCA	0.14283243	0.15616	0.349234	0.222054
tsrna-24988	GGTTAGGCCTCTTTTTACCACC	0.07141622	0	0.069847	0
tsrna-24987	GGTTAGGCCTCTTTTTACCAC	0	0.07808	0	0

tsrna-24986	GGTTAGGCCTCTTTTTACCA	0	0	0.069847	0
tsrna-24985	GGTTAGGCCTCTTTTTACC	0	0	0.069847	0
tsrna-24983	GGTTAGGCCTCTTTTTA	0	0	0	0
tsrna-24981	GGTTAGGATTCGGCGCTCTCATC	0.28566487	0	0	0
tsrna-24980	GGTTAGGATTCGGCGCTCTCAT	0	0.390401	0.069847	0.222054
tsrna-24979	GGTTAGGATTCGGCGCTCTCACCGCCGCGGCCCGGGTT	0.4284973	0.546561	0.349234	0.333081
tsrna-24978	GGTTAGGATTCGGCGCTCTCACCGCCGCGGCCCGGG	0.4284973	1.093122	0.20954	0.222054
tsrna-24977	GGTTAGGATTCGGCGCTCTCACCGCCGCGGCCC	0.64274595	1.639682	0.20954	0.444109
tsrna-24976	GGTTAGGATTCGGCGCTCTCACCGCCGCGG	0.28566487	1.483522	0.139694	0.222054
tsrna-24975	GGTTAGGATTCGGCGCTCTCACCGCC	0.35708108	0.858881	0.419081	0.111027
tsrna-24974	GGTTAGGATTCGGCGCTCTCACCGC	0	0.390401	0.069847	0
tsrna-24973	GGTTAGGATTCGGCGCTCTCACCG	0.21424865	0.15616	0.139694	0.111027
tsrna-24972	GGTTAGGATTCGGCGCTCTCACC	0	0.31232	0.139694	0
tsrna-24971	GGTTAGGATTCGGCGCTCTCAC	0.07141622	0.31232	0.139694	0
tsrna-24970	GGTTAGGATTCGGCGCTCTCA	0	0.07808	0	0
tsrna-24969	GGTTAGGATTCGGCGCTCTC	0.14283243	0.15616	0	0.222054
tsrna-24968	GGTTAGGATTCGGCGCTCT	0.07141622	0.07808	0.139694	0
tsrna-24967	GGTTAGGATTCGGCGCTC	0.07141622	0.23424	0	0.111027
tsrna-24966	GGTTAGGATTCGGCGCT	0.14283243	0.15616	0	0.111027
tsrna-24965	GGTTAGGATTCGGCGC	0.07141622	0	0	0
tsrna-24964	GGTTAGGATTCCTGGTTTTCACC	0.28566487	0.31232	0.419081	0.999244
tsrna-24963	GGTTAGGATTCCTGGTTTTCAC	0.64274595	0	0	0.333081
tsrna-24962	GGTTAGGATTCCTGGTTTTC	0	0	0	0
tsrna-24960	GGTTAGGATTCCTGGTTT	0	0.07808	0	0
tsrna-24959	GGTTAGGATTCCTGGTT	0	0	0	0
tsrna-24955	GGTTAGCTCAGTTGGTTAGA	0	0	0.069847	0
tsrna-24946	GGTTAGCGCGTTCGGCTGTTAA	0	0.07808	0	0
tsrna-24941	GGTTAGCGCGCGGTACTTATA	0	0.07808	0	0
tsrna-24940	GGTTAGCGCGCGGTACTTA	0.21424865	0	0	0
tsrna-24937	GGTTAGCATAGCTGCCTTCCAAGC	0	0.07808	0	0
tsrna-24936	GGTTAGCATAGCTGCCTTCCAAG	0	0	0	0
tsrna-24930	GGTTAGCACTCTGGACTTTGAATCCAGCGATCCGAGTT	0.49991351	1.405442	0.20954	0.333081
tsrna-24929	GGTTAGCACTCTGGACTTTGAATCCAGCGATCCGAGT	0.07141622	0.31232	0.139694	0.111027
tsrna-24928	GGTTAGCACTCTGGACTTTGAATCCAGCGATCCGAG	0.35708108	0.31232	0.139694	0
tsrna-24927	GGTTAGCACTCTGGACTTTGAATCCAGCAATCCGAG	0.35708108	0.23424	0.20954	0.111027
tsrna-24926	GGTTAGCACTCTGGACTTTGAATCCAGC	0.14283243	0.07808	0.139694	0.555136
tsrna-24925	GGTTAGCACTCTGGACTTTGAATCC	0.14283243	0.858881	0.20954	0.222054
tsrna-24924	GGTTAGCACTCTGGACTTTGAATC	0	0.390401	0	0
tsrna-24922	GGTTAGCACTCTGGACTTTGAA	0	0.07808	0	0
tsrna-24918	GGTTAGCACTCTGGACTT	0	0	0	0
tsrna-24917	GGTTAGCACTCTGGACTCTGAATCCAGCGATCCGAGTTC	0.35708108	0.546561	0.139694	0
tsrna-24916	GGTTAGCACTCTGGACTCTGAATCCAGCGATCCGAGTT	0	0.07808	0.139694	0.111027
tsrna-24915	GGTTAGCACTCTGGACTCTGAATCCAGCGATCCGAGT	0.28566487	0.390401	0.069847	0
tsrna-24914	GGTTAGCACTCTGGACTCTGAATCCAGCGATCCGAG	0.35708108	0.546561	0.069847	0.222054
tsrna-24913	GGTTAGCACTCTGGACTCTGAATCCAGCGATCCG	0.07141622	0.31232	0.20954	0
tsrna-24912	GGTTAGCACTCTGGACTCTGAATCCAGCGATCC	0.14283243	0.546561	0.139694	0.222054
tsrna-24911	GGTTAGCACTCTGGACTCTGAATCCAGCGATC	0.14283243	0.31232	0.139694	0
tsrna-24910	GGTTAGCACTCTGGACTCTGAATCCAGCG	0	0.390401	0.069847	0.222054
tsrna-24909	GGTTAGCACTCTGGACTCTGAATCCAGC	0.21424865	0.23424	0.279387	0
tsrna-24908	GGTTAGCACTCTGGACTCTGAATCCAG	0.07141622	0.23424	0.069847	0
tsrna-24907	GGTTAGCACTCTGGACTCTGAATCCA	0.07141622	0.07808	0	0
tsrna-24906	GGTTAGCACTCTGGACTCTGAATCC	0.07141622	0.23424	0	0
tsrna-24905	GGTTAGCACTCTGGACTCTGAATC	0	0.07808	0	0
tsrna-24904	GGTTAGCACTCTGGACTCTGAAT	0	0	0	0
tsrna-24903	GGTTAGCACTCTGGACTCTGAA	0	0	0.069847	0
tsrna-24897	GGTTAGCACTCTGGAC	0	0	0	0
tsrna-24884	GGTTAAGGCGTTGGACT	0	0	0	0
tsrna-24882	GGTTAAGGCGATGGACTAGAAA	0	0.07808	0	0
tsrna-24879	GGTTAAATACAGACCAAGAGCCTTCA	0.64274595	0.390401	0.908008	0.555136
tsrna-24878	GGTTAAATACAGACCAAGAGCC	0.07141622	0	0.069847	0.111027
tsrna-24876	GGTTAAATACAGACCAAGAG	0	0	0.069847	0.111027
tsrna-24875	GGTTAAATACAGACCAAGA	0	0	0.069847	0
tsrna-24872	GGTGTGAGAGGTCCCGGGT	0.07141622	0	0	0
tsrna-24870	GGTGTAGTGGTATCATGCAAGATTCCCA	0	0	0	0

tsrna-24869	GGTGTAGTGGTATCATGCAAGATTCCC	0.07141622	0	0	0
tsrna-24867	GGTGTAGTGGTATCATGCAAGATTC	0	0	0	0
tsrna-24865	GGTGTAGTGGTATCATGCAAGAT	0	0	0.069847	0
tsrna-24861	GGTGTAGTGGTATCATGCA	0.07141622	0	0	0
tsrna-24859	GGTGTAGTGGTATCATG	0.07141622	0	0	0
tsrna-24858	GGTGTAGCTCAGTGGTAGAGCGCGTGC	0.4284973	1.093122	0.139694	0.111027
tsrna-24857	GGTGTAGCTCAGTGGTAGAGCGCG	0	0	0.069847	0
tsrna-24855	GGTGTAGCTCAGTGGTAGAGCATTTGACT	0.21424865	0.07808	0	0
tsrna-24854	GGTGTAGCTCAGTGGTAGAGC	0.07141622	0	0	0
tsrna-24848	GGTGTAATGGTTAGCACTCTGGGCT	0	0.07808	0	0
tsrna-24847	GGTGTAATGGTTAGCACTCTGGACTTTGA	0	0	0.069847	0
tsrna-24846	GGTGTAATGGTTAGCACTCTGGACTTTG	0.14283243	0.07808	0	0.111027
tsrna-24845	GGTGTAATGGTTAGCACTCTGGACTTT	0.07141622	0	0.139694	0.111027
tsrna-24844	GGTGTAATGGTTAGCACTCTGGACTT	0.07141622	0	0	0
tsrna-24843	GGTGTAATGGTTAGCACTCTGGACTCTGAATCCAGC	0.49991351	0.468481	0.628621	0.77719
tsrna-24842	GGTGTAATGGTTAGCACTCTGGACTCTGA	0.14283243	0	0.069847	0.222054
tsrna-24841	GGTGTAATGGTTAGCACTCTGGACTCTG	0.07141622	0.07808	0	0.111027
tsrna-24840	GGTGTAATGGTTAGCACTCTGGACTCT	0	0.15616	0.069847	0.222054
tsrna-24839	GGTGTAATGGTTAGCACTCTGGACTC	0.07141622	0	0.069847	0
tsrna-24838	GGTGTAATGGTTAGCACTCTGGACT	0	0	0.069847	0
tsrna-24837	GGTGTAATGGTTAGCACTCTGGAC	0	0	0	0.111027
tsrna-24836	GGTGTAATGGTTAGCACTCTGGA	0	0.07808	0	0.222054
tsrna-24835	GGTGTAATGGTTAGCACTCTGG	0	0.07808	0	0
tsrna-24832	GGTGTAATGGTGAGCACTCTGGACTC	0.07141622	0	0.069847	0
tsrna-24830	GGTGTAATGGTGAGCACTCTGGAC	0	0.07808	0	0
tsrna-24829	GGTGTAATGGTGAGCACTCTGGA	0	0	0	0
tsrna-24828	GGTGTAATGGTGAGCACTCTGG	0.07141622	0	0	0
tsrna-24826	GGTGTAATGGTCAGCACTCTGGACT	0.07141622	0	0	0
tsrna-24824	GGTGTAATGGTAAGCACTCTGGACTC	0	0	0	0
tsrna-24821	GGTGGTTTAGTGGTAGAATTCTCGCCT	0.07141622	0.390401	0	0
tsrna-24820	GGTGGTTTAGTGGTAGAATTCTCGCC	0.35708108	0.31232	0.139694	0.111027
tsrna-24819	GGTGGTTTAGTGGTAGAATTCTCGC	0.07141622	0	0	0
tsrna-24818	GGTGGTTTAGTGGTAGAATTCTCG	0.07141622	0	0	0
tsrna-24817	GGTGGTTTAGTGGTAGAATTCTC	0	0	0	0.111027
tsrna-24815	GGTGGTTTAGTGGTAGAAT	0	0.07808	0	0
tsrna-24813	GGTGGTTCAGTGGTAGAATTCTT	0.14283243	0.15616	0.069847	0.111027
tsrna-24812	GGTGGTTCAGTGGTAGAATTCTCGCCTTTC	0.78557838	0.624641	0.349234	0.222054
tsrna-24811	GGTGGTTCAGTGGTAGAATTCTCGCCTGCC	1.49974054	2.654724	0.628621	0.666163
tsrna-24810	GGTGGTTCAGTGGTAGAATTCTCGCCTGC	1.07124325	0.858881	0.279387	0.111027
tsrna-24809	GGTGGTTCAGTGGTAGAATTCTCGCCTG	0.78557838	0.858881	0.488928	0.333081
tsrna-24808	GGTGGTTCAGTGGTAGAATTCTCGCCTCC	0.57132973	0.780801	0.349234	0.999244
tsrna-24807	GGTGGTTCAGTGGTAGAATTCTCGCCTC	0.4284973	0.702721	0.279387	0.444109
tsrna-24806	GGTGGTTCAGTGGTAGAATTCTCGCCT	0.07141622	0.780801	0.20954	0.77719
tsrna-24805	GGTGGTTCAGTGGTAGAATTCTCGCC	0.28566487	0.468481	0.20954	0.222054
tsrna-24804	GGTGGTTCAGTGGTAGAATTCTCGC	0.14283243	0.23424	0.069847	0.222054
tsrna-24803	GGTGGTTCAGTGGTAGAATTCTCG	0.14283243	0	0	0.111027
tsrna-24802	GGTGGTTCAGTGGTAGAATTCTC	0	0.390401	0.069847	0
tsrna-24801	GGTGGTTCAGTGGTAGAATTCT	0.14283243	0.15616	0.069847	0
tsrna-24800	GGTGGTTCAGTGGTAGAATTC	0.21424865	0.07808	0.069847	0
tsrna-24799	GGTGGTTCAGTGGTAGAATT	0.14283243	0.23424	0.069847	0
tsrna-24798	GGTGGTTCAGTGGTAGAAT	0.28566487	0.15616	0.069847	0.111027
tsrna-24797	GGTGGTTCAGTGGTAGAA	0.14283243	0.07808	0.069847	0.111027
tsrna-24796	GGTGGTTCAGTGGTAGA	0	0.07808	0	0
tsrna-24794	GGTGGTTCAATGGTAGAATTCTCGCCT	0.07141622	0	0.069847	0
tsrna-24793	GGTGGTCTAGTGGTTAGGATTCGGCGCTCT	2.78523244	3.357445	1.396936	1.776434
tsrna-24792	GGTGGTCTAGTGGTTAGGATTCGGCGCTC	2.49956757	2.967044	1.53663	1.221299
tsrna-24791	GGTGGTCTAGTGGTTAGGATTCGGCGCT	2.35673514	2.888964	1.606477	2.109516
tsrna-24790	GGTGGTCTAGTGGTTAGGATTCGGCGC	1.49974054	1.795843	0.558774	0.77719
tsrna-24789	GGTGGTCTAGTGGTTAGGATTCGGCG	1.07124325	0.702721	0.488928	0.888217
tsrna-24788	GGTGGTCTAGTGGTTAGGATTCGGC	0.57132973	0.31232	0.349234	0.77719
tsrna-24787	GGTGGTCTAGTGGTTAGGATTCGG	0.28566487	0	0.069847	0
tsrna-24786	GGTGGTCTAGTGGTTAGGATTCG	0.28566487	0.15616	0	0.111027
tsrna-24785	GGTGGTCTAGTGGTTAGGATTC	0.07141622	0	0.069847	0.111027
tsrna-24784	GGTGGTCTAGTGGTTAGGATT	0.14283243	0.23424	0.139694	0

tsrna-24783	GGTGGTCTAGTGGTTAGGAT	0.07141622	0	0.069847	0
tsrna-24778	GGTGGTCTAGTGGCTAGGATTCGGCGCT	1.07124325	3.123204	0.419081	3.219787
tsrna-24777	GGTGGTCTAGTGGCTAGGATTCGGCGC	0.8569946	2.576644	0.628621	4.330058
tsrna-24776	GGTGGTCTAGTGGCTAGGATTCGGCG	0.49991351	2.342403	0.488928	3.441841
tsrna-24775	GGTGGTCTAGTGGCTAGGATTCGGC	0.71416216	2.108163	0.20954	1.110271
tsrna-24774	GGTGGTCTAGTGGCTAGGATTCGG	0.07141622	0.23424	0	0.111027
tsrna-24773	GGTGGTCTAGTGGCTAGGATTCG	0.21424865	0.07808	0	0
tsrna-24772	GGTGGTCTAGTGGCTAGGATT	0.14283243	0.23424	0	0
tsrna-24771	GGTGGTCTAGTGGCTAGGAT	0.07141622	0	0.069847	0.222054
tsrna-24770	GGTGGTCTAGTGGCTAGGA	0	0	0.069847	0
tsrna-24766	GGTGGTATAGTGGTTAGCATAGCTGCCT	0	0	0	0.111027
tsrna-24764	GGTGGTATAGTGGTTAGCATAGCTGC	0.07141622	0	0	0
tsrna-24763	GGTGGTATAGTGGTTAGCATAGCTG	0	0	0	0
tsrna-24755	GGTGGTATAGTGGTGAGCATAGCTGCCTTC	0.07141622	0.07808	0	0
tsrna-24754	GGTGGTATAGTGGTGAGCATAGCTGCCTT	0.07141622	0	0	0
tsrna-24753	GGTGGTATAGTGGTGAGCATAGCTGCCT	0.07141622	0.07808	0	0
tsrna-24752	GGTGGTATAGTGGTGAGCATAGCTGCC	0.07141622	0.07808	0.069847	0
tsrna-24751	GGTGGTATAGTGGTGAGCATAGCTGC	0.07141622	0.07808	0	0
tsrna-24750	GGTGGTATAGTGGTGAGCATAGCTG	0.14283243	0	0	0
tsrna-24749	GGTGGTATAGTGGTGAGCATAGCT	0.21424865	0	0	0
tsrna-24748	GGTGGTATAGTGGTGAGCATAGC	0	0.07808	0	0
tsrna-24746	GGTGGTATAGTGGTGAGCATA	0	0	0	0
tsrna-24744	GGTGGTATAGTGGTGAGCA	0	0.07808	0	0
tsrna-24741	GGTGGTATAGTGGTAAGCATAGCTGCC	0	0.07808	0	0
tsrna-24740	GGTGGTATAGTGGTAAGCATAGCTG	0.14283243	0	0	0
tsrna-24739	GGTGGTATAGTGGTAAGCATAGC	0.07141622	0	0	0
tsrna-24733	GGTGGTAGAGCATTTGACTGC	0.07141622	0.07808	0	0.111027
tsrna-24732	GGTGGTAGAGCATTTGACTG	0	0	0	0.111027
tsrna-24730	GGTGGTAGAGCATTTGA	0	0.07808	0	0
tsrna-24722	GGTGGCCCGGGTTCGACTCCCGGTATGGG	0	0.15616	0	0
tsrna-24721	GGTGGCCCGGGTTCGACTCCCGGTATGG	0	0.07808	0	0
tsrna-24716	GGTGGCACGGAGAATTTTG	0	0	0.069847	0
tsrna-24715	GGTGGAGTTAAAGACTTTTTCTCTGACCA	1.85682163	2.810884	1.257243	1.887461
tsrna-24714	GGTGGAGTTAAAGACTTTTTCTCTGACC	0.21424865	0.858881	0.279387	0.333081
tsrna-24713	GGTGGAGTTAAAGACTTTTTCTCTGAC	0.07141622	0.390401	0	0
tsrna-24712	GGTGGAGTTAAAGACTTTTTCTCTGA	0.07141622	0.31232	0.139694	0
tsrna-24711	GGTGGAGTTAAAGACTTTTTCTCTG	0	0.15616	0	0
tsrna-24710	GGTGGAGTTAAAGACTTTTTCTCT	0	0.15616	0	0
tsrna-24709	GGTGGAGTTAAAGACTTTTTCTC	0	0.15616	0	0
tsrna-24706	GGTGGAGTTAAAGACTTTTT	0	0.15616	0	0
tsrna-24705	GGTGGAGTTAAAGACTTTT	0	0.07808	0	0
tsrna-24704	GGTGGAGTTAAAGACTTT	0	0	0.069847	0
tsrna-24700	GGTGCTTAGCTGTTAACT	0	0	0.069847	0
tsrna-24698	GGTGCTAATGGTGGAGTTAAAGACTTTTTCTCTGACCA	2.49956757	3.904006	2.025557	1.998488
tsrna-24697	GGTGCTAATGGTGGAGTTAAAGACTTTTTCTCTGACC	0.35708108	2.498564	0.349234	0
tsrna-24696	GGTGCTAATGGTGGAGTTAAAGACTTTTTCTCTGAC	0.78557838	1.639682	0.069847	0.222054
tsrna-24695	GGTGCTAATGGTGGAGTTAAAGACTTTTTC	0.28566487	0.624641	0.20954	0
tsrna-24694	GGTGCTAATGGTGGAGTTAAAGACT	0.21424865	0.468481	0.139694	0.111027
tsrna-24693	GGTGCTAATGGTGGAGTTAAAGAC	0	0	0.139694	0.111027
tsrna-24692	GGTGCTAATGGTGGAGTTAAAGA	0.14283243	0.23424	0.069847	0
tsrna-24691	GGTGCTAATGGTGGAGTTAAAG	0	0.23424	0.069847	0
tsrna-24690	GGTGCTAATGGTGGAGTTAAA	0.14283243	0.23424	0.139694	0
tsrna-24689	GGTGCTAATGGTGGAGTTAA	0.14283243	0.15616	0	0
tsrna-24688	GGTGCTAATGGTGGAGTTA	0	0.15616	0.069847	0
tsrna-24687	GGTGCTAATGGTGGAGTT	0	0.07808	0	0
tsrna-24684	GGTGCGAGAGGTCCCGGGTTCAAATCCCGG	0	0.23424	0.069847	0
tsrna-24683	GGTGCGAGAGGTCCCGGGTTCAAATCCCG	0.07141622	0.31232	0	0.111027
tsrna-24682	GGTGCGAGAGGTCCCGGGTTCAAA	0	0.390401	0.069847	0
tsrna-24681	GGTGCGAGAGGTCCCGGGTTCAA	0	0.390401	0	0
tsrna-24680	GGTGCGAGAGGTCCCGGGTTCA	0	0.15616	0	0
tsrna-24679	GGTGCGAGAGGTCCCGGGTTC	0.07141622	0.23424	0	0
tsrna-24678	GGTGCGAGAGGTCCCGGGTT	0	0.31232	0	0
tsrna-24677	GGTGCGAGAGGTCCCGGGT	0	0.15616	0.069847	0
tsrna-24673	GGTGCAGTGGTAGAATTCTCGCCT	0.4284973	0.23424	0.419081	0

tsrna-24672	GGTGCAGTGGTAGAATTCTCGCC	0.07141622	0.07808	0.069847	0.111027
tsrna-24671	GGTGCAGTGGTAGAATTCTCGC	0	0.31232	0	0
tsrna-24665	GGTGCAACTCCAAATAAAAGTAC	0	0	0	0
tsrna-24659	GGTGAGTATCCCCGCCTGTCACGCGGGAG	1.07124325	3.435525	0.698468	0.444109
tsrna-24658	GGTGAGTATCCCCGCCTGTCACGCG	0.14283243	0.624641	0	0
tsrna-24657	GGTGAGTATCCCCGCCTGTCACGC	0.21424865	0.23424	0	0.111027
tsrna-24656	GGTGAGTATCCCCGCCTGTCACG	0.07141622	0.31232	0.069847	0.222054
tsrna-24655	GGTGAGTATCCCCGCCTGTCAC	0.28566487	0.31232	0.069847	0.111027
tsrna-24654	GGTGAGTATCCCCGCCTGTCA	0.07141622	0.15616	0.069847	0.111027
tsrna-24653	GGTGAGTATCCCCGCCTGTC	0.28566487	0.858881	0	0.111027
tsrna-24652	GGTGAGTATCCCCGCCTGT	0	0.23424	0	0
tsrna-24651	GGTGAGTATCCCCGCCTG	0	0.15616	0	0
tsrna-24650	GGTGAGTATCCCCGCCT	0	0	0	0.222054
tsrna-24649	GGTGAGTATCCCCGCC	0	0.15616	0	0
tsrna-24647	GGTGAGCATAGCTGCCTTCCAAG	0	0.07808	0.069847	0
tsrna-24645	GGTGAGCATAGCTGCCTTC	0	0	0.069847	0
tsrna-24642	GGTGAGCACTCTGGACTCTGAATCCAGCGATCCGAG	0.07141622	0.15616	0	0.111027
tsrna-24641	GGTGAGCACTCTGGACTCTGAATCCAGCG	0.14283243	0.31232	0	0.111027
tsrna-24640	GGTGAGCACTCTGGACTCTGAATCCAGC	0	0.468481	0.20954	0.222054
tsrna-24639	GGTGAGCACTCTGGACTCTGAATCCA	0	0.15616	0.069847	0
tsrna-24638	GGTGAGCACTCTGGACTCTGAATCC	0	0.15616	0.069847	0.111027
tsrna-24637	GGTGAGCACTCTGGACTCTGAATC	0	0	0	0
tsrna-24628	GGTCTCTGTGGCGCAATGGAC	0	0	0	0
tsrna-24627	GGTCTCGTAAACCGAAGATCAC	0	0.07808	0.069847	0
tsrna-24626	GGTCTCCGGATGGAGGCGTGG	0.07141622	0.07808	0.139694	0
tsrna-24625	GGTCTCCGGATGGAGGCG	0	0.07808	0	0
tsrna-24623	GGTCTCCAATGGAGGCGTGGGT	1.21407568	1.483522	0.419081	0
tsrna-24619	GGTCTAGTGGTTAGGATTCGGCGCTCTCAC	2.35673514	4.138246	1.396936	1.998488
tsrna-24618	GGTCTAGTGGTTAGGATTCGGCGCTCTCA	2.92806487	4.216326	1.396936	1.443353
tsrna-24617	GGTCTAGTGGTTAGGATTCGGCGCTCTC	3.42797839	3.669765	1.885864	1.332326
tsrna-24616	GGTCTAGTGGTTAGGATTCGGCGCTCT	2.21390271	3.591685	1.396936	1.776434
tsrna-24615	GGTCTAGTGGTTAGGATTCGGCGCTC	2.35673514	3.123204	1.816017	1.332326
tsrna-24614	GGTCTAGTGGTTAGGATTCGGCGCT	2.71381622	3.904006	1.327089	1.665407
tsrna-24613	GGTCTAGTGGTTAGGATTCGGCGC	1.21407568	0.936961	0.908008	1.443353
tsrna-24612	GGTCTAGTGGTTAGGATTCGGCG	0.64274595	0.858881	0.349234	0.77719
tsrna-24611	GGTCTAGTGGTTAGGATTCGGC	0.35708108	0.624641	0.20954	0.333081
tsrna-24610	GGTCTAGTGGTTAGGATTCGG	0.28566487	0.15616	0.139694	0
tsrna-24609	GGTCTAGTGGTTAGGATTCG	0	0.15616	0.069847	0
tsrna-24608	GGTCTAGTGGTTAGGATTC	0.21424865	0	0	0
tsrna-24607	GGTCTAGTGGTTAGGATT	0	0.15616	0.139694	0
tsrna-24606	GGTCTAGTGGTTAGGAT	0	0	0	0
tsrna-24604	GGTCTAGTGGCTAGGATTCGGCGCTTT	0.57132973	3.201285	0.488928	4.108004
tsrna-24603	GGTCTAGTGGCTAGGATTCGGCGCTT	1.14265946	2.576644	0.558774	4.552113
tsrna-24602	GGTCTAGTGGCTAGGATTCGGCGCT	0.71416216	4.997127	0.20954	3.441841
tsrna-24601	GGTCTAGTGGCTAGGATTCGGCGC	0.71416216	3.045124	0.279387	4.219031
tsrna-24600	GGTCTAGTGGCTAGGATTCGGCG	0.57132973	2.108163	0.139694	2.442597
tsrna-24599	GGTCTAGTGGCTAGGATTCGGC	0.14283243	0.936961	0.349234	0.444109
tsrna-24598	GGTCTAGTGGCTAGGATTCGG	0	0	0.069847	0
tsrna-24597	GGTCTAGTGGCTAGGATTCG	0.07141622	0	0	0
tsrna-24596	GGTCTAGGGGTATGATTCTCGGTTT	0.4284973	0	0.139694	0
tsrna-24595	GGTCTAGGGGTATGATTCTCGGTT	0.49991351	0	0.069847	0.222054
tsrna-24594	GGTCTAGGGGTATGATTCTCGGT	0.21424865	0.31232	0	0.333081
tsrna-24593	GGTCTAGGGGTATGATTCTCGG	0.07141622	0	0.069847	0
tsrna-24592	GGTCTAGGGGTATGATTCTCGCTTT	0.78557838	1.015041	0.698468	1.887461
tsrna-24591	GGTCTAGGGGTATGATTCTCGCTTC	1.49974054	1.171202	0.838162	1.110271
tsrna-24590	GGTCTAGGGGTATGATTCTCGCTT	1.21407568	0.780801	0.768315	1.221299
tsrna-24589	GGTCTAGGGGTATGATTCTCGCT	1.57115676	1.639682	0.698468	1.443353
tsrna-24588	GGTCTAGGGGTATGATTCTCGC	0.71416216	0.468481	0.419081	0.999244
tsrna-24587	GGTCTAGGGGTATGATTCTCG	0	0	0	0
tsrna-24586	GGTCTAGGGGTATGATTCTC	0	0	0	0.111027
tsrna-24585	GGTCTAGGGGTATGATTCT	0	0	0.069847	0
tsrna-24584	GGTCTAGGGGTATGATTC	0	0	0	0
tsrna-24581	GGTCTAGCGGTTAGGATTCCTGGTTTTC	0.57132973	0.546561	0.279387	0
tsrna-24580	GGTCTAGCGGTTAGGATTCCTGGTTTT	0.21424865	0.468481	0.20954	0.444109

tsrna-24579	GGTCTAGCGGTTAGGATTCCTGGTTT	0.28566487	0.858881	0.069847	0
tsrna-24578	GGTCTAGCGGTTAGGATTCCTGGTT	0.21424865	0.31232	0.349234	0
tsrna-24577	GGTCTAGCGGTTAGGATTCCTGGT	0.14283243	0.390401	0	0.111027
tsrna-24576	GGTCTAGCGGTTAGGATTCCTGG	0.35708108	0.23424	0.069847	0.333081
tsrna-24575	GGTCTAGCGGTTAGGATTCCTG	0	0.07808	0	0
tsrna-24567	GGTCTAAGGCGCTGGATTAAGG	0	0	0	0.111027
tsrna-24566	GGTCTAAGGCGCTGGATTAAG	0	0.07808	0	0
tsrna-24565	GGTCTAAGGCGCTGGATT	0	0	0.069847	0
tsrna-24555	GGTCGTGGTTGTAGTCCGTGCGAGAATACCA	0.71416216	2.810884	1.187396	0.999244
tsrna-24554	GGTCGTGGTTGTAGTCCGTGCGAGAATACC	0.21424865	0.468481	0.279387	0.222054
tsrna-24553	GGTCGTGGTTGTAGTCCGTGCGAGAATAC	0.07141622	0.07808	0.349234	0.111027
tsrna-24552	GGTCGTGGTTGTAGTCCGTGCGAGAATA	0.14283243	0.15616	0.139694	0
tsrna-24551	GGTCGTGGTTGTAGTCCGTGCGAGAAT	0.07141622	0.15616	0.279387	0
tsrna-24550	GGTCGTGGTTGTAGTCCGTGCGAGAA	0.07141622	0.23424	0.139694	0
tsrna-24549	GGTCGTGGTTGTAGTCCGTGCGAGA	0.14283243	0	0.069847	0
tsrna-24548	GGTCGTGGTTGTAGTCCGTGCGAG	0	0.07808	0	0
tsrna-24547	GGTCGTGGTTGTAGTCCGTGCGA	0.07141622	0.07808	0	0
tsrna-24546	GGTCGTGGTTGTAGTCCGTGCG	0	0	0	0
tsrna-24545	GGTCGTGGTTGTAGTCCGTGC	0	0	0	0
tsrna-24531	GGTCGCTGGTTCGAATCCGGCTCGAAGGAC	0.07141622	0.07808	0.069847	0
tsrna-24529	GGTCGCTGGTTCGAATCCGG	0	0.07808	0	0
tsrna-24528	GGTCGCTGGTTCGAATCC	0	0	0	0
tsrna-24527	GGTCGCGGGTTCGATCCCCGTACGGG	0	0.07808	0	0
tsrna-24515	GGTCGCGAGTTCAAATCTCGCTGGGGCCTC	0	0	0	0
tsrna-24512	GGTCGCAGTCTCCCCTGGAGGCGTGGGTTC	0.21424865	9.838094	0.20954	0
tsrna-24511	GGTCGCAGTCTCCCCTGGAGGC	0	0.390401	0.069847	0
tsrna-24510	GGTCGCAGTCTCCCCTGGAGG	0	0.07808	0	0
tsrna-24508	GGTCGATTCCCCGACGGGGAGCCA	0.21424865	1.327362	0	0
tsrna-24507	GGTCGATTCCCCGACGGGGAGCC	0	0.07808	0	0
tsrna-24501	GGTCGATGGATCGAAACCATCCTCTGCTAC	0	0	0	0
tsrna-24495	GGTCGAAACCGAGCGGAAACACCA	0	0.07808	0	0
tsrna-24489	GGTCCTGGGTTCGAGCCCCAGTGGAACCACC	0.14283243	0.390401	0.20954	0.111027
tsrna-24488	GGTCCTGGGTTCGAGCCCCAGTGGAACCAC	0	0	0.279387	0
tsrna-24487	GGTCCTGGGTTCGAGCCCCAGTGGAACCA	0	0.07808	0	0
tsrna-24485	GGTCCTGGGTTCGAGCCCCAGTGGA	0	0.07808	0	0
tsrna-24473	GGTCCGAGAGGTCCCGGGTTC	0	0	0	0.111027
tsrna-24472	GGTCCGAGAGGTCCCGGGTT	0	0.15616	0	0
tsrna-24471	GGTCCGAGAGGTCCCGGGT	0	0	0	0
tsrna-24468	GGTCCCTGGTTCGATCCCGG	0.07141622	0	0	0
tsrna-24461	GGTCCCGGGTTCAAATCCCGGACGAGCCCC	0.35708108	0.07808	0	0
tsrna-24460	GGTCCCGGGTTCAAATCCCGGACGAGCCC	0	0.07808	0	0
tsrna-24459	GGTCCCGGGTTCAAATCCCGGACGAGCC	0.07141622	0.15616	0	0
tsrna-24453	GGTCCCCGGTTCGAAACCGGGCGGAAACAC	0.21424865	0	0	0.111027
tsrna-24448	GGTCCCATGGTGTAATGGTTAGCACTCTGGACTTTG	0	0.15616	0	0.111027
tsrna-24447	GGTCCCATGGTGTAATGGTTAGCACTCTGGACT	0	0.15616	0.069847	0
tsrna-24446	GGTCCCATGGTGTAATGGTTAGCACTCTGGAC	0.07141622	0.15616	0	0
tsrna-24445	GGTCCCATGGTGTAATGGTTAGCACTCTGGA	0.07141622	0	0.069847	0.222054
tsrna-24444	GGTCCCATGGTGTAATGGTTAGCACTCTGG	0	0.23424	0	0.111027
tsrna-24443	GGTCCCATGGTGTAATGGTTAGCACTCTG	0	0.07808	0	0
tsrna-24432	GGTCCCATGGTGTAATGG	0	0.07808	0	0
tsrna-24429	GGTCCAGTGGTAGAATTCTC	0	0	0	0.111027
tsrna-24426	GGTCCAGGGTTCAAGTCCCTGTTCGGGCGC	0	0.07808	0	0
tsrna-24425	GGTCCAGGGTTCAAGTCCCTGTTCGGGCG	0	0	0	0
tsrna-24423	GGTCAGCTAAATAAGCTATCGGGCCCATAC	0	0.07808	0.279387	0.111027
tsrna-24422	GGTCAGCTAAATAAGCTATCGGGCCCA	0	0	0.139694	0
tsrna-24420	GGTCAGCTAAATAAGCTATCGGGCC	0.07141622	0	0	0
tsrna-24411	GGTCAGCACTCTGGACTCTGAATCCAGC	0.07141622	0.31232	0.069847	0
tsrna-24410	GGTCAGCACTCTGGACTCTGAATCC	0.07141622	0.23424	0	0
tsrna-24400	GGTATTAGAAAAACCATTTCATAACTTTGT	0	0	0.069847	0
tsrna-24398	GGTATGATTCTCGGTTTGGGTC	0	0.07808	0	0
tsrna-24396	GGTATGATTCTCGGTTTGGG	0	0.07808	0	0
tsrna-24395	GGTATGATTCTCGGTTTGG	0	0	0.069847	0
tsrna-24391	GGTATGATTCTCGCTTTGGGTGC	0	0	0	0.111027
tsrna-24390	GGTATGATTCTCGCTTTGGGTG	0.14283243	0	0	0

tsrna-24388	GGTATGATTCTCGCTTTG	0	0	0.139694	0.111027
tsrna-24387	GGTATGATTCTCGCTTT	0	0.07808	0	0
tsrna-24386	GGTATGATTCTCGCTTCGGGTGTG	0	0.15616	0	0
tsrna-24385	GGTATGATTCTCGCTTCGGGTGC	0	0	0	0
tsrna-24384	GGTATGATTCTCGCTTCGGGTG	0.14283243	0	0.069847	0
tsrna-24383	GGTATGATTCTCGCTTCGG	0.07141622	0.15616	0	0
tsrna-24381	GGTATGATTCTCGCTTC	0	0.07808	0	0
tsrna-24380	GGTATGATTCTCGCTT	0	0.07808	0	0
tsrna-24379	GGTATCATGCAAGATTCCCATTC	0	0	0	0
tsrna-24364	GGTATAGTGGTGAGCATAGCTGCCTTCCA	0	0.15616	0	0
tsrna-24362	GGTATAGTGGTGAGCATAGCTGCC	0	0.07808	0	0
tsrna-24360	GGTATAGTGGTGAGCATAGCTG	0	0.07808	0	0
tsrna-24356	GGTATAGTGGTAAGCATAGCTGC	0	0	0	0
tsrna-24352	GGTATAGCTCAGTGGTAGAGCATTTGACTG	0	0	0.139694	0.222054
tsrna-24351	GGTATAGCTCAGTGGTAGAGCATTTGACT	0.07141622	0.07808	0.139694	0
tsrna-24350	GGTATAGCTCAGTGGTAGAGCATTTGAC	0.14283243	0	0	0
tsrna-24349	GGTATAGCTCAGTGGTAGAGCATTTG	0.14283243	0.15616	0.069847	0
tsrna-24348	GGTATAGCTCAGTGGTAGAGCA	0	0	0	0
tsrna-24346	GGTATAGCTCAGTGGTAGAG	0	0.07808	0	0
tsrna-24344	GGTATAGCTCAGTGGGTAGAGCATTTGACT	0.07141622	0.15616	0.069847	0.111027
tsrna-24343	GGTATAGCTCAGTGGGTAGAG	0	0	0	0
tsrna-24342	GGTATAGCTCAGGGGTAGAGCATTTGACTG	0.07141622	0.07808	0.069847	0.111027
tsrna-24341	GGTATAGCTCAGGGGTAGAGCATTTGACT	0.07141622	0	0	0.111027
tsrna-24337	GGTAGTGTGGCCGAGCGGTCTAAGGCGCTGGATT	0	0.15616	0.069847	0
tsrna-24336	GGTAGTGTGGCCGAGCGGTCTAAGGCGCTG	0	0	0	0
tsrna-24335	GGTAGTGTGGCCGAGCGGTCTAAGGCGCT	0	0	0	0.111027
tsrna-24333	GGTAGTGTGGCCGAGCGGTCTAAGGC	0	0.07808	0	0
tsrna-24332	GGTAGTGTGGCCGAGCGGTCTAAGG	0.07141622	0	0	0
tsrna-24331	GGTAGTGTGGCCGAGCGGTCTAAG	0	0	0.069847	0
tsrna-24330	GGTAGTGTGGCCGAGCGGTCTAA	0	0	0	0
tsrna-24329	GGTAGTGTGGCCGAGCGGTCTA	0	0	0	0
tsrna-24328	GGTAGTGTGGCCGAGCGGTCT	0	0	0.069847	0
tsrna-24324	GGTAGTGTGGCCGAGCG	0.07141622	0	0	0
tsrna-24321	GGTAGTCGTGGCCGAGTGG	0.64274595	0.624641	0.20954	0.444109
tsrna-24310	GGTAGCGTGGCCGAGTGGTCTAAGGCGCTGGATTT	0.21424865	0.31232	0.488928	0.444109
tsrna-24309	GGTAGCGTGGCCGAGTGGTCTAAGGCGCTGGATT	0.14283243	0.546561	0.139694	0.222054
tsrna-24308	GGTAGCGTGGCCGAGTGGTCTAAGGCGCTG	0.21424865	0.31232	0.20954	0
tsrna-24307	GGTAGCGTGGCCGAGTGGTCTAAGGCGCT	0.21424865	0.31232	0.069847	0.111027
tsrna-24306	GGTAGCGTGGCCGAGTGGTCTAAGGCGC	0.14283243	0.31232	0.279387	0.111027
tsrna-24305	GGTAGCGTGGCCGAGTGGTCTAAGGCG	0.14283243	0.468481	0	0.111027
tsrna-24304	GGTAGCGTGGCCGAGTGGTCTAAGGC	0.07141622	0.31232	0.279387	0
tsrna-24303	GGTAGCGTGGCCGAGTGGTCTAAGG	0.07141622	0.390401	0.139694	0.111027
tsrna-24302	GGTAGCGTGGCCGAGTGGTCTAAGAC	0.28566487	0.546561	0.139694	0
tsrna-24301	GGTAGCGTGGCCGAGTGGTCTAAG	0.07141622	0.546561	0.139694	0
tsrna-24300	GGTAGCGTGGCCGAGTGGTCTAA	0.07141622	0.15616	0	0.333081
tsrna-24299	GGTAGCGTGGCCGAGTGGTCTA	0.07141622	0.546561	0.069847	0.333081
tsrna-24298	GGTAGCGTGGCCGAGTGGTCT	0.07141622	0.31232	0.139694	0.444109
tsrna-24297	GGTAGCGTGGCCGAGTGGTC	0.14283243	0.468481	0.069847	0.333081
tsrna-24296	GGTAGCGTGGCCGAGTGGT	0.07141622	0.390401	0.139694	0.111027
tsrna-24295	GGTAGCGTGGCCGAGTGG	0.14283243	0.546561	0.279387	0.111027
tsrna-24294	GGTAGCGTGGCCGAGTG	0.28566487	0.31232	0.069847	0.111027
tsrna-24293	GGTAGCGTGGCCGAGT	0.07141622	0.07808	0	0
tsrna-24292	GGTAGCGTGGCCGAGCGGTCTAAGGCGCTGGATT	0.4284973	0.780801	0.279387	0.555136
tsrna-24291	GGTAGCGTGGCCGAGCGGTCTAAGGCGCTGGAT	0	0.624641	0.20954	0.666163
tsrna-24290	GGTAGCGTGGCCGAGCGGTCTAAGGCGCTG	0.28566487	0.15616	0	0.222054
tsrna-24289	GGTAGCGTGGCCGAGCGGTCTAAGGCGCT	0.14283243	0.31232	0	0.222054
tsrna-24288	GGTAGCGTGGCCGAGCGGTCTAAGGCGC	0.21424865	0.390401	0.139694	0.111027
tsrna-24287	GGTAGCGTGGCCGAGCGGTCTAAGGCG	0	0	0.279387	0.444109
tsrna-24286	GGTAGCGTGGCCGAGCGGTCTAAGGC	0.21424865	0.624641	0	0.111027
tsrna-24285	GGTAGCGTGGCCGAGCGGTCTAAGG	0.07141622	0.624641	0	0.111027
tsrna-24284	GGTAGCGTGGCCGAGCGGTCTAAG	0.07141622	0.390401	0.069847	0.333081
tsrna-24283	GGTAGCGTGGCCGAGCGGTCTAA	0.14283243	0.23424	0	0.333081
tsrna-24282	GGTAGCGTGGCCGAGCGGTCTA	0.14283243	0.15616	0.139694	0.222054
tsrna-24281	GGTAGCGTGGCCGAGCGGTCT	0.14283243	0.23424	0.069847	0.444109

tsrna-24280	GGTAGCGTGGCCGAGCGGTC	0.14283243	0.390401	0	0.111027
tsrna-24279	GGTAGCGTGGCCGAGCGGT	0.07141622	0.07808	0.139694	0
tsrna-24278	GGTAGCGTGGCCGAGCGG	0	0	0	0
tsrna-24277	GGTAGCGTGGCCGAGCG	0	0	0.069847	0
tsrna-24276	GGTAGCGTGGCCGAGC	0	0	0	0
tsrna-24275	GGTAGCGGGATCGATGCCCGCATCCTCCAC	0.07141622	0	0.069847	0
tsrna-24274	GGTAGCGGGATCGATGCC	0	0	0.069847	0
tsrna-24272	GGTAGCGCGTCTGACTCC	0.07141622	0.07808	0	0
tsrna-24264	GGTAGAGCATTTGACTGCAGATC	0	0	0	0
tsrna-24261	GGTAGAGCATTTGACTG	0	0	0	0
tsrna-24259	GGTAGAGCATGGGACTCTTAATCCC	0.35708108	0.390401	0.349234	0.444109
tsrna-24258	GGTAGAGCATGGGACTCTTAATCC	0.28566487	0.624641	0.279387	0.222054
tsrna-24257	GGTAGAGCATGGGACTCT	0.35708108	0.07808	0	0
tsrna-24256	GGTAGAGCATGGGACTC	0.07141622	0	0	0
tsrna-24255	GGTAGAGCATGGGACT	0.07141622	0.31232	0.069847	0.333081
tsrna-24254	GGTAGAGCATGAGACTCTTAATCT	0.14283243	0.624641	0.069847	0.222054
tsrna-24253	GGTAGAGCATGAGACTCTTA	0.07141622	0.23424	0.139694	0.111027
tsrna-24252	GGTAGAGCATGAGACTC	0.07141622	0.390401	0	0
tsrna-24251	GGTAGAGCATGAGACT	0.14283243	0.390401	0	0.111027
tsrna-24250	GGTAGAGCATCAGACTTTTAATCTGAGGGTCCAGGGT	0.78557838	2.342403	0.419081	0.111027
tsrna-24249	GGTAGAGCATCAGACTTTTAATC	0.07141622	0	0	0
tsrna-24244	GGTAGAATTCTCGCCTGCCACGCGGGAGGCCCGGGTT	4.42780542	6.012168	2.793872	1.443353
tsrna-24243	GGTAGAATTCTCGCCTGCCACGCGGGAGGCCCGGGT	3.64222704	6.090249	1.74617	1.221299
tsrna-24242	GGTAGAATTCTCGCCTGCCACGCGGGAGGCCC	2.85664866	3.045124	1.396936	1.110271
tsrna-24241	GGTAGAATTCTCGCCTGCCACGCGGGAGGC	2.57098379	3.591685	1.466783	1.443353
tsrna-24240	GGTAGAATTCTCGCCTGCCACGCGGGAG	3.9278919	2.498564	1.466783	1.110271
tsrna-24239	GGTAGAATTCTCGCCTGCCACGCGGGA	3.07089731	3.357445	1.327089	1.998488
tsrna-24238	GGTAGAATTCTCGCCTGCCACGCGG	1.85682163	3.201285	1.606477	2.109516
tsrna-24237	GGTAGAATTCTCGCCTGCCACGC	2.28531893	3.201285	1.466783	1.55438
tsrna-24236	GGTAGAATTCTCGCCTGCCACG	2.49956757	1.795843	1.257243	0.999244
tsrna-24235	GGTAGAATTCTCGCCTGCCAC	0.64274595	0.390401	0.279387	0.222054
tsrna-24234	GGTAGAATTCTCGCCTGCCA	0.28566487	0.31232	0	0
tsrna-24233	GGTAGAATTCTCGCCTGCC	0.21424865	0.468481	0.20954	0.222054
tsrna-24232	GGTAGAATTCTCGCCTGC	0.07141622	0.23424	0.069847	0
tsrna-24231	GGTAGAATTCTCGCCTG	0.07141622	0.15616	0	0
tsrna-24230	GGTAGAATTCTCGCCTCCCACGCGGGAGACCCGGGTT	0.14283243	0.07808	0.20954	0
tsrna-24229	GGTAGAATTCTCGCCTCCCACGCGGGAGACCCGGGT	0.07141622	0.31232	0	0
tsrna-24228	GGTAGAATTCTCGCCTCCCACG	0.14283243	0.15616	0.069847	0
tsrna-24227	GGTAGAATTCTCGCCTCCCA	0	0.23424	0	0.333081
tsrna-24226	GGTAGAATTCTCGCCTCCC	0	0.07808	0.069847	0
tsrna-24225	GGTAGAATTCTCGCCTCC	0.28566487	0.15616	0	0.222054
tsrna-24224	GGTAGAATTCTCGCCTC	0	0	0	0
tsrna-24223	GGTAGAATTCTCGCCT	0.14283243	0.23424	0.069847	0.111027
tsrna-24222	GGTAATCGCATAAAACTTAAAACTTTACAG	0.14283243	0.07808	0.419081	0
tsrna-24221	GGTAATCGCATAAAACTTAAAACTTTACA	0.28566487	0.15616	0.279387	0
tsrna-24220	GGTAATCGCATAAAACTTAAAACTTTA	0.21424865	0.31232	0.488928	0
tsrna-24219	GGTAATCGCATAAAACTTAAAACTTT	0.07141622	0.31232	0.349234	0
tsrna-24218	GGTAATCGCATAAAACTTAAAACTT	0	0	0.069847	0
tsrna-24216	GGTAATCGCATAAAACTTAAAAC	0	0	0	0
tsrna-24203	GGTAAGCACTCTGGACTCTGAATCCAGC	0	0.31232	0.069847	0.222054
tsrna-24202	GGTAAGCACTCTGGACTCTGAATCCA	0	0	0	0
tsrna-24201	GGTAAGCACTCTGGACTCTGAATCC	0.14283243	0.15616	0	0
tsrna-24191	GGTAAATATAGTTTAACCAAAACATCAGATTGTGAATCTGACA	0	0.31232	0.139694	0
tsrna-24190	GGTAAATATAGTTTAACCAAAACATCAGATTGTGA	0.07141622	0	0.20954	0.111027
tsrna-24189	GGTAAATATAGTTTAACCAAAACATCAGATTGT	0	0	0.069847	0
tsrna-24188	GGTAAATATAGTTTAACCAAAACATCAGAT	0.07141622	0	0	0
tsrna-24187	GGTAAATATAGTTTAACCAAAACATCAG	0.07141622	0	0	0.111027
tsrna-24184	GGTAAAATGGCTGAGTGAAGCATTGGACTGTAAATCT	0.8569946	0.702721	1.466783	0.888217
tsrna-24183	GGTAAAATGGCTGAGTGAAGCATTGGACTGTAAATC	1.21407568	0.702721	1.466783	0.666163
tsrna-24182	GGTAAAATGGCTGAGTGAAGCATTGGACTGTAAA	1.21407568	1.015041	1.117549	1.55438
tsrna-24181	GGTAAAATGGCTGAGTGAAGCATTGGACTGTAA	1.42832433	0.546561	0.908008	1.665407
tsrna-24180	GGTAAAATGGCTGAGTGAAGCATTGGACTGTA	0.71416216	0.936961	0.558774	1.110271
tsrna-24179	GGTAAAATGGCTGAGTGAAGCATTGGACTGT	0.14283243	0.468481	0.139694	0.222054
tsrna-24178	GGTAAAATGGCTGAGTGAAGCATTGGACTG	0.07141622	0.07808	0	0

tsrna-24177	GGTAAAATGGCTGAGTGAAGCATTGGACT	0	0.07808	0	0.111027
tsrna-24176	GGTAAAATGGCTGAGTGAAGCATTGGAC	0.07141622	0.07808	0	0.111027
tsrna-24175	GGTAAAATGGCTGAGTGAAGCATTGGA	0	0.31232	0	0
tsrna-24174	GGTAAAATGGCTGAGTGAAGCATTGG	0	0.15616	0	0
tsrna-24173	GGTAAAATGGCTGAGTGAAGCATTG	0	0.15616	0	0
tsrna-24172	GGTAAAATGGCTGAGTGAAGCATT	0	0.07808	0	0
tsrna-24171	GGTAAAATGGCTGAGTGAAGCAT	0.14283243	0	0	0
tsrna-24170	GGTAAAATGGCTGAGTGAAGCA	0	0	0	0.111027
tsrna-24169	GGTAAAATGGCTGAGTGAAGC	0	0.07808	0	0
tsrna-24168	GGTAAAATGGCTGAGTGAAG	0	0	0	0
tsrna-24167	GGTAAAATGGCTGAGTGAA	0	0.15616	0	0
tsrna-24166	GGTAAAATGGCTGAGTGA	0	0	0	0
tsrna-24165	GGTAAAATGGCTGAGTG	0	0	0	0
tsrna-24163	GGGTTTTGCAGTCCTTACCA	0	0.23424	0	0
tsrna-24161	GGGTTTTGCAGTCCTTAC	0	0	0.069847	0
tsrna-24160	GGGTTTTGCAGTCCTTA	0	0.07808	0.069847	0
tsrna-24159	GGGTTTTGCAGTCCTT	0	0.07808	0.069847	0
tsrna-24158	GGGTTTCCCCGCGCAGGTTCG	0	0	0	0
tsrna-24154	GGGTTTCCCCGCACAGGTT	0.14283243	0	0	0
tsrna-24153	GGGTTTCCCCGCACAGGT	0.07141622	0	0	0
tsrna-24152	GGGTTTAAGTCCCATTGGTCTAGCCA	0.14283243	0.23424	0	0
tsrna-24151	GGGTTTAAGTCCCATTGGTCTAGCC	0	0.07808	0	0
tsrna-24150	GGGTTTAAGTCCCATTGGTCTAGC	0.07141622	0	0	0
tsrna-24149	GGGTTTAAGTCCCATTGGTCTAG	0	0.07808	0	0
tsrna-24148	GGGTTTAAGTCCCATTGGTCTA	0.07141622	0.07808	0.069847	0
tsrna-24147	GGGTTTAAGTCCCATTGGTCT	0	0.07808	0	0
tsrna-24144	GGGTTTAAGTCCCATTG	0	0.15616	0	0
tsrna-24142	GGGTTCGATTCTCATAGTCCTAGCCA	0.07141622	0.15616	0	0.111027
tsrna-24141	GGGTTCGATTCTCATAGTCCTAGCC	0.07141622	0.15616	0	0.111027
tsrna-24140	GGGTTCGATTCTCATAGTCCTAGC	0.07141622	0.390401	0	0
tsrna-24139	GGGTTCGATTCTCATAGTCCTAG	0	0.15616	0	0
tsrna-24138	GGGTTCGATTCTCATAGTCCT	0	0.23424	0.069847	0
tsrna-24137	GGGTTCGATTCTCATAGTCC	0.07141622	0.15616	0.069847	0
tsrna-24136	GGGTTCGATTCTCATAGTC	0	0.15616	0	0
tsrna-24134	GGGTTCGATTCCTTCCTTTTTTGCCA	0.07141622	0.15616	0.069847	0.222054
tsrna-24133	GGGTTCGATTCCTTCCTTTTTTGCC	0	0.31232	0.20954	0
tsrna-24132	GGGTTCGATTCCTTCCTTTTTTGC	0.07141622	0.07808	0	0
tsrna-24130	GGGTTCGATTCCCGGTCAGGGAACCA	0.49991351	22.79939	0.279387	0.333081
tsrna-24129	GGGTTCGATTCCCGGTCAGGGAACC	0	0.858881	0.069847	0
tsrna-24128	GGGTTCGATTCCCGGTCAGGGAAC	0.07141622	0.15616	0.069847	0
tsrna-24127	GGGTTCGATTCCCGGTCAGGGAA	0	0.07808	0	0
tsrna-24126	GGGTTCGATTCCCGGTCAGGGA	0	0.07808	0	0
tsrna-24124	GGGTTCGATTCCCGGTCAGG	0	0.07808	0	0
tsrna-24121	GGGTTCGATTCCCGGGCGGCGCACCA	0.64274595	4.840967	0.349234	0.333081
tsrna-24120	GGGTTCGATTCCCGGCCCATGCACCA	1.14265946	2.498564	0.419081	1.332326
tsrna-24119	GGGTTCGATTCCCGGCCAATGCACCA	3.21372974	13.11746	1.187396	1.443353
tsrna-24118	GGGTTCGATTCCCCGACGGGGAGCCA	0.14283243	1.639682	0.139694	0.111027
tsrna-24110	GGGTTCGATCCCCGGCATCTCCACCA	16.0686487	29.98276	9.778553	8.882171
tsrna-24109	GGGTTCGATCCCCGGCATCTCCACC	1.14265946	1.952003	0.349234	0.444109
tsrna-24108	GGGTTCGATCCCCGGCACCTCCACCA	9.92685408	23.89251	6.495753	5.995465
tsrna-24106	GGGTTCGATCCCCAGTACCTCCACCA	1.71398919	5.465608	1.187396	0.999244
tsrna-24105	GGGTTCGATCCCCAGCATCTCCACCA	0.64274595	1.015041	0	0.444109
tsrna-24104	GGGTTCGATCCCCAGCATCTCCACC	0.14283243	0.07808	0	0
tsrna-24100	GGGTTCGAGTCCCTTCGTGGTCGCCA	0	0.390401	0	0
tsrna-24098	GGGTTCGAGTCCCTTCGTGGTCGC	0.07141622	0	0	0
tsrna-24095	GGGTTCGAGTCCCATCTGGGTCGCCA	0.14283243	1.249282	0.279387	0
tsrna-24094	GGGTTCGAGTCCCACCAGAGTCGCCA	0.14283243	0.15616	0	0.111027
tsrna-24091	GGGTTCGAGCCCCAGTGGAACCACCA	3.21372974	1.483522	1.676323	1.443353
tsrna-24090	GGGTTCGAGCCCCAGTGGAACCACC	0.35708108	0.07808	0	0.222054
tsrna-24089	GGGTTCGAGCCCCAGTGGAACCAC	0	0	0.069847	0
tsrna-24088	GGGTTCGAGCCCCAGTGGAACCA	0	0.07808	0	0
tsrna-24087	GGGTTCGAGCCCCAGTGGAACC	0.07141622	0	0.069847	0
tsrna-24085	GGGTTCGAGCCCCACGTTGGGCGCCA	0.99982703	14.83522	0.488928	0.111027
tsrna-24084	GGGTTCGAGCCCCACGTTGGGCGCC	0	0.546561	0	0

tsrna-24081	GGGTTCGACTCCCGGTGTGGGAACCA	4.49922163	21.00355	1.885864	2.220543
tsrna-24080	GGGTTCGACTCCCGGTGTGGGAACC	0.4284973	1.483522	0	0
tsrna-24079	GGGTTCGACTCCCGGTGTGGGAAC	0	0.07808	0	0
tsrna-24078	GGGTTCGACTCCCGGTGTGGGAA	0	0	0.069847	0
tsrna-24077	GGGTTCGACTCCCGGTGTGGGA	0	0	0.069847	0
tsrna-24076	GGGTTCGACTCCCGGTGTGGG	0	0	0.069847	0
tsrna-24071	GGGTTCGACTCCCGGTATGGGAACCA	0.57132973	6.636809	0.279387	0.333081
tsrna-24070	GGGTTCGACTCCCGGTATGGGAAC	0	0.15616	0	0
tsrna-24068	GGGTTCGACTCCCAGCGGGGCCTCCA	0	0	0	0.111027
tsrna-24066	GGGTTCGAATCCCTTCGTGGTTGCCA	0	0.31232	0	0
tsrna-24064	GGGTTCGAATCCCTTCGTGGTTGC	0	0.07808	0	0
tsrna-24062	GGGTTCGAATCCCATCCTCGTCGCCA	0.35708108	1.015041	0.20954	0
tsrna-24061	GGGTTCGAATCCCATCCTCGTCGCC	0	0.07808	0	0
tsrna-24059	GGGTTCGAATCCCATCCTCGTCG	0	0	0	0
tsrna-24058	GGGTTCGAATCCCAGCGGTGCCTCCA	2.07107028	3.201285	0.349234	0.333081
tsrna-24056	GGGTTCGAATCCCAGCGGGGCCTCCA	0.14283243	0.31232	0.069847	0.111027
tsrna-24055	GGGTTCGAATCCCACTTCTGACACCA	1.14265946	1.873923	0.698468	0.999244
tsrna-24054	GGGTTCGAATCCCACTTCTGACACC	0	0.15616	0.069847	0.111027
tsrna-24053	GGGTTCGAATCCCACTTCTGACAC	0	0.07808	0	0
tsrna-24052	GGGTTCGAATCCCACTCCTGACACCA	7.35587029	8.042251	3.562187	3.219787
tsrna-24051	GGGTTCGAATCCCACTCCTGACAC	0.07141622	0	0	0
tsrna-24050	GGGTTCGAATCCCACCGCTGCCACCA	1.2854919	2.810884	0.069847	0
tsrna-24048	GGGTTCGAATCCCACCAGAGTCGCCA	0.21424865	0.702721	0	0
tsrna-24045	GGGTTCAATTCCCCGACGGGGAGCCA	0	0.23424	0	0
tsrna-24044	GGGTTCAATTCCCCGACGGGGAGCC	0.07141622	0.07808	0	0
tsrna-24042	GGGTTCAATCCCCGGCATCTCCACCA	8.99844327	12.5709	5.517898	5.551357
tsrna-24041	GGGTTCAATCCCCGGCACCTCCACCA	15.6401514	26.23492	7.962536	5.884438
tsrna-24040	GGGTTCAATCCCCGGCACCTCCAC	0	0	0	0.111027
tsrna-24039	GGGTTCAATCCCCGGCACCTCCA	0.07141622	0	0	0
tsrna-24038	GGGTTCAAGTCCCTGTTCGGGCGCCA	0.49991351	0.31232	0.279387	0.333081
tsrna-24033	GGGTTCAAGTCCCTGTCCAGGCGCCA	0	0.23424	0	0
tsrna-24027	GGGTTAGGCCTCTTTTTACCACCA	0.14283243	0.07808	0.20954	0.111027
tsrna-24025	GGGTTAGGCCTCTTTTTACCAC	0	0	0	0
tsrna-24024	GGGTTAGGCCTCTTTTTACCA	0	0.07808	0	0.111027
tsrna-24023	GGGTTAGGCCTCTTTTTACC	0	0	0	0
tsrna-24022	GGGTTAGGCCTCTTTTTAC	0	0	0.069847	0
tsrna-24021	GGGTTAGGCCTCTTTTTA	0	0	0	0
tsrna-24018	GGGTGTGATAGGTGGCACGGAGAATTTTG	0.07141622	0.07808	0	0
tsrna-24017	GGGTGTGATAGGTGGCACGGAGAA	0	0.07808	0	0
tsrna-24014	GGGTGTGATAGGTGGCACGGA	0	0	0	0
tsrna-24007	GGGTGTAGCTCAGTGGTAGAGCGCGTGC	0.49991351	0.936961	0.139694	0
tsrna-24006	GGGTGTAGCTCAGTGGTAGAGC	0	0	0	0
tsrna-24005	GGGTGTAGCTCAGTGGTAGAG	0	0	0	0
tsrna-24003	GGGTGTAGCTCAGTGGTAG	0	0	0	0
tsrna-23999	GGGTGGTTCAGTGGTAGAATTCTCGCCTGC	0.57132973	0.936961	0.488928	0.222054
tsrna-23998	GGGTGGTTCAGTGGTAGAATTCTCGCCT	0.4284973	0.858881	0.20954	0.333081
tsrna-23997	GGGTGGTTCAGTGGTAGAATTCTCGCC	0.4284973	0.468481	0.139694	0.222054
tsrna-23996	GGGTGGTTCAGTGGTAGAATTCTCGC	0.28566487	0.07808	0.069847	0.111027
tsrna-23995	GGGTGGTTCAGTGGTAGAATTCTCG	0.14283243	0.15616	0.139694	0.111027
tsrna-23994	GGGTGGTTCAGTGGTAGAATTCTC	0.21424865	0.15616	0.139694	0
tsrna-23993	GGGTGGTTCAGTGGTAGAATTCT	0	0.07808	0	0
tsrna-23992	GGGTGGTTCAGTGGTAGAA	0.07141622	0	0	0
tsrna-23989	GGGTGCTTAGCTGTTAACTAAG	0.07141622	0	0	0
tsrna-23988	GGGTGCTTAGCTGTTAACTAA	0	0	0	0
tsrna-23982	GGGTGCTAATGGTGGAGTTAAAGACTTTTTCTCTGACCA	1.64257298	2.576644	1.396936	2.553624
tsrna-23981	GGGTGCTAATGGTGGAGTTAAAGACTTTTT	0.35708108	0.546561	0.349234	0
tsrna-23980	GGGTGCTAATGGTGGAGTTAAAGACT	0	0.23424	0.20954	0
tsrna-23979	GGGTGCTAATGGTGGAGTTAAAGAC	0	0.07808	0.069847	0.222054
tsrna-23978	GGGTGCTAATGGTGGAGTTAAAGA	0.07141622	0.31232	0.069847	0
tsrna-23977	GGGTGCTAATGGTGGAGTTAAAG	0	0.546561	0.139694	0
tsrna-23976	GGGTGCTAATGGTGGAGTTAAA	0.07141622	0.15616	0.069847	0
tsrna-23975	GGGTGCTAATGGTGGAGTTAA	0.14283243	0.23424	0.069847	0
tsrna-23974	GGGTGCTAATGGTGGAGTTA	0.07141622	0.23424	0	0
tsrna-23973	GGGTGCTAATGGTGGAGTT	0.07141622	0	0	0

tsrna-23970	GGGTGCGAGAGGTCCCGGGTTCAAATCCCG	0	0	0.069847	0
tsrna-23969	GGGTGCGAGAGGTCCCGGGTTC	0	0.07808	0	0
tsrna-23968	GGGTGCGAGAGGTCCCGGGTT	0.07141622	0.31232	0.069847	0
tsrna-23967	GGGTGCGAGAGGTCCCGGGT	0.07141622	0.07808	0	0
tsrna-23963	GGGTGCGAGAGGTCCC	0	0	0	0
tsrna-23962	GGGTCTCCCCGCGCAGGT	0	0.23424	0	0
tsrna-23956	GGGTCGATTCCCCGACGGGGAGCCA	0.07141622	0.702721	0.069847	0
tsrna-23954	GGGTCGATTCCCCGACGGGGAGC	0	0.07808	0	0
tsrna-23953	GGGTCGATTCCCCGACGGGGAG	0	0.07808	0	0
tsrna-23946	GGGTCCGAGAGGTCCCGGGTTCAAATCCCG	0.07141622	0	0.069847	0
tsrna-23945	GGGTCCGAGAGGTCCCGGGTTC	0.07141622	0.07808	0.069847	0
tsrna-23944	GGGTCCGAGAGGTCCCGGGTT	0	0.07808	0	0
tsrna-23943	GGGTCCGAGAGGTCCCGGGT	0	0.07808	0	0
tsrna-23934	GGGTCCAGGGTTCAAGTCCCTGTTCGGGCG	0	0	0	0
tsrna-23929	GGGTATGATTCTCGGTTTGGG	0	0	0	0
tsrna-23927	GGGTATGATTCTCGGTTTG	0.07141622	0	0	0
tsrna-23923	GGGTATGATTCTCGCTTTGGGTG	0.14283243	0	0	0
tsrna-23922	GGGTATGATTCTCGCTTTGGG	0.21424865	0.07808	0	0.111027
tsrna-23921	GGGTATGATTCTCGCTTTGG	0.07141622	0.07808	0.139694	0
tsrna-23920	GGGTATGATTCTCGCTTTG	0	0.15616	0	0.222054
tsrna-23919	GGGTATGATTCTCGCTTT	0	0.23424	0	0
tsrna-23918	GGGTATGATTCTCGCTTCGG	0.07141622	0.23424	0.139694	0
tsrna-23917	GGGTATGATTCTCGCTTCG	0.07141622	0	0.069847	0
tsrna-23916	GGGTATGATTCTCGCTTC	0	0.23424	0	0
tsrna-23914	GGGTATGATTCTCGCT	0	0	0	0.111027
tsrna-23913	GGGTATAGCTCAGTGGTAGAGCGCGTGC	0.8569946	2.030083	0.139694	0
tsrna-23912	GGGTATAGCTCAGTGGTAGAGCATTTGACT	0.28566487	0.15616	0	0
tsrna-23910	GGGTATAGCTCAGTGGTAGAG	0	0	0.069847	0.111027
tsrna-23909	GGGTATAGCTCAGTGGTAGA	0.07141622	0.15616	0	0
tsrna-23907	GGGTATAGCTCAGTGGGTAGAGCATTTGAC	0	0	0.069847	0
tsrna-23906	GGGTATAGCTCAGTGG	0	0	0	0
tsrna-23905	GGGTATAGCTCAGGGGTAGAGCATTTGACT	0	0.624641	0.069847	0
tsrna-23904	GGGTATAGCTCAGGGGTAGAGCA	0	0	0	0.222054
tsrna-23903	GGGTATAGCTCAGGGGTAGAGC	0	0	0	0.222054
tsrna-23902	GGGTATAGCTCAGGGGTAGAG	0	0.23424	0.069847	0
tsrna-23901	GGGTATAGCTCAGGGGTAGA	0	0	0	0
tsrna-23900	GGGTATAGCTCAGGGGTAG	0.21424865	0.07808	0.139694	0.111027
tsrna-23899	GGGTATAGCTCAGGGG	0	0.07808	0.069847	0.333081
tsrna-23898	GGGTATAGCTCAGCGGTAGAGCGCGTGC	0	0.702721	0	0.111027
tsrna-23896	GGGTAGCGTGGCCGAGCGGTCT	0	0.390401	0	0.222054
tsrna-23895	GGGTAGCGTGGCCGAGCGGTC	0	0.390401	0.069847	0.333081
tsrna-23894	GGGTAGCGTGGCCGAGCGGT	0	0.31232	0.069847	0.333081
tsrna-23893	GGGTAGCGTGGCCGAGCGG	0.07141622	0	0.139694	0.111027
tsrna-23890	GGGTAGAGCATTTGACTGC	0.07141622	0	0	0
tsrna-23887	GGGGTTTTGCAGTCCTTACCA	0.07141622	0.07808	0	0.111027
tsrna-23886	GGGGTTTTGCAGTCCTTACC	0	0.07808	0.069847	0
tsrna-23885	GGGGTTTTGCAGTCCTTAC	0	0	0	0
tsrna-23883	GGGGTTTTGCAGTCCTT	0	0.07808	0	0
tsrna-23882	GGGGTTCGATTCCTTCCTTTTTTGCCA	0.21424865	0.07808	0	0.111027
tsrna-23881	GGGGTTCGATTCCTTCCTTTTTTGCC	0	0.390401	0	0
tsrna-23880	GGGGTTCGATTCCTTCCTTTTTTGC	0.07141622	0.15616	0	0
tsrna-23879	GGGGTTCGATTCCTTCCTTT	0	0.07808	0	0
tsrna-23877	GGGGTTCGATTCCCCGACGGGGAGCCA	0	1.483522	0	0.111027
tsrna-23874	GGGGTTCGATTCCCCGACGGGGAG	0.07141622	0.07808	0	0
tsrna-23873	GGGGTTCGATTCCCCGACGGGGA	0	0.07808	0	0
tsrna-23869	GGGGTTCAATTCCCCGACGGGGAGCCA	0	0.23424	0	0
tsrna-23867	GGGGTTCAATTCCCCGACGGGGAGC	0	0.07808	0	0
tsrna-23866	GGGGTTAGGCCTCTTTTTACCACCA	0.21424865	0.07808	0.279387	0.111027
tsrna-23865	GGGGTTAGGCCTCTTTTTACCACC	0	0	0.069847	0
tsrna-23864	GGGGTTAGGCCTCTTTTTACCAC	0	0	0.139694	0
tsrna-23863	GGGGTTAGGCCTCTTTTTACCA	0.07141622	0	0	0
tsrna-23862	GGGGTTAGGCCTCTTTTTACC	0.07141622	0	0	0
tsrna-23861	GGGGTTAGGCCTCTTTTTAC	0	0	0	0
tsrna-23859	GGGGTTAGGCCTCTTTTT	0	0	0	0

tsrna-23858	GGGGTTAGGCCTCTTTT	0	0	0	0
tsrna-23857	GGGGTTAGGCCTCTTT	0	0.07808	0	0
tsrna-23856	GGGGTTAGAGCACTGGT	0	0	0.069847	0
tsrna-23852	GGGGTGTGATAGGTGGCACGGA	0	0	0	0
tsrna-23846	GGGGTGTAGCTCAGTGGTAGAGCGCGTGCT	0.57132973	2.108163	0.139694	0
tsrna-23845	GGGGTGTAGCTCAGTGGTAGAGCGCGTGC	0.28566487	2.186243	0.069847	0
tsrna-23844	GGGGTGTAGCTCAGTGGTAGAGCGCGTG	0.07141622	0	0	0
tsrna-23842	GGGGTGTAGCTCAGTGGTAGAGCATTTGAC	0.07141622	0	0	0
tsrna-23839	GGGGTGTAGCTCAGTGGTAGA	0.07141622	0	0	0
tsrna-23838	GGGGTGTAGCTCAGTGGTAG	0	0	0	0
tsrna-23832	GGGGTCGATTCCCCGACGGGGAGCCA	0	1.015041	0	0
tsrna-23831	GGGGTCGATTCCCCGACGGGGAGCC	0	0	0	0
tsrna-23829	GGGGTATGATTCTCGGTTTGG	0.07141622	0	0	0
tsrna-23828	GGGGTATGATTCTCGGTTTG	0.07141622	0.07808	0	0
tsrna-23827	GGGGTATGATTCTCGGTTT	0.07141622	0	0	0
tsrna-23823	GGGGTATGATTCTCGCTTTGGGTG	0.07141622	0.15616	0.069847	0.111027
tsrna-23822	GGGGTATGATTCTCGCTTTGG	0	0	0	0
tsrna-23821	GGGGTATGATTCTCGCTTTG	0.07141622	0.07808	0.139694	0
tsrna-23820	GGGGTATGATTCTCGCTTT	0.07141622	0.07808	0.20954	0
tsrna-23819	GGGGTATGATTCTCGCTTCGG	0.14283243	0	0.139694	0
tsrna-23818	GGGGTATGATTCTCGCTTCG	0	0	0	0.111027
tsrna-23817	GGGGTATGATTCTCGCTTC	0	0.07808	0.069847	0
tsrna-23816	GGGGTATGATTCTCGCTTA	0.07141622	0.07808	0.139694	0.111027
tsrna-23815	GGGGTATGATTCTCGCTT	0.07141622	0.07808	0.139694	0.111027
tsrna-23814	GGGGTATGATTCTCGCT	0.14283243	0.15616	0	0.111027
tsrna-23813	GGGGTATGATTCTCGC	0.14283243	0.15616	0.069847	0
tsrna-23812	GGGGTATAGCTCAGTGGTAGAGCATTTGACT	0.14283243	0.390401	0	0.111027
tsrna-23811	GGGGTATAGCTCAGTGGTAGAGCATTTGAC	0.07141622	0	0	0.111027
tsrna-23810	GGGGTATAGCTCAGTGGTAGAGCATTTGA	0.14283243	0.15616	0	0
tsrna-23809	GGGGTATAGCTCAGTGGTAGAGCAT	0.07141622	0.07808	0	0
tsrna-23808	GGGGTATAGCTCAGTGGTAGAGCA	0	0.07808	0	0.111027
tsrna-23807	GGGGTATAGCTCAGTGGTAGAGC	0.07141622	0.15616	0	0
tsrna-23806	GGGGTATAGCTCAGTGGTAGAG	0	0	0	0.222054
tsrna-23805	GGGGTATAGCTCAGTGGTAGA	0	0.07808	0.139694	0
tsrna-23804	GGGGTATAGCTCAGTGGTAG	0.07141622	0.07808	0	0.111027
tsrna-23803	GGGGTATAGCTCAGTGGT	0	0.07808	0	0
tsrna-23802	GGGGTATAGCTCAGTGGGTAGAGCATTTG	0	0.07808	0	0
tsrna-23800	GGGGTATAGCTCAGTGGGTAGAGC	0.07141622	0	0	0
tsrna-23797	GGGGTATAGCTCAGGTGGTAGAGCATTTG	0	0	0.069847	0.111027
tsrna-23796	GGGGTATAGCTCAGGGGTAGAGCATTTGAC	0	0	0.069847	0.111027
tsrna-23795	GGGGTATAGCTCAGGGGTAGAGCATT	0.07141622	0	0	0
tsrna-23794	GGGGTATAGCTCAGGGGTAGAGCAT	0	0.15616	0.069847	0.222054
tsrna-23793	GGGGTATAGCTCAGGGGTAGAGCA	0.07141622	0.07808	0.069847	0.333081
tsrna-23792	GGGGTATAGCTCAGGGGTAGAGC	0	0	0.069847	0.111027
tsrna-23791	GGGGTATAGCTCAGGGGTAGAG	0.07141622	0	0	0
tsrna-23790	GGGGTATAGCTCAGGGGTAGA	0	0.15616	0.139694	0
tsrna-23789	GGGGTATAGCTCAGGGGTAG	0	0.15616	0	0
tsrna-23788	GGGGTATAGCTCAGGGG	0.07141622	0.07808	0	0
tsrna-23787	GGGGTATAGCTCAGGG	0	0	0.069847	0.111027
tsrna-23786	GGGGTAGAGCATTTGACTGCAGATCAAG	0	0.23424	0	0
tsrna-23784	GGGGTAGAGCATTTGACTGC	0.07141622	0.07808	0	0
tsrna-23780	GGGGGTTCGATTCCTTCCTTTTTTGCCA	0.14283243	0.31232	0	0.222054
tsrna-23779	GGGGGTTCGATTCCTTCCTTTTTTGCC	0.14283243	0.31232	0.139694	0
tsrna-23778	GGGGGTTCGATTCCTTCCTTTTTTGC	0	0.23424	0	0.111027
tsrna-23777	GGGGGTTCGATTCCTTCCTT	0	0.31232	0	0
tsrna-23776	GGGGGTGTAGCTCAGTGGTAGAGCGT	0.07141622	0.468481	0.139694	0
tsrna-23775	GGGGGTGTAGCTCAGTGGTAGAGCGCGTGCTTC	0.92841081	2.420483	0.558774	0.666163
tsrna-23774	GGGGGTGTAGCTCAGTGGTAGAGCGCGTGCTT	0.78557838	1.795843	0.558774	0.444109
tsrna-23773	GGGGGTGTAGCTCAGTGGTAGAGCGCGTGCT	1.2854919	2.654724	0.558774	0
tsrna-23772	GGGGGTGTAGCTCAGTGGTAGAGCGCGTGC	0.64274595	2.498564	0.349234	0.222054
tsrna-23771	GGGGGTGTAGCTCAGTGGTAGAGCGCGTG	0.35708108	0.468481	0.349234	0.333081
tsrna-23770	GGGGGTGTAGCTCAGTGGTAGAGCGCGT	0.21424865	0.390401	0	0.333081
tsrna-23769	GGGGGTGTAGCTCAGTGGTAGAGCGCG	0.35708108	0.31232	0.20954	0.333081
tsrna-23768	GGGGGTGTAGCTCAGTGGTAGAGCGCATGCTT	0.35708108	1.093122	0.139694	0.999244

tsrna-23767	GGGGGTGTAGCTCAGTGGTAGAGCGCATGC	0.14283243	0.546561	0.139694	0.444109
tsrna-23766	GGGGGTGTAGCTCAGTGGTAGAGCGCAT	0.07141622	0.15616	0.139694	0.333081
tsrna-23765	GGGGGTGTAGCTCAGTGGTAGAGCGCA	0.14283243	0.31232	0.139694	0.333081
tsrna-23764	GGGGGTGTAGCTCAGTGGTAGAGCGC	0.35708108	0.468481	0.139694	0.333081
tsrna-23763	GGGGGTGTAGCTCAGTGGTAGAGCG	0.14283243	0.31232	0.069847	0.111027
tsrna-23762	GGGGGTGTAGCTCAGTGGTAGAGCATTTGACTG	0.64274595	1.093122	0.419081	1.221299
tsrna-23761	GGGGGTGTAGCTCAGTGGTAGAGCATTTGACT	0.49991351	0.858881	0.419081	0.666163
tsrna-23760	GGGGGTGTAGCTCAGTGGTAGAGCATTTGA	0.14283243	0.390401	0.279387	0.333081
tsrna-23759	GGGGGTGTAGCTCAGTGGTAGAGCATTTG	0.35708108	0.07808	0	0.444109
tsrna-23758	GGGGGTGTAGCTCAGTGGTAGAGCATT	0.35708108	0.468481	0.139694	0.222054
tsrna-23757	GGGGGTGTAGCTCAGTGGTAGAGCAT	0.21424865	0.07808	0	0.333081
tsrna-23756	GGGGGTGTAGCTCAGTGGTAGAGCACATGC	0.21424865	0.23424	0	0.666163
tsrna-23755	GGGGGTGTAGCTCAGTGGTAGAGCAC	0.49991351	0.07808	0.069847	0.333081
tsrna-23754	GGGGGTGTAGCTCAGTGGTAGAGCA	0.14283243	0.468481	0.419081	0.111027
tsrna-23753	GGGGGTGTAGCTCAGTGGTAGAGC	0.21424865	0.468481	0.279387	0
tsrna-23752	GGGGGTGTAGCTCAGTGGTAGAG	0.28566487	0.390401	0.069847	0.444109
tsrna-23751	GGGGGTGTAGCTCAGTGGTAGA	0.28566487	0.390401	0	0.222054
tsrna-23750	GGGGGTGTAGCTCAGTGGTAG	0.28566487	0.23424	0.069847	0.222054
tsrna-23749	GGGGGTGTAGCTCAGTGGTA	0.07141622	0.31232	0.20954	0.333081
tsrna-23748	GGGGGTGTAGCTCAGTGGT	0.07141622	0.702721	0	0
tsrna-23747	GGGGGTGTAGCTCAGTGG	0.21424865	0.07808	0.069847	0
tsrna-23746	GGGGGTGTAGCTCAGTG	0.07141622	0.23424	0	0.222054
tsrna-23745	GGGGGTGTAGCTCAGT	0.07141622	0	0.069847	0
tsrna-23740	GGGGGTCGATTCCCCGACGGGGAGCCA	0.07141622	1.015041	0	0
tsrna-23739	GGGGGTATAGTTCAGGGGTAG	0	0	0	0.111027
tsrna-23738	GGGGGTATAGCTTAGCGGTAGAGCATTTGACT	0.28566487	0.624641	0.698468	0.77719
tsrna-23737	GGGGGTATAGCTTAGCGGTAGAGCATTTG	0.21424865	0.15616	0.20954	0.222054
tsrna-23736	GGGGGTATAGCTTAGCGGTAGAGC	0.07141622	0	0.488928	0
tsrna-23735	GGGGGTATAGCTTAGCGGTA	0.14283243	0.15616	0.069847	0.222054
tsrna-23734	GGGGGTATAGCTTAGCGG	0.21424865	0.31232	0.419081	0
tsrna-23733	GGGGGTATAGCTTAGC	0.21424865	0.390401	0.20954	0.444109
tsrna-23732	GGGGGTATAGCTCAGTGGTAGAGCGCGTGCTT	0.8569946	2.576644	0.419081	0.444109
tsrna-23731	GGGGGTATAGCTCAGTGGTAGAGCGCGTGC	0.8569946	2.342403	0.488928	0.555136
tsrna-23730	GGGGGTATAGCTCAGTGGTAGAGCATTTGACTGCA	3.07089731	2.654724	1.466783	0.888217
tsrna-23729	GGGGGTATAGCTCAGTGGTAGAGCATTTGACTGC	3.07089731	2.810884	1.53663	0.77719
tsrna-23728	GGGGGTATAGCTCAGTGGTAGAGCATTTGACTG	2.57098379	1.561602	0.419081	0.77719
tsrna-23727	GGGGGTATAGCTCAGTGGTAGAGCATTTGACT	1.07124325	1.405442	0.698468	0.77719
tsrna-23726	GGGGGTATAGCTCAGTGGTAGAGCATTTGAC	0.21424865	0.31232	0.419081	0.444109
tsrna-23725	GGGGGTATAGCTCAGTGGTAGAGCATTTGA	0.28566487	0.23424	0.20954	0.444109
tsrna-23724	GGGGGTATAGCTCAGTGGTAGAGCATTTG	0.07141622	0.468481	0.069847	0.222054
tsrna-23723	GGGGGTATAGCTCAGTGGTAGAGCATTT	0.49991351	0.390401	0.139694	0.333081
tsrna-23722	GGGGGTATAGCTCAGTGGTAGAGCATT	0.57132973	0.07808	0.488928	0.666163
tsrna-23721	GGGGGTATAGCTCAGTGGTAGAGCAT	0.14283243	0.15616	0.069847	0
tsrna-23720	GGGGGTATAGCTCAGTGGTAGAGCA	0.4284973	0.23424	0.20954	0.555136
tsrna-23719	GGGGGTATAGCTCAGTGGTAGAGC	0.28566487	0	0.139694	0.111027
tsrna-23718	GGGGGTATAGCTCAGTGGTAGAG	0.14283243	0.31232	0.349234	0.222054
tsrna-23717	GGGGGTATAGCTCAGTGGTAGA	0.35708108	0.23424	0.139694	0.333081
tsrna-23716	GGGGGTATAGCTCAGTGGTAG	0.14283243	0.31232	0.20954	0.222054
tsrna-23715	GGGGGTATAGCTCAGTGGTA	0.21424865	0.23424	0.349234	0
tsrna-23714	GGGGGTATAGCTCAGTGGT	0.07141622	0.15616	0.349234	0.555136
tsrna-23713	GGGGGTATAGCTCAGTGGGTAGAGCATTTGACTGC	0.64274595	0.31232	0.139694	0.222054
tsrna-23712	GGGGGTATAGCTCAGTGGGTAGAGCATTTGACTG	0.4284973	0.468481	0	0.555136
tsrna-23711	GGGGGTATAGCTCAGTGGGTAGAGCATTTGACT	0.28566487	0.31232	0.069847	0.222054
tsrna-23710	GGGGGTATAGCTCAGTGGGTAGAGCATTTG	0.07141622	0.23424	0.069847	0.333081
tsrna-23709	GGGGGTATAGCTCAGTGGGTAGAGCATTT	0	0.31232	0.069847	0.222054
tsrna-23708	GGGGGTATAGCTCAGTGGGTAGAGCATT	0.14283243	0.23424	0.279387	0
tsrna-23707	GGGGGTATAGCTCAGTGGGTAGAGCAT	0.14283243	0.23424	0.069847	0.111027
tsrna-23706	GGGGGTATAGCTCAGTGGGTAGAGCA	0	0.07808	0.139694	0.222054
tsrna-23705	GGGGGTATAGCTCAGTGGGTAGAGC	0.14283243	0.15616	0	0.222054
tsrna-23704	GGGGGTATAGCTCAGTGGGTAGAG	0.21424865	0.07808	0.279387	0
tsrna-23703	GGGGGTATAGCTCAGTGGGTAGA	0.07141622	0.07808	0.069847	0
tsrna-23702	GGGGGTATAGCTCAGTGGGTAG	0.28566487	0.07808	0	0.111027
tsrna-23701	GGGGGTATAGCTCAGTGGGTA	0	0.07808	0.20954	0.222054
tsrna-23700	GGGGGTATAGCTCAGTGGGT	0	0	0.069847	0.222054

tsrna-23699	GGGGGTATAGCTCAGTGGG	0.07141622	0.31232	0.069847	0.222054
tsrna-23698	GGGGGTATAGCTCAGTGG	0.21424865	0.15616	0	0
tsrna-23697	GGGGGTATAGCTCAGTG	0.07141622	0.15616	0.139694	0
tsrna-23696	GGGGGTATAGCTCAGT	0.07141622	0.07808	0.139694	0
tsrna-23695	GGGGGTATAGCTCAGGTGGTAGAGCATTTGACTGC	0.14283243	0.15616	0.20954	0
tsrna-23694	GGGGGTATAGCTCAGGTGGTAGAGCATTTGACTG	0	0.23424	0.139694	0.222054
tsrna-23693	GGGGGTATAGCTCAGGTGGTAGAGCATTTGACT	0.07141622	0.07808	0	0.111027
tsrna-23692	GGGGGTATAGCTCAGGTGGTAGAGCATTTG	0.07141622	0.15616	0	0
tsrna-23691	GGGGGTATAGCTCAGGTGGTAGAGCATT	0.07141622	0	0.069847	0.222054
tsrna-23690	GGGGGTATAGCTCAGGTGGTAGAGCAT	0.07141622	0	0	0.333081
tsrna-23689	GGGGGTATAGCTCAGGTGGTAGAGCA	0	0.15616	0.20954	0.111027
tsrna-23688	GGGGGTATAGCTCAGGTGGTAGAGC	0	0.15616	0	0.222054
tsrna-23687	GGGGGTATAGCTCAGGTGGTAGAG	0	0.23424	0.069847	0
tsrna-23686	GGGGGTATAGCTCAGGTGGTAGA	0.14283243	0.15616	0	0.222054
tsrna-23685	GGGGGTATAGCTCAGGTGGTAG	0.14283243	0.15616	0.069847	0.111027
tsrna-23684	GGGGGTATAGCTCAGGTGGTA	0.07141622	0.15616	0.069847	0.111027
tsrna-23683	GGGGGTATAGCTCAGGTGGT	0.07141622	0	0.139694	0.111027
tsrna-23682	GGGGGTATAGCTCAGGTGG	0.21424865	0	0	0.111027
tsrna-23681	GGGGGTATAGCTCAGGT	0	0.07808	0.069847	0
tsrna-23680	GGGGGTATAGCTCAGGGGTAGAGCATTTGACTGC	0.64274595	0.936961	0.349234	0.444109
tsrna-23679	GGGGGTATAGCTCAGGGGTAGAGCATTTGACTG	0.4284973	1.015041	0.139694	0.555136
tsrna-23678	GGGGGTATAGCTCAGGGGTAGAGCATTTGACT	0.35708108	0.858881	0.419081	0.222054
tsrna-23677	GGGGGTATAGCTCAGGGGTAGAGCATTTGA	0	0.23424	0	0.666163
tsrna-23676	GGGGGTATAGCTCAGGGGTAGAGCATTTG	0	0.23424	0.139694	0.555136
tsrna-23675	GGGGGTATAGCTCAGGGGTAGAGCATTT	0.14283243	0.23424	0.139694	0.333081
tsrna-23674	GGGGGTATAGCTCAGGGGTAGAGCATT	0.14283243	0.07808	0.139694	0.444109
tsrna-23673	GGGGGTATAGCTCAGGGGTAGAGCAT	0.07141622	0.07808	0.069847	0.555136
tsrna-23672	GGGGGTATAGCTCAGGGGTAGAGCACTTG	0.14283243	0.15616	0	0.222054
tsrna-23671	GGGGGTATAGCTCAGGGGTAGAGCA	0.07141622	0.07808	0.069847	0.666163
tsrna-23670	GGGGGTATAGCTCAGGGGTAGAGC	0	0.07808	0.139694	0.222054
tsrna-23669	GGGGGTATAGCTCAGGGGTAGAG	0.07141622	0	0	0
tsrna-23668	GGGGGTATAGCTCAGGGGTAGA	0.07141622	0.15616	0.139694	0.222054
tsrna-23667	GGGGGTATAGCTCAGGGGTAG	0.07141622	0.31232	0	0.111027
tsrna-23666	GGGGGTATAGCTCAGGGGTA	0	0.15616	0.069847	0.222054
tsrna-23665	GGGGGTATAGCTCAGGGGT	0.14283243	0.23424	0.20954	0
tsrna-23664	GGGGGTATAGCTCAGGGG	0	0.07808	0.069847	0.222054
tsrna-23663	GGGGGTATAGCTCAGGG	0	0.23424	0	0.111027
tsrna-23662	GGGGGTATAGCTCAGG	0	0.07808	0	0.111027
tsrna-23661	GGGGGTATAGCTCAGCGGTAGAGCGCGTGCT	0.4284973	1.795843	0.20954	0.666163
tsrna-23660	GGGGGTATAGCTCAGCGGTAGAGCGCGTGC	0.35708108	1.015041	0.069847	0.999244
tsrna-23659	GGGGGTATAGCTCAGCGGTAGAGCGCG	0.21424865	0.31232	0.069847	0.222054
tsrna-23658	GGGGGTATAGCTCAGCGGTAGAGC	0.21424865	0.23424	0	0.111027
tsrna-23657	GGGGGTATAGCTCAGCGGTAGAG	0.07141622	0.31232	0.069847	0.222054
tsrna-23656	GGGGGTATAGCTCAGCGGTAGA	0.21424865	0.07808	0.069847	0.555136
tsrna-23655	GGGGGTATAGCTCAGCGGTAG	0	0	0.069847	0.555136
tsrna-23654	GGGGGTATAGCTCAGCGGTA	0	0.15616	0.069847	0.444109
tsrna-23653	GGGGGTATAGCTCAGCGGT	0.14283243	0.390401	0.139694	0.111027
tsrna-23652	GGGGGTATAGCTCAGCGG	0	0.15616	0	0.111027
tsrna-23651	GGGGGTATAGCTCAGCG	0.07141622	0.15616	0	0.444109
tsrna-23650	GGGGGTATAGCTCAGC	0.07141622	0.15616	0.069847	0.111027
tsrna-23649	GGGGGGTTCGATTCCTTCCTTTTTTGCCA	0.21424865	0.390401	0.139694	0.333081
tsrna-23648	GGGGGGTTCGATTCCTTCCTTTTTTGCC	0.14283243	0.468481	0	0
tsrna-23647	GGGGGGTTCGATTCCTTCCTTTTTTGC	0.14283243	0	0	0
tsrna-23646	GGGGGGTTCGATTCCTTCCTTTTTTG	0	0.468481	0	0
tsrna-23645	GGGGGGTTCGATTCCTTCCTTT	0.07141622	0.15616	0.069847	0
tsrna-23644	GGGGGGTTCGATTCCTTCCTT	0	0.07808	0	0
tsrna-23641	GGGGGGTGTAGCTCAGTGG	0.07141622	0.07808	0	0.111027
tsrna-23638	GGGGGCATAGCTCAGTGGTAGAGCATTTGACTGC	0.14283243	0.780801	0.488928	0.999244
tsrna-23637	GGGGGCATAGCTCAGTGGTAGAGCATTTGACTG	0.07141622	0.390401	0.069847	0.555136
tsrna-23636	GGGGGCATAGCTCAGTGGTAGAGCATTTGACT	0.14283243	0.546561	0.069847	0.222054
tsrna-23635	GGGGGCATAGCTCAGTGGTAGAGCATTTGA	0.07141622	0.07808	0	0.111027
tsrna-23634	GGGGGCATAGCTCAGTGGTAGAGCATTTG	0.21424865	0.31232	0	0.111027
tsrna-23633	GGGGGCATAGCTCAGTGGTAGAGCA	0	0.15616	0.139694	0
tsrna-23632	GGGGGCATAGCTCAGTGGTAGAGC	0	0.07808	0.139694	0

tsrna-23631	GGGGGCATAGCTCAGTGGTAGA	0	0	0	0.444109
tsrna-23630	GGGGGCATAGCTCAGTGGT	0.14283243	0.15616	0.069847	0.444109
tsrna-23629	GGGGGCATAGCTCAGTGG	0	0.07808	0	0.111027
tsrna-23627	GGGGGCAGAGCATTTGACTG	0.07141622	0	0	0
tsrna-23625	GGGGGATTAGCTCAAG	0.4284973	0.702721	0.279387	0.111027
tsrna-23624	GGGGGATTAGCTCAAATGGTAGAGCGCTCGCTT	0.99982703	2.108163	0.698468	0.555136
tsrna-23623	GGGGGATTAGCTCAAATGGTAGAGCGCTCGCT	1.57115676	2.030083	0.628621	0.555136
tsrna-23622	GGGGGATTAGCTCAAATGGTAGAGCGCTCGC	1.21407568	1.483522	0.977855	1.443353
tsrna-23621	GGGGGATTAGCTCAAATGGTAGAGCGCTCG	1.49974054	1.639682	1.187396	0.999244
tsrna-23620	GGGGGATTAGCTCAAATGGTAGAGCGCTC	0.8569946	1.952003	0.838162	0.111027
tsrna-23619	GGGGGATTAGCTCAAATGGTAGAGCGCT	0.99982703	1.093122	0.419081	0.111027
tsrna-23618	GGGGGATTAGCTCAAATGGTAGAGCGC	1.21407568	1.639682	0.698468	0.666163
tsrna-23617	GGGGGATTAGCTCAAATGGTAGAGCG	0.64274595	1.873923	0.768315	0.888217
tsrna-23616	GGGGGATTAGCTCAAATGGTAGAGC	1.21407568	1.093122	0.698468	0.999244
tsrna-23615	GGGGGATTAGCTCAAATGGTAGAG	1.71398919	1.795843	0.977855	0.555136
tsrna-23614	GGGGGATTAGCTCAAATGGTAGA	1.21407568	1.952003	0.488928	0.444109
tsrna-23613	GGGGGATTAGCTCAAATGGTAG	1.14265946	1.171202	0.768315	0.555136
tsrna-23612	GGGGGATTAGCTCAAATGGTA	0.71416216	1.249282	0.977855	0.444109
tsrna-23611	GGGGGATTAGCTCAAATGGT	1.21407568	1.327362	0.698468	0.444109
tsrna-23610	GGGGGATTAGCTCAAATGG	0.71416216	1.405442	0.419081	0.333081
tsrna-23609	GGGGGATTAGCTCAAATG	0.64274595	1.639682	0.558774	0.222054
tsrna-23608	GGGGGATTAGCTCAAAT	1.14265946	1.639682	0.768315	0.999244
tsrna-23607	GGGGGATTAGCTCAAA	1.14265946	1.561602	0.977855	0.444109
tsrna-23605	GGGGCGTGGGTTCGAATC	0	0.07808	0	0
tsrna-23598	GGGGATTGTGGGTTCGAGTCCCATCTGGGTCGCC	0	0.15616	0.069847	0
tsrna-23597	GGGGATTGTGGGTTCGAGTCCCATC	0	0.07808	0	0
tsrna-23596	GGGGATTGTGGGTTCGAGTCCCA	0	0.15616	0	0
tsrna-23595	GGGGATTGTGGGTTCGAGTCCC	0	0.23424	0	0
tsrna-23592	GGGGATTGTGGGTTCG	0	0	0	0
tsrna-23591	GGGGATTAGCTCAAATGGTAGAGC	0.71416216	1.171202	0.349234	0.333081
tsrna-23590	GGGGATTAGCTCAAATGGTAGAG	0.8569946	0.546561	0.558774	0.222054
tsrna-23589	GGGGATTAGCTCAAATGGTAGA	0.71416216	1.093122	0.488928	0.666163
tsrna-23588	GGGGATTAGCTCAAATGGTAG	1.21407568	1.093122	0.349234	0.333081
tsrna-23587	GGGGATTAGCTCAAATGGT	0.78557838	0.624641	0.139694	0.555136
tsrna-23586	GGGGATTAGCTCAAATGG	0.71416216	1.015041	0.488928	0.222054
tsrna-23585	GGGGATTAGCTCAAAT	0.4284973	1.249282	0.419081	0.666163
tsrna-23584	GGGGATGTAGCTCAGTGGTAGAGCGCGCGCTTC	0.78557838	1.639682	0.558774	1.443353
tsrna-23583	GGGGATGTAGCTCAGTGGTAGAGCGCGCGCTT	0.99982703	1.483522	0.069847	0.77719
tsrna-23582	GGGGATGTAGCTCAGTGGTAGAGCGCGCGCT	0.8569946	1.405442	0.488928	1.110271
tsrna-23581	GGGGATGTAGCTCAGTGGTAGAGCGCGCGC	0.4284973	1.171202	0.488928	0.444109
tsrna-23580	GGGGATGTAGCTCAGTGGTAGAGCGCGCG	0.64274595	0.936961	0	0.555136
tsrna-23579	GGGGATGTAGCTCAGTGGTAGAGCGCGC	0.4284973	1.015041	0.279387	0.666163
tsrna-23578	GGGGATGTAGCTCAGTGGTAGAGCGCG	0.8569946	0.624641	0.419081	0.666163
tsrna-23577	GGGGATGTAGCTCAGTGGTAGAGCGCATGCTTTGCA	0.49991351	0.390401	0.488928	0.444109
tsrna-23576	GGGGATGTAGCTCAGTGGTAGAGCGCATGCTT	0.21424865	0.858881	0	0.77719
tsrna-23575	GGGGATGTAGCTCAGTGGTAGAGCGCATGCT	0.4284973	0.936961	0.349234	0.888217
tsrna-23574	GGGGATGTAGCTCAGTGGTAGAGCGCATGC	0.4284973	0.702721	0.279387	0.444109
tsrna-23573	GGGGATGTAGCTCAGTGGTAGAGCGCATG	0.49991351	1.015041	0.20954	0.555136
tsrna-23572	GGGGATGTAGCTCAGTGGTAGAGCGCAT	0.35708108	0.624641	0.069847	0.444109
tsrna-23571	GGGGATGTAGCTCAGTGGTAGAGCGCA	0.35708108	1.015041	0.139694	0.444109
tsrna-23570	GGGGATGTAGCTCAGTGGTAGAGCGC	0.28566487	1.093122	0.139694	0.555136
tsrna-23569	GGGGATGTAGCTCAGTGGTAGAGCG	0.49991351	1.093122	0.139694	0.666163
tsrna-23568	GGGGATGTAGCTCAGTGGTAGAGC	0.4284973	0.936961	0.279387	0.444109
tsrna-23567	GGGGATGTAGCTCAGTGGTAGAG	0.21424865	0.702721	0.139694	0.333081
tsrna-23566	GGGGATGTAGCTCAGTGGTAGA	0.07141622	0.780801	0.20954	0.666163
tsrna-23565	GGGGATGTAGCTCAGTGGTAG	0.57132973	0.31232	0.349234	0.666163
tsrna-23564	GGGGATGTAGCTCAGTGGTA	0.35708108	0.546561	0.20954	0.333081
tsrna-23563	GGGGATGTAGCTCAGTGGT	0.21424865	0.468481	0.069847	0.333081
tsrna-23562	GGGGATGTAGCTCAGTGG	0.14283243	0.31232	0	0
tsrna-23561	GGGGATGTAGCTCAGTG	0.07141622	0.23424	0	0
tsrna-23560	GGGGATGTAGCTCAGT	0.28566487	0.15616	0	0
tsrna-23559	GGGGATATAGCTCAGGGGTAGAGCATTTG	0.07141622	0	0.069847	0.111027
tsrna-23558	GGGGAATTAGCTCAGGCGGTAGAGC	0	0.15616	0	0.111027
tsrna-23557	GGGGAATTAGCTCAGGCGGTAGAG	0.07141622	0	0	0

tsrna-23556	GGGGAATTAGCTCAGGCGGTAGA	0.07141622	0	0	0
tsrna-23555	GGGGAATTAGCTCAGGCGGTA	0	0.07808	0	0
tsrna-23551	GGGGAATTAGCTCAAGTGGTAGAGCGCTTG	0.14283243	0.07808	0.069847	0.111027
tsrna-23547	GCAGTTGACCCGGGTTCGATTCCCGGCCA	0.07141622	0.31232	0	0
tsrna-23546	GCAGTTGACCCGGGTTCGA	0	0	0.069847	0
tsrna-23545	GCAGTTGACCCGGGTTCG	0	0.07808	0	0
tsrna-23544	GCAGTTGACCCGGGTTC	0	0	0	0
tsrna-23543	GCAGTGGTAGAATTCTCGCCT	0.21424865	0	0.139694	0.111027
tsrna-23542	GCAGTGGTAGAATTCTCGCC	0.07141622	0	0.20954	0
tsrna-23541	GCAGTGGTAGAATTCTCGC	0.07141622	0.07808	0.069847	0
tsrna-23540	GCAGTGGTAGAATTCTCG	0.14283243	0.07808	0	0
tsrna-23539	GCAGTCTCCCCTGGAGGCGTGGGT	0	0.15616	0.069847	0
tsrna-23533	GCAGGTTCGAGTCCTGCCGCGGTCGCCA	0.14283243	0.858881	0	0
tsrna-23531	GCAGGTTCGAATCCTGCTCACAGCGCCA	0.21424865	1.327362	0.349234	0.111027
tsrna-23529	GCAGGTTCGAATCCTGCTCACAG	0	0	0.069847	0
tsrna-23528	GCAGGTTCGAATCCTGCCGACTACGC	0	0.15616	0	0.111027
tsrna-23525	GCAGGTTCGAACCCTGCTCGCTGCGCCA	0.71416216	2.498564	0.349234	0
tsrna-23524	GCAGCTTCAAACCTGCCGGGGCTTCC	0.07141622	0.07808	0	0
tsrna-23520	GCAGCGGAAGCGTGCTGGGCCC	0.28566487	3.201285	0.628621	0.77719
tsrna-23519	GCAGCGGAAGCGTGCTGGGCC	0.35708108	1.873923	0.20954	0.222054
tsrna-23518	GCAGCGGAAGCGTGCTGGGC	0.35708108	1.717762	0.349234	0
tsrna-23517	GCAGCGATGGCCGAGTGGTTAAGGCGTTGG	0.07141622	0.31232	0	0
tsrna-23516	GCAGCGATGGCCGAGTGGTTAAGGCGTTG	0.14283243	0.390401	0.20954	0.111027
tsrna-23515	GCAGCGATGGCCGAGTGGTTAAGGC	0.07141622	0.390401	0	0
tsrna-23514	GCAGCGATGGCCGAGTGGTTAAGG	0.07141622	0.390401	0	0
tsrna-23513	GCAGCGATGGCCGAGTGGTTAAG	0.28566487	0.390401	0.069847	0.111027
tsrna-23512	GCAGCGATGGCCGAGTGGTTAA	0.21424865	0.23424	0.069847	0
tsrna-23511	GCAGCGATGGCCGAGTGGTTA	0	0.07808	0.069847	0.111027
tsrna-23510	GCAGCGATGGCCGAGTGGTT	0.21424865	0.07808	0	0
tsrna-23509	GCAGCGATGGCCGAGTGGT	0.21424865	0.31232	0.069847	0
tsrna-23508	GCAGCGATGGCCGAGTGG	0.07141622	0.468481	0	0
tsrna-23507	GCAGCGATGGCCGAGT	0.07141622	0	0.069847	0.111027
tsrna-23506	GCAGCAACCTCGGTTCGAATCCGAGTCACG	0.07141622	0	0	0
tsrna-23502	GCAGCAACCTCGGTTCGAA	0	0	0.069847	0
tsrna-23501	GCAGCAACCTCGGTTCGA	0.07141622	0	0	0
tsrna-23500	GCAGCAACCTCGGTTCG	0	0	0	0
tsrna-23498	GCAGATCAAGAGGTCCCTGGTTCA	0.14283243	0.546561	0.069847	0.111027
tsrna-23497	GCAGATCAAGAGGTCCCTGGTTC	0.21424865	1.093122	0.20954	0.111027
tsrna-23496	GCAGATCAAGAGGTCCCTGGTT	0.14283243	0.624641	0.349234	0
tsrna-23495	GCAGATCAAGAGGTCCCTGGT	0	0.31232	0.419081	0.222054
tsrna-23493	GCAGATCAAGAGGTCCCCGGTTCA	0	0.390401	0	0.111027
tsrna-23492	GCAGATCAAGAGGTCCCCGGTTC	0	0.468481	0	0
tsrna-23491	GCAGATCAAGAGGTCCCCGGTT	0.07141622	0.07808	0	0.111027
tsrna-23490	GCAGATCAAGAGGTCCCCGGT	0	0.07808	0	0.111027
tsrna-23487	GCAGATCAAGAGGTCCCCAGT	0	0.07808	0	0
tsrna-23483	GCAGATCAAGAAGTCCCCGGTT	0.07141622	0.624641	0	0
tsrna-23482	GCAGATCAAGAAGTCCCCGGT	0.07141622	0.546561	0	0.111027
tsrna-23480	GCAGATCAAAAGGTCCCTGGT	0.07141622	0.468481	0.069847	0.111027
tsrna-23479	GCAGAGTGGGGTTTTGCAGTCCTTACCA	0	0.07808	0.069847	0.111027
tsrna-23478	GCAGAGTGGGGTTTTGCAGTCCTTACC	0	0.07808	0	0
tsrna-23477	GCAGAGTGGGGTTTTGCAGTCCTTAC	0	0	0	0
tsrna-23476	GCAGAGTGGGGTTTTGCAGTCCTTA	0	0	0.139694	0
tsrna-23475	GCAGAGTGGGGTTTTGCAGTCCTT	0	0	0.069847	0
tsrna-23474	GCAGAGTGGGGTTTTGCAGTCCT	0.14283243	0	0	0
tsrna-23473	GCAGAGTGGGGTTTTGCAGTCC	0	0.15616	0	0
tsrna-23472	GCAGAGTGGGGTTTTGCAGTC	0	0	0	0
tsrna-23471	GCAGAGTGGGGTTTTGCAGT	0.07141622	0	0	0
tsrna-23470	GCAGAGTGGGGTTTTGCAG	0	0	0	0
tsrna-23469	GCAGAGTGGGGTTTTGCA	0	0	0.069847	0
tsrna-23467	GCAGAGTGGGGTTTTG	0	0.07808	0	0
tsrna-23466	GCAGAGTGGCGCAGCGGAAGCGTGCTGGGC	0.21424865	2.186243	0.20954	0.111027
tsrna-23465	GCAGAGTGGCGCAGCGGAAGCGTGCTGGG	0.21424865	2.420483	0.069847	0
tsrna-23464	GCAGAGTGGCGCAGCGGAAGCGTGCTGG	0	0.31232	0	0
tsrna-23463	GCAGAGTGGCGCAGCGGAAGCGTGCTG	0	0.15616	0	0

tsrna-23451	GCAGAGCCCGGTAATCGCATAAAACTTAA	0.07141622	0	0.139694	0
tsrna-23450	GCAGAGCCCGGTAATCGCATAAAACTTA	0	0.07808	0.139694	0
tsrna-23449	GCAGAGCCCGGTAATCGCATAAAACTT	0	0	0.069847	0
tsrna-23448	GCAGAGCCCGGTAATCGCATAAAACT	0	0	0	0
tsrna-23447	GCAGAGCCCGGTAATCGCATAAAAC	0	0.07808	0	0
tsrna-23446	GCAGAGCCCGGTAATCGCATAAAA	0	0	0	0.111027
tsrna-23444	GCAGAGCCCGGTAATCGCATAA	0	0	0.069847	0
tsrna-23442	GCAGAGCCCGGTAATCGCAT	0	0.07808	0	0
tsrna-23437	GCAGAAGGTCCTGGGTTCGAGCCCCAGTGGAACCACCA	3.9278919	3.201285	2.235098	2.997733
tsrna-23436	GCAGAAGGTCCTGGGTTCGAGCCCCAGTGGAACCACC	0.35708108	0.780801	0.349234	0.111027
tsrna-23435	GCAGAAGGTCCTGGGTTCGAGCCCCAGTGGAACCAC	0.35708108	0.07808	0.139694	0
tsrna-23434	GCAGAAGGTCCTGGGTTCGAGCCCCAGTGGAACC	0.14283243	0.15616	0.279387	0
tsrna-23433	GCAGAAGGTCCTGGGTTCGAGCCCCAGTGG	0.07141622	0.15616	0	0
tsrna-23432	GCAGAAGGTCCTGGGTTCGAGCCCCAGTG	0	0.23424	0.139694	0
tsrna-23431	GCAGAAGGTCCTGGGT	0	0.23424	0	0
tsrna-23430	GCACTGGTGGTTCAGTGGTAGAATTCTCGCCT	9.78402165	2.576644	11.87396	0.888217
tsrna-23429	GCACTGGTGGTTCAGTGGTAGAATTCTCGCC	5.99896218	2.342403	4.749583	0.888217
tsrna-23428	GCACTGGTGGTTCAGTGGTAGAATTCTCGC	1.14265946	0.546561	0.419081	0.333081
tsrna-23427	GCACTGGTGGTTCAGTGGTAGAATTCTCG	0.28566487	0.23424	0.279387	0.555136
tsrna-23426	GCACTGGTGGTTCAGTGGTAGAATTCTC	0.21424865	0.07808	0.488928	0.333081
tsrna-23425	GCACTGGTGGTTCAGTGGTAGAATTCT	0.21424865	0.23424	0.139694	0
tsrna-23424	GCACTGGTGGTTCAGTGGTAGAATTC	0.14283243	0.15616	0.20954	0
tsrna-23423	GCACTGGTGGTTCAGTGGTAGAATT	0.07141622	0.31232	0.069847	0
tsrna-23422	GCACTGGTGGTTCAGTGGTAGAAT	0.07141622	0	0.20954	0
tsrna-23421	GCACTGGTGGTTCAGTGGTAGAA	0.07141622	0.07808	0	0
tsrna-23420	GCACTGGTGGTTCAGTGGTAGA	0.14283243	0.07808	0	0
tsrna-23416	GCACTCTGGACTTTGAATCCAGC	0.07141622	0.15616	0.279387	0
tsrna-23415	GCACTCTGGACTTTGAATCCA	0.14283243	0.23424	0.139694	0
tsrna-23414	GCACTCTGGACTTTGAATCC	0.14283243	0.23424	0.139694	0
tsrna-23413	GCACTCTGGACTTTGAATC	0.14283243	0.31232	0	0
tsrna-23409	GCACTCTGGACTCTGAATCCG	0	0.15616	0	0
tsrna-23408	GCACTCTGGACTCTGAATCCAGCGATCCGAGTTCA	0.14283243	0.15616	0.069847	0
tsrna-23407	GCACTCTGGACTCTGAATCCAGCGATCCGAGTTC	0.07141622	0.23424	0.139694	0
tsrna-23406	GCACTCTGGACTCTGAATCCAGCGATCCGAGTT	0.14283243	0.23424	0.069847	0.111027
tsrna-23405	GCACTCTGGACTCTGAATCCAGCGATCCGAGT	0.14283243	0.23424	0.069847	0.111027
tsrna-23404	GCACTCTGGACTCTGAATCCAGCGATCCGAG	0.07141622	0.468481	0	0.111027
tsrna-23403	GCACTCTGGACTCTGAATCCAGCGATCCG	0.07141622	0.15616	0	0.111027
tsrna-23402	GCACTCTGGACTCTGAATCCAGCGATCC	0.14283243	0.31232	0.139694	0
tsrna-23401	GCACTCTGGACTCTGAATCCAGCGATC	0	0.390401	0.069847	0.111027
tsrna-23400	GCACTCTGGACTCTGAATCCAGCGAT	0	0.15616	0	0
tsrna-23399	GCACTCTGGACTCTGAATCCAGCGA	0	0.31232	0	0.111027
tsrna-23398	GCACTCTGGACTCTGAATCCAGCG	0	0.31232	0	0
tsrna-23397	GCACTCTGGACTCTGAATCCAGC	0	0.23424	0	0
tsrna-23396	GCACTCTGGACTCTGAATCCAG	0.07141622	0.23424	0.139694	0.111027
tsrna-23395	GCACTCTGGACTCTGAATCCA	0	0.15616	0	0
tsrna-23394	GCACTCTGGACTCTGAATCC	0	0.07808	0	0
tsrna-23392	GCACTCTGGACTCTGAAT	0	0.07808	0	0
tsrna-23389	GCACGTATGAGGCCCCGGGTT	0.07141622	0.31232	0.069847	0.222054
tsrna-23388	GCACGTATGAGGCCCCGGGT	0.07141622	0.31232	0	0.111027
tsrna-23386	GCACGAGGTCCTGGGTTCGATC	0.14283243	0.702721	0	0.333081
tsrna-23385	GCACGAGGTCCTGGGTTCGAT	0	0.780801	0.069847	0
tsrna-23384	GCACGAGGTCCTGGGTT	0.14283243	0.468481	0.279387	0.111027
tsrna-23383	GCACGAGGTCCTGGGT	0.14283243	0.624641	0.069847	0.111027
tsrna-23382	GCACGAGGCCCCGGGTTCAATCCCCGGCACCT	0	0.07808	0	0
tsrna-23381	GCACGAGGCCCCGGGTTCAATC	0.07141622	0	0	0
tsrna-23380	GCACGAGGCCCCGGGTTCAA	0.07141622	0.15616	0.139694	0
tsrna-23379	GCACGAGGCCCCGGGTTCA	0	0.07808	0	0
tsrna-23378	GCACGAGGCCCCGGGTTC	0	0.07808	0	0
tsrna-23377	GCACGAGGCCCCGGGTT	0	0	0.069847	0
tsrna-23375	GCACCCTGGACTCTGAATCCAGC	0.07141622	0.07808	0	0
tsrna-23373	GCACCCAACTTACACTTAGGAGATTTCAACTTAACTTGACCG(	0.07141622	0.23424	0.768315	0
tsrna-23372	GCACCCAACTTACACTTAGGAGATTTCAACTTAACTTGACC	0.07141622	0	0.768315	0
tsrna-23371	GCACCCAACTTACACTTAGGAGATTTCAAC	0	0	0	0
tsrna-23369	GCACCCAACTTACACTTAGGAGATTTCA	0	0.07808	0	0

tsrna-23366	GCACCCAACTTACACTTAGGAGATT	0.07141622	0	0	0
tsrna-23356	GCAATGGTGGTTCAGTGGTAGAATTCTCGCCTT	3.42797839	1.952003	4.400349	0.666163
tsrna-23355	GCAATGGTGGTTCAGTGGTAGAATTCTCGCCT	3.21372974	1.639682	2.444638	0.111027
tsrna-23354	GCAATGGTGGTTCAGTGGTAGAATTCTCGCC	1.99965406	1.327362	2.304945	0.222054
tsrna-23353	GCAATGGTGGTTCAGTGGTAGAATTCTCGC	0.78557838	0.390401	0.419081	0.333081
tsrna-23352	GCAATGGTGGTTCAGTGGTAGAATTCTCG	0.35708108	0.31232	0.349234	0
tsrna-23351	GCAATGGTGGTTCAGTGGTAGAATTCTC	0	0.31232	0.139694	0
tsrna-23350	GCAATGGTGGTTCAGTGGTAGAATTCT	0	0	0.069847	0
tsrna-23349	GCAATGGTGGTTCAGTGGTAGAATTC	0.07141622	0.23424	0.139694	0.111027
tsrna-23348	GCAATGGTGGTTCAGTGGTAGAATT	0.21424865	0.07808	0.069847	0
tsrna-23347	GCAATGGTGGTTCAGTGGTAGAAT	0.21424865	0.15616	0.069847	0.222054
tsrna-23346	GCAATGGTGGTTCAGTGGTAGAA	0.07141622	0.07808	0	0
tsrna-23342	GCAATGGATAGCGCATTGGACT	0.64274595	0.624641	0.20954	0.333081
tsrna-23341	GCAATGGATAGCGCATTGGA	0.28566487	0.936961	0.279387	0.111027
tsrna-23340	GCAATGGATAGCGCATTGG	0.28566487	0.31232	0.139694	0.111027
tsrna-23339	GCAATGGATAGCGCATTG	0	0	0	0
tsrna-23333	GCAATACTTAATTTCTGCCA	0.21424865	0	0.20954	0.111027
tsrna-23332	GCAATACTTAATTTCTGCC	0.07141622	0	0	0
tsrna-23329	GCAATACACTGAAAATGTTTAGACGGGCTCAC	2.07107028	1.171202	0.698468	0
tsrna-23328	GCAATACACTGAAAATGTTTAGACGGGCTC	0.28566487	0.468481	0.139694	0.444109
tsrna-23327	GCAAGATTCCCATTCTTGCGACCCG	0	0.15616	0.069847	0.111027
tsrna-23326	GCAAGATTCCCATTCTTGCGA	0.07141622	0.07808	0	0
tsrna-23318	GCAAATTCGAAGAAGCAGCTTCAAACCTGCCGGGGCTT	0.07141622	0.15616	0.139694	0
tsrna-23317	GCAAATTCGAAGAAGCAGCTTCAAACCTGC	0.07141622	0	0	0
tsrna-23316	GCAAATTCGAAGAAGCAGCTTC	0	0	0	0
tsrna-23314	GCAAATTCGAAGAAGCAGCT	0	0	0.069847	0
tsrna-23308	GATTTGCGTTCAGTTGATGCAGAGTGGGGT	0.07141622	0	0	0
tsrna-23307	GATTTGCGTTCAGTTGATGCAGA	0	0	0.069847	0
tsrna-23306	GATTTGCGTTCAGTTGATGCAG	0.07141622	0.07808	0	0
tsrna-23305	GATTTGCGTTCAGTTGATGCA	0	0	0	0
tsrna-23300	GATTTCGACTCATTAAATTATGATAATC	0.21424865	0.07808	0.20954	0
tsrna-23299	GATTTCGACTCATTAAATTATGATA	0.35708108	0.23424	0.139694	0
tsrna-23298	GATTTCGACTCATTAAATTATGA	0	0	0.069847	0
tsrna-23297	GATTTCGACTCATTAAATTATG	0	0	0	0
tsrna-23296	GATTTCGACTCATTAAATTA	0	0	0.069847	0
tsrna-23292	GATTTCAACTTAACTTGACCGCTCTGACCA	1.78540541	2.264323	2.654179	1.110271
tsrna-23291	GATTTCAACTTAACTTGACCGCTCTGACC	0.07141622	0.31232	0.349234	0.222054
tsrna-23290	GATTTCAACTTAACTTGACCGCTCTGAC	0	0.15616	0.139694	0
tsrna-23289	GATTTCAACTTAACTTGACCGCTCTGA	0.07141622	0	0	0
tsrna-23288	GATTTCAACTTAACTTGACCGCTCTG	0	0.07808	0.139694	0
tsrna-23287	GATTTCAACTTAACTTGACCGCTCT	0	0	0	0
tsrna-23286	GATTTCAACTTAACTTGACCGCTC	0	0	0.069847	0
tsrna-23276	GATTTAGGCTCCAGTCTCTTCGGAGGCGTG	0.07141622	0	0	0
tsrna-23275	GATTTAGGCTCCAGTCTCTTCGG	0	0.07808	0	0
tsrna-23268	GATTGTGAATCTGACAACAGAGGCTTACGACCCCTTATTTAC(	2.21390271	1.015041	1.676323	0.111027
tsrna-23267	GATTGTGAATCTGACAACAGAGGCTTACGACCCCTTATTTAC(	1.42832433	0.390401	1.885864	0
tsrna-23266	GATTGTGAATCTGACAACAGAGGCTTACGACCCCTTATTTAC(	2.14248649	0.702721	2.235098	0
tsrna-23265	GATTGTGAATCTGACAACAGAGGCTTACGACCCCTTATTTAC(	1.85682163	1.249282	2.304945	0
tsrna-23264	GATTGTGAATCTGACAACAGAGGCTTACGACCCCTT	0.14283243	0.23424	0.419081	0
tsrna-23263	GATTGTGAATCTGACAACAGAGGCTTACGACCCCT	0.35708108	0.23424	0.349234	0
tsrna-23262	GATTGTGAATCTGACAACAGAGGCTTACGACCCC	0.14283243	0.31232	0.20954	0
tsrna-23261	GATTGTGAATCTGACAACAGAGGCTTACGACCC	0	0.23424	0.279387	0
tsrna-23260	GATTGTGAATCTGACAACAGAGGCTTACGACC	0.07141622	0	0	0
tsrna-23259	GATTGTGAATCTGACAACAGAGGCTTACGA	0	0.31232	0.069847	0.111027
tsrna-23258	GATTGTGAATCTGACAACAGAGGCTTACG	0	0.07808	0.069847	0
tsrna-23257	GATTGTGAATCTGACAACAGAGGCTTAC	0	0.31232	0.139694	0
tsrna-23256	GATTGTGAATCTGACAACAGAGGCTTA	0.07141622	0.31232	0	0
tsrna-23255	GATTGTGAATCTGACAACAGAGGCTT	0	0.15616	0.069847	0
tsrna-23254	GATTGTGAATCTGACAACAGAGGCT	0	0.390401	0.139694	0
tsrna-23253	GATTGTGAATCTGACAACAGAGGC	0	0.31232	0.069847	0
tsrna-23252	GATTGTGAATCTGACAACAGAGG	0.14283243	0.15616	0.069847	0
tsrna-23251	GATTGTGAATCTGACAACAGAG	0.07141622	0.07808	0.069847	0
tsrna-23250	GATTGTGAATCTGACAACAGA	0	0.07808	0	0
tsrna-23249	GATTGTGAATCTGACAACAG	0.07141622	0.07808	0	0

tsrna-23248	GATTGTGAATCTGACAACA	0	0	0.069847	0
tsrna-23247	GATTGTGAATCTGACAAC	0	0.15616	0	0
tsrna-23246	GATTGTGAATCTGACAA	0	0.07808	0	0
tsrna-23245	GATTGTGAATCTGACA	0	0.23424	0.069847	0.111027
tsrna-23244	GATTGCAGGTTCGAGTCCTGCCGCGGTCGCCA	0.14283243	0.390401	0.20954	0
tsrna-23240	GATTGAGGGTTCGAGTCCCTTCGTGGTCGCCA	0.14283243	0.07808	0	0
tsrna-23239	GATTGAGGGTTCGAGTCCCTTCGTGGTCGCC	0	0.07808	0	0
tsrna-23234	GATTGAGGGTTCGAATCCCTCCGTGGTTAC	0.07141622	0.07808	0	0
tsrna-23233	GATTGAAGCCAGTTGATTAGGGTGCTTAGC	0	0	0	0
tsrna-23231	GATTGAAGCCAGTTGATTAG	0	0	0	0
tsrna-23230	GATTGAAGCCAGTTGATTA	0	0	0	0
tsrna-23225	GATTCTCATAGTCCTAGCCA	0	0	0	0
tsrna-23224	GATTCTCATAGTCCTAGCC	0	0	0	0
tsrna-23223	GATTCTCATAGTCCTAGC	0.07141622	0.07808	0	0
tsrna-23221	GATTCTCAGGGATGGGTTCG	0	0.15616	0.069847	0
tsrna-23220	GATTCTAGGTTCGACTCCTGGCTGGCTCGC	0	0	0.069847	0
tsrna-23219	GATTCTAGGTTCGACTCCTGGCTGGCTCG	0	0.07808	0	0
tsrna-23216	GATTCTAGGTTCGACTCCTG	0	0	0	0
tsrna-23215	GATTCGGCGCTTTCACCGCCGCGGCCC	0.07141622	0.390401	0.069847	0.555136
tsrna-23214	GATTCGGCGCTCTCACCGCCGCGGCCCGGGTT	0.07141622	0.390401	0	0.111027
tsrna-23213	GATTCGGCGCTCTCACCGCCGCGGCCCGGGT	0	0.07808	0	0
tsrna-23212	GATTCGGCGCTCTCACCGCCGCGGCCCGGG	0.07141622	0.15616	0	0
tsrna-23211	GATTCGGCGCTCTCACCGCCGCGGCCCGG	0	0.15616	0.069847	0
tsrna-23210	GATTCGGCGCTCTCACCGCCGCGGCCCG	0.07141622	0	0	0
tsrna-23209	GATTCGGCGCTCTCACCGCCGCGGCCC	0	0	0	0
tsrna-23208	GATTCGGCGCTCTCACCGCCGCGGC	0	0.07808	0	0
tsrna-23207	GATTCGGCGCTCTCACCGCCGCGG	0.07141622	0.15616	0.069847	0
tsrna-23206	GATTCGGCGCTCTCACCGCCGC	0.07141622	0.15616	0	0
tsrna-23205	GATTCGGCGCTCTCACCGCCG	0	0	0	0.111027
tsrna-23204	GATTCGGCGCTCTCACCGCC	0	0	0	0
tsrna-23203	GATTCGGCGCTCTCACCGC	0.07141622	0	0	0
tsrna-23202	GATTCGGCGCTCTCACCG	0	0	0	0
tsrna-23201	GATTCGGCGCTCTCACC	0	0	0	0
tsrna-23200	GATTCCTTCCTTTTTTGCCA	0	0.07808	0	0
tsrna-23198	GATTCCTTCCTTTTTTGC	0	0	0	0
tsrna-23195	GATTCCTGGTTTTCACCCA	0.07141622	0.07808	0	0
tsrna-23193	GATTCCGGCTCGAAGGACCA	4.14214055	10.07233	1.955711	0.999244
tsrna-23192	GATTCCGGCTCGAAGGACC	0.21424865	0.624641	0	0
tsrna-23191	GATTCCGGCTCGAAGGAC	0	0.07808	0	0
tsrna-23188	GATTCCGGATCAGAAGATTGAGGGT	0.64274595	0.390401	0.279387	0.333081
tsrna-23182	GATTCCCGGTCAGGGAACCA	0.28566487	0.15616	0	0.222054
tsrna-23178	GATTCCCGGGCGGCGCACCA	0.49991351	2.654724	0.419081	0
tsrna-23176	GATTCCCGGCCCATGCACCA	0.4284973	0.780801	0.419081	0.555136
tsrna-23175	GATTCCCGGCCCATGCACC	0.07141622	0.15616	0	0
tsrna-23173	GATTCCCGGCCAATGCACCA	0.14283243	0.31232	0.139694	0
tsrna-23172	GATTCCCGGCCAATGCACC	0.14283243	0.07808	0	0
tsrna-23170	GATTCCCGGCCAACGCACCA	0	0.07808	0.069847	0.222054
tsrna-23169	GATTCCCGGCCAACGCACC	0.07141622	0.07808	0	0.111027
tsrna-23165	GATTCCCCGACGGGGAGCCA	0.07141622	0	0	0
tsrna-23160	GATTCCCATTCTTGCGACCCGGGTTCG	0.28566487	0.702721	0	0
tsrna-23159	GATTCCCATTCTTGCGACCCGGGTT	0.21424865	0.23424	0.069847	0
tsrna-23158	GATTCCCATTCTTGCGACCCGGGT	0.07141622	0.07808	0	0
tsrna-23157	GATTCCCATTCTTGCGACCCGGG	0	0.15616	0	0
tsrna-23156	GATTCCCATTCTTGCGACCCGG	0.07141622	0	0	0.111027
tsrna-23155	GATTCCCATTCTTGCGACCCG	0.07141622	0	0	0
tsrna-23154	GATTCCCATTCTTGCGACCC	0.21424865	0.15616	0	0
tsrna-23153	GATTCCCATTCTTGCGACC	0	0.15616	0	0
tsrna-23152	GATTCCCATTCTTGCGAC	0	0.07808	0.069847	0
tsrna-23151	GATTCCCATTCTTGCGA	0	0	0	0
tsrna-23150	GATTCCCATTCTTGCG	0.07141622	0	0	0
tsrna-23148	GATTCCAGGTTCGACTCCTGGCTGGCTCG	0	0	0	0
tsrna-23135	GATTCATATCCGCGTGGGT	0.07141622	0.31232	0	0.111027
tsrna-23134	GATTAGGGTGCTTAGCTGTTAACTAAGTGT	0.57132973	0.07808	0.488928	0
tsrna-23133	GATTAGGGTGCTTAGCTGTTAACTAAG	0.21424865	0.23424	0.419081	0

tsrna-23132	GATTAGGGTGCTTAGCTGTTAACTA	0.21424865	0.07808	0.628621	0
tsrna-23131	GATTAGGGTGCTTAGCTGTTAACT	0.35708108	0.07808	0.558774	0
tsrna-23130	GATTAGGGTGCTTAGCTGTTAAC	0.07141622	0	0.139694	0
tsrna-23129	GATTAGGGTGCTTAGCTGTTAA	0	0.07808	0.139694	0
tsrna-23128	GATTAGGGTGCTTAGCTGTTA	0.21424865	0.07808	0.069847	0
tsrna-23127	GATTAGGGTGCTTAGCTGTT	0	0	0	0
tsrna-23126	GATTAGGGTGCTTAGCTGT	0	0.15616	0.069847	0
tsrna-23122	GATTAGCTCAAATGGTAGAGCGCTCGCTT	0	0	0	0
tsrna-23116	GATTAAGGCTCCAGTCTCT	0.07141622	0	0	0
tsrna-23115	GATTAAGAGAACCAACACCTCTTTACAGTGACCA	0.07141622	0.07808	0.069847	0
tsrna-23112	GATTAAGAGAACCAACACCTCTTTACAGTG	0	0	0	0
tsrna-23110	GATTAAGAGAACCAACACCTCTTTACAG	0	0	0	0
tsrna-23108	GATTAAGAGAACCAACACCTCTTTAC	0	0.07808	0	0
tsrna-23097	GATGTAGCTCAGTGGTAGAGCGCAT	0.07141622	0	0	0
tsrna-23093	GATGTAGCTCAGTGGTAGAG	0	0	0	0
tsrna-23089	GATGGTTTTTCATATCATTGGTCGTGGTTGTAGTCCGTGCGA(	0.71416216	1.015041	1.187396	1.776434
tsrna-23088	GATGGTTTTTCATATCATTGGTCGTGGTTGTAGTCCGTGCGA(	0.71416216	1.483522	1.257243	1.443353
tsrna-23087	GATGGTTTTTCATATCATTGGTCGTGGTTGTAGTCCGTGCGA(	0.57132973	1.093122	1.606477	0.888217
tsrna-23086	GATGGTTTTTCATATCATTGGTCGTGGTTGTAGTCC	0.07141622	0.23424	0.419081	0
tsrna-23085	GATGGTTTTTCATATCATTGGTCGTGGTTGTAGTC	0.07141622	0.07808	0	0.111027
tsrna-23084	GATGGTTTTTCATATCATTGGTCGTGGTTGTAGT	0.07141622	0.07808	0.069847	0
tsrna-23083	GATGGTTTTTCATATCATTGGTCGTGGTTG	0	0.07808	0.069847	0.111027
tsrna-23082	GATGGTTTTTCATATCATTGGTCGTGGT	0.14283243	0	0	0
tsrna-23081	GATGGTTTTTCATATCATTGGTCGTGG	0	0.07808	0	0
tsrna-23075	GATGGGTTCGATTCTCATAGTCCTAGCCA	0.07141622	0.390401	0.20954	0.111027
tsrna-23074	GATGGGTTCGATTCTCATAGTCCTAGC	0	0.390401	0.069847	0.444109
tsrna-23073	GATGGGTTCGATTCTCATAGTCCTAG	0	0.23424	0.20954	0.111027
tsrna-23072	GATGGGTTCGATTCTCATAGTCC	0	0.15616	0.139694	0.111027
tsrna-23071	GATGGGTTCGATTCTCATAGTC	0	0.07808	0	0
tsrna-23070	GATGGGGTGTGATAGGTGGCACGGAGAATTTTG	0.07141622	0.15616	0.069847	0.111027
tsrna-23069	GATGGGGTGTGATAGGTGGCACGGAGA	0	0	0	0
tsrna-23068	GATGGGGTGTGATAGGTGGCACGGAG	0	0	0	0
tsrna-23058	GATGGCGTGGGTTCGAATCCCACC	0	0	0	0
tsrna-23054	GATGGCCGAGTGGTCTAAGGCGCCAGAC	0.07141622	0	0	0
tsrna-23047	GATGGCCGAGCGGTCTAAGGCGCTGCGTTC	0	0.07808	0	0
tsrna-23046	GATGGCCGAGCGGTCTAAGGCGCTGCGTT	0	0.07808	0	0
tsrna-23033	GATGGCAGAGCCCGGTAATCGCATAAA	0.07141622	0	0	0
tsrna-23030	GATGGCAGAGCCCGGTAATCGCA	0	0.07808	0	0
tsrna-23022	GATGGATCGAAACCATCCTCTGCTACCA	1.14265946	1.717762	1.047702	1.443353
tsrna-23020	GATGGAAACCATCCTCTGCTACC	0	0	0	0
tsrna-23019	GATGCGAGAGGTCCCGGGT	0	0	0.069847	0
tsrna-23017	GATGCCCGCATTCTCCACCA	0.07141622	0.23424	0	0
tsrna-23016	GATGCCCGCATCCTCCACCA	0	0.15616	0.069847	0
tsrna-23015	GATGCCCGCATCCTCCACC	0.07141622	0	0.069847	0
tsrna-23012	GATGCAGAGTGGGGTTTTGCAGTCCTTACC	0.21424865	0	0	0
tsrna-23011	GATGCAGAGTGGGGTTTTGCAGTCCTTAC	0.14283243	0.15616	0.069847	0
tsrna-23010	GATGCAGAGTGGGGTTTTGCAGTCCTTA	0.07141622	0.07808	0	0
tsrna-23009	GATGCAGAGTGGGGTTTTGCAGTCCTT	0	0.07808	0.069847	0
tsrna-23008	GATGCAGAGTGGGGTTTTGCAGTCCT	0.14283243	0	0.069847	0.111027
tsrna-23007	GATGCAGAGTGGGGTTTTGCAGTCC	0	0	0	0
tsrna-23006	GATGCAGAGTGGGGTTTTGCAGTC	0.07141622	0.15616	0.139694	0
tsrna-23005	GATGCAGAGTGGGGTTTTGCAGT	0	0	0	0
tsrna-23004	GATGCAGAGTGGGGTTTTGCAG	0	0.07808	0	0
tsrna-23003	GATGCAGAGTGGGGTTTTGCA	0	0	0	0
tsrna-23002	GATGCAGAGTGGGGTTTTGC	0	0	0	0
tsrna-22997	GATGAAAACCTTTTTCCAAGGACACCA	0.21424865	0.31232	0.20954	0
tsrna-22996	GATGAAAACCTTTTTCCAAGGACACC	0.07141622	0	0	0
tsrna-22994	GATGAAAACCTTTTTCCAAGGACA	0	0	0	0
tsrna-22987	GATCGTATAGTGGTTAGTACTCTGCGTTGT	2.07107028	0.702721	0.908008	0.999244
tsrna-22986	GATCGTATAGTGGTTAGTACTCTGCGTTG	1.49974054	1.015041	0.488928	0.666163
tsrna-22985	GATCGTATAGTGGTTAGTACTCTGCGTT	1.14265946	0.702721	0.488928	0.666163
tsrna-22984	GATCGTATAGTGGTTAGTACTCTGCGT	0.78557838	0.31232	0.20954	0.111027
tsrna-22983	GATCGTATAGTGGTTAGTACTCTGCGCTG	0.8569946	0.31232	0.349234	0.222054
tsrna-22982	GATCGTATAGTGGTTAGTACTCTGCG	0.14283243	0.31232	0	0.111027

tsrna-22981	GATCGTATAGTGGTTAGTACTCTGC	0.14283243	0.07808	0.139694	0.111027
tsrna-22980	GATCGTATAGTGGTTAGTACTCTG	0.14283243	0.15616	0	0
tsrna-22979	GATCGTATAGTGGTTAGTACTCT	0.07141622	0	0.20954	0
tsrna-22978	GATCGTATAGTGGTTAGTACTC	0.07141622	0.07808	0	0.111027
tsrna-22977	GATCGTATAGTGGTTAGTACT	0.07141622	0	0.069847	0
tsrna-22976	GATCGTATAGTGGTTAGTAC	0	0	0	0
tsrna-22971	GATCGATGCCCGCATTCTCCACCA	0.14283243	0.23424	0	0
tsrna-22970	GATCGATGCCCGCATTCTCCACC	0	0.07808	0	0
tsrna-22968	GATCGATGCCCGCATCCTCCACCA	0.07141622	0.468481	0.069847	0
tsrna-22960	GATCGAAACCATCCTCTGCTACCA	1.35690811	1.561602	0.558774	1.221299
tsrna-22959	GATCGAAACCATCCTCTGCTACC	0.21424865	0.468481	0.139694	0.222054
tsrna-22958	GATCGAAACCATCCTCTGCTAC	0.07141622	0	0	0
tsrna-22952	GATCCTTAGGTCGCTGGTTCG	0	0	0	0
tsrna-22946	GATCCTCGCTGGGGCCTCCA	0.4284973	0.624641	0.279387	0.555136
tsrna-22945	GATCCTCACCTGGAGCACCA	0.07141622	0.15616	0	0
tsrna-22944	GATCCTCACACGGGGCACCA	0	0.31232	0	0
tsrna-22943	GATCCGAGTTCAAATCTCGGTGGAACCTCC	0.71416216	0.858881	0.488928	0.222054
tsrna-22942	GATCCCGGGTTTCGGCACCA	3.14231352	5.231367	0.628621	1.443353
tsrna-22941	GATCCCGGGTTTCGGCACC	0.35708108	0.23424	0	0
tsrna-22937	GATCCCGGGCGGAAACACCA	1.14265946	2.186243	0.768315	0.555136
tsrna-22936	GATCCCCGTACTGGCCACCA	0.07141622	0.15616	0.20954	0
tsrna-22935	GATCCCCGTACGGGCCACCA	0.71416216	4.528646	0.20954	0
tsrna-22934	GATCCCCGGCATCTCCACCA	7.28445408	8.666892	3.003413	4.441086
tsrna-22933	GATCCCCGGCATCTCCACC	0.21424865	0.546561	0.139694	0.222054
tsrna-22930	GATCCCCGGCACCTCCACCA	9.9982703	18.19267	6.076672	2.220543
tsrna-22929	GATCCCCGACACCTCCACCA	0.21424865	0.468481	0	0.111027
tsrna-22928	GATCCCCAGTACCTCCACCA	0.07141622	0.15616	0	0
tsrna-22927	GATCCCCAGTACCTCCACC	0	0	0.069847	0
tsrna-22926	GATCCCCAGCATCTCCACCA	0.07141622	0.390401	0.069847	0
tsrna-22923	GATCCCACCCAGGGACGCCA	0.07141622	0.468481	0	0.111027
tsrna-22922	GATCAGAAGGCTGCGTGTTCGAA	0	0.07808	0	0
tsrna-22921	GATCAGAAGGCTGCGTGTTCG	0.07141622	0	0.069847	0
tsrna-22920	GATCAGAAGGCTGCGTGTTC	0	0.07808	0	0
tsrna-22918	GATCAGAAGATTCTAGGTTCGACTCCTGGC	0.14283243	0.858881	0.069847	0
tsrna-22917	GATCAGAAGATTCCAGGTTC	0	0	0.069847	0
tsrna-22916	GATCAAGAGGTCCCTGGTT	0.28566487	0.15616	0.069847	0.333081
tsrna-22915	GATCAAGAGGTCCCCGGTTCA	0.07141622	0.390401	0.139694	0
tsrna-22914	GATCAAGAGGTCCCCGGTTC	0	0.390401	0.139694	0
tsrna-22913	GATCAAGAGGTCCCCGGTT	0.07141622	0.15616	0	0.222054
tsrna-22912	GATCAAGAGGTCCCCGGT	0.07141622	0.31232	0.069847	0
tsrna-22911	GATCAAAAGGTCCCTGGTT	0.64274595	0.702721	0.139694	0.888217
tsrna-22910	GATCAAAACCAGGCGGAAACACCA	0.4284973	0.468481	0.20954	0
tsrna-22909	GATAGGTGGCACGGAGAATTTTGGATT	0.07141622	0.31232	0.069847	0
tsrna-22908	GATAGGTGGCACGGAGAATTTTGGAT	0.07141622	0.15616	0.069847	0
tsrna-22907	GATAGGTGGCACGGAGAATTTTGGA	0.07141622	0.07808	0.069847	0
tsrna-22906	GATAGGTGGCACGGAGAATTTTGG	0	0.07808	0.069847	0
tsrna-22905	GATAGGTGGCACGGAGAATTTTG	0	0.15616	0	0
tsrna-22904	GATAGGTGGCACGGAGAATTTT	0	0	0	0
tsrna-22903	GATAGGTGGCACGGAGAATTT	0	0	0	0
tsrna-22902	GATAGGTGGCACGGAGAATT	0	0	0	0
tsrna-22898	GATAGCTCAGTTGGTAGAGCGGAGGACTGT	0	0.07808	0.139694	0.111027
tsrna-22896	GATAGCTCAGTTGGTAGAGC	0.07141622	0.15616	0.069847	0
tsrna-22895	GATAGCTCAGTTGGTAGAG	0	0	0	0.111027
tsrna-22894	GATAGCTCAGTTGGTAGAACA	0	0	0.069847	0
tsrna-22893	GATAGCTCAGTTGGGAGAGC	0	0.07808	0	0
tsrna-22891	GATAGCTCAGTCGGTAGAGCATCAGACTTTT	1.99965406	0.780801	1.257243	1.998488
tsrna-22890	GATAGCTCAGTCGGTAGAGCATCAGACTTT	0.64274595	1.561602	0.419081	0.111027
tsrna-22889	GATAGCTCAGTCGGTAGAGCATCAGACT	0.78557838	0.546561	0.838162	0.222054
tsrna-22888	GATAGCTCAGTCGGTAGAGCATCAGAC	0.64274595	0.936961	0.419081	0.222054
tsrna-22887	GATAGCTCAGTCGGTAGAGCATC	0.35708108	0.31232	0.20954	0.111027
tsrna-22881	GATAGCTCAGCTGGTAGAGCGGAGGACTGT	0	0	0	0
tsrna-22878	GATAGAGTAAATAATAGGAGCTTAAACCCC	0	0	0.069847	0
tsrna-22877	GATAGAGTAAATAATAGGAGCTTAAACCC	0	0	0	0
tsrna-22876	GATAGAGTAAATAATAGGAGCTTA	0.07141622	0	0.069847	0

tsrna-22875	GATAGAGTAAATAATAGGAGCTT	0	0	0	0.111027
tsrna-22874	GATAGAGTAAATAATAGGAGCT	0	0	0.069847	0
tsrna-22870	GATAATCATATTTACCAACCA	0	0	0	0
tsrna-22863	GATAAGGCGTCTGACTTCG	0.07141622	0	0	0
tsrna-22855	GATAACAGCTATCCATTGGTCTTAGGCCCC	0.07141622	0.07808	0.069847	0.111027
tsrna-22854	GATAACAGCTATCCATTGGTCTTAGGCCC	0	0	0.139694	0
tsrna-22853	GATAACAGCTATCCATTGGTCTTAGGCC	0	0	0	0
tsrna-22852	GATAACAGCTATCCATTGGTCTTAGGC	0	0	0.069847	0
tsrna-22851	GATAACAGCTATCCATTGGTCTTAGG	0	0	0	0
tsrna-22850	GATAACAGCTATCCATTGGTCTTAG	0	0	0	0
tsrna-22849	GATAACAGCTATCCATTGGTCTTA	0	0	0	0
tsrna-22848	GATAACAGCTATCCATTGGTCTT	0	0	0	0
tsrna-22841	GATAACACCAAGGTCGCGGGCTCG	0	0.07808	0	0
tsrna-22840	GATAACACCAAGGTCGCGGGCT	0.14283243	0.15616	0	0
tsrna-22838	GATAAAAGAGTTACTTTGATAGAGTAAATAATAGGAGCT	0.07141622	0.390401	0.349234	0
tsrna-22837	GATAAAAGAGTTACTTTGATAGAGTA	0.07141622	0.31232	0.139694	0
tsrna-22836	GATAAAAGAGTTACTTTGATAGAG	0.14283243	0.15616	0.069847	0
tsrna-22835	GATAAAAGAGTTACTTTGATAGA	0	0.31232	0.069847	0
tsrna-22834	GATAAAAGAGTTACTTTGATAG	0.07141622	0	0	0.111027
tsrna-22832	GAGTTCGATCCTCACACGGGGCACCA	0	0.07808	0	0
tsrna-22830	GAGTTCGAGTCTCGGTGGAACCTCCA	4.64205407	10.61889	1.396936	4.108004
tsrna-22829	GAGTTCGAGTCTCGGTGGAACCTC	0.14283243	0.07808	0	0
tsrna-22828	GAGTTCGAGTCTCGGTGGAAC	0	0.15616	0	0
tsrna-22826	GAGTTCGAGCCTCACCTGGAGCACCA	0	0.23424	0	0
tsrna-22823	GAGTTCGAACCTCAGAGGGGGCACCA	0	0.546561	0	0
tsrna-22821	GAGTTCAAATCTCGGTGGGACCTCCA	5.57046488	7.02721	1.74617	3.663896
tsrna-22820	GAGTTCAAATCTCGGTGGAACCTCCA	11.8550919	17.09954	3.352647	2.664651
tsrna-22819	GAGTTCAAATCTCGGTGGAACCTC	0.07141622	0.23424	0.069847	0
tsrna-22818	GAGTTCAAATCTCGGTGGAACCT	0	0.07808	0	0
tsrna-22815	GAGTTCAAATCTCGCTGGGGCCTC	0	0	0	0
tsrna-22814	GAGTTAAAGACTTTTTCTCTGACCA	1.42832433	2.654724	0.977855	1.221299
tsrna-22813	GAGTTAAAGACTTTTTCTCTGACC	0.21424865	1.171202	0	0.333081
tsrna-22812	GAGTTAAAGACTTTTTCTCTGAC	0.07141622	0	0	0
tsrna-22810	GAGTTAAAGACTTTTTCTCTG	0	0	0	0
tsrna-22792	GAGTGGTCTAAGGCGCTGGATT	0	0.07808	0.069847	0.111027
tsrna-22788	GAGTGGGGTTTTGCAGTCCTTACCA	0.07141622	0.31232	0.139694	0
tsrna-22787	GAGTGGGGTTTTGCAGTCCTTACC	0.14283243	0.07808	0	0
tsrna-22786	GAGTGGGGTTTTGCAGTCCTTAC	0.07141622	0.15616	0	0
tsrna-22785	GAGTGGGGTTTTGCAGTCCTTA	0	0	0	0
tsrna-22784	GAGTGGGGTTTTGCAGTCCTT	0	0	0	0
tsrna-22783	GAGTGGGGTTTTGCAGTCCT	0	0.07808	0.069847	0
tsrna-22782	GAGTGGGGTTTTGCAGTCC	0	0	0	0
tsrna-22778	GAGTGGCGCAGCGGAAGCGTGCTGGGCCC	0.8569946	4.372486	0.279387	0.666163
tsrna-22777	GAGTGGCGCAGCGGAAGCGTGCTGGGCC	0.28566487	2.498564	0	0
tsrna-22776	GAGTGGCGCAGCGGAAGCGTGCTGGGC	0.14283243	2.030083	0.069847	0.111027
tsrna-22775	GAGTGGCGCAGCGGAAGCGTGCTGGG	0.28566487	2.030083	0.069847	0
tsrna-22774	GAGTGGCGCAGCGGAAGCGTGCTGG	0	0.468481	0	0
tsrna-22769	GAGTGAAGCATTGGACTGTAAATCTAAAGA	0.14283243	0.07808	0.069847	0
tsrna-22768	GAGTGAAGCATTGGACTGTAAATCTAAAG	0.14283243	0	0.069847	0
tsrna-22767	GAGTGAAGCATTGGACTGTAAATCTAAA	0	0.15616	0	0
tsrna-22766	GAGTGAAGCATTGGACTGTAAATCTAA	0.07141622	0.15616	0.069847	0.111027
tsrna-22765	GAGTGAAGCATTGGACTGTAAATCTA	0.07141622	0.07808	0.069847	0
tsrna-22764	GAGTGAAGCATTGGACTGTAAATCT	0	0.07808	0	0.111027
tsrna-22763	GAGTGAAGCATTGGACTGTAAATC	0	0.15616	0.069847	0
tsrna-22762	GAGTGAAGCATTGGACTGTAAAT	0.07141622	0	0	0
tsrna-22761	GAGTGAAGCATTGGACTGTAAA	0	0.07808	0	0
tsrna-22760	GAGTGAAGCATTGGACTGTAA	0.07141622	0.07808	0	0
tsrna-22759	GAGTGAAGCATTGGACTGTA	0	0	0	0.111027
tsrna-22754	GAGTCTCGGTGGAACCTCCA	2.14248649	8.042251	1.466783	1.110271
tsrna-22753	GAGTCTCGGTGGAACCTCC	0	0.390401	0.20954	0.111027
tsrna-22751	GAGTCCTGCCGCGGTCGCCA	0.35708108	0.468481	0	0
tsrna-22750	GAGTCCTGCCGCGGTCGCC	0	0	0.069847	0
tsrna-22746	GAGTCCCGGCGGAGTCGCCA	0	0.23424	0	0
tsrna-22744	GAGTCCCATCTGGGTCGCCA	0	0.31232	0	0

tsrna-22743	GAGTCCCATCTGGGGTGCCA	0	0.07808	0	0
tsrna-22742	GAGTCCCACCAGAGTCGCCA	0	0.31232	0	0
tsrna-22739	GAGTATCCCCGCCTGTCACGCGGGAGACCG	0.99982703	5.309447	0.768315	0.111027
tsrna-22738	GAGTATCCCCGCCTGTCACGCGGGAGACC	1.14265946	4.216326	0.628621	0.222054
tsrna-22737	GAGTATCCCCGCCTGTCACGC	0	0.07808	0	0
tsrna-22736	GAGTATCCCCGCCTGTCACG	0.07141622	0	0	0
tsrna-22734	GAGTATCCCCGCCTGTCA	0	0	0	0.111027
tsrna-22733	GAGTATCCCCGCCTGTC	0	0	0.069847	0
tsrna-22731	GAGTAAATAATAGGAGCTTAAACCCCCTT	0.07141622	0	0	0
tsrna-22730	GAGTAAATAATAGGAGCTTAAACCC	0	0.07808	0	0
tsrna-22729	GAGTAAATAATAGGAGCTTAAACC	0.07141622	0	0.069847	0.111027
tsrna-22726	GAGTAAATAATAGGAGCTTA	0	0	0	0
tsrna-22725	GAGTAAATAATAGGAGCTT	0	0	0	0
tsrna-22721	GAGGTTCCGGGTTCGAGTCCCGGC	0	0	0	0
tsrna-22720	GAGGTTCAATTCCTCTTCT	0	0.15616	0	0
tsrna-22719	GAGGTTCAATTCCTCTT	0	0.07808	0	0
tsrna-22717	GAGGTGGCCGAGTGGTTAAGGC	0	0.07808	0	0
tsrna-22710	GAGGTCGATGGATCGAAACCATCCTCTGCT	0	0	0	0
tsrna-22709	GAGGTCGATGGATCGAAACCATCC	0	0.07808	0	0
tsrna-22707	GAGGTCGATGGATCGAAACCA	0	0	0	0
tsrna-22702	GAGGTCCTGGGTTCGATCCCC	0	0.07808	0	0
tsrna-22701	GAGGTCCCTGGTTCAAATCCGGGTGC	0	0.07808	0	0
tsrna-22696	GAGGTCCCGGGTTCGATCCCCGGCATC	0	0	0	0.111027
tsrna-22695	GAGGTCCCGGGTTCGATCCCCGGCAT	0	0.07808	0	0
tsrna-22689	GAGGTCCCGGGTTCGATCCCCA	0	0.07808	0	0
tsrna-22687	GAGGTCCCGGGTTCGATCCC	0	0	0	0.111027
tsrna-22684	GAGGTCCCGGGTTCAAATCCCGGACGAGCC	0.14283243	0.07808	0.069847	0
tsrna-22683	GAGGTCCCGGGTTCAAATCCCGGACGAGC	0	0.23424	0	0
tsrna-22678	GAGGTCCCCGGTTCAAA	0	0	0	0
tsrna-22677	GAGGTATGATTCTCGCTT	0	0.07808	0.069847	0
tsrna-22676	GAGGGTTCGAGTCCCTTCGTGGTCGCCA	0	0.31232	0	0
tsrna-22674	GAGGGTTCGAGTCCCTTCGTGGTCGC	0	0.07808	0	0
tsrna-22673	GAGGGTTCGAATCCCTTCGTGGTTGC	0	0	0	0
tsrna-22669	GAGGGTCCAGGGTTCATGTCCC	0	0	0	0
tsrna-22665	GAGGGTCCAGGGTTCAAGTCCCTGTTCGGGCGCCA	0.35708108	0.936961	0.279387	0
tsrna-22660	GAGGGTCCAGGGTTCAAGTCCCT	0	0	0	0
tsrna-22658	GAGGGTCCAGGGTTCAAGTC	0	0.07808	0	0
tsrna-22657	GAGGGTCCAGGGTTCA	0.07141622	0	0	0
tsrna-22656	GAGGCTTACGACCCCTTATTTACCCCA	0.07141622	0.23424	0	0
tsrna-22645	GAGGCGTGGGTTCGAATCCCACTTCTGAC	0	0	0	0
tsrna-22644	GAGGCGTGGGTTCGAATCCCACT	0	0	0.069847	0
tsrna-22643	GAGGCGTGGGTTCGAATCCCAC	0	0	0	0.111027
tsrna-22636	GAGGCCCCGGGTTCGATCCCCGGCATCTCC	0.07141622	0.15616	0	0
tsrna-22635	GAGGCCCCGGGTTCGATCCCCGGCATC	0	0.07808	0	0
tsrna-22626	GAGGCCCCGGGTTCAATCCCC	0	0.07808	0	0
tsrna-22623	GAGGACTGTAGATCCTTAGGTCGCTGGTTC	0.14283243	0.07808	0	0
tsrna-22622	GAGCTTAAACCCCCTTATTTCTACCA	0.14283243	0.15616	0.20954	0
tsrna-22621	GAGCTTAAACCCCCTTATTTCTACC	0.14283243	0.15616	0	0
tsrna-22620	GAGCTTAAACCCCCTTATTTCTAC	0	0.23424	0	0
tsrna-22619	GAGCTTAAACCCCCTTATTTCTA	0.14283243	0	0.069847	0
tsrna-22618	GAGCTTAAACCCCCTTATTTCT	0	0.15616	0	0
tsrna-22617	GAGCTTAAACCCCCTTATTTC	0.07141622	0.07808	0.139694	0
tsrna-22616	GAGCTTAAACCCCCTTATTT	0	0.15616	0	0
tsrna-22613	GAGCTTAAACCCCCTTA	0	0	0	0
tsrna-22611	GAGCTGGGGATTGTGGGTTCG	0	0	0	0
tsrna-22609	GAGCTGGGGATTGTGGGTT	0	0.23424	0	0
tsrna-22608	GAGCTGGGGATTGTGGGT	0	0.23424	0.069847	0
tsrna-22605	GAGCGTTAGACTGAAGATCTAAAGGTCCCT	0	0.390401	0.069847	0.222054
tsrna-22604	GAGCGTTAGACTGAAGATCTAAAGGTC	0	0.31232	0.069847	0.111027
tsrna-22598	GAGCGGTCTAAGGCGCTGGATT	0	0.07808	0	0.111027
tsrna-22587	GAGCCTCACCTGGAGCACCA	0	0	0.069847	0
tsrna-22585	GAGCCTACCCAGGGACGCCA	0	0.15616	0	0
tsrna-22584	GAGCCCGGTAATCGCATAAAACTTAAAACT	0.35708108	0.07808	0.349234	0
tsrna-22583	GAGCCCGGTAATCGCATAAAACTTAAAAC	0	0	0.069847	0

tsrna-22582	GAGCCCGGTAATCGCATAAAACTTAAAA	0	0.07808	0.139694	0
tsrna-22581	GAGCCCGGTAATCGCATAAAACTTAAA	0	0	0.139694	0
tsrna-22580	GAGCCCGGTAATCGCATAAAACTTAA	0	0.07808	0	0
tsrna-22579	GAGCCCGGTAATCGCATAAAACTTA	0	0	0.069847	0
tsrna-22576	GAGCCCGGTAATCGCATAAAAC	0	0	0.069847	0
tsrna-22575	GAGCCCGGTAATCGCATAAAA	0	0	0	0
tsrna-22572	GAGCCCGGTAATCGCATA	0	0	0	0
tsrna-22569	GAGCCCCAGTGGAACCACCA	0.07141622	0.23424	0.349234	0
tsrna-22568	GAGCCCCAGTGGAACCACC	0	0	0	0
tsrna-22567	GAGCCCCACGTTGGGCGCCA	0.64274595	1.873923	0.20954	0.222054
tsrna-22566	GAGCCCACCCAGGGACGCCA	0.21424865	4.762887	0	0.111027
tsrna-22565	GAGCCCACCCAGGGACGCC	0	0.23424	0	0
tsrna-22562	GAGCATGGACTCTTAATCCCAGGGTCGTG	0.07141622	0.07808	0.069847	0
tsrna-22557	GAGCACTCTGGACTCTGAATCCAGCGATCC	0	0.23424	0.069847	0
tsrna-22556	GAGCACTCTGGACTCTGAATCCAGCGATC	0.07141622	0.07808	0.069847	0
tsrna-22555	GAGCACTCTGGACTCTGAATCCAGCGAT	0.07141622	0.23424	0.069847	0.222054
tsrna-22554	GAGCACTCTGGACTCTGAATCCAGCGA	0	0.15616	0.139694	0
tsrna-22553	GAGCACTCTGGACTCTGAATCCAGCG	0.07141622	0.15616	0	0
tsrna-22552	GAGCACTCTGGACTCTGAATCCAGC	0.07141622	0	0	0
tsrna-22551	GAGCACTCTGGACTCTGAATCCAG	0	0	0	0
tsrna-22550	GAGCACTCTGGACTCTGAATCCA	0	0.390401	0	0
tsrna-22549	GAGCACTCTGGACTCTGAATCC	0	0.23424	0	0
tsrna-22548	GAGCACTCTGGACTCTGAATC	0.07141622	0	0	0
tsrna-22542	GAGATTTCAACTTAACTTGACCGCTCTGACCA	5.35621623	3.825925	3.841574	2.775678
tsrna-22541	GAGATTTCAACTTAACTTGACCGCTCTGACC	0.57132973	0.702721	0.488928	0.222054
tsrna-22540	GAGATTTCAACTTAACTTGACCGCTCTGAC	0.35708108	0.15616	0	0
tsrna-22539	GAGATTTCAACTTAACTTGACCGCTCTGA	0.07141622	0.23424	0.069847	0
tsrna-22538	GAGATTTCAACTTAACTTGACCGCTCTG	0	0.31232	0	0
tsrna-22537	GAGATTTCAACTTAACTTGACCGCTCT	0	0.07808	0	0
tsrna-22536	GAGATTTCAACTTAACTTGACCGCTC	0	0	0.069847	0
tsrna-22533	GAGATTTCAACTTAACTTGACCG	0	0	0.069847	0
tsrna-22527	GAGATGAAAACCTTTTTCCAAGGACACCA	0.14283243	0.390401	0.069847	0
tsrna-22526	GAGATGAAAACCTTTTTCCAAGGACACC	0.21424865	0.15616	0	0
tsrna-22525	GAGATGAAAACCTTTTTCCAAGGACA	0	0	0.069847	0
tsrna-22518	GAGAGGTCCCGGGTTCAAATCCCGGACGAG	0.07141622	0.07808	0.069847	0
tsrna-22517	GAGAGGTCCCGGGTTCAAA	0	0	0.069847	0
tsrna-22516	GAGAGGTCCCGGGTTCA	0.07141622	0	0	0
tsrna-22506	GAGACCGGGGTTCGATTCCCCGACGGGGAGCCA	0.07141622	1.639682	0	0
tsrna-22505	GAGACCGGGGTTCGATTCCCCGACGGGGAGCC	0	0.15616	0	0
tsrna-22504	GAGACCGGGGTTCGATTCCCCGACGGGGAGC	0	0.07808	0	0
tsrna-22501	GAGACCGGGGTTCGATTCCCCGACGGGG	0	0.07808	0	0
tsrna-22500	GAGACCGGGGTTCGATTCCCCGACGGG	0	0	0	0
tsrna-22486	GAGAATTTTGGATTCTCAGGGATG	0	0	0	0
tsrna-22485	GAGAATAGTTTAAATTAGAATCTTAGCTTTGG	0.78557838	0.23424	0.488928	0.222054
tsrna-22484	GAGAATAGTTTAAATTAGAATCTTAGCTTT	0.28566487	0.07808	0.279387	0.111027
tsrna-22476	GAGAACCAACACCTCTTTACAGTG	0	0	0.069847	0
tsrna-22468	GAGAAAGCTCACAAGAACTGCTAACTCATGCCCCCATGTCTA	63.0605191	163.734	51.05801	50.51735
tsrna-22467	GAGAAAGCTCACAAGAACTGCTAACTCATGCCCCCATGTCTA	64.4174272	167.3257	50.42939	48.51886
tsrna-22466	GAGAAAGCTCACAAGAACTGCTAACTCATGCCCCCATGTCTA	65.6315029	166.3887	50.56909	50.7394
tsrna-22465	GAGAAAGCTCACAAGAACTGCTAACTCATGCCCCCATGTCT	59.5611245	164.4367	47.28629	48.07475
tsrna-22464	GAGAAAGCTCACAAGAACTGCTAACTCATGCCCCCA	60.4181191	164.0463	44.00349	48.85194
tsrna-22463	GAGAAAGCTCACAAGAACTGCTAACTCATGCCCCC	62.8462705	162.1724	48.61338	52.84892
tsrna-22462	GAGAAAGCTCACAAGAACTGCTAACTCATGCCCC	62.4177732	159.2834	48.54353	51.07248
tsrna-22461	GAGAAAGCTCACAAGAACTGCTAACTCATGCCC	59.9182056	160.2204	43.93364	44.96599
tsrna-22460	GAGAAAGCTCACAAGAACTGCTAACTCATGCC	56.0617299	162.4847	48.40384	52.62686
tsrna-22459	GAGAAAGCTCACAAGAACTGCTAACTCATG	61.4179461	161.4697	45.88935	52.84892
tsrna-22458	GAGAAAGCTCACAAGAACTGCTAACTCAT	56.7758921	162.7189	48.26414	48.74091
tsrna-22457	GAGAAAGCTCACAAGAACTGCTAACTCA	60.9180326	158.5026	47.35613	51.84967
tsrna-22456	GAGAAAGCTCACAAGAACTGCTAACTC	59.7039569	160.4546	45.8195	52.95994
tsrna-22455	GAGAAAGCTCACAAGAACTGCTAACT	58.418465	155.0671	45.8195	50.29529
tsrna-22454	GAGAAAGCTCACAAGAACTGCTAAC	56.7758921	140.0757	41.69854	47.96372
tsrna-22453	GAGAAAGCTCACAAGAACTGCTAA	21.781946	78.00203	16.55369	15.43277
tsrna-22452	GAGAAAGCTCACAAGAACTGCTA	20.0679568	70.2721	14.45829	12.10196
tsrna-22451	GAGAAAGCTCACAAGAACTGCT	15.5687352	68.78858	14.80752	14.43353

tsrna-22450	GAGAAAGCTCACAAGAACTGC	5.78471353	18.58307	4.400349	2.553624
tsrna-22449	GAGAAAGCTCACAAGAACTG	4.57063785	15.38178	3.981268	1.332326
tsrna-22448	GAGAAAGCTCACAAGAACT	4.64205407	12.64898	4.260655	1.110271
tsrna-22447	GAGAAAGCTCACAAGAAC	0	0.546561	0.139694	0
tsrna-22446	GAGAAAGCTCACAAGAA	0	0	0	0
tsrna-22445	GAGAAAGCTCACAAGA	0	0.07808	0	0
tsrna-22444	GACTTTTTCTCTGACCA	0.21424865	0.07808	0.279387	0.333081
tsrna-22443	GACTTTTTCTCTGACC	0	0	0	0
tsrna-22442	GACTTTTAATCTGAGGGTCCAGG	0.64274595	2.654724	0.279387	0.333081
tsrna-22441	GACTTTTAATCTGAGGGTCCAG	0.4284973	2.810884	0.279387	0
tsrna-22440	GACTTTTAATCTGAGGGTCC	0.4284973	1.873923	0.20954	0
tsrna-22439	GACTTTGAATCCAGCGATCCGAGTTC	0.14283243	0.390401	0.139694	0
tsrna-22438	GACTTTGAATCCAGCGATCCGAGT	0.21424865	0.15616	0.139694	0
tsrna-22437	GACTTTGAATCCAGCGATCCGAG	0.07141622	0	0.139694	0
tsrna-22436	GACTTTGAATCCAGCGATCCGA	0.07141622	0.15616	0	0
tsrna-22435	GACTTTGAATCCAGCGATCCG	0.07141622	0	0.069847	0
tsrna-22434	GACTTTGAATCCAGCGATCC	0.21424865	0.07808	0.20954	0.111027
tsrna-22433	GACTTTGAATCCAGCGATC	0.07141622	0.07808	0	0
tsrna-22432	GACTTTGAATCCAGCGA	0	0.31232	0.069847	0
tsrna-22431	GACTTTGAATCCAGCG	0.07141622	0	0	0
tsrna-22430	GACTTTGAATCCAGCAATCCGA	0	0.31232	0.069847	0
tsrna-22429	GACTTTGAATCCAGCAATCCG	0	0.15616	0.069847	0.111027
tsrna-22425	GACTTCGGATCAGAAGATTGAGGGT	0.14283243	0.546561	0.349234	0.222054
tsrna-22413	GACTGTAGATCCTTAGGTCGCTGG	0	0	0	0.111027
tsrna-22410	GACTGTAAATCTAAAGACAGGGGTTAGGCC	0	0.07808	0	0
tsrna-22409	GACTGTAAATCTAAAGACAGGGGTTAGG	0	0.07808	0	0
tsrna-22408	GACTGTAAATCTAAAGACAGGGGTTAG	0.07141622	0	0	0
tsrna-22407	GACTGTAAATCTAAAGACAGGGGTT	0	0	0	0
tsrna-22405	GACTGTAAATCTAAAGACAGGGG	0	0	0.069847	0
tsrna-22404	GACTGTAAATCTAAAGACAGGG	0.07141622	0	0	0
tsrna-22398	GACTGCAGATCAAGAGGTCCCTGGT	0.07141622	0.624641	0.069847	0.444109
tsrna-22397	GACTGCAGATCAAGAGGTCCCCGGT	0.14283243	0.31232	0.069847	0.111027
tsrna-22392	GACTGAAGATCTAAAGGTCCCTGGT	0.8569946	0.936961	0.628621	0.333081
tsrna-22391	GACTCTTAATCTCAGGGTC	0.07141622	0	0.069847	0
tsrna-22390	GACTCTGAATCCAGCGATCCGAGTTCAAGT	0	0.07808	0	0.111027
tsrna-22389	GACTCTGAATCCAGCGATCCGAGTTCAA	0	0.23424	0.139694	0
tsrna-22388	GACTCTGAATCCAGCGATCCGAGTTCA	0	0.31232	0	0
tsrna-22387	GACTCTGAATCCAGCGATCCGAGTTC	0	0.07808	0	0
tsrna-22386	GACTCTGAATCCAGCGATCCGAGT	0	0.15616	0	0
tsrna-22385	GACTCTGAATCCAGCGATCCGAG	0	0	0	0
tsrna-22384	GACTCTGAATCCAGCGATCCGA	0	0.07808	0	0
tsrna-22383	GACTCTGAATCCAGCGATCCG	0	0	0	0.111027
tsrna-22382	GACTCTGAATCCAGCGATCC	0	0.07808	0	0
tsrna-22380	GACTCTGAATCCAGCGAT	0	0	0	0.111027
tsrna-22377	GACTCGAAATCCAATGGGG	0.07141622	1.015041	0	0
tsrna-22376	GACTCCTGGCTGGCTCGCCA	0.71416216	4.762887	0	0.333081
tsrna-22375	GACTCCTGGCTGGCTCGCC	0	0	0	0
tsrna-22372	GACTCCCGGTGTGGGAACCA	0.07141622	0.31232	0.069847	0.111027
tsrna-22370	GACTCCCGGTGTGGGAAC	0	0	0	0
tsrna-22367	GACTCCCGGTATGGGAACCA	0	0	0	0.111027
tsrna-22365	GACTCCAGATCAGAAGGTTGCGTGTTCA	0.21424865	0.31232	0	0
tsrna-22364	GACTCCAGATCAGAAGGTTGCGTGTT	0	0.31232	0	0
tsrna-22363	GACTCCAGATCAGAAGGTTGCGTGT	0	0.07808	0	0
tsrna-22362	GACTCCAGATCAGAAGGTTGCGTG	0.07141622	0	0	0
tsrna-22355	GACTCCAGATCAGAAGGCTGCGTGTTCG	0.07141622	0	0	0
tsrna-22354	GACTCCAGATCAGAAGGCTGCGTGTT	0	0	0.139694	0.111027
tsrna-22339	GACTCAAGTTCTGGTCTCCGGATGGAGGCG	0.07141622	0.07808	0.069847	0
tsrna-22338	GACTCAAGTTCTGGTCTCCGGA	0.14283243	0.15616	0	0.111027
tsrna-22337	GACTCAAGTTCTGGTCTCCGG	0.21424865	0.23424	0	0.222054
tsrna-22336	GACTCAAGTTCTGGTCTCCG	0.21424865	0.23424	0	0
tsrna-22335	GACTCAAGTTCTGGTCTCCAATGGAGGCGT	0	0.390401	0.069847	0
tsrna-22334	GACTCAAGTTCTGGTCTCC	0	0	0	0
tsrna-22333	GACTCAAGTTCTGGTCTC	0.14283243	0.15616	0.069847	0.111027
tsrna-22332	GACTCAAGTTCTGGTC	0	0.31232	0	0.111027

tsrna-22331	GACTACGGATCAGAAGATTCTAGGTTCGAC	0.28566487	0.468481	0	0.111027
tsrna-22330	GACGGGCTCACATCACCCCATAAACACCA	0.78557838	0.15616	0.488928	0.77719
tsrna-22329	GACGGGCTCACATCACCCCATAAACACC	0	0.07808	0.069847	0.222054
tsrna-22317	GACGAGGTGGCCGAGTGGTTAAGGCGATGG	1.14265946	4.294406	0.698468	0.666163
tsrna-22316	GACGAGGTGGCCGAGTGGTTAAGGCGATG	1.21407568	3.669765	0.488928	0.888217
tsrna-22315	GACGAGGTGGCCGAGTGGTTAAGGCGA	0.99982703	4.372486	0.698468	0.999244
tsrna-22314	GACGAGGTGGCCGAGTGGTTAAGGCG	1.2854919	3.357445	0.698468	0.666163
tsrna-22313	GACGAGGTGGCCGAGTGGTTAAGGC	1.2854919	3.904006	1.117549	0.77719
tsrna-22312	GACGAGGTGGCCGAGTGGTTAAGG	0.4284973	4.919047	0.279387	1.221299
tsrna-22311	GACGAGGTGGCCGAGTGGTTAAG	0.99982703	5.153287	0.977855	0.666163
tsrna-22310	GACGAGGTGGCCGAGTGGTTAA	0.78557838	3.669765	0.628621	0.888217
tsrna-22309	GACGAGGTGGCCGAGTGGTTA	0.71416216	3.201285	0.768315	0.999244
tsrna-22308	GACGAGGTGGCCGAGTGGTT	0.78557838	4.450566	0.698468	0.666163
tsrna-22307	GACGAGGTGGCCGAGTGGT	1.14265946	3.435525	0.698468	0.555136
tsrna-22306	GACGAGGTGGCCGAGTGG	0.64274595	3.591685	0.419081	0.333081
tsrna-22305	GACGAGGTGGCCGAGTG	0.78557838	4.684807	0.419081	0.333081
tsrna-22304	GACGAGGTGGCCGAGT	1.21407568	3.513605	0.628621	0.444109
tsrna-22303	GACCTCGTGGCGCAATGGTAGCGCGTCTGACTCC	0.07141622	0.15616	0	0
tsrna-22302	GACCTCGTGGCGCAATGGTAGCGCGTCTGACT	0	0.07808	0	0
tsrna-22300	GACCTCGTGGCGCAATGGTAGCGCGTCTG	0	0	0.069847	0
tsrna-22299	GACCTCGTGGCGCAATGGTAGCGC	0	0.07808	0.069847	0
tsrna-22296	GACCTCGTGGCGCAATGGTAG	0	0	0.069847	0
tsrna-22293	GACCTCGTGGCGCAATGG	0	0	0	0.111027
tsrna-22290	GACCTCGTGGCGCAACGGTAGCGCGTCTGACTCC	0.14283243	0.546561	0.069847	0.222054
tsrna-22289	GACCTCGTGGCGCAACGGTAGCGCGTCTGACT	0	0.23424	0	0
tsrna-22285	GACCTCGTGGCGCAACGGTAGCGCG	0	0.07808	0	0
tsrna-22272	GACCGGGGTTCGATTCCCCGACGGGGAGCCA	0	1.093122	0.069847	0
tsrna-22271	GACCGGGGTTCGATTCCCCGACGGGGAGCC	0	0	0	0.111027
tsrna-22270	GACCGGGGTTCGATTCCCCGACGGGGAGC	0	0.15616	0	0
tsrna-22269	GACCGGGGTTCGATTCCCCGACGGGGAG	0.07141622	0	0	0
tsrna-22267	GACCGGGGTTCGATTCCCCGACGGGG	0	0.07808	0	0
tsrna-22266	GACCGGGGTTCGATTCCCCGACGGG	0	0	0	0
tsrna-22252	GACCGCGTGGCCTAATGGATAAGGCGTCTGACTT	0	0	0.069847	0
tsrna-22251	GACCGCGTGGCCTAATGGATAAGGCGTCTGACT	0	0.07808	0	0
tsrna-22237	GACCCGGGTTCGATTCCCGGCCAACGCACC	0.8569946	1.639682	0	0.222054
tsrna-22230	GACCCCTTATTTACCCCA	0	0.07808	0	0
tsrna-22227	GACCCCCGGCTCCTCCACCA	0.92841081	1.952003	0.279387	0.222054
tsrna-22226	GACCCAGTGGCCTAATGGATAAGGCATCAGCCTCC	0.35708108	0.31232	0.069847	0
tsrna-22225	GACCCAGTGGCCTAATGGATAAGGCATCAGCCTC	0.28566487	0.23424	0.279387	0.111027
tsrna-22224	GACCCAGTGGCCTAATGGATAAGGCATCAGCCT	0.28566487	0.23424	0.349234	0
tsrna-22223	GACCCAGTGGCCTAATGGATAAGGCATCAGCC	0.4284973	0.390401	0.139694	0
tsrna-22222	GACCCAGTGGCCTAATGGATAAGGCATCAGC	0.28566487	0.15616	0.20954	0
tsrna-22221	GACCCAGTGGCCTAATGGATAAGGCATCAG	0.07141622	0.390401	0.069847	0
tsrna-22220	GACCCAGTGGCCTAATGGATAAGGCATCA	0.14283243	0.07808	0.349234	0
tsrna-22219	GACCCAGTGGCCTAATGGATAAGGCATC	0	0.15616	0.139694	0.111027
tsrna-22218	GACCCAGTGGCCTAATGGATAAGGCAT	0	0	0	0
tsrna-22210	GACCCAGTGGCCTAATGGA	0	0	0	0
tsrna-22204	GACCACGTGGCCTAATGGATAAG	0	0	0	0
tsrna-22198	GACCAAGAGCCTTCAAAGCCCTC	0	0.07808	0	0
tsrna-22195	GACCAAGAGCCTTCAAAGCC	0	0.07808	0	0
tsrna-22193	GACCAAGAGCCTTCAAA	0.07141622	0	0	0
tsrna-22192	GACATATGTCCGCGTGGGT	0.35708108	0.468481	0.20954	0
tsrna-22191	GACATATGTCCGCGTGGG	0.07141622	0	0	0
tsrna-22190	GACAGGGGTTAGGCCTCTTTTTACCACCA	0.07141622	0.15616	0.139694	0.111027
tsrna-22189	GACAGGGGTTAGGCCTCTTTTTACCACC	0	0.07808	0.139694	0
tsrna-22188	GACAGGGGTTAGGCCTCTTTTTACCAC	0	0.07808	0.139694	0
tsrna-22187	GACAGGGGTTAGGCCTCTTTTTACCA	0	0.07808	0	0
tsrna-22186	GACAGGGGTTAGGCCTCTTTTTACC	0	0	0.139694	0.111027
tsrna-22185	GACAGGGGTTAGGCCTCTTT	0.07141622	0	0	0
tsrna-22184	GACAGGGGTTAGGCCTCTT	0	0	0	0
tsrna-22183	GACAGGGGTTAGGCCTCT	0	0.07808	0	0
tsrna-22180	GACAACATTCAAAAAAGAGTACCA	0	0	0	0
tsrna-22174	GACAACAGAGGCTTACGACCCCTTATTTAC	0	0	0	0
tsrna-22169	GAATTTTGGATTCTCAGGGATG	0	0	0	0

tsrna-22166	GAATTGCAAATTCGAAGAAGCAGCTTCAAACCTGCCGGGGCT	0.07141622	0.23424	0.279387	0
tsrna-22165	GAATTGCAAATTCGAAGAAGCAGCTTCAAACCTGCCGGGGCT	0.07141622	0.07808	0.069847	0
tsrna-22164	GAATTGCAAATTCGAAGAAGCAGCTTCAAACCTGCCGGGGCT	0	0.15616	0.069847	0
tsrna-22163	TGGTCTAGTGGCTAGGATTCGGCGC	0.71416216	2.420483	0.488928	3.552868
tsrna-22162	TGGTCTAGTGGCTAGGATTCGGCG	0.71416216	2.342403	0.419081	2.775678
tsrna-22161	TGGTCTAGTGGCTAGGATTCGGC	0.49991351	1.483522	0.279387	1.110271
tsrna-22160	TGGTCTAGTGGCTAGGATTCGG	0	0.15616	0.069847	0
tsrna-22159	TGGTCTAGTGGCTAGGATTCG	0.07141622	0.07808	0	0.111027
tsrna-22158	TGGTCTAGGGGTATGATTCTCGGTT	0.14283243	0.15616	0.279387	0.111027
tsrna-22157	TGGTCTAGGGGTATGATTCTCGGT	0.57132973	0.390401	0.139694	0
tsrna-22156	TGGTCTAGGGGTATGATTCTCGG	0.07141622	0.07808	0.069847	0
tsrna-22155	TGGTCTAGGGGTATGATTCTCGCTTC	1.35690811	1.249282	1.047702	1.998488
tsrna-22154	TGGTCTAGGGGTATGATTCTCGCTT	1.42832433	2.576644	0.977855	1.887461
tsrna-22153	TGGTCTAGGGGTATGATTCTCGCT	1.21407568	1.249282	0.628621	1.55438
tsrna-22152	TGGTCTAGGGGTATGATTCTCGC	0.92841081	0.702721	0.488928	1.221299
tsrna-22151	TGGTCTAGGGGTATGATTCTCG	0	0.07808	0	0.111027
tsrna-22150	TGGTCTAGGGGTATGATTCTC	0	0	0	0
tsrna-22149	TGGTCTAGGGGTATGATTCT	0	0	0	0
tsrna-22144	TGGTCTAGCGGTTAGGATTCCTGGTTTTCACCCAG	0.99982703	1.483522	0.977855	0.555136
tsrna-22143	TGGTCTAGCGGTTAGGATTCCTGGTTTTCACCC	0.92841081	1.015041	0.628621	0.222054
tsrna-22142	TGGTCTAGCGGTTAGGATTCCTGGTTTTCA	0.8569946	0.624641	0.419081	0.444109
tsrna-22141	TGGTCTAGCGGTTAGGATTCCTGGTTTTC	0.8569946	0.31232	0.488928	0.111027
tsrna-22140	TGGTCTAGCGGTTAGGATTCCTGGTTTT	0.35708108	0.390401	0.20954	0.333081
tsrna-22139	TGGTCTAGCGGTTAGGATTCCTGGTTT	0.35708108	0.702721	0	0.111027
tsrna-22138	TGGTCTAGCGGTTAGGATTCCTGGTT	0.28566487	0.702721	0.20954	0.222054
tsrna-22137	TGGTCTAGCGGTTAGGATTCCTGGT	0.49991351	0.31232	0.139694	0.111027
tsrna-22136	TGGTCTAGCGGTTAGGATTCCTGG	0.35708108	0.390401	0.139694	0
tsrna-22135	TGGTCTAGCGGTTAGGATTCCTG	0.07141622	0.15616	0.139694	0
tsrna-22134	TGGTCTAGCGGTTAGGATTCCT	0.07141622	0.07808	0.20954	0
tsrna-22128	TGGTCTAAGGCGCTGGATTTAG	0.21424865	0.390401	0.069847	0
tsrna-22127	TGGTCTAAGGCGCTGGATTT	0.14283243	0.15616	0.139694	0
tsrna-22126	TGGTCTAAGGCGCTGGATT	0	0.15616	0	0
tsrna-22125	TGGTCTAAGGCGCTGGAT	0	0.07808	0	0
tsrna-22124	TGGTCTAAGGCGCTGGA	0.07141622	0.07808	0	0
tsrna-22122	TGGTCTAAGGCGCCAGACTCAAG	0.28566487	0.07808	0.139694	0.555136
tsrna-22121	TGGTCTAAGGCGCCAGACTCA	0.21424865	0	0.139694	0.111027
tsrna-22120	TGGTCTAAGGCGCCAGACTC	0.14283243	0.07808	0.139694	0.222054
tsrna-22117	TGGTCGTGGTTGTAGTCCGTGCGAGAATACCA	1.49974054	3.904006	2.374791	1.55438
tsrna-22116	TGGTCGTGGTTGTAGTCCGTGCGAGAATACC	0.4284973	0.546561	0.419081	0.111027
tsrna-22115	TGGTCGTGGTTGTAGTCCGTGCGAGAATAC	0.28566487	0.546561	0.558774	0.333081
tsrna-22114	TGGTCGTGGTTGTAGTCCGTGCGAGAATA	0	0.31232	0.139694	0.111027
tsrna-22113	TGGTCGTGGTTGTAGTCCGTGCGAGAAT	0	0.23424	0.20954	0.111027
tsrna-22112	TGGTCGTGGTTGTAGTCCGTGCGAGAA	0	0.31232	0.139694	0.111027
tsrna-22111	TGGTCGTGGTTGTAGTCCGTGCGAGA	0.07141622	0.390401	0.139694	0.444109
tsrna-22110	TGGTCGTGGTTGTAGTCCGTGCGAG	0	0.07808	0.139694	0
tsrna-22109	TGGTCGTGGTTGTAGTCCGTGCGA	0	0.07808	0	0
tsrna-22108	TGGTCGTGGTTGTAGTCCGTGCG	0	0.07808	0.069847	0
tsrna-22107	TGGTCGTGGTTGTAGTCCGTGC	0.07141622	0	0.069847	0
tsrna-22106	TGGTCGTGGTTGTAGTCCGTG	0.14283243	0.07808	0	0
tsrna-22105	TGGTCGTGGTTGTAGTCCGT	0	0	0	0
tsrna-22104	TGGTCGTGGTTGTAGTCCG	0	0	0.069847	0
tsrna-22103	TGGTCGTGGTTGTAGTCC	0	0	0	0
tsrna-22100	TGGTCATCACGTTCGCC	0	0	0.069847	0
tsrna-22098	TGGTCAGCACTCTGGACTCTGAATCCA	0.07141622	0.31232	0.139694	0
tsrna-22097	TGGTCAGCACTCTGGACTCTGAATC	0	0.07808	0	0
tsrna-22093	TGGTCAGCACTCTGGACT	0	0	0	0
tsrna-22091	TGGTATGATTCTCGCT	0.07141622	0.07808	0	0.222054
tsrna-22088	TGGTATCATGCAAGATTC	0	0.07808	0	0
tsrna-22086	TGGTATATAGTTTAAACAAAACGAATGATTTCGACTCA	0.07141622	0.15616	0.069847	0
tsrna-22085	TGGTATATAGTTTAAACAAAACGAATGATTTCGACTC	0	0.07808	0	0
tsrna-22084	TGGTATATAGTTTAAACAAAACGAATGATTTCGACT	0	0	0	0
tsrna-22083	TGGTATATAGTTTAAACAAAACGAATGATT	0	0	0.069847	0
tsrna-22076	TGGTATAGTGGTTAGCATAGCTG	0.07141622	0	0	0
tsrna-22074	TGGTATAGTGGTGAGCATAGCTGC	0	0.15616	0	0.111027

tsrna-22073	TGGTATAGTGGTGAGCATAGCTG	0	0	0	0
tsrna-22072	TGGTATAGTGGTGAGCATAGC	0	0.07808	0	0
tsrna-22070	TGGTATAGTGGTAAGCATAGCTGC	0	0.15616	0	0
tsrna-22069	TGGTATAGTGGTAAGCATAGCTG	0.07141622	0	0	0
tsrna-22050	TGGTAGAGCGCTCGCTTAGC	0.07141622	0	0.069847	0.111027
tsrna-22049	TGGTAGAGCGCTCGCTTAG	0	0	0.069847	0
tsrna-22048	TGGTAGAGCGCGTGCTTCGCA	0.4284973	2.498564	0.279387	0
tsrna-22047	TGGTAGAGCGCGTGCTTCGC	0.57132973	2.030083	0.419081	0
tsrna-22046	TGGTAGAGCGCGTGCTTAGC	0.92841081	2.967044	0.419081	0.111027
tsrna-22045	TGGTAGAGCGCGTGCTTAG	0.49991351	1.639682	0.139694	0
tsrna-22044	TGGTAGAGCGCGTGCT	0.4284973	1.249282	0.139694	0
tsrna-22042	TGGTAGAGCATTTGACTGCAGATCAAGAGG	0	0	0	0
tsrna-22041	TGGTAGAGCATTTGACTGCAGATCAAG	0	0.15616	0	0
tsrna-22040	TGGTAGAGCATTTGACTGCAGATCA	0	0.07808	0	0
tsrna-22039	TGGTAGAGCATTTGACTGCAGATC	0	0.07808	0.069847	0
tsrna-22037	TGGTAGAGCATTTGACTGCA	0	0.15616	0	0
tsrna-22036	TGGTAGAGCATTTGACTGC	0.07141622	0	0	0
tsrna-22035	TGGTAGAGCATTTGACTG	0	0.07808	0	0
tsrna-22034	TGGTAGAGCATTTGACT	0.07141622	0	0	0
tsrna-22032	TGGTAGAGCATGGGACTCT	0.21424865	0.390401	0.20954	0
tsrna-22028	TGGTAGAATTCTTGCCTGCC	0	0	0.069847	0
tsrna-22025	TGGTAGAATTCTCGCCTGCCACGCGGGAGGCCCGGGTTT	9.64118922	14.5229	9.429319	2.997733
tsrna-22024	TGGTAGAATTCTCGCCTGCCACGCGGGAGGCCCGGGTT	9.42694057	14.44482	8.800698	2.997733
tsrna-22023	TGGTAGAATTCTCGCCTGCCACGCGGGAGGCCCGGGT	8.85561084	12.33666	7.264068	2.33157
tsrna-22022	TGGTAGAATTCTCGCCTGCCACGCGGGAGGCCCGGG	5.28480002	11.3997	3.073259	2.997733
tsrna-22021	TGGTAGAATTCTCGCCTGCCACGCGGGAGGCCCGG	5.71329731	11.16546	3.911421	1.998488
tsrna-22020	TGGTAGAATTCTCGCCTGCCACGCGGGAGGCCCG	4.99913515	10.22849	3.003413	2.442597
tsrna-22019	TGGTAGAATTCTCGCCTGCCACGCGGGAGGCCC	5.42763245	9.760014	3.143106	2.220543
tsrna-22018	TGGTAGAATTCTCGCCTGCCACGCGGGAGGCC	4.3563892	9.916174	2.724025	1.998488
tsrna-22017	TGGTAGAATTCTCGCCTGCCACGCGGGAGG	5.35621623	9.291533	2.514485	2.33157
tsrna-22016	TGGTAGAATTCTCGCCTGCCACGCGGGAG	3.71364325	11.3997	2.374791	2.109516
tsrna-22015	TGGTAGAATTCTCGCCTGCCACGCGGGA	3.78505947	9.291533	2.025557	2.220543
tsrna-22014	TGGTAGAATTCTCGCCTGCCACGCGGG	4.92771893	8.744972	2.793872	2.442597
tsrna-22013	TGGTAGAATTCTCGCCTGCCACGCGG	4.28497299	8.823052	2.933566	2.997733
tsrna-22012	TGGTAGAATTCTCGCCTGCCACGCG	4.3563892	7.41761	2.724025	3.10876
tsrna-22011	TGGTAGAATTCTCGCCTGCCACGC	4.28497299	6.480649	2.235098	2.442597
tsrna-22010	TGGTAGAATTCTCGCCTGCCACG	3.28514596	7.02721	1.396936	1.776434
tsrna-22009	TGGTAGAATTCTCGCCTGCCAC	1.07124325	2.342403	0.908008	0.444109
tsrna-22008	TGGTAGAATTCTCGCCTGCCA	0.71416216	2.108163	0.488928	0.111027
tsrna-22007	TGGTAGAATTCTCGCCTGCC	0.71416216	2.810884	0.768315	0.111027
tsrna-22006	TGGTAGAATTCTCGCCTGC	0.35708108	0.23424	0.349234	0.444109
tsrna-22005	TGGTAGAATTCTCGCCTG	0	0.31232	0.069847	0
tsrna-22004	TGGTAGAATTCTCGCCTCCCACGCGGGAGACCCGGGT	0.21424865	1.249282	0.20954	1.221299
tsrna-22003	TGGTAGAATTCTCGCCTCCCACGCGGGAGACCC	0.4284973	1.015041	0.279387	1.332326
tsrna-22002	TGGTAGAATTCTCGCCTCCCACGCGGGAG	0.4284973	0.858881	0.20954	0.888217
tsrna-22001	TGGTAGAATTCTCGCCTCCCACG	0.35708108	0.702721	0.139694	0.666163
tsrna-22000	TGGTAGAATTCTCGCCTCCCA	0.21424865	1.405442	0.069847	0.111027
tsrna-21999	TGGTAGAATTCTCGCCTCCC	0.21424865	1.015041	0.20954	0.999244
tsrna-21998	TGGTAGAATTCTCGCCTCC	0.14283243	0.858881	0.069847	0
tsrna-21997	TGGTAGAATTCTCGCCTC	0.14283243	0.07808	0.139694	0.222054
tsrna-21996	TGGTAGAATTCTCGCCT	0.14283243	0.07808	0	0
tsrna-21995	TGGTAGAATTCTCGCC	0.07141622	0	0	0
tsrna-21994	TGGTAGAATTCTCACC	0.07141622	0.07808	0	0
tsrna-21985	TGGTAAGCACTCTGGACTCTGAATCCA	0.07141622	0.07808	0.139694	0
tsrna-21984	TGGTAAGCACTCTGGACTCTGAATCC	0.14283243	0.15616	0.139694	0.222054
tsrna-21983	TGGTAAGCACTCTGGACTCTGAATC	0	0	0.139694	0
tsrna-21973	TGGGTTTAAGTCCCATTGGTCTAGCCA	0.07141622	0	0	0.111027
tsrna-21972	TGGGTTTAAGTCCCATTGGTCTAGCC	0.14283243	0.07808	0	0.111027
tsrna-21971	TGGGTTTAAGTCCCATTGGTCTAGC	0	0	0.069847	0
tsrna-21968	TGGGTTTAAGTCCCATTGGTCT	0	0	0	0
tsrna-21967	TGGGTTTAAGTCCCATTGGTC	0.07141622	0	0.069847	0
tsrna-21962	TGGGTTCGATTCTCATAGTCCTAGCCA	0.14283243	0.31232	0	0.222054
tsrna-21961	TGGGTTCGATTCTCATAGTCCTAGCC	0.14283243	0.390401	0	0.111027
tsrna-21960	TGGGTTCGATTCTCATAGTCCTAGC	0.14283243	0.31232	0.069847	0.111027

tsrna-21959	TGGGTTCGATTCTCATAGTCCTAG	0.07141622	0.468481	0	0
tsrna-21958	TGGGTTCGATTCTCATAGTCCTA	0	0.07808	0	0
tsrna-21957	TGGGTTCGATTCTCATAGTCC	0	0.15616	0.069847	0
tsrna-21956	TGGGTTCGATTCTCATAGTC	0	0	0.069847	0
tsrna-21955	TGGGTTCGATCCCCAGTACCTCCACCA	2.57098379	4.216326	0.977855	1.443353
tsrna-21954	TGGGTTCGAGTCCCATCTGGGTCGCCA	0.07141622	0.31232	0	0
tsrna-21953	TGGGTTCGAGTCCCATCTGGGTCGCC	0	0	0.069847	0
tsrna-21950	TGGGTTCGAGTCCCACCAGAGTCGCCA	0	0.546561	0	0
tsrna-21948	TGGGTTCGAGCCCCAGTGGAACCACCA	2.21390271	2.810884	0.838162	1.665407
tsrna-21947	TGGGTTCGAGCCCCAGTGGAACCA	0.07141622	0.07808	0	0
tsrna-21946	TGGGTTCGAGCCCCAGTGGAACC	0.07141622	0	0.069847	0
tsrna-21945	TGGGTTCGAGCCCCACGTTGGGCGCCA	0.64274595	15.53794	0.488928	0.444109
tsrna-21944	TGGGTTCGAATCCCATCCTCGTCGCCA	0.4284973	1.405442	0.139694	0.111027
tsrna-21943	TGGGTTCGAATCCCATCCTCGTCGCC	0.07141622	0.15616	0	0
tsrna-21941	TGGGTTCGAATCCCATCCTCGTCG	0.07141622	0	0	0
tsrna-21940	TGGGTTCGAATCCCAGCGGTGCCTCCA	1.71398919	4.684807	0.838162	0.333081
tsrna-21939	TGGGTTCGAATCCCAGCGGGGCCTCCA	0	0.546561	0	0.111027
tsrna-21938	TGGGTTCGAATCCCACTTCTGACACCA	0.57132973	2.342403	0.349234	0.555136
tsrna-21937	TGGGTTCGAATCCCACTTCTGACAC	0	0	0	0
tsrna-21936	TGGGTTCGAATCCCACCGCTGCCACC	0.07141622	0.390401	0	0
tsrna-21935	TGGGTTCGAATCCCACCAGAGTCGCCA	0.4284973	0.15616	0	0.222054
tsrna-21934	TGGGTTCGAATCCCACCAGAGTCG	0	0.07808	0	0
tsrna-21933	TGGGTGGTTCAGTGGTAGAATTCTCGCCTGCC	1.07124325	2.888964	1.187396	0.444109
tsrna-21932	TGGGTGGTTCAGTGGTAGAATTCTCGCCTG	0.49991351	1.561602	0.139694	0.77719
tsrna-21931	TGGGTGGTTCAGTGGTAGAATTCTCGCCT	0.8569946	1.561602	0.279387	0.333081
tsrna-21930	TGGGTGGTTCAGTGGTAGAATTCTCGCC	0.78557838	0.624641	0	0.222054
tsrna-21929	TGGGTGGTTCAGTGGTAGAATTCTCGC	0.92841081	0.15616	0.069847	0.111027
tsrna-21928	TGGGTGGTTCAGTGGTAGAATTCTCG	0.14283243	0.23424	0.20954	0.222054
tsrna-21927	TGGGTGGTTCAGTGGTAGAATTCTC	0.14283243	0.390401	0.139694	0.111027
tsrna-21926	TGGGTGGTTCAGTGGTAGAATTCT	0.21424865	0.390401	0.069847	0.111027
tsrna-21925	TGGGTGGTTCAGTGGTAGAATTC	0	0.15616	0.139694	0
tsrna-21924	TGGGTGGTTCAGTGGTAGAATT	0.49991351	0.15616	0.279387	0
tsrna-21923	TGGGTGGTTCAGTGGTAGAAT	0	0.31232	0.069847	0.111027
tsrna-21922	TGGGTGGTTCAGTGGTAGAA	0.07141622	0.07808	0	0.111027
tsrna-21921	TGGGTGGTTCAGTGGTAGA	0	0	0	0
tsrna-21920	TGGGTGGTTCAGTGGTAG	0.14283243	0	0	0
tsrna-21919	TGGGTGGTTCAGTGGTA	0	0	0	0
tsrna-21917	TGGGTGCTAATGGTGGAGTTAAAGACTTTT	0.21424865	0.546561	0.419081	0.222054
tsrna-21916	TGGGTGCTAATGGTGGAGTTAAAGACT	0.14283243	0.468481	0.20954	0.111027
tsrna-21915	TGGGTGCTAATGGTGGAGTTAAAGAC	0.07141622	0.31232	0	0
tsrna-21914	TGGGTGCTAATGGTGGAGTTAAAGA	0.07141622	0.15616	0.069847	0.222054
tsrna-21913	TGGGTGCTAATGGTGGAGTTAAAG	0.14283243	0.624641	0.139694	0.111027
tsrna-21912	TGGGTGCTAATGGTGGAGTTAAA	0.07141622	0.15616	0.069847	0.111027
tsrna-21911	TGGGTGCTAATGGTGGAGTTAA	0.14283243	0.468481	0	0
tsrna-21910	TGGGTGCTAATGGTGGAGTTA	0	0.15616	0.069847	0
tsrna-21909	TGGGTGCTAATGGTGGAGTT	0	0.07808	0	0
tsrna-21906	TGGGTGCGAGAGGTCCCGGGTT	0.07141622	0.15616	0	0
tsrna-21905	TGGGTGCGAGAGGTCCCGGGT	0	0.07808	0	0
tsrna-21903	TGGGTCCGAGAGGTCCCGGGT	0	0.07808	0	0
tsrna-21899	TGGGTAGAGCATTTGACTGC	0.07141622	0	0.069847	0
tsrna-21894	TGGGGTTTTGCAGTCCTTACCA	0	0.23424	0.069847	0
tsrna-21893	TGGGGTTTTGCAGTCCTTACC	0	0	0	0
tsrna-21891	TGGGGTTTCCCCGCGCAGGTTCG	0	0.07808	0	0
tsrna-21889	TGGGGTTTCCCCGCGCAGGTT	0	0	0	0
tsrna-21880	TGGGGGTTCCCCGCGCAGGT	0.21424865	0.15616	0.069847	0
tsrna-21879	TGGGGGGTTCGATTCCTTCCTTTTTTGCC	0	0.07808	0	0.111027
tsrna-21878	TGGGGGGTTCGATTCCTTCCTTTTTTGC	0	0.07808	0	0.222054
tsrna-21877	TGGGGGGTTCGATTCCTTCCTTTTTTG	0.07141622	0.15616	0.069847	0
tsrna-21876	TGGGGGGTTCGATTCCTTCCTT	0	0.07808	0	0
tsrna-21874	TGGGGATTGTGGGTTCGAGTCCCATCTGGGTCGCC	0	0.07808	0.069847	0
tsrna-21873	TGGGGATTGTGGGTTCGAGTCCCATC	0	0.07808	0	0
tsrna-21872	TGGGGATTGTGGGTTCGAGTCCC	0	0.15616	0	0
tsrna-21871	TGGGGATTGTGGGTTCG	0	0.07808	0	0
tsrna-21869	TGGGCCCATAACCCAGAGGTCGATGGATCG	0.35708108	0.23424	0.069847	0.111027

tsrna-21868	TGGGCCCATAACCCAGAGGTCGATGGA	0.21424865	0.390401	0	0
tsrna-21867	TGGGCCCATAACCCAGAGGTCGATG	0.07141622	0.07808	0	0
tsrna-21866	TGGGCCCATAACCCAGAGGTCGAT	0.07141622	0	0	0
tsrna-21864	TGGGCCCATAACCCAGAGGTCG	0	0.07808	0	0
tsrna-21860	TGGGAGAGCGTTAGACTGAAGA	0	0.07808	0	0
tsrna-21854	TGGGAGAGCATTAGACTGAA	0	0	0	0
tsrna-21853	TGGGACTCTTAATCCCAGGGTCGTGGGTTC	0	0.23424	0	0
tsrna-21848	TGGCTGAGTGAAGCATTGGACTGTAAATC	0.14283243	0	0	0
tsrna-21847	TGGCTGAGTGAAGCATTGGACTGTAAAT	0	0.07808	0.069847	0
tsrna-21846	TGGCTGAGTGAAGCATTGGACTGTAAA	0	0.15616	0.139694	0
tsrna-21845	TGGCTGAGTGAAGCATTGGACTGTAA	0.07141622	0	0	0
tsrna-21844	TGGCTGAGTGAAGCATTGGACTGTA	0.07141622	0.07808	0	0
tsrna-21842	TGGCTGAGTGAAGCATTGGACTG	0	0.07808	0.069847	0
tsrna-21841	TGGCTGAGTGAAGCATTGGACT	0	0	0	0
tsrna-21833	TGGCTAGGATTCGGCGC	0.07141622	0.858881	0	0.555136
tsrna-21832	TGGCTAGGATTCGGCG	0.28566487	0.15616	0.069847	0.111027
tsrna-21831	TGGCGGTCTAGTGGTTAGGATTCGG	0.4284973	0.858881	0.20954	0.111027
tsrna-21830	TGGCGGTCTAGTGGTTAGGATTCG	0.14283243	1.093122	0.488928	0.111027
tsrna-21829	TGGCGCAGCGGAAGCGTGCTGGGCC	0.28566487	2.342403	0.069847	0.333081
tsrna-21828	TGGCGCAGCGGAAGCGTGCTGGGC	0.14283243	2.030083	0.139694	0
tsrna-21827	TGGCGCAGCGGAAGCGTGCTGGG	0.21424865	1.639682	0.139694	0.111027
tsrna-21826	TGGCGCAATGGATAGCGCATTG	0.14283243	0.07808	0	0
tsrna-21825	TGGCCTCCTAAGCCAGGGATTGTGG	0	0.07808	0.069847	0
tsrna-21824	TGGCCTAATGGATAAGGCGTCTGA	0	0.15616	0	0
tsrna-21823	TGGCCTAATGGATAAGGCATTGGCCTCCT	0.35708108	0.546561	0.279387	0.111027
tsrna-21822	TGGCCTAATGGATAAGGCATTGGCC	0.49991351	0.23424	0.069847	0.444109
tsrna-21821	TGGCCTAATGGATAAGGCATTG	0.07141622	0.546561	0.279387	0
tsrna-21820	TGGCCTAATGGATAAGGCATCAGCCT	0.28566487	0.23424	0	0.222054
tsrna-21817	TGGCCGCAGCAACCTCGGTTCGAAT	0	0.07808	0	0
tsrna-21816	TGGCCGCAGCAACCTCGGTTCGAA	0.07141622	0.07808	0	0
tsrna-21815	TGGCCGCAGCAACCTCGGTTCGA	0	0.15616	0	0
tsrna-21814	TGGCCGCAGCAACCTCGGTTCG	0	0.07808	0	0
tsrna-21813	TGGCCGCAGCAACCTCGGTTC	0	0.07808	0	0
tsrna-21804	TGGCCGAGTGGTCTAAGGCGCCAGAC	0	0.07808	0	0
tsrna-21801	TGGCCGAGCGGTCTAAGGCGCTGGATT	0.07141622	0.07808	0.069847	0
tsrna-21800	TGGCCGAGCGGTCTAAGGCGCTGCGTTC	0	0.07808	0	0.111027
tsrna-21789	TGGATTTAGGCTCCAGTCTCTTCGG	0.07141622	0	0	0
tsrna-21787	TGGATCGAAACCATCCTCTGCTACCA	0.78557838	1.873923	0.977855	0.666163
tsrna-21786	TGGATCGAAACCATCCTCTGCTACC	0.35708108	0.390401	0.139694	0.111027
tsrna-21785	TGGATCGAAACCATCCTCTGCTAC	0	0.07808	0	0
tsrna-21784	TGGATCGAAACCATCCTCTGCTA	0	0	0	0
tsrna-21777	TGGATAGCTCAGTCGGTAGAG	0	0.07808	0	0
tsrna-21774	TGGATAGCGCATTGGAC	0.14283243	0.15616	0.069847	0
tsrna-21773	TGGATAGCGCATTGGA	0.14283243	0	0.139694	0
tsrna-21772	TGGATAAGGCGTCTGATTCC	0.07141622	0.23424	0.069847	0.111027
tsrna-21771	TGGATAAGGCGTCTGATT	0.07141622	0	0	0
tsrna-21770	TGGATAAGGCGTCTGACTTCGGATCAGAAG	0	0.23424	0.069847	0.111027
tsrna-21769	TGGATAAGGCGTCTGACTTCGGATCA	0.07141622	0	0	0
tsrna-21768	TGGATAAGGCGTCTGACTTCGGATC	0.07141622	0.07808	0	0
tsrna-21767	TGGATAAGGCGTCTGACTTCGGAT	0	0.07808	0	0
tsrna-21765	TGGATAAGGCGTCTGACTTCGG	0.07141622	0.07808	0	0
tsrna-21764	TGGATAAGGCGTCTGACTTCG	0.07141622	0	0	0
tsrna-21763	TGGATAAGGCGTCTGACTTC	0	0	0	0
tsrna-21761	TGGATAAGGCGTCTGACT	0	0	0	0.111027
tsrna-21758	TGGATAAGGCATTGGCCTCCTAAGCCAGGG	0.21424865	0.23424	0.139694	0
tsrna-21757	TGGATAAGGCATTGGCCTCCTAAG	0.14283243	0.07808	0.20954	0
tsrna-21756	TGGATAAGGCATTGGCCTC	0	0	0.139694	0
tsrna-21754	TGGATAAGGCATTGGCC	0	0	0.139694	0
tsrna-21753	TGGATAAGGCATTGGC	0.14283243	0	0	0
tsrna-21752	TGGATAAGGCATCAGCCT	0	0	0	0
tsrna-21751	TGGATAAGGCACTGGC	0.64274595	1.015041	0.698468	0.666163
tsrna-21750	TGGATAACGCGTCTGACTACGGATC	0.14283243	0.07808	0	0.111027
tsrna-21749	TGGATAACGCGTCTGACTACGGAT	0	0	0.069847	0.111027
tsrna-21748	TGGATAACGCGTCTGACTACGGA	0.14283243	0.07808	0.069847	0.222054

tsrna-21747	TGGATAACGCGTCTGACTACGG	0	0.07808	0	0
tsrna-21746	TGGATAACGCGTCTGACTACG	0.07141622	0	0.139694	0
tsrna-21745	TGGATAACGCGTCTGACTAC	0	0	0	0.111027
tsrna-21743	TGGAGTTAAAGACTTTTTCTCTGACCA	1.92823784	2.888964	1.117549	1.998488
tsrna-21742	TGGAGTTAAAGACTTTTTCTCTGACC	0.28566487	0.31232	0.069847	0
tsrna-21741	TGGAGTTAAAGACTTTTTCTCTGAC	0	0.468481	0	0
tsrna-21740	TGGAGTTAAAGACTTTTTCTCTGA	0.07141622	0.15616	0	0
tsrna-21739	TGGAGTTAAAGACTTTTTCTCTG	0	0.23424	0	0
tsrna-21738	TGGAGGCGTGGGTTCGAATCCCACT	0	0.23424	0	0
tsrna-21736	TGGAGGCGTGGGTTCGAATCCC	0	0	0	0
tsrna-21731	TGGAGGCGTGGGTTCG	0	0.07808	0	0
tsrna-21730	TGGAGGCCATGGGGTTGGCTTG	0	0.07808	0	0
tsrna-21727	TGGACTTTGAATCCAGCGATCCGAG	0	0.15616	0.139694	0
tsrna-21726	TGGACTTTGAATCCAGCGATCCG	0	0.15616	0	0
tsrna-21725	TGGACTTTGAATCCAGCGATCC	0.07141622	0.23424	0.139694	0.111027
tsrna-21724	TGGACTTTGAATCCAGCGA	0	0.23424	0	0.111027
tsrna-21723	TGGACTTTGAATCCAGC	0	0.07808	0	0.111027
tsrna-21722	TGGACTTGAAATCCATTGGGG	0	0.31232	0	0
tsrna-21721	TGGACTTCTAATTCAAAGGTTC	0	0.07808	0	0
tsrna-21714	TGGACTGTAAATCTAAAGACAGGGG	0.07141622	0	0	0
tsrna-21707	TGGACTGCTAATCCATTGTGCT	0.14283243	0	0.139694	0
tsrna-21706	TGGACTGCTAATCCATTGTGC	0	0	0.139694	0
tsrna-21705	TGGACTGCTAATCCATTGTG	0	0.07808	0	0
tsrna-21700	TGGACTCTGAATCCAGCGATCCGAG	0	0.15616	0	0
tsrna-21698	TGGACTCTGAATCCAGCGATCCG	0	0.07808	0	0
tsrna-21696	TGGACTCTGAATCCAGCGATC	0	0.07808	0	0.111027
tsrna-21692	TGGACTCTGAATCCAGC	0	0	0	0.111027
tsrna-21690	TGGACATATGTCCGCGTGGGTT	2.42815136	2.732804	1.816017	0.444109
tsrna-21689	TGGACATATGTCCGCGTGGGT	1.78540541	1.483522	1.187396	0.111027
tsrna-21688	TGGACATATGTCCGCGTGGG	0.07141622	0	0.069847	0
tsrna-21687	TGGACATATGTCCGCGTGG	0.14283243	0.07808	0.069847	0
tsrna-21686	TGCTTTGCACGTATGAGGCCCCGGGT	1.92823784	4.216326	0.838162	3.10876
tsrna-21685	TGCTTTGCACGTATGAGGCCCC	0.07141622	0.15616	0	0
tsrna-21684	TGCTTTGCACGCGTGGGTT	0	0.468481	0	0
tsrna-21683	TGCTTTGCACGCGTGGGT	0.14283243	0.390401	0	0
tsrna-21682	TGCTTTACACGCAGAAGGTCCTGGGT	0.71416216	2.810884	0.558774	0.222054
tsrna-21681	TGCTTTACACGCAGAAGGTCCTG	0.14283243	0	0	0
tsrna-21680	TGCTTAGCTGTTAACTAAGTGTTTGTGGGT	0.07141622	0	0	0.222054
tsrna-21678	TGCTCTGCACGCGTGGGT	0.14283243	0.468481	0	0
tsrna-21677	TGCTACTAATGCCAGGGTCGAGGTTT	0.07141622	0.23424	0.139694	0.666163
tsrna-21676	TGCTACTAATGCCAGGGTCGAGGTT	0	0	0.20954	0.333081
tsrna-21673	TGCTAATGGTGGAGTTAAAGACTTTTTCTCTGACCA	2.78523244	3.591685	1.396936	1.55438
tsrna-21672	TGCTAATGGTGGAGTTAAAGACTTTTTCTCTGACC	0.8569946	1.015041	0.279387	0.111027
tsrna-21671	TGCTAATGGTGGAGTTAAAGACTTTTTCTC	0.4284973	0.31232	0.279387	0.111027
tsrna-21670	TGCTAATGGTGGAGTTAAAGACT	0.21424865	0.390401	0.069847	0.111027
tsrna-21669	TGCTAATGGTGGAGTTAAAGAC	0.07141622	0.15616	0	0
tsrna-21668	TGCTAATGGTGGAGTTAAAGA	0.07141622	0.31232	0.069847	0
tsrna-21667	TGCTAATGGTGGAGTTAAAG	0.14283243	0.15616	0	0
tsrna-21666	TGCTAATGGTGGAGTTAAA	0.14283243	0.23424	0	0
tsrna-21665	TGCTAATGGTGGAGTTAA	0.07141622	0.07808	0.069847	0
tsrna-21664	TGCTAATGGTGGAGTTA	0.07141622	0.07808	0.069847	0
tsrna-21662	TGCTAATCCATTGTGCTTTGCACGCGTGGG	0	0.07808	0.069847	0
tsrna-21661	TGCTAATCCATTGTGCTTTGCACGC	0	0.15616	0	0
tsrna-21659	TGCTAATCCATTGTGCTTTGCA	0	0.07808	0	0
tsrna-21654	TGCTAATCCATTGTGCTCTGC	0	0.23424	0.069847	0
tsrna-21653	TGCTAATCCATTGTGCTCTG	0	0.07808	0	0
tsrna-21652	TGCTAATCCATTGTGCTC	0	0.07808	0	0
tsrna-21651	TGCTAATCCATTGTGCT	0.07141622	0	0	0
tsrna-21649	TGCTAACTCATGCCCCCATGTCTAACAACATGGCTTTCTCAC(	1.49974054	0.23424	2.374791	0.111027
tsrna-21648	TGCTAACTCATGCCCCCATGTCTAACAACATGGCTTTCTCAC(	0.71416216	0.23424	1.117549	0
tsrna-21647	TGCTAACTCATGCCCCCATGTCTAACAAC	0	0	0	0
tsrna-21646	TGCTAACTCATGCCCCCATGTCTAACA	0	0	0	0
tsrna-21642	TGCTAACTCATGCCCCCATGTCT	0	0	0	0
tsrna-21640	TGCTAACTCATGCCCCCATGT	0	0	0	0

tsrna-21632	TGCGTGTTCAAGTCACGTCGGGGTCA	0	0	0	0
tsrna-21626	TGCGAGAGGTCCCGGGTTCAAATCCCGGAC	0	0.390401	0	0
tsrna-21625	TGCGAGAGGTCCCGGGTTCA	0	0.07808	0	0
tsrna-21624	TGCGAGAGGTCCCGGGTTC	0.07141622	0.15616	0	0
tsrna-21623	TGCGAGAGGTCCCGGGTT	0	0.23424	0	0
tsrna-21622	TGCGAGAGGTCCCGGGT	0	0.07808	0	0
tsrna-21621	TGCGAGAGGTAGCGGGA	0.07141622	0.936961	0.139694	0
tsrna-21620	TGCGACCCGGGTTCGATTCCCGGGCGGCG	0	0.23424	0	0
tsrna-21619	TGCCTTCCAAGCAGTTGACCCGGGTTCG	2.14248649	2.888964	0.908008	1.332326
tsrna-21618	TGCCTTCCAAGCAGTTGACCCGGGTTC	2.35673514	2.108163	1.466783	0.999244
tsrna-21617	TGCCTTCCAAGCAGTTGACCCGGGT	2.07107028	2.186243	0.908008	1.55438
tsrna-21616	TGCCTTCCAAGCAGTTGACCCGGG	0.21424865	0.23424	0.20954	0.444109
tsrna-21615	TGCCTTCCAAGCAGTTGACCCGG	0.07141622	0	0.069847	0.222054
tsrna-21614	TGCCTTCCAAGCAGTTGACCCG	0.07141622	0.15616	0.20954	0.333081
tsrna-21613	TGCCTTCCAAGCAGTTGACCC	0.07141622	0.07808	0.069847	0
tsrna-21612	TGCCTGTCACGCGGGAGACCGG	1.57115676	3.123204	0.628621	0.555136
tsrna-21611	TGCCGTGATCGTATAGTGGTTAGTACTCTGCGTTGTGGCCGC	2.35673514	1.249282	1.117549	0.999244
tsrna-21610	TGCCGTGATCGTATAGTGGTTAGTACTCTGCGTTGTGGCC	2.42815136	1.327362	1.117549	0.77719
tsrna-21609	TGCCGTGATCGTATAGTGGTTAGTACTCTGCGTTGTG	2.14248649	0.936961	0.908008	0.555136
tsrna-21608	TGCCGTGATCGTATAGTGGTTAGTACTCTGCGTTGT	2.71381622	1.483522	0.908008	0.888217
tsrna-21607	TGCCGTGATCGTATAGTGGTTAGTACTCTGCGTTG	2.21390271	1.249282	1.047702	0.444109
tsrna-21606	TGCCGTGATCGTATAGTGGTTAGTACTCTGCGTT	1.64257298	0.546561	0.768315	0.666163
tsrna-21605	TGCCGTGATCGTATAGTGGTTAGTACTCTGCGT	0.21424865	0.23424	0.069847	0.111027
tsrna-21604	TGCCGTGATCGTATAGTGGTTAGTACTCTGCG	0.28566487	0.390401	0.349234	0.333081
tsrna-21603	TGCCGTGATCGTATAGTGGTTAGTACTCTGC	0.07141622	0.07808	0.139694	0.222054
tsrna-21602	TGCCGTGATCGTATAGTGGTTAGTACTCTG	0	0	0.20954	0.222054
tsrna-21601	TGCCGTGATCGTATAGTGGTTAGTACTCT	0.14283243	0.07808	0.069847	0
tsrna-21600	TGCCGTGATCGTATAGTGGTTAGTACTC	0.14283243	0.15616	0	0
tsrna-21597	TGCCGTGATCGTATAGTGGTTAGTA	0	0	0.069847	0.111027
tsrna-21592	TGCCGTGATCGTATAGTGGT	0	0	0	0.111027
tsrna-21591	TGCCGTGATCGTATAGTGG	0	0	0	0.111027
tsrna-21587	TGCCGAGGTTGTGAGTTCG	0	0	0	0
tsrna-21584	TGCCCGCATTCTCCACCA	0.07141622	0.390401	0	0.111027
tsrna-21581	TGCCCGCATCCTCCACCA	0	0.07808	0.069847	0
tsrna-21580	TGCCCGCATCCTCCACC	0	0.07808	0	0
tsrna-21578	TGCCCCCATGTCTAACAACATGGCTTTCTCACCA	0.49991351	0.31232	0.488928	0
tsrna-21577	TGCCCCCATGTCTAACAACATGGCTTTCTCACC	0.21424865	0.07808	0.069847	0
tsrna-21576	TGCCCCCATGTCTAACAACATGGCTTTCTCA	0.07141622	0	0.069847	0
tsrna-21575	TGCCCCCATGTCTAACAACATGGCTTTCTC	0.07141622	0	0	0.111027
tsrna-21573	TGCCCCCATGTCTAACAACATGGCTTTC	0	0.07808	0	0
tsrna-21566	TGCCCCCATGTCTAACAACA	0	0	0.069847	0
tsrna-21561	TGCCCACATTCTCCACCA	0.07141622	0.07808	0	0.111027
tsrna-21560	TGCCATGATCGTATAGTGGTTAGTACTCTG	0	0.15616	0	0.111027
tsrna-21558	TGCCATGATCGTATAGTGGTTAGT	0	0.07808	0	0
tsrna-21553	TGCCACGCGGGAGGCCCGGGTTCGATTCCCGGCCCATGCAC	1.71398919	3.747845	0.768315	1.665407
tsrna-21552	TGCCACGCGGGAGGCCCGGGTTCGATTCCCGGCCAATGCAC	3.42797839	12.1805	1.117549	1.332326
tsrna-21551	TGCCACGCGGGAGGCCCGGGTTCG	0	0.468481	0.139694	0.111027
tsrna-21550	TGCCACGCGGGAGGCCCGGGTTC	0.21424865	0.546561	0.139694	0
tsrna-21549	TGCCACGCGGGAGGCCCGGGTT	0.07141622	0.23424	0	0
tsrna-21548	TGCCACGCGGGAGGCCCGGGT	0.07141622	0.390401	0	0
tsrna-21547	TGCCACGCGGGAGGCCCGGG	0	0	0.069847	0
tsrna-21546	TGCCACGCGGGAGGCCCGG	0	0.07808	0	0
tsrna-21545	TGCCACGCGGGAGGCCCG	0	0.07808	0	0
tsrna-21543	TGCATTGGTGGTTCAGTGGTAGAATTCTCG	2.64240001	1.327362	4.190808	4.774167
tsrna-21542	TGCATGTATGAGGTCCCGGGTT	0	0.390401	0	0
tsrna-21541	TGCATGGGTGGTTCAGTGGTAGAATTCTCG	1.78540541	1.249282	1.53663	5.218275
tsrna-21540	TGCATGGGTGGTTCAGTGGTAGA	0.35708108	0.390401	0.279387	0.888217
tsrna-21539	TGCATGAGGTCCCGGGTTCGATCCCCAGC	0.07141622	0.858881	0	0.111027
tsrna-21538	TGCATGAGGTCCCGGGTTCG	0	0.31232	0	0
tsrna-21537	TGCATGAGGTCCCGGGTTC	0.07141622	0.31232	0.069847	0.111027
tsrna-21536	TGCATGAGGTCCCGGGT	0.14283243	0.15616	0	0
tsrna-21535	TGCAGTGGTAGAATTCTCGCCT	0.14283243	0.546561	0.139694	0.111027
tsrna-21534	TGCAGTGGTAGAATTCTCGCC	0.21424865	0.390401	0.139694	0
tsrna-21533	TGCAGTGGTAGAATTCTCGC	0	0	0	0.111027

tsrna-21532	TGCAGTGGTAGAATTCTCG	0	0	0	0
tsrna-21529	TGCAGATCAAGAGGTCCCTGGT	0.07141622	0.31232	0	0.111027
tsrna-21528	TGCAGATCAAGAGGTCCCCGGT	0	0.390401	0.139694	0
tsrna-21526	TGCAGAGTGGGGTTTTGCAGTCCTTACCA	0.07141622	0.23424	0.069847	0
tsrna-21525	TGCAGAGTGGGGTTTTGCAGTCCT	0	0	0.069847	0
tsrna-21524	TGCAGAGTGGGGTTTTGCAGTCC	0	0.07808	0	0
tsrna-21523	TGCAGAGTGGGGTTTTGCAGTC	0	0	0	0
tsrna-21522	TGCAGAGTGGGGTTTTGCAGT	0	0	0	0
tsrna-21520	TGCAGAGTGGGGTTTTGC	0	0	0	0
tsrna-21517	TGCACGTATGAGGCCCCGGGTTCA	0.07141622	0.468481	0.279387	0
tsrna-21516	TGCACGTATGAGGCCCCGGGTT	0.14283243	0.15616	0.069847	0.111027
tsrna-21515	TGCACGTATGAGGCCCCGGGT	0.14283243	0.31232	0.069847	0.111027
tsrna-21514	TGCACGTATGAGGCCCCGGG	0	0	0	0.111027
tsrna-21512	TGCACGCGTGGGTTCGAATCCCATCCTCGTCGC	0	0	0	0.111027
tsrna-21511	TGCACGCGTGGGTTCGAATCCCATCCTCGT	0	0.07808	0	0
tsrna-21510	TGCACGCGTGGGTTCGAATCCCATC	0.07141622	0	0	0
tsrna-21508	TGCACGCGTGGGTTCGAATCCCA	0	0	0.069847	0
tsrna-21506	TGCACGCGTGGGTTCGAATCC	0	0.07808	0	0
tsrna-21504	TGCACGCGTGGGTTCGAAT	0	0.07808	0.069847	0
tsrna-21502	TGCACGAGGTCCTGGGT	0.07141622	0.468481	0.069847	0
tsrna-21501	TGCACGAGGCCCCGGGTTCAATCCCCGGCACCTCCACC	1.21407568	2.186243	0.908008	0.333081
tsrna-21500	TGCACGAGGCCCCGGGTTCAATCCCCGGCACCT	0	0.23424	0	0
tsrna-21499	TGCACGAGGCCCCGGGTTCAATCCCCGGC	0	0.07808	0	0
tsrna-21498	TGCACGAGGCCCCGGGTTCA	0.07141622	0.23424	0.069847	0
tsrna-21497	TGCACGAGGCCCCGGGTTC	0	0.390401	0.069847	0
tsrna-21496	TGCACGAGGCCCCGGGTT	0	0.07808	0	0
tsrna-21493	TGCACACGTGGGTTCGAATCCCATCCTCGT	0.07141622	0	0	0
tsrna-21492	TGCACACGTGGGTTCGAATCCC	0.07141622	0	0	0
tsrna-21491	TGCAATACTTAATTTCTGCCA	0.14283243	0	0	0.111027
tsrna-21490	TGCAATACTTAATTTCTGCC	0.14283243	0	0	0
tsrna-21487	TGCAAGATTCCCATTCTTGCGACCCG	0	0.23424	0	0.111027
tsrna-21486	TGCAACTCCAAATAAAAGTACCA	0	0	0	0
tsrna-21479	TGCAAATTCGAAGAAGCAGCTTCAAACCTGCCGGGGCTTCCA	0.8569946	1.795843	0.419081	0.999244
tsrna-21478	TGCAAATTCGAAGAAGCAGCTTCAAACCTGCCGGGGCTT	0	0.31232	0.069847	0
tsrna-21475	TGATTTGCGTTCAGTTGATGCAGAGT	0.07141622	0	0	0
tsrna-21474	TGATTTGCGTTCAGTTGATGCA	0.07141622	0	0.069847	0
tsrna-21472	TGATTTCGACTCATTAAATTATGATAATCATATTTACCAACC	2.57098379	0.468481	4.889276	0.333081
tsrna-21471	TGATTTCGACTCATTAAATTATGATAATC	0.49991351	0.31232	0.419081	0
tsrna-21466	TGATTCTCGCTTTGGGTGCGAG	0	0	0	0
tsrna-21465	TGATTCTCGCTTTGGGTGCGA	0	0	0	0
tsrna-21456	TGATTCCGGATCAGAAGATTGAGGGT	0.21424865	0.468481	0	0.222054
tsrna-21455	TGATTCCGGATCAGAAGATTGAGG	0	0.07808	0	0
tsrna-21451	TGATTAGGGTGCTTAGCTGTTA	0.21424865	0	0.279387	0.111027
tsrna-21450	TGATTAGGGTGCTTAGCTGTT	0	0	0.069847	0
tsrna-21448	TGATGCAGAGTGGGGTTTTGCAGTCCTTAC	0	0.15616	0	0.111027
tsrna-21447	TGATGCAGAGTGGGGTTTTGCAGTCC	0	0	0	0
tsrna-21446	TGATGCAGAGTGGGGTTTTGCAGTC	0	0	0	0
tsrna-21445	TGATGCAGAGTGGGGTTTTGCA	0	0.07808	0	0
tsrna-21444	TGATGCAGAGTGGGGTTTTGC	0	0	0	0
tsrna-21443	TGATCGTATAGTGGTTAGTACTCTGCGTTG	1.57115676	0.780801	0.628621	1.110271
tsrna-21442	TGATCGTATAGTGGTTAGTACTCTGCGTT	1.14265946	0.702721	0.698468	0.555136
tsrna-21441	TGATCGTATAGTGGTTAGTACTCTGCGT	0.49991351	0.468481	0.069847	0.222054
tsrna-21440	TGATCGTATAGTGGTTAGTACTCTGCGCTG	0.4284973	0.15616	0.139694	0.444109
tsrna-21439	TGATCGTATAGTGGTTAGTACTCTGCGC	0.21424865	0.390401	0.139694	0.222054
tsrna-21438	TGATCGTATAGTGGTTAGTACTCTGCG	0.28566487	0.15616	0.069847	0.222054
tsrna-21437	TGATCGTATAGTGGTTAGTACTCTGC	0.14283243	0	0.069847	0.222054
tsrna-21436	TGATCGTATAGTGGTTAGTACTCTG	0.14283243	0.31232	0.139694	0
tsrna-21435	TGATCGTATAGTGGTTAGTACTCT	0.14283243	0.07808	0	0.222054
tsrna-21434	TGATCGTATAGTGGTTAGTACTC	0	0	0.069847	0
tsrna-21433	TGATCGTATAGTGGTTAGTACT	0	0.07808	0	0
tsrna-21431	TGATCGTATAGTGGTTAGTA	0	0	0	0
tsrna-21427	TGATAGGTGGCACGGAGAATTTTGGATT	0	0.07808	0.069847	0
tsrna-21426	TGATAGGTGGCACGGAGAATTTTGGA	0.07141622	0.15616	0	0
tsrna-21425	TGATAGGTGGCACGGAGAATTTTGG	0.07141622	0	0.069847	0.111027

tsrna-21424	TGATAGGTGGCACGGAGAATTTTG	0	0.07808	0.069847	0.111027
tsrna-21422	TGATAGGTGGCACGGAGAATTT	0	0	0.069847	0
tsrna-21421	TGATAGGTGGCACGGAGAATT	0	0.07808	0.069847	0
tsrna-21420	TGATAGGTGGCACGGAGAAT	0	0	0	0
tsrna-21418	TGATAGGTGGCACGGAGA	0	0	0	0
tsrna-21416	TGATAATCATATTTACCAACCA	0	0.07808	0	0
tsrna-21410	TGATAACACCAAGGTCGCGGGC	0	0.468481	0.279387	0
tsrna-21408	TGAGTTCGATCCTCACACGGGGCACCA	0	0.468481	0	0
tsrna-21407	TGAGTTCGAGCCTCACCTGGAGCACCA	0	0.390401	0	0
tsrna-21402	TGAGTGAAGCATTGGACTGTAAATCTAAAG	0.07141622	0.07808	0.069847	0
tsrna-21401	TGAGTGAAGCATTGGACTGTAAATCTAAA	0.07141622	0.15616	0.20954	0.111027
tsrna-21400	TGAGTGAAGCATTGGACTGTAAATCTAA	0.14283243	0	0.069847	0
tsrna-21399	TGAGTGAAGCATTGGACTGTAAATCTA	0	0.07808	0.139694	0
tsrna-21398	TGAGTGAAGCATTGGACTGTAAATCT	0	0.15616	0.069847	0
tsrna-21397	TGAGTGAAGCATTGGACTGTAAATC	0	0	0.279387	0.111027
tsrna-21396	TGAGTGAAGCATTGGACTGTAAAT	0	0	0	0
tsrna-21395	TGAGTGAAGCATTGGACTGTAAA	0.07141622	0.07808	0.069847	0
tsrna-21394	TGAGTGAAGCATTGGACTGTAA	0	0	0	0
tsrna-21393	TGAGTGAAGCATTGGACTGTA	0	0.07808	0.139694	0
tsrna-21392	TGAGTGAAGCATTGGACTGT	0	0	0	0
tsrna-21388	TGAGTATCCCCGCCTGTCACGCGGGAGACC	1.49974054	3.669765	0.558774	0.333081
tsrna-21387	TGAGTATCCCCGCCTGTCACGCGGG	0.71416216	1.639682	0.20954	0.333081
tsrna-21386	TGAGTATCCCCGCCTGTCACGC	0	0.15616	0	0
tsrna-21385	TGAGTATCCCCGCCTGTC	0	0.07808	0	0
tsrna-21384	TGAGTATCCCCGCCTGT	0.07141622	0	0	0
tsrna-21380	TGAGGTCCCGGGTTCGATCCCCAGC	0	0.07808	0.069847	0.111027
tsrna-21379	TGAGGTCCCGGGTTCGATCCCC	0	0.07808	0	0
tsrna-21378	TGAGGTCCCGGGTTCGATCCC	0	0.07808	0	0
tsrna-21377	TGAGGTCCCGGGTTCGATCC	0	0.15616	0	0.111027
tsrna-21376	TGAGGTCCCGGGTTCG	0	0.15616	0	0
tsrna-21374	TGAGGGTCCAGGGTTCAAGTCCCTGTTCGGGCGC	0	0.23424	0	0.111027
tsrna-21373	TGAGGGTCCAGGGTTCAAGTCCCTGTTCGGGCG	0	0	0.139694	0
tsrna-21372	TGAGGGTCCAGGGTTCAAGTCCCTGTTCGGGC	0	0.23424	0	0
tsrna-21371	TGAGGGTCCAGGGTTCAAGTCCCT	0	0.07808	0	0
tsrna-21366	TGAGGGTCCAGGGTTC	0	0	0	0
tsrna-21365	TGAGGCCCCGGGTTCGATCCCCGGCATC	0	0.07808	0	0
tsrna-21364	TGAGGCCCCGGGTTCGATCCCCGGC	0	0.07808	0	0
tsrna-21363	TGAGGCCCCGGGTTCGATCCCC	0.07141622	0.31232	0	0
tsrna-21362	TGAGCACTCTGGACTCTGAATCCAGCG	0.07141622	0.23424	0.069847	0.111027
tsrna-21361	TGAGCACTCTGGACTCTGAATCCAGC	0.14283243	0.23424	0	0
tsrna-21360	TGAGCACTCTGGACTCTGAATCCA	0	0.23424	0.20954	0
tsrna-21359	TGAGCACTCTGGACTCTGAATCC	0.07141622	0.15616	0.139694	0
tsrna-21358	TGAGCACTCTGGACTCTGAATC	0	0.07808	0	0
tsrna-21352	TGAGACTCTTAATCTC	0	0.07808	0	0
tsrna-21349	TGACTTCGGATCAGAAGATTGAGGGT	0.07141622	0.390401	0.069847	0.222054
tsrna-21348	TGACTTCGGATCAGAAGAT	0.07141622	0	0	0
tsrna-21347	TGACTGCAGATCAAGAGGTCCCTGGTTC	0.14283243	0.858881	0	0.111027
tsrna-21346	TGACTGCAGATCAAGAGGTCCCTGGTT	0	0.468481	0.139694	0.444109
tsrna-21345	TGACTGCAGATCAAGAGGTCCCTGGT	0.28566487	0.468481	0.279387	0.111027
tsrna-21341	TGACTGCAGATCAAGAGGTCCCCGGTTC	0.07141622	0.390401	0.069847	0.222054
tsrna-21340	TGACTGCAGATCAAGAGGTCCCCGGTT	0.21424865	0.31232	0	0.111027
tsrna-21339	TGACTGCAGATCAAGAGGTCCCCGGT	0.07141622	0.546561	0.139694	0
tsrna-21329	TGACTCCAGATCAGAAGGTTGCGTGTT	0.07141622	0.31232	0	0.111027
tsrna-21328	TGACTCCAGATCAGAAGGTTGCGTGT	0	0.23424	0.069847	0
tsrna-21327	TGACTCCAGATCAGAAGGTTGCGTG	0	0.07808	0.069847	0
tsrna-21322	TGACTCCAGATCAGAAGGCTGCGTGTT	0	0.07808	0.069847	0
tsrna-21315	TGACTACGGATCAGAAGATTCTAGGTTCG	0.14283243	0.624641	0.139694	0.111027
tsrna-21314	TGACCCGGGTTCGATTCCCGGCCAACGCACCA	3.28514596	6.012168	1.466783	1.332326
tsrna-21310	GTGGGTTCGAATCCCACCCTCGTCGC	0	0	0	0
tsrna-21309	GTGGGTTCGAATCCCACCACTGCCAC	0	0.07808	0	0
tsrna-21307	GTGGGTTCGAACCCCACTCCTGGTACCA	0	0.07808	0	0
tsrna-21306	GTGGGTTCGAACCCCACTCCTGGTACC	0	0	0	0
tsrna-21301	GTGGGTAGAGCATTTGACTGC	0.07141622	0.15616	0	0.111027
tsrna-21299	GTGGGTAGAGCATTTGACT	0	0.15616	0	0

tsrna-21298	GTGGGTAGAGCATTTGAC	0	0.07808	0	0
tsrna-21296	GTGGGGTTTTGCAGTCCTTACCA	0	0.07808	0	0
tsrna-21295	GTGGGGTTTTGCAGTCCTTACC	0	0	0	0
tsrna-21294	GTGGGGTTTTGCAGTCCTTA	0	0.15616	0	0
tsrna-21293	GTGGGGTTTTGCAGTCCTT	0	0.07808	0	0
tsrna-21292	GTGGGGTTTTGCAGTCCT	0	0	0	0
tsrna-21284	GTGGCTAGGATTCGGCGCTTTC	0.35708108	1.327362	0.558774	1.110271
tsrna-21283	GTGGCTAGGATTCGGCGCTTT	0.35708108	1.327362	0.139694	0.999244
tsrna-21282	GTGGCTAGGATTCGGCGCTT	0.28566487	1.717762	0.069847	0.555136
tsrna-21281	GTGGCTAGGATTCGGCGCT	0.35708108	1.015041	0.20954	1.110271
tsrna-21280	GTGGCTAGGATTCGGCGC	0.07141622	0.858881	0.349234	0.555136
tsrna-21279	GTGGCTAGGATTCGGCG	0	1.171202	0.069847	1.221299
tsrna-21278	GTGGCTAGGATTCGGC	0.21424865	1.093122	0.069847	0.111027
tsrna-21277	GTGGCGCAGCGGAAGCGTGCTGGGCCC	0.71416216	3.747845	0.349234	0.77719
tsrna-21276	GTGGCGCAGCGGAAGCGTGCTGGGCC	0.71416216	2.420483	0.139694	0.111027
tsrna-21275	GTGGCGCAGCGGAAGCGTGCTGGGC	0.14283243	2.732804	0	0.111027
tsrna-21274	GTGGCGCAGCGGAAGCGTGCTGGG	0	1.639682	0.279387	0.111027
tsrna-21273	GTGGCGCAGCGGAAGCGTGCTG	0	0.07808	0	0
tsrna-21271	GTGGCGCAATGGATAGCGCATTGG	0.14283243	0.546561	0.069847	0.111027
tsrna-21270	GTGGCGCAATGGATAGCGCATTG	0	0	0	0
tsrna-21268	GTGGCGCAACGGTAGCGCGTCTGACT	0	0.15616	0.069847	0
tsrna-21267	GTGGCCTAATGGATAAGGCGTCTGATTCCG	0.07141622	0.780801	0.069847	0.222054
tsrna-21266	GTGGCCTAATGGATAAGGCATTGGCC	0.4284973	0.390401	0.349234	0
tsrna-21265	GTGGCCTAATGGATAAGGCATTG	0.35708108	0.07808	0.069847	0
tsrna-21264	GTGGCCTAATGGATAAGGCATCAGCCTC	0	0.31232	0	0.111027
tsrna-21263	GTGGCCTAATGGATAAGGCATCAGCCT	0.28566487	0.390401	0.20954	0
tsrna-21262	GTGGCCTAATGGATAAGGCATCAGC	0.35708108	0.15616	0.20954	0.111027
tsrna-21261	GTGGCCTAATGGATAAGGCATCAG	0.14283243	0.31232	0.069847	0
tsrna-21260	GTGGCCTAATGGATAAGGCACTG	5.07055137	2.810884	3.49234	1.887461
tsrna-21258	GTGGCCGCAGCAACCTCGGTTCG	0	0.15616	0	0
tsrna-21257	GTGGCCGCAGCAACCTCGGTT	0.07141622	0.07808	0	0
tsrna-21254	GTGGCCGCAGCAACCTCG	0	0	0	0
tsrna-21248	GTGGCCGAGCGGTCTAAGGCGCT	0	0	0	0
tsrna-21244	GTGGCACGGAGAATTTTGGATT	0	0.07808	0	0
tsrna-21240	GTGGAGTTAAAGACTTTTTCTCTGACCA	1.71398919	2.967044	0.768315	2.442597
tsrna-21239	GTGGAGTTAAAGACTTTTTCTCTGACC	0.28566487	1.015041	0.069847	0.111027
tsrna-21238	GTGGAGTTAAAGACTTTTTCTCTGAC	0	0.07808	0	0
tsrna-21236	GTGGAGTTAAAGACTTTTTCTCTG	0	0.15616	0.069847	0
tsrna-21235	GTGGAGTTAAAGACTTTTTCTCT	0	0.15616	0	0
tsrna-21233	GTGGAGTTAAAGACTTTTTCT	0	0	0.069847	0
tsrna-21231	GTGGAGTTAAAGACTTTTT	0	0	0	0
tsrna-21228	GTGGAGTTAAAGACTT	0	0.07808	0	0
tsrna-21227	GTGGAGTGGTTATCACGTTCGCCT	0	0.07808	0	0
tsrna-21225	GTGCTTTGCACGCGTGGGTT	0.07141622	0.15616	0.069847	0
tsrna-21222	GTGCTGGGCCCATAACCCAGAGGTCGATGGATC	0.35708108	0.780801	0.069847	0.111027
tsrna-21218	GTGCTAATGGTGGAGTTAAAGACTTTTTCTCTGACC	0.4284973	1.717762	0.349234	0.111027
tsrna-21217	GTGCTAATGGTGGAGTTAAAGACTTTTTCT	0.49991351	1.171202	0.279387	0
tsrna-21216	GTGCTAATGGTGGAGTTAAAGACT	0	0.468481	0.279387	0
tsrna-21215	GTGCTAATGGTGGAGTTAAAGAC	0.07141622	0.31232	0	0
tsrna-21214	GTGCTAATGGTGGAGTTAAAGA	0.07141622	0.15616	0.069847	0
tsrna-21213	GTGCTAATGGTGGAGTTAAAG	0.07141622	0	0	0.222054
tsrna-21212	GTGCTAATGGTGGAGTTAA	0	0.390401	0.139694	0
tsrna-21211	GTGCTAATGGTGGAGTTA	0	0.23424	0	0
tsrna-21210	GTGCTAATGGTGGAGTT	0	0	0	0
tsrna-21208	GTGCGAGAGGTCCCGGGTTC	0	0.31232	0	0
tsrna-21207	GTGCGAGAGGTCCCGGGTT	0	0.07808	0	0
tsrna-21206	GTGCGAGAGGTCCCGGGT	0.07141622	0	0	0
tsrna-21204	GTGCAGTGGTAGAATTCTCGCCT	0.07141622	0.23424	0	0.222054
tsrna-21203	GTGCAGTGGTAGAATTCTCGCC	0.14283243	0.546561	0.069847	0.111027
tsrna-21202	GTGCAGTGGTAGAATTCTCGC	0	0.07808	0	0
tsrna-21201	GTGCAGTGGTAGAATTCTCG	0	0	0	0
tsrna-21198	GTGCAACTCCAAATAAAAGTACCA	0	0	0.069847	0
tsrna-21197	GTGCAACTCCAAATAAAAGTACC	0	0.15616	0	0
tsrna-21196	GTGCAACTCCAAATAAAAGTAC	0	0.07808	0	0

tsrna-21193	GTGCAACTCCAAATAAAAG	0	0	0	0.111027
tsrna-21192	GTGCAACTCCAAATAAAA	0	0.07808	0	0
tsrna-21190	GTGCAACTCCAAATAA	0	0	0.069847	0
tsrna-21187	GTGATGGCCGAGTGGTTAAGGC	0	0.07808	0	0
tsrna-21185	GTGATCGTATAGTGGTTAGTACTCTGCGTTG	1.71398919	1.249282	0.908008	0.888217
tsrna-21184	GTGATCGTATAGTGGTTAGTACTCTGCGTT	1.64257298	1.327362	0.349234	0.222054
tsrna-21183	GTGATCGTATAGTGGTTAGTACTCTGCGT	0.57132973	0.23424	0	0.111027
tsrna-21182	GTGATCGTATAGTGGTTAGTACTCTGCG	0.35708108	0.15616	0.069847	0
tsrna-21181	GTGATCGTATAGTGGTTAGTACTCTGC	0.21424865	0.15616	0	0
tsrna-21180	GTGATCGTATAGTGGTTAGTACTCTG	0	0.07808	0.069847	0.222054
tsrna-21179	GTGATCGTATAGTGGTTAGTACTCT	0.07141622	0.07808	0.069847	0.111027
tsrna-21178	GTGATCGTATAGTGGTTAGTACTC	0	0	0	0.111027
tsrna-21177	GTGATCGTATAGTGGTTAGTACT	0.07141622	0	0.069847	0
tsrna-21169	GTGATAGGTGGCACGGAGAATTTTG	0	0.31232	0.069847	0
tsrna-21165	GTGAGTATCCCCGCCTGTCACGCGGGAGAC	1.21407568	6.012168	0.698468	0.444109
tsrna-21164	GTGAGTATCCCCGCCTGTCACGCGGG	0.28566487	2.186243	0.419081	0
tsrna-21163	GTGAGTATCCCCGCCTGTCACGC	0.28566487	0.546561	0	0
tsrna-21162	GTGAGTATCCCCGCCTGTCACG	0.14283243	0.23424	0.069847	0
tsrna-21161	GTGAGTATCCCCGCCTGTCAC	0.07141622	0.23424	0.069847	0
tsrna-21160	GTGAGTATCCCCGCCTGTCA	0	0.23424	0.069847	0.111027
tsrna-21159	GTGAGTATCCCCGCCTGTC	0.21424865	0.31232	0.069847	0.111027
tsrna-21158	GTGAGTATCCCCGCCTGT	0	0	0	0.111027
tsrna-21157	GTGAGTATCCCCGCCTG	0	0	0	0
tsrna-21156	GTGAGTATCCCCGCCT	0	0.07808	0	0.111027
tsrna-21155	GTGAGCATAGCTGCCTTCCAAGCAG	0	0	0	0
tsrna-21154	GTGAGCATAGCTGCCTTCCAAGCA	0.07141622	0	0	0
tsrna-21151	GTGAGCACTCTGGACTCTGAATCCAGCG	0.14283243	0.15616	0.069847	0
tsrna-21150	GTGAGCACTCTGGACTCTGAATCCAGC	0.21424865	0.31232	0.069847	0.111027
tsrna-21149	GTGAGCACTCTGGACTCTGAATCCA	0.07141622	0.15616	0	0.111027
tsrna-21148	GTGAGCACTCTGGACTCTGAATCC	0.07141622	0	0	0
tsrna-21147	GTGAGCACTCTGGACTCTGAATC	0.07141622	0.07808	0	0
tsrna-21140	GTGAGAGGTCCCGGGTTCAAATCCCGGACG	0.07141622	0.07808	0	0
tsrna-21138	GTGAATCTGACAACAGAGGCTTACGACCCCTTATTTACCCCA	1.71398919	0.702721	0.908008	0
tsrna-21137	GTGAATCTGACAACAGAGGCTTACGACCCCTTATTTACCCC	1.21407568	0.546561	0.488928	0
tsrna-21136	GTGAATCTGACAACAGAGGCTTACGACCCCTTATTTACC	0.71416216	0.624641	0.349234	0
tsrna-21135	GTGAATCTGACAACAGAGGCTTACGACCCCTTATTT	0.99982703	0.468481	0.419081	0
tsrna-21134	GTGAATCTGACAACAGAGGCTTACGACCCC	0.07141622	0.07808	0	0
tsrna-21133	GTGAATCTGACAACAGAGGCTTACGACCC	0	0	0	0
tsrna-21132	GTGAATCTGACAACAGAGGCTTACGA	0	0	0.069847	0
tsrna-21129	GTGAATCTGACAACAGAGGCTTA	0	0	0	0
tsrna-21128	GTGAATCTGACAACAGAGGCTT	0	0.07808	0	0
tsrna-21127	GTGAATCTGACAACAGAGGCT	0	0.07808	0	0
tsrna-21126	GTGAATCTGACAACAGAGGC	0	0.07808	0	0
tsrna-21121	GTGAAGCATTGGACTGTAAATCTAAAGAC	0	0.15616	0.069847	0
tsrna-21120	GTGAAGCATTGGACTGTAAATCTAAA	0	0	0.069847	0
tsrna-21119	GTGAAGCATTGGACTGTAAATCTAA	0.07141622	0	0	0
tsrna-21113	GTGAAGCATTGGACTGTAA	0	0	0	0
tsrna-21112	GTGAAGCATTGGACTGTA	0	0	0.069847	0
tsrna-21109	GTCTTGTAAACCAGGGGTCGCGA	0.07141622	0.23424	0.069847	0.111027
tsrna-21108	GTCTTGTAAACCAGGGGTCGCG	0.07141622	0.468481	0.069847	0
tsrna-21104	GTCTCTTCGGGGGCGTGGGTTCG	0.07141622	0.31232	0	0
tsrna-21103	GTCTCTTCGGGGGCGTGGGTTC	0.07141622	0.07808	0	0.111027
tsrna-21102	GTCTCTTCGGGGGCGTGGGTT	0	0.31232	0	0.111027
tsrna-21088	GTCTCTGTGGCGCAATGGGT	0.07141622	0	0.069847	0
tsrna-21087	GTCTCTGTGGCGCAATGGG	0	0.07808	0	0
tsrna-21086	GTCTCTGTGGCGCAATGGACGAGCGC	0	0	0	0
tsrna-21083	GTCTCTGTGGCGCAATGGACGAG	0	0.07808	0	0
tsrna-21082	GTCTCTGTGGCGCAATGGACGA	0	0	0	0
tsrna-21080	GTCTCTGTGGCGCAATGGAC	0	0	0	0
tsrna-21079	GTCTCTGTGGCGCAATGGA	0	0	0	0
tsrna-21078	GTCTCTGTGGCGCAATGG	0	0	0.069847	0
tsrna-21077	GTCTCTGTGGCGCAATG	0	0	0.139694	0
tsrna-21076	GTCTCTGTGGCGCAATCGGTTAGCGCGTTC	0	0.15616	0	0
tsrna-21075	GTCTCTGTGGCGCAATCGGTTAGCGCATTC	0.07141622	0	0.069847	0

tsrna-21074	GTCTCTGTGGCGCAATCGGTTAGCGC	0	0.15616	0	0
tsrna-21073	GTCTCTGTGGCGCAATCGGTTAGCG	0	0	0	0
tsrna-21071	GTCTCTGTGGCGCAATCGGTTAG	0.14283243	0	0	0
tsrna-21070	GTCTCTGTGGCGCAATCGGTTA	0	0	0	0
tsrna-21068	GTCTCTGTGGCGCAATCGGTC	0	0.07808	0	0
tsrna-21067	GTCTCTGTGGCGCAATCGGT	0	0	0	0
tsrna-21060	GTCTCTGTGGAACCTCCA	0	0.15616	0.139694	0
tsrna-21059	GTCTCGGTGGAACCTCCA	2.99948109	7.33953	0.488928	1.221299
tsrna-21058	GTCTCGGTGGAACCTCC	0.35708108	0.31232	0.069847	0.111027
tsrna-21057	GTCTCGGTGGAACCTC	0.07141622	0.07808	0	0
tsrna-21056	GTCTCCGGATGGAGGCGTGGG	0.07141622	0.624641	0.069847	0
tsrna-21055	GTCTCCGGATGGAGGCGTGG	0.07141622	0.468481	0	0
tsrna-21054	GTCTCCGGATGGAGGCGTG	0.14283243	0.390401	0	0
tsrna-21050	GTCTCATAATCTGAAGGTCGTGAGT	1.78540541	3.904006	1.74617	1.221299
tsrna-21049	GTCTCATAATCTGAAGGTCGTG	0.21424865	0.546561	0.20954	0.222054
tsrna-21048	GTCTCATAATCTGAAGGTCCTGAGT	9.21269192	25.45412	5.797285	6.883683
tsrna-21047	GTCTCATAATCTGAAGGTCCTGA	0.14283243	1.639682	0.488928	0.333081
tsrna-21046	GTCTCATAATCTGAAGGTCCTG	0.28566487	1.171202	0.279387	0.555136
tsrna-21045	GTCTCATAATCTGAAGGTCCT	0.07141622	0.23424	0.069847	0
tsrna-21044	GTCTCATAATCTGAAGGTCC	0	0.390401	0.139694	0
tsrna-21043	GTCTAGTGGTTAGGATTCGGCGCTCTCACCGCCGCGGCCCG	3.64222704	4.919047	2.304945	2.775678
tsrna-21042	GTCTAGTGGTTAGGATTCGGCGCTCTCACCGCCGCGGCCCG	4.92771893	5.699848	2.444638	1.887461
tsrna-21041	GTCTAGTGGTTAGGATTCGGCGCTCTCACC	3.28514596	3.669765	1.257243	1.887461
tsrna-21040	GTCTAGTGGTTAGGATTCGGCGCTCTCA	2.64240001	3.123204	1.955711	2.997733
tsrna-21039	GTCTAGTGGTTAGGATTCGGCGCTCTC	2.99948109	3.279365	1.53663	2.442597
tsrna-21038	GTCTAGTGGTTAGGATTCGGCGCTCT	1.57115676	3.747845	1.955711	1.332326
tsrna-21037	GTCTAGTGGTTAGGATTCGGCGCTC	3.71364325	3.904006	1.53663	1.776434
tsrna-21036	GTCTAGTGGTTAGGATTCGGCGCT	2.28531893	3.201285	1.257243	1.55438
tsrna-21035	GTCTAGTGGTTAGGATTCGGCGC	1.21407568	1.093122	1.117549	0.888217
tsrna-21034	GTCTAGTGGTTAGGATTCGGCG	0.92841081	0.702721	0.628621	0.333081
tsrna-21033	GTCTAGTGGTTAGGATTCGGC	0.4284973	0.390401	0.279387	0.111027
tsrna-21032	GTCTAGTGGTTAGGATTCGG	0.28566487	0	0.20954	0
tsrna-21031	GTCTAGTGGTTAGGATTCG	0.28566487	0	0.069847	0.111027
tsrna-21030	GTCTAGTGGTTAGGATTC	0.07141622	0	0.069847	0
tsrna-21029	GTCTAGTGGTTAGGATT	0.14283243	0	0.069847	0
tsrna-21028	GTCTAGTGGTTAGGAT	0.07141622	0	0	0
tsrna-21026	GTCTAGTGGCTAGGATTCGGCGCTTT	0.78557838	3.201285	0.628621	2.997733
tsrna-21025	GTCTAGTGGCTAGGATTCGGCGCT	1.21407568	3.591685	0.419081	3.10876
tsrna-21024	GTCTAGTGGCTAGGATTCGGCGC	0.71416216	2.420483	0.349234	2.220543
tsrna-21023	GTCTAGTGGCTAGGATTCGGC	0.49991351	1.717762	0.20954	0.999244
tsrna-21020	GTCTAGGGGTATGATTCTCGGTTTG	0.35708108	0.23424	0	0.111027
tsrna-21019	GTCTAGGGGTATGATTCTCGGTT	0.71416216	0.15616	0.20954	0.222054
tsrna-21018	GTCTAGGGGTATGATTCTCGGT	0	0.15616	0.069847	0.111027
tsrna-21017	GTCTAGGGGTATGATTCTCGG	0.07141622	0	0.069847	0.222054
tsrna-21016	GTCTAGGGGTATGATTCTCGCTTC	1.21407568	1.639682	0.977855	1.665407
tsrna-21015	GTCTAGGGGTATGATTCTCGCTT	1.14265946	1.015041	0.488928	1.332326
tsrna-21014	GTCTAGGGGTATGATTCTCGCT	0.99982703	1.093122	0.838162	1.998488
tsrna-21013	GTCTAGGGGTATGATTCTCGC	0.99982703	0.702721	0.488928	1.332326
tsrna-21012	GTCTAGGGGTATGATTCTCG	0	0	0	0
tsrna-21011	GTCTAGGGGTATGATTCTC	0	0	0	0.111027
tsrna-21008	GTCTAGCGGTTAGGATTCCTGGTTTTCACC	0.64274595	1.015041	0.419081	0.444109
tsrna-21007	GTCTAGCGGTTAGGATTCCTGGTTTTC	0.35708108	0.702721	0.069847	0.555136
tsrna-21006	GTCTAGCGGTTAGGATTCCTGGTTTT	0.14283243	0.31232	0	0.111027
tsrna-21005	GTCTAGCGGTTAGGATTCCTGGTTT	0.21424865	0.31232	0.069847	0
tsrna-21004	GTCTAGCGGTTAGGATTCCTGGTT	0.57132973	0.468481	0.069847	0.111027
tsrna-21003	GTCTAGCGGTTAGGATTCCTGGT	0.07141622	0.23424	0	0.333081
tsrna-21002	GTCTAGCGGTTAGGATTCCTGG	0	0.23424	0.069847	0
tsrna-21001	GTCTAGCGGTTAGGATTCCTG	0.07141622	0	0	0
tsrna-20993	GTCTAAGGCGCCAGACTCAAG	0	0	0	0.111027
tsrna-20992	GTCTAACAACATGGCTTTCTCACCA	0	0.23424	0.139694	0
tsrna-20991	GTCTAACAACATGGCTTTCTCACC	0	0.07808	0	0
tsrna-20983	GTCGTGGTTGTAGTCCGTGCGAGAATACCA	0.92841081	1.717762	0.838162	1.332326
tsrna-20982	GTCGTGGTTGTAGTCCGTGCGAGAATACC	0.21424865	0.31232	0.20954	0.111027
tsrna-20981	GTCGTGGTTGTAGTCCGTGCGAGAATAC	0.14283243	0.15616	0.139694	0.111027

tsrna-20980	GTCGTGGTTGTAGTCCGTGCGAGAATA	0.07141622	0.23424	0.069847	0
tsrna-20979	GTCGTGGTTGTAGTCCGTGCGAGAAT	0	0.23424	0	0
tsrna-20978	GTCGTGGTTGTAGTCCGTGCGAGAA	0.14283243	0.07808	0.20954	0
tsrna-20977	GTCGTGGTTGTAGTCCGTGCGAGA	0	0.23424	0.069847	0
tsrna-20976	GTCGTGGTTGTAGTCCGTGCGAG	0	0.07808	0.069847	0
tsrna-20975	GTCGTGGTTGTAGTCCGTGCGA	0	0	0.139694	0
tsrna-20974	GTCGTGGTTGTAGTCCGTGCG	0.07141622	0	0.069847	0
tsrna-20968	GTCGTGGGTTCGAGCCCCACGTTGGGCGCCA	1.07124325	16.8653	0.20954	0.222054
tsrna-20967	GTCGTGGGTTCGAGCCCCACGTTGGGCGC	0	0.15616	0	0
tsrna-20966	GTCGTGGGTTCGAGCCCCACGTTGGGCG	0	0.07808	0	0
tsrna-20963	GTCGTGGCCGAGTGGTTAAG	0	0	0	0
tsrna-20961	GTCGGTGGAGCATGGGACT	0.14283243	0	0	0.222054
tsrna-20960	GTCGGTGGAGCATGGGAC	0.14283243	0	0	0
tsrna-20958	GTCGGTAGAGCATGGGACTCTTAATCCC	0.71416216	2.108163	1.047702	0.555136
tsrna-20957	GTCGGTAGAGCATGGGACTCTTAATCC	0.99982703	2.108163	0.558774	0.666163
tsrna-20956	GTCGGTAGAGCATGGGACTCTTAATC	0.64274595	1.249282	0.279387	0.111027
tsrna-20955	GTCGGTAGAGCATGGGACTCTTAAT	0.21424865	1.249282	0.20954	0.111027
tsrna-20954	GTCGGTAGAGCATGGGACTCTTA	0.64274595	1.405442	0.279387	0.222054
tsrna-20953	GTCGGTAGAGCATGGGACTCTT	0.57132973	1.327362	0.139694	0.222054
tsrna-20952	GTCGGTAGAGCATGGGACTCT	0.28566487	1.015041	0.279387	0
tsrna-20951	GTCGGTAGAGCATGGGACTC	0.78557838	1.093122	0.20954	0
tsrna-20950	GTCGGTAGAGCATGGGACT	0.4284973	0.31232	0.139694	0.222054
tsrna-20949	GTCGGTAGAGCATGGGAC	0.21424865	0.858881	0.419081	0.222054
tsrna-20948	GTCGGTAGAGCATGGGA	0.49991351	0.624641	0.279387	0
tsrna-20947	GTCGGTAGAGCATGGG	0.07141622	0.15616	0	0
tsrna-20946	GTCGGTAGAGCATGAGACTCTTAATCTC	0.99982703	1.249282	0.419081	0.888217
tsrna-20945	GTCGGTAGAGCATGAGACTCTTAATCT	0.78557838	1.873923	0.768315	0.111027
tsrna-20944	GTCGGTAGAGCATGAGACTCTTAA	0.78557838	1.327362	0.20954	0.888217
tsrna-20943	GTCGGTAGAGCATGAGACTCTT	0.71416216	2.342403	0.20954	0.333081
tsrna-20942	GTCGGTAGAGCATGAGACTCT	1.2854919	1.249282	0.419081	0.444109
tsrna-20941	GTCGGTAGAGCATGAGACTC	0.8569946	2.108163	0.279387	0.333081
tsrna-20940	GTCGGTAGAGCATGAGACT	0.49991351	1.952003	0.419081	0
tsrna-20939	GTCGGTAGAGCATGAGAC	0.71416216	1.483522	0.069847	0.222054
tsrna-20938	GTCGGTAGAGCATGAGA	0.07141622	0.468481	0	0.111027
tsrna-20937	GTCGGTAGAGCATGAG	0.14283243	0.31232	0.069847	0
tsrna-20936	GTCGGTAGAGCATCAGACTTTTAATCT	0.35708108	0.546561	0.139694	0
tsrna-20935	GTCGGTAGAGCATCAGACTTTTAATC	0.14283243	0.23424	0.069847	0
tsrna-20934	GTCGGTAGAGCATCAGACTTTTAAT	0.28566487	0.390401	0	0
tsrna-20933	GTCGGTAGAGCATCAGACTTTTAA	0.07141622	0.15616	0.069847	0
tsrna-20932	GTCGGTAGAGCATCAGACTTTTA	0.28566487	0.23424	0.069847	0
tsrna-20931	GTCGGTAGAGCATCAGACTTTT	0	0.23424	0	0
tsrna-20930	GTCGGTAGAGCATCAGACTTT	0	0.07808	0.139694	0
tsrna-20929	GTCGGTAGAGCATCAGACTT	0.14283243	0	0.139694	0
tsrna-20928	GTCGGTAGAGCATCAGACT	0.07141622	0.468481	0	0
tsrna-20927	GTCGGTAGAGCATCAGAC	0	0.07808	0.20954	0
tsrna-20926	GTCGGTAGAGCATCAGA	0.14283243	0	0	0
tsrna-20925	GTCGGTAGAGCATCAG	0.07141622	0	0	0
tsrna-20924	GTCGCTGGTTCGATTCCGGCTCGAAGGACC	0.4284973	2.108163	0.069847	0.555136
tsrna-20917	GTCGCAGTCTCCCCTGGAGG	0	0.15616	0	0
tsrna-20916	GTCGATTCCCCGACGGGGAGCCA	0	1.015041	0	0
tsrna-20914	GTCGATGGATCGAAACCATCCTCTGCTACC	0.21424865	0.15616	0	0
tsrna-20912	GTCCTTGTAGTATAAACTAATACACCAGTC	0.07141622	0.07808	0.20954	0.111027
tsrna-20911	GTCCTTGTAGTATAAACTAATACACCAGT	0.4284973	0.15616	0.279387	0.222054
tsrna-20910	GTCCTTGTAGTATAAACTAATACACCAG	0.49991351	0.15616	0.069847	0.333081
tsrna-20909	GTCCTTGTAGTATAAACTAATACACCA	0.4284973	0.07808	0	0
tsrna-20908	GTCCTTGTAGTATAAACTAATACACC	0.71416216	0.07808	0	0.333081
tsrna-20907	GTCCTTGTAGTATAAACTAATACAC	0.57132973	0.07808	0.069847	0.222054
tsrna-20906	GTCCTTGTAGTATAAACTAATACA	0.4284973	0.07808	0.139694	0.333081
tsrna-20905	GTCCTTGTAGTATAAACTAATAC	0.49991351	0.07808	0.139694	0.222054
tsrna-20904	GTCCTTGTAGTATAAACTAATA	0.4284973	0.07808	0.139694	0.111027
tsrna-20903	GTCCTTGTAGTATAAACTAAT	0.21424865	0.15616	0	0
tsrna-20902	GTCCTTGTAGTATAAACTAA	0.21424865	0.23424	0.069847	0
tsrna-20901	GTCCTTGTAGTATAAACTA	0	0	0	0.111027
tsrna-20900	GTCCTTGTAGTATAAACT	0	0	0.069847	0.111027

tsrna-20899	GTCCTTGTAGTATAAAC	0.07141622	0	0	0
tsrna-20891	GTCCTGCCGCGGTCGCCA	0.07141622	0.31232	0.139694	0
tsrna-20887	GTCCTCGTTAGTATAGTGGTGAG	0.07141622	0	0	0
tsrna-20886	GTCCGTGCGAGAATACCA	0.07141622	0	0	0
tsrna-20883	GTCCCTTCGTGGTCGCCA	0	0.07808	0	0
tsrna-20881	GTCCCTGTTCGGGCGCCA	0.57132973	0.702721	0.20954	0.222054
tsrna-20878	GTCCCTGTTCAGGCGCCA	0	0	0	0
tsrna-20872	GTCCCTGGTTCGATCCCGG	0	0	0	0
tsrna-20863	GTCCCGGGTTCAAATCCCGGACGAG	0	0	0	0
tsrna-20855	GTCCCGGCGGAGTCGCCA	0	0	0	0
tsrna-20845	GTCCCATTGGTCTAGCCA	0	0.07808	0	0
tsrna-20842	GTCCCATGGTGTAATGGTTAGCACTCTGG	0	0.07808	0.069847	0
tsrna-20837	GTCCCATCTGGGTCGCCA	0.07141622	0.31232	0.069847	0.111027
tsrna-20836	GTCCCATCTGGGTCGCC	0	0.15616	0	0
tsrna-20835	GTCCCATCTGGGGTGCCA	0	0	0	0
tsrna-20828	GTCCCACCAGAGTCGCCA	0	0.07808	0	0
tsrna-20825	GTCCAGGGTTCAAGTCCCTGTTCGGGCGCCA	0.35708108	1.093122	0.20954	0.333081
tsrna-20824	GTCCAGGGTTCAAGTCCCTGTTCGGGCGCC	0.07141622	0	0	0
tsrna-20823	GTCCAGGGTTCAAGTCCCTGTTCGGGCGC	0	0	0	0.111027
tsrna-20822	GTCATTTCGATGGCGTGGGTTCG	0.21424865	0.31232	0.349234	0
tsrna-20821	GTCATTTCGATGGCGTGG	0	0.07808	0	0
tsrna-20820	GTCATGGAGGCCATGGGGTTGGCTTGA	0	0.07808	0.069847	0.111027
tsrna-20819	GTCATGGAGGCCATGGGGTTGGC	0	0.07808	0	0
tsrna-20818	GTCATGGAGGCCATGGGGTTGG	0	0.07808	0	0
tsrna-20817	GTCATGGAGGCCATGGGGTTG	0	0.07808	0	0
tsrna-20816	GTCATGGAGGCCATGGGGTT	0	0	0.069847	0
tsrna-20811	GTCAGGATGGCCGAGTGGTCTAAGGCGCCAGACT	0.14283243	0.546561	0.139694	0
tsrna-20810	GTCAGGATGGCCGAGTGGTCTAAGGCGCCAGAC	0	0.468481	0.20954	0.111027
tsrna-20809	GTCAGGATGGCCGAGTGGTCTAAGGCGCCAGA	0.21424865	0.390401	0	0.111027
tsrna-20808	GTCAGGATGGCCGAGTGGTCTAAGGCGCCA	0.21424865	0.390401	0.139694	0.666163
tsrna-20807	GTCAGGATGGCCGAGTGGTCTAAGGCGCC	0.4284973	0.23424	0.349234	0.444109
tsrna-20806	GTCAGGATGGCCGAGTGGTCTAAGGCGC	0.14283243	0.546561	0	0.444109
tsrna-20805	GTCAGGATGGCCGAGTGGTCTAAGGC	0.21424865	0.936961	0.279387	0.444109
tsrna-20804	GTCAGGATGGCCGAGTGGTCTAAGG	0	0.390401	0	0.333081
tsrna-20803	GTCAGGATGGCCGAGTGGTCTAAG	0.14283243	0.546561	0	0
tsrna-20802	GTCAGGATGGCCGAGTGGTCTAA	0.21424865	0.546561	0.069847	0.111027
tsrna-20801	GTCAGGATGGCCGAGTGGTCTA	0	0.468481	0.20954	0.111027
tsrna-20800	GTCAGGATGGCCGAGTGGTCT	0.07141622	0.780801	0	0.222054
tsrna-20799	GTCAGGATGGCCGAGTGGTC	0.28566487	0.468481	0.139694	0.111027
tsrna-20798	GTCAGGATGGCCGAGTGGT	0.35708108	0.15616	0.20954	0.222054
tsrna-20797	GTCAGGATGGCCGAGTGG	0	0.23424	0.069847	0
tsrna-20796	GTCAGGATGGCCGAGTG	0.21424865	0.15616	0.139694	0
tsrna-20795	GTCAGGATGGCCGAGT	0.07141622	0.468481	0.20954	0
tsrna-20794	GTCAGGATGGCCGAGCGGTCTAAGGCGCTGCGTTC	0.35708108	0.936961	0.069847	0.333081
tsrna-20793	GTCAGGATGGCCGAGCGGTCTAAGGCGCTGCGTT	0.14283243	0.858881	0.20954	0.666163
tsrna-20792	GTCAGGATGGCCGAGCGGTCTAAGGCGCTGCGT	0.35708108	1.015041	0.20954	0
tsrna-20791	GTCAGGATGGCCGAGCGGTCTAAGGCGCTGCG	0.4284973	1.249282	0.349234	0.222054
tsrna-20790	GTCAGGATGGCCGAGCGGTCTAAGGCGCTGC	0.28566487	1.093122	0.279387	0.222054
tsrna-20789	GTCAGGATGGCCGAGCGGTCTAAGGCGCTG	0.4284973	1.405442	0.279387	0.444109
tsrna-20788	GTCAGGATGGCCGAGCGGTCTAAGGCGCT	0.21424865	0.936961	0.139694	0.666163
tsrna-20787	GTCAGGATGGCCGAGCGGTCTAAGGCGC	0.21424865	0.858881	0.069847	0.555136
tsrna-20786	GTCAGGATGGCCGAGCGGTCTAAGGCG	0.57132973	1.561602	0	0.555136
tsrna-20785	GTCAGGATGGCCGAGCGGTCTAAGGC	0.28566487	1.249282	0.139694	0.333081
tsrna-20784	GTCAGGATGGCCGAGCGGTCTAAGG	0.35708108	0.780801	0.279387	0.333081
tsrna-20783	GTCAGGATGGCCGAGCGGTCTAAG	0.21424865	0.390401	0.20954	0.333081
tsrna-20782	GTCAGGATGGCCGAGCGGTCTAA	0.21424865	0.31232	0	0.333081
tsrna-20781	GTCAGGATGGCCGAGCGGTCTA	0.14283243	0.468481	0.279387	0.222054
tsrna-20780	GTCAGGATGGCCGAGCGGTCT	0.07141622	0.390401	0.069847	0.333081
tsrna-20779	GTCAGGATGGCCGAGCGGTC	0.14283243	0.624641	0	0.222054
tsrna-20778	GTCAGGATGGCCGAGCGGT	0	0.702721	0	0.111027
tsrna-20777	GTCAGGATGGCCGAGCGG	0	0.546561	0	0.111027
tsrna-20776	GTCAGGATGGCCGAGCG	0.07141622	1.249282	0.139694	0.111027
tsrna-20775	GTCAGGATGGCCGAGC	0.07141622	0.624641	0	0.111027
tsrna-20774	GTCAGCTAAATAAGCTATCGGGCCCAT	0.07141622	0.07808	0	0

tsrna-20773	GTCAGCTAAATAAGCTATCGGGCCCA	0	0.07808	0	0
tsrna-20772	GTCAGCTAAATAAGCTATCGGGCCC	0.07141622	0	0	0
tsrna-20767	GTCAGCACTCTGGACTCTGAATCCAGC	0.21424865	0.31232	0	0
tsrna-20766	GTCAGCACTCTGGACTCTGAATCC	0	0	0	0
tsrna-20765	GTCAGCACTCTGGACTCTGAATC	0.07141622	0	0	0
tsrna-20758	GTCACGTCGGGGTCACCA	0.49991351	0.546561	0.069847	0.444109
tsrna-20757	GTCACGTCGGGGTCACC	0	0	0	0
tsrna-20755	GTCACGGTGGCCGAGTGGTTAAGGCGTTGG	0.07141622	0.390401	0.139694	0
tsrna-20754	GTCACGGTGGCCGAGTGGTTAAGGCGTTG	0.21424865	0.468481	0	0.111027
tsrna-20753	GTCACGGTGGCCGAGTGGTTAAGGC	0.07141622	0.390401	0.139694	0.111027
tsrna-20752	GTCACGGTGGCCGAGTGGTTAAGG	0.21424865	0.702721	0.069847	0
tsrna-20751	GTCACGGTGGCCGAGTGGTTAAG	0.07141622	0.936961	0	0
tsrna-20750	GTCACGGTGGCCGAGTGGTTAA	0.07141622	0.31232	0	0.111027
tsrna-20749	GTCACGGTGGCCGAGTGGTTA	0.14283243	0.546561	0.069847	0
tsrna-20748	GTCACGGTGGCCGAGTGGTT	0.07141622	0.468481	0	0.111027
tsrna-20747	GTCACGGTGGCCGAGTGGT	0.07141622	0.31232	0	0
tsrna-20746	GTCACGGTGGCCGAGTGG	0.07141622	0.858881	0	0
tsrna-20745	GTCACGGTGGCCGAGTG	0.07141622	0.23424	0	0
tsrna-20744	GTCACGGTGGCCGAGT	0	0.23424	0	0
tsrna-20743	GTCACGCGGGAGACCGGGGTTCGATTCCCCGACGGGGAGC(	0	1.561602	0.069847	0
tsrna-20737	GTCAAAGTTAAATTATAGGCTAAATCCTA	0.49991351	0	0.139694	0.111027
tsrna-20736	GTCAAAGTTAAATTATAGGCTAAATCCT	0.14283243	0.07808	0.069847	0
tsrna-20735	GTCAAAGTTAAATTATAGGCT	0	0	0.069847	0
tsrna-20734	GTCAAAGTTAAATTATAGGC	0	0	0.139694	0
tsrna-20733	GTCAAAGTTAAATTATAGG	0	0.07808	0	0
tsrna-20730	GTATGATTCTCGGTTTGGGTCCGAGA	0.14283243	0	0	0
tsrna-20728	GTATGATTCTCGGTTTGGGTC	0	0	0.069847	0
tsrna-20727	GTATGATTCTCGGTTTGGGT	0.07141622	0	0	0
tsrna-20725	GTATGATTCTCGGTTTGG	0	0.07808	0	0
tsrna-20722	GTATGATTCTCGCTTTGGGTGCGAGAGGTCCCGGGT	0.57132973	4.294406	0.488928	1.665407
tsrna-20721	GTATGATTCTCGCTTTGGGTG	0.07141622	0	0	0
tsrna-20718	GTATGATTCTCGCTTT	0	0.07808	0	0
tsrna-20715	GTATGATTCTCGCTTCGGGTGCGAGAGGTC	0.07141622	0	0	0
tsrna-20712	GTATGATTCTCGCTTCGGGTG	0.07141622	0.07808	0	0
tsrna-20711	GTATGATTCTCGCTTCGGG	0.07141622	0.07808	0	0
tsrna-20710	GTATGATTCTCGCTTCGG	0.07141622	0	0	0
tsrna-20707	GTATGAGGTCCCGGGTTCGATCCCCGGCATCTCCACC	1.14265946	2.186243	0.419081	0.333081
tsrna-20706	GTATGAGGTCCCGGGTTCGATCCCCGGC	0.28566487	0.07808	0.069847	0
tsrna-20705	GTATGAGGTCCCGGGTTCGAT	0	0.31232	0	0
tsrna-20704	GTATGAGGTCCCGGGTTCG	0	0.31232	0.069847	0.222054
tsrna-20703	GTATGAGGTCCCGGGTTC	0.07141622	0.15616	0	0
tsrna-20702	GTATGAGGTCCCGGGTT	0	0.31232	0	0
tsrna-20701	GTATGAGGTCCCGGGT	0	0.390401	0	0
tsrna-20700	GTATGAGGCCTCGGGT	0.07141622	0.390401	0.139694	0
tsrna-20699	GTATGAGGCCCCGGGTTCGATCCCCGGCATCTCCACC	0.78557838	1.561602	0.768315	0
tsrna-20698	GTATGAGGCCCCGGGTTCGATCCCCGGC	0	0.15616	0	0.111027
tsrna-20697	GTATGAGGCCCCGGGTTCGATCC	0	0.23424	0	0.111027
tsrna-20696	GTATGAGGCCCCGGGTTCGAT	0	0.390401	0	0
tsrna-20695	GTATGAGGCCCCGGGTTCG	0.07141622	0	0.069847	0
tsrna-20694	GTATGAGGCCCCGGGTTCAATCCCCGGC	0.21424865	0.07808	0.069847	0
tsrna-20693	GTATGAGGCCCCGGGTTC	0	0.31232	0	0.111027
tsrna-20692	GTATGAGGCCCCGGGTT	0	0.15616	0	0
tsrna-20691	GTATGAGGCCCCGGGT	0	0.07808	0	0
tsrna-20690	GTATCCCCGCCTGTCACGCGGGAGACCGG	0.64274595	3.201285	0.768315	0.444109
tsrna-20689	GTATCCCCGCCTGTCACGCGGG	0.4284973	2.264323	0.139694	0.111027
tsrna-20683	GTATCATGCAAGATTCCCATTC	0	0	0	0
tsrna-20678	GTATAGTGGTTAGTATCCCCG	0	0	0.069847	0
tsrna-20673	GTATAGTGGTTAGTACTCTGCGTTGTGGCC	1.78540541	0.858881	0.977855	1.110271
tsrna-20672	GTATAGTGGTTAGTACTCTGCGTTGTGGC	2.49956757	1.171202	0.349234	0.666163
tsrna-20671	GTATAGTGGTTAGTACTCTGCGTTGTG	1.71398919	1.015041	0.768315	0.77719
tsrna-20670	GTATAGTGGTTAGTACTCTGCGTTGT	1.85682163	1.327362	1.047702	0.888217
tsrna-20669	GTATAGTGGTTAGTACTCTGCGTTG	1.35690811	1.249282	0.698468	1.110271
tsrna-20668	GTATAGTGGTTAGTACTCTGCGTT	0.8569946	0.546561	0.628621	0.666163
tsrna-20667	GTATAGTGGTTAGTACTCTGCGT	0.28566487	0.23424	0	0.222054

tsrna-20666	GTATAGTGGTTAGTACTCTGCG	0.14283243	0.07808	0	0.111027
tsrna-20665	GTATAGTGGTTAGTACTCTGC	0.07141622	0	0	0.111027
tsrna-20664	GTATAGTGGTTAGTACTCTG	0	0	0.069847	0
tsrna-20662	GTATAGTGGTTAGTACTC	0	0	0	0
tsrna-20658	GTATAGTGGTTAGCATAGCTGCCTT	0	0	0	0
tsrna-20653	GTATAGTGGTGAGTATCCCCGCCTGTC	0.14283243	0.858881	0.069847	0.111027
tsrna-20652	GTATAGTGGTGAGTATCCCCGCCTGT	0.07141622	0.468481	0	0.111027
tsrna-20651	GTATAGTGGTGAGTATCCCCGCCTG	0.14283243	0.23424	0	0.111027
tsrna-20650	GTATAGTGGTGAGTATCCCCGCCT	0.07141622	0.468481	0	0
tsrna-20649	GTATAGTGGTGAGTATCCCCGCC	0.21424865	0.31232	0.139694	0
tsrna-20648	GTATAGTGGTGAGTATCCCCGC	0.07141622	0.23424	0	0
tsrna-20647	GTATAGTGGTGAGTATCCCCG	0	0.07808	0	0
tsrna-20645	GTATAGTGGTGAGTATCCC	0	0.07808	0	0
tsrna-20644	GTATAGTGGTGAGTATCC	0.07141622	0.07808	0	0
tsrna-20640	GTATAGTGGTGAGCATAGCTGCCTTC	0	0.07808	0	0
tsrna-20639	GTATAGTGGTGAGCATAGCTGCCTT	0.07141622	0.07808	0	0.111027
tsrna-20638	GTATAGTGGTGAGCATAGCTGCCT	0	0.07808	0.069847	0
tsrna-20636	GTATAGTGGTGAGCATAGCTGC	0	0.07808	0	0
tsrna-20635	GTATAGTGGTGAGCATAGCTG	0	0.07808	0	0
tsrna-20633	GTATAGTGGTGAGCATAGC	0.07141622	0	0	0
tsrna-20629	GTATAGCTCAGTGGTAGAGCATTTGACTGC	0.57132973	0.468481	0.279387	0
tsrna-20628	GTATAGCTCAGTGGTAGAGCATTTGACTG	0.4284973	0.07808	0	0.111027
tsrna-20627	GTATAGCTCAGTGGTAGAGCATTTGACTAC	0.21424865	0.31232	0	0.111027
tsrna-20626	GTATAGCTCAGTGGTAGAGCATTTGACT	0.07141622	0.15616	0.069847	0
tsrna-20625	GTATAGCTCAGTGGTAGAGCATTTGAC	0.28566487	0	0	0
tsrna-20624	GTATAGCTCAGTGGTAGAGCATTTG	0.07141622	0.07808	0	0
tsrna-20621	GTATAGCTCAGTGGGTAGAGCATTTGACTG	0.14283243	0	0	0.111027
tsrna-20620	GTATAGCTCAGTGGGTAGAGCATTTGACT	0	0.07808	0	0
tsrna-20619	GTATAGCTCAGGTGGTAGAGCATTTGACTG	0.07141622	0	0	0
tsrna-20618	GTATAGCTCAGGGGTAGAGCATTTGACTGC	0.14283243	0.23424	0.20954	0.888217
tsrna-20617	GTATAGCTCAGGGGTAGAGCATTTGACTG	0	0	0	0.222054
tsrna-20616	GTATAGCTCAGGGGTAGAGCATTTGACT	0	0.23424	0	0.222054
tsrna-20615	GTATAAATAGTACCGTTAACTTCC	0.07141622	0	0.069847	0
tsrna-20614	GTATAAATAGTACCGTTAACTTC	0	0	0	0
tsrna-20600	GTAGTGTGGCCGAGCGGTCT	0	0	0	0
tsrna-20599	GTAGTGTAGTGGTTATCACGTTCGCCTC	0.07141622	0.07808	0	0
tsrna-20598	GTAGTGTAGTGGTTATCACGTTCGCCT	0.14283243	0	0	0
tsrna-20597	GTAGTGTAGTGGTTATCACGTTCGCC	0	0.07808	0	0
tsrna-20596	GTAGTGTAGTGGTTATCACGTTCGC	0.14283243	0	0.069847	0
tsrna-20595	GTAGTGTAGTGGTTATCACGTTCG	0	0	0	0.111027
tsrna-20594	GTAGTGTAGTGGTTATCACGTTC	0	0	0	0
tsrna-20592	GTAGTGTAGTGGTTATCACG	0	0.07808	0	0
tsrna-20591	GTAGTGTAGTGGTTATCAC	0	0.07808	0	0.111027
tsrna-20583	GTAGTGTAGTGGTCATC	0.14283243	0	0	0
tsrna-20582	GTAGTGTAGTGGTCAT	0.07141622	0	0	0
tsrna-20580	GTAGTGTAGCGGTTATCAC	0.07141622	0	0	0
tsrna-20574	GTAGTGGTTATCACGTTCGCCTA	0.07141622	0	0	0
tsrna-20571	GTAGTGGTTATCACGTTCGC	0	0	0	0
tsrna-20567	GTAGTGGTTATCACGTCTGCTTTAC	0.21424865	0	0.139694	0
tsrna-20566	GTAGTGGTTATCACGTCTGCTTTA	0.07141622	0	0	0
tsrna-20565	GTAGTGGTTATCACGTCTGCTTT	0	0	0	0
tsrna-20554	GTAGTGGTTATCACATCTGCTT	0	0	0	0
tsrna-20552	GTAGTGGTCATCACGTTCGCC	0	0.07808	0	0
tsrna-20551	GTAGTGGTCATCACGTTCGC	0	0.07808	0	0
tsrna-20550	GTAGTGGTATCATGCAAGATTCCCATTCTT	0.07141622	0.07808	0.20954	0
tsrna-20549	GTAGTGGTATCATGCAAGATTCCC	0	0	0	0
tsrna-20548	GTAGTGGTATCATGCAAGATTCC	0	0	0	0
tsrna-20547	GTAGTGGTATCATGCAAGATTC	0	0	0	0
tsrna-20545	GTAGTGGTATCATGCAAGAT	0	0	0.069847	0
tsrna-20542	GTAGTCGTGGCCGAGTGGTTAAGGTGATGG	0.35708108	1.249282	0.349234	0.77719
tsrna-20541	GTAGTCGTGGCCGAGTGGTTAAGGTGA	0.4284973	1.873923	0.20954	0.999244
tsrna-20540	GTAGTCGTGGCCGAGTGGTTAAGGT	0.99982703	1.561602	0.349234	0.444109
tsrna-20539	GTAGTCGTGGCCGAGTGGTTAAGGCGATGG	0.4284973	1.483522	0.139694	1.110271
tsrna-20538	GTAGTCGTGGCCGAGTGGTTAAGGCGATG	0.71416216	1.561602	0.628621	0.555136

tsrna-20537	GTAGTCGTGGCCGAGTGGTTAAGGCGA	0.92841081	1.093122	0.419081	0.111027
tsrna-20536	GTAGTCGTGGCCGAGTGGTTAAGGCG	0.78557838	1.093122	0.20954	0.444109
tsrna-20535	GTAGTCGTGGCCGAGTGGTTAAGGC	0.71416216	1.405442	0.628621	0.444109
tsrna-20534	GTAGTCGTGGCCGAGTGGTTAAGG	0.28566487	1.327362	0.419081	0.444109
tsrna-20533	GTAGTCGTGGCCGAGTGGTTAAG	0.28566487	1.249282	0.419081	0.888217
tsrna-20532	GTAGTCGTGGCCGAGTGGTTAA	0.49991351	1.873923	0.20954	0.333081
tsrna-20531	GTAGTCGTGGCCGAGTGGTTA	0.78557838	1.249282	0.20954	0.222054
tsrna-20530	GTAGTCGTGGCCGAGTGGTT	0.99982703	1.327362	0.488928	0.77719
tsrna-20529	GTAGTCGTGGCCGAGTGGT	0.64274595	1.093122	0.349234	0.222054
tsrna-20528	GTAGTCGTGGCCGAGTGG	0.28566487	1.405442	0.20954	0.333081
tsrna-20527	GTAGTCGTGGCCGAGTG	0.07141622	0.468481	0.069847	0.444109
tsrna-20526	GTAGTCGTGGCCGAGT	0.21424865	0.546561	0.139694	0.222054
tsrna-20525	GTAGTCCGTGCGAGAATACCA	0.07141622	0.23424	0	0
tsrna-20523	GTAGTCCGTGCGAGAATAC	0	0	0	0
tsrna-20521	GTAGTCCGTGCGAGAAT	0	0	0	0
tsrna-20519	GTAGTATAAACTAATACACCAGT	0	0	0.069847	0
tsrna-20517	GTAGTATAAACTAATACACCA	0	0	0.069847	0
tsrna-20514	GTAGCTTACCTCCTCAAAGCAATA	0	0	0	0
tsrna-20508	GTAGCTCAGTGGTAGAGCGCGTGC	0.8569946	2.030083	0.20954	0
tsrna-20507	GTAGCTCAGTGGTAGAGCGCATGCTT	0	0	0	0
tsrna-20505	GTAGCTCAGTGGTAGAGCGCATGC	0	0	0	0
tsrna-20500	GTAGCTCAGTGGTAGAGCATTTGACTGC	0.07141622	0.15616	0.069847	0.222054
tsrna-20499	GTAGCTCAGTGGTAGAGCATTTGACTG	0.21424865	0	0	0
tsrna-20498	GTAGCTCAGTGGTAGAGCATTTGACT	0	0.15616	0.069847	0
tsrna-20497	GTAGCTCAGTGGTAGAGCATTTGAC	0	0	0	0
tsrna-20490	GTAGCGTGGCCGAGTGGTCTAAGGCGCT	0	0	0	0
tsrna-20489	GTAGCGTGGCCGAGTGGTCTAAGGC	0	0	0	0.111027
tsrna-20488	GTAGCGTGGCCGAGTGGTCTAAGG	0.07141622	0	0	0
tsrna-20487	GTAGCGTGGCCGAGTGGTCTA	0.14283243	0	0	0
tsrna-20483	GTAGCGTGGCCGAGCGGTCTAAGGCGCTG	0	0.07808	0	0
tsrna-20482	GTAGCGTGGCCGAGCGGTCTAAGGCGCT	0	0.23424	0	0
tsrna-20481	GTAGCGTGGCCGAGCGGTCTAAGGC	0	0.07808	0	0
tsrna-20479	GTAGCGTGGCCGAGCGGTCTAAG	0	0	0.069847	0
tsrna-20477	GTAGCGTGGCCGAGCGGTCTA	0	0	0	0
tsrna-20476	GTAGCGTGGCCGAGCGGTCT	0	0.07808	0	0
tsrna-20475	GTAGCGTGGCCGAGCGGTC	0	0	0.069847	0
tsrna-20474	GTAGCGTGGCCGAGCGGT	0	0.07808	0	0
tsrna-20473	GTAGCGTGGCCGAGCGG	0	0	0	0.111027
tsrna-20472	GTAGCGTGGCCGAGCG	0.07141622	0	0	0.111027
tsrna-20471	GTAGCGGTTATCACGTCTGCTTT	0	0	0	0
tsrna-20463	GTAGATTGAAGCCAGTTGATTAGGGTGCTT	0.14283243	0.07808	0.139694	0.111027
tsrna-20462	GTAGATTGAAGCCAGTTGATT	0.14283243	0.07808	0	0
tsrna-20455	GTAGAGCATTTGACTGC	0	0.07808	0	0
tsrna-20454	GTAGAGCATTTGACTG	0	0	0	0
tsrna-20453	GTAGAGCATGGGACTCTTAATCCCAGGGTCGTGGGT	0.21424865	0.546561	0.139694	0.222054
tsrna-20452	GTAGAGCATGGGACTCTTAATCC	0.07141622	0.23424	0	0
tsrna-20450	GTAGAGCATGAGACTCTTAATCTCAGGGTCGTGGGT	0.71416216	1.093122	0.279387	0.77719
tsrna-20449	GTAGAGCATGAGACTC	0	0	0	0
tsrna-20448	GTAGAGCATCAGACTTTTAATCTGAGGGTCCAGGGT	0.92841081	3.513605	0.488928	0.444109
tsrna-20447	GTAGAGCATCAGACTTTTAATC	0.07141622	0	0	0
tsrna-20442	GTAGAATTCTCGCCTGCCACGCGGGAGGCCCGGGTTT	3.78505947	6.714889	2.654179	2.33157
tsrna-20441	GTAGAATTCTCGCCTGCCACGCGGGAGGCCCGGGTTC	3.28514596	5.777928	3.562187	1.887461
tsrna-20440	GTAGAATTCTCGCCTGCCACGCGGGAGGCCCGGGTT	3.71364325	6.94913	1.955711	1.221299
tsrna-20439	GTAGAATTCTCGCCTGCCACGCGGGAGGCCCGGGT	4.07072434	4.372486	1.53663	1.55438
tsrna-20438	GTAGAATTCTCGCCTGCCACGCGGGAGGCCCGGG	3.4993946	3.669765	2.025557	1.776434
tsrna-20437	GTAGAATTCTCGCCTGCCACGCGGGAGGCCCGG	2.85664866	3.435525	2.095404	1.221299
tsrna-20436	GTAGAATTCTCGCCTGCCACGCGGGAGGCCCG	2.99948109	3.357445	1.885864	1.665407
tsrna-20435	GTAGAATTCTCGCCTGCCACGCGGGAGGCCC	3.57081082	2.654724	1.257243	1.332326
tsrna-20434	GTAGAATTCTCGCCTGCCACGCGGGAGGCC	2.57098379	3.201285	1.396936	1.887461
tsrna-20433	GTAGAATTCTCGCCTGCCACGCGGGAGGC	2.99948109	3.591685	1.466783	1.776434
tsrna-20432	GTAGAATTCTCGCCTGCCACGCGGGAGG	3.21372974	2.498564	1.606477	1.221299
tsrna-20431	GTAGAATTCTCGCCTGCCACGCGGGAG	1.92823784	3.591685	1.955711	2.442597
tsrna-20430	GTAGAATTCTCGCCTGCCACGCGGGA	2.85664866	3.747845	1.53663	1.776434
tsrna-20429	GTAGAATTCTCGCCTGCCACGCGGG	2.35673514	2.498564	1.53663	1.221299

tsrna-20428	GTAGAATTCTCGCCTGCCACGCGG	2.64240001	3.435525	0.908008	1.443353
tsrna-20427	GTAGAATTCTCGCCTGCCACGCG	2.78523244	3.045124	1.74617	2.220543
tsrna-20426	GTAGAATTCTCGCCTGCCACGC	2.64240001	1.952003	1.117549	1.887461
tsrna-20425	GTAGAATTCTCGCCTGCCACG	2.14248649	2.498564	0.698468	1.55438
tsrna-20424	GTAGAATTCTCGCCTGCCAC	0.49991351	0.15616	0.349234	0.444109
tsrna-20423	GTAGAATTCTCGCCTGCCA	0.21424865	0.23424	0	0.111027
tsrna-20422	GTAGAATTCTCGCCTGCC	0.14283243	0	0	0.111027
tsrna-20421	GTAGAATTCTCGCCTGC	0.14283243	0.07808	0.069847	0.111027
tsrna-20420	GTAGAATTCTCGCCTG	0.14283243	0	0	0
tsrna-20419	GTAGAATTCTCGCCTCCCACGCGGGAGACCCGGGTT	0.14283243	0.15616	0	0.111027
tsrna-20418	GTAGAATTCTCGCCTCCCACGCGGGAGACCCGGGT	0.14283243	0.23424	0	0.222054
tsrna-20417	GTAGAATTCTCGCCTCCCACG	0.21424865	0.07808	0	0.222054
tsrna-20416	GTAGAATTCTCGCCTCCC	0.07141622	0	0.069847	0
tsrna-20415	GTAGAATTCTCGCCTCC	0.14283243	0	0.069847	0.111027
tsrna-20414	GTAGAATTCTCGCCTC	0.07141622	0	0	0
tsrna-20413	GTACTTATAATGCCGAGGTTGTG	0.14283243	0.780801	0.069847	0.111027
tsrna-20411	GTACTCTGCGTTGTGGCCGCA	0.07141622	0	0	0
tsrna-20405	GTACGAGGCCCCGGGTTCG	0	0.31232	0	0
tsrna-20403	GTACCGTTAACTTCCAATTAACTAGTTTTG	0	0	0.069847	0
tsrna-20399	GTACCGTTAACTTCCAATTA	0	0	0.069847	0
tsrna-20395	GTAATGGTTAGCACTCTGGGCTT	0	0	0	0
tsrna-20393	GTAATGGTTAGCACTCTGGGC	0	0	0	0
tsrna-20392	GTAATGGTTAGCACTCTGGACTTTGAATCC	0.4284973	0.390401	0.20954	0.111027
tsrna-20391	GTAATGGTTAGCACTCTGGACTTTGAATC	0.07141622	0.31232	0.139694	0
tsrna-20390	GTAATGGTTAGCACTCTGGACTTTGAAT	0	0.07808	0.069847	0
tsrna-20389	GTAATGGTTAGCACTCTGGACTTTGAA	0	0	0.139694	0
tsrna-20388	GTAATGGTTAGCACTCTGGACTTTGA	0.07141622	0.07808	0	0
tsrna-20387	GTAATGGTTAGCACTCTGGACTTTG	0	0	0.069847	0
tsrna-20386	GTAATGGTTAGCACTCTGGACTTT	0	0	0	0.111027
tsrna-20385	GTAATGGTTAGCACTCTGGACTT	0.14283243	0.07808	0	0
tsrna-20384	GTAATGGTTAGCACTCTGGACTCTGAATCCAGCGAT	0.35708108	0.936961	0.279387	0.222054
tsrna-20383	GTAATGGTTAGCACTCTGGACTCTGAATCC	0.35708108	0.23424	0.069847	0
tsrna-20382	GTAATGGTTAGCACTCTGGACTCTGAATC	0	0	0	0.111027
tsrna-20381	GTAATGGTTAGCACTCTGGACTCTGAAT	0	0.07808	0	0
tsrna-20380	GTAATGGTTAGCACTCTGGACTCTGAA	0.14283243	0	0	0
tsrna-20379	GTAATGGTTAGCACTCTGGACTCTGA	0	0	0	0
tsrna-20378	GTAATGGTTAGCACTCTGGACTCTG	0	0	0	0
tsrna-20377	GTAATGGTTAGCACTCTGGACTCT	0.07141622	0	0	0
tsrna-20376	GTAATGGTTAGCACTCTGGACTC	0	0.07808	0	0
tsrna-20375	GTAATGGTTAGCACTCTGGACT	0	0	0	0
tsrna-20374	GTAATGGTTAGCACTCTGGAC	0.07141622	0.07808	0.069847	0
tsrna-20369	GTAATGGTTAGCACTC	0	0	0	0
tsrna-20368	GTAATGGTGAGCACTTTGGACTCTGA	0	0.31232	0	0
tsrna-20367	GTAATGGTGAGCACTCTGGACTCTG	0	0	0	0
tsrna-20364	GTAATGGTGAGCACTCTGGACT	0	0.07808	0	0
tsrna-20363	GTAATGGTGAGCACTCTGGAC	0	0.07808	0	0.111027
tsrna-20352	GTAATGGTAAGCACTCTGGACTCTGAATC	0	0	0.069847	0
tsrna-20351	GTAATGGTAAGCACTCTGGACTCTG	0	0	0	0.111027
tsrna-20346	GTAATGGTAAGCACTCTGG	0	0.07808	0	0
tsrna-20345	GTAATGGTAAGCACTCTG	0	0	0	0
tsrna-20344	GTAATCGCATAAAACTTAAAACTTTACAGT	0.28566487	0	0.069847	0.111027
tsrna-20343	GTAATCGCATAAAACTTAAAACTTTACAG	0.14283243	0.23424	0.20954	0
tsrna-20342	GTAATCGCATAAAACTTAAAACTTTACA	0.14283243	0	0.349234	0.111027
tsrna-20341	GTAATCGCATAAAACTTAAAACTT	0	0.07808	0	0
tsrna-20337	GTAATCGCATAAAACTTA	0	0.07808	0	0
tsrna-20336	GTAAGTTGCAATACTTAATTTCTGCCA	0.14283243	0	0.069847	0
tsrna-20335	GTAAGTTGCAATACTTAATTTCTGCC	0	0	0	0
tsrna-20330	GTAAGGTCAGCTAAATAAGCTATCGGGCCC	0.07141622	0	0.069847	0
tsrna-20325	GTAAGGTCAGCTAAATAAGCT	0	0	0	0
tsrna-20324	GTAAGGTCAGCTAAATAAGC	0	0	0.069847	0
tsrna-20319	GTAAGCACTCTGGACTCTGAATCC	0	0.07808	0.069847	0
tsrna-20318	GTAAGCACTCTGGACTCTGAATC	0	0.07808	0	0
tsrna-20312	GTAAATCTAAAGACAGGGGTTAGGCCT	0	0.07808	0	0
tsrna-20311	GTAAATCTAAAGACAGGGGTTAGGC	0.07141622	0	0	0

tsrna-20307	GTAAATATAGTTTAACCAAAACATCAGAT	0	0	0.139694	0
tsrna-20305	GTAAATAATAGGAGCTTAAACCCCCTTATT	0.14283243	0	0.069847	0
tsrna-20304	GTAAATAATAGGAGCTTAAACCCCCTTAT	0	0	0	0.111027
tsrna-20303	GTAAATAATAGGAGCTTAAACCCCCTTA	0.14283243	0	0	0
tsrna-20302	GTAAATAATAGGAGCTTAAACCCCCT	0	0	0	0
tsrna-20291	GTAAACCGGAGATGAAAACCTTTTTCCAAGGACACCA	3.07089731	3.279365	8.032383	0.333081
tsrna-20290	GTAAACCGGAGATGAAAACCTTTTTCCAAGGACACC	0.78557838	1.639682	0.628621	0
tsrna-20289	GTAAACCGGAGATGAAAACCTTTTTCCAAG	0.14283243	0.780801	0.069847	0
tsrna-20288	GTAAACCGGAGATGAAAACCTT	0	0.07808	0	0
tsrna-20286	GTAAACCGGAGATGAAAACC	0	0.07808	0	0
tsrna-20285	GTAAACCGGAGATGAAAAC	0.07141622	0	0	0
tsrna-20284	GTAAACCGAAGATCGCGGGTTCGAACCCCGTCCGTGCCTCC	0.99982703	3.201285	0.069847	0.333081
tsrna-20283	GTAAACCGAAGATCGCGGGT	0.14283243	1.717762	0.069847	0.222054
tsrna-20279	GTAAACCGAAGATCACGGGTTCGAACCCCGTCCGTGCCTCC	0.28566487	1.405442	0.069847	0.333081
tsrna-20278	GTAAACCGAAGATCACGGGTTC	0	0.23424	0.069847	0
tsrna-20276	GTAAACCAGGGGTCGCGAGTTCA	0.78557838	2.186243	0.279387	0.666163
tsrna-20275	GTAAACCAGGGGTCGCGAGTTC	0.8569946	2.030083	0.558774	0.555136
tsrna-20274	GTAAACCAGGGGTCGCGAGTT	0.49991351	2.030083	0.349234	0.666163
tsrna-20273	GTAAACCAGGGGTCGCGAGT	0.78557838	0.936961	0.628621	0.333081
tsrna-20272	GTAAACCAGGGGTCGCGAG	0	0.07808	0	0
tsrna-20271	GTAAACCAGGGGTCGCGA	0.14283243	0.23424	0	0
tsrna-20270	GTAAACCAGGGGTCGCG	0	0.15616	0	0
tsrna-20268	GTAAACAGGAGATCCTGGGT	0.8569946	1.405442	0.20954	0.222054
tsrna-20267	GTAAAATGGCTGAGTGAAGCATTGGACTGT	0.07141622	0.07808	0	0
tsrna-20266	GTAAAATGGCTGAGTGAAGCATTGGACTG	0	0	0.069847	0
tsrna-20265	GTAAAATGGCTGAGTGAAGCATTGGACT	0.07141622	0.15616	0	0
tsrna-20264	GTAAAATGGCTGAGTGAAGCATTGGAC	0	0	0	0
tsrna-20263	GTAAAATGGCTGAGTGAAGCATTGG	0	0	0.069847	0
tsrna-20262	GTAAAATGGCTGAGTGAAGCATTG	0	0.07808	0.069847	0
tsrna-20261	GTAAAATGGCTGAGTGAAGCATT	0.07141622	0	0.069847	0
tsrna-20260	GTAAAATGGCTGAGTGAAGCA	0.07141622	0.15616	0	0
tsrna-20259	GTAAAATGGCTGAGTGAAGC	0.14283243	0.07808	0	0
tsrna-20258	GTAAAATGGCTGAGTGAAG	0.07141622	0	0	0
tsrna-20257	GTAAAATGGCTGAGTGAA	0.07141622	0	0	0
tsrna-20256	GGTTTTTCATATCATTGGTCGTGGTTGTAGTCCGTGCGAGAAT	11.1409298	11.94626	14.87737	6.328547
tsrna-20255	GGTTTTTCATATCATTGGTCGTGGTTGTAGTCCGTGCGAGAAT	1.57115676	2.654724	2.584332	1.998488
tsrna-20254	GGTTTTTCATATCATTGGTCGTGGTTGTAGTCCGTGCGAGAAT	0.99982703	1.561602	1.047702	1.221299
tsrna-20253	GGTTTTTCATATCATTGGTCGTGGTTGTAGTCCGTGCGAGAAT	0.78557838	1.249282	0.908008	1.55438
tsrna-20252	GGTTTTTCATATCATTGGTCGTGGTTGTAGTCCGTGCGAGAA	0.57132973	1.483522	1.676323	1.110271
tsrna-20251	GGTTTTTCATATCATTGGTCGTGGTTGTAGTCCGTGCGAGA	0.49991351	1.327362	1.117549	1.110271
tsrna-20250	GGTTTTTCATATCATTGGTCGTGGTTGTAGTCCGTGCGAG	0.78557838	0.624641	0.838162	2.220543
tsrna-20249	GGTTTTTCATATCATTGGTCGTGGTTGTAGTCCGTGCGA	0.4284973	0.858881	0.977855	0.444109
tsrna-20248	GGTTTTTCATATCATTGGTCGTGGTTGTAGTCCGTGCG	0.28566487	0.390401	0.698468	0.77719
tsrna-20247	GGTTTTTCATATCATTGGTCGTGGTTGTAGTCCGTGC	0.57132973	0.23424	1.117549	0
tsrna-20246	GGTTTTTCATATCATTGGTCGTGGTTGTAGTCCGTG	0.07141622	0.468481	0.139694	0.444109
tsrna-20245	GGTTTTTCATATCATTGGTCGTGGTTGTAGTCC	0.07141622	0.23424	0.069847	0
tsrna-20244	GGTTTTTCATATCATTGGTCGTGGTTGTAGTC	0.07141622	0.07808	0	0
tsrna-20243	GGTTTTTCATATCATTGGTCGTGGTTGTAG	0.21424865	0	0.069847	0.111027
tsrna-20242	GGTTTTTCATATCATTGGTCGTGGTTGTA	0	0.07808	0.069847	0
tsrna-20241	GGTTTTTCATATCATTGGTCGTGGTTGT	0	0	0	0
tsrna-20240	GGTTTTTCATATCATTGGTCGTGGTTG	0	0.07808	0	0
tsrna-20239	GGTTTTTCATATCATTGGTCGTGGTT	0	0	0	0
tsrna-20238	GGTTTTTCATATCATTGGTCGTGGT	0	0	0	0
tsrna-20237	GGTTTTTCATATCATTGGTCGTGG	0	0	0	0
tsrna-20236	GGTTTTTCATATCATTGGTCGTG	0	0	0	0.111027
tsrna-20235	GGTTTTTCATATCATTGGTCGT	0	0.07808	0	0
tsrna-20234	GGTTTTTCATATCATTGGTCG	0	0.07808	0	0
tsrna-20228	GGTTTTGCAGTCCTTACCA	0.07141622	0	0	0
tsrna-20226	GGTTTTGCAGTCCTTAC	0	0	0.069847	0
tsrna-20224	GGTTTTCACCCAGGTGGCCCGG	0.21424865	0.390401	0.279387	0
tsrna-20223	GGTTTTCACCCAGGTGGCCCG	0.28566487	0.07808	0.139694	0.111027
tsrna-20222	GGTTTTCACCCAGGTGGCCC	0.21424865	0	0.279387	0
tsrna-20221	GGTTTTCACCCAGGCGGCCCGGGT	0.21424865	0.468481	0	0
tsrna-20220	GGTTTTCACCCAGGCGGCCCGG	0.14283243	0	0.069847	0

tsrna-20219	GGTTTTCACCCAGGCGGCCCG	0.07141622	0.15616	0	0
tsrna-20218	GGTTTTCACCCAGGCGGCCC	0.14283243	0.31232	0	0
tsrna-20217	GGTTTTCACCCAGGCGGCC	0.07141622	0	0	0
tsrna-20212	GGTTTCCGTAGTGTAGTGGTTATCACGTTCGCCT	28.4236541	11.55586	29.82459	12.76812
tsrna-20211	GGTTTCCGTAGTGTAGTGGTTATCACGTTCGCC	26.3525839	13.11746	26.40209	10.99169
tsrna-20210	GGTTTCCGTAGTGTAGTGGTTATCACGTTC	7.07020543	3.513605	4.679736	8.104981
tsrna-20209	GGTTTCCGTAGTGTAGTGGTTATC	6.92737299	2.576644	4.120962	7.438818
tsrna-20208	GGTTTCCGTAGTGTAGTGGTTAT	1.49974054	1.015041	0.419081	0.888217
tsrna-20207	GGTTTCCGTAGTGTAGTGGTT	1.14265946	1.249282	0.139694	0.77719
tsrna-20206	GGTTTCCGTAGTGTAGTGGTC	1.14265946	1.015041	0.488928	0.444109
tsrna-20205	GGTTTCCGTAGTGTAGTGGT	0.99982703	1.171202	0.488928	0.77719
tsrna-20204	GGTTTCCGTAGTGTAGTGG	1.14265946	1.015041	0.20954	0
tsrna-20203	GGTTTCCGTAGTGTAGCGGTT	0.57132973	0.780801	0.488928	0.77719
tsrna-20202	GGTTTCCGTAGTGTAG	0.49991351	1.171202	0.20954	0
tsrna-20200	GGTTTCCCCGCGCAGGTTC	0	0	0	0
tsrna-20198	GGTTTCCCCGCGCAGGT	0	0.07808	0	0
tsrna-20196	GGTTTCCCCGCACAGGTT	0	0.07808	0	0
tsrna-20194	GGTTTAGTGGTAGAATTCTCGCCT	0.49991351	0.390401	0.279387	0
tsrna-20193	GGTTTAGTGGTAGAATTCTCGCC	0	0.390401	0	0.222054
tsrna-20192	GGTTTAGTGGTAGAATTCTCGC	0	0	0	0
tsrna-20191	GGTTTAGTGGTAGAATTCTCG	0.07141622	0.07808	0	0
tsrna-20187	GGTTTAAGTCCCATTGGTCTAGCCA	0	0.15616	0	0
tsrna-20185	GGTTTAAGTCCCATTGGTCTAGC	0	0	0.20954	0
tsrna-20183	GGTTTAAGTCCCATTGGTCTA	0	0	0	0
tsrna-20182	GGTTTAAGTCCCATTGGTCT	0	0	0	0
tsrna-20181	GGTTTAAGTCCCATTGGTC	0.07141622	0	0	0
tsrna-20180	GGTTTAAGTCCCATTGGT	0	0.07808	0	0
tsrna-20179	GGTTTAAGTCCCATTGG	0	0	0	0
tsrna-20174	GGTTGTGGGTTCGAATCCCACCAGAGTCGC	0	0	0	0
tsrna-20172	GGTTGTAGTCCGTGCGAGAATACCA	0.14283243	0.15616	0.069847	0.111027
tsrna-20171	GGTTGTAGTCCGTGCGAGAATACC	0.14283243	0.31232	0	0
tsrna-20170	GGTTGTAGTCCGTGCGAGAATAC	0	0	0	0
tsrna-20169	GGTTGTAGTCCGTGCGAGAATA	0.07141622	0.07808	0.069847	0
tsrna-20168	GGTTGTAGTCCGTGCGAGAAT	0.07141622	0	0	0
tsrna-20167	GGTTGTAGTCCGTGCGAGAA	0.07141622	0	0	0
tsrna-20166	GGTTGTAGTCCGTGCGAGA	0	0.07808	0.069847	0
tsrna-20165	GGTTGTAGTCCGTGCGAG	0	0	0	0
tsrna-20160	GGTTGGTGGTTCGATCCCACCCAGGGACGC	0	0.23424	0	0
tsrna-20159	GGTTGGTGGTTCGAGCCCACCCAGGGACGC	0.07141622	0.15616	0	0
tsrna-20158	GGTTGGTGGTTCGAGCCCACCCAGGGACG	0	0.15616	0	0
tsrna-20157	GGTTGCGTGTTCAAGTCACGTCGGGGTCAC	0	0	0	0
tsrna-20153	GGTTGCGTGTTCAAATCACGTCGGGGTCAC	0	0	0	0
tsrna-20151	GGTTCTTGTAGTTGAAAT	0	0	0	0
tsrna-20150	GGTTCGATTCTCATAGTCCTAGCCA	0.07141622	0.390401	0.069847	0
tsrna-20149	GGTTCGATTCTCATAGTCCTAGCC	0	0.23424	0	0.111027
tsrna-20148	GGTTCGATTCTCATAGTCCTAGC	0	0.07808	0	0.111027
tsrna-20147	GGTTCGATTCTCATAGTCCTAG	0	0.07808	0	0
tsrna-20146	GGTTCGATTCTCATAGTCCTA	0.07141622	0	0	0.111027
tsrna-20145	GGTTCGATTCTCATAGTCCT	0.07141622	0.07808	0	0.111027
tsrna-20144	GGTTCGATTCTCATAGTCC	0	0.07808	0	0
tsrna-20140	GGTTCGATTCCTTCCTTTTTTGCCA	0	0.07808	0.069847	0
tsrna-20139	GGTTCGATTCCTTCCTTTTTTGCC	0	0	0	0
tsrna-20138	GGTTCGATTCCTTCCTTTTTTGC	0	0.07808	0	0.222054
tsrna-20137	GGTTCGATTCCTTCCTTTTTTG	0	0.15616	0.069847	0
tsrna-20136	GGTTCGATTCCTTCCTTTTTT	0	0.07808	0	0
tsrna-20135	GGTTCGATTCCTTCCTTTTT	0	0	0	0
tsrna-20134	GGTTCGATTCCTTCCTTTT	0	0	0	0
tsrna-20132	GGTTCGATTCCTTCCTT	0	0.07808	0	0
tsrna-20130	GGTTCGATTCCGGCTCGAAGGACCA	5.14196758	12.02434	1.74617	2.775678
tsrna-20121	GGTTCGATTCCCGGTCAGGGAACCA	0.71416216	22.33091	0.069847	0.333081
tsrna-20120	GGTTCGATTCCCGGTCAGGGAACC	0	0.858881	0.069847	0
tsrna-20119	GGTTCGATTCCCGGTCAGGGAAC	0	0.23424	0	0
tsrna-20118	GGTTCGATTCCCGGTCAGGGAA	0	0.23424	0	0
tsrna-20117	GGTTCGATTCCCGGTCAGGGA	0	0.23424	0	0

tsrna-20116	GGTTCGATTCCCGGTCAGGG	0	0.07808	0	0
tsrna-20111	GGTTCGATTCCCGGGCGGCGCACCA	0.64274595	4.762887	0.349234	0.111027
tsrna-20110	GGTTCGATTCCCGGGCGGCGCACC	0.35708108	0.624641	0	0
tsrna-20101	GGTTCGATTCCCGGCCCATGCACCA	1.2854919	2.264323	0.768315	0.999244
tsrna-20100	GGTTCGATTCCCGGCCCATGCACC	0.28566487	0.15616	0.069847	0.111027
tsrna-20099	GGTTCGATTCCCGGCCCATGCAC	0	0.07808	0	0
tsrna-20096	GGTTCGATTCCCGGCCAGGGAACCA	0	0.468481	0	0
tsrna-20095	GGTTCGATTCCCGGCCAGGGAACC	0	0.07808	0	0
tsrna-20090	GGTTCGATTCCCGGCCAATGCACCA	2.92806487	9.838094	0.558774	1.443353
tsrna-20089	GGTTCGATTCCCGGCCAATGCACC	0.78557838	0.624641	0	0.111027
tsrna-20086	GGTTCGATTCCCGGCCAATGC	0	0.07808	0	0
tsrna-20085	GGTTCGATTCCCGGCCAACGCACCA	3.35656217	8.354572	1.327089	1.221299
tsrna-20084	GGTTCGATTCCCGGCCAACGCACC	0.57132973	0.858881	0.20954	0.222054
tsrna-20083	GGTTCGATTCCCGGCCAACGCAC	0	0.15616	0	0
tsrna-20082	GGTTCGATTCCCGGCCAACGCA	0	0.07808	0	0
tsrna-20081	GGTTCGATTCCCGGCCAACGC	0	0	0	0
tsrna-20078	GGTTCGATTCCCCGACGGGGAGCCA	0.14283243	1.639682	0.139694	0
tsrna-20077	GGTTCGATTCCCCGACGGGGAGCC	0	0.07808	0	0
tsrna-20075	GGTTCGATTCCCCGACGGGGAG	0	0	0	0
tsrna-20060	GGTTCGATCCCGGGTTTCGGCACCA	2.28531893	8.979213	0.488928	1.332326
tsrna-20059	GGTTCGATCCCGGGTTTCGGCAC	0	0	0.069847	0
tsrna-20056	GGTTCGATCCCGGGTTTCGG	0	0	0	0
tsrna-20053	GGTTCGATCCCGGGCGGA	0	0.07808	0	0
tsrna-20051	GGTTCGATCCCCGTACTGGCCACCA	0.4284973	0.936961	0.20954	0.111027
tsrna-20050	GGTTCGATCCCCGTACGGGCCACCA	0.35708108	4.762887	0.20954	0.444109
tsrna-20044	GGTTCGATCCCCGGCATCTCCACCA	17.5683892	30.9978	8.31177	9.770388
tsrna-20043	GGTTCGATCCCCGGCATCTCCACC	0.8569946	1.483522	0.279387	0.111027
tsrna-20042	GGTTCGATCCCCGGCATCTCCAC	0	0.07808	0	0.111027
tsrna-20041	GGTTCGATCCCCGGCATCTCCA	0.07141622	0	0	0
tsrna-20040	GGTTCGATCCCCGGCATCTCC	0	0.07808	0	0
tsrna-20039	GGTTCGATCCCCGGCATCTC	0	0	0	0
tsrna-20035	GGTTCGATCCCCGGCACCTCCACCA	12.854919	20.76931	7.613302	5.329303
tsrna-20034	GGTTCGATCCCCGGCACCTCCACC	0.4284973	1.639682	0.139694	0.222054
tsrna-20033	GGTTCGATCCCCGGCACCTCCAC	0.07141622	0	0	0
tsrna-20031	GGTTCGATCCCCGGCACCTCC	0	0	0.069847	0
tsrna-20029	GGTTCGATCCCCGACACCTCCACCA	1.42832433	1.952003	0	0.555136
tsrna-20028	GGTTCGATCCCCAGTACCTCCACCA	1.57115676	4.919047	0.908008	0.888217
tsrna-20027	GGTTCGATCCCCAGTACCTCCACC	0.57132973	0.390401	0.069847	0
tsrna-20019	GGTTCGATCCCCAGCATCTCCACCA	0.78557838	1.015041	0.488928	0.111027
tsrna-20018	GGTTCGATCCCCAGCATCTCCACC	0	0.07808	0	0
tsrna-20014	GGTTCGATCCCCAGCATCTC	0	0.07808	0	0
tsrna-20009	GGTTCGAGTCCTGCCGCGGTCGCCA	0.14283243	0.390401	0.069847	0
tsrna-20007	GGTTCGAGTCCTGCCGCGGTCGC	0	0	0	0
tsrna-20002	GGTTCGAGTCCCTTCGTGGTCGCCA	0.07141622	0.15616	0	0
tsrna-20001	GGTTCGAGTCCCTTCGTGGTCGCC	0	0.07808	0	0
tsrna-19996	GGTTCGAGTCCCGGCGGAGTCGCCA	0	0.390401	0	0
tsrna-19988	GGTTCGAGTCCCATCTGGGTCGCCA	0.07141622	0.31232	0	0.111027
tsrna-19987	GGTTCGAGTCCCATCTGGGTCGCC	0	0.15616	0	0.111027
tsrna-19984	GGTTCGAGTCCCATCTGGGGTGCCA	0	0.702721	0	0
tsrna-19976	GGTTCGAGTCCCACCAGAGTCGCCA	0.14283243	0.15616	0	0
tsrna-19975	GGTTCGAGTCCCACCAGAGTCGCC	0	0.07808	0	0
tsrna-19973	GGTTCGAGTCCCACCAGAGTCG	0	0.07808	0	0
tsrna-19969	GGTTCGAGCCCCAGTGGAACCACCA	3.14231352	2.264323	1.74617	1.998488
tsrna-19968	GGTTCGAGCCCCAGTGGAACCAC	0.14283243	0	0.069847	0
tsrna-19967	GGTTCGAGCCCCAGTGGAACC	0.07141622	0	0	0
tsrna-19961	GGTTCGAGCCCCACGTTGGGCGCCA	0.99982703	16.70914	0.279387	0.333081
tsrna-19960	GGTTCGAGCCCCACGTTGGGCGCC	0.14283243	0.546561	0	0
tsrna-19952	GGTTCGAGCCCACCCAGGGACGCCA	0.49991351	7.573771	0	0.222054
tsrna-19951	GGTTCGAGCCCACCCAGGGACGCC	0	0.546561	0	0
tsrna-19950	GGTTCGAGCCCACCCAGGGACGC	0	0.07808	0	0
tsrna-19943	GGTTCGACTCCTGGCTGGCTCGCCA	3.07089731	8.666892	0.698468	0.555136
tsrna-19942	GGTTCGACTCCTGGCTGGCTCGCC	0.07141622	0.468481	0	0.111027
tsrna-19941	GGTTCGACTCCTGGCTGGCTCGC	0	0	0	0
tsrna-19939	GGTTCGACTCCTGGCTGGCTC	0	0	0	0

tsrna-19935	GGTTCGACTCCCGGTGTGGGAACCA	5.14196758	21.00355	1.606477	1.55438
tsrna-19934	GGTTCGACTCCCGGTGTGGGAACC	0.07141622	1.717762	0	0.222054
tsrna-19933	GGTTCGACTCCCGGTGTGGGAAC	0	0.15616	0	0
tsrna-19932	GGTTCGACTCCCGGTGTGGGAA	0	0.23424	0	0
tsrna-19930	GGTTCGACTCCCGGTGTGGG	0	0.07808	0	0
tsrna-19929	GGTTCGACTCCCGGTGTGG	0	0.07808	0	0
tsrna-19925	GGTTCGACTCCCGGTATGGGAACCA	0.78557838	4.294406	0.069847	0.222054
tsrna-19924	GGTTCGACTCCCGGTATGGGAACC	0.28566487	0.624641	0.069847	0
tsrna-19923	GGTTCGACTCCCGGTATGGGAAC	0.07141622	0.15616	0	0
tsrna-19922	GGTTCGACTCCCGGTATGGGAA	0.07141622	0.15616	0	0
tsrna-19920	GGTTCGACTCCCGGTATGGG	0	0.07808	0	0
tsrna-19915	GGTTCGACTCCCAGCGGGGCCTCC	0	0	0.069847	0
tsrna-19914	GGTTCGACTCCCAGCGGGGC	0	0	0	0
tsrna-19913	GGTTCGACCCCCGGCTCCTCCACCA	0.8569946	1.873923	0.628621	0.555136
tsrna-19910	GGTTCGAATCCTGTTCGTGACGCC	0.14283243	0	0	0.222054
tsrna-19909	GGTTCGAATCCTGTTCGTGACGC	0	0	0	0
tsrna-19908	GGTTCGAATCCTGTCG	0	0.07808	0	0
tsrna-19907	GGTTCGAATCCTGCTCACAGCGCCA	0.92841081	2.576644	0.279387	0
tsrna-19906	GGTTCGAATCCTGCTCACAGCGCC	0.07141622	0.15616	0.069847	0
tsrna-19904	GGTTCGAATCCTGCCGACTACGCCA	6.42745948	19.05155	1.816017	2.886706
tsrna-19903	GGTTCGAATCCTGCCGACTACGCC	0.35708108	1.093122	0.20954	0.222054
tsrna-19902	GGTTCGAATCCTGCCGACTACGC	0	0	0	0
tsrna-19899	GGTTCGAATCCTGCCGAC	0	0	0	0
tsrna-19889	GGTTCGAATCCGAGTCACGGCACCA	0.07141622	0.07808	0	0
tsrna-19883	GGTTCGAATCCCTTCGTGGTTGCCA	0	0.15616	0	0
tsrna-19880	GGTTCGAATCCCATCCTCGTCGCCA	0.28566487	0.936961	0.279387	0.333081
tsrna-19879	GGTTCGAATCCCATCCTCGTCGCC	0.07141622	0.15616	0	0
tsrna-19872	GGTTCGAATCCCAGCGGTGCCTCCA	1.71398919	4.919047	0.768315	0.666163
tsrna-19871	GGTTCGAATCCCAGCGGTGCCTCC	0.07141622	0.15616	0.069847	0
tsrna-19865	GGTTCGAATCCCAGCGGGGCCTCCA	0.07141622	0.702721	0	0
tsrna-19864	GGTTCGAATCCCAGCGGGGCCTCC	0.07141622	0.07808	0	0
tsrna-19857	GGTTCGAATCCCACTTCTGACACCA	0.71416216	2.108163	0.558774	0.888217
tsrna-19856	GGTTCGAATCCCACTTCTGACACC	0.14283243	0.31232	0.069847	0
tsrna-19855	GGTTCGAATCCCACTTCTGACAC	0.07141622	0	0.069847	0
tsrna-19854	GGTTCGAATCCCACTTCTGACA	0	0	0	0
tsrna-19851	GGTTCGAATCCCACTCCTGACACCA	7.49870272	9.213453	3.981268	3.330814
tsrna-19850	GGTTCGAATCCCACTCCTGACACC	0.35708108	1.093122	0.139694	0
tsrna-19849	GGTTCGAATCCCACTCCTGACAC	0.07141622	0	0	0
tsrna-19848	GGTTCGAATCCCACTCCTGACA	0	0	0.069847	0
tsrna-19844	GGTTCGAATCCCACCTTCGTCGCCA	0	0.468481	0.069847	0.111027
tsrna-19840	GGTTCGAATCCCACCGCTGCCACCA	1.35690811	2.108163	0.139694	0.222054
tsrna-19839	GGTTCGAATCCCACCGCTGCCACC	0.07141622	0.23424	0.069847	0
tsrna-19838	GGTTCGAATCCCACCGCTGCCAC	0	0.07808	0	0
tsrna-19832	GGTTCGAATCCCACCCTCGTCGCCA	0.21424865	1.327362	0.279387	0.555136
tsrna-19831	GGTTCGAATCCCACCAGAGTCGCCA	0.14283243	0.23424	0	0
tsrna-19830	GGTTCGAATCCCACCAGAGTCGCC	0.07141622	0.15616	0	0
tsrna-19824	GGTTCGAACCCTGCTCGCTGCGCCA	0.78557838	2.030083	0.069847	0.111027
tsrna-19823	GGTTCGAACCCTGCTCGCTGCGCC	0	0.15616	0	0
tsrna-19817	GGTTCGAACCCCGTCCGTGCCTCCA	0.21424865	0.858881	0.139694	0
tsrna-19813	GGTTCGAACCCCGTCCGTGCC	0	0	0	0
tsrna-19810	GGTTCGAACCCCACTCCTGGTACCA	0.07141622	0	0	0
tsrna-19805	GGTTCGAAACTGGGCGGAAACACC	0	0.780801	0.139694	0.111027
tsrna-19804	GGTTCGAAACCGGGCGGAAACACCA	18.6396325	56.29576	8.31177	4.885194
tsrna-19803	GGTTCGAAACCGGGCGGAAACACC	5.57046488	14.44482	2.025557	0.666163
tsrna-19802	GGTTCGAAACCGGGCGGAAACAC	0.07141622	0.23424	0	0
tsrna-19801	GGTTCGAAACCGGGCGGAAACA	0	0.15616	0	0
tsrna-19797	GGTTCGAAACCGGGCGGA	0	0.07808	0	0
tsrna-19794	GGTTCGAAACCGGGCAGAAGCACCA	2.35673514	10.30657	0.628621	0.555136
tsrna-19793	GGTTCGAAACCGGGCAGAAGCA	0	0.07808	0	0
tsrna-19789	GGTTCCGGGTTCGAGTCCCGGCAGAGATGC	0	0	0.069847	0
tsrna-19786	GGTTCCATGGTGTAATGGTTAGCACTCTGGACTCTGAATCCA	0.71416216	1.171202	0.628621	0.888217
tsrna-19785	GGTTCCATGGTGTAATGGTTAGCACTCTGGACTCTGAATCCA	1.21407568	1.249282	0.349234	0.555136
tsrna-19784	GGTTCCATGGTGTAATGGTTAGCACTCTGGACTCTGAATCCA	0.92841081	1.171202	0.419081	1.110271
tsrna-19783	GGTTCCATGGTGTAATGGTTAGCACTCTGGACTCTGAATCCA	1.07124325	1.249282	0.558774	0.333081

tsrna-19782	GGTTCCATGGTGTAATGGTTAGCACTCTGGACTCTGAATCCA	0.64274595	1.249282	0.069847	1.221299
tsrna-19781	GGTTCCATGGTGTAATGGTTAGCACTCTGGACTCTGAATCC	0.4284973	0.936961	0.069847	0.77719
tsrna-19780	GGTTCCATGGTGTAATGGTTAGCACTCTGGACTCTGAATC	0.21424865	0.23424	0.069847	0.111027
tsrna-19779	GGTTCCATGGTGTAATGGTTAGCACTCTGGACTCTG	0.14283243	0.23424	0.139694	0.111027
tsrna-19778	GGTTCCATGGTGTAATGGTTAGCACTCTGGACTCT	0	0.31232	0	0
tsrna-19777	GGTTCCATGGTGTAATGGTTAGCACTCTGGACTC	0	0.31232	0	0.444109
tsrna-19776	GGTTCCATGGTGTAATGGTTAGCACTCTGGACT	0.14283243	0.624641	0.069847	0
tsrna-19775	GGTTCCATGGTGTAATGGTTAGCACTCTGGAC	0.14283243	0.546561	0	0
tsrna-19774	GGTTCCATGGTGTAATGGTTAGCACTCTGGA	0	0.546561	0.139694	0.111027
tsrna-19773	GGTTCCATGGTGTAATGGTTAGCACTCTGG	0.14283243	0.31232	0	0
tsrna-19772	GGTTCCATGGTGTAATGGTTAGCACTCTG	0	0.31232	0	0
tsrna-19771	GGTTCCATGGTGTAATGGTTAGCACTCT	0	0.07808	0.069847	0.111027
tsrna-19770	GGTTCCATGGTGTAATGGTTAGCACTC	0	0.31232	0	0.111027
tsrna-19769	GGTTCCATGGTGTAATGGTTAGCACT	0	0.15616	0	0
tsrna-19768	GGTTCCATGGTGTAATGGTTAGCAC	0	0.07808	0	0
tsrna-19767	GGTTCCATGGTGTAATGGTTAGCA	0	0.390401	0	0
tsrna-19766	GGTTCCATGGTGTAATGGTTAGC	0	0.23424	0	0
tsrna-19765	GGTTCCATGGTGTAATGGTTAG	0	0.31232	0.139694	0.111027
tsrna-19764	GGTTCCATGGTGTAATGGTTA	0	0.15616	0	0
tsrna-19763	GGTTCCATGGTGTAATGGTT	0	0.390401	0	0
tsrna-19762	GGTTCCATGGTGTAATGGTGAGCACTCTGGACTCT	0	0.15616	0	0
tsrna-19761	GGTTCCATGGTGTAATGGTGAGCACTCTGGACTC	0.07141622	0.31232	0	0
tsrna-19760	GGTTCCATGGTGTAATGGTGAGCACTCTGGACT	0.07141622	0.390401	0	0.111027
tsrna-19759	GGTTCCATGGTGTAATGGTGAGCACTCTGGAC	0	0.15616	0	0
tsrna-19758	GGTTCCATGGTGTAATGGTGAGCACTCTGGA	0.07141622	0.468481	0	0
tsrna-19757	GGTTCCATGGTGTAATGGTGAGCACTCTGG	0.14283243	0.31232	0	0
tsrna-19756	GGTTCCATGGTGTAATGGTGAGCACTCTG	0	0.624641	0	0
tsrna-19755	GGTTCCATGGTGTAATGGTGAGCACTCT	0.07141622	0.31232	0	0
tsrna-19754	GGTTCCATGGTGTAATGGTGAGCACTC	0	0.468481	0	0
tsrna-19753	GGTTCCATGGTGTAATGGTGAGCACT	0.07141622	0	0	0
tsrna-19752	GGTTCCATGGTGTAATGGTGAGCA	0	0.07808	0	0
tsrna-19751	GGTTCCATGGTGTAATGGTGAGC	0.07141622	0.23424	0	0
tsrna-19750	GGTTCCATGGTGTAATGGTGAG	0.14283243	0.15616	0	0
tsrna-19749	GGTTCCATGGTGTAATGGTGA	0.07141622	0.31232	0.069847	0.111027
tsrna-19748	GGTTCCATGGTGTAATGGTG	0	0.23424	0	0
tsrna-19747	GGTTCCATGGTGTAATGGTAAGCACTCTGG	0	0.468481	0	0.111027
tsrna-19746	GGTTCCATGGTGTAATGGTAAGCACTCTG	0.07141622	0.23424	0	0.111027
tsrna-19745	GGTTCCATGGTGTAATGGTAAGC	0	0.31232	0	0
tsrna-19744	GGTTCCATGGTGTAATGGTA	0	0.23424	0	0.111027
tsrna-19743	GGTTCCATGGTGTAATGGT	0	0.546561	0	0
tsrna-19742	GGTTCCATGGTGTAATGG	0.07141622	0.15616	0	0
tsrna-19741	GGTTCCATGGTGTAATG	0.07141622	0.31232	0.069847	0.111027
tsrna-19740	GGTTCCATGGTGTAAT	0	0.624641	0	0
tsrna-19739	GGTTCCATAGTGTAGTGGTTATCACGTCTGCTTTACACGCAG.	3.64222704	1.717762	5.098817	0.555136
tsrna-19738	GGTTCCATAGTGTAGTGGTTATCACGTCTGCTTTAC	3.85647569	1.873923	3.701881	0.333081
tsrna-19737	GGTTCCATAGTGTAGTGGTTATCACGTCTGCTTTA	3.28514596	1.483522	2.933566	0.444109
tsrna-19736	GGTTCCATAGTGTAGTGGTTATCACGTCTGCTTT	3.07089731	1.249282	2.863719	0.222054
tsrna-19735	GGTTCCATAGTGTAGTGGTTATCACGTCTGCTT	1.2854919	1.015041	1.047702	0.444109
tsrna-19734	GGTTCCATAGTGTAGTGGTTATCACGTCTGCT	1.21407568	1.015041	1.327089	0.222054
tsrna-19733	GGTTCCATAGTGTAGTGGTTATCACGTCTGC	1.35690811	0.624641	1.187396	0
tsrna-19732	GGTTCCATAGTGTAGTGGTTATCACGTCTG	0.14283243	0.31232	0.069847	0.111027
tsrna-19731	GGTTCCATAGTGTAGTGGTTATCACGTCT	0.14283243	0.31232	0.139694	0.222054
tsrna-19730	GGTTCCATAGTGTAGTGGTTATCACGTC	0.28566487	0.15616	0.20954	0
tsrna-19729	GGTTCCATAGTGTAGTGGTTATCACGT	0.14283243	0.07808	0.069847	0.222054
tsrna-19728	GGTTCCATAGTGTAGTGGTTATCACG	0.21424865	0.07808	0.20954	0.111027
tsrna-19727	GGTTCCATAGTGTAGTGGTTATCACATCTGCTTT	0.64274595	0.23424	0.838162	0.444109
tsrna-19726	GGTTCCATAGTGTAGTGGTTATCACATCTGCTT	0.49991351	0.390401	0.069847	0.111027
tsrna-19725	GGTTCCATAGTGTAGTGGTTATCACATCTGCT	0.35708108	0.468481	0.139694	0.555136
tsrna-19724	GGTTCCATAGTGTAGTGGTTATCACATCTGC	0.07141622	0	0.279387	0.222054
tsrna-19723	GGTTCCATAGTGTAGTGGTTATCACATCTG	0.28566487	0.15616	0	0.222054
tsrna-19722	GGTTCCATAGTGTAGTGGTTATCACATCT	0.28566487	0.15616	0.069847	0.222054
tsrna-19721	GGTTCCATAGTGTAGTGGTTATCACATC	0.21424865	0.07808	0.20954	0.111027
tsrna-19720	GGTTCCATAGTGTAGTGGTTATCACAT	0.28566487	0.15616	0.279387	0
tsrna-19719	GGTTCCATAGTGTAGTGGTTATCACA	0.21424865	0.23424	0.139694	0.222054

tsrna-19718	GGTTCCATAGTGTAGTGGTTATCAC	0.21424865	0.31232	0.069847	0.111027
tsrna-19717	GGTTCCATAGTGTAGTGGTTATCA	0.21424865	0.15616	0.20954	0.111027
tsrna-19716	GGTTCCATAGTGTAGTGGTTATC	0.28566487	0.07808	0.20954	0.222054
tsrna-19715	GGTTCCATAGTGTAGTGGTTAT	0.21424865	0.624641	0.20954	0
tsrna-19714	GGTTCCATAGTGTAGTGGTTA	0.14283243	0.390401	0.139694	0.222054
tsrna-19713	GGTTCCATAGTGTAGTGGTT	0.14283243	0.390401	0.349234	0.111027
tsrna-19712	GGTTCCATAGTGTAGTGGT	0.21424865	0.15616	0.069847	0
tsrna-19711	GGTTCCATAGTGTAGTGG	0.35708108	0.390401	0.069847	0
tsrna-19710	GGTTCCATAGTGTAGTG	0.14283243	0.15616	0.139694	0.111027
tsrna-19709	GGTTCCATAGTGTAGT	0	0.15616	0	0.111027
tsrna-19708	GGTTCCATAGTGTAGCGGTTATCACGTCTGCTTTACACGCAG	1.42832433	0.390401	1.117549	0.555136
tsrna-19707	GGTTCCATAGTGTAGCGGTTATCACGTCTGCTTTAC	1.57115676	0.702721	1.74617	0.333081
tsrna-19706	GGTTCCATAGTGTAGCGGTTATCACGTCTGCTTT	1.42832433	0.468481	1.466783	0.111027
tsrna-19705	GGTTCCATAGTGTAGCGGTTATCACGTCTGCTT	0.49991351	0.546561	0.558774	0.111027
tsrna-19704	GGTTCCATAGTGTAGCGGTTATCACGTCTGCT	0.57132973	0.390401	0.768315	0.222054
tsrna-19703	GGTTCCATAGTGTAGCGGTTATCACGTCTGC	0.64274595	0.546561	0.139694	0
tsrna-19702	GGTTCCATAGTGTAGCGGTTATCACGTCTG	0.21424865	0.23424	0.20954	0.222054
tsrna-19701	GGTTCCATAGTGTAGCGGTTATCACGTCT	0	0.15616	0.20954	0.111027
tsrna-19700	GGTTCCATAGTGTAGCGGTTATCACGTC	0.28566487	0.15616	0.139694	0
tsrna-19699	GGTTCCATAGTGTAGCGGTTATCACGT	0.21424865	0.390401	0.139694	0
tsrna-19698	GGTTCCATAGTGTAGCGGTTATCACG	0.28566487	0.07808	0.20954	0
tsrna-19697	GGTTCCATAGTGTAGCGGTTATCAC	0.14283243	0.468481	0.279387	0
tsrna-19696	GGTTCCATAGTGTAGCGGTTATCA	0.21424865	0.468481	0.488928	0
tsrna-19695	GGTTCCATAGTGTAGCGGTTATC	0.07141622	0.23424	0	0
tsrna-19694	GGTTCCATAGTGTAGCGGTTAT	0.28566487	0.31232	0	0.111027
tsrna-19693	GGTTCCATAGTGTAGCGGTTA	0.14283243	0.23424	0.139694	0
tsrna-19692	GGTTCCATAGTGTAGCGGTT	0.14283243	0.23424	0.069847	0
tsrna-19691	GGTTCCATAGTGTAGCGGT	0.28566487	0.546561	0	0
tsrna-19690	GGTTCCATAGTGTAGCGG	0.35708108	0.15616	0	0
tsrna-19689	GGTTCCATAGTGTAGCG	0.07141622	0.390401	0.139694	0.222054
tsrna-19688	GGTTCCATAGTGTAGC	0.07141622	0.31232	0.069847	0
tsrna-19685	GGTTCAGTGGTAGAATTCTTGCCTGC	0	0	0	0.111027
tsrna-19684	GGTTCAGTGGTAGAATTCTTGCCT	0	0	0	0
tsrna-19683	GGTTCAGTGGTAGAATTCTTGCC	0	0.07808	0	0
tsrna-19682	GGTTCAGTGGTAGAATTCTTGC	0.07141622	0	0	0
tsrna-19680	GGTTCAGTGGTAGAATTCTCGCCTT	0.28566487	0.31232	0.139694	0.222054
tsrna-19679	GGTTCAGTGGTAGAATTCTCGCCTGCCAT	2.21390271	5.856008	1.606477	0.888217
tsrna-19678	GGTTCAGTGGTAGAATTCTCGCCTGCCACG	3.85647569	7.18337	1.816017	1.665407
tsrna-19677	GGTTCAGTGGTAGAATTCTCGCCTGCCAC	1.99965406	3.747845	0.628621	1.332326
tsrna-19676	GGTTCAGTGGTAGAATTCTCGCCTGCCA	0.99982703	2.810884	0.558774	0.333081
tsrna-19675	GGTTCAGTGGTAGAATTCTCGCCTGCC	0.92841081	3.201285	0.698468	0.77719
tsrna-19674	GGTTCAGTGGTAGAATTCTCGCCTGC	0.71416216	1.015041	0.488928	0.333081
tsrna-19673	GGTTCAGTGGTAGAATTCTCGCCTG	0.57132973	0.702721	0.139694	0.222054
tsrna-19672	GGTTCAGTGGTAGAATTCTCGCCTCCC	0.8569946	1.717762	0.139694	0.888217
tsrna-19671	GGTTCAGTGGTAGAATTCTCGCCTCC	0.21424865	1.327362	0.20954	0.999244
tsrna-19670	GGTTCAGTGGTAGAATTCTCGCCTC	0.35708108	0.702721	0.069847	0.333081
tsrna-19669	GGTTCAGTGGTAGAATTCTCGCCT	0.21424865	0.546561	0.139694	0.111027
tsrna-19668	GGTTCAGTGGTAGAATTCTCGCC	0.07141622	0.15616	0	0.111027
tsrna-19667	GGTTCAGTGGTAGAATTCTCGC	0	0.07808	0	0.111027
tsrna-19666	GGTTCAGTGGTAGAATTCTCG	0	0	0	0
tsrna-19665	GGTTCAGTGGTAGAATTCTC	0	0.07808	0	0
tsrna-19663	GGTTCAGTGGTAGAATTC	0	0	0	0.111027
tsrna-19660	GGTTCAATTCCTCTTCTTAACACCA	0.28566487	0	0	0.111027
tsrna-19659	GGTTCAATTCCTCTTCTTAACACC	0	0	0	0.111027
tsrna-19657	GGTTCAATTCCTCTTCTTAAC	0.07141622	0	0	0
tsrna-19653	GGTTCAATTCCCGGTCAGGGAACCA	0.07141622	1.249282	0.139694	0
tsrna-19652	GGTTCAATTCCCCGACGGGGAGCCA	0	0.31232	0	0
tsrna-19642	GGTTCAATGGTAGAATTCTCGCCT	0.14283243	0.07808	0	0
tsrna-19641	GGTTCAATGGTAGAATTCTCGC	0.07141622	0.07808	0	0
tsrna-19640	GGTTCAATGGTAGAATTCTCG	0	0	0	0.111027
tsrna-19639	GGTTCAATCCCCGGCATCTCCACCA	8.64136219	13.42978	4.889276	4.66314
tsrna-19638	GGTTCAATCCCCGGCACCTCCACCA	13.9261622	24.20483	8.730851	7.549845
tsrna-19637	GGTTCAATCCCCGGCACCTCCACC	0.8569946	1.483522	0.419081	0.222054
tsrna-19636	GGTTCAATCCCCGGCACCTCCAC	0	0	0	0

tsrna-19632	GGTTCAATCCCCAGCACCTCCACCA	0.4284973	0.936961	0.279387	0.222054
tsrna-19631	GGTTCAATCCCCAGCACCTCCACC	0	0.15616	0	0
tsrna-19629	GGTTCAAGTCCCTGTTCGGGCGCCA	0.57132973	1.015041	0.20954	0.333081
tsrna-19622	GGTTCAAGTCCCTGTTCG	0	0	0	0
tsrna-19618	TCACGTCTGCTTTACACGC	0.07141622	0	0	0
tsrna-19617	TCACGTCTGCTTTACACG	0	0.07808	0	0
tsrna-19616	TCACGTCGGGGTCACCA	0.21424865	0.07808	0.139694	0.111027
tsrna-19612	TCACGCGGGAGACCGGGGTTCGATTCCCCGACGGGGAGCC/	0.14283243	1.405442	0	0.111027
tsrna-19611	TCACGCGGGAGACCGGGGTTCGATTCCCCGACGGGGAGCC	0	0.390401	0.069847	0
tsrna-19610	TCACGCGGGAGACCGGGGTTCGATTCCCCGACGGGGAGC	0	0.07808	0	0
tsrna-19609	TCACGCGGGAGACCGGGGTTCGATTCCCCGACGGGGAG	0	0.07808	0.069847	0
tsrna-19608	TCACGCGGGAGACCGGGGTTCGATTCCCCGACGGGGA	0	0.07808	0	0
tsrna-19607	TCACGCGGGAGACCGGGGTTCGATTCCCCGACGGGG	0	0	0	0
tsrna-19606	TCACGCGGGAGACCGGGGTTCGATTCCCCGACGGG	0	0	0	0
tsrna-19603	TCACGCGGGAGACCGGGGTTCGA	0	0.07808	0	0
tsrna-19602	TCACGCGGGAGACCGGGGTTCAATTCCCCGACGGGGAGCC/	0	0.468481	0	0
tsrna-19599	TCACGCGGGAGACCGGGGTTCAATTCCCCGACGGGG	0.07141622	0	0	0
tsrna-19598	TCACGCGGGAGACCGGGGTTCAATTCCCCG	0	0.07808	0	0
tsrna-19596	TCACCGCCGCGGCCCGGGTTCGATTCCCGGTCAGGGAACCA	3.21372974	23.42403	1.327089	1.332326
tsrna-19595	TCACCGCCGCGGCCCGGGTTCGATTCCCGGTCAGGGAACC	0.35708108	0.858881	0.768315	0
tsrna-19594	TCACCGCCGCGGCCCGGGTTCGATTCCCGGTCAGGGAAC	0.07141622	0.31232	0.139694	0.111027
tsrna-19593	TCACCGCCGCGGCCCGGGTTCGATTCCCGGTCAGGGAA	0	0.07808	0	0.111027
tsrna-19592	TCACCGCCGCGGCCCGGGTTCGATTCCCGGTCAGGGA	0	0.23424	0.139694	0
tsrna-19591	TCACCGCCGCGGCCCGGGTTCGATTCCCGGTCAGGG	0	0.07808	0	0
tsrna-19590	TCACCGCCGCGGCCCGGGTTCGATTCCCGG	0	0.07808	0	0
tsrna-19588	TCACCGCCGCGGCCCGGGTTCG	0	0.07808	0	0
tsrna-19587	TCACCGCCGCGGCCCGGGTTC	0	0.07808	0	0
tsrna-19581	TCACCCCATAAACACCA	0	0.07808	0	0
tsrna-19577	TCACCCAGGCGGCCCGGGTTCGACTCCCGGTGTGGGAACC/	6.35604326	23.18979	3.49234	2.997733
tsrna-19576	TCACCCAGGCGGCCCGGGTTCGACTCCCGGTGTGGGAACC	0.35708108	0.936961	0.488928	0.333081
tsrna-19575	TCACCCAGGCGGCCCGGGTTCGACTCCCGGTGTGGGAAC	0.21424865	0.15616	0.139694	0
tsrna-19573	TCACCCAGGCGGCCCGGGTTCGACTCCCGGTGTG	0	0.07808	0	0
tsrna-19570	TCACCCAGGCGGCCCGGGTTCGACTCCC	0	0	0	0
tsrna-19564	TCACCCAGGCGGCCCGGG	0	0	0	0
tsrna-19561	TCACATCACCCCATAAACACCA	0.21424865	0.07808	0.279387	0.333081
tsrna-19554	TCACACGCGAAAGGTCCCCGGTTT	0.14283243	0.936961	0	0
tsrna-19553	TCACACGCGAAAGGTCCCCGGTTCGAAACC	0.21424865	0.546561	0	0.111027
tsrna-19552	TCACACGCGAAAGGTCCCCGGTT	0.14283243	0.468481	0	0.111027
tsrna-19551	TCACACGCGAAAGGTCCCCGGT	0.14283243	0.468481	0	0
tsrna-19549	TCACACGCGAAAGGTCCCCG	0	0.07808	0	0
tsrna-19547	TCACAAGAACTGCTAACTCATGCCCCCATGTCTAACAACATG(	0	0.23424	0.279387	0.222054
tsrna-19546	TCACAAGAACTGCTAACTCATGCCCCCATGTCTAACAACATG(	0	0.15616	0.139694	0
tsrna-19545	TCACAAGAACTGCTAACTCATGCCCCCATGTCTAACAACATG(	0.07141622	0.15616	0.139694	0
tsrna-19544	TCACAAGAACTGCTAACTCATGCCCCCATGTCTAACAACA	0.07141622	0.07808	0.069847	0
tsrna-19543	TCACAAGAACTGCTAACTCATGCCCCCATGTCTAACA	0	0	0	0.111027
tsrna-19542	TCACAAGAACTGCTAACTCATGCCCCCATGTCTAAC	0.07141622	0.07808	0.20954	0.111027
tsrna-19541	TCACAAGAACTGCTAACTCATGCCCCCATG	0.21424865	0.07808	0.069847	0
tsrna-19540	TCACAAGAACTGCTAACTCATGCCCCC	0	0	0	0
tsrna-19539	TCACAAGAACTGCTAACTCATGCCC	0	0	0.139694	0
tsrna-19538	TCACAAGAACTGCTAACTCATGCC	0.07141622	0.07808	0	0
tsrna-19537	TCACAAGAACTGCTAACTCATGC	0.07141622	0	0	0
tsrna-19536	TCACAAGAACTGCTAACTCATG	0	0.07808	0	0
tsrna-19534	TCACAAGAACTGCTAACTCA	0.14283243	0	0	0
tsrna-19533	TCACAAGAACTGCTAACTC	0	0	0.069847	0
tsrna-19532	TCACAAGAACTGCTAACT	0.07141622	0	0	0
tsrna-19531	TCACAAGAACTGCTAAC	0	0	0	0.111027
tsrna-19529	TCAATTCTCGCTGGGGCCTCCA	2.78523244	3.279365	1.187396	1.998488
tsrna-19528	TCAATTCCTCTTCTTAACACCA	0	0.23424	0	0.111027
tsrna-19527	TCAATTCCGGCTCGAAGGACCA	3.42797839	11.08738	1.53663	1.776434
tsrna-19526	TCAATTCCCGGCCAATGCACCA	0.78557838	1.171202	0.558774	1.776434
tsrna-19525	TCAATTCCCGGCCAATGCACC	0.21424865	0.23424	0	0.111027
tsrna-19523	TCAATTCCCCGACGGGGAGCC	0.07141622	0	0	0
tsrna-19520	TCAATGGTAGAATTCTCGCCT	0.14283243	0.23424	0.20954	0
tsrna-19519	TCAATGGTAGAATTCTCGCC	0.21424865	0.15616	0	0

tsrna-19518	TCAATGGTAGAATTCTCGC	0	0	0	0
tsrna-19517	TCAATCCCTGGCACCTCCACCA	0.21424865	0.15616	0.069847	0
tsrna-19516	TCAATCCCGGGTTTCGGCACCA	2.49956757	6.324489	0.349234	0.999244
tsrna-19515	TCAATCCCGGGTTTCGGCACC	0.21424865	1.093122	0.069847	0.111027
tsrna-19514	TCAATCCCCGGCATCTCCACCA	9.9982703	11.24354	3.981268	4.774167
tsrna-19513	TCAATCCCCGGCATCTCCACC	0.64274595	0.31232	0.349234	0
tsrna-19510	TCAATCCCCGGCACCTCCACCA	15.6401514	25.5322	8.800698	6.328547
tsrna-19509	TCAATCCCCGGCACCTCCACC	0.78557838	1.327362	0.558774	0.222054
tsrna-19508	TCAATCCCCGGCACCTCCAC	0	0	0.069847	0
tsrna-19504	TCAATCCCCAGCACCTCCACCA	0.28566487	0.624641	0.069847	0.333081
tsrna-19503	TCAATCCCCAGCACCTCCACC	0.07141622	0	0	0
tsrna-19502	TCAATCCCCAATACCTCCACCA	0	0.07808	0.139694	0
tsrna-19501	TCAAGTTCTGGTCTCCGGATGGA	0	0.07808	0	0
tsrna-19498	TCAAGTTCTGGTCTCCGGA	0	0	0.069847	0
tsrna-19494	TCAAGTCTCGGTGGAACCTCCA	3.99930812	8.510732	1.257243	1.443353
tsrna-19493	TCAAGTCTCGGTGGAACCTCC	0.14283243	0.390401	0.069847	0.111027
tsrna-19492	TCAAGTCCCTGTTCGGGCGCCA	0.35708108	0.858881	0.349234	0.222054
tsrna-19488	TCAAGTCCCTGTTCAGGCGCCA	0	0.15616	0	0
tsrna-19487	TCAAGTCCCTGTTCAGGCGCC	0	0.07808	0	0.111027
tsrna-19486	TCAAGTCCCTGTTCAGGCACCA	0	0.23424	0.139694	0
tsrna-19485	TCAAGTCCCTGTCCAGGCGCCA	0	0.07808	0	0
tsrna-19484	TCAAGTCACGTCGGGGTCACCA	0.28566487	0.31232	0.279387	0.444109
tsrna-19483	TCAAGTCACGTCGGGGTCACC	0	0.07808	0.069847	0
tsrna-19477	TCAAGCCCCAGTGGAACCACCA	0.14283243	0.390401	0.139694	0.555136
tsrna-19476	TCAAGAGGTCCCTGGTTCAAATCCGGGTGCCCCCTCC	0.57132973	1.093122	0.069847	0.222054
tsrna-19475	TCAAGAGGTCCCCGGTTCAAATCCGGGTGCCCCCTCC	0.35708108	0.546561	0.20954	0.222054
tsrna-19474	TCAAGAGGTCCCCGGTTCAAATCCGGGTGCCCCCTC	0	0.31232	0.20954	0
tsrna-19473	TCAAGAGGTCCCCGGTTCAAATCCGGGTGCCCCCT	0.07141622	0.23424	0.139694	0.111027
tsrna-19472	TCAAGAGGTCCCCGGTTCAAATCCGGGTGC	0	0.23424	0	0.222054
tsrna-19471	TCAAGAGGTCCCCGGTTCAAA	0.28566487	0.31232	0	0.111027
tsrna-19470	TCAAGAGGTCCCCGGTTCA	0.14283243	0.07808	0.069847	0
tsrna-19469	TCAAGAGGTCCCCGGTTC	0.14283243	0.23424	0.069847	0
tsrna-19468	TCAAGAGGTCCCCGGTT	0.07141622	0.390401	0	0.222054
tsrna-19467	TCAAGAGGTCCCCGGT	0.07141622	0.31232	0	0.111027
tsrna-19466	TCAACTTAACTTGACCGCTCTGACCA	0.99982703	1.795843	0.698468	0.77719
tsrna-19465	TCAACTTAACTTGACCGCTCTGACC	0.21424865	0.15616	0.20954	0
tsrna-19464	TCAACTTAACTTGACCGCTCTGAC	0.14283243	0	0	0
tsrna-19463	TCAACTTAACTTGACCGCTCTGA	0.07141622	0	0	0
tsrna-19462	TCAACTTAACTTGACCGCTCTG	0.14283243	0.07808	0.069847	0
tsrna-19455	TCAAATCTGGGTGCCCCCTCCA	0.07141622	0.07808	0	0
tsrna-19454	TCAAATCTCGGTGGGACCTCCA	5.71329731	8.354572	2.514485	2.664651
tsrna-19453	TCAAATCTCGGTGGGACCTCC	0.49991351	0.624641	0	0
tsrna-19452	TCAAATCTCGGTGGGACCTC	0	0	0	0
tsrna-19451	TCAAATCTCGGTGGGACC	0	0.07808	0	0
tsrna-19450	TCAAATCTCGGTGGAACCTCCA	11.1409298	17.02146	3.2828	1.776434
tsrna-19449	TCAAATCTCGGTGGAACCTCC	1.14265946	1.015041	0.069847	0
tsrna-19448	TCAAATCTCGGTGGAACCTC	0.07141622	0.31232	0	0
tsrna-19447	TCAAATCTCGGTGGAACCT	0.07141622	0.07808	0	0
tsrna-19444	TCAAATCTCGCTGGGGCCTCCA	2.64240001	4.372486	1.117549	2.33157
tsrna-19443	TCAAATCTCGCTGGGGCCTCC	0.49991351	0.390401	0.069847	0.111027
tsrna-19442	TCAAATCTCGCTGGGGCCTC	0	0	0	0
tsrna-19441	TCAAATCCGGGTGCCCCCTCCA	10.5696	12.88322	5.378204	2.109516
tsrna-19440	TCAAATCCGGGTGCCCCCTCC	0.21424865	0.858881	0	0
tsrna-19439	TCAAATCCGGGTGCCCCCTC	0	0.23424	0	0
tsrna-19436	TCAAATCCCGGACGAGCCCCCA	8.35569732	14.5229	3.003413	6.550601
tsrna-19435	TCAAATCCCGGACGAGCCCCC	0.35708108	0.546561	0.069847	0.333081
tsrna-19434	TCAAATCCCGGACGAGCCCC	0.07141622	0.23424	0.069847	0
tsrna-19433	TCAAATCCCGGACGAGCCC	0	0	0	0
tsrna-19432	TCAAATCCCGGACGAGCC	0	0	0	0
tsrna-19429	TCAAATCCCACCGCTGCCACCA	0	0	0	0
tsrna-19428	TCAAATCCAGGTGCCCCCTCCA	0.14283243	0.31232	0	0.222054
tsrna-19427	TCAAATCACGTCGGGGTCACCA	0.35708108	0.780801	0.488928	0.999244
tsrna-19426	TCAAATCACGTCGGGGTCACC	0.07141622	0	0.069847	0
tsrna-19420	TCAAAGTTAAATTATAGGCTAAATCCTATATATCTTACCA	0.49991351	0.31232	0.558774	0

tsrna-19419	TCAAAGTTAAATTATAGGCTAAATCCTATATATCTTACC	0.35708108	0	0.558774	0.111027
tsrna-19418	TCAAAGTTAAATTATAGGCTAAATCCTATATATCTT	0.64274595	0.15616	0.838162	0
tsrna-19417	TCAAAGTTAAATTATAGGCTAAATCCTATATATCT	0.57132973	0.07808	0.908008	0.111027
tsrna-19416	TCAAAGTTAAATTATAGGCTAAATCCTATATATC	0.4284973	0.23424	0.628621	0.111027
tsrna-19415	TCAAAGTTAAATTATAGGCTAAATCCTATATA	0.64274595	0.15616	0.349234	0
tsrna-19414	TCAAAGTTAAATTATAGGCTAAATCCTAT	0.21424865	0.07808	0.069847	0
tsrna-19409	TCAAAGGTTGTGGGTTCGAGTCCCACCAGAGTCGCCA	0.28566487	1.093122	0.069847	0.222054
tsrna-19408	TCAAAGGTTGTGGGTTCGAGTCCCACCAGAGTCGCC	0	0.15616	0	0
tsrna-19407	TCAAAGGTTGTGGGTTCGAGTCCCACCAGA	0	0.23424	0	0
tsrna-19406	TCAAAGGTTGTGGGTTCGAGTCCCACCAG	0	0.07808	0.069847	0
tsrna-19405	TCAAAGGTTGTGGGTTCGAATCCCACCAGAGTCGCCA	0.4284973	0.780801	0	0
tsrna-19404	TCAAAGGTTGTGGGTTCGAATCCCACCAGA	0.07141622	0	0	0
tsrna-19403	TCAAAGGTTGTGGGTTCGAATCCCACCAG	0	0.07808	0	0
tsrna-19402	TCAAAGGTTGTGGGTTC	0.07141622	0.07808	0	0.222054
tsrna-19401	TCAAAGGTTGTGGGTT	0	0.07808	0	0
tsrna-19400	TCAAAGGTTCCGGGTTCGAGTCCCGGCGGAGTCGCCA	0.07141622	0.23424	0	0
tsrna-19399	TCAAAGGTTCCGGGTTCGAGTCCCGGCGGAGTCGCC	0	0	0	0
tsrna-19398	TCAAAGGTTCCGGGTTCGAGTCCCGGCGGAGTCGC	0	0	0.069847	0
tsrna-19397	TCAAAGGTTCCGGGTTCGAGTCCCGGCGGAGTC	0	0	0	0
tsrna-19396	TCAAAGGTTCCGGGTTCGAGTCCCGGCGGA	0	0.07808	0	0
tsrna-19393	TCAAAGGTTCCGGGTTC	0.07141622	0.07808	0	0
tsrna-19392	TCAAAGGTTCCGGGTT	0	0.07808	0	0
tsrna-19385	TCAAAGCAATACACTGAAAATGTTTAGACGGGCTCA	0.92841081	1.717762	0.488928	0
tsrna-19384	TCAAAGCAATACACTGAAAATGTTTAGACG	0	0.07808	0	0
tsrna-19383	TCAAAGCAATACACTGAA	0	0	0	0
tsrna-19380	TCAAACCTGCCGGGGCTTCCA	0.07141622	0.468481	0.139694	0.222054
tsrna-19379	TCAAACCTGCCGGGGCTTCC	0	0	0	0
tsrna-19376	TCAAACCTGCCGGGGCT	0.07141622	0	0	0
tsrna-19373	TATTGAATTGCAAATTCGAAGAAGCAGCTTCAAACCTGCCGG	0.07141622	0	0	0
tsrna-19372	TATTGAATTGCAAATTCGAAGAAGCAGCTTCAAACCTGCCGG	0	0.07808	0.069847	0
tsrna-19370	TATGTCCGCGTGGGTTCGAACC	0.35708108	0.31232	0.069847	0
tsrna-19369	TATGTCCGCGTGGGTTCGAAC	0.35708108	0.468481	0.069847	0
tsrna-19368	TATGTCCGCGTGGGTTCGAA	0.21424865	0.624641	0.20954	0
tsrna-19367	TATGTCCGCGTGGGTTCGA	0.07141622	0.390401	0.069847	0
tsrna-19366	TATGTCCGCGTGGGTTCG	0.21424865	0.07808	0	0
tsrna-19365	TATGTCCGCGTGGGTT	0.07141622	0.31232	0.139694	0
tsrna-19364	TATGTAGCTTACCTCCTCAAAGCA	0	0	0	0.111027
tsrna-19360	TATGTAGCTTACCTCC	0.07141622	0	0	0
tsrna-19359	TATGGTCTAGCGGTTAGGATTCCTGGTTTT	0.35708108	0.702721	0.698468	0.222054
tsrna-19358	TATGGTCTAGCGGTTAGGATTCCTGGTT	0.35708108	0.390401	0.279387	0.222054
tsrna-19357	TATGGTCTAGCGGTTAGGATTCCTGG	0.28566487	0.31232	0.139694	0
tsrna-19356	TATGGTCTAGCGGTTAGGATTCCTG	0.07141622	0.07808	0	0
tsrna-19355	TATGGTCTAGCGGTTAGGATTCCT	0.14283243	0.07808	0.069847	0.111027
tsrna-19354	TATGGTCTAGCGGTTAGGATTCC	0	0	0	0
tsrna-19347	TATGATTCTCGGTTTGGGTC	0.07141622	0	0	0
tsrna-19344	TATGATTCTCGGTTTGG	0	0.07808	0	0
tsrna-19342	TATGATTCTCGCTTTGGGTGCGAGAGGTCCCGGGTT	0.71416216	5.856008	0.279387	2.664651
tsrna-19341	TATGATTCTCGCTTTGGGTGCGAGAGGTCCCGGGT	0.64274595	4.684807	0.20954	1.110271
tsrna-19338	TATGATTCTCGCTTCGGGTGTGAGAGGTCCCGGGT	0.14283243	0.07808	0	0
tsrna-19337	TATGATTCTCGCTTCGGGTGTGAGAGGTCC	0.07141622	0	0	0
tsrna-19333	TATGATTCTCGCTTCGGGTGCGAGAGGTCCCGGGT	0	0.780801	0.069847	0
tsrna-19329	TATGATTCTCGCTTCGGGTG	0.14283243	0	0	0
tsrna-19326	TATGATAATCATATTTACCAACC	0	0	0.069847	0
tsrna-19320	TATGAGGTCCCGGGTTCGATCCCCGGC	0	0.31232	0	0
tsrna-19319	TATGAGGTCCCGGGTTCGA	0	0.468481	0	0
tsrna-19318	TATGAGGCCCCGGGTTCGATCCCCGGC	0.07141622	0.468481	0	0
tsrna-19317	TATGAGGCCCCGGGTTCAATCCCCGGC	0	0.23424	0	0
tsrna-19316	TATCGGGCCCATACCCCGAAAATGTTGGTTATACCC	0.07141622	0.15616	0.069847	0
tsrna-19315	TATCGGGCCCATACCCCGAAAATG	0.14283243	0	0	0
tsrna-19314	TATCGGGCCCATACCCCGAAA	0	0.07808	0	0
tsrna-19313	TATCGGGCCCATACCC	0	0.07808	0	0
tsrna-19312	TATCCCCGCCTGTCACGCGGGAGACCGG	0.8569946	4.060166	0.628621	0.77719
tsrna-19307	TATCCATTGGTCTTAGGCCCCA	0.14283243	0.07808	0	0
tsrna-19306	TATCCATTGGTCTTAGGCCCC	0	0	0	0

tsrna-19303	TATCCATTGGTCTTAGGC	0	0.07808	0	0
tsrna-19301	TATCCATTGGTCTTAG	0	0	0	0
tsrna-19300	TATCATTGGTCGTGGTTGTAGTCCGTGCGAGAATACCA	9.06985949	12.9613	11.10564	5.995465
tsrna-19299	TATCATTGGTCGTGGTTGTAGTCCGTGCGAGAATACC	1.07124325	1.249282	2.095404	1.776434
tsrna-19298	TATCATTGGTCGTGGTTGTAGTCCGTGCGAGAATAC	0.78557838	1.483522	0.838162	1.332326
tsrna-19297	TATCATTGGTCGTGGTTGTAGTCCGTGCGAGAATA	1.49974054	1.015041	0.768315	1.887461
tsrna-19296	TATCATTGGTCGTGGTTGTAGTCCGTGCGAGAAT	0.71416216	0.936961	0.768315	1.110271
tsrna-19295	TATCATTGGTCGTGGTTGTAGTCCGTGCGAGAA	0.92841081	1.093122	0.628621	0.999244
tsrna-19294	TATCATTGGTCGTGGTTGTAGTCCGTGCGAGA	0.57132973	0.936961	0.419081	0.77719
tsrna-19293	TATCATTGGTCGTGGTTGTAGTCCGTGCGAG	0.57132973	0.546561	0.349234	0.555136
tsrna-19292	TATCATTGGTCGTGGTTGTAGTCCGTGCGA	0.35708108	0.15616	0.279387	0.555136
tsrna-19291	TATCATTGGTCGTGGTTGTAGTCCGTGCG	0.14283243	0.702721	0.698468	0.222054
tsrna-19290	TATCATTGGTCGTGGTTGTAGTCCGTGC	0.07141622	0.31232	0.139694	0.666163
tsrna-19289	TATCATTGGTCGTGGTTGTAGTCCGTG	0.21424865	0.23424	0.069847	0.111027
tsrna-19288	TATCATTGGTCGTGGTTGTAGTCCGT	0.21424865	0.23424	0.20954	0
tsrna-19287	TATCATTGGTCGTGGTTGTAGTCCG	0.14283243	0	0.139694	0
tsrna-19286	TATCATTGGTCGTGGTTGTAGTCC	0.14283243	0.07808	0.139694	0
tsrna-19285	TATCATTGGTCGTGGTTGTAGTC	0.07141622	0	0	0
tsrna-19284	TATCATTGGTCGTGGTTGTAGT	0	0	0	0
tsrna-19283	TATCATTGGTCGTGGTTGTAG	0	0	0	0
tsrna-19275	TATCACGTTCGCCTCACACGC	0	0	0	0
tsrna-19270	TATCACGTCTGCTTTACACGC	0	0.07808	0	0
tsrna-19269	TATCACGTCTGCTTTACACG	0	0.07808	0	0
tsrna-19266	TATAGTGGTTAGTACTCTGCGTTGTGGCCG	2.07107028	0.858881	1.047702	0.666163
tsrna-19265	TATAGTGGTTAGTACTCTGCGTTGTGGCC	2.35673514	1.327362	0.908008	0.666163
tsrna-19264	TATAGTGGTTAGTACTCTGCGTTGTGGC	1.71398919	0.390401	0.768315	1.55438
tsrna-19263	TATAGTGGTTAGTACTCTGCGTTGTGG	2.21390271	0.936961	1.047702	0.77719
tsrna-19262	TATAGTGGTTAGTACTCTGCGTTGTG	2.21390271	1.249282	0.628621	0.888217
tsrna-19261	TATAGTGGTTAGTACTCTGCGTTGT	1.57115676	0.390401	0.698468	0.77719
tsrna-19260	TATAGTGGTTAGTACTCTGCGTTG	1.49974054	0.624641	0.628621	0.666163
tsrna-19259	TATAGTGGTTAGTACTCTGCGTT	1.21407568	0.31232	0.419081	0.444109
tsrna-19258	TATAGTGGTTAGTACTCTGCGT	0.35708108	0.07808	0.20954	0
tsrna-19257	TATAGTGGTTAGTACTCTGCGCT	0.35708108	0.23424	0.349234	0.222054
tsrna-19256	TATAGTGGTTAGTACTCTGCGC	0.14283243	0.15616	0.139694	0
tsrna-19255	TATAGTGGTTAGTACTCTGCG	0.14283243	0.23424	0.069847	0
tsrna-19254	TATAGTGGTTAGTACTCTGC	0.07141622	0.15616	0.069847	0
tsrna-19253	TATAGTGGTTAGTACTCTG	0	0	0	0.111027
tsrna-19252	TATAGTGGTTAGTACTCT	0.07141622	0	0	0
tsrna-19250	TATAGTGGTGAGTATCCCCGCCTGTC	0.21424865	0.624641	0.139694	0.444109
tsrna-19249	TATAGTGGTGAGTATCCCCGCCTGT	0.07141622	0.23424	0	0.111027
tsrna-19248	TATAGTGGTGAGTATCCCCGCCT	0.07141622	0.31232	0	0
tsrna-19247	TATAGTGGTGAGTATCCCCGCC	0.07141622	0.468481	0	0
tsrna-19246	TATAGTGGTGAGTATCCCCGC	0.07141622	0.15616	0	0
tsrna-19244	TATAGTGGTGAGCATAGCTGCCT	0	0.07808	0	0
tsrna-19243	TATAGTGGTGAGCATAGCTGC	0	0.07808	0	0
tsrna-19241	TATAGCTCAGTGGTAGAGCATTTGACTGC	0.4284973	0.23424	0.069847	0.222054
tsrna-19240	TATAGCTCAGTGGTAGAGCATTTGACTG	0.07141622	0	0.069847	0
tsrna-19239	TATAGCTCAGTGGTAGAGCATTTGACT	0.14283243	0.07808	0.139694	0
tsrna-19237	TATAGCTCAGTGGTAGAGCATTTG	0	0	0.069847	0
tsrna-19236	TATAGCTCAGTGGGTAGAGCATTTGACTG	0	0.15616	0	0
tsrna-19235	TATAGCTCAGTGGGTAGAGCATTTGACT	0	0	0.069847	0
tsrna-19233	TATAGCTCAGGTGGTAGAGCATTTGACTG	0.07141622	0.15616	0	0
tsrna-19232	TATAGCTCAGGGGTTAGAGCACTGG	0	0.07808	0	0
tsrna-19231	TATAGCTCAGGGGTAGAGCATTTGACTGC	0.07141622	0	0.069847	0.444109
tsrna-19230	TATAGCTCAGGGGTAGAGCATTTGACTG	0	0.15616	0	0.222054
tsrna-19229	TATAGCTCAGGGGTAGAGCATTTGACT	0	0	0	0
tsrna-19228	TATAGCTCAGGGGTAGAGCATTTG	0	0.07808	0	0.111027
tsrna-19227	TATACCCTTCCCGTACTACCA	0.07141622	0.15616	0.069847	0
tsrna-19226	TATACCCTTCCCGTACTACC	0.07141622	0	0	0
tsrna-19222	TATAATGCCGAGGTTGTGAGTTCG	0.35708108	1.873923	0.558774	0.111027
tsrna-19221	TATAATGCCGAGGTTGTGAGTTC	0.07141622	0.546561	0.139694	0
tsrna-19220	TATAATGCCGAGGTTGTGAGTT	0.07141622	0.702721	0.069847	0.111027
tsrna-19219	TATAATGCCGAGGTTGTGAGT	0.21424865	0.15616	0	0
tsrna-19218	TATAATGCCGAGGTTGTGAG	0.21424865	0.468481	0.069847	0

tsrna-19217	TATAATGCCGAGGTTGTG	0	0.15616	0.069847	0
tsrna-19216	TATAAATAGTACCGTTAACTTCCA	0	0	0.069847	0
tsrna-19215	TATAAATAGTACCGTTAACTTCC	0	0	0	0
tsrna-19214	TATAAATAGTACCGTTAACTTC	0	0	0	0
tsrna-19212	TAGTTTTGACAACATTCAAAAAAGAGTACC	0	0	0.069847	0.222054
tsrna-19204	TAGTGTAGTGGTTATCACGTTCGCCT	0	0.07808	0	0
tsrna-19201	TAGTGTAGTGGTTATCACGTCTGCTTTACACGCAGA	0.21424865	0	0.139694	0.111027
tsrna-19200	TAGTGTAGTGGTTATCACGTCTGCTTT	0	0	0	0.111027
tsrna-19199	TAGTGTAGTGGTTATCACGTCTGCTT	0	0	0	0.111027
tsrna-19192	TAGTGTAGTGGTCATCACGTTCGCCT	0	0.07808	0	0
tsrna-19191	TAGTGTAGTGGTCATCACGTTCGCC	0	0	0	0
tsrna-19188	TAGTGTAGTGGTCATC	0	0	0	0
tsrna-19187	TAGTGTAGCGGTTATCACGTCTGC	0	0	0	0
tsrna-19186	TAGTGTAGCGGTTATCACATTCGCC	0.07141622	0	0.069847	0
tsrna-19185	TAGTGTAGCGGTTATC	0.07141622	0	0	0
tsrna-19184	TAGTGGTTATCACGTTCGCCTCACACGC	0.07141622	0	0	0
tsrna-19182	TAGTGGTTATCACGTTCGCCTC	0	0	0	0
tsrna-19180	TAGTGGTTATCACGTTCGCC	0	0.07808	0	0
tsrna-19177	TAGTGGTTATCACGTCTGCTTTACA	0.07141622	0	0.20954	0
tsrna-19175	TAGTGGTTATCACGTCTGCTT	0.07141622	0	0	0
tsrna-19172	TAGTGGTTAGTATCCCCGCCTGTCACGCGG	0.92841081	0.390401	0.279387	0.666163
tsrna-19171	TAGTGGTTAGTATCCCCGCCTGTC	0.14283243	0.07808	0.279387	0.111027
tsrna-19170	TAGTGGTTAGTATCCCCGCCTGT	0.14283243	0	0	0.111027
tsrna-19169	TAGTGGTTAGTATCCCCGCCTG	0	0.07808	0.069847	0
tsrna-19168	TAGTGGTTAGTATCCCCGCCT	0.07141622	0	0	0
tsrna-19167	TAGTGGTTAGTATCCCCGCC	0.07141622	0	0	0
tsrna-19166	TAGTGGTTAGTATCCCCGC	0.14283243	0	0	0
tsrna-19165	TAGTGGTTAGTATCCCCG	0.07141622	0	0	0
tsrna-19164	TAGTGGTTAGTACTCTGCGTTGTGGCCGCAGCAACCT	3.78505947	1.561602	1.187396	0.666163
tsrna-19163	TAGTGGTTAGTACTCTGCGTTGTGGCCGC	1.35690811	0.546561	0.977855	1.221299
tsrna-19162	TAGTGGTTAGTACTCTGCGTTGTGGCC	1.92823784	0.858881	0.419081	0.333081
tsrna-19161	TAGTGGTTAGTACTCTGCGTTGTGGC	1.85682163	0.780801	0.558774	0.888217
tsrna-19160	TAGTGGTTAGTACTCTGCGTTGTGG	1.92823784	1.171202	1.327089	0.555136
tsrna-19159	TAGTGGTTAGTACTCTGCGTTGTG	2.57098379	0.858881	0.349234	0.77719
tsrna-19158	TAGTGGTTAGTACTCTGCGTTGT	1.57115676	0.702721	0.768315	0.666163
tsrna-19157	TAGTGGTTAGTACTCTGCGTTG	1.85682163	1.015041	0.419081	0.444109
tsrna-19156	TAGTGGTTAGTACTCTGCGTT	1.57115676	0.390401	0.349234	0.444109
tsrna-19155	TAGTGGTTAGTACTCTGCGT	0.4284973	0.23424	0.139694	0.111027
tsrna-19154	TAGTGGTTAGTACTCTGCGCTGT	0.57132973	0	0.349234	0.222054
tsrna-19153	TAGTGGTTAGTACTCTGCGCTG	0.28566487	0.23424	0.139694	0.111027
tsrna-19152	TAGTGGTTAGTACTCTGCGCT	0.14283243	0.07808	0.279387	0
tsrna-19151	TAGTGGTTAGTACTCTGCGC	0.4284973	0.07808	0.069847	0.111027
tsrna-19150	TAGTGGTTAGTACTCTGCG	0.07141622	0.07808	0	0
tsrna-19149	TAGTGGTTAGTACTCTGC	0	0	0	0.111027
tsrna-19147	TAGTGGTTAGTACTCT	0	0	0	0
tsrna-19146	TAGTGGTTAGGATTCGGCGCTCTCACCGCCGCGGCCCGGGT	3.64222704	5.699848	2.793872	1.998488
tsrna-19145	TAGTGGTTAGGATTCGGCGCTCTCACCGCCGCGGCCCG	4.07072434	6.636809	2.514485	1.55438
tsrna-19144	TAGTGGTTAGGATTCGGCGCTCTCACCGCCGCGGCCC	4.21355677	5.934088	2.374791	2.664651
tsrna-19143	TAGTGGTTAGGATTCGGCGCTCTCACCGCCGCGGCC	3.07089731	3.123204	1.74617	2.886706
tsrna-19142	TAGTGGTTAGGATTCGGCGCTCTCACCGCC	2.78523244	4.294406	2.235098	2.33157
tsrna-19141	TAGTGGTTAGGATTCGGCGCTCTCACCGC	1.78540541	3.747845	1.74617	2.109516
tsrna-19140	TAGTGGTTAGGATTCGGCGCTCTCACC	2.57098379	3.045124	2.025557	1.55438
tsrna-19139	TAGTGGTTAGGATTCGGCGCTCTCAC	2.49956757	3.201285	1.466783	2.109516
tsrna-19138	TAGTGGTTAGGATTCGGCGCTCTCA	2.78523244	3.045124	1.606477	1.665407
tsrna-19137	TAGTGGTTAGGATTCGGCGCTCTC	3.07089731	2.967044	1.606477	1.665407
tsrna-19136	TAGTGGTTAGGATTCGGCGCTCT	3.99930812	4.138246	1.955711	1.221299
tsrna-19135	TAGTGGTTAGGATTCGGCGCTC	2.35673514	3.123204	1.466783	0.999244
tsrna-19134	TAGTGGTTAGGATTCGGCGCT	1.99965406	3.357445	1.327089	1.332326
tsrna-19133	TAGTGGTTAGGATTCGGCGC	1.35690811	1.327362	0.698468	0.888217
tsrna-19132	TAGTGGTTAGGATTCGGCG	1.07124325	0.702721	0.768315	0.333081
tsrna-19131	TAGTGGTTAGGATTCGGC	0.8569946	0.31232	0.279387	0.111027
tsrna-19130	TAGTGGTTAGGATTCGG	0.21424865	0	0	0.222054
tsrna-19129	TAGTGGTTAGGATTCG	0	0	0	0
tsrna-19128	TAGTGGTTAGGATTCAGCGCT	1.14265946	1.639682	0.908008	0.444109

tsrna-19127	TAGTGGTTAGCATAGCTGCCTTCC	0	0.07808	0	0
tsrna-19124	TAGTGGTTAGCATAGCTGCCT	0	0.07808	0	0
tsrna-19122	TAGTGGTTAGCATAGCTGC	0.07141622	0	0	0
tsrna-19118	TAGTGGTGAGTATCCCCGCCTGTCACGCGG	0.8569946	1.717762	0.628621	1.332326
tsrna-19117	TAGTGGTGAGTATCCCCGCCTGTCACGC	0.57132973	1.952003	0.768315	0.999244
tsrna-19116	TAGTGGTGAGTATCCCCGCCTGTCAC	0.35708108	1.093122	0.279387	0.666163
tsrna-19115	TAGTGGTGAGTATCCCCGCCTGTCA	0.28566487	0.936961	0	0.111027
tsrna-19114	TAGTGGTGAGTATCCCCGCCTGTC	0.35708108	0.702721	0.069847	0.111027
tsrna-19113	TAGTGGTGAGTATCCCCGCCTGT	0.07141622	0.23424	0	0
tsrna-19112	TAGTGGTGAGTATCCCCGCCTG	0.07141622	0.15616	0	0
tsrna-19111	TAGTGGTGAGTATCCCCGCCT	0	0.390401	0	0
tsrna-19110	TAGTGGTGAGTATCCCCGCC	0.14283243	0.15616	0	0.111027
tsrna-19109	TAGTGGTGAGTATCCCCGC	0.14283243	0.07808	0	0
tsrna-19108	TAGTGGTGAGTATCCCCG	0	0.07808	0	0
tsrna-19105	TAGTGGTGAGCATAGCTGCCTTCC	0	0	0.069847	0
tsrna-19104	TAGTGGTGAGCATAGCTGCCTTC	0.07141622	0	0	0
tsrna-19103	TAGTGGTGAGCATAGCTGCCTT	0.21424865	0.07808	0	0
tsrna-19101	TAGTGGTGAGCATAGCTGCC	0.07141622	0	0	0
tsrna-19097	TAGTGGTGAGCATAGC	0.07141622	0	0	0
tsrna-19096	TAGTGGTCATCACGTTCGCCT	0	0	0.069847	0
tsrna-19095	TAGTGGTCATCACGTTCGCC	0	0.07808	0	0
tsrna-19094	TAGTGGTATGATTCTCGCTTT	0.92841081	0.23424	0.069847	0.111027
tsrna-19093	TAGTGGTATGATTCTCGCTT	0.4284973	0.07808	0.279387	0.666163
tsrna-19092	TAGTGGTATGATTCTCGCT	0.4284973	0.15616	0.139694	0.666163
tsrna-19091	TAGTGGTATGATTCTCGC	0.57132973	0.07808	0	0.666163
tsrna-19083	TAGTGGTAGAATTCTCGCCT	0	0.390401	0.139694	0.111027
tsrna-19082	TAGTGGTAGAATTCTCGCC	0.07141622	0.468481	0.069847	0
tsrna-19081	TAGTGGTAGAATTCTCGC	0	0.07808	0	0.111027
tsrna-19080	TAGTGGTAGAATTCTCG	0	0.07808	0	0
tsrna-19074	TAGTGGCTAGGATTCGGCGCTTTCACCGCCGCGGCCC	1.57115676	4.919047	1.257243	4.66314
tsrna-19073	TAGTGGCTAGGATTCGGCGCTTTC	1.21407568	3.513605	0.698468	3.552868
tsrna-19072	TAGTGGCTAGGATTCGGCGCTTT	0.8569946	3.201285	0.558774	3.441841
tsrna-19071	TAGTGGCTAGGATTCGGCGCTT	1.21407568	2.810884	0.349234	3.552868
tsrna-19070	TAGTGGCTAGGATTCGGCGCT	1.2854919	2.888964	0.419081	3.996977
tsrna-19069	TAGTGGCTAGGATTCGGCGC	0.4284973	2.186243	0.419081	2.553624
tsrna-19068	TAGTGGCTAGGATTCGGCG	0.57132973	1.561602	0.419081	2.553624
tsrna-19067	TAGTGGCTAGGATTCGGC	0.4284973	1.093122	0.279387	0.77719
tsrna-19066	TAGTGGCTAGGATTCGG	0.07141622	0	0	0
tsrna-19065	TAGTCGTGGCCGAGTGGTTAAGGT	0	0	0.069847	0
tsrna-19064	TAGTCGTGGCCGAGTGGTTAAGGC	0	0	0	0
tsrna-19063	TAGTCGTGGCCGAGTGGTTAAGG	0.07141622	0.07808	0	0
tsrna-19062	TAGTCGTGGCCGAGTGGTTAAG	0	0	0	0.111027
tsrna-19061	TAGTCGTGGCCGAGTGGTTAA	0.07141622	0	0	0
tsrna-19060	TAGTCGTGGCCGAGTGGTTA	0.07141622	0	0	0
tsrna-19059	TAGTCGTGGCCGAGTGG	0.07141622	0.07808	0	0
tsrna-19058	TAGTCCGTGCGAGAATACCA	0.07141622	0	0	0
tsrna-19057	TAGTCCGTGCGAGAATACC	0	0	0	0
tsrna-19056	TAGTCCGTGCGAGAATAC	0	0	0	0
tsrna-19054	TAGTATCCCCGCCTGTCACGC	0	0.07808	0	0
tsrna-19052	TAGTATCCCCGCCTGTC	0	0.07808	0	0
tsrna-19049	TAGTACTCTGCGTTGTGGCCGCAGCAACCTCGGT	0.78557838	0.15616	0.279387	0.222054
tsrna-19048	TAGTACTCTGCGTTGTGGCCGCAGCAACCT	0.07141622	0.15616	0	0
tsrna-19047	TAGTACTCTGCGTTGTGGCCGCAGC	0	0.15616	0	0
tsrna-19045	TAGTACTCTGCGTTGTGGCCGC	0.07141622	0	0	0
tsrna-19044	TAGTACTCTGCGTTGTGGCCG	0.07141622	0.15616	0	0
tsrna-19043	TAGTACTCTGCGTTGTGGCC	0.14283243	0	0	0
tsrna-19042	TAGTACTCTGCGTTGTGGC	0.07141622	0	0	0
tsrna-19041	TAGTACTCTGCGTTGTGG	0	0	0	0
tsrna-19040	TAGTACTCTGCGTTGTG	0	0	0	0
tsrna-19039	TAGTACTCTGCGTTGT	0	0	0	0
tsrna-19038	TAGTACCGTTAACTTCCAATTAACTAGTTT	0	0.15616	0	0
tsrna-19037	TAGTACCGTTAACTTCCAATTA	0.07141622	0	0.069847	0
tsrna-19036	TAGTACCGTTAACTTCCAATT	0	0	0	0
tsrna-19035	TAGTACCGTTAACTTCCA	0.07141622	0	0	0

tsrna-19032	TAGGTTCGACTCCTGGCTGGCTCGCCA	2.78523244	10.77506	0.838162	0.888217
tsrna-19031	TAGGTTCGACTCCTGGCTGGCTCGCC	0	0.31232	0.069847	0.111027
tsrna-19030	TAGGTTCGACTCCTGGCTGGCTCG	0	0.15616	0	0
tsrna-19029	TAGGTTCGACTCCTGGCTGGCTC	0	0	0	0
tsrna-19027	TAGGTGGTTCAGTGGTAGAATTCT	0.35708108	0.15616	0.069847	0.333081
tsrna-19026	TAGGTGGCACGGAGAATTTTGGATT	0	0	0	0
tsrna-19025	TAGGTGGCACGGAGAATTTTGGAT	0	0.15616	0.069847	0
tsrna-19024	TAGGTGGCACGGAGAATTTTGGA	0.07141622	0	0	0
tsrna-19023	TAGGTGGCACGGAGAATTTTG	0	0	0	0.111027
tsrna-19021	TAGGTCGCTGGTTCGATTCCGGC	0	0.07808	0.069847	0
tsrna-19020	TAGGTCGCTGGTTCGAATCCGGCT	0.07141622	0	0	0
tsrna-19019	TAGGTCGCTGGTTCGAATCCGGC	0	0	0	0.111027
tsrna-19018	TAGGTCGCTGGTTCGAATCCGG	0	0	0.139694	0
tsrna-19016	TAGGGTGCTTAGCTGTTAACTA	0	0	0	0.111027
tsrna-19013	TAGGGGTATGATTCTCGGTTTGG	0.28566487	0.07808	0.139694	0
tsrna-19012	TAGGGGTATGATTCTCGGTTTG	0.35708108	0.07808	0.139694	0
tsrna-19011	TAGGGGTATGATTCTCGGTTT	0.21424865	0.15616	0	0.111027
tsrna-19010	TAGGGGTATGATTCTCGGTT	0.14283243	0.07808	0.139694	0.222054
tsrna-19009	TAGGGGTATGATTCTCGGT	0.07141622	0	0.069847	0
tsrna-19008	TAGGGGTATGATTCTCGG	0.07141622	0	0	0
tsrna-19007	TAGGGGTATGATTCTCGCTTTG	1.14265946	0.780801	0.349234	0.555136
tsrna-19006	TAGGGGTATGATTCTCGCTTT	0.99982703	0.936961	0.349234	1.221299
tsrna-19005	TAGGGGTATGATTCTCGCTTCG	0.92841081	1.093122	0.558774	1.221299
tsrna-19004	TAGGGGTATGATTCTCGCTTC	0.71416216	0.780801	0.279387	2.33157
tsrna-19003	TAGGGGTATGATTCTCGCTT	0.64274595	0.702721	0.279387	0.666163
tsrna-19002	TAGGGGTATGATTCTCGCT	0.92841081	0.780801	0.488928	0.111027
tsrna-19001	TAGGGGTATGATTCTCGC	0.78557838	0.702721	0.628621	0.666163
tsrna-19000	TAGGGGTATGATTCTCG	0	0	0.069847	0
tsrna-18994	TAGGCTAAATCCTATATATCTTACCA	0	0	0	0
tsrna-18993	TAGGCTAAATCCTATATATCTTACC	0.14283243	0	0	0.111027
tsrna-18992	TAGGCTAAATCCTATATATCT	0	0.07808	0	0
tsrna-18991	TAGGCTAAATCCTATATATC	0.07141622	0	0	0
tsrna-18990	TAGGCCTCTTTTTACCACCA	0.07141622	0	0	0.111027
tsrna-18985	TAGGATTCGGTGCTCTCA	0	0	0.069847	0
tsrna-18983	TAGGATTCGGCGCTTTCACCGCCGCGGCCC	0.07141622	1.795843	0.488928	0.999244
tsrna-18982	TAGGATTCGGCGCTTT	0	0.31232	0.139694	0.111027
tsrna-18981	TAGGATTCGGCGCTCTCAT	0	0	0	0
tsrna-18980	TAGGATTCGGCGCTCTCACCGCCGCGGCCCGGGTTC	0.49991351	1.093122	0.20954	0.444109
tsrna-18979	TAGGATTCGGCGCTCTCACCGCCGCGGCCCGGGTT	0.35708108	1.483522	0.139694	0.222054
tsrna-18978	TAGGATTCGGCGCTCTCACCGCCGCGGCCCGGG	0.21424865	0.546561	0.279387	0.222054
tsrna-18977	TAGGATTCGGCGCTCTCACCGCCGCGGCCC	0.28566487	0.780801	0.349234	0
tsrna-18976	TAGGATTCGGCGCTCTCACCGCCGCGGCC	0.14283243	0.936961	0.419081	0
tsrna-18975	TAGGATTCGGCGCTCTCACCGCCGCGGC	0.64274595	1.015041	0.279387	0.555136
tsrna-18974	TAGGATTCGGCGCTCTCACCGCC	0.21424865	0.936961	0.139694	0.333081
tsrna-18973	TAGGATTCGGCGCTCTCACCGC	0.14283243	0.546561	0.069847	0
tsrna-18972	TAGGATTCGGCGCTCTCACCG	0.07141622	0.15616	0.069847	0.111027
tsrna-18971	TAGGATTCGGCGCTCTCACC	0.14283243	0.23424	0.069847	0.111027
tsrna-18970	TAGGATTCGGCGCTCTCAC	0.07141622	0.15616	0	0
tsrna-18969	TAGGATTCGGCGCTCTCA	0.07141622	0	0.069847	0
tsrna-18968	TAGGATTCGGCGCTCTC	0	0.31232	0	0.111027
tsrna-18967	TAGGATTCGGCGCTCT	0	0.15616	0.139694	0.111027
tsrna-18966	TAGGATTCCTGGTTTTCACCCAGGCGGCCCGGGTTCGACTC(	0.57132973	1.405442	0.349234	0.333081
tsrna-18965	TAGGATTCCTGGTTTTCACCCAGGCGGCCCGGGTTCGACTC	0.92841081	1.639682	0.628621	0.555136
tsrna-18964	TAGGATTCCTGGTTTTCACCCA	0.57132973	0.390401	0.20954	0.111027
tsrna-18963	TAGGATTCCTGGTTTTCACCC	0.57132973	0.858881	0.349234	0.111027
tsrna-18962	TAGGATTCCTGGTTTTC	0.07141622	0	0.20954	0
tsrna-18961	TAGGATGTGGTGTGATAGG	0	0	0	0.111027
tsrna-18960	TAGGATGGGGTGTGATAGGTGGCACGGAGAATTTTGGATTC	3.9278919	3.982086	5.168664	3.996977
tsrna-18959	TAGGATGGGGTGTGATAGGTGGCACGGAGAATTTTGG	3.99930812	3.513605	5.02897	5.329303
tsrna-18958	TAGGATGGGGTGTGATAGGTGGCACGGAGAATTTTG	3.9278919	2.888964	4.609889	3.774923
tsrna-18957	TAGGATGGGGTGTGATAGGTGGCACGGAGAATTTT	2.57098379	3.357445	3.212953	2.997733
tsrna-18956	TAGGATGGGGTGTGATAGGTGGCACGGAGAATTT	2.35673514	3.825925	3.073259	4.219031
tsrna-18955	TAGGATGGGGTGTGATAGGTGGCACGGAGAATT	2.71381622	2.576644	2.793872	1.887461
tsrna-18954	TAGGATGGGGTGTGATAGGTGGCACGGAGAAT	1.78540541	2.342403	1.466783	1.998488

tsrna-18953	TAGGATGGGGTGTGATAGGTGGCACGGAGAA	1.49974054	2.420483	1.676323	1.665407
tsrna-18952	TAGGATGGGGTGTGATAGGTGGCACGGAGA	1.49974054	2.888964	1.885864	2.664651
tsrna-18951	TAGGATGGGGTGTGATAGGTGGCACGGAG	0.49991351	1.327362	0.558774	1.887461
tsrna-18950	TAGGATGGGGTGTGATAGGTGGCACGGA	0.71416216	0.780801	0.488928	1.443353
tsrna-18949	TAGGATGGGGTGTGATAGGTGGCACGG	0.35708108	0.858881	0.628621	0.555136
tsrna-18948	TAGGATGGGGTGTGATAGGTGGCACG	0.64274595	0.702721	0.069847	0.77719
tsrna-18947	TAGGATGGGGTGTGATAGGTGGCAC	0.21424865	0.15616	0.349234	0.222054
tsrna-18946	TAGGATGGGGTGTGATAGGTGGCA	0.21424865	0.390401	0.069847	0.111027
tsrna-18945	TAGGATGGGGTGTGATAGGTGGC	0	0.624641	0.069847	0.111027
tsrna-18944	TAGGATGGGGTGTGATAGGTGG	0.21424865	0.31232	0	0
tsrna-18943	TAGGATGGGGTGTGATAGGTG	0.28566487	0.390401	0.069847	0
tsrna-18942	TAGGATGGGGTGTGATAGGT	0.07141622	0.15616	0	0
tsrna-18941	TAGGATGGGGTGTGATAGG	0	0.23424	0	0.111027
tsrna-18940	TAGGATGGGGTGTGATAG	0.21424865	0.15616	0	0.111027
tsrna-18939	TAGGATGGGGTGTGATA	0.07141622	0.15616	0	0.111027
tsrna-18938	TAGGATGGGGTGTGAT	0.07141622	0.15616	0	0.111027
tsrna-18937	TAGGAGCTTAAACCCCCTTATTTCTACCA	0.14283243	0.31232	0.139694	0
tsrna-18936	TAGGAGCTTAAACCCCCTTATTTCTACC	0.14283243	0.15616	0	0
tsrna-18932	TAGGAGATTTCAACTTAACTTGACCGCTCTGACCA	7.35587029	3.357445	6.076672	2.220543
tsrna-18931	TAGGAGATTTCAACTTAACTTGACCGCTCTGACC	1.21407568	0.546561	0.349234	0.333081
tsrna-18930	TAGGAGATTTCAACTTAACTTGACCGCTCTGAC	0.14283243	0.07808	0.069847	0
tsrna-18929	TAGGAGATTTCAACTTAACTTGACCGCTCT	0.28566487	0	0	0
tsrna-18928	TAGGAGATTTCAACTTAACTTGACCGCTC	0.14283243	0	0.139694	0
tsrna-18927	TAGGAGATTTCAACTTAACTTGACCGCT	0.07141622	0	0	0
tsrna-18926	TAGGAGATTTCAACTTAACTTGACCGC	0.07141622	0	0	0
tsrna-18925	TAGGAGATTTCAACTTAACTTGACCG	0	0	0	0
tsrna-18924	TAGGAGATTTCAACTTAACTTGACC	0	0	0	0
tsrna-18923	TAGGAGATTTCAACTTAACTTGAC	0.07141622	0.07808	0	0
tsrna-18922	TAGGAGATTTCAACTTAACTTGA	0	0	0.069847	0
tsrna-18917	TAGCTTTGGGTGCTAATGGTGGAGTTAAAGACTTTTTCTCTGA	0.92841081	1.952003	0.558774	0
tsrna-18916	TAGCTTTGGGTGCTAATGGTGGAGTTAAAGACTTTT	0.14283243	0.858881	0.139694	0
tsrna-18915	TAGCTTTGGGTGCTAATGGTGGAGTTAAAG	0.14283243	0.390401	0	0
tsrna-18914	TAGCTTTGGGTGCTAATGGTGGAGTTAAA	0	0.624641	0.139694	0
tsrna-18913	TAGCTTTGGGTGCTAATGGTGGAGTTAA	0.35708108	0.23424	0	0
tsrna-18912	TAGCTTTGGGTGCTAATGGTGGAGTTA	0.21424865	0.23424	0.069847	0.111027
tsrna-18911	TAGCTTTGGGTGCTAATGGTGGAGTT	0.07141622	0.07808	0	0
tsrna-18910	TAGCTTTGGGTGCTAATGGTGGAGT	0	0.07808	0	0
tsrna-18909	TAGCTTTGGGTGCTAATGGTGGAG	0	0.15616	0	0
tsrna-18908	TAGCTTTGGGTGCTAATGGTGGA	0	0.07808	0	0
tsrna-18903	TAGCTTAGCGGTAGAGCATTTGACTG	0	0	0	0
tsrna-18901	TAGCTGTTAACTAAGTGTTTGTGGGTTTAAGTCCCATTGGTCT	0.64274595	0.07808	0.628621	0.444109
tsrna-18900	TAGCTGTTAACTAAGTGTTTGTGGGTTTAAGTCCCATTGGTCT	0.35708108	0.15616	0.628621	0.222054
tsrna-18899	TAGCTGTTAACTAAGTGTTTGTGGGTTTAAGTCCCA	0.21424865	0.07808	0.069847	0.111027
tsrna-18898	TAGCTGTTAACTAAGTGTTTGTGGGTTTA	0.07141622	0.15616	0	0.111027
tsrna-18897	TAGCTGTTAACTAAGTGTTTGTGGGTTT	0.07141622	0.23424	0	0
tsrna-18896	TAGCTGTTAACTAAGTGTTTGTGG	0	0	0	0
tsrna-18893	TAGCTGCCTTCCAAGCAGTTGACCCGG	0.21424865	0.23424	0.069847	0
tsrna-18892	TAGCTGCCTTCCAAGCAGTTGACCCG	0	0.23424	0.069847	0
tsrna-18891	TAGCTGCCTTCCAAGCAGTTGACCC	0	0.07808	0	0.222054
tsrna-18884	TAGCTCAGTGGTAGAGCATTTGACTGCA	0.35708108	0.07808	0.20954	0
tsrna-18883	TAGCTCAGTGGTAGAGCATTTGACTGC	0.14283243	0.07808	0.069847	0.111027
tsrna-18882	TAGCTCAGTGGTAGAGCATTTGACTG	0.14283243	0.15616	0	0
tsrna-18881	TAGCTCAGTGGTAGAGCATTTGACTA	0	0.23424	0	0
tsrna-18880	TAGCTCAGTGGTAGAGCATTTGACT	0.07141622	0.31232	0.069847	0
tsrna-18879	TAGCTCAGTGGTAGAGCATTTGAC	0.07141622	0	0	0
tsrna-18878	TAGCTCAGTGGTAGAGCATTTGA	0.07141622	0	0	0
tsrna-18877	TAGCTCAGTGGTAGAGCATTTG	0.21424865	0.07808	0	0
tsrna-18876	TAGCTCAGTGGTAGAGCATTT	0.07141622	0.07808	0	0
tsrna-18874	TAGCTCAGTGGGTAGAGCATTTGACTGC	0.21424865	0.31232	0	0.111027
tsrna-18873	TAGCTCAGTGGGTAGAGCATTTGACTG	0	0.31232	0	0
tsrna-18872	TAGCTCAGTGGGTAGAGCATTTGACT	0	0	0	0
tsrna-18871	TAGCTCAGTCGGTAGAGCATGGGACTCTT	0.71416216	1.561602	0.349234	0.222054
tsrna-18870	TAGCTCAGTCGGTAGAGCATGGGACTCT	1.42832433	2.030083	0.558774	0.333081
tsrna-18869	TAGCTCAGTCGGTAGAGCATGGGACTC	0.92841081	1.561602	0.419081	0.333081

tsrna-18868	TAGCTCAGTCGGTAGAGCATGGGACT	1.14265946	1.171202	0.768315	0.222054
tsrna-18867	TAGCTCAGTCGGTAGAGCATGGGAC	0.4284973	1.561602	0.349234	0.333081
tsrna-18866	TAGCTCAGTCGGTAGAGCATGGGA	0.64274595	1.249282	0.279387	0.111027
tsrna-18865	TAGCTCAGTCGGTAGAGCATGGG	0.14283243	0.07808	0.069847	0.111027
tsrna-18864	TAGCTCAGTCGGTAGAGCATGG	0.07141622	0.15616	0	0
tsrna-18863	TAGCTCAGTCGGTAGAGCATGAGACTCTT	1.49974054	2.420483	0.419081	0.666163
tsrna-18862	TAGCTCAGTCGGTAGAGCATGAGACTCT	1.14265946	2.576644	0.698468	0.111027
tsrna-18861	TAGCTCAGTCGGTAGAGCATGAGACT	0.8569946	0.936961	0.20954	0.555136
tsrna-18860	TAGCTCAGTCGGTAGAGCATGAGAC	0.57132973	1.873923	0.349234	0.222054
tsrna-18859	TAGCTCAGTCGGTAGAGCATGAGA	0.14283243	0.702721	0.139694	0.222054
tsrna-18858	TAGCTCAGTCGGTAGAGCATGAG	0.14283243	0.15616	0.20954	0
tsrna-18857	TAGCTCAGTCGGTAGAGCATGA	0.14283243	0	0	0
tsrna-18856	TAGCTCAGTCGGTAGAGCATG	0.07141622	0	0.139694	0
tsrna-18855	TAGCTCAGTCGGTAGAGCATCAGACTTTT	0.64274595	0.936961	1.257243	0.555136
tsrna-18854	TAGCTCAGTCGGTAGAGCATCAGACTTT	1.14265946	0.390401	0.838162	0.333081
tsrna-18853	TAGCTCAGTCGGTAGAGCATCAGACT	0.92841081	0.702721	0.698468	0.222054
tsrna-18852	TAGCTCAGTCGGTAGAGCATCAGAC	1.14265946	1.093122	0.349234	0.333081
tsrna-18851	TAGCTCAGTCGGTAGAGCATCAGA	0.35708108	0.468481	0.20954	0.222054
tsrna-18850	TAGCTCAGTCGGTAGAGCATCAG	0.14283243	0.07808	0.279387	0.111027
tsrna-18849	TAGCTCAGTCGGTAGAGCATC	0.28566487	0.23424	0.20954	0
tsrna-18844	TAGCTCAGGTGGTAGAGCATTTGACTG	0	0	0	0
tsrna-18842	TAGCTCAGGGGTTAGAGCACTGGTCT	0.21424865	0.390401	0.279387	0.888217
tsrna-18841	TAGCTCAGGGGTAGAGCATTTGACTGC	0.14283243	0.23424	0.069847	0.333081
tsrna-18840	TAGCTCAGGGGTAGAGCATTTGACTG	0.07141622	0.15616	0	0.111027
tsrna-18839	TAGCTCAGGGGTAGAGCATTTGACT	0	0	0	0
tsrna-18837	TAGCTCAGGGGTAGAGCATTTGA	0	0.07808	0	0
tsrna-18832	TAGCGTGGCCGAGTGGTCTAAGG	0	0.07808	0	0
tsrna-18831	TAGCGTGGCCGAGTGGTCTAAG	0	0.07808	0	0
tsrna-18830	TAGCGTGGCCGAGCGGTCTAAGGCGCTGG	0	0.07808	0	0
tsrna-18827	TAGCGTGGCCGAGCGGTCTAAGG	0.07141622	0.15616	0	0
tsrna-18825	TAGCGTGGCCGAGCGGTCTAA	0	0.07808	0	0
tsrna-18824	TAGCGTGGCCGAGCGGTCTA	0.07141622	0	0	0
tsrna-18821	TAGCGTGGCCGAGCGG	0	0.07808	0	0
tsrna-18817	TAGCGGTTAGGATTCCTGGTTTTCACCCAG	1.14265946	1.327362	0.558774	0.77719
tsrna-18816	TAGCGGTTAGGATTCCTGGTTTTCACC	0.8569946	1.015041	0.628621	0.444109
tsrna-18815	TAGCGGTTAGGATTCCTGGTTTTCAC	0.64274595	0.390401	0.349234	0.222054
tsrna-18814	TAGCGGTTAGGATTCCTGGTTTTCA	0.92841081	0.624641	0.349234	0.333081
tsrna-18813	TAGCGGTTAGGATTCCTGGTTTTC	0.14283243	0.468481	0.279387	0.444109
tsrna-18812	TAGCGGTTAGGATTCCTGGTTTT	0.4284973	0	0.139694	0
tsrna-18811	TAGCGGTTAGGATTCCTGGTTT	0.35708108	0.390401	0.069847	0.111027
tsrna-18810	TAGCGGTTAGGATTCCTGGTT	0.07141622	0.390401	0.069847	0.111027
tsrna-18809	TAGCGGTTAGGATTCCTGGT	0.07141622	0.23424	0	0.222054
tsrna-18808	TAGCGGTTAGGATTCCTGG	0.14283243	0.15616	0.069847	0.111027
tsrna-18807	TAGCGGTTAGGATTCCTG	0	0.07808	0.069847	0
tsrna-18804	TAGCGGTAGAGCATTTGACTGC	0	0	0	0
tsrna-18803	TAGCGGTAGAGCATTTGACTG	0.07141622	0.15616	0	0
tsrna-18802	TAGCGGTAGAGCATTTGACT	0	0.07808	0	0
tsrna-18801	TAGCGGGATCGATGCCCGCATTCTCCACC	0	0	0	0
tsrna-18800	TAGCGGGATCGATGCCCGCATCCTCCACC	0	0.07808	0	0
tsrna-18798	TAGCGCGTTCGGCTGTTAACCGA	0.14283243	1.249282	0.20954	0
tsrna-18797	TAGCGCGTTCGGCTGTTAACCG	0.49991351	1.249282	0.20954	0
tsrna-18796	TAGCGCGTTCGGCTGTTAACCA	0.64274595	2.967044	0.768315	0
tsrna-18795	TAGCGCGTTCGGCTGTTAACC	0.07141622	0.15616	0	0.111027
tsrna-18793	TAGCGCGTTCGGCTGTTAA	0	0	0	0
tsrna-18791	TAGCGCGTTCGGCTGTT	0	0	0	0
tsrna-18788	TAGCGCGTCTGACTCC	0	0	0	0.111027
tsrna-18787	TAGCGCATTGGACTTCTAATTCAAAGGTTGTGGGT	3.14231352	6.79297	1.955711	1.887461
tsrna-18786	TAGCGCATTGGACTTCTAATTCAAAGGTTCCGGG	0.21424865	0.23424	0.139694	0.111027
tsrna-18785	TAGCATTAACCTTTTAAGTTAAAGATTAAGAGAACC	0	0	0.139694	0
tsrna-18780	TAGCACTCTGGACTTTGAATCCAGCGATCCGAGTT	0.21424865	0.624641	0.069847	0
tsrna-18779	TAGCACTCTGGACTTTGAATCCAGC	0.21424865	0.390401	0.069847	0.111027
tsrna-18778	TAGCACTCTGGACTTTGAATCCA	0.07141622	0.390401	0.20954	0
tsrna-18777	TAGCACTCTGGACTTTGAATCC	0.21424865	0.468481	0.069847	0.111027
tsrna-18776	TAGCACTCTGGACTTTGAATC	0.14283243	0.15616	0.069847	0.111027

tsrna-18770	TAGCACTCTGGACTCTGAATCCAGCGATCCGAGTTCA	0	0.15616	0.20954	0
tsrna-18769	TAGCACTCTGGACTCTGAATCCAGCGATCCGAGTTC	0.07141622	0.07808	0	0
tsrna-18768	TAGCACTCTGGACTCTGAATCCAGCGATCCGAGTT	0.14283243	0.31232	0.069847	0
tsrna-18767	TAGCACTCTGGACTCTGAATCCAGCGATCCGAGT	0	0.546561	0	0
tsrna-18766	TAGCACTCTGGACTCTGAATCCAGCGATCCGAG	0.14283243	0.390401	0	0
tsrna-18765	TAGCACTCTGGACTCTGAATCCAGCGATCC	0.07141622	0.31232	0.069847	0.111027
tsrna-18764	TAGCACTCTGGACTCTGAATCCAGCGATC	0.14283243	0.15616	0	0
tsrna-18763	TAGCACTCTGGACTCTGAATCCAGCGAT	0	0.546561	0.069847	0
tsrna-18762	TAGCACTCTGGACTCTGAATCCAGCGA	0.21424865	0.31232	0.069847	0
tsrna-18761	TAGCACTCTGGACTCTGAATCCAGCG	0.07141622	0	0.069847	0
tsrna-18760	TAGCACTCTGGACTCTGAATCCAGC	0.21424865	0.23424	0	0.111027
tsrna-18759	TAGCACTCTGGACTCTGAATCCAG	0	0.390401	0.139694	0
tsrna-18758	TAGCACTCTGGACTCTGAATCCA	0	0.07808	0	0
tsrna-18757	TAGCACTCTGGACTCTGAATCC	0	0.15616	0	0
tsrna-18756	TAGCACTCTGGACTCTGAATC	0	0.07808	0	0
tsrna-18750	TAGATTGAAGCCAGTTGATTAGGGTGCTTAGCTGTTAACT	2.99948109	0.31232	2.793872	0.111027
tsrna-18749	TAGATTGAAGCCAGTTGATTAGGGTGCTTAGCTGTT	1.2854919	0.15616	2.095404	0.222054
tsrna-18748	TAGATTGAAGCCAGTTGATTAGGGTGCTTAGCT	0.35708108	0.15616	0.558774	0
tsrna-18747	TAGATTGAAGCCAGTTGATTAGGGTGCTTA	0.07141622	0	0.20954	0
tsrna-18746	TAGATTGAAGCCAGTTGATTAGGGTGCTT	0.07141622	0.07808	0.139694	0.111027
tsrna-18745	TAGATTGAAGCCAGTTGATTAGGGTGCT	0.07141622	0	0.069847	0
tsrna-18744	TAGATTGAAGCCAGTTGATTAGGGTGC	0	0.07808	0.069847	0
tsrna-18743	TAGATTGAAGCCAGTTGATTAGGGTG	0	0.07808	0	0
tsrna-18742	TAGATTGAAGCCAGTTGATTAGGGT	0	0.07808	0.139694	0
tsrna-18741	TAGATTGAAGCCAGTTGATTAGGG	0	0.15616	0	0
tsrna-18740	TAGATTGAAGCCAGTTGATTAGG	0	0	0	0
tsrna-18739	TAGATTGAAGCCAGTTGATTAG	0.07141622	0.07808	0.139694	0
tsrna-18738	TAGATTGAAGCCAGTTGATTA	0	0	0.139694	0
tsrna-18737	TAGATTGAAGCCAGTTGATT	0	0.07808	0	0.111027
tsrna-18736	TAGATTGAAGCCAGTTGAT	0.14283243	0.07808	0	0.111027
tsrna-18735	TAGATTGAAGCCAGTTGA	0.07141622	0	0	0.111027
tsrna-18734	TAGATTGAAGCCAGTTG	0	0.07808	0	0.111027
tsrna-18733	TAGATTGAAGCCAGTT	0.07141622	0	0	0
tsrna-18721	TAGAGCATGGGACTCTTAATCCCAGGGTCGTGGGTT	0.14283243	0.390401	0	0.111027
tsrna-18720	TAGAGCATGGGACTCTTAATCCCAGGGTCGTGGGT	0.14283243	0	0.139694	0.111027
tsrna-18719	TAGAGCATGGGACTCTTAATCCCAGGGTCGTGG	0	0.23424	0	0
tsrna-18718	TAGAGCATGGGACTCTTAATCCCAGGGTCG	0	0.07808	0.069847	0
tsrna-18717	TAGAGCATGGGACTCTTAATCCC	0.07141622	0.23424	0.069847	0
tsrna-18716	TAGAGCATGGGACTCTTAATCC	0.14283243	0.07808	0	0.111027
tsrna-18715	TAGAGCATGGGACTCTTAATC	0	0	0	0
tsrna-18713	TAGAGCATGAGACTCTTAATCTCAGGGTCGTGGGTT	0.4284973	1.717762	0.419081	0.77719
tsrna-18712	TAGAGCATGAGACTCTTAATCTCAGGGTCGTGGGT	0.64274595	1.561602	0.069847	0.77719
tsrna-18711	TAGAGCATGAGACTCTTAATCT	0.14283243	0.468481	0	0.222054
tsrna-18710	TAGAGCATCAGACTTTTAATCTGAGGGTCCAGGGT	1.07124325	3.747845	0.838162	0.555136
tsrna-18708	TAGACGGGCTCACATCACCCCATAAACACCA	1.2854919	0.468481	1.466783	0.999244
tsrna-18707	TAGACGGGCTCACATCACCCCATAAACACC	0.49991351	0.15616	0.768315	0.111027
tsrna-18706	TAGACGGGCTCACATCACCCCATAAACA	0.07141622	0.07808	0.20954	0
tsrna-18705	TAGACGGGCTCACATCACCCCATA	0.07141622	0.07808	0.20954	0.111027
tsrna-18704	TAGACGGGCTCACATCACCCCAT	0.07141622	0	0.139694	0
tsrna-18703	TAGACGGGCTCACATCACCCCA	0	0	0.069847	0
tsrna-18702	TAGACGGGCTCACATCACCCC	0.14283243	0.15616	0.139694	0
tsrna-18701	TAGACGGGCTCACATCACCC	0	0	0	0.111027
tsrna-18700	TAGACGGGCTCACATCACC	0	0	0.349234	0
tsrna-18699	TAGACGGGCTCACATCAC	0.14283243	0.15616	0.069847	0
tsrna-18698	TAGACGGGCTCACATCA	0.07141622	0	0.139694	0
tsrna-18697	TAGACGGGCTCACATC	0.07141622	0	0.069847	0
tsrna-18696	TAGAATTCTCGCCTGCCACGCGGGAGGCCC	2.07107028	2.888964	1.885864	0.999244
tsrna-18695	TAGAATTCTCGCCTGCCACGCGGG	3.64222704	3.357445	1.047702	1.998488
tsrna-18694	TAGAATTCTCGCCTGCCACGCGG	1.99965406	3.123204	1.327089	1.332326
tsrna-18693	TAGAATTCTCGCCTGCCACGCG	2.92806487	3.825925	1.466783	1.443353
tsrna-18692	TAGAATTCTCGCCTGCCACGC	2.57098379	2.264323	0.977855	0.77719
tsrna-18691	TAGAATTCTCGCCTGCCACG	2.35673514	2.498564	1.466783	0.666163
tsrna-18690	TAGAATTCTCGCCTGCCAC	0.21424865	0.624641	0	0.333081
tsrna-18689	TAGAATTCTCGCCTGCCA	0.14283243	0.15616	0.069847	0.111027

tsrna-18688	TAGAATTCTCGCCTGCC	0.21424865	0.07808	0.069847	0.111027
tsrna-18687	TAGAATTCTCGCCTGC	0.07141622	0.07808	0.139694	0
tsrna-18686	TAGAATTCTCGCCTCCCACGC	0.14283243	0	0	0.111027
tsrna-18685	TAGAATTCTCGCCTCCCACG	0.14283243	0.07808	0.069847	0
tsrna-18684	TAGAATTCTCGCCTCCC	0	0	0.069847	0.111027
tsrna-18683	TAGAATTCTCGCCTCC	0.07141622	0.07808	0.069847	0
tsrna-18682	TAGAATCTTAGCTTTGGGTGCTAATGGTGGAGTTAA	0.07141622	0.15616	0.069847	0.111027
tsrna-18681	TAGAATCTTAGCTTTGGGTGCTAATGGTGG	0.07141622	0.07808	0	0
tsrna-18679	TAGAATCTTAGCTTTGGGTGC	0	0	0	0
tsrna-18677	TAGAATCTTAGCTTTGGGT	0.07141622	0	0.069847	0
tsrna-18676	TAGAATCTTAGCTTTGG	0.07141622	0	0	0
tsrna-18675	TAGAATCTTAGCTTTG	0	0	0	0
tsrna-18674	TAGAAATCCATTGGGGTTTC	0	0.546561	0	0
tsrna-18673	TACTTTGATAGAGTAAATAATAGGAGCT	0	0	0.069847	0
tsrna-18672	TACTTATAATGCCGAGGTTGTG	0.14283243	0.31232	0.20954	0.111027
tsrna-18669	TACTCTGCGTTGTGGCCGCAGC	0	0.15616	0	0
tsrna-18668	TACTCTGCGTTGTGGCCGCAG	0	0	0.069847	0
tsrna-18661	TACTCGTTAGTATAGTGGT	0	0	0	0.111027
tsrna-18658	TACGACCCCTTATTTACCCCA	0	0	0	0
tsrna-18653	TACCTCCTCAAAGCAATACACTGAAAATGT	0.4284973	0.07808	0.349234	0.222054
tsrna-18652	TACCTCCTCAAAGCAATACACTGAA	0.14283243	0.390401	0.349234	0.222054
tsrna-18651	TACCTCCTCAAAGCAATACACTGA	0.07141622	0.23424	0.069847	0
tsrna-18650	TACCTCCTCAAAGCAATACACTG	0	0.23424	0	0.111027
tsrna-18649	TACCTCCTCAAAGCAATACACT	0.21424865	0	0.069847	0
tsrna-18648	TACCTCCTCAAAGCAATACAC	0	0	0	0
tsrna-18647	TACCTCCTCAAAGCAATACA	0	0	0	0
tsrna-18639	TACCGTTAACTTCCAATTA	0	0	0.069847	0
tsrna-18638	TACCCTTCCCGTACTACCA	0	0	0	0
tsrna-18635	TACCCCGAAAATGTTGGTTATACCCTTCCCGTACTA	0	0	0.20954	0
tsrna-18634	TACCCCGAAAATGTTGGTTATACCCTTCCC	0.07141622	0	0	0
tsrna-18630	TACAGTCAGAGGTTCA	0.07141622	0	0	0
tsrna-18629	TACAGACCAAGAGCCTTCAAAG	0.4284973	0	0	0.111027
tsrna-18628	TACAGACCAAGAGCCTTCA	0	0.15616	0	0
tsrna-18627	TACAGACCAAGAGCCTTC	0	0.07808	0	0
tsrna-18626	TACACTTAGGAGATTTCAACTTAACTTGACCGCTCTGACCA	6.57029191	3.982086	5.517898	1.55438
tsrna-18625	TACACTTAGGAGATTTCAACTTAACTTGACCGCTCTGACC	1.21407568	0.780801	0.977855	0.111027
tsrna-18624	TACACTTAGGAGATTTCAACTTAACTTGACCGCTCTGA	0.35708108	0.31232	0.069847	0
tsrna-18623	TACACTTAGGAGATTTCAACTTAACTTGACCGCTCTG	0	0	0	0.111027
tsrna-18622	TACACTTAGGAGATTTCAACTTAACTTGACCGCTCT	0.07141622	0.15616	0	0
tsrna-18621	TACACTTAGGAGATTTCAACTTAACTTGACCGCTC	0	0	0.20954	0.111027
tsrna-18620	TACACTTAGGAGATTTCAACTTAACTTGACCGCT	0.07141622	0	0.069847	0
tsrna-18619	TACACTTAGGAGATTTCAACTTAACTTGACC	0	0	0	0
tsrna-18618	TACACTTAGGAGATTTCAACTTAACTTGAC	0.07141622	0	0.139694	0
tsrna-18617	TACACTTAGGAGATTTCAACTTAACTTGA	0	0	0	0
tsrna-18615	TACACTTAGGAGATTTCAACTTAACT	0	0	0.139694	0
tsrna-18607	TACACTGAAAATGTTTAGACGGGCTCAC	2.14248649	1.327362	0.558774	0.222054
tsrna-18606	TACACTGAAAATGTTTAGAC	0	0	0	0
tsrna-18604	TACACCAGTCTTGTAAACCGG	0.14283243	0.07808	0	0
tsrna-18603	TACAACGATGGTTTTTCATATCATTGGTCGTGGTTGTAGTC	0.14283243	0.07808	0.20954	0.333081
tsrna-18602	TACAACGATGGTTTTTCATATCATTGGTCGTGGTTGTAGT	0.07141622	0.15616	0.069847	0
tsrna-18601	TACAACGATGGTTTTTCATATCATTGGTCG	0.07141622	0	0	0
tsrna-18597	TAATTCAAAGGTTGTGGGTTCGAGTCCCACCAGAGTCGCCA	0.14283243	1.717762	0.069847	0.111027
tsrna-18596	TAATTCAAAGGTTGTGGGTTCGAATCCCACCAGAGTCGCCA	0.71416216	1.483522	0.20954	0.222054
tsrna-18594	TAATTCAAAGGTTCCGGGTTCGAGTCCCGGCGGAGTCGCCA	0.07141622	0.31232	0	0
tsrna-18593	TAATTCAAAGGTTCCGGGTTCGAGTCCCGGCGGAGTCGCC	0.07141622	0.23424	0	0
tsrna-18592	TAATTCAAAGGTTCCGGGTTCGAGTCCCGG	0	0.07808	0	0
tsrna-18591	TAATTCAAAGGTTCCGGGTT	0	0.07808	0	0
tsrna-18590	TAATTAAAGTGGCTGATTTGCGTTCA	0	0.07808	0	0
tsrna-18589	TAATTAAAGTGGCTGATTTGCGTTC	0	0	0	0
tsrna-18588	TAATTAAAGTGGCTGATTTGCG	0	0	0	0
tsrna-18587	TAATTAAAGTGGCTGATTTGC	0	0	0	0
tsrna-18586	TAATTAAAGTGGCTGATTTG	0	0.07808	0	0
tsrna-18584	TAATGGTTAGCACTCTGGGCT	0	0	0	0
tsrna-18583	TAATGGTTAGCACTCTGGGC	0.07141622	0	0	0

tsrna-18582	TAATGGTTAGCACTCTGGACTTTGAATCC	0.21424865	0.624641	0.139694	0.111027
tsrna-18581	TAATGGTTAGCACTCTGGACTTTGAATC	0.28566487	0.07808	0.069847	0
tsrna-18580	TAATGGTTAGCACTCTGGACTTTGAAT	0	0	0	0.111027
tsrna-18579	TAATGGTTAGCACTCTGGACTTTGAA	0.07141622	0.07808	0	0
tsrna-18578	TAATGGTTAGCACTCTGGACTTTGA	0	0	0	0.111027
tsrna-18577	TAATGGTTAGCACTCTGGACTTTG	0	0	0	0
tsrna-18576	TAATGGTTAGCACTCTGGACTTT	0.07141622	0	0	0
tsrna-18574	TAATGGTTAGCACTCTGGACTCTGAATCCAGCGATCCGAGTT	0.28566487	0.468481	0.139694	0.222054
tsrna-18573	TAATGGTTAGCACTCTGGACTCTGAATCCAGCGATC	0.57132973	0.468481	0.20954	0.222054
tsrna-18572	TAATGGTTAGCACTCTGGACTCTGAATCC	0	0.390401	0.069847	0.333081
tsrna-18571	TAATGGTTAGCACTCTGGACTCTGAATC	0.07141622	0.07808	0.20954	0
tsrna-18570	TAATGGTTAGCACTCTGGACTCTGAAT	0	0.07808	0	0
tsrna-18569	TAATGGTTAGCACTCTGGACTCTGAA	0.14283243	0	0	0
tsrna-18567	TAATGGTTAGCACTCTGGACTCTG	0	0	0	0.111027
tsrna-18566	TAATGGTTAGCACTCTGGACTCT	0	0.07808	0	0.111027
tsrna-18565	TAATGGTTAGCACTCTGGACTC	0.07141622	0.07808	0	0
tsrna-18564	TAATGGTTAGCACTCTGGACT	0.07141622	0	0	0
tsrna-18562	TAATGGTTAGCACTCTGGA	0	0	0	0
tsrna-18558	TAATGGTGGAGTTAAAGACTTTTTCTCTGACCA	2.07107028	4.919047	1.396936	2.33157
tsrna-18557	TAATGGTGGAGTTAAAGACTTTTTCTCTGACC	0.4284973	1.873923	0.279387	0.222054
tsrna-18556	TAATGGTGGAGTTAAAGACTTTTTCTCTGAC	0.35708108	1.327362	0.20954	0.111027
tsrna-18555	TAATGGTGGAGTTAAAGACTTTTTCTCTGA	0.71416216	1.015041	0	0
tsrna-18554	TAATGGTGGAGTTAAAGACTTTTTCTCTG	0.57132973	1.483522	0.20954	0.111027
tsrna-18553	TAATGGTGGAGTTAAAGACTTTTTC	0.07141622	1.015041	0.139694	0.222054
tsrna-18552	TAATGGTGGAGTTAAAGACTTTTT	0	0.780801	0.069847	0
tsrna-18551	TAATGGTGGAGTTAAAGACTTTT	0.21424865	0.546561	0.279387	0
tsrna-18550	TAATGGTGGAGTTAAAGACTTT	0.14283243	0.546561	0.279387	0
tsrna-18549	TAATGGTGGAGTTAAAGACTT	0.14283243	0.390401	0.139694	0
tsrna-18548	TAATGGTGGAGTTAAAGACT	0.14283243	0.15616	0.069847	0
tsrna-18547	TAATGGTGGAGTTAAAGAC	0	0.23424	0.069847	0
tsrna-18546	TAATGGTGGAGTTAAAGA	0.21424865	0.15616	0.069847	0
tsrna-18545	TAATGGTGGAGTTAAAG	0	0.15616	0.069847	0.111027
tsrna-18544	TAATGGTGGAGTTAAA	0	0.07808	0	0
tsrna-18543	TAATGGTGAGCACTTTGGACTCTGA	0	0.15616	0	0
tsrna-18542	TAATGGTGAGCACTTTGGACTCTG	0.07141622	0.07808	0	0
tsrna-18540	TAATGGTGAGCACTCTGGACTCTGAATCC	0.14283243	0.390401	0	0.111027
tsrna-18537	TAATGGTGAGCACTCTGGACTCT	0	0	0	0
tsrna-18534	TAATGGTGAGCACTCTGGAC	0	0	0.069847	0
tsrna-18530	TAATGGTCAGCACTCTGGACTCTGAA	0	0	0	0
tsrna-18522	TAATGGTAAGCACTCTGGACTCTGAATCC	0	0.07808	0.069847	0
tsrna-18512	TAATGGATAAGGCGTCTGATTCC	0	0.23424	0	0
tsrna-18510	TAATGGATAAGGCGTCTGACTTCGGATC	0	0.15616	0.069847	0
tsrna-18509	TAATGGATAAGGCGTCTGACTTCGGAT	0.07141622	0.07808	0	0.111027
tsrna-18508	TAATGGATAAGGCGTCTGACTTCGGA	0	0	0	0.111027
tsrna-18507	TAATGGATAAGGCGTCTGACTTCG	0	0.07808	0.069847	0
tsrna-18506	TAATGGATAAGGCGTCTGACTTC	0.07141622	0	0	0.111027
tsrna-18504	TAATGGATAAGGCGTCTGACT	0	0	0.069847	0
tsrna-18503	TAATGGATAAGGCGTCTGAC	0.07141622	0	0	0
tsrna-18501	TAATGGATAAGGCGTCTG	0	0.07808	0	0
tsrna-18500	TAATGGATAAGGCGTCT	0.07141622	0	0	0
tsrna-18499	TAATGGATAAGGCATTGG	0.21424865	0.15616	0.139694	0
tsrna-18498	TAATGGATAAGGCATTG	0.14283243	0.07808	0.069847	0
tsrna-18497	TAATGGATAAGGCATT	0	0	0.069847	0
tsrna-18496	TAATGGATAAGGCATCAGCCTCC	0.14283243	0.15616	0.20954	0.111027
tsrna-18495	TAATGGATAAGGCATCAGCCTC	0.57132973	0.23424	0.349234	0
tsrna-18494	TAATGGATAAGGCATCAGCCT	0.07141622	0.15616	0.139694	0
tsrna-18493	TAATGGATAAGGCATCAGCC	0.28566487	0.07808	0.139694	0.111027
tsrna-18492	TAATGGATAAGGCATCAGC	0.4284973	0.15616	0.069847	0.111027
tsrna-18491	TAATGGATAAGGCATCAG	0.14283243	0.15616	0.069847	0
tsrna-18490	TAATGGATAAGGCATCA	0.4284973	0.15616	0.139694	0.111027
tsrna-18489	TAATGGATAAGGCATC	0	0.07808	0.20954	0.222054
tsrna-18488	TTCACCGCCGCGGCCCGGGTT	0	0	0	0
tsrna-18483	TTCACCCAGGCGGCCCGGGTTCGACTCCCGGTGTGGGAACC	5.35621623	26.39108	2.584332	2.553624
tsrna-18478	TTCAATTCTCGCTGGGGCCTCCA	2.71381622	3.669765	1.606477	2.109516

tsrna-18476	TTCAATTCCCCGACGGGGAGCCA	0	0.15616	0	0
tsrna-18474	TTCAATGGTAGAATTCTCGCCT	0.07141622	0.390401	0.069847	0
tsrna-18473	TTCAATCCCGGGTTTCGGCACCA	2.64240001	7.26145	0.908008	1.332326
tsrna-18472	TTCAATCCCCGGCATCTCCACCA	9.06985949	10.38465	4.120962	5.44033
tsrna-18469	TTCAATCCCCGGCACCTCCACCA	17.9254703	23.18979	8.032383	4.108004
tsrna-18468	TTCAATCCCCGGCACCTCCACC	0.49991351	1.717762	0.558774	0.555136
tsrna-18467	TTCAATCCCCGGCACCTCCAC	0	0	0	0.111027
tsrna-18465	TTCAATCCCCAGCACCTCCACCA	0.21424865	1.015041	0.139694	0.666163
tsrna-18464	TTCAAGTCTCGGTGGAACCTCCA	4.85630272	8.276492	2.025557	1.998488
tsrna-18463	TTCAAGTCCCTGTTCGGGCGCCA	0.71416216	0.936961	0.20954	0.333081
tsrna-18457	TTCAAGTCCCTGTTCAGGCGCCA	0.07141622	0.23424	0	0
tsrna-18455	TTCAAGTCCCTGTCCAGGCGCCA	0	0	0	0
tsrna-18454	TTCAAGTCACGTCGGGGTCACCA	0.35708108	0.546561	0.069847	0.111027
tsrna-18453	TTCAAGTCACGTCGGGGTCACC	0	0	0.139694	0
tsrna-18452	TTCAAGTCACGTCGGGGTCAC	0.07141622	0	0	0
tsrna-18446	TTCAAGCCTCACCTGGAGCACCA	0	0.07808	0.069847	0
tsrna-18445	TTCAACTTAACTTGACCGCTCTGACCA	1.2854919	1.639682	2.304945	0.555136
tsrna-18444	TTCAACTTAACTTGACCGCTCTGACC	0	0.31232	0.069847	0
tsrna-18443	TTCAACTTAACTTGACCGCTCTGAC	0	0	0	0
tsrna-18442	TTCAACTTAACTTGACCGCTCTGA	0	0	0	0.111027
tsrna-18441	TTCAACTTAACTTGACCGCTCTG	0.07141622	0	0	0
tsrna-18440	TTCAACTTAACTTGACCGCTCT	0	0	0	0
tsrna-18439	TTCAACTTAACTTGACCGCTC	0	0	0	0
tsrna-18437	TTCAACTTAACTTGACCGC	0	0	0	0
tsrna-18433	TTCAAATCTGGGTGCCCCCTCCA	0	0	0	0
tsrna-18432	TTCAAATCTCGGTGGGACCTCCA	5.07055137	8.198412	2.235098	2.109516
tsrna-18431	TTCAAATCTCGGTGGGACCTCC	0.21424865	0.702721	0.139694	0.222054
tsrna-18429	TTCAAATCTCGGTGGGACCT	0	0	0	0
tsrna-18425	TTCAAATCTCGGTGGAACCTCCA	11.3551784	14.83522	3.981268	3.219787
tsrna-18424	TTCAAATCTCGGTGGAACCTCC	0.28566487	0.546561	0.20954	0
tsrna-18423	TTCAAATCTCGGTGGAACCTC	0	0.15616	0.069847	0
tsrna-18422	TTCAAATCTCGGTGGAACCT	0	0.23424	0.069847	0.222054
tsrna-18421	TTCAAATCTCGGTGGAACC	0	0.15616	0	0
tsrna-18418	TTCAAATCTCGCTGGGGCCTCCA	2.21390271	4.138246	1.74617	1.665407
tsrna-18417	TTCAAATCTCGCTGGGGCCTCC	0.28566487	0.468481	0.139694	0
tsrna-18416	TTCAAATCTCGCTGGGGCCTC	0	0.07808	0	0
tsrna-18414	TTCAAATCCGGGTGCCCCCTCCA	12.1407568	12.64898	4.120962	2.664651
tsrna-18413	TTCAAATCCGGGTGCCCCCTCC	0.28566487	0.546561	0.069847	0.222054
tsrna-18412	TTCAAATCCGGGTGCCCCCTC	0.14283243	0.07808	0	0
tsrna-18409	TTCAAATCCCGGACGAGCCCCCA	10.4981838	12.1805	4.120962	4.219031
tsrna-18408	TTCAAATCCCGGACGAGCCCCC	0.49991351	0.546561	0.20954	0
tsrna-18407	TTCAAATCCCGGACGAGCCCC	0.07141622	0.15616	0.069847	0
tsrna-18406	TTCAAATCCCGGACGAGCCC	0.14283243	0	0.069847	0
tsrna-18405	TTCAAATCCCGGACGAGCC	0	0.07808	0	0
tsrna-18401	TTCAAATCCAGGTGCCCCCTCCA	0.07141622	0.15616	0	0.111027
tsrna-18400	TTCAAATCACGTCGGGGTCACCA	0.28566487	0.624641	0.908008	0.111027
tsrna-18399	TTCAAATCACGTCGGGGTCACC	0	0.07808	0	0.111027
tsrna-18398	TTCAAATCACGTCGGGGTCAC	0	0	0	0
tsrna-18393	TTCAAAGGTTGTGGGTTCGAGTCCCACCAGAGTCGCCA	0.07141622	0.702721	0	0
tsrna-18392	TTCAAAGGTTGTGGGTTCGAGTCCCACCAGAGTCGCC	0.07141622	0.23424	0	0.111027
tsrna-18391	TTCAAAGGTTGTGGGTTCGAGTCCCACCAGAGTCGC	0.07141622	0.15616	0	0
tsrna-18390	TTCAAAGGTTGTGGGTTCGAGTCCCACCAG	0.07141622	0	0	0
tsrna-18389	TTCAAAGGTTGTGGGTTCGAGTCCCACCA	0	0.07808	0	0
tsrna-18388	TTCAAAGGTTGTGGGTTCGAATCCCACCAGAGTCGCCA	0.21424865	0.858881	0	0.111027
tsrna-18387	TTCAAAGGTTGTGGGTTCGAATCCCACCAGAGTCGCC	0.07141622	0.468481	0.069847	0.111027
tsrna-18385	TTCAAAGGTTGTGGGTTCGAATCCCACCA	0.14283243	0.23424	0	0
tsrna-18384	TTCAAAGGTTCCGGGTTCGAGTCCCGGCGGAGTCGCCA	0	0.546561	0	0
tsrna-18383	TTCAAAGGTTCCGGGTTCGAGTCCCGGCGGAGTCGCC	0	0	0	0
tsrna-18381	TTCAAAGGTTCCGGGTTCGAGTCCCGGCGGAGTC	0	0	0	0
tsrna-18379	TTCAAAGGTTCCGGGTTCGAGTCCCGGCG	0.07141622	0	0	0
tsrna-18378	TTCAAAGGTTCCGGGTTCG	0	0	0	0
tsrna-18373	TTCAAACCTGCCGGGGCTTCCA	0	0.15616	0.069847	0
tsrna-18372	TTCAAACCTGCCGGGGCTTCC	0	0	0	0.111027
tsrna-18365	TTATGTAGCTTACCTCC	0.07141622	0	0	0

tsrna-18356	TTATCACGTTCGCCTCACACGC	0	0.07808	0	0
tsrna-18352	TTATCACGTCTGCTTTACACG	0	0	0.069847	0
tsrna-18349	TTATACCCTTCCCGTACTACCA	0.07141622	0.23424	0	0
tsrna-18348	TTATACCCTTCCCGTACTACC	0	0.07808	0	0
tsrna-18345	TTATAATGCCGAGGTTGTGAGTTC	0.07141622	1.249282	0.558774	0.333081
tsrna-18344	TTATAATGCCGAGGTTGTGAGTT	0.28566487	0.624641	0.20954	0
tsrna-18343	TTATAATGCCGAGGTTGTGAGT	0.49991351	0.702721	0.069847	0.111027
tsrna-18342	TTATAATGCCGAGGTTGTGAG	0.28566487	0.702721	0	0
tsrna-18341	TTATAATGCCGAGGTTGTGA	0.07141622	0.390401	0	0.111027
tsrna-18340	TTAGTGGTAGAATTCTCGCCT	0.14283243	0.546561	0.069847	0.111027
tsrna-18339	TTAGTGGTAGAATTCTCGCC	0.14283243	0.31232	0	0
tsrna-18338	TTAGTGGTAGAATTCTCGC	0.07141622	0.07808	0	0.111027
tsrna-18337	TTAGTGGTAGAATTCTCG	0	0	0	0
tsrna-18334	TTAGTATAGTGGTGAGTATCCCCG	0.07141622	0	0	0.111027
tsrna-18333	TTAGTACTCTGCGTTGTGGCCGCAGCAACCTCGGT	1.21407568	0.468481	0.419081	0.333081
tsrna-18332	TTAGTACTCTGCGTTGTGGCCGCAGCAACC	0	0.15616	0	0
tsrna-18331	TTAGTACTCTGCGTTGTGGCCGCAGC	0.28566487	0.15616	0	0
tsrna-18330	TTAGTACTCTGCGTTGTGGCCGCA	0.14283243	0	0	0
tsrna-18329	TTAGTACTCTGCGTTGTGGCCGC	0	0	0.139694	0
tsrna-18328	TTAGTACTCTGCGTTGTGGCCG	0.07141622	0.390401	0.069847	0
tsrna-18327	TTAGTACTCTGCGTTGTGGCC	0.14283243	0	0	0
tsrna-18326	TTAGTACTCTGCGTTGTGGC	0.07141622	0.15616	0	0
tsrna-18325	TTAGTACTCTGCGTTGTGG	0	0.07808	0	0
tsrna-18324	TTAGTACTCTGCGTTGTG	0.07141622	0.15616	0.139694	0
tsrna-18323	TTAGTACTCTGCGTTGT	0	0	0	0
tsrna-18322	TTAGTACTCTGCGTTG	0.07141622	0	0	0
tsrna-18320	TTAGGTCGCTGGTTCGATTCCGGCTCGAAGGACC	0.8569946	2.420483	0.419081	0.555136
tsrna-18319	TTAGGTCGCTGGTTCGATTCCGGCTCGAAG	0	0.07808	0.069847	0
tsrna-18318	TTAGGTCGCTGGTTCGATTCCGGC	0.07141622	0	0	0
tsrna-18316	TTAGGGTGCTTAGCTGTTAACTAAGTGTTTGTGGGT	0.14283243	0.07808	0	0.111027
tsrna-18315	TTAGGGTGCTTAGCTGTTAACTA	0.07141622	0	0.139694	0
tsrna-18314	TTAGGGTGCTTAGCTGTTAACT	0	0.07808	0	0
tsrna-18312	TTAGGGTGCGAGAGGTCCCGGGTT	0	0.546561	0.139694	0.333081
tsrna-18311	TTAGGGTGCGAGAGGTCCCGGGT	0	0.546561	0.069847	0.111027
tsrna-18310	TTAGGGTGCGAGAGGTCCCGG	0	0.31232	0	0
tsrna-18306	TTAGGCTCCAGTCTCTTCGG	0	0.07808	0	0
tsrna-18303	TTAGGCCTCTTTTTACCACCA	0	0.15616	0.069847	0.111027
tsrna-18298	TTAGGCCCCAAAAATTTTGGTGCAACTCCAAATAAAAGTACC	1.2854919	9.525773	0.628621	0
tsrna-18297	TTAGGATTCGGCGCTCTCACCGCCGCGGCCCGGGTTC	0.49991351	1.171202	0.349234	0.222054
tsrna-18296	TTAGGATTCGGCGCTCTCACCGCCGCGGCCCGGGTT	0.21424865	1.171202	0.279387	0.111027
tsrna-18295	TTAGGATTCGGCGCTCTCACCGCCGCGGCCCGGG	0.07141622	0.546561	0.20954	0.333081
tsrna-18294	TTAGGATTCGGCGCTCTCACCGCCGCGGCCC	0.21424865	0.780801	0.20954	0.222054
tsrna-18293	TTAGGATTCGGCGCTCTCACCGCCGCGGCC	0.35708108	1.405442	0.139694	0.222054
tsrna-18292	TTAGGATTCGGCGCTCTCACCGCCGCGGC	0.21424865	1.015041	0.069847	0.111027
tsrna-18291	TTAGGATTCGGCGCTCTCACCGCC	0.4284973	1.015041	0.069847	0.111027
tsrna-18290	TTAGGATTCGGCGCTCTCACCGC	0.14283243	0.31232	0.069847	0
tsrna-18289	TTAGGATTCGGCGCTCTCACCG	0.07141622	0.546561	0.069847	0
tsrna-18288	TTAGGATTCGGCGCTCTCACC	0.14283243	0.23424	0.069847	0
tsrna-18287	TTAGGATTCGGCGCTCTCAC	0	0.15616	0	0
tsrna-18286	TTAGGATTCGGCGCTCTCA	0	0.15616	0.069847	0
tsrna-18285	TTAGGATTCGGCGCTCTC	0	0.23424	0	0
tsrna-18284	TTAGGATTCGGCGCTCT	0	0.07808	0.069847	0
tsrna-18283	TTAGGATTCGGCGCTC	0	0.23424	0	0
tsrna-18282	TTAGGAGATTTCAACTTAACTTGACCGCTCTGACCA	5.2133838	3.825925	6.006825	1.776434
tsrna-18281	TTAGGAGATTTCAACTTAACTTGACCGCTCTGACC	0.57132973	0.702721	0.838162	0.333081
tsrna-18280	TTAGGAGATTTCAACTTAACTTGACCGCTC	0.07141622	0	0.069847	0.111027
tsrna-18279	TTAGGAGATTTCAACTTAACTTGACCGCT	0	0.07808	0	0
tsrna-18276	TTAGGAGATTTCAACTTAACTTGACC	0	0	0	0.111027
tsrna-18275	TTAGGAGATTTCAACTTAACTTGAC	0.07141622	0	0	0
tsrna-18269	TTAGCTTTGGGTGCTAATGGTGGAGTTAAAGACTTTTTCTCTG	0.8569946	1.483522	0.698468	0.111027
tsrna-18268	TTAGCTTTGGGTGCTAATGGTGGAGTTAA	0.14283243	0.23424	0	0
tsrna-18267	TTAGCTTTGGGTGCTAATGGTGGAGTTA	0.07141622	0.468481	0	0
tsrna-18266	TTAGCTTTGGGTGCTAATGGTGGAGTT	0	0.07808	0	0.111027
tsrna-18264	TTAGCTTTGGGTGCTAATGGTGGA	0	0.07808	0	0

tsrna-18263	TTAGCTTTGGGTGCTAATGGTGG	0.07141622	0	0	0
tsrna-18258	TTAGCTGTTAACTAAGTGTTTGTGGGTTTAAGTCCCATTGGTC	0.57132973	0.390401	1.257243	0
tsrna-18257	TTAGCTGTTAACTAAGTGTTTGTGGGTTTAAGTCCC	0.14283243	0.15616	0	0
tsrna-18254	TTAGCGGTAGAGCATTTGACTGC	0.07141622	0	0	0.111027
tsrna-18253	TTAGCGGTAGAGCATTTGACTG	0	0	0	0
tsrna-18252	TTAGCGGTAGAGCATTTGACT	0	0.07808	0	0
tsrna-18251	TTAGCGCGTTCGGCTGTTAACCG	0.21424865	0.858881	0.069847	0
tsrna-18250	TTAGCGCGTTCGGCTGTTAACC	0	0.15616	0	0.111027
tsrna-18244	TTAGCATTAACCTTTTAAGTTAAAGATTAAGAGAACC	0	0	0	0.111027
tsrna-18243	TTAGCATTAACCTTTTAAGTTAAAGATTAAGAGAAC	0.07141622	0.07808	0	0
tsrna-18238	TTAGCACTCTGGGCTTTGAATC	0.21424865	0	0	0
tsrna-18236	TTAGCACTCTGGACTTTGAATCCAGCGATCCGAGTT	0.21424865	0.780801	0.20954	0.222054
tsrna-18235	TTAGCACTCTGGACTTTGAATCCAGCG	0.21424865	0.15616	0.20954	0
tsrna-18234	TTAGCACTCTGGACTTTGAATCCAGC	0.14283243	0.23424	0	0.111027
tsrna-18233	TTAGCACTCTGGACTTTGAATCCA	0.07141622	0.390401	0.069847	0
tsrna-18232	TTAGCACTCTGGACTTTGAATCC	0.14283243	0.780801	0	0.333081
tsrna-18231	TTAGCACTCTGGACTTTGAATC	0.07141622	0.23424	0.139694	0
tsrna-18224	TTAGCACTCTGGACTCTGAATCCAGCGATCCGAGTTCA	0.35708108	0.07808	0	0.111027
tsrna-18223	TTAGCACTCTGGACTCTGAATCCAGCGATCCGAGTTC	0.14283243	0.546561	0.069847	0
tsrna-18222	TTAGCACTCTGGACTCTGAATCCAGCGATCCGAGTT	0.07141622	0.23424	0	0
tsrna-18221	TTAGCACTCTGGACTCTGAATCCAGCGATCCGAGT	0.14283243	0.15616	0.069847	0
tsrna-18220	TTAGCACTCTGGACTCTGAATCCAGCGATCCGAG	0.28566487	0.15616	0	0.111027
tsrna-18219	TTAGCACTCTGGACTCTGAATCCAGCGAT	0.14283243	0.546561	0	0
tsrna-18218	TTAGCACTCTGGACTCTGAATCCAGCGA	0.07141622	0.31232	0.069847	0
tsrna-18217	TTAGCACTCTGGACTCTGAATCCAGCG	0	0.15616	0.069847	0
tsrna-18216	TTAGCACTCTGGACTCTGAATCCAGC	0.07141622	0.546561	0.069847	0
tsrna-18215	TTAGCACTCTGGACTCTGAATCCAG	0.07141622	0.390401	0	0
tsrna-18214	TTAGCACTCTGGACTCTGAATCCA	0.07141622	0.15616	0.069847	0
tsrna-18213	TTAGCACTCTGGACTCTGAATCC	0	0.31232	0	0
tsrna-18205	TTAGAGCCAGACTGCCTGGGTTTGA	0.14283243	0	0.069847	0
tsrna-18204	TTAGAGCCAGACTGCCTGGGTTTG	0.07141622	0.15616	0.349234	0.111027
tsrna-18203	TTAGAGCCAGACTGCCTGGGTTT	0.21424865	0.07808	0.139694	0.111027
tsrna-18202	TTAGAGCCAGACTGCCTGGGTT	0	0	0.069847	0
tsrna-18201	TTAGAGCCAGACTGCCTGGGT	0.07141622	0	0	0
tsrna-18197	TTAGACGGGCTCACATCACCCCATAAACACCA	2.21390271	1.093122	2.304945	2.220543
tsrna-18196	TTAGACGGGCTCACATCACCCCATAAACACC	0.8569946	0.390401	0.488928	0.111027
tsrna-18195	TTAGACGGGCTCACATCACCCCATAAACAC	0.4284973	0.07808	0.698468	0.111027
tsrna-18194	TTAGACGGGCTCACATCACCCCATAAACA	0.49991351	0.07808	0.419081	0.111027
tsrna-18193	TTAGACGGGCTCACATCACCCCATAAAC	0.14283243	0.15616	0.628621	0.111027
tsrna-18192	TTAGACGGGCTCACATCACCCCATAAA	0.07141622	0	0.139694	0
tsrna-18191	TTAGACGGGCTCACATCACCCCATAA	0.07141622	0.07808	0.279387	0
tsrna-18190	TTAGACGGGCTCACATCACCCCATA	0.07141622	0.07808	0.349234	0
tsrna-18189	TTAGACGGGCTCACATCACCCCAT	0.28566487	0	0.20954	0
tsrna-18188	TTAGACGGGCTCACATCACCCCA	0.07141622	0	0.139694	0
tsrna-18187	TTAGACGGGCTCACATCACCCC	0.21424865	0	0.279387	0
tsrna-18186	TTAGACGGGCTCACATCACCC	0	0.07808	0.139694	0.111027
tsrna-18185	TTAGACGGGCTCACATCACC	0.28566487	0.07808	0.20954	0
tsrna-18184	TTAGACGGGCTCACATCAC	0.14283243	0	0.069847	0
tsrna-18183	TTAGACGGGCTCACATCA	0	0.07808	0.139694	0
tsrna-18182	TTAGACGGGCTCACATC	0.07141622	0.15616	0.139694	0
tsrna-18181	TTAGACGGGCTCACAT	0	0	0.20954	0
tsrna-18180	TTAGAATCTTAGCTTTGGGTGCT	0.07141622	0	0	0
tsrna-18179	TTAGAATCTTAGCTTTGGGTGC	0	0	0.069847	0.111027
tsrna-18178	TTAGAATCTTAGCTTTGGGTG	0.07141622	0	0	0
tsrna-18177	TTAGAATCTTAGCTTTGGGT	0	0	0	0.111027
tsrna-18176	TTAGAATCTTAGCTTTGG	0	0	0	0
tsrna-18174	TTACGACCCCTTATTTACCCCA	0	0.07808	0.069847	0.111027
tsrna-18169	TTACCTCCTCAAAGCAATACACTGAAAATG	0.64274595	0.390401	0.349234	0.111027
tsrna-18168	TTACCTCCTCAAAGCAATACACT	0.14283243	0.07808	0.139694	0
tsrna-18167	TTACCTCCTCAAAGCAATACA	0.07141622	0	0	0
tsrna-18166	TTACCTCCTCAAAGCAATAC	0	0.07808	0	0
tsrna-18165	TTACCTCCTCAAAGCAATA	0.07141622	0	0	0
tsrna-18163	TTACAGTCAGAGGTTCAATTC	0.07141622	0.15616	0	0
tsrna-18161	TTACAGTCAGAGGTTC	0	0	0.069847	0

tsrna-18160	TTACACTTAGGAGATTTCAACTTAACTTGACCGCTCTGACCA	6.64170813	4.372486	7.333915	1.887461
tsrna-18159	TTACACTTAGGAGATTTCAACTTAACTTGACCGCTCTGACC	1.42832433	0.936961	1.047702	0
tsrna-18158	TTACACTTAGGAGATTTCAACTTAACTTGACCGCTCTGAC	0.07141622	0.624641	0.349234	0
tsrna-18157	TTACACTTAGGAGATTTCAACTTAACTTGACCGCTCTG	0.14283243	0.23424	0.20954	0
tsrna-18156	TTACACTTAGGAGATTTCAACTTAACTTGACCGCT	0.07141622	0.15616	0	0.111027
tsrna-18155	TTACACTTAGGAGATTTCAACTTAACTTGACC	0.07141622	0	0.139694	0
tsrna-18154	TTACACTTAGGAGATTTCAACTTAACTTGA	0	0	0.069847	0
tsrna-18153	TTACACTTAGGAGATTTCAACTTAACTTG	0	0	0	0
tsrna-18150	TTACACTTAGGAGATTTCAACT	0	0	0.069847	0
tsrna-18145	TTACACGCAGAAGGTCCTGGGTTCGAGCCCCAGTGGAACCA	3.42797839	3.825925	3.143106	2.33157
tsrna-18144	TTACACGCAGAAGGTCCTGGGT	0.07141622	0.23424	0.069847	0
tsrna-18142	TTAATCTGAGGGTCCAGGGTTCATG	0.14283243	0.858881	0.279387	0.111027
tsrna-18141	TTAATCTGAGGGTCCAGGGTTCAT	0.28566487	1.249282	0	0
tsrna-18140	TTAATCTGAGGGTCCAGGGTTCAAGTCCCTGTTCGGGCGCCA	0.92841081	4.216326	0.558774	0.333081
tsrna-18139	TTAATCTGAGGGTCCAGGGTTCAAGT	0.57132973	2.264323	0.20954	0.111027
tsrna-18138	TTAATCTGAGGGTCCAGGGTTCAAG	0.4284973	1.717762	0.069847	0.222054
tsrna-18137	TTAATCTGAGGGTCCAGGGTTCAA	0.35708108	1.327362	0	0.111027
tsrna-18136	TTAATCTGAGGGTCCAGGGTTCA	0.35708108	1.171202	0.139694	0.222054
tsrna-18135	TTAATCTGAGGGTCCAGGGTTC	0.28566487	0.702721	0.349234	0
tsrna-18134	TTAATCTGAGGGTCCAGGGTT	0.28566487	1.015041	0.069847	0
tsrna-18133	TTAATCTGAGGGTCCAGGGT	0.07141622	0.702721	0	0.111027
tsrna-18132	TTAATCTGAGGGTCCAGGG	0.07141622	0.624641	0.139694	0
tsrna-18131	TTAATCTGAGGGTCCAGG	0.28566487	0.468481	0.069847	0.222054
tsrna-18130	TTAATCTGAGGGTCCAG	0.07141622	0.390401	0	0.222054
tsrna-18129	TTAATCTGAGGGTCCA	0.07141622	0.936961	0.069847	0
tsrna-18128	TTAATCTCAGGGTCGTGGGTTCGAGCCCCACGTTGGGCGCCA	1.21407568	21.78435	0.768315	0.555136
tsrna-18127	TTAATCTCAGGGTCGTGGGTTC	0.28566487	0.468481	0.139694	0
tsrna-18126	TTAATCTCAGGGTCGTGGGTT	0.21424865	0.858881	0.419081	0.111027
tsrna-18125	TTAATCTCAGGGTCGTGGGT	0.14283243	0.624641	0.279387	0.222054
tsrna-18124	TTAATCTCAGGGTCGTGGGC	0.28566487	0.624641	0.20954	0.111027
tsrna-18123	TTAATCTCAGGGTCGTGGG	0.35708108	0.546561	0.069847	0.111027
tsrna-18122	TTAATCTCAGGGTCGTGG	0.35708108	0.390401	0	0.333081
tsrna-18121	TTAATCTCAGGGTCGTG	0.4284973	0.624641	0	0.111027
tsrna-18120	TTAATCCCAGGGTCGTGGGT	0	0	0.069847	0
tsrna-18119	TTAATCCCAGGGTCGTGGG	0.07141622	0.07808	0	0
tsrna-18118	TTAATCCCAGGGTCGTGG	0	0.07808	0	0
tsrna-18116	TTAATCCCAGGGTCGT	0	0	0.069847	0
tsrna-18115	TTAAGTTAAAGATTAAGAGAACCAACACCTCTTTACAGTGACC	0.49991351	0.468481	0.628621	0.111027
tsrna-18114	TTAAGTTAAAGATTAAGAGAACCAACACCTCTTTACAGTGACC	0.4284973	0.07808	0.419081	0.222054
tsrna-18113	TTAAGTTAAAGATTAAGAGAACCAACACCTCTTTACAGTGAC	0.14283243	0	0.20954	0
tsrna-18112	TTAAGTTAAAGATTAAGAGAACCAACACCTCTTTACAGTGA	0.07141622	0	0.139694	0.111027
tsrna-18111	TTAAGTTAAAGATTAAGAGAACCAACACCT	0	0	0.069847	0.111027
tsrna-18110	TTAAGTTAAAGATTAAGAGAACCAACACC	0.14283243	0	0	0.333081
tsrna-18109	TTAAGTTAAAGATTAAGAGAACCA	0	0	0	0
tsrna-18108	TTAAGTTAAAGATTAAGAGAACC	0	0	0.069847	0
tsrna-18107	TTAAGTTAAAGATTAAGAGAAC	0.07141622	0	0	0
tsrna-18104	TTAAGTCCCATTGGTCTAGCC	0.07141622	0	0	0
tsrna-18099	TTAAGGCTCCAGTCTCTTCGGG	0	0.07808	0	0
tsrna-18098	TTAAGGCTCCAGTCTCTTCGG	0.07141622	0	0	0
tsrna-18097	TTAAGGCTCCAGTCTCTTCG	0	0	0.069847	0
tsrna-18087	TTAAGAGAACCAACACCTCTTTACAGTGACCA	0.07141622	0	0.069847	0.222054
tsrna-18086	TTAAGAGAACCAACACCTCTTTACAGTGACC	0	0	0	0.111027
tsrna-18085	TTAAGAGAACCAACACCTCTTTACAGTGAC	0	0.07808	0	0
tsrna-18080	TTAACTTGACCGCTCTGACCA	0.4284973	1.327362	0.698468	0.222054
tsrna-18079	TTAACTTGACCGCTCTGACC	0	0.468481	0.069847	0
tsrna-18078	TTAACTTGACCGCTCTGAC	0	0	0	0
tsrna-18077	TTAACTTGACCGCTCTGA	0	0	0.069847	0
tsrna-18074	TTAACTTCCAATTAACTAGTTTTGAC	0	0	0	0
tsrna-18065	TTAACTAAGTGTTTGTGGGTTTAAGTCCCATTGGTCTAGCCA	0.49991351	0.07808	0.139694	0.111027
tsrna-18064	TTAACTAAGTGTTTGTGGGTTTAAGTCCCATTGGTCTAGCC	0.49991351	0.15616	0.20954	0
tsrna-18063	TTAACTAAGTGTTTGTGGGTTTAAGTCCCATTGGTCTAGC	0.14283243	0.07808	0	0
tsrna-18062	TTAACTAAGTGTTTGTGGGTTTAAGTCCCATTGGTCTAG	0.35708108	0.15616	0.279387	0.111027
tsrna-18061	TTAACTAAGTGTTTGTGGGTTTAAGTCCCATTGGTCTA	0.49991351	0.15616	0.069847	0
tsrna-18060	TTAACTAAGTGTTTGTGGGTTTAAGTCCCATTGGTCT	0.21424865	0.23424	0.349234	0.111027

tsrna-18059	TTAACTAAGTGTTTGTGGGTTTAAGTCCCATTGGTC	0.07141622	0.07808	0.419081	0
tsrna-18058	TTAACTAAGTGTTTGTGGGTTTAAGTCCC	0.07141622	0.15616	0	0
tsrna-18057	TTAACTAAGTGTTTGTGGGTTTAAGTCC	0.14283243	0.15616	0	0
tsrna-18056	TTAACTAAGTGTTTGTGGGTTTAAGTC	0.07141622	0	0.069847	0
tsrna-18055	TTAACTAAGTGTTTGTGGGTTTAAGT	0.14283243	0.07808	0.069847	0
tsrna-18054	TTAACTAAGTGTTTGTGGGTTTAAG	0.07141622	0.07808	0.069847	0
tsrna-18053	TTAACTAAGTGTTTGTGGGTTTA	0.07141622	0.07808	0	0
tsrna-18052	TTAACTAAGTGTTTGTGGGTTT	0.07141622	0	0.069847	0
tsrna-18051	TTAACTAAGTGTTTGTGGGTT	0	0	0	0
tsrna-18050	TTAACTAAGTGTTTGTGGGT	0	0.07808	0	0
tsrna-18046	TTAACCTTTTAAGTTAAAGATTAAGAGAACC	0	0	0	0
tsrna-18045	TTAACCTTTTAAGTTAAAGATTAAGAGAAC	0	0	0	0
tsrna-18043	TTAACCGAAAGGTTGGTGGTTCGATCCCACCCAGGGACGCC/	0.28566487	1.093122	0.419081	0.222054
tsrna-18042	TTAACCGAAAGGTTGGTGGTTCGAGCCCACCCAGGGACGCC	1.64257298	9.447693	0.349234	0.222054
tsrna-18041	TTAACCGAAAGGTTGGTGGTTCGAGCCCACCCAGGGACGCC	0.78557838	1.561602	0.419081	0
tsrna-18040	TTAACCGAAAGGTTGGTGGTTCGAGCCCACCCAGGGACG	0.28566487	0.780801	0.20954	0
tsrna-18039	TTAACCGAAAGGTTGGTGGTTCGAGCCCACCCAGGGAC	0	0.468481	0.069847	0
tsrna-18038	TTAACCGAAAGGTTGGTGGTTCGAGCCCACCCAGGG	0.07141622	0.390401	0.069847	0
tsrna-18037	TTAACCGAAAGGTTGGTGGTTCGAGCCCAC	0.14283243	0.23424	0.069847	0
tsrna-18036	TTAACCGAAAGGTTGGTGGTTCGA	0.21424865	0.546561	0.069847	0
tsrna-18035	TTAACCGAAAGGTTGGTGGTTCG	0	0.390401	0	0
tsrna-18034	TTAACCGAAAGGTTGGTGGTTC	0	0	0.069847	0
tsrna-18033	TTAACCGAAAGGTTGGTGGTT	0	0.07808	0	0
tsrna-18032	TTAACCGAAAGGTTGGTGGT	0	0	0.069847	0
tsrna-18031	TTAACCGAAAGGTTGGTGG	0	0	0	0
tsrna-18027	TTAACCAAAACATCAGATTGTGAATCT	0	0	0	0
tsrna-18018	TTAACACAAAGCACCCAACTTACACTTAGG	0	0	0	0
tsrna-18013	TTAAATTATGATAATCATATTTACCAAC	0	0	0.069847	0
tsrna-18011	TTAAATACAGACCAAGAGCC	0.07141622	0	0	0
tsrna-18010	TTAAATACAGACCAAGA	0	0.07808	0	0
tsrna-18008	TTAAAGTGGCTGATTTGCGTTCA	0	0	0	0
tsrna-18007	TTAAAGTGGCTGATTTGCGTTC	0	0.07808	0	0
tsrna-18006	TTAAAGTGGCTGATTTGC	0	0.07808	0	0
tsrna-18004	TTAAAGATTAAGAGAACCAACACCTCTTTACAGTGACCA	0.71416216	0.15616	0.488928	0
tsrna-18003	TTAAAGATTAAGAGAACCAACACCTCTTTACAGTGACC	0.35708108	0	0.139694	0.111027
tsrna-18002	TTAAAGATTAAGAGAACCAACACCTCTTTACAGTGAC	0.4284973	0	0.139694	0
tsrna-18001	TTAAAGATTAAGAGAACCAACACCTCTTT	0	0	0	0
tsrna-17997	TTAAAGATTAAGAGAACCAAC	0	0	0	0
tsrna-17996	TTAAAGATTAAGAGAACCAA	0.07141622	0	0	0
tsrna-17993	TTAAAGACTTTTTCTCTGACCA	1.14265946	1.873923	0.628621	1.332326
tsrna-17992	TTAAAGACTTTTTCTCTGACC	0.21424865	0.858881	0.139694	0.333081
tsrna-17988	TTAAACCCCCTTATTTCTACCA	0	0.07808	0.069847	0
tsrna-17987	TTAAACCCCCTTATTTCTACC	0	0.15616	0	0
tsrna-17985	TTAAACCCCCTTATTTC	0	0.07808	0	0
tsrna-17983	TGTTTGTGGGTTTAAGTCCCATTGGTCTAGCCA	0.14283243	0	0.139694	0
tsrna-17982	TGTTTGTGGGTTTAAGTCCCATTGGTCTAGCC	0.07141622	0	0.069847	0
tsrna-17981	TGTTTGTGGGTTTAAGTCCCATTGGTCTAGC	0.07141622	0	0.069847	0
tsrna-17980	TGTTTGTGGGTTTAAGTCCCATTGGTCTAG	0	0.07808	0	0
tsrna-17979	TGTTTGTGGGTTTAAGTCCCATTGG	0	0.07808	0	0
tsrna-17973	TGTTTAGACGGGCTCACATCACCCCATAAACACC	2.35673514	0.702721	1.396936	0.555136
tsrna-17972	TGTTTAGACGGGCTCACATCACCCCATAAA	1.21407568	0.858881	1.466783	0.555136
tsrna-17971	TGTTTAGACGGGCTCACATCACCCCATAA	1.21407568	0.780801	1.396936	0.444109
tsrna-17970	TGTTTAGACGGGCTCACATCACCCCATA	1.78540541	0.546561	0.908008	0.666163
tsrna-17969	TGTTTAGACGGGCTCACATCACCCCAT	1.35690811	0.624641	1.396936	0.333081
tsrna-17968	TGTTTAGACGGGCTCACATCACCCCA	0.78557838	0.624641	1.047702	0.333081
tsrna-17967	TGTTTAGACGGGCTCACATCACCCC	1.49974054	0.390401	0.908008	0.111027
tsrna-17966	TGTTTAGACGGGCTCACATCACCC	1.07124325	0.468481	1.117549	0.333081
tsrna-17965	TGTTTAGACGGGCTCACATCACC	1.07124325	0.624641	0.977855	0.111027
tsrna-17964	TGTTTAGACGGGCTCACATCAC	0.92841081	0.702721	0.558774	0.111027
tsrna-17963	TGTTTAGACGGGCTCACATCA	0.8569946	0.468481	0.558774	0.333081
tsrna-17962	TGTTTAGACGGGCTCACATC	0.71416216	0.390401	0.908008	0.222054
tsrna-17961	TGTTTAGACGGGCTCACAT	0.71416216	0.702721	0.488928	0.111027
tsrna-17960	TGTTTAGACGGGCTCACA	0.49991351	0.390401	0.488928	0.111027
tsrna-17959	TGTTTAGACGGGCTCAC	0.49991351	0.23424	0.279387	0.222054

tsrna-17958	TGTTTAGACGGGCTCA	0.21424865	0	0.069847	0
tsrna-17957	TGTTGGTTATACCCTTCCCGTACTACCA	0.28566487	0.23424	0.139694	0
tsrna-17956	TGTTGGTTATACCCTTCCCGTACTACC	0	0.07808	0.069847	0
tsrna-17954	TGTTGGTTATACCCTTCCCGTACTA	0.07141622	0	0	0
tsrna-17953	TGTTGGTTATACCCTTCCCGTACT	0.07141622	0	0.069847	0
tsrna-17952	TGTTGGTTATACCCTTCCCGTAC	0	0	0	0
tsrna-17949	TGTTGGTTATACCCTTCCCG	0.07141622	0	0	0
tsrna-17946	TGTTCGAATCACGTCGGGGTCACCA	0.4284973	0.624641	0.279387	0.555136
tsrna-17945	TGTTCGAATCACGTCGGGGTCACC	0	0.23424	0	0
tsrna-17937	TGTTCAAGTCACGTCGGGGTCACCA	0.49991351	0.390401	0.20954	0.222054
tsrna-17936	TGTTCAAGTCACGTCGGGGTCACC	0	0.23424	0	0
tsrna-17934	TGTTCAAGTCACGTCGGGGTCA	0	0.07808	0	0
tsrna-17928	TGTTCAAATCACGTCGGGGTCACCA	0.21424865	0.780801	0.20954	0.555136
tsrna-17926	TGTTCAAATCACGTCGGGGTCAC	0	0	0	0
tsrna-17919	TGTTAACTAAGTGTTTGTGGGTTTAAGTCCCATTGGTCTAGC	0.49991351	0.390401	0.139694	0
tsrna-17918	TGTTAACTAAGTGTTTGTGGGTTTAAGTCC	0	0	0	0
tsrna-17917	TGTTAACTAAGTGTTTGTGGGTTTAAGTC	0.07141622	0	0.069847	0
tsrna-17916	TGTTAACTAAGTGTTTGTGGGTTTA	0.07141622	0.07808	0.069847	0
tsrna-17915	TGTTAACTAAGTGTTTGTGGGTTT	0	0.23424	0	0.111027
tsrna-17914	TGTTAACTAAGTGTTTGTGGGTT	0	0	0	0
tsrna-17913	TGTTAACTAAGTGTTTGTGGGT	0.07141622	0.15616	0	0
tsrna-17912	TGTTAACTAAGTGTTTGTGGG	0	0	0	0.111027
tsrna-17911	TGTTAACTAAGTGTTTGTGG	0.07141622	0	0	0
tsrna-17910	TGTTAACTAAGTGTTTGTG	0	0	0.069847	0
tsrna-17909	TGTTAACCGAAAGGTTGGTGGT	0.21424865	0.23424	0	0
tsrna-17907	TGTGTGAGGTCCCGGGTTCA	0.07141622	0.468481	0	0
tsrna-17906	TGTGTGAGGTCCCGGGTTC	0	0.15616	0.139694	0
tsrna-17905	TGTGTGAGGTCCCGGGT	0.14283243	0.15616	0	0
tsrna-17904	TGTGGTTAGGATTCGGCGC	0	0	0.279387	0
tsrna-17901	TGTGGGTTTAAGTCCCATTGGTCTAGCCA	0	0	0.069847	0
tsrna-17900	TGTGGGTTTAAGTCCCATTGGTCTAGCC	0	0.07808	0	0
tsrna-17899	TGTGGGTTTAAGTCCCATTGGTCTAGC	0	0.15616	0.20954	0
tsrna-17898	TGTGGGTTTAAGTCCCATTGGTCTAG	0.07141622	0.07808	0.069847	0
tsrna-17897	TGTGGGTTTAAGTCCCATTGGTCTA	0.07141622	0.07808	0.069847	0
tsrna-17896	TGTGGGTTTAAGTCCCATTGGTCT	0	0.07808	0	0
tsrna-17890	TGTGGGTTCGAGTCCCATCTGGGTCGCCA	0	1.015041	0	0.111027
tsrna-17889	TGTGGGTTCGAGTCCCATCTGGGTCGCC	0	0.07808	0	0
tsrna-17881	TGTGGCCGCAGCAACCTCGGTT	0	0.07808	0	0
tsrna-17880	TGTGGCCGCAGCAACCTCGGT	0.07141622	0.07808	0	0
tsrna-17876	TGTGCTTTGCACGCGTGGGTTC	0.14283243	0.468481	0.069847	0
tsrna-17875	TGTGATGGCCGAGTGGTTAAGG	0	0	0	0
tsrna-17873	TGTGAATCTGACAACAGAGGCTTACGACCCCTTATTTACCCA	1.57115676	0.780801	0.908008	0.111027
tsrna-17872	TGTGAATCTGACAACAGAGGCTTACGACCCCTTATTTACCCC	0.92841081	0.624641	0.838162	0
tsrna-17871	TGTGAATCTGACAACAGAGGCTTACGACCCCTTATTTACCC	1.42832433	1.015041	0.977855	0
tsrna-17870	TGTGAATCTGACAACAGAGGCTTACGACCCCTTATT	0.64274595	0.780801	0.419081	0
tsrna-17869	TGTGAATCTGACAACAGAGGCTTACGACCC	0	0	0	0
tsrna-17868	TGTGAATCTGACAACAGAGGCTTACGACC	0.07141622	0.07808	0	0
tsrna-17867	TGTGAATCTGACAACAGAGGCTTACGA	0	0	0	0
tsrna-17866	TGTGAATCTGACAACAGAGGCTTACG	0	0	0	0
tsrna-17865	TGTGAATCTGACAACAGAGGCTTAC	0	0	0	0
tsrna-17864	TGTGAATCTGACAACAGAGGCTTA	0	0.07808	0	0
tsrna-17862	TGTGAATCTGACAACAGAGGCT	0	0	0	0
tsrna-17860	TGTGAATCTGACAACAGAGG	0.07141622	0	0	0
tsrna-17859	TGTGAATCTGACAACAGAG	0	0	0.069847	0
tsrna-17858	TGTGAATCTGACAACAGA	0	0.07808	0	0
tsrna-17855	TGTCTAACAACATGGCTTTCTCACCA	0.21424865	0	0	0
tsrna-17853	TGTCTAACAACATGGCTTTCTCAC	0	0	0	0
tsrna-17852	TGTCTAACAACATGGCTTTCTCA	0.07141622	0	0	0
tsrna-17851	TGTCTAACAACATGGCTTTCTC	0.07141622	0	0	0
tsrna-17849	TGTCTAACAACATGGCTTTC	0	0	0	0
tsrna-17846	TGTCCGCGTGGGTTCGAACCCCACTCCTGGTACC	0.07141622	0.07808	0	0
tsrna-17844	TGTCACGCGGGAGACCGGGGTTCGATTCCCCGACGGGGAG(	0.35708108	1.405442	0.20954	0.111027
tsrna-17843	TGTCACGCGGGAGACCGGGGTTCGATTCCC	0	0.07808	0	0
tsrna-17842	TGTCACGCGGGAGACCGGGGTTCG	0	0.15616	0.139694	0

tsrna-17841	TGTCACGCGGGAGACCGGGGTTCAATTCCCCGACGGGGAG(	0	0.31232	0.069847	0
tsrna-17840	TGTCACGCGGGAGACCGGGGTT	0.14283243	0.15616	0	0
tsrna-17839	TGTCACGCGGGAGACCGGGGT	0	0.07808	0	0
tsrna-17838	TGTCACGCGGGAGACCGGGG	0.14283243	0.07808	0	0
tsrna-17837	TGTCACGCGGGAGACCGG	0.14283243	0.31232	0	0
tsrna-17836	TGTCACGCGGGAGACCG	0	0.23424	0	0.111027
tsrna-17835	TGTCACGCGGGAGACC	0	0.07808	0	0
tsrna-17834	TGTCAAAGTTAAATTATAGGCTAAATCCT	0.49991351	0.07808	0.279387	0
tsrna-17833	TGTATGAGGTCCCGGGTTCGAT	0.21424865	0.390401	0	0
tsrna-17832	TGTATGAGGTCCCGGGTTCGA	0.21424865	0.468481	0	0
tsrna-17831	TGTATGAGGTCCCGGGTTCG	0	0.23424	0	0.111027
tsrna-17830	TGTATGAGGTCCCGGGTTC	0.07141622	0.15616	0	0
tsrna-17829	TGTATGAGGTCCCGGGTT	0.14283243	0.390401	0	0
tsrna-17828	TGTATGAGGTCCCGGGT	0.07141622	0.23424	0	0
tsrna-17827	TGTATGAGGCCCCGGGTTCGATCCCCGGCATCTCCACC	1.2854919	2.030083	0.419081	0.333081
tsrna-17826	TGTATGAGGCCCCGGGTTCGATCCCCGGCATCTCCAC	0	0.390401	0.20954	0
tsrna-17825	TGTATGAGGCCCCGGGTTCGATCCCCGGC	0.14283243	0.546561	0.279387	0
tsrna-17824	TGTATGAGGCCCCGGGTTCGATC	0	0.31232	0	0
tsrna-17823	TGTATGAGGCCCCGGGTTCGA	0.21424865	0.23424	0	0
tsrna-17822	TGTATGAGGCCCCGGGTTCG	0.07141622	0.468481	0.069847	0
tsrna-17821	TGTATGAGGCCCCGGGT	0	0.31232	0.069847	0
tsrna-17820	TGTAGTTGAAATACAACGATGGTTTTTC	0	0.07808	0.069847	0
tsrna-17819	TGTAGTGTAGTGGTTATCACGTTCGCCT	0	0.07808	0	0
tsrna-17818	TGTAGTGTAGTGGTTATCACGTTCGCC	0.07141622	0	0.069847	0
tsrna-17816	TGTAGTGGTTATCACGTTCGCCTCAC	0	0	0	0.111027
tsrna-17815	TGTAGTGGTTATCACGTTCGCCTCA	0	0	0.069847	0
tsrna-17800	TGTAGTGGTTATCACATTCGCC	0	0.07808	0	0
tsrna-17798	TGTAGTGGTCATCACGTTCGCCT	0	0.07808	0	0
tsrna-17795	TGTAGTGGTATCATGCAAGATTCCC	0	0	0	0
tsrna-17793	TGTAGTGGTATCATGCAAGATTC	0	0.07808	0.069847	0
tsrna-17788	TGTAGTCGTGGCCGAGTGG	0.49991351	1.015041	0.558774	0.555136
tsrna-17787	TGTAGTCGTGGCCGAGTG	0.28566487	0.23424	0	0.444109
tsrna-17786	TGTAGTCGTGGCCGAG	0	0.23424	0	0.333081
tsrna-17785	TGTAGTCCGTGCGAGAATACCA	0	0	0.069847	0
tsrna-17784	TGTAGTCCGTGCGAGAATACC	0	0	0	0
tsrna-17783	TGTAGTCCGTGCGAGAATAC	0	0.07808	0	0
tsrna-17782	TGTAGTCCGTGCGAGAATA	0	0.07808	0	0
tsrna-17778	TGTAGTATAAACTAATACACCAG	0	0	0.069847	0
tsrna-17777	TGTAGCTTACCTCCTCAAAGCAATA	0	0.07808	0	0
tsrna-17774	TGTAGCTTACCTCCTCAAAG	0	0	0	0
tsrna-17769	TGTAGCTCAGTGGTAGAGCGCGTGCTT	0.4284973	2.342403	0.069847	0
tsrna-17768	TGTAGCTCAGTGGTAGAGCGCGTGC	0.57132973	1.405442	0.069847	0
tsrna-17767	TGTAGCTCAGTGGTAGAGCGCGTG	0	0.07808	0	0
tsrna-17760	TGTAGCTCAGTGGTAGAGCATTTGACTGC	0.14283243	0.23424	0.069847	0.222054
tsrna-17759	TGTAGCTCAGTGGTAGAGCATTTGACTG	0.07141622	0.31232	0.139694	0
tsrna-17758	TGTAGCTCAGTGGTAGAGCATTTGACT	0.07141622	0.23424	0	0.111027
tsrna-17748	TGTAGCGGTTATCACATTCGCC	0	0	0	0
tsrna-17744	TGTAGATCCTTAGGTCGCTGGT	0	0.07808	0	0
tsrna-17740	TGTACGAGGCCCCGGGTTCG	0.07141622	0.15616	0	0.111027
tsrna-17739	TGTACGAGGCCCCGGGTTC	0.07141622	0.23424	0	0
tsrna-17738	TGTACGAGGCCCCGGGT	0	0.15616	0	0
tsrna-17735	TGTAATGGTTAGCACTCTGGACTTTGAATCCA	0.8569946	0.390401	0.279387	0.555136
tsrna-17734	TGTAATGGTTAGCACTCTGGACTTTGAATC	0.28566487	0.07808	0.139694	0
tsrna-17733	TGTAATGGTTAGCACTCTGGACTTTGAA	0.07141622	0.07808	0	0
tsrna-17732	TGTAATGGTTAGCACTCTGGACTTTGA	0	0	0.069847	0.222054
tsrna-17731	TGTAATGGTTAGCACTCTGGACTTTG	0	0	0	0
tsrna-17730	TGTAATGGTTAGCACTCTGGACTTT	0.21424865	0	0.069847	0.111027
tsrna-17729	TGTAATGGTTAGCACTCTGGACTT	0	0	0.069847	0
tsrna-17728	TGTAATGGTTAGCACTCTGGACTCTGAATCCAGCGA	0.35708108	0.31232	0.279387	0.444109
tsrna-17727	TGTAATGGTTAGCACTCTGGACTCTGAATCCA	0.28566487	0.468481	0.20954	0.333081
tsrna-17726	TGTAATGGTTAGCACTCTGGACTCTGAATCC	0.21424865	0.546561	0.139694	0.666163
tsrna-17725	TGTAATGGTTAGCACTCTGGACTCTGAATC	0	0.15616	0.069847	0.222054
tsrna-17724	TGTAATGGTTAGCACTCTGGACTCTGAAT	0.14283243	0.07808	0.069847	0
tsrna-17723	TGTAATGGTTAGCACTCTGGACTCTGAA	0	0.07808	0	0

tsrna-17722	TGTAATGGTTAGCACTCTGGACTCTGA	0.07141622	0	0	0.111027
tsrna-17721	TGTAATGGTTAGCACTCTGGACTCTG	0	0.15616	0	0
tsrna-17720	TGTAATGGTTAGCACTCTGGACTCT	0.07141622	0.07808	0.069847	0
tsrna-17719	TGTAATGGTTAGCACTCTGGACTC	0.07141622	0	0	0
tsrna-17718	TGTAATGGTTAGCACTCTGGACT	0	0.07808	0	0
tsrna-17717	TGTAATGGTTAGCACTCTGGAC	0	0	0	0.222054
tsrna-17716	TGTAATGGTTAGCACTCTGGA	0	0	0	0.111027
tsrna-17714	TGTAATGGTTAGCACTCTG	0.07141622	0	0	0
tsrna-17711	TGTAATGGTGAGCACTCTGGACTCTGAATCCA	0	0.936961	0.069847	0.333081
tsrna-17710	TGTAATGGTGAGCACTCTGGACTCTGA	0	0	0	0
tsrna-17709	TGTAATGGTGAGCACTCTGGACTCTG	0.07141622	0	0	0
tsrna-17708	TGTAATGGTGAGCACTCTGGACTCT	0	0	0	0
tsrna-17706	TGTAATGGTGAGCACTCTGGACT	0	0	0	0
tsrna-17705	TGTAATGGTGAGCACTCTGGAC	0	0	0	0.111027
tsrna-17703	TGTAATGGTGAGCACTCTGG	0	0	0	0
tsrna-17702	TGTAATGGTGAGCACTCTG	0	0.07808	0	0
tsrna-17696	TGTAATGGTAAGCACTCTGGACTCTGA	0	0.15616	0	0
tsrna-17695	TGTAATGGTAAGCACTCTGGACTCTG	0	0	0	0
tsrna-17692	TGTAATGGTAAGCACTCTGGACT	0	0	0	0
tsrna-17690	TGTAATGGTAAGCACTCTGGA	0	0	0	0
tsrna-17679	TGTAAACCGGAGATGAAAACCTTTTTCCAAGGACACCA	2.71381622	2.654724	6.495753	0
tsrna-17678	TGTAAACCGGAGATGAAAACCTTTTTCCAAGGACACC	0.49991351	1.483522	1.117549	0
tsrna-17677	TGTAAACCGGAGATGAAAACCTTTTTCCAAGGACAC	0.14283243	1.249282	0.069847	0
tsrna-17676	TGTAAACCGGAGATGAAAACCTTTTTCCA	0.28566487	0.546561	0.069847	0
tsrna-17675	TGTAAACCGGAGATGAAAACCTT	0	0	0.069847	0
tsrna-17674	TGTAAACCGGAGATGAAAACCT	0.07141622	0	0	0
tsrna-17673	TGTAAACCGGAGATGAAAACC	0	0.07808	0	0
tsrna-17672	TGTAAACCAGGGGTCGCGAGTTC	0.57132973	2.108163	0.558774	0.333081
tsrna-17671	TGTAAACCAGGGGTCGCGAGT	0.99982703	1.873923	0.419081	0.222054
tsrna-17670	TGTAAACCAGGGGTCGCGAG	0.14283243	0.390401	0.069847	0
tsrna-17669	TGTAAACCAGGGGTCGCGA	0	0.23424	0	0
tsrna-17668	TGTAAACCAGGGGTCGCG	0	0	0.069847	0
tsrna-17667	TGTAAACCAGGGGTCG	0.07141622	0	0	0
tsrna-17666	TGGTTTTTCATATCATTGGTCGTGGTTGTAGTCCGTGCGAGA/	1.21407568	1.483522	1.53663	1.55438
tsrna-17665	TGGTTTTTCATATCATTGGTCGTGGTTGTAGTCCGT	0.28566487	0.31232	0.349234	0
tsrna-17664	TGGTTTTTCATATCATTGGTCGTGGTTGTA	0.07141622	0.07808	0.139694	0
tsrna-17663	TGGTTTTTCATATCATTGGTCGTGGTTGT	0	0	0	0
tsrna-17662	TGGTTTTTCATATCATTGGTCGTGGTTG	0	0.15616	0.139694	0
tsrna-17661	TGGTTTTTCATATCATTGGTCGTGGTT	0	0.07808	0	0
tsrna-17659	TGGTTTTTCATATCATTGGTCGTGG	0	0	0	0
tsrna-17657	TGGTTTTTCATATCATTGGTCGT	0	0	0	0
tsrna-17651	TGGTTTTCACCCAGGTGGCCCGG	0.14283243	0.390401	0.279387	0
tsrna-17650	TGGTTTTCACCCAGGTGGCCCG	0.07141622	0.546561	0.139694	0
tsrna-17649	TGGTTTTCACCCAGGTGGCC	0.14283243	0.23424	0.20954	0
tsrna-17648	TGGTTTTCACCCAGGCGGCCCGGGT	0.07141622	0.15616	0.069847	0
tsrna-17647	TGGTTTTCACCCAGGCGGCCCGG	0.07141622	0.31232	0	0.111027
tsrna-17646	TGGTTTTCACCCAGGCGGCCCG	0	0.390401	0	0
tsrna-17645	TGGTTTTCACCCAGGCGGCCC	0.14283243	0.07808	0.069847	0.111027
tsrna-17644	TGGTTTTCACCCAGGCGGCC	0	0.23424	0	0
tsrna-17643	TGGTTTAGTGGTAGAATTCTCGCCT	0.21424865	0.15616	0.069847	0.111027
tsrna-17642	TGGTTTAGTGGTAGAATTCTCGCC	0.07141622	0.468481	0.069847	0.222054
tsrna-17641	TGGTTTAGTGGTAGAATTCTCGC	0.14283243	0.07808	0.069847	0
tsrna-17640	TGGTTTAGTGGTAGAATTCTCG	0	0	0	0
tsrna-17639	TGGTTTAGTGGTAGAATTCTC	0	0	0.069847	0
tsrna-17637	TGGTTGTAGTCCGTGCGAGAATACCA	0	0.390401	0.069847	0.222054
tsrna-17636	TGGTTGTAGTCCGTGCGAGAATACC	0	0	0.069847	0.111027
tsrna-17635	TGGTTGTAGTCCGTGCGAGAATAC	0.14283243	0	0	0
tsrna-17634	TGGTTGTAGTCCGTGCGAGAATA	0.07141622	0.07808	0	0
tsrna-17633	TGGTTGTAGTCCGTGCGAGAAT	0	0.15616	0	0
tsrna-17632	TGGTTGTAGTCCGTGCGAGAA	0	0.07808	0	0
tsrna-17631	TGGTTGTAGTCCGTGCGAGA	0	0	0	0.111027
tsrna-17630	TGGTTGTAGTCCGTGCGAG	0	0	0	0
tsrna-17629	TGGTTGTAGTCCGTGCGA	0	0	0	0
tsrna-17626	TGGTTCGATTCCGGCTCGAAGGACCA	5.28480002	12.33666	1.396936	2.664651

tsrna-17625	TGGTTCGATTCCGGCTCGAAGGACC	0.49991351	0.936961	0.139694	0.333081
tsrna-17623	TGGTTCGATTCCGGCTCGAAGGA	0	0	0	0
tsrna-17621	TGGTTCGATTCCGGCTCGAAG	0.07141622	0	0	0
tsrna-17620	TGGTTCGATTCCAGCTCGAAGGACCA	0	0	0	0
tsrna-17619	TGGTTCGATCCCGGGTTTCGGCACCA	2.71381622	6.480649	0.977855	1.110271
tsrna-17617	TGGTTCGATCCCGGGTTTCGG	0	0.07808	0	0
tsrna-17615	TGGTTCGAGCCCACCCAGGGACGCCA	0.14283243	9.369613	0	0.333081
tsrna-17611	TGGTTCGAGCCCACCCAG	0	0.07808	0	0
tsrna-17608	TGGTTCGAATCCGGCTCGAAGGACCA	1.64257298	3.279365	0.977855	0.999244
tsrna-17607	TGGTTCGAATCCGGCTCGAAGGACC	0.21424865	0.390401	0	0.111027
tsrna-17606	TGGTTCGAATCCGGCTCGAAGGAC	0	0.07808	0	0
tsrna-17602	TGGTTCAGTGGTAGAATTCTT	0.14283243	0.15616	0.069847	0
tsrna-17601	TGGTTCAGTGGTAGAATTCTCGCCTGCCAC	1.71398919	5.153287	0.908008	0.999244
tsrna-17600	TGGTTCAGTGGTAGAATTCTCGCCTGCC	1.14265946	2.732804	0.419081	0.999244
tsrna-17599	TGGTTCAGTGGTAGAATTCTCGCCTGC	1.07124325	1.249282	0.279387	0.555136
tsrna-17598	TGGTTCAGTGGTAGAATTCTCGCCTG	0.28566487	0.624641	0.139694	0.222054
tsrna-17597	TGGTTCAGTGGTAGAATTCTCGCCTCC	0.49991351	1.249282	0.069847	1.443353
tsrna-17596	TGGTTCAGTGGTAGAATTCTCGCCTC	0.8569946	0.702721	0.139694	0.111027
tsrna-17595	TGGTTCAGTGGTAGAATTCTCGCCT	0.49991351	0.624641	0.20954	0.333081
tsrna-17594	TGGTTCAGTGGTAGAATTCTCGCC	0.49991351	0.15616	0.139694	0.222054
tsrna-17593	TGGTTCAGTGGTAGAATTCTCGC	0.14283243	0	0	0.222054
tsrna-17592	TGGTTCAGTGGTAGAATTCTCG	0.21424865	0	0.069847	0
tsrna-17591	TGGTTCAGTGGTAGAATTCTC	0	0.15616	0.069847	0
tsrna-17590	TGGTTCAGTGGTAGAATTCT	0.21424865	0	0	0.111027
tsrna-17589	TGGTTCAGTGGTAGAATTC	0.4284973	0.23424	0	0.222054
tsrna-17588	TGGTTCAGTGGTAGAATT	0.07141622	0.23424	0.069847	0
tsrna-17587	TGGTTCAGTGGTAGAAT	0.07141622	0.15616	0	0.111027
tsrna-17586	TGGTTCAGTGGTAGAA	0	0.07808	0.069847	0.111027
tsrna-17584	TGGTTCAAATCCGGGTGCCCCCTCCA	8.92702705	16.0845	4.889276	1.665407
tsrna-17583	TGGTTATCACGTTCGCCTCACAC	0.07141622	0	0	0
tsrna-17582	TGGTTATCACGTTCGCCTCACA	0.14283243	0	0.069847	0.111027
tsrna-17576	TGGTTATCACGTTCGC	0	0.07808	0	0
tsrna-17575	TGGTTATCACGTCTGCTTTACACGCAGAAGGTCCTGGGT	0.99982703	3.123204	0.628621	0.666163
tsrna-17574	TGGTTATCACGTCTGCTTTACACGC	0	0.07808	0.069847	0
tsrna-17573	TGGTTATCACGTCTGCTTTACA	0	0.07808	0.20954	0
tsrna-17572	TGGTTATCACGTCTGCTTTAC	0	0	0	0.111027
tsrna-17568	TGGTTATCACATTCGCCTCAC	0.07141622	0	0	0
tsrna-17567	TGGTTATACCCTTCCCGTACTACCA	0.07141622	0.15616	0	0
tsrna-17558	TGGTTAGTATCCCCGCCTGTCACGCGGGAG	1.64257298	4.762887	1.047702	0.333081
tsrna-17557	TGGTTAGTATCCCCGCCTGTCACGC	0.14283243	0.15616	0.069847	0.555136
tsrna-17556	TGGTTAGTATCCCCGCCTGTCAC	0	0.23424	0.20954	0.222054
tsrna-17555	TGGTTAGTATCCCCGCCTGTC	0.14283243	0.07808	0.069847	0.222054
tsrna-17554	TGGTTAGTATCCCCGCCTGT	0	0	0	0
tsrna-17551	TGGTTAGTATCCCCGC	0	0	0	0
tsrna-17550	TGGTTAGTACTCTGCGTTGTGGCCGCAGC	0.64274595	0.624641	0.139694	0.111027
tsrna-17549	TGGTTAGTACTCTGCGTTGTGGCCGCA	0.99982703	0.390401	0.349234	0.222054
tsrna-17548	TGGTTAGTACTCTGCGTTGTGGCCGC	0.49991351	0.23424	0.069847	0.333081
tsrna-17547	TGGTTAGTACTCTGCGTTGTGGCCG	0.71416216	0.390401	0.279387	0.111027
tsrna-17546	TGGTTAGTACTCTGCGTTGTGGCC	0.57132973	0.23424	0.349234	0.222054
tsrna-17545	TGGTTAGTACTCTGCGTTGTGGC	0.64274595	0.23424	0.349234	0.111027
tsrna-17544	TGGTTAGTACTCTGCGTTGTGG	0.71416216	0.23424	0.419081	0.111027
tsrna-17543	TGGTTAGTACTCTGCGTTGTG	0.57132973	0.07808	0.279387	0.111027
tsrna-17542	TGGTTAGTACTCTGCGTTGT	0.64274595	0.390401	0.628621	0.222054
tsrna-17541	TGGTTAGTACTCTGCGTTG	0.35708108	0.23424	0.20954	0
tsrna-17540	TGGTTAGTACTCTGCGTT	0.64274595	0.31232	0.349234	0.222054
tsrna-17539	TGGTTAGTACTCTGCGT	0	0	0.069847	0
tsrna-17538	TGGTTAGTACTCTGCGCTGTG	0	0.07808	0.069847	0
tsrna-17537	TGGTTAGTACTCTGCGCTGT	0.07141622	0.15616	0	0
tsrna-17536	TGGTTAGTACTCTGCGCTG	0.14283243	0	0.139694	0
tsrna-17535	TGGTTAGTACTCTGCGCT	0.07141622	0	0.139694	0
tsrna-17534	TGGTTAGTACTCTGCGC	0.07141622	0	0.069847	0
tsrna-17532	TGGTTAGGATTCGGCGCTCTCAT	0.71416216	0.468481	0.20954	0.333081
tsrna-17531	TGGTTAGGATTCGGCGCTCTCACCGCCGCGGCCCGGGT	0.64274595	1.873923	0.768315	1.443353
tsrna-17530	TGGTTAGGATTCGGCGCTCTCACCGCCGCGGCCCGGG	0.71416216	1.639682	0.488928	0.222054

tsrna-17529	TGGTTAGGATTCGGCGCTCTCACCGCCGCGGCCCGG	0.99982703	1.873923	0.558774	0.666163
tsrna-17528	TGGTTAGGATTCGGCGCTCTCACCGCCGCGGCCC	0.57132973	1.873923	0.558774	0.888217
tsrna-17527	TGGTTAGGATTCGGCGCTCTCACCGCCGCGGC	0.8569946	1.249282	0.838162	0.666163
tsrna-17526	TGGTTAGGATTCGGCGCTCTCACCGCCGCG	0.92841081	1.483522	0.558774	0.444109
tsrna-17525	TGGTTAGGATTCGGCGCTCTCACCGCCGC	0.49991351	1.327362	0.768315	0.444109
tsrna-17524	TGGTTAGGATTCGGCGCTCTCACCGCC	0.71416216	1.093122	0.488928	0.77719
tsrna-17523	TGGTTAGGATTCGGCGCTCTCACCGC	0.92841081	0.936961	0.279387	0.888217
tsrna-17522	TGGTTAGGATTCGGCGCTCTCACCG	0.57132973	0.780801	0.349234	0.666163
tsrna-17521	TGGTTAGGATTCGGCGCTCTCACC	0.49991351	0.23424	0.349234	0.333081
tsrna-17520	TGGTTAGGATTCGGCGCTCTCAC	0.8569946	0.468481	0.20954	0.333081
tsrna-17519	TGGTTAGGATTCGGCGCTCTCA	0.57132973	0.936961	0.279387	0.333081
tsrna-17518	TGGTTAGGATTCGGCGCTCTC	0.71416216	0.31232	0.419081	0.333081
tsrna-17517	TGGTTAGGATTCGGCGCTCT	0.49991351	0.468481	0.20954	0.333081
tsrna-17516	TGGTTAGGATTCGGCGCTC	0.35708108	0.624641	0.20954	0.222054
tsrna-17515	TGGTTAGGATTCGGCGCT	0.07141622	0.31232	0.139694	0.333081
tsrna-17514	TGGTTAGGATTCGGCGC	0	0	0	0.111027
tsrna-17512	TGGTTAGCATAGCTGCCTTCCAAG	0	0	0.069847	0
tsrna-17506	TGGTTAGCACTCTGGACTTTGAATCCAGCG	0.49991351	0.468481	0.139694	0.222054
tsrna-17505	TGGTTAGCACTCTGGACTTTGAATCCAGC	0.28566487	0.23424	0.139694	0.333081
tsrna-17504	TGGTTAGCACTCTGGACTTTGAATCCA	0.21424865	0.31232	0.279387	0.222054
tsrna-17503	TGGTTAGCACTCTGGACTTTGAATCC	0.14283243	0.390401	0.349234	0
tsrna-17502	TGGTTAGCACTCTGGACTTTGAATC	0	0.15616	0.069847	0.111027
tsrna-17499	TGGTTAGCACTCTGGACTTTGA	0	0	0	0
tsrna-17495	TGGTTAGCACTCTGGACTCTGAATCCAGCGATCCGAGTTCA	0.4284973	0.23424	0.279387	0
tsrna-17494	TGGTTAGCACTCTGGACTCTGAATCCAGCGATCCGAGTTC	0.14283243	0.31232	0.20954	0
tsrna-17493	TGGTTAGCACTCTGGACTCTGAATCCAGCGATCCGA	0.21424865	0.15616	0.069847	0
tsrna-17492	TGGTTAGCACTCTGGACTCTGAATCCAGCG	0.14283243	0.390401	0.139694	0
tsrna-17491	TGGTTAGCACTCTGGACTCTGAATCCAGC	0.28566487	0.23424	0.279387	0.111027
tsrna-17490	TGGTTAGCACTCTGGACTCTGAATCCAG	0.21424865	0.23424	0.139694	0.111027
tsrna-17489	TGGTTAGCACTCTGGACTCTGAATCCA	0.28566487	0.23424	0.069847	0
tsrna-17488	TGGTTAGCACTCTGGACTCTGAATCC	0.14283243	0.07808	0	0.111027
tsrna-17487	TGGTTAGCACTCTGGACTCTGAATC	0	0	0.069847	0
tsrna-17486	TGGTTAGCACTCTGGACTCTGAAT	0	0	0.069847	0
tsrna-17483	TGGTTAGCACTCTGGACTCTG	0	0.07808	0.069847	0
tsrna-17481	TGGTTAGCACTCTGGACTC	0	0	0	0
tsrna-17480	TGGTTAGCACTCTGGACT	0.07141622	0	0	0
tsrna-17478	TGGTTAGCACTCTGGA	0.07141622	0	0	0.111027
tsrna-17477	TGGTTAGAGCGTGGTGCTAATA	0.14283243	0.31232	0.20954	0.111027
tsrna-17475	TGGTTAAGGCGTTGGACTTGAAATC	0.07141622	0	0	0
tsrna-17474	TGGTTAAGGCGTTGGACTTGAAAT	0	0	0	0
tsrna-17471	TGGTTAAGGCGTTGGACTTAAGATC	0	0	0.069847	0
tsrna-17470	TGGTTAAGGCGTTGGACTTAAGAT	0	0	0	0
tsrna-17469	TGGTTAAGGCGTTGGACTTAAGA	0	0	0	0
tsrna-17467	TGGTTAAGGCGTTGGACTTAA	0	0.07808	0	0
tsrna-17466	TGGTTAAGGCGTTGGACTTA	0	0.07808	0	0
tsrna-17456	TGGTTAAGGCGATGGACTGCTAAT	0.14283243	0.15616	0	0.111027
tsrna-17455	TGGTTAAGGCGATGGACTGCTAA	0	0	0	0.222054
tsrna-17454	TGGTTAAGGCGATGGACTGCTA	0.07141622	0	0	0
tsrna-17453	TGGTTAAGGCGATGGACTAGAAATC	0.07141622	0.23424	0	0
tsrna-17452	TGGTTAAGGCGATGGACTAGAA	0	0.07808	0	0
tsrna-17451	TGGTTAAGGCGATGGACTAGA	0	0.07808	0	0
tsrna-17450	TGGTTAAGGCGATGGACTAG	0	0.15616	0	0.222054
tsrna-17444	TGGTGTAGTGGTATCATGCAAGATTC	0.14283243	0	0	0
tsrna-17443	TGGTGTAGTGGTATCATGCAAGATT	0.07141622	0.15616	0	0
tsrna-17442	TGGTGTAGTGGTATCATGCAAGA	0	0.15616	0.20954	0
tsrna-17441	TGGTGTAGTGGTATCATGCAAG	0.07141622	0	0	0
tsrna-17438	TGGTGTAGTGGTATCATG	0.07141622	0	0	0
tsrna-17437	TGGTGTAATGGTTAGCACTCTGGGCT	0.07141622	0.07808	0	0
tsrna-17436	TGGTGTAATGGTTAGCACTCTGGACTTTGAATCC	1.07124325	0.936961	0.419081	0.999244
tsrna-17435	TGGTGTAATGGTTAGCACTCTGGACTTTGAATC	0.57132973	0.546561	0.20954	0.999244
tsrna-17434	TGGTGTAATGGTTAGCACTCTGGACTTTG	0	0	0.069847	0.111027
tsrna-17433	TGGTGTAATGGTTAGCACTCTGGACTTT	0.07141622	0.07808	0.069847	0
tsrna-17432	TGGTGTAATGGTTAGCACTCTGGACTT	0	0	0.20954	0.333081
tsrna-17431	TGGTGTAATGGTTAGCACTCTGGACTCTGAATCCAGCGATCC	0.8569946	0.702721	0.419081	0.444109

tsrna-17430	TGGTGTAATGGTTAGCACTCTGGACTCTGAATCCAGCGATCC	0.4284973	1.327362	0.349234	0.333081
tsrna-17429	TGGTGTAATGGTTAGCACTCTGGACTCTGAATCCAG	0.8569946	0.702721	0.628621	0.444109
tsrna-17428	TGGTGTAATGGTTAGCACTCTGGACTCTGAATCCA	0.49991351	0.624641	0.139694	0.111027
tsrna-17427	TGGTGTAATGGTTAGCACTCTGGACTCTGAATC	0.14283243	0.390401	0	0.444109
tsrna-17426	TGGTGTAATGGTTAGCACTCTGGACTCTGAAT	0.21424865	0.15616	0.069847	0.222054
tsrna-17425	TGGTGTAATGGTTAGCACTCTGGACTCTG	0.28566487	0.15616	0.069847	0.222054
tsrna-17424	TGGTGTAATGGTTAGCACTCTGGACTCT	0	0.07808	0	0.111027
tsrna-17423	TGGTGTAATGGTTAGCACTCTGGACTC	0.14283243	0.15616	0.139694	0
tsrna-17422	TGGTGTAATGGTTAGCACTCTGGACT	0.07141622	0.07808	0.069847	0
tsrna-17421	TGGTGTAATGGTTAGCACTCTGGAC	0.07141622	0.07808	0	0
tsrna-17420	TGGTGTAATGGTTAGCACTCTGGA	0	0.15616	0.069847	0
tsrna-17419	TGGTGTAATGGTTAGCACTCTGG	0	0	0.069847	0
tsrna-17414	TGGTGTAATGGTGAGCACTCTGGACTCTG	0	0	0	0
tsrna-17413	TGGTGTAATGGTGAGCACTCTGGACTCT	0	0.07808	0	0.111027
tsrna-17412	TGGTGTAATGGTGAGCACTCTGGACTC	0.07141622	0	0	0
tsrna-17411	TGGTGTAATGGTGAGCACTCTGGACT	0.07141622	0	0	0
tsrna-17410	TGGTGTAATGGTGAGCACTCTGGAC	0	0.07808	0	0
tsrna-17406	TGGTGTAATGGTCAGCACTCTGGACTC	0	0	0	0
tsrna-17403	TGGTGTAATGGTCAGCACTCTGGA	0.07141622	0	0	0
tsrna-17401	TGGTGTAATGGTAAGCACTCTGGACTC	0	0	0	0
tsrna-17400	TGGTGTAATGGTAAGCACTCTGGACT	0	0	0	0.111027
tsrna-17394	TGGTGGTTCAGTGGTAGAATTCTCGCCTGC	0.78557838	0.780801	0.488928	0.222054
tsrna-17393	TGGTGGTTCAGTGGTAGAATTCTCGCCTG	0.21424865	0.546561	0.139694	0.444109
tsrna-17392	TGGTGGTTCAGTGGTAGAATTCTCGCCT	0.49991351	0.31232	0.558774	0.333081
tsrna-17391	TGGTGGTTCAGTGGTAGAATTCTCGCC	0.28566487	0.936961	0.349234	0
tsrna-17390	TGGTGGTTCAGTGGTAGAATTCTCGC	0.28566487	0.390401	0.139694	0
tsrna-17389	TGGTGGTTCAGTGGTAGAATTCTCG	0.07141622	0	0.069847	0
tsrna-17388	TGGTGGTTCAGTGGTAGAATTCTC	0.35708108	0.390401	0.069847	0
tsrna-17387	TGGTGGTTCAGTGGTAGAATTCT	0.14283243	0.468481	0.139694	0.222054
tsrna-17386	TGGTGGTTCAGTGGTAGAATT	0.21424865	0.15616	0	0
tsrna-17385	TGGTGGTTCAGTGGTAGAAT	0.14283243	0.07808	0.069847	0.111027
tsrna-17384	TGGTGGTTCAGTGGTAGAA	0.28566487	0	0	0
tsrna-17383	TGGTGGTTCAGTGGTAGA	0	0.07808	0	0.111027
tsrna-17380	TGGTGGTCTAGTGGTTAGGATTCGGCGCTCTCACC	2.64240001	4.138246	2.095404	1.776434
tsrna-17379	TGGTGGTCTAGTGGTTAGGATTCGGCGCTC	2.57098379	4.216326	1.885864	1.665407
tsrna-17378	TGGTGGTCTAGTGGTTAGGATTCGGCGCT	2.07107028	2.576644	1.187396	1.221299
tsrna-17377	TGGTGGTCTAGTGGTTAGGATTCGGCGC	0.92841081	1.093122	0.558774	0.888217
tsrna-17376	TGGTGGTCTAGTGGTTAGGATTCGGCG	0.99982703	1.093122	1.047702	0.555136
tsrna-17375	TGGTGGTCTAGTGGTTAGGATTCGGC	0.4284973	0.936961	0.20954	0.555136
tsrna-17374	TGGTGGTCTAGTGGTTAGGATTCGG	0.4284973	0.31232	0.20954	0
tsrna-17373	TGGTGGTCTAGTGGTTAGGATTCG	0.14283243	0	0.069847	0.222054
tsrna-17372	TGGTGGTCTAGTGGTTAGGATTC	0.07141622	0.07808	0.069847	0.111027
tsrna-17371	TGGTGGTCTAGTGGTTAGGATT	0.21424865	0	0.139694	0.111027
tsrna-17370	TGGTGGTCTAGTGGTTAGGAT	0.21424865	0	0	0
tsrna-17365	TGGTGGTCTAGTGGCTAGGATTCGGCGC	0.35708108	2.967044	0.558774	3.441841
tsrna-17364	TGGTGGTCTAGTGGCTAGGATTCGGCG	1.21407568	3.591685	0.20954	2.775678
tsrna-17363	TGGTGGTCTAGTGGCTAGGATTCGGC	0.28566487	1.249282	0.279387	1.887461
tsrna-17362	TGGTGGTCTAGTGGCTAGGATTCGG	0	0.31232	0.069847	0.333081
tsrna-17361	TGGTGGTCTAGTGGCTAGGATTCG	0.28566487	0.15616	0	0.666163
tsrna-17360	TGGTGGTCTAGTGGCTAGGAT	0	0.07808	0.069847	0.444109
tsrna-17358	TGGTGGTCTAGTGGCTAG	0	0.07808	0	0
tsrna-17357	TGGTGGAGTTAAAGACTTTTTCTCTGACCA	1.64257298	2.810884	1.047702	1.665407
tsrna-17356	TGGTGGAGTTAAAGACTTTTTCTCTGACC	0.14283243	1.015041	0.139694	0
tsrna-17355	TGGTGGAGTTAAAGACTTTTTCTCTGAC	0.07141622	0.390401	0.139694	0
tsrna-17354	TGGTGGAGTTAAAGACTTTTTCTCTGA	0.07141622	0	0.20954	0
tsrna-17353	TGGTGGAGTTAAAGACTTTTTCTCTG	0.14283243	0.390401	0.069847	0
tsrna-17351	TGGTGGAGTTAAAGACTTTTTCT	0	0	0	0
tsrna-17350	TGGTGGAGTTAAAGACTTTTT	0	0	0	0
tsrna-17349	TGGTGGAGTTAAAGACTTTT	0	0.07808	0.069847	0
tsrna-17348	TGGTGGAGTTAAAGACTTT	0	0.07808	0	0
tsrna-17347	TGGTGGAGTTAAAGACTT	0.07141622	0	0	0
tsrna-17344	TGGTGCAACTCCAAATAAAAGTACCA	0.07141622	0	0.069847	0
tsrna-17343	TGGTGCAACTCCAAATAAAAGTACC	0.07141622	0	0	0
tsrna-17340	TGGTGAGTATCCCCGCCTGTCACGCGGGAG	2.49956757	4.450566	2.235098	0.666163

tsrna-17339	TGGTGAGTATCCCCGCCTGTCACGC	0.28566487	0.390401	0	0
tsrna-17338	TGGTGAGTATCCCCGCCTGTCACG	0.35708108	0.546561	0.069847	0.333081
tsrna-17337	TGGTGAGTATCCCCGCCTGTCAC	0.57132973	0.546561	0.20954	0
tsrna-17336	TGGTGAGTATCCCCGCCTGTC	0.35708108	1.015041	0.20954	0
tsrna-17335	TGGTGAGTATCCCCGCCTGT	0.14283243	0.23424	0.069847	0
tsrna-17334	TGGTGAGTATCCCCGCCTG	0.07141622	0.23424	0.069847	0
tsrna-17333	TGGTGAGTATCCCCGCCT	0.07141622	0.15616	0	0.111027
tsrna-17332	TGGTGAGTATCCCCGCC	0	0.07808	0	0
tsrna-17331	TGGTGAGTATCCCCGC	0.07141622	0.07808	0	0
tsrna-17330	TGGTGAGCATAGCTGCCTTCCAAG	0	0.07808	0	0
tsrna-17329	TGGTGAGCATAGCTGCCTTCC	0	0.07808	0	0
tsrna-17324	TGGTGAGCACTCTGGACTCTGAATCCAGC	0.07141622	0.390401	0	0.222054
tsrna-17323	TGGTGAGCACTCTGGACTCTGAATCCA	0.07141622	0.31232	0	0
tsrna-17322	TGGTGAGCACTCTGGACTCTGAATCC	0.14283243	0.07808	0	0
tsrna-17321	TGGTGAGCACTCTGGACTCTGAATC	0.07141622	0.07808	0	0
tsrna-17320	TGGTGAGCACTCTGGACTCTGAAT	0	0.07808	0	0
tsrna-17311	TGGTGAGCACCCTGGACTCTGA	0	0	0	0
tsrna-17310	TGGTCTCCGGATGGAGGCGTGGGTT	0.49991351	3.045124	0.279387	0
tsrna-17309	TGGTCTCCGGATGGAGGCGTGG	0.07141622	0.23424	0.069847	0
tsrna-17306	TGGTCTCCAATGGAGGCGTGGGTT	1.07124325	2.030083	0.698468	0
tsrna-17305	TGGTCTCCAATGGAGGCGTGGGT	1.07124325	1.952003	1.117549	0
tsrna-17304	TGGTCTAGTGGTTAGGATTCGGCGCTCTCA	2.78523244	5.153287	1.955711	1.776434
tsrna-17303	TGGTCTAGTGGTTAGGATTCGGCGCTCTC	2.85664866	4.684807	1.816017	1.887461
tsrna-17302	TGGTCTAGTGGTTAGGATTCGGCGCTCT	3.28514596	3.123204	1.606477	3.441841
tsrna-17301	TGGTCTAGTGGTTAGGATTCGGCGCTC	2.64240001	3.591685	2.095404	2.664651
tsrna-17300	TGGTCTAGTGGTTAGGATTCGGCGCT	2.28531893	3.045124	1.955711	1.221299
tsrna-17299	TGGTCTAGTGGTTAGGATTCGGCGC	1.14265946	0.936961	0.628621	0.999244
tsrna-17298	TGGTCTAGTGGTTAGGATTCGGCG	1.07124325	0.546561	0.488928	0.888217
tsrna-17297	TGGTCTAGTGGTTAGGATTCGGC	0.64274595	0.468481	0.349234	0.111027
tsrna-17296	TGGTCTAGTGGTTAGGATTCGG	0.21424865	0.23424	0.139694	0.111027
tsrna-17295	TGGTCTAGTGGTTAGGATTCG	0	0.15616	0.279387	0
tsrna-17294	TGGTCTAGTGGTTAGGATTC	0	0	0.069847	0.111027
tsrna-17293	TGGTCTAGTGGTTAGGATT	0.07141622	0	0	0.111027
tsrna-17290	TGGTCTAGTGGTATGATTCTCGC	0.4284973	0	0.349234	0.222054
tsrna-17289	TTTTTCCAAGGACACCA	0.07141622	0	0.139694	0
tsrna-17288	TTTTTCCAAGGACACC	0	0	0.069847	0.111027
tsrna-17287	TTTTTCATATCATTGGTCGTGGTTGTAGTCCGTGCGAGAATAC(	9.85543787	12.1805	12.36288	5.773411
tsrna-17286	TTTTTCATATCATTGGTCGTGGTTGTAGTCCGTGCGAGAATAC(	1.35690811	2.654724	1.606477	1.998488
tsrna-17285	TTTTTCATATCATTGGTCGTGGTTGTAGTCCGTGCGAGAATA	1.21407568	1.639682	0.908008	1.776434
tsrna-17284	TTTTTCATATCATTGGTCGTGGTTGTAGTCCGTGCGAGAAT	0.78557838	1.249282	0.558774	1.665407
tsrna-17283	TTTTTCATATCATTGGTCGTGGTTGTAGTCCGTGCGAGAA	0.57132973	1.327362	0.838162	1.110271
tsrna-17282	TTTTTCATATCATTGGTCGTGGTTGTAGTCCGTGCGAGA	0.35708108	1.249282	0.698468	0.77719
tsrna-17281	TTTTTCATATCATTGGTCGTGGTTGTAGTCCGTGCG	0.49991351	0.23424	0.349234	0.77719
tsrna-17280	TTTTTCATATCATTGGTCGTGGTTGTAGTC	0.14283243	0.07808	0.069847	0
tsrna-17279	TTTTTCATATCATTGGTCGTGGTTGTAGT	0.07141622	0.07808	0	0
tsrna-17278	TTTTTCATATCATTGGTCGTGGTTGTAG	0	0	0.069847	0
tsrna-17277	TTTTTCATATCATTGGTCGTGGTTGTA	0	0	0	0.111027
tsrna-17275	TTTTTCATATCATTGGTCGTGGTTG	0	0	0	0
tsrna-17263	TTTTGCAGTCCTTACCA	0	0.15616	0	0
tsrna-17261	TTTTCCAAGGACACCA	0.07141622	0.07808	0.069847	0
tsrna-17260	TTTTCATATCATTGGTCGTGGTTGTAGTCCGTGCGAGAATACC(	10.9980973	12.72706	12.29304	6.661628
tsrna-17259	TTTTCATATCATTGGTCGTGGTTGTAGTCCGTGCGAGAATACC(	1.49974054	2.108163	1.117549	1.998488
tsrna-17258	TTTTCATATCATTGGTCGTGGTTGTAGTCCGTGCGAGAATA	0.71416216	1.873923	0.768315	0.999244
tsrna-17257	TTTTCATATCATTGGTCGTGGTTGTAGTCCGTGCGAGAAT	0.71416216	1.639682	0.908008	1.55438
tsrna-17256	TTTTCATATCATTGGTCGTGGTTGTAGTCCGTGCGAGAA	0.78557838	1.483522	0.628621	1.55438
tsrna-17255	TTTTCATATCATTGGTCGTGGTTGTAGTCCGTGCGAGA	0.49991351	0.31232	0.838162	0.666163
tsrna-17254	TTTTCATATCATTGGTCGTGGTTGTAGTCCGTGCGA	0.49991351	0.31232	0.279387	0.666163
tsrna-17253	TTTTCATATCATTGGTCGTGGTTGTAGTCC	0	0.15616	0.069847	0.111027
tsrna-17252	TTTTCATATCATTGGTCGTGGTTGTAGTC	0	0.07808	0.139694	0
tsrna-17251	TTTTCATATCATTGGTCGTGGTTGTAGT	0	0.07808	0.139694	0.111027
tsrna-17250	TTTTCATATCATTGGTCGTGGTTGTAG	0.07141622	0	0	0
tsrna-17249	TTTTCATATCATTGGTCGTGGTTGTA	0	0	0	0
tsrna-17248	TTTTCATATCATTGGTCGTGGTTGT	0	0	0	0.111027
tsrna-17247	TTTTCATATCATTGGTCGTGGTTG	0	0.07808	0	0

tsrna-17239	TTTTCACCCAGGTGGCCCGGG	0.14283243	0.15616	0.139694	0
tsrna-17238	TTTTCACCCAGGTGGCCCGG	0.28566487	0.31232	0.20954	0
tsrna-17237	TTTTCACCCAGGTGGCCCG	0.07141622	0.31232	0.069847	0
tsrna-17236	TTTTCACCCAGGTGGCCC	0.35708108	0.07808	0.069847	0
tsrna-17235	TTTTCACCCAGGCGGCCCGGGTTCGACTCCCGGTGTGGGAA	6.21321083	27.79652	3.352647	3.10876
tsrna-17234	TTTTCACCCAGGCGGCCCGGGTTCGACTCCCGGTGTGG	0.07141622	0.31232	0	0
tsrna-17233	TTTTCACCCAGGCGGCCCGGGTTCGACTCCCGGTGTG	0	0.07808	0.069847	0
tsrna-17231	TTTTCACCCAGGCGGCCCGGGTTCGACTCC	0.14283243	0.07808	0	0
tsrna-17230	TTTTCACCCAGGCGGCCCGGGT	0.28566487	0.07808	0	0.111027
tsrna-17229	TTTTCACCCAGGCGGCCCGGG	0.07141622	0	0	0
tsrna-17228	TTTTCACCCAGGCGGCCCGG	0.14283243	0.15616	0	0
tsrna-17227	TTTTCACCCAGGCGGCCCG	0	0.23424	0	0
tsrna-17225	TTTTCACCCAGGCGGCC	0	0.07808	0	0
tsrna-17224	TTTTAATCTGAGGGTCCAGGGTTCAAGTCCCTGTTCGGGCGC	1.14265946	4.060166	0.768315	0.555136
tsrna-17223	TTTTAATCTGAGGGTCCAGGGT	0.28566487	0.780801	0.069847	0.111027
tsrna-17222	TTTTAATCTGAGGGTCCAGGG	0.07141622	1.405442	0.139694	0
tsrna-17221	TTTTAATCTGAGGGTCCAGG	0.14283243	0.858881	0.139694	0
tsrna-17220	TTTTAATCTGAGGGTCCAG	0.21424865	1.093122	0.279387	0
tsrna-17219	TTTTAATCTGAGGGTCCA	0.35708108	0.702721	0.419081	0.111027
tsrna-17218	TTTTAATCTGAGGGTCC	0.14283243	0.624641	0.069847	0.111027
tsrna-17217	TTTTAAGTTAAAGATTAAGAGAACCAACACCTCTTTACAGTGA	0.35708108	0.07808	0.628621	0.222054
tsrna-17216	TTTTAAGTTAAAGATTAAGAGAACCAACACCTCTTTACAGTGA	0.28566487	0.07808	0.419081	0
tsrna-17215	TTTTAAGTTAAAGATTAAGAGAACCAACACCTCTTTACAGTGA	0.28566487	0.07808	0.20954	0
tsrna-17214	TTTTAAGTTAAAGATTAAGAGAACCAACACC	0	0	0	0.111027
tsrna-17213	TTTTAAGTTAAAGATTAAGAGAACCAACAC	0	0	0	0
tsrna-17212	TTTTAAGTTAAAGATTAAGAGAACC	0	0	0	0
tsrna-17206	TTTGTGGGTTTAAGTCCCATTGGTCTAGCCA	0.07141622	0.07808	0.069847	0.111027
tsrna-17205	TTTGTGGGTTTAAGTCCCATTGGTCTAGCC	0.14283243	0	0.069847	0
tsrna-17203	TTTGTGGGTTTAAGTCCCATTGGTC	0	0	0.069847	0
tsrna-17200	TTTGTGGGTTTAAGTC	0	0	0	0
tsrna-17199	TTTGTCAAAGTTAAATTATAGGCT	0.21424865	0	0	0
tsrna-17198	TTTGTCAAAGTTAAATTATAG	0.21424865	0	0.069847	0
tsrna-17197	TTTGGTGCAACTCCAAATAAAAGTACCA	0	0.468481	0.139694	0
tsrna-17195	TTTGGTCTAGGGGTATGAT	0.07141622	0	0	0
tsrna-17193	TTTGGGTGCTAATGGTGGAGTTAAAGACTTTTTCTC	0.4284973	1.327362	0.419081	0
tsrna-17192	TTTGGGTGCTAATGGTGGAGTTAAAGACTT	0.21424865	0.468481	0.139694	0.222054
tsrna-17191	TTTGGGTGCTAATGGTGGAGTTAAAGAC	0.14283243	0.23424	0	0.222054
tsrna-17190	TTTGGGTGCTAATGGTGGAGTTAAAGA	0.07141622	0.15616	0.20954	0
tsrna-17189	TTTGGGTGCTAATGGTGGAGTTAAAG	0.07141622	0.546561	0	0.111027
tsrna-17188	TTTGGGTGCTAATGGTGGAGTTAAA	0	0.31232	0.069847	0
tsrna-17187	TTTGGGTGCTAATGGTGGAGTTAA	0	0.15616	0	0
tsrna-17186	TTTGGGTGCTAATGGTGGAGTTA	0.07141622	0.07808	0	0.111027
tsrna-17179	TTTGGGTGCGAGAGGTCCCGGGTT	0.78557838	5.231367	0.419081	2.886706
tsrna-17178	TTTGGGTGCGAGAGGTCCCGGGT	0.57132973	3.279365	0.139694	0.77719
tsrna-17177	TTTGGGTGCGAGAGGTCCCGGG	0.14283243	0.15616	0	0
tsrna-17175	TTTGGGTGCGAGAGGTCCCG	0	0.07808	0	0
tsrna-17173	TTTGGGTGCGAGAGGTCC	0	0.07808	0	0
tsrna-17171	TTTGGGTCCGAGAGGTCCCGGGTTCA	0	0.15616	0	0
tsrna-17170	TTTGGGTCCGAGAGGTCCCGGGTT	0	0.07808	0	0
tsrna-17169	TTTGGGTCCGAGAGGTCCCGGGT	0	0.07808	0	0
tsrna-17163	TTTGGGGGGTTCGATTCCTTCCTTTTTTGC	0.07141622	0.15616	0	0.111027
tsrna-17159	TTTGCATGTATGAGGTCCCGGGT	0.07141622	0.31232	0.069847	0.222054
tsrna-17158	TTTGCATGTATGAGGCCTC	0	0	0	0.111027
tsrna-17157	TTTGCATGTATGAGGCCCCGGGT	0.14283243	0.15616	0	0
tsrna-17154	TTTGCACGTATGAGGCCCCGGGTT	0.71416216	2.108163	0.488928	1.665407
tsrna-17153	TTTGCACGTATGAGGCCCCGGGT	0.57132973	2.030083	0.349234	1.110271
tsrna-17152	TTTGCACGTATGAGGCCCCGGG	0.07141622	0	0	0
tsrna-17151	TTTGCACGTATGAGGCCCCGG	0.14283243	0	0	0.111027
tsrna-17150	TTTGCACGTATGAGGCCCCG	0.07141622	0.15616	0	0
tsrna-17149	TTTGCACGTATGAGGCCCC	0.07141622	0	0.139694	0.111027
tsrna-17148	TTTGCACGTATGAGGCCC	0	0	0	0
tsrna-17147	TTTGATAGAGTAAATAATAGGAGCTTAAACCC	0.07141622	0	0	0
tsrna-17146	TTTGATAGAGTAAATAATAGGAGCT	0	0	0	0
tsrna-17142	TTTGACTGCAGATCAAGAGGTCCCTGGT	0.21424865	0.468481	0.139694	0.444109

tsrna-17140	TTTGACTGCAGATCAAGAGGTCC	0	0	0.069847	0
tsrna-17138	TTTGACTGCAGATCAAGAG	0	0.15616	0	0
tsrna-17137	TTTGACAACATTCAAAAAAGAGTACCA	0	0	0	0
tsrna-17136	TTTGACAACATTCAAAAAAGAGTA	0	0	0	0
tsrna-17135	TTTGAATCCAGCGATCCGAGTT	0	0	0	0.111027
tsrna-17131	TTTGAATCCAGCGATCCG	0	0	0	0.111027
tsrna-17130	TTTGAATCCAGCAATCCGAGT	0.07141622	0.624641	0	0.111027
tsrna-17129	TTTCGACTCATTAAATTATGATAATCATAT	0.35708108	0.07808	0.139694	0
tsrna-17128	TTTCGACTCATTAAATTATGATAATCAT	0.14283243	0.15616	0	0
tsrna-17127	TTTCCGTAGTGTAGTGGTTATCACGTTCGCCTCACACGCGAA	0.71416216	0.546561	1.117549	0.444109
tsrna-17126	TTTCCGTAGTGTAGTGGTTATCACGTTCGCCTCACACG	1.21407568	0.702721	0.977855	0.666163
tsrna-17125	TTTCCGTAGTGTAGTGGTTATCACGTTCGCCTCACA	1.35690811	0.390401	0.698468	0.333081
tsrna-17124	TTTCCGTAGTGTAGTGGTTATCACGTTCGCCTCAC	1.21407568	0.546561	0.908008	0.222054
tsrna-17123	TTTCCGTAGTGTAGTGGTTATCACGTTCGCCTCA	0.8569946	0.624641	0.349234	0.111027
tsrna-17122	TTTCCGTAGTGTAGTGGTTATCACGTTCGCCTC	1.71398919	0.31232	0.488928	0.222054
tsrna-17121	TTTCCGTAGTGTAGTGGTTATCACGTTCGCCT	0.99982703	0.07808	0.628621	0.222054
tsrna-17120	TTTCCGTAGTGTAGTGGTTATCACGTTCGCC	1.14265946	0.546561	0.628621	0.222054
tsrna-17119	TTTCCGTAGTGTAGTGGTTATCACGTTCGC	0.78557838	0.15616	0.20954	0.111027
tsrna-17118	TTTCCGTAGTGTAGTGGTTATCACGTTCG	0.4284973	0.23424	0.069847	0.222054
tsrna-17117	TTTCCGTAGTGTAGTGGTTATCACGTTC	1.07124325	0.390401	0.20954	0.222054
tsrna-17116	TTTCCGTAGTGTAGTGGTTATCACGTT	0.57132973	0	0.349234	0.222054
tsrna-17115	TTTCCGTAGTGTAGTGGTTATCACG	0.57132973	0.15616	0.279387	0.222054
tsrna-17114	TTTCCGTAGTGTAGTGGTTATCA	0.49991351	0	0.349234	0.222054
tsrna-17113	TTTCCGTAGTGTAGTGGTTATC	0.4284973	0.07808	0.279387	0.111027
tsrna-17112	TTTCCGTAGTGTAGTGGTT	0.07141622	0.15616	0	0
tsrna-17111	TTTCCGTAGTGTAGTGGTCATCACGTTCGCCT	0.28566487	0.15616	0.139694	0.111027
tsrna-17110	TTTCCGTAGTGTAGTGGTCATCACGTTCGCC	0.35708108	0.07808	0.279387	0.333081
tsrna-17109	TTTCCGTAGTGTAGTGGTCATCACGTTCGC	0.64274595	0.07808	0.069847	0.222054
tsrna-17108	TTTCCGTAGTGTAGTGGTCATCACGTTCG	0.57132973	0.07808	0.069847	0.222054
tsrna-17107	TTTCCGTAGTGTAGTGGTCATCACGTTC	0.14283243	0	0.069847	0
tsrna-17106	TTTCCGTAGTGTAGTGGTCATCACGTT	0.28566487	0.07808	0.20954	0.333081
tsrna-17105	TTTCCGTAGTGTAGTGGTCATCAC	0.21424865	0.07808	0.139694	0.111027
tsrna-17104	TTTCCGTAGTGTAGTGGTCATC	0.14283243	0	0.139694	0
tsrna-17102	TTTCCGTAGTGTAGTGGT	0.07141622	0	0	0
tsrna-17101	TTTCCGTAGTGTAGTGG	0	0	0.069847	0
tsrna-17100	TTTCCGTAGTGTAGCGGTTATCACATTCGCCT	0.4284973	0.07808	0.349234	0.111027
tsrna-17099	TTTCCGTAGTGTAGCGGTTATCACATTCGCC	0.28566487	0.15616	0.349234	0
tsrna-17098	TTTCCGTAGTGTAGCGGTTATCACATTC	0.14283243	0.23424	0.139694	0
tsrna-17093	TTTCCCCGCACAGGTTCGAATC	0.21424865	0.07808	0	0
tsrna-17092	TTTCATATCATTGGTCGTGGTTGTAGTCCGTGCGAGAATACC/	11.3551784	12.25858	13.27089	5.662384
tsrna-17091	TTTCATATCATTGGTCGTGGTTGTAGTCCGTGCGAGAATACC	1.99965406	2.342403	1.676323	1.887461
tsrna-17090	TTTCATATCATTGGTCGTGGTTGTAGTCCGTGCGAGAATA	0.99982703	1.561602	1.257243	1.55438
tsrna-17089	TTTCATATCATTGGTCGTGGTTGTAGTCCGTGCGAGAAT	0.49991351	0.936961	0.768315	0.666163
tsrna-17088	TTTCATATCATTGGTCGTGGTTGTAGTCCGTGCGAGAA	0.4284973	1.405442	0.838162	1.443353
tsrna-17087	TTTCATATCATTGGTCGTGGTTGTAGTCCGTGCGAGA	0.07141622	1.171202	0.768315	1.998488
tsrna-17086	TTTCATATCATTGGTCGTGGTTGTAGTCCGTGCGAG	0.49991351	0.624641	0.838162	0.444109
tsrna-17085	TTTCATATCATTGGTCGTGGTTGTAGTCCG	0.21424865	0	0	0.111027
tsrna-17084	TTTCATATCATTGGTCGTGGTTGTAGTCC	0.14283243	0.07808	0.069847	0
tsrna-17083	TTTCATATCATTGGTCGTGGTTGTAGTC	0.14283243	0	0.069847	0.111027
tsrna-17082	TTTCATATCATTGGTCGTGGTTGTAGT	0	0	0	0
tsrna-17081	TTTCATATCATTGGTCGTGGTTGTAG	0.07141622	0	0	0
tsrna-17080	TTTCATATCATTGGTCGTGGTTGTA	0	0	0	0
tsrna-17079	TTTCATATCATTGGTCGTGGTTGT	0.07141622	0	0.069847	0
tsrna-17075	TTTCATATCATTGGTCGTG	0.07141622	0	0	0
tsrna-17073	TTTCATAACTTTGTCAAAGTTAAATTATAGGCT	0.28566487	0.624641	0.698468	0.111027
tsrna-17072	TTTCATAACTTTGTCAAAGTTAAA	0.14283243	0.15616	0.069847	0
tsrna-17071	TTTCATAACTTTGTCAAAGTTA	0.14283243	0.07808	0	0
tsrna-17069	TTTCACCGCCGCGGCCCGGGTTCGATTCCCGGTCAGGGAAC	2.64240001	25.06372	1.117549	0.333081
tsrna-17068	TTTCACCGCCGCGGCCCGGGT	0	0.07808	0	0
tsrna-17066	TTTCACCGCCGCGGCCC	0	0	0	0.111027
tsrna-17064	TTTCACCCAGGCGGCCCGGGTTCGACTCCCGGTGTGGGAAC	5.6418811	27.56228	3.701881	2.442597
tsrna-17063	TTTCACCCAGGCGGCCCGGGTTCGACTCCCGGTGTGGGAAC	0.64274595	1.327362	0.419081	0
tsrna-17062	TTTCACCCAGGCGGCCCGGGTTCGACTCCCGGTGTG	0.07141622	0	0	0
tsrna-17056	TTTCAACTTAACTTGACCGCTCTGACCA	1.35690811	2.420483	1.74617	1.332326

tsrna-17055	TTTCAACTTAACTTGACCGCTCTGACC	0.21424865	0.390401	0.139694	0
tsrna-17054	TTTCAACTTAACTTGACCGCTCTGAC	0.14283243	0.15616	0	0.111027
tsrna-17053	TTTCAACTTAACTTGACCGCTCTGA	0.07141622	0.07808	0	0
tsrna-17052	TTTCAACTTAACTTGACCGCTCTG	0	0.15616	0	0
tsrna-17051	TTTCAACTTAACTTGACCGCTCT	0	0.07808	0.069847	0
tsrna-17044	TTTATGTAGCTTACCTCCTCAAA	0.14283243	0	0	0.111027
tsrna-17043	TTTATGTAGCTTACCTCCTCA	0	0	0.069847	0
tsrna-17042	TTTATGTAGCTTACCTCCTC	0.07141622	0	0.069847	0
tsrna-17040	TTTATGTAGCTTACCTCC	0	0	0	0.111027
tsrna-17039	TTTATGTAGCTTACCTC	0	0	0	0.111027
tsrna-17038	TTTAGTGGTAGAATTCTCGCCT	0.07141622	0.468481	0.069847	0.444109
tsrna-17037	TTTAGTGGTAGAATTCTCGCC	0.14283243	0.468481	0.139694	0.222054
tsrna-17036	TTTAGTGGTAGAATTCTCGC	0	0	0	0
tsrna-17035	TTTAGTGGTAGAATTCTCG	0	0	0	0
tsrna-17032	TTTAGGCTCCAGTCTCTTCGGAGGCGTGGG	0.07141622	0	0	0
tsrna-17024	TTTAGGCTCCAGTCATTTCGATGGCGTGGG	0	0.15616	0.069847	0
tsrna-17016	TTTAGACGGGCTCACATCACCCCATAAACACCA	5.42763245	0.624641	3.073259	1.332326
tsrna-17015	TTTAGACGGGCTCACATCACCCCATAAACACC	0.99982703	0.15616	0.628621	0.222054
tsrna-17014	TTTAGACGGGCTCACATCACCCCATAAACAC	0.4284973	0.15616	0.628621	0.111027
tsrna-17013	TTTAGACGGGCTCACATCACCCCATAAACA	0.4284973	0.15616	0.419081	0
tsrna-17012	TTTAGACGGGCTCACATCACCCCATAAAC	0.4284973	0.15616	0.419081	0
tsrna-17011	TTTAGACGGGCTCACATCACCCCATAAA	0.49991351	0	0.419081	0
tsrna-17010	TTTAGACGGGCTCACATCACCCCATAA	0.35708108	0.07808	0.419081	0
tsrna-17009	TTTAGACGGGCTCACATCACCCCATA	0.14283243	0.07808	0.628621	0
tsrna-17008	TTTAGACGGGCTCACATCACCCCA	0	0	0.488928	0.111027
tsrna-17007	TTTAGACGGGCTCACATCACCCC	0.14283243	0	0.419081	0
tsrna-17006	TTTAGACGGGCTCACATCACCC	0.14283243	0.15616	0.279387	0
tsrna-17005	TTTAGACGGGCTCACATCACC	0.21424865	0.15616	0.558774	0
tsrna-17004	TTTAGACGGGCTCACATCAC	0.21424865	0	0.139694	0
tsrna-17003	TTTAGACGGGCTCACATCA	0.07141622	0	0.279387	0
tsrna-17002	TTTAGACGGGCTCACATC	0.21424865	0.07808	0.069847	0
tsrna-17001	TTTAGACGGGCTCACAT	0.14283243	0.15616	0.069847	0
tsrna-17000	TTTAGACGGGCTCACA	0.21424865	0	0	0.111027
tsrna-16999	TTTACAGTCAGAGGTTCA	0	0	0.139694	0
tsrna-16996	TTTACACGCAGAAGGTCCTGGGT	0.4284973	2.732804	0.349234	0.222054
tsrna-16995	TTTACACGCAGAAGGTCCTG	0	0.07808	0	0
tsrna-16993	TTTACACGCAGAAGGTCC	0.07141622	0	0	0.111027
tsrna-16992	TTTAATCTGAGGGTCCAGGGTTCAAGTCCCTGTTCGGGCGC(	1.35690811	3.279365	0.628621	0.111027
tsrna-16991	TTTAATCTGAGGGTCCAG	0.28566487	0.31232	0.139694	0
tsrna-16990	TTTAAGTTAAAGATTAAGAGAACCAACACCTCTTTACAGTGAC	0.4284973	0	0.977855	0.333081
tsrna-16989	TTTAAGTTAAAGATTAAGAGAACCAACACCTCTTTACAGTGAC	0.28566487	0.15616	0.419081	0
tsrna-16988	TTTAAGTTAAAGATTAAGAGAACCAACACCTCTTTACAGTGAC	0.35708108	0.07808	0.488928	0
tsrna-16987	TTTAAGTTAAAGATTAAGAGAACCAACACCT	0	0	0	0.222054
tsrna-16986	TTTAAGTTAAAGATTAAGAGAACCAACACC	0.07141622	0.07808	0	0
tsrna-16985	TTTAAGTTAAAGATTAAGAGAACC	0	0	0	0
tsrna-16968	TTTAAATTAGAATCTTAGCTTTGG	0.35708108	0	0.419081	0.222054
tsrna-16967	TTTAAAGGATAACAGCTATCCATTGGTC	0.07141622	0	0	0
tsrna-16966	TTGTGGGTTTAAGTCCCATTGGTCTAGCCA	0.14283243	0	0.069847	0
tsrna-16965	TTGTGGGTTTAAGTCCCATTGGTCTAGCC	0.07141622	0	0	0
tsrna-16964	TTGTGGGTTTAAGTCCCATTGGTCTAGC	0.07141622	0	0	0
tsrna-16963	TTGTGGGTTTAAGTCCCATTGGTCTAG	0	0.07808	0	0
tsrna-16957	TTGTGGGTTCGAATCCCACCAGAGTCGCC	0.07141622	0.23424	0	0
tsrna-16955	TTGTGGCCGCAGCAACCTCGGTT	0	0.07808	0	0
tsrna-16954	TTGTGGCCGCAGCAACCTCGGT	0.07141622	0	0	0
tsrna-16949	TTGTGGCCGCAGCAACC	0	0	0	0
tsrna-16948	TTGTGCTCTGCACGCGTGGGTTCG	0.21424865	0.702721	0	0
tsrna-16947	TTGTGAGTTCGATCCTCACCTGGAGCACC	0.14283243	0.546561	0.069847	0
tsrna-16946	TTGTGAGTTCGAGCCTCACCTGGAGCACC	0	0.702721	0.279387	0.111027
tsrna-16943	TTGTGAATCTGACAACAGAGGCTTACGACCCCTTATTTACCC(	1.07124325	0.780801	1.74617	0
tsrna-16942	TTGTGAATCTGACAACAGAGGCTTACGACCCCTTATTTACCC(	1.92823784	0.780801	0.558774	0
tsrna-16941	TTGTGAATCTGACAACAGAGGCTTACGACCCCTTATTTACCC	1.21407568	1.249282	1.047702	0
tsrna-16940	TTGTGAATCTGACAACAGAGGCTTACGACCCCTTATTTACC	1.2854919	0.546561	1.53663	0.111027
tsrna-16939	TTGTGAATCTGACAACAGAGGCTTACGACCCCTTAT	0.8569946	0.702721	0.558774	0
tsrna-16938	TTGTGAATCTGACAACAGAGGCTTACGACCCCT	0.07141622	0.31232	0.20954	0

tsrna-16937	TTGTGAATCTGACAACAGAGGCTTACGACCCC	0.07141622	0.07808	0	0
tsrna-16936	TTGTGAATCTGACAACAGAGGCTTACGACCC	0.07141622	0.23424	0	0
tsrna-16935	TTGTGAATCTGACAACAGAGGCTTACGACC	0	0.07808	0	0
tsrna-16934	TTGTGAATCTGACAACAGAGGCTTACGAC	0.07141622	0	0	0
tsrna-16933	TTGTGAATCTGACAACAGAGGCTTACGA	0.07141622	0	0	0
tsrna-16931	TTGTGAATCTGACAACAGAGGCTTAC	0	0.07808	0	0
tsrna-16929	TTGTGAATCTGACAACAGAGGCTT	0	0.07808	0	0
tsrna-16928	TTGTGAATCTGACAACAGAGGCT	0	0.07808	0	0
tsrna-16927	TTGTGAATCTGACAACAGAGGC	0	0.07808	0	0
tsrna-16926	TTGTGAATCTGACAACAGAGG	0	0	0.069847	0
tsrna-16925	TTGTGAATCTGACAACAGAG	0.07141622	0.07808	0.069847	0.111027
tsrna-16924	TTGTGAATCTGACAACAGA	0	0	0.069847	0
tsrna-16922	TTGTGAATCTGACAACA	0.07141622	0.07808	0	0
tsrna-16921	TTGTGAATCTGACAAC	0	0	0.069847	0
tsrna-16920	TTGTCAAAGTTAAATTATAGGCTAAATCCT	0.21424865	0	0.419081	0
tsrna-16919	TTGTCAAAGTTAAATTATAGGCT	0.07141622	0	0.349234	0.111027
tsrna-16918	TTGTCAAAGTTAAATTATAGG	0.14283243	0	0	0.111027
tsrna-16917	TTGTCAAAGTTAAATTATAG	0	0	0.139694	0
tsrna-16916	TTGTAGTTGAAATACAACGATGGTTTTTC	0	0	0	0
tsrna-16915	TTGTAGTTGAAATACAACGATGGTT	0.07141622	0	0.069847	0
tsrna-16912	TTGTAGTCCGTGCGAGAATACCA	0.07141622	0.15616	0	0.222054
tsrna-16911	TTGTAGTCCGTGCGAGAATACC	0.07141622	0	0	0
tsrna-16910	TTGTAGTCCGTGCGAGAATAC	0	0	0	0
tsrna-16909	TTGTAGTCCGTGCGAGAATA	0.07141622	0	0.069847	0
tsrna-16906	TTGTAGTCCGTGCGAGA	0	0	0	0
tsrna-16902	TTGTAAACCGGAGATGAAAACCTTTTTCCAAGGACACCA	2.28531893	3.045124	7.752995	0
tsrna-16901	TTGTAAACCGGAGATGAAAACCTTTTTCCAAGGACACC	0.57132973	1.093122	0.977855	0.111027
tsrna-16900	TTGTAAACCGGAGATGAAAACCTTTTTCCAAGGACA	0.35708108	0.858881	0.139694	0.333081
tsrna-16899	TTGTAAACCGGAGATGAAAACCTTTTTCCAAGGAC	0.21424865	0.390401	0.349234	0
tsrna-16898	TTGTAAACCGGAGATGAAAACCTTTTTCC	0.14283243	0.780801	0	0.111027
tsrna-16897	TTGTAAACCGGAGATGAAAACCTT	0	0	0	0
tsrna-16896	TTGTAAACCGGAGATGAAAACCT	0	0.07808	0	0.111027
tsrna-16894	TTGTAAACCGGAGATGAAAAC	0	0.07808	0	0
tsrna-16893	TTGTAAACCGGAGATGAAAA	0.07141622	0	0	0
tsrna-16888	TTGTAAACCAGGGGTCGCGAGTTCAA	2.99948109	7.964171	1.396936	1.443353
tsrna-16887	TTGTAAACCAGGGGTCGCGAGTTCA	1.99965406	7.808011	0.698468	1.665407
tsrna-16886	TTGTAAACCAGGGGTCGCGAGTT	2.14248649	7.26145	1.187396	1.665407
tsrna-16885	TTGTAAACCAGGGGTCGCGAGT	1.92823784	4.372486	0.768315	1.221299
tsrna-16884	TTGTAAACCAGGGGTCGCGAG	0.21424865	0.624641	0.139694	0
tsrna-16883	TTGTAAACCAGGGGTCGCGA	0.07141622	0	0.069847	0
tsrna-16882	TTGTAAACCAGGGGTCGCG	0	0.07808	0.069847	0
tsrna-16881	TTGTAAACCAGGGGTCGC	0.07141622	0	0.069847	0
tsrna-16880	TTGTAAACCAGGGGTCG	0	0.07808	0	0
tsrna-16879	TTGGTTATACCCTTCCCGTACTACCA	0.21424865	0.624641	0	0.111027
tsrna-16878	TTGGTTATACCCTTCCCGTACTACC	0.14283243	0.07808	0	0
tsrna-16877	TTGGTTATACCCTTCCCGTACTAC	0	0	0.069847	0
tsrna-16876	TTGGTTATACCCTTCCCGTACTA	0	0	0	0
tsrna-16868	TTGGTGGTTTAGTGGTAGAATTCTCGC	0	0	0	0
tsrna-16867	TTGGTGGTTTAGTGGTAGAATTCTCG	0	0	0	0
tsrna-16866	TTGGTGGTTTAGTGGTAGAATTCTC	0	0.07808	0	0
tsrna-16861	TTGGTGGTTTAGTGGTAGAA	0.07141622	0	0	0
tsrna-16859	TTGGTGGTTCGATCCCACCCAGGGACGCC	0	0.15616	0	0
tsrna-16857	TTGGTGGTTCGAGCCCATCCAGGGACGCC	0.07141622	0.546561	0	0
tsrna-16856	TTGGTGGTTCGAGCCCACCCAGGGACGCC	0.14283243	0.624641	0	0
tsrna-16855	TTGGTGGTTCGAGCCCACCCAGGGACG	0	0.15616	0	0.111027
tsrna-16854	TTGGTGGTTCGAGCCCACCCAGGGAC	0	0	0	0
tsrna-16848	TTGGTGGTTCAGTGGTAGAATTCTCGCCTGCCACGC	5.49904866	8.744972	2.095404	2.553624
tsrna-16847	TTGGTGGTTCAGTGGTAGAATTCTCGCCTGCCA	1.35690811	4.060166	0.908008	0.555136
tsrna-16846	TTGGTGGTTCAGTGGTAGAATTCTCGCCTGCC	1.49974054	2.888964	1.117549	0.555136
tsrna-16845	TTGGTGGTTCAGTGGTAGAATTCTCGCCTGC	0.78557838	1.015041	0.628621	0.666163
tsrna-16844	TTGGTGGTTCAGTGGTAGAATTCTCGCCTG	0.8569946	1.093122	0.488928	0.333081
tsrna-16843	TTGGTGGTTCAGTGGTAGAATTCTCGCCTCCC	1.21407568	1.483522	0.488928	1.443353
tsrna-16842	TTGGTGGTTCAGTGGTAGAATTCTCGCCTCC	0.92841081	1.639682	0.419081	1.221299
tsrna-16841	TTGGTGGTTCAGTGGTAGAATTCTCGCCTC	0.35708108	1.171202	0.349234	0.555136

tsrna-16840	TTGGTGGTTCAGTGGTAGAATTCTCGCCT	0.64274595	0.546561	0.349234	0.222054
tsrna-16839	TTGGTGGTTCAGTGGTAGAATTCTCGCC	0.49991351	0.780801	0.698468	0.222054
tsrna-16838	TTGGTGGTTCAGTGGTAGAATTCTCGC	0.28566487	0.23424	0.20954	0.111027
tsrna-16837	TTGGTGGTTCAGTGGTAGAATTCTCG	0.35708108	0.23424	0.20954	0
tsrna-16836	TTGGTGGTTCAGTGGTAGAATTCTC	0.4284973	0.624641	0.419081	0.111027
tsrna-16835	TTGGTGGTTCAGTGGTAGAATTCT	0.07141622	0.31232	0	0
tsrna-16834	TTGGTGGTTCAGTGGTAGAATTC	0.21424865	0.702721	0.279387	0.333081
tsrna-16833	TTGGTGGTTCAGTGGTAGAATT	0.28566487	0.15616	0.139694	0.222054
tsrna-16832	TTGGTGGTTCAGTGGTAGAAT	0.07141622	0.23424	0.20954	0
tsrna-16831	TTGGTGGTTCAGTGGTAGAA	0.07141622	0.07808	0.069847	0
tsrna-16830	TTGGTGGTTCAGTGGTAGA	0.14283243	0.07808	0.069847	0
tsrna-16826	TTGGTGGTGCAGTGGTAGAATTCTCGC	0	0	0	0.111027
tsrna-16825	TTGGTGGTATAGTGGTTAGCATAGCTGCC	0.07141622	0.07808	0	0.111027
tsrna-16824	TTGGTGGTATAGTGGTTAGCATAGCTGC	0	0	0	0.111027
tsrna-16823	TTGGTGGTATAGTGGTTAGCATAGCTG	0	0.07808	0	0
tsrna-16822	TTGGTGGTATAGTGGTTAGCATAGC	0	0	0	0
tsrna-16817	TTGGTGGTATAGTGGTGAGCATAGCTGC	0.07141622	0.23424	0	0.111027
tsrna-16816	TTGGTGGTATAGTGGTGAGCATAGCTG	0	0.07808	0	0
tsrna-16815	TTGGTGGTATAGTGGTGAGCATAGCT	0	0.15616	0	0
tsrna-16813	TTGGTGGTATAGTGGTGAGCATAG	0	0.07808	0	0
tsrna-16812	TTGGTGGTATAGTGGTGAGCATA	0	0.07808	0	0
tsrna-16811	TTGGTGGTATAGTGGTGAGCAT	0	0	0	0
tsrna-16810	TTGGTGGTATAGTGGTGAGCA	0	0.15616	0	0
tsrna-16806	TTGGTGGTATAGTGGTAAGCATAG	0	0.07808	0	0
tsrna-16805	TTGGTGGTATAGTGGTAAGCATA	0	0	0.069847	0
tsrna-16803	TTGGTGCAACTCCAAATAAAAGTACCA	0	0.23424	0	0
tsrna-16798	TTGGTCTAGTGGTATGATTCTCGCTT	0.49991351	0.23424	0.069847	0.555136
tsrna-16797	TTGGTCTAGTGGTATGATTCTCGC	0.28566487	0.07808	0.139694	0.222054
tsrna-16795	TTGGTCTAGGGGTATGATTCTCGGTTTG	0.07141622	0.15616	0.069847	0.111027
tsrna-16794	TTGGTCTAGGGGTATGATTCTCGGTTT	0.35708108	0.31232	0	0.111027
tsrna-16793	TTGGTCTAGGGGTATGATTCTCGGTT	0	0.390401	0.139694	0.222054
tsrna-16792	TTGGTCTAGGGGTATGATTCTCGGT	0.21424865	0.23424	0	0
tsrna-16791	TTGGTCTAGGGGTATGATTCTCGG	0.07141622	0	0.069847	0
tsrna-16790	TTGGTCTAGGGGTATGATTCTCGCTTT	2.64240001	1.639682	1.327089	2.442597
tsrna-16789	TTGGTCTAGGGGTATGATTCTCGCTTCG	2.14248649	2.108163	0.908008	1.665407
tsrna-16788	TTGGTCTAGGGGTATGATTCTCGCTTC	1.78540541	2.186243	0.628621	1.887461
tsrna-16787	TTGGTCTAGGGGTATGATTCTCGCTT	1.14265946	2.030083	1.187396	2.442597
tsrna-16786	TTGGTCTAGGGGTATGATTCTCGCT	1.78540541	1.717762	0.838162	1.55438
tsrna-16785	TTGGTCTAGGGGTATGATTCTCGC	0.71416216	1.327362	0.698468	0.999244
tsrna-16784	TTGGTCTAGGGGTATGATTCTCG	0	0.07808	0	0
tsrna-16782	TTGGTCTAGGGGTATGATTCT	0	0.07808	0	0
tsrna-16776	TTGGTCGTGGTTGTAGTCCGTGCGAGAATACCA	4.99913515	8.823052	6.146519	2.886706
tsrna-16775	TTGGTCGTGGTTGTAGTCCGTGCGAGAATACC	0.99982703	1.717762	0.768315	0.888217
tsrna-16774	TTGGTCGTGGTTGTAGTCCGTGCGAGAATAC	0.57132973	0.780801	0.279387	0.77719
tsrna-16773	TTGGTCGTGGTTGTAGTCCGTGCGAGAATA	0.14283243	0.624641	0.20954	0.555136
tsrna-16772	TTGGTCGTGGTTGTAGTCCGTGCGAGAAT	0.14283243	0.468481	0.419081	0.666163
tsrna-16771	TTGGTCGTGGTTGTAGTCCGTGCGAGAA	0.21424865	0.624641	0.349234	0.111027
tsrna-16770	TTGGTCGTGGTTGTAGTCCGTGCGAGA	0.14283243	0.702721	0.349234	0.555136
tsrna-16769	TTGGTCGTGGTTGTAGTCCGTGCGAG	0.07141622	0.468481	0.139694	0.111027
tsrna-16768	TTGGTCGTGGTTGTAGTCCGTGCGA	0.07141622	0.31232	0.20954	0.111027
tsrna-16767	TTGGTCGTGGTTGTAGTCCGTGCG	0.14283243	0.31232	0.069847	0.111027
tsrna-16766	TTGGTCGTGGTTGTAGTCCGTGC	0.07141622	0.23424	0	0
tsrna-16765	TTGGTCGTGGTTGTAGTCCGTG	0	0.23424	0.139694	0.222054
tsrna-16764	TTGGTCGTGGTTGTAGTCCGT	0	0	0	0
tsrna-16763	TTGGTCGTGGTTGTAGTCCG	0	0	0	0.111027
tsrna-16762	TTGGTCGTGGTTGTAGTCC	0	0	0	0
tsrna-16761	TTGGTCGTGGTTGTAGTC	0	0.07808	0	0
tsrna-16759	TTGGTCGTGGTTGTAG	0	0	0	0
tsrna-16747	TTGGTAGAGCATCAGAC	0.07141622	0	0	0
tsrna-16746	TTGGTAGAGCATCAGA	0.14283243	0	0	0
tsrna-16740	TTGGGTGCTAATGGTGGAGTTAAAGACTTTTTCTCTGACCA	2.71381622	3.357445	1.396936	2.664651
tsrna-16739	TTGGGTGCTAATGGTGGAGTTAAAGACTTTTTCTCTGACC	0.57132973	1.639682	0.419081	0.222054
tsrna-16738	TTGGGTGCTAATGGTGGAGTTAAAGACTTTTTCTCT	0.57132973	1.405442	0.20954	0
tsrna-16737	TTGGGTGCTAATGGTGGAGTTAAAGACTTT	0.21424865	0.390401	0.20954	0.111027

tsrna-16736	TTGGGTGCTAATGGTGGAGTTAAAGACT	0.21424865	0.390401	0.279387	0.111027
tsrna-16735	TTGGGTGCTAATGGTGGAGTTAAAGAC	0	0	0	0.111027
tsrna-16734	TTGGGTGCTAATGGTGGAGTTAAAGA	0.14283243	0.546561	0.139694	0
tsrna-16733	TTGGGTGCTAATGGTGGAGTTAAAG	0.14283243	0.31232	0	0
tsrna-16732	TTGGGTGCTAATGGTGGAGTTAAA	0	0.31232	0	0.111027
tsrna-16731	TTGGGTGCTAATGGTGGAGTTAA	0.07141622	0.07808	0	0.111027
tsrna-16730	TTGGGTGCTAATGGTGGAGTTA	0.21424865	0.15616	0	0.111027
tsrna-16729	TTGGGTGCTAATGGTGGAGTT	0	0	0	0
tsrna-16723	TTGGGTGCGAGAGGTCCCGGGTT	0.28566487	5.153287	0.069847	2.33157
tsrna-16722	TTGGGTGCGAGAGGTCCCGGGT	0.57132973	3.045124	0.349234	1.776434
tsrna-16721	TTGGGTGCGAGAGGTCCCGGG	0	0.07808	0	0
tsrna-16719	TTGGGTGCGAGAGGTCCCG	0	0.07808	0	0
tsrna-16718	TTGGGTGCGAGAGGTCCC	0	0.15616	0	0
tsrna-16716	TTGGGTCCGAGAGGTCCCGGGTT	0	0.15616	0	0
tsrna-16709	TTGGGGTTTCCCCGCGCAGGT	0	0.23424	0	0
tsrna-16708	TTGGGGGGTTCGATTCCTTCCTTTTTTGCC	0	0.468481	0.069847	0.111027
tsrna-16707	TTGGGGGGTTCGATTCCTTCCTTTTTTGC	0	0.31232	0	0
tsrna-16706	TTGGGGGGTTCGATTCCTTCCTTTTTTG	0.07141622	0.23424	0.069847	0
tsrna-16705	TTGGGGGGTTCGATTCCTTC	0.07141622	0	0	0
tsrna-16704	TTGGGGGGTTCGATTCCTT	0	0	0	0
tsrna-16703	TTGGGGGGTTCGATTCCT	0	0	0	0
tsrna-16702	TTGGGGGGTTCGATTCC	0	0	0.139694	0
tsrna-16699	TTGGGAGAGCGTTAGACTGAAGAT	0	0	0	0.111027
tsrna-16698	TTGGGAGAGCGTTAGACTGAAGA	0	0.07808	0	0
tsrna-16696	TTGGGAGAGCGTTAGACTGAA	0	0.07808	0	0
tsrna-16692	TTGGCCTCCTAAGCCAGGGATTGTGG	0.07141622	0.23424	0.279387	0
tsrna-16691	TTGGATTCTCAGGGATGGGTTCGATTCTCATAGTCCTAGCCA	0.78557838	1.795843	1.327089	2.442597
tsrna-16690	TTGGATTCTCAGGGATGGGTTCG	0.07141622	0.15616	0.069847	0
tsrna-16689	TTGGACTTCTAATTCAAAGGTTGTGGGT	0.92841081	3.747845	0.698468	0.444109
tsrna-16686	TTGGACTGTAAATCTAAAGACAGGGGTTAGGCCTCT	0.35708108	0.546561	0.20954	0.333081
tsrna-16685	TTGGACTGTAAATCTAAAGACAGGGGTTAG	0	0.07808	0	0
tsrna-16684	TTGGACTGTAAATCTAAAGACAGGGGTT	0	0	0	0
tsrna-16682	TTGGACTGTAAATCTAAAGACAGGGG	0	0	0.069847	0
tsrna-16673	TTGCGTTCAGTTGATGCAGAGTGGGGTTTT	0.14283243	0.15616	0.069847	0.222054
tsrna-16672	TTGCGTTCAGTTGATGCAGAGTGGG	0	0.15616	0.069847	0
tsrna-16670	TTGCGTTCAGTTGATGCAGAGTG	0	0	0.20954	0
tsrna-16668	TTGCGTTCAGTTGATGCAGAG	0	0	0.069847	0
tsrna-16662	TTGCGTGTTCAAGTCACGTCGGGGTCACC	0.07141622	0.23424	0	0
tsrna-16655	TTGCGTGTTCAAATCACGTCGGGGTCACC	0	0.15616	0	0
tsrna-16651	TTGCGACCCGGGTTCGATTCCCGGGCGGCGCACCA	1.2854919	5.153287	0.069847	0
tsrna-16650	TTGCGACCCGGGTTCGATTCCCGGGCGGCGCACC	0.4284973	1.015041	0	0
tsrna-16649	TTGCGACCCGGGTTCGATTCCCGGGC	0.07141622	0.07808	0	0
tsrna-16648	TTGCGACCCGGGTTCGATTCCCGGG	0	0.468481	0	0
tsrna-16646	TTGCATGTATGAGGCCTCGGGT	0.28566487	0.858881	0.139694	0.111027
tsrna-16645	TTGCATGTATGAGGCCTC	0	0.07808	0	0
tsrna-16644	TTGCATGTATGAGGCCCCGGGT	0.21424865	0.390401	0	0
tsrna-16641	TTGCACGTATGAGGCCCCGGGTTCA	0.4284973	1.015041	0.20954	0.222054
tsrna-16640	TTGCACGTATGAGGCCCCGGGTTC	0.21424865	0.702721	0.20954	0.666163
tsrna-16639	TTGCACGTATGAGGCCCCGGGTT	0.4284973	0.780801	0	0.111027
tsrna-16638	TTGCACGTATGAGGCCCCGGGT	0.4284973	0.624641	0.139694	0
tsrna-16637	TTGCACGTATGAGGCCCCGGG	0.07141622	0	0	0
tsrna-16636	TTGCACGTATGAGGCCCCGG	0	0.07808	0	0
tsrna-16632	TTGCACGCGTGGGTTCGAATCCCATCCTCGTCGC	0	0.07808	0.069847	0
tsrna-16631	TTGCACGCGTGGGTTCGAAT	0	0.15616	0	0
tsrna-16630	TTGCAATACTTAATTTCTGCCA	0.21424865	0.07808	0	0
tsrna-16629	TTGCAATACTTAATTTCTGCC	0.07141622	0	0.069847	0
tsrna-16628	TTGCAATACTTAATTTCTGC	0.07141622	0	0	0
tsrna-16625	TTGCAAATTCGAAGAAGCAGCTTCAAACCTGCCGGGGCTT	0.07141622	0.07808	0.069847	0
tsrna-16622	TTGCAAATTCGAAGAAGCAGCTT	0.07141622	0	0	0
tsrna-16617	TTGATTAGGGTGCTTAGCTGTTAACTAAGTGTTTGT	0.49991351	0	0.488928	0
tsrna-16616	TTGATTAGGGTGCTTAGCTGTTAAC	0.07141622	0.07808	0.139694	0
tsrna-16615	TTGATTAGGGTGCTTAGCTGTTA	0.14283243	0.07808	0.139694	0
tsrna-16614	TTGATTAGGGTGCTTAGCTGTT	0.07141622	0.07808	0.069847	0
tsrna-16613	TTGATTAGGGTGCTTAGCTGT	0.07141622	0.07808	0	0

tsrna-16612	TTGATTAGGGTGCTTAGCTG	0	0	0	0
tsrna-16608	TTGATGCAGAGTGGGGTTTTGCAGTCC	0.14283243	0.07808	0.069847	0
tsrna-16607	TTGATGCAGAGTGGGGTTTTGCAGTC	0	0	0	0
tsrna-16606	TTGATGCAGAGTGGGGTTTTGCA	0	0	0	0
tsrna-16605	TTGATGCAGAGTGGGGTTTTGC	0	0	0	0
tsrna-16604	TTGATGCAGAGTGGGGTTTTG	0	0	0	0
tsrna-16603	TTGATAGAGTAAATAATAGGAGCTTAAACCC	0.07141622	0.07808	0.139694	0.111027
tsrna-16602	TTGATAGAGTAAATAATAGGAGCT	0	0	0	0
tsrna-16596	TTGACTGCAGATCAAGAGGTCCCTGGTT	0.28566487	0.936961	0.20954	0.77719
tsrna-16595	TTGACTGCAGATCAAGAGGTCCCTGGT	0.07141622	0.23424	0.069847	0.444109
tsrna-16594	TTGACTGCAGATCAAGAGGTCCCTGG	0.07141622	0.15616	0.069847	0
tsrna-16593	TTGACTGCAGATCAAGAGGTCCCT	0	0.07808	0	0.111027
tsrna-16592	TTGACTGCAGATCAAGAGGTCCCCGGT	0.21424865	0.468481	0.139694	0.222054
tsrna-16591	TTGACTGCAGATCAAGAGGTCCCCGG	0.07141622	0.15616	0	0
tsrna-16590	TTGACTGCAGATCAAGAGGTCCCCG	0	0.15616	0	0
tsrna-16589	TTGACTGCAGATCAAGAGGTCCCCAGT	0	0.07808	0.069847	0
tsrna-16587	TTGACTGCAGATCAAGAGGTCCC	0	0	0.069847	0
tsrna-16585	TTGACTGCAGATCAAGAG	0	0.07808	0	0
tsrna-16582	TTGACTGCAGATCAAAAGGTCCCTGGT	0.49991351	0.780801	0.20954	0.222054
tsrna-16581	TTGACCGCTCTGACCA	0	0.07808	0	0.111027
tsrna-16579	TTGACAACATTCAAAAAAGAGTACCA	0	0.07808	0	0
tsrna-16576	TTGAATTGCAAATTCGAAGAAGCAGCTTCAAACCTGCCGGGG	0	0.23424	0.069847	0
tsrna-16575	TTGAATTGCAAATTCGAAGAAGCAGCTTCAAACCTGCCGGGG	0	0.390401	0.069847	0
tsrna-16574	TTGAATCCCACCGCTGCCACCA	0	0.07808	0	0
tsrna-16573	TTGAATCCAGCGATCCGAGTT	0	0.07808	0	0
tsrna-16569	TTGAAGCCAGTTGATTAGGGTGCTTAGCTG	0	0	0	0
tsrna-16567	TTGAAATCCATTGGGGTTTCC	0.07141622	4.997127	0.069847	0.222054
tsrna-16566	TTGAAATCCATTGGGGTTTC	0	5.231367	0.069847	0.111027
tsrna-16565	TTGAAATCCATTGGGG	0.07141622	0.468481	0	0
tsrna-16564	TTGAAATACAACGATGGTTTTTCATATC	0	0.15616	0	0
tsrna-16563	TTGAAACCAGCTTTGGGGGGTTCGA	0.07141622	0.07808	0	0
tsrna-16562	TTGAAACCAGCTTTGGGGGGTTCG	0	0.15616	0	0
tsrna-16561	TTGAAACCAGCTTTGGGGGGTTC	0	0.07808	0.139694	0
tsrna-16560	TTGAAACCAGCTTTGGGGGGTT	0	0.07808	0	0
tsrna-16559	TTGAAACCAGCTTTGGGGGGT	0	0.07808	0	0
tsrna-16557	TTCTTGCGACCCGGGTTCGATTCCCGGGCGGCGCACC	0.35708108	0.858881	0.139694	0
tsrna-16556	TTCTTGCGACCCGGGTTCGATTCCCGGGCGGCG	0	0.31232	0	0
tsrna-16555	TTCTTGCGACCCGGGTTCGATTCCCGGGCGGC	0	0.31232	0.069847	0
tsrna-16554	TTCTTGCGACCCGGGTTCGATTCCCGGGCG	0.07141622	0.390401	0	0
tsrna-16553	TTCTTGCGACCCGGGTTCGA	0	0.07808	0	0
tsrna-16552	TTCTTGCGACCCGGGTTCG	0	0.07808	0	0
tsrna-16551	TTCTTGCGACCCGGGTTC	0	0.07808	0	0
tsrna-16548	TTCTGTAGTGTAGTGGTTATCACGTTCGCCT	0	0.07808	0	0
tsrna-16547	TTCTGTAGTGTAGTGGTTATCACGTTCGCC	0	0	0	0
tsrna-16545	TTCTGTAGTGTAGTGGTTATCACGTTCG	0	0	0	0
tsrna-16543	TTCTGTAGTGTAGTGGTTATCACGTT	0.07141622	0	0	0
tsrna-16541	TTCTGTAGTGTAGTGGTTATCACG	0	0	0.069847	0
tsrna-16539	TTCTGTAGTGTAGTGGTTATC	0	0	0	0
tsrna-16535	TTCTGGTCTCCGGATGGAGGCGTGGGTTCGAATCCC	0.99982703	4.840967	0.419081	0.222054
tsrna-16534	TTCTGGTCTCCGGATGGAGGCGTGGGTTCG	0.71416216	3.825925	0.20954	0.333081
tsrna-16533	TTCTGGTCTCCGGATGGAGGCGTGGG	0.07141622	0.390401	0.139694	0
tsrna-16532	TTCTGGTCTCCGGATGGAGGCGTGG	0	0.31232	0.069847	0
tsrna-16531	TTCTGGTCTCCGGATGGAGGCGT	0	0.23424	0.20954	0
tsrna-16530	TTCTGGTCTCCGGATGGAGGCG	0	0.07808	0	0
tsrna-16525	TTCTGGTCTCCGGATGG	0	0	0	0.111027
tsrna-16523	TTCTGGTCTCCAATGGAGGCGTGGG	0.14283243	0.15616	0.139694	0.111027
tsrna-16521	TTCTGGTCTCCAATGGAGGCGTG	0	0.07808	0	0
tsrna-16520	TTCTGGTCTCCAATGGAGGCG	0	0.07808	0	0
tsrna-16510	TTCTCGCTGGGGCCTCCA	1.92823784	3.201285	0.908008	1.110271
tsrna-16509	TTCTCGCTGGGGCCTCC	0.07141622	0.546561	0	0.111027
tsrna-16508	TTCTCGCCTGCCACGCGGGAGGCCCGGGT	0.35708108	2.576644	0.349234	0.222054
tsrna-16507	TTCTCGCCTGCCACGCGGGAGGCCCGG	0	0.546561	0	0
tsrna-16506	TTCTCGCCTGCCACGCGGGAGGCCCG	0.07141622	0.31232	0.139694	0
tsrna-16505	TTCTCGCCTGCCACGCGGGAGGCCC	0	0.468481	0.069847	0

tsrna-16504	TTCTCGCCTGCCACGCGGGA	0.07141622	0.31232	0.069847	0
tsrna-16503	TTCTCGCCTGCCACGCGG	0.07141622	0.624641	0	0.111027
tsrna-16502	TTCTCGCCTGCCACGCG	0	0.390401	0	0
tsrna-16501	TTCTCGCCTGCCACGC	0.07141622	0.31232	0	0
tsrna-16500	TTCTCATAGTCCTAGCCA	0.07141622	0	0	0
tsrna-16499	TTCTCATAGTCCTAGCC	0	0.07808	0	0
tsrna-16498	TTCTCATAGTCCTAGC	0	0	0	0.111027
tsrna-16497	TTCTCAGGGATGGGTTCGATTCTCATAGTCCTAGCCA	1.07124325	1.483522	1.117549	2.109516
tsrna-16496	TTCTCAGGGATGGGTTCGATTCT	0.28566487	0.390401	0.419081	0
tsrna-16495	TTCTCAGGGATGGGTTCGATTC	0.21424865	0.23424	0.20954	0
tsrna-16494	TTCTCAGGGATGGGTTCG	0	0.07808	0	0
tsrna-16493	TTCTAGGTTCGACTCCTGGCTGGCTCGC	0.07141622	0	0	0
tsrna-16492	TTCTAGGTTCGACTCCTGGCTGGCTCG	0	0	0	0
tsrna-16491	TTCTAATTCAAAGGTTGTGGGTTCGAGTCC	0.28566487	0.702721	0	0.333081
tsrna-16490	TTCTAATTCAAAGGTTGTGGGTTCGAATCC	0.4284973	0.546561	0.20954	0.222054
tsrna-16489	TTCTAATTCAAAGGTTGTGGGT	0	0.23424	0	0
tsrna-16486	TTCTAATTCAAAGGTTCCGGGTTCGAGTCCCGGCGGAGTCG(	0.21424865	0.858881	0	0.111027
tsrna-16485	TTCTAATTCAAAGGTTCCGGGTTCGAGTCC	0	0.23424	0	0.111027
tsrna-16484	TTCTAATTCAAAGGTTCCGGGTT	0.07141622	0.23424	0	0.222054
tsrna-16483	TTCTAATTCAAAGGTTCCGGGT	0.07141622	0.15616	0	0
tsrna-16477	TTCGGGTGTGAGAGGTCCCGGGT	0	0.15616	0	0
tsrna-16475	TTCGGGTGCGAGAGGTCCCGGGTT	0	0.546561	0.139694	0
tsrna-16474	TTCGGGTGCGAGAGGTCCCGGGT	0.07141622	0.15616	0	0
tsrna-16473	TTCGGGTGCGAGAGGTCCCGG	0.07141622	0.07808	0	0
tsrna-16471	TTCGGGTGCGAGAGGTCCC	0.07141622	0	0	0
tsrna-16469	TTCGGCTGTTAACCGA	0.49991351	0.702721	0.139694	0
tsrna-16468	TTCGGCGCTCTCACCGCCGCGGCCCGGGTTCGATTCC	0	0	0	0
tsrna-16467	TTCGGCGCTCTCACCGCCGCGGCCCGGGTTCGATTC	0	0	0	0
tsrna-16466	TTCGGCGCTCTCACCGCCGCGGCCCGGGTTCGATT	0.07141622	0.23424	0	0
tsrna-16465	TTCGGCGCTCTCACCGCCGCGGCCCGGGTTCGAT	0	0.23424	0	0.222054
tsrna-16464	TTCGGCGCTCTCACCGCCGCGGCCCGGGTTCG	0	0.07808	0	0
tsrna-16463	TTCGGCGCTCTCACCGCCGCGGCCCGGGTTC	0	0.15616	0.069847	0
tsrna-16462	TTCGGCGCTCTCACCGCCGCGGCCCGGGTT	0	0.07808	0.069847	0
tsrna-16461	TTCGGCGCTCTCACCGCCGCGGCCCGGGT	0	0	0	0
tsrna-16460	TTCGGCGCTCTCACCGCCGCGGCCCGGG	0	0.31232	0	0
tsrna-16459	TTCGGCGCTCTCACCGCCGCGGCCCGG	0	0.07808	0	0
tsrna-16458	TTCGGCGCTCTCACCGCCGCGGCCCG	0	0	0.069847	0.111027
tsrna-16457	TTCGGCGCTCTCACCGCCGCGGCCC	0	0	0	0
tsrna-16456	TTCGGCGCTCTCACCGCC	0	0	0.069847	0
tsrna-16453	TTCGGATCAGAAGATTGCAGGT	0	0	0	0
tsrna-16450	TTCGGATCAGAAGATTGAGGGT	0.21424865	0.15616	0.139694	0
tsrna-16447	TTCGATTCTCATAGTCCTAGCCA	0	0	0	0
tsrna-16446	TTCGATTCTCATAGTCCTAGCC	0	0.07808	0	0
tsrna-16445	TTCGATTCTCATAGTCCTAGC	0	0	0.069847	0
tsrna-16443	TTCGATTCCTTCCTTTTTTGCCA	0	0	0	0
tsrna-16442	TTCGATTCCTTCCTTTTTTGCC	0	0	0	0
tsrna-16440	TTCGATTCCTTCCTTTTTTG	0	0	0	0
tsrna-16439	TTCGATTCCGGCTCGAAGGACCA	5.49904866	12.72706	1.816017	2.442597
tsrna-16438	TTCGATTCCGGCTCGAAGGACC	0.07141622	0.858881	0.069847	0
tsrna-16437	TTCGATTCCGGCTCGAAGGAC	0	0	0	0
tsrna-16434	TTCGATTCCCGGTCAGGGAACCA	0.8569946	23.50211	0.20954	0
tsrna-16433	TTCGATTCCCGGTCAGGGAACC	0.07141622	0.546561	0	0
tsrna-16432	TTCGATTCCCGGTCAGGGAAC	0	0.468481	0	0
tsrna-16431	TTCGATTCCCGGTCAGGGAA	0	0.07808	0	0
tsrna-16430	TTCGATTCCCGGTCAGGGA	0	0.07808	0	0
tsrna-16426	TTCGATTCCCGGGCGGCGCACCA	0.99982703	4.528646	0.488928	0.333081
tsrna-16425	TTCGATTCCCGGGCGGCGCACC	0.28566487	0.546561	0.069847	0
tsrna-16424	TTCGATTCCCGGGCGGCGCAC	0.07141622	0	0	0
tsrna-16418	TTCGATTCCCGGCCCATGCACCA	1.42832433	3.045124	0.558774	1.998488
tsrna-16417	TTCGATTCCCGGCCCATGCACC	0.35708108	0.23424	0.139694	0.222054
tsrna-16415	TTCGATTCCCGGCCAATGCACCA	2.78523244	10.61889	0.279387	1.110271
tsrna-16414	TTCGATTCCCGGCCAATGCACC	0.21424865	1.015041	0.139694	0
tsrna-16413	TTCGATTCCCGGCCAATGCAC	0	0.15616	0	0
tsrna-16411	TTCGATTCCCGGCCAATGC	0	0.07808	0	0

tsrna-16410	TTCGATTCCCGGCCAACGCACCA	3.42797839	7.651851	1.955711	1.998488
tsrna-16409	TTCGATTCCCGGCCAACGCACC	0.28566487	1.093122	0.20954	0.333081
tsrna-16408	TTCGATTCCCGGCCAACGCAC	0	0.15616	0	0
tsrna-16407	TTCGATTCCCGGCCAACGCA	0	0.07808	0	0
tsrna-16406	TTCGATTCCCGGCCAACGC	0	0.07808	0	0
tsrna-16405	TTCGATTCCCCGACGGGGAGCCA	0.07141622	0.936961	0	0
tsrna-16404	TTCGATTCCCCGACGGGGAGCC	0	0.07808	0	0
tsrna-16403	TTCGATTCCCCGACGGGGAGC	0	0.07808	0	0
tsrna-16397	TTCGATTCCAGCTCGAAGGACCA	0	0	0	0
tsrna-16395	TTCGATCCTCGCTGGGGCCTCCA	0.71416216	0.702721	0	0.222054
tsrna-16394	TTCGATCCTCACCTGGAGCACCA	0.07141622	0.31232	0.069847	0
tsrna-16393	TTCGATCCTCACCTGGAGCACC	0	0.07808	0	0
tsrna-16392	TTCGATCCTCACACGGGGCACCA	0	0.390401	0	0
tsrna-16390	TTCGATCCCGGGTTTCGGCACCA	2.07107028	7.729931	0.908008	1.110271
tsrna-16389	TTCGATCCCGGGTTTCGGCACC	0.35708108	0.858881	0.069847	0.222054
tsrna-16383	TTCGATCCCGGGCGGAAACACCA	1.57115676	4.840967	0.977855	0.555136
tsrna-16381	TTCGATCCCCGTACTGGCCACCA	0.28566487	0.546561	0.419081	0
tsrna-16380	TTCGATCCCCGTACGGGCCACCA	0.78557838	4.997127	0.349234	0.222054
tsrna-16379	TTCGATCCCCGTACGGGCCACC	0.07141622	0.15616	0.069847	0
tsrna-16377	TTCGATCCCCGGCATCTCCACCA	17.2827244	28.73348	8.730851	8.54909
tsrna-16376	TTCGATCCCCGGCATCTCCACC	0.64274595	1.639682	0.349234	0.222054
tsrna-16369	TTCGATCCCCGGCACCTCCACCA	9.42694057	21.94051	5.448051	3.996977
tsrna-16368	TTCGATCCCCGACACCTCCACCA	0.78557838	2.108163	0	0.77719
tsrna-16367	TTCGATCCCCAGTACCTCCACCA	2.64240001	3.669765	1.187396	1.887461
tsrna-16366	TTCGATCCCCAGCATCTCCACCA	0.92841081	1.249282	0.20954	0.222054
tsrna-16365	TTCGATCCCCAGCATCTCCACC	0	0.07808	0	0
tsrna-16362	TTCGATCCCACCCAGGGACGCCA	0.21424865	0.546561	0	0
tsrna-16361	TTCGATAGCTCAGTTGGTAGAGCGGAGGAC	0	0.15616	0	0
tsrna-16360	TTCGATAGCTCAGTTGGTAGA	0	0	0.069847	0.111027
tsrna-16359	TTCGATAGCTCAGCTGGTAGAGCGGAGGACT	0.14283243	0	0	0
tsrna-16358	TTCGATAGCTCAGCTGGTAGA	0.21424865	0.15616	0.139694	0
tsrna-16357	TTCGAGTCTCGGTGGAACCTCCA	4.28497299	11.24354	1.606477	3.219787
tsrna-16356	TTCGAGTCTCGGTGGAACCTCC	0.14283243	1.015041	0.20954	0
tsrna-16355	TTCGAGTCTCGGTGGAACCTC	0	0	0.069847	0
tsrna-16351	TTCGAGTCCTGCCGCGGTCGCCA	0.07141622	0.546561	0.139694	0
tsrna-16346	TTCGAGTCCCTTCGTGGTCGCCA	0.07141622	0.15616	0	0
tsrna-16345	TTCGAGTCCCGGCGGAGTCGCCA	0	0.31232	0.069847	0
tsrna-16340	TTCGAGTCCCATCTGGGTCGCCA	0.07141622	0.702721	0.069847	0
tsrna-16339	TTCGAGTCCCATCTGGGGTGCCA	0.07141622	0.23424	0	0
tsrna-16337	TTCGAGTCCCACCTGGGGTGCCA	0.07141622	0.468481	0	0
tsrna-16336	TTCGAGTCCCACCAGAGTCGCCA	0	0.546561	0	0
tsrna-16335	TTCGAGCCTCAGAGAGGGCACCA	0	0.07808	0	0
tsrna-16334	TTCGAGCCTCACCTGGAGCACCA	0.07141622	0.23424	0	0.111027
tsrna-16333	TTCGAGCCTCACCTGGAGCACC	0	0.15616	0	0
tsrna-16330	TTCGAGCCTCACCTGGAGC	0	0.07808	0	0
tsrna-16329	TTCGAGCCTCACCTGGA	0	0.07808	0	0
tsrna-16327	TTCGAGCCCCAGTGGAACCACCA	2.28531893	2.888964	1.74617	1.55438
tsrna-16326	TTCGAGCCCCAGTGGAACCACC	0.28566487	0.390401	0	0
tsrna-16325	TTCGAGCCCCAGTGGAACCAC	0.07141622	0	0	0
tsrna-16324	TTCGAGCCCCAGTGGAACCA	0	0	0	0
tsrna-16323	TTCGAGCCCCAGTGGAACC	0	0.07808	0	0
tsrna-16322	TTCGAGCCCCACGTTGGGCGCCA	0.78557838	16.0845	0.20954	0.111027
tsrna-16317	TTCGAGCCCACCCAGGGACGCCA	0.21424865	6.324489	0.139694	0
tsrna-16316	TTCGAGCCCACCCAGGGACGCC	0	0.07808	0	0.111027
tsrna-16312	TTCGACTCCTGGCTGGCTCGCCA	2.92806487	9.135373	0.838162	0.444109
tsrna-16311	TTCGACTCCTGGCTGGCTCGCC	0.07141622	0.546561	0	0.111027
tsrna-16310	TTCGACTCCTGGCTGGCTCGC	0.07141622	0	0	0
tsrna-16305	TTCGACTCCCGGTGTGGGAACCA	5.2133838	21.86243	1.676323	1.998488
tsrna-16304	TTCGACTCCCGGTGTGGGAACC	0.35708108	0.23424	0	0
tsrna-16303	TTCGACTCCCGGTGTGGGAAC	0	0.23424	0	0
tsrna-16297	TTCGACTCCCGGTATGGGAACCA	0.57132973	5.153287	0.488928	0.333081
tsrna-16296	TTCGACTCCCGGTATGGGAAC	0	0.15616	0	0
tsrna-16293	TTCGACTCATTAAATTATGATAATCATATT	0.14283243	0	0.069847	0
tsrna-16291	TTCGAATCTCGGTGGGACCTCCA	3.9278919	7.26145	1.53663	2.109516

tsrna-16290	TTCGAATCCTGTTCGTGACGCCA	0.28566487	0.702721	0.20954	0.222054
tsrna-16289	TTCGAATCCTGTCGGCTACGCCA	0.35708108	1.015041	0.488928	0.888217
tsrna-16288	TTCGAATCCTGCTCACAGCGCCA	0.28566487	1.327362	0.279387	0.222054
tsrna-16287	TTCGAATCCTGCTCACAGCGCC	0	0.07808	0	0.111027
tsrna-16285	TTCGAATCCTGCCGACTACGCCA	7.92720002	18.50499	2.584332	1.776434
tsrna-16284	TTCGAATCCTGCCGACTACGCC	0.28566487	1.405442	0	0
tsrna-16283	TTCGAATCCTGCCGACTACGC	0	0.07808	0	0
tsrna-16281	TTCGAATCCGGCTCGGAGGACCA	0	0.07808	0	0
tsrna-16280	TTCGAATCCGGCTCGAAGGACCA	1.57115676	3.747845	0.838162	0.666163
tsrna-16279	TTCGAATCCGGCTCGAAGGACC	0.14283243	0.390401	0	0
tsrna-16270	TTCGAATCCCTTCGTGGTTGCCA	0	0.624641	0	0
tsrna-16269	TTCGAATCCCTCCGTGGTTACCA	0.14283243	1.015041	0.069847	0
tsrna-16268	TTCGAATCCCATCCTCGTCGCCA	0.35708108	1.171202	0.139694	0
tsrna-16267	TTCGAATCCCATCCTCGTCGCC	0	0.07808	0.069847	0
tsrna-16266	TTCGAATCCCATCCTCGTCGC	0	0.07808	0	0
tsrna-16265	TTCGAATCCCATCCTCGTCG	0	0.07808	0	0
tsrna-16264	TTCGAATCCCAGTAGAGCCTCCA	0.99982703	1.561602	0.349234	1.110271
tsrna-16263	TTCGAATCCCAGCGGTGCCTCCA	2.71381622	3.591685	0.558774	0.444109
tsrna-16262	TTCGAATCCCAGCGGTGCCTCC	0.14283243	0.07808	0	0
tsrna-16256	TTCGAATCCCAGCGGGGCCTCCA	0	0.31232	0	0.111027
tsrna-16253	TTCGAATCCCACTTCTGACACCA	0.78557838	2.186243	0.768315	1.221299
tsrna-16252	TTCGAATCCCACTTCTGACACC	0.4284973	0.23424	0.20954	0
tsrna-16251	TTCGAATCCCACTTCTGACAC	0	0	0	0
tsrna-16250	TTCGAATCCCACTTCTGACA	0	0	0	0
tsrna-16248	TTCGAATCCCACTCCTGACACCA	6.42745948	7.729931	4.190808	3.219787
tsrna-16247	TTCGAATCCCACTCCTGACACC	0.4284973	0.780801	0.139694	0.222054
tsrna-16246	TTCGAATCCCACTCCTGACAC	0	0.15616	0	0
tsrna-16242	TTCGAATCCCACCTTCGTCGCCA	0.14283243	0.31232	0	0.111027
tsrna-16241	TTCGAATCCCACCGCTGCCACCA	0.92841081	2.342403	0.628621	0.444109
tsrna-16240	TTCGAATCCCACCGCTGCCACC	0	0.390401	0	0
tsrna-16235	TTCGAATCCCACCAGAGTCGCCA	0.28566487	0.468481	0	0
tsrna-16233	TTCGAATCCCACCACTGCCACCA	0.14283243	1.561602	0	0
tsrna-16232	TTCGAATCACGTCGGGGTCACCA	0.4284973	0.702721	0.279387	0.222054
tsrna-16231	TTCGAATCACGTCGGGGTCACC	0	0.07808	0.069847	0
tsrna-16230	TTCGAATCACGTCGGGGTCAC	0	0	0	0.111027
tsrna-16224	TTCGAAGAAGCAGCTTCAAACCTGCCGGGGCTT	0.07141622	0.07808	0	0
tsrna-16223	TTCGAAGAAGCAGCTTCAAACCTGCCGGGGCT	0	0	0.069847	0
tsrna-16222	TTCGAAGAAGCAGCTTCAAACCTGCCGGGG	0	0.07808	0	0
tsrna-16219	TTCGAACCTCAGAGGGGGCACCA	0	0.546561	0	0
tsrna-16215	TTCGAACCCTGCTCGCTGCGCCA	0.49991351	1.873923	0.419081	0.444109
tsrna-16214	TTCGAACCCTGCTCGCTGCGCC	0	0.390401	0	0
tsrna-16211	TTCGAACCCCGTCCGTGCCTCCA	0.35708108	0.858881	0.279387	0.222054
tsrna-16206	TTCGAACCCCACTTCTGGTACCA	0	0.546561	0	0
tsrna-16205	TTCGAACCCCACTCCTGGTACCA	0	0.07808	0	0
tsrna-16201	TTCGAAACCGGGCGGAAACACCA	22.4961082	56.1396	8.241923	4.108004
tsrna-16200	TTCGAAACCGGGCGGAAACACC	4.92771893	17.02146	1.74617	0.222054
tsrna-16199	TTCGAAACCGGGCGGAAACAC	0.07141622	0.31232	0	0
tsrna-16198	TTCGAAACCGGGCGGAAACA	0.07141622	0.07808	0	0
tsrna-16193	TTCGAAACCGGGCAGAAGCACCA	2.21390271	11.16546	0.628621	0.555136
tsrna-16192	TTCGAAACCGGGCAGAAGCACC	0	0.468481	0	0
tsrna-16191	TTCGAAACCGGGCAGAAGCAC	0	0.07808	0	0
tsrna-16190	TTCGAAACCGGGCAGAAGCA	0	0	0	0
tsrna-16186	TTCCTTCCTTTTTTGCCA	0.07141622	0	0	0
tsrna-16184	TTCCTGGTTTTCACCCAGGTGGCCCG	0.21424865	0.468481	0.20954	0
tsrna-16183	TTCCTGGTTTTCACCCAGGCGGCCCGGGTTCGACTCCCGGT(	0	0.23424	0	0
tsrna-16182	TTCCTGGTTTTCACCCAGGCGGCCCG	0.14283243	0.31232	0	0.111027
tsrna-16180	TTCCTGGCCAATGCACCA	0	0	0.20954	0
tsrna-16179	TTCCTCTTCTTAACACCA	0.07141622	0	0	0
tsrna-16176	TTCCTCGTTAGTATAGTGGTGAGT	0.21424865	0.624641	0.20954	0.555136
tsrna-16175	TTCCTCGTTAGTATAGTGGTGAG	0	0.07808	0	0.222054
tsrna-16174	TTCCTCGTTAGTATAGTGG	0.07141622	0	0	0
tsrna-16173	TTCCGTAGTGTAGTGGTTATCACGTTCGCCTCAC	0.49991351	0.31232	0.20954	0.111027
tsrna-16172	TTCCGTAGTGTAGTGGTTATCACGTTCGCCTCA	0.14283243	0	0.069847	0.333081
tsrna-16171	TTCCGTAGTGTAGTGGTTATCACGTTCGCCTC	0.35708108	0.07808	0.20954	0

tsrna-16170	TTCCGTAGTGTAGTGGTTATCACGTTCGCCT	0.07141622	0	0.349234	0.77719
tsrna-16169	TTCCGTAGTGTAGTGGTTATCACGTTCGCC	0.14283243	0.23424	0.419081	0.333081
tsrna-16168	TTCCGTAGTGTAGTGGTTATCACGTTCGC	0.21424865	0.07808	0	0
tsrna-16167	TTCCGTAGTGTAGTGGTTATCACGTTCG	0.4284973	0.07808	0.069847	0
tsrna-16166	TTCCGTAGTGTAGTGGTTATCACGTTC	0.14283243	0	0.20954	0
tsrna-16165	TTCCGTAGTGTAGTGGTTATCACGTT	0.07141622	0.07808	0.139694	0.111027
tsrna-16164	TTCCGTAGTGTAGTGGTTATCACG	0.07141622	0.07808	0.20954	0.111027
tsrna-16163	TTCCGTAGTGTAGTGGTTATCA	0.14283243	0	0	0.111027
tsrna-16162	TTCCGTAGTGTAGTGGTTATC	0.21424865	0.07808	0.069847	0
tsrna-16160	TTCCGTAGTGTAGTGGTCATCACGTTCGCCT	0.14283243	0.07808	0	0.111027
tsrna-16159	TTCCGTAGTGTAGTGGTCATCACGTTCGCC	0.07141622	0.07808	0.279387	0
tsrna-16158	TTCCGTAGTGTAGTGGTCATCACGTT	0.07141622	0	0.069847	0.333081
tsrna-16157	TTCCGTAGTGTAGTGGTCATC	0.07141622	0	0	0.111027
tsrna-16155	TTCCGTAGTGTAGTGGT	0	0	0	0.111027
tsrna-16153	TTCCGTAGTGTAGCGGTTATCACATTCGCCT	0.07141622	0.07808	0.069847	0
tsrna-16152	TTCCGTAGTGTAGCGGTTATCACATTCGCC	0	0	0.139694	0
tsrna-16151	TTCCGTAGTGTAGCGGTTATCACATT	0	0.15616	0.069847	0
tsrna-16150	TTCCGGGTTCGAGTCCCGGCGGAGTCGCCA	0.14283243	0.07808	0	0
tsrna-16146	TTCCGGCTCGAAGGACCA	4.28497299	8.979213	1.117549	1.332326
tsrna-16145	TTCCGGCTCGAAGGACC	0.14283243	0.07808	0.069847	0.111027
tsrna-16143	TTCCGGATCAGAAGATTGAGGGTTCG	0.21424865	0.858881	0.20954	0.222054
tsrna-16142	TTCCGGATCAGAAGATTGAGGGTT	0.14283243	0.780801	0.419081	0
tsrna-16141	TTCCGGATCAGAAGATTGAGGGT	0.14283243	0.31232	0.20954	0.111027
tsrna-16135	TTCCCTGGTGGTCTAGTGGTTAGGATTCGG	1.07124325	2.654724	1.816017	2.33157
tsrna-16134	TTCCCTGGTGGTCTAGTGGTTAGGATTCG	1.78540541	2.342403	1.466783	1.998488
tsrna-16133	TTCCCTGGTGGTCTAGTGGTTAGG	1.35690811	2.108163	2.025557	1.998488
tsrna-16132	TTCCCTGGTGGTCTAGTGGTTAG	1.42832433	2.732804	2.095404	1.776434
tsrna-16131	TTCCCTGGTGGTCTAGTGGTTA	2.21390271	1.952003	1.396936	1.443353
tsrna-16130	TTCCCTGGTGGTCTAGTGG	0.07141622	1.015041	0	0.444109
tsrna-16129	TTCCCTGGTGGTCTAGTG	0.21424865	1.171202	0.20954	0.444109
tsrna-16127	TTCCCGGTCAGGGAACCA	0.21424865	0.468481	0.069847	0.111027
tsrna-16124	TTCCCGGGCGGCGCACCA	0.57132973	2.342403	0.419081	0
tsrna-16123	TTCCCGGGCGGCGCACC	0.49991351	0.390401	0.069847	0
tsrna-16121	TTCCCGGCCCATGCACCA	0.28566487	0.780801	0.139694	0.111027
tsrna-16120	TTCCCGGCCCATGCACC	0.14283243	0.07808	0	0
tsrna-16118	TTCCCGGCCACTGCACCA	0	0.23424	0	0
tsrna-16117	TTCCCGGCCAATGCACCA	0	0	0.139694	0
tsrna-16116	TTCCCGGCCAATGCACC	0.07141622	0	0	0
tsrna-16110	TTCCCCGCACAGGTTCGAATCCT	0.35708108	1.171202	0.139694	0
tsrna-16109	TTCCCCGCACAGGTTCGAA	0	0	0	0
tsrna-16105	TTCCCATTCTTGCGACCCGGGTT	0.07141622	0.390401	0.139694	0
tsrna-16104	TTCCCATTCTTGCGACCCGGGT	0.07141622	0.15616	0	0
tsrna-16102	TTCCCATTCTTGCGACCCGG	0.07141622	0.07808	0	0
tsrna-16099	TTCCCATTCTTGCGACC	0	0	0	0
tsrna-16097	TTCCCATATGGTCTAGCGGTTAGGATTCCT	0.07141622	0.07808	0.139694	0
tsrna-16096	TTCCCATATGGTCTAGCGGTTAGGATT	0.28566487	0	0.069847	0.111027
tsrna-16095	TTCCCATATGGTCTAGCGGTTAGG	0	0.07808	0.139694	0.333081
tsrna-16094	TTCCCATATGGTCTAGCGGTTAG	0.07141622	0.07808	0.139694	0.111027
tsrna-16093	TTCCCACATGGTCTAGCGGTTAGGATTCCT	0.07141622	0.624641	0.558774	1.443353
tsrna-16092	TTCCCACATGGTCTAGCGGTTAGGATT	0.35708108	0.468481	0.558774	1.776434
tsrna-16091	TTCCCACATGGTCTAGCGGTTAGG	0.21424865	0.31232	0.698468	1.665407
tsrna-16090	TTCCATGGTGTAATGGTTAGCACTCTGGACTCT	0.07141622	0.07808	0	0
tsrna-16089	TTCCATGGTGTAATGGTTAGCACTCTGGACT	0	0.15616	0.069847	0.111027
tsrna-16088	TTCCATGGTGTAATGGTTAGCACTCTGGAC	0.07141622	0	0	0.111027
tsrna-16087	TTCCATGGTGTAATGGTTAGCACTCTGGA	0	0	0.069847	0
tsrna-16083	TTCCATGGTGTAATGGTGAGCACTCTGGAC	0.07141622	0	0	0
tsrna-16079	TTCCATAGTGTAGTGGTTATCACGTCTGCTTTAC	0.28566487	0	0.488928	0
tsrna-16078	TTCCATAGTGTAGTGGTTATCACGTCTGCTTT	0.07141622	0	0.069847	0
tsrna-16077	TTCCATAGTGTAGTGGTTATCACGTCTGCTT	0.07141622	0	0	0
tsrna-16075	TTCCATAGTGTAGTGGTTATCACGTCTGC	0	0	0.069847	0
tsrna-16074	TTCCATAGTGTAGTGGTTATCACGTCTG	0.07141622	0	0	0
tsrna-16072	TTCCATAGTGTAGCGGTTATCACGTCTG	0	0	0.069847	0
tsrna-16071	TTCCAGGTTCGACTCCTGGCTGGCTCGCC	0	0.07808	0	0.111027
tsrna-16070	TTCCAGGTTCGACTCCTGGCTGGCTCGC	0	0	0	0

tsrna-16069	TTCCAGGTTCGACTCCTGGCTGGCTCG	0	0.07808	0	0
tsrna-16061	TTCCAATTAACTAGTTTTGACAACATTCAAAAAAGAGTACCA	0	0.07808	0	0
tsrna-16058	TTCCAAGCAGTTGACCCGGGTTCGATTCCCGGCCAACGCACC	4.21355677	10.38465	2.025557	1.665407
tsrna-16057	TTCCAAGCAGTTGACCCGGGT	0	0.468481	0.139694	0
tsrna-16056	TTCCAAGCAGTTGACCCGG	0.07141622	0.07808	0	0
tsrna-16053	TTCATATTGAATTGCAAATTCG	0	0	0	0
tsrna-16052	TTCATATTGAATTGCAAATTC	0.07141622	0	0	0
tsrna-16049	TTCATATCCGCGTGGGT	0	0.15616	0	0
tsrna-16048	TTCATATCATTGGTCGTGGTTGTAGTCCGTGCGAGAATACCA	9.78402165	11.47778	15.43614	5.107248
tsrna-16047	TTCATATCATTGGTCGTGGTTGTAGTCCGTGCGAGAATACC	0.92841081	2.810884	1.606477	2.33157
tsrna-16046	TTCATATCATTGGTCGTGGTTGTAGTCCGTGCGAGAATA	1.14265946	1.405442	0.558774	1.332326
tsrna-16045	TTCATATCATTGGTCGTGGTTGTAGTCCGTGCGAGAAT	0.28566487	1.249282	0.768315	1.332326
tsrna-16044	TTCATATCATTGGTCGTGGTTGTAGTCCGTGCGAGAA	0.4284973	1.249282	0.488928	0.999244
tsrna-16043	TTCATATCATTGGTCGTGGTTGTAGTCCGTGCGAGA	0.49991351	1.171202	0.628621	1.221299
tsrna-16042	TTCATATCATTGGTCGTGGTTGTAGTCCGTGCGAG	0.14283243	0.780801	0.558774	0.444109
tsrna-16041	TTCATATCATTGGTCGTGGTTGTAGTCCGTGCGA	0.14283243	0.390401	0.20954	0.333081
tsrna-16040	TTCATATCATTGGTCGTGGTTGTAGTCCGTGCG	0.4284973	0.23424	0.279387	0.333081
tsrna-16039	TTCATATCATTGGTCGTGGTTGTAGTCCGT	0.07141622	0	0.20954	0.111027
tsrna-16038	TTCATATCATTGGTCGTGGTTGTAGTCCG	0.07141622	0.23424	0	0
tsrna-16037	TTCATATCATTGGTCGTGGTTGTAGTCC	0.07141622	0.07808	0.139694	0
tsrna-16036	TTCATATCATTGGTCGTGGTTGTAGTC	0.07141622	0.15616	0	0
tsrna-16035	TTCATATCATTGGTCGTGGTTGTAGT	0.07141622	0	0	0.111027
tsrna-16034	TTCATATCATTGGTCGTGGTTGTAG	0	0.07808	0.069847	0
tsrna-16033	TTCATATCATTGGTCGTGGTTGTA	0	0.07808	0	0
tsrna-16032	TTCATATCATTGGTCGTGGTTGT	0	0	0	0
tsrna-16028	TTCATATCATTGGTCGTGG	0	0	0	0
tsrna-16025	TTCATAACTTTGTCAAAGTTAAATTATAGGCT	0.35708108	0.468481	0.349234	0.111027
tsrna-16024	TTCATAACTTTGTCAAAGTTAAATTATAGGC	0.28566487	0.390401	0.698468	0
tsrna-16023	TTCAGTTGATGCAGAGTGGGGTTTTGCAGTCCTTACCA	1.49974054	0.624641	1.816017	0.333081
tsrna-16022	TTCAGTTGATGCAGAGTGGGGTTTTGCAGTCCTTACC	0.4284973	0.390401	0.349234	0.111027
tsrna-16021	TTCAGTTGATGCAGAGTGGGGTTTTGCAGTCCTTAC	0.21424865	0.15616	0.419081	0.111027
tsrna-16020	TTCAGTTGATGCAGAGTGGGGTTTTGCAGTCCT	0	0.624641	0.069847	0.555136
tsrna-16019	TTCAGTTGATGCAGAGTGGGGTTTTGCAGTC	0.07141622	0.15616	0	0
tsrna-16018	TTCAGTTGATGCAGAGTGGGGTTTTGCAGT	0.07141622	0.15616	0.139694	0
tsrna-16017	TTCAGTTGATGCAGAGTGGGGTTTTGCAG	0.07141622	0.07808	0	0
tsrna-16016	TTCAGTTGATGCAGAGTGGGGTTTTGCA	0.07141622	0.15616	0.069847	0
tsrna-16015	TTCAGTTGATGCAGAGTGGGGTTTTGC	0.14283243	0	0	0
tsrna-16014	TTCAGTTGATGCAGAGTGGGGTTTTG	0	0	0.069847	0
tsrna-16013	TTCAGTTGATGCAGAGTGGGGTTTT	0	0	0.069847	0
tsrna-16012	TTCAGTTGATGCAGAGTGGGGTTT	0	0	0.069847	0.111027
tsrna-16011	TTCAGTTGATGCAGAGTGGGGTT	0	0	0	0
tsrna-16010	TTCAGTTGATGCAGAGTGGGGT	0	0	0	0
tsrna-16009	TTCAGTTGATGCAGAGTGGGG	0	0	0	0
tsrna-16007	TTCAGTTGATGCAGAGTGG	0	0.07808	0.069847	0
tsrna-16006	TTCAGTGGTAGAATTTTC	0.07141622	0	0	0
tsrna-16005	TTCAGTGGTAGAATTCTTGCCTGC	0	0	0	0
tsrna-16001	TTCAGTGGTAGAATTCTCGCCTT	0.28566487	0.390401	0.20954	0.444109
tsrna-16000	TTCAGTGGTAGAATTCTCGCCTGCCACGC	3.07089731	7.964171	1.816017	2.553624
tsrna-15999	TTCAGTGGTAGAATTCTCGCCTGCCA	0.57132973	2.498564	0.349234	0.444109
tsrna-15998	TTCAGTGGTAGAATTCTCGCCTGCC	0.99982703	3.357445	0.558774	0.555136
tsrna-15997	TTCAGTGGTAGAATTCTCGCCTGC	0.14283243	0.936961	0.279387	0
tsrna-15996	TTCAGTGGTAGAATTCTCGCCTG	0.21424865	0.468481	0.349234	0.222054
tsrna-15995	TTCAGTGGTAGAATTCTCGCCTCC	0.14283243	1.093122	0.20954	0.333081
tsrna-15994	TTCAGTGGTAGAATTCTCGCCTC	0.35708108	0.31232	0.20954	0.333081
tsrna-15993	TTCAGTGGTAGAATTCTCGCCT	0.21424865	0.858881	0.139694	0.111027
tsrna-15992	TTCAGTGGTAGAATTCTCGCC	0.14283243	0.23424	0	0
tsrna-15991	TTCAGTGGTAGAATTCTCGC	0.07141622	0.07808	0.069847	0
tsrna-15985	TTCAGGTCGCAGTCTCCCCTG	0	0.07808	0	0
tsrna-15979	GCTGTTAACCGAAAGGTTGGTGGT	0.14283243	2.576644	0.069847	0.333081
tsrna-15978	GCTGTTAACCGAAAGGTTGGTGG	0	0.31232	0	0
tsrna-15977	GCTGTTAACCGAAAGGTTGGTG	0	0	0	0
tsrna-15976	GCTGTTAACCGAAAGGTTGGT	0	0.15616	0	0
tsrna-15975	GCTGTTAACCGAAAGGTTGG	0	0.07808	0	0
tsrna-15974	GCTGTGATGGCCGAGTGGTTAAGGTGTTGG	0.07141622	0.23424	0	0.111027

tsrna-15973	GCTGTGATGGCCGAGTGGTTAAGGTGTTG	0.07141622	0.31232	0	0
tsrna-15972	GCTGTGATGGCCGAGTGGTTAAGGT	0.07141622	0.31232	0.069847	0
tsrna-15971	GCTGTGATGGCCGAGTGGTTAAGGCGTTGG	0.07141622	0.390401	0	0
tsrna-15970	GCTGTGATGGCCGAGTGGTTAAGGCGTTG	0.07141622	0.23424	0.069847	0
tsrna-15969	GCTGTGATGGCCGAGTGGTTAAGGCGT	0	0.15616	0	0
tsrna-15968	GCTGTGATGGCCGAGTGGTTAAGGC	0	0.31232	0	0
tsrna-15967	GCTGTGATGGCCGAGTGGTTAAGG	0.07141622	0.468481	0.069847	0
tsrna-15966	GCTGTGATGGCCGAGTGGTTAAG	0.07141622	0.390401	0.069847	0
tsrna-15965	GCTGTGATGGCCGAGTGGTTAA	0.07141622	0.468481	0	0
tsrna-15964	GCTGTGATGGCCGAGTGGTTA	0	0.23424	0	0
tsrna-15963	GCTGTGATGGCCGAGTGGTT	0.07141622	0.390401	0.069847	0
tsrna-15962	GCTGTGATGGCCGAGTGGT	0.07141622	0.468481	0	0
tsrna-15961	GCTGTGATGGCCGAGTGG	0	0.390401	0	0
tsrna-15960	GCTGTGATGGCCGAGTG	0.07141622	0	0	0
tsrna-15959	GCTGTGATGGCCGAGT	0	0.07808	0	0
tsrna-15958	GCTGGTTCGATTCCGGCTCGAAGGACCA	5.71329731	11.7901	2.165251	2.775678
tsrna-15955	GCTGGTTCGAATCCGGCTCGAAGGACCA	2.07107028	4.138246	0.558774	0.888217
tsrna-15953	GCTGGTGTAGTGGTATCATGCAAGATTCCCATTCTT	0.35708108	0.23424	0.488928	0
tsrna-15952	GCTGGTGTAGTGGTATCATGCAAGATTCCC	0.14283243	0.15616	0.139694	0.111027
tsrna-15951	GCTGGTGTAGTGGTATCATGCAAGATTCC	0.21424865	0.07808	0.279387	0.111027
tsrna-15950	GCTGGTGTAGTGGTATCATGCAAGATTC	0.21424865	0.07808	0	0.111027
tsrna-15949	GCTGGTGTAGTGGTATCATGCAAGATT	0.07141622	0.15616	0.139694	0
tsrna-15948	GCTGGTGTAGTGGTATCATGCAAGAT	0	0.15616	0	0
tsrna-15947	GCTGGTGTAGTGGTATCATGCAAGA	0.21424865	0.23424	0.139694	0
tsrna-15946	GCTGGTGTAGTGGTATCATGCAAG	0.07141622	0.15616	0	0
tsrna-15945	GCTGGTGTAGTGGTATCATGCAA	0	0	0	0
tsrna-15944	GCTGGTGTAGTGGTATCATGCA	0	0	0	0
tsrna-15943	GCTGGTGTAGTGGTATCATGC	0.07141622	0	0	0
tsrna-15942	GCTGGTGTAGTGGTATCATG	0.07141622	0	0	0
tsrna-15941	GCTGGTGTAGTGGTATCAT	0.07141622	0	0	0
tsrna-15940	GCTGGTGTAGTGGTATCA	0	0	0.069847	0
tsrna-15939	GCTGGTGTAGTGGTATC	0	0	0	0.111027
tsrna-15935	GCTGGGGATTGTGGGTTCGAGTCCCATCTGGGTCGCCA	0.35708108	1.249282	0	0.111027
tsrna-15934	GCTGGGGATTGTGGGTTCGAGTCCCATCTGGGTCGCC	0.14283243	0.15616	0.139694	0
tsrna-15933	GCTGGGGATTGTGGGTTCGAGTCCCATCTG	0.07141622	0.31232	0.069847	0.111027
tsrna-15932	GCTGGGGATTGTGGGTTCGAGTC	0	0.07808	0	0.111027
tsrna-15931	GCTGGGGATTGTGGGTTCGAG	0.07141622	0.07808	0	0
tsrna-15930	GCTGGGGATTGTGGGTTCG	0	0	0	0
tsrna-15929	GCTGGGGATTGTGGGTTC	0.07141622	0.07808	0	0
tsrna-15928	GCTGGGGATTGTGGGTT	0	0.07808	0.069847	0
tsrna-15925	GCTGCCTTCCAAGCAGTTGACCCGGGT	1.78540541	2.888964	1.047702	1.332326
tsrna-15924	GCTGCCTTCCAAGCAGTTGACCCGGG	0.14283243	0	0.069847	0.222054
tsrna-15923	GCTGCCTTCCAAGCAGTTGACCCGG	0.21424865	0.07808	0	0.111027
tsrna-15922	GCTGCCTTCCAAGCAGTTGACCCG	0.14283243	0.15616	0.069847	0
tsrna-15921	GCTGCCTTCCAAGCAGTTGACCC	0.07141622	0	0	0.111027
tsrna-15920	GCTGATTTGCGTTCAGTTGATGCAG	0	0	0	0.111027
tsrna-15919	GCTGATTTGCGTTCAGTTGATG	0.07141622	0	0	0
tsrna-15917	GCTGAGTGAAGCATTGGACTGTAAATCTA	0.07141622	0.07808	0	0
tsrna-15916	GCTGAGTGAAGCATTGGACTGTAAATCT	0	0.23424	0	0
tsrna-15915	GCTGAGTGAAGCATTGGACTGTAAATC	0	0	0.069847	0
tsrna-15914	GCTGAGTGAAGCATTGGACTGTAAAT	0	0.07808	0	0.222054
tsrna-15913	GCTGAGTGAAGCATTGGACTGTAAA	0	0.07808	0	0
tsrna-15912	GCTGAGTGAAGCATTGGACTGTAA	0.07141622	0.23424	0.069847	0
tsrna-15911	GCTGAGTGAAGCATTGGACTGTA	0	0.07808	0	0
tsrna-15909	GCTGAGTGAAGCATTGGACTG	0	0.07808	0	0
tsrna-15907	GCTGAGTGAAGCATTGGAC	0	0.07808	0	0
tsrna-15902	GCTCTGTGGCGCAATGGATAGCGCATTG	0.07141622	0	0.069847	0
tsrna-15897	GCTCTGCACGCGTGGGT	0	0.07808	0	0
tsrna-15895	GCTCTCACCGCCGCGGCCCGGGTTCGATTCCCGGTCAGGGA	2.35673514	23.81443	1.466783	0.666163
tsrna-15894	GCTCTCACCGCCGCGGCCCGGGTTCGATTCCCGGTCAGG	0	0.15616	0	0
tsrna-15890	GCTCTCACCGCCGCGGCCCGGGTT	0.07141622	0.23424	0	0.111027
tsrna-15889	GCTCTCACCGCCGCGGCCCGGGT	0	0.07808	0	0
tsrna-15887	GCTCTCACCGCCGCGGCCCGG	0	0.07808	0	0
tsrna-15880	GCTCGTTGGTCTAGGGGTATGATTCTCGGT	0.49991351	0.15616	0	0

tsrna-15879	GCTCGTTGGTCTAGGGGTATGATTCTCGG	0	0.15616	0	0
tsrna-15878	GCTCGTTGGTCTAGGGGTATGATTCTCGCTT	1.64257298	1.873923	1.047702	2.33157
tsrna-15877	GCTCGTTGGTCTAGGGGTATGATTCTCGCT	1.57115676	2.186243	1.187396	2.33157
tsrna-15876	GCTCGTTGGTCTAGGGGTATGATTCTCGC	0.8569946	0.702721	0.279387	1.776434
tsrna-15875	GCTCGTTGGTCTAGGGGTATGATTCTCG	0	0.15616	0.069847	0
tsrna-15874	GCTCGTTGGTCTAGGGGTATGATTCTC	0.07141622	0	0.069847	0
tsrna-15873	GCTCGTTGGTCTAGGGGTATGATTCT	0	0	0	0
tsrna-15871	GCTCGTTGGTCTAGGGGTATGATT	0	0	0	0
tsrna-15858	GCTCCGAGGTGATTTTCATATTGAATTGC	0.07141622	0.23424	0	0
tsrna-15857	GCTCCGAGGTGATTTTCATATTGAATTG	0.14283243	0	0	0
tsrna-15856	GCTCCGAGGTGATTTTCATATTGAATT	0.07141622	0	0	0
tsrna-15852	GCTCCGAGGTGATTTTCATATTG	0.07141622	0	0	0
tsrna-15844	GCTCCATAGCTCAGTGGT	0.14283243	0	0	0
tsrna-15842	GCTCCATAGCTCAGGGGT	0	0.07808	0	0
tsrna-15826	GCTCCAGTCTCTTCGGGGGCGTGGGTTCG	0.07141622	0	0.069847	0
tsrna-15824	GCTCCAGTCTCTTCGGGGGCGTGGG	0	0	0	0
tsrna-15819	GCTCCAGTCTCTTCGGGGGC	0	0.07808	0	0
tsrna-15815	GCTCCAGTCTCTTCGGAGGCGTGGGTTCG	0	0	0	0
tsrna-15814	GCTCCAGTCTCTTCGGAGGCGTGGGTTC	0.07141622	0.15616	0.069847	0
tsrna-15813	GCTCCAGTCTCTTCGGAGGCGTGG	0	0	0	0
tsrna-15812	GCTCCAGTCTCTTCGGAGGCGTG	0	0.07808	0	0
tsrna-15804	GCTCCAGTCATTTCGATGGCGTGGGTTCG	0.21424865	0.468481	0.20954	0
tsrna-15803	GCTCAGTTGGTAGAGCGGAGGACTGTAG	0	0	0	0.111027
tsrna-15800	GCTCAGTTGGTAGAGCATCAG	0.07141622	0	0	0
tsrna-15797	GCTCAGTGGTAGAGCGCGTGCTT	0.28566487	1.405442	0.139694	0
tsrna-15796	GCTCAGTGGTAGAGCGCGTGC	0.14283243	1.561602	0.069847	0
tsrna-15795	GCTCAGTGGTAGAGCATTTGACTGC	0	0.07808	0.069847	0
tsrna-15794	GCTCAGTGGTAGAGCATTTGACTG	0.35708108	0.15616	0	0
tsrna-15793	GCTCAGTGGTAGAGCATTTGACTAC	0.07141622	0	0	0.111027
tsrna-15792	GCTCAGTGGTAGAGCATTTGACTA	0	0.23424	0.069847	0.111027
tsrna-15791	GCTCAGTGGTAGAGCATTTGACT	0.07141622	0	0.069847	0
tsrna-15790	GCTCAGTGGTAGAGCATTTGAC	0.21424865	0.07808	0	0
tsrna-15789	GCTCAGTGGTAGAGCATTTGA	0.07141622	0.15616	0	0
tsrna-15788	GCTCAGTGGTAGAGCATTTG	0	0	0.069847	0
tsrna-15787	GCTCAGTGGTAGAGCATTTAACT	0	0.07808	0	0.222054
tsrna-15786	GCTCAGTGGTAGAGCATTT	0	0.15616	0	0
tsrna-15784	GCTCAGTGGGTAGAGCATTTGACTGC	0	0.23424	0	0
tsrna-15783	GCTCAGTGGGTAGAGCATTTGACTG	0.21424865	0	0	0
tsrna-15782	GCTCAGTGGGTAGAGCATTTGACT	0	0.15616	0.069847	0
tsrna-15781	GCTCAGTCGGTAGAGCATGGGACTCTTAATCC	1.64257298	1.717762	0.698468	0.666163
tsrna-15780	GCTCAGTCGGTAGAGCATGGGACTCTTA	0.64274595	1.405442	0.349234	0.222054
tsrna-15779	GCTCAGTCGGTAGAGCATGGGACTCTT	0.78557838	1.561602	0.488928	0.222054
tsrna-15778	GCTCAGTCGGTAGAGCATGGGACTCT	1.07124325	1.873923	0.488928	0.111027
tsrna-15777	GCTCAGTCGGTAGAGCATGGGACTC	1.14265946	2.030083	0.558774	0.222054
tsrna-15776	GCTCAGTCGGTAGAGCATGGGACT	0.8569946	1.015041	0.628621	0.444109
tsrna-15775	GCTCAGTCGGTAGAGCATGGGAC	0.92841081	1.483522	0.20954	0.333081
tsrna-15774	GCTCAGTCGGTAGAGCATGGGA	0.49991351	1.171202	0.349234	0.111027
tsrna-15773	GCTCAGTCGGTAGAGCATGGG	0.21424865	0.23424	0	0
tsrna-15772	GCTCAGTCGGTAGAGCATGG	0.07141622	0.07808	0	0
tsrna-15771	GCTCAGTCGGTAGAGCATGAGACTC	1.07124325	2.498564	0.838162	0.999244
tsrna-15770	GCTCAGTCGGTAGAGCATGAGACT	0.57132973	2.030083	0.558774	0.333081
tsrna-15769	GCTCAGTCGGTAGAGCATGAGAC	1.35690811	2.498564	0.419081	0.77719
tsrna-15768	GCTCAGTCGGTAGAGCATGAGA	0.14283243	0.468481	0.20954	0.222054
tsrna-15767	GCTCAGTCGGTAGAGCATGAG	0.14283243	0.23424	0	0
tsrna-15765	GCTCAGTCGGTAGAGCATG	0	0.15616	0	0
tsrna-15764	GCTCAGTCGGTAGAGCATCAGACTTTTA	1.42832433	0.546561	0.558774	0.666163
tsrna-15763	GCTCAGTCGGTAGAGCATCAGACTTTT	0.78557838	1.171202	0.977855	0.77719
tsrna-15762	GCTCAGTCGGTAGAGCATCAGACTTT	0.49991351	1.249282	0.20954	0.222054
tsrna-15761	GCTCAGTCGGTAGAGCATCAGACTT	0.64274595	0.624641	0.419081	0.111027
tsrna-15760	GCTCAGTCGGTAGAGCATCAGACT	0.57132973	0.780801	0.20954	0
tsrna-15759	GCTCAGTCGGTAGAGCATCAGAC	0.64274595	1.015041	0.349234	0.222054
tsrna-15758	GCTCAGTCGGTAGAGCATCAGA	0.92841081	0.858881	0.20954	0
tsrna-15757	GCTCAGTCGGTAGAGCATCAG	0.07141622	0.31232	0.139694	0.111027
tsrna-15756	GCTCAGTCGGTAGAGCATCA	0.21424865	0.390401	0.069847	0

tsrna-15755	GCTCAGTCGGTAGAGCATC	0.21424865	0.07808	0.139694	0
tsrna-15754	GCTCAGTCGGTAGAGCAT	0	0.07808	0	0
tsrna-15751	GCTCAGGTGGTAGAGCATTTGACTGC	0.07141622	0	0.069847	0
tsrna-15750	GCTCAGGTGGTAGAGCATTTGACTG	0.07141622	0	0	0
tsrna-15749	GCTCAGGTGGTAGAGCATTTGACT	0.07141622	0	0	0.111027
tsrna-15748	GCTCAGGGGTTAGAGCACTGGT	0.07141622	0.07808	0	0
tsrna-15746	GCTCAGGGGTAGAGCATTTGACTGC	0	0.07808	0	0
tsrna-15745	GCTCAGGGGTAGAGCATTTGACTG	0.07141622	0	0	0
tsrna-15744	GCTCAGGGGTAGAGCATTTGACT	0	0.07808	0	0
tsrna-15739	GCTCACATCACCCCATAAACACCA	0.35708108	0.15616	0.069847	0
tsrna-15738	GCTCACATCACCCCATAAACACC	0.07141622	0	0	0
tsrna-15730	GCTCACAAGAACTGCTAACTCATGCCCCCATGTCTAACAACA[illegible]	0.35708108	0.07808	0.139694	0.111027
tsrna-15729	GCTCACAAGAACTGCTAACTCATGCCCCCATGTCTA	0.07141622	0.15616	0.139694	0.222054
tsrna-15728	GCTCACAAGAACTGCTAACTCATGCCCCC	0	0	0.069847	0
tsrna-15727	GCTCACAAGAACTGCTAACTCATGCCCC	0	0	0.069847	0
tsrna-15726	GCTCACAAGAACTGCTAACTCATGCC	0.07141622	0.23424	0	0
tsrna-15725	GCTCACAAGAACTGCTAACTCATGC	0.21424865	0	0.139694	0
tsrna-15724	GCTCACAAGAACTGCTAACTCATG	0.21424865	0.15616	0	0
tsrna-15723	GCTCACAAGAACTGCTAACTCAT	0	0	0.069847	0.111027
tsrna-15722	GCTCACAAGAACTGCTAACTCA	0	0	0	0
tsrna-15721	GCTCACAAGAACTGCTAACTC	0	0.07808	0.069847	0
tsrna-15720	GCTCACAAGAACTGCTAACT	0.07141622	0.07808	0	0.111027
tsrna-15719	GCTCACAAGAACTGCTAAC	0	0	0.069847	0
tsrna-15714	GCTATCGGGCCCATACCCCGAAAATGTTGG	0.28566487	0.07808	0.139694	0
tsrna-15713	GCTATCGGGCCCATACCCCGAAAATGTTG	0.14283243	0	0.20954	0
tsrna-15712	GCTATCGGGCCCATACCCCGAAAATG	0.28566487	0.07808	0.069847	0
tsrna-15711	GCTATCGGGCCCATACCCCGAAA	0	0	0	0
tsrna-15710	GCTATCGGGCCCATACCCCGAA	0	0.07808	0	0
tsrna-15709	GCTATCGGGCCCATACCCCGA	0	0.07808	0	0.111027
tsrna-15708	GCTATCGGGCCCATACCCCG	0	0	0	0
tsrna-15707	GCTATCGGGCCCATACCCC	0	0	0.20954	0
tsrna-15706	GCTATCGGGCCCATACCC	0	0	0.069847	0
tsrna-15704	GCTATCGGGCCCATAC	0	0.07808	0	0.111027
tsrna-15703	GCTATCCATTGGTCTTAGGCCCCA	0	0	0.069847	0
tsrna-15702	GCTATCCATTGGTCTTAGGCCCC	0	0	0	0
tsrna-15694	GCTAGGATTCGGCGCTTTCACCGCCGCGGCCC	0.49991351	1.639682	0.488928	1.55438
tsrna-15693	GCTAGGATTCGGCGCTTTCA	0.14283243	0.624641	0.349234	0.222054
tsrna-15692	GCTAGGATTCGGCGCTTTC	0.07141622	0.15616	0	0.111027
tsrna-15691	GCTAGGATTCGGCGCTTT	0	0.23424	0	0.111027
tsrna-15690	GCTAGGATTCGGCGCTT	0	0.23424	0	0.111027
tsrna-15689	GCTAGGATTCGGCGCT	0.07141622	0	0.139694	0
tsrna-15688	GCTAGCTCAGTCGGTAGAGCATGGGACTCT	1.2854919	1.249282	0.419081	0.222054
tsrna-15687	GCTAGCTCAGTCGGTAGAGCATGGGACTC	0.64274595	1.093122	0.698468	0.333081
tsrna-15686	GCTAGCTCAGTCGGTAGAGCATGGGACT	1.2854919	1.015041	0.558774	0.555136
tsrna-15685	GCTAGCTCAGTCGGTAGAGCATGGGAC	0.57132973	1.249282	0.768315	0.333081
tsrna-15684	GCTAGCTCAGTCGGTAGAGCATGGGA	0.28566487	1.093122	0.069847	0.111027
tsrna-15683	GCTAGCTCAGTCGGTAGAGCATGGG	0.28566487	0.15616	0.139694	0
tsrna-15682	GCTAGCTCAGTCGGTAGAGCATGG	0	0.15616	0.069847	0
tsrna-15681	GCTAGCTCAGTCGGTAGAGCATGAGACTCT	1.21407568	1.639682	0.349234	0.77719
tsrna-15680	GCTAGCTCAGTCGGTAGAGCATGAGACTC	1.14265946	2.264323	0.139694	0.666163
tsrna-15679	GCTAGCTCAGTCGGTAGAGCATGAGACT	0.49991351	1.093122	0.20954	0.333081
tsrna-15678	GCTAGCTCAGTCGGTAGAGCATGAGAC	1.2854919	1.483522	0.488928	0.333081
tsrna-15677	GCTAGCTCAGTCGGTAGAGCATGAGA	0	0.390401	0.069847	0.111027
tsrna-15676	GCTAGCTCAGTCGGTAGAGCATGAG	0.07141622	0.390401	0	0
tsrna-15675	GCTAGCTCAGTCGGTAGAGCATGA	0.07141622	0.15616	0.069847	0.111027
tsrna-15674	GCTAGCTCAGTCGGTAGAGCATG	0	0.07808	0	0
tsrna-15673	GCTAGCTCAGTCGGTAGAGCAT	0	0	0.069847	0
tsrna-15672	GCTAGCTCAGTCGGTAGAGCA	0	0.07808	0	0
tsrna-15666	GCTAATGGTGGAGTTAAAGACTTTTTCTCTGACCA	1.78540541	4.060166	1.327089	1.332326
tsrna-15665	GCTAATGGTGGAGTTAAAGACTTTTTCTCTGACC	1.21407568	1.795843	0.488928	0.111027
tsrna-15664	GCTAATGGTGGAGTTAAAGACTTTTTCTCTGAC	0.35708108	1.249282	0.20954	0
tsrna-15663	GCTAATGGTGGAGTTAAAGACTTTTTCTCT	0.78557838	1.015041	0.20954	0.111027
tsrna-15662	GCTAATGGTGGAGTTAAAGACTTTTTCTC	0.21424865	1.561602	0.349234	0.111027
tsrna-15661	GCTAATGGTGGAGTTAAAGACTTTTTCT	0.21424865	0.780801	0.069847	0.111027

tsrna-15660	GCTAATGGTGGAGTTAAAGACTTTTTC	0.64274595	0.702721	0.349234	0
tsrna-15659	GCTAATGGTGGAGTTAAAGACTTTTT	0.21424865	1.015041	0.139694	0.111027
tsrna-15658	GCTAATGGTGGAGTTAAAGACTTTT	0.14283243	0.390401	0.139694	0
tsrna-15657	GCTAATGGTGGAGTTAAAGACTTT	0.21424865	0.07808	0.139694	0.111027
tsrna-15656	GCTAATGGTGGAGTTAAAGACTT	0.14283243	0.23424	0.139694	0
tsrna-15655	GCTAATGGTGGAGTTAAAGACT	0.21424865	0.546561	0.139694	0
tsrna-15654	GCTAATGGTGGAGTTAAAGAC	0	0.31232	0	0
tsrna-15653	GCTAATGGTGGAGTTAAAGA	0.14283243	0.07808	0.069847	0
tsrna-15652	GCTAATGGTGGAGTTAAAG	0.35708108	0.546561	0	0
tsrna-15651	GCTAATGGTGGAGTTAAA	0.07141622	0.15616	0	0
tsrna-15650	GCTAATGGTGGAGTTAA	0.07141622	0.23424	0	0.111027
tsrna-15649	GCTAATGGTGGAGTTA	0	0.23424	0	0
tsrna-15648	GCTAATCCATTGTGCTTTGCACGCGTGGGT	0.07141622	0.702721	0.139694	0
tsrna-15639	GCTAACTCATGCCCCCATGTCTAACAACATGGCTTTCTCACC/	1.2854919	0.468481	2.724025	0.111027
tsrna-15638	GCTAACTCATGCCCCCATGTCTAACAACATGGCTTTCTCACC	0.64274595	0.23424	0.628621	0.111027
tsrna-15637	GCTAACTCATGCCCCCATGTCTAACAACATGGCTTTCTCA	0.21424865	0	0.20954	0
tsrna-15636	GCTAACTCATGCCCCCATGTCTAACAACATGGCT	0.14283243	0.07808	0.069847	0
tsrna-15635	GCTAACTCATGCCCCCATGTCTAACAACAT	0	0.07808	0	0
tsrna-15634	GCTAACTCATGCCCCCATGTCTAACAACA	0	0	0	0
tsrna-15633	GCTAACTCATGCCCCCATGTCTAACAAC	0	0	0	0
tsrna-15632	GCTAACTCATGCCCCCATGTCTAACA	0	0	0	0
tsrna-15631	GCTAACTCATGCCCCCATGTCTAAC	0	0	0	0
tsrna-15629	GCTAACTCATGCCCCCATGTCTA	0	0.07808	0	0
tsrna-15628	GCTAACTCATGCCCCCATGTCT	0.07141622	0	0	0
tsrna-15621	GCTAAATCCTATATATCTTACCA	0	0.07808	0	0
tsrna-15615	GCTAAATAAGCTATCGGGCCCATACCCCG	0.07141622	0.23424	0.139694	0
tsrna-15614	GCTAAATAAGCTATCGGGCCCATACC	0	0	0.069847	0.111027
tsrna-15613	GCTAAATAAGCTATCGGGCCCATA	0.07141622	0	0.069847	0
tsrna-15612	GCTAAATAAGCTATCGGGCCCAT	0	0	0	0
tsrna-15611	GCTAAATAAGCTATCGGGCCCA	0	0.15616	0	0
tsrna-15608	GCTAAATAAGCTATCGGGC	0.07141622	0	0	0
tsrna-15604	GCGTTTGGCTGTTAACTAAA	0	0	0	0.111027
tsrna-15598	GCGTTGGTGGTTTAGTGGTAGAATTCTCGC	0.14283243	0	0.139694	0
tsrna-15597	GCGTTGGTGGTTTAGTGGTAGAATTCTCG	0.07141622	0	0.069847	0
tsrna-15591	GCGTTGGTGGTGTAGTGGTGAGCA	0.21424865	0.07808	0	0
tsrna-15590	GCGTTGGTGGTGTAGTGGTGAGC	0	0.07808	0	0
tsrna-15582	GCGTTGGTGGTATAGTGGTTAGCATAGCTGCCTTCC	0.49991351	0.15616	0.628621	0.111027
tsrna-15581	GCGTTGGTGGTATAGTGGTTAGCATAGCTGCCTTC	0.21424865	0.468481	0.419081	0.111027
tsrna-15580	GCGTTGGTGGTATAGTGGTTAGCATAGCTGCCTT	0.35708108	0.07808	0.349234	0.111027
tsrna-15579	GCGTTGGTGGTATAGTGGTTAGCATAGCTGCCT	0.49991351	0.468481	0.419081	0
tsrna-15578	GCGTTGGTGGTATAGTGGTTAGCATAGCTGCC	0.28566487	0.31232	0.20954	0.111027
tsrna-15577	GCGTTGGTGGTATAGTGGTTAGCATAGCTGC	0.21424865	0.31232	0	0
tsrna-15576	GCGTTGGTGGTATAGTGGTTAGCATAGCTG	0.07141622	0.15616	0.069847	0
tsrna-15575	GCGTTGGTGGTATAGTGGTTAGCATAGCT	0	0.15616	0	0
tsrna-15574	GCGTTGGTGGTATAGTGGTTAGCATAGC	0	0	0	0
tsrna-15573	GCGTTGGTGGTATAGTGGTTAGCATAG	0	0.07808	0	0.111027
tsrna-15572	GCGTTGGTGGTATAGTGGTTAGCATA	0	0.07808	0.069847	0
tsrna-15571	GCGTTGGTGGTATAGTGGTTAGCAT	0	0.07808	0	0
tsrna-15570	GCGTTGGTGGTATAGTGGTTAGCA	0	0.07808	0	0
tsrna-15569	GCGTTGGTGGTATAGTGGTTAGC	0	0.07808	0	0
tsrna-15568	GCGTTGGTGGTATAGTGGTTAG	0	0.07808	0	0
tsrna-15567	GCGTTGGTGGTATAGTGGTTA	0	0.15616	0	0
tsrna-15566	GCGTTGGTGGTATAGTGGTT	0	0	0.069847	0
tsrna-15565	GCGTTGGTGGTATAGTGGTGAGCATAGT	0.21424865	1.171202	0.069847	0
tsrna-15564	GCGTTGGTGGTATAGTGGTGAGCATAGCTGCCTTCCAAGCA(	0.71416216	1.873923	0.698468	0.222054
tsrna-15563	GCGTTGGTGGTATAGTGGTGAGCATAGCTGCCTTCC	0.28566487	1.093122	0.349234	0.222054
tsrna-15562	GCGTTGGTGGTATAGTGGTGAGCATAGCTGCCTTC	0.14283243	0.936961	0	0
tsrna-15561	GCGTTGGTGGTATAGTGGTGAGCATAGCTGCCTT	0.21424865	0.936961	0.139694	0.111027
tsrna-15560	GCGTTGGTGGTATAGTGGTGAGCATAGCTGCCT	0	0.624641	0.069847	0
tsrna-15559	GCGTTGGTGGTATAGTGGTGAGCATAGCTGCC	0.28566487	0.468481	0	0
tsrna-15558	GCGTTGGTGGTATAGTGGTGAGCATAGCTGC	0.14283243	0.468481	0	0.111027
tsrna-15557	GCGTTGGTGGTATAGTGGTGAGCATAGCTG	0.07141622	0.390401	0	0.222054
tsrna-15556	GCGTTGGTGGTATAGTGGTGAGCATAGCT	0.07141622	0.858881	0.069847	0.111027
tsrna-15555	GCGTTGGTGGTATAGTGGTGAGCATAGC	0	0.780801	0	0.222054

tsrna-15554	GCGTTGGTGGTATAGTGGTGAGCATAG	0	0.624641	0.069847	0
tsrna-15553	GCGTTGGTGGTATAGTGGTGAGCATA	0.14283243	0.390401	0.069847	0
tsrna-15552	GCGTTGGTGGTATAGTGGTGAGCAT	0.07141622	0.858881	0.069847	0.111027
tsrna-15551	GCGTTGGTGGTATAGTGGTGAGCA	0	0.624641	0.069847	0.111027
tsrna-15550	GCGTTGGTGGTATAGTGGTGAGC	0.07141622	0.390401	0	0
tsrna-15549	GCGTTGGTGGTATAGTGGTGAG	0	0.23424	0	0
tsrna-15548	GCGTTGGTGGTATAGTGGTGA	0	0.07808	0.069847	0.111027
tsrna-15547	GCGTTGGTGGTATAGTGGTG	0.07141622	0	0	0
tsrna-15546	GCGTTGGTGGTATAGTGGTAAGCATAGCTGCCTTCC	0.14283243	0.15616	0.20954	0.222054
tsrna-15545	GCGTTGGTGGTATAGTGGTAAGCATAGCTGCCTTC	0.28566487	0.31232	0.20954	0.111027
tsrna-15544	GCGTTGGTGGTATAGTGGTAAGCATAGCTGCCT	0.28566487	0.23424	0.20954	0.111027
tsrna-15543	GCGTTGGTGGTATAGTGGTAAGCATAGCTGCC	0.14283243	0.15616	0.069847	0.222054
tsrna-15542	GCGTTGGTGGTATAGTGGTAAGCATAGCTGC	0.14283243	0.15616	0.069847	0.444109
tsrna-15541	GCGTTGGTGGTATAGTGGTAAGCATAGCTG	0.07141622	0.07808	0	0.111027
tsrna-15540	GCGTTGGTGGTATAGTGGTAAGCATAGCT	0.07141622	0	0	0.333081
tsrna-15539	GCGTTGGTGGTATAGTGGTAAGCATAGC	0	0.15616	0	0
tsrna-15538	GCGTTGGTGGTATAGTGGTAAGCATAG	0	0.07808	0	0.111027
tsrna-15537	GCGTTGGTGGTATAGTGGTAAGCATA	0	0	0	0.111027
tsrna-15535	GCGTTGGTGGTATAGTGGTAAGCA	0	0.07808	0	0
tsrna-15534	GCGTTGGTGGTATAGTGGTAAGC	0.14283243	0	0	0
tsrna-15533	GCGTTGGTGGTATAGTGGTAAG	0	0.07808	0	0.111027
tsrna-15532	GCGTTGGTGGTATAGTGGTAA	0	0.23424	0	0
tsrna-15531	GCGTTGGTGGTATAGTGGTA	0	0.07808	0	0
tsrna-15529	GCGTTGGTGGTATAGTGG	0.07141622	0.23424	0	0
tsrna-15528	GCGTTGGTGGTATAGTG	0.07141622	0.07808	0	0
tsrna-15527	GCGTTGGTGGTATAGT	0.07141622	0	0	0
tsrna-15526	GCGTTCGGCTGTTAACC	0.07141622	0.31232	0	0
tsrna-15524	GCGTTCGATTCCCGGTCAGGGAACCA	1.21407568	18.58307	0.349234	0.222054
tsrna-15523	GCGTTCAGTTGATGCAGAGTGGG	0	0.07808	0	0
tsrna-15521	GCGTTCAGTTGATGCAG	0.07141622	0	0	0
tsrna-15516	GCGTTAGACTGAAGATCT	0	0.07808	0	0
tsrna-15515	GCGTGGGTTCGAATCCCATCCTCGTCGCCA	0.49991351	0.858881	0.279387	0
tsrna-15514	GCGTGGGTTCGAATCCCACTTCTGACACC	0.07141622	0.23424	0.069847	0.111027
tsrna-15513	GCGTGGGTTCGAATCCCACTTCTGACAC	0	0.07808	0.069847	0
tsrna-15512	GCGTGGCCTAATGGATAAGGCGTCTGACTT	0.21424865	0	0	0.111027
tsrna-15511	GCGTGGCCTAATGGATAAGGCGTCTGA	0.07141622	0	0	0
tsrna-15510	GCGTGGCCTAATGGATAAGGCGTC	0.07141622	0	0	0
tsrna-15499	GCGTGGCCGAGCGGTCTAAGGC	0	0.07808	0	0
tsrna-15494	GCGTGCTGGGCCCATAACCCAGA	0	0.07808	0	0.111027
tsrna-15493	GCGTGCTGGGCCCATAACCCAG	0	0.07808	0	0
tsrna-15492	GCGTGCTGGGCCCATAACCCA	0	0.07808	0	0.111027
tsrna-15491	GCGTGCTGGGCCCATAACCC	0.14283243	0	0	0
tsrna-15490	GCGTGCTGGGCCCATAACC	0.07141622	0	0	0.111027
tsrna-15485	GCGGTTATCACGTCTGCTTTAC	0.14283243	0	0.069847	0
tsrna-15475	GCGGTTAGGATTCCTGGTTTTCACCCAGGC	0.99982703	2.030083	0.419081	0.666163
tsrna-15474	GCGGTTAGGATTCCTGGTTTTCACCC	0.78557838	1.015041	0.419081	0.666163
tsrna-15473	GCGGTTAGGATTCCTGGTTTTCACC	0.71416216	0.468481	0.349234	0.222054
tsrna-15472	GCGGTTAGGATTCCTGGTTTTCAC	0.4284973	0.546561	0.069847	0.666163
tsrna-15471	GCGGTTAGGATTCCTGGTTTTCA	0.4284973	0.15616	0.139694	0.222054
tsrna-15470	GCGGTTAGGATTCCTGGTTTTC	0.14283243	0.31232	0.139694	0.111027
tsrna-15469	GCGGTTAGGATTCCTGGTTTT	0.35708108	0.31232	0	0.111027
tsrna-15468	GCGGTTAGGATTCCTGGTTT	0.14283243	0.15616	0	0.111027
tsrna-15467	GCGGTTAGGATTCCTGGTT	0.07141622	0.23424	0.069847	0
tsrna-15466	GCGGTTAGGATTCCTGGT	0.14283243	0.23424	0.139694	0
tsrna-15465	GCGGTTAGGATTCCTGG	0.07141622	0	0.139694	0
tsrna-15464	GCGGTTAGGATTCCTG	0	0	0	0
tsrna-15461	GCGGTCTAAGGCGCTGGATT	0	0.07808	0	0
tsrna-15457	GCGGTCTAAGGCGCTGCGTTCA	0.07141622	0	0	0.111027
tsrna-15450	GCGGTAGAGCATTTGACTG	0.07141622	0.07808	0	0
tsrna-15448	GCGGGTTCGAACCCCGTACGGGCCACC	0	0	0	0
tsrna-15447	GCGGGAGGCCCGGGTTT	0.35708108	0.390401	0	0
tsrna-15446	GCGGGAGGCCCGGGTTCGATTCCCGGCCAATGCACC	0.35708108	0.780801	0.139694	0.222054
tsrna-15445	GCGGGAGGCCCGGGTTCGATTCCCGGCCAATGC	0	0.468481	0.069847	0
tsrna-15444	GCGGGAGGCCCGGGTTCGATTCCCGGCCAATG	0.07141622	0	0	0

tsrna-15443	GCGGGAGGCCCGGGTTCGATTCCCGGCCA	0.07141622	0.07808	0.069847	0
tsrna-15442	GCGGGAGGCCCGGGTTCG	0.07141622	0.31232	0.069847	0
tsrna-15441	GCGGGAGGCCCGGGTTC	0.07141622	0.546561	0.069847	0
tsrna-15440	GCGGGAGGCCCGGGTT	0.14283243	0.23424	0.069847	0
tsrna-15439	GCGGGAGACCGGGGTTCGATTCCCCGACGGGGAGCCA	0.07141622	0.936961	0	0
tsrna-15438	GCGGGAGACCGGGGTTCGATTCCCCGACGGGGAGCC	0	0.390401	0	0
tsrna-15437	GCGGGAGACCGGGGTTCGATTCCCCGACGGGGAGC	0	0.07808	0	0
tsrna-15436	GCGGGAGACCGGGGTTCGATTCCCCGACGGGGAG	0	0	0	0
tsrna-15435	GCGGGAGACCGGGGTTCGATTCCCCGACGGGGA	0.07141622	0	0	0
tsrna-15428	GCGGGAGACCGGGGTTCAATTCCCCGACGGGGAGCCA	0	0.23424	0	0
tsrna-15425	GCGGGAGACCGGGGTTCAATTCCCCGACGG	0	0	0.069847	0
tsrna-15422	GCGGCCCGGGTTCGATTCCCGGTCAGGGAACCA	0.92841081	22.33091	0	0.999244
tsrna-15421	GCGGCCCGGGTTCGATTCCCGGTCAGGGAACC	0	0.702721	0.069847	0.111027
tsrna-15420	GCGGCCCGGGTTCGATTCCCGGTCAGGGAA	0	0	0.069847	0
tsrna-15418	GCGGCCCGGGTTCGATTCCCGGTCAGGG	0	0.15616	0.069847	0
tsrna-15415	GCGGCCCGGGTTCGATTCCCGGTCA	0	0.07808	0	0
tsrna-15409	GCGGCCCGGGTTCGATTCC	0	0.07808	0	0
tsrna-15408	GCGGCCCGGGTTCGATTC	0.07141622	0.07808	0	0
tsrna-15406	GCGGCCCGGGTTCGACTCCCGGTGTGGGAACCA	4.99913515	25.06372	2.165251	3.441841
tsrna-15405	GCGGCCCGGGTTCGACTCCCGGTGTGGGAACC	0.21424865	0.858881	0.20954	0.111027
tsrna-15404	GCGGCCCGGGTTCGACTCCCGGTGTGGGAA	0	0.07808	0	0
tsrna-15403	GCGGCCCGGGTTCGACTCCCGGTGTGGGA	0.14283243	0	0.139694	0
tsrna-15402	GCGGCCCGGGTTCGACTCCCGGTGTGGG	0	0.07808	0	0
tsrna-15401	GCGGCCCGGGTTCGACTCCCGGTGTGG	0.07141622	0	0	0
tsrna-15397	GCGGCCCGGGTTCGACTCCCGGT	0	0	0	0
tsrna-15396	GCGGCCCGGGTTCGACTCCCGG	0	0	0.069847	0
tsrna-15390	GCGGAAGCGTGCTGGGCCCATAACCCAGAG	1.14265946	3.982086	0.558774	0.222054
tsrna-15389	GCGGAAGCGTGCTGGGCCCATAACC	0.64274595	2.967044	0.768315	1.110271
tsrna-15388	GCGGAAGCGTGCTGGGCCCATA	0.99982703	3.201285	0.558774	0.888217
tsrna-15387	GCGGAAGCGTGCTGGGCCCAT	0.71416216	3.045124	0	0.333081
tsrna-15386	GCGGAAGCGTGCTGGGCCCA	0.28566487	3.123204	0.488928	0.77719
tsrna-15385	GCGGAAGCGTGCTGGGCCC	0.57132973	3.669765	0.139694	0.444109
tsrna-15384	GCGGAAGCGTGCTGGGCC	0.4284973	1.639682	0.069847	0
tsrna-15383	GCGGAAGCGTGCTGGGC	0.14283243	1.639682	0.069847	0.333081
tsrna-15382	GCGGAAGCGTGCTGGG	0.21424865	1.561602	0.139694	0.111027
tsrna-15381	GCGCTTTCACCGCCGCGGCCCGGGT	0.07141622	0.546561	0.139694	0.333081
tsrna-15380	GCGCTTTCACCGCCGCGGCCCGG	0.14283243	0.23424	0	0.222054
tsrna-15379	GCGCTTTCACCGCCGCGGCCCG	0.07141622	0.31232	0.069847	0
tsrna-15378	GCGCTTTCACCGCCGCGGCCC	0.07141622	0.23424	0	0
tsrna-15377	GCGCTTTCACCGCCGCGGCC	0.07141622	0.15616	0.069847	0.333081
tsrna-15376	GCGCTCTCACCGCCGCGGCCCGGGTTCGATTCCCGGTCAGG	3.21372974	23.42403	1.53663	1.221299
tsrna-15375	GCGCTCTCACCGCCGCGGCCCGGGTTCGATTCCCGGTCAGG	0.49991351	0.858881	0.419081	0.222054
tsrna-15374	GCGCTCTCACCGCCGCGGCCCGGGTTCGATTCCCGGTCAGG	0.28566487	0.31232	0.419081	0
tsrna-15373	GCGCTCTCACCGCCGCGGCCCGGGTTCGATTCCCGGTCAGG	0.07141622	0.23424	0.139694	0
tsrna-15372	GCGCTCTCACCGCCGCGGCCCGGGTTCGATTCCCGGTCAGG	0	0.31232	0.069847	0
tsrna-15370	GCGCTCTCACCGCCGCGGCCCGGGTTCG	0.07141622	0.23424	0	0
tsrna-15369	GCGCTCTCACCGCCGCGGCCCGGGTTC	0	0.07808	0	0
tsrna-15368	GCGCTCTCACCGCCGCGGCCCGGGTT	0	0.15616	0	0
tsrna-15365	GCGCTCTCACCGCCGCGGCCCGG	0	0	0	0
tsrna-15363	GCGCTCTCACCGCCGCGGCCC	0	0.07808	0	0
tsrna-15362	GCGCTCTCACCGCCGCGGCC	0	0.07808	0	0
tsrna-15357	GCGCGTTTGGCTGTTAACT	0.07141622	0	0	0
tsrna-15356	GCGCGTTCGGCTGTTAACCGAAAGGTTGGT	0.4284973	2.264323	0.628621	0.555136
tsrna-15355	GCGCGTTCGGCTGTTAACCGAAAGGT	0.49991351	1.249282	0.558774	0.111027
tsrna-15354	GCGCGTTCGGCTGTTAACCGAAAGG	0.49991351	1.327362	0.349234	0.111027
tsrna-15353	GCGCGTTCGGCTGTTAACCGAAAG	0.28566487	1.405442	0.20954	0.111027
tsrna-15352	GCGCGTTCGGCTGTTAACCGAAA	0.21424865	1.561602	0.488928	0.111027
tsrna-15351	GCGCGTTCGGCTGTTAACC	0.14283243	0.07808	0	0
tsrna-15350	GCGCGTTCGGCTGTTAAC	0	0.07808	0	0
tsrna-15346	GCGCCGTGGCTTAGTTGGTTAAAGC	0	0	0	0.111027
tsrna-15344	GCGCCGCTGGTGTAGTGGTATCATGCAAGATTCCCATTCTTG	12.2835892	8.354572	15.92507	0.999244
tsrna-15343	GCGCCGCTGGTGTAGTGGTATCATGCAAGATTCCCA	8.85561084	6.168329	6.286213	0.77719
tsrna-15342	GCGCCGCTGGTGTAGTGGTATCATGCAAGATTCCC	7.78436759	6.324489	7.194221	0.999244
tsrna-15341	GCGCCGCTGGTGTAGTGGTATCATGCAAGATTCC	6.35604326	5.856008	5.448051	0.666163

tsrna-15340	GCGCCGCTGGTGTAGTGGTATCATGCAAGATTC	5.07055137	5.465608	3.841574	1.332326
tsrna-15339	GCGCCGCTGGTGTAGTGGTATCATGCAAGATT	3.42797839	4.606726	3.841574	0.888217
tsrna-15338	GCGCCGCTGGTGTAGTGGTATCATGCAAGAT	1.71398919	3.591685	1.676323	0.666163
tsrna-15337	GCGCCGCTGGTGTAGTGGTATCATGCAAGA	1.2854919	3.591685	1.396936	0.77719
tsrna-15336	GCGCCGCTGGTGTAGTGGTATCATGCAAG	0.8569946	1.952003	1.396936	0.333081
tsrna-15335	GCGCCGCTGGTGTAGTGGTATCATGCAA	0.78557838	0.936961	0.698468	0
tsrna-15334	GCGCCGCTGGTGTAGTGGTATCATGCA	0.64274595	1.171202	0.698468	0
tsrna-15333	GCGCCGCTGGTGTAGTGGTATCATGC	0.28566487	0.390401	0.069847	0
tsrna-15332	GCGCCGCTGGTGTAGTGGTATCATG	0.07141622	0.07808	0.20954	0
tsrna-15331	GCGCCGCTGGTGTAGTGGTATCAT	0.07141622	0.390401	0.139694	0.111027
tsrna-15330	GCGCCGCTGGTGTAGTGGTATCA	0	0.624641	0.069847	0
tsrna-15329	GCGCCGCTGGTGTAGTGGTATC	0	0.390401	0.069847	0
tsrna-15328	GCGCCGCTGGTGTAGTGGTAT	0	0.23424	0	0
tsrna-15327	GCGCCGCTGGTGTAGTGGTA	0.07141622	0.468481	0.139694	0.111027
tsrna-15326	GCGCCGCTGGTGTAGTGGT	0	0.23424	0	0
tsrna-15325	GCGCCGCTGGTGTAGTGG	0.07141622	0.23424	0	0
tsrna-15323	GCGCCGCTGGTGTAGT	0	0	0.069847	0
tsrna-15320	GCGCAGCGGAAGCGTGCTGGGCCCA	1.07124325	3.982086	0.558774	0.333081
tsrna-15319	GCGCAGCGGAAGCGTGCTGGGCCC	0.71416216	4.372486	0.349234	0.77719
tsrna-15318	GCGCAGCGGAAGCGTGCTGGGCC	0.07141622	2.498564	0.069847	0.111027
tsrna-15317	GCGCAGCGGAAGCGTGCTGGGC	0.14283243	2.420483	0.069847	0.111027
tsrna-15316	GCGCAGCGGAAGCGTGCTGGG	0.21424865	2.264323	0	0.222054
tsrna-15315	GCGCAGCGGAAGCGTGCTGG	0	0.390401	0	0
tsrna-15314	GCGCAGCGGAAGCGTGCTG	0	0.15616	0	0
tsrna-15313	GCGCAGCGGAAGCGTGCT	0	0.07808	0	0
tsrna-15311	GCGCAATGGATAGCGCATTGGACT	0.64274595	1.171202	0.139694	0.444109
tsrna-15310	GCGCAATGGATAGCGCATTGG	0.49991351	0.390401	0.20954	0
tsrna-15309	GCGCAATGGATAGCGCATTG	0.07141622	0	0	0
tsrna-15308	GCGCAATGGATAGCGCATT	0	0	0	0.111027
tsrna-15307	GCGCAATGGATAACGCGTCTGACTACGG	0.14283243	0.31232	0	0.333081
tsrna-15306	GCGCAATGGATAACGCGTCTGACT	0.07141622	0.07808	0	0
tsrna-15305	GCGCAATGGATAACGCGTCTGAC	0.14283243	0.07808	0	0
tsrna-15300	GCGATCCGAGTTCAAATCTCGGTGGAACCTC	0	0.23424	0	0
tsrna-15299	GCGATCCGAGTTCAAATCTCGGTGGAACCT	0	0.07808	0	0
tsrna-15296	GCGAGTTCAATTCTCGCTGGGGCCTC	0.07141622	0	0	0
tsrna-15294	GCGAGTTCAAATCTCGCTGGGGCCTCC	0.07141622	0.624641	0	0.333081
tsrna-15293	GCGAGTTCAAATCTCGCTGGGGCCTC	0.07141622	0.07808	0.069847	0
tsrna-15290	GCGAGAGGTCCCGGGTTCAAATCCCGGACGAGCCCC	0.35708108	0.546561	0.20954	0
tsrna-15289	GCGAGAGGTCCCGGGTTCAAATCCCGGACG	0	0.15616	0	0
tsrna-15288	GCGAGAGGTCCCGGGTTCAAA	0	0.15616	0	0
tsrna-15287	GCGAGAGGTCCCGGGTTCA	0	0.23424	0	0
tsrna-15286	GCGAGAGGTCCCGGGTTC	0	0.07808	0	0.222054
tsrna-15285	GCGAGAGGTCCCGGGTT	0	0.07808	0	0
tsrna-15283	GCGAGAGGTAGCGGGATCG	0.49991351	1.015041	0.628621	0
tsrna-15282	GCGAGAGGTAGCGGGATC	0.78557838	0.780801	0.20954	0
tsrna-15281	GCGAGAGGTAGCGGGAT	0.07141622	0.23424	0	0.111027
tsrna-15280	GCGAGAGGTAGCGGGA	0.07141622	0.858881	0	0
tsrna-15279	GCGACCCGGGTTCGATTCCCGGGCGGCGC	0	0.15616	0	0
tsrna-15273	GCGACCCGGGTTCGATTCCCGG	0	0.07808	0	0
tsrna-15271	GCGAAAGGTCCCTGGA	0	0.23424	0	0
tsrna-15270	GCGAAAGGTCCCCGGTTT	0	0	0	0
tsrna-15269	GCGAAAGGTCCCCGGTTCGATCCCGGGCGGAAACACC	0.14283243	0.546561	0.279387	0.111027
tsrna-15268	GCGAAAGGTCCCCGGTTCGATCCCGGGCGG	0	0.15616	0	0
tsrna-15267	GCGAAAGGTCCCCGGTTCGAAACCGGGCGGAAACACCA	20.3536217	55.20264	9.010238	4.552113
tsrna-15266	GCGAAAGGTCCCCGGTTCGAAACCGGGCGGAAACACC	5.57046488	14.9133	1.74617	0.666163
tsrna-15265	GCGAAAGGTCCCCGGTTCGAAACCGGGCGGAAACA	0	0.390401	0	0
tsrna-15263	GCGAAAGGTCCCCGGTTCGAAACCGGGCG	0	0.23424	0.069847	0
tsrna-15262	GCGAAAGGTCCCCGGTTCGAAACCGGGCAG	0	0.23424	0.069847	0
tsrna-15261	GCGAAAGGTCCCCGGTTCGAAACCGGGC	0.07141622	0	0	0
tsrna-15260	GCGAAAGGTCCCCGGTTCGAAACCGG	0	0.15616	0	0
tsrna-15259	GCGAAAGGTCCCCGGTTCGAAACC	0	0.15616	0	0
tsrna-15258	GCGAAAGGTCCCCGGTTCGAAAC	0.07141622	0.15616	0	0
tsrna-15257	GCGAAAGGTCCCCGGTTCGAAA	0.07141622	0.23424	0	0
tsrna-15256	GCGAAAGGTCCCCGGTTCG	0.14283243	0.07808	0	0

tsrna-15255	GCGAAAGGTCCCCGGTTC	0.07141622	0	0.069847	0
tsrna-15254	GCGAAAGGTCCCCGGTT	0	0.07808	0	0
tsrna-15251	GCCTTGGTGGTGCAGTGGTAGAATTCTCGC	0.21424865	0.07808	0	0
tsrna-15250	GCCTTGGTGGTGCAGTGGTAGAATTCTCG	0.21424865	0.07808	0	0
tsrna-15249	GCCTTCGATAGCTCAGTTGGTAGAGCGGAG	0.49991351	1.639682	0.488928	0.444109
tsrna-15248	GCCTTCCAAGCAGTTGACCCGGGT	2.14248649	2.810884	0.977855	1.887461
tsrna-15247	GCCTTCCAAGCAGTTGACCCGGG	0	0	0.20954	0.111027
tsrna-15246	GCCTTCCAAGCAGTTGACCCGG	0	0	0.20954	0.111027
tsrna-15245	GCCTTCCAAGCAGTTGACCCG	0.14283243	0.15616	0	0.111027
tsrna-15244	GCCTTCAAAGCCCTCAGTAAGTTGCAATACTTAATTTCTGCCA	0.64274595	0.23424	0.419081	0.333081
tsrna-15242	GCCTGTCACGCGGGAGACCGGGGTTCGATTCCCCGACGGG(	1.49974054	5.231367	0.768315	0.222054
tsrna-15241	GCCTGTCACGCGGGAGACCGGGGTTCGATTCCCCGACGGG(	1.14265946	3.357445	0.419081	0.333081
tsrna-15240	GCCTGTCACGCGGGAGACCGGGGTTCGATTCCCCGACGGG(	1.07124325	4.919047	0.349234	0.555136
tsrna-15239	GCCTGTCACGCGGGAGACCGGGGTTCGATTCCCCGACGGG(	1.21407568	4.762887	0.628621	0.222054
tsrna-15238	GCCTGTCACGCGGGAGACCGGGGTTCGATTCCCCGACGGG	0.71416216	3.435525	0.488928	0.666163
tsrna-15237	GCCTGTCACGCGGGAGACCGGGGTTCGATT	1.14265946	4.138246	0.558774	0.444109
tsrna-15236	GCCTGTCACGCGGGAGACCGGGGTTCG	0.92841081	3.591685	0.558774	0.111027
tsrna-15235	GCCTGTCACGCGGGAGACCGGGGTTCAATT	0.57132973	4.606726	0.488928	0.333081
tsrna-15234	GCCTGTCACGCGGGAGACCGGGGTTC	0.8569946	2.576644	0.628621	0.333081
tsrna-15233	GCCTGTCACGCGGGAGACCGGGGTT	1.07124325	4.372486	0.698468	0.222054
tsrna-15232	GCCTGTCACGCGGGAGACCGGGGT	1.14265946	3.904006	0.349234	0.222054
tsrna-15231	GCCTGTCACGCGGGAGACCGGGG	0.78557838	3.982086	0.419081	0.111027
tsrna-15230	GCCTGTCACGCGGGAGACCGGG	0.64274595	3.201285	0.838162	0.444109
tsrna-15229	GCCTGTCACGCGGGAGACCGG	0.8569946	3.435525	0.279387	0.333081
tsrna-15228	GCCTGTCACGCGGGAGACCG	0.8569946	3.669765	0.628621	0.222054
tsrna-15227	GCCTGTCACGCGGGAGACC	0.99982703	3.357445	0.279387	0
tsrna-15226	GCCTGTCACGCGGGAGAC	1.21407568	3.982086	0.558774	0.555136
tsrna-15225	GCCTGTCACGCGGGAGA	1.21407568	4.138246	0.768315	0.444109
tsrna-15224	GCCTGTCACGCGGGAG	0.57132973	4.138246	0.908008	0.555136
tsrna-15223	GCCTGGGTAGCTCAGTCGGTAGAGCATCAGAC	1.07124325	1.015041	0.488928	0.111027
tsrna-15222	GCCTGGGTAGCTCAGTCGGTAGAGCATCAG	0.28566487	0.390401	0.349234	0
tsrna-15221	GCCTGGGTAGCTCAGTCGGTAGAGCATCA	0.35708108	0.15616	0.069847	0
tsrna-15220	GCCTGGGTAGCTCAGTCGGTAGAGCATC	0.14283243	0.15616	0.069847	0
tsrna-15219	GCCTGGGTAGCTCAGTCGGTAGAGCAT	0	0.07808	0	0
tsrna-15218	GCCTGGGTAGCTCAGTCGGTAGAGCA	0	0	0.069847	0
tsrna-15216	GCCTGGGTAGCTCAGTCGGTAGAG	0	0	0	0
tsrna-15211	GCCTGGGTAGCTCAGTCG	0.07141622	0	0	0
tsrna-15209	GCCTGGATAGCTCAGTTGGTAGAGCATCAGAC	0.21424865	0	0.20954	0
tsrna-15208	GCCTGGATAGCTCAGTTGGTAGAGCATCAG	0.07141622	0	0	0
tsrna-15207	GCCTGGATAGCTCAGTTGGTAGAGCATC	0	0	0	0
tsrna-15206	GCCTGGATAGCTCAGTTGGTAGAGCAT	0	0	0.069847	0.111027
tsrna-15205	GCCTGGATAGCTCAGTTGGTAGAGCA	0.07141622	0.07808	0	0
tsrna-15204	GCCTGGATAGCTCAGTTGGTAGAGC	0	0	0	0
tsrna-15203	GCCTGGATAGCTCAGTTGGTAGAG	0	0.23424	0.139694	0
tsrna-15202	GCCTGGATAGCTCAGTTGGTAG	0.07141622	0.07808	0.069847	0.111027
tsrna-15201	GCCTGGATAGCTCAGTTGGT	0	0	0	0
tsrna-15199	GCCTGGATAGCTCAGTCGGTAGAGCATCAGACT	1.21407568	1.639682	1.606477	0.444109
tsrna-15198	GCCTGGATAGCTCAGTCGGTAGAGCATCAGAC	0.78557838	0.624641	0.488928	0.333081
tsrna-15197	GCCTGGATAGCTCAGTCGGTAGAGCATCAG	0.21424865	0.624641	0.419081	0.333081
tsrna-15196	GCCTGGATAGCTCAGTCGGTAGAGCATCA	0.35708108	0.31232	0.349234	0.444109
tsrna-15195	GCCTGGATAGCTCAGTCGGTAGAGCATC	0.35708108	0.31232	0.20954	0
tsrna-15194	GCCTGGATAGCTCAGTCGGTAGAGCAT	0.07141622	0.07808	0.139694	0.333081
tsrna-15193	GCCTGGATAGCTCAGTCGGTAGAGCA	0.14283243	0.07808	0.069847	0.111027
tsrna-15192	GCCTGGATAGCTCAGTCGGTAGAGC	0	0	0.069847	0
tsrna-15191	GCCTGGATAGCTCAGTCGGTAGAG	0	0.07808	0.069847	0
tsrna-15190	GCCTGGATAGCTCAGTCGGTAGA	0	0	0	0.111027
tsrna-15189	GCCTGGATAGCTCAGTCGGTAG	0	0	0.069847	0
tsrna-15188	GCCTGGATAGCTCAGTCGGTA	0	0.07808	0	0
tsrna-15185	GCCTGGATAGCTCAGTCG	0	0	0	0
tsrna-15183	GCCTGCCACGCGGGAGGCCCGGGTTCG	0.35708108	1.483522	0.20954	0.111027
tsrna-15182	GCCTGCCACGCGGGAGGCCCGGGTT	0.21424865	1.171202	0.069847	0.111027
tsrna-15181	GCCTGCCACGCGGGAGGCCCGGGT	0.28566487	1.093122	0	0.222054
tsrna-15179	GCCTGCCACGCGGGAGGCCCGG	0	0.07808	0	0
tsrna-15178	GCCTGCCACGCGGGAGGCCCG	0	0.07808	0	0

tsrna-15177	GCCTGCCACGCGGGAGGCCC	0	0.07808	0	0
tsrna-15170	GCCTCGTTAGCGCAGTAGGTAGCGCGTCAGTC	0.07141622	0.07808	0	0
tsrna-15169	GCCTCGTTAGCGCAGTAGGTAGCGCGTCAG	0	0.07808	0	0
tsrna-15163	GCCTCGTTAGCGCAGTAGGTA	0	0.07808	0	0
tsrna-15156	GCCTCGTGGCGCAACGGTAGCGCGTCTGAC	0.07141622	0.07808	0	0
tsrna-15154	GCCTCCTTAGCGCAGTAGGCAGCG	0.07141622	0	0	0
tsrna-15151	GCCTCCTTAGCGCAGTAGGCA	0	0	0	0
tsrna-15150	GCCTCCTTAGCGCAGTAGG	0	0.15616	0	0
tsrna-15148	GCCTCCTTAGCGCAGTA	0	0	0	0
tsrna-15147	GCCTCCTTAGCGCAGT	0	0	0	0
tsrna-15146	GCCTCCTAAGCCAGGGATTGTGGGT	9.78402165	28.6554	6.216366	4.885194
tsrna-15145	GCCTCCTAAGCCAGGGATTGTGGG	0.07141622	0.31232	0.139694	0
tsrna-15144	GCCTCCTAAGCCAGGGATTGTGG	0	0	0	0
tsrna-15143	GCCTCCTAAGCCAGGGATTGTG	0	0.07808	0	0.111027
tsrna-15142	GCCTCCGGAGCTGGGGATTGTGGGT	0.14283243	0.07808	0	0.111027
tsrna-15141	GCCTCCGGAGCTGGGGATTGTGGG	0	0.07808	0	0
tsrna-15132	GCCTCACACGCGAAAGGTCCCCG	0	0	0	0
tsrna-15130	GCCTAATGGATAAGGCGTCTGACT	0	0	0.069847	0
tsrna-15129	GCCTAATGGATAAGGCATTGGCCTCCTAAG	0.4284973	0.546561	0.419081	0.111027
tsrna-15128	GCCTAATGGATAAGGCATTGGCCTCC	0.21424865	0.15616	0.279387	0.111027
tsrna-15127	GCCTAATGGATAAGGCATTGGCCTC	0.35708108	0.07808	0.139694	0.333081
tsrna-15126	GCCTAATGGATAAGGCATTGGCCT	0.4284973	0.23424	0.139694	0
tsrna-15125	GCCTAATGGATAAGGCATTGGCC	0.21424865	0.07808	0.20954	0
tsrna-15124	GCCTAATGGATAAGGCATTGGC	0.28566487	0.31232	0.20954	0.222054
tsrna-15123	GCCTAATGGATAAGGCATTGG	0.4284973	0.23424	0.279387	0.111027
tsrna-15122	GCCTAATGGATAAGGCATTG	0.28566487	0.15616	0.139694	0
tsrna-15121	GCCTAATGGATAAGGCATCAGCCTCCGGAG	0.28566487	0.07808	0.20954	0
tsrna-15120	GCCTAATGGATAAGGCATCAGCCTCC	0.28566487	0.23424	0.419081	0
tsrna-15119	GCCTAATGGATAAGGCATCAGCCTC	0.21424865	0.07808	0.139694	0.111027
tsrna-15118	GCCTAATGGATAAGGCATCAGCCT	0.28566487	0	0.069847	0.222054
tsrna-15117	GCCTAATGGATAAGGCATCAGCC	0.35708108	0.23424	0.069847	0
tsrna-15116	GCCTAATGGATAAGGCATCAGC	0.14283243	0.15616	0.279387	0
tsrna-15115	GCCTAATGGATAAGGCATCAG	0.07141622	0.468481	0.20954	0
tsrna-15114	GCCTAATGGATAAGGCATC	0.14283243	0.07808	0.069847	0
tsrna-15111	GCCGTGATCGTATAGTGGTTAGTACTCTGCGTTGTGGCCGCA	1.78540541	1.483522	0.908008	0.222054
tsrna-15110	GCCGTGATCGTATAGTGGTTAGTACTCTGCGTTGTGGCCG	1.64257298	1.483522	1.396936	0.888217
tsrna-15109	GCCGTGATCGTATAGTGGTTAGTACTCTGCGTTGTGGCC	2.21390271	0.936961	0.838162	0.77719
tsrna-15108	GCCGTGATCGTATAGTGGTTAGTACTCTGCGTTGTG	2.35673514	0.702721	1.396936	0.888217
tsrna-15107	GCCGTGATCGTATAGTGGTTAGTACTCTGCGTTGT	2.07107028	1.093122	0.977855	0.999244
tsrna-15106	GCCGTGATCGTATAGTGGTTAGTACTCTGCGTTG	1.64257298	1.171202	1.117549	0.666163
tsrna-15105	GCCGTGATCGTATAGTGGTTAGTACTCTGCGTT	1.49974054	0.780801	0.628621	0.999244
tsrna-15104	GCCGTGATCGTATAGTGGTTAGTACTCTGCGT	0.57132973	0.23424	0.20954	0.222054
tsrna-15103	GCCGTGATCGTATAGTGGTTAGTACTCTGCG	0.57132973	0.15616	0.139694	0.333081
tsrna-15102	GCCGTGATCGTATAGTGGTTAGTACTCTGC	0.14283243	0.15616	0.069847	0.222054
tsrna-15101	GCCGTGATCGTATAGTGGTTAGTACTCTG	0.14283243	0.07808	0.069847	0.222054
tsrna-15100	GCCGTGATCGTATAGTGGTTAGTACTCT	0	0	0.069847	0.111027
tsrna-15099	GCCGTGATCGTATAGTGGTTAGTACTC	0	0	0	0.111027
tsrna-15098	GCCGTGATCGTATAGTGGTTAGTACT	0.07141622	0.07808	0.069847	0
tsrna-15094	GCCGTGATCGTATAGTGGTTAG	0	0	0	0
tsrna-15087	GCCGGTTAGCTCAGTTGGTTAGA	0	0	0	0
tsrna-15086	GCCGGTTAGCTCAGTTGGTTAG	0.07141622	0	0	0
tsrna-15085	GCCGGTTAGCTCAGTTGGTT	0	0.07808	0	0
tsrna-15084	GCCGGTTAGCTCAGTTGGTCAGAGC	0	0	0	0.111027
tsrna-15083	GCCGGTTAGCTCAGTTGGTCAGA	0.14283243	0.07808	0	0
tsrna-15082	GCCGGTTAGCTCAGTTGGT	0	0	0.069847	0
tsrna-15081	GCCGGTTAGCTCAGTTGG	0.07141622	0	0	0
tsrna-15080	GCCGGTTAGCTCAGTTG	0	0	0.069847	0
tsrna-15079	GCCGGTTAGCTCAGTT	0	0	0.069847	0
tsrna-15078	GCCGGTTAGCTCAGTCGGC	0	0	0.069847	0.111027
tsrna-15077	GCCGGTTAGCTCAGTCGG	0	0	0	0.111027
tsrna-15076	GCCGCTGGTGTAGTGGTATCATGCAAGATTCCC	0.71416216	0.468481	0.908008	0.111027
tsrna-15075	GCCGCTGGTGTAGTGGTATCATGCAAGATT	0.35708108	0.23424	0.349234	0
tsrna-15074	GCCGCTGGTGTAGTGGTATCATGCAAGAT	0.14283243	0.07808	0.139694	0
tsrna-15073	GCCGCTGGTGTAGTGGTATCATGCAAGA	0.28566487	0	0.069847	0

tsrna-15072	GCCGCTGGTGTAGTGGTATCATGCAAG	0	0.07808	0.139694	0.111027
tsrna-15071	GCCGCTGGTGTAGTGGTATCATGCAA	0.07141622	0.07808	0.069847	0
tsrna-15070	GCCGCTGGTGTAGTGGTATCATGCA	0.14283243	0	0	0
tsrna-15068	GCCGCTGGTGTAGTGGTATCATG	0	0	0	0
tsrna-15066	GCCGCGTGGCCTAATGGATAAGGCGTCTG	0	0.15616	0	0.111027
tsrna-15058	GCCGCGGCCCGGGTTCGATTCCCGGT	0	0	0	0
tsrna-15057	GCCGCGGCCCGGGTTCGATTCCC	0	0.07808	0	0
tsrna-15055	GCCGCGGCCCGGGTTCGATTC	0	0	0	0
tsrna-15051	GCCGCGGCCCGGGTTCG	0	0.07808	0	0
tsrna-15049	GCCGCAGCAACCTCGGTTCGAATCCGAGTCACGGCACCA	0.57132973	2.654724	0.139694	0
tsrna-15048	GCCGCAGCAACCTCGG	0	0	0.069847	0
tsrna-15040	GCCGAGGTTGTGAGTTCG	0	0.07808	0	0
tsrna-15037	GCCGAGCGGTCTAAGGCGCTGCGTTC	0	0.07808	0	0
tsrna-15032	GCCGAGATAGCTCAGTTGGGA	0	0	0.139694	0
tsrna-15031	GCCGAGATAGCTCAGTTGGG	0	0.07808	0	0
tsrna-15030	GCCGAGATAGCTCAGTTGG	0	0.07808	0	0.111027
tsrna-15029	GCCGAAATAGCTCAGTTGGGAGAGCGTTAGACT	0.49991351	0.624641	0.419081	0.333081
tsrna-15028	GCCGAAATAGCTCAGTTGGGAGAGCGTTAG	0.28566487	0.624641	0.419081	0.444109
tsrna-15027	GCCGAAATAGCTCAGTTGGGAGAGC	0.71416216	0.31232	0.069847	0.444109
tsrna-15026	GCCGAAATAGCTCAGTTGGGAGAG	0.14283243	0.23424	0.349234	0.555136
tsrna-15025	GCCGAAATAGCTCAGTTGGGAGA	0.4284973	0.468481	0.628621	0.555136
tsrna-15024	GCCGAAATAGCTCAGTTGGGAG	0.71416216	0.31232	0.419081	0.333081
tsrna-15023	GCCGAAATAGCTCAGTTGGGA	0.78557838	0.31232	0.20954	0.666163
tsrna-15022	GCCGAAATAGCTCAGTTGGG	0.35708108	1.327362	0.349234	0.77719
tsrna-15021	GCCGAAATAGCTCAGTTGG	0.57132973	0.702721	0.349234	0.77719
tsrna-15020	GCCGAAATAGCTCAGTTG	0.49991351	0.468481	0.279387	0.444109
tsrna-15019	GCCGAAATAGCTCAGTT	0.14283243	0.31232	0.279387	0.444109
tsrna-15018	GCCGAAATAGCTCAGT	0.57132973	0.23424	0.419081	0.444109
tsrna-15017	GCCCTGTGGTCTAGTGGTTAGG	0.28566487	0.390401	0.488928	0.333081
tsrna-15014	GCCCTCTTAGCGCAGTGGG	0	0	0.069847	0
tsrna-15013	GCCCTCTTAGCGCAGCGGGCAGC	0.07141622	0.15616	0	0
tsrna-15012	GCCCTCTTAGCGCAGCGGG	0.07141622	0	0	0
tsrna-15011	GCCCTCTTAGCGCAGCGG	0	0	0	0
tsrna-15000	GCCCGGTAATCGCATAAAACTTAAAACTTT	0.21424865	0.624641	1.327089	0.111027
tsrna-14993	GCCCGGGTTCGATTCCCGGTCAGGGAACC	0	0.546561	0	0
tsrna-14992	GCCCGGGTTCGATTCCCGGTCAGGGAAC	0	0.23424	0	0
tsrna-14991	GCCCGGGTTCGATTCCCGGTCAGGGAA	0.07141622	0.15616	0	0
tsrna-14990	GCCCGGGTTCGATTCCCGGTCAGG	0	0.07808	0	0
tsrna-14989	GCCCGGGTTCGACTCCCGGTGTGGGAACC	0.14283243	0.624641	0	0.111027
tsrna-14988	GCCCGGGTTCGACTCCCGGTGTGGGAAC	0	0.07808	0	0
tsrna-14987	GCCCGGGTTCGACTCCCGGTGTGGGAA	0	0.15616	0	0
tsrna-14985	GCCCGGGTTCGACTCCCGGTGTGGG	0.14283243	0	0	0
tsrna-14984	GCCCGGGTTCGACTCCCGGTGTGG	0	0.15616	0	0
tsrna-14983	GCCCGGCTAGCTCAGTCGGTAGAGCATGGGACTCTTAATCT(	25.7812541	10.69698	31.98984	16.09893
tsrna-14982	GCCCGGCTAGCTCAGTCGGTAGAGCATGGGACTCTTAATCC(	31.0660541	10.30657	32.40892	15.43277
tsrna-14981	GCCCGGCTAGCTCAGTCGGTAGAGCATGGGACTCTTAATCC	23.4959352	11.3997	27.10056	15.43277
tsrna-14980	GCCCGGCTAGCTCAGTCGGTAGAGCATGGGACTCTTAATC	25.9240866	9.213453	28.00857	14.10045
tsrna-14979	GCCCGGCTAGCTCAGTCGGTAGAGCATGGGACTCTTAAT	21.3534487	10.22849	23.95745	15.21072
tsrna-14978	GCCCGGCTAGCTCAGTCGGTAGAGCATGGGACTCTTAA	23.2816866	9.057293	25.00516	14.54456
tsrna-14977	GCCCGGCTAGCTCAGTCGGTAGAGCATGGGACTCTT	23.3531028	9.603854	22.77006	15.76585
tsrna-14976	GCCCGGCTAGCTCAGTCGGTAGAGCATGGGACTCT	13.9261622	8.510732	14.3186	9.881415
tsrna-14975	GCCCGGCTAGCTCAGTCGGTAGAGCATGGGACTC	4.21355677	4.138246	3.841574	3.663896
tsrna-14974	GCCCGGCTAGCTCAGTCGGTAGAGCATGGGACT	1.64257298	2.108163	2.304945	1.443353
tsrna-14973	GCCCGGCTAGCTCAGTCGGTAGAGCATGGGAC	1.64257298	1.952003	1.047702	0.77719
tsrna-14972	GCCCGGCTAGCTCAGTCGGTAGAGCATGGGA	0.71416216	1.561602	0.628621	0.666163
tsrna-14971	GCCCGGCTAGCTCAGTCGGTAGAGCATGGG	0.4284973	0.23424	0.139694	0.444109
tsrna-14970	GCCCGGCTAGCTCAGTCGGTAGAGCATGG	0.14283243	0.702721	0.419081	0.222054
tsrna-14969	GCCCGGCTAGCTCAGTCGGTAGAGCATGAGACTCTTAATCT	29.0664001	23.58019	28.77688	14.98866
tsrna-14968	GCCCGGCTAGCTCAGTCGGTAGAGCATGAGACTCTTAATC	25.9240866	26.46916	22.63037	15.09969
tsrna-14967	GCCCGGCTAGCTCAGTCGGTAGAGCATGAGACTCTTAAT	27.2095785	22.17475	21.23343	12.99018
tsrna-14966	GCCCGGCTAGCTCAGTCGGTAGAGCATGAGACTCTTAA	24.7814271	21.00355	17.39185	13.21223
tsrna-14965	GCCCGGCTAGCTCAGTCGGTAGAGCATGAGACTCTT	25.1385082	23.97059	21.37312	13.32326
tsrna-14964	GCCCGGCTAGCTCAGTCGGTAGAGCATGAGACTCT	15.8544	16.94338	12.22319	9.659361
tsrna-14963	GCCCGGCTAGCTCAGTCGGTAGAGCATGAGACTC	7.42728651	8.666892	5.23851	6.439574

tsrna-14962	GCCCGGCTAGCTCAGTCGGTAGAGCATGAGACT	1.14265946	2.108163	0.768315	0.444109
tsrna-14961	GCCCGGCTAGCTCAGTCGGTAGAGCATGAGACCCTT	1.21407568	2.186243	0.628621	0.666163
tsrna-14960	GCCCGGCTAGCTCAGTCGGTAGAGCATGAGACCC	1.07124325	1.873923	0.558774	0.555136
tsrna-14959	GCCCGGCTAGCTCAGTCGGTAGAGCATGAGACC	1.42832433	2.030083	0.628621	1.221299
tsrna-14958	GCCCGGCTAGCTCAGTCGGTAGAGCATGAGAC	0.8569946	1.952003	0.139694	0.999244
tsrna-14957	GCCCGGCTAGCTCAGTCGGTAGAGCATGAGA	0.64274595	1.171202	0.698468	0.222054
tsrna-14956	GCCCGGCTAGCTCAGTCGGTAGAGCATGAG	0.35708108	0.31232	0.419081	0.222054
tsrna-14955	GCCCGGCTAGCTCAGTCGGTAGAGCATGA	0.49991351	0.31232	0.139694	0.111027
tsrna-14954	GCCCGGCTAGCTCAGTCGGTAGAGCATG	0.4284973	0.780801	0.279387	0.111027
tsrna-14953	GCCCGGCTAGCTCAGTCGGTAGAGCAT	0.28566487	0.23424	0.139694	0.222054
tsrna-14952	GCCCGGCTAGCTCAGTCGGTAGAGCA	0.07141622	0.546561	0.279387	0.444109
tsrna-14951	GCCCGGCTAGCTCAGTCGGTAGAGC	0.07141622	0.468481	0	0.444109
tsrna-14950	GCCCGGCTAGCTCAGTCGGTAGAG	0.28566487	0.546561	0.069847	0.333081
tsrna-14949	GCCCGGCTAGCTCAGTCGGTAGA	0.21424865	0.390401	0.279387	0.444109
tsrna-14948	GCCCGGCTAGCTCAGTCGGTAG	0.14283243	0.31232	0.20954	0.222054
tsrna-14947	GCCCGGCTAGCTCAGTCGGTA	0.14283243	0.31232	0.069847	0.333081
tsrna-14946	GCCCGGCTAGCTCAGTCGGT	0.14283243	0.702721	0.139694	0.333081
tsrna-14945	GCCCGGCTAGCTCAGTCGG	0.07141622	0.15616	0.139694	0.111027
tsrna-14944	GCCCGGCTAGCTCAGTCGATAGAGCATGAGACTC	0.35708108	0.468481	0.139694	0.444109
tsrna-14943	GCCCGGCTAGCTCAGTCGATAGAGCATGAGACT	0.35708108	0.468481	0.279387	0.111027
tsrna-14942	GCCCGGCTAGCTCAGTCGA	0.28566487	0.390401	0	0
tsrna-14941	GCCCGGCTAGCTCAGTCG	0	0.23424	0	0
tsrna-14940	GCCCGGCTAGCTCAGTC	0.28566487	0.07808	0.20954	0
tsrna-14939	GCCCGGCTAGCTCAGT	0	0	0	0.222054
tsrna-14938	GCCCGGATAGCTCAGTCGGTAGAGCATCAGACTTTT	93.4124111	59.49704	103.3034	24.09289
tsrna-14937	GCCCGGATAGCTCAGTCGGTAGAGCATCAGACTTT	86.9849516	55.82728	95.48058	22.98262
tsrna-14936	GCCCGGATAGCTCAGTCGGTAGAGCATCAGACTT	83.9854705	56.53	98.41415	19.87386
tsrna-14935	GCCCGGATAGCTCAGTCGGTAGAGCATCAGACT	80.9145732	54.81224	90.03253	22.98262
tsrna-14934	GCCCGGATAGCTCAGTCGGTAGAGCATCAGAC	2.49956757	2.732804	1.955711	0.888217
tsrna-14933	GCCCGGATAGCTCAGTCGGTAGAGCATCAGA	1.49974054	1.639682	1.396936	0.444109
tsrna-14932	GCCCGGATAGCTCAGTCGGTAGAGCATCAG	1.14265946	0.31232	0.558774	0.888217
tsrna-14931	GCCCGGATAGCTCAGTCGGTAGAGCATCA	1.21407568	0.780801	1.187396	0.444109
tsrna-14930	GCCCGGATAGCTCAGTCGGTAGAGCATC	0.78557838	1.171202	0.628621	0.555136
tsrna-14929	GCCCGGATAGCTCAGTCGGTAGAGCAT	0.49991351	0.07808	0.279387	0.999244
tsrna-14928	GCCCGGATAGCTCAGTCGGTAGAGCA	0.28566487	0	0.20954	0.444109
tsrna-14927	GCCCGGATAGCTCAGTCGGTAGAGC	0.21424865	0.31232	0.279387	0.444109
tsrna-14926	GCCCGGATAGCTCAGTCGGTAGAG	0.28566487	0.15616	0.20954	0.222054
tsrna-14925	GCCCGGATAGCTCAGTCGGTAGA	0.14283243	0.31232	0.069847	0.333081
tsrna-14924	GCCCGGATAGCTCAGTCGGTAG	0.07141622	0	0.20954	0.444109
tsrna-14923	GCCCGGATAGCTCAGTCGGTA	0.07141622	0.07808	0	0
tsrna-14922	GCCCGGATAGCTCAGTCGGT	0.07141622	0.31232	0.139694	0.111027
tsrna-14921	GCCCGGATAGCTCAGTCGG	0	0.23424	0.069847	0
tsrna-14920	GCCCGGATAGCTCAGTCG	0	0	0	0
tsrna-14919	GCCCGGATAGCTCAGTC	0	0	0	0.111027
tsrna-14918	GCCCGGATAGCTCAGT	0.07141622	0.07808	0	0
tsrna-14917	GCCCGCATTCTCCACCA	0	0	0.069847	0.111027
tsrna-14915	GCCCGCATCCTCCACCA	0	0.07808	0.069847	0
tsrna-14913	GCCCCGGTGGCCTAATGGATAAGGCATTGG	0.35708108	0.07808	0.279387	0.111027
tsrna-14912	GCCCCGGTGGCCTAATGGATAAGGCATTG	0.14283243	0.468481	0.349234	0.222054
tsrna-14901	GCCCCGGGTTCGATCCCCGGCATCTCCAC	0	0	0.069847	0
tsrna-14893	GCCCCCATGTCTAACAACATGGCTTTCTCACCA	0	0.15616	0.419081	0
tsrna-14892	GCCCCCATGTCTAACAACATGGCTTTCTCACC	0.28566487	0	0.139694	0
tsrna-14884	GCCCCCATGTCTAACAACATGGC	0	0.07808	0	0
tsrna-14879	GCCCCATGGTGTAATGGTTAGCACTCTGGA	0	0.15616	0	0
tsrna-14878	GCCCCATGGTGTAATGGTTAGCACTCTGG	0.07141622	0.07808	0	0
tsrna-14872	GCCCCAGTGGCCTAATGGATAAGGCATTGG	0.14283243	0.15616	0.419081	0
tsrna-14871	GCCCCAGTGGCCTAATGGATAAGGCATTG	0.07141622	0.07808	0.139694	0
tsrna-14868	GCCCCAGTGGCCTAATGGATAAGGCACTGG	4.85630272	2.888964	3.771728	2.997733
tsrna-14867	GCCCCAGTGGCCTAATGGATAAGGCACTG	5.35621623	2.498564	3.562187	1.665407
tsrna-14866	GCCCCAGTGGCCTAATGGATAAGGCACT	0.28566487	0.23424	0.139694	0.222054
tsrna-14865	GCCCCAGTGGCCTAATGGATAAGGCAC	0	0	0	0
tsrna-14853	GCCCCAGTGGAACCACCA	0.14283243	0.07808	0	0
tsrna-14852	GCCCCAGTGGAACCACC	0	0	0	0
tsrna-14850	GCCCCACGTTGGGCGCCA	0.35708108	1.873923	0.279387	0.444109

tsrna-14849	GCCCCACGTTGGGCGCC	0	0.15616	0	0
tsrna-14848	GCCCATACCCCGAAAATGTTGGTT	0.07141622	0	0	0
tsrna-14843	GCCCATACCCCGAAAA	0.07141622	0	0	0
tsrna-14842	GCCCATAACCCAGAGGTCGATGGATC	0.21424865	0.624641	0.139694	0.111027
tsrna-14841	GCCCATAACCCAGAGGTCGATGGA	0.07141622	0.546561	0	0.111027
tsrna-14840	GCCCATAACCCAGAGGTCGATGG	0.14283243	0.07808	0	0.111027
tsrna-14839	GCCCATAACCCAGAGGTCGATG	0	0.07808	0.069847	0
tsrna-14838	GCCCATAACCCAGAGGTCGAT	0.07141622	0.07808	0	0
tsrna-14837	GCCCATAACCCAGAGGTCGA	0	0	0	0
tsrna-14836	GCCCATAACCCAGAGGTCG	0	0.07808	0	0
tsrna-14834	GCCCAGCTAGCTCAGTCGGTAGAGCATGAG	0.14283243	0.468481	0.279387	0.222054
tsrna-14833	GCCCAGCTAGCTCAGTCGGTAGAGC	0	0	0	0
tsrna-14832	GCCCAGCTAGCTCAGTCGGTAGAG	0.07141622	0	0	0
tsrna-14829	GCCCACCCAGGGACGCCA	0.14283243	4.919047	0.069847	0
tsrna-14828	GCCCACCCAGGGACGCC	0	0.07808	0	0
tsrna-14826	GCCCACATTCTCCACCA	0.07141622	0	0	0
tsrna-14825	GCCATGGGGTTGGCTTGAAACC	0	0.15616	0	0
tsrna-14823	GCCATGATCGTATAGTGGTTAGTACTCTGC	0.4284973	0.15616	0.20954	0.111027
tsrna-14822	GCCATGATCGTATAGTGGTTAGTACTCTG	0.14283243	0.07808	0	0
tsrna-14821	GCCATGATCGTATAGTGGTTAGTACTC	0	0	0	0.111027
tsrna-14820	GCCAGTTGATTAGGGTGCTTAGCTGTTAAC	0	0.23424	0.069847	0
tsrna-14819	GCCAGTTGATTAGGGTGCTTAGCTG	0	0	0	0
tsrna-14818	GCCAGTTGATTAGGGTGCTTAGCT	0	0.07808	0	0
tsrna-14815	GCCAGTTGATTAGGGTGCTTA	0.07141622	0	0	0
tsrna-14812	GCCAGGGATTGTGGGTTCGAGTCCCATCTGGGGTGCCA	0.07141622	1.327362	0.069847	0
tsrna-14811	GCCAGGGATTGTGGGTTCGAGTCCCATCTGGGGTGCC	0.21424865	0.468481	0.20954	0.111027
tsrna-14810	GCCAGGGATTGTGGGTTCGAGTCCC	0.14283243	0.546561	0.069847	0.111027
tsrna-14809	GCCAGGGATTGTGGGTTCGAGTCC	0.07141622	0.31232	0.139694	0
tsrna-14808	GCCAGGGATTGTGGGTTCG	0.07141622	0.31232	0.20954	0.222054
tsrna-14807	GCCAGGGATTGTGGGTTC	0	0.780801	0.20954	0.111027
tsrna-14806	GCCAGGGATTGTGGGT	0.21424865	0.780801	0.279387	0.111027
tsrna-14805	GCCACGCGGGAGGCCCGGGTTT	0.14283243	0.23424	0	0
tsrna-14804	GCCACGCGGGAGGCCCGGGTTCGATTCCCGGCCAATGCAC(	2.35673514	12.64898	0.768315	1.221299
tsrna-14803	GCCACGCGGGAGGCCCGGGTTCG	0.21424865	0.23424	0	0.111027
tsrna-14802	GCCACGCGGGAGGCCCGGGTTC	0	0.31232	0.069847	0
tsrna-14801	GCCACGCGGGAGGCCCGGGTT	0.14283243	0.624641	0	0
tsrna-14800	GCCACGCGGGAGGCCCGGGT	0.21424865	0.702721	0	0
tsrna-14797	GCCACGCGGGAGGCCCG	0	0	0.069847	0
tsrna-14796	GCCACGCGGGAGGCCC	0	0.07808	0	0
tsrna-14795	GCCAAGGTCGCGGGTTCGATCCCCGTA	0	0.23424	0	0
tsrna-14794	GCCAAGGTCGCGGGTTCGAT	0	0	0.069847	0
tsrna-14793	GCCAAGGTCGCGGGTTCG	0.07141622	0.07808	0	0
tsrna-14789	GCATTGGTGGTTCAGTGGTAGAATTCTCGCCTGCCACGCGG(	373.506812	70.89674	417.0553	41.7462
tsrna-14788	GCATTGGTGGTTCAGTGGTAGAATTCTCGCCTGCCACGCGG(	376.077796	69.02282	422.3636	40.74696
tsrna-14787	GCATTGGTGGTTCAGTGGTAGAATTCTCGCCTGCCACGCGG(	368.793342	71.28714	411.8168	42.85648
tsrna-14786	GCATTGGTGGTTCAGTGGTAGAATTCTCGCCTGCCACGCGG(	376.93479	72.45834	417.9633	47.07551
tsrna-14785	GCATTGGTGGTTCAGTGGTAGAATTCTCGCCTGCCACGCGG(	374.149558	75.26923	420.2682	41.41312
tsrna-14784	GCATTGGTGGTTCAGTGGTAGAATTCTCGCCTGCCACGCGG(	370.221666	84.24844	411.7469	50.51735
tsrna-14783	GCATTGGTGGTTCAGTGGTAGAATTCTCGCCTGCCACGCGG	375.077969	67.46122	421.3159	41.52415
tsrna-14782	GCATTGGTGGTTCAGTGGTAGAATTCTCGCCTGCCACGCG	364.079871	69.33514	412.0263	42.30134
tsrna-14781	GCATTGGTGGTTCAGTGGTAGAATTCTCGCCTGCCACGC	373.578228	70.2721	415.7282	45.52113
tsrna-14780	GCATTGGTGGTTCAGTGGTAGAATTCTCGCCTGCCACG	371.935655	69.8817	407.3466	41.96826
tsrna-14779	GCATTGGTGGTTCAGTGGTAGAATTCTCGCCTGCCAC	365.365363	69.72554	413.982	40.63593
tsrna-14778	GCATTGGTGGTTCAGTGGTAGAATTCTCGCCTGCCA	362.22305	66.60233	409.6515	41.08004
tsrna-14777	GCATTGGTGGTTCAGTGGTAGAATTCTCGCCTGCC	364.29412	67.38313	410.2103	42.63442
tsrna-14776	GCATTGGTGGTTCAGTGGTAGAATTCTCGCCTGC	352.867525	62.07369	378.8491	42.30134
tsrna-14775	GCATTGGTGGTTCAGTGGTAGAATTCTCGCCTG	351.010704	61.52713	376.0552	41.19107
tsrna-14774	GCATTGGTGGTTCAGTGGTAGAATTCTCGCCTCCCACGCGG(	307.589644	50.43975	366.6957	41.41312
tsrna-14773	GCATTGGTGGTTCAGTGGTAGAATTCTCGCCTCCCACGCGG(	310.232044	55.67112	362.924	43.18956
tsrna-14772	GCATTGGTGGTTCAGTGGTAGAATTCTCGCCTCCCACGC	321.872887	49.81511	361.6668	41.63518
tsrna-14771	GCATTGGTGGTTCAGTGGTAGAATTCTCGCCTCCCAC	315.374012	54.81224	358.5237	43.18956
tsrna-14770	GCATTGGTGGTTCAGTGGTAGAATTCTCGCCTCCCA	312.803028	50.51783	357.3363	43.96675
tsrna-14769	GCATTGGTGGTTCAGTGGTAGAATTCTCGCCTCCC	314.017104	54.1876	360.1301	38.74847
tsrna-14768	GCATTGGTGGTTCAGTGGTAGAATTCTCGCCTCC	308.23239	55.67112	365.9973	39.63669

tsrna-14767	GCATTGGTGGTTCAGTGGTAGAATTCTCGCCTC	303.947417	50.59591	357.1966	40.74696
tsrna-14766	GCATTGGTGGTTCAGTGGTAGAATTCTCGCCT	307.018314	51.61095	350.4214	36.86101
tsrna-14765	GCATTGGTGGTTCAGTGGTAGAATTCTCGCC	194.466357	30.9978	207.1656	24.64802
tsrna-14764	GCATTGGTGGTTCAGTGGTAGAATTCTCGC	14.1404109	8.276492	14.1789	7.105737
tsrna-14763	GCATTGGTGGTTCAGTGGTAGAATTCTCG	3.64222704	2.342403	2.304945	5.44033
tsrna-14762	GCATTGGTGGTTCAGTGGTAGAATTCTC	2.71381622	1.405442	2.374791	5.44033
tsrna-14761	GCATTGGTGGTTCAGTGGTAGAATTCT	1.92823784	1.327362	1.606477	2.997733
tsrna-14760	GCATTGGTGGTTCAGTGGTAGAATTC	1.42832433	1.639682	1.606477	2.553624
tsrna-14759	GCATTGGTGGTTCAGTGGTAGAATT	1.71398919	0.936961	1.466783	2.664651
tsrna-14758	GCATTGGTGGTTCAGTGGTAGAAT	1.07124325	0.624641	0.279387	0.888217
tsrna-14757	GCATTGGTGGTTCAGTGGTAGAA	0.49991351	1.015041	0.349234	0.444109
tsrna-14756	GCATTGGTGGTTCAGTGGTAGA	0.57132973	0.468481	0.349234	0.666163
tsrna-14755	GCATTGGTGGTTCAGTGGTAG	0.35708108	0.780801	0.139694	0.111027
tsrna-14754	GCATTGGTGGTTCAGTGGTA	0.14283243	0.390401	0.069847	0.111027
tsrna-14753	GCATTGGTGGTTCAGTGGT	0.07141622	0.390401	0.069847	0.222054
tsrna-14752	GCATTGGTGGTTCAGTGG	0.14283243	0.702721	0	0.111027
tsrna-14751	GCATTGGTGGTTCAGTG	0	0.07808	0	0
tsrna-14749	GCATTGGTGGTTCAATGGTAGAATTCTCGCCT	3.14231352	0.780801	4.679736	0.555136
tsrna-14748	GCATTGGTGGTTCAATGGTAGAATTCTCGCC	1.57115676	1.015041	1.396936	0.333081
tsrna-14747	GCATTGGTGGTTCAATGGTAGAATTCTCGC	0.57132973	0.780801	0	0
tsrna-14746	GCATTGGTGGTTCAATGGTAGAATTCTCG	0.4284973	0.390401	0.20954	0
tsrna-14745	GCATTGGTGGTTCAATGGTAGAATTCTC	0.28566487	0.624641	0	0
tsrna-14744	GCATTGGTGGTTCAATGGTAGAATTCT	0.21424865	0.31232	0.279387	0
tsrna-14743	GCATTGGTGGTTCAATGGTAGAA	0.14283243	0.468481	0.139694	0
tsrna-14742	GCATTGGTGGTTCAATGGTAGA	0.21424865	0.468481	0.139694	0
tsrna-14741	GCATTGGTGGTTCAATG	0.14283243	0.390401	0.069847	0
tsrna-14740	GCATTGGCCTCCTAAGCCAGGGATTGTGG	0.14283243	0.390401	0.069847	0
tsrna-14738	GCATTGGACTGTAAATCTAAAGACAGGGGTTAGGCC	0	0	0.20954	0
tsrna-14736	GCATTGGACTGTAAATCTAAAGACAGGGG	0	0.15616	0	0
tsrna-14735	GCATTGGACTGTAAATCTAAAGACAGGG	0	0.15616	0.139694	0
tsrna-14733	GCATTGGACTGTAAATCTAAAGACAG	0	0.07808	0	0
tsrna-14732	GCATTGGACTGTAAATCTAAAGACA	0	0	0	0
tsrna-14731	GCATTGGACTGTAAATCTAAAGAC	0	0	0.069847	0
tsrna-14726	GCATTGGACTGTAAATCTA	0	0	0	0
tsrna-14722	GCATTCGGCTGTTAACC	0.28566487	0.390401	0.069847	0
tsrna-14721	GCATTAACCTTTTAAGTTAAAGATTAAGAGAACC	0.07141622	0	0.139694	0
tsrna-14712	GCATGTGTGAGGTCCCGGGTT	0	0.15616	0.069847	0
tsrna-14711	GCATGTGTGAGGTCCCGGGT	0	0.23424	0.069847	0
tsrna-14709	GCATGTATGAGGTCCCGGGT	0.49991351	0.390401	0	0
tsrna-14708	GCATGTATGAGGCCCCGGGTT	0.07141622	0.780801	0.069847	0.111027
tsrna-14707	GCATGTATGAGGCCCCGGGT	0	0	0.069847	0
tsrna-14706	GCATGTACGAGGCCCCGGGT	0	0.23424	0	0.111027
tsrna-14705	GCATGGGTGGTTCAGTGGTAGAATTCTCGCCTGCCACGCGG	103.267849	42.3975	143.5352	43.30058
tsrna-14704	GCATGGGTGGTTCAGTGGTAGAATTCTCGCCTGCCACGCG	107.838487	47.94119	138.157	36.4169
tsrna-14703	GCATGGGTGGTTCAGTGGTAGAATTCTCGCCTGCCACG	104.339092	39.50854	141.3001	38.74847
tsrna-14702	GCATGGGTGGTTCAGTGGTAGAATTCTCGCCTGCCA	103.339265	34.74565	140.3921	36.19485
tsrna-14701	GCATGGGTGGTTCAGTGGTAGAATTCTCGCCTGCC	100.839698	36.07301	139.2745	34.52944
tsrna-14700	GCATGGGTGGTTCAGTGGTAGAATTCTCGCCTGC	100.696865	31.7786	134.1757	31.64273
tsrna-14699	GCATGGGTGGTTCAGTGGTAGAATTCTCGCCTG	97.1260543	29.98276	137.3887	33.5302
tsrna-14698	GCATGGGTGGTTCAGTGGTAGAATTCTCGCCT	95.9119787	29.8266	130.1246	32.41992
tsrna-14697	GCATGGGTGGTTCAGTGGTAGAATTCTCGCC	61.5607786	19.05155	75.01547	23.64878
tsrna-14696	GCATGGGTGGTTCAGTGGTAGAATTCTCGC	5.14196758	4.294406	4.609889	6.661628
tsrna-14695	GCATGGGTGGTTCAGTGGTAGAATTCTCG	1.57115676	1.561602	1.676323	3.996977
tsrna-14694	GCATGGGTGGTTCAGTGGTAGAATTCTC	0.8569946	1.249282	0.628621	3.88595
tsrna-14693	GCATGGGTGGTTCAGTGGTAGAATTCT	0.8569946	1.171202	0.628621	3.219787
tsrna-14692	GCATGGGTGGTTCAGTGGTAGAATTC	0.57132973	0.936961	0.279387	2.664651
tsrna-14691	GCATGGGTGGTTCAGTGGTAGAATT	0.64274595	0.702721	0.558774	1.998488
tsrna-14690	GCATGGGTGGTTCAGTGGTAGAAT	0.4284973	0.468481	0.698468	0.77719
tsrna-14689	GCATGGGTGGTTCAGTGGTAGAA	0.49991351	0.23424	0.20954	0.77719
tsrna-14688	GCATGGGTGGTTCAGTGGTAGA	0.35708108	0.390401	0.279387	0.444109
tsrna-14687	GCATGGGTGGTTCAGTGGTAG	0.35708108	0.31232	0.069847	0.666163
tsrna-14686	GCATGGGTGGTTCAGTGGTA	0	0.31232	0.139694	0.222054
tsrna-14685	GCATGGGTGGTTCAGTGGT	0	0.23424	0.20954	0.222054
tsrna-14684	GCATGGGTGGTTCAGTGG	0.07141622	0.31232	0	0.111027

tsrna-14683	GCATGGGTGGTTCAGTG	0	0	0.069847	0.222054
tsrna-14682	GCATGGGTGGTTCAGT	0.07141622	0.23424	0.069847	0.111027
tsrna-14681	GCATGGGTGATTCAGTGGTAGAA	0	0	0	0.111027
tsrna-14680	GCATGGGTGATTCAGTGGTAGA	0.07141622	0	0	0
tsrna-14679	GCATGGGACTCTTAATCCCAGGGTCGTGGG	0.07141622	0.07808	0	0
tsrna-14678	GCATGGGACTCTTAATCCC	0.07141622	0	0	0
tsrna-14677	GCATGGGACTCTTAATCC	0.07141622	0	0	0.111027
tsrna-14675	GCATGGGACTCTTAAT	0	0	0.069847	0
tsrna-14674	GCATGCGAGAGGTAGCGGG	0	0	0	0
tsrna-14673	GCATGCATGAGGTCCCGGGT	0.14283243	0.23424	0	0
tsrna-14672	GCATGCACGAGGTCCTGGGTT	0.35708108	1.171202	0.069847	1.110271
tsrna-14671	GCATGCACGAGGTCCTGGGT	0.21424865	1.795843	0.069847	0.333081
tsrna-14670	GCATGCACGAGGTCCTGGG	0	0.07808	0	0
tsrna-14669	GCATGCACGAGGCCCTGGGT	0.14283243	0.702721	0.069847	0
tsrna-14668	GCATGCACGAGGCCCCGGGTT	0.07141622	0.07808	0	0.222054
tsrna-14667	GCATGCACGAGGCCCCGGGT	0	0.31232	0	0
tsrna-14663	GCATGAGGTCCCGGGTTCGATCCCCAGC	0.14283243	0.468481	0	0
tsrna-14662	GCATGAGGTCCCGGGTTCG	0	0.23424	0	0.111027
tsrna-14661	GCATGAGGTCCCGGGTTC	0	0.624641	0.069847	0
tsrna-14660	GCATGAGGTCCCGGGTT	0	0.23424	0	0.111027
tsrna-14659	GCATGAGGTCCCGGGT	0	0.07808	0	0
tsrna-14658	GCATGAGACTCTTAATCTC	0.14283243	0.390401	0.069847	0
tsrna-14657	GCATGAGACTCTTAATCT	0.07141622	0.07808	0.069847	0
tsrna-14656	GCATGAGACTCTTAATC	0	0.07808	0	0.111027
tsrna-14651	GCATCAGACTTTTAATCTGAGGGTCCAG	0.21424865	2.576644	0.628621	0.222054
tsrna-14650	GCATAGGTGGTTCAGTGGTAGAATTCT	0.21424865	0.23424	0.20954	0
tsrna-14649	GCATAGGTGGTTCAGTGGTAGAATTC	0.21424865	0.23424	0.139694	0.111027
tsrna-14648	GCATAGGTGGTTCAGTGGTAGAATT	0.14283243	0.07808	0.069847	0.111027
tsrna-14647	GCATAGGTGGTTCAGTGGTAGAA	0.14283243	0.15616	0	0
tsrna-14646	GCATAGGTGGTTCAGTGGTAGA	0	0	0.069847	0
tsrna-14645	GCATAGCTGCCTTCCAAGCAGTTGACCCGG	0	0.15616	0.139694	0.222054
tsrna-14644	GCATAGCTGCCTTCCAAGCAGTTGACCCG	0.21424865	0.15616	0.139694	0.111027
tsrna-14643	GCATAGCTGCCTTCCAAGCAGTT	0	0.23424	0	0
tsrna-14642	GCATAGCTCAGTGGTAGAGCATTTGACTG	0.28566487	0.07808	0.139694	0.111027
tsrna-14641	GCATAGCTCAGTGGTAGAGCATTTGACT	0	0.23424	0.069847	0
tsrna-14640	TGACAACATTCAAAAAAGAGTACCA	0	0	0	0.111027
tsrna-14639	TGACAACATTCAAAAAAGAGTACC	0.07141622	0	0	0
tsrna-14638	TGACAACAGAGGCTTACGACCCCTTATTT	0.07141622	0	0	0
tsrna-14637	TGAATTGCAAATTCGAAGAAGCAGCTTCAAACCTGCCGGGG(	0.07141622	0.23424	0	0
tsrna-14636	TGAATCTGACAACAGAGGCTTACGACCCCTTATTTACCCCA	1.21407568	0.858881	0.419081	0
tsrna-14635	TGAATCTGACAACAGAGGCTTACGACCCCTTATTTACCCC	1.07124325	0.31232	0.628621	0.111027
tsrna-14634	TGAATCTGACAACAGAGGCTTACGACCCCTTATTTACCC	0.71416216	0.546561	0.488928	0
tsrna-14633	TGAATCTGACAACAGAGGCTTACGACCCCTTATTTACC	0.92841081	0.546561	0.349234	0.111027
tsrna-14632	TGAATCTGACAACAGAGGCTTACGACCCCTTATTTAC	0.49991351	0.31232	0.488928	0
tsrna-14631	TGAATCTGACAACAGAGGCTTACGACCCCTTATTTA	1.42832433	0.624641	0.279387	0
tsrna-14630	TGAATCTGACAACAGAGGCTTACGACCCCTTATTT	1.2854919	0.546561	0.279387	0
tsrna-14629	TGAATCTGACAACAGAGGCTTACGACCCCTTATT	0.99982703	0.23424	0.069847	0
tsrna-14628	TGAATCTGACAACAGAGGCTTACGACCCCTTAT	0.64274595	0.936961	0.20954	0
tsrna-14627	TGAATCTGACAACAGAGGCTTACGACCCCTTA	0.4284973	0.546561	0.20954	0
tsrna-14626	TGAATCTGACAACAGAGGCTTACGACCCCTT	0.14283243	0.07808	0	0
tsrna-14625	TGAATCTGACAACAGAGGCTTACGACCCCT	0.07141622	0.07808	0.069847	0
tsrna-14624	TGAATCTGACAACAGAGGCTTACGACCCC	0	0	0	0
tsrna-14623	TGAATCTGACAACAGAGGCTTACGACCC	0	0	0	0
tsrna-14622	TGAATCTGACAACAGAGGCTTACGACC	0	0.07808	0	0
tsrna-14621	TGAATCTGACAACAGAGGCTTACGAC	0	0	0	0
tsrna-14620	TGAATCTGACAACAGAGGCTTACGA	0	0	0	0
tsrna-14607	TGAATCCAGCGATCCGAGTTCAAATCTCGGTGGAACCTCCA	10.9266811	17.2557	4.260655	2.997733
tsrna-14599	TGAATCCAGCGATCCGAGT	0	0.07808	0	0
tsrna-14593	TGAAGGTCCTGAGTTCGAA	0.28566487	0.546561	0.069847	0.111027
tsrna-14592	TGAAGGTCCTGAGTTCG	0.14283243	0.858881	0.139694	0.111027
tsrna-14591	TGAAGCCAGTTGATTAG	0.07141622	0	0	0
tsrna-14590	TGAAGCATTGGACTGTAAATCTAAAGACAG	0.07141622	0	0	0
tsrna-14589	TGAAGCATTGGACTGTAAATCTAAA	0	0	0.069847	0
tsrna-14588	TGAAGCATTGGACTGTAAATCTAA	0	0.07808	0.069847	0

tsrna-14582	TGAAGCATTGGACTGTAA	0	0	0	0
tsrna-14581	TGAAGCATTGGACTGTA	0	0	0.069847	0
tsrna-14579	TGAAGATCTAAAGGTCCCTGGT	0.71416216	1.561602	0.698468	0.222054
tsrna-14578	TGAAATACAACGATGGTTTTTCATATCATT	0.07141622	0	0	0.111027
tsrna-14576	TGAAACCAGCTTTGGGGGGTTCG	0	0	0	0.111027
tsrna-14574	TGAAAATGTTTAGACGGGCTCACA	3.71364325	2.654724	0.838162	0.222054
tsrna-14573	TGAAAATGTTTAGACGGGCTCAC	1.42832433	2.108163	0.20954	0
tsrna-14572	TGAAAATGTTTAGACGGGCTCA	0.99982703	1.015041	0.139694	0.222054
tsrna-14571	TGAAAATGTTTAGACGGGCTC	0.49991351	0.31232	0.279387	0.111027
tsrna-14570	TGAAAATGTTTAGACGGGCT	0	0.31232	0.069847	0
tsrna-14569	TGAAAATGTTTAGACGGGC	0	0.07808	0	0
tsrna-14568	TGAAAACCTTTTTCCAAGGACACCA	0.14283243	0.07808	0.139694	0
tsrna-14567	TGAAAACCTTTTTCCAAGGACACC	0.07141622	0	0	0
tsrna-14566	TGAAAACCTTTTTCCAAGGACAC	0	0	0	0
tsrna-14565	TGAAAACCTTTTTCCAAGGACA	0	0	0	0
tsrna-14560	TCTTGTAAACCAGGGGTCGCG	0	0.390401	0	0
tsrna-14559	TCTTGGTGGGACCTCCA	0.07141622	0.390401	0	0
tsrna-14558	TCTTGCGACCCGGGTTCGATTCCCGGGCGGCGCACCA	1.07124325	5.153287	0.419081	0.222054
tsrna-14557	TCTTGCGACCCGGGTTCGATTCCCGGGCGGCGCACC	0.21424865	0.936961	0.279387	0.111027
tsrna-14556	TCTTGCGACCCGGGTTCGATTCCCGGGCGGCGC	0	0.468481	0	0
tsrna-14555	TCTTGCGACCCGGGTTCGATTCCCGGGCGGCG	0.35708108	0.15616	0	0
tsrna-14554	TCTTGCGACCCGGGTTCGATTCCCGGGCGGC	0	0.390401	0	0
tsrna-14553	TCTTGCGACCCGGGTTCGATTCCCGGGCGG	0.07141622	0.15616	0	0
tsrna-14552	TCTTGCGACCCGGGTTCGATTCCCG	0	0.468481	0.069847	0
tsrna-14551	TCTTGCGACCCGGGTTCGATTCCC	0.07141622	0.15616	0	0
tsrna-14550	TCTTGCGACCCGGGTTCGATTCC	0	0.07808	0	0
tsrna-14548	TCTTGCGACCCGGGTTCGATT	0.07141622	0.07808	0	0
tsrna-14547	TCTTGCGACCCGGGTTCGAT	0.07141622	0.07808	0	0
tsrna-14546	TCTTGCGACCCGGGTTCGA	0.07141622	0	0	0
tsrna-14545	TCTTGCGACCCGGGTTCG	0	0.07808	0	0
tsrna-14543	TCTTGCGACCCGGGTT	0	0.07808	0	0
tsrna-14542	TCTTCGGGGGCGTGGGTTC	0	0.31232	0	0
tsrna-14541	TCTTCGGGGGCGTGGGTT	0.07141622	0.07808	0	0
tsrna-14540	TCTTCGGGGGCGTGGGT	0	0.31232	0	0
tsrna-14538	TCTTCGGAGGCGTGGGTTCG	0	0	0	0
tsrna-14537	TCTTCGGAGGCGTGGGT	0	0.07808	0	0
tsrna-14536	TCTTCGGAGGCGTGGG	0	0.15616	0	0
tsrna-14535	TCTTAGCTTTGGGTGCTAATGGTGGAGTTAAAGACT	0.14283243	0.468481	0.069847	0.333081
tsrna-14534	TCTTAGCTTTGGGTGCTAATGGTGGAGTT	0	0.23424	0.069847	0
tsrna-14532	TCTTAATCTCAGGGTCGTGGGT	0.49991351	0.702721	0.069847	0.666163
tsrna-14531	TCTTAATCTCAGGGTCGTGGG	0.49991351	0.702721	0.20954	0.333081
tsrna-14530	TCTTAATCTCAGGGTCGTGG	0.49991351	0.23424	0.349234	0.111027
tsrna-14529	TCTTAATCTCAGGGTCGTG	0.49991351	0.702721	0	0.222054
tsrna-14528	TCTTAATCCCAGGGTCGTGGGT	0.07141622	0	0	0.111027
tsrna-14527	TCTTAATCCCAGGGTCGTGG	0.07141622	0	0	0
tsrna-14526	TCTTAATCCCAGGGTCGTG	0.07141622	0	0	0
tsrna-14525	TCTTAATCCCAGGGTCGT	0.07141622	0	0	0
tsrna-14524	TCTTAATCCCAGGGTCG	0	0	0	0.111027
tsrna-14514	TCTGTAGTGTAGTGGTTATCACG	0	0	0	0
tsrna-14509	TCTGGTCTCCGGATGGAGGCGTGGGTTCGAATCCCACTTCT(	0.35708108	4.606726	0.698468	0.111027
tsrna-14508	TCTGGTCTCCGGATGGAGGCGTGG	0.07141622	0.702721	0	0.111027
tsrna-14504	TCTGGTCTCCAATGGAGGCGTGG	0	0.07808	0	0
tsrna-14503	TCTGGGTGCCCCCTCCA	0	0.15616	0	0
tsrna-14499	TCTGGACTCTGAATCCGGTAAT	0	0	0	0.111027
tsrna-14497	TCTGGACTCTGAATCCGGT	0	0.07808	0	0
tsrna-14495	TCTGGACTCTGAATCCAGCGATCCGAGTTC	0.07141622	0.15616	0.139694	0
tsrna-14494	TCTGGACTCTGAATCCAGCGATCCGAGTT	0.07141622	0.07808	0	0
tsrna-14493	TCTGGACTCTGAATCCAGCGATCCGAGT	0	0	0.069847	0
tsrna-14492	TCTGGACTCTGAATCCAGCGATCCGAG	0	0.23424	0	0
tsrna-14491	TCTGGACTCTGAATCCAGCGATCCGA	0	0.15616	0	0
tsrna-14490	TCTGGACTCTGAATCCAGCGATCCG	0.07141622	0.07808	0	0
tsrna-14489	TCTGGACTCTGAATCCAGCGATCC	0.07141622	0.07808	0.069847	0
tsrna-14488	TCTGGACTCTGAATCCAGCGATC	0	0	0.069847	0
tsrna-14487	TCTGGACTCTGAATCCAGCGAT	0	0.15616	0	0

tsrna-14486	TCTGGACTCTGAATCCAGCGA	0.07141622	0.23424	0	0
tsrna-14485	TCTGGACTCTGAATCCAGCG	0	0.07808	0	0
tsrna-14484	TCTGGACTCTGAATCCAGC	0.07141622	0	0	0
tsrna-14483	TCTGGACTCTGAATCCAG	0	0.15616	0	0
tsrna-14480	TCTGCTTTACACGCAGAAGGTCCTGGGTTC	0.99982703	2.420483	0.628621	0.222054
tsrna-14479	TCTGATTCCGGATCAGAAGATTGAGGGTTC	0.28566487	1.249282	0.139694	0.111027
tsrna-14475	TCTGAGGGTCCAGGGTTCATGTCCCTGTTC	0	0.07808	0	0
tsrna-14473	TCTGAGGGTCCAGGGTTCATGTCCCTG	0	0.07808	0	0
tsrna-14472	TCTGAGGGTCCAGGGTTCATGTCCCT	0	0.07808	0	0
tsrna-14471	TCTGAGGGTCCAGGGTTCATGTCCC	0	0.07808	0	0
tsrna-14470	TCTGAGGGTCCAGGGTTCATG	0	0.07808	0	0
tsrna-14469	TCTGAGGGTCCAGGGTTCAAGTCCCTGTTCGGGCGCCA	0.57132973	1.093122	0.349234	0.444109
tsrna-14468	TCTGAGGGTCCAGGGTTCAAGTCCCTGTTCGGGCGCC	0.07141622	0.23424	0.069847	0
tsrna-14467	TCTGAGGGTCCAGGGTTCAAGTCCCTGTTCGGGCGC	0	0.23424	0.069847	0
tsrna-14466	TCTGAGGGTCCAGGGTTCAAGTCCCTGTTCAGGCGCCA	0	0.546561	0	0
tsrna-14465	TCTGAGGGTCCAGGGTTCAAGTCCCTGTTC	0	0	0	0
tsrna-14464	TCTGAGGGTCCAGGGTTCAAGTCCCTGTCCAGGCGCCA	0.07141622	0.15616	0	0
tsrna-14463	TCTGAGGGTCCAGGGTTCAAGT	0	0.07808	0	0
tsrna-14462	TCTGAGGGTCCAGGGTTCAAG	0	0.07808	0.069847	0
tsrna-14461	TCTGAGGGTCCAGGGTTCAA	0	0.15616	0	0
tsrna-14460	TCTGAGGGTCCAGGGTTCA	0	0.07808	0	0
tsrna-14459	TCTGAGGGTCCAGGGTTC	0.07141622	0	0	0
tsrna-14454	TCTGACTTCGGATCAGAAGATTGC	0.07141622	0	0	0
tsrna-14453	TCTGACTTCGGATCAGAAGATTGAGGGTTC	0.49991351	0.546561	0.349234	0.111027
tsrna-14452	TCTGACTTCGGATCAGAAGATTGAGGGT	0.07141622	0.624641	0.069847	0.111027
tsrna-14443	TCTGACTCCAGATCAGAAGGTTGCGTGTT	0.14283243	0.31232	0.139694	0
tsrna-14432	TCTGACAACAGAGGCTTACGACCCCTTATTTACCCCA	0.14283243	0.07808	0	0
tsrna-14431	TCTGACAACAGAGGCTTACGACCCCTTATTTACCCC	0	0	0	0
tsrna-14428	TCTGACAACAGAGGCTTACGACCCCTTAT	0	0	0	0
tsrna-14427	TCTGACAACAGAGGCTTACGACCCCTTA	0	0	0	0
tsrna-14426	TCTGACAACAGAGGCTTACGACCCCTT	0	0	0.069847	0
tsrna-14425	TCTGACAACAGAGGCTTACGACCCCT	0	0	0	0
tsrna-14416	TCTGAATCCAGCGATCCGAGTTCGAGTCTCGGTGGAACCT	0.07141622	0.15616	0.069847	0.111027
tsrna-14415	TCTGAATCCAGCGATCCGAGTTCA	0	0.15616	0	0
tsrna-14414	TCTGAATCCAGCGATCCGAGTTC	0	0	0	0.111027
tsrna-14412	TCTGAATCCAGCGATCCGAGT	0	0	0.069847	0
tsrna-14410	TCTGAATCCAGCGATCCGA	0	0.07808	0	0
tsrna-14406	TCTGAAGGTCGTGAGTTCGATCCTCACACGGGGCACCA	0.21424865	1.795843	0.139694	0
tsrna-14405	TCTGAAGGTCGTGAGTTCGATCCTCACACGGGGCACC	0.35708108	0.858881	0.279387	0
tsrna-14404	TCTGAAGGTCGTGAGTTCGATCCTCACACGGGGCAC	0.14283243	0.31232	0.139694	0
tsrna-14403	TCTGAAGGTCGTGAGTTCGATCCTCACACG	0	0.468481	0	0
tsrna-14402	TCTGAAGGTCGTGAGTTCGAGCCTCACACGGGGCACCA	0.07141622	0.624641	0	0.111027
tsrna-14401	TCTGAAGGTCGTGAGTTCGAGCCTCACACGGGGCACC	0.14283243	0.546561	0.139694	0
tsrna-14400	TCTGAAGGTCGTGAGTTCGAGCCTCACACGGGGCAC	0.14283243	0.15616	0.139694	0
tsrna-14399	TCTGAAGGTCGTGAGTTCGAGCCTCACACG	0.07141622	0.23424	0.069847	0
tsrna-14398	TCTGAAGGTCGTGAGTTCG	0.14283243	0.23424	0.20954	0.111027
tsrna-14397	TCTGAAGGTCGTGAGTTC	0	0.15616	0	0
tsrna-14396	TCTGAAGGTCGTGAGTT	0.21424865	0.390401	0.069847	0
tsrna-14395	TCTGAAGGTCGTGAGT	0.14283243	0.390401	0.20954	0
tsrna-14394	TCTGAAGGTCCTGAGTTCGAGCCTCAGAGAGGGCACCA	0.71416216	2.810884	0.698468	0.555136
tsrna-14393	TCTGAAGGTCCTGAGTTCGAACCTCAGAGGGGGCACCA	0.78557838	3.591685	0.558774	0.111027
tsrna-14392	TCTGAAGGTCCTGAGTTCGAACCTCAGAGGGGGCACC	1.14265946	3.201285	0.908008	0.111027
tsrna-14391	TCTGAAGGTCCTGAGTTCGAACCTCAGAGGGGGCAC	0.99982703	2.810884	0.279387	0.111027
tsrna-14390	TCTGAAGGTCCTGAGTTCGAACCTCAGAGG	0.57132973	2.576644	0.698468	0.333081
tsrna-14389	TCTGAAGGTCCTGAGTTCGAACC	1.2854919	1.873923	0.279387	0.555136
tsrna-14388	TCTGAAGGTCCTGAGTTCGAAC	0.57132973	2.186243	0.908008	0.111027
tsrna-14387	TCTGAAGGTCCTGAGTTCGA	1.2854919	2.654724	0.488928	0.333081
tsrna-14386	TCTGAAGGTCCTGAGTTCG	1.42832433	2.654724	0.419081	0.333081
tsrna-14385	TCTGAAGGTCCTGAGTTC	0.4284973	3.825925	0.349234	0.666163
tsrna-14384	TCTGAAGGTCCTGAGT	0.35708108	2.576644	0.558774	0.444109
tsrna-14383	TCTCTTCGGGGGCGTGGGT	0	0.23424	0.069847	0
tsrna-14379	TCTCTTCGGAGGCGTGG	0.07141622	0.07808	0	0
tsrna-14371	TCTCTGTGGAACCTCCA	0	0.07808	0	0
tsrna-14369	TCTCGGTGGGACCTCCA	1.71398919	3.825925	0.628621	0.444109

tsrna-14368	TCTCGGTGGGACCTCC	0.14283243	0.390401	0	0.111027
tsrna-14367	TCTCGGTGGGACCCCCA	0	0.23424	0	0.111027
tsrna-14366	TCTCGGTGGAACCTCCA	2.64240001	7.495691	1.396936	1.110271
tsrna-14365	TCTCGGTGGAACCTCC	0.14283243	0.31232	0.139694	0
tsrna-14360	TCTCGCTGGGGCCTCCA	1.85682163	2.108163	1.187396	1.110271
tsrna-14359	TCTCGCTGGGGCCTCC	0.21424865	0.31232	0	0
tsrna-14358	TCTCGCCTGCCACGCGGGAGGCCCGGGT	0.71416216	2.030083	0.20954	0.333081
tsrna-14357	TCTCGCCTGCCACGCGGGAGGCCCGGG	0.14283243	0.07808	0	0
tsrna-14356	TCTCGCCTGCCACGCGGGAGGCCCG	0.14283243	0.15616	0	0
tsrna-14355	TCTCGCCTGCCACGCGGGAGGCCC	0	0.23424	0	0
tsrna-14354	TCTCGCCTGCCACGCGGGAGGCC	0.07141622	0.07808	0.069847	0.111027
tsrna-14353	TCTCGCCTGCCACGCGGGAGG	0	0.07808	0.069847	0
tsrna-14352	TCTCGCCTGCCACGCGGGA	0	0.07808	0	0
tsrna-14351	TCTCGCCTGCCACGCGGG	0	0.07808	0	0
tsrna-14349	TCTCGCCTGCCACGCG	0	0.07808	0	0
tsrna-14347	TCTCCGGATGGAGGCGTGGGTTCGAA	0.35708108	2.967044	0.488928	0.111027
tsrna-14346	TCTCCGGATGGAGGCGTGGGTTCG	0.8569946	3.591685	0.20954	0
tsrna-14345	TCTCCGGATGGAGGCGTGGGT	0.35708108	2.888964	0.069847	0.111027
tsrna-14344	TCTCCGGATGGAGGCGTGGG	0.07141622	0.31232	0	0.111027
tsrna-14343	TCTCCGGATGGAGGCGTGG	0.21424865	0.15616	0.069847	0
tsrna-14342	TCTCCGGATGGAGGCGTG	0.07141622	0.23424	0	0.111027
tsrna-14341	TCTCCGGATGGAGGCGT	0.07141622	0	0	0
tsrna-14340	TCTCCCCTGGAGGCGTGGGT	0	0.15616	0.069847	0
tsrna-14339	TCTCCCCTGGAGGCGTGG	0	0.07808	0	0
tsrna-14338	TCTCCAATGGAGGCGTGGGTTCGAATCCCACTTCTG	1.2854919	1.873923	0.908008	0
tsrna-14337	TCTCCAATGGAGGCGTGGGT	0.78557838	0.858881	0.628621	0.111027
tsrna-14336	TCTCATAGTCCTAGCCA	0	0	0.069847	0
tsrna-14334	TCTCATAATCTGAAGGTCGT	0	0	0.069847	0
tsrna-14333	TCTCATAATCTGAAGGTCCTGAGT	9.569773	27.95268	7.822842	7.327791
tsrna-14332	TCTCATAATCTGAAGGTCCTGAG	0.64274595	2.108163	0.558774	0.444109
tsrna-14331	TCTCATAATCTGAAGGTCCTGA	0.14283243	1.093122	0.558774	0.111027
tsrna-14330	TCTCATAATCTGAAGGTCCT	0.21424865	0.390401	0.20954	0
tsrna-14329	TCTCAGGGTCGTGGGTTCGAGCCCCACGTTGGGCGCCA	0.35708108	19.36387	0.20954	0.555136
tsrna-14328	TCTCAGGGTCGTGGGTTCGAGCCCCACGTTGGGCGC	0	0	0.069847	0.111027
tsrna-14327	TCTCAGGGTCGTGGGTTCGAGCCCCACGTT	0	0.07808	0	0.111027
tsrna-14326	TCTCAGGGTCGTGGGTTCG	0	0.07808	0.069847	0
tsrna-14325	TCTCAGGGATGGGTTCGATTCTCATAGTCCT	0.64274595	1.171202	0.628621	1.110271
tsrna-14324	TCTCAGGGATGGGTTCGATTCTC	0	0.702721	0.419081	0
tsrna-14323	TCTCAGGGATGGGTTCGATTCT	0.35708108	0.31232	0.139694	0.111027
tsrna-14322	TCTCAGGGATGGGTTCGATTC	0.21424865	0.31232	0	0
tsrna-14321	TCTCACCGCCGCGGCCCGGGTTCGATTCCCGGTCAGGGAAC	2.28531893	24.67331	1.327089	0.333081
tsrna-14320	TCTCACCGCCGCGGCCCGGGTTCGATTCCCGGTCAGGGAAC	0.57132973	1.015041	0.349234	0.111027
tsrna-14319	TCTCACCGCCGCGGCCCGGGTTCGATTCCCGGTCA	0	0.07808	0	0
tsrna-14318	TCTCACCGCCGCGGCCCGGGTTCGATTCCC	0	0.07808	0	0
tsrna-14315	TCTCACCGCCGCGGCCCGGGT	0	0.07808	0.069847	0
tsrna-14312	TCTCACCGCCGCGGCCCG	0	0.07808	0	0
tsrna-14309	TCTAGTGGTTAGGATTCGGCGCTCTCACCGCCGCGGCCC	4.49922163	6.558729	2.724025	2.220543
tsrna-14308	TCTAGTGGTTAGGATTCGGCGCTCTCACCGCCGCGG	3.14231352	4.528646	1.466783	2.442597
tsrna-14307	TCTAGTGGTTAGGATTCGGCGCTCTCACCGCC	2.57098379	3.825925	1.74617	1.665407
tsrna-14306	TCTAGTGGTTAGGATTCGGCGCTCTCACCG	2.64240001	3.201285	1.676323	1.665407
tsrna-14305	TCTAGTGGTTAGGATTCGGCGCTCTCACC	2.71381622	3.904006	1.606477	0.999244
tsrna-14304	TCTAGTGGTTAGGATTCGGCGCTCTCAC	2.92806487	4.216326	1.117549	1.776434
tsrna-14303	TCTAGTGGTTAGGATTCGGCGCTCTCA	3.57081082	2.732804	1.955711	1.887461
tsrna-14302	TCTAGTGGTTAGGATTCGGCGCTCTC	2.28531893	4.060166	1.53663	2.442597
tsrna-14301	TCTAGTGGTTAGGATTCGGCGCTCT	2.28531893	2.967044	1.327089	1.55438
tsrna-14300	TCTAGTGGTTAGGATTCGGCGCTC	2.14248649	3.982086	1.466783	1.665407
tsrna-14299	TCTAGTGGTTAGGATTCGGCGCT	2.21390271	2.654724	1.53663	0.888217
tsrna-14298	TCTAGTGGTTAGGATTCGGCGC	0.99982703	1.327362	0.558774	0.666163
tsrna-14297	TCTAGTGGTTAGGATTCGGCG	0.57132973	0.858881	0.139694	0.666163
tsrna-14296	TCTAGTGGTTAGGATTCGGC	0.92841081	0.15616	0.419081	0.222054
tsrna-14295	TCTAGTGGTTAGGATTCGG	0.07141622	0	0.139694	0.222054
tsrna-14294	TCTAGTGGTTAGGATTCG	0.21424865	0.07808	0.139694	0
tsrna-14293	TCTAGTGGTTAGGATTC	0.21424865	0.23424	0.069847	0
tsrna-14292	TCTAGTGGCTAGGATTCGGCGCT	0.92841081	3.123204	0.488928	2.664651

tsrna-14291	TCTAGTGGCTAGGATTCGGCGC	0.92841081	3.982086	0.558774	3.552868
tsrna-14290	TCTAGTGGCTAGGATTCGG	0	0	0	0
tsrna-14289	TCTAGGTTCGACTCCTGGCTGGCTCGCC	0.21424865	0.31232	0.069847	0
tsrna-14288	TCTAGGTTCGACTCCTGGCTGGCTCGC	0	0.07808	0	0
tsrna-14287	TCTAGGTTCGACTCCTGGCTGGCTCG	0	0	0	0
tsrna-14286	TCTAGGTTCGACTCCTGGCTGGCTC	0	0	0	0
tsrna-14285	TCTAGGTTCGACTCCTGGCTGGCT	0	0.07808	0	0
tsrna-14282	TCTAGGGGTATGATTCTCGGTT	0.21424865	0.07808	0.069847	0.333081
tsrna-14281	TCTAGGGGTATGATTCTCGGT	0.21424865	0.15616	0.139694	0
tsrna-14280	TCTAGGGGTATGATTCTCGG	0.14283243	0.07808	0.069847	0
tsrna-14279	TCTAGGGGTATGATTCTCGCTT	1.2854919	1.873923	0.838162	1.221299
tsrna-14278	TCTAGGGGTATGATTCTCGCT	1.35690811	2.264323	0.488928	1.776434
tsrna-14277	TCTAGGGGTATGATTCTCGC	0.4284973	0.546561	0.419081	1.332326
tsrna-14276	TCTAGGGGTATGATTCTCG	0	0	0	0
tsrna-14274	TCTAGCGGTTAGGATTCCTGGTTTT	0.49991351	0.624641	0.139694	0.333081
tsrna-14273	TCTAGCGGTTAGGATTCCTGGTT	0.21424865	0.624641	0.069847	0.111027
tsrna-14272	TCTAGCGGTTAGGATTCCTGGT	0.71416216	0.390401	0	0
tsrna-14271	TCTAGCGGTTAGGATTCCTGG	0.14283243	0	0.279387	0
tsrna-14270	TCTAGCGGTTAGGATTCCTG	0.14283243	0.07808	0.069847	0
tsrna-14268	TCTAATTCAAAGGTTGTGGGTTCGAGTCCCACCAGAGTCGCC	0.21424865	1.795843	0.139694	0.444109
tsrna-14265	TCTAACAACATGGCTTTCTCACCA	0.14283243	0	0.069847	0
tsrna-14264	TCTAACAACATGGCTTTCTCACC	0	0.07808	0	0
tsrna-14263	TCTAACAACATGGCTTTCTCAC	0.07141622	0	0	0
tsrna-14262	TCTAACAACATGGCTTTCTCA	0.07141622	0	0	0
tsrna-14259	TCTAAAGGTCCCTGGTTCGATCCCGGGTTTCGGCACCA	3.85647569	10.22849	1.53663	2.553624
tsrna-14258	TCTAAAGGTCCCTGGTTCGATCCCGGGTTTCGGCACC	1.64257298	2.108163	0.20954	0.666163
tsrna-14257	TCTAAAGGTCCCTGGTTCGATCCCGGGTTTCGGCAC	0.64274595	1.249282	0.558774	0.666163
tsrna-14256	TCTAAAGGTCCCTGGTTCGATCCCGGGTTT	0.71416216	1.327362	1.047702	0.222054
tsrna-14255	TCTAAAGGTCCCTGGTTCGATC	0.92841081	1.795843	0.628621	0.666163
tsrna-14254	TCTAAAGGTCCCTGGTTCGAT	1.2854919	1.093122	0.838162	0.111027
tsrna-14253	TCTAAAGGTCCCTGGTTCGA	1.2854919	1.171202	0.349234	0.555136
tsrna-14252	TCTAAAGGTCCCTGGTTCG	1.2854919	1.093122	0.628621	0.111027
tsrna-14251	TCTAAAGGTCCCTGGTT	0.78557838	1.717762	0.768315	0.444109
tsrna-14250	TCTAAAGGTCCCTGGT	0.4284973	1.171202	0.279387	0.111027
tsrna-14249	TCTAAAGACAGGGGTTAGGCCTCTTTTTAC	0.07141622	0.15616	0	0
tsrna-14248	TCTAAAGACAGGGGTTAGGCCTCT	0	0.07808	0.139694	0
tsrna-14247	TCTAAAGACAGGGGTTAGGCCTC	0.07141622	0.07808	0.069847	0
tsrna-14246	TCTAAAGACAGGGGTTAGGCCT	0.07141622	0	0.069847	0
tsrna-14244	TCGTTGGTCTAGTGGTATGATTCTCGC	0.64274595	0.390401	0.349234	0.111027
tsrna-14242	TCGTTGGTCTAGTGGTATGAT	0	0	0.069847	0
tsrna-14240	TCGTTGGTCTAGGGGTATGATTCTCGGTT	0.64274595	0.31232	0.20954	0.111027
tsrna-14239	TCGTTGGTCTAGGGGTATGATTCTCGGT	0.21424865	0.15616	0	0.333081
tsrna-14238	TCGTTGGTCTAGGGGTATGATTCTCGG	0	0.15616	0.20954	0
tsrna-14237	TCGTTGGTCTAGGGGTATGATTCTCGCTTC	1.85682163	2.108163	1.047702	1.665407
tsrna-14236	TCGTTGGTCTAGGGGTATGATTCTCGCTT	1.64257298	1.717762	1.327089	2.553624
tsrna-14235	TCGTTGGTCTAGGGGTATGATTCTCGCT	1.78540541	1.717762	0.908008	1.776434
tsrna-14234	TCGTTGGTCTAGGGGTATGATTCTCGC	0.49991351	0.468481	0.908008	2.33157
tsrna-14233	TCGTTGGTCTAGGGGTATGATTCTCG	0.07141622	0.07808	0	0
tsrna-14232	TCGTTGGTCTAGGGGTATGATTCTC	0.07141622	0	0.069847	0
tsrna-14231	TCGTTGGTCTAGGGGTATGATTCT	0	0	0	0
tsrna-14230	TCGTTGGTCTAGGGGTATGATTC	0	0	0	0
tsrna-14222	TCGTTAGTATAGTGGTTAGTATCCCCGCC	0.07141622	0.07808	0	0
tsrna-14221	TCGTTAGTATAGTGGTTAGTATCCCCG	0	0.07808	0	0
tsrna-14220	TCGTTAGTATAGTGGTTAGTATCCCC	0	0.07808	0	0
tsrna-14217	TCGTTAGTATAGTGGTTAGTATC	0	0.07808	0	0
tsrna-14216	TCGTTAGTATAGTGGTTAGT	0	0.07808	0	0
tsrna-14215	TCGTTAGTATAGTGGTGAGTATCCCCGCCT	0.07141622	0.390401	0.139694	0
tsrna-14214	TCGTTAGTATAGTGGTGAGTATCCCCGCC	0.14283243	0.468481	0	0.222054
tsrna-14213	TCGTTAGTATAGTGGTGAGTATCCCCGC	0.07141622	0.23424	0	0
tsrna-14212	TCGTTAGTATAGTGGTGAGTATCCCCG	0.07141622	0.23424	0	0
tsrna-14211	TCGTTAGTATAGTGGTGAGTATCCCC	0	0.15616	0	0
tsrna-14210	TCGTTAGTATAGTGGTGAGTATCCC	0	0.07808	0.069847	0
tsrna-14208	TCGTTAGTATAGTGGTGAGTATC	0	0	0.069847	0
tsrna-14206	TCGTTAGTATAGTGGTGAGTA	0	0.07808	0	0

tsrna-14205	TCGTTAGTATAGTGGTGAGT	0	0.07808	0	0
tsrna-14198	TCGTGGTTGTAGTCCGTGCGAGAATACCA	0.71416216	1.795843	0.698468	0.77719
tsrna-14197	TCGTGGTTGTAGTCCGTGCGAGAATACC	0.14283243	0.702721	0.419081	0
tsrna-14196	TCGTGGTTGTAGTCCGTGCGAGAATAC	0.14283243	0.07808	0.069847	0
tsrna-14195	TCGTGGTTGTAGTCCGTGCGAGAATA	0.21424865	0.07808	0.139694	0
tsrna-14194	TCGTGGTTGTAGTCCGTGCGAGAAT	0	0.23424	0.139694	0.111027
tsrna-14193	TCGTGGTTGTAGTCCGTGCGAGAA	0.07141622	0.07808	0	0
tsrna-14192	TCGTGGTTGTAGTCCGTGCGAGA	0	0	0	0
tsrna-14191	TCGTGGTTGTAGTCCGTGCGAG	0	0.15616	0	0
tsrna-14190	TCGTGGTTGTAGTCCGTGCGA	0	0	0	0
tsrna-14189	TCGTGGTTGTAGTCCGTGCG	0	0	0.069847	0.111027
tsrna-14187	TCGTGGTTGTAGTCCGTG	0	0	0.069847	0
tsrna-14184	TCGTGGGTTCGAGCCCCACGTTGGGCGCCA	0.78557838	18.50499	0.698468	0.555136
tsrna-14183	TCGTGGGTTCGAGCCCCACGTTGGGCGCC	0	0.936961	0.139694	0
tsrna-14182	TCGTGGGTTCGAGCCCCACGTTGGGCGC	0	0.15616	0	0
tsrna-14175	TCGTGAGTTCGATCCTCACACGGGGCACC	0	0.07808	0	0
tsrna-14173	TCGTATAGTGGTTAGTACTCTGCGTTGTGGCCGCAGCAACCT	3.14231352	1.717762	2.235098	1.332326
tsrna-14172	TCGTATAGTGGTTAGTACTCTGCGTTGTGG	2.42815136	1.171202	1.117549	0.999244
tsrna-14171	TCGTATAGTGGTTAGTACTCTGCGTTGTG	1.64257298	1.171202	1.187396	0.555136
tsrna-14170	TCGTATAGTGGTTAGTACTCTGCGTTGT	1.64257298	1.639682	0.838162	0.999244
tsrna-14169	TCGTATAGTGGTTAGTACTCTGCGTTG	2.28531893	1.015041	0.838162	0.444109
tsrna-14168	TCGTATAGTGGTTAGTACTCTGCGTT	1.92823784	0.858881	1.117549	0.999244
tsrna-14167	TCGTATAGTGGTTAGTACTCTGCGT	0.57132973	0.23424	0.279387	0.111027
tsrna-14166	TCGTATAGTGGTTAGTACTCTGCGCTG	0.49991351	0.23424	0.279387	0.333081
tsrna-14165	TCGTATAGTGGTTAGTACTCTGCGC	0.49991351	0.23424	0.279387	0.222054
tsrna-14164	TCGTATAGTGGTTAGTACTCTGCG	0.14283243	0.31232	0.20954	0.111027
tsrna-14163	TCGTATAGTGGTTAGTACTCTGC	0.14283243	0.07808	0	0
tsrna-14162	TCGTATAGTGGTTAGTACTCTG	0.07141622	0.07808	0.069847	0.222054
tsrna-14161	TCGTATAGTGGTTAGTACTCT	0.14283243	0.07808	0.139694	0
tsrna-14160	TCGTATAGTGGTTAGTACTC	0.14283243	0	0	0.222054
tsrna-14159	TCGTATAGTGGTTAGTACT	0.07141622	0	0	0
tsrna-14155	TCGTAAACCGAAGATCGCGGGTTCGAACCCCGTCCGTGCCT(	4.71347028	13.82018	1.885864	1.332326
tsrna-14154	TCGTAAACCGAAGATCGCGGGTTCGAACCCCGTCCGTGCCT(	2.78523244	15.61602	1.74617	0.999244
tsrna-14153	TCGTAAACCGAAGATCGCGGGTTCGAACCC	3.28514596	13.11746	2.304945	0.888217
tsrna-14152	TCGTAAACCGAAGATCGCGGGTT	2.99948109	12.72706	1.885864	1.110271
tsrna-14151	TCGTAAACCGAAGATCGCGGGT	3.14231352	12.41474	1.816017	0.444109
tsrna-14150	TCGTAAACCGAAGATCGCGGG	0.07141622	0.23424	0.069847	0
tsrna-14148	TCGTAAACCGAAGATCACGGGTTCGAACCCCGTCCGTGCCT(	1.71398919	7.495691	0.977855	0.555136
tsrna-14147	TCGTAAACCGAAGATCACGGGTTCGAACCC	0.64274595	6.558729	0.069847	0.222054
tsrna-14146	TCGTAAACCGAAGATCACGGGT	0.57132973	5.699848	0.279387	0.444109
tsrna-14145	TCGTAAACAGGAGATCCTGGGT	1.21407568	12.33666	0.977855	0.77719
tsrna-14144	TCGTAAACAGGAGATCC	0.35708108	0.390401	0	0.222054
tsrna-14141	TCGGTTAGCGCGTTCGGCTGTT	0	0	0.069847	0
tsrna-14139	TCGGTAGAGCTATCAGACTTTTA	0	0.07808	0	0.111027
tsrna-14136	TCGGTAGAGCTATCAGAC	0	0	0	0.222054
tsrna-14135	TCGGTAGAGCTATCAGA	0	0	0	0.111027
tsrna-14134	TCGGTAGAGCATGGGACTCTTAATCTC	0.71416216	2.030083	0.698468	0.111027
tsrna-14133	TCGGTAGAGCATGGGACTCTTAATCCCAGGGTCGTGGGTTC	1.71398919	2.264323	1.047702	0.444109
tsrna-14132	TCGGTAGAGCATGGGACTCTTAATCCCAGGGTCGTGGGT	1.14265946	1.795843	0.838162	0.444109
tsrna-14131	TCGGTAGAGCATGGGACTCTTAATCCCAGGGTCGTGGG	1.57115676	1.405442	1.047702	0.666163
tsrna-14130	TCGGTAGAGCATGGGACTCTTAATCCCAGGGTCGTGG	0.8569946	1.795843	0.558774	0.555136
tsrna-14129	TCGGTAGAGCATGGGACTCTTAATCCC	0.92841081	1.952003	0.628621	0.444109
tsrna-14128	TCGGTAGAGCATGGGACTCTTAATCC	1.07124325	1.249282	0.279387	0.555136
tsrna-14127	TCGGTAGAGCATGGGACTCTTAATC	0.4284973	1.249282	0.20954	0.333081
tsrna-14126	TCGGTAGAGCATGGGACTCTTAAT	0.35708108	0.390401	0.20954	0
tsrna-14125	TCGGTAGAGCATGGGACTCT	0.49991351	0.702721	0.139694	0.222054
tsrna-14124	TCGGTAGAGCATGGGACTC	0.64274595	0.936961	0.279387	0.222054
tsrna-14123	TCGGTAGAGCATGGGACT	0.28566487	0.702721	0.20954	0
tsrna-14122	TCGGTAGAGCATGGGAC	0.49991351	0.858881	0.419081	0.111027
tsrna-14121	TCGGTAGAGCATGGGA	0.35708108	0.546561	0.069847	0.111027
tsrna-14120	TCGGTAGAGCATGAGACTCTTAATCTCAGGGTCGTGGGT	1.35690811	2.967044	0.628621	1.443353
tsrna-14119	TCGGTAGAGCATGAGACTCTTAATCTC	0.57132973	1.717762	0.139694	0.444109
tsrna-14118	TCGGTAGAGCATGAGACTCTTAATCT	0.78557838	2.108163	0.20954	0.444109
tsrna-14117	TCGGTAGAGCATGAGACTCTTAATC	0.49991351	1.483522	0.279387	0.666163

tsrna-14116	TCGGTAGAGCATGAGACTCTTA	0.92841081	1.795843	0.558774	0.444109
tsrna-14115	TCGGTAGAGCATGAGACTCTT	0.64274595	1.795843	0.488928	0.444109
tsrna-14114	TCGGTAGAGCATGAGACTC	0.4284973	1.249282	0.279387	0.333081
tsrna-14113	TCGGTAGAGCATGAGACT	0.49991351	1.015041	0.20954	0.444109
tsrna-14112	TCGGTAGAGCATGAGACCC	0.35708108	1.327362	0.139694	0.333081
tsrna-14111	TCGGTAGAGCATGAGAC	0.4284973	0.936961	0.139694	0.222054
tsrna-14110	TCGGTAGAGCATGAGA	0.14283243	0.31232	0.069847	0
tsrna-14109	TCGGTAGAGCATCAGACTTTTAATCTG	0.21424865	0.390401	0.139694	0.111027
tsrna-14108	TCGGTAGAGCATCAGACTTTTAATCT	0	0.468481	0	0.111027
tsrna-14107	TCGGTAGAGCATCAGACTTTTAATC	0.35708108	0.15616	0.069847	0.111027
tsrna-14106	TCGGTAGAGCATCAGACTTTTAAT	0.21424865	0.15616	0	0
tsrna-14105	TCGGTAGAGCATCAGACTTTTA	0.14283243	0.31232	0.069847	0
tsrna-14104	TCGGTAGAGCATCAGACTTTT	0.07141622	0.15616	0.069847	0.222054
tsrna-14103	TCGGTAGAGCATCAGACTTT	0	0.15616	0.069847	0
tsrna-14102	TCGGTAGAGCATCAGACTT	0.14283243	0.31232	0	0.111027
tsrna-14101	TCGGTAGAGCATCAGACT	0.07141622	0.07808	0	0
tsrna-14100	TCGGTAGAGCATCAGAC	0	0.390401	0.069847	0
tsrna-14099	TCGGTAGAGCATCAGA	0.07141622	0.31232	0	0
tsrna-14098	TCGGTAGAGCATAAGAC	0.78557838	1.327362	0.419081	0.222054
tsrna-14097	TCGGGTGTGAGAGGTCCCGGGTT	0	0.546561	0	0
tsrna-14096	TCGGGTGTGAGAGGTCCCGGGT	0	0	0	0
tsrna-14095	TCGGGTGCGAGAGGTCCCGGGTT	0.21424865	0.15616	0	0.111027
tsrna-14094	TCGGGTGCGAGAGGTCCCGGGT	0	0.23424	0	0
tsrna-14092	TCGGGTGCGAGAGGTCCCGG	0	0.07808	0	0
tsrna-14085	TCGGCTGTTAACCGAAAGGTTGGTGGT	1.92823784	9.057293	1.466783	4.330058
tsrna-14084	TCGGCTGTTAACCGAAAGGTTGGTGG	0.28566487	1.639682	0.488928	0.666163
tsrna-14083	TCGGCTGTTAACCGAAAGGTTGGTG	0.14283243	2.576644	0.349234	1.110271
tsrna-14082	TCGGCTGTTAACCGAAAGGTTGG	0.35708108	0.780801	0	0.444109
tsrna-14081	TCGGCTGTTAACCGAAAGGTTG	0.28566487	1.093122	0.419081	0.333081
tsrna-14080	TCGGCGCTTTCACCGCCGCGGCCC	0	0.23424	0.069847	0.555136
tsrna-14079	TCGGCGCTCTCACCGCCGCGGCCCGGGTTC	0	0	0	0
tsrna-14077	TCGGCGCTCTCACCGCCGCGGCCCGGGT	0.07141622	0	0	0
tsrna-14074	TCGGCGCTCTCACCGCCGCGG	0	0.07808	0	0
tsrna-14073	TCGGCGCTCTCACCGCCG	0	0.07808	0	0
tsrna-14071	TCGGCGCTCTCACCGC	0	0	0	0.111027
tsrna-14070	TCGGATCAGAAGATTGCAGGTTCGAGTCCTGCCGCGGTCGC(	0.14283243	0.390401	0.069847	0
tsrna-14068	TCGGATCAGAAGATTGCAGGTT	0	0	0	0
tsrna-14064	TCGGATCAGAAGATTGAGGGTTCGAGTCCCTTCGTGGTCGC(	0.49991351	0.858881	0.279387	0.555136
tsrna-14063	TCGGATCAGAAGATTGAGGGTTCGAATCCCTTCGTGGTTGCC	0.35708108	1.249282	0.349234	0.222054
tsrna-14062	TCGGATCAGAAGATTGAGGGTTC	0.35708108	1.171202	0.069847	0.222054
tsrna-14061	TCGGATCAGAAGATTGAGGGT	0.28566487	0.468481	0.139694	0
tsrna-14055	TCGCTTCGGGTGTGAGAGGTCCC	0	0.07808	0	0
tsrna-14049	TCGCTGGTTCGATTCCGGCTCGAAGGACCA	5.49904866	51.22055	1.955711	3.441841
tsrna-14048	TCGCTGGTTCGATTCCGGCTCGAAGGACC	0.14283243	1.405442	0.20954	0.333081
tsrna-14047	TCGCTGGTTCGATTCCGGCTCGAAGGAC	0	0	0	0.111027
tsrna-14045	TCGCTGGTTCGATTCCGGCTCGAAGG	0	0.07808	0	0
tsrna-14043	TCGCTGGTTCGATTCCGGC	0	0.07808	0	0
tsrna-14041	TCGCTGGTTCGATTCCAGCTCGAAGGACC	0	0.07808	0	0
tsrna-14039	TCGCTGGTTCGAATCCGGCTCGAAGGACC	0	0.546561	0.139694	0
tsrna-14037	TCGCTGGTTCGAATCCGGCTCGAAGGA	0	0.15616	0	0
tsrna-14033	TCGCGGGTTCGATCCCCGTACTGGCCACC	0	0.07808	0.069847	0
tsrna-14032	TCGCGGGTTCGATCCCCGTACTGGCCAC	0	0	0	0
tsrna-14031	TCGCGGGTTCGATCCCCGTACTGGCCA	0	0.07808	0.069847	0
tsrna-14030	TCGCGGGTTCGATCCCCGTACGGGCCACCA	0.57132973	9.681934	0.349234	0.555136
tsrna-14029	TCGCGGGTTCGATCCCCGTACGGGCCACC	0.14283243	0.702721	0.069847	0.111027
tsrna-14023	TCGCGGGTTCGAACCCCGTACGGGCCACC	0	0	0	0
tsrna-14022	TCGCGAGTTCAAATCTCGCTGGGGCCTCC	0.07141622	0.31232	0.139694	0.222054
tsrna-14021	TCGCGAGTTCAAATCTCGCTGGGGCCTC	0.07141622	0	0	0
tsrna-14020	TCGCCTGCCACGCGGGAGGCCCGGGTT	0.14283243	2.264323	0.349234	0.333081
tsrna-14019	TCGCCTGCCACGCGGGAGGCCCGGGT	0.21424865	1.717762	0.279387	0.111027
tsrna-14018	TCGCCTGCCACGCGGGAGGCCCGGG	0.14283243	0	0	0
tsrna-14017	TCGCCTGCCACGCGGGAGGCCCGG	0	0.07808	0	0
tsrna-14013	TCGCATGTGTGAGGTCCCGGGT	0	0.31232	0.069847	0
tsrna-14011	TCGCATGTATGAGGTCCCGGGTT	0.07141622	0.390401	0	0.111027

tsrna-14010	TCGCATGTATGAGGTCCCGGGT	0	0.468481	0	0
tsrna-14009	TCGCATGTATGAGGTCCC	0	0	0.069847	0
tsrna-14008	TCGCATGTACGAGGCCCCGGGT	0	0.07808	0	0
tsrna-14007	TCGCATAAAACTTAAAACTT	0.07141622	0	0	0
tsrna-14006	TCGATTCTCATAGTCCTAGCCA	0	0.15616	0	0.111027
tsrna-14005	TCGATTCTCATAGTCCTAGCC	0	0	0	0
tsrna-14004	TCGATTCTCATAGTCCTAGC	0.07141622	0	0	0
tsrna-14003	TCGATTCTCATAGTCCTAG	0	0	0	0
tsrna-14002	TCGATTCTCATAGTCCTA	0	0.15616	0	0.111027
tsrna-14001	TCGATTCCTTCCTTTTTTGCCA	0	0.15616	0	0.111027
tsrna-13998	TCGATTCCTTCCTTTTTTG	0	0.07808	0	0
tsrna-13997	TCGATTCCGGCTCGAAGGACCA	4.57063785	12.41474	2.235098	1.998488
tsrna-13996	TCGATTCCGGCTCGAAGGACC	0.28566487	0.702721	0.069847	0
tsrna-13994	TCGATTCCGGCTCGAAGGA	0	0.07808	0	0
tsrna-13991	TCGATTCCCTGACGGGGAGCCA	0	0.15616	0	0
tsrna-13990	TCGATTCCCGGTCAGGGAACCA	0.71416216	22.09667	0.349234	0.444109
tsrna-13989	TCGATTCCCGGTCAGGGAACC	0.07141622	0.858881	0	0
tsrna-13988	TCGATTCCCGGTCAGGGAAC	0	0.390401	0	0
tsrna-13987	TCGATTCCCGGTCAGGGAA	0	0.15616	0	0
tsrna-13985	TCGATTCCCGGGCGGCGCACCA	1.57115676	5.856008	0.558774	0.333081
tsrna-13984	TCGATTCCCGGGCGGCGCACC	0.49991351	0.546561	0.069847	0
tsrna-13983	TCGATTCCCGGGCGGCGCAC	0	0.07808	0	0
tsrna-13979	TCGATTCCCGGCCCATGCACCA	2.28531893	2.888964	0.698468	1.443353
tsrna-13978	TCGATTCCCGGCCCATGCACC	0.14283243	0.15616	0	0.111027
tsrna-13975	TCGATTCCCGGCCAATGCACCA	2.21390271	10.93122	0.698468	1.110271
tsrna-13974	TCGATTCCCGGCCAATGCACC	0.07141622	0.624641	0	0
tsrna-13973	TCGATTCCCGGCCAATGCAC	0	0.07808	0	0
tsrna-13972	TCGATTCCCGGCCAATGCA	0	0.15616	0	0
tsrna-13971	TCGATTCCCGGCCAACGCACCA	3.14231352	8.120331	1.53663	1.776434
tsrna-13970	TCGATTCCCGGCCAACGCACC	1.07124325	0.858881	0.069847	0.111027
tsrna-13968	TCGATTCCCGGCCAACGCA	0	0	0	0
tsrna-13963	TCGATTCCCCGACGGGGAGCCA	0	1.561602	0.069847	0.111027
tsrna-13962	TCGATTCCCCGACGGGGAGCC	0	0	0	0
tsrna-13959	TCGATTCCCCGACGGGGA	0	0.07808	0	0
tsrna-13956	TCGATTCCCAGCCAATGCACCA	0.14283243	0.468481	0	0
tsrna-13952	TCGATGGATCGAAACCATCCTCTGCTACC	0	0.23424	0	0.111027
tsrna-13951	TCGATGGATCGAAACCATCCTCTGCTAC	0	0	0.069847	0
tsrna-13945	TCGATGCCCGCATTCTCCACCA	0.14283243	0.390401	0	0
tsrna-13944	TCGATGCCCGCATTCTCCACC	0	0.15616	0	0
tsrna-13942	TCGATGCCCGCATCCTCCACCA	0.14283243	0.31232	0.069847	0
tsrna-13941	TCGATGCCCGCATCCTCCACC	0	0.07808	0	0
tsrna-13936	TCGATCCTCGCTGGGGCCTCCA	0.71416216	0.702721	0.069847	0.333081
tsrna-13935	TCGATCCTCGCTGGGGCCTCC	0.07141622	0	0	0.111027
tsrna-13934	TCGATCCTCACCTGGAGCACCA	0	0.23424	0	0.111027
tsrna-13932	TCGATCCTCACACGGGGCACCA	0	0.23424	0	0
tsrna-13930	TCGATCCCGGGTTTCGGCACCA	2.85664866	8.120331	0.977855	0.888217
tsrna-13929	TCGATCCCGGGTTTCGGCACC	0.35708108	0.31232	0.20954	0.111027
tsrna-13924	TCGATCCCGGGCGGAAACACCA	1.2854919	2.888964	0.558774	0.77719
tsrna-13923	TCGATCCCGGGCGGAAACACC	0.28566487	0.468481	0.069847	0
tsrna-13921	TCGATCCCCGTACTGGCCACCA	0.28566487	0.15616	0.349234	0
tsrna-13919	TCGATCCCCGTACGGGCCACCA	0.8569946	5.699848	0.419081	0.77719
tsrna-13918	TCGATCCCCGTACGGGCCACC	0.14283243	0.07808	0	0
tsrna-13917	TCGATCCCCGGCATCTCCACCA	16.9970595	30.37316	9.429319	8.993198
tsrna-13916	TCGATCCCCGGCATCTCCACC	0.4284973	1.873923	0.139694	0.333081
tsrna-13915	TCGATCCCCGGCATCTCCAC	0	0.07808	0.069847	0
tsrna-13911	TCGATCCCCGGCACCTCCACCA	13.2834163	22.33091	6.635447	4.66314
tsrna-13910	TCGATCCCCGGCACCTCCACC	0.64274595	1.561602	0.419081	0.111027
tsrna-13909	TCGATCCCCGACACCTCCACCA	1.42832433	1.873923	0.20954	0.333081
tsrna-13908	TCGATCCCCAGTACCTCCACCA	2.92806487	4.919047	1.327089	1.221299
tsrna-13907	TCGATCCCCAGTACCTCCACC	0.14283243	0.390401	0.20954	0.222054
tsrna-13904	TCGATCCCCAGCATCTCCACCA	0.78557838	1.171202	0.488928	0.222054
tsrna-13902	TCGATCCCCAGCATCTCCAC	0	0	0	0
tsrna-13900	TCGATCCCACCCAGGGACGCCA	0.14283243	0.31232	0.069847	0
tsrna-13899	TCGATAGCTCAGTTGGTAGAGCGGAGGACT	0.07141622	0	0	0.333081

tsrna-13898	TCGATAGCTCAGTTGGTAGAGC	0.07141622	0	0.069847	0
tsrna-13897	TCGATAGCTCAGTTGGTAGAG	0	0	0.069847	0.222054
tsrna-13896	TCGATAGCTCAGTTGGTAGA	0.07141622	0.07808	0	0.111027
tsrna-13895	TCGATAGCTCAGTTGGTAG	0	0	0.139694	0
tsrna-13894	TCGATAGCTCAGTTGG	0	0	0.139694	0
tsrna-13893	TCGATAGCTCAGCTGGTAGAGCGGAGGACT	0	0	0	0
tsrna-13892	TCGATAGCTCAGCTGGTAGAGCGG	0.07141622	0	0	0
tsrna-13891	TCGATAGCTCAGCTGGTAGAGC	0.07141622	0.07808	0	0
tsrna-13890	TCGATAGCTCAGCTGGTAGA	0	0	0.069847	0.222054
tsrna-13889	TCGATAGCTCAGCTGGTAG	0.07141622	0	0.069847	0
tsrna-13888	TCGAGTCTCGGTGGAACCTCCA	4.3563892	9.291533	1.327089	2.664651
tsrna-13887	TCGAGTCTCGGTGGAACCTCC	0.49991351	0.23424	0	0.111027
tsrna-13886	TCGAGTCTCGGTGGAACCTC	0	0.07808	0	0
tsrna-13885	TCGAGTCCTGCCGCGGTCGCCA	0.21424865	0.468481	0.069847	0.111027
tsrna-13884	TCGAGTCCTGCCGCGGTCGCC	0	0	0.069847	0
tsrna-13881	TCGAGTCCCTTCGTGGTCGCCA	0.07141622	0	0	0
tsrna-13880	TCGAGTCCCTTCGTGGTCGCC	0	0.07808	0	0
tsrna-13879	TCGAGTCCCGGCGGAGTCGCCA	0	0.23424	0	0
tsrna-13873	TCGAGTCCCATCTGGGTCGCCA	0.21424865	0.624641	0	0
tsrna-13872	TCGAGTCCCATCTGGGTCGCC	0.07141622	0	0	0
tsrna-13870	TCGAGTCCCATCTGGGGTGCCA	0	0.31232	0	0
tsrna-13868	TCGAGTCCCATCTGGGGTGC	0	0.07808	0	0
tsrna-13867	TCGAGTCCCACCTGGGGTGCCA	0.07141622	0.390401	0.069847	0
tsrna-13866	TCGAGTCCCACCTGGGGTACCA	0	0.468481	0	0
tsrna-13864	TCGAGTCCCACCCGGGGTACCA	0	0.07808	0	0
tsrna-13863	TCGAGTCCCACCAGAGTCGCCA	0	0.15616	0	0
tsrna-13862	TCGAGTCCCACCAGAGTCGCC	0	0	0	0
tsrna-13860	TCGAGCCTCAGAGAGGGCACCA	0	0.07808	0	0
tsrna-13859	TCGAGCCTCACCTGGAGCACCA	0	0.31232	0.139694	0
tsrna-13858	TCGAGCCTCACCTGGAGCACC	0	0.07808	0	0
tsrna-13854	TCGAGCCCCAGTGGAACCACCA	2.71381622	2.186243	1.955711	1.887461
tsrna-13853	TCGAGCCCCAGTGGAACCACC	0.14283243	0.468481	0.069847	0.222054
tsrna-13852	TCGAGCCCCAGTGGAACCAC	0	0	0	0
tsrna-13850	TCGAGCCCCAGTGGAACC	0	0.15616	0	0
tsrna-13847	TCGAGCCCCACGTTGGGCGCCA	0.64274595	15.06946	0.558774	0.444109
tsrna-13846	TCGAGCCCACCCAGGGACGCCA	0.35708108	8.120331	0	0
tsrna-13845	TCGAGCCCACCCAGGGACGCC	0	0.468481	0	0
tsrna-13842	TCGACTCCTGGCTGGCTCGCCA	2.14248649	9.213453	1.396936	0.333081
tsrna-13841	TCGACTCCTGGCTGGCTCGCC	0	0.23424	0	0
tsrna-13840	TCGACTCCTGGCTGGCTCGC	0	0.15616	0	0
tsrna-13836	TCGACTCCCGGTGTGGGAACCA	3.85647569	20.30083	1.816017	2.109516
tsrna-13835	TCGACTCCCGGTGTGGGAACC	0.07141622	0.858881	0.069847	0
tsrna-13832	TCGACTCCCGGTGTGGGA	0	0.07808	0	0
tsrna-13829	TCGACTCCCGGTATGGGAACCA	0.64274595	6.246409	0.069847	0.666163
tsrna-13827	TCGACTCCCAGCGGGGCCTCCA	0.07141622	0	0	0
tsrna-13826	TCGACTCATTAAATTATGATAATCATATTT	0	0	0.20954	0
tsrna-13825	TCGACTCATTAAATTATGATAATCAT	0.07141622	0	0	0
tsrna-13824	TCGACCCCCGGCTCCTCCACCA	1.2854919	1.795843	0.838162	0.222054
tsrna-13822	TCGAATCTCGGTGGGACCTCCA	4.21355677	10.38465	1.676323	2.775678
tsrna-13821	TCGAATCCTGTTCGTGACGCCA	1.07124325	0.468481	0.20954	0.111027
tsrna-13820	TCGAATCCTGTTCGTGACGCC	0.14283243	0.23424	0.069847	0
tsrna-13819	TCGAATCCTGTCGGCTACGCCA	0.28566487	1.327362	0.419081	0.888217
tsrna-13818	TCGAATCCTGCTCACAGCGCCA	0.57132973	2.030083	0.20954	0.222054
tsrna-13817	TCGAATCCTGCTCACAGCGCC	0.07141622	0.23424	0	0
tsrna-13814	TCGAATCCTGCCGACTACGCCA	7.35587029	19.05155	1.885864	1.665407
tsrna-13813	TCGAATCCTGCCGACTACGCC	0.64274595	1.015041	0.069847	0
tsrna-13812	TCGAATCCTGCCGACTACGC	0	0.15616	0	0
tsrna-13811	TCGAATCCGGCTCGGAGGACCA	0.14283243	0	0.069847	0
tsrna-13808	TCGAATCCGGCTCGAAGGACCA	1.49974054	4.450566	0.628621	0.444109
tsrna-13807	TCGAATCCGGCTCGAAGGACC	0.07141622	0.390401	0	0.111027
tsrna-13802	TCGAATCCGAGTCACGGCACCA	0.07141622	0.23424	0	0
tsrna-13801	TCGAATCCGAGTCACGGCACC	0	0	0	0
tsrna-13797	TCGAATCCCTTCGTGGTTGCCA	0	0.546561	0	0
tsrna-13795	TCGAATCCCTCCGTGGTTACCA	0.07141622	0.31232	0.139694	0

tsrna-13794	TCGAATCCCATCCTCGTCGCCA	0.57132973	1.015041	0.139694	0.111027
tsrna-13793	TCGAATCCCATCCTCGTCGCC	0.07141622	0.07808	0	0
tsrna-13789	TCGAATCCCAGTAGAGCCTCCA	0.78557838	1.405442	0.698468	1.110271
tsrna-13788	TCGAATCCCAGTAGAGCCTCC	0.21424865	0.546561	0	0
tsrna-13787	TCGAATCCCAGCGGTGCCTCCA	1.71398919	3.825925	0.838162	0.444109
tsrna-13786	TCGAATCCCAGCGGTGCCTCC	0	0.07808	0	0
tsrna-13782	TCGAATCCCAGCGGGGCCTCCA	0	0.23424	0	0.111027
tsrna-13781	TCGAATCCCAGCGGGGCCTCC	0	0.15616	0	0
tsrna-13779	TCGAATCCCAGCGAGGCCTCCA	0	0	0	0
tsrna-13778	TCGAATCCCACTTTTGACACCA	0	0	0	0.111027
tsrna-13777	TCGAATCCCACTTCTGACACCA	0.64274595	1.639682	0.558774	0.999244
tsrna-13776	TCGAATCCCACTTCTGACACC	0.07141622	0.15616	0.069847	0
tsrna-13775	TCGAATCCCACTTCTGACAC	0	0	0	0
tsrna-13772	TCGAATCCCACTCCTGACACCA	8.14144867	8.588812	4.470196	2.775678
tsrna-13771	TCGAATCCCACTCCTGACACC	0.21424865	0.624641	0.069847	0.444109
tsrna-13770	TCGAATCCCACTCCTGACAC	0	0	0.069847	0
tsrna-13769	TCGAATCCCACTCCTGACA	0.07141622	0	0	0
tsrna-13767	TCGAATCCCACCTTCGTCGCCA	0	0.31232	0	0
tsrna-13766	TCGAATCCCACCTTCGTCGCC	0.14283243	0.07808	0	0
tsrna-13764	TCGAATCCCACCGCTGCCACCA	1.07124325	2.810884	0.279387	0.222054
tsrna-13763	TCGAATCCCACCGCTGCCACC	0.07141622	0.15616	0	0
tsrna-13759	TCGAATCCCACCCTCGTCGCCA	0.21424865	1.795843	0.20954	0.555136
tsrna-13758	TCGAATCCCACCCTCGTCGCC	0	0.07808	0	0.111027
tsrna-13757	TCGAATCCCACCAGAGTCGCCA	0.28566487	0.31232	0.069847	0
tsrna-13756	TCGAATCCCACCAGAGTCGCC	0.14283243	0	0	0
tsrna-13754	TCGAATCCCACCACTGCCACCA	0.07141622	1.639682	0	0.222054
tsrna-13753	TCGAATCCCACCACTGCCACC	0.07141622	0.07808	0	0
tsrna-13752	TCGAATCACGTCGGGGTCACCA	0.28566487	0.702721	0.349234	0.222054
tsrna-13751	TCGAATCACGTCGGGGTCACC	0	0.07808	0.069847	0
tsrna-13745	TCGAAGAAGCAGCTTCAAACCTGCCGGGGCTT	0	0	0	0
tsrna-13744	TCGAAGAAGCAGCTTCAAACCTGCCGGGGCT	0.14283243	0	0	0
tsrna-13742	TCGAACCTCAGAGGGGGCACCA	0	0.07808	0	0
tsrna-13738	TCGAACCCTGCTCGCTGCGCCA	0.57132973	2.888964	0.279387	0.111027
tsrna-13737	TCGAACCCTGCTCGCTGCGCC	0.07141622	0.23424	0	0
tsrna-13734	TCGAACCCCGTCCGTGCCTCCA	0.57132973	0.624641	0.139694	0.111027
tsrna-13733	TCGAACCCCGTCCGTGCCTCC	0	0.07808	0.069847	0
tsrna-13729	TCGAACCCCACTTCTGGTACCA	0.14283243	0.858881	0	0
tsrna-13727	TCGAACCCCACTCCTGGTACC	0	0.07808	0	0
tsrna-13724	TCGAAATCCAATGGGGTTTCCCC	0.64274595	30.13892	0.139694	0.222054
tsrna-13723	TCGAAACCGGGCGGAAACACCA	21.2820325	55.90536	8.730851	4.330058
tsrna-13722	TCGAAACCGGGCGGAAACACC	4.85630272	14.28866	1.676323	0.333081
tsrna-13721	TCGAAACCGGGCGGAAACAC	0	0.07808	0.069847	0
tsrna-13720	TCGAAACCGGGCGGAAACA	0	0.15616	0	0
tsrna-13716	TCGAAACCGGGCAGAAGCACCA	2.35673514	9.916174	1.117549	0.555136
tsrna-13715	TCGAAACCGGGCAGAAGCACC	0.07141622	0.702721	0.069847	0
tsrna-13710	TCGAAACCATCCTCTGCTACCA	0.8569946	1.093122	0.908008	1.221299
tsrna-13709	TCGAAACCATCCTCTGCTACC	0.35708108	0.468481	0.139694	0.111027
tsrna-13707	TCGAAACCATCCTCTGCTA	0	0	0	0
tsrna-13706	TCGAAACCATCCTCTGCT	0	0.07808	0	0
tsrna-13702	TCCTTGTAGTATAAACTAATACACCAG	0.07141622	0	0	0
tsrna-13701	TCCTTGGTGGTCTAGTGGCTAGGATTCGG	0.21424865	0.546561	0	0.555136
tsrna-13700	TCCTTGGTGGTCTAGTGGC	0	0.07808	0.139694	0
tsrna-13699	TCCTTGGTGGTCTAGTGG	0	0	0	0
tsrna-13696	TCCTTAGGTCGCTGGTTCGATTCCGGCTCG	0.07141622	0	0.279387	0
tsrna-13695	TCCTTAGGTCGCTGGTTCGATT	0.07141622	0.07808	0	0
tsrna-13693	TCCTTAGGTCGCTGGTTCGAATCCGGCTCG	0	0.07808	0	0
tsrna-13691	TCCTTAGGTCGCTGGTTCG	0	0	0	0
tsrna-13690	TCCTTAGGTCGCTGGTTC	0	0.07808	0	0
tsrna-13689	TCCTTAGGTCGCTGGTT	0	0	0.069847	0
tsrna-13688	TCCTTAGGTCGCTGGT	0	0	0.069847	0
tsrna-13687	TCCTGTTCGTGACGCCA	0.07141622	0.23424	0.069847	0
tsrna-13685	TCCTGTCGGCTACGCCA	0.07141622	0	0	0
tsrna-13684	TCCTGGTTTTCACCCAGGTGGCCCG	0.35708108	0.07808	0.069847	0
tsrna-13683	TCCTGGGTTCGATCCCCAGTACCTCCACC	0.14283243	0.31232	0.069847	0

tsrna-13682	TCCTGGGTTCGAGCCCCAGTGGAACCACC	0.07141622	0.23424	0.069847	0.111027
tsrna-13681	TCCTGGGTTCGAGCCCCAGTGGAACCAC	0	0	0	0
tsrna-13680	TCCTGGGTTCGAGCCCCAGTGGAACCA	0.14283243	0.15616	0	0.111027
tsrna-13679	TCCTGGGTTCGAATCCCAGCGGTGCCTCC	0.14283243	0	0	0
tsrna-13678	TCCTGGGTTCAATCCCCAATACCTCCACC	0	0	0	0
tsrna-13677	TCCTGGCTGGCTCGCCA	0.28566487	3.045124	0	0
tsrna-13675	TCCTGCTCACAGCGCCA	0.14283243	0.390401	0.069847	0
tsrna-13673	TCCTGCCGCGGTCGCCA	0	0.07808	0	0
tsrna-13671	TCCTGCCGACTACGCCA	1.2854919	1.717762	0.069847	0
tsrna-13670	TCCTGCCGACTACGCC	0.07141622	0.23424	0	0
tsrna-13665	TCCTGAGTTCGAACCTCAGAGGGGGCACC	0	0.07808	0	0
tsrna-13659	TCCTCTTCTTAACACCA	0	0.07808	0.069847	0
tsrna-13657	TCCTCGTTAGTATGGTGG	0.07141622	0	0	0
tsrna-13656	TCCTCGTTAGTATAGTGGTTAGTATCCCCGCCTGTC	0.57132973	0.23424	0.279387	0.333081
tsrna-13655	TCCTCGTTAGTATAGTGGTTAGTATCCCCGCCTGT	0.57132973	0.23424	0.139694	0.333081
tsrna-13654	TCCTCGTTAGTATAGTGGTTAGTATCCCCGCCTG	0.14283243	0.15616	0.279387	0.555136
tsrna-13653	TCCTCGTTAGTATAGTGGTTAGTATCCCCGCCT	0.07141622	0.23424	0.139694	0.111027
tsrna-13652	TCCTCGTTAGTATAGTGGTTAGTATCCCCGCC	0.07141622	0	0.139694	0.333081
tsrna-13651	TCCTCGTTAGTATAGTGGTTAGTATCCCCG	0	0.31232	0.20954	0.111027
tsrna-13650	TCCTCGTTAGTATAGTGGTTAGTATCCCC	0.07141622	0.07808	0	0
tsrna-13649	TCCTCGTTAGTATAGTGGTTAGTATCCC	0.14283243	0	0	0.222054
tsrna-13648	TCCTCGTTAGTATAGTGGTTAGTATCC	0	0	0.069847	0.222054
tsrna-13647	TCCTCGTTAGTATAGTGGTTAGTATC	0	0.23424	0.069847	0.333081
tsrna-13646	TCCTCGTTAGTATAGTGGTTAGT	0.14283243	0	0.20954	0.111027
tsrna-13645	TCCTCGTTAGTATAGTGGTTAG	0	0	0	0.222054
tsrna-13644	TCCTCGTTAGTATAGTGGTT	0.07141622	0	0.069847	0
tsrna-13643	TCCTCGTTAGTATAGTGGTGAGTATCCCCGCCTGTC	2.35673514	2.186243	1.53663	1.776434
tsrna-13642	TCCTCGTTAGTATAGTGGTGAGTATCCCCGCCTGT	1.14265946	1.639682	1.327089	1.55438
tsrna-13641	TCCTCGTTAGTATAGTGGTGAGTATCCCCGCCTG	0.99982703	1.327362	0.768315	0.888217
tsrna-13640	TCCTCGTTAGTATAGTGGTGAGTATCCCCGCCT	1.2854919	1.249282	0.628621	1.443353
tsrna-13639	TCCTCGTTAGTATAGTGGTGAGTATCCCCGCC	1.42832433	1.015041	0.279387	1.332326
tsrna-13638	TCCTCGTTAGTATAGTGGTGAGTATCCCCGC	0.64274595	0.858881	0.279387	0.888217
tsrna-13637	TCCTCGTTAGTATAGTGGTGAGTATCCCCG	0.35708108	1.405442	0.139694	0.444109
tsrna-13636	TCCTCGTTAGTATAGTGGTGAGTATCCCC	0.49991351	0.780801	0.139694	0.444109
tsrna-13635	TCCTCGTTAGTATAGTGGTGAGTATCCC	0.35708108	0.624641	0.069847	0.444109
tsrna-13634	TCCTCGTTAGTATAGTGGTGAGTATCC	0.21424865	0.936961	0.139694	0.555136
tsrna-13633	TCCTCGTTAGTATAGTGGTGAGTATC	0.14283243	0.31232	0	0.666163
tsrna-13632	TCCTCGTTAGTATAGTGGTGAGTAT	0.49991351	0.546561	0.139694	0.333081
tsrna-13631	TCCTCGTTAGTATAGTGGTGAGTA	0.4284973	0.546561	0.069847	0.222054
tsrna-13630	TCCTCGTTAGTATAGTGGTGAGT	0.49991351	0.936961	0.20954	0.555136
tsrna-13629	TCCTCGTTAGTATAGTGGTGAG	0.14283243	0.07808	0.139694	0.444109
tsrna-13628	TCCTCGTTAGTATAGTGGTGA	0.07141622	0	0.069847	0.222054
tsrna-13627	TCCTCGTTAGTATAGTGGTG	0	0	0.069847	0
tsrna-13626	TCCTCGTTAGTATAGTGGT	0	0	0.069847	0
tsrna-13625	TCCTCGTTAGTATAGTGG	0.07141622	0	0	0
tsrna-13624	TCCTCGTTAGTATAGTG	0	0	0	0.222054
tsrna-13623	TCCTCGTTAGTATAGT	0.07141622	0	0	0
tsrna-13622	TCCTCGCTGGGGCCTCCA	0.57132973	0.468481	0.279387	0.333081
tsrna-13621	TCCTCACCTGGAGCACCA	0.07141622	0.07808	0	0
tsrna-13619	TCCTCACACGGGGCACCA	0	0.15616	0	0
tsrna-13612	TCCTAAGCCAGGGATTGTGGGTTCGAGTCCCATCTGGGGTG(	12.2835892	37.94693	7.194221	4.885194
tsrna-13611	TCCTAAGCCAGGGATTGTGGGTTCGAGTCCCATCTGGGGTG(	10.8552649	37.94693	7.752995	5.662384
tsrna-13610	TCCTAAGCCAGGGATTGTGGGTTCGAGTCCCACCCGGGGTA(	10.2125189	37.24421	6.635447	6.883683
tsrna-13609	TCCTAAGCCAGGGATTGTGGGTTCGAGTCC	10.7838487	35.44837	7.543455	5.662384
tsrna-13608	TCCTAAGCCAGGGATTGTGGGTTCG	13.4976649	35.99493	7.962536	6.21752
tsrna-13607	TCCTAAGCCAGGGATTGTGGGTTC	12.4264217	37.55653	7.543455	5.662384
tsrna-13606	TCCTAAGCCAGGGATTGTGGGTT	10.7838487	36.22917	6.914834	4.330058
tsrna-13605	TCCTAAGCCAGGGATTGTGGGT	10.4267676	33.96485	5.657591	4.774167
tsrna-13604	TCCTAAGCCAGGGATTGTGGG	0.07141622	0.468481	0.279387	0.222054
tsrna-13603	TCCTAAGCCAGGGATTGTGG	0.07141622	0.07808	0.069847	0
tsrna-13602	TCCTAAGCCAGGGATTGTG	0	0.07808	0	0.111027
tsrna-13599	TCCGTGGTGGTCTAGTGGCTAGGATTCGGC	0.64274595	1.717762	0.419081	1.332326
tsrna-13598	TCCGTGGTGGTCTAGTGGCTAGGATTCGG	0.07141622	0.31232	0	0.444109
tsrna-13597	TCCGTGGTGGTCTAGTGGC	0	0	0.069847	0

tsrna-13593	TCCGTGCGAGAATACCA	0.07141622	0	0	0
tsrna-13591	TCCGTAGTGTAGTGGTTATCACGTTCGCCTCAC	0.14283243	0.07808	0.069847	0.111027
tsrna-13590	TCCGTAGTGTAGTGGTTATCACGTTCGCCTC	0.14283243	0	0.069847	0
tsrna-13589	TCCGTAGTGTAGTGGTTATCACGTTCGCCT	0.14283243	0.07808	0.139694	0
tsrna-13588	TCCGTAGTGTAGTGGTTATCACGTTCGCC	0.14283243	0	0	0.111027
tsrna-13587	TCCGTAGTGTAGTGGTTATCACGTTCGC	0.07141622	0.23424	0.069847	0.111027
tsrna-13586	TCCGTAGTGTAGTGGTTATCACGTTCG	0.07141622	0	0.069847	0.111027
tsrna-13585	TCCGTAGTGTAGTGGTTATCACGTTC	0.21424865	0	0	0
tsrna-13584	TCCGTAGTGTAGTGGTTATCACGTT	0.21424865	0.07808	0.069847	0.111027
tsrna-13583	TCCGTAGTGTAGTGGTTATCACG	0.28566487	0	0	0.111027
tsrna-13582	TCCGTAGTGTAGTGGTTATCA	0	0	0.069847	0.111027
tsrna-13581	TCCGTAGTGTAGTGGTTATC	0.14283243	0.07808	0.069847	0
tsrna-13578	TCCGTAGTGTAGTGGTCATCACGTTCGCCT	0.07141622	0	0	0.111027
tsrna-13577	TCCGTAGTGTAGTGGTCATCACGTTCGCC	0.07141622	0.07808	0	0
tsrna-13576	TCCGTAGTGTAGTGGTCATCACGTTC	0	0	0.069847	0
tsrna-13575	TCCGTAGTGTAGTGGTCATCACG	0.21424865	0	0.20954	0
tsrna-13574	TCCGTAGTGTAGTGGTCATC	0.07141622	0	0	0
tsrna-13571	TCCGTAGTGTAGCGGTTATCACATT	0.07141622	0	0	0
tsrna-13570	TCCGGGTTCGAGTCCCGGCGGAGTCGCCA	0	0.31232	0	0
tsrna-13569	TCCGGGTTCGAGTCCCGGCGGAGTCGCC	0	0.07808	0	0
tsrna-13566	TCCGGGTGCCCCCTCCA	7.21303786	9.838094	3.422493	1.332326
tsrna-13565	TCCGGGTGCCCCCTCC	0	0.07808	0	0
tsrna-13564	TCCGGGTGCCCCCCCCA	0.71416216	1.015041	0.279387	0
tsrna-13563	TCCGGGGTTCAAGTCCCTGTTCGGGCGCC	0	0	0	0
tsrna-13560	TCCGGCTCGAAGGACCA	0.07141622	0.23424	0	0.111027
tsrna-13559	TCCGGCTCGAAGGACC	0.07141622	0.23424	0	0
tsrna-13558	TCCGGATGGAGGCGTGGGTTCGAATCCCACTTCTG	0.07141622	2.342403	0.279387	0.222054
tsrna-13557	TCCGGATGGAGGCGTGGGTTCGAATC	0.4284973	2.654724	0.279387	0.222054
tsrna-13556	TCCGGATGGAGGCGTGGGTTCGAA	0.4284973	3.279365	0.139694	0.111027
tsrna-13555	TCCGGATGGAGGCGTGGGTTCG	0.4284973	2.732804	0.279387	0
tsrna-13554	TCCGGATGGAGGCGTGGGTT	0.64274595	2.888964	0.20954	0.222054
tsrna-13553	TCCGGATGGAGGCGTGGGT	0.21424865	2.654724	0.20954	0.111027
tsrna-13552	TCCGGATGGAGGCGTGGG	0.07141622	0.780801	0.069847	0
tsrna-13551	TCCGGATGGAGGCGTGG	0.07141622	0.23424	0	0
tsrna-13550	TCCGGATGGAGGCGTG	0.14283243	0.546561	0.069847	0.111027
tsrna-13549	TCCGGATCAGAAGATTGAGGGT	0.21424865	0.624641	0.139694	0
tsrna-13547	TCCGGAGCTGGGGATTGTGGGT	0	0.15616	0	0
tsrna-13545	TCCGGAGCTGGGGATTGTGG	0	0	0	0
tsrna-13542	TCCGCGTGGGTTCGAACCCCACTCCTGG	0	0.07808	0	0
tsrna-13534	TCCCTGTTCGGGCGCCA	0.14283243	0.468481	0.419081	0.444109
tsrna-13531	TCCCTGTGGTCTAGTGGTTAGGA	14.4260757	26.39108	16.97277	32.3089
tsrna-13530	TCCCTGTGGTCTAGTGGTTAGG	13.1405838	23.97059	18.02048	18.31948
tsrna-13529	TCCCTGTGGTCTAGTGGTTAG	9.9982703	22.95555	16.20446	18.09742
tsrna-13528	TCCCTGTGGTCTAGTGGTTA	10.9266811	22.09667	18.85864	16.43202
tsrna-13527	TCCCTGTGGTCTAGTGGTT	4.7848865	13.82018	4.959123	3.219787
tsrna-13526	TCCCTGTGGTCTAGTGGT	3.57081082	15.45986	3.841574	4.108004
tsrna-13525	TCCCTGTGGTCTAGTGG	3.42797839	14.67906	4.470196	4.885194
tsrna-13524	TCCCTGTGGTCTAGTG	4.07072434	16.0845	4.330502	5.551357
tsrna-13523	TCCCTGTCCAGGCGCCA	0	0	0	0
tsrna-13521	TCCCTGGTTCGATCCCGGGTTTCGGCACC	0.4284973	0.468481	0.069847	0.111027
tsrna-13519	TCCCTGGTTCGATCCCGGGTTTCGGCA	0	0.07808	0	0
tsrna-13518	TCCCTGGTTCGATCCCGGGTTTCGGC	0	0	0	0
tsrna-13516	TCCCTGGTTCGATCCCGGGTT	0	0.07808	0	0
tsrna-13512	TCCCTGGTTCAATCCCGGGTTTCGGCACC	0.57132973	0.390401	0.20954	0
tsrna-13511	TCCCTGGTTCAAATCCGGGTGCCCCCTCC	0.28566487	0.390401	0.139694	0
tsrna-13510	TCCCTGGTGGTCTAGTGGTTAGGATTCGGCGCTCTCACCGC(	59.4897083	25.5322	86.26081	19.6518
tsrna-13509	TCCCTGGTGGTCTAGTGGTTAGGATTCGGCGCTCTCACCGC(	59.6325407	27.71844	83.1177	16.43202
tsrna-13508	TCCCTGGTGGTCTAGTGGTTAGGATTCGGCGCTCTCACCGC(	59.1326272	27.40612	84.86387	15.98791
tsrna-13507	TCCCTGGTGGTCTAGTGGTTAGGATTCGGCGCTCTCACCGC(	58.418465	26.78148	77.94904	18.87461
tsrna-13506	TCCCTGGTGGTCTAGTGGTTAGGATTCGGCGCTCTCACCGC	51.5625083	22.79939	65.93538	15.21072
tsrna-13505	TCCCTGGTGGTCTAGTGGTTAGGATTCGGCGCTCTCACCG	53.5621623	24.12675	66.14492	13.76737
tsrna-13504	TCCCTGGTGGTCTAGTGGTTAGGATTCGGCGCTCTCACC	48.1345299	25.45412	58.0427	14.65558
tsrna-13503	TCCCTGGTGGTCTAGTGGTTAGGATTCGGCGCTCTCAC	43.9923893	22.09667	48.12445	11.76888
tsrna-13502	TCCCTGGTGGTCTAGTGGTTAGGATTCGGCGCTCTCA	45.9920434	21.78435	48.12445	9.548334

tsrna-13501	TCCCTGGTGGTCTAGTGGTTAGGATTCGGCGCTCTC	46.9918704	23.18979	48.89276	12.87915
tsrna-13500	TCCCTGGTGGTCTAGTGGTTAGGATTCGGCGCTCT	45.206465	20.84739	45.54012	12.21299
tsrna-13499	TCCCTGGTGGTCTAGTGGTTAGGATTCGGCGCTC	11.4980108	9.057293	7.752995	5.995465
tsrna-13498	TCCCTGGTGGTCTAGTGGTTAGGATTCGGCGCT	4.28497299	6.402569	2.933566	3.552868
tsrna-13497	TCCCTGGTGGTCTAGTGGTTAGGATTCGGCGC	4.07072434	3.435525	2.235098	3.552868
tsrna-13496	TCCCTGGTGGTCTAGTGGTTAGGATTCGGCG	2.49956757	4.216326	2.095404	3.552868
tsrna-13495	TCCCTGGTGGTCTAGTGGTTAGGATTCGGC	2.49956757	3.357445	2.444638	2.997733
tsrna-13494	TCCCTGGTGGTCTAGTGGTTAGGATTCGG	1.57115676	2.810884	1.606477	2.109516
tsrna-13493	TCCCTGGTGGTCTAGTGGTTAGGATTCG	1.49974054	2.888964	2.235098	2.775678
tsrna-13492	TCCCTGGTGGTCTAGTGGTTAGGATTC	1.57115676	3.201285	1.53663	2.553624
tsrna-13491	TCCCTGGTGGTCTAGTGGTTAGGATT	1.64257298	2.888964	1.466783	1.998488
tsrna-13490	TCCCTGGTGGTCTAGTGGTTAGGAT	1.92823784	2.264323	1.676323	1.665407
tsrna-13489	TCCCTGGTGGTCTAGTGGTTAGGA	1.35690811	2.576644	1.606477	1.55438
tsrna-13488	TCCCTGGTGGTCTAGTGGTTAGG	1.64257298	3.201285	1.74617	1.998488
tsrna-13487	TCCCTGGTGGTCTAGTGGTTAG	1.71398919	2.810884	1.885864	1.665407
tsrna-13486	TCCCTGGTGGTCTAGTGGTTA	1.64257298	2.108163	2.095404	1.110271
tsrna-13485	TCCCTGGTGGTCTAGTGGTT	0.14283243	1.093122	0.139694	0.666163
tsrna-13484	TCCCTGGTGGTCTAGTGGT	0.57132973	1.405442	0.20954	0.333081
tsrna-13483	TCCCTGGTGGTCTAGTGGCTAGGATTCGGCGCTTTCACC	5.14196758	7.26145	7.822842	10.10347
tsrna-13482	TCCCTGGTGGTCTAGTGGCTAGGATTCGGCGCTTTC	5.49904866	6.012168	5.657591	6.21752
tsrna-13481	TCCCTGGTGGTCTAGTGGCTAGGATTCGGCGCTTT	5.42763245	5.856008	5.098817	4.441086
tsrna-13480	TCCCTGGTGGTCTAGTGGCTAGGATTCGGCGCTT	2.78523244	5.153287	3.2828	4.774167
tsrna-13479	TCCCTGGTGGTCTAGTGGCTAGGATTCGGCGCT	1.78540541	3.825925	0.908008	4.996221
tsrna-13478	TCCCTGGTGGTCTAGTGGCTAGGATTCGGCGC	0.99982703	4.294406	0.698468	4.441086
tsrna-13477	TCCCTGGTGGTCTAGTGGCTAGGATTCGGCG	1.14265946	3.982086	0.698468	3.88595
tsrna-13476	TCCCTGGTGGTCTAGTGGCTAGGATTCGGC	1.14265946	2.342403	0.419081	1.998488
tsrna-13475	TCCCTGGTGGTCTAGTGGCTAGGATTCGG	0.49991351	1.795843	0.279387	0.444109
tsrna-13474	TCCCTGGTGGTCTAGTGGCTAGGATTCG	0.4284973	0.780801	0.069847	1.665407
tsrna-13473	TCCCTGGTGGTCTAGTGGCTAGGATTC	0.28566487	1.015041	0.20954	0.666163
tsrna-13472	TCCCTGGTGGTCTAGTGGCTAGGATT	0.4284973	2.030083	0.349234	1.332326
tsrna-13471	TCCCTGGTGGTCTAGTGGCTAGGAT	0.35708108	1.483522	0.139694	0.555136
tsrna-13470	TCCCTGGTGGTCTAGTGGCTAGGA	0.35708108	1.327362	0.488928	0.888217
tsrna-13469	TCCCTGGTGGTCTAGTGGCTAGG	0.21424865	1.795843	0.139694	0.222054
tsrna-13468	TCCCTGGTGGTCTAGTGGCTAG	0.28566487	1.327362	0.139694	0.444109
tsrna-13467	TCCCTGGTGGTCTAGTGGCTA	0.21424865	1.171202	0.139694	0.444109
tsrna-13466	TCCCTGGTGGTCTAGTGGCT	0.07141622	1.795843	0.20954	0.111027
tsrna-13465	TCCCTGGTGGTCTAGTGGC	0.07141622	1.561602	0	0.111027
tsrna-13464	TCCCTGGTGGTCTAGTGG	0.14283243	1.327362	0.20954	0.222054
tsrna-13463	TCCCTGGTGGTCTAGTG	0.28566487	1.639682	0.488928	0.333081
tsrna-13462	TCCCTGGTGGTCTAGT	0.28566487	0.936961	0.139694	0.222054
tsrna-13461	TCCCTGGTGGTCTAATGGTTAGG	0.57132973	2.264323	0.488928	0.222054
tsrna-13460	TCCCTGGTGGTCTAATGGTTAG	0.14283243	2.498564	0.419081	0.555136
tsrna-13459	TCCCTGGTCTAGTGGTTAGGATT	0.28566487	0.546561	0.349234	0.555136
tsrna-13458	TCCCTGGTCTAGTGGTTAGGAT	0	0.07808	0	0.111027
tsrna-13457	TCCCTGGTCTAGTGGTTAGGA	0	0.15616	0	0
tsrna-13456	TCCCTGGTCTAGTGGTTAGG	0	0	0	0
tsrna-13455	TCCCTGGTCTAGTGGTTAG	0	0	0.139694	0
tsrna-13454	TCCCTGGTCTAGTGGTTA	0	0	0	0
tsrna-13453	TCCCTGGTAGTCTAGTGGC	0	0.07808	0	0
tsrna-13452	TCCCTGGTAGTCTAGTGG	0	0	0	0
tsrna-13451	TCCCTGGTAGTCTAGTG	0	0.23424	0	0
tsrna-13450	TCCCTGGTAGTCTAGT	0	0	0	0
tsrna-13449	TCCCTGGCACCTCCACCA	0	0	0	0
tsrna-13448	TCCCTCCGTGGTTACCA	0	0.07808	0	0
tsrna-13447	TCCCGGTGTGGGAACCA	0	0.15616	0	0
tsrna-13445	TCCCGGTCAGGGAACCA	0.07141622	0.07808	0	0
tsrna-13442	TCCCGGGTTTCGGCACCA	2.71381622	8.510732	0.908008	0.999244
tsrna-13441	TCCCGGGTTTCGGCACC	0.14283243	0.468481	0.139694	0
tsrna-13440	TCCCGGGTTTCGGCAC	0	0.07808	0	0
tsrna-13439	TCCCGGGTTCGATCCCCGGCATCTCCACC	0.4284973	1.171202	0.279387	0.333081
tsrna-13438	TCCCGGGTTCGATCCCCGGCACCTCCACC	0.57132973	1.717762	0.349234	0.222054
tsrna-13436	TCCCGGGTTCGATCCCCAGCATCTCCACC	0.07141622	0.23424	0	0.222054
tsrna-13432	TCCCGGGTTCAATCCCCGGCATCTCCACC	0.14283243	0.468481	0.069847	0.111027
tsrna-13431	TCCCGGGTTCAATCCCCGGCACCTCCACC	0.71416216	1.561602	0.20954	0.111027

tsrna-13430	TCCCGGGTTCAAATCCCGGACGAGCCCCC	0.4284973	0.858881	0.069847	0
tsrna-13429	TCCCGGGTTCAAATCCCGGACGAG	0	0	0	0
tsrna-13428	TCCCGGGCGGCGCACCA	0.21424865	0.15616	0	0.111027
tsrna-13427	TCCCGGGCGGCGCACC	0	0.15616	0.069847	0
tsrna-13426	TCCCGGGCGGAAACACCA	1.14265946	1.952003	0.558774	0.444109
tsrna-13425	TCCCGGGCGGAAACACC	0.28566487	0.546561	0	0.111027
tsrna-13424	TCCCGGCGGAGTCGCCA	0	0	0	0
tsrna-13423	TCCCGGCGGAGTCGCC	0	0	0	0
tsrna-13422	TCCCGGCCCATGCACCA	0.07141622	0.15616	0.139694	0.111027
tsrna-13420	TCCCGGCCAATGCACCA	0.07141622	0	0	0.222054
tsrna-13419	TCCCGGCCAATGCACC	0.07141622	0.07808	0.069847	0
tsrna-13416	TCCCGGACGAGCCCCCA	1.71398919	3.357445	0.768315	1.776434
tsrna-13415	TCCCGGACGAGCCCCC	0.07141622	0.07808	0	0
tsrna-13412	TCCCCGTACTGGCCACCA	0.07141622	0.624641	0.139694	0
tsrna-13411	TCCCCGTACTGGCCACC	0.07141622	0	0	0
tsrna-13410	TCCCCGTACGGGCCACCA	0.71416216	3.435525	0.279387	0.333081
tsrna-13409	TCCCCGTACGGGCCACC	0	0.15616	0	0
tsrna-13408	TCCCCGGTTCGATCCCGGGCGGAAACACC	0.21424865	0.390401	0.139694	0
tsrna-13407	TCCCCGGTTCGATCCCGGGCGGA	0	0	0.069847	0
tsrna-13405	TCCCCGGTTCGAAACCGGGCGGAAACACC	4.28497299	15.14754	2.095404	0.222054
tsrna-13401	TCCCCGGTTCAAATCCGGGTGCCCCCTCC	0.35708108	0.15616	0.069847	0.222054
tsrna-13400	TCCCCGGCATCTCCACCA	7.14162164	9.760014	3.841574	3.552868
tsrna-13399	TCCCCGGCATCTCCACC	0.21424865	0.15616	0.20954	0
tsrna-13398	TCCCCGGCATCTCCAC	0	0.07808	0	0
tsrna-13397	TCCCCGGCACCTCCACCA	9.85543787	18.58307	5.448051	5.218275
tsrna-13396	TCCCCGGCACCTCCACC	0.64274595	0.702721	0.279387	0
tsrna-13395	TCCCCGGCACCTCCAC	0	0	0.069847	0
tsrna-13394	TCCCCGCGCAGGTTCGAATCCTGCCGACT	0	0.15616	0	0.111027
tsrna-13393	TCCCCGCGCAGGTTCGAATCCTG	0	0.23424	0	0
tsrna-13392	TCCCCGCGCAGGTTCGAATCCT	0.07141622	0.15616	0	0
tsrna-13389	TCCCCGCCTGTCACGCGGGAGACCGGGGTTCGATTC	1.07124325	4.919047	0.419081	0.888217
tsrna-13388	TCCCCGCCTGTCACGCGGGAGACCGGGGTT	0.78557838	4.372486	0.349234	0.222054
tsrna-13387	TCCCCGCCTGTCACGCGGGAGACCGG	0.92841081	3.435525	0.488928	0.222054
tsrna-13386	TCCCCGCCTGTCACGCGGGAGACCG	0.71416216	3.591685	0.768315	0.555136
tsrna-13382	TCCCCGACACCTCCACCA	0.49991351	0.07808	0.139694	0.111027
tsrna-13381	TCCCCGACACCTCCACC	0.07141622	0	0	0
tsrna-13378	TCCCCAGTACCTCCACCA	0.07141622	0.23424	0.069847	0.111027
tsrna-13377	TCCCCAGTACCTCCACC	0	0.07808	0	0
tsrna-13375	TCCCCAGCATCTCCACCA	0.07141622	0.23424	0.069847	0
tsrna-13372	TCCCCAGCACTTCCACCA	0	0	0	0
tsrna-13371	TCCCCAGCACCTCCACCA	0	0.07808	0	0
tsrna-13362	TCCCATGGTGTAATGGTTAGCACTCTGGACT	0.07141622	0.07808	0.069847	0
tsrna-13361	TCCCATGGTGTAATGGTTAGCACTCTGGAC	0	0.15616	0	0.333081
tsrna-13360	TCCCATGGTGTAATGGTTAGCACTCTGG	0	0	0	0.111027
tsrna-13359	TCCCATGGTGTAATGGTTAGCACTCTG	0.07141622	0	0	0
tsrna-13358	TCCCATCTGGGTCGCCA	0.14283243	0.15616	0.069847	0
tsrna-13357	TCCCATCTGGGGTGCCA	0	0.07808	0	0
tsrna-13356	TCCCATCCTCGTCGCCA	0.28566487	0	0.279387	0
tsrna-13355	TCCCATCCTCGTCGCC	0.07141622	0.07808	0	0
tsrna-13354	TCCCATATGGTCTAGCGGTTAGGATTCCTGGTTTTCACCCA	3.14231352	1.483522	6.076672	0.999244
tsrna-13353	TCCCATATGGTCTAGCGGTTAGGATTCCTGGTTTTCACCC	2.99948109	0.858881	5.448051	0.999244
tsrna-13352	TCCCATATGGTCTAGCGGTTAGGATTCCTGGTTTTCACC	2.42815136	1.327362	4.540042	1.110271
tsrna-13351	TCCCATATGGTCTAGCGGTTAGGATTCCTGGTTTTCA	2.64240001	0.468481	4.540042	0.555136
tsrna-13350	TCCCATATGGTCTAGCGGTTAGGATTCCTGGTTTTC	1.92823784	0.390401	4.81943	0.666163
tsrna-13349	TCCCATATGGTCTAGCGGTTAGGATTCCTGGTTTT	1.85682163	0.702721	3.2828	0.333081
tsrna-13348	TCCCATATGGTCTAGCGGTTAGGATTCCTGGTTT	1.71398919	0.624641	3.212953	0.333081
tsrna-13347	TCCCATATGGTCTAGCGGTTAGGATTCCTGGTT	0.14283243	0.31232	0.488928	0.222054
tsrna-13346	TCCCATATGGTCTAGCGGTTAGGATTCCTGGT	0.49991351	0.858881	0.419081	0.444109
tsrna-13345	TCCCATATGGTCTAGCGGTTAGGATTCCTGG	0.28566487	0.468481	0.069847	0.111027
tsrna-13344	TCCCATATGGTCTAGCGGTTAGGATTCCTG	0.35708108	0.15616	0	0.333081
tsrna-13343	TCCCATATGGTCTAGCGGTTAGGATTCCT	0.14283243	0.07808	0.069847	0.111027
tsrna-13342	TCCCATATGGTCTAGCGGTTAGGATTCC	0.07141622	0.07808	0.139694	0.111027
tsrna-13341	TCCCATATGGTCTAGCGGTTAGGATTC	0.07141622	0	0.069847	0.444109
tsrna-13340	TCCCATATGGTCTAGCGGTTAGGATT	0.14283243	0	0.069847	0.333081

tsrna-13339	TCCCATATGGTCTAGCGGTTAGGAT	0	0	0.069847	0.222054
tsrna-13338	TCCCATATGGTCTAGCGGTTAGGA	0	0	0.279387	0.222054
tsrna-13337	TCCCATATGGTCTAGCGGTTAGG	0.07141622	0.07808	0.139694	0.333081
tsrna-13336	TCCCATATGGTCTAGCGGTTAG	0	0.07808	0	0
tsrna-13335	TCCCATATGGTCTAGCGGTTA	0	0	0	0
tsrna-13334	TCCCATATGGTCTAGCGGTT	0	0	0	0
tsrna-13333	TCCCATATGGTCTAGCGGT	0	0	0	0
tsrna-13332	TCCCATATGGTCTAGCGG	0	0	0	0
tsrna-13331	TCCCATATGGTCTAGCG	0	0	0	0
tsrna-13329	TCCCAGTAGAGCCTCCA	0.07141622	0.15616	0	0
tsrna-13328	TCCCAGTAGAGCCTCC	0.07141622	0	0	0
tsrna-13327	TCCCAGCGGTGCCTCCA	0	0	0.069847	0
tsrna-13322	TCCCACTTCTGACACCA	0	0	0	0.222054
tsrna-13321	TCCCACTTCTGACACC	0.07141622	0.07808	0	0
tsrna-13320	TCCCACTCCTGACACCA	0.8569946	0.858881	0.698468	0.555136
tsrna-13319	TCCCACTCCTGACACC	0.07141622	0	0.069847	0
tsrna-13318	TCCCACCTTCGTCGCCA	0	0.07808	0	0
tsrna-13315	TCCCACCTGGGGTACCA	0	0.07808	0	0
tsrna-13314	TCCCACCGCTGCTACCA	0.07141622	0	0	0
tsrna-13311	TCCCACCCTCGTCGCCA	0	0.23424	0	0.333081
tsrna-13310	TCCCACCCTCGTCGCC	0	0.07808	0	0
tsrna-13309	TCCCACCCGGGGTACCA	0.07141622	0	0	0
tsrna-13308	TCCCACCCAGGGACGCCA	0.28566487	0.31232	0	0.222054
tsrna-13306	TCCCACCAGAGTCGCCA	0	0	0	0
tsrna-13303	TCCCACATGGTCTAGCGGTTAGGATTCCTGGTTTTCACCCAG	8.49852975	3.357445	20.53496	3.219787
tsrna-13302	TCCCACATGGTCTAGCGGTTAGGATTCCTGGTTTTCACCCAG	7.78436759	2.264323	17.67124	2.220543
tsrna-13301	TCCCACATGGTCTAGCGGTTAGGATTCCTGGTTTTCACCCA	7.78436759	2.186243	19.27772	3.219787
tsrna-13300	TCCCACATGGTCTAGCGGTTAGGATTCCTGGTTTTCACCC	8.49852975	2.030083	16.27431	2.664651
tsrna-13299	TCCCACATGGTCTAGCGGTTAGGATTCCTGGTTTTCACC	8.64136219	1.952003	17.39185	2.442597
tsrna-13298	TCCCACATGGTCTAGCGGTTAGGATTCCTGGTTTTCA	8.92702705	2.888964	17.32201	3.552868
tsrna-13297	TCCCACATGGTCTAGCGGTTAGGATTCCTGGTTTTC	6.64170813	2.030083	16.55369	2.442597
tsrna-13296	TCCCACATGGTCTAGCGGTTAGGATTCCTGGTTTT	7.78436759	1.561602	13.62013	2.442597
tsrna-13295	TCCCACATGGTCTAGCGGTTAGGATTCCTGGTTT	6.99878921	1.483522	10.19763	1.665407
tsrna-13294	TCCCACATGGTCTAGCGGTTAGGATTCCTGGTT	0.21424865	0.780801	0.349234	1.221299
tsrna-13293	TCCCACATGGTCTAGCGGTTAGGATTCCTGGT	0.28566487	1.249282	0.768315	1.665407
tsrna-13292	TCCCACATGGTCTAGCGGTTAGGATTCCTGG	0.71416216	0.468481	1.117549	0.77719
tsrna-13291	TCCCACATGGTCTAGCGGTTAGGATTCCTG	0.57132973	0.390401	0.698468	1.998488
tsrna-13290	TCCCACATGGTCTAGCGGTTAGGATTCCT	0.35708108	0.858881	0.628621	1.776434
tsrna-13289	TCCCACATGGTCTAGCGGTTAGGATTCC	0.35708108	0.390401	0.908008	1.332326
tsrna-13288	TCCCACATGGTCTAGCGGTTAGGATTC	0.21424865	1.015041	0.628621	0.666163
tsrna-13287	TCCCACATGGTCTAGCGGTTAGGATT	0.4284973	0.31232	0.488928	1.998488
tsrna-13286	TCCCACATGGTCTAGCGGTTAGGAT	0.57132973	0.624641	0.838162	1.55438
tsrna-13285	TCCCACATGGTCTAGCGGTTAGGA	0.35708108	0.546561	0.349234	1.221299
tsrna-13284	TCCCACATGGTCTAGCGGTTAGG	0.21424865	1.093122	0.558774	1.55438
tsrna-13283	TCCCACATGGTCTAGCGGTTAG	0.28566487	0.468481	0.698468	1.110271
tsrna-13282	TCCCACATGGTCTAGCGGTTA	0.28566487	0.546561	0.628621	1.332326
tsrna-13281	TCCCACATGGTCTAGCGGTT	0	0.31232	0.069847	0.999244
tsrna-13280	TCCCACATGGTCTAGCGGT	0.28566487	0.15616	0.069847	0.222054
tsrna-13279	TCCCACATGGTCTAGCGG	0.07141622	0.390401	0.349234	0.555136
tsrna-13278	TCCCACATGGTCTAGCG	0.21424865	0.07808	0.20954	0.555136
tsrna-13277	TCCCACATGGTCTAGC	0	0.23424	0.419081	0.333081
tsrna-13276	TCCATTGTGCTTTGCACGCGTGGGTTCGAATCCCATCCTCGT	1.07124325	2.654724	0.558774	0
tsrna-13275	TCCATTGTGCTTTGCACGCGTGGGTTCGAATCCCATCCTCGT	0.4284973	0.858881	0.139694	0
tsrna-13274	TCCATTGTGCTTTGCACGCGTGGGTTCGA	0.07141622	0.858881	0	0
tsrna-13272	TCCATTGGTCTTAGGCCCCA	0	0	0	0
tsrna-13271	TCCATTGGTCTTAGGCCCC	0	0	0.069847	0
tsrna-13267	TCCATTGGGGTTTCCCCGCGCAGGTTCGAATCCTGCCGACTA	9.35552435	21.55011	2.793872	2.220543
tsrna-13266	TCCATTGGGGTTTCCCCGCGCAGGTTCGAATCCTGCCGACTA	0.78557838	1.249282	0.139694	0.111027
tsrna-13265	TCCATTGGGGTTTCCCCGCGCAGGTTCGA	0	0.07808	0	0
tsrna-13264	TCCATTGGGGTTTCCCCGCGCAGGTT	0.07141622	0	0	0
tsrna-13263	TCCATTGGGGTTTCCCCGCGCAGGT	0	0.07808	0	0
tsrna-13254	TCCATGGTGTAATGGTTAGCACTCTGGACT	0.14283243	0.23424	0	0
tsrna-13253	TCCATGGTGTAATGGTTAGCACTCTGGAC	0.21424865	0.07808	0	0.111027
tsrna-13252	TCCATGGTGTAATGGTTAGCACTCTGGA	0.07141622	0.07808	0.069847	0.222054

tsrna-13251	TCCATGGTGTAATGGTTAGCACTCTGG	0	0.07808	0	0
tsrna-13247	TCCATGGTGTAATGGTGAGCACTCTG	0	0	0	0
tsrna-13246	TCCATAGTGTAGTGGTTATCACGTCTGCTT	0.07141622	0	0	0
tsrna-13245	TCCATAGTGTAGTGGTTATCACGTCTGCT	0	0.07808	0.139694	0
tsrna-13243	TCCATAGTGTAGTGGTTATCACG	0	0	0	0
tsrna-13242	TCCATAGTGTAGCGGTTATCACGTCTG	0	0	0.069847	0
tsrna-13240	TCCAGTCTCTTCGGGGGCGTG	0	0.07808	0	0
tsrna-13238	TCCAGTCTCTTCGGGGG	0	0.07808	0	0
tsrna-13236	TCCAGGTTCGACTCCTGGCTGGCTCGCC	0	0.07808	0	0
tsrna-13235	TCCAGGTTCGACTCCTGGCTGGCTCG	0	0	0	0
tsrna-13231	TCCAGGTGCCCCCTCCA	0	0.15616	0	0
tsrna-13229	TCCAGGGTTCAAGTCCCTGTTCGGGCGCCA	0.57132973	1.327362	0.349234	0
tsrna-13228	TCCAGGGTTCAAGTCCCTGTTCGGGCGCC	0	0.07808	0	0
tsrna-13227	TCCAGGGTTCAAGTCCCTGTTCGGGCGC	0	0.07808	0	0
tsrna-13226	TCCAGGGTTCAAGTCCCTGTTCGGGCG	0	0	0	0
tsrna-13222	TCCAGGGTTCAAGTCCCTGTTCAGGCGCC	0	0	0.069847	0.111027
tsrna-13221	TCCAGGGTTCAAGTCCCTGTCCAGGCGCC	0	0	0	0
tsrna-13216	TCCAGCGATCCGAGTTCAAATCTCGGTGG	0	0	0	0
tsrna-13210	TCCAGATCAGAAGGTTGCGTGTT	0.14283243	0.31232	0	0.222054
tsrna-13209	TCCAGATCAGAAGGTTGCGTGT	0	0.31232	0.069847	0
tsrna-13204	TCCAGAGGTTCCGGGTTCGAGTCCCGGCAG	0	0	0	0.111027
tsrna-13203	TCCAATTAACTAGTTTTGACAACATTCAAAAAAGAGTACCA	0.14283243	0	0.069847	0
tsrna-13195	TCCAATGGGGTCTCCCCGCGCAGGT	0.07141622	0.07808	0	0
tsrna-13193	TCCAATGGAGGCGTGGGTTCGAATCCCACTTCTGACACCA	2.21390271	7.10529	1.327089	1.110271
tsrna-13192	TCCAATGGAGGCGTGGGTTCGAATCCCACTTCTG	0.8569946	2.186243	0.488928	0.111027
tsrna-13191	TCCAATGGAGGCGTGGGTTCGAA	0.99982703	1.873923	0.488928	0.111027
tsrna-13190	TCCAATGGAGGCGTGGGTTCG	1.2854919	2.030083	0.628621	0.111027
tsrna-13189	TCCAATGGAGGCGTGGGTTC	0.71416216	1.561602	0.488928	0.111027
tsrna-13188	TCCAATGGAGGCGTGGGTT	0.92841081	1.483522	0.698468	0.222054
tsrna-13187	TCCAATGGAGGCGTGGGT	0.4284973	1.327362	0.419081	0
tsrna-13186	TCCAATGGAGGCGTGGG	0	0.15616	0	0
tsrna-13185	TCCAATGGACATATGTCCGCGTGG	0.14283243	0.15616	0.139694	0
tsrna-13181	TCATTTCGATGGCGTGGGT	0.21424865	0.15616	0.20954	0
tsrna-13180	TCATTTCGATGGCGTGGG	0.07141622	0.07808	0	0
tsrna-13179	TCATTTCGATGGCGTGG	0	0.15616	0	0
tsrna-13178	TCATTTCGATGGCGTG	0	0	0.069847	0
tsrna-13177	TCATTGGTCGTGGTTGTAGTCCGTGCGAGAATACCA	8.14144867	10.22849	9.778553	5.44033
tsrna-13176	TCATTGGTCGTGGTTGTAGTCCGTGCGAGAATACC	0.71416216	1.952003	1.396936	1.332326
tsrna-13175	TCATTGGTCGTGGTTGTAGTCCGTGCGAGAATA	0.64274595	1.327362	1.047702	0.999244
tsrna-13174	TCATTGGTCGTGGTTGTAGTCCGTGCGAGAAT	0.21424865	0.858881	0.488928	1.110271
tsrna-13173	TCATTGGTCGTGGTTGTAGTCCGTGCGAGAA	0.4284973	0.858881	0.628621	1.110271
tsrna-13172	TCATTGGTCGTGGTTGTAGTCCGTGCGAGA	0.28566487	1.093122	0.419081	0.999244
tsrna-13171	TCATTGGTCGTGGTTGTAGTCCGTGCGAG	0.28566487	0.468481	0.20954	0.999244
tsrna-13170	TCATTGGTCGTGGTTGTAGTCCGTGCGA	0	0.468481	0.139694	0.333081
tsrna-13169	TCATTGGTCGTGGTTGTAGTCCGTGCG	0.28566487	0.31232	0.20954	0.333081
tsrna-13168	TCATTGGTCGTGGTTGTAGTCCGTGC	0.14283243	0	0	0.111027
tsrna-13167	TCATTGGTCGTGGTTGTAGTCCGTG	0.07141622	0.23424	0.069847	0.222054
tsrna-13166	TCATTGGTCGTGGTTGTAGTCCGT	0	0	0.069847	0
tsrna-13165	TCATTGGTCGTGGTTGTAGTCCG	0.07141622	0.15616	0	0
tsrna-13164	TCATTGGTCGTGGTTGTAGTCC	0	0	0.069847	0.111027
tsrna-13163	TCATTGGTCGTGGTTGTAGTC	0	0.15616	0	0
tsrna-13162	TCATTGGTCGTGGTTGTAGT	0	0	0	0
tsrna-13160	TCATTGGTCGTGGTTGTA	0	0	0.069847	0
tsrna-13157	TCATGGAGGCCATGGGGTTGGCTTGA	0	0	0	0
tsrna-13156	TCATGGAGGCCATGGGGTTGG	0	0	0	0
tsrna-13155	TCATGGAGGCCATGGGGTT	0	0.07808	0	0
tsrna-13154	TCATGCCCCCATGTCTAACAACATGGCTTTCTCACCA	0.78557838	0.624641	0.838162	0
tsrna-13153	TCATGCCCCCATGTCTAACAACATGGCTTTCTCACC	0.14283243	0.15616	0.349234	0
tsrna-13152	TCATGCCCCCATGTCTAACAACATGGCTTT	0	0	0.069847	0
tsrna-13151	TCATGCCCCCATGTCTAACAACATGGCT	0	0.07808	0	0
tsrna-13149	TCATGCCCCCATGTCTAACAACATG	0	0.07808	0	0
tsrna-13148	TCATGCCCCCATGTCTAACAACA	0	0.07808	0	0
tsrna-13144	TCATGCAAGATTCCCATTCTTGCGACCCGG	0.07141622	0	0.069847	0
tsrna-13142	TCATATCATTGGTCGTGGTTGTAGTCCGTGCGAGAATACCA	12.0693406	10.54081	12.85181	5.329303

tsrna-13141	TCATATCATTGGTCGTGGTTGTAGTCCGTGCGAGAATACC	1.2854919	1.873923	1.676323	2.220543
tsrna-13140	TCATATCATTGGTCGTGGTTGTAGTCCGTGCGAGAATA	1.42832433	1.015041	0.768315	0.999244
tsrna-13139	TCATATCATTGGTCGTGGTTGTAGTCCGTGCGAGAAT	0.4284973	1.015041	0.419081	1.55438
tsrna-13138	TCATATCATTGGTCGTGGTTGTAGTCCGTGCGAGAA	0.21424865	0.780801	0.768315	0.77719
tsrna-13137	TCATATCATTGGTCGTGGTTGTAGTCCGTGCGAGA	0.4284973	1.171202	1.047702	1.221299
tsrna-13136	TCATATCATTGGTCGTGGTTGTAGTCCGTG	0.14283243	0.468481	0.139694	0.111027
tsrna-13135	TCATATCATTGGTCGTGGTTGTAGTCCGT	0.21424865	0	0.069847	0.111027
tsrna-13134	TCATATCATTGGTCGTGGTTGTAGTCCG	0.07141622	0.15616	0.139694	0.111027
tsrna-13133	TCATATCATTGGTCGTGGTTGTAGTCC	0.07141622	0.390401	0.139694	0
tsrna-13132	TCATATCATTGGTCGTGGTTGTAGTC	0.07141622	0.07808	0.139694	0
tsrna-13131	TCATATCATTGGTCGTGGTTGTAGT	0.07141622	0	0	0
tsrna-13130	TCATATCATTGGTCGTGGTTGTAG	0	0	0	0
tsrna-13129	TCATATCATTGGTCGTGGTTGTA	0	0	0	0
tsrna-13128	TCATATCATTGGTCGTGGTTGT	0	0	0	0
tsrna-13123	TCATAATCTGAAGGTCGTGAGTTCGATCCTCACACGGGGCAC	1.92823784	5.231367	1.187396	0.999244
tsrna-13122	TCATAATCTGAAGGTCGTGAGTTCGAGCCTCACACGGGGCAC	2.28531893	4.216326	1.955711	0.77719
tsrna-13121	TCATAATCTGAAGGTCGTGAGTTC	1.57115676	2.576644	0.977855	0.555136
tsrna-13120	TCATAATCTGAAGGTCGTGAGT	1.71398919	3.357445	1.53663	0.666163
tsrna-13119	TCATAATCTGAAGGTCGTGAG	0.14283243	0.546561	0.069847	0.111027
tsrna-13118	TCATAATCTGAAGGTCGTG	0	0.468481	0	0.111027
tsrna-13117	TCATAATCTGAAGGTCCTGAGTTCGAACCTCAGAGGGGCAC	8.28428111	23.26787	6.146519	6.328547
tsrna-13116	TCATAATCTGAAGGTCCTGAGT	6.78454056	21.55011	6.356059	5.551357
tsrna-13115	TCATAATCTGAAGGTCCTGAG	0.49991351	2.264323	0.139694	0.222054
tsrna-13114	TCATAATCTGAAGGTCCTG	0.28566487	1.405442	0.139694	0.333081
tsrna-13113	TCATAACTTTGTCAAAGTTAAATT	0	0.23424	0	0
tsrna-13112	TCAGTTGGTTAGAGCGTGGTGC	0	0	0	0.333081
tsrna-13110	TCAGTTGGTAGAGCATCAG	0.14283243	0	0	0
tsrna-13108	TCAGTTGATGCAGAGTGGGGTTTTGCAGTC	0.07141622	0.07808	0	0
tsrna-13107	TCAGTTGATGCAGAGTGGGGTTTT	0.07141622	0	0	0.111027
tsrna-13106	TCAGTTGATGCAGAGTGGGGTT	0	0	0	0
tsrna-13105	TCAGTGGTCTAGTGGTTAGGATTC	0.07141622	0	0.069847	0
tsrna-13102	TCAGTGGTAGAGCATTTGACTGCA	0.14283243	0.468481	0.139694	0
tsrna-13101	TCAGTGGTAGAGCATTTGACTGC	0.28566487	0.23424	0	0.111027
tsrna-13100	TCAGTGGTAGAGCATTTGACTG	0	0.23424	0	0
tsrna-13099	TCAGTGGTAGAGCATTTGACT	0.14283243	0.15616	0.069847	0
tsrna-13098	TCAGTGGTAGAGCATTTGAC	0.07141622	0	0	0
tsrna-13097	TCAGTGGTAGAGCATTTGA	0.14283243	0	0	0
tsrna-13096	TCAGTGGTAGAGCATTTG	0.14283243	0.15616	0.069847	0
tsrna-13095	TCAGTGGTAGAATTTTC	0.07141622	0	0	0
tsrna-13088	TCAGTGGTAGAATTCTCGCCTTT	0.28566487	0.546561	0.279387	0.111027
tsrna-13087	TCAGTGGTAGAATTCTCGCCTT	0.35708108	0.624641	0.139694	0
tsrna-13086	TCAGTGGTAGAATTCTCGCCTGCCACG	3.4993946	7.573771	1.466783	2.553624
tsrna-13085	TCAGTGGTAGAATTCTCGCCTGCCA	1.21407568	3.123204	0.628621	0.444109
tsrna-13084	TCAGTGGTAGAATTCTCGCCTGCC	0.64274595	2.888964	0.698468	0.222054
tsrna-13083	TCAGTGGTAGAATTCTCGCCTGC	0.49991351	0.546561	0.279387	0.444109
tsrna-13082	TCAGTGGTAGAATTCTCGCCTG	0.21424865	0.31232	0.20954	0.111027
tsrna-13081	TCAGTGGTAGAATTCTCGCCTCCC	0.28566487	0.702721	0.069847	1.55438
tsrna-13080	TCAGTGGTAGAATTCTCGCCTCC	0.21424865	0.702721	0.419081	0.444109
tsrna-13079	TCAGTGGTAGAATTCTCGCCTC	0.28566487	0.624641	0.279387	0.333081
tsrna-13078	TCAGTGGTAGAATTCTCGCCT	0.21424865	0.390401	0.069847	0
tsrna-13077	TCAGTGGTAGAATTCTCGCC	0.14283243	0.07808	0.069847	0.111027
tsrna-13076	TCAGTGGTAGAATTCTCGC	0	0	0	0
tsrna-13072	TCAGTGGGTAGAGCATTTGACTG	0	0	0	0
tsrna-13071	TCAGTGGGTAGAGCATTTGACT	0.07141622	0.15616	0.069847	0
tsrna-13070	TCAGTGGGTAGAGCATTTGA	0	0	0.069847	0
tsrna-13069	TCAGTGGGTAGAGCATCAGAC	0	0.07808	0	0
tsrna-13068	TCAGTCTCATAATCTGAAGGTCG	0	0.31232	0.069847	0.111027
tsrna-13067	TCAGTCTCATAATCTGAAGGTC	0	0.390401	0.069847	0
tsrna-13066	TCAGTCGGTAGAGCTATC	0.14283243	0	0	0
tsrna-13065	TCAGTCGGTAGAGCATGGGACTCTTAATCC	1.07124325	2.030083	1.257243	0.222054
tsrna-13064	TCAGTCGGTAGAGCATGGGACTCTTAATC	1.07124325	1.015041	0.419081	0.222054
tsrna-13063	TCAGTCGGTAGAGCATGGGACTCTTAAT	0.92841081	1.405442	0.558774	0.222054
tsrna-13062	TCAGTCGGTAGAGCATGGGACTCTTAA	0.99982703	1.795843	0.628621	0.222054
tsrna-13061	TCAGTCGGTAGAGCATGGGACTCTTA	0.8569946	1.639682	0.838162	0.111027

tsrna-13060	TCAGTCGGTAGAGCATGGGACTCTT	0.92841081	1.795843	0.349234	0.444109
tsrna-13059	TCAGTCGGTAGAGCATGGGACTCT	0.64274595	1.952003	0.558774	0.333081
tsrna-13058	TCAGTCGGTAGAGCATGGGACTC	0.99982703	1.327362	0.279387	0
tsrna-13057	TCAGTCGGTAGAGCATGGGACT	1.21407568	1.327362	0.349234	0.222054
tsrna-13056	TCAGTCGGTAGAGCATGGGAC	0.78557838	1.249282	0.279387	0.111027
tsrna-13055	TCAGTCGGTAGAGCATGGGA	0.49991351	0.780801	0.349234	0
tsrna-13054	TCAGTCGGTAGAGCATGGG	0.21424865	0.31232	0.069847	0.111027
tsrna-13053	TCAGTCGGTAGAGCATGG	0.14283243	0.07808	0.069847	0
tsrna-13052	TCAGTCGGTAGAGCATGAGACTCTTA	0.92841081	2.420483	0.279387	0.999244
tsrna-13051	TCAGTCGGTAGAGCATGAGACTCTT	1.21407568	2.732804	0.558774	0.888217
tsrna-13050	TCAGTCGGTAGAGCATGAGACTCT	1.49974054	2.420483	0.419081	0.333081
tsrna-13049	TCAGTCGGTAGAGCATGAGACTC	0.99982703	2.420483	0.279387	0.333081
tsrna-13048	TCAGTCGGTAGAGCATGAGACT	0.4284973	1.873923	0.069847	0.666163
tsrna-13047	TCAGTCGGTAGAGCATGAGAC	0.8569946	1.171202	0.279387	0.222054
tsrna-13046	TCAGTCGGTAGAGCATGAGA	0.4284973	0.780801	0.139694	0.111027
tsrna-13045	TCAGTCGGTAGAGCATGAG	0.21424865	0.624641	0.069847	0
tsrna-13044	TCAGTCGGTAGAGCATGA	0	0.31232	0	0
tsrna-13043	TCAGTCGGTAGAGCATG	0.14283243	0.07808	0.139694	0
tsrna-13042	TCAGTCGGTAGAGCATCAGACTTTTAATC	0.78557838	1.093122	0.698468	0.333081
tsrna-13041	TCAGTCGGTAGAGCATCAGACTTTTAA	0.64274595	1.171202	0.628621	0.222054
tsrna-13040	TCAGTCGGTAGAGCATCAGACTTTTA	0.64274595	0.702721	0.349234	0.111027
tsrna-13039	TCAGTCGGTAGAGCATCAGACTTTT	0.71416216	0.780801	0.628621	0.222054
tsrna-13038	TCAGTCGGTAGAGCATCAGACTTT	1.07124325	1.015041	0.279387	0.222054
tsrna-13037	TCAGTCGGTAGAGCATCAGACTT	0.78557838	0.468481	0.349234	0.111027
tsrna-13036	TCAGTCGGTAGAGCATCAGACT	0.49991351	0.702721	0.558774	0.222054
tsrna-13035	TCAGTCGGTAGAGCATCAGAC	0.99982703	0.936961	0.558774	0.111027
tsrna-13034	TCAGTCGGTAGAGCATCAGA	0.49991351	1.327362	0.20954	0
tsrna-13033	TCAGTCGGTAGAGCATCAG	0.4284973	0.23424	0.20954	0.111027
tsrna-13032	TCAGTCGGTAGAGCATCA	0.35708108	0.390401	0.20954	0.222054
tsrna-13031	TCAGTCGGTAGAGCATC	0.07141622	0.15616	0.139694	0.111027
tsrna-13030	TCAGTCGGTAGAGCAT	0	0	0	0
tsrna-13029	TCAGTCAGTAGAGCATCAGAC	0.14283243	0	0	0.111027
tsrna-13024	TCAGGTGGTAGAGCATTTGACTG	0.07141622	0	0	0
tsrna-13023	TCAGGTGGTAGAGCATTTGACT	0	0.07808	0	0
tsrna-13019	TCAGGGTCGTGGGTTCGAGCC	0	0.07808	0	0
tsrna-13018	TCAGGGTCGTGGGTTCGAGC	0	0	0	0
tsrna-13017	TCAGGGTCGTGGGTTCGAG	0	0	0	0
tsrna-13015	TCAGGGTCGTGGGTTCG	0	0	0	0
tsrna-13014	TCAGGGTCGTGGGTTC	0	0	0	0
tsrna-13013	TCAGGGGTTAGAGCACTGG	0	0.07808	0	0
tsrna-13012	TCAGGGGTAGAGCATTTGACTGC	0	0	0	0
tsrna-13009	TCAGGGATGGGTTCGATTCTCA	0.21424865	0.468481	0.139694	0.111027
tsrna-13008	TCAGGGATGGGTTCGATTCTC	0.35708108	0.23424	0.069847	0
tsrna-13007	TCAGGGATGGGTTCGATTCT	0.07141622	0.15616	0.279387	0.111027
tsrna-13006	TCAGGGATGGGTTCGA	0.14283243	0	0.069847	0
tsrna-13005	TCAGGATGGCCGAGTGGTCTAAG	0	0.15616	0	0
tsrna-13004	TCAGGATGGCCGAGCGGTCTAAGGCGCTGCGTTC	0.07141622	0.390401	0.139694	0.222054
tsrna-13003	TCAGGATGGCCGAGCGGTCTAAGGCGCTGCGTT	0.14283243	0.468481	0.069847	0
tsrna-13002	TCAGGATGGCCGAGCGGTCTAAGGCGCTGCG	0.07141622	0.15616	0.069847	0.222054
tsrna-13001	TCAGGATGGCCGAGCGGTCTAAGGCGCTGC	0	0.702721	0.069847	0
tsrna-13000	TCAGGATGGCCGAGCGGTCTAAGGCGCTG	0.07141622	0.23424	0.069847	0
tsrna-12999	TCAGGATGGCCGAGCGGTCTAAGGC	0.21424865	0	0.20954	0
tsrna-12998	TCAGGATGGCCGAGCGGTCTAAGG	0.07141622	0.31232	0	0
tsrna-12997	TCAGGATGGCCGAGCGGTCTAAG	0	0.15616	0.069847	0
tsrna-12996	TCAGGATGGCCGAGCGGTCTA	0	0.07808	0	0
tsrna-12994	TCAGGATGGCCGAGCGGTC	0.07141622	0	0	0.111027
tsrna-12993	TCAGGATGGCCGAGCGG	0.07141622	0	0	0
tsrna-12992	TCAGGATGGCCGAGCG	0	0.07808	0	0
tsrna-12989	TCAGCTAAATAAGCTATCGGGCCCATACCCCGAAA	0.35708108	0.23424	0.279387	0.111027
tsrna-12988	TCAGCTAAATAAGCTATCGGGCCCA	0.07141622	0.23424	0.139694	0
tsrna-12987	TCAGCTAAATAAGCTATCGGGCCC	0	0	0	0
tsrna-12986	TCAGCTAAATAAGCTATCGGGCC	0	0	0	0
tsrna-12984	TCAGCCTCCGGAGCTGGGGATTGTGGGT	0.07141622	0.15616	0.139694	0.111027
tsrna-12983	TCAGCCTCCGGAGCTGGGGATTGTGGG	0	0.07808	0	0.111027

tsrna-12978	TCAGCACTCTGGACTCTGAATCC	0	0.07808	0	0
tsrna-12977	TCAGCACTCTGGACTCTGAATC	0	0.07808	0	0
tsrna-12972	TCAGATTGTGAATCTGACAACAGAGGCTTACGACCC	0.07141622	0.624641	0.628621	0
tsrna-12971	TCAGATTGTGAATCTGACAACAGAGGCTT	0.07141622	0.23424	0.139694	0
tsrna-12970	TCAGATTGTGAATCTGACAACAGAGGCT	0	0.31232	0.069847	0.111027
tsrna-12969	TCAGATTGTGAATCTGACAACAG	0	0.31232	0.139694	0
tsrna-12968	TCAGATTGTGAATCTGACAACA	0.07141622	0.23424	0.069847	0
tsrna-12967	TCAGATTGTGAATCTGACAAC	0	0.07808	0	0.111027
tsrna-12966	TCAGATTGTGAATCTGACA	0	0	0.069847	0
tsrna-12962	TCAGAGTTCAAGTCTCACTGGGA	0	0.07808	0	0
tsrna-12960	TCAGACTTTTAATCTGAGGGTCCAGGGT	1.49974054	3.357445	0.419081	0.999244
tsrna-12959	TCAGACTTTTAATCTGAGGGTCCAG	0.8569946	3.045124	0.628621	0.333081
tsrna-12958	TCAGAAGGTTGCGTGTTCAAGTCACGTCGGGGTCACCA	0.64274595	1.171202	0.20954	0.111027
tsrna-12957	TCAGAAGGTTGCGTGTTCAAGTCACGTCGGGGTCACC	0.14283243	0.468481	0.279387	0
tsrna-12956	TCAGAAGGTTGCGTGTTCAAGTCACGTCGG	0.14283243	0.468481	0.069847	0
tsrna-12955	TCAGAAGGTTGCGTGTTCAAG	0.28566487	0.31232	0	0
tsrna-12954	TCAGAAGGTTGCGTGTTCAAATCACGTCGGGGTCACCA	0.57132973	2.264323	0.558774	0.666163
tsrna-12953	TCAGAAGGTTGCGTGTTCAAATCACGTCGGGGTCACC	0.35708108	0.780801	0	0
tsrna-12952	TCAGAAGGTTGCGTGTTCAAATCACGTCGG	0.07141622	0.15616	0	0
tsrna-12951	TCAGAAGGTTGCGTGTTCAAA	0.07141622	0.15616	0	0
tsrna-12950	TCAGAAGGTTGCGTGTTCAA	0.07141622	0.390401	0.069847	0
tsrna-12949	TCAGAAGGTTGCGTGTTCA	0.28566487	0.15616	0	0.222054
tsrna-12948	TCAGAAGGTTGCGTGTTC	0.28566487	0.390401	0.069847	0
tsrna-12947	TCAGAAGGTTGCGTGTT	0.07141622	0.31232	0.069847	0
tsrna-12946	TCAGAAGGTTGCGTGT	0.07141622	0.23424	0	0
tsrna-12945	TCAGAAGGCTGCGTGTTCGAATCACGTCGGGGTCACCA	0.49991351	1.327362	0.419081	0.555136
tsrna-12944	TCAGAAGGCTGCGTGTTCGAATCACGTCGGGGTCACC	0.07141622	0.07808	0.069847	0
tsrna-12943	TCAGAAGGCTGCGTGTTCGAATCACGTCGGGGTCAC	0	0.15616	0.139694	0
tsrna-12942	TCAGAAGGCTGCGTGTTCGAATCACGTCGGGGTC	0.14283243	0.07808	0.069847	0
tsrna-12941	TCAGAAGGCTGCGTGTTCGAATCACGTCGGGG	0	0.15616	0	0
tsrna-12940	TCAGAAGGCTGCGTGTTCGAATCACGTCGGG	0	0.31232	0	0.111027
tsrna-12939	TCAGAAGGCTGCGTGTTCGAATCACGTCGG	0.14283243	0.07808	0.069847	0
tsrna-12938	TCAGAAGGCTGCGTGTTCGAATCACGTCG	0.14283243	0.07808	0	0
tsrna-12937	TCAGAAGGCTGCGTGTTCGAATCA	0	0.07808	0	0
tsrna-12936	TCAGAAGGCTGCGTGTTCGAATC	0	0.15616	0	0
tsrna-12935	TCAGAAGGCTGCGTGTTCGAAT	0	0.15616	0.069847	0.111027
tsrna-12933	TCAGAAGGCTGCGTGTTCGA	0	0.07808	0.069847	0
tsrna-12932	TCAGAAGGCTGCGTGTTCG	0	0	0	0
tsrna-12931	TCAGAAGGCTGCGTGTTC	0.07141622	0	0	0
tsrna-12928	TCAGAAGATTGCAGGTTCGAGTCCTGCCGCGGTCGCCA	0.21424865	0.546561	0.139694	0
tsrna-12927	TCAGAAGATTGCAGGTTCGAGTCCTGCCGCGGTCGCC	0	0.07808	0	0
tsrna-12925	TCAGAAGATTGAGGGTTCGAGTCCCTTCGTGGTCGCCA	0.28566487	1.249282	0.349234	0.444109
tsrna-12924	TCAGAAGATTGAGGGTTCGAGTCCCTTCGTGGTCGCC	0.21424865	0.546561	0.419081	0.333081
tsrna-12923	TCAGAAGATTGAGGGTTCGAGTCCCTTCGT	0.49991351	0.702721	0.139694	0
tsrna-12922	TCAGAAGATTGAGGGTTCGAATCCCTTCGTGGTTGCCA	0.07141622	1.171202	0.069847	0.222054
tsrna-12921	TCAGAAGATTGAGGGTTCGAATCCCTTCGTGGTTGCC	0.64274595	0.624641	0	0.222054
tsrna-12920	TCAGAAGATTGAGGGTTCGAATCCCTTCGT	0.21424865	1.015041	0.139694	0.222054
tsrna-12919	TCAGAAGATTGAGGGTTCGAATCCCTCCGT	0.35708108	0.390401	0.069847	0.222054
tsrna-12918	TCAGAAGATTCTAGGTTCGACTCCTGGCTGGCTCGCCA	2.78523244	11.24354	1.396936	1.110271
tsrna-12917	TCAGAAGATTCTAGGTTCGACTCCTGGCTGGCTCGCC	0.57132973	1.171202	0.139694	0
tsrna-12916	TCAGAAGATTCTAGGTTCGACTCCTGGCTG	0	0.468481	0.069847	0.111027
tsrna-12915	TCAGAAGATTCCAGGTTCGACTCCTGGCTGGCTCGCCA	2.14248649	10.46273	1.466783	1.443353
tsrna-12914	TCAGAAGATTCCAGGTTCGACTCCTGGCTGGCTCGCC	0.14283243	0.390401	0.279387	0
tsrna-12913	TCAGAAGATTCCAGGTTCGACTCCTGGCTGGCTCGC	0	0.15616	0	0.111027
tsrna-12912	TCAGAAGATTCCAGGTTCG	0.28566487	0.23424	0	0
tsrna-12911	TCAGAAGATTCCAGGTTC	0.14283243	0.07808	0	0
tsrna-12910	TAATGGATAAGGCACTGGC	1.64257298	1.873923	0.977855	0.77719
tsrna-12909	TAATGGATAAGGCACTGG	0.78557838	1.015041	0.558774	0.333081
tsrna-12908	TAATGGATAAGGCACTG	1.21407568	1.249282	0.419081	0.444109
tsrna-12907	TAATGGATAAGGCACT	0.4284973	0.07808	0.069847	0.111027
tsrna-12906	TAATGCCGAGGTTGTGAGTTCGAG	0.21424865	1.639682	0.139694	0
tsrna-12905	TAATGCCGAGGTTGTGAGTTCGA	0.07141622	0.702721	0.069847	0
tsrna-12904	TAATGCCGAGGTTGTGAGTTCG	0.07141622	0.936961	0	0
tsrna-12903	TAATGCCGAGGTTGTGAGTTCA	0.21424865	0.858881	0.139694	0

tsrna-12902	TAATGCCGAGGTTGTGAGTTC	0.07141622	0.23424	0.139694	0
tsrna-12901	TAATGCCGAGGTTGTGAGTT	0	0.23424	0.069847	0
tsrna-12900	TAATGCCGAGGTTGTGAGT	0	0	0	0
tsrna-12899	TAATGCCGAGGTTGTGAG	0	0	0	0
tsrna-12896	TAATCTGAGGGTCCAGGGTTCAAGTCCCTGTTCGGGCGCCA	0.4284973	1.795843	0.279387	0.222054
tsrna-12895	TAATCTGAGGGTCCAGGGTTCA	0	0.31232	0	0
tsrna-12894	TAATCTGAGGGTCCAGGGTTC	0.07141622	0.07808	0.069847	0
tsrna-12893	TAATCTGAGGGTCCAGGGTT	0	0.07808	0	0
tsrna-12892	TAATCTGAAGGTCGTGAGTTCGATCCTCACACGGGGCACCA	0.28566487	3.123204	0.139694	0.333081
tsrna-12891	TAATCTGAAGGTCGTGAGTTCGATCCTCACACGGGGCACC	0.57132973	0.702721	0.349234	0.222054
tsrna-12890	TAATCTGAAGGTCGTGAGTTCGATCCTCACACGGGGCAC	0.49991351	1.093122	0.279387	0.111027
tsrna-12889	TAATCTGAAGGTCGTGAGTTCGATCCTCACACGGGGC	0.78557838	1.249282	0.349234	0
tsrna-12888	TAATCTGAAGGTCGTGAGTTCGAGCCTCACACGGGGCACCA	0.64274595	1.405442	0.698468	0.333081
tsrna-12887	TAATCTGAAGGTCGTGAGTTCGAGCCTCACACGGGGCACC	0.21424865	1.249282	0.419081	0.222054
tsrna-12886	TAATCTGAAGGTCGTGAGTTCGAGCCTCACACGGGGCAC	0.49991351	0.936961	0.20954	0.111027
tsrna-12885	TAATCTGAAGGTCGTGAGTTCGAGCCTCACACGGGGC	0.71416216	1.171202	0.628621	0
tsrna-12884	TAATCTGAAGGTCGTGAGTTCGAGCCTCACACGGGG	0.21424865	1.249282	0	0.111027
tsrna-12883	TAATCTGAAGGTCGTGAGTTCGAGC	0.35708108	1.483522	0.349234	0
tsrna-12882	TAATCTGAAGGTCGTGAGTTCG	0.35708108	1.093122	0.349234	0.111027
tsrna-12881	TAATCTGAAGGTCGTGAGTTC	0.28566487	0.702721	0.20954	0.444109
tsrna-12880	TAATCTGAAGGTCGTGAGT	0.21424865	0.546561	0.20954	0
tsrna-12879	TAATCTGAAGGTCGTGAG	0.07141622	0.23424	0	0
tsrna-12878	TAATCTGAAGGTCGTGA	0.07141622	0.31232	0	0
tsrna-12877	TAATCTGAAGGTCGTG	0.07141622	0.07808	0	0
tsrna-12876	TAATCTGAAGGTCCTGAGTTCGAGCCTCAGAGAGGGC	5.2133838	13.3517	2.584332	2.442597
tsrna-12875	TAATCTGAAGGTCCTGAGTTCGAACCTCAGAGGGGGCACCA	4.64205407	13.19554	2.724025	2.664651
tsrna-12874	TAATCTGAAGGTCCTGAGTTCGAACCTCAGAGGGGGCACC	3.64222704	12.25858	2.514485	2.775678
tsrna-12873	TAATCTGAAGGTCCTGAGTTCGAACCTCAGAGGGGGCAC	3.28514596	11.08738	2.793872	2.220543
tsrna-12872	TAATCTGAAGGTCCTGAGTTCGAACCTCAGAGGGGGC	4.3563892	13.11746	3.2828	1.776434
tsrna-12871	TAATCTGAAGGTCCTGAGTTCGAACCTCAG	4.49922163	11.63394	3.212953	1.665407
tsrna-12870	TAATCTGAAGGTCCTGAGTTCGAACC	4.3563892	13.66402	2.235098	2.997733
tsrna-12869	TAATCTGAAGGTCCTGAGTTCG	4.64205407	11.08738	3.212953	1.665407
tsrna-12868	TAATCTGAAGGTCCTGAGT	4.49922163	12.1805	2.584332	2.220543
tsrna-12867	TAATCTGAAGGTCCTGAG	0.14283243	1.015041	0.069847	0
tsrna-12866	TAATCTGAAGGTCCTGA	0.14283243	0.624641	0.20954	0.111027
tsrna-12865	TAATCTGAAGGTCCTG	0.21424865	0.468481	0	0
tsrna-12864	TAATCTCAGGGTCGTGGGTTCG	0	0.07808	0	0
tsrna-12863	TAATCTCAGGGTCGTGGGTTC	0.07141622	0.07808	0	0
tsrna-12862	TAATCTCAGGGTCGTGGGTT	0	0.07808	0	0
tsrna-12861	TAATCTCAGGGTCGTGGGT	0.07141622	0	0	0
tsrna-12860	TAATCTCAGGGTCGTGG	0	0	0	0
tsrna-12856	TAATCCATTGTGCTTTGCACGCGTGGGTTC	0	0.702721	0.139694	0
tsrna-12855	TAATCCAGAGGTTCCGGGTTCGAG	0	0	0	0
tsrna-12854	TAATCCAGAGGTTCCGGGTTCGA	0.14283243	0	0	0.111027
tsrna-12847	TAATAGGAGCTTAAACCCCCTTATTTCTAC	0	0	0.069847	0
tsrna-12846	TAATAGGAGCTTAAACCCCCT	0	0	0.069847	0
tsrna-12840	TAATAACGCCAAGGTCGCGGGT	0.57132973	1.483522	0.20954	0.333081
tsrna-12839	TAAGTTGCAATACTTAATTTCTGCCA	0.35708108	0	0.069847	0.333081
tsrna-12838	TAAGTTGCAATACTTAATTTCTGCC	0	0	0.069847	0
tsrna-12837	TAAGTTAAAGATTAAGAGAACCAACACCTCTTTACAGTGACCA	0.28566487	0.07808	1.117549	0.111027
tsrna-12836	TAAGTTAAAGATTAAGAGAACCAACACCTCTTTACAGTGACC	0.4284973	0.15616	0.069847	0
tsrna-12835	TAAGTTAAAGATTAAGAGAACCAACACC	0	0	0	0
tsrna-12834	TAAGTTAAAGATTAAGAGAACC	0.07141622	0	0	0
tsrna-12833	TAAGTTAAAGATTAAGAGAAC	0	0	0	0
tsrna-12832	TAAGTGTTTGTGGGTTTAAGTCCCATTGGTCTAGCCA	0	0.07808	0.069847	0.111027
tsrna-12831	TAAGTGTTTGTGGGTTTAAGTCCCATTGGTCTAGC	0.07141622	0.23424	0	0
tsrna-12830	TAAGTGTTTGTGGGTTTAAGTCCCATTGGT	0.07141622	0	0	0
tsrna-12829	TAAGTGTTTGTGGGTTTAAGTCCCATT	0	0	0.20954	0.111027
tsrna-12828	TAAGTGTTTGTGGGTTTAAGTCC	0	0	0	0
tsrna-12827	TAAGTGTTTGTGGGTTTAAGTC	0.07141622	0	0.069847	0
tsrna-12825	TAAGTGTTTGTGGGTTTAAG	0.07141622	0	0	0
tsrna-12821	TAAGTCCCATTGGTCTAGCCA	0	0.07808	0	0
tsrna-12818	TAAGGTCAGCTAAATAAGCTATCGGGCCCA	0.07141622	0	0	0
tsrna-12817	TAAGGTCAGCTAAATAAGCTATCGGGCCC	0	0	0	0

tsrna-12795	TAAGGCGTCTGACTTCGGATC	0	0	0	0
tsrna-12791	TAAGGCGTCTGACTTCG	0	0.07808	0	0
tsrna-12789	TAAGGCGCCAGACTCAAG	0	0	0	0
tsrna-12788	TAAGCTATCGGGCCCATACCCCGAAAATGTTGGTTA	0.21424865	0.23424	0.488928	0
tsrna-12787	TAAGCTATCGGGCCCATACCCCGAAAATGTTGGTT	0.21424865	0	0.349234	0.111027
tsrna-12786	TAAGCTATCGGGCCCATACCCCGAAAATGTT	0	0.15616	0.069847	0
tsrna-12785	TAAGCTATCGGGCCCATACCCCGAAAATGT	0	0.23424	0	0.111027
tsrna-12784	TAAGCTATCGGGCCCATACCCCGAAA	0	0.07808	0.069847	0
tsrna-12783	TAAGCTATCGGGCCCATACCCCGAA	0.07141622	0	0	0
tsrna-12782	TAAGCTATCGGGCCCATACCCCGA	0	0.15616	0.069847	0
tsrna-12781	TAAGCTATCGGGCCCATACCCC	0.07141622	0.07808	0	0.111027
tsrna-12780	TAAGCTATCGGGCCCATACC	0	0.07808	0.069847	0.111027
tsrna-12779	TAAGCTATCGGGCCCATA	0	0.07808	0.069847	0
tsrna-12778	TAAGCTATCGGGCCCA	0.07141622	0	0	0
tsrna-12777	TAAGCCAGGGATTGTGGGTTCGAGTCCCATCTGGGGTGCCA	1.71398919	5.543688	1.117549	1.110271
tsrna-12776	TAAGCCAGGGATTGTGGGTTCGAGTCCCATCTGGGGTGCC	2.07107028	3.591685	0.698468	0.999244
tsrna-12775	TAAGCCAGGGATTGTGGGTTCGAGTCCCATCTGGGGTGC	2.14248649	4.216326	0.698468	0.666163
tsrna-12774	TAAGCCAGGGATTGTGGGTTCGAGTCCCACCTGGGGTACC	1.85682163	4.138246	0.838162	0.555136
tsrna-12773	TAAGCCAGGGATTGTGGGTTCGAGTCCCACCCGGGGTACC	1.57115676	4.840967	1.047702	0.555136
tsrna-12772	TAAGCCAGGGATTGTGGGTTCGAGTCCC	1.2854919	4.372486	0.838162	0.77719
tsrna-12771	TAAGCCAGGGATTGTGGGTTCGAGT	1.92823784	4.606726	0.908008	0.888217
tsrna-12770	TAAGCCAGGGATTGTGGGTTCGAG	1.07124325	3.669765	0.698468	0.77719
tsrna-12769	TAAGCCAGGGATTGTGGGTTCGA	1.2854919	4.138246	1.327089	0.333081
tsrna-12768	TAAGCCAGGGATTGTGGGTTCG	1.35690811	4.216326	0.838162	0.77719
tsrna-12767	TAAGCCAGGGATTGTGGGTTC	1.78540541	3.747845	0.628621	0.77719
tsrna-12766	TAAGCCAGGGATTGTGGGTT	1.2854919	4.684807	0.977855	0.999244
tsrna-12765	TAAGCCAGGGATTGTGGGT	1.35690811	3.904006	0.768315	1.110271
tsrna-12764	TAAGCCAGGGATTGTGGG	0	0	0	0
tsrna-12761	TAAGCACTCTGGACTCTGAATCCAGCG	0.14283243	0.31232	0.069847	0.111027
tsrna-12760	TAAGCACTCTGGACTCTGAATCCAGC	0	0.31232	0	0.222054
tsrna-12759	TAAGCACTCTGGACTCTGAATCCA	0	0.31232	0.069847	0
tsrna-12758	TAAGCACTCTGGACTCTGAATCC	0	0.07808	0.069847	0
tsrna-12757	TAAGCACTCTGGACTCTGAATC	0.07141622	0	0.069847	0
tsrna-12751	TAAGATGGCAGAGCCCGGTAATCGCATAAAACTTAA	0	0	0	0.111027
tsrna-12746	TAAGAGAACCAACACCTCTTTACAGTGACCA	0.07141622	0	0.069847	0
tsrna-12745	TAAGAGAACCAACACCTCTTTACAGTGACC	0	0	0	0
tsrna-12738	TAACTTTGTCAAAGTTAAATTATAGGCT	0.78557838	0.23424	0.349234	0.111027
tsrna-12737	TAACTTTGTCAAAGTTAAATT	0	0.15616	0.069847	0
tsrna-12735	TAACTTGACCGCTCTGACCA	0.71416216	1.093122	0.558774	0
tsrna-12734	TAACTTGACCGCTCTGACC	0.07141622	0.468481	0.069847	0
tsrna-12733	TAACTTGACCGCTCTGAC	0	0.07808	0	0
tsrna-12725	TAACTCATGCCCCCATGTCTAACAACATGGCTTTCTCACCA	0.71416216	0.780801	2.165251	0
tsrna-12724	TAACTCATGCCCCCATGTCTAACAACATGGCTTTCTCACC	0.99982703	0.31232	0.768315	0
tsrna-12723	TAACTCATGCCCCCATGTCTAACAACATGGCT	0	0.07808	0.069847	0
tsrna-12719	TAACTAAGTGTTTGTGGGTTT	0	0.15616	0	0
tsrna-12718	TAACTAAGTGTTTGTGGGTT	0.07141622	0.07808	0	0
tsrna-12717	TAACGCTAAGGTCGCGGGTTCGA	4.71347028	9.603854	5.797285	2.109516
tsrna-12715	TAACGCGTCTGACTACGGATCAGAAGA	0	0.07808	0	0
tsrna-12713	TAACGCGTCTGACTACGGAT	0	0	0	0
tsrna-12711	TAACGCCAAGGTCGCGGGTTCGATCCCCGTACGGGCCACCA	1.35690811	11.0093	0.419081	0.444109
tsrna-12710	TAACGCCAAGGTCGCGGGTTCGATCCCCGTACGGGCCACC	0.49991351	1.015041	0.20954	0
tsrna-12709	TAACGCCAAGGTCGCGGGTTCGATCCCCGTACGGGC	0.07141622	0.15616	0.069847	0
tsrna-12708	TAACGCCAAGGTCGCGGGTTCGATC	0.28566487	0.390401	0	0
tsrna-12707	TAACGCCAAGGTCGCGGGTTCGAT	0.14283243	0.936961	0	0
tsrna-12706	TAACGCCAAGGTCGCGGGTTCGAA	0.35708108	0.390401	0.069847	0
tsrna-12705	TAACGCCAAGGTCGCGGGTTCGA	0.21424865	0.936961	0.069847	0
tsrna-12704	TAACGCCAAGGTCGCGGGTTCG	0	0.546561	0.139694	0
tsrna-12703	TAACGCCAAGGTCGCGGGTTC	0.14283243	0.15616	0.069847	0
tsrna-12702	TAACGCCAAGGTCGCGGGTT	0	0.07808	0	0
tsrna-12701	TAACGCCAAGGTCGCGGGT	0	0.23424	0	0
tsrna-12698	TAACCTTTTAAGTTAAAGATTAAGAGAACC	0.07141622	0	0	0
tsrna-12697	TAACCGAAAGGTTGGTGGTTCGAGCCCACCCAGGGACGCCA	0.78557838	8.666892	0.139694	0.333081
tsrna-12696	TAACCGAAAGGTTGGTGGTTCGAGCCCACCCAGGGACGCC	0.28566487	1.015041	0.069847	0.111027
tsrna-12695	TAACCGAAAGGTTGGTGGTTCGAGCCCACCCAGGGAC	0.07141622	0.31232	0.069847	0.111027

tsrna-12694	TAACCGAAAGGTTGGTGGTTCGA	0	0.07808	0	0
tsrna-12693	TAACCGAAAGGTTGGTGGTTCG	0.07141622	0	0	0
tsrna-12692	TAACCGAAAGGTTGGTGGTT	0	0.23424	0	0
tsrna-12691	TAACCCAGAGGTCGATGGATCT	0.92841081	1.952003	0.20954	0.222054
tsrna-12690	TAACCCAGAGGTCGATGGATCGAAACCATC	0.35708108	0.23424	0	0
tsrna-12689	TAACCCAGAGGTCGATGGATCGAAACCA	0	0.23424	0	0
tsrna-12688	TAACCCAGAGGTCGATGGATCGAAACC	0.14283243	0.07808	0	0
tsrna-12687	TAACCCAGAGGTCGATGGATCGAAAC	0.07141622	0.31232	0.139694	0
tsrna-12686	TAACCCAGAGGTCGATGGATCGAAA	0	0.390401	0	0
tsrna-12685	TAACCCAGAGGTCGATGGATCGAA	0.21424865	0.31232	0	0
tsrna-12684	TAACCCAGAGGTCGATGGATCGA	0	0.31232	0.069847	0.111027
tsrna-12683	TAACCCAGAGGTCGATGGATCG	0	0.07808	0.069847	0
tsrna-12682	TAACCCAGAGGTCGATGGATC	0.07141622	0.23424	0.069847	0
tsrna-12681	TAACCCAGAGGTCGATGGAT	0	0.31232	0	0.111027
tsrna-12680	TAACCCAGAGGTCGATGGA	0.21424865	0.07808	0.069847	0
tsrna-12679	TAACCCAGAGGTCGATGG	0	0.23424	0	0.111027
tsrna-12678	TAACCCAGAGGTCGATG	0.07141622	0	0	0
tsrna-12677	TAACCCAGAGGTCGAT	0	0.07808	0	0
tsrna-12676	TAACCCAGAGGTCAATGGATCGA	0	0.07808	0	0
tsrna-12675	TAACCAAAACATCAGATTGTGAATCTGACAACAGAGGCTTAC(	0.35708108	0.780801	0.628621	0.111027
tsrna-12674	TAACCAAAACATCAGATTGTGAATCTGACAACAGAGGCT	0	0.390401	0.349234	0
tsrna-12673	TAACCAAAACATCAGATTGTGAATCTGACAACAGAGG	0.07141622	0.15616	0.069847	0
tsrna-12672	TAACCAAAACATCAGATTGTGAATCTGAC	0	0	0.069847	0
tsrna-12662	TAACAGCTATCCATTGGTCTTAGGCCCC	0.07141622	0.07808	0	0
tsrna-12661	TAACAGCTATCCATTGGTCTTAGGCCC	0	0	0	0
tsrna-12660	TAACAGCTATCCATTGGTCTTAGGCC	0	0	0.069847	0
tsrna-12659	TAACAGCTATCCATTGGTCTTAGGC	0	0	0	0
tsrna-12658	TAACAGCTATCCATTGGTCTTAGG	0	0	0	0
tsrna-12657	TAACAGCTATCCATTGGTCTTAG	0	0	0.069847	0
tsrna-12656	TAACAGCTATCCATTGGTCTTA	0	0.07808	0	0
tsrna-12655	TAACAGCTATCCATTGGTCTT	0	0	0	0
tsrna-12650	TAACACCAAGGTCGCGGGCTCGAC	0	0	0	0
tsrna-12648	TAACACCAAGGTCGCGGGCTCG	0	0.07808	0	0
tsrna-12647	TAACACCAAGGTCGCGGGCT	0	0	0.139694	0
tsrna-12644	TAACACAAAGCACCCAACTTACACTTAGGA	0	0	0.069847	0
tsrna-12643	TAACACAAAGCACCCAACTTACACTTAGG	0	0	0	0
tsrna-12640	TAACACAAAGCACCCAACTTACACTT	0	0	0	0
tsrna-12634	TAACACAAAGCACCCAACTT	0	0	0	0
tsrna-12633	TAACACAAAGCACCCAACT	0	0	0	0
tsrna-12629	TAACAACATGGCTTTCTCACCA	0	0	0.069847	0
tsrna-12628	TAACAACATGGCTTTCTCACC	0	0	0	0
tsrna-12627	TAACAACATGGCTTTCTCAC	0	0	0	0
tsrna-12626	TAACAACATGGCTTTCTCA	0.07141622	0	0	0
tsrna-12621	TAAATTATAGGCTAAATCCTATATATCTT	0.07141622	0	0.069847	0
tsrna-12619	TAAATTAGAATCTTAGCTTTGGGTGCTAATGGTGGAGTTAAA(	0.4284973	0.468481	0.488928	0
tsrna-12618	TAAATTAGAATCTTAGCTTTGGGTGCTAATGG	0.28566487	0.15616	0.20954	0.111027
tsrna-12617	TAAATTAGAATCTTAGCTTTGGGTGCTA	0.07141622	0.15616	0.279387	0
tsrna-12616	TAAATTAGAATCTTAGCTTTGGGTGCT	0.4284973	0.07808	0.069847	0.111027
tsrna-12615	TAAATTAGAATCTTAGCTTTGGGTG	0.21424865	0.31232	0.069847	0.222054
tsrna-12614	TAAATTAGAATCTTAGCTTTGGGT	0.49991351	0.07808	0.20954	0
tsrna-12613	TAAATTAGAATCTTAGCTTTGG	0.14283243	0	0.20954	0.111027
tsrna-12612	TAAATTAGAATCTTAGCTTTG	0.07141622	0	0.279387	0.222054
tsrna-12611	TAAATTAGAATCTTAGCTTT	0.07141622	0.15616	0.279387	0.111027
tsrna-12609	TAAATCTAAAGACAGGGGTTAGGCCTCTTTTTACCACCA	5.28480002	3.513605	4.540042	0.666163
tsrna-12608	TAAATCTAAAGACAGGGGTTAGGCCTCTTTTTACCACC	3.07089731	3.747845	1.885864	0.222054
tsrna-12607	TAAATCTAAAGACAGGGGTTAGGCCTCTTT	0.49991351	1.405442	0.139694	0
tsrna-12606	TAAATCTAAAGACAGGGGTTAGGCCTCTT	0.35708108	0.468481	0.139694	0
tsrna-12605	TAAATCTAAAGACAGGGGTTAGGCCTC	0.07141622	0.07808	0	0
tsrna-12604	TAAATCTAAAGACAGGGGTTAGGCCT	0	0	0	0
tsrna-12602	TAAATCTAAAGACAGGGG	0.07141622	0	0	0
tsrna-12598	TAAATAGTACCGTTAACTTCCA	0.07141622	0.15616	0.20954	0
tsrna-12596	TAAATACAGACCAAGAGCCTTCAAAGCCCT	1.57115676	0.468481	1.047702	0.222054
tsrna-12595	TAAATACAGACCAAGAGCCTTCAA	1.2854919	0.07808	0.488928	0.666163
tsrna-12594	TAAATACAGACCAAGAGCCTTCA	0.8569946	0.23424	0.628621	0.333081

tsrna-12593	TAAATACAGACCAAGAGCCTTC	0.71416216	0	0.558774	0
tsrna-12592	TAAATACAGACCAAGAGCCTT	0	0	0.069847	0
tsrna-12591	TAAATACAGACCAAGAGCCT	0.07141622	0	0.069847	0
tsrna-12590	TAAATACAGACCAAGAGCC	0	0	0	0
tsrna-12586	TAAATAATAGGAGCTTAAACCC	0	0.07808	0	0
tsrna-12585	TAAATAATAGGAGCTTAAACC	0	0	0	0
tsrna-12583	TAAATAATAGGAGCTT	0	0	0	0
tsrna-12582	TAAATAAGCTATCGGGCCCATA	0.07141622	0.07808	0.069847	0
tsrna-12581	TAAATAAGCTATCGGGCCCA	0.07141622	0.07808	0.069847	0
tsrna-12580	TAAATAAGCTATCGGGCCC	0	0	0	0
tsrna-12577	TAAAGTGGCTGATTTGCGTTCA	0	0	0	0
tsrna-12576	TAAAGTGGCTGATTTGCGTTC	0	0.07808	0	0
tsrna-12575	TAAAGGTCCCTGGTTCGATCCC	0.8569946	0.858881	0.488928	0.222054
tsrna-12574	TAAAGGTCCCTGGTTCG	0.78557838	1.327362	0.488928	0.666163
tsrna-12572	TAAAGATTAAGAGAACCAACACCTCTTTACAGTGACCA	0.71416216	0.07808	0.628621	0
tsrna-12571	TAAAGATTAAGAGAACCAACACCTCTTTACAGTGACC	0.21424865	0.15616	0.20954	0.111027
tsrna-12570	TAAAGATTAAGAGAACCAACACCTCTTTACAGTGAC	0.14283243	0	0.069847	0
tsrna-12569	TAAAGATTAAGAGAACCAACACCTCTTTACAGTGA	0.07141622	0.07808	0.139694	0
tsrna-12568	TAAAGATTAAGAGAACCAACACCTCTTTAC	0.07141622	0	0	0
tsrna-12567	TAAAGATTAAGAGAACCAACACCTCTTTA	0.07141622	0.07808	0	0
tsrna-12566	TAAAGATTAAGAGAACCAACACC	0	0	0.069847	0
tsrna-12564	TAAAGATTAAGAGAACCAACA	0	0	0	0
tsrna-12562	TAAAGATTAAGAGAACCAA	0	0	0	0
tsrna-12561	TAAAGATTAAGAGAACCA	0	0	0	0
tsrna-12558	TAAAGACTTTTTCTCTGACCA	0.57132973	1.171202	0.977855	1.221299
tsrna-12557	TAAAGACTTTTTCTCTGACC	0	0.07808	0.20954	0
tsrna-12556	TAAAGACTTTTTCTCTGAC	0	0.15616	0	0
tsrna-12554	TAAAGACTTTTTCTCTG	0	0.07808	0	0
tsrna-12553	TAAAGACAGGGGTTAGGCCTCTTTTTACCACCA	0.4284973	0.468481	0.349234	0.111027
tsrna-12552	TAAAGACAGGGGTTAGGCCTCTTTTTACCACC	0.14283243	0.31232	0.069847	0.222054
tsrna-12551	TAAAGACAGGGGTTAGGCCTCTTTTTACC	0.07141622	0.390401	0.069847	0
tsrna-12550	TAAAGACAGGGGTTAGGCCT	0	0	0	0.111027
tsrna-12549	TAAAGACAGGGGTTAGGCC	0	0	0	0
tsrna-12547	TAAACCGGAGATGAAAACCTTTTTCCAAGGACACCA	3.07089731	2.498564	6.5656	0
tsrna-12546	TAAACCGGAGATGAAAACCTTTTTCCAAGGACACC	0.57132973	0.936961	0.419081	0
tsrna-12545	TAAACCGGAGATGAAAACCTTTTTCCAAGG	0.14283243	0.858881	0.069847	0
tsrna-12544	TAAACCGGAGATGAAAACCTTTTTCCAAG	0.21424865	0.702721	0	0
tsrna-12543	TAAACCGGAGATGAAAACCT	0	0	0	0
tsrna-12540	TAAACCGAAGATCGCGGGTTCGAACCCCGTCCGTGCCTCCA	0.99982703	2.576644	0.139694	0
tsrna-12539	TAAACCGAAGATCGCGGGTTCGAACCCCGT	0.21424865	1.405442	0.069847	0
tsrna-12538	TAAACCGAAGATCGCGGGTTCGAAC	0.21424865	0.624641	0	0
tsrna-12537	TAAACCGAAGATCGCGGGTTCGAA	0.07141622	0.624641	0.069847	0
tsrna-12536	TAAACCGAAGATCGCGGGTTCGA	0.14283243	0.468481	0.20954	0
tsrna-12535	TAAACCGAAGATCGCGGGTTCG	0.21424865	0.31232	0.139694	0
tsrna-12534	TAAACCGAAGATCGCGGGT	0.14283243	0.468481	0	0.222054
tsrna-12532	TAAACCGAAGATCACGGGTTCGAACCCCGTCCGTGCCTCCA	0.28566487	1.249282	0.20954	0
tsrna-12531	TAAACCGAAGATCACGGGTTCGA	0	0.15616	0.139694	0
tsrna-12530	TAAACCCCCTTATTTCTACCA	0.07141622	0.23424	0	0
tsrna-12529	TAAACCCCCTTATTTCTACC	0.07141622	0.07808	0.069847	0
tsrna-12528	TAAACCCCCTTATTTCTAC	0	0.07808	0	0
tsrna-12527	TAAACCAGGGGTCGCGAGTTCGA	0	0.07808	0.069847	0.111027
tsrna-12526	TAAACCAGGGGTCGCGAGTTCG	0	0.390401	0	0.111027
tsrna-12525	TAAACCAGGGGTCGCGAGTTCAAATCTCGCTGGGGCCTCCA	2.92806487	10.38465	1.606477	2.442597
tsrna-12524	TAAACCAGGGGTCGCGAGTTCAAATCTCGCTGGGGCCT	0.07141622	0.23424	0.069847	0
tsrna-12523	TAAACCAGGGGTCGCGAGTTCAAA	0.07141622	0	0.139694	0
tsrna-12522	TAAACCAGGGGTCGCGAGTTCAA	0	0.15616	0.069847	0
tsrna-12521	TAAACCAGGGGTCGCGAGTTCA	0.07141622	0.07808	0	0
tsrna-12520	TAAACCAGGGGTCGCGAGTTC	0.07141622	0.23424	0	0
tsrna-12519	TAAACCAGGGGTCGCGAGTT	0.14283243	0.07808	0	0.111027
tsrna-12518	TAAACCAGGGGTCGCGAGT	0	0.15616	0	0
tsrna-12516	TAAACCAGGGGTCGCGA	0	0.07808	0	0
tsrna-12514	TAAACAGGAGATCCTGGGTTCGAATCCCAGCGGTGCCTC	0.78557838	2.342403	0.419081	0.222054
tsrna-12513	TAAACAGGAGATCCTGGGTTCGAATCCCAGCGGTGCCT	0.35708108	1.717762	0.558774	0
tsrna-12512	TAAACAGGAGATCCTGGGTTCGAATCCCAGCGGTGCC	0.71416216	2.420483	0.20954	0.333081

tsrna-12511	TAAACAGGAGATCCTGGGTTCGAATCCCAGCGGTGC	1.21407568	2.108163	0.279387	0.111027
tsrna-12510	TAAACAGGAGATCCTGGGTTCGAATCCCAGCGGGGCCTC	0.71416216	1.639682	0.419081	0.222054
tsrna-12509	TAAACAGGAGATCCTGGGTTCGAATCCCAGCGGGGCCT	0.35708108	1.483522	0.558774	0.111027
tsrna-12508	TAAACAGGAGATCCTGGGTTCGAATCCCAGCGGGGCC	0.78557838	1.717762	0.488928	0.222054
tsrna-12507	TAAACAGGAGATCCTGGGTTCGAATCCCAGCGGGGC	0.35708108	2.108163	0.139694	0.111027
tsrna-12506	TAAACAGGAGATCCTGGGTTCGA	0.64274595	1.561602	0.279387	0.222054
tsrna-12505	TAAACAGGAGATCCTGGGTTCG	0.57132973	1.795843	0.838162	0
tsrna-12504	TAAACAAAACGAATGATTTCGACTCATTAAATTATG	0.21424865	0.780801	0.628621	0.111027
tsrna-12503	TAAACAAAACGAATGATTTCGACTC	0.07141622	0.23424	0.139694	0
tsrna-12501	TAAAATGGCTGAGTGAAGCATTGGACTGT	0	0	0.069847	0
tsrna-12500	TAAAATGGCTGAGTGAAGCATTGGACTG	0	0	0.069847	0
tsrna-12499	TAAAATGGCTGAGTGAAGCATTGGACT	0	0.07808	0	0
tsrna-12498	TAAAATGGCTGAGTGAAGCATTGGAC	0	0	0	0
tsrna-12497	TAAAATGGCTGAGTGAAGCATTGG	0.07141622	0	0	0
tsrna-12496	TAAAATGGCTGAGTGAAGCATTG	0.07141622	0.07808	0	0
tsrna-12492	TAAAACTTAAAACTTTACAGTCAG	0	0	0	0.111027
tsrna-12490	GTTTTTCATATCATTGGTCGTGGTTGTAGTCCGTGCGAGAAT/	10.3553514	12.72706	12.71212	6.883683
tsrna-12489	GTTTTTCATATCATTGGTCGTGGTTGTAGTCCGTGCGAGAAT/	1.2854919	2.888964	1.466783	2.442597
tsrna-12488	GTTTTTCATATCATTGGTCGTGGTTGTAGTCCGTGCGAGAAT/	0.78557838	2.576644	0.768315	1.665407
tsrna-12487	GTTTTTCATATCATTGGTCGTGGTTGTAGTCCGTGCGAGAAT	0.49991351	1.327362	0.768315	1.443353
tsrna-12486	GTTTTTCATATCATTGGTCGTGGTTGTAGTCCGTGCGAGAA	0.64274595	0.936961	0.558774	1.332326
tsrna-12485	GTTTTTCATATCATTGGTCGTGGTTGTAGTCCGTGCGAGA	0.64274595	1.171202	0.628621	1.665407
tsrna-12484	GTTTTTCATATCATTGGTCGTGGTTGTAGTCCGTGCGA	0.35708108	0.780801	0.349234	0.77719
tsrna-12483	GTTTTTCATATCATTGGTCGTGGTTGTAGTCCGTGCG	0.57132973	0.468481	0.628621	0.77719
tsrna-12482	GTTTTTCATATCATTGGTCGTGGTTGTAGTCCGTGC	0.07141622	0.31232	0.20954	0.222054
tsrna-12481	GTTTTTCATATCATTGGTCGTGGTTGTAGTCC	0.14283243	0.07808	0.069847	0.111027
tsrna-12480	GTTTTTCATATCATTGGTCGTGGTTGTAGT	0	0	0.069847	0
tsrna-12479	GTTTTTCATATCATTGGTCGTGGTTGTAG	0	0	0	0
tsrna-12478	GTTTTTCATATCATTGGTCGTGGTTGTA	0	0.07808	0	0
tsrna-12477	GTTTTTCATATCATTGGTCGTGGTTGT	0	0.07808	0	0
tsrna-12476	GTTTTTCATATCATTGGTCGTGGTTG	0.07141622	0	0	0
tsrna-12475	GTTTTTCATATCATTGGTCGTGGTT	0.07141622	0	0	0
tsrna-12474	GTTTTTCATATCATTGGTCGTGGT	0	0	0	0
tsrna-12473	GTTTTTCATATCATTGGTCGTGG	0	0.07808	0	0.111027
tsrna-12465	GTTTTGCAGTCCTTACCA	0	0	0.069847	0
tsrna-12463	GTTTTGACAACATTCAAAAAAGAGTACCA	0	0.07808	0.069847	0
tsrna-12462	GTTTTCACCCAGGTGGCCCGGGTT	0.21424865	0.546561	0.069847	0.111027
tsrna-12461	GTTTTCACCCAGGTGGCCCGGGT	0.35708108	0.390401	0.139694	0
tsrna-12460	GTTTTCACCCAGGTGGCCCGG	0.21424865	0.07808	0.069847	0
tsrna-12459	GTTTTCACCCAGGTGGCCCG	0.35708108	0.624641	0.069847	0.111027
tsrna-12458	GTTTTCACCCAGGTGGCCC	0.21424865	0.468481	0.139694	0
tsrna-12457	GTTTTCACCCAGGTGGCC	0.4284973	0.23424	0.139694	0
tsrna-12456	GTTTTCACCCAGGCGGCCCGGGTTCGACTCCCGGTGTGGGA	5.92754596	25.9226	3.352647	2.220543
tsrna-12455	GTTTTCACCCAGGCGGCCCGGGT	0.07141622	0	0.069847	0
tsrna-12454	GTTTTCACCCAGGCGGCCCGGG	0.07141622	0.15616	0.069847	0
tsrna-12453	GTTTTCACCCAGGCGGCCCGG	0	0.31232	0	0
tsrna-12452	GTTTTCACCCAGGCGGCCCG	0.14283243	0.390401	0	0
tsrna-12451	GTTTTCACCCAGGCGGCCC	0.21424865	0.15616	0.069847	0
tsrna-12450	GTTTTCACCCAGGCGGCC	0.21424865	0	0	0
tsrna-12449	GTTTTCACCCAGGCGGC	0	0.07808	0.069847	0
tsrna-12448	GTTTTCACCCAGGCGG	0.07141622	0.23424	0.069847	0.111027
tsrna-12447	GTTTGTGGGTTTAAGTCCCATTGGTCTAGCCA	0	0.07808	0.069847	0
tsrna-12445	GTTTGTGGGTTTAAGTCCCATTGGTCTAGC	0	0	0	0
tsrna-12444	GTTTGTGGGTTTAAGTCCCATTGGTCTA	0.07141622	0	0.069847	0
tsrna-12443	GTTTGTGGGTTTAAGTCCCATTGGTCT	0	0	0.069847	0
tsrna-12442	GTTTGTGGGTTTAAGTCCCATTGGTC	0	0.07808	0	0
tsrna-12440	GTTTGTGGGTTTAAGTCCCATTGG	0	0.07808	0	0
tsrna-12436	GTTTGTGGGTTTAAGTCCCA	0	0	0	0
tsrna-12433	GTTTGTGGGTTTAAGTC	0	0	0	0
tsrna-12432	GTTTGTGGGTTTAAGT	0	0	0	0
tsrna-12431	GTTTGGGTCCGAGAGGTCCCGGGT	0	0.15616	0	0
tsrna-12426	GTTTCTGTGGTGTAGTGGTTATC	0.21424865	0.07808	0.069847	0.111027
tsrna-12425	GTTTCCGTGGTGTAGTGGTTATCACATTCGCCT	0.92841081	0.15616	0.768315	0.77719
tsrna-12424	GTTTCCGTGGTGTAGTGGTTATCACATTCG	0.71416216	0.07808	0.069847	1.221299

tsrna-12423	GTTTCCGTGGTGTAGTGGTTATCACA	0.35708108	0.31232	0.349234	0.888217
tsrna-12422	GTTTCCGTGGTGTAGTGGTTATC	0.21424865	0.468481	0.279387	0.444109
tsrna-12421	GTTTCCGTGGTGTAGTGGTTAT	0.07141622	0.07808	0.069847	0.111027
tsrna-12420	GTTTCCGTGGTGTAGTGGTT	0.07141622	0.15616	0	0
tsrna-12419	GTTTCCGTGGTGTAGTGGT	0	0.07808	0.069847	0.333081
tsrna-12418	GTTTCCGTGGTGTAGTGG	0	0.07808	0	0.111027
tsrna-12417	GTTTCCGTGGTGTAGT	0	0	0	0
tsrna-12416	GTTTCCGTAGTGTAGTGGTTATT	13.4262487	5.934088	12.92166	7.216764
tsrna-12415	GTTTCCGTAGTGTAGTGGTTATCACGTTTGCCT	7.99861624	3.591685	5.308357	5.329303
tsrna-12414	GTTTCCGTAGTGTAGTGGTTATCACGTTTGCC	8.21286489	3.747845	4.749583	8.438062
tsrna-12413	GTTTCCGTAGTGTAGTGGTTATCACGTTTG	8.21286489	3.513605	4.330502	7.993954
tsrna-12412	GTTTCCGTAGTGTAGTGGTTATCACGTTCGCCTCACACGCGA	31.7088001	11.24354	29.68489	11.99093
tsrna-12411	GTTTCCGTAGTGTAGTGGTTATCACGTTCGCCTCACACGCGA	32.8514596	14.5229	31.29137	13.21223
tsrna-12410	GTTTCCGTAGTGTAGTGGTTATCACGTTCGCCTCACACGCGA	32.1372974	11.47778	31.43106	11.99093
tsrna-12409	GTTTCCGTAGTGTAGTGGTTATCACGTTCGCCTCACACGCG	28.9949839	12.88322	29.75474	9.770388
tsrna-12408	GTTTCCGTAGTGTAGTGGTTATCACGTTCGCCTCACACGC	31.7802163	11.86818	31.01198	12.65709
tsrna-12407	GTTTCCGTAGTGTAGTGGTTATCACGTTCGCCTCACACG	29.5663136	13.3517	31.15168	10.76963
tsrna-12406	GTTTCCGTAGTGTAGTGGTTATCACGTTCGCCTCACAC	34.636865	12.41474	29.96428	11.21374
tsrna-12405	GTTTCCGTAGTGTAGTGGTTATCACGTTCGCCTCACA	29.9233947	13.19554	32.75815	13.32326
tsrna-12404	GTTTCCGTAGTGTAGTGGTTATCACGTTCGCCTCAC	31.994465	14.60098	32.05968	10.88066
tsrna-12403	GTTTCCGTAGTGTAGTGGTTATCACGTTCGCCTCA	30.2804758	14.21058	31.36122	11.54682
tsrna-12402	GTTTCCGTAGTGTAGTGGTTATCACGTTCGCCTC	30.0662271	12.72706	28.3578	10.99169
tsrna-12401	GTTTCCGTAGTGTAGTGGTTATCACGTTCGCCTAAC	31.2803028	13.19554	29.19596	11.76888
tsrna-12400	GTTTCCGTAGTGTAGTGGTTATCACGTTCGCCTAA	33.2085406	12.72706	32.40892	10.32552
tsrna-12399	GTTTCCGTAGTGTAGTGGTTATCACGTTCGCCTA	30.4233082	12.72706	32.47876	11.65785
tsrna-12398	GTTTCCGTAGTGTAGTGGTTATCACGTTCGCCT	30.4233082	12.10242	31.50091	10.76963
tsrna-12397	GTTTCCGTAGTGTAGTGGTTATCACGTTCGCC	28.5664866	12.72706	26.19255	11.10271
tsrna-12396	GTTTCCGTAGTGTAGTGGTTATCACGTTCGC	10.0696865	5.699848	7.962536	6.772655
tsrna-12395	GTTTCCGTAGTGTAGTGGTTATCACGTTCG	8.28428111	3.357445	5.308357	8.54909
tsrna-12394	GTTTCCGTAGTGTAGTGGTTATCACGTTC	7.21303786	2.810884	5.587744	8.216008
tsrna-12393	GTTTCCGTAGTGTAGTGGTTATCACGTT	8.07003246	3.357445	4.81943	9.104225
tsrna-12392	GTTTCCGTAGTGTAGTGGTTATCACGT	6.85595678	2.732804	4.81943	8.216008
tsrna-12391	GTTTCCGTAGTGTAGTGGTTATCACG	7.64153516	3.201285	5.23851	6.328547
tsrna-12390	GTTTCCGTAGTGTAGTGGTTATCAC	7.07020543	2.967044	3.841574	7.7719
tsrna-12389	GTTTCCGTAGTGTAGTGGTTATCA	7.14162164	3.435525	5.797285	8.438062
tsrna-12388	GTTTCCGTAGTGTAGTGGTTATC	7.57011894	1.952003	4.609889	6.328547
tsrna-12387	GTTTCCGTAGTGTAGTGGTTAT	1.2854919	1.873923	0.768315	0.444109
tsrna-12386	GTTTCCGTAGTGTAGTGGTTA	0.78557838	1.327362	0.838162	0.555136
tsrna-12385	GTTTCCGTAGTGTAGTGGTT	1.42832433	1.171202	0.558774	0.888217
tsrna-12384	GTTTCCGTAGTGTAGTGGTCATCACGTTCGCCTA	9.35552435	4.294406	7.613302	4.885194
tsrna-12383	GTTTCCGTAGTGTAGTGGTCATCACGTTCGCCT	9.42694057	5.543688	7.054527	5.884438
tsrna-12382	GTTTCCGTAGTGTAGTGGTCATCACGTTCGCC	6.64170813	3.123204	4.81943	4.885194
tsrna-12381	GTTTCCGTAGTGTAGTGGTCATCACGTTCGC	7.21303786	2.654724	4.330502	4.219031
tsrna-12380	GTTTCCGTAGTGTAGTGGTCATCACGTTCG	6.21321083	2.498564	3.422493	4.330058
tsrna-12379	GTTTCCGTAGTGTAGTGGTCATCACGTTC	7.14162164	3.825925	4.120962	3.996977
tsrna-12378	GTTTCCGTAGTGTAGTGGTCATCACGTT	5.42763245	3.357445	3.562187	4.66314
tsrna-12377	GTTTCCGTAGTGTAGTGGTCATCACGT	5.35621623	3.123204	3.841574	5.551357
tsrna-12376	GTTTCCGTAGTGTAGTGGTCATCACG	5.2133838	3.513605	3.771728	4.330058
tsrna-12375	GTTTCCGTAGTGTAGTGGTCATCAC	4.85630272	1.952003	4.260655	5.107248
tsrna-12374	GTTTCCGTAGTGTAGTGGTCATCA	5.42763245	2.030083	3.841574	3.219787
tsrna-12373	GTTTCCGTAGTGTAGTGGTCATC	6.14179461	1.639682	2.933566	3.663896
tsrna-12372	GTTTCCGTAGTGTAGTGGTCAT	1.57115676	1.327362	0.488928	0.999244
tsrna-12371	GTTTCCGTAGTGTAGTGGTCA	1.64257298	1.717762	0.558774	0.888217
tsrna-12370	GTTTCCGTAGTGTAGTGGTC	0.78557838	0.780801	0.768315	0.444109
tsrna-12369	GTTTCCGTAGTGTAGTGGT	1.49974054	1.561602	0.349234	0.77719
tsrna-12368	GTTTCCGTAGTGTAGTGG	0.57132973	1.405442	0.488928	0.222054
tsrna-12367	GTTTCCGTAGTGTAGTG	0.78557838	1.171202	0.628621	0.999244
tsrna-12366	GTTTCCGTAGTGTAGT	0.35708108	0.936961	0.349234	0.333081
tsrna-12365	GTTTCCGTAGTGTAGCGGTTATCACATTCGCCTCAC	15.5687352	7.33953	13.1312	1.998488
tsrna-12364	GTTTCCGTAGTGTAGCGGTTATCACATTCGCCTCA	14.3546595	5.231367	13.1312	2.997733
tsrna-12363	GTTTCCGTAGTGTAGCGGTTATCACATTCGCCTC	12.9263352	5.699848	12.64227	1.776434
tsrna-12362	GTTTCCGTAGTGTAGCGGTTATCACATTCGCCT	8.92702705	4.840967	8.800698	2.109516
tsrna-12361	GTTTCCGTAGTGTAGCGGTTATCACATTCGCC	6.99878921	3.669765	4.81943	2.220543
tsrna-12360	GTTTCCGTAGTGTAGCGGTTATCACATTCGC	1.99965406	2.888964	2.165251	1.221299

tsrna-12359	GTTTCCGTAGTGTAGCGGTTATCACATTCG	0.92841081	2.264323	0.698468	1.110271
tsrna-12358	GTTTCCGTAGTGTAGCGGTTATCACATTC	0.8569946	1.795843	1.117549	0.555136
tsrna-12357	GTTTCCGTAGTGTAGCGGTTATCACATT	1.49974054	1.483522	1.047702	1.221299
tsrna-12356	GTTTCCGTAGTGTAGCGGTTATCACAT	1.14265946	1.249282	0.488928	0.77719
tsrna-12355	GTTTCCGTAGTGTAGCGGTTATCACA	1.49974054	1.717762	0.768315	0.333081
tsrna-12354	GTTTCCGTAGTGTAGCGGTTATCAC	1.21407568	1.171202	0.977855	0.77719
tsrna-12353	GTTTCCGTAGTGTAGCGGTTATCA	0.99982703	1.405442	0.908008	0.888217
tsrna-12352	GTTTCCGTAGTGTAGCGGTTATC	0.99982703	1.093122	0.558774	0.666163
tsrna-12351	GTTTCCGTAGTGTAGCGGTTAT	0.71416216	1.327362	0.20954	0.444109
tsrna-12350	GTTTCCGTAGTGTAGCGGTTA	0.64274595	1.327362	0.768315	0.444109
tsrna-12349	GTTTCCGTAGTGTAGCGGTT	0.78557838	0.858881	0.488928	0.666163
tsrna-12348	GTTTCCGTAGTGTAGCGGT	0.4284973	0.858881	0.139694	0.333081
tsrna-12347	GTTTCCGTAGTGTAGCGG	0.49991351	1.015041	0.349234	0.444109
tsrna-12346	GTTTCCGTAGTGTAGCG	0.28566487	0.936961	0.20954	0.555136
tsrna-12345	GTTTCCGTAGTGTAGC	0.35708108	1.093122	0.139694	0
tsrna-12344	GTTTCCGTAGTGGAGTGGTTATCACGTTCGCCTC	0	0.07808	0.139694	0.111027
tsrna-12343	GTTTCCGTAGTGGAGTGGTTATCACGTTCGCCT	0	0.07808	0.069847	0.444109
tsrna-12342	GTTTCCGTAGTGGAGTGGTTATCACGTTCGCC	0	0.07808	0.069847	0
tsrna-12341	GTTTCCGTAGTGGAGTGGTTATCACGTTCGC	0.14283243	0	0	0
tsrna-12340	GTTTCCGTAGTGGAGTGGTTATCACGTTCG	0	0	0	0
tsrna-12339	GTTTCCGTAGTGGAGTGGTTATCACGTT	0.07141622	0	0	0.111027
tsrna-12338	GTTTCCGTAGTGGAGTGGTTATCACG	0.07141622	0	0	0
tsrna-12337	GTTTCCGTAGTGGAGTGGTTATCAC	0	0.07808	0.139694	0.111027
tsrna-12336	GTTTCCGTAGTGGAGTGGTTATCA	0.14283243	0	0	0
tsrna-12335	GTTTCCGTAGTGGAGTGGTTATC	0	0	0.139694	0.222054
tsrna-12333	GTTTCCGTAGTGGAGTGGTT	0	0	0.069847	0
tsrna-12322	GTTTATGTAGCTTACCTCCTCAAAGCAATACACTGAA	4.92771893	2.967044	4.120962	4.441086
tsrna-12321	GTTTATGTAGCTTACCTCCTCAAAGCAATACACTGA	5.07055137	2.967044	2.793872	3.88595
tsrna-12320	GTTTATGTAGCTTACCTCCTCAAAGCAATACACTG	4.28497299	3.123204	2.793872	3.774923
tsrna-12319	GTTTATGTAGCTTACCTCCTCAAAGCAATACACT	3.64222704	2.654724	2.095404	5.107248
tsrna-12318	GTTTATGTAGCTTACCTCCTCAAAGCAATACA	2.49956757	1.561602	1.606477	2.220543
tsrna-12317	GTTTATGTAGCTTACCTCCTCAAAGCAATA	1.78540541	1.171202	1.606477	3.219787
tsrna-12316	GTTTATGTAGCTTACCTCCTCAAAGCAAT	1.21407568	0.858881	0.768315	2.220543
tsrna-12315	GTTTATGTAGCTTACCTCCTCAAAGCAA	0.92841081	0.390401	0.698468	1.665407
tsrna-12314	GTTTATGTAGCTTACCTCCTCAAAGCA	0.57132973	0.23424	0.977855	1.887461
tsrna-12313	GTTTATGTAGCTTACCTCCTCAAAGC	0.71416216	0.23424	0.698468	2.109516
tsrna-12312	GTTTATGTAGCTTACCTCCTCAAAG	0.92841081	0.07808	0.838162	2.33157
tsrna-12311	GTTTATGTAGCTTACCTCCTCAAA	0.57132973	0.23424	1.117549	1.665407
tsrna-12310	GTTTATGTAGCTTACCTCCTCAA	0.35708108	0.07808	0.419081	1.443353
tsrna-12309	GTTTATGTAGCTTACCTCCTCA	0.8569946	0	0.488928	1.332326
tsrna-12308	GTTTATGTAGCTTACCTCCTC	0.64274595	0	0.349234	0.666163
tsrna-12307	GTTTATGTAGCTTACCTCCT	0.28566487	0	0.139694	1.110271
tsrna-12306	GTTTATGTAGCTTACCTCC	0.07141622	0.07808	0.069847	0.111027
tsrna-12305	GTTTATGTAGCTTACCTC	0.28566487	0	0.279387	0.333081
tsrna-12304	GTTTATGTAGCTTACCT	0.21424865	0	0.069847	0.111027
tsrna-12302	GTTTAGTGGTAGAATTCTCGCCT	0.21424865	0.31232	0.069847	0.111027
tsrna-12301	GTTTAGTGGTAGAATTCTCGCC	0	0.390401	0	0
tsrna-12300	GTTTAGTGGTAGAATTCTCGC	0	0	0	0
tsrna-12297	GTTTAGTGGTAGAATTCT	0	0	0	0
tsrna-12295	GTTTAGACGGGCTCACATCACCCCATAAACACCA	5.42763245	0.858881	3.981268	1.665407
tsrna-12294	GTTTAGACGGGCTCACATCACCCCATAAACACC	1.14265946	0.390401	1.187396	0.666163
tsrna-12293	GTTTAGACGGGCTCACATCACCCCATAAACA	0.64274595	0.31232	1.257243	0.222054
tsrna-12292	GTTTAGACGGGCTCACATCACCCCATAAAC	0.71416216	0.31232	1.257243	0.111027
tsrna-12291	GTTTAGACGGGCTCACATCACCCCATAAA	0.99982703	0.546561	1.327089	0
tsrna-12290	GTTTAGACGGGCTCACATCACCCCATAA	0.57132973	0.390401	0.768315	0
tsrna-12289	GTTTAGACGGGCTCACATCACCCCATA	0.57132973	0.31232	1.187396	0.111027
tsrna-12288	GTTTAGACGGGCTCACATCACCCCAT	0.57132973	0.31232	1.117549	0.222054
tsrna-12287	GTTTAGACGGGCTCACATCACCCCA	0.64274595	0.15616	0.977855	0.222054
tsrna-12286	GTTTAGACGGGCTCACATCACCCC	0.92841081	0.07808	0.558774	0.222054
tsrna-12285	GTTTAGACGGGCTCACATCACCC	0.57132973	0.23424	0.558774	0
tsrna-12284	GTTTAGACGGGCTCACATCACC	0.57132973	0.31232	0.558774	0
tsrna-12283	GTTTAGACGGGCTCACATCAC	0.57132973	0.15616	0.488928	0.111027
tsrna-12282	GTTTAGACGGGCTCACATCA	0.64274595	0.23424	0.419081	0.111027
tsrna-12281	GTTTAGACGGGCTCACATC	0.28566487	0.07808	0.419081	0

tsrna-12280	GTTTAGACGGGCTCACAT	0.28566487	0.07808	0.558774	0
tsrna-12279	GTTTAGACGGGCTCACA	0.49991351	0.23424	0.279387	0
tsrna-12278	GTTTAGACGGGCTCAC	0.07141622	0.31232	0.20954	0
tsrna-12277	GTTTAAGTCCCATTGGTCTAGCCA	0	0.15616	0.20954	0
tsrna-12276	GTTTAAGTCCCATTGGTCTAGCC	0	0.07808	0	0
tsrna-12275	GTTTAAGTCCCATTGGTCTAGC	0	0	0	0
tsrna-12272	GTTTAAGTCCCATTGGTCT	0	0	0	0
tsrna-12268	GTTTAACCAAAACATCAGATTGTGAATCTGACAACAGAGGCT	0.21424865	0.624641	0.069847	0
tsrna-12260	GTTTAAATTAGAATCTTAGCTTTGGGTGCT	0.92841081	0.390401	0.628621	0.222054
tsrna-12259	GTTTAAATTAGAATCTTAGCTTTGGGTGC	0.64274595	0.07808	0.349234	0
tsrna-12258	GTTTAAATTAGAATCTTAGCTTTGGGTG	0.4284973	0.15616	0.419081	0.333081
tsrna-12257	GTTTAAATTAGAATCTTAGCTTTGG	0.35708108	0.23424	0.628621	0.333081
tsrna-12256	GTTTAAATTAGAATCTTAGCTTTG	0.49991351	0	0.139694	0
tsrna-12255	GTTTAAATTAGAATCTTAGCTTT	0.14283243	0	0.279387	0.111027
tsrna-12254	GTTTAAATTAGAATCTTAGCT	0.21424865	0.23424	0.069847	0
tsrna-12253	GTTTAAATTAGAATCTTAGC	0.14283243	0	0	0
tsrna-12249	GTTGTGGCCGCAGCAACCTCGGT	0	0.15616	0	0.111027
tsrna-12246	GTTGTGGCCGCAGCAACCTC	0	0	0	0.111027
tsrna-12244	GTTGTCTAGTGGCTAGGATTCGG	0	0.07808	0	0
tsrna-12243	GTTGTAGTCCGTGCGAGAATACCA	0.07141622	0.15616	0	0.111027
tsrna-12242	GTTGTAGTCCGTGCGAGAATACC	0.07141622	0.07808	0	0
tsrna-12241	GTTGTAGTCCGTGCGAGAATAC	0	0	0	0
tsrna-12240	GTTGTAGTCCGTGCGAGAATA	0	0	0.069847	0.111027
tsrna-12239	GTTGTAGTCCGTGCGAGAAT	0	0	0	0
tsrna-12238	GTTGTAGTCCGTGCGAGAA	0	0	0	0
tsrna-12234	GTTGGTTATACCCTTCCCGTACTACCA	0.28566487	0	0	0.111027
tsrna-12233	GTTGGTTATACCCTTCCCGTACTACC	0	0.15616	0	0.111027
tsrna-12231	GTTGGTTATACCCTTCCCGTACTA	0	0	0	0
tsrna-12230	GTTGGTTATACCCTTCCCGTACT	0.07141622	0	0	0
tsrna-12220	GTTGGTGGTTCGAGCCCACCCAGGGACGCC	0.07141622	0.468481	0.069847	0
tsrna-12219	GTTGGTGGTATAGTGGTTAGCATAGCTGCC	0	0.15616	0	0
tsrna-12218	GTTGGTGGTATAGTGGTTAGCATAGCTGC	0.14283243	0	0	0
tsrna-12217	GTTGGTGGTATAGTGGTTAGCATAGCTG	0.07141622	0.07808	0	0
tsrna-12212	GTTGGTGGTATAGTGGTTAGCAT	0	0	0	0
tsrna-12208	GTTGGTGGTATAGTGGTGAGCATAGCTGCC	0.14283243	0	0	0
tsrna-12207	GTTGGTGGTATAGTGGTGAGCATAGCTGC	0.07141622	0	0.139694	0
tsrna-12206	GTTGGTGGTATAGTGGTGAGCATAGCTG	0	0.15616	0	0.111027
tsrna-12205	GTTGGTGGTATAGTGGTGAGCATAGCT	0	0.15616	0	0
tsrna-12204	GTTGGTGGTATAGTGGTGAGCATAGC	0	0.07808	0	0
tsrna-12203	GTTGGTGGTATAGTGGTGAGCATAG	0	0	0.069847	0
tsrna-12202	GTTGGTGGTATAGTGGTGAGCATA	0	0	0	0
tsrna-12201	GTTGGTGGTATAGTGGTGAGCAT	0	0.07808	0	0
tsrna-12200	GTTGGTGGTATAGTGGTGAGCA	0	0.15616	0	0
tsrna-12199	GTTGGTGGTATAGTGGTGAGC	0	0	0.069847	0
tsrna-12197	GTTGGTGGTATAGTGGTGA	0	0	0	0
tsrna-12195	GTTGGTGGTATAGTGGTAAGCATAGCTGC	0	0.15616	0	0.111027
tsrna-12187	GTTGGTCTAGGGGTATGATTCTCGGTT	0.28566487	0.07808	0.139694	0.111027
tsrna-12186	GTTGGTCTAGGGGTATGATTCTCGGT	0.49991351	0.390401	0.139694	0.333081
tsrna-12185	GTTGGTCTAGGGGTATGATTCTCGCTTT	1.78540541	1.327362	0.977855	1.443353
tsrna-12184	GTTGGTCTAGGGGTATGATTCTCGCTT	1.78540541	1.015041	0.838162	1.221299
tsrna-12183	GTTGGTCTAGGGGTATGATTCTCGCT	1.2854919	1.561602	0.698468	3.330814
tsrna-12182	GTTGGTCTAGGGGTATGATTCTCGC	0.92841081	0.702721	0.349234	1.332326
tsrna-12181	GTTGGTCTAGGGGTATGATTCT	0.07141622	0	0.069847	0
tsrna-12167	GTTGGGAGAGCGTTAGACTGAAGATC	0	0.15616	0	0
tsrna-12166	GTTGGGAGAGCGTTAGACTGAAGAT	0	0.07808	0	0
tsrna-12164	GTTGGGAGAGCGTTAGACTGAAG	0	0	0	0.111027
tsrna-12159	GTTGGCTTGAAACCAGCTTTGGGG	0	0	0	0
tsrna-12157	GTTGCAATACTTAATTTCTGCCA	0	0	0	0.111027
tsrna-12156	GTTGCAATACTTAATTTCTGCC	0	0	0	0
tsrna-12154	GTTGCAATACTTAATTTCTG	0.07141622	0	0	0
tsrna-12150	GTTGATTAGGGTGCTTAGCTGTTAACTAAGTGTTTGTGGGTTT	0.21424865	0.624641	0.838162	0.111027
tsrna-12149	GTTGATTAGGGTGCTTAGCTGTTAACTAAGTGTTTG	0.35708108	0.23424	0.349234	0
tsrna-12148	GTTGATTAGGGTGCTTAGCTGTTAACTAAG	0.4284973	0.07808	0.628621	0.111027
tsrna-12147	GTTGATTAGGGTGCTTAGCTGTTAACTA	0.21424865	0.07808	0.698468	0

tsrna-12146	GTTGATTAGGGTGCTTAGCTGTTAACT	0.35708108	0.07808	0.558774	0.222054
tsrna-12145	GTTGATTAGGGTGCTTAGCTGTTAAC	0	0.07808	0.139694	0
tsrna-12144	GTTGATTAGGGTGCTTAGCTGTTAA	0.07141622	0.15616	0.139694	0
tsrna-12143	GTTGATTAGGGTGCTTAGCTGTTA	0.07141622	0	0.139694	0
tsrna-12142	GTTGATTAGGGTGCTTAGCTGTT	0	0	0	0
tsrna-12141	GTTGATTAGGGTGCTTAGCTGT	0	0.15616	0.069847	0
tsrna-12140	GTTGATTAGGGTGCTTAGCTG	0	0	0.069847	0
tsrna-12139	GTTGATTAGGGTGCTTAGCT	0	0	0	0
tsrna-12138	GTTGATTAGGGTGCTTAGC	0	0.07808	0.069847	0
tsrna-12134	GTTGATGCAGAGTGGGGTTTTGCAGTCCTT	0.07141622	0.15616	0	0.111027
tsrna-12133	GTTGATGCAGAGTGGGGTTTTGCAGTCCT	0	0	0.069847	0.111027
tsrna-12132	GTTGATGCAGAGTGGGGTTTTGCAGTCC	0.07141622	0.07808	0.069847	0.111027
tsrna-12131	GTTGATGCAGAGTGGGGTTTTGCAGTC	0	0	0.20954	0
tsrna-12130	GTTGATGCAGAGTGGGGTTTTGCAGT	0	0	0	0
tsrna-12129	GTTGATGCAGAGTGGGGTTTTGCAG	0	0.07808	0	0
tsrna-12128	GTTGATGCAGAGTGGGGTTTTGCA	0	0.07808	0	0
tsrna-12127	GTTGATGCAGAGTGGGGTTTTGC	0	0	0.069847	0
tsrna-12126	GTTGATGCAGAGTGGGGTTTTG	0.07141622	0	0	0
tsrna-12125	GTTGATGCAGAGTGGGGTTTT	0	0	0	0
tsrna-12124	GTTGATGCAGAGTGGGGTTT	0	0	0	0
tsrna-12123	GTTGATGCAGAGTGGGGTT	0	0	0	0
tsrna-12122	GTTGATGCAGAGTGGGGT	0	0	0	0
tsrna-12119	GTTGACCCGGGTTCGATTCCCGGC	0	0	0	0
tsrna-12117	GTTGAAATACAACGATGGTTTTTCATATCATTGGTCGTGGTTG	0	0.15616	0.069847	0.111027
tsrna-12116	GTTGAAATACAACGATGGTTTTTCATATCATTGGTC	0	0.07808	0.069847	0.111027
tsrna-12115	GTTGAAATACAACGATGGTTTTTCATATC	0	0	0.069847	0
tsrna-12113	GTTGAAATACAACGATGGTTTTTCA	0.07141622	0	0	0
tsrna-12110	GTTCTTGTAGTTGAAATACAACGATGGTTTTTCATATCATTGG	0.28566487	2.186243	0.20954	0.333081
tsrna-12109	GTTCTTGTAGTTGAAATACAACGATGGTTTTTCATATCA	0.78557838	1.249282	0.419081	0.77719
tsrna-12108	GTTCTTGTAGTTGAAATACAACGATGGTTTTTCATATC	0.57132973	1.795843	0.20954	0.222054
tsrna-12107	GTTCTTGTAGTTGAAATACAACGATGGTTTTTCATA	0.35708108	1.795843	0.139694	0.222054
tsrna-12106	GTTCTTGTAGTTGAAATACAACGATGGTTTTTCAT	0.64274595	1.405442	0.279387	0.222054
tsrna-12105	GTTCTTGTAGTTGAAATACAACGATGGTTTTTCA	0.4284973	1.327362	0.419081	0.444109
tsrna-12104	GTTCTTGTAGTTGAAATACAACGATGGTTTTTC	0.28566487	1.717762	0.20954	0
tsrna-12103	GTTCTTGTAGTTGAAATACAACGATGGTTT	0.4284973	1.483522	0.279387	0
tsrna-12102	GTTCTTGTAGTTGAAATACAACGATGGTT	0.07141622	1.405442	0.139694	0
tsrna-12101	GTTCTTGTAGTTGAAATACAACGATGGT	0.35708108	1.639682	0.279387	0
tsrna-12100	GTTCTTGTAGTTGAAATACAACGATGG	0.07141622	0.780801	0.069847	0.111027
tsrna-12099	GTTCTTGTAGTTGAAATACAACGATG	0.14283243	1.249282	0.20954	0.222054
tsrna-12098	GTTCTTGTAGTTGAAATACAACGAT	0.14283243	1.171202	0	0.333081
tsrna-12097	GTTCTTGTAGTTGAAATACAACGA	0.21424865	1.093122	0.069847	0.222054
tsrna-12096	GTTCTTGTAGTTGAAATACAACG	0.35708108	0.624641	0.069847	0.222054
tsrna-12095	GTTCTTGTAGTTGAAATACAAC	0.35708108	0.23424	0	0.222054
tsrna-12094	GTTCTTGTAGTTGAAATACAA	0.14283243	0.07808	0.069847	0
tsrna-12093	GTTCTTGTAGTTGAAATACA	0.35708108	0.31232	0.069847	0.222054
tsrna-12092	GTTCTTGTAGTTGAAATAC	0.14283243	0.07808	0	0.111027
tsrna-12091	GTTCTTGTAGTTGAAATA	0.21424865	0.07808	0.069847	0
tsrna-12090	GTTCTTGTAGTTGAAAT	0	0	0	0
tsrna-12088	GTTCTGGTCTCCGGATGGAGGCGTGGGTTC	0.71416216	2.654724	0.419081	0
tsrna-12087	GTTCTGGTCTCCGGATGGAGGCGT	0.07141622	0.07808	0	0
tsrna-12082	GTTCTGGTCTCCAATGGAGGCGTG	0.07141622	0	0	0
tsrna-12081	GTTCGATTCTCATAGTCCTAGCCA	0	0.15616	0	0.111027
tsrna-12080	GTTCGATTCTCATAGTCCTAGCC	0.07141622	0.07808	0	0.111027
tsrna-12079	GTTCGATTCTCATAGTCCTAGC	0	0.07808	0.069847	0
tsrna-12078	GTTCGATTCTCATAGTCCTAG	0.07141622	0.07808	0	0
tsrna-12077	GTTCGATTCTCATAGTCCTA	0	0.07808	0.069847	0
tsrna-12076	GTTCGATTCTCATAGTCCT	0.07141622	0.07808	0	0
tsrna-12075	GTTCGATTCTCATAGTCC	0	0	0	0.111027
tsrna-12072	GTTCGATTCCTTCCTTTTTTGCCA	0	0	0.069847	0.111027
tsrna-12071	GTTCGATTCCTTCCTTTTTTGCC	0	0	0.069847	0
tsrna-12069	GTTCGATTCCTTCCTTTTTTG	0	0.07808	0	0
tsrna-12068	GTTCGATTCCTTCCTTTTTT	0	0.07808	0	0
tsrna-12063	GTTCGATTCCTGGTCAGGGAACCA	0.07141622	2.420483	0	0.111027
tsrna-12062	GTTCGATTCCTGGTCAGGG	0.07141622	0	0	0

tsrna-12061	GTTCGATTCCGGCTCGAAGGACCA	5.28480002	14.28866	1.74617	3.10876
tsrna-12060	GTTCGATTCCGGCTCGAAGGACC	0.07141622	0.624641	0.069847	0
tsrna-12057	GTTCGATTCCGGCTCGAAGG	0.07141622	0	0	0
tsrna-12054	GTTCGATTCCCGGTCATGGAACCA	0	0.15616	0	0
tsrna-12053	GTTCGATTCCCGGTCATGGAAC	0	0.07808	0	0
tsrna-12051	GTTCGATTCCCGGTCAGGGAACCA	0.92841081	21.47203	0.279387	0.222054
tsrna-12050	GTTCGATTCCCGGTCAGGGAACC	0	0.390401	0	0
tsrna-12049	GTTCGATTCCCGGTCAGGGAAC	0	0.31232	0	0
tsrna-12048	GTTCGATTCCCGGTCAGGGAA	0	0.23424	0	0
tsrna-12047	GTTCGATTCCCGGTCAGGGA	0	0.23424	0	0
tsrna-12045	GTTCGATTCCCGGTCAGGAAACCA	0.07141622	0.858881	0	0
tsrna-12044	GTTCGATTCCCGGTCAGGA	0	0.15616	0	0
tsrna-12040	GTTCGATTCCCGGGCGGCGCACCA	1.49974054	4.684807	0.139694	0.222054
tsrna-12039	GTTCGATTCCCGGGCGGCGCACC	0.21424865	0.936961	0.069847	0.111027
tsrna-12037	GTTCGATTCCCGGGCGGCGCA	0	0	0	0
tsrna-12031	GTTCGATTCCCGGCCCATGCACCA	1.57115676	2.888964	0.628621	1.55438
tsrna-12030	GTTCGATTCCCGGCCCATGCACC	0.14283243	0.390401	0.069847	0.222054
tsrna-12023	GTTCGATTCCCGGCCAGGGAACCA	0	0.858881	0	0
tsrna-12022	GTTCGATTCCCGGCCAGGGAACC	0	0.07808	0	0
tsrna-12017	GTTCGATTCCCGGCCAATGCACCA	2.64240001	9.681934	0.908008	0.77719
tsrna-12016	GTTCGATTCCCGGCCAATGCACC	0.57132973	0.936961	0	0
tsrna-12013	GTTCGATTCCCGGCCAATGC	0	0	0	0
tsrna-12010	GTTCGATTCCCGGCCAACGCACCA	2.92806487	7.651851	1.257243	1.443353
tsrna-12009	GTTCGATTCCCGGCCAACGCACC	0.57132973	2.030083	0.139694	0
tsrna-12008	GTTCGATTCCCGGCCAACGCAC	0	0.07808	0	0
tsrna-12007	GTTCGATTCCCGGCCAACGCA	0	0.07808	0	0
tsrna-12005	GTTCGATTCCCGGCCAACG	0	0.07808	0	0
tsrna-12001	GTTCGATTCCCCGACGGGGAGCCA	0.14283243	1.327362	0	0
tsrna-12000	GTTCGATTCCCCGACGGGGAGCC	0	0.07808	0	0
tsrna-11997	GTTCGATTCCCCGACGGGGA	0	0.07808	0	0
tsrna-11984	GTTCGATCCTCGCTGGGGCCTCCA	0.4284973	0.936961	0	0.444109
tsrna-11983	GTTCGATCCTCGCTGGGGCCTC	0	0	0	0
tsrna-11978	GTTCGATCCTCACCTGGAGCACCA	0.07141622	0.23424	0	0
tsrna-11977	GTTCGATCCTCACCTGGAGCACC	0.14283243	0	0	0
tsrna-11974	GTTCGATCCTCACCTGGAGC	0	0.15616	0	0
tsrna-11971	GTTCGATCCTCACACGGGGCACCA	0	0.31232	0	0
tsrna-11964	GTTCGATCCCGGGTTTCGGCACCA	1.85682163	7.573771	0.977855	1.776434
tsrna-11963	GTTCGATCCCGGGTTTCGGCACC	0.35708108	0.390401	0.069847	0.111027
tsrna-11962	GTTCGATCCCGGGTTTCGGCAC	0	0.07808	0	0
tsrna-11955	GTTCGATCCCGGGCGGAAACACCA	1.99965406	3.123204	0.488928	1.110271
tsrna-11953	GTTCGATCCCGGGCGGA	0	0	0.069847	0
tsrna-11952	GTTCGATCCCCGTACTGGCCACCA	0.21424865	0.31232	0.488928	0.111027
tsrna-11951	GTTCGATCCCCGTACGGGCCACCA	0.35708108	4.528646	0.279387	0.333081
tsrna-11950	GTTCGATCCCCGTACGGGCCACC	0	0.31232	0	0
tsrna-11944	GTTCGATCCCCGGCATCTCCACCA	16.8542271	29.74852	9.359472	8.104981
tsrna-11943	GTTCGATCCCCGGCATCTCCACC	0.78557838	1.327362	0.419081	0.222054
tsrna-11942	GTTCGATCCCCGGCATCTCCAC	0	0	0	0
tsrna-11940	GTTCGATCCCCGGCATCTCC	0	0.07808	0	0
tsrna-11935	GTTCGATCCCCGGCACCTCCACCA	12.2835892	25.84452	5.867132	4.330058
tsrna-11934	GTTCGATCCCCGGCACCTCCACC	0.92841081	0.936961	0.279387	0
tsrna-11933	GTTCGATCCCCGGCACCTCCAC	0	0.07808	0	0
tsrna-11932	GTTCGATCCCCGGCACCTCCA	0	0	0	0.222054
tsrna-11931	GTTCGATCCCCGGCACCTCC	0	0.07808	0	0
tsrna-11930	GTTCGATCCCCGGCACCTC	0.07141622	0.07808	0	0
tsrna-11929	GTTCGATCCCCGACACCTCCACCA	0.99982703	2.264323	0	0.222054
tsrna-11928	GTTCGATCCCCGACACCTCCACC	0	0.31232	0.069847	0
tsrna-11927	GTTCGATCCCCAGTACCTCCACCA	2.71381622	5.777928	0.838162	1.110271
tsrna-11926	GTTCGATCCCCAGTACCTCCACC	0.14283243	0.390401	0	0
tsrna-11925	GTTCGATCCCCAGTACCTCCAC	0	0.07808	0	0
tsrna-11918	GTTCGATCCCCAGCATCTCCACCA	0.57132973	1.561602	0.349234	0.444109
tsrna-11917	GTTCGATCCCCAGCATCTCCACC	0	0.07808	0	0
tsrna-11910	GTTCGATCCCACCCAGGGACGCCA	0.07141622	0.468481	0	0
tsrna-11908	GTTCGAGTCTCGGTGGAACCTCCA	2.85664866	10.61889	0.768315	2.664651
tsrna-11907	GTTCGAGTCTCGGTGGAACCTCC	0.35708108	0.936961	0.139694	0.444109

tsrna-11906	GTTCGAGTCTCGGTGGAACCTC	0.07141622	0.23424	0	0.111027
tsrna-11905	GTTCGAGTCTCGGTGGAACCT	0	0.07808	0	0
tsrna-11902	GTTCGAGTCCTGCCGCGGTCGCCA	0.21424865	0.31232	0	0
tsrna-11895	GTTCGAGTCCCTTCGTGGTCGCCA	0	0.15616	0	0
tsrna-11887	GTTCGAGTCCCGGCGGAGTCGCCA	0.07141622	0.07808	0	0
tsrna-11880	GTTCGAGTCCCATCTGGGTCGCCA	0.14283243	0.624641	0	0.111027
tsrna-11879	GTTCGAGTCCCATCTGGGTCGCC	0	0.15616	0	0
tsrna-11875	GTTCGAGTCCCATCTGGGGTGCCA	0.07141622	0.624641	0	0
tsrna-11874	GTTCGAGTCCCATCTGGGGTGCC	0	0.07808	0	0
tsrna-11867	GTTCGAGTCCCACCTGGGGTGCCA	0.07141622	0.390401	0	0
tsrna-11865	GTTCGAGTCCCACCTGGGGTACCA	0	0.31232	0	0
tsrna-11859	GTTCGAGTCCCACCCGGGGTACCA	0	0.07808	0	0
tsrna-11855	GTTCGAGTCCCACCAGAGTCGCCA	0	0.23424	0.069847	0
tsrna-11851	GTTCGAGCCTCAGAGAGGGCACCA	0	0	0	0
tsrna-11849	GTTCGAGCCTCACCTGGAGCACC	0.07141622	0	0	0
tsrna-11846	GTTCGAGCCTCACCTGGAGC	0	0.07808	0	0
tsrna-11835	GTTCGAGCCCCAGTGGAACCACCA	2.14248649	1.952003	2.095404	2.109516
tsrna-11834	GTTCGAGCCCCAGTGGAACCACC	0.07141622	0.15616	0	0
tsrna-11833	GTTCGAGCCCCAGTGGAACCAC	0	0	0.069847	0
tsrna-11832	GTTCGAGCCCCAGTGGAACCA	0.07141622	0	0	0.111027
tsrna-11831	GTTCGAGCCCCAGTGGAACC	0	0.07808	0	0
tsrna-11829	GTTCGAGCCCCAGTGGAA	0	0	0.069847	0
tsrna-11826	GTTCGAGCCCCACGTTGGGCGCCA	0.71416216	14.9133	0.20954	0.555136
tsrna-11825	GTTCGAGCCCCACGTTGGGCGCC	0.14283243	0.780801	0	0
tsrna-11818	GTTCGAGCCCACCCAGGGACGCCA	0.4284973	7.33953	0.069847	0
tsrna-11817	GTTCGAGCCCACCCAGGGACGCC	0.14283243	0.780801	0	0
tsrna-11816	GTTCGAGCCCACCCAGGGACGC	0	0.07808	0	0
tsrna-11809	GTTCGACTCCTGGCTGGCTCGCCA	3.28514596	7.495691	0.908008	0.666163
tsrna-11808	GTTCGACTCCTGGCTGGCTCGCC	0.14283243	0.546561	0.069847	0
tsrna-11807	GTTCGACTCCTGGCTGGCTCGC	0	0.07808	0	0
tsrna-11804	GTTCGACTCCTGGCTGGCT	0	0	0	0
tsrna-11801	GTTCGACTCCCGGTGTGGGAACCA	4.71347028	22.79939	2.304945	1.776434
tsrna-11800	GTTCGACTCCCGGTGTGGGAACC	0.14283243	0.624641	0	0.111027
tsrna-11799	GTTCGACTCCCGGTGTGGGAAC	0	0.15616	0	0
tsrna-11795	GTTCGACTCCCGGTGTGG	0	0.07808	0	0
tsrna-11794	GTTCGACTCCCGGTGTG	0.07141622	0	0	0
tsrna-11792	GTTCGACTCCCGGTATGGGAACCA	0.57132973	5.231367	0.279387	0.444109
tsrna-11791	GTTCGACTCCCGGTATGGGAACC	0.07141622	0.31232	0	0
tsrna-11790	GTTCGACTCCCGGTATGGGAAC	0.07141622	0.31232	0	0
tsrna-11788	GTTCGACTCCCGGTATGGGA	0	0.07808	0	0
tsrna-11783	GTTCGACTCCCAGCGGGGCCTCCA	0.21424865	0	0	0
tsrna-11782	GTTCGACTCCCAGCGGGGCCTCC	0	0.07808	0	0
tsrna-11781	GTTCGACTCCCAGCGGGGC	0	0	0.069847	0
tsrna-11780	GTTCGACTCCCAGCGGGG	0	0.15616	0	0
tsrna-11779	GTTCGACTCCCAGCGG	0	0.15616	0.069847	0
tsrna-11778	GTTCGACCCCCGGCTCCTCCACCA	1.49974054	1.171202	0.349234	0.444109
tsrna-11774	GTTCGAATCTCGGTGGGACCTCCA	4.28497299	8.354572	2.165251	1.776434
tsrna-11772	GTTCGAATCCTGTTCGTGACGCCA	0.4284973	0.702721	0.20954	0.555136
tsrna-11771	GTTCGAATCCTGTTCGTGACG	0	0.07808	0	0
tsrna-11770	GTTCGAATCCTGTCGGCTACGCCA	0.49991351	1.249282	0.349234	1.110271
tsrna-11767	GTTCGAATCCTGCTCACAGCGCCA	0.78557838	1.483522	0.20954	0.111027
tsrna-11766	GTTCGAATCCTGCTCACAGCGCC	0.14283243	0.07808	0	0
tsrna-11763	GTTCGAATCCTGCTCACAGC	0	0	0	0
tsrna-11762	GTTCGAATCCTGCCGACTACGCCA	7.14162164	18.11459	1.885864	2.442597
tsrna-11761	GTTCGAATCCTGCCGACTACGCC	0.49991351	1.015041	0.20954	0.333081
tsrna-11754	GTTCGAATCCGGCTCGGAGGACCA	0.14283243	0.15616	0	0
tsrna-11749	GTTCGAATCCGGCTCGAAGGACCA	1.64257298	2.810884	0.908008	0.555136
tsrna-11748	GTTCGAATCCGGCTCGAAGGACC	0.14283243	0.546561	0.069847	0.111027
tsrna-11742	GTTCGAATCCGAGTCACGGCACCA	0	0.07808	0	0
tsrna-11733	GTTCGAATCCCTTCGTGGTTGCCA	0	0.858881	0.069847	0
tsrna-11730	GTTCGAATCCCTTCGTGGTTG	0	0.07808	0	0
tsrna-11728	GTTCGAATCCCTCCGTGGTTACCA	0.21424865	0.31232	0.139694	0
tsrna-11725	GTTCGAATCCCATCCTCGTCGCCA	0.49991351	1.015041	0.419081	0.111027
tsrna-11724	GTTCGAATCCCATCCTCGTCGCC	0.07141622	0	0	0

tsrna-11717	GTTCGAATCCCAGTAGAGCCTCCA	1.49974054	3.201285	0.977855	1.110271
tsrna-11716	GTTCGAATCCCAGCGGTGCCTCCA	1.78540541	4.684807	0.908008	0.333081
tsrna-11715	GTTCGAATCCCAGCGGTGCCTCC	0	0.15616	0	0
tsrna-11709	GTTCGAATCCCAGCGGGGCCTCCA	0	0.31232	0	0
tsrna-11700	GTTCGAATCCCAGCGAGGCCTCCA	0	0.15616	0	0
tsrna-11699	GTTCGAATCCCACTTCTGACACCA	1.07124325	2.810884	0.838162	0.888217
tsrna-11698	GTTCGAATCCCACTTCTGACACC	0.07141622	0.468481	0	0.111027
tsrna-11697	GTTCGAATCCCACTTCTGACAC	0	0	0	0
tsrna-11691	GTTCGAATCCCACTCCTGACACCA	7.57011894	8.432652	3.701881	2.775678
tsrna-11690	GTTCGAATCCCACTCCTGACACC	0.57132973	0.780801	0.20954	0
tsrna-11689	GTTCGAATCCCACTCCTGACAC	0.07141622	0	0.139694	0.111027
tsrna-11688	GTTCGAATCCCACTCCTGACA	0	0	0	0
tsrna-11687	GTTCGAATCCCACTCCTGAC	0.07141622	0	0	0
tsrna-11682	GTTCGAATCCCACCTTCGTCGCCA	0.07141622	0.390401	0	0
tsrna-11681	GTTCGAATCCCACCGCTGCCACCA	0.64274595	2.888964	0.349234	0.333081
tsrna-11680	GTTCGAATCCCACCGCTGCCACC	0	0.15616	0	0
tsrna-11679	GTTCGAATCCCACCGCTGCCAC	0	0	0	0
tsrna-11677	GTTCGAATCCCACCGCTGCC	0	0	0	0.222054
tsrna-11672	GTTCGAATCCCACCCTCGTCGCCA	0.4284973	1.015041	0.069847	0
tsrna-11670	GTTCGAATCCCACCAGAGTCGCCA	0.07141622	0.468481	0	0.111027
tsrna-11668	GTTCGAATCCCACCACTGCCACCA	0.28566487	1.952003	0.069847	0
tsrna-11667	GTTCGAATCACGTCGGGGTCACCA	0.14283243	0.546561	0.139694	0.111027
tsrna-11666	GTTCGAATCACGTCGGGGTCACC	0	0	0	0
tsrna-11658	GTTCGAACCTCAGAGGGGGCACCA	0.14283243	0.546561	0	0
tsrna-11653	GTTCGAACCCTGCTCGCTGCGCCA	0.78557838	2.108163	0.20954	0.444109
tsrna-11652	GTTCGAACCCTGCTCGCTGCGCC	0	0.23424	0.069847	0
tsrna-11646	GTTCGAACCCCGTCCGTGCCTCCA	0.28566487	0.702721	0.139694	0.111027
tsrna-11645	GTTCGAACCCCGTCCGTGCCTCC	0	0.07808	0	0
tsrna-11644	GTTCGAACCCCGTCCGTGCCTC	0	0	0	0
tsrna-11638	GTTCGAACCCCGTACGGGCCACCA	0.07141622	0.23424	0.20954	0
tsrna-11637	GTTCGAACCCCACTCCTGGTACCA	0	0.07808	0	0
tsrna-11630	GTTCGAAACCGGGCGGAAACACCA	21.9961947	55.3588	7.822842	4.996221
tsrna-11629	GTTCGAAACCGGGCGGAAACACC	4.71347028	14.67906	2.025557	0.444109
tsrna-11628	GTTCGAAACCGGGCGGAAACAC	0.14283243	0	0.069847	0
tsrna-11627	GTTCGAAACCGGGCGGAAACA	0	0.07808	0	0
tsrna-11626	GTTCGAAACCGGGCGGAAAC	0	0.07808	0	0
tsrna-11622	GTTCGAAACCGGGCAGAAGCACCA	2.42815136	10.61889	1.257243	0.333081
tsrna-11617	GTTCCATGGTGTAATGGTTAGCACTCTGGACTCTGA	0	0.15616	0	0.222054
tsrna-11616	GTTCCATGGTGTAATGGTTAGCACTCTGGACT	0.07141622	0.15616	0	0.444109
tsrna-11615	GTTCCATGGTGTAATGGTTAGCACTCTGGA	0.07141622	0.07808	0	0.111027
tsrna-11614	GTTCCATGGTGTAATGGTTAGCACTCTGG	0	0.07808	0.069847	0
tsrna-11610	GTTCCATGGTGTAATGGTTAGC	0	0	0	0
tsrna-11607	GTTCCATGGTGTAATGGTGAGCACTCTG	0.07141622	0	0	0
tsrna-11605	GTTCCATGGTGTAATGGT	0	0.07808	0	0
tsrna-11603	GTTCCATAGTGTAGTGGTTATCACGTCTGCTTT	0.21424865	0	0.20954	0
tsrna-11602	GTTCCATAGTGTAGTGGTTATCACGTCTGCTT	0.14283243	0	0.139694	0.111027
tsrna-11601	GTTCCATAGTGTAGTGGTTATCACGTCTGCT	0.07141622	0.07808	0	0
tsrna-11600	GTTCCATAGTGTAGTGGTTATCACGTCTGC	0.14283243	0.07808	0.069847	0
tsrna-11599	GTTCCATAGTGTAGTGGTTATCACGTCTG	0.07141622	0	0	0
tsrna-11597	GTTCCATAGTGTAGTGGTTATCACGTC	0.07141622	0	0	0
tsrna-11594	GTTCCATAGTGTAGTGGTTATCACATCTGC	0.07141622	0	0	0.111027
tsrna-11592	GTTCCATAGTGTAGTGGTTATCAC	0.21424865	0	0.069847	0
tsrna-11587	GTTCCATAGTGTAGTGG	0	0	0	0
tsrna-11585	GTTCCATAGTGTAGCGGTTATCACGTCTG	0	0.07808	0	0
tsrna-11583	GTTCCATAGTGTAGCGGTTATCACGTC	0	0	0.069847	0
tsrna-11582	GTTCCATAGTGTAGCGGTTATC	0	0.07808	0.069847	0
tsrna-11581	GTTCCATAGTGTAGCGGTT	0	0.07808	0	0
tsrna-11579	GTTCCATAGTGTAGCGG	0	0	0	0
tsrna-11578	GTTCCATAGTGTAGCG	0	0	0	0
tsrna-11576	GTTCAGTTGATGCAGAGTGGGGTTTTG	0	0	0	0
tsrna-11575	GTTCAGTTGATGCAGAGTGGGGTTTT	0.07141622	0	0	0
tsrna-11572	GTTCAGTGGTAGAATTCTTGCCTG	0	0.07808	0	0
tsrna-11570	GTTCAGTGGTAGAATTCTTGCC	0	0.07808	0	0
tsrna-11569	GTTCAGTGGTAGAATTCTTGC	0.07141622	0	0	0

tsrna-11568	GTTCAGTGGTAGAATTCTT	0	0	0	0
tsrna-11567	GTTCAGTGGTAGAATTCTCGCCTT	0.4284973	0.546561	0.139694	0
tsrna-11566	GTTCAGTGGTAGAATTCTCGCCTGCCACGC	3.85647569	7.41761	2.444638	2.664651
tsrna-11565	GTTCAGTGGTAGAATTCTCGCCTGCCA	1.42832433	3.435525	0.908008	0.222054
tsrna-11564	GTTCAGTGGTAGAATTCTCGCCTGCC	1.2854919	3.045124	0.349234	0.333081
tsrna-11563	GTTCAGTGGTAGAATTCTCGCCTGC	0.21424865	0.624641	0.628621	0.222054
tsrna-11562	GTTCAGTGGTAGAATTCTCGCCTG	0.35708108	0.546561	0.139694	0.111027
tsrna-11561	GTTCAGTGGTAGAATTCTCGCCTCCCACGCGGGAGACCCGG	0.35708108	1.171202	0.698468	1.110271
tsrna-11560	GTTCAGTGGTAGAATTCTCGCCTCCC	0.35708108	1.093122	0.20954	0.999244
tsrna-11559	GTTCAGTGGTAGAATTCTCGCCTCC	0.21424865	1.171202	0.069847	1.110271
tsrna-11558	GTTCAGTGGTAGAATTCTCGCCTC	0.21424865	0.390401	0	0.222054
tsrna-11557	GTTCAGTGGTAGAATTCTCGCCT	0.28566487	0.31232	0	0.222054
tsrna-11556	GTTCAGTGGTAGAATTCTCGCC	0.07141622	0.390401	0.139694	0
tsrna-11555	GTTCAGTGGTAGAATTCTCGC	0.07141622	0.15616	0	0
tsrna-11554	GTTCAGTGGTAGAATTCTCG	0.14283243	0	0	0
tsrna-11553	GTTCAGTGGTAGAATTCTC	0	0	0	0
tsrna-11552	GTTCAGTGGTAGAATTCT	0	0.07808	0	0
tsrna-11549	GTTCAGGTCGCAGTCTCCCCTGGAGGCGTG	0	1.015041	0	0
tsrna-11547	GTTCAGGTCGCAGTCTCCCCTGG	0	0.07808	0	0
tsrna-11539	GTTCAATTCTCGCTGGGGCCTC	0	0	0	0
tsrna-11538	GTTCAATTCCTCTTCTTAACACCA	0	0.07808	0.069847	0
tsrna-11537	GTTCAATTCCTCTTCTTAACACC	0.07141622	0.07808	0	0
tsrna-11534	GTTCAATTCCCGGTCAGGGAACCA	0.07141622	1.405442	0.069847	0
tsrna-11533	GTTCAATTCCCGGTCAGGGAACC	0.07141622	0.07808	0	0
tsrna-11532	GTTCAATTCCCGGTCAGGGAAC	0	0.07808	0	0
tsrna-11531	GTTCAATTCCCCGACGGGGAGCCA	0	0.31232	0	0
tsrna-11522	GTTCAATGGTAGAATTCTCGCCT	0	0.23424	0	0.111027
tsrna-11521	GTTCAATGGTAGAATTCTCGCC	0	0.15616	0	0
tsrna-11520	GTTCAATCCCGGGTTTCGGCACCA	2.49956757	8.042251	0.488928	1.110271
tsrna-11518	GTTCAATCCCCGGCATCTCCACCA	7.57011894	13.42978	6.286213	5.44033
tsrna-11517	GTTCAATCCCCGGCATCTCCACC	0.21424865	0.780801	0.139694	0.333081
tsrna-11516	GTTCAATCCCCGGCATCTCCAC	0	0.15616	0	0
tsrna-11515	GTTCAATCCCCGGCATCTCCA	0	0	0	0.111027
tsrna-11511	GTTCAATCCCCGGCACCTCCACCA	15.9258163	22.33091	8.870544	5.995465
tsrna-11510	GTTCAATCCCCGGCACCTCCACC	0.64274595	0.936961	0.279387	0.111027
tsrna-11509	GTTCAATCCCCGGCACCTCCAC	0	0.07808	0	0
tsrna-11507	GTTCAATCCCCGGCACCTCC	0	0	0	0
tsrna-11503	GTTCAATCCCCAGCACCTCCACCA	0.49991351	0.702721	0.069847	0.222054
tsrna-11502	GTTCAAGTCTCGGTGGAACCTCCA	3.9278919	9.760014	1.53663	2.109516
tsrna-11501	GTTCAAGTCTCGGTGGAACCTCC	0.49991351	0.780801	0	0
tsrna-11500	GTTCAAGTCTCGGTGGAACCTC	0	0.07808	0	0
tsrna-11499	GTTCAAGTCTCGGTGGAACCT	0	0.07808	0	0
tsrna-11495	GTTCAAGTCCCTGTTCGGGCGCCA	0.57132973	0.624641	0.20954	0.333081
tsrna-11494	GTTCAAGTCCCTGTTCGGGCGCC	0	0.07808	0	0
tsrna-11487	GTTCAAGTCCCTGTTCAGGCGCCA	0.14283243	0.07808	0.069847	0
tsrna-11486	GTTCAAGTCACGTCGGGGTCACCA	0.28566487	0.31232	0	0.333081
tsrna-11485	GTTCAAGTCACGTCGGGGTCACC	0	0	0.139694	0
tsrna-11477	GTTCAAATCTCGGTGGGACCTCCA	3.85647569	8.432652	2.304945	2.442597
tsrna-11476	GTTCAAATCTCGGTGGGACCTCC	0.4284973	0.23424	0.20954	0.222054
tsrna-11475	GTTCAAATCTCGGTGGGACCTC	0	0.07808	0	0
tsrna-11474	GTTCAAATCTCGGTGGGACC	0	0	0	0
tsrna-11473	GTTCAAATCTCGGTGGGAC	0	0	0	0
tsrna-11472	GTTCAAATCTCGGTGGGA	0	0.07808	0	0
tsrna-11470	GTTCAAATCTCGGTGGAACCTCCA	11.6408433	17.88035	3.212953	2.886706
tsrna-11469	GTTCAAATCTCGGTGGAACCTCC	0.21424865	0.780801	0.069847	0
tsrna-11468	GTTCAAATCTCGGTGGAACCTC	0	0.468481	0	0
tsrna-11467	GTTCAAATCTCGGTGGAACCT	0.07141622	0.07808	0.069847	0
tsrna-11466	GTTCAAATCTCGGTGGAACC	0	0	0	0
tsrna-11461	GTTCAAATCTCGCTGGGGCCTCCA	2.85664866	4.138246	1.047702	1.665407
tsrna-11460	GTTCAAATCTCGCTGGGGCCTCC	0.28566487	0.23424	0.069847	0.222054
tsrna-11459	GTTCAAATCTCGCTGGGGCCTC	0	0	0	0
tsrna-11458	GTTCAAATCTCGCTGGGGCCT	0	0.07808	0	0
tsrna-11456	GTTCAAATCTCGCTGGGGC	0	0	0	0
tsrna-11454	GTTCAAATCCGGGTGCCCCCTCCA	9.71260543	13.03938	5.378204	2.33157

tsrna-11453	GTTCAAATCCGGGTGCCCCCTCC	0.49991351	0.858881	0.069847	0.111027
tsrna-11452	GTTCAAATCCGGGTGCCCCCTC	0	0.15616	0	0
tsrna-11451	GTTCAAATCCGGGTGCCCCCT	0	0.07808	0	0
tsrna-11445	GTTCAAATCCCGGACGAGCCCCCA	8.42711354	14.5229	3.212953	5.551357
tsrna-11444	GTTCAAATCCCGGACGAGCCCCC	0.4284973	1.327362	0.279387	0.222054
tsrna-11443	GTTCAAATCCCGGACGAGCCCC	0.14283243	0.07808	0	0
tsrna-11442	GTTCAAATCCCGGACGAGCCC	0.07141622	0	0	0
tsrna-11441	GTTCAAATCCCGGACGAGCC	0.14283243	0	0	0
tsrna-11436	GTTCAAATCCAGGTGCCCCCTCCA	0.07141622	0.07808	0.139694	0.222054
tsrna-11434	GTTCAAATCACGTCGGGGTCACCA	0.78557838	1.405442	0.20954	0.333081
tsrna-11433	GTTCAAATCACGTCGGGGTCACC	0	0	0.139694	0
tsrna-11425	GTTATCACGTTCGCCTCACACG	0	0.07808	0	0
tsrna-11421	GTTATCACGTCTGCTTTACACGC	0	0	0.069847	0
tsrna-11413	GTTATACCCTTCCCGTACTACCA	0.07141622	0.23424	0.069847	0
tsrna-11407	GTTATACCCTTCCCGTA	0	0	0	0
tsrna-11405	GTTAGTATCCCCGCCTGTC	0.07141622	0	0	0
tsrna-11404	GTTAGTATCCCCGCCTGT	0.14283243	0	0	0
tsrna-11402	GTTAGTATAGTGGTTAGTATCCCCGCC	0.14283243	0.15616	0	0
tsrna-11396	GTTAGTATAGTGGTGAGTATCCCCGCC	0.07141622	0.31232	0.069847	0.222054
tsrna-11395	GTTAGTATAGTGGTGAGTATCCCCGC	0.14283243	0.23424	0	0
tsrna-11394	GTTAGTATAGTGGTGAGTATCCCCG	0.14283243	0	0.069847	0
tsrna-11393	GTTAGTATAGTGGTGAGTATCCCC	0	0	0	0
tsrna-11385	GTTAGTACTCTGCGTTGTGGCCGCAGCAACCT	0.14283243	0.07808	0.069847	0.111027
tsrna-11384	GTTAGTACTCTGCGTTGTGGCCGCAGC	0.28566487	0.15616	0.139694	0
tsrna-11383	GTTAGTACTCTGCGTTGTGGCCGCA	0.21424865	0	0.139694	0
tsrna-11382	GTTAGTACTCTGCGTTGTGGCCGC	0.07141622	0	0	0.111027
tsrna-11381	GTTAGTACTCTGCGTTGTGGCCG	0.14283243	0	0.069847	0
tsrna-11380	GTTAGTACTCTGCGTTGTGGCC	0.35708108	0.07808	0	0
tsrna-11379	GTTAGTACTCTGCGTTGTGGC	0	0.07808	0.279387	0
tsrna-11378	GTTAGTACTCTGCGTTGTGG	0.07141622	0	0	0
tsrna-11377	GTTAGTACTCTGCGTTGTG	0.14283243	0.15616	0	0
tsrna-11376	GTTAGTACTCTGCGTTGT	0	0	0	0
tsrna-11373	GTTAGGCCTCTTTTTACCACCA	0.14283243	0.23424	0.139694	0.111027
tsrna-11372	GTTAGGCCTCTTTTTACCACC	0	0.07808	0.069847	0
tsrna-11371	GTTAGGCCTCTTTTTACCAC	0	0	0	0
tsrna-11370	GTTAGGCCTCTTTTTACCA	0	0	0	0.111027
tsrna-11369	GTTAGGCCTCTTTTTACC	0	0	0.069847	0
tsrna-11368	GTTAGGCCTCTTTTTAC	0.07141622	0	0	0
tsrna-11367	GTTAGGCCTCTTTTTA	0	0	0	0
tsrna-11366	GTTAGGATTCGGCGCTCTCAT	0.14283243	0	0	0
tsrna-11365	GTTAGGATTCGGCGCTCTCACCGCCGCGGCCCGGGTTC	0.35708108	1.639682	0.279387	0.555136
tsrna-11364	GTTAGGATTCGGCGCTCTCACCGCCGCGGCCCGGGTT	0.4284973	0.858881	0.139694	0.444109
tsrna-11363	GTTAGGATTCGGCGCTCTCACCGCCGCGGCCCGGGT	0.21424865	1.093122	0.139694	0.444109
tsrna-11362	GTTAGGATTCGGCGCTCTCACCGCCGCGGCCCGGG	0.28566487	1.561602	0.139694	0.555136
tsrna-11361	GTTAGGATTCGGCGCTCTCACCGCCGCGGCCC	0.28566487	1.952003	0.139694	0.333081
tsrna-11360	GTTAGGATTCGGCGCTCTCACCGCCGCGGCC	0.28566487	0.936961	0.419081	0.555136
tsrna-11359	GTTAGGATTCGGCGCTCTCACCGCCGCGGC	0.49991351	1.405442	0.139694	0.333081
tsrna-11358	GTTAGGATTCGGCGCTCTCACCGCC	0.4284973	1.327362	0.20954	0.111027
tsrna-11357	GTTAGGATTCGGCGCTCTCACCGC	0.07141622	0.468481	0.20954	0.111027
tsrna-11356	GTTAGGATTCGGCGCTCTCACCG	0.07141622	0.390401	0.139694	0.111027
tsrna-11355	GTTAGGATTCGGCGCTCTCACC	0.21424865	0.15616	0.069847	0
tsrna-11354	GTTAGGATTCGGCGCTCTCAC	0.07141622	0.15616	0.069847	0
tsrna-11353	GTTAGGATTCGGCGCTCTCA	0	0	0.069847	0
tsrna-11352	GTTAGGATTCGGCGCTCTC	0	0.07808	0	0
tsrna-11351	GTTAGGATTCGGCGCTCT	0	0.07808	0	0
tsrna-11350	GTTAGGATTCGGCGCTC	0.07141622	0.23424	0	0.111027
tsrna-11349	GTTAGGATTCGGCGCT	0	0	0	0
tsrna-11348	GTTAGGATTCCTGGTTTTCACC	0.4284973	0.468481	0	0.111027
tsrna-11347	GTTAGGATTCCTGGTTTTCA	0.07141622	0.07808	0.069847	0
tsrna-11346	GTTAGGATTCCTGGTTTTC	0.07141622	0.07808	0.279387	0
tsrna-11339	GTTAGCGCGTTCGGCTGTTAACC	0	0.546561	0.069847	0
tsrna-11336	GTTAGCGCGTTCGGCTGTTA	0	0	0.069847	0
tsrna-11327	GTTAGCACTCTGGACTTTGAATCCAGCGATCCGAGT	0.35708108	0.31232	0.139694	0.444109
tsrna-11326	GTTAGCACTCTGGACTTTGAATCCAGCG	0.71416216	0.546561	0.069847	0

tsrna-11325	GTTAGCACTCTGGACTTTGAATCCAGC	0	0.390401	0.139694	0.111027
tsrna-11324	GTTAGCACTCTGGACTTTGAATCCA	0.35708108	0.624641	0.20954	0
tsrna-11323	GTTAGCACTCTGGACTTTGAATCC	0.07141622	0.390401	0	0
tsrna-11322	GTTAGCACTCTGGACTTTGAATC	0.14283243	0.31232	0.069847	0.111027
tsrna-11315	GTTAGCACTCTGGACTCTGAATCCAGCGATCCGAGTTCA	0.07141622	0.23424	0	0.222054
tsrna-11314	GTTAGCACTCTGGACTCTGAATCCAGCGATCCGAGTTC	0.28566487	0.390401	0.139694	0.111027
tsrna-11313	GTTAGCACTCTGGACTCTGAATCCAGCGATCCGAGT	0.07141622	0.23424	0.069847	0
tsrna-11312	GTTAGCACTCTGGACTCTGAATCCAGCGATCCGAG	0.35708108	0.23424	0.279387	0
tsrna-11311	GTTAGCACTCTGGACTCTGAATCCAGCGA	0.07141622	0.31232	0.069847	0
tsrna-11310	GTTAGCACTCTGGACTCTGAATCCAGCG	0	0.390401	0.069847	0.222054
tsrna-11309	GTTAGCACTCTGGACTCTGAATCCAGC	0	0.23424	0.069847	0
tsrna-11308	GTTAGCACTCTGGACTCTGAATCCAG	0	0.390401	0.069847	0
tsrna-11307	GTTAGCACTCTGGACTCTGAATCCA	0	0	0.069847	0.111027
tsrna-11306	GTTAGCACTCTGGACTCTGAATCC	0	0.23424	0.139694	0
tsrna-11305	GTTAGCACTCTGGACTCTGAATC	0.07141622	0	0	0
tsrna-11303	GTTAGCACTCTGGACTCTGAA	0	0	0	0
tsrna-11288	GTTAAGATGGCAGAGCCTGGTAAT	0.07141622	0.07808	0	0
tsrna-11281	GTTAAGATGGCAGAGCCCGGTAATCGCATAAAACTTAAAACT	2.42815136	0.390401	3.911421	0.555136
tsrna-11280	GTTAAGATGGCAGAGCCCGGTAATCGCATAAAACTTAAAACT	0.8569946	0.23424	3.073259	0.444109
tsrna-11279	GTTAAGATGGCAGAGCCCGGTAATCGCATAAAACTTAAAACT	0.92841081	0.23424	2.793872	0.222054
tsrna-11278	GTTAAGATGGCAGAGCCCGGTAATCGCATAAAACTTAAAACT	1.21407568	0.468481	3.143106	0.111027
tsrna-11277	GTTAAGATGGCAGAGCCCGGTAATCGCATAAAACTTAAAACT	0.49991351	0.23424	0.628621	0
tsrna-11276	GTTAAGATGGCAGAGCCCGGTAATCGCATAAAACTTAAAACT	0.21424865	0.07808	0.279387	0.111027
tsrna-11275	GTTAAGATGGCAGAGCCCGGTAATCGCATAAAACTTAAAAC	0	0	0	0
tsrna-11274	GTTAAGATGGCAGAGCCCGGTAATCGCATAAAACTTAAAA	0	0.07808	0	0
tsrna-11273	GTTAAGATGGCAGAGCCCGGTAATCGCATAAAACTTAAA	0.07141622	0	0.20954	0.111027
tsrna-11272	GTTAAGATGGCAGAGCCCGGTAATCGCATAAAACTTAA	0.07141622	0	0.069847	0
tsrna-11271	GTTAAGATGGCAGAGCCCGGTAATCGCATAAAACTTA	0	0.15616	0	0
tsrna-11270	GTTAAGATGGCAGAGCCCGGTAATCGCATAAAACTT	0	0.15616	0.069847	0
tsrna-11269	GTTAAGATGGCAGAGCCCGGTAATCGCATAAAACT	0.07141622	0.15616	0	0.111027
tsrna-11268	GTTAAGATGGCAGAGCCCGGTAATCGCATAAAAC	0	0	0.139694	0
tsrna-11267	GTTAAGATGGCAGAGCCCGGTAATCGCATAAAA	0	0	0.069847	0
tsrna-11266	GTTAAGATGGCAGAGCCCGGTAATCGCATAAA	0	0.07808	0.20954	0
tsrna-11265	GTTAAGATGGCAGAGCCCGGTAATCGCATAA	0	0	0	0
tsrna-11264	GTTAAGATGGCAGAGCCCGGTAATCGCATA	0	0	0	0
tsrna-11263	GTTAAGATGGCAGAGCCCGGTAATCGCAT	0	0.07808	0	0
tsrna-11261	GTTAAGATGGCAGAGCCCGGTAATCGC	0.07141622	0	0	0.111027
tsrna-11258	GTTAAGATGGCAGAGCCCGGTAAT	0	0.07808	0	0
tsrna-11257	GTTAAGATGGCAGAGCCCGGTAA	0	0.07808	0	0
tsrna-11251	GTTAAGATGGCAGAGCCCGGC	0	0.07808	0	0
tsrna-11236	GTTAACTTCCAATTAACTAGT	0.07141622	0	0	0
tsrna-11230	GTTAACTAAGTGTTTGTGGGTTTAAGTCCCATTGGTCTAGCCA	0.35708108	0	0.20954	0.111027
tsrna-11229	GTTAACTAAGTGTTTGTGGGTTTAAGTCCCATTGGTCTAGCC	0.14283243	0.07808	0.20954	0.111027
tsrna-11228	GTTAACTAAGTGTTTGTGGGTTTAAGTCCCATTGGT	0.14283243	0.07808	0.349234	0
tsrna-11227	GTTAACTAAGTGTTTGTGGGTTTAAGTCCC	0.07141622	0	0	0.111027
tsrna-11226	GTTAACTAAGTGTTTGTGGGTTTAAGTCC	0.28566487	0	0	0
tsrna-11225	GTTAACTAAGTGTTTGTGGGTTTAAGTC	0.07141622	0.07808	0.069847	0.111027
tsrna-11224	GTTAACTAAGTGTTTGTGGGTTTAAGT	0.14283243	0	0	0
tsrna-11223	GTTAACTAAGTGTTTGTGGGTTTAAG	0	0	0.069847	0
tsrna-11222	GTTAACTAAGTGTTTGTGGGTTTAA	0	0	0.069847	0
tsrna-11221	GTTAACTAAGTGTTTGTGGGTTTA	0	0	0.20954	0
tsrna-11220	GTTAACTAAGTGTTTGTGGGTTT	0	0	0.069847	0
tsrna-11219	GTTAACTAAGTGTTTGTGGGTT	0.07141622	0	0	0
tsrna-11218	GTTAACTAAGTGTTTGTGGGT	0	0	0.069847	0
tsrna-11212	GTTAACCGAAAGGTTGGTGGTTCGAGCCCA	0.07141622	0.546561	0	0
tsrna-11211	GTTAACCGAAAGGTTGGTGGTTCGAGCCC	0.07141622	0.23424	0	0
tsrna-11210	GTTAACCGAAAGGTTGGTGGTTC	0.07141622	0.15616	0	0
tsrna-11209	GTTAACCGAAAGGTTGGTGGTT	0.07141622	0.15616	0	0
tsrna-11208	GTTAACCGAAAGGTTGGTGGT	0.07141622	0.07808	0	0
tsrna-11207	GTTAACCGAAAGGTTGGTGG	0	0.07808	0	0
tsrna-11203	GTTAAATTATAGGCTAAATCCTATATATC	0.07141622	0.07808	0	0
tsrna-11200	GTTAAATTATAGGCTAAATCCTATA	0	0.07808	0	0
tsrna-11199	GTTAAATTATAGGCTAAATCCTAT	0	0.15616	0	0
tsrna-11191	GTTAAATTATAGGCTA	0	0	0.069847	0

tsrna-11190	GTTAAATACAGACCAAGAGCCTTCAAAGCC	1.21407568	0.858881	0.838162	0.77719
tsrna-11189	GTTAAATACAGACCAAGAGCCTTCA	0.78557838	0	0.768315	0.111027
tsrna-11188	GTTAAATACAGACCAAGAGCCTTC	0.8569946	0.07808	0.768315	0.333081
tsrna-11187	GTTAAATACAGACCAAGAGCC	0	0.07808	0.069847	0
tsrna-11184	GTTAAATACAGACCAAGA	0.07141622	0	0	0
tsrna-11182	GTTAAATACAGACCAA	0	0	0	0
tsrna-11181	GTTAAAGATTAAGAGAACCAACACCTCTTTACAGTGACCA	0.49991351	0.07808	0.279387	0.111027
tsrna-11180	GTTAAAGATTAAGAGAACCAACACCTCTTTACAGTGACC	0.28566487	0	0.20954	0.111027
tsrna-11179	GTTAAAGATTAAGAGAACCAACACCTCTTTACAGTGAC	0.07141622	0	0.139694	0.111027
tsrna-11178	GTTAAAGATTAAGAGAACCAACACCTCTTTACAGTGA	0.07141622	0	0.279387	0
tsrna-11177	GTTAAAGATTAAGAGAACCAACACCTCTTT	0.07141622	0	0.069847	0.222054
tsrna-11176	GTTAAAGATTAAGAGAACCAACACCTCTT	0	0	0	0
tsrna-11175	GTTAAAGATTAAGAGAACCAACACCTCT	0.14283243	0.07808	0	0.222054
tsrna-11174	GTTAAAGATTAAGAGAACCAACACCT	0	0	0	0.111027
tsrna-11173	GTTAAAGATTAAGAGAACCAACACC	0	0	0	0
tsrna-11172	GTTAAAGATTAAGAGAACCAACAC	0	0	0	0
tsrna-11171	GTTAAAGATTAAGAGAACCAACA	0	0	0.069847	0
tsrna-11170	GTTAAAGATTAAGAGAACCAAC	0.07141622	0	0	0
tsrna-11169	GTTAAAGATTAAGAGAACCAA	0	0	0	0
tsrna-11167	GTTAAAGATTAAGAGAACC	0	0	0	0
tsrna-11163	GTTAAAGACTTTTTCTCTGACCA	1.64257298	2.732804	0.838162	1.665407
tsrna-11162	GTTAAAGACTTTTTCTCTGACC	0.28566487	0.702721	0	0
tsrna-11161	GTTAAAGACTTTTTCTCTGAC	0.07141622	0.07808	0	0
tsrna-11160	GTTAAAGACTTTTTCTCTGA	0	0	0.069847	0
tsrna-11158	GTTAAAGACTTTTTCTCT	0.07141622	0	0	0
tsrna-11155	GTGTTTGTGGGTTTAAGTCCCATTGGTCTAGCCA	0	0.07808	0	0.111027
tsrna-11154	GTGTTTGTGGGTTTAAGTCCCATTGGTCTAGCC	0.14283243	0.07808	0.069847	0
tsrna-11153	GTGTTTGTGGGTTTAAGTCCCATTGGTCTAGC	0.07141622	0	0	0
tsrna-11152	GTGTTTGTGGGTTTAAGTCCCATTGGTCT	0	0	0.069847	0
tsrna-11150	GTGTTTGTGGGTTTAAGTCCCATTG	0	0.23424	0.069847	0
tsrna-11149	GTGTTTGTGGGTTTAAGTCCCATT	0	0.07808	0	0.111027
tsrna-11140	GTGTTCGAATCACGTCGGGGTCACCA	0.14283243	0.624641	0.279387	0.222054
tsrna-11133	GTGTTCAAGTCACGTCGGGGTCACCA	0.4284973	0.468481	0.069847	0.222054
tsrna-11129	GTGTGATAGGTGGCACGGAGAATTTTG	0.14283243	0.23424	0.139694	0
tsrna-11128	GTGTGATAGGTGGCACGGAGA	0	0	0	0
tsrna-11124	GTGTGAGGTCCCGGGTTC	0	0.23424	0	0
tsrna-11123	GTGTGAGGTCCCGGGTT	0.21424865	0	0	0.111027
tsrna-11122	GTGTGAGGTCCCGGGT	0.07141622	0.23424	0.139694	0
tsrna-11121	GTGTGAGGCCCCGGGT	0	0.23424	0	0
tsrna-11120	GTGTAGTGGTTATCACGTTCGCCTCA	0.07141622	0	0	0
tsrna-11118	GTGTAGTGGTTATCACGTTCGCCT	0	0.07808	0	0
tsrna-11117	GTGTAGTGGTTATCACGTTCGCC	0	0	0.139694	0
tsrna-11103	GTGTAGTGGTCATCACGTTCGCCT	0	0.07808	0	0
tsrna-11097	GTGTAGTGGTATCATGCAAGATTCC	0.07141622	0	0	0
tsrna-11091	GTGTAGCTTAACACAAAGCACCCAACTTAC	0	0	0	0
tsrna-11089	GTGTAGCTCAGTGGTAGAGCGCGTGC	0.14283243	1.171202	0.279387	0
tsrna-11088	GTGTAGCTCAGTGGTAGAGCGC	0	0	0	0
tsrna-11087	GTGTAGCTCAGTGGTAGAGCATTTGACTGC	0.35708108	0.07808	0	0.111027
tsrna-11086	GTGTAGCTCAGTGGTAGAGCATTTGACT	0	0.15616	0.069847	0
tsrna-11084	GTGTAGCTCAGTGGTAGAG	0	0	0	0
tsrna-11079	GTGTAGCGGTTATCACATTCGCCTC	0	0	0	0
tsrna-11077	GTGTAGCGGTTATCACATTCGCC	0	0	0	0
tsrna-11073	GTGTAATGGTTAGCACTCTGGGCT	0.07141622	0.07808	0.069847	0
tsrna-11071	GTGTAATGGTTAGCACTCTGGACTTTGA	0.07141622	0.07808	0.069847	0
tsrna-11070	GTGTAATGGTTAGCACTCTGGACTTTG	0	0.15616	0	0
tsrna-11069	GTGTAATGGTTAGCACTCTGGACTTT	0.21424865	0.31232	0	0
tsrna-11068	GTGTAATGGTTAGCACTCTGGACTT	0	0.07808	0	0.333081
tsrna-11067	GTGTAATGGTTAGCACTCTGGACTCTGAATCCAGCG	0.71416216	0.780801	0.279387	0.333081
tsrna-11066	GTGTAATGGTTAGCACTCTGGACTCTGAA	0.07141622	0	0.069847	0
tsrna-11065	GTGTAATGGTTAGCACTCTGGACTCTGA	0.35708108	0	0	0.111027
tsrna-11064	GTGTAATGGTTAGCACTCTGGACTCTG	0.28566487	0.07808	0.069847	0.111027
tsrna-11063	GTGTAATGGTTAGCACTCTGGACTCT	0.07141622	0.15616	0.069847	0
tsrna-11062	GTGTAATGGTTAGCACTCTGGACTC	0	0.23424	0	0.111027
tsrna-11061	GTGTAATGGTTAGCACTCTGGACT	0	0.07808	0	0.222054

tsrna-11060	GTGTAATGGTTAGCACTCTGGAC	0.07141622	0	0	0.111027
tsrna-11059	GTGTAATGGTTAGCACTCTGGA	0.07141622	0	0	0
tsrna-11052	GTGTAATGGTGAGCACTCTGGACTCTGA	0.07141622	0	0.139694	0
tsrna-11051	GTGTAATGGTGAGCACTCTGGACTCTG	0	0	0	0.111027
tsrna-11050	GTGTAATGGTGAGCACTCTGGACTCT	0.14283243	0	0	0
tsrna-11049	GTGTAATGGTGAGCACTCTGGACTC	0.21424865	0	0	0
tsrna-11048	GTGTAATGGTGAGCACTCTGGACT	0.07141622	0	0	0.111027
tsrna-11047	GTGTAATGGTGAGCACTCTGGAC	0	0	0	0
tsrna-11046	GTGTAATGGTGAGCACTCTGGA	0	0	0	0
tsrna-11045	GTGTAATGGTGAGCACTCTGG	0	0.07808	0	0
tsrna-11043	GTGTAATGGTCAGCACTCTGGACTCTGA	0	0.07808	0	0
tsrna-11041	GTGTAATGGTCAGCACTCTGGACTC	0	0	0	0
tsrna-11034	GTGTAATGGTAAGCACTCTGGACTC	0	0	0	0
tsrna-11031	GTGGTTTAGTGGTAGAATTCTCGCCT	0.07141622	0.15616	0	0.222054
tsrna-11030	GTGGTTTAGTGGTAGAATTCTCGCC	0.14283243	0.15616	0.069847	0.111027
tsrna-11029	GTGGTTTAGTGGTAGAATTCTCGC	0.07141622	0.07808	0	0
tsrna-11028	GTGGTTTAGTGGTAGAATTCTCG	0	0.15616	0	0
tsrna-11027	GTGGTTTAGTGGTAGAATTCTC	0	0	0	0
tsrna-11025	GTGGTTTAGTGGTAGAA	0	0.07808	0	0
tsrna-11024	GTGGTTGTAGTCCGTGCGAGAATACCA	0.57132973	1.327362	0.628621	0.666163
tsrna-11023	GTGGTTGTAGTCCGTGCGAGAATACC	0.07141622	0.390401	0.139694	0.111027
tsrna-11022	GTGGTTGTAGTCCGTGCGAGAATAC	0.14283243	0.07808	0.20954	0
tsrna-11021	GTGGTTGTAGTCCGTGCGAGAATA	0.14283243	0.15616	0.069847	0.111027
tsrna-11020	GTGGTTGTAGTCCGTGCGAGAAT	0	0.07808	0.069847	0
tsrna-11019	GTGGTTGTAGTCCGTGCGAGAA	0	0.15616	0.139694	0
tsrna-11018	GTGGTTGTAGTCCGTGCGAGA	0	0	0	0
tsrna-11017	GTGGTTGTAGTCCGTGCGAG	0	0	0	0
tsrna-11016	GTGGTTGTAGTCCGTGCGA	0	0	0	0
tsrna-11015	GTGGTTGTAGTCCGTGCG	0	0	0	0
tsrna-11012	GTGGTTCGAGCCCACCCAGGGACGCCA	0.35708108	7.18337	0.279387	0
tsrna-11010	GTGGTTCAGTGGTAGAATTCTTGCCT	0.07141622	0	0.139694	0
tsrna-11009	GTGGTTCAGTGGTAGAATTCTT	0.14283243	0.15616	0.20954	0
tsrna-11008	GTGGTTCAGTGGTAGAATTCTCGCCTGCC	1.2854919	3.747845	0.628621	0.333081
tsrna-11007	GTGGTTCAGTGGTAGAATTCTCGCCTGC	0.99982703	0.468481	0.20954	0.222054
tsrna-11006	GTGGTTCAGTGGTAGAATTCTCGCCTG	0.4284973	0.780801	0.20954	0.444109
tsrna-11005	GTGGTTCAGTGGTAGAATTCTCGCCTCC	0.64274595	0.936961	0.069847	1.443353
tsrna-11004	GTGGTTCAGTGGTAGAATTCTCGCCT	0.64274595	0.546561	0	0.222054
tsrna-11003	GTGGTTCAGTGGTAGAATTCTCGCC	0.35708108	0.31232	0.069847	0
tsrna-11002	GTGGTTCAGTGGTAGAATTCTCGC	0.21424865	0.15616	0.069847	0.111027
tsrna-11001	GTGGTTCAGTGGTAGAATTCTCG	0.14283243	0.07808	0.139694	0
tsrna-11000	GTGGTTCAGTGGTAGAATTCTC	0.14283243	0.23424	0.139694	0.111027
tsrna-10999	GTGGTTCAGTGGTAGAATTCT	0.14283243	0.07808	0.069847	0
tsrna-10998	GTGGTTCAGTGGTAGAATTC	0.07141622	0.07808	0	0.111027
tsrna-10997	GTGGTTCAGTGGTAGAATT	0.21424865	0.07808	0.139694	0
tsrna-10996	GTGGTTCAGTGGTAGAAT	0.14283243	0.15616	0.139694	0
tsrna-10995	GTGGTTCAGTGGTAGAA	0	0.07808	0.069847	0
tsrna-10993	GTGGTTATCACGTTCGCCTCACACGC	0.28566487	0.15616	0	0.111027
tsrna-10992	GTGGTTATCACGTTCGCCTCACACG	0	0	0	0
tsrna-10991	GTGGTTATCACGTTCGCCTCACAC	0	0.23424	0	0
tsrna-10990	GTGGTTATCACGTTCGCCTCACA	0	0	0	0.222054
tsrna-10989	GTGGTTATCACGTTCGCCTCAC	0	0	0.069847	0
tsrna-10988	GTGGTTATCACGTTCGCCTCA	0	0.07808	0	0
tsrna-10986	GTGGTTATCACGTTCGCCTA	0	0.07808	0	0
tsrna-10984	GTGGTTATCACGTTCGCC	0.07141622	0	0	0
tsrna-10982	GTGGTTATCACGTCTGCTTTACACGCAGAAGGTCCTGGGT	0.8569946	2.498564	0.419081	0
tsrna-10981	GTGGTTATCACGTCTGCTTTACACG	0.28566487	0	0.20954	0.111027
tsrna-10980	GTGGTTATCACGTCTGCTTTAC	0.14283243	0	0.069847	0
tsrna-10977	GTGGTTATCACGTCTGCTT	0.07141622	0	0	0
tsrna-10970	GTGGTTAGTATCCCCGCCTGTCACGCGGG	0.71416216	3.123204	0.628621	0.666163
tsrna-10969	GTGGTTAGTATCCCCGCCTGTCA	0.07141622	0.07808	0.069847	0
tsrna-10968	GTGGTTAGTATCCCCGCCTGTC	0.21424865	0	0	0.111027
tsrna-10967	GTGGTTAGTATCCCCGCCTGT	0.14283243	0	0	0
tsrna-10966	GTGGTTAGTATCCCCGCCTG	0.07141622	0	0	0
tsrna-10965	GTGGTTAGTATCCCCGCCT	0.14283243	0.07808	0	0

tsrna-10964	GTGGTTAGTATCCCCGCC	0	0	0	0
tsrna-10963	GTGGTTAGTATCCCCGC	0.07141622	0	0.069847	0
tsrna-10961	GTGGTTAGTACTCTGCGTTGTGGCCGCAGC	0.99982703	0.23424	0.069847	0.111027
tsrna-10960	GTGGTTAGTACTCTGCGTTGTGGCCGC	0.78557838	0.31232	0.419081	0.333081
tsrna-10959	GTGGTTAGTACTCTGCGTTGTGGCCG	0.78557838	0.390401	0.419081	0.333081
tsrna-10958	GTGGTTAGTACTCTGCGTTGTGGCC	0.71416216	0.546561	0.279387	0.222054
tsrna-10957	GTGGTTAGTACTCTGCGTTGTGGC	0.8569946	0.31232	0.20954	0.222054
tsrna-10956	GTGGTTAGTACTCTGCGTTGTGG	0.71416216	0	0.279387	0.444109
tsrna-10955	GTGGTTAGTACTCTGCGTTGTG	0.71416216	0.15616	0.488928	0.444109
tsrna-10954	GTGGTTAGTACTCTGCGTTGT	0.8569946	0.15616	0.20954	0.222054
tsrna-10953	GTGGTTAGTACTCTGCGTTG	0.57132973	0.15616	0.349234	0
tsrna-10952	GTGGTTAGTACTCTGCGTT	0.49991351	0.390401	0.20954	0.111027
tsrna-10951	GTGGTTAGTACTCTGCGT	0	0.07808	0	0
tsrna-10950	GTGGTTAGTACTCTGCGCTGTGGCC	0	0.15616	0.069847	0
tsrna-10949	GTGGTTAGTACTCTGCGCTGTGG	0.07141622	0	0	0
tsrna-10948	GTGGTTAGTACTCTGCGCTGTG	0	0.07808	0	0.222054
tsrna-10947	GTGGTTAGTACTCTGCGCTGT	0.14283243	0.07808	0	0
tsrna-10946	GTGGTTAGTACTCTGCGCTG	0.14283243	0.07808	0.069847	0
tsrna-10945	GTGGTTAGTACTCTGCGCT	0.07141622	0.15616	0	0
tsrna-10944	GTGGTTAGTACTCTGCGC	0.21424865	0.07808	0	0.111027
tsrna-10943	GTGGTTAGTACTCTGCG	0	0	0	0
tsrna-10941	GTGGTTAGGATTCGGCGCTCTCAT	0.64274595	0.624641	0.349234	0.666163
tsrna-10940	GTGGTTAGGATTCGGCGCTCTCACCGCCGCGGCCCGGGTT	1.14265946	1.639682	0.768315	0.77719
tsrna-10939	GTGGTTAGGATTCGGCGCTCTCACCGCCGCGGCCCGGGT	1.21407568	2.186243	0.977855	0.77719
tsrna-10938	GTGGTTAGGATTCGGCGCTCTCACCGCCGCGGCCCGGG	0.92841081	1.795843	0.628621	0.444109
tsrna-10937	GTGGTTAGGATTCGGCGCTCTCACCGCCGCGGCCCG	0.49991351	1.952003	0.628621	0.666163
tsrna-10936	GTGGTTAGGATTCGGCGCTCTCACCGCCGCGGCCC	0.78557838	2.498564	0.349234	1.110271
tsrna-10935	GTGGTTAGGATTCGGCGCTCTCACCGCCGC	0.99982703	1.639682	0.558774	0.666163
tsrna-10934	GTGGTTAGGATTCGGCGCTCTCACCGCCG	1.07124325	1.405442	0.628621	1.221299
tsrna-10933	GTGGTTAGGATTCGGCGCTCTCACCGCC	1.07124325	2.030083	0.419081	1.332326
tsrna-10932	GTGGTTAGGATTCGGCGCTCTCACCGC	0.57132973	1.093122	0.558774	1.110271
tsrna-10931	GTGGTTAGGATTCGGCGCTCTCACCG	0.99982703	1.093122	0.419081	0.555136
tsrna-10930	GTGGTTAGGATTCGGCGCTCTCACC	0.64274595	0.780801	0.279387	0.666163
tsrna-10929	GTGGTTAGGATTCGGCGCTCTCAC	0.57132973	0.936961	0.628621	0.999244
tsrna-10928	GTGGTTAGGATTCGGCGCTCTCA	0.71416216	1.171202	0.488928	1.332326
tsrna-10927	GTGGTTAGGATTCGGCGCTCTC	0.14283243	0.702721	0.279387	0.222054
tsrna-10926	GTGGTTAGGATTCGGCGCTCT	0.64274595	1.249282	0.419081	0.333081
tsrna-10925	GTGGTTAGGATTCGGCGCTC	0.4284973	0.858881	0.279387	0
tsrna-10924	GTGGTTAGGATTCGGCGCT	0.28566487	0.780801	0.349234	0.444109
tsrna-10923	GTGGTTAGGATTCGGCGC	0.14283243	0.390401	0.139694	0.111027
tsrna-10922	GTGGTTAGGATTCGGCG	0.14283243	0	0	0
tsrna-10921	GTGGTTAGGATTCGGC	0.14283243	0	0.069847	0
tsrna-10909	GTGGTTAAGGCGTTGGACTTG	0	0	0.069847	0
tsrna-10896	GTGGTTAAGGCGATGGA	0	0.07808	0	0
tsrna-10892	GTGGTGAGTATCCCTGCCTGT	0	0.07808	0	0
tsrna-10891	GTGGTGAGTATCCCCGCCTGTCACGCGGGA	2.14248649	3.747845	0.977855	0.555136
tsrna-10890	GTGGTGAGTATCCCCGCCTGTCACGCGGG	2.28531893	4.138246	1.187396	0.444109
tsrna-10889	GTGGTGAGTATCCCCGCCTGTCACGCG	0.28566487	0.780801	0.20954	0
tsrna-10888	GTGGTGAGTATCCCCGCCTGTCACGC	0.21424865	0.702721	0.628621	0.222054
tsrna-10887	GTGGTGAGTATCCCCGCCTGTCACG	0.14283243	0.780801	0	0
tsrna-10886	GTGGTGAGTATCCCCGCCTGTCAC	0.14283243	0.624641	0	0.222054
tsrna-10885	GTGGTGAGTATCCCCGCCTGTCA	0.14283243	0.780801	0.069847	0.111027
tsrna-10884	GTGGTGAGTATCCCCGCCTGTC	0.07141622	0.624641	0.069847	0.333081
tsrna-10883	GTGGTGAGTATCCCCGCCTGT	0.21424865	0.15616	0	0
tsrna-10882	GTGGTGAGTATCCCCGCCTG	0	0.624641	0.139694	0.111027
tsrna-10881	GTGGTGAGTATCCCCGCCT	0.07141622	0.23424	0	0.222054
tsrna-10880	GTGGTGAGTATCCCCGCC	0.07141622	0.07808	0	0
tsrna-10879	GTGGTGAGTATCCCCGC	0	0	0	0
tsrna-10878	GTGGTGAGTATCCCCG	0	0.07808	0	0
tsrna-10874	GTGGTGAGCATAGCTGCCT	0	0	0.069847	0
tsrna-10873	GTGGTGAGCATAGCTGCC	0	0	0	0
tsrna-10872	GTGGTGAGCATAGCTGC	0	0.07808	0	0
tsrna-10871	GTGGTGAGCATAGCTG	0	0	0	0
tsrna-10870	GTGGTCTAGTGGTTAGGATTCGGCGCTCTC	2.28531893	2.888964	1.53663	2.220543

tsrna-10869	GTGGTCTAGTGGTTAGGATTCGGCGCTCT	2.78523244	3.357445	1.676323	2.886706
tsrna-10868	GTGGTCTAGTGGTTAGGATTCGGCGCTC	2.42815136	3.669765	1.606477	1.665407
tsrna-10867	GTGGTCTAGTGGTTAGGATTCGGCGCT	3.4993946	3.825925	1.606477	1.55438
tsrna-10866	GTGGTCTAGTGGTTAGGATTCGGCGC	1.21407568	0.936961	0.488928	0.666163
tsrna-10865	GTGGTCTAGTGGTTAGGATTCGGCG	0.92841081	0.780801	0.488928	0.666163
tsrna-10864	GTGGTCTAGTGGTTAGGATTCGGC	0.35708108	0.546561	0.349234	0.444109
tsrna-10863	GTGGTCTAGTGGTTAGGATTCGG	0.28566487	0.15616	0.069847	0.111027
tsrna-10862	GTGGTCTAGTGGTTAGGATTCG	0.14283243	0.15616	0.069847	0
tsrna-10861	GTGGTCTAGTGGTTAGGATTC	0.14283243	0.15616	0.139694	0
tsrna-10860	GTGGTCTAGTGGTTAGGATT	0.07141622	0.07808	0	0
tsrna-10859	GTGGTCTAGTGGTTAGGAT	0	0	0	0.111027
tsrna-10855	GTGGTCTAGTGGCTAGGATTCGGCGC	0.64274595	2.108163	0.349234	3.552868
tsrna-10854	GTGGTCTAGTGGCTAGGATTCGGCG	0.71416216	1.639682	0.349234	2.664651
tsrna-10853	GTGGTCTAGTGGCTAGGATTCGG	0.21424865	0.15616	0	0.111027
tsrna-10852	GTGGTCTAGTGGCTAGGATTCG	0.14283243	0	0	0.111027
tsrna-10851	GTGGTCTAGTGGCTAGGATT	0	0.15616	0	0
tsrna-10849	GTGGTCTAAGGCGCTGGA	0	0	0	0
tsrna-10844	GTGGTATGATTCTCGCTTTG	0.14283243	0.07808	0.139694	0
tsrna-10843	GTGGTATGATTCTCGCTTT	0.07141622	0.15616	0	0
tsrna-10842	GTGGTATGATTCTCGCTT	0.21424865	0.07808	0.139694	0
tsrna-10841	GTGGTATGATTCTCGC	0	0.15616	0.20954	0
tsrna-10832	GTGGTATAGTGGTTAGCATAGCTGCC	0	0.07808	0.069847	0
tsrna-10828	GTGGTATAGTGGTGAGCATAGCTGCC	0	0.07808	0	0
tsrna-10827	GTGGTATAGTGGTGAGCATAGCTGC	0	0.31232	0	0
tsrna-10826	GTGGTATAGTGGTGAGCATAGCTG	0.07141622	0.07808	0	0.222054
tsrna-10825	GTGGTATAGTGGTGAGCATAGCT	0.14283243	0	0	0
tsrna-10824	GTGGTATAGTGGTGAGCATAGC	0	0	0.069847	0
tsrna-10823	GTGGTATAGTGGTGAGCATAG	0	0	0	0
tsrna-10822	GTGGTATAGTGGTGAGCATA	0.07141622	0.07808	0.069847	0
tsrna-10821	GTGGTATAGTGGTGAGCA	0	0.07808	0	0
tsrna-10818	GTGGTATAGTGGTAAGCATAGCTG	0	0.07808	0	0
tsrna-10815	GTGGTAGAGCGCTCGC	0	0.07808	0	0
tsrna-10814	GTGGTAGAGCGCGTGCT	0.64274595	1.639682	0.139694	0
tsrna-10813	GTGGTAGAGCGCGTGC	0.28566487	1.327362	0.349234	0
tsrna-10810	GTGGTAGAGCATTTGACTGCAGATCAAG	0	0.23424	0	0
tsrna-10809	GTGGTAGAGCATTTGACTGCAGATCA	0	0.07808	0	0
tsrna-10808	GTGGTAGAGCATTTGACTGCAGATC	0	0.07808	0	0
tsrna-10807	GTGGTAGAGCATTTGACTGCA	0	0.23424	0	0
tsrna-10806	GTGGTAGAGCATTTGACTGC	0.07141622	0.07808	0	0
tsrna-10805	GTGGTAGAGCATTTGACTG	0	0	0	0.111027
tsrna-10804	GTGGTAGAGCATTTGACT	0	0.07808	0	0
tsrna-10801	GTGGTAGAATTCTTGCCTGC	0	0	0	0
tsrna-10800	GTGGTAGAATTCTCGCCTT	0	0.23424	0.069847	0
tsrna-10799	GTGGTAGAATTCTCGCCTGCCACG	3.07089731	6.402569	2.025557	1.998488
tsrna-10798	GTGGTAGAATTCTCGCCTGCCAC	1.42832433	3.045124	0.488928	0.555136
tsrna-10797	GTGGTAGAATTCTCGCCTGCCA	0.92841081	3.201285	0.349234	0.111027
tsrna-10796	GTGGTAGAATTCTCGCCTGCC	1.21407568	2.420483	0.419081	0.333081
tsrna-10795	GTGGTAGAATTCTCGCCTGC	0.4284973	0.702721	0.069847	0.333081
tsrna-10794	GTGGTAGAATTCTCGCCTG	0.07141622	0.702721	0.069847	0.333081
tsrna-10793	GTGGTAGAATTCTCGCCTCCCACGCGGGAGACCCGGGT	0.64274595	1.405442	0.139694	1.221299
tsrna-10792	GTGGTAGAATTCTCGCCTCCCACG	0.35708108	0.468481	0.069847	0.999244
tsrna-10791	GTGGTAGAATTCTCGCCTCCC	0.14283243	0.858881	0.069847	0.77719
tsrna-10790	GTGGTAGAATTCTCGCCTCC	0.07141622	0.702721	0.069847	0.555136
tsrna-10789	GTGGTAGAATTCTCGCCTC	0.14283243	0.23424	0.069847	0.111027
tsrna-10788	GTGGTAGAATTCTCGCCT	0.07141622	0.31232	0	0.222054
tsrna-10787	GTGGTAGAATTCTCGCC	0.07141622	0.23424	0	0
tsrna-10786	GTGGTAGAATTCTCGC	0	0.15616	0	0
tsrna-10783	GTGGTAAGCATAGCTGCCTTCC	0.07141622	0	0	0
tsrna-10782	GTGGTAAGCATAGCTGCCTTC	0	0.07808	0	0
tsrna-10778	GTGGGTTTAAGTCCCATTGGTCTAGCCA	0.07141622	0.15616	0.069847	0.111027
tsrna-10777	GTGGGTTTAAGTCCCATTGGTCTAGCC	0	0	0	0
tsrna-10776	GTGGGTTTAAGTCCCATTGGTCTAGC	0	0	0.069847	0
tsrna-10775	GTGGGTTTAAGTCCCATTGGTCTAG	0.07141622	0.15616	0.069847	0
tsrna-10774	GTGGGTTTAAGTCCCATTGGTCTA	0	0	0	0

tsrna-10772	GTGGGTTCGAGTCCCATCTGGGTCGCCA	0.14283243	0.936961	0	0.111027
tsrna-10771	GTGGGTTCGAGTCCCATCTGGGTCGCC	0.07141622	0	0.069847	0
tsrna-10770	GTGGGTTCGAATCCCATCCTCGTCGCCA	0.4284973	0.858881	0.279387	0.222054
tsrna-10769	GTGGGTTCGAATCCCATCCTCGTCGCC	0	0.15616	0	0
tsrna-10768	GTGGGTTCGAATCCCATCCTCGTCGC	0	0.07808	0	0
tsrna-10767	GTGGGTTCGAATCCCATCCTCGTCG	0.14283243	0	0	0
tsrna-10766	GTGGGTTCGAATCCCATCCTCGTC	0	0	0	0
tsrna-10763	GTGGGTTCGAATCCCACTTCTGACACCA	1.99965406	2.186243	0.768315	1.110271
tsrna-10762	GTGGGTTCGAATCCCACTTCTGACACC	0.14283243	0.15616	0.069847	0.222054
tsrna-10756	GTGGGTTCGAATCCCACTCCTGACACCA	7.49870272	9.213453	3.981268	4.108004
tsrna-10755	GTGGGTTCGAATCCCACTCCTGACACC	0.21424865	0.858881	0.20954	0.111027
tsrna-10754	GTGGGTTCGAATCCCACTCCTGACAC	0	0.07808	0.069847	0
tsrna-10752	GTGGGTTCGAATCCCACTCCTGAC	0.07141622	0.07808	0	0
tsrna-10748	GTGGGTTCGAATCCCACCGCTGCCACCA	0.8569946	3.357445	0.139694	0.555136
tsrna-10746	GGGGAATTAGCTCAAGTGGTAGAGC	0	0.15616	0	0.222054
tsrna-10745	GGGGAATTAGCTCAAGTGGTAGA	0	0.31232	0	0.222054
tsrna-10744	GGGGAATTAGCTCAAGTGGTAG	0	0	0	0.444109
tsrna-10743	GGGGAATTAGCTCAAGTGGTA	0	0.07808	0.069847	0.111027
tsrna-10742	GGGGAATTAGCTCAAGTGGT	0.07141622	0.23424	0	0
tsrna-10741	GGGGAATTAGCTCAAGTGG	0.07141622	0.15616	0	0.111027
tsrna-10740	GGGGAATTAGCTCAAGT	0	0	0.069847	0.111027
tsrna-10739	GGGGAATTAGCTCAAGCGGTAGAGCGCTTG	0	0	0	0.222054
tsrna-10738	GGGGAATTAGCTCAAGCGGTAGAGC	0	0.07808	0	0.111027
tsrna-10737	GGGGAATTAGCTCAAGCGGTAGAG	0.28566487	0.390401	0	0.444109
tsrna-10736	GGGGAATTAGCTCAAGCGGTAGA	0.07141622	0.15616	0	0.222054
tsrna-10735	GGGGAATTAGCTCAAGCGGTA	0	0	0	0.222054
tsrna-10734	GGGGAATTAGCTCAAGCGGT	0	0.15616	0	0.111027
tsrna-10733	GGGGAATTAGCTCAAGCGG	0.07141622	0.07808	0	0.111027
tsrna-10732	GGGGAATTAGCTCAAGCG	0	0	0	0.111027
tsrna-10731	GGGGAATTAGCTCAAGC	0	0.07808	0	0.222054
tsrna-10730	GGGGAATTAGCTCAAATGGTAGAGCGCTCGCTT	0.49991351	0.546561	0.20954	0.333081
tsrna-10729	GGGGAATTAGCTCAAATGGTAGAGCGCTCGCT	0.49991351	0.702721	0.139694	0.77719
tsrna-10728	GGGGAATTAGCTCAAATGGTAGAGCGCTCG	0.57132973	0.702721	0.279387	0.77719
tsrna-10727	GGGGAATTAGCTCAAATGGTAGAGCGC	0.49991351	0.390401	0.069847	0.666163
tsrna-10726	GGGGAATTAGCTCAAATGGTAGAGC	0.21424865	1.093122	0.139694	0.111027
tsrna-10725	GGGGAATTAGCTCAAATGGTAGAG	0.64274595	0.702721	0.069847	0.444109
tsrna-10724	GGGGAATTAGCTCAAATGGTAGA	0.21424865	0.780801	0.20954	0.666163
tsrna-10723	GGGGAATTAGCTCAAATGGTAG	0.57132973	0.15616	0.139694	1.110271
tsrna-10722	GGGGAATTAGCTCAAATGGTA	0.35708108	0.780801	0.20954	0.555136
tsrna-10721	GGGGAATTAGCTCAAATGGT	0.21424865	0.390401	0.139694	0.444109
tsrna-10720	GGGGAATTAGCTCAAATGG	0.21424865	0.780801	0.069847	0.222054
tsrna-10719	GGGGAATTAGCTCAAATG	0.35708108	0.23424	0.139694	0.444109
tsrna-10718	GGGGAATTAGCTCAAAT	0.35708108	0.858881	0.139694	0.111027
tsrna-10717	GGGGAATTAGCTCAAA	0.35708108	0.702721	0.069847	0.111027
tsrna-10716	GGGCTTAGCTTAATTAAAGTGGCTGATTTG	0.14283243	0	0.069847	0
tsrna-10713	GGGCTCCGTGGCGCAATGGA	0	0	0	0.111027
tsrna-10712	GGGCTCACATCACCCCATAAACACCA	0.4284973	0.23424	0.139694	0.111027
tsrna-10703	GGGCGTATAGCTCAGGGGTAGAGCATTTG	0	0.07808	0	0
tsrna-10701	GGGCGTATAGCTCAGGGGTAGAGC	0	0	0	0
tsrna-10699	GGGCCCATACCCCGAAAATGTTGGTTATACCCTTCC	0.07141622	0	0	0
tsrna-10698	GGGCCCATACCCCGAAAATGTTGGTTATACC	0.07141622	0	0	0
tsrna-10697	GGGCCCATACCCCGAAAATGTTGGTTATAC	0	0	0	0
tsrna-10696	GGGCCCATACCCCGAAAATGTTGGTTA	0	0.07808	0	0
tsrna-10692	GGGCCCATACCCCGAAAATG	0	0.07808	0	0
tsrna-10691	GGGCCCATAACCCAGAGGTCGATGGA	0.78557838	0.468481	0	0.222054
tsrna-10690	GGGCCCATAACCCAGAGGTCGATG	0.14283243	0.07808	0	0.111027
tsrna-10689	GGGCCCATAACCCAGAGGTCGAT	0.07141622	0.07808	0	0
tsrna-10688	GGGCCCATAACCCAGAGGTCGA	0	0	0	0
tsrna-10683	GGGCCAGTGGCGCAATGGATAACGCGTCTGACT	0.07141622	0	0.069847	0.222054
tsrna-10682	GGGCCAGTGGCGCAATGGATAACGCGTCTGAC	0	0	0.069847	0
tsrna-10681	GGGCCAGTGGCGCAATGGATAACGCGTCTG	0	0.07808	0	0
tsrna-10679	GGGCCAGTGGCGCAATGGATAACGCGTC	0	0.07808	0	0
tsrna-10677	GGGCCAGTGGCGCAATGGATAACG	0.07141622	0	0	0
tsrna-10676	GGGCCAGTGGCGCAATGGATAAC	0	0	0	0

tsrna-10673	GGGCCAGTGGCGCAATGGAT	0	0.07808	0	0
tsrna-10671	GGGCCAGTGGCGCAATGG	0	0	0	0
tsrna-10668	GGGCAGAGCATTTGACTGC	0	0	0	0
tsrna-10666	GGGATTGTGGGTTCGAGTCCCATCTGGGTCGCC	0.07141622	0.15616	0.069847	0
tsrna-10665	GGGATTGTGGGTTCGAGTCCCATCTGGGGTGCCA	0	0.31232	0	0
tsrna-10664	GGGATTGTGGGTTCGAGTCCCATCTGGGGTGCC	0	0.23424	0	0
tsrna-10663	GGGATTGTGGGTTCGAGTCCCATC	0	0.07808	0	0
tsrna-10662	GGGATTGTGGGTTCGAGTCCCACC	0	0.07808	0	0
tsrna-10660	GGGATTGTGGGTTCGAGTCCC	0	0	0	0
tsrna-10659	GGGATTGTGGGTTCGAGTCC	0	0	0	0
tsrna-10657	GGGATTAGCTCAAATGGTAGAGCGCTCGC	0.4284973	1.171202	0.419081	0.111027
tsrna-10656	GGGATTAGCTCAAATGGTAGAGC	0.49991351	1.015041	0.279387	0.444109
tsrna-10655	GGGATTAGCTCAAATGGTAGA	0.78557838	0.936961	0.349234	0.333081
tsrna-10654	GGGATTAGCTCAAATGGTAG	0.4284973	0.468481	0.20954	0.444109
tsrna-10653	GGGATGTAGCTCAGTGGTAGAGC	0	0.07808	0	0
tsrna-10651	GGGATGTAGCTCAGTGGTAGA	0	0	0	0
tsrna-10648	GGGATGTAGCTCAGTGGT	0.07141622	0.07808	0	0
tsrna-10645	GGGATGGGTTCGATTCTCATAGTC	0.07141622	0.07808	0	0
tsrna-10644	GGGATGGGTTCGATTCTCATAGT	0	0	0.139694	0
tsrna-10643	GGGATGGGTTCGATTCTCATAG	0	0	0	0
tsrna-10642	GGGATGGGTTCGATTCTCATA	0	0.07808	0	0
tsrna-10641	GGGATGGGTTCGATTCTCA	0	0.15616	0.069847	0
tsrna-10640	GGGATGGGTTCGATTCT	0	0.07808	0	0
tsrna-10639	GGGATGGGTTCGATTC	0	0	0.069847	0
tsrna-10638	GGGATCGATGCCCGCATCCTCCACCA	0.21424865	0.546561	0.139694	0
tsrna-10637	GGGATCGATGCCCGCATCCTCCACC	0	0.07808	0	0
tsrna-10635	GGGAGGCCCGGGTTCGATTCCCGG	0.07141622	0	0.069847	0
tsrna-10626	GGGAGACCGGGGTTCGATTCCCCGACGGGGAGCCA	0.07141622	1.015041	0	0
tsrna-10619	GGGAGACCGGGGTTCGATTCCCCGAC	0	0	0	0
tsrna-10611	GGGACTCTTAATCCCAGGGTCGTGGGTT	0.07141622	0	0	0
tsrna-10610	GGGAATTAGCTCAAATGGTAGAGC	0	0.31232	0.069847	0.111027
tsrna-10609	GGGAATTAGCTCAAATGGTAGAG	0.07141622	0.07808	0	0
tsrna-10608	GGGAATTAGCTCAAATGGTAGA	0	0.23424	0	0
tsrna-10607	GGGAATTAGCTCAAATGGT	0	0	0.069847	0.111027
tsrna-10606	GGCTTGAAACCAGCTTTGGGGG	0	0	0	0
tsrna-10600	GGCTTACGACCCCTTATTTACCCCA	0.28566487	0.07808	0.139694	0
tsrna-10597	GGCTTACGACCCCTTATTTACC	0	0	0.069847	0
tsrna-10591	GGCTGTTAACCGAAAGGTTGGTGGT	0.28566487	2.498564	0.139694	0.555136
tsrna-10590	GGCTGTTAACCGAAAGGTTGGTG	0.07141622	0.07808	0	0
tsrna-10589	GGCTGTTAACCGAAAGGTTGGT	0	0.07808	0	0
tsrna-10588	GGCTGTTAACCGAAAGGTTGG	0.07141622	0.07808	0	0
tsrna-10586	GGCTGTTAACCGAAAGGTT	0	0	0	0.111027
tsrna-10584	GGCTGTATAGCTCAGTGGTAGAGCATTTG	0.14283243	0.15616	0	0
tsrna-10581	GGCTGGTTAGCTCAGTTGGTTAGAGC	0	0.15616	0	0
tsrna-10575	GGCTGAGTGAAGCATTGGACTGTAAATCT	0	0	0.139694	0.111027
tsrna-10574	GGCTGAGTGAAGCATTGGACTGTAAATC	0.07141622	0.07808	0.069847	0
tsrna-10573	GGCTGAGTGAAGCATTGGACTGTAAAT	0.14283243	0.07808	0.069847	0.111027
tsrna-10572	GGCTGAGTGAAGCATTGGACTGTAAA	0.07141622	0.15616	0.069847	0
tsrna-10571	GGCTGAGTGAAGCATTGGACTGTAA	0	0.07808	0	0.222054
tsrna-10570	GGCTGAGTGAAGCATTGGACTGTA	0	0.07808	0	0
tsrna-10569	GGCTGAGTGAAGCATTGGACTGT	0	0.07808	0	0
tsrna-10568	GGCTGAGTGAAGCATTGGACTG	0	0	0	0
tsrna-10558	GGCTCTGTGGCGCAATGGATAGCGCATTGGACT	0.49991351	0.780801	0.139694	0.333081
tsrna-10557	GGCTCTGTGGCGCAATGGATAGCGCATTGG	0.49991351	0.468481	0.069847	0
tsrna-10556	GGCTCTGTGGCGCAATGGATAGCGCATTG	0.07141622	0.15616	0	0
tsrna-10548	GGCTCTGTGGCGCAATGGAT	0	0	0.069847	0
tsrna-10544	GGCTCTATGGCTTAGTTGGTTAAAGC	0	0.15616	0	0
tsrna-10542	GGCTCTATGGCTTAGTTGG	0	0.07808	0	0
tsrna-10541	GGCTCGTTGGTCTAGTGGTATGATTCTCGC	0.4284973	0.07808	0.069847	0.555136
tsrna-10540	GGCTCGTTGGTCTAGTGGTATGATTCTCG	0	0	0.069847	0
tsrna-10539	GGCTCGTTGGTCTAGTGGTATGATTCTC	0	0.07808	0	0
tsrna-10537	GGCTCGTTGGTCTAGTGGTATGATTC	0	0	0.069847	0
tsrna-10526	GGCTCGTTGGTCTAGGGGTATGATTCTCGGTTT	0.4284973	0.468481	0.139694	0.444109
tsrna-10525	GGCTCGTTGGTCTAGGGGTATGATTCTCGGTT	0.07141622	0.23424	0.139694	0.111027

tsrna-10524	GGCTCGTTGGTCTAGGGGTATGATTCTCGGT	0.07141622	0.07808	0.139694	0.222054
tsrna-10523	GGCTCGTTGGTCTAGGGGTATGATTCTCGG	0.21424865	0	0.069847	0.222054
tsrna-10522	GGCTCGTTGGTCTAGGGGTATGATTCTCGCTTT	3.64222704	4.840967	5.727438	6.550601
tsrna-10521	GGCTCGTTGGTCTAGGGGTATGATTCTCGCTTC	5.42763245	4.919047	5.797285	5.662384
tsrna-10520	GGCTCGTTGGTCTAGGGGTATGATTCTCGCTT	3.71364325	4.684807	4.470196	5.662384
tsrna-10519	GGCTCGTTGGTCTAGGGGTATGATTCTCGCT	3.9278919	4.528646	3.143106	6.328547
tsrna-10518	GGCTCGTTGGTCTAGGGGTATGATTCTCGC	0.57132973	0.936961	0.279387	0.77719
tsrna-10517	GGCTCGTTGGTCTAGGGGTATGATTCTCG	0	0	0	0
tsrna-10516	GGCTCGTTGGTCTAGGGGTATGATTCTC	0.14283243	0	0	0
tsrna-10515	GGCTCGTTGGTCTAGGGGTATGATTCT	0	0	0	0
tsrna-10514	GGCTCGTTGGTCTAGGGGTATGATTC	0.07141622	0	0	0
tsrna-10513	GGCTCGTTGGTCTAGGGGTATGATT	0	0.07808	0	0
tsrna-10500	GGCTCCGTGGCTTAGCTGGTT	0.07141622	0	0	0
tsrna-10494	GGCTCCGTGGCGCAATGGATAGCGCATTGGACT	0.57132973	1.093122	0.20954	0.222054
tsrna-10493	GGCTCCGTGGCGCAATGGATAGCGCATTGGAC	0.92841081	0.858881	0.279387	0
tsrna-10492	GGCTCCGTGGCGCAATGGATAGCGCATTGG	0.14283243	0.468481	0.069847	0
tsrna-10491	GGCTCCGTGGCGCAATGGATAGCGCATTG	0.14283243	0	0	0
tsrna-10487	GGCTCCGTGGCGCAATGGATAGCGC	0.07141622	0	0	0
tsrna-10485	GGCTCCGTGGCGCAATGGATAGC	0	0.15616	0	0
tsrna-10483	GGCTCCGTGGCGCAATGGATA	0.07141622	0	0	0
tsrna-10482	GGCTCCGTGGCGCAATGGAT	0	0.07808	0	0
tsrna-10477	GGCTCCGTAGCTTAGTTGGTTAAAGC	0	0	0.069847	0
tsrna-10475	GGCTCCATAGCTCAGTGGTTAGAGCACTGG	0.28566487	0.31232	0.20954	0
tsrna-10474	GGCTCCATAGCTCAGTGGTTAGAGCA	0.4284973	0.07808	0.069847	0
tsrna-10473	GGCTCCATAGCTCAGTGGTTAGAGC	0.07141622	0.07808	0	0.111027
tsrna-10472	GGCTCCATAGCTCAGTGGTTAGAG	0.21424865	0	0.069847	0
tsrna-10471	GGCTCCATAGCTCAGTGGTTAGA	0.14283243	0	0	0
tsrna-10470	GGCTCCATAGCTCAGTGGT	0.14283243	0.07808	0.069847	0
tsrna-10469	GGCTCCATAGCTCAGTGG	0.14283243	0.07808	0	0
tsrna-10468	GGCTCCATAGCTCAGGGGTTAGAGCACTGGTCTT	0.21424865	0.702721	0.628621	1.110271
tsrna-10467	GGCTCCATAGCTCAGGGGTTAGAGCACTGGTCT	0.35708108	0.31232	0.139694	0.666163
tsrna-10466	GGCTCCATAGCTCAGGGGTTAGAGCACTGGTC	0.21424865	0.23424	0.139694	0.666163
tsrna-10465	GGCTCCATAGCTCAGGGGTTAGAGCACTGG	0.21424865	0	0	0
tsrna-10464	GGCTCCATAGCTCAGGGGTTAGAGCACTG	0	0.07808	0	0
tsrna-10463	GGCTCCATAGCTCAGGGGTTAGAGCACT	0	0	0	0
tsrna-10461	GGCTCCATAGCTCAGGGGTTAGAGCA	0.07141622	0	0	0
tsrna-10460	GGCTCCATAGCTCAGGGGTTAGAGC	0.07141622	0	0.069847	0
tsrna-10459	GGCTCCATAGCTCAGGGGTTAGAG	0	0.07808	0	0
tsrna-10457	GGCTCCATAGCTCAGGGGTTAG	0	0	0	0
tsrna-10455	GGCTCCATAGCTCAGGGGT	0.07141622	0	0	0
tsrna-10453	GGCTCCATAGCTCAGGG	0	0	0	0.111027
tsrna-10449	GGCTCCAGTCTCTTCGGGGGCGTGGGTTCG	0	0.468481	0	0
tsrna-10448	GGCTCCAGTCTCTTCGGGGGCGTGGGTTC	0.14283243	0.23424	0	0.111027
tsrna-10447	GGCTCCAGTCTCTTCGGGGGCGTGGGT	0.07141622	0.15616	0	0
tsrna-10446	GGCTCCAGTCTCTTCGGGGGCGTGGG	0	0	0	0
tsrna-10444	GGCTCCAGTCTCTTCGGGGGCGTG	0.07141622	0	0	0
tsrna-10437	GGCTCCAGTCTCTTCGGAGGCGTGGGTTCG	0	0	0	0.111027
tsrna-10436	GGCTCCAGTCTCTTCGGAGGCGTGGGTTC	0	0.468481	0	0
tsrna-10435	GGCTCCAGTCTCTTCGGAGGCGTGG	0	0.15616	0	0
tsrna-10427	GGCTCCAGTCATTTCGATGGCGTGGGTTCG	0.28566487	0.390401	0.20954	0
tsrna-10426	GGCTCCAGTCATTTCGATGGCGTGG	0	0.07808	0	0
tsrna-10423	GGCTCACATCACCCCATAAACACCA	0.35708108	0.23424	0.139694	0.222054
tsrna-10422	GGCTCACATCACCCCATAAACACC	0	0.07808	0	0
tsrna-10413	GGCTAGGATTCGGCGCTTTC	0.07141622	0.23424	0	0
tsrna-10412	GGCTAGGATTCGGCGCTTT	0.14283243	0.31232	0.20954	0
tsrna-10411	GGCTAGGATTCGGCGCTT	0	0.07808	0	0.111027
tsrna-10410	GGCTAGGATTCGGCGCT	0	0.15616	0	0.222054
tsrna-10409	GGCTAGGATTCGGCGC	0	0.15616	0	0
tsrna-10408	GGCTAGCTCAGTCGGTAGAGCATGGGACTCT	1.42832433	1.639682	0.838162	0.555136
tsrna-10407	GGCTAGCTCAGTCGGTAGAGCATGGGACTC	1.07124325	0.858881	0.558774	0.111027
tsrna-10406	GGCTAGCTCAGTCGGTAGAGCATGGGACT	1.07124325	1.249282	0.139694	0.222054
tsrna-10405	GGCTAGCTCAGTCGGTAGAGCATGGGAC	0.8569946	1.093122	0.139694	0
tsrna-10404	GGCTAGCTCAGTCGGTAGAGCATGGGA	0.92841081	0.546561	0.558774	0.111027
tsrna-10403	GGCTAGCTCAGTCGGTAGAGCATGGG	0.21424865	0.390401	0.279387	0

tsrna-10402	GGCTAGCTCAGTCGGTAGAGCATGG	0.28566487	0.15616	0	0
tsrna-10401	GGCTAGCTCAGTCGGTAGAGCATGAGACTC	0.8569946	2.264323	0.279387	0.77719
tsrna-10400	GGCTAGCTCAGTCGGTAGAGCATGAGACT	0.78557838	1.405442	0.419081	0.222054
tsrna-10399	GGCTAGCTCAGTCGGTAGAGCATGAGAC	0.64274595	2.186243	0.419081	0.77719
tsrna-10398	GGCTAGCTCAGTCGGTAGAGCATGAGA	0.4284973	0.624641	0.139694	0
tsrna-10397	GGCTAGCTCAGTCGGTAGAGCATGAG	0	0.07808	0	0
tsrna-10396	GGCTAGCTCAGTCGGTAGAGCATGA	0.07141622	0.23424	0	0
tsrna-10395	GGCTAGCTCAGTCGGTAGAGCATG	0	0.07808	0	0
tsrna-10394	GGCTAGCTCAGTCGGTAGAGCAT	0	0	0.069847	0
tsrna-10391	GGCTAGCTCAGTCGGTAGAG	0	0	0.069847	0
tsrna-10382	GGCGTGGGTTCGAATCCCACTTCTGACAC	0.07141622	0	0	0.111027
tsrna-10376	GGCGGTCTAGTGGTTAGGATTCGGCGCTCT	3.71364325	4.606726	1.676323	1.332326
tsrna-10375	GGCGGTCTAGTGGTTAGGATTCGGCGCT	2.42815136	4.528646	1.606477	2.109516
tsrna-10374	GGCGGTCTAGTGGTTAGGATTCGGCGC	1.42832433	2.108163	1.117549	0.666163
tsrna-10373	GGCGGTCTAGTGGTTAGGATTCGGCG	0.99982703	1.093122	0.488928	0.77719
tsrna-10372	GGCGGTCTAGTGGTTAGGATTCGGC	0.8569946	1.249282	0.279387	0.333081
tsrna-10371	GGCGGTCTAGTGGTTAGGATT	0.28566487	1.327362	0.349234	0.111027
tsrna-10370	GGCGGTCTAGTGGTTAGG	0.35708108	0.702721	0.20954	0.111027
tsrna-10369	GGCGGCCCGGGTTCGACTCCCGGTGTGGGAACC	0.49991351	1.093122	0.069847	0.111027
tsrna-10368	GGCGGCCCGGGTTCGACTCCCGGTGTGGG	0.07141622	0.23424	0	0
tsrna-10367	GGCGGCCCGGGTTCGACTCCCGGTGTGG	0	0.07808	0	0
tsrna-10364	GGCGGCCCGGGTTCGACTCCCGGTG	0	0	0	0
tsrna-10357	GGCGCTTTCACCGCCGCGGCCCG	0	0.15616	0	0.111027
tsrna-10356	GGCGCTCTCACCGCCGCGGCCCGGGTT	0	0	0.069847	0
tsrna-10355	GGCGCTCTCACCGCCGCGGCCCGGG	0.07141622	0	0	0
tsrna-10354	GGCGCTCTCACCGCCGCGGCCCGG	0	0.07808	0	0
tsrna-10353	GGCGCTCTCACCGCCGCGGCCCG	0	0.07808	0	0
tsrna-10352	GGCGCTCTCACCGCCGCGGCCC	0	0.07808	0	0
tsrna-10350	GGCGCGGTGGCCAAGTGGTAAGGCGTCGGTCT	0	0.07808	0	0
tsrna-10349	GGCGCGGTGGCCAAGTGGTAAGGCGTCGGT	0	0	0	0
tsrna-10348	GGCGCGGTGGCCAAGTGGTAAGGCGTCGG	0	0	0	0
tsrna-10347	GGCGCGGTGGCCAAGTGGTAAGGCGTCG	0	0.07808	0	0
tsrna-10346	GGCGCGGTGGCCAAGTGGTAAGGCGTC	0	0	0	0
tsrna-10344	GGCGCGGTGGCCAAGTGGTAAGGCG	0	0	0	0
tsrna-10342	GGCGCGGTGGCCAAGTGGTAAGG	0	0	0	0
tsrna-10341	GGCGCGGTGGCCAAGTGGTAAG	0	0	0	0
tsrna-10331	GGCGCCGTGGCTTAGTTGGTTAA	0	0	0	0.111027
tsrna-10330	GGCGCCGTGGCTTAGTTGGTTA	0	0	0	0.111027
tsrna-10329	GGCGCCGTGGCTTAGTTGGTT	0	0	0.069847	0
tsrna-10322	GGCGCCGTGGCTTAGCTGGT	0.07141622	0	0	0
tsrna-10321	GGCGCCGTGGCTTAGCTGG	0	0	0.069847	0
tsrna-10318	GGCGCAGCGGAAGCGTGCTGGGCCC	0.35708108	3.904006	0.558774	1.110271
tsrna-10317	GGCGCAGCGGAAGCGTGCTGGGCC	0.35708108	2.420483	0	0.111027
tsrna-10316	GGCGCAGCGGAAGCGTGCTGGGC	0.21424865	2.420483	0.139694	0.111027
tsrna-10315	GGCGCAGCGGAAGCGTGCTGGG	0.14283243	1.873923	0	0.111027
tsrna-10314	GGCGCAATGGATAGCGCATTGGA	0.28566487	1.171202	0.139694	0
tsrna-10313	GGCGCAATGGATAGCGCATTGG	0.21424865	0.936961	0	0
tsrna-10312	GGCGCAATGGATAGCGCATTG	0.07141622	0.07808	0	0
tsrna-10311	GGCGCAATGGATAACGCGTCTGACTACGG	0.07141622	0.468481	0	0
tsrna-10310	GGCGCAACGGTAGCGCGTCTGACTCCAG	0.07141622	0.15616	0.069847	0.111027
tsrna-10306	GGCCTCGTGGCGCAACGGTAGCGCGTCTGACTCC	0.07141622	0.780801	0.069847	0.333081
tsrna-10305	GGCCTCGTGGCGCAACGGTAGCGCGTCTGACT	0	0.07808	0	0.111027
tsrna-10304	GGCCTCGTGGCGCAACGGTAGCGCGTCTG	0	0	0.069847	0
tsrna-10303	GGCCTCGTGGCGCAACGGTAGCGC	0	0	0	0
tsrna-10298	GGCCTCGTGGCGCAACGGT	0	0	0	0
tsrna-10294	GGCCTCCTAAGCCAGGGATTGTGGGT	10.4981838	29.12388	5.448051	4.774167
tsrna-10293	GGCCTCCTAAGCCAGGGATTGTGG	0.07141622	0	0	0
tsrna-10291	GGCCTAATGGATAAGGCATTG	0.21424865	0.07808	0.069847	0
tsrna-10290	GGCCTAATGGATAAGGCATCAGCCTC	0.28566487	0.07808	0.069847	0
tsrna-10289	GGCCTAATGGATAAGGCATCAGCCT	0.21424865	0.546561	0.069847	0
tsrna-10288	GGCCTAATGGATAAGGCATCAG	0.14283243	0.07808	0.349234	0.222054
tsrna-10283	GGCCGTGATCGTATAGTGGTTAGTACTCTGCGTTGTG	2.64240001	1.171202	1.53663	1.665407
tsrna-10282	GGCCGTGATCGTATAGTGGTTAGTACTCTGCGTTGT	2.99948109	1.015041	1.885864	0.999244
tsrna-10281	GGCCGTGATCGTATAGTGGTTAGTACTCTGCGTTG	1.85682163	0.702721	1.187396	0.222054

tsrna-10280	GGCCGTGATCGTATAGTGGTTAGTACTCTGCGTT	2.07107028	1.249282	1.257243	0.888217
tsrna-10279	GGCCGTGATCGTATAGTGGTTAGTACTCTGCGT	0.14283243	0.31232	0.419081	0.333081
tsrna-10278	GGCCGTGATCGTATAGTGGTTAGTACTCTGCG	0.4284973	0.07808	0.279387	0
tsrna-10277	GGCCGTGATCGTATAGTGGTTAGTACTCTGC	0	0.31232	0.139694	0.111027
tsrna-10276	GGCCGTGATCGTATAGTGGTTAGTACTCTG	0	0.23424	0.069847	0
tsrna-10275	GGCCGTGATCGTATAGTGGTTAGTACTCT	0.07141622	0	0.139694	0.111027
tsrna-10274	GGCCGTGATCGTATAGTGGTTAGTACTC	0	0.15616	0.069847	0
tsrna-10273	GGCCGTGATCGTATAGTGGTTAGTACT	0	0.07808	0.069847	0
tsrna-10271	GGCCGTGATCGTATAGTGGTTAGTA	0	0	0	0
tsrna-10269	GGCCGTGATCGTATAGTGGTTAG	0	0	0	0
tsrna-10264	GGCCGTGATCGTATAGTG	0	0	0	0
tsrna-10263	GGCCGTGATCGTATAGT	0	0.07808	0	0
tsrna-10261	GGCCGGTTAGCTCAGTTGGTTAGAGCGTGGTGCT	0.14283243	0.15616	0.139694	0.111027
tsrna-10260	GGCCGGTTAGCTCAGTTGGTTAGAGCGTGG	0.07141622	0.15616	0	0.111027
tsrna-10259	GGCCGGTTAGCTCAGTTGGTTAGAGCGTG	0.07141622	0	0	0.111027
tsrna-10258	GGCCGGTTAGCTCAGTTGGTTAGAGC	0.07141622	0.23424	0	0
tsrna-10257	GGCCGGTTAGCTCAGTTGGTTAGAG	0.14283243	0	0	0.111027
tsrna-10256	GGCCGGTTAGCTCAGTTGGTTAGA	0	0.07808	0.069847	0.111027
tsrna-10255	GGCCGGTTAGCTCAGTTGGTTAG	0.07141622	0.07808	0	0.111027
tsrna-10254	GGCCGGTTAGCTCAGTTGGTTA	0	0.07808	0	0
tsrna-10253	GGCCGGTTAGCTCAGTTGGTT	0.07141622	0	0.069847	0
tsrna-10252	GGCCGGTTAGCTCAGTTGGTCAGAGCGTGG	0	0	0	0.111027
tsrna-10251	GGCCGGTTAGCTCAGTTGGTCAGAGC	0.07141622	0	0	0
tsrna-10250	GGCCGGTTAGCTCAGTTGGTCAGAG	0.07141622	0	0	0
tsrna-10249	GGCCGGTTAGCTCAGTTGGTCAGA	0.07141622	0.15616	0	0
tsrna-10248	GGCCGGTTAGCTCAGTTGGTC	0.07141622	0.07808	0.069847	0
tsrna-10247	GGCCGGTTAGCTCAGTTGGTAAGAGC	0.07141622	0	0.069847	0
tsrna-10246	GGCCGGTTAGCTCAGTTGGTAAGA	0.14283243	0.23424	0	0
tsrna-10245	GGCCGGTTAGCTCAGTTGGTA	0.14283243	0	0.069847	0
tsrna-10244	GGCCGGTTAGCTCAGTTGGT	0.07141622	0	0	0
tsrna-10243	GGCCGGTTAGCTCAGTTGG	0.14283243	0.15616	0.069847	0
tsrna-10242	GGCCGGTTAGCTCAGTTG	0	0	0.069847	0
tsrna-10241	GGCCGGTTAGCTCAGTT	0.07141622	0.07808	0	0
tsrna-10240	GGCCGGTTAGCTCAGTCGGCTAGAGCGTGG	0.35708108	0.23424	0.139694	0
tsrna-10239	GGCCGGTTAGCTCAGTCGGCTAGAGC	0.14283243	0.07808	0.069847	0
tsrna-10238	GGCCGGTTAGCTCAGTCGGCTAGA	0.21424865	0.07808	0.069847	0.111027
tsrna-10237	GGCCGGTTAGCTCAGTCGGC	0	0	0	0
tsrna-10236	GGCCGGTTAGCTCAGTCGG	0.07141622	0	0	0.111027
tsrna-10234	GGCCGGTTAGCTCAGT	0.07141622	0	0	0
tsrna-10233	GGCCGCGTGGCCTAATGGATAAGGCGTCTGATT	0.07141622	0.15616	0	0.111027
tsrna-10232	GGCCGCGTGGCCTAATGGATAAGGCGTCTG	0	0	0.069847	0
tsrna-10217	GGCCGCAGCAACCTCGGTTCG	0	0.07808	0	0
tsrna-10208	GGCCGAGCGGTCTAAGGCGCTGGATT	0	0.07808	0.069847	0.222054
tsrna-10206	GGCCGAGCGGTCTAAGGCGCTGCGTTCAG	0	0.15616	0	0.111027
tsrna-10201	GGCCGAGCGGTCTAAGGCGCTG	0	0.07808	0	0
tsrna-10200	GGCCGAGCGGTCTAAGGCGCT	0	0	0	0
tsrna-10193	GGCCCTGTGGCTTAGC	0	0	0	0.111027
tsrna-10192	GGCCCTATAGCTCAGGGGTTAGAGCACTGG	0.07141622	0.07808	0	0
tsrna-10191	GGCCCTATAGCTCAGGGGTTAGAGCACTG	0	0.07808	0	0.333081
tsrna-10190	GGCCCTATAGCTCAGGGGTTAGAGCACT	0.07141622	0.07808	0	0
tsrna-10187	GGCCCTATAGCTCAGGGGTTAGAGC	0	0	0	0
tsrna-10183	GGCCCTATAGCTCAGGGGTT	0	0	0	0.111027
tsrna-10178	GGCCCGGGTTCGATTCCCGGTCAGGGAACC	0.07141622	0.780801	0	0
tsrna-10177	GGCCCGGGTTCGATTCCCGGTCAGGGAAC	0	0.390401	0	0
tsrna-10176	GGCCCGGGTTCGATTCCCGGTCAGGGAA	0	0.15616	0	0
tsrna-10175	GGCCCGGGTTCGATTCCCGGTCAGGGA	0	0.23424	0	0
tsrna-10174	GGCCCGGGTTCGATTCCCGGTCAGGG	0	0.07808	0	0
tsrna-10171	GGCCCGGGTTCGATTCCCGGCCAATGCACC	0.14283243	1.093122	0.069847	0
tsrna-10164	GGCCCGGGTTCGACTCCCGGTGTGGGAACC	0.21424865	0.858881	0	0.222054
tsrna-10163	GGCCCGGGTTCGACTCCCGGTGTGGGAAC	0	0.15616	0.139694	0
tsrna-10162	GGCCCGGGTTCGACTCCCGGTGTGGGAA	0	0.07808	0	0
tsrna-10161	GGCCCGGGTTCGACTCCCGGTGTGGG	0	0	0.139694	0
tsrna-10160	GGCCCGGGTTCGACTCCCGGTGTGG	0.07141622	0	0	0
tsrna-10159	GGCCCGGGTTCGACTCCCGGTATGGGAAC	0	0.23424	0	0

tsrna-10158	GGCCCGGGTTCGACTCCCGGTATGGGA	0	0.15616	0	0
tsrna-10157	GGCCCGGGTTCGACTCCCGGTATGG	0	0.07808	0	0
tsrna-10154	GGCCCCGGGTTCGATCCCCGGCATCTCCAC	0.07141622	0.07808	0	0.111027
tsrna-10149	GGCCCCATGGTGTAATGGTTAGCACTCTGGACTTTG	0.14283243	0.23424	0.069847	0
tsrna-10148	GGCCCCATGGTGTAATGGTTAGCACTCTGGACT	0.07141622	0.15616	0.139694	0
tsrna-10147	GGCCCCATGGTGTAATGGTTAGCACTCTGGAC	0.07141622	0.15616	0.069847	0
tsrna-10144	GGCCCCATGGTGTAATGGTTAGCACTCTG	0	0.07808	0	0
tsrna-10141	GGCCCCATGGTGTAATGGTTAGCACT	0	0	0	0.111027
tsrna-10133	GGCCCCATGGTGTAATGGTCAGC	0	0.07808	0	0
tsrna-10127	GGCCCATACCCCGAAAATGTTGGTTATACC	0.07141622	0	0	0
tsrna-10116	GGCCCATAACCCAGAGGTCGATGGAT	0.07141622	0.23424	0	0.111027
tsrna-10115	GGCCCATAACCCAGAGGTCGATGG	0.07141622	0.07808	0	0
tsrna-10114	GGCCCATAACCCAGAGGTCGATG	0.07141622	0.15616	0	0
tsrna-10113	GGCCCATAACCCAGAGGTCGAT	0	0.07808	0	0
tsrna-10106	GGCATTGGTGGTTCAGTGGTAGAATTCTCGCCT	306.304152	48.48775	349.1642	35.30663
tsrna-10105	GGCATTGGTGGTTCAGTGGTAGAATTCTCG	3.71364325	1.873923	3.771728	5.329303
tsrna-10103	GGCATCAGCCTCCGGAGCTGGGGATTGTGG	0	0.07808	0	0
tsrna-10097	GGCATAGCTCAGTGGTAGAGCATTTGACTG	0.35708108	0.15616	0.069847	0
tsrna-10096	GGCATAGCTCAGTGGTAGAGCATTTGACT	0.07141622	0.15616	0.139694	0
tsrna-10095	GGCAGCGATGGCCGAGTGG	0.07141622	0.23424	0	0.111027
tsrna-10092	GGCAGAGCCCGGTAATCGCATA	0	0	0.069847	0
tsrna-10091	GGCAGAGCCCGGTAATCGCA	0	0.07808	0	0
tsrna-10087	GGCAGAGCATTTGACTG	0	0	0.069847	0
tsrna-10085	GGCACGGAGAATTTTGGATTCTCAGGGATG	0	0.468481	0.069847	0
tsrna-10084	GGATTTAGGCTCCAGTCTCTTCGGA	0.07141622	0	0	0
tsrna-10078	GGATTCTCAGGGATGGGTTCG	0	0.07808	0.069847	0
tsrna-10077	GGATTCTCAGGGATGGGTTC	0.07141622	0	0.069847	0
tsrna-10076	GGATTCGGCGCTCTCACCGCCGCGGCCCGGGT	0	0.15616	0.069847	0
tsrna-10075	GGATTCGGCGCTCTCACCGCCGCGGCCCGG	0	0.546561	0	0
tsrna-10074	GGATTCGGCGCTCTCACCGCCGCGGCCC	0	0.31232	0	0
tsrna-10073	GGATTCGGCGCTCTCACCGCCGCGGCC	0.07141622	0.468481	0.069847	0.111027
tsrna-10072	GGATTCGGCGCTCTCACCGCCGCGG	0	0	0	0
tsrna-10071	GGATTCGGCGCTCTCACCGCCGCG	0	0.07808	0	0
tsrna-10070	GGATTCGGCGCTCTCACCGCCGC	0	0.23424	0.139694	0
tsrna-10069	GGATTCGGCGCTCTCACCGCCG	0	0.07808	0	0
tsrna-10068	GGATTCGGCGCTCTCACCGCC	0	0.23424	0.139694	0.111027
tsrna-10067	GGATTCGGCGCTCTCACCGC	0	0.07808	0	0
tsrna-10066	GGATTCGGCGCTCTCACCG	0.07141622	0.23424	0	0
tsrna-10065	GGATTCGGCGCTCTCACC	0	0.07808	0	0
tsrna-10062	GGATTCCTGGTTTTCACCCAGGC	0	0.23424	0	0
tsrna-10061	GGATTCCTGGTTTTCACCCAG	0.14283243	0.15616	0.069847	0
tsrna-10060	GGATTCCTGGTTTTCACCC	0.07141622	0.07808	0	0
tsrna-10059	GGATTCCTGGTTTTCACC	0	0.07808	0	0
tsrna-10056	GGATTAGCTCAAATGGTAGAGCGCTCGCTT	0	0.07808	0	0
tsrna-10051	GGATGTAGCTCAGTGGTAGAGCGCATGCTT	0	0.07808	0	0
tsrna-10048	GGATGTAGCTCAGTGGTAGA	0	0	0	0.111027
tsrna-10047	GGATGTAGCTCAGTGGTAG	0	0	0.069847	0
tsrna-10041	GGATGGGGTGTGATAGGTGGCACGGAGA	0	0.07808	0	0
tsrna-10038	GGATGGGGTGTGATAGGTGGCACGG	0	0	0	0
tsrna-10037	GGATGGGGTGTGATAGGTGGCACG	0	0.07808	0	0
tsrna-10027	GGATGGCCGAGTGGTCTAAGGCGCCAGAC	0.07141622	0	0	0
tsrna-10021	GGATGGCCGAGCGGTCTAAGGCGCTGCG	0	0.15616	0	0
tsrna-10020	GGATGGCCGAGCGGTCTAAGGCGCTGC	0.07141622	0	0	0.111027
tsrna-10017	GGATGGCCGAGCGGTCTAAGGCG	0	0.07808	0	0
tsrna-10011	GGATGGAGGCGTGGGTTCGAA	0	0.07808	0	0
tsrna-10009	GGATGGAGGCGTGGGTTC	0.07141622	0	0	0
tsrna-10006	GGATGCGAGAGGTCCCGGGTT	0.14283243	0.15616	0	0
tsrna-10005	GGATGCGAGAGGTCCCGGGT	0	0.15616	0	0.111027
tsrna-10004	GGATCGATGCCCGCATTCTCCACCA	0.28566487	0.23424	0.069847	0
tsrna-10003	GGATCGATGCCCGCATCCTCCACCA	0.07141622	0.23424	0.069847	0
tsrna-10002	GGATCGATGCCCGCATCCTCCACC	0	0.07808	0	0
tsrna-10001	GGATCGATGCCCGCATCCTCCAC	0	0.07808	0	0
tsrna-10000	GGATCGAAACCATCCTCTGCTACCA	0.99982703	1.717762	0.628621	0.666163
tsrna-09999	GGATCGAAACCATCCTCTGCTACC	0.21424865	0.390401	0.139694	0

tsrna-09996	GGATCGAAACCATCCTCTGCT	0	0	0	0
tsrna-09993	GGATCAGAAGATTGCAGGTTCGAGTCCTGCCGCGGTCGCCA	0.07141622	0.390401	0.069847	0.111027
tsrna-09988	GGATCAGAAGATTGAGGGTTCGAGTCCCTT	0.21424865	1.015041	0.20954	0.111027
tsrna-09987	GGATCAGAAGATTGAGGGTTCGAG	0.4284973	0.780801	0.349234	0
tsrna-09986	GGATCAGAAGATTGAGGGTTCG	0.4284973	0.546561	0.279387	0.333081
tsrna-09985	GGATCAGAAGATTGAGGGTTC	0.21424865	0.780801	0.069847	0.111027
tsrna-09984	GGATCAGAAGATTGAGGGTT	0.28566487	0.390401	0.20954	0.222054
tsrna-09983	GGATCAGAAGATTGAGGGT	0.28566487	0.15616	0.139694	0
tsrna-09981	GGATCAGAAGATTCTAGGTTC	0.07141622	0.624641	0	0.111027
tsrna-09979	GGATCAGAAGATTCCAGGTTCG	0.14283243	0.07808	0.069847	0
tsrna-09978	GGATCAGAAGATTCCAGGTTC	0	0.390401	0.139694	0
tsrna-09977	GGATAGCTCAGTTGGTAGAGCATCAGAC	0	0.15616	0.139694	0.222054
tsrna-09976	GGATAGCTCAGTCGGTAGAGCATCAGACTTTT	1.99965406	1.405442	1.396936	2.775678
tsrna-09975	GGATAGCTCAGTCGGTAGAGCATCAGACTTT	1.14265946	0.780801	0.628621	0.555136
tsrna-09974	GGATAGCTCAGTCGGTAGAGCATCAGACTT	0.57132973	1.015041	0.698468	0.111027
tsrna-09973	GGATAGCTCAGTCGGTAGAGCATCAGACT	0.49991351	0.780801	0.419081	0.222054
tsrna-09972	GGATAGCTCAGTCGGTAGAGCATCAGAC	0.78557838	0.936961	0.419081	0.111027
tsrna-09971	GGATAGCTCAGTCGGTAGAGCATCAGA	0.57132973	1.015041	0.279387	0.111027
tsrna-09970	GGATAGCTCAGTCGGTAGAGCATCAG	0.49991351	0.468481	0.069847	0
tsrna-09969	GGATAGCTCAGTCGGTAGAGCATC	0.07141622	0.390401	0.139694	0
tsrna-09967	GGATAGCTCAGTCGGTAGAGCA	0.07141622	0.07808	0	0
tsrna-09965	GGATAGCTCAGTCGGTAGAG	0	0	0.069847	0
tsrna-09962	GGATAGCGCATTGGACT	0.07141622	0.15616	0	0
tsrna-09961	GGATAGCGCATTGGAC	0	0.15616	0	0
tsrna-09954	GGATAAGGCGTCTGACTTC	0	0.07808	0	0
tsrna-09953	GGATAAGGCGTCTGACTT	0	0	0	0
tsrna-09950	GGATAAGGCATCAGCCTCCGGAGCTGGGGATTGT	0	0	0	0.222054
tsrna-09947	GGATAAGGCATCAGCCTC	0	0	0	0
tsrna-09946	GGATAAGGCATCAGCCT	0	0.07808	0	0
tsrna-09943	GGATAACGCGTCTGACT	0	0	0.069847	0
tsrna-09942	GGATAACAGCTATCCATTGGTCTTAGGCCC	0	0.07808	0	0
tsrna-09941	GGATAACAGCTATCCATTGGTCTTAGGCC	0	0	0	0
tsrna-09940	GGATAACAGCTATCCATTGGTCTTAGGC	0.07141622	0	0	0
tsrna-09939	GGATAACAGCTATCCATTGGTCTTAGG	0	0.15616	0	0
tsrna-09938	GGATAACAGCTATCCATTGGTCTTAG	0	0	0.069847	0
tsrna-09937	GGATAACAGCTATCCATTGGTCTTA	0.07141622	0.07808	0	0
tsrna-09936	GGATAACAGCTATCCATTGGTCTT	0	0.07808	0	0
tsrna-09935	GGATAACAGCTATCCATTGGTCT	0	0	0	0
tsrna-09929	GGAGTTAAAGACTTTTTCTCTGACCA	2.14248649	2.108163	0.977855	1.665407
tsrna-09928	GGAGTTAAAGACTTTTTCTCTGACC	0.35708108	0.624641	0.069847	0.222054
tsrna-09927	GGAGTTAAAGACTTTTTCTCTGAC	0	0.15616	0	0
tsrna-09926	GGAGTTAAAGACTTTTTCTCTGA	0	0	0	0
tsrna-09925	GGAGTTAAAGACTTTTTCTCTG	0	0	0	0
tsrna-09920	GGAGGCGTGGGTTCGAATC	0.07141622	0	0	0
tsrna-09917	GGAGCTTAAACCCCCTTATTTCTACCA	0.14283243	0.31232	0	0.111027
tsrna-09916	GGAGCTTAAACCCCCTTATTTCTACC	0	0.31232	0.069847	0
tsrna-09915	GGAGCTTAAACCCCCTTATTTCTAC	0.07141622	0	0.069847	0
tsrna-09914	GGAGCTTAAACCCCCTTATTTCTA	0.07141622	0	0	0
tsrna-09913	GGAGCTTAAACCCCCTTATTTCT	0.07141622	0.15616	0	0
tsrna-09912	GGAGCTTAAACCCCCTTATTTC	0	0.15616	0	0.111027
tsrna-09911	GGAGCTTAAACCCCCTTATTT	0	0.07808	0.069847	0
tsrna-09905	GGAGCTGGGGATTGTGGGTTCGAGTCCCATCTGGGTCGCCA	0.21424865	1.952003	0.20954	0
tsrna-09904	GGAGCTGGGGATTGTGGGTTCGAGTCCC	0.14283243	0.15616	0	0
tsrna-09903	GGAGCTGGGGATTGTGGGTTCG	0	0.15616	0	0
tsrna-09902	GGAGCTGGGGATTGTGGGTTC	0.07141622	0.07808	0	0
tsrna-09901	GGAGCTGGGGATTGTGGGTT	0	0.15616	0	0
tsrna-09900	GGAGCTGGGGATTGTGGGT	0	0.07808	0.139694	0
tsrna-09896	GGAGATTTCAACTTAACTTGACCGCTCTGACCA	6.42745948	4.684807	5.168664	1.332326
tsrna-09895	GGAGATTTCAACTTAACTTGACCGCTCTGACC	0.71416216	0.23424	0.977855	0.111027
tsrna-09894	GGAGATTTCAACTTAACTTGACCGCTCTGAC	0.14283243	0.15616	0	0
tsrna-09893	GGAGATTTCAACTTAACTTGACCGCTCTGA	0.35708108	0.07808	0.20954	0
tsrna-09892	GGAGATTTCAACTTAACTTGACCGCTCTG	0	0.07808	0.069847	0
tsrna-09891	GGAGATTTCAACTTAACTTGACCGCTCT	0.07141622	0	0	0
tsrna-09887	GGAGATTTCAACTTAACTTGACCG	0	0.07808	0	0

tsrna-09886	GGAGATTTCAACTTAACTTGACC	0	0	0.139694	0
tsrna-09883	GGAGATTTCAACTTAACTTG	0	0.07808	0	0
tsrna-09879	GGAGATGAAAACCTTTTTCCAAGGACACCA	0.07141622	0.23424	0.139694	0.111027
tsrna-09878	GGAGATGAAAACCTTTTTCCAAGGACACC	0	0.07808	0.069847	0
tsrna-09877	GGAGATGAAAACCTTTTTCCAAGGACAC	0	0	0	0
tsrna-09864	GGAGATCCTGGGTTCGAAT	0.14283243	0.546561	0.069847	0.222054
tsrna-09863	GGAGATCCTGGGTTCG	0.28566487	0.31232	0.139694	0.222054
tsrna-09856	GGAGACCGGGGTTCGATTCCCCGACGGGGAGCCA	0.21424865	1.561602	0.069847	0.111027
tsrna-09855	GGAGACCGGGGTTCGATTCCCCGACGGGGAGCC	0	0.15616	0	0
tsrna-09854	GGAGACCGGGGTTCGATTCCCCGACGGGGAGC	0	0.23424	0	0
tsrna-09853	GGAGACCGGGGTTCGATTCCCCGACGGGG	0	0	0	0
tsrna-09839	GGAGAATTTTGGATTCTCAGGGA	0	0.07808	0	0
tsrna-09837	GGACTTTGAATCCAGCGATCCGAGT	0	0.07808	0.139694	0
tsrna-09836	GGACTTTGAATCCAGCGATCCGAG	0.07141622	0.07808	0.20954	0.111027
tsrna-09835	GGACTTTGAATCCAGCGATCCGA	0.14283243	0.07808	0	0
tsrna-09834	GGACTTTGAATCCAGCGATCCG	0	0.15616	0	0
tsrna-09833	GGACTTTGAATCCAGCGATCC	0.28566487	0.15616	0	0.111027
tsrna-09832	GGACTTTGAATCCAGCGATC	0.07141622	0.390401	0	0
tsrna-09828	GGACTGTAGATCCTTAGGTCGCTGGT	0	0.07808	0	0
tsrna-09822	GGACTGTAAATCTAAAGACAGGGGTTAGGC	0	0	0.069847	0
tsrna-09814	GGACTGCTAATCCATTGTGCTCTG	0	0	0.139694	0
tsrna-09813	GGACTCTTAATCCCAGGGTCGTGGGTTC	0.07141622	0.07808	0	0
tsrna-09812	GGACTCTTAATCCCAGGGTCGTGGGTT	0	0.23424	0.069847	0
tsrna-09811	GGACTCTTAATCCCAGGGTCGTGGG	0	0.07808	0.069847	0.111027
tsrna-09810	GGACTCTTAATCCCAGGGTCGTGG	0	0.07808	0	0
tsrna-09809	GGACTCTTAATCCCAGGG	0.07141622	0	0	0
tsrna-09805	GGACTCTGAATCCAGCGATCCGAGTTCAAG	0	0.15616	0	0
tsrna-09804	GGACTCTGAATCCAGCGATCCGAGTTCAA	0	0.07808	0	0
tsrna-09803	GGACTCTGAATCCAGCGATCCGAGTTC	0.07141622	0	0	0
tsrna-09802	GGACTCTGAATCCAGCGATCCGAGTT	0	0	0.069847	0
tsrna-09800	GGACTCTGAATCCAGCGATCCGAG	0	0.07808	0.069847	0
tsrna-09799	GGACTCTGAATCCAGCGATCCGA	0	0.07808	0	0
tsrna-09798	GGACTCTGAATCCAGCGATCCG	0	0.07808	0	0
tsrna-09797	GGACTCTGAATCCAGCGATCC	0	0.15616	0	0
tsrna-09789	GGAAGCGTGCTGGGCCCATAACCCAGA	0	0.07808	0	0.333081
tsrna-09788	GGAAGCGTGCTGGGCCCATAACCCAG	0.07141622	0.390401	0	0.222054
tsrna-09787	GGAAGCGTGCTGGGCCCATAACCC	0.07141622	0.15616	0	0.111027
tsrna-09786	GGAAGCGTGCTGGGCCCATAAC	0	0.15616	0	0
tsrna-09785	GGAAGCGTGCTGGGCCCATA	0	0.15616	0	0
tsrna-09784	GGAAGCGTGCTGGGCCCAT	0	0.23424	0.069847	0
tsrna-09783	GGAAGCGTGCTGGGCCCA	0	0.15616	0	0
tsrna-09782	GGAAGCGTGCTGGGCCC	0.14283243	0.23424	0	0
tsrna-09781	GGAAGCGTGCTGGGCC	0	0.31232	0.069847	0.111027
tsrna-09780	GGAAAAAGTCATGGAGGCCATGGGGTT	5.99896218	25.45412	3.841574	2.33157
tsrna-09779	GGAAAAAGTCATGGAGGCCATGGGG	1.85682163	7.808011	1.187396	0.888217
tsrna-09778	GCTTTGGGTGCTAATGGTGGAGTTAAAGACTTTTTCTCTGAC(	1.78540541	4.997127	1.466783	1.998488
tsrna-09777	GCTTTGGGTGCTAATGGTGGAGTTAAAGACTTTTTCTCTGAC(	1.14265946	1.717762	0.768315	0
tsrna-09776	GCTTTGGGTGCTAATGGTGGAGTTAAAGACTTTTTCTCTGAC	0.8569946	1.249282	0.349234	0.111027
tsrna-09775	GCTTTGGGTGCTAATGGTGGAGTTAAAGACTTTTTC	0.35708108	1.015041	0.279387	0.222054
tsrna-09774	GCTTTGGGTGCTAATGGTGGAGTTAAAGAC	0.07141622	0.624641	0	0
tsrna-09773	GCTTTGGGTGCTAATGGTGGAGTTAAAGA	0.28566487	0.390401	0.069847	0
tsrna-09772	GCTTTGGGTGCTAATGGTGGAGTTAAAG	0.07141622	0.31232	0.069847	0.111027
tsrna-09771	GCTTTGGGTGCTAATGGTGGAGTTAAA	0.21424865	0	0.069847	0
tsrna-09770	GCTTTGGGTGCTAATGGTGGAGTTAA	0.14283243	0.23424	0.139694	0
tsrna-09769	GCTTTGGGTGCTAATGGTGGAGTTA	0.07141622	0.23424	0.139694	0
tsrna-09768	GCTTTGGGTGCTAATGGTGGAGTT	0.07141622	0.07808	0.069847	0
tsrna-09760	GCTTTGGGTGCGAGAGGTCCCGG	0	0.07808	0.069847	0
tsrna-09759	GCTTTGGGTGCGAGAGGTCCCG	0	0.07808	0	0
tsrna-09758	GCTTTGGGTGCGAGAGGTCCC	0	0.07808	0	0.111027
tsrna-09757	GCTTTGGGTGCGAGAGGTCC	0	0.15616	0	0
tsrna-09754	GCTTTGGGGGGTTCGATTCCTTCCTTTTTTGCCA	0	0.780801	0.279387	0.333081
tsrna-09753	GCTTTGGGGGGTTCGATTCCTTCCTTTTTT	0.21424865	0.31232	0.069847	0.111027
tsrna-09752	GCTTTGGGGGGTTCGATTCCTTCC	0.07141622	0	0	0
tsrna-09751	GCTTTGGGGGGTTCGATTCCTTC	0	0	0.069847	0

tsrna-09750	GCTTTGGGGGGTTCGATTCCTT	0	0	0	0.111027
tsrna-09749	GCTTTGGGGGGTTCGATTCCT	0	0.07808	0	0.111027
tsrna-09748	GCTTTGGGGGGTTCGATTCC	0	0	0.069847	0.111027
tsrna-09747	GCTTTGGGGGGTTCGATTC	0	0	0.069847	0
tsrna-09746	GCTTTGGGGGGTTCGATT	0	0.07808	0	0
tsrna-09743	GCTTTGCATGTATGAGGCCCCGGGT	0.14283243	0.546561	0.069847	0
tsrna-09740	GCTTTGCACGTATGAGGCCCCGGGTT	2.85664866	3.513605	1.53663	3.330814
tsrna-09739	GCTTTGCACGTATGAGGCCCCGGGT	1.71398919	2.654724	0.977855	2.886706
tsrna-09738	GCTTTGCACGTATGAGGCCCCGGG	0.07141622	0.15616	0	0
tsrna-09737	GCTTTGCACGTATGAGGCCCCGG	0.07141622	0.15616	0	0
tsrna-09736	GCTTTGCACGTATGAGGCCCCG	0.07141622	0.07808	0	0
tsrna-09735	GCTTTGCACGTATGAGGCCCC	0.07141622	0	0	0.111027
tsrna-09732	GCTTTGCACGCGTGGGTT	0	0.468481	0.069847	0
tsrna-09731	GCTTTGCACGCGTGGGT	0	0.15616	0	0
tsrna-09730	GCTTTCACCGCCGCGGCCCGGGT	0.07141622	0.624641	0	0.111027
tsrna-09729	GCTTTCACCGCCGCGGCCCGG	0.21424865	0	0	0.555136
tsrna-09728	GCTTTCACCGCCGCGGCCCG	0.07141622	0.23424	0	0.333081
tsrna-09727	GCTTTCACCGCCGCGGCCC	0.07141622	0.23424	0	0.222054
tsrna-09726	GCTTTCACCGCCGCGGCC	0.14283243	0	0	0
tsrna-09725	GCTTTCACCGCCGCGGC	0.14283243	0.31232	0.069847	0.111027
tsrna-09724	GCTTTCACCGCCGCGG	0.14283243	0.23424	0	0
tsrna-09723	GCTTTACACGCAGAAGGTCCTGGGT	0.64274595	3.435525	0.20954	0
tsrna-09722	GCTTTACACGCAGAAGGTCCTGG	0.07141622	0	0	0
tsrna-09721	GCTTTACACGCAGAAGGTCCTG	0.14283243	0	0	0
tsrna-09718	GCTTCTGTAGTGTAGTGGTTATCACGTTCGCCTCACACGCGA	24.0672649	11.47778	23.39868	7.549845
tsrna-09717	GCTTCTGTAGTGTAGTGGTTATCACGTTCGCCTCACACG	25.424173	8.354572	19.06818	7.549845
tsrna-09716	GCTTCTGTAGTGTAGTGGTTATCACGTTCGCCTCACA	25.5670055	9.603854	19.34757	6.883683
tsrna-09715	GCTTCTGTAGTGTAGTGGTTATCACGTTCGCCTCAC	26.7096649	9.916174	22.00174	7.216764
tsrna-09714	GCTTCTGTAGTGTAGTGGTTATCACGTTCGCCTCA	23.9958487	11.0093	18.5094	7.549845
tsrna-09713	GCTTCTGTAGTGTAGTGGTTATCACGTTCGCCTC	24.6385947	10.54081	20.39527	6.772655
tsrna-09712	GCTTCTGTAGTGTAGTGGTTATCACGTTCGCCT	21.2820325	8.276492	18.36971	7.993954
tsrna-09711	GCTTCTGTAGTGTAGTGGTTATCACGTTCGCC	18.139719	8.042251	15.15676	6.439574
tsrna-09710	GCTTCTGTAGTGTAGTGGTTATCACGTTCGC	5.71329731	4.528646	4.260655	5.107248
tsrna-09709	GCTTCTGTAGTGTAGTGGTTATCACGTTCG	2.21390271	0.546561	2.025557	3.441841
tsrna-09708	GCTTCTGTAGTGTAGTGGTTATCACGTTC	2.71381622	0.624641	1.327089	3.88595
tsrna-09707	GCTTCTGTAGTGTAGTGGTTATCACGTT	1.92823784	0.936961	1.955711	2.886706
tsrna-09706	GCTTCTGTAGTGTAGTGGTTATCACGT	2.71381622	0.546561	1.606477	2.997733
tsrna-09705	GCTTCTGTAGTGTAGTGGTTATCACG	1.71398919	0.31232	1.606477	3.219787
tsrna-09704	GCTTCTGTAGTGTAGTGGTTATCAC	2.35673514	0.624641	1.885864	2.220543
tsrna-09703	GCTTCTGTAGTGTAGTGGTTATCA	2.07107028	0.390401	0.838162	1.776434
tsrna-09702	GCTTCTGTAGTGTAGTGGTTATC	1.57115676	0.468481	1.117549	2.33157
tsrna-09701	GCTTCTGTAGTGTAGTGGTTAT	0.07141622	0	0	0
tsrna-09700	GCTTCTGTAGTGTAGTGGTTA	0.14283243	0	0	0
tsrna-09699	GCTTCTGTAGTGTAGTGGTT	0	0	0	0.111027
tsrna-09698	GCTTCTGTAGTGTAGTGGT	0	0.07808	0	0
tsrna-09692	GCTTCGCATGTATGAGGTCCCGGGT	0.28566487	0.23424	0.069847	0
tsrna-09691	GCTTCGCATGTATGAGGCCCCGGGT	0.28566487	0.390401	0.069847	0
tsrna-09690	GCTTCAAACCTGCCGGGGCTTCCA	0.35708108	0.15616	0.20954	0
tsrna-09689	GCTTCAAACCTGCCGGGGCTTCC	0.07141622	0	0	0
tsrna-09688	GCTTCAAACCTGCCGGGGCTTC	0	0	0.069847	0
tsrna-09681	GCTTAGTTGGTTAAAGCGCCTGTCT	0.28566487	0.07808	0.069847	0.555136
tsrna-09680	GCTTAGTTGGTTAAAGCGCCTGT	0.21424865	0	0.069847	0.222054
tsrna-09675	GCTTACGACCCCTTATTTACCCCA	0.14283243	0.15616	0	0
tsrna-09674	GCTTACGACCCCTTATTTACCCC	0	0	0.069847	0
tsrna-09666	GCTTACCTCCTCAAAGCAATACACTG	0.14283243	0.07808	0.20954	0
tsrna-09665	GCTTACCTCCTCAAAGCAATACACT	0.21424865	0.15616	0.069847	0.111027
tsrna-09664	GCTTACCTCCTCAAAGCAATACAC	0.07141622	0	0	0
tsrna-09663	GCTTACCTCCTCAAAGCAATACA	0	0	0.20954	0
tsrna-09661	GCTTACCTCCTCAAAGCAATA	0.07141622	0	0	0
tsrna-09659	GCTTACCTCCTCAAAGC	0.07141622	0	0	0
tsrna-09657	GCTTAATTAAAGTGGCTGATTTGC	0	0	0.139694	0
tsrna-09656	GCTTAATTAAAGTGGCTGA	0	0	0	0
tsrna-09654	GCTTAACACAAAGCACCCAACTTACACTT	0	0	0	0.111027
tsrna-09646	GCTTAAACCCCCTTATTTCTACCA	0.07141622	0	0	0

tsrna-09645	GCTTAAACCCCCTTATTTCTACC	0.07141622	0.31232	0	0
tsrna-09644	GCTTAAACCCCCTTATTTCTAC	0.07141622	0.15616	0	0
tsrna-09643	GCTTAAACCCCCTTATTTCTA	0.07141622	0	0	0
tsrna-09642	GCTTAAACCCCCTTATTTCT	0.07141622	0.15616	0.069847	0
tsrna-09641	GCTTAAACCCCCTTATTTC	0	0.07808	0	0
tsrna-09640	GCTTAAACCCCCTTATTT	0.07141622	0	0	0
tsrna-09637	GCTGTTAACTGAAAGGTTGGTGGT	0.07141622	0.07808	0	0
tsrna-09636	GCTGTTAACTAAGTGTTTGTGGGTTTAAGTCCCATTGGTCTAC	0.57132973	0.23424	0.20954	0.222054
tsrna-09635	GCTGTTAACTAAGTGTTTGTGGGTTTAAGTCCCATTGGTCTAC	0.07141622	0.23424	0.069847	0.111027
tsrna-09634	GCTGTTAACTAAGTGTTTGTGGGTTTAAGTCCCATTGGTCTAC	0.49991351	0.07808	0.349234	0
tsrna-09633	GCTGTTAACTAAGTGTTTGTGGGTTTAAGT	0	0	0	0
tsrna-09632	GCTGTTAACTAAGTGTTTGTGGGTTT	0	0.07808	0.139694	0
tsrna-09631	GCTGTTAACTAAGTGTTTGTGGGTT	0	0.07808	0.069847	0
tsrna-09630	GCTGTTAACTAAGTGTTTGTGGGT	0	0	0.069847	0
tsrna-09624	ACCTTTTTCCAAGGACACCA	0.14283243	0	0.349234	0
tsrna-09623	ACCTTTTTCCAAGGACACC	0.07141622	0	0.069847	0
tsrna-09622	ACCTTTTAAGTTAAAGATTAAGAGAACCAACACCTCTTTACAG	0	0	0.488928	0
tsrna-09621	ACCTTTTAAGTTAAAGATTAAGAGAACCA	0	0	0.069847	0
tsrna-09620	ACCTTTTAAGTTAAAGATTAAGAGAACC	0	0.15616	0	0
tsrna-09619	ACCTTCGATAGCTCAGTTGGTAGAGC	0.99982703	0.858881	0.558774	0.222054
tsrna-09618	ACCTTCGATAGCTCAGCTGGTAGAGC	3.9278919	2.498564	1.816017	2.33157
tsrna-09617	ACCTGCCGGGGCTTCCA	0.07141622	0	0.069847	0
tsrna-09616	ACCTGCCGGGGCTTCC	0	0	0	0
tsrna-09614	ACCTCTTTACAGTGACC	0	0	0	0
tsrna-09608	ACCTCGTGGCGCAACGGTAGCGCGTCTGAC	0	0.07808	0	0
tsrna-09602	ACCTCCTCAAAGCAATACACT	0.07141622	0	0	0
tsrna-09598	ACCTCCTCAAAGCAATA	0	0	0	0.111027
tsrna-09597	ACCTCAGAGGGGGCACCA	0.07141622	0.15616	0	0
tsrna-09596	ACCGTTAACTTCCAATTAACTAGTTTTGAC	0	0	0	0
tsrna-09595	ACCGGGGTTCGATTCCCCGACGGGGAGCCA	0.14283243	1.327362	0	0
tsrna-09594	ACCGGGGTTCGATTCCCCGACGGGGAGCC	0	0.07808	0	0
tsrna-09593	ACCGGGGTTCGATTCCCCGACGGGGAGC	0	0	0	0
tsrna-09592	ACCGGGGTTCGATTCCCCGACGGGGAG	0.07141622	0	0	0
tsrna-09591	ACCGGGGTTCGATTCCCCGACGGGGA	0	0.07808	0.069847	0
tsrna-09589	ACCGGGGTTCGATTCCCCGACGGG	0	0.07808	0	0
tsrna-09586	ACCGGGCGGAAACACCA	11.9265081	30.13892	3.422493	1.887461
tsrna-09585	ACCGGGCGGAAACACC	2.35673514	7.41761	1.327089	0.333081
tsrna-09584	ACCGGGCAGAAGCACCA	0.8569946	4.294406	0.488928	0.111027
tsrna-09583	ACCGGGCAGAAGCACC	0	0.390401	0	0
tsrna-09582	ACCGGGATGGCCGAGTGGTTAAGGC	0.07141622	1.561602	0.069847	1.665407
tsrna-09581	ACCGGAGATGAAAACCTTTTTCCAAGGACACCA	1.99965406	2.498564	4.190808	0.222054
tsrna-09580	ACCGGAGATGAAAACCTTTTTCCAAGGACACC	0.21424865	0.858881	0.628621	0
tsrna-09579	ACCGGAGATGAAAACCTTTTTCCAAGGACA	0.21424865	0.702721	0.279387	0
tsrna-09578	ACCGGAGATGAAAACCTTTTTCCAAGGAC	0.07141622	0.390401	0.139694	0
tsrna-09577	ACCGGAGATGAAAACCTTTTTCCAAGG	0.14283243	0.468481	0.069847	0
tsrna-09576	ACCGGAGATGAAAACCTTTTTCCAAG	0	0.390401	0	0
tsrna-09575	ACCGGAGATGAAAACCTTTTTCCAA	0.07141622	0.23424	0.20954	0
tsrna-09574	ACCGGAGATGAAAACCTTTTTCCA	0.21424865	0.390401	0	0
tsrna-09573	ACCGGAGATGAAAACCTTTTTCC	0.14283243	0.23424	0	0
tsrna-09572	ACCGGAGATGAAAACCTT	0	0	0	0
tsrna-09571	ACCGGAGATGAAAACCT	0	0	0	0
tsrna-09569	ACCGCGTGGCCTAATGGATAAGGCGTCTGA	0	0.07808	0.069847	0
tsrna-09564	ACCGCCGCGGCCCGGGTTCGATTCCCGGTCAGGGAACCA	2.35673514	24.12675	1.187396	0.444109
tsrna-09563	ACCGCCGCGGCCCGGGTTCGATTCCCGGTCAGGGAACC	0.28566487	1.093122	0.20954	0.111027
tsrna-09562	ACCGCCGCGGCCCGGGTTCGATTCCCGGTCAGGGAAC	0.14283243	0.624641	0.139694	0
tsrna-09561	ACCGCCGCGGCCCGGGTTCGATTCCCGGTCAGGGAA	0.07141622	0	0	0
tsrna-09560	ACCGCCGCGGCCCGGGTTCGATTCCCGGTCAGGGA	0	0.07808	0	0
tsrna-09559	ACCGCCGCGGCCCGGGTTCGATTCCCGGTCAGGG	0	0.07808	0	0
tsrna-09558	ACCGCCGCGGCCCGGGTTCGATTCCCGGTCAGG	0	0.07808	0	0
tsrna-09556	ACCGCCGCGGCCCGGGTTCGATTCCCGGTC	0.14283243	0.15616	0	0
tsrna-09555	ACCGCCGCGGCCCGGGTTCGATTCCCGGT	0	0	0	0.111027
tsrna-09550	ACCGCCGCGGCCCGGGTTCG	0	0	0	0
tsrna-09545	ACCGAGCGGAAACACCA	0.07141622	0	0	0
tsrna-09544	ACCGAAGATCGCGGGTTCGAACCCCGTCCGTGCCTCCA	0.35708108	1.405442	0.20954	0.111027

tsrna-09543	ACCGAAGATCGCGGGTTCGAACCCCGTCCGTGCCTCC	0.21424865	1.093122	0	0
tsrna-09542	ACCGAAGATCGCGGGTTCGAACCCCGTCCGTGCCTC	0.14283243	0.624641	0	0.111027
tsrna-09541	ACCGAAGATCGCGGGTTCGAACCCCGTCCG	0.14283243	0.546561	0.069847	0
tsrna-09540	ACCGAAGATCGCGGGTTCGA	0	0.390401	0.139694	0
tsrna-09539	ACCGAAGATCGCGGGTTCG	0.21424865	0.468481	0.20954	0.111027
tsrna-09538	ACCGAAGATCGCGGGT	0.21424865	0.23424	0.069847	0
tsrna-09537	ACCGAAGATCACGGGTTCGAACCCCGTCCGTGCCTCCA	0.4284973	0.624641	0.419081	0.222054
tsrna-09536	ACCGAAGATCACGGGTTCGAACCCCGTCCGTGCCTC	0	0	0	0
tsrna-09535	ACCGAAGATCACGGGTTCGAACCCCGTCCGTGCCT	0	0.23424	0.20954	0
tsrna-09534	ACCGAAGATCACGGGTTCGAACCCCGTCCG	0.14283243	0.23424	0	0
tsrna-09533	ACCGAAAGGTTGGTGGTTCG	0	0	0	0
tsrna-09532	ACCGAAAGGTTGGTGGTT	0.07141622	0.07808	0	0
tsrna-09528	ACCCTGGACTCTGAATCCAGC	0	0.07808	0	0
tsrna-09525	ACCCTGCTCGCTGCGCCA	1.14265946	2.498564	0.139694	0
tsrna-09524	ACCCTGCTCGCTGCGCC	0.14283243	0.31232	0	0
tsrna-09522	ACCCCTTATTTACCCCA	0	0	0.069847	0
tsrna-09520	ACCCCGTCCGTGCCTCCA	0.28566487	0.390401	0.139694	0.222054
tsrna-09517	ACCCCGTACGGCCACCA	0.14283243	0.23424	0	0.111027
tsrna-09516	ACCCCGAAAATGTTGGTTATACCCTTCCCGTACTAC	0	0	0	0
tsrna-09515	ACCCCGAAAATGTTGGTTATACCCTTCCCG	0	0	0.069847	0
tsrna-09513	ACCCCCTTATTTCTACCA	0	0	0	0.111027
tsrna-09511	ACCCCCTTATTTCTAC	0	0.07808	0	0
tsrna-09509	ACCCCACTCTCGGTACCA	0	0.23424	0	0.222054
tsrna-09505	ACCCAGTGGCCTAATGGATAAGGCATCAGC	0.21424865	0.07808	0	0.111027
tsrna-09504	ACCCAGTGGCCTAATGGATAAGGCATC	0.14283243	0.07808	0.069847	0
tsrna-09500	ACCCAGGTGGCCCGGGTTCGACTCCCGGTATGGGAACCA	1.21407568	5.699848	0.838162	0.333081
tsrna-09499	ACCCAGGTGGCCCGGGTTCGACTCCCGGTATGGGAACC	0.14283243	0.624641	0.069847	0
tsrna-09498	ACCCAGGTGGCCCGGGTTCGACTCCCGGTATGGGAAC	0.14283243	0.390401	0	0
tsrna-09497	ACCCAGGTGGCCCGGGTTCGACTCCCGGTATGGGAA	0	0.15616	0	0
tsrna-09495	ACCCAGGTGGCCCGGGTTCGACTCCCGGT	0	0.07808	0	0
tsrna-09491	ACCCAGGCGGCCCGGGTTCGACTCCCGGTGTGGGAACCA	6.28462705	25.76644	3.073259	3.663896
tsrna-09490	ACCCAGGCGGCCCGGGTTCGACTCCCGGTGTGGGAACC	0.21424865	1.483522	0.558774	0.222054
tsrna-09489	ACCCAGGCGGCCCGGGTTCGACTCCCGGTGTGGGAAC	0.07141622	0.23424	0.349234	0
tsrna-09488	ACCCAGGCGGCCCGGGTTCGACTCCCGGTGTGGGAA	0	0.15616	0	0
tsrna-09487	ACCCAGGCGGCCCGGGTTCGACTCCCGGTGTGGGA	0	0.15616	0	0
tsrna-09486	ACCCAGGCGGCCCGGGTTCGACTCCCGGTGTGGG	0	0.15616	0	0
tsrna-09478	ACCCAGGCGGCCCGGGTTCGA	0	0.07808	0	0
tsrna-09475	ACCCAGAGGTCGATGGATCGAAACC	0	0.15616	0	0
tsrna-09474	ACCCAGAGGTCGATGGATCGAAAC	0	0	0	0.111027
tsrna-09473	ACCCAGAGGTCGATGGATCGAAA	0.07141622	0.15616	0	0.111027
tsrna-09472	ACCCAGAGGTCGATGGATCGAA	0	0.07808	0	0.111027
tsrna-09471	ACCCAGAGGTCGATGGATCGA	0.14283243	0.31232	0.069847	0
tsrna-09470	ACCCAGAGGTCGATGGATCG	0.07141622	0.23424	0	0
tsrna-09469	ACCCAGAGGTCGATGGATC	0	0.23424	0	0
tsrna-09468	ACCCAGAGGTCGATGGAT	0	0.23424	0	0
tsrna-09467	ACCCAGAGGTCGATGGA	0	0.31232	0	0
tsrna-09466	ACCCAGAGGTCGATGG	0	0	0.069847	0
tsrna-09465	ACCCACCCAGAGGCGCCA	0.07141622	0	0	0
tsrna-09464	ACCCAACTTACACTTAGGAGATTTCAACTTAACTTGACCGCTC	0.21424865	0.23424	1.327089	0.111027
tsrna-09463	ACCCAACTTACACTTAGGAGATTTCAACTTAACTTGACCGCTC	0.64274595	0.15616	0.908008	0
tsrna-09462	ACCCAACTTACACTTAGGAGATTTCAACTTAACTTGACCGCTC	0.21424865	0.15616	0.838162	0
tsrna-09461	ACCCAACTTACACTTAGGAGATTTCAACTT	0.07141622	0	0	0
tsrna-09460	ACCCAACTTACACTTAGGAGATTTCAACT	0	0	0	0
tsrna-09456	ACCCAACTTACACTTAG	0	0	0	0
tsrna-09455	ACCATTTCATAACTTTGTCAAAGTT	0.28566487	0.23424	0.279387	0.222054
tsrna-09454	ACCATTTCATAACTTTGTC	0	0	0.20954	0
tsrna-09453	ACCATCCTCTGCTACCA	0.28566487	0.07808	0.069847	0.111027
tsrna-09452	ACCATCCTCTGCTACC	0	0.07808	0	0
tsrna-09451	ACCAGGGGTCGCGAGTTCAA	0	0.07808	0	0
tsrna-09450	ACCAGGGGTCGCGAGTTCA	0.21424865	0.07808	0	0
tsrna-09449	ACCAGGGGTCGCGAGTTC	0	0.15616	0	0
tsrna-09448	ACCAGGGGTCGCGAGTT	0.07141622	0.07808	0	0
tsrna-09447	ACCAGGGGTCGCGAGT	0	0	0	0
tsrna-09446	ACCAGGCGGAAACACCA	0.21424865	0.07808	0.069847	0

tsrna-09445	ACCAGGATGGCCGAGTGGTTAAGGCGTTGG	0.07141622	0.702721	0.20954	0
tsrna-09444	ACCAGGATGGCCGAGTGGTTAAGGCG	0.4284973	0.468481	0.349234	0.111027
tsrna-09443	ACCAGGATGGCCGAGTGGTTAAGGC	0.21424865	1.015041	0.349234	0.111027
tsrna-09442	ACCAGGATGGCCGAGTGGTTAAGG	0.14283243	0.780801	0.20954	0.222054
tsrna-09441	ACCAGGATGGCCGAGTGGTTAAG	0.14283243	0.390401	0.139694	0.111027
tsrna-09440	ACCAGGATGGCCGAGTGGTTAA	0.14283243	0.702721	0	0.111027
tsrna-09439	ACCAGGATGGCCGAGTGGTTA	0.21424865	0.31232	0.069847	0
tsrna-09438	ACCAGGATGGCCGAGTGGTT	0	0.624641	0.069847	0
tsrna-09437	ACCAGGATGGCCGAGTGGT	0	0.31232	0.069847	0
tsrna-09436	ACCAGGATGGCCGAGTGG	0.07141622	0.31232	0	0
tsrna-09435	ACCAGGATGGCCGAGTG	0.21424865	0.23424	0	0.111027
tsrna-09434	ACCAGGATGGCCGAGT	0.07141622	0.15616	0.069847	0
tsrna-09433	ACCAGCTTTGGGGGGTTCG	0	0	0	0
tsrna-09432	ACCAGCTTTGGGGGGTTC	0	0	0	0
tsrna-09431	ACCAGCTTTGGGGGGTT	0.07141622	0	0	0
tsrna-09430	ACCAGAATGGCCGAGTGGTTAAGGC	0	0.390401	0	0.111027
tsrna-09429	ACCAGAATGGCCGAGTGGTTAAGG	0.14283243	0.07808	0	0
tsrna-09428	ACCAGAATGGCCGAGTGGTTAAG	0.07141622	0.31232	0	0
tsrna-09427	ACCAGAATGGCCGAGTGGTTAA	0.21424865	0.23424	0	0
tsrna-09426	ACCAGAATGGCCGAGTGGTTA	0	0	0.20954	0
tsrna-09425	ACCAGAATGGCCGAGTGGTT	0.07141622	0	0.069847	0
tsrna-09424	ACCAGAATGGCCGAGTGG	0.07141622	0.07808	0.069847	0
tsrna-09423	ACCAGAATGGCCGAGTG	0	0.15616	0.069847	0
tsrna-09422	ACCAGAATGGCCGAGT	0.07141622	0.07808	0.069847	0
tsrna-09407	ACCAAAACATCAGATTGTGAATCTGACAAC	0.07141622	0.23424	0.139694	0
tsrna-09406	ACCAAAACATCAGATTGTGAATCTGACA	0	0.07808	0.069847	0
tsrna-09399	ACATGGTCTAGCGGTTAGGATTCCTGGTTT	0.4284973	0.546561	0.349234	0.222054
tsrna-09398	ACATGGTCTAGCGGTTAGGATTCCTGGTT	0.14283243	0.546561	0.139694	0.111027
tsrna-09397	ACATGGTCTAGCGGTTAGGATTCCTGGT	0.28566487	0.468481	0.20954	0.444109
tsrna-09396	ACATGGTCTAGCGGTTAGGATTCCTGG	0.21424865	0.23424	0.20954	0.222054
tsrna-09395	ACATGGTCTAGCGGTTAGGATTCCTG	0.21424865	0	0.139694	0
tsrna-09394	ACATGGTCTAGCGGTTAGGATTCCT	0.07141622	0	0.139694	0
tsrna-09393	ACATGGTCTAGCGGTTAGGATTCC	0	0	0	0
tsrna-09390	ACATGGTCTAGCGGTTAGGAT	0.07141622	0	0	0
tsrna-09386	ACATGGCTTTCTCACC	0	0	0	0
tsrna-09385	ACATCAGATTGTGAATCTGACAACAGAGGCTTACGA	0.21424865	0.624641	0.419081	0
tsrna-09384	ACATCAGATTGTGAATCTGACAACAGAGGC	0.14283243	0.468481	0.069847	0
tsrna-09383	ACATCAGATTGTGAATCTGACAACAGAGG	0.07141622	0.23424	0.279387	0
tsrna-09382	ACATCAGATTGTGAATCTGACAACAGAG	0.14283243	0.31232	0.139694	0
tsrna-09381	ACATCAGATTGTGAATCTGACAACAGA	0.14283243	0.07808	0.279387	0
tsrna-09380	ACATCAGATTGTGAATCTGACAACAG	0.14283243	0.23424	0.139694	0
tsrna-09379	ACATCAGATTGTGAATCTGACAACA	0	0.07808	0.139694	0
tsrna-09378	ACATCAGATTGTGAATCTGACAAC	0.14283243	0	0.069847	0
tsrna-09377	ACATCAGATTGTGAATCTGACAA	0.07141622	0	0.069847	0
tsrna-09376	ACATCAGATTGTGAATCTGACA	0	0.15616	0	0
tsrna-09371	ACATCACCCCATAAACACCA	0	0.15616	0	0.111027
tsrna-09366	ACATATGTCCGCGTGGGTT	0.99982703	1.171202	0.279387	0.222054
tsrna-09365	ACATATGTCCGCGTGGGT	0.57132973	0.624641	0.069847	0
tsrna-09364	ACATATGTCCGCGTGGG	0.14283243	0	0	0
tsrna-09363	ACAGGTTCGAATCCTGTTCGTGACGC	0	0	0.069847	0
tsrna-09362	ACAGGGGTTAGGCCTCTTTTTACCACCA	0.07141622	0.07808	0.069847	0.111027
tsrna-09361	ACAGGGGTTAGGCCTCTTTTTACCACC	0.07141622	0.15616	0.069847	0
tsrna-09360	ACAGGGGTTAGGCCTCTTTTTACCAC	0.14283243	0	0.069847	0
tsrna-09359	ACAGGGGTTAGGCCTCTTTTTACCA	0	0	0	0
tsrna-09357	ACAGGAGATCCTGGGTTCGACTCCCAGCGG	0.71416216	1.249282	0.279387	0.333081
tsrna-09356	ACAGGAGATCCTGGGTTCGAATCCCAGCGGTGCCTCCA	2.85664866	5.934088	0.977855	0.333081
tsrna-09355	ACAGGAGATCCTGGGTTCGAATCCCAGCGGTGCCTCC	0.57132973	2.030083	0.558774	0.111027
tsrna-09354	ACAGGAGATCCTGGGTTCGAATCCCAGCGGTGCCTC	0.49991351	1.639682	0.558774	0.222054
tsrna-09353	ACAGGAGATCCTGGGTTCGAATCCCAGCGGTGCCT	0.71416216	1.795843	0.20954	0.111027
tsrna-09352	ACAGGAGATCCTGGGTTCGAATCCCAGCGGTGCC	0.49991351	2.264323	0.419081	0.333081
tsrna-09351	ACAGGAGATCCTGGGTTCGAATCCCAGCGGTGC	0.49991351	1.561602	0.698468	0
tsrna-09350	ACAGGAGATCCTGGGTTCGAATCCCAGCGGGGCCTCCA	0.49991351	2.420483	0.279387	0.111027
tsrna-09349	ACAGGAGATCCTGGGTTCGAATCCCAGCGGGGCCTCC	0.57132973	2.186243	0.279387	0.666163
tsrna-09348	ACAGGAGATCCTGGGTTCGAATCCCAGCGGGGCCTC	0.49991351	1.873923	0.139694	0.111027

tsrna-09347	ACAGGAGATCCTGGGTTCGAATCCCAGCGGGGCCT	0.35708108	1.483522	0.20954	0.111027
tsrna-09346	ACAGGAGATCCTGGGTTCGAATCCCAGCGGGGCC	0.4284973	1.249282	0.20954	0.333081
tsrna-09345	ACAGGAGATCCTGGGTTCGAATCCCAGCGG	0.57132973	2.030083	0.20954	0.444109
tsrna-09344	ACAGCTATCCATTGGTCTTAGGCCCCA	0	0	0.279387	0
tsrna-09343	ACAGCTATCCATTGGTCTTAGGCCCC	0	0.07808	0	0
tsrna-09340	ACAGCTATCCATTGGTCTTAGGC	0	0	0.069847	0
tsrna-09339	ACAGCTATCCATTGGTCTTAGG	0	0	0	0
tsrna-09332	ACAGAGGCTTACGACCCCTTATTTACCCC	0.07141622	0	0.069847	0
tsrna-09331	ACAGAGGCTTACGACCCCTTATTTACCC	0	0	0.069847	0
tsrna-09323	ACAGAGAATAGTTTAAATTAGAATCTTAGC	6.64170813	0.546561	1.396936	1.221299
tsrna-09322	ACAGACCAAGAGCCTTCAAAGCCC	0.49991351	0.23424	0.069847	0
tsrna-09321	ACAGACCAAGAGCCTTCAAAGCC	0.4284973	0.468481	0.279387	0
tsrna-09320	ACAGACCAAGAGCCTTCAAAGC	0.21424865	0.468481	0.139694	0
tsrna-09319	ACAGACCAAGAGCCTTCAAAG	0.4284973	0.31232	0.069847	0
tsrna-09318	ACAGACCAAGAGCCTTCA	0.07141622	0.07808	0	0
tsrna-09317	ACAGACCAAGAGCCTTC	0	0.07808	0	0
tsrna-09316	ACACTTAGGAGATTTCAACTTAACTTGACCGCTCTGACCA	7.07020543	3.279365	6.984681	1.776434
tsrna-09315	ACACTTAGGAGATTTCAACTTAACTTGACCGCTCTGACC	0.71416216	0.936961	0.488928	0.333081
tsrna-09314	ACACTTAGGAGATTTCAACTTAACTTGACCGCTCTGAC	0.21424865	0.390401	0.279387	0
tsrna-09313	ACACTTAGGAGATTTCAACTTAACTTGACCGCTCTG	0.07141622	0.07808	0.139694	0.111027
tsrna-09312	ACACTTAGGAGATTTCAACTTAACTTGACCGCT	0	0.07808	0.20954	0
tsrna-09311	ACACTTAGGAGATTTCAACTTAACTTGACC	0	0	0.069847	0
tsrna-09310	ACACTTAGGAGATTTCAACTTAACTTGAC	0.07141622	0.07808	0.139694	0
tsrna-09309	ACACTTAGGAGATTTCAACTTAACT	0	0	0	0
tsrna-09308	ACACTTAGGAGATTTCAACTTA	0	0	0	0
tsrna-09307	ACACTTAGGAGATTTCAACTT	0	0	0	0
tsrna-09302	ACACTGAAAATGTTTAGACGGGCTCACATCACCCCATAAACA	13.212	4.372486	6.984681	3.330814
tsrna-09301	ACACTGAAAATGTTTAGACGGGCTCACATCACCCCATAAACA	8.14144867	4.528646	4.330502	0.999244
tsrna-09300	ACACTGAAAATGTTTAGACGGGCTCACATCACC	6.28462705	3.123204	3.352647	0.666163
tsrna-09299	ACACTGAAAATGTTTAGACGGGCTCACAT	3.07089731	2.342403	0.908008	0.333081
tsrna-09298	ACACTGAAAATGTTTAGACGGGCTCACA	2.99948109	2.342403	1.396936	0.333081
tsrna-09297	ACACTGAAAATGTTTAGACGGGCTCAC	1.14265946	1.093122	0.279387	0.333081
tsrna-09296	ACACTGAAAATGTTTAGACGGGCTCA	0.99982703	0.936961	0.20954	0.555136
tsrna-09295	ACACTGAAAATGTTTAGACGGGCTC	0.64274595	0.31232	0	0
tsrna-09294	ACACTGAAAATGTTTAGACGGGCT	0	0.23424	0	0
tsrna-09293	ACACTGAAAATGTTTAGACGGGC	0	0.23424	0	0
tsrna-09292	ACACTGAAAATGTTTAGACGGG	0	0.15616	0	0
tsrna-09291	ACACTGAAAATGTTTAGACGG	0	0	0.139694	0
tsrna-09288	ACACGCGAAAGGTCCCCGGTTT	0	0.31232	0.069847	0
tsrna-09287	ACACGCGAAAGGTCCCCGGTTCGAAACCGGGCGGAAACACC	5.14196758	14.44482	1.955711	0.666163
tsrna-09286	ACACGCGAAAGGTCCCCGGTTCGAAACCGGGCGGAAAC	0	0.15616	0	0
tsrna-09285	ACACGCGAAAGGTCCCCGGTTC	0.07141622	0.23424	0	0
tsrna-09284	ACACGCGAAAGGTCCCCGGTT	0	0.390401	0	0
tsrna-09283	ACACGCGAAAGGTCCCCGGT	0	0.07808	0	0
tsrna-09279	ACACGCAGAAGGTCCTGGGTTCGAGCCCCAGTGGAACCACC	2.49956757	2.732804	3.49234	2.553624
tsrna-09278	ACACGCAGAAGGTCCTGGGTTCGAGCCCCAGTGGAACCACC	1.14265946	1.249282	0.977855	0.222054
tsrna-09277	ACACGCAGAAGGTCCTGGGTTCGAGCCCCAGTGGAACCAC	0.64274595	0.624641	0.977855	0
tsrna-09276	ACACGCAGAAGGTCCTGGGTTCGAGCCCCAGTGGAACCA	0.49991351	0.624641	0.628621	0.111027
tsrna-09275	ACACGCAGAAGGTCCTGGGTTCGAGCCCCAGTGGAACC	0.57132973	0.390401	0.558774	0.222054
tsrna-09274	ACACGCAGAAGGTCCTGGGTTCGAGCCCCAGTGGAAC	0.07141622	0.23424	0	0
tsrna-09273	ACACGCAGAAGGTCCTGGGTTCGAGCCCC	0.07141622	0.07808	0.069847	0
tsrna-09271	ACACCTCTTTACAGTGACCA	0.07141622	0	0	0
tsrna-09270	ACACCTCTTTACAGTGACC	0	0	0	0
tsrna-09267	ACACCAGTCTTGTAAACC	0	0.07808	0	0
tsrna-09266	ACACAAAGCACCCAACTTACACTTAGGAG	0	0	0	0
tsrna-09265	ACACAAAGCACCCAACTTACACTTAG	0	0	0	0
tsrna-09258	ACAAGAACTGCTAACTCATGCCCCCATGTCTAACAACATGGC	0.4284973	0.23424	0.20954	0
tsrna-09257	ACAAGAACTGCTAACTCATGCCCCCATGTCTAACAACATGGC	0.07141622	0.07808	0.419081	0
tsrna-09256	ACAAGAACTGCTAACTCATGCCCCCATGTCTAACAACATGGC	0.35708108	0.07808	0.139694	0
tsrna-09255	ACAAGAACTGCTAACTCATGCCCCCATGTCTAACAACATGGC	0.07141622	0	0.279387	0.111027
tsrna-09254	ACAAGAACTGCTAACTCATGCCCCCATGTCTAACAACATGGC	0	0.15616	0.139694	0.111027
tsrna-09253	ACAAGAACTGCTAACTCATGCCCCCATGTCTAACAACATGG	0.28566487	0	0.20954	0.333081
tsrna-09252	ACAAGAACTGCTAACTCATGCCCCCATGTCTAACAACATG	0.14283243	0.23424	0.419081	0
tsrna-09251	ACAAGAACTGCTAACTCATGCCCCCATGTCTAACAACA	0.21424865	0.07808	0.069847	0.111027

tsrna-09250	ACAAGAACTGCTAACTCATGCCCCCATGTCTAACAA	0.07141622	0.23424	0.139694	0
tsrna-09249	ACAAGAACTGCTAACTCATGCCCCCATGTCTAACA	0.07141622	0	0.069847	0
tsrna-09248	ACAAGAACTGCTAACTCATGCCCCCATGTCT	0	0.15616	0	0
tsrna-09247	ACAAGAACTGCTAACTCATGCCCCCATGTC	0.14283243	0	0.20954	0
tsrna-09246	ACAAGAACTGCTAACTCATGCCCCCATGT	0	0	0.069847	0
tsrna-09245	ACAAGAACTGCTAACTCATGCCCCCATG	0.14283243	0.07808	0	0
tsrna-09244	ACAAGAACTGCTAACTCATGCCCCCA	0	0.07808	0.069847	0
tsrna-09243	ACAAGAACTGCTAACTCATGCCCCC	0.07141622	0	0	0
tsrna-09242	ACAAGAACTGCTAACTCATGCCCC	0	0	0	0
tsrna-09241	ACAAGAACTGCTAACTCATGCCC	0	0.07808	0.069847	0
tsrna-09240	ACAAGAACTGCTAACTCATGCC	0.07141622	0	0	0
tsrna-09239	ACAAGAACTGCTAACTCATGC	0.07141622	0	0	0
tsrna-09237	ACAAGAACTGCTAACTCAT	0.07141622	0	0	0
tsrna-09236	ACAAGAACTGCTAACTCA	0.07141622	0	0	0
tsrna-09235	ACAAGAACTGCTAACTC	0.07141622	0	0	0
tsrna-09233	ACAACGATGGTTTTTCATATCATTGGTCGT	0	0.07808	0.069847	0
tsrna-09232	ACAACGATGGTTTTTCATATC	0	0.07808	0	0
tsrna-09229	ACAACATGGCTTTCTCACCA	0.07141622	0	0	0
tsrna-09224	ACAAAGCACCCAACTTACACTTAGGAGATT	0	0	0	0
tsrna-09223	ACAAAGCACCCAACTTACACTTAGGAG	0	0.07808	0.069847	0
tsrna-09221	ACAAAGCACCCAACTTACAC	0	0	0	0
tsrna-09220	ACAAAACGAATGATTTCGACTCA	0	0.15616	0.20954	0
tsrna-09219	ACAAAACGAATGATTTCGACTC	0	0.07808	0.069847	0
tsrna-09218	AATTTTGGTGCAACTCCAAATAAAAGTACC	0	0	0	0
tsrna-09213	AATTTTGGATTCTCAGGGATGGGTTCGATT	0.07141622	0.07808	0.20954	0
tsrna-09212	AATTTTGGATTCTCAGGGATGGGTTCG	0.07141622	0.07808	0	0.111027
tsrna-09211	AATTTTGGATTCTCAGGGATG	0	0	0	0
tsrna-09209	AATTTGACTAGAGATCAAGA	1.71398919	5.699848	1.047702	0
tsrna-09208	AATTGCAAATTCGAAGAAGCAGCTTCAAACCTGCCGGGGCTT	0.07141622	0.15616	0	0
tsrna-09207	AATTGCAAATTCGAAGAAGCAGCTTCAAACCTGCCGGGGCT	0	0.07808	0	0
tsrna-09206	AATTGCAAATTCGAAGAAGCAGCT	0.07141622	0.07808	0	0
tsrna-09204	AATTCTCGCTGGGGCCTCCA	2.85664866	2.888964	0.838162	1.776434
tsrna-09203	AATTCTCGCCTGCCACGCGGGAGGCCCGG	0.28566487	0.468481	0	0.111027
tsrna-09202	AATTCTCGCCTGCCACGCGGGAGGCCCG	0.21424865	0.624641	0.139694	0.111027
tsrna-09201	AATTCTCGCCTGCCACGCGGGAGGCCC	0.35708108	0.624641	0.069847	0
tsrna-09200	AATTCTCGCCTGCCACGCGGG	0.28566487	0.780801	0	0
tsrna-09199	AATTCTCGCCTGCCACGCGG	0.07141622	0.390401	0	0.222054
tsrna-09198	AATTCTCGCCTGCCACGCG	0	0.780801	0.069847	0.111027
tsrna-09197	AATTCTCGCCTGCCACGC	0.07141622	0.468481	0.139694	0
tsrna-09196	AATTCTCGCCTGCCACG	0.14283243	0.780801	0.069847	0
tsrna-09195	AATTCTCGCCTGCCAC	0	0.07808	0.069847	0
tsrna-09194	AATTCGAAGAAGCAGCTTCAAACCTGCCGGGGCTTCC	0	0.390401	0	0
tsrna-09193	AATTCGAAGAAGCAGCTTCAAACCTGCCGGGGCTT	0	0.15616	0	0
tsrna-09192	AATTCGAAGAAGCAGCTTCAAACCTGCCGGGGCT	0.14283243	0	0.069847	0
tsrna-09191	AATTCGAAGAAGCAGCTTCAAACCTGCCGG	0	0.15616	0	0
tsrna-09190	AATTCCTCTTCTTAACACCA	0	0.31232	0	0.111027
tsrna-09189	AATTCCGGCTCGAAGGACCA	4.92771893	12.41474	2.095404	1.443353
tsrna-09187	AATTCCCGGCCAATGCACCA	0.28566487	0.546561	0	0.111027
tsrna-09186	AATTCCCCGACGGGGAGCCA	0	0	0	0
tsrna-09185	AATTCAAAGGTTCCGGGTTCGAGTCCCGGCGGAGTCGCCA	0	0.390401	0.069847	0
tsrna-09184	AATTCAAAGGTTCCGGGTTC	0	0	0.139694	0
tsrna-09180	AATTATGATAATCATATTTACCAA	0	0	0.069847	0
tsrna-09172	AATTAGAATCTTAGCTTTGGGTGCTAATGGTGGAGTTAAAGA(	0.28566487	0.468481	0.419081	0.222054
tsrna-09171	AATTAGAATCTTAGCTTTGGGTGCTAATGGTGGAGT	0.07141622	0.07808	0	0.111027
tsrna-09170	AATTAGAATCTTAGCTTTGGGTGCTAATGG	0.07141622	0	0	0
tsrna-09169	AATTAGAATCTTAGCTTTGGGTGCT	0.14283243	0.15616	0.069847	0
tsrna-09168	AATTAGAATCTTAGCTTTGGGTGC	0	0.07808	0	0
tsrna-09167	AATTAGAATCTTAGCTTTGGGTG	0.21424865	0.07808	0.20954	0.111027
tsrna-09166	AATTAGAATCTTAGCTTTGGGT	0.07141622	0.07808	0.069847	0
tsrna-09165	AATTAGAATCTTAGCTTTGGG	0	0	0	0
tsrna-09164	AATTAGAATCTTAGCTTTGG	0	0.07808	0	0
tsrna-09163	AATTAGAATCTTAGCTTTG	0	0.15616	0	0.222054
tsrna-09162	AATTAACTAGTTTTGACAACATTCAAAAAA	0	0	0	0
tsrna-09161	AATTAACTAGTTTTGACAACATTCAAAAA	0	0	0	0

tsrna-09158	AATTAACTAGTTTTGACAACATTCA	0.14283243	0	0	0
tsrna-09157	AATTAACTAGTTTTGACAACATTC	0	0	0.069847	0
tsrna-09156	AATTAACTAGTTTTGACAACATT	0	0	0	0
tsrna-09155	AATTAACTAGTTTTGACAACAT	0	0.07808	0	0
tsrna-09151	AATTAACTAGTTTTGACA	0.07141622	0	0	0
tsrna-09148	AATTAAAGTGGCTGATTTGCGTTCA	0.07141622	0	0	0
tsrna-09147	AATTAAAGTGGCTGATTTGCGTTC	0	0	0	0
tsrna-09146	AATTAAAGTGGCTGATTTGCGTT	0	0	0	0
tsrna-09145	AATTAAAGTGGCTGATTTGC	0	0	0.069847	0
tsrna-09144	AATTAAAGTGGCTGATTTG	0	0	0.069847	0
tsrna-09143	AATGTTTAGACGGGCTCACATCACCCCATAAACACCA	7.14162164	0.936961	4.679736	2.220543
tsrna-09142	AATGTTTAGACGGGCTCACATCACCCCATAAACACC	2.71381622	1.483522	2.374791	1.221299
tsrna-09141	AATGTTTAGACGGGCTCACATCACCCCATAAACA	2.64240001	0.858881	2.304945	1.110271
tsrna-09140	AATGTTTAGACGGGCTCACATCACCCCATAAA	2.21390271	1.015041	1.885864	0.77719
tsrna-09139	AATGTTTAGACGGGCTCACATCACCCCATAA	1.71398919	0.546561	1.816017	0.666163
tsrna-09138	AATGTTTAGACGGGCTCACATCACCCCATA	2.14248649	0.858881	1.74617	0.444109
tsrna-09137	AATGTTTAGACGGGCTCACATCACCCCA	2.14248649	1.093122	1.466783	0.888217
tsrna-09136	AATGTTTAGACGGGCTCACATCACCCC	1.35690811	0.390401	1.187396	0.333081
tsrna-09135	AATGTTTAGACGGGCTCACATCACCC	2.28531893	0.390401	1.257243	0.555136
tsrna-09134	AATGTTTAGACGGGCTCACATCACC	1.2854919	0.780801	0.908008	0.333081
tsrna-09133	AATGTTTAGACGGGCTCACATCA	0.92841081	0.624641	0.698468	0.555136
tsrna-09132	AATGTTTAGACGGGCTCACATC	1.07124325	0.780801	0.838162	0.222054
tsrna-09131	AATGTTTAGACGGGCTCACAT	1.14265946	0.702721	1.396936	0.222054
tsrna-09130	AATGTTTAGACGGGCTCACA	0.8569946	0.858881	0.768315	0.222054
tsrna-09129	AATGTTTAGACGGGCTCAC	0.57132973	0.31232	0.558774	0
tsrna-09128	AATGTTTAGACGGGCTCA	0.49991351	0.15616	0	0.111027
tsrna-09127	AATGTTTAGACGGGCTC	0.14283243	0.15616	0.069847	0
tsrna-09126	AATGTTTAGACGGGCT	0	0	0.069847	0
tsrna-09125	AATGTTGGTTATACCCTTCCCGTACTACCA	0.49991351	1.015041	0	0.555136
tsrna-09124	AATGTTGGTTATACCCTTCCCGTACTACC	0	0.07808	0.069847	0
tsrna-09119	AATGTTGGTTATACCC	0	0.07808	0	0
tsrna-09118	AATGGTTAGCACTCTGGGCT	0.07141622	0	0	0
tsrna-09116	AATGGTTAGCACTCTGGACTTTGAATCCAG	0.78557838	0.23424	0.279387	0.444109
tsrna-09115	AATGGTTAGCACTCTGGACTTTGAATCCA	0.28566487	0.858881	0.279387	0.555136
tsrna-09114	AATGGTTAGCACTCTGGACTTTGAATCC	0.49991351	0.468481	0.20954	0.333081
tsrna-09113	AATGGTTAGCACTCTGGACTTTGAATC	0.14283243	0.468481	0.069847	0.111027
tsrna-09112	AATGGTTAGCACTCTGGACTTTGAAT	0.07141622	0	0	0
tsrna-09111	AATGGTTAGCACTCTGGACTTTGAA	0.07141622	0	0	0.111027
tsrna-09110	AATGGTTAGCACTCTGGACTTTGA	0	0.07808	0.139694	0
tsrna-09106	AATGGTTAGCACTCTGGACTCTGAATCCAGCGATCCGAGTTC	0.28566487	0.390401	0.349234	0.222054
tsrna-09105	AATGGTTAGCACTCTGGACTCTGAATCCAGCGATCCGAGT	0.21424865	0.546561	0.20954	0.222054
tsrna-09104	AATGGTTAGCACTCTGGACTCTGAATCCAGCGATCCGAG	0.21424865	0.390401	0	0.222054
tsrna-09103	AATGGTTAGCACTCTGGACTCTGAATCCAGCGATCC	0.14283243	0.390401	0.20954	0.222054
tsrna-09102	AATGGTTAGCACTCTGGACTCTGAATCCAGCG	0.35708108	0.31232	0.139694	0.111027
tsrna-09101	AATGGTTAGCACTCTGGACTCTGAATCCAGC	0.21424865	0.07808	0.279387	0
tsrna-09100	AATGGTTAGCACTCTGGACTCTGAATCCAG	0.28566487	0.624641	0.349234	0.111027
tsrna-09099	AATGGTTAGCACTCTGGACTCTGAATCCA	0.21424865	0.31232	0.069847	0.111027
tsrna-09098	AATGGTTAGCACTCTGGACTCTGAATCC	0.21424865	0.390401	0.279387	0
tsrna-09097	AATGGTTAGCACTCTGGACTCTGAATC	0	0.15616	0.139694	0
tsrna-09096	AATGGTTAGCACTCTGGACTCTGAAT	0	0	0	0.111027
tsrna-09095	AATGGTTAGCACTCTGGACTCTGAA	0.14283243	0	0	0
tsrna-09094	AATGGTTAGCACTCTGGACTCTGA	0.07141622	0	0.139694	0
tsrna-09093	AATGGTTAGCACTCTGGACTCTG	0.07141622	0.07808	0	0.222054
tsrna-09091	AATGGTTAGCACTCTGGACTC	0.07141622	0	0	0
tsrna-09090	AATGGTTAGCACTCTGGACT	0	0.07808	0	0
tsrna-09088	AATGGTTAGCACTCTGGA	0	0	0.069847	0
tsrna-09085	AATGGTGGTTCAGTGGTAGAATTCTCGCCT	1.99965406	1.639682	1.187396	0.333081
tsrna-09084	AATGGTGGTTCAGTGGTAGAATTCTCGC	0.21424865	0.624641	0	0.222054
tsrna-09083	AATGGTGGTTCAGTGGTAGAATTCTCG	0.21424865	0.31232	0.20954	0.222054
tsrna-09082	AATGGTGGTTCAGTGGTAGAATTCTC	0.07141622	0.31232	0.069847	0
tsrna-09081	AATGGTGGTTCAGTGGTAGAATTCT	0.21424865	0	0.139694	0.111027
tsrna-09080	AATGGTGGAGTTAAAGACTTTTTCTCTGACCA	1.92823784	4.060166	1.257243	1.443353
tsrna-09079	AATGGTGGAGTTAAAGACTTTTTCTCTGACC	0.57132973	1.873923	0.419081	0.222054
tsrna-09078	AATGGTGGAGTTAAAGACTTTTTCTCTGAC	0.14283243	0.780801	0.20954	0

tsrna-09077	AATGGTGGAGTTAAAGACTTTTTCTCTGA	0.28566487	0.702721	0.069847	0.111027
tsrna-09076	AATGGTGGAGTTAAAGACTTTTTCTCTG	0.35708108	0.780801	0.139694	0.111027
tsrna-09075	AATGGTGGAGTTAAAGACTTTTTCTCT	0.14283243	1.093122	0.349234	0
tsrna-09074	AATGGTGGAGTTAAAGACTTTTTCTC	0.21424865	0.780801	0	0
tsrna-09073	AATGGTGGAGTTAAAGACTTTTTCT	0.4284973	1.093122	0.20954	0
tsrna-09072	AATGGTGGAGTTAAAGACTTTTTC	0.64274595	0.546561	0.20954	0
tsrna-09071	AATGGTGGAGTTAAAGACTTTTT	0.14283243	0.624641	0.20954	0
tsrna-09070	AATGGTGGAGTTAAAGACTTTT	0.14283243	0.23424	0.139694	0
tsrna-09069	AATGGTGGAGTTAAAGACTTT	0	0.15616	0	0
tsrna-09068	AATGGTGGAGTTAAAGACTT	0.21424865	0.23424	0.069847	0
tsrna-09067	AATGGTGGAGTTAAAGACT	0.14283243	0.15616	0.139694	0.111027
tsrna-09066	AATGGTGGAGTTAAAGAC	0.07141622	0.07808	0.069847	0
tsrna-09065	AATGGTGGAGTTAAAGA	0.07141622	0.15616	0	0
tsrna-09064	AATGGTGGAGTTAAAG	0	0.15616	0	0
tsrna-09063	AATGGTGAGCACTTTGGACTCTGA	0	0	0	0
tsrna-09062	AATGGTGAGCACTTTGGACTCTG	0	0	0	0
tsrna-09061	AATGGTGAGCACTTTGGACTCT	0	0.07808	0	0
tsrna-09058	AATGGTGAGCACTCTGGACTCTGAATCCAG	0.28566487	0.468481	0	0
tsrna-09057	AATGGTGAGCACTCTGGACTCTGAATCCA	0	0.31232	0	0
tsrna-09056	AATGGTGAGCACTCTGGACTCTGAATCC	0.07141622	0.31232	0	0.333081
tsrna-09055	AATGGTGAGCACTCTGGACTCTGAATC	0	0.07808	0	0
tsrna-09054	AATGGTGAGCACTCTGGACTCTGA	0	0.15616	0	0
tsrna-09048	AATGGTGAGCACTCTGGA	0	0	0	0
tsrna-09038	AATGGTAGAATTCTCG	0	0	0.069847	0
tsrna-09037	AATGGTAAGCACTCTGGACTCTGAATCC	0.14283243	0.31232	0	0
tsrna-09034	AATGGTAAGCACTCTGGACTCTG	0	0	0	0
tsrna-09026	AATGGCTGAGTGAAGCATTGGACTGTAAA	0.28566487	0.15616	0	0
tsrna-09025	AATGGCTGAGTGAAGCATTGGACTGTAA	0	0.15616	0.069847	0
tsrna-09024	AATGGCTGAGTGAAGCATTGGACTGTA	0.07141622	0.07808	0.069847	0
tsrna-09023	AATGGCTGAGTGAAGCATTGGACTGT	0.07141622	0.07808	0	0
tsrna-09022	AATGGCTGAGTGAAGCATTGGACTG	0	0	0	0
tsrna-09021	AATGGCTGAGTGAAGCATTGGACT	0.07141622	0.07808	0	0
tsrna-09020	AATGGCTGAGTGAAGCATTGGAC	0	0.23424	0.069847	0
tsrna-09019	AATGGCTGAGTGAAGCATTGGA	0	0.07808	0.069847	0.111027
tsrna-09018	AATGGCTGAGTGAAGCATTGG	0.14283243	0	0.139694	0
tsrna-09017	AATGGCTGAGTGAAGCATTG	0	0	0.069847	0
tsrna-09016	AATGGCTGAGTGAAGCATT	0	0.07808	0	0
tsrna-09015	AATGGCTGAGTGAAGCA	0	0	0	0
tsrna-09014	AATGGCTGAGTGAAGC	0	0	0	0
tsrna-09012	AATGGATAGCGCATTGGACTTC	0.49991351	1.561602	0.069847	0
tsrna-09011	AATGGATAGCGCATTGGACT	0.35708108	1.093122	0.069847	0.333081
tsrna-09010	AATGGATAGCGCATTGGAC	0.35708108	1.249282	0.069847	0.111027
tsrna-09009	AATGGATAGCGCATTGGA	0.35708108	0.468481	0	0.333081
tsrna-09008	AATGGATAGCGCATTGG	0.35708108	0.31232	0.069847	0.111027
tsrna-09007	AATGGATAAGGCGTCTGATTCC	0.14283243	0.468481	0.069847	0
tsrna-09006	AATGGATAAGGCGTCTGACTTCGGATC	0.07141622	0.07808	0.069847	0
tsrna-09005	AATGGATAAGGCGTCTGACTTCGGAT	0	0.07808	0	0
tsrna-09004	AATGGATAAGGCGTCTGACTTCG	0.07141622	0	0.069847	0.111027
tsrna-09003	AATGGATAAGGCGTCTGACTTC	0.07141622	0.07808	0	0
tsrna-09002	AATGGATAAGGCGTCTGACTT	0	0.07808	0	0.111027
tsrna-09001	AATGGATAAGGCGTCTGACT	0	0.15616	0	0
tsrna-09000	AATGGATAAGGCGTCTGA	0	0.07808	0	0
tsrna-08999	AATGGATAAGGCATTGGC	0.07141622	0.07808	0.069847	0
tsrna-08998	AATGGATAAGGCATTGG	0.07141622	0.07808	0.069847	0
tsrna-08997	AATGGATAAGGCATTG	0.07141622	0.15616	0	0
tsrna-08996	AATGGATAAGGCATCAGCCTCC	0.07141622	0	0.069847	0.111027
tsrna-08995	AATGGATAAGGCATCAGCCTC	0.14283243	0.15616	0.139694	0.111027
tsrna-08994	AATGGATAAGGCATCAGCCT	0.4284973	0.07808	0.349234	0.111027
tsrna-08993	AATGGATAAGGCACTGGC	1.35690811	1.639682	0.698468	0.444109
tsrna-08992	AATGGATAAGGCACTGG	0.78557838	1.405442	0.349234	0.444109
tsrna-08991	AATGGATAAGGCACTG	0.49991351	0.936961	0.698468	0.333081
tsrna-08990	AATGGATAACGCGTCTGACTACGGA	0.14283243	0	0.20954	0.111027
tsrna-08989	AATGGATAACGCGTCTGACTACGG	0.07141622	0	0.069847	0
tsrna-08988	AATGGATAACGCGTCTGACTACG	0.07141622	0.15616	0.069847	0.333081

tsrna-08986	AATGGAGGCGTGGGTTCGAATCCCACTTCTGACACCA	1.71398919	4.372486	0.628621	1.332326
tsrna-08985	AATGGAGGCGTGGGTTCGAATCCCACTTCTGACACC	0.14283243	0.624641	0.419081	0
tsrna-08984	AATGGAGGCGTGGGTTCGAATCCCACTTCTGACA	0	0.23424	0	0
tsrna-08983	AATGGAGGCGTGGGTT	0.07141622	0.31232	0	0
tsrna-08982	AATGCCGAGGTTGTGAGTTCGATC	0.07141622	0.780801	0	0
tsrna-08981	AATGCCGAGGTTGTGAGTTCGAT	0	0.390401	0	0
tsrna-08980	AATGCCGAGGTTGTGAGTTCGAGC	0.49991351	5.777928	0.20954	0
tsrna-08979	AATGCCGAGGTTGTGAGTTCGAG	0.07141622	1.015041	0.069847	0
tsrna-08978	AATGCCGAGGTTGTGAGTTCGA	0.14283243	0.468481	0	0
tsrna-08977	AATGCCGAGGTTGTGAGTTCG	0.14283243	0.15616	0.069847	0
tsrna-08976	AATGCCGAGGTTGTGAGTTCAAGC	0	0.23424	0	0
tsrna-08975	AATGCCGAGGTTGTGAGTTC	0	0.23424	0	0
tsrna-08974	AATGCCGAGGTTGTGAGTT	0.14283243	0.15616	0	0
tsrna-08970	AATGATTTCGACTCATTA	0	0.07808	0	0
tsrna-08969	AATGATTTCGACTCATT	0	0.07808	0	0
tsrna-08967	AATCTTAGCTTTGGGTGCTAATGGTGGAGTTAAAGA	0.21424865	0.07808	0.069847	0
tsrna-08966	AATCTTAGCTTTGGGTGCTAATGGTGGAGT	0.07141622	0	0	0
tsrna-08962	AATCTGAGGGTCCAGGGTTCAAGT	0.14283243	0.15616	0	0
tsrna-08961	AATCTGAGGGTCCAGGGTTCAAG	0.07141622	0.31232	0	0
tsrna-08960	AATCTGACAACAGAGGCTTACGACCCCTTATTTACCCCA	0.28566487	0.23424	0.279387	0.111027
tsrna-08959	AATCTGACAACAGAGGCTTACGACCCCTTATTTACCCC	0.14283243	0	0.20954	0
tsrna-08958	AATCTGACAACAGAGGCTTACGACCCCTTATTTACCC	0.14283243	0.07808	0.069847	0
tsrna-08957	AATCTGACAACAGAGGCTTACGACCCCTTATTTACC	0.21424865	0	0	0
tsrna-08956	AATCTGACAACAGAGGCTTACGACCCCTT	0	0	0	0
tsrna-08955	AATCTGACAACAGAGGCTTACGACCCCT	0	0	0	0
tsrna-08949	AATCTGAAGGTCGTGAGTTCGATCCTCACACGGGGCACCA	0.49991351	3.045124	0.139694	0
tsrna-08948	AATCTGAAGGTCGTGAGTTCGATCCTCACACGGGGCACC	0.28566487	0.936961	0.488928	0
tsrna-08947	AATCTGAAGGTCGTGAGTTCGATCCTCACACGGGGCAC	0.28566487	0.624641	0.20954	0
tsrna-08946	AATCTGAAGGTCGTGAGTTCGAGCCTCACACGGGGCACCA	0.78557838	1.405442	0.069847	0.111027
tsrna-08945	AATCTGAAGGTCGTGAGTTCGAG	0.21424865	0.624641	0.139694	0.111027
tsrna-08944	AATCTGAAGGTCGTGAGT	0	0.468481	0.139694	0
tsrna-08943	AATCTGAAGGTCCTGAGTTCGAACCTCAGAGGGGGCACCA	1.2854919	3.279365	0.838162	0.555136
tsrna-08942	AATCTGAAGGTCCTGAGTTCGA	0.8569946	3.279365	0.768315	0.222054
tsrna-08941	AATCTGAAGGTCCTGAGT	0.64274595	2.108163	1.187396	0.333081
tsrna-08940	AATCTGAAGGTCCTGA	0	0.23424	0	0
tsrna-08939	AATCTCGGTGGGACCTCCA	3.71364325	7.26145	1.74617	0.999244
tsrna-08938	AATCTCGGTGGGACCTCC	0.07141622	0.390401	0.069847	0
tsrna-08937	AATCTCGGTGGAACCTCCA	7.21303786	14.21058	3.422493	1.998488
tsrna-08936	AATCTCGGTGGAACCTCC	0.21424865	1.015041	0.139694	0.111027
tsrna-08935	AATCTCGGTGGAACCTC	0	0.15616	0	0
tsrna-08934	AATCTCGGTGGAACCT	0.07141622	0.15616	0.069847	0
tsrna-08933	AATCTCGCTGGGGCCTCCA	3.78505947	4.606726	1.53663	1.887461
tsrna-08932	AATCTCAGGGTCGTGGGTTCGAG	0	0.15616	0	0
tsrna-08931	AATCTCAGGGTCGTGGGTTCG	0	0	0	0
tsrna-08929	AATCTAAAGACAGGGGTTAGGCCTCTTTTTACCACC	2.42815136	1.561602	1.117549	0.111027
tsrna-08928	AATCTAAAGACAGGGGTTAGGCCTCTTTTTACC	1.71398919	1.483522	0.768315	0
tsrna-08927	AATCTAAAGACAGGGGTTAGGCCTCTTTTT	0.71416216	1.561602	0.628621	0.111027
tsrna-08926	AATCTAAAGACAGGGGTTAGGCCTCT	0.35708108	0.15616	0	0
tsrna-08925	AATCTAAAGACAGGGGTTAGG	0	0	0	0
tsrna-08924	AATCTAAAGACAGGGGTTAG	0	0.07808	0	0
tsrna-08923	AATCGGTTAGCGCGTTCGGCT	0	0.07808	0	0
tsrna-08922	AATCCTGTTCGTGACGCCA	0.14283243	0.546561	0.069847	0.111027
tsrna-08921	AATCCTGTTCGTGACGCC	0.07141622	0	0	0
tsrna-08919	AATCCTGTTCGTGACG	0	0	0	0
tsrna-08918	AATCCTGCTCACAGCGCCA	0.28566487	1.015041	0.139694	0.222054
tsrna-08917	AATCCTGCTCACAGCGCC	0.07141622	0.07808	0.139694	0
tsrna-08916	AATCCTGCCGACTACGCCA	6.78454056	19.36387	2.514485	1.665407
tsrna-08915	AATCCTGCCGACTACGCC	0.35708108	0.858881	0.20954	0.222054
tsrna-08914	AATCCTGCCGACTACGC	0	0.15616	0	0
tsrna-08910	AATCCGGGTGCCCCCTCCA	9.9982703	15.85026	3.771728	2.997733
tsrna-08909	AATCCGGGTGCCCCCTCC	0.21424865	0.390401	0.069847	0
tsrna-08908	AATCCGGGTGCCCCCTC	0	0.07808	0	0
tsrna-08907	AATCCGGCTCGGAGGACCA	0.07141622	0.31232	0	0
tsrna-08904	AATCCGGCTCGAAGGACCA	1.14265946	1.795843	0.838162	0.222054

tsrna-08903	AATCCGGCTCGAAGGACC	0.07141622	0.15616	0	0
tsrna-08898	AATCCCGGGTTTCGGCACCA	3.64222704	5.543688	1.187396	1.55438
tsrna-08896	AATCCCGGACGAGCCCCCA	8.78419462	12.25858	2.654179	3.663896
tsrna-08895	AATCCCGGACGAGCCCCC	0.35708108	0.780801	0.069847	0.333081
tsrna-08894	AATCCCGGACGAGCCCC	0.07141622	0.07808	0	0.111027
tsrna-08893	AATCCCGGACGAGCCC	0	0	0	0
tsrna-08892	AATCCCCGGCATCTCCACCA	8.07003246	9.681934	4.120962	1.998488
tsrna-08891	AATCCCCGGCATCTCCACC	0.4284973	0.390401	0.069847	0.222054
tsrna-08888	AATCCCCGGCACCTCCACCA	11.9979244	19.44195	6.006825	2.775678
tsrna-08887	AATCCCCGGCACCTCCACC	0.49991351	1.327362	0.279387	0
tsrna-08884	AATCCCCAGCACCTCCACCA	0	0	0	0
tsrna-08882	AATCCCATCCTCGTCGCCA	0.07141622	0.468481	0.069847	0
tsrna-08878	AATCCCAGTAGAGCCTCCA	0.14283243	0.31232	0.139694	0.666163
tsrna-08876	AATCCCAGCGGTGCCTCCA	0	0	0	0
tsrna-08873	AATCCCAGCGGGGCCTCCA	0	0.07808	0	0
tsrna-08872	AATCCCACTTCTGACACCA	0.21424865	0.468481	0.139694	0.444109
tsrna-08871	AATCCCACTTCTGACACC	0	0.07808	0	0
tsrna-08868	AATCCCACTCCTGACACCA	5.35621623	4.528646	2.724025	2.109516
tsrna-08867	AATCCCACTCCTGACACC	0.14283243	0.23424	0.069847	0
tsrna-08866	AATCCCACTCCTGACAC	0	0	0.069847	0
tsrna-08864	AATCCCACCTTCGTCGCCA	0	0.468481	0.139694	0.222054
tsrna-08861	AATCCCACCGCTGCCACCA	0	0.07808	0	0
tsrna-08859	AATCCCACCGCTGCCAC	0	0	0	0
tsrna-08858	AATCCCACCCTCGTCGCCA	0.07141622	1.327362	0.069847	0.222054
tsrna-08857	AATCCCACCCTCGTCGCC	0	0.07808	0	0.111027
tsrna-08854	AATCCCACCAGAGTCGCCA	0.14283243	0.468481	0.069847	0
tsrna-08853	AATCCCACCAGAGTCGCC	0	0.07808	0	0
tsrna-08849	AATCCATTGGGGTTTCCCCGCGCAGGTTCGAATCCTGCCGAC	0.14283243	0.390401	0	0
tsrna-08848	AATCCATTGGGGTTTCCCCGCGCAGGTTCGAATCCTGCCGAC	0.07141622	0.31232	0	0
tsrna-08847	AATCCATTGGGGTTTCCCCGCGCAGGTTCGAATCCTGCCGA	0.21424865	0.390401	0	0
tsrna-08842	AATCCAGGTGCCCCCTCCA	0.14283243	0.07808	0	0.111027
tsrna-08840	AATCCAGCGATCCGAGTTCGAG	0	0.07808	0	0
tsrna-08837	AATCCAGCGATCCGAGTTCAAGTC	0	0.07808	0	0
tsrna-08836	AATCCAGCGATCCGAGTTCAAGT	0	0	0.069847	0
tsrna-08835	AATCCAGCGATCCGAGTTCAAG	0	0.07808	0	0
tsrna-08834	AATCCAGCGATCCGAGTTCAAATCTCGGTGGAACCTC	0	0	0.069847	0
tsrna-08832	AATCCAGCGATCCGAGTTCAAATCT	0	0	0.069847	0
tsrna-08822	AATCCAGCAATCCGAGTTCGAAT	0	0.23424	0	0
tsrna-08821	AATCCAGCAATCCGAGTTCGAA	0	0.15616	0	0
tsrna-08820	AATCCAGCAATCCGAGTTCGA	0	0.15616	0	0
tsrna-08819	AATCCAGCAATCCGAGTTCG	0.07141622	0.15616	0	0
tsrna-08818	AATCATATTTACCAACCA	0	0	0.069847	0
tsrna-08815	AATCACGTCGGGGTCACCA	0.28566487	0.624641	0.139694	0.222054
tsrna-08814	AATCACGTCGGGGTCACC	0	0	0.069847	0
tsrna-08810	AATATAGTTTAACCAAAACATCAGATTGTG	0	0	0	0
tsrna-08809	AATAGTACCGTTAACTTCCAATTAACTAGTTTTGAC	0.4284973	0.23424	0.279387	0
tsrna-08808	AATAGTACCGTTAACTTCCAATTAACTAGT	0.21424865	0.15616	0.20954	0
tsrna-08807	AATAGTACCGTTAACTTCCAATTA	0.07141622	0.15616	0.419081	0
tsrna-08805	AATAGTACCGTTAACTTCC	0	0.07808	0	0
tsrna-08803	AATAGTACCGTTAACTT	0	0	0	0
tsrna-08801	AATAGGAGCTTAAACCCCCTTATTTCTACC	0.07141622	0.468481	0.069847	0
tsrna-08800	AATAGGAGCTTAAACCCCC	0	0	0.069847	0
tsrna-08798	AATAGGAGCTTAAACCC	0	0	0	0
tsrna-08792	AATACTTAATTTCTGCCA	0.14283243	0	0	0
tsrna-08790	AATACAGACCAAGAGCCTTCA	0	0	0	0
tsrna-08789	AATACAGACCAAGAGCCTTC	0	0	0	0
tsrna-08788	AATACAGACCAAGAGCC	0	0	0	0
tsrna-08786	AATACACTGAAAATGTTTAGACGGGCTCACATCACCCCATAA/	9.1412757	4.060166	5.517898	1.776434
tsrna-08785	AATACACTGAAAATGTTTAGACGGGCTCAC	1.71398919	1.561602	0.977855	0.111027
tsrna-08784	AATACACTGAAAATGTTTAGACGGGCTC	0.4284973	1.015041	0.279387	0
tsrna-08783	AATACACCAGTCTTGTAAACC	0.64274595	0.23424	0.279387	0.111027
tsrna-08782	AATACAACGATGGTTTTTCATATCATTGGTCGTGGTTGTAGTC	0.35708108	0.31232	0.558774	0.111027
tsrna-08781	AATACAACGATGGTTTTTCATATCATTGGTCGTGGTTGTA	0.14283243	0.15616	0.139694	0
tsrna-08780	AATACAACGATGGTTTTTCATATCATTGGT	0	0	0	0

tsrna-08778	AATACAACGATGGTTTTTC	0.07141622	0	0	0
tsrna-08777	AATAATAGGAGCTTAAACCCCCTTATTTCT	0.14283243	0.07808	0	0
tsrna-08776	AATAATAGGAGCTTAAACCCCCTT	0.07141622	0	0	0
tsrna-08773	AATAATAGGAGCTTAAACCCC	0	0	0	0
tsrna-08772	AATAATAGGAGCTTAAACCC	0	0	0	0.111027
tsrna-08771	AATAATAGGAGCTTAAACC	0	0	0	0
tsrna-08770	AATAAGCTATCGGGCCCATACCCCGAAA	0	0.07808	0.069847	0
tsrna-08769	AATAAGCTATCGGGCCCATACC	0	0.07808	0.069847	0.111027
tsrna-08768	AATAAGCTATCGGGCCCATA	0	0	0	0
tsrna-08767	AATAAGCTATCGGGCCCAT	0	0	0	0
tsrna-08766	AATAAGCTATCGGGCCCA	0	0.15616	0	0
tsrna-08765	AATAAGCTATCGGGCCC	0	0	0	0
tsrna-08763	AATAACGCCAAGGTCGCGGGTTCGA	0.64274595	0.702721	0.20954	0
tsrna-08762	AATAACGCCAAGGTCGCGGGTT	0.21424865	0.468481	0	0
tsrna-08761	AATAACGCCAAGGTCGCGGGT	0.28566487	0.468481	0	0
tsrna-08760	AATAACGCCAAGGTCGCGG	0	0.07808	0	0
tsrna-08759	AAGTTGCAATACTTAATTTCTGCCA	0.14283243	0	0	0.111027
tsrna-08758	AAGTTGCAATACTTAATTTCTGCC	0	0	0	0
tsrna-08757	AAGTTGCAATACTTAATTTCTGC	0.07141622	0	0	0
tsrna-08756	AAGTTCTGGTCTCCGGATGGAGGCGTGGGT	0.35708108	2.888964	0.349234	0.111027
tsrna-08754	AAGTTCTGGTCTCCGGATGGAGG	0	0.07808	0	0
tsrna-08753	AAGTTCTGGTCTCCGGATGGAG	0	0	0	0.111027
tsrna-08745	AAGTTAAAGATTAAGAGAACCAACACCTCTTTACAGTGACCA	0.57132973	0.31232	0.628621	0.111027
tsrna-08744	AAGTTAAAGATTAAGAGAACCAACACCTCTTTACAGTGACC	0.35708108	0.07808	0.419081	0.111027
tsrna-08743	AAGTTAAAGATTAAGAGAACCAACACCTCTTTACAGTGAC	0.21424865	0.07808	0.558774	0.333081
tsrna-08742	AAGTTAAAGATTAAGAGAACCAACACC	0	0	0	0
tsrna-08741	AAGTTAAAGATTAAGAGAACC	0	0	0	0
tsrna-08739	AAGTGTTTGTGGGTTTAAGTCCCATTGGTCTAGCCA	0.14283243	0.23424	0.069847	0
tsrna-08738	AAGTGTTTGTGGGTTTAAGTCCCATTGGTCTAGCC	0.07141622	0	0	0
tsrna-08737	AAGTGTTTGTGGGTTTAAGTCCCATTGGTCTAGC	0	0	0.139694	0
tsrna-08736	AAGTGTTTGTGGGTTTAAGTCCCATTGGTCTA	0	0.15616	0	0
tsrna-08735	AAGTGTTTGTGGGTTTAAGTCCCATTGGTC	0	0.07808	0	0
tsrna-08734	AAGTGTTTGTGGGTTTAAGTCCCATTG	0	0	0	0
tsrna-08733	AAGTGTTTGTGGGTTTAAGTCCCATT	0	0.23424	0	0
tsrna-08732	AAGTGTTTGTGGGTTTAAGTCCCA	0	0	0	0
tsrna-08731	AAGTGTTTGTGGGTTTAAGTCCC	0	0	0.069847	0
tsrna-08730	AAGTGTTTGTGGGTTTAAGTCC	0	0.07808	0	0
tsrna-08729	AAGTGTTTGTGGGTTTAAGTC	0	0	0	0
tsrna-08724	AAGTGTTTGTGGGTTT	0	0	0.069847	0
tsrna-08721	AAGTGGCTGATTTGCGTTCAGTTGATGC	0	0.07808	0	0
tsrna-08720	AAGTGGCTGATTTGCGTTCAGTTGATG	0	0.15616	0.139694	0
tsrna-08719	AAGTGGCTGATTTGCGTTCAGT	0	0.07808	0	0
tsrna-08718	AAGTGGCTGATTTGCGTTCAG	0	0	0	0
tsrna-08717	AAGTGGCTGATTTGCGTTCA	0	0	0	0
tsrna-08715	AAGTGGCTGATTTGCGTT	0	0	0	0
tsrna-08712	AAGTCTCGGTGGAACCTCCA	3.57081082	8.354572	1.047702	1.221299
tsrna-08711	AAGTCCCTGTTCGGGCGCCA	0.4284973	0.702721	0.20954	0
tsrna-08709	AAGTCCCTGTTCAGGCGCCA	0	0	0	0
tsrna-08706	AAGTCATGGAGGCCATGGGGTTGG	0	0.23424	0.069847	0
tsrna-08705	AAGTCATGGAGGCCATGGGGTTG	0	0.23424	0	0
tsrna-08704	AAGTCATGGAGGCCATGGGGTT	0	0.07808	0	0
tsrna-08703	AAGTCATGGAGGCCATGGGGT	0	0.07808	0	0
tsrna-08702	AAGTCATGGAGGCCATGGGG	0	0	0.069847	0
tsrna-08698	AAGTCACGTCGGGGTCACCA	0.14283243	0.23424	0.20954	0
tsrna-08697	AAGTCACGTCGGGGTCACC	0	0	0	0.111027
tsrna-08696	AAGTCACGTCGGGGTCAC	0	0	0	0
tsrna-08695	AAGTCACGTCGGGGTCA	0	0.07808	0	0
tsrna-08694	AAGTCACGTCGGGGTC	0	0.07808	0	0
tsrna-08693	AAGGTTGTGGGTTCGAGTCCCACC	0	0	0.069847	0
tsrna-08692	AAGGTTGGTGGTTCGAGCCCACCCAGGGAC	0.07141622	0.07808	0	0
tsrna-08688	AAGGTTGCGTGTTCAAATCACGTCGGGGTC	0	0.15616	0	0.111027
tsrna-08687	AAGGTTGCGTGTTCAAATCA	0	0.07808	0	0
tsrna-08684	AAGGTTCCGGGTTCGAGTCCCGGCG	0	0	0	0
tsrna-08681	AAGGTTCCGGGTTCGAGTCCCG	0	0	0.069847	0

tsrna-08676	AAGGTCGTGAGTTCGAGCCTCACACGGGGC	0	0	0	0
tsrna-08673	AAGGTCGCGGGTTCGATCCCCGTACGGGCC	0.07141622	0	0.069847	0
tsrna-08671	AAGGTCGCGGGTTCGATCCCCG	0	0.15616	0	0
tsrna-08670	AAGGTCGCGGGTTCGATCCCC	0	0.07808	0.069847	0
tsrna-08668	AAGGTCGCGGGTTCGATCC	0	0.07808	0	0
tsrna-08667	AAGGTCGCGGGTTCGATC	0.07141622	0	0.069847	0
tsrna-08666	AAGGTCGCGGGTTCGA	0	0	0	0
tsrna-08665	AAGGTCCTGGGTTCGAGCCCCAGTGGAACCACCA	3.35656217	2.030083	1.257243	1.443353
tsrna-08664	AAGGTCCTGGGTTCGAGCCCCAGTGGAACC	0.07141622	0.07808	0.069847	0
tsrna-08663	AAGGTCCTGGGTTCGAGCCCCAGTGGAAC	0	0.07808	0	0
tsrna-08661	AAGGTCCTGGGTTCGAGCCCCAGTGGA	0.07141622	0	0	0
tsrna-08658	AAGGTCCTGGGTTCGAGCCCCAGT	0	0	0	0
tsrna-08657	AAGGTCCTGGGTTCGAGCCCCAG	0	0	0	0
tsrna-08656	AAGGTCCTGGGTTCGAGCCCCA	0	0.15616	0	0
tsrna-08654	AAGGTCCTGGGTTCGAGCCC	0.07141622	0	0.069847	0
tsrna-08653	AAGGTCCTGGGTTCGAGCC	0.07141622	0	0	0
tsrna-08652	AAGGTCCTGGGTTCGAGC	0.07141622	0	0	0
tsrna-08651	AAGGTCCTGGGTTCGAG	0.07141622	0.07808	0	0
tsrna-08650	AAGGTCCTGAGTTCGAACCTC	0	0	0	0.111027
tsrna-08647	AAGGTCCTGAGTTCGAA	0.07141622	0	0	0
tsrna-08646	AAGGTCCCTGGTTCGATCCCGGGTTTCGGC	0.28566487	0.702721	0.279387	0.111027
tsrna-08645	AAGGTCCCTGGTTCGATCCCGGGTTTCGG	0.4284973	0.858881	0.279387	0.111027
tsrna-08644	AAGGTCCCTGGTTCGATCCCGGG	0.35708108	0.624641	0.349234	0.333081
tsrna-08643	AAGGTCCCTGGTTCGATCCCGG	0.35708108	0.624641	0.20954	0.111027
tsrna-08642	AAGGTCCCTGGTTCGATCCC	0.4284973	1.093122	0.069847	0.111027
tsrna-08641	AAGGTCCCTGGTTCGAT	0.28566487	0.936961	0.069847	0.222054
tsrna-08638	AAGGTCCCCGGTTCGAAACC	0.07141622	0.15616	0	0
tsrna-08637	AAGGTCCCCGGTTCGAAAC	0.14283243	0	0	0
tsrna-08634	AAGGTCAGCTAAATAAGCTATCGGGCCCA	0	0.07808	0.069847	0
tsrna-08633	AAGGTCAGCTAAATAAGCTATCGGGCCC	0	0	0	0
tsrna-08626	AAGGTCAGCTAAATAAGC	0	0	0.069847	0
tsrna-08623	AAGGTATTAGAAAAACCATTTCATAACTTTGTCAAAGTTA	1.07124325	1.171202	1.257243	0.111027
tsrna-08622	AAGGTATTAGAAAAACCATTTCATAACTTTGTCAAA	1.21407568	0.780801	0.628621	0.222054
tsrna-08621	AAGGTATTAGAAAAACCATTTCATAACTTT	0.07141622	0.07808	0.139694	0.111027
tsrna-08620	AAGGTATTAGAAAAACCATTTCATA	0	0	0.069847	0.111027
tsrna-08619	AAGGTATTAGAAAAACCATTTCA	0	0	0.069847	0
tsrna-08616	AAGGTATTAGAAAAACC	0	0.07808	0	0
tsrna-08614	AAGGGCTTAGCTTAATTAAAGTGGCTGATT	0.64274595	1.717762	0.349234	3.441841
tsrna-08613	AAGGGCTTAGCTTAATTAAAGTGGCTGA	0.35708108	0.936961	0.349234	2.220543
tsrna-08612	AAGGGCTTAGCTTAATTAAAGTGGCTG	0.21424865	1.093122	0.349234	1.887461
tsrna-08611	AAGGGCTTAGCTTAATTAAAGTGGCT	0.21424865	0.624641	0.139694	0.77719
tsrna-08610	AAGGGCTTAGCTTAATTAAAGTGGC	0.35708108	0.624641	0.069847	0.888217
tsrna-08609	AAGGGCTTAGCTTAATTAAAGTGG	0.35708108	0.468481	0.279387	0.77719
tsrna-08608	AAGGGCTTAGCTTAATTAAAGTG	0.28566487	0.390401	0.069847	1.332326
tsrna-08607	AAGGGCTTAGCTTAATTAAAGT	0.14283243	0.23424	0.069847	0.222054
tsrna-08606	AAGGGCTTAGCTTAATTAAAG	0.21424865	0.468481	0.069847	0.333081
tsrna-08605	AAGGGCTTAGCTTAATTA	0.07141622	0.23424	0	0.555136
tsrna-08604	AAGGGCTTAGCTTAATT	0.14283243	0.07808	0	0
tsrna-08587	AAGGATAACAGCTATCCATTGGTCTTAG	0	0	0	0
tsrna-08585	AAGGATAACAGCTATCCATTGGTCT	0	0.07808	0	0
tsrna-08581	AAGCTCCGAGGTGATTTTCATA	0.07141622	0	0	0
tsrna-08580	AAGCTCACAAGAACTGCTAACTCATGCCCCCATGTCTAACAA(	0	0.07808	0.20954	0.222054
tsrna-08579	AAGCTCACAAGAACTGCTAACTCATGCCCCCATGTC	0.14283243	0.15616	0	0.111027
tsrna-08578	AAGCTCACAAGAACTGCTAACTCATGCCCC	0.28566487	0.07808	0.069847	0
tsrna-08577	AAGCTCACAAGAACTGCTAACTCATGCC	0.14283243	0	0	0
tsrna-08576	AAGCTCACAAGAACTGCTAACTCATGC	0	0.07808	0	0.111027
tsrna-08575	AAGCTCACAAGAACTGCTAACTCATG	0	0.07808	0	0.222054
tsrna-08574	AAGCTCACAAGAACTGCTAACTCA	0.07141622	0	0	0
tsrna-08573	AAGCTCACAAGAACTGCTAACTC	0.07141622	0.07808	0	0
tsrna-08572	AAGCTCACAAGAACTGCTAACT	0	0	0	0.111027
tsrna-08571	AAGCTCACAAGAACTGCTAAC	0.07141622	0	0	0.111027
tsrna-08570	AAGCTCACAAGAACTGCT	0.07141622	0	0	0
tsrna-08569	AAGCTATCGGGCCCATACCCCGAAAATGTT	0.07141622	0.23424	0.069847	0
tsrna-08568	AAGCTATCGGGCCCATACCCCGAAA	0	0	0.069847	0

tsrna-08567	AAGCTATCGGGCCCATACCCCGAA	0	0.15616	0	0
tsrna-08566	AAGCTATCGGGCCCATACCCC	0	0.07808	0.069847	0
tsrna-08565	AAGCTATCGGGCCCATACC	0	0	0	0.222054
tsrna-08563	AAGCGTGCTGGGCCCATAAC	0	0	0	0
tsrna-08562	AAGCGTGCTGGGCCCA	0	0	0	0
tsrna-08560	AAGCCAGGGATTGTGGGTTCGAGTCCCATCTGGGGTGCCA	0.57132973	1.795843	0.349234	0.222054
tsrna-08559	AAGCCAGGGATTGTGGGTTCGAGTCCCATCTGGGGTGCC	0.35708108	1.093122	0.20954	0.111027
tsrna-08558	AAGCCAGGGATTGTGGGTTCGAGTCCCATCTGGGGTGC	0.35708108	1.561602	0.139694	0.444109
tsrna-08557	AAGCCAGGGATTGTGGGTTCGAGTCCCATCTGGGGTG	0.28566487	0.858881	0.419081	0
tsrna-08556	AAGCCAGGGATTGTGGGTTCGAGTCCCACCTGGGGTGCCA	0.57132973	1.483522	0.069847	0
tsrna-08555	AAGCCAGGGATTGTGGGTTCGAGTCCCACCTGGGGTACC	0.28566487	1.405442	0.279387	0.333081
tsrna-08554	AAGCCAGGGATTGTGGGTTCGAGTCCCACCCGGGGTACC	0.35708108	1.093122	0.628621	0.333081
tsrna-08553	AAGCCAGGGATTGTGGGTTCGAGTCCCACCCGGGGTA	0.4284973	1.795843	0.20954	0.111027
tsrna-08552	AAGCCAGGGATTGTGGGTTCGAGTCCC	0.4284973	1.015041	0.419081	0.111027
tsrna-08551	AAGCCAGGGATTGTGGGTTCGAGTCC	0.14283243	0.858881	0.419081	0.333081
tsrna-08550	AAGCCAGGGATTGTGGGTTCGAGTC	0.14283243	0.936961	0.279387	0
tsrna-08549	AAGCCAGGGATTGTGGGTTCG	0.4284973	1.015041	0.20954	0.111027
tsrna-08548	AAGCCAGGGATTGTGGGTTC	0.14283243	0.936961	0.20954	0
tsrna-08547	AAGCCAGGGATTGTGGGTT	0.49991351	1.171202	0.139694	0.222054
tsrna-08546	AAGCCAGGGATTGTGGGT	0.28566487	0.702721	0.488928	0.222054
tsrna-08543	AAGCATTGGACTGTAAATCTAAAGACAGGGGTTAGG	0	0	0.139694	0.111027
tsrna-08542	AAGCATTGGACTGTAAATCTAAAGACAGGG	0	0	0	0
tsrna-08541	AAGCATTGGACTGTAAATCTAAAGACAGG	0.14283243	0	0.069847	0
tsrna-08540	AAGCATTGGACTGTAAATCTAAAGACA	0	0.15616	0.069847	0
tsrna-08538	AAGCATTGGACTGTAAATCTAAAGA	0	0	0	0
tsrna-08537	AAGCATTGGACTGTAAATCTAAAG	0	0	0.069847	0
tsrna-08536	AAGCATTGGACTGTAAATCTAAA	0	0	0	0
tsrna-08533	AAGCATTGGACTGTAAATCT	0	0	0.069847	0
tsrna-08528	AAGCATAGCTGCCTTCCAAGC	0	0	0.069847	0
tsrna-08527	AAGCAGTTGACCCGGGTTCGATTCCCGGCCAACGCACC	0.8569946	2.264323	0.139694	0.111027
tsrna-08526	AAGCAGTTGACCCGGGTTCG	0	0.07808	0.069847	0
tsrna-08525	AAGCAGTTGACCCGGGT	0.07141622	0.07808	0.069847	0
tsrna-08524	AAGCAGCTTCAAACCTGCCGGGGCTTCC	0.07141622	0	0.139694	0
tsrna-08523	AAGCAGCTTCAAACCTGCCGGGGCTT	0	0	0	0
tsrna-08522	AAGCAGCTTCAAACCTGCCGGGGCT	0	0.07808	0	0
tsrna-08521	AAGCAGCTTCAAACCTGCCGGGGC	0	0.07808	0	0
tsrna-08518	AAGCAGAGTGGCGCAGCGGAAGCGTGCTGG	0	0.702721	0	0.111027
tsrna-08517	AAGCACTCTGGACTCTGAATCCAGCGATCC	0.14283243	0.31232	0.069847	0.111027
tsrna-08516	AAGCACTCTGGACTCTGAATCCAGCGA	0.07141622	0.31232	0	0.111027
tsrna-08515	AAGCACTCTGGACTCTGAATCCAGCG	0.14283243	0.07808	0	0
tsrna-08514	AAGCACTCTGGACTCTGAATCCAGC	0.14283243	0.15616	0.069847	0.111027
tsrna-08512	AAGCACTCTGGACTCTGAATCC	0.14283243	0.23424	0	0
tsrna-08505	AAGCACCCAACTTACACTTAGGAGATTTC	0	0.07808	0.139694	0
tsrna-08504	AAGCACCCAACTTACACTTAGGAGATTT	0	0	0	0
tsrna-08503	AAGCACCCAACTTACACTTAGGAGATT	0	0	0	0
tsrna-08502	AAGCACCCAACTTACACTTAGGAGAT	0	0	0	0
tsrna-08501	AAGCACCCAACTTACACTTAGGAGA	0	0.15616	0	0
tsrna-08500	AAGCACCCAACTTACACTTAGGAG	0	0.07808	0	0
tsrna-08499	AAGCACCCAACTTACACTTAGGA	0.07141622	0	0	0
tsrna-08491	AAGCAATACACTGAAAATGTTTAGACGGGCTC	0.92841081	1.093122	0.279387	0.222054
tsrna-08490	AAGCAATACACTGAAAATGTTTAGACGGGCT	0	0.15616	0	0
tsrna-08489	AAGCAATACACTGAAAATGTTTAGACGGGC	0.28566487	0.23424	0	0.111027
tsrna-08487	AAGATTGAGGGTTCGAGTCCCTTCGTGGTCGCCA	0.35708108	1.405442	0.279387	0.222054
tsrna-08486	AAGATTGAGGGTTCGAGTCCCTTCGTGGTCGCC	0.21424865	0.390401	0.279387	0.222054
tsrna-08485	AAGATTGAGGGTTCGAGTCCCT	0.28566487	0.468481	0.069847	0.111027
tsrna-08484	AAGATTCTAGGTTCGACTCCTGGCTGGCTC	0.07141622	0.31232	0.069847	0.111027
tsrna-08483	AAGATTCTAGGTTCGACTCCTG	0.07141622	0.31232	0	0.111027
tsrna-08482	AAGATTCTAGGTTCGACTCC	0	0.31232	0.139694	0.111027
tsrna-08481	AAGATTCCCATTCTTGCGACCCGG	0.07141622	0.15616	0.20954	0
tsrna-08480	AAGATTCCCATTCTTGCGACCCG	0.21424865	0.31232	0	0
tsrna-08478	AAGATTCCCATTCTTGCGAC	0.14283243	0.15616	0	0
tsrna-08477	AAGATTCCAGGTTCGACTCCTGGCTGGCTC	0.07141622	0.07808	0	0
tsrna-08476	AAGATTCCAGGTTCGACTCCTG	0.07141622	0.07808	0	0
tsrna-08475	AAGATTCCAGGTTCGACTCC	0.07141622	0	0	0

tsrna-08474	AAGATTCCAGGTTCGACTC	0	0	0	0
tsrna-08473	AAGATTCCAGGTTCGACT	0	0	0	0
tsrna-08472	AAGATTCCAGGTTCGAC	0.07141622	0	0	0.111027
tsrna-08471	AAGATTAAGAGAACCAACACCTCTTTACAGTGACCA	0.14283243	0.07808	0.20954	0.111027
tsrna-08470	AAGATTAAGAGAACCAACACCTCTTTACAGTGACC	0.07141622	0.15616	0.139694	0
tsrna-08469	AAGATTAAGAGAACCAACACCTCTTTACAGTGAC	0	0	0	0
tsrna-08468	AAGATTAAGAGAACCAACACCTCTTTACAG	0	0	0	0
tsrna-08467	AAGATTAAGAGAACCAACACCTCTTTACA	0	0	0	0
tsrna-08466	AAGATTAAGAGAACCAACACCTCT	0	0	0	0
tsrna-08465	AAGATTAAGAGAACCAACACCTC	0.07141622	0	0	0
tsrna-08464	AAGATTAAGAGAACCAACACCT	0	0	0	0
tsrna-08463	AAGATTAAGAGAACCAACACC	0	0	0	0
tsrna-08448	AAGATCACGGGTTCGAACCCCG	0	0	0.069847	0
tsrna-08447	AAGAGGTCCCTGGTTCA	0.07141622	0.624641	0.139694	0.111027
tsrna-08446	AAGAGGTCCCTGGTTC	0.14283243	0.23424	0.20954	0
tsrna-08445	AAGAGGTCCCGGGTTCAAATCC	0	0.15616	0	0.111027
tsrna-08444	AAGAGGTCCCCGGTTCAAATCCGGGTGCCCCCTCC	0.49991351	0.546561	0.139694	0.111027
tsrna-08443	AAGAGGTCCCCGGTTCAAA	0.21424865	0.31232	0	0
tsrna-08442	AAGAGGTCCCCGGTTCA	0.07141622	0.23424	0	0
tsrna-08441	AAGAGGTCCCCGGTTC	0.21424865	0.07808	0	0
tsrna-08436	AAGAGAACCAACACCTCTTTACAGTGACCA	0	0.15616	0	0
tsrna-08435	AAGAGAACCAACACCTCTTTACAGTGACC	0.07141622	0.15616	0.069847	0
tsrna-08433	AAGAGAACCAACACCTCTTTACAGTG	0	0.07808	0	0
tsrna-08427	AAGACTTTTTCTCTGACCA	0.78557838	0.546561	0.279387	0.888217
tsrna-08426	AAGACTTTTTCTCTGACC	0	0	0.20954	0
tsrna-08425	AAGACTTTTTCTCTGAC	0.07141622	0	0	0.111027
tsrna-08423	AAGACAGGGGTTAGGCCTCTTTTTACCACCA	0.28566487	0.390401	0.558774	0.111027
tsrna-08422	AAGACAGGGGTTAGGCCTCTTTTTACCACC	0	0.07808	0.139694	0
tsrna-08421	AAGACAGGGGTTAGGCCTCTTTTTACCAC	0.28566487	0	0	0
tsrna-08420	AAGACAGGGGTTAGGCCTCTTTTTACCA	0.07141622	0.15616	0.069847	0.111027
tsrna-08419	AAGACAGGGGTTAGGCCTCTTTTTACC	0	0.07808	0.139694	0
tsrna-08418	AAGACAGGGGTTAGGCCTCTTTTTAC	0	0.07808	0	0
tsrna-08417	AAGACAGGGGTTAGGCCTCTT	0	0	0.069847	0
tsrna-08416	AAGACAGGGGTTAGGCCTCT	0.07141622	0.07808	0	0
tsrna-08415	AAGACAGGGGTTAGGCCTC	0	0.07808	0	0
tsrna-08413	AAGACAGGGGTTAGGCC	0	0.07808	0	0
tsrna-08412	AAGAAGCAGCTTCAAACCTGCCGGGGCTTCC	0	0.468481	0	0
tsrna-08411	AAGAAGCAGCTTCAAACCTGCCGGGGCTTC	0	0.07808	0	0
tsrna-08410	AAGAAGCAGCTTCAAACCTGCCGGGGCTT	0.07141622	0	0	0
tsrna-08409	AAGAAGCAGCTTCAAACCTGCCGGGGCT	0	0	0	0
tsrna-08408	AAGAAGCAGCTTCAAACCTGCCGGGGC	0.07141622	0	0.069847	0
tsrna-08405	AAGAAGCAGCTTCAAACC	0	0.07808	0	0
tsrna-08404	AAGAACTGCTAACTCATGCCCCCATGTCTAACAACATGGCTT	0.49991351	0.23424	0.558774	0
tsrna-08403	AAGAACTGCTAACTCATGCCCCCATGTCTAACAACATGGCTT	0.28566487	0	0.349234	0.111027
tsrna-08402	AAGAACTGCTAACTCATGCCCCCATGTCTAACAACATGGCTT	0.21424865	0.07808	0.139694	0.111027
tsrna-08401	AAGAACTGCTAACTCATGCCCCCATGTCTAACAACATGGCTT	0.07141622	0	0.069847	0
tsrna-08400	AAGAACTGCTAACTCATGCCCCCATGTCTAACAACATGGCTT	0.14283243	0.31232	0.139694	0
tsrna-08399	AAGAACTGCTAACTCATGCCCCCATGTCTAACAACATGGCT	0.14283243	0.07808	0.069847	0
tsrna-08398	AAGAACTGCTAACTCATGCCCCCATGTCTAACAACATGGC	0.07141622	0.15616	0.139694	0.111027
tsrna-08397	AAGAACTGCTAACTCATGCCCCCATGTCTAACAACATGG	0	0.31232	0.20954	0
tsrna-08396	AAGAACTGCTAACTCATGCCCCCATGTCTAACAACATG	0.14283243	0.23424	0.279387	0
tsrna-08395	AAGAACTGCTAACTCATGCCCCCATGTCTAACAACA	0.07141622	0	0.139694	0
tsrna-08394	AAGAACTGCTAACTCATGCCCCCATGTCTAACA	0.14283243	0.07808	0.069847	0
tsrna-08393	AAGAACTGCTAACTCATGCCCCCATGTCTAAC	0.07141622	0	0	0.111027
tsrna-08392	AAGAACTGCTAACTCATGCCCCCATGTCT	0	0.23424	0.069847	0
tsrna-08391	AAGAACTGCTAACTCATGCCCCCATGTC	0.07141622	0.07808	0	0.111027
tsrna-08390	AAGAACTGCTAACTCATGCCCCCATGT	0	0.07808	0	0
tsrna-08389	AAGAACTGCTAACTCATGCCCCCATG	0	0	0	0
tsrna-08388	AAGAACTGCTAACTCATGCCCCCAT	0	0	0.069847	0
tsrna-08387	AAGAACTGCTAACTCATGCCCCCA	0	0	0.069847	0.111027
tsrna-08386	AAGAACTGCTAACTCATGCCCCC	0	0	0	0
tsrna-08385	AAGAACTGCTAACTCATGCCCC	0	0	0	0
tsrna-08384	AAGAACTGCTAACTCATGCCC	0	0.07808	0	0
tsrna-08382	AAGAACTGCTAACTCATGC	0	0	0	0

tsrna-08381	AAGAACTGCTAACTCATG	0.07141622	0	0	0
tsrna-08379	AAGAACTGCTAACTCA	0	0	0	0
tsrna-08378	AACTTTGTCAAAGTTAAATTATAGGCTAA	0.35708108	0.07808	0.768315	0
tsrna-08377	AACTTTGTCAAAGTTAAATTATAGGCT	0.35708108	0.07808	0.419081	0.111027
tsrna-08376	AACTTTGTCAAAGTTAAATTATAGGC	0.4284973	0.07808	0.558774	0.111027
tsrna-08375	AACTTTACAGTCAGAGGTTCA	0.07141622	0	0.069847	0
tsrna-08374	AACTTTACAGTCAGAGGTTC	0.14283243	0.07808	0	0
tsrna-08370	AACTTGACCGCTCTGACCA	0.49991351	1.171202	0.279387	0
tsrna-08369	AACTTGACCGCTCTGACC	0.07141622	0.15616	0.069847	0
tsrna-08368	AACTTGACCGCTCTGAC	0	0	0.069847	0
tsrna-08366	AACTTCCAATTAACTAGTTTTGACAACATT	0.07141622	0.07808	0	0
tsrna-08363	AACTTCCAATTAACTAGTTTTGACAAC	0	0	0	0
tsrna-08362	AACTTCCAATTAACTAGTTTTGACAA	0	0	0.069847	0
tsrna-08361	AACTTCCAATTAACTAGTTTTGACA	0	0	0.069847	0
tsrna-08355	AACTTAGCATTAACCTTTTAAGTTAAAGATTAAGAGAACC	0	0.07808	0	0
tsrna-08354	AACTTAGCATTAACCTTTTAAGTTAAAGATTAAGAG	0.07141622	0	0	0
tsrna-08353	AACTTAGCATTAACCTTTTAAGTTA	0.07141622	0	0	0
tsrna-08347	AACTTACACTTAGGAGATTTCAACTTAACTTGACCGCTCTGAC	6.49887569	3.669765	6.076672	1.110271
tsrna-08346	AACTTACACTTAGGAGATTTCAACTTAACTTGACCGCTCTGAC	0.92841081	1.249282	1.257243	0.111027
tsrna-08345	AACTTACACTTAGGAGATTTCAACTTAACTTGACCGCTCTGAC	0.28566487	0	0.558774	0
tsrna-08344	AACTTACACTTAGGAGATTTCAACTTAACTTGACCGCTCTGA	0.28566487	0.31232	0.488928	0
tsrna-08343	AACTTACACTTAGGAGATTTCAACTTAACTTGACCGCTCTG	0.35708108	0.07808	0.419081	0
tsrna-08342	AACTTACACTTAGGAGATTTCAACTTAACTTGACCGCTCT	0	0.15616	0.349234	0
tsrna-08341	AACTTACACTTAGGAGATTTCAACTTAACTTGACCGCTC	0	0.31232	0.628621	0.111027
tsrna-08340	AACTTACACTTAGGAGATTTCAACTTAACTTGACCGCT	0.21424865	0.07808	0.488928	0
tsrna-08339	AACTTACACTTAGGAGATTTCAACTTAACTTGACCGC	0	0.07808	0.488928	0
tsrna-08338	AACTTACACTTAGGAGATTTCAACTTAACTTGACCG	0.21424865	0	1.047702	0
tsrna-08337	AACTTACACTTAGGAGATTTCAACTTAACTTGACC	0.4284973	0.07808	0.419081	0
tsrna-08336	AACTTACACTTAGGAGATTTCAACTTAACT	0	0	0.139694	0
tsrna-08334	AACTTACACTTAGGAGATTTCAACTTA	0	0	0.279387	0
tsrna-08333	AACTTACACTTAGGAGATTTCAACTT	0	0	0.069847	0
tsrna-08332	AACTTACACTTAGGAGATTTCAACT	0.14283243	0	0.069847	0
tsrna-08331	AACTTACACTTAGGAGATTTCAAC	0	0	0	0
tsrna-08329	AACTTACACTTAGGAGATTTCA	0	0	0.069847	0
tsrna-08328	AACTTACACTTAGGAGATTTC	0	0.07808	0	0
tsrna-08322	AACTTAACTTGACCGCTCTGACCA	1.49974054	1.327362	1.117549	0.111027
tsrna-08321	AACTTAACTTGACCGCTCTGACC	0.07141622	0.07808	0.20954	0
tsrna-08320	AACTTAACTTGACCGCTCTGAC	0	0	0	0
tsrna-08319	AACTTAACTTGACCGCTCTGA	0.21424865	0	0	0
tsrna-08318	AACTTAACTTGACCGCTCTG	0	0	0	0.111027
tsrna-08317	AACTTAACTTGACCGCTCT	0	0	0	0
tsrna-08308	AACTTAAAACTTTACAATCAG	0	0	0	0
tsrna-08307	AACTGCTAACTCATGCCCCCATGTCTAACAACATGGCTTTCTC	0.78557838	0.390401	2.933566	0
tsrna-08306	AACTGCTAACTCATGCCCCCATGTCTAACAACATGGCTTTCTC	0.57132973	0.07808	1.466783	0
tsrna-08305	AACTGCTAACTCATGCCCCCATGTCTAACAACATGGCTTTCTC	0.07141622	0.390401	0.20954	0
tsrna-08304	AACTGCTAACTCATGCCCCCATGTCTAACAACATGGCT	0.07141622	0.07808	0	0.111027
tsrna-08303	AACTGCTAACTCATGCCCCCATGTCTAACAACATGGC	0	0.07808	0	0.111027
tsrna-08302	AACTGCTAACTCATGCCCCCATGTCTAACAACATGG	0.07141622	0.07808	0	0
tsrna-08301	AACTGCTAACTCATGCCCCCATGTCTAACAACA	0.07141622	0	0	0
tsrna-08300	AACTGCTAACTCATGCCCCCATGTCTAACA	0.07141622	0	0	0
tsrna-08299	AACTGCTAACTCATGCCCCCATGTCTAAC	0	0.07808	0	0
tsrna-08298	AACTGCTAACTCATGCCCCCATGTCT	0	0	0	0
tsrna-08296	AACTGCTAACTCATGCCCCCATGT	0	0.07808	0	0
tsrna-08295	AACTGCTAACTCATGCCCCCATG	0	0	0	0.111027
tsrna-08294	AACTGCTAACTCATGCCCCCAT	0	0	0	0
tsrna-08284	AACTCATGCCCCCATGTCTAACAACATGGCTTTCTCACCA	1.14265946	0.780801	2.304945	0.111027
tsrna-08283	AACTCATGCCCCCATGTCTAACAACATGGCTTTCTCACC	0.21424865	0.07808	0.698468	0.111027
tsrna-08282	AACTCATGCCCCCATGTCTAACAACATGGCT	0	0	0.139694	0
tsrna-08281	AACTCATGCCCCCATGTCTAACAACATGGC	0	0	0	0
tsrna-08277	AACTCATGCCCCCATGTCTAACA	0	0	0	0
tsrna-08266	AACTAATACACCAGTCTTG	0.07141622	0.15616	0.279387	0
tsrna-08265	AACTAAGTGTTTGTGGGTTTAAGTCCCATTGGTCTAGCCA	0.14283243	0.15616	0.279387	0.111027
tsrna-08264	AACTAAGTGTTTGTGGGTTTAAGTCCCATTGGTCTA	0.28566487	0	0.20954	0
tsrna-08263	AACTAAGTGTTTGTGGGTTTA	0	0.07808	0	0

tsrna-08262	AACTAAGTGTTTGTGGGTTT	0.07141622	0	0.069847	0.111027
tsrna-08261	AACTAAGTGTTTGTGGGTT	0	0	0	0
tsrna-08260	AACTAAGTGTTTGTGGG	0	0.07808	0	0
tsrna-08258	AACGGTAGCGCGTCTGACTCC	0.21424865	0.390401	0	0.666163
tsrna-08257	AACGGTAGCGCGTCTGACT	0	0.15616	0	0
tsrna-08255	AACGCCAAGGTCGCGGGTTCG	0	0.07808	0.139694	0
tsrna-08253	AACGATGGTTTTTCATATCATTGGTCGTGGTTGTAGTCCGTG(	1.35690811	1.483522	1.257243	1.221299
tsrna-08252	AACGATGGTTTTTCATATCATTGGTCGTGGTTGTAGTCC	0.28566487	0.07808	0.419081	0
tsrna-08251	AACGATGGTTTTTCATATCATTGGTCGTGGTTGTAGTC	0	0.390401	0.139694	0
tsrna-08250	AACGATGGTTTTTCATATCATTGGTCGTGGTTGTAGT	0.28566487	0.07808	0.349234	0
tsrna-08249	AACGATGGTTTTTCATATCATTGGTCGTGG	0.07141622	0	0	0
tsrna-08248	AACGATGGTTTTTCATATCATTGGTCG	0	0	0	0
tsrna-08247	AACGATGGTTTTTCATATCA	0	0.07808	0	0
tsrna-08246	AACGATGGTTTTTCATATC	0	0	0.069847	0
tsrna-08245	AACGAATGATTTCGACTCATTAAATTATG	0.07141622	0.15616	0.20954	0
tsrna-08244	AACGAATGATTTCGACTCATT	0	0.23424	0	0.111027
tsrna-08243	AACGAATGATTTCGACTCA	0	0.15616	0.069847	0
tsrna-08240	AACCTTTTTCCAAGGACACCA	0.28566487	0.15616	0.20954	0
tsrna-08239	AACCTTTTTCCAAGGACACC	0.07141622	0	0	0
tsrna-08238	AACCTTTTAAGTTAAAGATTAAGAGAACC	0	0.07808	0	0
tsrna-08237	AACCTGCCGGGGCTTCCA	0	0.07808	0	0
tsrna-08233	AACCGGGCGGAAACACCA	18.3539676	54.89032	7.194221	4.441086
tsrna-08232	AACCGGGCGGAAACACC	4.49922163	14.9133	2.863719	0.999244
tsrna-08231	AACCGGGCGGAAACAC	0	0.07808	0	0
tsrna-08230	AACCGGGCAGAAGCACCA	2.35673514	9.681934	0.908008	0.222054
tsrna-08229	AACCGGGCAGAAGCACC	0	0.390401	0.139694	0.111027
tsrna-08228	AACCGGGCAGAAGCAC	0	0	0	0
tsrna-08227	AACCGGAGATGAAAACCTTTTTCCAAGGACACCA	2.42815136	2.888964	7.194221	0
tsrna-08226	AACCGGAGATGAAAACCTTTTTCCAAGGACACC	0.4284973	1.483522	0.838162	0.111027
tsrna-08225	AACCGGAGATGAAAACCTTTTTCCAAGGAC	0.07141622	1.015041	0.279387	0
tsrna-08224	AACCGGAGATGAAAACCTTTTTCCAAGG	0.14283243	0.780801	0	0
tsrna-08223	AACCGGAGATGAAAACCTTTTTCCAAG	0.21424865	0.780801	0.139694	0
tsrna-08222	AACCGGAGATGAAAACCTTTTTCCAA	0	0.390401	0.069847	0
tsrna-08221	AACCGGAGATGAAAACCTT	0.07141622	0	0	0
tsrna-08220	AACCGGAGATGAAAACCT	0	0	0	0
tsrna-08219	AACCGGAGATGAAAACC	0	0	0	0
tsrna-08217	AACCGAAGATCGCGGGTTCGAACC	0.14283243	0.858881	0.20954	0
tsrna-08216	AACCGAAGATCGCGGGTTCGAAC	0.28566487	0.23424	0	0
tsrna-08215	AACCGAAGATCGCGGGTTCGAA	0.14283243	1.093122	0.069847	0.111027
tsrna-08214	AACCGAAGATCGCGGGTTCGA	0.49991351	0.468481	0	0
tsrna-08213	AACCGAAAGGTTGGTGGTTCG	0	0.07808	0	0
tsrna-08211	AACCCTGCTCGCTGCGCCA	1.07124325	2.186243	0.349234	0.111027
tsrna-08210	AACCCCGTCCGTGCCTCCA	0.28566487	0.858881	0	0.111027
tsrna-08209	AACCCCCTTATTTCTACCA	0	0.07808	0.069847	0
tsrna-08208	AACCCCCTTATTTCTACC	0	0.15616	0	0
tsrna-08205	AACCCAGAGGTCGATGGATCGAAACC	0.07141622	0.07808	0.069847	0.111027
tsrna-08203	AACCCAGAGGTCGATGGATCGAA	0.07141622	0.15616	0.069847	0.111027
tsrna-08202	AACCCAGAGGTCGATGGATCGA	0	0.07808	0.069847	0
tsrna-08201	AACCCAGAGGTCGATGGATCG	0	0.07808	0	0
tsrna-08200	AACCCAGAGGTCGATGGATC	0	0.15616	0.069847	0.111027
tsrna-08199	AACCCAGAGGTCGATGGAT	0	0.23424	0	0
tsrna-08198	AACCCAGAGGTCGATGGA	0	0.23424	0	0
tsrna-08197	AACCCAGAGGTCGATGG	0.07141622	0	0	0
tsrna-08196	AACCCAGAGGTCGATG	0.07141622	0	0	0
tsrna-08195	AACCCAGAGGTCAATGGATCGAAG	0	0	0	0.111027
tsrna-08194	AACCCAGAGGTCAATGGATCGA	0	0	0	0.111027
tsrna-08193	AACCATTTCATAACTTTGTCA	0	0.07808	0	0
tsrna-08192	AACCATTTCATAACTTTGTC	0	0.07808	0.069847	0
tsrna-08191	AACCATCCTCTGCTACCA	0.14283243	0.468481	0.069847	0
tsrna-08190	AACCATCCTCTGCTACC	0.07141622	0	0	0
tsrna-08189	AACCATCCTCTGCTAC	0	0	0	0
tsrna-08188	AACCAGGGGTCGCGAGTTCA	0	0	0	0
tsrna-08187	AACCAGGGGTCGCGAGTTC	0	0.15616	0	0
tsrna-08186	AACCAGGGGTCGCGAGTT	0	0.07808	0	0

tsrna-08185	AACCAGGGGTCGCGAGT	0	0.07808	0.139694	0
tsrna-08184	AACCAGGGGTCGCGAG	0	0	0	0
tsrna-08183	AACCAGGCGGAAACACCA	0.28566487	1.093122	0.20954	0
tsrna-08182	AACCAGCTTTGGGGGGTTC	0	0.07808	0	0
tsrna-08180	AACCAACACCTCTTTACAGTGACCA	0	0	0.069847	0
tsrna-08173	AACCAAAACATCAGATTGTGAATCTGACAACAGAGGCTTACG/	0.14283243	0.702721	0.488928	0.111027
tsrna-08172	AACCAAAACATCAGATTGTGAATCTGACAACAGAGGCT	0.21424865	0.23424	0.20954	0
tsrna-08171	AACCAAAACATCAGATTGTGAATCTGACAACAGAGG	0.07141622	0.23424	0.069847	0
tsrna-08170	AACCAAAACATCAGATTGTGAATCTGACAA	0.07141622	0.15616	0.069847	0
tsrna-08169	AACCAAAACATCAGATTGTGAATCTGACA	0.07141622	0.07808	0.139694	0
tsrna-08168	AACCAAAACATCAGATTGTGAATCTGAC	0	0.07808	0	0
tsrna-08167	AACCAAAACATCAGATTGTGAATCT	0	0.07808	0	0
tsrna-08157	AACATGGCTTTCTCACCA	0	0.07808	0	0
tsrna-08154	AACATCAGATTGTGAATCTGACAACAGAGGCTTACGACCCCT	1.78540541	1.249282	2.793872	0
tsrna-08153	AACATCAGATTGTGAATCTGACAACAGAGGCTTACGACCCCT	0.71416216	1.171202	0.558774	0
tsrna-08152	AACATCAGATTGTGAATCTGACAACAGAGGCTTACGACCCC	0.35708108	0.936961	0.558774	0
tsrna-08151	AACATCAGATTGTGAATCTGACAACAGAGGCTTACGACCC	0.14283243	0.546561	0.349234	0
tsrna-08150	AACATCAGATTGTGAATCTGACAACAGAGGCTTACGACC	0.28566487	0.390401	0.558774	0
tsrna-08149	AACATCAGATTGTGAATCTGACAACAGAGGCTTACGAC	0.28566487	0.546561	0.349234	0.111027
tsrna-08148	AACATCAGATTGTGAATCTGACAACAGAGGCTTACGA	0.14283243	0.546561	0.20954	0.111027
tsrna-08147	AACATCAGATTGTGAATCTGACAACAGAGGCTTACG	0.14283243	0.546561	0.279387	0.111027
tsrna-08146	AACATCAGATTGTGAATCTGACAACAGAGGCTTAC	0.14283243	0.546561	0.419081	0
tsrna-08145	AACATCAGATTGTGAATCTGACAACAGAGGCTT	0.07141622	0.858881	0.20954	0.111027
tsrna-08144	AACATCAGATTGTGAATCTGACAACAGAGGCT	0.07141622	0.624641	0.279387	0.111027
tsrna-08143	AACATCAGATTGTGAATCTGACAACAGAGGC	0.14283243	0.624641	0.069847	0
tsrna-08142	AACATCAGATTGTGAATCTGACAACAGAGG	0.21424865	0.390401	0.139694	0
tsrna-08141	AACATCAGATTGTGAATCTGACAACAGAG	0.14283243	0.23424	0.419081	0
tsrna-08140	AACATCAGATTGTGAATCTGACAACAGA	0.07141622	0.15616	0.20954	0
tsrna-08139	AACATCAGATTGTGAATCTGACAACAG	0	0.23424	0.279387	0
tsrna-08138	AACATCAGATTGTGAATCTGACAACA	0.07141622	0.15616	0.069847	0
tsrna-08137	AACATCAGATTGTGAATCTGACAAC	0.07141622	0.23424	0.069847	0
tsrna-08136	AACATCAGATTGTGAATCTGACAA	0	0.15616	0.139694	0
tsrna-08135	AACATCAGATTGTGAATCTGACA	0	0	0.069847	0
tsrna-08128	AACAGGAGATCCTGGGTTCGAATCCCAGCGGTGCCT	0.4284973	1.639682	0.20954	0.222054
tsrna-08127	AACAGGAGATCCTGGGTTCGAATCCCAGCGGTGCC	0.71416216	1.327362	0.279387	0.111027
tsrna-08126	AACAGGAGATCCTGGGTTCGAATCCCAGCGGTGC	0.4284973	1.873923	0.279387	0
tsrna-08125	AACAGGAGATCCTGGGTTCGAATCCCAGCGGGGCCT	0.4284973	1.717762	0.419081	0.222054
tsrna-08124	AACAGGAGATCCTGGGTTCGAATCCCAGCGGGGCC	0.49991351	1.015041	0.419081	0.111027
tsrna-08123	AACAGCTATCCATTGGTCTTAGGCCCC	0.07141622	0	0	0
tsrna-08122	AACAGCTATCCATTGGTCTTAGGCCC	0	0	0	0
tsrna-08121	AACAGCTATCCATTGGTCTTAGGCC	0	0	0	0
tsrna-08120	AACAGCTATCCATTGGTCTTAGGC	0	0	0	0
tsrna-08119	AACAGCTATCCATTGGTCTTAGG	0	0	0	0
tsrna-08118	AACAGCTATCCATTGGTCTTAG	0	0.07808	0	0
tsrna-08117	AACAGCTATCCATTGGTCTTA	0	0	0	0
tsrna-08115	AACAGCTATCCATTGGTCT	0	0	0.069847	0
tsrna-08101	AACACCAAGGTCGCGGGCTCGA	0	0.07808	0.069847	0
tsrna-08100	AACACAAAGCACCCAACTTACACTTAGGAG	0	0	0	0
tsrna-08099	AACACAAAGCACCCAACTTACACTTAGGA	0	0	0	0
tsrna-08098	AACACAAAGCACCCAACTTACACTTAG	0	0	0.069847	0
tsrna-08097	AACACAAAGCACCCAACTTACACTTA	0	0	0	0
tsrna-08095	AACACAAAGCACCCAACTTACA	0	0	0.069847	0
tsrna-08089	AACAACATGGCTTTCTCACCA	0	0.07808	0.069847	0
tsrna-08088	AACAACATGGCTTTCTCACC	0	0	0	0
tsrna-08087	AACAACATGGCTTTCTCAC	0	0	0	0
tsrna-08084	AACAAAACGAATGATTTCGACTCA	0.14283243	0.07808	0.069847	0
tsrna-08083	AACAAAACGAATGATTTCGACTC	0	0.31232	0.139694	0
tsrna-08080	AAATTTTGGTGCAACTCCAAATA	0	0.15616	0	0
tsrna-08079	AAATTTTGGTGCAACTCCA	0	0.07808	0	0
tsrna-08076	AAATTCGAAGAAGCAGCTTCAAACCTGCCGGGGCTT	0	0.15616	0.139694	0
tsrna-08075	AAATTCGAAGAAGCAGCTTCAAACCTGCCGGGGCT	0	0.07808	0.069847	0
tsrna-08073	AAATTATGATAATCATATTTACCAACC	0	0	0	0
tsrna-08069	AAATTAGAATCTTAGCTTTGGGTGCTAATGGTGGAGTTAAAG/	0.4284973	0.15616	0.139694	0.111027
tsrna-08068	AAATTAGAATCTTAGCTTTGGGTGCTAATGGTGGAG	0.07141622	0.31232	0.20954	0.111027

tsrna-08067	AAATTAGAATCTTAGCTTTGGGTGCTAATG	0.49991351	0	0.279387	0
tsrna-08066	AAATTAGAATCTTAGCTTTGGGTGC	0.21424865	0.23424	0.069847	0
tsrna-08065	AAATTAGAATCTTAGCTTTGGGTG	0	0.07808	0.139694	0.111027
tsrna-08064	AAATTAGAATCTTAGCTTTGG	0.14283243	0	0.069847	0.222054
tsrna-08063	AAATGTTTAGACGGGCTCACATCACCCCATAAA	3.28514596	1.171202	2.095404	0.222054
tsrna-08062	AAATGTTTAGACGGGCTCACATCACCCCATA	3.64222704	1.171202	1.676323	0.77719
tsrna-08061	AAATGTTTAGACGGGCTCACATCACC	1.49974054	1.093122	1.74617	0.444109
tsrna-08060	AAATGTTTAGACGGGCTCACAT	1.71398919	0.390401	0.838162	0.666163
tsrna-08059	AAATGTTTAGACGGGCTCACA	1.21407568	0.546561	0.628621	0.222054
tsrna-08058	AAATGTTTAGACGGGCTCAC	0.92841081	0.780801	0.279387	0.111027
tsrna-08057	AAATGTTTAGACGGGCTCA	0.21424865	0.07808	0.139694	0
tsrna-08056	AAATGTTTAGACGGGCTC	0.21424865	0.23424	0.069847	0.111027
tsrna-08055	AAATGTTTAGACGGGCT	0	0	0	0.111027
tsrna-08054	AAATGTTTAGACGGGC	0	0.07808	0	0
tsrna-08053	AAATGTTGGTTATACCCTTCCCGTACTACCA	0.21424865	1.561602	0.139694	0.333081
tsrna-08052	AAATGTTGGTTATACCCTTCCCGTACTACC	0.07141622	0	0	0
tsrna-08046	AAATGGTAGAGCGCTCGC	0.07141622	0.07808	0	0
tsrna-08045	AAATGGCTGAGTGAAGCATTGGACTGTAA	0.14283243	0.31232	0.069847	0
tsrna-08044	AAATGGCTGAGTGAAGCATTGGACTGTA	0.07141622	0.15616	0	0
tsrna-08043	AAATGGCTGAGTGAAGCATTGGACTGT	0	0.23424	0.069847	0
tsrna-08042	AAATGGCTGAGTGAAGCATTGGACTG	0.14283243	0	0	0
tsrna-08041	AAATGGCTGAGTGAAGCATTGGACT	0	0	0.069847	0
tsrna-08040	AAATGGCTGAGTGAAGCATTGGAC	0	0.07808	0.069847	0
tsrna-08039	AAATGGCTGAGTGAAGCATTGGA	0.07141622	0.07808	0	0
tsrna-08037	AAATGGCTGAGTGAAGCATTG	0	0	0.069847	0
tsrna-08036	AAATGGCTGAGTGAAGC	0	0.15616	0.069847	0
tsrna-08034	AAATCTGGGTGCCCCCTCCA	0.07141622	0	0.069847	0
tsrna-08033	AAATCTCGGTGGGACCTCCA	4.07072434	7.41761	1.74617	2.220543
tsrna-08032	AAATCTCGGTGGGACCTCC	0.35708108	0.624641	0.20954	0.111027
tsrna-08031	AAATCTCGGTGGGACCTC	0	0	0	0
tsrna-08030	AAATCTCGGTGGGACCCCCA	0.35708108	0.624641	0.139694	0.111027
tsrna-08029	AAATCTCGGTGGAACCTCCA	7.49870272	13.58594	1.606477	2.33157
tsrna-08028	AAATCTCGGTGGAACCTCC	0.57132973	0.702721	0.419081	0
tsrna-08027	AAATCTCGGTGGAACCTC	0	0.15616	0	0
tsrna-08026	AAATCTCGGTGGAACCT	0	0.07808	0	0
tsrna-08025	AAATCTCGGTGGAACC	0	0.07808	0	0
tsrna-08024	AAATCTCGCTGGGGCCTCCA	2.28531893	4.528646	1.53663	0.999244
tsrna-08023	AAATCTAAAGACAGGGGTTAGGCCTCTTTTTACC	2.57098379	2.498564	1.257243	0
tsrna-08022	AAATCTAAAGACAGGGGTTAGGCCTC	0.07141622	0.15616	0	0
tsrna-08021	AAATCCTATATATCTTACCA	0	0.07808	0	0
tsrna-08019	AAATCCGGGTGCCCCCTCCA	9.78402165	14.05442	4.330502	1.998488
tsrna-08018	AAATCCGGGTGCCCCCTCC	0.21424865	0.624641	0	0
tsrna-08017	AAATCCGGGTGCCCCCTC	0.07141622	0	0	0
tsrna-08016	AAATCCCGGACGAGCCCCCA	7.85578381	12.02434	3.352647	4.330058
tsrna-08015	AAATCCCGGACGAGCCCCC	0.49991351	0.468481	0	0.222054
tsrna-08014	AAATCCCGGACGAGCCCC	0.21424865	0.23424	0.069847	0
tsrna-08013	AAATCCCGGACGAGCCC	0.14283243	0.23424	0	0
tsrna-08011	AAATCCAGGTGCCCCCTCCA	0	0.15616	0.069847	0.222054
tsrna-08010	AAATCACGTCGGGGTCACCA	0.07141622	0.546561	0.069847	0.77719
tsrna-08009	AAATCACGTCGGGGTCACC	0	0.07808	0.069847	0
tsrna-08004	AAATAGTACCGTTAACTTCCAATTAACTAG	0.21424865	0.07808	0	0
tsrna-08003	AAATAGTACCGTTAACTTCCA	0	0	0.069847	0
tsrna-08002	AAATAGTACCGTTAACTTCC	0	0	0	0
tsrna-08000	AAATAGTACCGTTAACT	0	0.07808	0	0
tsrna-07997	AAATACAGACCAAGAGCCTTCA	0.14283243	0.07808	0.20954	0.222054
tsrna-07996	AAATACAGACCAAGAGCCTTC	0.07141622	0	0	0
tsrna-07995	AAATACAGACCAAGAGCCT	0	0	0	0
tsrna-07994	AAATACAGACCAAGAGCC	0	0	0	0
tsrna-07991	AAATACAACGATGGTTTTTCATATCATTGGTCGTGGTTGTAGT	0.35708108	0.07808	0.279387	0.111027
tsrna-07990	AAATACAACGATGGTTTTTCATATCATTGGTCGTGGTTGTAGT	0.21424865	0.07808	0.279387	0
tsrna-07989	AAATACAACGATGGTTTTTCATATCATTGGTCGTGGTTGTA	0.07141622	0	0.488928	0
tsrna-07988	AAATACAACGATGGTTTTTCATATCATTGGTCGT	0.07141622	0	0	0
tsrna-07986	AAATACAACGATGGTTTTTC	0	0	0.069847	0
tsrna-07985	AAATAATAGGAGCTTAAACCCCCTTATTTCTACC	0.14283243	0	0	0

tsrna-07984	AAATAATAGGAGCTTAAACCCCCTTATTTC	0.07141622	0	0	0.111027
tsrna-07983	AAATAATAGGAGCTTAAACCCCCTT	0	0	0	0
tsrna-07982	AAATAATAGGAGCTTAAACCCCCT	0	0.07808	0	0
tsrna-07980	AAATAATAGGAGCTTAAACCCC	0	0	0	0.111027
tsrna-07977	AAATAAGCTATCGGGCCCATACCCCGAAA	0	0.23424	0.069847	0
tsrna-07976	AAATAAGCTATCGGGCCCATACCCCGAA	0	0.15616	0.20954	0.111027
tsrna-07975	AAATAAGCTATCGGGCCCATACCCCGA	0.07141622	0.23424	0.139694	0
tsrna-07974	AAATAAGCTATCGGGCCCATACCCCG	0	0	0	0
tsrna-07973	AAATAAGCTATCGGGCCCATACC	0.07141622	0	0.20954	0
tsrna-07972	AAATAAGCTATCGGGCCCATA	0.07141622	0.15616	0	0
tsrna-07971	AAATAAGCTATCGGGCCCAT	0	0	0	0
tsrna-07970	AAATAAGCTATCGGGCCCA	0	0.07808	0	0
tsrna-07967	AAAGTTAAATTATAGGCT	0.07141622	0	0	0
tsrna-07966	AAAGTGGCTGATTTGCGTTCA	0	0.15616	0	0
tsrna-07962	AAAGTGGCTGATTTGCG	0	0	0	0
tsrna-07961	AAAGTGGCTGATTTGC	0	0.07808	0	0
tsrna-07960	AAAGTCATGGAGGCCATGGGGTTGG	0.14283243	0.23424	0.069847	0
tsrna-07959	AAAGTCATGGAGGCCATGGGGTTG	0.07141622	0.15616	0.139694	0
tsrna-07958	AAAGTCATGGAGGCCATGGGGTT	0	0.15616	0.069847	0
tsrna-07957	AAAGTCATGGAGGCCATGGGGT	0	0	0	0
tsrna-07956	AAAGTCATGGAGGCCATGGGG	0	0	0	0
tsrna-07954	AAAGTCATGGAGGCCATGG	0	0	0	0
tsrna-07952	AAAGTCATGGAGGCCA	0	0	0	0
tsrna-07951	AAAGGTTGGTGGTTCGAGCCCACCCAGGGACGCCA	0.35708108	8.744972	0.139694	0.111027
tsrna-07949	AAAGGTTCCGGGTTCGAGTCCCGGCGGAGTCGC	0	0	0	0
tsrna-07948	AAAGGTTCCGGGTTCG	0.07141622	0	0	0
tsrna-07947	AAAGGTCCCTGGTTCGATCCC	0.64274595	1.171202	0.069847	0.222054
tsrna-07946	AAAGGTCCCTGGTTCGATCC	0.35708108	1.171202	0.139694	0.444109
tsrna-07945	AAAGGTCCCTGGTTCG	0.28566487	0.390401	0.20954	0.222054
tsrna-07944	AAAGGTCCCTGGTTCAATCCC	0.49991351	0.702721	0.488928	0.111027
tsrna-07943	AAAGGTCCCCGGTTCGAAACC	0	0.15616	0	0
tsrna-07942	AAAGGTCCCCGGTTCGAAAC	0	0.23424	0	0.111027
tsrna-07940	AAAGGATAACAGCTATCCATTGGTCTTAG	0	0	0	0
tsrna-07939	AAAGGATAACAGCTATCCATTGGTCTTA	0	0.07808	0	0
tsrna-07938	AAAGGATAACAGCTATCCATTGGTCTT	0	0	0	0
tsrna-07937	AAAGGATAACAGCTATCCATTGGTCT	0.07141622	0	0	0
tsrna-07935	AAAGGATAACAGCTATCCATTGG	0	0	0	0
tsrna-07932	AAAGCTCACAAGAACTGCTAACTCATGCCCCCATGT	0	0.23424	0	0.111027
tsrna-07931	AAAGCTCACAAGAACTGCTAACTCATGCCC	0	0.15616	0	0
tsrna-07930	AAAGCTCACAAGAACTGCTAACTCATGCC	0.07141622	0	0	0
tsrna-07929	AAAGCTCACAAGAACTGCTAACTCATGC	0.07141622	0.07808	0	0
tsrna-07928	AAAGCTCACAAGAACTGCTAACTCATG	0	0	0	0
tsrna-07927	AAAGCTCACAAGAACTGCTAACTCAT	0.07141622	0.07808	0	0
tsrna-07926	AAAGCTCACAAGAACTGCTAACTCA	0.07141622	0.15616	0.139694	0
tsrna-07925	AAAGCTCACAAGAACTGCTAACTC	0.07141622	0.07808	0	0
tsrna-07924	AAAGCTCACAAGAACTGCTAACT	0	0.07808	0	0
tsrna-07923	AAAGCTCACAAGAACTGCT	0.07141622	0	0	0
tsrna-07919	AAAGCACCCAACTTACACTTAGGAGATTT	0	0	0	0
tsrna-07918	AAAGCACCCAACTTACACTTAGGAG	0	0	0	0
tsrna-07917	AAAGCACCCAACTTACACTTAGG	0	0	0	0
tsrna-07912	AAAGCAATACACTGAAAATGTTTAGACGGGCTCACATCACC	8.28428111	3.669765	6.216366	0.888217
tsrna-07911	AAAGCAATACACTGAAAATGTTTAGACGGGCTC	0.49991351	0.702721	0.279387	0.111027
tsrna-07910	AAAGCAATACACTGAAAATGTTTAGACGGGCT	0	0.31232	0.139694	0
tsrna-07909	AAAGCAATACACTGAAAATGTTTAGACGGG	0.14283243	0.07808	0.069847	0
tsrna-07907	AAAGATTAAGAGAACCAACACCTCTTTACAGTGACCA	0.21424865	0.468481	0.628621	0
tsrna-07906	AAAGATTAAGAGAACCAACACCTCTTTACAGTGACC	0	0.15616	0.069847	0
tsrna-07905	AAAGATTAAGAGAACCAACACCTCTTTACAGTGAC	0	0	0.069847	0
tsrna-07904	AAAGATTAAGAGAACCAACACCTCTTTACA	0.07141622	0.15616	0	0
tsrna-07903	AAAGATTAAGAGAACCAACACCTCTTTAC	0.14283243	0	0.069847	0
tsrna-07902	AAAGATTAAGAGAACCAACACCTCT	0	0	0.20954	0.111027
tsrna-07901	AAAGATTAAGAGAACCAACACCTC	0	0	0	0
tsrna-07900	AAAGATTAAGAGAACCAACACCT	0	0	0.069847	0
tsrna-07899	AAAGATTAAGAGAACCAACACC	0	0	0	0
tsrna-07895	AAAGATTAAGAGAACCAA	0	0	0	0

tsrna-07894	AAAGATTAAGAGAACCA	0	0	0	0
tsrna-07892	AAAGACTTTTTCTCTGACCA	0.92841081	1.015041	0.628621	0.555136
tsrna-07891	AAAGACTTTTTCTCTGACC	0.14283243	0.15616	0.20954	0
tsrna-07890	AAAGACTTTTTCTCTGAC	0	0	0	0
tsrna-07889	AAAGACTTTTTCTCTGA	0	0	0.069847	0
tsrna-07887	AAAGACAGGGGTTAGGCCTCTTTTTACCACCA	0.71416216	0.31232	0.628621	0.444109
tsrna-07886	AAAGACAGGGGTTAGGCCTCTTTTTACCACC	0.14283243	0.15616	0.069847	0
tsrna-07885	AAAGACAGGGGTTAGGCCTCTTTTTACCAC	0.07141622	0.31232	0	0
tsrna-07884	AAAGACAGGGGTTAGGCCTCTTTTTACCA	0	0.07808	0	0
tsrna-07883	AAAGACAGGGGTTAGGCCTCTTTTTACC	0	0.07808	0.139694	0
tsrna-07882	AAAGACAGGGGTTAGGCCTCT	0	0	0	0
tsrna-07881	AAAGACAGGGGTTAGGCCTC	0	0	0	0
tsrna-07880	AAAGACAGGGGTTAGGCCT	0	0	0.069847	0
tsrna-07879	AAAGACAGGGGTTAGGCC	0	0	0	0
tsrna-07877	AAACTTTACAGTCAGAGGTTCA	0	0	0	0
tsrna-07876	AAACTTTACAGTCAGAGGTTC	0	0	0	0
tsrna-07874	AAACTTAAAACTTTACAGTCAGAGGTTC	0	0.15616	0	0
tsrna-07871	AAACGAATGATTTCGACTCATTAAATTATG	0.28566487	0.15616	0.419081	0.111027
tsrna-07870	AAACGAATGATTTCGACTCATT	0	0.07808	0.069847	0
tsrna-07869	AAACGAATGATTTCGACTCA	0	0	0.069847	0
tsrna-07868	AAACGAATGATTTCGACTC	0.07141622	0.23424	0.139694	0
tsrna-07866	AAACGAATGATTTCGAC	0	0	0	0
tsrna-07865	AAACCTTTTTCCAAGGACACCA	0.28566487	0.15616	0.279387	0
tsrna-07864	AAACCTTTTTCCAAGGACACC	0.07141622	0	0	0
tsrna-07863	AAACCTGCCGGGGCTTCCA	0.14283243	0.07808	0.139694	0
tsrna-07859	AAACCGGGCGGAAACACCA	20.6392865	56.84232	6.705293	3.996977
tsrna-07858	AAACCGGGCGGAAACACC	4.99913515	17.33378	1.327089	0.555136
tsrna-07856	AAACCCCCTTATTTCTACC	0.07141622	0.07808	0	0
tsrna-07854	AAACCATTTCATAACTTTGTCAAAGTTA	0.4284973	0.624641	0.558774	0.111027
tsrna-07853	AAACCATTTCATAACTTTGTCA	0.28566487	0.07808	0.20954	0.111027
tsrna-07852	AAACCATTTCATAACTTTGTC	0.07141622	0	0.20954	0
tsrna-07850	AAACCATCCTCTGCTACCA	0.21424865	0.468481	0.069847	0.333081
tsrna-07849	AAACCATCCTCTGCTACC	0	0	0	0
tsrna-07847	AAACCATCCTCTGCTA	0	0	0	0
tsrna-07846	AAACCAGGGGTCGCGAGTTC	0	0	0	0
tsrna-07845	AAACCAGGGGTCGCGAGTT	0	0.07808	0.069847	0
tsrna-07844	AAACCAGGGGTCGCGAGT	0.07141622	0.07808	0	0
tsrna-07843	AAACCAGCTTTGGGGGGTTC	0	0	0	0
tsrna-07842	AAACATCAGATTGTGAATCTGACAACAGAGGCTTACGACCCC	0.71416216	0.702721	0.768315	0.111027
tsrna-07841	AAACATCAGATTGTGAATCTGACAACAGAGGCTTACGACCCC	0.28566487	0.858881	0.698468	0
tsrna-07840	AAACATCAGATTGTGAATCTGACAACAGAGGCTTACGACCCC	0.35708108	0.468481	0.488928	0.111027
tsrna-07839	AAACATCAGATTGTGAATCTGACAACAGAGGCTTACGACCC	0.35708108	0.858881	0.419081	0
tsrna-07838	AAACATCAGATTGTGAATCTGACAACAGAGGCTTACGACC	0.14283243	0.858881	0.419081	0.222054
tsrna-07837	AAACATCAGATTGTGAATCTGACAACAGAGGCTTACGAC	0.49991351	0.702721	0.488928	0.333081
tsrna-07836	AAACATCAGATTGTGAATCTGACAACAGAGGCTTACGA	0.28566487	0.780801	0.349234	0.111027
tsrna-07835	AAACATCAGATTGTGAATCTGACAACAGAGGCTTACG	0	0.23424	0.488928	0
tsrna-07834	AAACATCAGATTGTGAATCTGACAACAGAGGCTTAC	0.14283243	0.31232	0.419081	0
tsrna-07833	AAACATCAGATTGTGAATCTGACAACAGAGGCTTA	0.28566487	0.15616	0.558774	0
tsrna-07832	AAACATCAGATTGTGAATCTGACAACAGAGGCTT	0	0.390401	0.279387	0
tsrna-07831	AAACATCAGATTGTGAATCTGACAACAGAGGCT	0.07141622	0.390401	0.349234	0
tsrna-07830	AAACATCAGATTGTGAATCTGACAACAGAGGC	0.07141622	0.15616	0.349234	0
tsrna-07829	AAACATCAGATTGTGAATCTGACAACAGAGG	0	0.07808	0.20954	0
tsrna-07828	AAACATCAGATTGTGAATCTGACAACAGAG	0.07141622	0.07808	0.069847	0
tsrna-07827	AAACATCAGATTGTGAATCTGACAACAGA	0.14283243	0.07808	0.069847	0
tsrna-07826	AAACATCAGATTGTGAATCTGACAACAG	0.07141622	0.23424	0.069847	0
tsrna-07825	AAACATCAGATTGTGAATCTGACAACA	0.07141622	0.07808	0.069847	0.111027
tsrna-07824	AAACATCAGATTGTGAATCTGACAAC	0	0.23424	0	0
tsrna-07823	AAACATCAGATTGTGAATCTGACAA	0	0.07808	0.069847	0
tsrna-07822	AAACATCAGATTGTGAATCTGACA	0	0.15616	0.069847	0
tsrna-07821	AAACATCAGATTGTGAATCTGAC	0	0	0	0
tsrna-07818	AAACATCAGATTGTGAATCT	0	0	0	0
tsrna-07814	AAACAGGAGATCCTGGGTTCGAATCCCAGCGGTGCCT	0.35708108	1.483522	0.698468	0.222054
tsrna-07813	AAACAAAACGAATGATTTCGACTCA	0	0.23424	0	0
tsrna-07810	AAAATTTTGGTGCAACTCCAAATA	0	0.07808	0	0

tsrna-07809	AAAATTTTGGTGCAACTCCA	0.07141622	0	0.069847	0
tsrna-07805	AAAATGTTTAGACGGGCTCACATCACCCCATAAACACCA	7.35587029	2.264323	5.797285	1.998488
tsrna-07804	AAAATGTTTAGACGGGCTCACATCACCCCATAAA	3.71364325	1.873923	3.422493	0.666163
tsrna-07803	AAAATGTTTAGACGGGCTCACATCACCCCATA	3.57081082	1.561602	1.74617	0.999244
tsrna-07802	AAAATGTTTAGACGGGCTCACATCACCCC	2.42815136	1.483522	1.327089	0.555136
tsrna-07801	AAAATGTTTAGACGGGCTCACATCACCC	2.42815136	1.171202	1.955711	0.666163
tsrna-07800	AAAATGTTTAGACGGGCTCACATCACC	2.14248649	0.858881	0.977855	0.555136
tsrna-07799	AAAATGTTTAGACGGGCTCACATCA	1.78540541	0.546561	1.187396	0.222054
tsrna-07798	AAAATGTTTAGACGGGCTCACAT	1.78540541	1.405442	0.977855	0
tsrna-07797	AAAATGTTTAGACGGGCTCACA	1.64257298	1.015041	0.698468	0.111027
tsrna-07796	AAAATGTTTAGACGGGCTCAC	1.07124325	0.468481	0.349234	0
tsrna-07795	AAAATGTTTAGACGGGCTCA	0.49991351	0.23424	0.20954	0.111027
tsrna-07794	AAAATGTTTAGACGGGCTC	0.28566487	0.31232	0	0
tsrna-07793	AAAATGTTTAGACGGGCT	0.07141622	0.07808	0	0
tsrna-07791	AAAATGTTGGTTATACCCTTCCCGTACTACCA	0.49991351	1.639682	0.558774	0.222054
tsrna-07790	AAAATGTTGGTTATACCCTTCCCGTACTAC	0	0.07808	0.069847	0
tsrna-07782	AAAATGGCTGAGTGAAGCATTGGACTGTA	0	0.07808	0	0
tsrna-07781	AAAATGGCTGAGTGAAGCATTGGACTGT	0	0.07808	0	0
tsrna-07780	AAAATGGCTGAGTGAAGCATTGGACTG	0	0.23424	0.069847	0
tsrna-07779	AAAATGGCTGAGTGAAGCATTGGACT	0.14283243	0.07808	0.069847	0
tsrna-07778	AAAATGGCTGAGTGAAGCATTGGAC	0	0.15616	0	0
tsrna-07777	AAAATGGCTGAGTGAAGC	0.07141622	0.07808	0	0
tsrna-07776	AAAATGGCTGAGTGAAG	0	0	0	0
tsrna-07775	AAAAGTCATGGAGGCCATGGGGTTGG	0.07141622	0.468481	0.139694	0
tsrna-07774	AAAAGTCATGGAGGCCATGGGGTT	0.14283243	0.624641	0.139694	0
tsrna-07773	AAAAGTCATGGAGGCCATGGGG	0.07141622	0	0	0
tsrna-07772	AAAAGTCATGGAGGCCATGGG	0	0.15616	0	0
tsrna-07771	AAAAGTCATGGAGGCCATG	0	0.07808	0	0
tsrna-07768	AAAACTTAAAACTTTACAGTCAGAGGTTCAATTCCTCTTC	1.07124325	1.483522	0.628621	0.444109
tsrna-07765	AAAACGAATGATTTCGACTCATTAAATT	0	0.468481	0.069847	0
tsrna-07764	AAAACGAATGATTTCGACTCATT	0.14283243	0.390401	0.069847	0
tsrna-07763	AAAACGAATGATTTCGACTCA	0	0.07808	0	0
tsrna-07762	AAAACGAATGATTTCGACTC	0	0.15616	0.139694	0
tsrna-07759	AAAACCTTTTTCCAAGGACACCA	0.07141622	0.07808	0	0
tsrna-07758	AAAACCTTTTTCCAAGGACACC	0.07141622	0	0	0
tsrna-07756	AAAACCTTTTTCCAAGGACA	0	0	0	0
tsrna-07754	AAAACCATTTCATAACTTTGTC	0.07141622	0.07808	0.20954	0
tsrna-07753	AAAACATCAGATTGTGAATCTGACAACAGAGGCTTACGACCC	0.07141622	0.702721	0.628621	0
tsrna-07752	AAAACATCAGATTGTGAATCTGACAACAGAGGCTTACGACCC	0.4284973	0.702721	0.419081	0
tsrna-07751	AAAACATCAGATTGTGAATCTGACAACAGAGGCTTACGACCC	0.07141622	0.858881	0.838162	0.111027
tsrna-07750	AAAACATCAGATTGTGAATCTGACAACAGAGGCTTACGACC	0.21424865	0.702721	0.349234	0.111027
tsrna-07749	AAAACATCAGATTGTGAATCTGACAACAGAGGCTTACGA	0.14283243	0.15616	0.768315	0
tsrna-07748	AAAACATCAGATTGTGAATCTGACAACAGAGGCTTACG	0.14283243	0.780801	0.488928	0.111027
tsrna-07747	AAAACATCAGATTGTGAATCTGACAACAGAGGCTTA	0.28566487	0.390401	0.069847	0
tsrna-07746	AAAACATCAGATTGTGAATCTGACAACAGAGGCTT	0.21424865	0.468481	0.139694	0
tsrna-07745	AAAACATCAGATTGTGAATCTGACAACAGAGGCT	0.28566487	0.468481	0.419081	0
tsrna-07744	AAAACATCAGATTGTGAATCTGACAACAGAGGC	0	0.390401	0	0
tsrna-07743	AAAACATCAGATTGTGAATCTGACAACAGAGG	0	0.15616	0.488928	0
tsrna-07742	AAAACATCAGATTGTGAATCTGACAACAGA	0.07141622	0.468481	0.20954	0
tsrna-07741	AAAACATCAGATTGTGAATCTGACAACAG	0.07141622	0.23424	0.069847	0
tsrna-07740	AAAACATCAGATTGTGAATCTGACAACA	0.21424865	0.23424	0	0.111027
tsrna-07739	AAAACATCAGATTGTGAATCTGACAAC	0.07141622	0.31232	0	0
tsrna-07738	AAAACATCAGATTGTGAATCTGACAA	0.14283243	0.07808	0.069847	0
tsrna-07737	AAAACATCAGATTGTGAATCTGACA	0	0.07808	0.139694	0
tsrna-07736	AAAACATCAGATTGTGAATCTGAC	0	0.07808	0	0
tsrna-07734	AAAACATCAGATTGTGAATCTG	0	0	0	0
tsrna-07727	AAAAATTTTGGTGCAACTCCAAATAAAAGTACC	0	0.23424	0.069847	0
tsrna-07726	AAAAATTTTGGTGCAACTCCAAATAAAAGTAC	0	0	0.069847	0.111027
tsrna-07725	AAAAATTTTGGTGCAACTCCAAATAAAAGT	0	0	0.069847	0
tsrna-07724	AAAAATTTTGGTGCAACTCCAAATA	0	0	0.139694	0
tsrna-07723	AAAAATTTTGGTGCAACTCCAAAT	0	0	0	0
tsrna-07722	AAAAATTTTGGTGCAACTCCAAA	0	0	0	0
tsrna-07720	AAAAATTTTGGTGCAACTCC	0	0.15616	0.069847	0
tsrna-07719	AAAAATTTTGGTGCAACTC	0	0.07808	0	0

tsrna-07717	AAAAAGTCATGGAGGCCATGGGGTTGG	0.07141622	1.717762	0.139694	0.111027
tsrna-07716	AAAAAGTCATGGAGGCCATGGGGTT	0.07141622	0.936961	0.069847	0.111027
tsrna-07715	AAAAAGTCATGGAGGCCATGGGG	0	0.07808	0	0
tsrna-07714	AAAAAGTCATGGAGGCCATGGG	0	0.390401	0	0
tsrna-07713	AAAAAGTCATGGAGGCCATG	0	0.15616	0	0
tsrna-07712	AAAAAGTCATGGAGGCC	0.07141622	0.15616	0	0
tsrna-07711	AAAAACCATTTCATAACTTTGTCA	0.4284973	0.07808	0.20954	0.111027
tsrna-07710	ATTTGATTCCCGGTCAGGGAACCA	0.28566487	1.405442	0	0.333081
tsrna-07709	ATTTGATTCCCGGTCAGGGAACC	0	0.15616	0	0
tsrna-07708	ATTTCGATGGCGTGGGT	0.14283243	0.07808	0.069847	0
tsrna-07707	ATTTCGACTCATTAAATTATGATAATCAT	0.14283243	0	0.069847	0.111027
tsrna-07706	ATTTCATAACTTTGTCAAAGTTAAATTATAGGCT	0.57132973	0.468481	0.698468	0.111027
tsrna-07705	ATTTCATAACTTTGTCAAAGTTAAATTAT	0.28566487	0.546561	0	0
tsrna-07704	ATTTCATAACTTTGTCAAAGTT	0.14283243	0.15616	0.069847	0
tsrna-07700	ATTTCAACTTAACTTGACCGCTCTGACCA	1.42832433	2.108163	1.885864	1.110271
tsrna-07699	ATTTCAACTTAACTTGACCGCTCTGACC	0.14283243	0.31232	0.349234	0.222054
tsrna-07698	ATTTCAACTTAACTTGACCGCTCTGAC	0.07141622	0	0.139694	0
tsrna-07697	ATTTCAACTTAACTTGACCGCTCTGA	0	0	0.069847	0.111027
tsrna-07696	ATTTCAACTTAACTTGACCGCTCTG	0	0	0.069847	0
tsrna-07695	ATTTCAACTTAACTTGACCGCTCT	0	0	0.139694	0
tsrna-07688	ATTTAGGTTAAATACAGACCAAG	0	0	0	0
tsrna-07682	ATTGTGAATCTGACAACAGAGGCTTACGACCCCTTATTTACC(	1.57115676	1.405442	1.816017	0.111027
tsrna-07681	ATTGTGAATCTGACAACAGAGGCTTACGACCCCTTATTTACC(	1.2854919	0.936961	1.885864	0
tsrna-07680	ATTGTGAATCTGACAACAGAGGCTTACGACCCCTTATTTACC(	1.49974054	1.249282	1.327089	0
tsrna-07679	ATTGTGAATCTGACAACAGAGGCTTACGACCCCTTATTTACC	1.14265946	0.31232	1.676323	0.111027
tsrna-07678	ATTGTGAATCTGACAACAGAGGCTTACGACCCCTTA	1.2854919	0.546561	0.838162	0
tsrna-07677	ATTGTGAATCTGACAACAGAGGCTTACGACCCCT	0.35708108	0.15616	0.279387	0
tsrna-07676	ATTGTGAATCTGACAACAGAGGCTTACGACCCC	0.35708108	0.07808	0.139694	0
tsrna-07675	ATTGTGAATCTGACAACAGAGGCTTACGACCC	0.07141622	0.15616	0.20954	0
tsrna-07674	ATTGTGAATCTGACAACAGAGGCTTACGACC	0	0.07808	0	0
tsrna-07673	ATTGTGAATCTGACAACAGAGGCTTACGAC	0	0.31232	0.069847	0
tsrna-07672	ATTGTGAATCTGACAACAGAGGCTTACGA	0.14283243	0	0	0
tsrna-07671	ATTGTGAATCTGACAACAGAGGCTTACG	0	0.15616	0	0
tsrna-07670	ATTGTGAATCTGACAACAGAGGCTTAC	0	0.31232	0.069847	0
tsrna-07669	ATTGTGAATCTGACAACAGAGGCTTA	0	0.15616	0.069847	0
tsrna-07668	ATTGTGAATCTGACAACAGAGGCTT	0	0.07808	0.069847	0
tsrna-07667	ATTGTGAATCTGACAACAGAGGCT	0	0.15616	0.069847	0
tsrna-07666	ATTGTGAATCTGACAACAGAGGC	0.07141622	0.15616	0	0
tsrna-07665	ATTGTGAATCTGACAACAGAGG	0	0	0	0
tsrna-07664	ATTGTGAATCTGACAACAGAG	0.07141622	0	0	0
tsrna-07663	ATTGTGAATCTGACAACAGA	0	0.15616	0	0
tsrna-07662	ATTGTGAATCTGACAACAG	0	0.07808	0	0
tsrna-07659	ATTGTGAATCTGACAA	0	0	0	0
tsrna-07658	ATTGGTGGTTCAGTGGTAGAATTCTCGCCTGCCATG	4.85630272	5.699848	4.679736	1.110271
tsrna-07657	ATTGGTGGTTCAGTGGTAGAATTCTCGCCTGCCACG	7.49870272	8.432652	4.889276	2.109516
tsrna-07656	ATTGGTGGTTCAGTGGTAGAATTCTCGCCTGCCA	3.35656217	4.919047	4.051115	1.776434
tsrna-07655	ATTGGTGGTTCAGTGGTAGAATTCTCGCCTGCC	3.71364325	3.357445	3.073259	1.221299
tsrna-07654	ATTGGTGGTTCAGTGGTAGAATTCTCGCCTGC	1.49974054	1.483522	1.257243	0.222054
tsrna-07653	ATTGGTGGTTCAGTGGTAGAATTCTCGCCTG	1.2854919	1.327362	1.466783	0.111027
tsrna-07652	ATTGGTGGTTCAGTGGTAGAATTCTCGCCT	1.64257298	1.249282	1.257243	0.555136
tsrna-07651	ATTGGTGGTTCAGTGGTAGAATTCTCGCC	1.07124325	1.249282	1.047702	0.333081
tsrna-07650	ATTGGTGGTTCAGTGGTAGAATTCTCGC	0.35708108	0.468481	0.20954	0.333081
tsrna-07649	ATTGGTGGTTCAGTGGTAGAATTCTCG	0.21424865	0.31232	0.279387	0.333081
tsrna-07648	ATTGGTGGTTCAGTGGTAGAATTCTC	0.35708108	0.390401	0.349234	0
tsrna-07647	ATTGGTGGTTCAGTGGTAGAATTCT	0.49991351	0.390401	0.279387	0.222054
tsrna-07646	ATTGGTGGTTCAGTGGTAGAATTC	0.14283243	0.390401	0.20954	0.222054
tsrna-07645	ATTGGTGGTTCAGTGGTAGAATT	0.28566487	0.624641	0.279387	0.222054
tsrna-07644	ATTGGTGGTTCAGTGGTAGAAT	0.28566487	0.15616	0	0.222054
tsrna-07643	ATTGGTGGTTCAGTGGTAGAA	0.07141622	0.07808	0.349234	0
tsrna-07642	ATTGGTGGTTCAGTGGTAGA	0	0.15616	0.069847	0
tsrna-07641	ATTGGTGGTTCAGTGGTAG	0	0	0	0.111027
tsrna-07640	ATTGGTGGTTCAGTGGTA	0	0.07808	0	0
tsrna-07638	ATTGGTGGTTCAGTGG	0	0	0	0
tsrna-07636	ATTGGTCGTGGTTGTAGTCCGTGCGAGAATACCA	6.49887569	11.0093	7.822842	4.774167

tsrna-07635	ATTGGTCGTGGTTGTAGTCCGTGCGAGAATACC	0.49991351	1.795843	0.558774	1.332326
tsrna-07634	ATTGGTCGTGGTTGTAGTCCGTGCGAGAATAC	0.57132973	0.858881	0.488928	0.999244
tsrna-07633	ATTGGTCGTGGTTGTAGTCCGTGCGAGAATA	0.35708108	1.249282	0.558774	0.999244
tsrna-07632	ATTGGTCGTGGTTGTAGTCCGTGCGAGAAT	0.21424865	0.780801	0.279387	0.888217
tsrna-07631	ATTGGTCGTGGTTGTAGTCCGTGCGAGAA	0.64274595	0.31232	0.349234	0.77719
tsrna-07630	ATTGGTCGTGGTTGTAGTCCGTGCGAGA	0.35708108	0.468481	0.419081	0.888217
tsrna-07629	ATTGGTCGTGGTTGTAGTCCGTGCGAG	0.21424865	0.23424	0.628621	0.222054
tsrna-07628	ATTGGTCGTGGTTGTAGTCCGTGCGA	0.14283243	0.546561	0.139694	0.111027
tsrna-07627	ATTGGTCGTGGTTGTAGTCCGTGCG	0.14283243	0.07808	0.279387	0.111027
tsrna-07626	ATTGGTCGTGGTTGTAGTCCGTGC	0.07141622	0.390401	0	0.111027
tsrna-07625	ATTGGTCGTGGTTGTAGTCCGTG	0.07141622	0.07808	0	0
tsrna-07624	ATTGGTCGTGGTTGTAGTCCGT	0.07141622	0.15616	0.069847	0
tsrna-07623	ATTGGTCGTGGTTGTAGTCCG	0	0.07808	0	0
tsrna-07622	ATTGGTCGTGGTTGTAGTCC	0.14283243	0.07808	0	0
tsrna-07621	ATTGGTCGTGGTTGTAGTC	0	0	0	0
tsrna-07620	ATTGGTCGTGGTTGTAGT	0	0.07808	0	0
tsrna-07613	ATTGGACTGTAAATCTAAAGACAGGGGTTAGGCCTC	0.14283243	0.15616	0	0
tsrna-07602	ATTGCAGGTTCGAGTCCTGCCGCGGTCGCCA	0.14283243	0.546561	0.069847	0
tsrna-07598	ATTGCAAATTCGAAGAAGCAGCTTCAAACCTGCCGGGGCTT	0.07141622	0.07808	0.069847	0
tsrna-07597	ATTGCAAATTCGAAGAAGCAGCTTCAAACCTGCCGGGGCT	0	0.23424	0.069847	0
tsrna-07596	ATTGAGGGTTCGAGTCCCTTCGTGGTCGCCA	0.07141622	0.31232	0	0
tsrna-07594	ATTGAAGCCAGTTGATTAGGGTGCTTAGC	0	0.07808	0.069847	0
tsrna-07593	ATTGAAGCCAGTTGATTAGGGTGCTTAG	0	0	0	0
tsrna-07592	ATTGAAGCCAGTTGATTAGGG	0.07141622	0	0	0
tsrna-07590	ATTCTTGCGACCCGGGTTCGATTCCCGGGCGGCGCACCA	1.07124325	6.636809	0.698468	0
tsrna-07589	ATTCTTGCGACCCGGGTTCGATTCCCGGGCGGCGCACC	0.28566487	1.171202	0.069847	0.222054
tsrna-07588	ATTCTTGCGACCCGGGTTCGATTCCCGGGCGGCGCAC	0.07141622	0.546561	0.20954	0.222054
tsrna-07587	ATTCTTGCGACCCGGGTTCGATTCCCGGGCGGCGCA	0.14283243	0.780801	0	0
tsrna-07586	ATTCTTGCGACCCGGGTTCGATTCCCGGGCGGCGC	0.14283243	0.546561	0	0
tsrna-07585	ATTCTTGCGACCCGGGTTCGATTCCCGGGCGGCG	0.14283243	0.390401	0	0
tsrna-07584	ATTCTTGCGACCCGGGTTCGATTCCCGGGCGGC	0.07141622	0.23424	0.069847	0
tsrna-07583	ATTCTTGCGACCCGGGTTCGATTCCCGGGCGG	0.07141622	0.31232	0	0
tsrna-07582	ATTCTTGCGACCCGGGTTCGATTCCCGGGC	0	0.936961	0.069847	0
tsrna-07581	ATTCTTGCGACCCGGGTTCGATT	0	0.31232	0	0
tsrna-07580	ATTCTTGCGACCCGGGTTCGAT	0	0.23424	0	0
tsrna-07579	ATTCTTGCGACCCGGGTTCGA	0.07141622	0	0.069847	0
tsrna-07578	ATTCTTGCGACCCGGGTTCG	0	0.07808	0	0
tsrna-07577	ATTCTTGCGACCCGGGTTC	0	0.23424	0	0
tsrna-07576	ATTCTTGCGACCCGGGTT	0	0.07808	0	0
tsrna-07566	ATTCTCGCTGGGGCCTCCA	2.07107028	3.279365	1.53663	1.998488
tsrna-07565	ATTCTCGCCTGCCACGCGGGAGGCCCGGGT	0.71416216	3.435525	0.558774	0.222054
tsrna-07564	ATTCTCGCCTGCCACGCGGGAGGCCCGG	0.14283243	0.546561	0	0
tsrna-07563	ATTCTCGCCTGCCACGCGGGAGGCCCG	0.07141622	0.468481	0	0.111027
tsrna-07562	ATTCTCGCCTGCCACGCGG	0.14283243	0.468481	0.069847	0
tsrna-07561	ATTCTCGCCTGCCACGC	0.07141622	0.468481	0	0
tsrna-07560	ATTCTCGCCTGCCACG	0	0.468481	0.069847	0
tsrna-07556	ATTCTCATAGTCCTAG	0	0	0	0
tsrna-07555	ATTCTCAGGGATGGGTTCGATTCTCATAGTCCT	0.71416216	1.795843	1.466783	1.55438
tsrna-07554	ATTCTAGGTTCGACTCCTGGCTGGCTCGCC	0.21424865	0.390401	0	0
tsrna-07553	ATTCTAGGTTCGACTCCTGGCTGGCTCGC	0	0	0	0
tsrna-07552	ATTCTAGGTTCGACTCCTGGCTGGCTCG	0	0.15616	0	0
tsrna-07549	ATTCGGTGCTGAAAGA	0.07141622	0.936961	0	0
tsrna-07548	ATTCGGCGCTTTCACCGCCGCGGCCC	0	0.31232	0.139694	0
tsrna-07547	ATTCGGCGCTCTCACCGCCGCGGCCCGGGTTCGATT	0	0.07808	0	0
tsrna-07546	ATTCGGCGCTCTCACCGCCGCGGCCCGGGTTC	0.07141622	0.07808	0	0
tsrna-07545	ATTCGGCGCTCTCACCGCCGCGGCCCGGGTT	0	0.31232	0	0.111027
tsrna-07544	ATTCGGCGCTCTCACCGCCGCGGCCCGGGT	0	0	0	0
tsrna-07543	ATTCGGCGCTCTCACCGCCGCGGCCCGGG	0.07141622	0.07808	0	0
tsrna-07542	ATTCGGCGCTCTCACCGCCGCGGCCCGG	0	0.07808	0	0
tsrna-07541	ATTCGGCGCTCTCACCGCCGCGGCCCG	0.07141622	0.07808	0	0
tsrna-07540	ATTCGGCGCTCTCACCGCCGCGGCCC	0.07141622	0.07808	0	0
tsrna-07538	ATTCGGCGCTCTCACCGC	0.07141622	0	0	0
tsrna-07537	ATTCGGCGCTCTCACC	0	0.07808	0	0
tsrna-07536	ATTCGAAGAAGCAGCTTCAAACCTGCCGGGGCTTCC	0	0.546561	0.069847	0

tsrna-07535	ATTCGAAGAAGCAGCTTCAAACCTGCCGGGGCTT	0	0.07808	0.069847	0
tsrna-07534	ATTCGAAGAAGCAGCTTCAAACCTGCCGGGGCT	0	0.07808	0	0
tsrna-07531	ATTCCTTCCTTTTTTGCCA	0	0	0	0
tsrna-07528	ATTCCTTCCTTTTTTG	0	0	0	0
tsrna-07527	ATTCCTGGTTTTCACCCAGGTGGCCCG	0.14283243	0.15616	0	0
tsrna-07526	ATTCCTGGTTTTCACCCAGGCGGCCCGGGTTCGACTCCCGG'	0.14283243	0.546561	0.069847	0.111027
tsrna-07524	ATTCCTCTTCTTAACACCA	0	0.390401	0.139694	0
tsrna-07523	ATTCCGGCTCGAAGGACCA	3.71364325	9.681934	1.816017	1.776434
tsrna-07522	ATTCCGGCTCGAAGGACC	0.07141622	0.31232	0	0
tsrna-07520	ATTCCGGCTCGAAGGA	0	0.07808	0	0
tsrna-07516	ATTCCCGGTCAGGGAACCA	0.14283243	0.468481	0.139694	0.111027
tsrna-07515	ATTCCCGGTCAGGGAACC	0	0.07808	0	0
tsrna-07513	ATTCCCGGGCGGCGCACCA	0.64274595	2.654724	0.349234	0.111027
tsrna-07512	ATTCCCGGCCCATGCACCA	0.21424865	1.093122	0.139694	0.555136
tsrna-07511	ATTCCCGGCCCATGCACC	0	0.23424	0	0
tsrna-07510	ATTCCCGGCCAATGCACCA	0	0.858881	0.139694	0
tsrna-07509	ATTCCCGGCCAATGCACC	0	0.07808	0.069847	0
tsrna-07507	ATTCCCGGCCAACGCACCA	0.14283243	0.23424	0.069847	0
tsrna-07506	ATTCCCGGCCAACGCACC	0.14283243	0	0	0
tsrna-07505	ATTCCCCGACGGGGAGCCA	0	0.15616	0	0
tsrna-07503	ATTCCCATTCTTGCGACCCGGG	0.21424865	0	0.069847	0
tsrna-07502	ATTCCCATTCTTGCGACCCGG	0.14283243	0.07808	0	0
tsrna-07501	ATTCCCATTCTTGCGACCCG	0	0	0	0
tsrna-07500	ATTCCCATTCTTGCGACC	0	0.07808	0	0
tsrna-07499	ATTCCAGGTTCGACTCCTGGCTGGCTCGCC	0.14283243	0.23424	0.069847	0
tsrna-07498	ATTCCAGGTTCGACTCCTGGCTGGCTCGC	0	0	0	0.111027
tsrna-07497	ATTCCAGGTTCGACTCCTGGCTGGCTCG	0	0	0	0
tsrna-07495	ATTCCAGGTTCGACTCCTGGC	0	0.07808	0	0
tsrna-07487	ATTCAAAGGTTGTGGGTTCGAGTCCCACCAGAGTCGCCA	0.07141622	1.249282	0	0.111027
tsrna-07486	ATTCAAAGGTTGTGGGTTCGAGTCCCACCAGAGTCG	0	0.15616	0	0
tsrna-07485	ATTCAAAGGTTGTGGGTTCGAATCCCACCAGAGTCGCCA	0.28566487	0.936961	0.069847	0.333081
tsrna-07484	ATTCAAAGGTTGTGGGTTCGAATCCCACCAGAGTCG	0	0.468481	0	0
tsrna-07483	ATTCAAAGGTTGTGGGTT	0.07141622	0.07808	0	0
tsrna-07482	ATTCAAAGGTTGTGGGT	0	0.07808	0	0
tsrna-07480	ATTCAAAGGTTCCGGGTTCGAGTCCCGGCGGAGTCGCCA	0.14283243	0.390401	0	0.111027
tsrna-07479	ATTCAAAGGTTCCGGGTTCGAGTCCCGGCGGAGTCGCC	0.07141622	0.15616	0	0
tsrna-07478	ATTCAAAGGTTCCGGGTTCGAGTCCCGGCGGAGTCG	0	0.07808	0.139694	0
tsrna-07477	ATTCAAAGGTTCCGGGTTCGAGTCCCGGCG	0	0.468481	0	0
tsrna-07476	ATTCAAAGGTTCCGGGTTCGAG	0	0.07808	0	0
tsrna-07474	ATTCAAAGGTTCCGGGT	0	0.07808	0	0
tsrna-07464	ATTAGGGTGCTTAGCTGTTAACTAAGTGTTTGTGGG	0.14283243	0.07808	0.349234	0.111027
tsrna-07463	ATTAGGGTGCTTAGCTGTTAACTAAGTGTT	0	0.15616	0.139694	0
tsrna-07462	ATTAGGGTGCTTAGCTGTTAACTA	0.07141622	0.390401	0.20954	0
tsrna-07461	ATTAGGGTGCTTAGCTGTTAACT	0.14283243	0.15616	0.20954	0
tsrna-07460	ATTAGGGTGCTTAGCTGTTAAC	0.07141622	0.15616	0.069847	0
tsrna-07459	ATTAGGGTGCTTAGCTGTTAA	0	0.07808	0	0
tsrna-07458	ATTAGGGTGCTTAGCTGTTA	0.07141622	0	0	0
tsrna-07457	ATTAGGGTGCTTAGCTGTT	0.07141622	0	0	0
tsrna-07451	ATTAGAATCTTAGCTTTGGGTGCTAATGGTGGAGTTAAAGAC'	0.14283243	0.23424	0.139694	0.111027
tsrna-07450	ATTAGAATCTTAGCTTTGGGTGCTAATGGTGGAGTT	0.14283243	0.23424	0.069847	0
tsrna-07449	ATTAGAATCTTAGCTTTGGGTGCTAATGGT	0.07141622	0.07808	0.139694	0
tsrna-07448	ATTAGAATCTTAGCTTTGGGTGCT	0	0	0	0
tsrna-07447	ATTAGAATCTTAGCTTTGGGTGC	0.07141622	0	0.139694	0
tsrna-07446	ATTAGAATCTTAGCTTTGGGTG	0	0.07808	0.069847	0
tsrna-07445	ATTAGAATCTTAGCTTTGGGT	0.07141622	0	0	0
tsrna-07444	ATTAGAATCTTAGCTTTGGG	0	0.07808	0	0
tsrna-07443	ATTAGAATCTTAGCTTTGG	0	0	0	0.111027
tsrna-07442	ATTAGAATCTTAGCTTTG	0.07141622	0	0	0
tsrna-07441	ATTAAGAGAACCAACACCTCTTTACAGTGACCA	0.14283243	0.07808	0.20954	0
tsrna-07440	ATTAAGAGAACCAACACCTCTTTACAGTGACC	0	0.07808	0	0
tsrna-07439	ATTAAGAGAACCAACACCTCTTTACAGTGAC	0	0	0.069847	0
tsrna-07438	ATTAAGAGAACCAACACCTCTTTACAGTGA	0.07141622	0	0	0
tsrna-07433	ATTAAGAGAACCAACACC	0	0	0	0
tsrna-07424	ATTAAATTATGATAATCATATTTACCAACC	0	0	0	0

tsrna-07423	ATTAAAGTGGCTGATTTGCGTTCA	0	0.07808	0	0.111027
tsrna-07422	ATTAAAGTGGCTGATTTGCGTTC	0	0.15616	0	0
tsrna-07421	ATTAAAGTGGCTGATTTGCGTT	0	0	0	0
tsrna-07420	ATTAAAGTGGCTGATTTGC	0	0	0	0
tsrna-07418	ATGTTTAGACGGGCTCACATCACCCCATAAACACCA	6.85595678	1.639682	4.470196	1.665407
tsrna-07417	ATGTTTAGACGGGCTCACATCACCCCATAAACACC	2.57098379	1.015041	1.676323	0.888217
tsrna-07416	ATGTTTAGACGGGCTCACATCACCCCATAAACA	2.35673514	0.780801	1.955711	0.77719
tsrna-07415	ATGTTTAGACGGGCTCACATCACCCCATAAA	1.92823784	0.624641	1.53663	0.666163
tsrna-07414	ATGTTTAGACGGGCTCACATCACCCCATAA	1.85682163	1.405442	1.466783	0.555136
tsrna-07413	ATGTTTAGACGGGCTCACATCACCCCATA	2.07107028	0.780801	1.816017	0.555136
tsrna-07412	ATGTTTAGACGGGCTCACATCACCCCAT	1.78540541	0.468481	2.304945	0.444109
tsrna-07411	ATGTTTAGACGGGCTCACATCACCCCA	1.64257298	1.093122	2.165251	0.999244
tsrna-07410	ATGTTTAGACGGGCTCACATCACCCC	1.14265946	0.702721	1.047702	0.333081
tsrna-07409	ATGTTTAGACGGGCTCACATCACCC	1.85682163	0.624641	1.466783	0.444109
tsrna-07408	ATGTTTAGACGGGCTCACATCACC	1.2854919	0.546561	1.047702	0.111027
tsrna-07407	ATGTTTAGACGGGCTCACATCAC	1.42832433	0.546561	1.047702	0.222054
tsrna-07406	ATGTTTAGACGGGCTCACATCA	1.35690811	0.702721	1.117549	0
tsrna-07405	ATGTTTAGACGGGCTCACATC	1.14265946	0.780801	0.488928	0
tsrna-07404	ATGTTTAGACGGGCTCACAT	1.07124325	1.171202	0.698468	0.111027
tsrna-07403	ATGTTTAGACGGGCTCACA	0.57132973	0.624641	0.558774	0
tsrna-07402	ATGTTTAGACGGGCTCAC	0.92841081	0.468481	0.20954	0.111027
tsrna-07401	ATGTTTAGACGGGCTCA	0.07141622	0.07808	0.069847	0.333081
tsrna-07400	ATGTTTAGACGGGCTC	0.07141622	0	0	0
tsrna-07399	ATGTTGGTTATACCCTTCCCGTACTACCA	0.14283243	0.702721	0.20954	0.333081
tsrna-07398	ATGTTGGTTATACCCTTCCCGTACTACC	0.07141622	0.15616	0	0
tsrna-07395	ATGTTGGTTATACCCTTCCCGTACT	0	0.07808	0	0
tsrna-07392	ATGTTGGTTATACCCTTCCCGT	0	0	0	0
tsrna-07388	ATGTGTGAGGTCCCGGGT	0.07141622	0.23424	0	0
tsrna-07387	ATGTCTAACAACATGGCTTTCTCACCA	0	0.15616	0.20954	0
tsrna-07386	ATGTCTAACAACATGGCTTTCTCACC	0	0.15616	0.139694	0
tsrna-07385	ATGTCTAACAACATGGCTTTCTCAC	0	0	0	0
tsrna-07383	ATGTCTAACAACATGGCTTTCTC	0	0	0.069847	0
tsrna-07377	ATGTCCGCGTGGGTTCGAACCCCACTCCTGGTACCA	0.14283243	0.468481	0.069847	0
tsrna-07376	ATGTCCGCGTGGGTTCGAACCCCACTCCTGGTACC	0.35708108	0.468481	0.069847	0
tsrna-07375	ATGTCCGCGTGGGTTCGAACCCCACTCCTGGTA	0.21424865	0.07808	0	0
tsrna-07374	ATGTCCGCGTGGGTTCGAACCCCACTCCTGGT	0.14283243	0.390401	0.069847	0
tsrna-07373	ATGTCCGCGTGGGTTCGAACCCCACTCCTGG	0	0.15616	0.069847	0
tsrna-07372	ATGTCCGCGTGGGTTCGAACCCCACTCCTG	0.07141622	0.15616	0	0
tsrna-07371	ATGTATGAGGTCCCGGGT	0	0.23424	0.069847	0
tsrna-07365	ATGTAGCTCAGTGGTAGAGCGCATGCTT	0	0	0	0
tsrna-07364	ATGTAGCTCAGTGGTAGAGCGCA	0	0	0	0
tsrna-07362	ATGTAGCTCAGTGGTAGAG	0	0	0.069847	0
tsrna-07359	ATGGTTTTTCATATCATTGGTCGTGGTTGTAGTCCGTGCGAG/	1.14265946	1.952003	1.117549	1.998488
tsrna-07358	ATGGTTTTTCATATCATTGGTCGTGGTTGTAGTCCGTGCGAG/	0.8569946	1.093122	0.977855	1.110271
tsrna-07357	ATGGTTTTTCATATCATTGGTCGTGGTTGTAGTCCGTGCGAG/	0.8569946	1.795843	0.908008	1.332326
tsrna-07356	ATGGTTTTTCATATCATTGGTCGTGGTTGTAGTCCGTGCGAG/	0.28566487	1.717762	1.257243	1.332326
tsrna-07355	ATGGTTTTTCATATCATTGGTCGTGGTTGTAGTCCGTGCGAG	0.71416216	0.702721	0.977855	0.444109
tsrna-07354	ATGGTTTTTCATATCATTGGTCGTGGTTGTAGTCCGTGCGA	0.35708108	0.936961	0.488928	0.77719
tsrna-07353	ATGGTTTTTCATATCATTGGTCGTGGTTGTAGTCCGTGCG	0.71416216	0.780801	0.838162	0.444109
tsrna-07352	ATGGTTTTTCATATCATTGGTCGTGGTTGTAGTCCGTGC	0.35708108	0.468481	0.419081	0
tsrna-07351	ATGGTTTTTCATATCATTGGTCGTGGTTGTAGTCCG	0.21424865	0.23424	0.20954	0.222054
tsrna-07350	ATGGTTTTTCATATCATTGGTCGTGGTTGTAGTCC	0.35708108	0	0.139694	0
tsrna-07349	ATGGTTTTTCATATCATTGGTCGTGGTTGTAGTC	0.4284973	0.15616	0.349234	0
tsrna-07348	ATGGTTTTTCATATCATTGGTCGTGGTTGTAGT	0.14283243	0.15616	0	0.111027
tsrna-07347	ATGGTTTTTCATATCATTGGTCGTGGTTGTA	0	0.07808	0.069847	0.111027
tsrna-07346	ATGGTTTTTCATATCATTGGTCGTGGTTGT	0.07141622	0.07808	0.069847	0
tsrna-07345	ATGGTTTTTCATATCATTGGTCGTGGTTG	0	0	0	0
tsrna-07344	ATGGTTTTTCATATCATTGGTCGTGG	0.07141622	0	0	0.111027
tsrna-07337	ATGGTTAGCACTCTGGGCT	0	0	0	0
tsrna-07335	ATGGTTAGCACTCTGGG	0	0.07808	0	0
tsrna-07334	ATGGTTAGCACTCTGGACTTTGAATCCAGC	0.71416216	0.702721	0.20954	0.444109
tsrna-07333	ATGGTTAGCACTCTGGACTTTGAATCCA	0.14283243	0.31232	0.139694	0.111027
tsrna-07332	ATGGTTAGCACTCTGGACTTTGAATCC	0.14283243	0.390401	0.20954	0
tsrna-07331	ATGGTTAGCACTCTGGACTTTGAATC	0.28566487	0.468481	0.069847	0

tsrna-07329	ATGGTTAGCACTCTGGACTTTGAA	0	0	0	0.111027
tsrna-07327	ATGGTTAGCACTCTGGACTTTG	0.14283243	0	0	0
tsrna-07325	ATGGTTAGCACTCTGGACTT	0	0	0	0
tsrna-07324	ATGGTTAGCACTCTGGACTCTGAATCCAGCGATCCGAGTTCA	0.49991351	0.31232	0.069847	0
tsrna-07323	ATGGTTAGCACTCTGGACTCTGAATCCAGCGATCCGAGTTC	0.4284973	0.390401	0.20954	0.111027
tsrna-07322	ATGGTTAGCACTCTGGACTCTGAATCCAGCGATCCGAGTT	0.21424865	0.702721	0.20954	0.222054
tsrna-07321	ATGGTTAGCACTCTGGACTCTGAATCCAGCGATCCGAGT	0.4284973	0.07808	0	0.444109
tsrna-07320	ATGGTTAGCACTCTGGACTCTGAATCCAGCGATCCGAG	0.07141622	0.31232	0.069847	0
tsrna-07319	ATGGTTAGCACTCTGGACTCTGAATCCAGCGATCCG	0.14283243	0	0.139694	0.222054
tsrna-07318	ATGGTTAGCACTCTGGACTCTGAATCCAGCG	0.4284973	0.468481	0.069847	0.111027
tsrna-07317	ATGGTTAGCACTCTGGACTCTGAATCCAGC	0.14283243	0.390401	0.069847	0.222054
tsrna-07316	ATGGTTAGCACTCTGGACTCTGAATCCAG	0.14283243	0.390401	0.20954	0.222054
tsrna-07315	ATGGTTAGCACTCTGGACTCTGAATCCA	0.14283243	0.31232	0.069847	0.111027
tsrna-07314	ATGGTTAGCACTCTGGACTCTGAATCC	0	0.07808	0.139694	0
tsrna-07313	ATGGTTAGCACTCTGGACTCTGAATC	0.28566487	0	0	0
tsrna-07312	ATGGTTAGCACTCTGGACTCTGAAT	0	0	0	0
tsrna-07310	ATGGTTAGCACTCTGGACTCTGA	0	0.07808	0.069847	0
tsrna-07309	ATGGTTAGCACTCTGGACTCTG	0	0	0.069847	0
tsrna-07308	ATGGTTAGCACTCTGGACTCT	0	0	0.069847	0.111027
tsrna-07307	ATGGTTAGCACTCTGGACTC	0	0	0	0
tsrna-07306	ATGGTTAGCACTCTGGACT	0	0.07808	0	0
tsrna-07305	ATGGTTAGCACTCTGGAC	0	0	0	0
tsrna-07301	ATGGTGTAATGGTTAGCACTCTGGACTTTG	0.21424865	0	0	0
tsrna-07300	ATGGTGTAATGGTTAGCACTCTGGACTTT	0	0	0	0
tsrna-07299	ATGGTGTAATGGTTAGCACTCTGGACTT	0.21424865	0	0.069847	0.111027
tsrna-07298	ATGGTGTAATGGTTAGCACTCTGGACTCTGAATCCA	0.4284973	0.468481	0.419081	0.333081
tsrna-07297	ATGGTGTAATGGTTAGCACTCTGGACTCTG	0	0	0	0.222054
tsrna-07296	ATGGTGTAATGGTTAGCACTCTGGACTCT	0	0	0	0.111027
tsrna-07295	ATGGTGTAATGGTTAGCACTCTGGACTC	0.14283243	0.390401	0	0
tsrna-07294	ATGGTGTAATGGTTAGCACTCTGGACT	0	0.390401	0.139694	0.111027
tsrna-07293	ATGGTGTAATGGTTAGCACTCTGGAC	0.14283243	0.15616	0	0.111027
tsrna-07292	ATGGTGTAATGGTTAGCACTCTGGA	0	0.07808	0	0
tsrna-07290	ATGGTGTAATGGTTAGCACTCTG	0	0	0	0
tsrna-07289	ATGGTGTAATGGTTAGCACTCT	0.07141622	0	0	0
tsrna-07288	ATGGTGTAATGGTGAGCACTCTGGACTC	0.07141622	0	0	0
tsrna-07287	ATGGTGTAATGGTGAGCACTCTGGACT	0	0.15616	0	0
tsrna-07286	ATGGTGTAATGGTGAGCACTCTGGAC	0	0.07808	0	0.111027
tsrna-07282	ATGGTGTAATGGTCAGCACTCTGGACT	0.07141622	0	0	0
tsrna-07278	ATGGTGGTTCAGTGGTAGAATTCTCGCCT	2.07107028	1.171202	1.466783	0.222054
tsrna-07277	ATGGTGGTTCAGTGGTAGAATTCTCGCC	1.78540541	1.093122	0.768315	0.222054
tsrna-07276	ATGGTGGTTCAGTGGTAGAATTCTCGC	0.14283243	0.31232	0.279387	0.111027
tsrna-07275	ATGGTGGTTCAGTGGTAGAATTCTCG	0.07141622	0.07808	0.20954	0
tsrna-07274	ATGGTGGTTCAGTGGTAGAATTCTC	0.14283243	0.07808	0.069847	0
tsrna-07273	ATGGTGGTTCAGTGGTAGAATTCT	0.14283243	0.390401	0.069847	0.111027
tsrna-07272	ATGGTGGTTCAGTGGTAGAATTC	0.14283243	0.23424	0.069847	0
tsrna-07271	ATGGTGGTTCAGTGGTAGAA	0	0	0	0.111027
tsrna-07270	ATGGTGGAGTTAAAGACTTTTTCTCTGACCA	2.14248649	3.591685	0.838162	1.665407
tsrna-07269	ATGGTGGAGTTAAAGACTTTTTCTCTGACC	0.21424865	1.405442	0.069847	0.111027
tsrna-07268	ATGGTGGAGTTAAAGACTTTTTCTCTGAC	0.07141622	0.546561	0.069847	0
tsrna-07267	ATGGTGGAGTTAAAGACTTTTTCTCTGA	0	0.390401	0.20954	0
tsrna-07266	ATGGTGGAGTTAAAGACTTTTTCTCTG	0.07141622	0.31232	0	0
tsrna-07265	ATGGTGGAGTTAAAGACTTTTTCTC	0	0.31232	0	0
tsrna-07264	ATGGTGGAGTTAAAGACTTTTTCT	0.07141622	0.15616	0.139694	0
tsrna-07263	ATGGTGGAGTTAAAGACTTTTTC	0	0.15616	0	0
tsrna-07262	ATGGTGGAGTTAAAGACTTTTT	0	0.31232	0.139694	0
tsrna-07261	ATGGTGGAGTTAAAGACTTTT	0.07141622	0.07808	0.20954	0
tsrna-07258	ATGGTGGAGTTAAAGACT	0	0	0	0
tsrna-07255	ATGGTGAGCACTTTGGACTCTGA	0	0.07808	0	0
tsrna-07252	ATGGTGAGCACTCTGGACTCTGAATCCAGC	0	0.31232	0.20954	0.111027
tsrna-07251	ATGGTGAGCACTCTGGACTCTGAATCCA	0.07141622	0.07808	0.069847	0
tsrna-07250	ATGGTGAGCACTCTGGACTCTGAATCC	0.07141622	0.390401	0.069847	0.111027
tsrna-07249	ATGGTGAGCACTCTGGACTCTGAATC	0.07141622	0.23424	0	0
tsrna-07246	ATGGTGAGCACTCTGGACTCTGA	0	0.07808	0	0
tsrna-07245	ATGGTGAGCACTCTGGACTCTG	0	0.07808	0	0

tsrna-07243	ATGGTGAGCACTCTGGACTC	0	0	0	0
tsrna-07242	ATGGTGAGCACTCTGGACT	0	0	0	0
tsrna-07238	ATGGTGAGCACCCTGGACT	0	0	0	0
tsrna-07237	ATGGTCTAGCGGTTAGGATTCCTGGTTTTC	0.4284973	0.624641	0.419081	0.666163
tsrna-07236	ATGGTCTAGCGGTTAGGATTCCTGGTTTT	0.4284973	0.390401	0.279387	0.222054
tsrna-07235	ATGGTCTAGCGGTTAGGATTCCTGGTTT	0.4284973	0.702721	0	0.222054
tsrna-07234	ATGGTCTAGCGGTTAGGATTCCTGGTT	0.07141622	0.624641	0.20954	0.111027
tsrna-07233	ATGGTCTAGCGGTTAGGATTCCTGGT	0.35708108	0.468481	0.349234	0
tsrna-07232	ATGGTCTAGCGGTTAGGATTCCTGG	0.21424865	0.468481	0.069847	0.111027
tsrna-07231	ATGGTCTAGCGGTTAGGATTCCTG	0.14283243	0.07808	0	0
tsrna-07230	ATGGTCTAGCGGTTAGGATTCCT	0	0.07808	0.069847	0
tsrna-07224	ATGGTCAGCACTCTGGACTCTGAATCCAGC	0.07141622	0.23424	0.20954	0
tsrna-07223	ATGGTCAGCACTCTGGACTCTGAAT	0	0	0	0
tsrna-07215	ATGGTAGCGCGTCTGACTCC	0	0.07808	0	0
tsrna-07214	ATGGTAGCGCGTCTGACT	0	0	0	0.111027
tsrna-07211	ATGGTAGAGCGCTCGCTT	0.07141622	0.15616	0	0
tsrna-07210	ATGGTAGAGCGCTCGC	0.07141622	0.15616	0	0
tsrna-07209	ATGGTAGAATTCTCGCCT	0.07141622	0.23424	0.069847	0.111027
tsrna-07208	ATGGTAAGCACTCTGGACTCTGAATCCA	0	0.31232	0.139694	0.111027
tsrna-07207	ATGGTAAGCACTCTGGACTCTGAATCC	0.21424865	0.07808	0.139694	0
tsrna-07200	ATGGTAAGCACTCTGGACTC	0	0	0	0
tsrna-07195	ATGGGTTCGATTCTCATAGTCCTAGC	0	0.31232	0	0.111027
tsrna-07194	ATGGGTTCGATTCTCATAGTCCTAG	0	0.390401	0.069847	0.111027
tsrna-07193	ATGGGTGGTTCAGTGGTAGAATTCTCGCCTGCCA	2.64240001	4.216326	2.374791	0.999244
tsrna-07192	ATGGGTGGTTCAGTGGTAGAATTCTCGCCTGCC	2.78523244	3.591685	1.955711	0.666163
tsrna-07191	ATGGGTGGTTCAGTGGTAGAATTCTCGCCTGC	1.78540541	2.420483	1.816017	0.333081
tsrna-07190	ATGGGTGGTTCAGTGGTAGAATTCTCGCCTG	1.42832433	1.327362	1.117549	0.333081
tsrna-07189	ATGGGTGGTTCAGTGGTAGAATTCTCGCCT	1.35690811	1.717762	1.74617	0.666163
tsrna-07188	ATGGGTGGTTCAGTGGTAGAATTCTCGCC	0.8569946	1.483522	1.396936	0.222054
tsrna-07187	ATGGGTGGTTCAGTGGTAGAATTCTCGC	0.35708108	0.15616	0.20954	0.111027
tsrna-07186	ATGGGTGGTTCAGTGGTAGAATTCTCG	0.21424865	0.23424	0.20954	0.111027
tsrna-07185	ATGGGTGGTTCAGTGGTAGAATTCTC	0.14283243	0.23424	0.139694	0.111027
tsrna-07184	ATGGGTGGTTCAGTGGTAGAATTCT	0.28566487	0.23424	0.069847	0.111027
tsrna-07183	ATGGGTGGTTCAGTGGTAGAATTC	0.14283243	0.23424	0	0.111027
tsrna-07182	ATGGGTGGTTCAGTGGTAGAATT	0.21424865	0.15616	0	0
tsrna-07181	ATGGGTGGTTCAGTGGTAGAAT	0.14283243	0.07808	0.20954	0
tsrna-07180	ATGGGTGGTTCAGTGGTAGAA	0.21424865	0.07808	0	0
tsrna-07179	ATGGGTGGTTCAGTGGTAGA	0	0.07808	0	0.111027
tsrna-07174	ATGGGGTGTGATAGGTGGCACGGAG	0	0	0	0
tsrna-07167	ATGGGACTCTTAATCCCAGGGTCGTGGGTT	0.07141622	0.15616	0.069847	0
tsrna-07166	ATGGGACTCTTAATCC	0	0.07808	0.069847	0
tsrna-07165	ATGGCTGAGTGAAGCATTGGACTGTAAAT	0	0.15616	0.139694	0
tsrna-07164	ATGGCTGAGTGAAGCATTGGACTGTAAA	0.28566487	0	0.069847	0
tsrna-07163	ATGGCTGAGTGAAGCATTGGACTGTAA	0.07141622	0.23424	0.139694	0
tsrna-07162	ATGGCTGAGTGAAGCATTGGACTGTA	0	0.15616	0.069847	0
tsrna-07161	ATGGCTGAGTGAAGCATTGGACTGT	0	0	0.069847	0
tsrna-07160	ATGGCTGAGTGAAGCATTGGACTG	0	0	0.069847	0
tsrna-07159	ATGGCTGAGTGAAGCATTGGACT	0	0.15616	0	0
tsrna-07158	ATGGCTGAGTGAAGCATTGGAC	0.07141622	0.07808	0	0
tsrna-07157	ATGGCTGAGTGAAGCATTGGA	0	0.23424	0.139694	0.111027
tsrna-07156	ATGGCTGAGTGAAGCATTGG	0	0.07808	0.069847	0
tsrna-07154	ATGGCTGAGTGAAGCATT	0	0.07808	0	0
tsrna-07149	ATGGCCGAGCGGTCTAAGGCGCTGCGTTC	0	0	0.069847	0
tsrna-07136	ATGGATCGAAACCATCCTCTGCTACCA	0.92841081	1.873923	0.838162	0.888217
tsrna-07135	ATGGATCGAAACCATCCTCTGCTACC	0.14283243	0.15616	0.139694	0.111027
tsrna-07134	ATGGATCGAAACCATCCTCTGCTA	0	0	0	0
tsrna-07133	ATGGATCGAAACCATCCTCTGC	0	0	0	0
tsrna-07131	ATGGATAGCGCATTGGACTTCTAATTCAAAGGTTCCGGGT	2.49956757	2.732804	0.488928	0.999244
tsrna-07130	ATGGATAGCGCATTGGACTTC	0.14283243	0.780801	0.139694	0.222054
tsrna-07129	ATGGATAGCGCATTGGAC	0.21424865	0.546561	0.069847	0.111027
tsrna-07128	ATGGATAGCGCATTGGA	0.21424865	0.546561	0.139694	0
tsrna-07127	ATGGATAGCGCATTGG	0.35708108	0.31232	0	0
tsrna-07126	ATGGATAAGGCGTCTGATTCC	0	0.624641	0	0.111027
tsrna-07125	ATGGATAAGGCGTCTGATTC	0.07141622	0.15616	0	0.111027

tsrna-07124	ATGGATAAGGCGTCTGATT	0	0.07808	0	0
tsrna-07123	ATGGATAAGGCGTCTGACTTCGGATCAGA	0	0	0.069847	0.111027
tsrna-07122	ATGGATAAGGCGTCTGACTTCGGATC	0.14283243	0	0	0.111027
tsrna-07121	ATGGATAAGGCGTCTGACTTCGGAT	0	0	0.069847	0
tsrna-07120	ATGGATAAGGCGTCTGACTTCGGA	0.07141622	0.15616	0.069847	0
tsrna-07119	ATGGATAAGGCGTCTGACTTCGG	0.07141622	0	0	0
tsrna-07118	ATGGATAAGGCGTCTGACTTCG	0	0.07808	0	0
tsrna-07117	ATGGATAAGGCGTCTGACTTC	0.07141622	0.07808	0.069847	0
tsrna-07115	ATGGATAAGGCGTCTGACT	0	0	0	0
tsrna-07114	ATGGATAAGGCGTCTGAC	0.07141622	0.07808	0.069847	0
tsrna-07112	ATGGATAAGGCGTCTG	0.07141622	0	0	0
tsrna-07111	ATGGATAAGGCATTGGC	0.07141622	0.07808	0	0
tsrna-07110	ATGGATAAGGCATTGG	0.14283243	0.15616	0	0
tsrna-07109	ATGGATAAGGCATCAGCCTCCGGAGCTGGG	0.4284973	0.23424	0	0
tsrna-07108	ATGGATAAGGCATCAGCCTCCGGAGCTGG	0.21424865	0.15616	0.20954	0
tsrna-07107	ATGGATAAGGCATCAGCCTCCGGAGCTG	0.14283243	0.15616	0.20954	0.111027
tsrna-07106	ATGGATAAGGCATCAGCCTCC	0.21424865	0.15616	0.069847	0
tsrna-07105	ATGGATAAGGCATCAGCCTC	0.21424865	0.23424	0.069847	0.111027
tsrna-07104	ATGGATAAGGCATCAGCCT	0.28566487	0.31232	0.139694	0
tsrna-07103	ATGGATAAGGCATCAGCC	0.21424865	0.31232	0.069847	0
tsrna-07102	ATGGATAAGGCATCAGC	0.07141622	0.15616	0.139694	0.222054
tsrna-07101	ATGGATAAGGCATCAG	0.07141622	0.31232	0.20954	0
tsrna-07100	ATGGATAAGGCACTGGC	1.14265946	1.327362	0.419081	0.444109
tsrna-07099	ATGGATAAGGCACTGG	0.99982703	0.468481	0.488928	0.222054
tsrna-07098	ATGGATAACGCGTCTGACTACGGA	0.07141622	0.07808	0.069847	0
tsrna-07097	ATGGATAACGCGTCTGACTACGG	0.14283243	0.07808	0.069847	0.333081
tsrna-07096	ATGGATAACGCGTCTGACTACG	0	0.07808	0	0
tsrna-07095	ATGGATAACGCGTCTGACTAC	0	0	0	0.111027
tsrna-07093	ATGGATAACGCGTCTGACT	0	0	0	0
tsrna-07090	ATGGAGGCGTGGGTTCGAATCCCACTTCTGACACCA	1.42832433	3.747845	0.558774	0.666163
tsrna-07089	ATGGAGGCGTGGGTTCGAATCCCACTTCTGACACC	0.35708108	0.780801	0	0.111027
tsrna-07088	ATGGAGGCGTGGGTTCGAATCCCACTTCTGACA	0	0	0	0
tsrna-07087	ATGGAGGCCATGGGGTTGGCTTG	0	0.15616	0	0
tsrna-07086	ATGGAGGCCATGGGGTTGGCTT	0	0.15616	0	0
tsrna-07085	ATGGAGGCCATGGGGTTGGCT	0.07141622	0	0	0
tsrna-07084	ATGGAGGCCATGGGGTTGGC	0	0	0	0
tsrna-07083	ATGGAGGCCATGGGGTTGG	0.14283243	0	0.069847	0
tsrna-07082	ATGGAGGCCATGGGGTTG	0	0	0	0
tsrna-07081	ATGGAGGCCATGGGGTT	0	0.07808	0	0
tsrna-07080	ATGGAGGCCATGGGGT	0	0	0	0
tsrna-07079	ATGCTTTGCACGTATGAGGCCCCGGGT	2.07107028	6.168329	1.53663	3.441841
tsrna-07078	ATGCGAGAGGTAGCGGGA	0.21424865	1.171202	0	0
tsrna-07077	ATGCCGAGGTTGTGAGTTCGATC	0.14283243	0.15616	0.069847	0
tsrna-07076	ATGCCGAGGTTGTGAGTTCG	0	0.15616	0	0
tsrna-07074	ATGCCGAGGTTGTGAGTT	0	0.07808	0	0
tsrna-07073	ATGCCGAGGTTGTGAGT	0	0.15616	0	0
tsrna-07072	ATGCCCGCATTCTCCACCA	0.07141622	0.390401	0.069847	0
tsrna-07071	ATGCCCGCATCCTCCACCA	0.14283243	0.23424	0	0
tsrna-07070	ATGCCCGCATCCTCCACC	0	0.15616	0	0
tsrna-07069	ATGCCCGCATCCTCCAC	0	0.07808	0	0
tsrna-07067	ATGCCCCCATGTCTAACAACATGGCTTTCTCACCA	0.35708108	0.390401	0.698468	0.111027
tsrna-07066	ATGCCCCCATGTCTAACAACATGGCTTTCTCACC	0.21424865	0	0.20954	0
tsrna-07065	ATGCCCCCATGTCTAACAACATGGCTTTCT	0	0.07808	0.069847	0
tsrna-07064	ATGCCCCCATGTCTAACAACATGGCTTTC	0	0.15616	0	0
tsrna-07062	ATGCCCCCATGTCTAACAACATGGC	0	0.07808	0	0
tsrna-07059	ATGCCCCCATGTCTAACAACA	0	0.07808	0	0
tsrna-07054	ATGCAGAGTGGGGTTTTGCAGTCCTTACC	0.07141622	0.07808	0	0.111027
tsrna-07053	ATGCAGAGTGGGGTTTTGCAGTCCT	0	0.07808	0	0.111027
tsrna-07052	ATGCAGAGTGGGGTTTTGCAGTCC	0	0.07808	0.069847	0
tsrna-07051	ATGCAGAGTGGGGTTTTGCAGTC	0	0	0	0
tsrna-07050	ATGCAGAGTGGGGTTTTGCAG	0	0	0	0
tsrna-07049	ATGCAGAGTGGGGTTTTGCA	0	0	0	0
tsrna-07046	ATGCACGAGGTCCTGGGT	0.21424865	0.546561	0.069847	0.111027
tsrna-07045	ATGCACGAGGCCCCGGGT	0	0.07808	0	0

tsrna-07044	ATGCAAGATTCCCATTCTTGCGACCCGG	0.14283243	0.23424	0	0.111027
tsrna-07043	ATGCAAGATTCCCATTCTTGCGACCCG	0	0.07808	0.139694	0
tsrna-07042	ATGATTTCGACTCATTAAATTATGATA	0.4284973	0	0.139694	0
tsrna-07036	ATGATTCTCGGTTTGGGT	0	0.07808	0	0
tsrna-07031	ATGATTCTCGCTTTGGGT	0	0	0.069847	0
tsrna-07028	ATGATTCTCGCTTCGGGTGTGA	0.07141622	0	0	0
tsrna-07025	ATGATTCTCGCTTCGGGTGCGAG	0	0.07808	0	0
tsrna-07021	ATGATCGTATAGTGGTTAGTACTCTGCG	0.57132973	0.546561	0.20954	0
tsrna-07020	ATGATCGTATAGTGGTTAGTACTCTGC	0.14283243	0.390401	0	0.222054
tsrna-07019	ATGATCGTATAGTGGTTAGTACTCTG	0	0.07808	0	0
tsrna-07018	ATGATCGTATAGTGGTTAGTACTCT	0.14283243	0	0.069847	0
tsrna-07017	ATGATCGTATAGTGGTTAGTACTC	0	0.07808	0	0
tsrna-07009	ATGAGGTCCCGGGTTCGATCCCCGGC	0.07141622	0.31232	0	0
tsrna-07008	ATGAGGTCCCGGGTTCGATCCCCAGC	0.07141622	0.390401	0.069847	0
tsrna-07007	ATGAGGTCCCGGGTTCGATCCCC	0	0.07808	0	0
tsrna-07006	ATGAGGTCCCGGGTTCGATCCC	0.07141622	0.15616	0.069847	0
tsrna-07005	ATGAGGTCCCGGGTTCGATCC	0.07141622	0.15616	0	0
tsrna-07004	ATGAGGTCCCGGGTTCGATC	0	0.31232	0	0
tsrna-07003	ATGAGGTCCCGGGTTCG	0.14283243	0.15616	0.069847	0
tsrna-07002	ATGAGGCCCCGGGTTCGATCCCCGGC	0	0.15616	0	0
tsrna-07001	ATGAGGCCCCGGGTTCGATCCC	0.14283243	0.23424	0.069847	0
tsrna-07000	ATGAGGCCCCGGGTTCGATCC	0	0.07808	0	0.111027
tsrna-06999	ATGAGGCCCCGGGTTCGATC	0	0.15616	0	0
tsrna-06998	ATGAGGCCCCGGGTTCG	0.07141622	0.780801	0	0.111027
tsrna-06997	ATGAAAACCTTTTTCCAAGGACACCA	0.14283243	0.07808	0.279387	0
tsrna-06996	ATGAAAACCTTTTTCCAAGGACACC	0	0.15616	0	0
tsrna-06995	ATGAAAACCTTTTTCCAAGGACA	0	0.15616	0	0
tsrna-06990	ATCTTAGCTTTGGGTGCTAATGGTGGAGTT	0.07141622	0.07808	0	0
tsrna-06986	ATCTGGGTGCCCCCTCCA	0	0.07808	0	0
tsrna-06985	ATCTGAGGGTCCAGGGTTT	0.07141622	0.702721	0	0
tsrna-06984	ATCTGAGGGTCCAGGGTTCATGTCCCTGTT	0	0.07808	0	0
tsrna-06983	ATCTGAGGGTCCAGGGTTCAAGTCCCTGTTCGGGCGCCA	0.49991351	1.873923	0.349234	0.555136
tsrna-06982	ATCTGAGGGTCCAGGGTTCAAGTCCCTGTTCGGGCGCC	0.07141622	0.31232	0	0.111027
tsrna-06981	ATCTGAGGGTCCAGGGTTCAAGTCCCTGTTCGGGCG	0.07141622	0.15616	0	0
tsrna-06980	ATCTGAGGGTCCAGGGTTCAAGT	0	0.31232	0.069847	0.111027
tsrna-06979	ATCTGACAACAGAGGCTTACGACCCCTTATTTACCC	0	0	0.069847	0.111027
tsrna-06976	ATCTGAAGGTCGTGAGTTCGATCCTCACACGGGGCACCA	0.21424865	2.186243	0.139694	0
tsrna-06975	ATCTGAAGGTCGTGAGTTCGATCCTCACACGGGGCACC	0.14283243	0.468481	0.139694	0.111027
tsrna-06974	ATCTGAAGGTCGTGAGTTCGATCCTCACACGGGGCAC	0	0.31232	0.139694	0
tsrna-06973	ATCTGAAGGTCGTGAGTTCGAGCCTCACACGGGGCACCA	0.35708108	0.468481	0.139694	0
tsrna-06972	ATCTGAAGGTCGTGAGTTCGAGCCTCACACGGGGCACC	0.07141622	0.31232	0.139694	0
tsrna-06971	ATCTGAAGGTCCTGAGTTCGAACCTCAGAGGGGGCACCA	0.78557838	2.732804	0.488928	0.333081
tsrna-06970	ATCTGAAGGTCCTGAGTTCGAACCTCAGAGGGGGCACC	1.07124325	3.747845	0.419081	0.222054
tsrna-06969	ATCTGAAGGTCCTGAGT	1.21407568	2.342403	0.908008	0.77719
tsrna-06968	ATCTCGGTGGTACCTCCA	0	0.23424	0.069847	0
tsrna-06967	ATCTCGGTGGGACCTCCA	3.78505947	6.714889	0.908008	2.109516
tsrna-06966	ATCTCGGTGGGACCTCC	0.28566487	0.624641	0.139694	0.111027
tsrna-06965	ATCTCGGTGGGACCTC	0	0.07808	0	0
tsrna-06964	ATCTCGGTGGAACCTCCA	7.49870272	12.10242	2.654179	1.776434
tsrna-06963	ATCTCGGTGGAACCTCC	0.21424865	0.858881	0.20954	0.111027
tsrna-06962	ATCTCGGTGGAACCTC	0	0.15616	0	0
tsrna-06961	ATCTCGCTGGGGCCTCCA	2.49956757	4.919047	1.53663	1.221299
tsrna-06960	ATCTCGCTGGGGCCTCC	0.14283243	0.546561	0	0
tsrna-06958	ATCTAAAGGTCCCTGGTTCGATCCCGGGTTTCGGCACCA	3.71364325	12.10242	1.466783	1.887461
tsrna-06957	ATCTAAAGGTCCCTGGTTCGATCCCGGGTTTCGGCACC	1.92823784	3.747845	0.628621	0.999244
tsrna-06956	ATCTAAAGGTCCCTGGTTCGATCCCGGGTT	0.64274595	3.123204	0.628621	0.222054
tsrna-06955	ATCTAAAGGTCCCTGGTTCGATC	1.78540541	2.108163	0.558774	0.333081
tsrna-06954	ATCTAAAGGTCCCTGGTTCGAT	1.14265946	2.420483	0.349234	0.444109
tsrna-06953	ATCTAAAGGTCCCTGGTTCGA	1.2854919	2.030083	0.349234	0.555136
tsrna-06952	ATCTAAAGGTCCCTGGTTCG	0.78557838	2.186243	0.20954	0.444109
tsrna-06951	ATCTAAAGGTCCCTGGTTC	1.35690811	1.952003	0.419081	0.555136
tsrna-06950	ATCTAAAGGTCCCTGGTT	1.14265946	1.952003	0.488928	0.222054
tsrna-06949	ATCTAAAGGTCCCTGGT	0.57132973	1.093122	0.488928	0.555136
tsrna-06948	ATCTAAAGGTCCCTGG	0	0	0	0.111027

tsrna-06947	ATCTAAAGACAGGGGTTAGGCCTCTTTTTACCACC	0.35708108	0.780801	0.628621	0
tsrna-06946	ATCTAAAGACAGGGGTTAGGCCTCTTTTT	0.28566487	0.624641	0.069847	0
tsrna-06945	ATCTAAACCATCCTCTGCTACCA	0.07141622	0.468481	0	0.111027
tsrna-06944	ATCGTATAGTGGTTAGTACTCTGCGTTGTG	2.42815136	0.858881	0.628621	1.110271
tsrna-06943	ATCGTATAGTGGTTAGTACTCTGCGTTGT	1.92823784	0.858881	1.257243	1.332326
tsrna-06942	ATCGTATAGTGGTTAGTACTCTGCGTTG	1.99965406	0.936961	1.187396	0.555136
tsrna-06941	ATCGTATAGTGGTTAGTACTCTGCGTT	1.71398919	0.780801	0.908008	0.666163
tsrna-06940	ATCGTATAGTGGTTAGTACTCTGCGT	0.4284973	0.31232	0.488928	0.111027
tsrna-06939	ATCGTATAGTGGTTAGTACTCTGCG	0.4284973	0.390401	0.139694	0.333081
tsrna-06938	ATCGTATAGTGGTTAGTACTCTGC	0.35708108	0.07808	0.20954	0.111027
tsrna-06937	ATCGTATAGTGGTTAGTACTCTG	0.14283243	0	0.139694	0.111027
tsrna-06936	ATCGTATAGTGGTTAGTACTCT	0.07141622	0	0.069847	0
tsrna-06935	ATCGTATAGTGGTTAGTACTC	0.07141622	0.07808	0	0.111027
tsrna-06931	ATCGGGCCCATACCCCGAAAATGTTGGTT	0	0.07808	0	0
tsrna-06927	ATCGATGCCCGCATTCTCCACCA	0.07141622	0.31232	0	0
tsrna-06925	ATCGATGCCCGCATCCTCCACCA	0.07141622	0.23424	0	0.111027
tsrna-06921	ATCGAAACCATCCTCTGCTACCA	1.42832433	2.030083	0.488928	0.888217
tsrna-06920	ATCGAAACCATCCTCTGCTACC	0.4284973	0.23424	0.069847	0
tsrna-06918	ATCGAAACCATCCTCTGC	0	0	0	0
tsrna-06916	ATCCTTAGGTCGCTGGTTCGATTCCGGCTCGAAGGA	0.14283243	0.390401	0.139694	0.222054
tsrna-06915	ATCCTTAGGTCGCTGGTTCGATTCCGGCTC	0.21424865	0.15616	0.069847	0.111027
tsrna-06914	ATCCTTAGGTCGCTGGTTCGATT	0.14283243	0.15616	0.139694	0
tsrna-06913	ATCCTTAGGTCGCTGGTTCGAAT	0	0	0.20954	0
tsrna-06912	ATCCTTAGGTCGCTGGTTCGA	0	0.15616	0	0
tsrna-06911	ATCCTTAGGTCGCTGGTTCG	0.07141622	0	0.139694	0
tsrna-06910	ATCCTTAGGTCGCTGGTTC	0.07141622	0.07808	0.069847	0.111027
tsrna-06909	ATCCTTAGGTCGCTGGTT	0	0.07808	0	0
tsrna-06906	ATCCTGTTCGTGACGCCA	0.28566487	0.468481	0.349234	0.222054
tsrna-06903	ATCCTGTCGGCTACGCCA	0.28566487	0.546561	0.139694	0.77719
tsrna-06902	ATCCTGTCGGCTACGCC	0.07141622	0.07808	0	0
tsrna-06901	ATCCTGGGTTCGAATCCCAGCGGTGCCTCC	0.28566487	0.23424	0	0
tsrna-06900	ATCCTGGGTTCGAATCCCAGCGGGGCCTCC	0	0.07808	0	0
tsrna-06899	ATCCTGCTCACAGCGCCA	0.49991351	1.015041	0.139694	0.111027
tsrna-06898	ATCCTGCTCACAGCGCC	0.21424865	0.07808	0	0
tsrna-06897	ATCCTGCTCACAGCGC	0	0	0.069847	0
tsrna-06896	ATCCTGCCGACTACGCCA	6.42745948	17.88035	2.235098	2.220543
tsrna-06895	ATCCTGCCGACTACGCC	0.35708108	0.936961	0.139694	0
tsrna-06894	ATCCTGCCGACTACGC	0	0.07808	0	0
tsrna-06893	ATCCTCGCTGGGGCCTCCA	0.92841081	0.624641	0.069847	0.444109
tsrna-06892	ATCCTCACCTGGAGCACCA	0.07141622	0.15616	0	0.111027
tsrna-06889	ATCCGGGTGCCCCCTCCA	9.78402165	14.75714	4.120962	1.665407
tsrna-06888	ATCCGGGTGCCCCCTCC	0.35708108	0.31232	0.069847	0.222054
tsrna-06887	ATCCGGGTGCCCCCTC	0	0.23424	0	0
tsrna-06886	ATCCGGCTCGGAGGACCA	0.07141622	0.23424	0	0
tsrna-06885	ATCCGGCTCGGAGGACC	0	0.07808	0	0
tsrna-06883	ATCCGGCTCGAAGGACCA	1.71398919	2.264323	0.768315	0.333081
tsrna-06882	ATCCGGCTCGAAGGACC	0	0.15616	0	0
tsrna-06881	ATCCGGCTCGAAGGAC	0	0	0	0
tsrna-06876	ATCCCTGGTGGTCTAGTGGTTAGGATTCGG	1.42832433	3.201285	1.53663	2.109516
tsrna-06875	ATCCCTGGTGGTCTAGTGGTTAGGATTCG	1.42832433	3.045124	1.466783	2.109516
tsrna-06874	ATCCCTGGTGGTCTAGTGGTTAGGATTC	1.85682163	2.654724	1.955711	2.442597
tsrna-06873	ATCCCTCCGTGGTTACCA	0.21424865	0.780801	0	0.111027
tsrna-06872	ATCCCTCCGTGGTTACC	0	0.31232	0	0
tsrna-06871	ATCCCGGGTTTCGGCACCA	2.78523244	7.729931	0.768315	0.77719
tsrna-06870	ATCCCGGGTTTCGGCACC	0	0.468481	0.069847	0.333081
tsrna-06869	ATCCCGGGTTTCGGCAC	0	0.07808	0	0
tsrna-06867	ATCCCGGGCGGAAACACCA	1.14265946	2.342403	0.349234	0.666163
tsrna-06866	ATCCCGGACGAGCCCCCA	8.78419462	12.02434	3.212953	6.106493
tsrna-06865	ATCCCGGACGAGCCCCC	0.57132973	0.858881	0	0
tsrna-06864	ATCCCGGACGAGCCCC	0.07141622	0.15616	0	0
tsrna-06863	ATCCCCGTACTGGCCACCA	0.07141622	0.23424	0.139694	0.222054
tsrna-06862	ATCCCCGTACGGGCCACCA	0.49991351	3.982086	0.279387	0.444109
tsrna-06861	ATCCCCGGCATCTCCACCA	7.28445408	9.838094	2.863719	3.219787
tsrna-06860	ATCCCCGGCATCTCCACC	0.21424865	0.31232	0.069847	0.222054

tsrna-06857	ATCCCCGGCACCTCCACCA	11.3551784	20.14467	5.936978	2.997733
tsrna-06856	ATCCCCGGCACCTCCACC	0.4284973	0.858881	0.20954	0.333081
tsrna-06855	ATCCCCGGCACCTCCAC	0.07141622	0.07808	0	0
tsrna-06854	ATCCCCGGCACCTCCA	0	0	0	0
tsrna-06853	ATCCCCGCCTGTCACGCGGGAGACCGGGGTTCGATTC	0.92841081	3.591685	0.558774	0.555136
tsrna-06852	ATCCCCGCCTGTCACGCGGGAGACCGG	1.42832433	4.528646	0.628621	0.111027
tsrna-06851	ATCCCCGCCTGTCACGCGGGAGACCG	0.8569946	3.747845	0.279387	0.444109
tsrna-06849	ATCCCCGACACCTCCACCA	0.35708108	0.15616	0.069847	0.111027
tsrna-06848	ATCCCCAGTACCTCCACCA	0	0.15616	0.069847	0
tsrna-06844	ATCCCCAGCATCTCCACCA	0.07141622	0.390401	0	0.111027
tsrna-06842	ATCCCCAGCACCTCCACCA	0	0.07808	0	0
tsrna-06839	ATCCCATCCTCGTCGCCA	0.35708108	0.07808	0	0.111027
tsrna-06838	ATCCCATCCTCGTCGCC	0	0.07808	0	0
tsrna-06836	ATCCCATATGGTCTAGCGGTTAGGATTCCT	0.14283243	0.07808	0	0
tsrna-06835	ATCCCAGTAGAGCCTCCA	0.21424865	0.702721	0.20954	0.333081
tsrna-06834	ATCCCAGTAGAGCCTCC	0	0.07808	0	0
tsrna-06826	ATCCCAGCGAGGCCTCCA	0	0	0	0.111027
tsrna-06825	ATCCCACTTCTGACACCA	0.28566487	0.15616	0.139694	0
tsrna-06823	ATCCCACTTCTGACAC	0	0	0	0
tsrna-06822	ATCCCACTGCTGCCACCA	0	0.23424	0	0
tsrna-06821	ATCCCACTCCTGACACCA	3.9278919	5.075207	3.212953	2.997733
tsrna-06820	ATCCCACTCCTGACACC	0	0	0	0
tsrna-06819	ATCCCACTCCTGACAC	0	0	0	0.111027
tsrna-06818	ATCCCACCTTCGTCGCCA	0.07141622	0	0	0
tsrna-06817	ATCCCACCTTCGTCGCC	0	0	0	0
tsrna-06815	ATCCCACCGCTGCTACCA	0.07141622	0.390401	0.069847	0
tsrna-06814	ATCCCACCGCTGCCACCA	0	0	0	0
tsrna-06813	ATCCCACCGCTGCCACC	0	0.07808	0	0
tsrna-06812	ATCCCACCGCTGCCAC	0	0	0.069847	0
tsrna-06811	ATCCCACCCTCGTCGCCA	0.28566487	1.171202	0.139694	0.222054
tsrna-06810	ATCCCACCCTCGTCGCC	0	0.07808	0	0.111027
tsrna-06808	ATCCCACCCAGGGACGCCA	0.14283243	0.23424	0	0
tsrna-06807	ATCCCACCAGAGTCGCCA	0.14283243	0.546561	0.069847	0.111027
tsrna-06806	ATCCCACCAGAGTCGCC	0.07141622	0.23424	0	0
tsrna-06802	ATCCCACATGGTCTAGCGGTTAGGATTCCT	0.71416216	0.702721	0.349234	1.110271
tsrna-06801	ATCCCACATGGTCTAGCGGTTAGGATT	0.07141622	0.936961	0.838162	1.221299
tsrna-06800	ATCCCACATGGTCTAGCGGT	0.21424865	0.23424	0.069847	0.222054
tsrna-06799	ATCCATTGTGCTTTGCACGCGTGGGTTCG	0.07141622	0.31232	0	0
tsrna-06798	ATCCATTGGTCTTAGGCCCCAAAAATTTTGGTGCAACTCCAA	1.85682163	4.294406	2.235098	0.222054
tsrna-06797	ATCCATTGGTCTTAGGCCCCAAAAATTTTGGTGCAACTCC	0.21424865	0.15616	1.53663	0
tsrna-06796	ATCCATTGGTCTTAGGCCCCAAAAATTTTGGTGCAA	0.07141622	0.15616	0.279387	0
tsrna-06795	ATCCATTGGTCTTAGGCCCCAA	0	0	0.279387	0
tsrna-06794	ATCCATTGGTCTTAGGCCCCA	0	0	0.139694	0
tsrna-06792	ATCCATTGGTCTTAGGCCC	0	0	0.069847	0
tsrna-06788	ATCCAGGTGCCCCTTCCA	0	0.15616	0.069847	0
tsrna-06787	ATCCAGGTGCCCCCTCCA	0	0.23424	0	0.444109
tsrna-06784	ATCCAGCGATCCGAGTTCAAATC	0.07141622	0	0	0
tsrna-06776	ATCATTGGTCGTGGTTGTAGTCCGTGCGAGAATACCA	8.7127784	10.22849	10.82625	4.330058
tsrna-06775	ATCATTGGTCGTGGTTGTAGTCCGTGCGAGAATACC	1.49974054	2.576644	1.466783	1.55438
tsrna-06774	ATCATTGGTCGTGGTTGTAGTCCGTGCGAGAATA	1.07124325	0.546561	0.768315	0.666163
tsrna-06773	ATCATTGGTCGTGGTTGTAGTCCGTGCGAGAAT	0.78557838	0.858881	0.349234	1.332326
tsrna-06772	ATCATTGGTCGTGGTTGTAGTCCGTGCGAGAA	0.71416216	1.405442	0.838162	0.888217
tsrna-06771	ATCATTGGTCGTGGTTGTAGTCCGTGCGAGA	0.35708108	0.624641	0.558774	0.666163
tsrna-06770	ATCATTGGTCGTGGTTGTAGTCCGTGCGAG	0.28566487	0.936961	0.20954	0.666163
tsrna-06769	ATCATTGGTCGTGGTTGTAGTCCGTGCGA	0.14283243	0.624641	0.558774	0.333081
tsrna-06768	ATCATTGGTCGTGGTTGTAGTCCGTGCG	0.21424865	0.23424	0.139694	0.444109
tsrna-06767	ATCATTGGTCGTGGTTGTAGTCCGTGC	0.21424865	0.31232	0.139694	0.111027
tsrna-06766	ATCATTGGTCGTGGTTGTAGTCCGTG	0.07141622	0.468481	0.069847	0
tsrna-06765	ATCATTGGTCGTGGTTGTAGTCCGT	0	0.07808	0.069847	0
tsrna-06764	ATCATTGGTCGTGGTTGTAGTCCG	0	0.15616	0.139694	0
tsrna-06763	ATCATTGGTCGTGGTTGTAGTCC	0	0	0	0
tsrna-06762	ATCATTGGTCGTGGTTGTAGTC	0	0	0	0
tsrna-06761	ATCATTGGTCGTGGTTGTAGT	0	0.15616	0.069847	0
tsrna-06760	ATCATTGGTCGTGGTTGTAG	0	0	0	0

tsrna-06758	ATCATTGGTCGTGGTTGT	0	0	0	0
tsrna-06755	ATCATGCAAGATTCCCATTCTT	0	0	0	0.111027
tsrna-06751	ATCAGATTGTGAATCTGACAACAG	0.07141622	0	0.139694	0
tsrna-06750	ATCAGATTGTGAATCTGACAACA	0	0.07808	0.139694	0
tsrna-06749	ATCAGATTGTGAATCTGACA	0	0	0.139694	0
tsrna-06747	ATCAGATTGTGAATCTGA	0	0	0	0
tsrna-06744	ATCAGACTTTTAATCTGAGGGTCCAG	0.78557838	2.576644	0.279387	0.555136
tsrna-06743	ATCAGAAGGTTGCGTGTTCA	0.07141622	0.31232	0.069847	0
tsrna-06742	ATCAGAAGGTTGCGTGTTC	0.14283243	0.15616	0.139694	0
tsrna-06741	ATCAGAAGGTTGCGTGTT	0.07141622	0.31232	0	0.111027
tsrna-06740	ATCAGAAGGCTGCGTGTTCGAA	0	0.15616	0.069847	0
tsrna-06739	ATCAGAAGGCTGCGTGTTC	0	0.07808	0	0
tsrna-06736	ATCAGAAGATTGAGGGTTCGAGTCCCTTCGTGGTCGCCA	0.14283243	1.171202	0.349234	0.444109
tsrna-06735	ATCAGAAGATTGAGGGTTCGAGTCCCTTCGTGGTCGCC	0.35708108	0.780801	0.20954	0.111027
tsrna-06734	ATCAGAAGATTGAGGGTTCGAGTCCCTTCGTGGTCGC	0.57132973	1.015041	0	0.111027
tsrna-06733	ATCAGAAGATTCTAGGTTCGACTCCTGGCTGGCTCGCC	0.92841081	1.327362	0.768315	0.333081
tsrna-06732	ATCAGAAGATTCTAGGTTCGACTCCTGGCTGGCTC	0.35708108	0.702721	0.069847	0
tsrna-06731	ATCAGAAGATTCCAGGTTCGACTCCTGGCTGGCTCGCC	0.49991351	0.15616	0.20954	0.111027
tsrna-06730	ATCAGAAGATTCCAGGTTCGACTCCTGGCT	0	0.15616	0	0
tsrna-06729	ATCACGTTCGCCTCACACGCGA	0.07141622	0.07808	0	0
tsrna-06728	ATCACGTTCGCCTCACACGCG	0	0	0	0
tsrna-06723	ATCACGTTCGCCTCAC	0	0	0	0
tsrna-06719	ATCACGTCTGCTTTACAC	0.07141622	0	0	0
tsrna-06716	ATCACGTCGGGGTCACCA	0.21424865	0.624641	0.20954	0.444109
tsrna-06715	ATCACGTCGGGGTCACC	0.07141622	0	0.069847	0
tsrna-06713	ATCACCCCATAAACACCA	0.07141622	0.07808	0	0
tsrna-06710	ATCAAGAGGTCCCCGGTTC	0.07141622	0.23424	0	0
tsrna-06709	ATCAAGAGGTCCCCGGTT	0	0.31232	0	0.111027
tsrna-06708	ATCAAGAGGTCCCCGGT	0.07141622	0.23424	0.139694	0
tsrna-06706	ATATTGAATTGCAAATTCGAAGAAGCAGCTTCAAACCTGCCG(	0.07141622	0.07808	0.069847	0
tsrna-06705	ATATTGAATTGCAAATTCGAAGAAGCAGCTTCAAACCTGCCG(	0.07141622	0.15616	0	0
tsrna-06702	ATATGTCCGCGTGGGTT	0.28566487	0.390401	0	0
tsrna-06701	ATATGTCCGCGTGGGT	0.07141622	0.15616	0.069847	0
tsrna-06700	ATATGGTCTAGCGGTTAGGATTCCTGGTTT	0.35708108	0.23424	0.419081	0.333081
tsrna-06699	ATATGGTCTAGCGGTTAGGATTCCTGGTT	0.35708108	0.468481	0.20954	0.222054
tsrna-06698	ATATGGTCTAGCGGTTAGGATTCCTGGT	0.4284973	0.390401	0.349234	0.333081
tsrna-06697	ATATGGTCTAGCGGTTAGGATTCCTGG	0.21424865	0.31232	0	0
tsrna-06696	ATATGGTCTAGCGGTTAGGATTCCTG	0.21424865	0	0.139694	0
tsrna-06695	ATATGGTCTAGCGGTTAGGATTCCT	0	0	0	0.111027
tsrna-06694	ATATGGTCTAGCGGTTAGGATTCC	0	0	0	0
tsrna-06693	ATATGGTCTAGCGGTTAGGATTC	0	0	0	0
tsrna-06689	ATATCATTGGTCGTGGTTGTAGTCCGTGCGAGAATACCA	10.7124325	10.30657	13.62013	5.662384
tsrna-06688	ATATCATTGGTCGTGGTTGTAGTCCGTGCGAGAATACC	1.57115676	2.576644	1.327089	1.55438
tsrna-06687	ATATCATTGGTCGTGGTTGTAGTCCGTGCGAGAATA	0.64274595	1.639682	0.628621	0.999244
tsrna-06686	ATATCATTGGTCGTGGTTGTAGTCCGTGCGAGAAT	0.8569946	1.015041	0.768315	0.222054
tsrna-06685	ATATCATTGGTCGTGGTTGTAGTCCGTGCGAGAA	0.49991351	0.780801	0.698468	0.555136
tsrna-06684	ATATCATTGGTCGTGGTTGTAGTCCGTGCGAGA	0.4284973	1.717762	0.488928	1.221299
tsrna-06683	ATATCATTGGTCGTGGTTGTAGTCCGTGCGAG	0.28566487	0.858881	0.139694	0.444109
tsrna-06682	ATATCATTGGTCGTGGTTGTAGTCCGTGCGA	0.35708108	0.31232	0.628621	0.888217
tsrna-06681	ATATCATTGGTCGTGGTTGTAGTCCGTGCG	0.21424865	0.31232	0	0.555136
tsrna-06680	ATATCATTGGTCGTGGTTGTAGTCCGTGC	0.14283243	0.23424	0	0
tsrna-06679	ATATCATTGGTCGTGGTTGTAGTCCGTG	0.35708108	0.23424	0.139694	0
tsrna-06678	ATATCATTGGTCGTGGTTGTAGTCCGT	0.14283243	0.07808	0.069847	0
tsrna-06677	ATATCATTGGTCGTGGTTGTAGTCCG	0	0.15616	0	0.111027
tsrna-06676	ATATCATTGGTCGTGGTTGTAGTCC	0.21424865	0.07808	0	0.111027
tsrna-06675	ATATCATTGGTCGTGGTTGTAGTC	0.07141622	0	0.069847	0
tsrna-06674	ATATCATTGGTCGTGGTTGTAGT	0	0	0	0
tsrna-06673	ATATCATTGGTCGTGGTTGTAG	0	0	0	0
tsrna-06663	ATAGTGTAGTGGTTATCACGTCTGCTTT	0	0	0.069847	0
tsrna-06659	ATAGTGTAGTGGTTATC	0.07141622	0	0.069847	0
tsrna-06658	ATAGTGGTTAGTATCCCCGCCTGT	0	0.07808	0.069847	0.111027
tsrna-06657	ATAGTGGTTAGTATCCCCGCCT	0.07141622	0.07808	0.069847	0
tsrna-06656	ATAGTGGTTAGTACTCTGCGTTGTGGCCG	1.49974054	1.015041	1.047702	0.888217
tsrna-06655	ATAGTGGTTAGTACTCTGCGTTGTGGCC	1.71398919	1.093122	0.838162	0.999244

tsrna-06654	ATAGTGGTTAGTACTCTGCGTTGTGGC	1.57115676	1.015041	0.349234	0.77719
tsrna-06653	ATAGTGGTTAGTACTCTGCGTTGTGG	1.57115676	0.858881	0.419081	0.77719
tsrna-06652	ATAGTGGTTAGTACTCTGCGTTGTG	1.78540541	0.390401	0.698468	1.332326
tsrna-06651	ATAGTGGTTAGTACTCTGCGTTGT	1.71398919	0.702721	0.768315	0.999244
tsrna-06650	ATAGTGGTTAGTACTCTGCGTTG	1.07124325	0.546561	0.908008	0.77719
tsrna-06649	ATAGTGGTTAGTACTCTGCGTT	1.42832433	0.702721	0.419081	0.555136
tsrna-06648	ATAGTGGTTAGTACTCTGCGT	0.49991351	0.07808	0.20954	0.111027
tsrna-06647	ATAGTGGTTAGTACTCTGCGC	0.28566487	0.23424	0.069847	0.111027
tsrna-06646	ATAGTGGTTAGTACTCTGCG	0.14283243	0.07808	0.069847	0.111027
tsrna-06645	ATAGTGGTTAGTACTCTGC	0.07141622	0.23424	0	0
tsrna-06641	ATAGTGGTGAGTATCCCCGCCTGTC	0.49991351	0.546561	0.069847	0.222054
tsrna-06640	ATAGTGGTGAGTATCCCCGCCTGT	0.14283243	0.23424	0	0.111027
tsrna-06639	ATAGTGGTGAGTATCCCCGCCTG	0.07141622	0.390401	0	0
tsrna-06638	ATAGTGGTGAGTATCCCCGCCT	0.14283243	0.390401	0.069847	0
tsrna-06637	ATAGTGGTGAGTATCCCCGCC	0	0.624641	0.069847	0
tsrna-06636	ATAGTGGTGAGTATCCCCGC	0.21424865	0.31232	0	0
tsrna-06635	ATAGTGGTGAGTATCCCCG	0.07141622	0.23424	0	0
tsrna-06633	ATAGTGGTGAGCATAGCTGCC	0.07141622	0	0	0
tsrna-06631	ATAGTACCGTTAACTTCCAATTAACTAGTT	0.14283243	0	0.20954	0
tsrna-06630	ATAGTACCGTTAACTTCCAATTA	0	0	0.069847	0
tsrna-06629	ATAGTACCGTTAACTTCCA	0.07141622	0	0	0
tsrna-06626	ATAGGTGGTTCAGTGGTAGAATTCT	0.14283243	0.07808	0.069847	0
tsrna-06625	ATAGGTGGTTCAGTGGTAGAATTC	0.07141622	0.31232	0.139694	0.111027
tsrna-06624	ATAGGTGGTTCAGTGGTAGAA	0	0.15616	0.069847	0
tsrna-06623	ATAGGTGGTTCAGTGGTAGA	0	0.07808	0.069847	0
tsrna-06621	ATAGGTGGCACGGAGAATTTTGGATT	0.14283243	0.07808	0	0
tsrna-06620	ATAGGTGGCACGGAGAATTTTGGAT	0	0.07808	0.139694	0
tsrna-06619	ATAGGTGGCACGGAGAATTTTGGA	0.07141622	0.15616	0.139694	0
tsrna-06618	ATAGGTGGCACGGAGAATTTTGG	0	0.15616	0	0
tsrna-06617	ATAGGTGGCACGGAGAATTTTG	0	0.15616	0.139694	0
tsrna-06616	ATAGGTGGCACGGAGAATTTT	0.07141622	0	0	0
tsrna-06614	ATAGGTGGCACGGAGAATT	0	0	0	0
tsrna-06613	ATAGGTGGCACGGAGAAT	0.07141622	0	0	0
tsrna-06611	ATAGGTGGCACGGAGA	0	0	0	0
tsrna-06610	ATAGGAGCTTAAACCCCCTTATTTCTACC	0.28566487	0.15616	0.069847	0
tsrna-06605	ATAGCTTAGGGGTAGAGCATTTGACTGC	0.14283243	0	0	0
tsrna-06604	ATAGCTTAGCGGTAGAGCATTTGACTGC	0	0	0	0.555136
tsrna-06603	ATAGCTTAGCGGTAGAGCATTTGACTG	0.07141622	0	0	0.111027
tsrna-06601	ATAGCTGCCTTCCAAGCAGTTGACCCGG	0.07141622	0.31232	0	0
tsrna-06600	ATAGCTGCCTTCCAAGCAGTTGACCCG	0	0.15616	0.069847	0.111027
tsrna-06598	ATAGCTCAGTTGGTAGAGCGGAGGA	0	0.07808	0	0
tsrna-06597	ATAGCTCAGTTGGTAGAACATCAGA	0.07141622	0	0	0
tsrna-06593	ATAGCTCAGTGGTAGAGCATTTGACTGCAG	0.21424865	0.23424	0.139694	0
tsrna-06592	ATAGCTCAGTGGTAGAGCATTTGACTGCA	0.28566487	0	0	0
tsrna-06591	ATAGCTCAGTGGTAGAGCATTTGACTGC	0	0.15616	0.139694	0.222054
tsrna-06590	ATAGCTCAGTGGTAGAGCATTTGACTG	0.28566487	0.07808	0.069847	0
tsrna-06589	ATAGCTCAGTGGTAGAGCATTTGACTAC	0.14283243	0.15616	0.069847	0.111027
tsrna-06588	ATAGCTCAGTGGTAGAGCATTTGACTA	0.35708108	0.15616	0.069847	0
tsrna-06587	ATAGCTCAGTGGTAGAGCATTTGACT	0.35708108	0	0	0
tsrna-06586	ATAGCTCAGTGGTAGAGCATTTGAC	0.14283243	0.07808	0	0
tsrna-06585	ATAGCTCAGTGGTAGAGCATTTGA	0.07141622	0	0	0
tsrna-06584	ATAGCTCAGTGGTAGAGCATTTG	0	0.07808	0	0
tsrna-06583	ATAGCTCAGTGGTAGAGCATTT	0.14283243	0.07808	0	0
tsrna-06582	ATAGCTCAGTGGTAGAGCATT	0.07141622	0.15616	0	0
tsrna-06580	ATAGCTCAGTGGGTAGAGCATTTGACTGC	0	0.07808	0.069847	0
tsrna-06579	ATAGCTCAGTGGGTAGAGCATTTGACTG	0.07141622	0.15616	0.069847	0.111027
tsrna-06578	ATAGCTCAGTGGGTAGAGCATTTGACT	0	0.15616	0	0
tsrna-06577	ATAGCTCAGTGGGTAGAGCATTTGAC	0.07141622	0.15616	0	0
tsrna-06575	ATAGCTCAGTCGGTAGAGCATCAGACTTTT	1.42832433	0.858881	0.908008	1.221299
tsrna-06574	ATAGCTCAGTCGGTAGAGCATCAGACTTT	1.21407568	0.468481	0.279387	0.444109
tsrna-06573	ATAGCTCAGTCGGTAGAGCATCAGACT	1.21407568	0.936961	0.279387	0.111027
tsrna-06572	ATAGCTCAGTCGGTAGAGCATCAGAC	0.78557838	0.546561	0.908008	0
tsrna-06571	ATAGCTCAGTCGGTAGAGCATCAGA	0.57132973	0.546561	0.419081	0.111027
tsrna-06570	ATAGCTCAGTCGGTAGAGCATCAG	0.35708108	0.31232	0.279387	0

tsrna-06569	ATAGCTCAGTCGGTAGAGCATC	0.07141622	0.15616	0.069847	0.111027
tsrna-06564	ATAGCTCAGGTGGTAGAGCATTTGACTGC	0.14283243	0	0	0
tsrna-06563	ATAGCTCAGGTGGTAGAGCATTTGACTG	0	0.07808	0	0
tsrna-06562	ATAGCTCAGGTGGTAGAGCATTTGACT	0	0	0	0
tsrna-06559	ATAGCTCAGGGGTAGAGCATTTGACTGC	0.14283243	0.15616	0.069847	0.888217
tsrna-06558	ATAGCTCAGGGGTAGAGCATTTGACTG	0.07141622	0	0	0.222054
tsrna-06557	ATAGCTCAGGGGTAGAGCATTTGACT	0.07141622	0.07808	0	0
tsrna-06556	ATAGCTCAGGGGTAGAGCATTTGAC	0	0	0	0.111027
tsrna-06555	ATAGCTCAGGGGTAGAGCATTTGA	0.07141622	0	0	0
tsrna-06554	ATAGCTCAGGGGTAGAGCATTTG	0	0.07808	0	0
tsrna-06553	ATAGAGTAAATAATAGGAGCTTAAACCCCCTTATTTCTACC	0.4284973	0.15616	0.419081	0
tsrna-06552	ATAGAGTAAATAATAGGAGCTTAAACCCCCTTATTTCT	0.28566487	0.15616	0.488928	0
tsrna-06551	ATAGAGTAAATAATAGGAGCTTAAACCCCC	0.14283243	0	0	0
tsrna-06550	ATAGAGTAAATAATAGGAGCTTAAACCC	0	0	0	0
tsrna-06549	ATAGAGTAAATAATAGGAGCTT	0.14283243	0	0	0
tsrna-06548	ATAGAGTAAATAATAGGAGCT	0	0	0	0
tsrna-06544	ATACTTAATTTCTGCCA	0.07141622	0	0	0
tsrna-06543	ATACTTAATTTCTGCC	0	0	0	0
tsrna-06542	ATACCCTTCCCGTACTACCA	0.07141622	0.07808	0	0
tsrna-06541	ATACCCTTCCCGTACTACC	0.14283243	0	0	0
tsrna-06538	ATACCCCGAAAATGTTGGTTATACCCTTCC	0.07141622	0	0	0
tsrna-06535	ATACAGACCAAGAGCCTTCAAAG	0.49991351	0.390401	0.20954	0.111027
tsrna-06533	ATACAGACCAAGAGCCTTC	0.07141622	0	0	0
tsrna-06531	ATACACTGAAAATGTTTAGACGGGCTC	0.57132973	0.390401	0.139694	0.222054
tsrna-06530	ATACACTGAAAATGTTTAGACGGGCT	0.14283243	0.15616	0	0.111027
tsrna-06526	ATACACCAGTCTTGTAAACCGG	0.14283243	0.31232	0.139694	0
tsrna-06525	ATACACCAGTCTTGTAAACCG	0.07141622	0.23424	0.139694	0
tsrna-06524	ATACACCAGTCTTGTAAACC	0	0.07808	0	0
tsrna-06523	ATACAACGATGGTTTTTCATATCATTGGTCGTGGTTGTAGTCC	0	0.23424	0.349234	0
tsrna-06522	ATACAACGATGGTTTTTCATATCATTGGTCGTGGTTGTAGTC	0.21424865	0	0.279387	0.111027
tsrna-06521	ATACAACGATGGTTTTTCATATCATTGGTCGTGGTTGTAGT	0.21424865	0.23424	0.349234	0
tsrna-06520	ATACAACGATGGTTTTTCATATCATTGGTCGTGGTTGTA	0.07141622	0.07808	0.349234	0
tsrna-06519	ATACAACGATGGTTTTTCATATCATTGGTCGTGGTTGT	0	0.07808	0	0
tsrna-06518	ATACAACGATGGTTTTTCATATCATTGGTC	0	0.07808	0	0
tsrna-06512	ATAATGCCGAGGTTGTGAGTTCGA	0.28566487	1.639682	0.069847	0.222054
tsrna-06511	ATAATGCCGAGGTTGTGAGTTCG	0.21424865	1.873923	0.069847	0.333081
tsrna-06510	ATAATGCCGAGGTTGTGAGTTCA	0.35708108	1.561602	0	0
tsrna-06509	ATAATGCCGAGGTTGTGAGTTC	0.14283243	0.546561	0	0
tsrna-06508	ATAATGCCGAGGTTGTGAGTT	0.07141622	0.702721	0.139694	0.111027
tsrna-06507	ATAATGCCGAGGTTGTGAGT	0.14283243	0.780801	0.069847	0
tsrna-06506	ATAATGCCGAGGTTGTGAG	0.07141622	0.23424	0.069847	0
tsrna-06505	ATAATGCCGAGGTTGTGA	0	0.07808	0.069847	0
tsrna-06504	ATAATGCCGAGGTTGTG	0.07141622	0.07808	0	0
tsrna-06502	ATAATCTGAAGGTCGTGAGTTCGATCCTCACACGGGGCACCA	0.28566487	3.825925	0.349234	0.222054
tsrna-06501	ATAATCTGAAGGTCGTGAGTTCGAGCCTCACACGGGGCACCA	0.8569946	1.639682	0.768315	0.222054
tsrna-06500	ATAATCTGAAGGTCGTGAGTTCGAGCCTCACACGGG	0.78557838	1.639682	0.488928	0.333081
tsrna-06499	ATAATCTGAAGGTCGTGAGTTCGAGCCTCA	0.49991351	1.873923	0.558774	0.222054
tsrna-06498	ATAATCTGAAGGTCGTGAGTTCGAGCCTC	0.28566487	1.873923	0.349234	0.333081
tsrna-06497	ATAATCTGAAGGTCGTGAGTTCG	0.4284973	0.936961	0.768315	0.555136
tsrna-06496	ATAATCTGAAGGTCGTGAGTTC	0.71416216	0.468481	0.279387	0.222054
tsrna-06495	ATAATCTGAAGGTCGTGAGTT	0.4284973	1.093122	0.698468	0.333081
tsrna-06494	ATAATCTGAAGGTCGTGAGT	0.64274595	0.858881	0.349234	0.444109
tsrna-06493	ATAATCTGAAGGTCGTGAG	0.07141622	0.07808	0	0.111027
tsrna-06492	ATAATCTGAAGGTCGTG	0	0.23424	0.139694	0.111027
tsrna-06491	ATAATCTGAAGGTCCTGAGTTCG	4.85630272	13.3517	4.260655	2.220543
tsrna-06490	ATAATCTGAAGGTCCTGAGTT	4.28497299	11.63394	3.003413	2.553624
tsrna-06489	ATAATCTGAAGGTCCTGAGT	4.21355677	12.41474	2.863719	3.552868
tsrna-06488	ATAATCTGAAGGTCCTGAG	0.14283243	0.936961	0.279387	0.222054
tsrna-06487	ATAATCTGAAGGTCCTG	0.21424865	0.390401	0.069847	0
tsrna-06481	ATAATAGGAGCTTAAACCCCCTTATTTCT	0	0.23424	0	0.111027
tsrna-06476	ATAATAGGAGCTTAAACCCC	0	0	0	0
tsrna-06475	ATAATAGGAGCTTAAACCC	0	0	0.069847	0
tsrna-06470	ATAAGGCGTCTGACTTCGG	0	0.07808	0	0
tsrna-06469	ATAAGGCGTCTGACTTCG	0	0	0	0

tsrna-06468	ATAAGCTATCGGGCCCATACC	0	0	0.069847	0
tsrna-06467	ATAAGCTATCGGGCCCATA	0	0	0	0
tsrna-06466	ATAAGCTATCGGGCCCA	0	0.07808	0	0
tsrna-06465	ATAAGCTATCGGGCCC	0	0.07808	0	0
tsrna-06464	ATAACTTTGTCAAAGTTAAATTATAGGCT	0.4284973	0.15616	0.558774	0.222054
tsrna-06461	ATAACGCCAAGGTCGCGGGTTCGATCCCCGTACGGGCCACC	0.4284973	1.405442	0.139694	0
tsrna-06460	ATAACGCCAAGGTCGCGGGTTCGA	0.28566487	1.093122	0.349234	0.222054
tsrna-06459	ATAACGCCAAGGTCGCGGGTTCG	0.49991351	0.624641	0.069847	0.222054
tsrna-06458	ATAACGCCAAGGTCGCGGGTT	0.35708108	0.468481	0	0.111027
tsrna-06457	ATAACGCCAAGGTCGCGGGT	0.07141622	0.624641	0.279387	0
tsrna-06455	ATAACGCCAAGGTCGCGG	0.07141622	0	0	0
tsrna-06454	ATAACCCAGAGGTCGATGGATCTA	0.64274595	2.654724	0.488928	1.332326
tsrna-06453	ATAACCCAGAGGTCGATGGATCT	0.92841081	2.264323	0.558774	0.666163
tsrna-06452	ATAACCCAGAGGTCGATGGATCGAAACCA	0	0.390401	0	0.222054
tsrna-06451	ATAACCCAGAGGTCGATGGATCGAAACC	0	0.780801	0	0.222054
tsrna-06450	ATAACCCAGAGGTCGATGGATCGAAAC	0.28566487	0.468481	0.069847	0
tsrna-06449	ATAACCCAGAGGTCGATGGATCGAAA	0	0.31232	0.139694	0
tsrna-06448	ATAACCCAGAGGTCGATGGATCGAA	0.14283243	0.780801	0	0.111027
tsrna-06447	ATAACCCAGAGGTCGATGGATCGA	0.35708108	0.15616	0.069847	0.111027
tsrna-06446	ATAACCCAGAGGTCGATGGATCG	0.07141622	0.23424	0.069847	0.111027
tsrna-06445	ATAACCCAGAGGTCGATGGATC	0.07141622	0.31232	0.139694	0.222054
tsrna-06444	ATAACCCAGAGGTCGATGGAT	0.07141622	0.468481	0.139694	0.111027
tsrna-06443	ATAACCCAGAGGTCGATGGA	0.14283243	0.31232	0	0.111027
tsrna-06442	ATAACCCAGAGGTCGATGG	0	0.15616	0	0
tsrna-06441	ATAACCCAGAGGTCGATG	0.14283243	0.23424	0	0
tsrna-06440	ATAACCCAGAGGTCGAT	0.14283243	0.07808	0.069847	0
tsrna-06438	ATAACAGCTATCCATTGGTCTTAGGCCCC	0	0	0	0
tsrna-06437	ATAACAGCTATCCATTGGTCTTAGGCCC	0	0	0.069847	0
tsrna-06436	ATAACAGCTATCCATTGGTCTTAGGCC	0.07141622	0	0.069847	0
tsrna-06435	ATAACAGCTATCCATTGGTCTTAGGC	0	0	0.069847	0
tsrna-06434	ATAACAGCTATCCATTGGTCTTAGG	0	0	0.069847	0
tsrna-06433	ATAACAGCTATCCATTGGTCTTAG	0	0	0	0
tsrna-06432	ATAACAGCTATCCATTGGTCTTA	0.07141622	0.07808	0	0
tsrna-06431	ATAACAGCTATCCATTGGTCTT	0	0	0	0
tsrna-06424	ATAACACCAAGGTCGCGGGCTCG	0	0.07808	0	0
tsrna-06423	ATAACACCAAGGTCGCGGGCT	0	0.15616	0.069847	0
tsrna-06422	ATAACACCAAGGTCGCGGGC	0	0.15616	0.069847	0
tsrna-06420	ATAAATAGTACCGTTAACTTCC	0.14283243	0.07808	0.139694	0
tsrna-06417	AGTTTTGACAACATTCAAAAAAGAGTACC	0	0	0.069847	0
tsrna-06415	AGTTTTGACAACATTCAAAAAAGAGT	0	0.07808	0	0
tsrna-06414	AGTTTCTGTGGTGTAG	0.07141622	0	0	0
tsrna-06413	AGTTTCCGTAGTGTAGTGGTTATCACGTTC	7.92720002	3.279365	5.378204	8.771144
tsrna-06412	AGTTTCCGTAGTGTAGTGGTTATC	7.64153516	2.888964	4.120962	6.883683
tsrna-06411	AGTTTCCGTAGTGTAGTGGTTA	1.35690811	1.795843	0.419081	0.888217
tsrna-06410	AGTTTCCGTAGTGTAGTGGTT	0.64274595	1.327362	0.419081	0.77719
tsrna-06409	AGTTTCCGTAGTGTAGTGGTC	1.14265946	1.405442	0.349234	0.555136
tsrna-06408	AGTTTCCGTAGTGTAGTGG	0.99982703	1.795843	0.20954	0.222054
tsrna-06407	AGTTTCCGTAGTGTAG	0.4284973	1.249282	0.20954	0.333081
tsrna-06406	AGTTTATGTAGCTTACCTCCTCA	0.49991351	0.23424	0.349234	0.77719
tsrna-06405	AGTTTATGTAGCTTACCTCCTC	0.28566487	0	0.139694	0.888217
tsrna-06404	AGTTTATGTAGCTTACCTCCT	0.64274595	0	0.349234	1.110271
tsrna-06403	AGTTTATGTAGCTTACCTCC	0.14283243	0	0.20954	0.333081
tsrna-06402	AGTTTATGTAGCTTACCTC	0.14283243	0	0.20954	0.333081
tsrna-06401	AGTTTAACCAAAACATCAGATTGTGAATCTGACAACAGAGGCT	0.14283243	0.468481	0.20954	0
tsrna-06396	AGTTTAAATTAGAATCTTAGCTTTG	0.4284973	0.23424	0.139694	0
tsrna-06395	AGTTGGTTAGAGCGTGGT	0	0	0	0
tsrna-06386	AGTTGGTAGAGCATCAGAC	0	0.07808	0	0
tsrna-06385	AGTTGGTAGAGCATCAGA	0.07141622	0.07808	0	0
tsrna-06381	AGTTGGGAGAGCGTTAGACTGAAGAT	0.07141622	0	0	0
tsrna-06379	AGTTGGGAGAGCGTTAGACTGAAG	0	0.07808	0	0
tsrna-06375	AGTTGCAATACTTAATTTCTGCCA	0	0	0.139694	0.111027
tsrna-06374	AGTTGCAATACTTAATTTCTGCC	0.07141622	0	0	0
tsrna-06369	AGTTGATTAGGGTGCTTAGCTGTTAACTAAGTGTTTGTGGGT	0.8569946	0.702721	1.327089	0.222054
tsrna-06368	AGTTGATTAGGGTGCTTAGCTGTTAACTAAGTGTTT	0.64274595	0.468481	0.488928	0.222054

tsrna-06367	AGTTGATTAGGGTGCTTAGCTGTTAACTAA	0.49991351	0.390401	0.977855	0.333081
tsrna-06366	AGTTGATTAGGGTGCTTAGCTGTTAACTA	0.35708108	0.390401	0.628621	0.111027
tsrna-06365	AGTTGATTAGGGTGCTTAGCTGTTAACT	0.07141622	0.23424	0.419081	0.111027
tsrna-06364	AGTTGATTAGGGTGCTTAGCTGTTAAC	0.07141622	0.15616	0.20954	0.111027
tsrna-06363	AGTTGATTAGGGTGCTTAGCTGTTA	0	0.15616	0	0
tsrna-06362	AGTTGATTAGGGTGCTTAGCTGTT	0	0.07808	0.139694	0.111027
tsrna-06361	AGTTGATTAGGGTGCTTAGCTGT	0	0	0.139694	0
tsrna-06360	AGTTGATTAGGGTGCTTAGCTG	0.07141622	0	0	0
tsrna-06359	AGTTGATTAGGGTGCTTAGCT	0	0	0	0
tsrna-06358	AGTTGATTAGGGTGCTTAGC	0.07141622	0	0	0
tsrna-06353	AGTTGATGCAGAGTGGGGTTTTGCAGTCCT	0.14283243	0.07808	0	0
tsrna-06352	AGTTGATGCAGAGTGGGGTTTTGCAGTCC	0	0.15616	0.069847	0
tsrna-06351	AGTTGATGCAGAGTGGGGTTTTGCAGTC	0	0	0.20954	0
tsrna-06350	AGTTGATGCAGAGTGGGGTTTTGCA	0	0	0	0.111027
tsrna-06349	AGTTGATGCAGAGTGGGGTTTTGC	0	0.07808	0.069847	0
tsrna-06348	AGTTGATGCAGAGTGGGGTTTTG	0	0.23424	0	0
tsrna-06347	AGTTGATGCAGAGTGGGGTTTT	0	0	0	0
tsrna-06346	AGTTGATGCAGAGTGGGGTTT	0	0.07808	0	0
tsrna-06345	AGTTGATGCAGAGTGGGGTT	0	0	0	0
tsrna-06336	AGTTGAAATACAACGATGGTTTTTCAT	0.07141622	0	0	0
tsrna-06335	AGTTCTTGTAGTTGAAATACAACG	0.28566487	0.546561	0.139694	0.444109
tsrna-06334	AGTTCTGGTCTCCGGATGGAGGCGT	0.14283243	0.31232	0.069847	0
tsrna-06333	AGTTCTGGTCTCCGGATGGAGGCG	0	0.15616	0	0
tsrna-06331	AGTTCTGGTCTCCGGATGGAGG	0	0.07808	0	0
tsrna-06325	AGTTCGATCCTCGCTGGGGCCTCCA	0.35708108	1.015041	0.349234	0.333081
tsrna-06324	AGTTCGATCCTCGCTGGGGCCTC	0	0	0	0
tsrna-06323	AGTTCGATCCTCGCTGGGGCCT	0	0	0	0
tsrna-06320	AGTTCGATCCTCACCTGGAGCACC	0.07141622	0	0	0
tsrna-06319	AGTTCGATCCTCACCTGGAGCA	0	0.07808	0	0
tsrna-06316	AGTTCGATCCTCACACGGGGCACCA	0	0.23424	0.069847	0
tsrna-06311	AGTTCGAGTCTCGGTGGAACCTCCA	4.42780542	11.63394	1.816017	2.220543
tsrna-06310	AGTTCGAGTCTCGGTGGAACC	0	0	0	0
tsrna-06309	AGTTCGAGTCTCGGTGGAAC	0	0.07808	0	0
tsrna-06307	AGTTCGAGCCTCACCTGGAGCACCA	0	0.07808	0	0
tsrna-06306	AGTTCGAGCCTCACCTGGAGCACC	0	0.07808	0	0
tsrna-06296	AGTTCGAATCTCGGTGGGACCTC	0	0	0	0
tsrna-06294	AGTTCGAACCTCAGAGGGGGCACCA	0.14283243	0.546561	0	0
tsrna-06290	AGTTCAATTCTCGCTGGGGCCTC	0.07141622	0	0	0
tsrna-06289	AGTTCAAGTCTCGGTGGAACCTCCA	4.42780542	7.964171	1.257243	1.332326
tsrna-06288	AGTTCAAGTCTCGGTGGAACCTC	0.07141622	0.23424	0	0
tsrna-06285	AGTTCAAATCTCGGTGGGACCTCCA	5.07055137	9.135373	1.955711	2.33157
tsrna-06284	AGTTCAAATCTCGGTGGGACCTC	0	0.07808	0	0
tsrna-06282	AGTTCAAATCTCGGTGGGACC	0	0	0	0
tsrna-06281	AGTTCAAATCTCGGTGGGAC	0	0.07808	0	0
tsrna-06280	AGTTCAAATCTCGGTGGGA	0	0	0.069847	0
tsrna-06278	AGTTCAAATCTCGGTGGAACCTCCA	11.2837622	13.66402	4.120962	3.441841
tsrna-06277	AGTTCAAATCTCGGTGGAACCTCC	0.71416216	1.015041	0.349234	0.222054
tsrna-06276	AGTTCAAATCTCGGTGGAACCTC	0	0.31232	0.069847	0
tsrna-06275	AGTTCAAATCTCGGTGGAACCT	0	0	0	0
tsrna-06274	AGTTCAAATCTCGGTGGAACC	0.07141622	0.15616	0	0
tsrna-06268	AGTTCAAATCTCGCTGGGGCCTCCA	3.07089731	4.294406	1.257243	2.109516
tsrna-06267	AGTTCAAATCTCGCTGGGGCCTCC	0.35708108	1.093122	0.069847	0.111027
tsrna-06266	AGTTCAAATCTCGCTGGGGCCTC	0	0	0	0
tsrna-06262	AGTTCAAATCTCGCTGGG	0	0	0	0
tsrna-06261	AGCGGTTAGGATTCCTGGTTTTCAC	0.64274595	0.31232	0.488928	0.333081
tsrna-06260	AGCGGTTAGGATTCCTGGTTTTCA	0.57132973	0.31232	0.279387	0.555136
tsrna-06259	AGCGGTTAGGATTCCTGGTTTTC	0.92841081	0.23424	0	0
tsrna-06258	AGCGGTTAGGATTCCTGGTTTT	0.07141622	0.23424	0.069847	0.111027
tsrna-06257	AGCGGTTAGGATTCCTGGTTT	0.28566487	0.23424	0.069847	0.222054
tsrna-06256	AGCGGTTAGGATTCCTGGTT	0.14283243	0.390401	0	0
tsrna-06255	AGCGGTTAGGATTCCTGGT	0.14283243	0.546561	0.069847	0
tsrna-06254	AGCGGTTAGGATTCCTGG	0.28566487	0.15616	0.069847	0.111027
tsrna-06253	AGCGGTTAGGATTCCTG	0	0	0.069847	0
tsrna-06252	AGCGGTCTAAGGCGCTGGATT	0.14283243	0.07808	0.069847	0.111027

tsrna-06250	AGCGGTCTAAGGCGCTGCGTTC	0	0	0	0
tsrna-06248	AGCGGTAGAGCATTTGACTGC	0	0.15616	0.069847	0.111027
tsrna-06247	AGCGGTAGAGCATTTGACTG	0	0	0.069847	0
tsrna-06242	AGCGGAAGCGTGCTGGGCCC	0.57132973	2.810884	0.349234	0.333081
tsrna-06241	AGCGGAAGCGTGCTGGGCC	0.28566487	1.561602	0.069847	0
tsrna-06240	AGCGCGTTCGGCTGTTAACCGAAAGGTTGG	0.64274595	3.045124	0.908008	1.221299
tsrna-06239	AGCGCGTTCGGCTGTTAACCGAAAGGT	0.64274595	1.327362	0.488928	0
tsrna-06238	AGCGCGTTCGGCTGTTAACCGAAAGG	0.64274595	1.249282	0.349234	0.222054
tsrna-06237	AGCGCGTTCGGCTGTTAACCGAAAG	0.64274595	1.405442	0.20954	0.222054
tsrna-06236	AGCGCGTTCGGCTGTTAACCGAAA	0.8569946	1.093122	0.488928	0.444109
tsrna-06235	AGCGCGTTCGGCTGTTAACCGA	0.21424865	0.390401	0.279387	0
tsrna-06234	AGCGCGTTCGGCTGTTAACCG	0.35708108	1.171202	0.139694	0
tsrna-06233	AGCGCGTTCGGCTGTTAACC	0.07141622	0	0.069847	0
tsrna-06227	AGCGCCGCTGGTGTAGTGGTATCATGCAAG	0.49991351	1.327362	1.117549	0.333081
tsrna-06222	AGCGATCCGAGTTCAAATCTCGGTGGAACCTC	0	0.23424	0	0
tsrna-06218	AGCCTCCGGAGCTGGGGATTGTGGGT	0.07141622	0.23424	0.069847	0
tsrna-06217	AGCCTCCGGAGCTGGGGATTGTGGG	0	0.07808	0	0
tsrna-06215	AGCCTCACCTGGAGCACCA	0.07141622	0.07808	0.069847	0
tsrna-06214	AGCCGTGATCGTATAGTGGTTAGTACTCTGCGTTGT	2.35673514	1.093122	1.117549	0.555136
tsrna-06213	AGCCGTGATCGTATAGTGGTTAGTACTCTGCGTTG	2.49956757	1.483522	0.908008	0.444109
tsrna-06212	AGCCGTGATCGTATAGTGGTTAGTACTCTGCGTT	1.64257298	0.468481	0.698468	0.333081
tsrna-06211	AGCCGTGATCGTATAGTGGTTAGTACTCTGCGT	0.14283243	0.23424	0.20954	0.111027
tsrna-06210	AGCCGTGATCGTATAGTGGTTAGTACTCTGCG	0.35708108	0.23424	0.139694	0.111027
tsrna-06209	AGCCGTGATCGTATAGTGGTTAGTACTCTGC	0.14283243	0.23424	0.20954	0.222054
tsrna-06208	AGCCGTGATCGTATAGTGGTTAGTACTCTG	0.14283243	0.07808	0	0
tsrna-06207	AGCCGTGATCGTATAGTGGTTAGTACTCT	0.35708108	0	0	0
tsrna-06205	AGCCGTGATCGTATAGTGGTTAGTACT	0.07141622	0.07808	0	0
tsrna-06196	AGCCGTGATCGTATAGTG	0	0	0	0
tsrna-06194	AGCCCTCAGTAAGTTGCAATACTTAATTTCTGCCA	0.07141622	0.07808	0.139694	0.111027
tsrna-06192	AGCCCTCAGTAAGTTGCAATACT	0	0	0.069847	0
tsrna-06186	AGCCCGGTAATCGCATAAAACTTAAAACTT	0.21424865	0.07808	0.20954	0
tsrna-06181	AGCCCGGCTAGCTCAGTCGGTAGAGCATG	0.14283243	0.31232	0.139694	0.444109
tsrna-06180	AGCCCGGCTAGCTCAGTCGGTAGAG	0.14283243	0.546561	0.069847	0
tsrna-06179	AGCCCGGCTAGCTCAGTCGG	0.07141622	0.07808	0	0.333081
tsrna-06178	AGCCCGGCTAGCTCAG	0.07141622	0	0	0
tsrna-06177	AGCCCGGATAGCTCAGTCGGTAGAGCATCAGACTTTT	97.4117192	57.3108	102.605	20.54002
tsrna-06176	AGCCCGGATAGCTCAGTCGGTAGAGCATCAGACTTT	86.6992867	56.68616	97.78553	21.76132
tsrna-06175	AGCCCGGATAGCTCAGTCGGTAGAGCATC	1.21407568	0.390401	1.047702	0.333081
tsrna-06174	AGCCCGGATAGCTCAGTCGGTAGAGCA	0.28566487	0.15616	0.139694	0.444109
tsrna-06173	AGCCCGGATAGCTCAGTCGGTAGAG	0.28566487	0.15616	0.20954	0.444109
tsrna-06172	AGCCCGGATAGCTCAGTCGGTAGA	0.21424865	0.468481	0.349234	0.555136
tsrna-06171	AGCCCGGATAGCTCAGTCGGTAG	0.07141622	0.23424	0.139694	0.222054
tsrna-06170	AGCCCGGATAGCTCAGTCGGTA	0.35708108	0.15616	0	0
tsrna-06169	AGCCCGGATAGCTCAGTCGG	0.07141622	0.390401	0	0
tsrna-06166	AGCCCCAGTGGAACCACCA	0.14283243	0.15616	0.069847	0
tsrna-06164	AGCCCCACGTTGGGCGCCA	0.49991351	2.576644	0.349234	0.555136
tsrna-06163	AGCCCACCCAGGGACGCCA	0.21424865	3.904006	0.069847	0.111027
tsrna-06161	AGCCAGGGATTGTGGGTTCGAGTCCCATCTGGGGTGCCA	0.49991351	1.717762	0.279387	0
tsrna-06160	AGCCAGGGATTGTGGGTTCGAGTCCCATCTGGGGTGCC	0.4284973	0.780801	0.419081	0.111027
tsrna-06159	AGCCAGGGATTGTGGGTTCGAGTCCCACCCGGGGTA	0.4284973	0.702721	0.279387	0.222054
tsrna-06158	AGCCAGGGATTGTGGGTTCGAGTCCC	0.35708108	0.780801	0.069847	0
tsrna-06157	AGCCAGGGATTGTGGGTTCG	0	0.624641	0.20954	0.111027
tsrna-06156	AGCCAGGGATTGTGGGTTC	0.07141622	0.624641	0.069847	0.111027
tsrna-06155	AGCCAGGGATTGTGGGTT	0.28566487	0.702721	0.069847	0
tsrna-06154	AGCCAGGGATTGTGGGT	0.07141622	0.702721	0.069847	0
tsrna-06150	AGCATTGGTGGTTCAGTGGTAGAATTCTCGCCT	307.018314	48.17543	360.3397	38.19334
tsrna-06149	AGCATTGGTGGTTCAGTGGTAGAATTCTCG	3.64222704	0.858881	3.771728	6.106493
tsrna-06148	AGCATTGGTGGTTCAGTGGTAGAATTCTC	3.14231352	1.171202	2.514485	4.66314
tsrna-06147	AGCATTGGTGGTTCAGTGGTAGAATTCT	1.07124325	1.639682	1.187396	3.330814
tsrna-06146	AGCATTGGTGGTTCAGTGGTAGA	0.57132973	0.780801	0.20954	0.555136
tsrna-06145	AGCATTGGTGGTTCAGTGGT	0.14283243	0.390401	0.069847	0
tsrna-06144	AGCATTGGACTGTAAATCTAAAGACAGGGGTTAGGC	0	0	0	0
tsrna-06143	AGCATTGGACTGTAAATCTAAAGACAGGGG	0	0.07808	0	0
tsrna-06142	AGCATTGGACTGTAAATCTAAAGACAGG	0.07141622	0	0	0

tsrna-06141	AGCATTGGACTGTAAATCTAAAGACA	0.07141622	0	0	0
tsrna-06138	AGCATTGGACTGTAAATCTAAAG	0	0.07808	0	0
tsrna-06136	AGCATTGGACTGTAAATCTAA	0	0	0	0
tsrna-06135	AGCATTGGACTGTAAATCTA	0.07141622	0	0	0
tsrna-06133	AGCATTGGACTGTAAATC	0	0.07808	0	0
tsrna-06130	AGCATTAACCTTTTAAGTTAAAGATTAAGAGAACC	0.07141622	0.07808	0.20954	0.111027
tsrna-06126	AGCATGGGTGGTTCAGTGGTAGAATTCTCG	1.57115676	1.561602	1.74617	3.88595
tsrna-06125	AGCATGGGTGGTTCAGTGGTAGAATTCT	1.2854919	1.093122	0.628621	2.553624
tsrna-06124	AGCATGGGTGGTTCAGTGGTAGA	0.35708108	0.468481	0.139694	0.333081
tsrna-06123	AGCATGGGACTCTTAATCCC	0.07141622	0.07808	0	0
tsrna-06122	AGCATGGGACTCTTAATCC	0.07141622	0.07808	0	0
tsrna-06120	AGCATGGGACTCTTAAT	0	0.07808	0	0
tsrna-06118	AGCATGCACGAGGCCCCGGGT	0	0.15616	0	0
tsrna-06117	AGCATGAGACTCTTAATCT	0.21424865	0.15616	0	0.333081
tsrna-06116	AGCATAGCTGCCTTCCAAGCAGTTGACCCG	0.07141622	0.15616	0.20954	0
tsrna-06115	AGCATAGCTGCCTTCCAAG	0	0	0.069847	0
tsrna-06114	AGCATAGCTGCCTTCCAA	0	0	0	0
tsrna-06112	AGCAGTTGACCCGGGTTCGATTCCCGGCCAACG	0.07141622	0.07808	0	0
tsrna-06111	AGCAGTTGACCCGGGTTCGATTCCCGGCC	0	0.23424	0	0
tsrna-06110	AGCAGTTGACCCGGGTTCGA	0	0	0.069847	0.111027
tsrna-06109	AGCAGTTGACCCGGGTTCG	0.07141622	0.07808	0.069847	0
tsrna-06108	AGCAGTTGACCCGGGTTC	0	0.23424	0.069847	0
tsrna-06107	AGCAGTTGACCCGGGTT	0.07141622	0	0	0
tsrna-06104	AGCAGAGTGGCGCAGCGGAAGCGTGCTGGGCCCATA	0.71416216	5.075207	0.558774	0.555136
tsrna-06103	AGCAGAGTGGCGCAGCGGAAGCGTGCTGGGCCCAT	0.99982703	5.153287	0.349234	0.77719
tsrna-06102	AGCAGAGTGGCGCAGCGGAAGCGTGCTGGGCCCA	0.4284973	5.465608	0.488928	0.77719
tsrna-06101	AGCAGAGTGGCGCAGCGGAAGCGTGCTGGGCCC	0.57132973	3.904006	0.279387	0.77719
tsrna-06100	AGCAGAGTGGCGCAGCGGAAGCGTGCTGGGCC	0.14283243	2.654724	0.279387	0.111027
tsrna-06099	AGCAGAGTGGCGCAGCGGAAGCGTGCTGGGC	0.21424865	1.717762	0.20954	0.111027
tsrna-06098	AGCAGAGTGGCGCAGCGGAAGCGTGCTGGG	0.21424865	1.405442	0.069847	0.111027
tsrna-06097	AGCAGAGTGGCGCAGCGGAAGCGTGCTGG	0.07141622	0.23424	0	0.111027
tsrna-06095	AGCAGAGTGGCGCAGCGGAAGCGTGCT	0	0.07808	0	0
tsrna-06082	AGCACTCTGGACTTTGAATCCAGC	0.14283243	0.15616	0.20954	0
tsrna-06081	AGCACTCTGGACTTTGAATCCAG	0	0.07808	0.069847	0
tsrna-06080	AGCACTCTGGACTTTGAATCCA	0.28566487	0.390401	0.069847	0.111027
tsrna-06079	AGCACTCTGGACTTTGAATCC	0.21424865	0.15616	0.20954	0
tsrna-06078	AGCACTCTGGACTTTGAATC	0.14283243	0.31232	0.069847	0.111027
tsrna-06073	AGCACTCTGGACTCTGAATCCAGCGATCCGAGTTCA	0.14283243	0.23424	0	0
tsrna-06072	AGCACTCTGGACTCTGAATCCAGCGATCCGAGTTC	0.07141622	0.23424	0.20954	0
tsrna-06071	AGCACTCTGGACTCTGAATCCAGCGATCCGAGTT	0	0.468481	0	0
tsrna-06070	AGCACTCTGGACTCTGAATCCAGCGATCCGAGT	0.14283243	0.31232	0.069847	0
tsrna-06069	AGCACTCTGGACTCTGAATCCAGCGATCCGAG	0.21424865	0.390401	0	0
tsrna-06068	AGCACTCTGGACTCTGAATCCAGCGATCCG	0.07141622	0.07808	0.069847	0
tsrna-06067	AGCACTCTGGACTCTGAATCCAGCGATCC	0.21424865	0.31232	0	0.111027
tsrna-06066	AGCACTCTGGACTCTGAATCCAGCGATC	0.07141622	0.23424	0.069847	0
tsrna-06065	AGCACTCTGGACTCTGAATCCAGCGAT	0.07141622	0.07808	0	0
tsrna-06064	AGCACTCTGGACTCTGAATCCAGCGA	0.07141622	0.23424	0.139694	0.111027
tsrna-06063	AGCACTCTGGACTCTGAATCCAGCG	0.14283243	0.07808	0	0
tsrna-06062	AGCACTCTGGACTCTGAATCCAGC	0	0.07808	0	0
tsrna-06061	AGCACTCTGGACTCTGAATCCAG	0.14283243	0.390401	0.139694	0
tsrna-06060	AGCACTCTGGACTCTGAATCCA	0.07141622	0.31232	0.069847	0
tsrna-06059	AGCACTCTGGACTCTGAATCC	0	0.15616	0.069847	0
tsrna-06058	AGCACTCTGGACTCTGAATC	0	0.15616	0	0.111027
tsrna-06052	AGCACCCAACTTACACTTAGGAGATTTCAA	0	0	0	0
tsrna-06051	AGCACCCAACTTACACTTAGGAGATTTCA	0	0	0.069847	0
tsrna-06049	AGCACCCAACTTACACTTAGGAGATTT	0	0	0	0
tsrna-06047	AGCACCCAACTTACACTTAGGAGAT	0	0	0	0
tsrna-06046	AGCACCCAACTTACACTTAGGAGA	0	0	0	0
tsrna-06037	AGCAATACACTGAAAATGTTTAGACGGGCTC	0.4284973	0.858881	0.139694	0
tsrna-06036	AGCAATACACTGAAAATGTTTAGACGGGCT	0.14283243	0.31232	0.139694	0
tsrna-06035	AGATTTCAACTTAACTTGACCGCTCTGACCA	4.99913515	3.591685	3.771728	1.887461
tsrna-06034	AGATTTCAACTTAACTTGACCGCTCTGACC	1.07124325	0.546561	0.488928	0
tsrna-06033	AGATTTCAACTTAACTTGACCGCTCTGAC	0.14283243	0.390401	0.069847	0
tsrna-06032	AGATTTCAACTTAACTTGACCGCTCTGA	0.14283243	0.07808	0	0

tsrna-06031	AGATTTCAACTTAACTTGACCGCTCTG	0	0.15616	0	0
tsrna-06030	AGATTTCAACTTAACTTGACCGCTCT	0	0	0	0
tsrna-06029	AGATTTCAACTTAACTTGACCGCTC	0.07141622	0	0	0
tsrna-06028	AGATTTCAACTTAACTTGACCGCT	0	0	0	0
tsrna-06024	AGATTTCAACTTAACTTGAC	0	0.07808	0	0
tsrna-06020	AGATTGTGAATCTGACAACAGAGGCTTACGACCCCTTATTTA(	1.92823784	0.858881	4.330502	0.111027
tsrna-06019	AGATTGTGAATCTGACAACAGAGGCTTACGACCCCTTATTTA(	2.28531893	1.405442	5.02897	0.111027
tsrna-06018	AGATTGTGAATCTGACAACAGAGGCTTACGACCCCTTATTTA(	2.99948109	1.717762	5.587744	0
tsrna-06017	AGATTGTGAATCTGACAACAGAGGCTTACGACCCCTTATTTA(	2.14248649	1.171202	4.260655	0.111027
tsrna-06016	AGATTGTGAATCTGACAACAGAGGCTTACGACCCCTT	0.57132973	0.858881	0.838162	0.111027
tsrna-06015	AGATTGTGAATCTGACAACAGAGGCTTACGACCCCT	0.28566487	0.624641	0.558774	0.111027
tsrna-06014	AGATTGTGAATCTGACAACAGAGGCTTACGACCCC	0.07141622	0.15616	0.349234	0
tsrna-06013	AGATTGTGAATCTGACAACAGAGGCTTACGACCC	0.14283243	0.468481	0.20954	0
tsrna-06012	AGATTGTGAATCTGACAACAGAGGCTTACGACC	0.21424865	0.31232	0.279387	0
tsrna-06011	AGATTGTGAATCTGACAACAGAGGCTTACGA	0.21424865	0.390401	0.20954	0.111027
tsrna-06010	AGATTGTGAATCTGACAACAGAGGCTTACG	0.28566487	0.23424	0.279387	0.111027
tsrna-06009	AGATTGTGAATCTGACAACAGAGGCTTAC	0.07141622	0.31232	0.419081	0.111027
tsrna-06008	AGATTGTGAATCTGACAACAGAGGCTT	0	0.468481	0.20954	0
tsrna-06007	AGATTGTGAATCTGACAACAGAGGCT	0.21424865	0.31232	0.488928	0
tsrna-06006	AGATTGTGAATCTGACAACAGAGGC	0.14283243	0.468481	0.20954	0
tsrna-06005	AGATTGTGAATCTGACAACAGAGG	0.07141622	0	0.20954	0
tsrna-06004	AGATTGTGAATCTGACAACAGAG	0.14283243	0.31232	0.139694	0
tsrna-06003	AGATTGTGAATCTGACAACAGA	0.14283243	0.23424	0.069847	0
tsrna-06002	AGATTGTGAATCTGACAACAG	0	0	0.069847	0.222054
tsrna-06001	AGATTGTGAATCTGACAACA	0.07141622	0.15616	0.069847	0
tsrna-06000	AGATTGTGAATCTGACAAC	0.07141622	0.23424	0.20954	0
tsrna-05999	AGATTGTGAATCTGACAA	0	0	0.069847	0
tsrna-05998	AGATTGTGAATCTGACA	0	0	0.069847	0
tsrna-05996	AGATTGCAGGTTCGAGTCCTGCCGCGGTCGCC	0	0.07808	0	0
tsrna-05994	AGATTGAGGGTTCGAGTCCCTTCGTGGTCGCCA	0	0.23424	0.069847	0
tsrna-05993	AGATTGAGGGTTCGAGTCCCTTCGTGGTCGCC	0	0.23424	0.069847	0
tsrna-05992	AGATTGAGGGTTCGAGTCCCTTCGTGGTCGC	0	0	0	0
tsrna-05991	AGATTGAGGGTTCGAGTCCCTTCGTGGTCG	0	0.07808	0	0
tsrna-05990	AGATTGAGGGTTCGAGTC	0.07141622	0	0.069847	0
tsrna-05988	AGATTGAAGCCAGTTGATTAGGGTGCTTAG	0	0	0.069847	0
tsrna-05987	AGATTGAAGCCAGTTGATTAGGG	0.14283243	0	0.069847	0
tsrna-05985	AGATTCTAGGTTCGACTCCTGGCTGGCTCG	0	0.07808	0	0
tsrna-05984	AGATTCTAGGTTCGACTCCTG	0	0.23424	0	0
tsrna-05983	AGATTCTAGGTTCGACTCC	0	0.23424	0	0
tsrna-05982	AGATTCCCATTCTTGCGACCCGGGTTCG	0.21424865	0.936961	0.069847	0.111027
tsrna-05981	AGATTCCCATTCTTGCGACCCGGGT	0	0.390401	0.069847	0
tsrna-05980	AGATTCCCATTCTTGCGACCCGGG	0	0.31232	0	0
tsrna-05979	AGATTCCCATTCTTGCGACCCGG	0	0	0	0
tsrna-05978	AGATTCCCATTCTTGCGACCCG	0	0.07808	0	0
tsrna-05977	AGATTCCCATTCTTGCGACCC	0.07141622	0.07808	0	0
tsrna-05976	AGATTCCCATTCTTGCGACC	0.07141622	0.07808	0	0
tsrna-05975	AGATTCCCATTCTTGCGAC	0	0.23424	0	0
tsrna-05973	AGATTCCAGGTTCGACTCCTGGC	0.07141622	0	0	0
tsrna-05967	AGATTAAGAGAACCAACACCTCTTTACAGTGACCA	0	0.07808	0.069847	0.111027
tsrna-05966	AGATTAAGAGAACCAACACCTCTTTACAGTGACC	0.07141622	0.07808	0	0
tsrna-05965	AGATTAAGAGAACCAACACCTCTTTACAGTGAC	0	0.07808	0	0
tsrna-05964	AGATTAAGAGAACCAACACCTCTTTACAGT	0	0.07808	0	0
tsrna-05963	AGATTAAGAGAACCAACACCTCTTTACAG	0	0.07808	0	0
tsrna-05960	AGATTAAGAGAACCAACACCTCTTTA	0	0	0	0
tsrna-05959	AGATTAAGAGAACCAACACCTCT	0	0	0	0
tsrna-05958	AGATTAAGAGAACCAACACCTC	0	0	0	0
tsrna-05956	AGATTAAGAGAACCAACACC	0	0	0.069847	0
tsrna-05949	AGATGGCAGAGCCCGGTAATCG	0	0	0	0
tsrna-05945	AGATGAAAACCTTTTTCCAAGGACACCA	0.4284973	0.07808	0.139694	0
tsrna-05944	AGATGAAAACCTTTTTCCAAGGACACC	0	0	0	0
tsrna-05939	AGATCTAAAGGTCCCTGGT	0.78557838	1.093122	0.488928	0.111027
tsrna-05937	AGATCCTTAGGTCGCTGGT	0	0.15616	0	0
tsrna-05933	AGATCAGAAGGTTGCGTGTTCAAGTCACGTCGGGGTCACC	0.35708108	0.23424	0.069847	0.111027
tsrna-05932	AGATCAGAAGGCTGCGTGTTCGAA	0	0	0.069847	0

tsrna-05931	AGATCAGAAGGCTGCGTGTTCGA	0	0.07808	0	0
tsrna-05930	AGATCAGAAGGCTGCGTGTTCG	0	0.07808	0	0
tsrna-05929	AGATCAGAAGGCTGCGTGTTC	0.07141622	0	0.069847	0
tsrna-05928	AGATCAGAAGGCTGCGTGTT	0	0	0.069847	0
tsrna-05927	AGATCAGAAGGCTGCGTGT	0.07141622	0	0	0
tsrna-05925	AGATCAAGAGGTCCCTGGTT	0.21424865	0.468481	0	0.444109
tsrna-05924	AGATCAAGAGGTCCCTGGT	0.14283243	0.546561	0.069847	0.111027
tsrna-05923	AGATCAAGAGGTCCCCGGTTCA	0.14283243	0.31232	0.069847	0
tsrna-05922	AGATCAAGAGGTCCCCGGTTC	0.14283243	0.23424	0	0
tsrna-05921	AGATCAAGAGGTCCCCGGTT	0.14283243	0.468481	0	0.111027
tsrna-05920	AGATCAAGAGGTCCCCGGT	0.14283243	0.468481	0.139694	0
tsrna-05914	AGATCAAAAGGTCCCTGGT	0.35708108	0.624641	0.20954	0.222054
tsrna-05912	AGAGTTCAAGTCTCACTGGG	0	0.07808	0	0
tsrna-05909	AGAGTGTAGCTTAACACAAAGCACCCAACT	0	0.07808	0	0
tsrna-05908	AGAGTGTAGCTTAACACA	0	0	0	0
tsrna-05907	AGAGTGGGGTTTTGCAGTCCTTACCA	0.07141622	0.07808	0.069847	0
tsrna-05906	AGAGTGGGGTTTTGCAGTCCTTACC	0	0	0	0.111027
tsrna-05905	AGAGTGGGGTTTTGCAGTCCT	0	0	0	0
tsrna-05904	AGAGTGGGGTTTTGCAGTCC	0	0	0	0
tsrna-05902	AGAGTGGGGTTTTGCAGT	0	0	0.069847	0
tsrna-05899	AGAGTGGCGCAGCGGAAGCGTGCTGGGCCC	0.57132973	3.513605	0.419081	0.666163
tsrna-05898	AGAGTGGCGCAGCGGAAGCGTGCTGGGCC	0.14283243	2.420483	0.139694	0.111027
tsrna-05897	AGAGTGGCGCAGCGGAAGCGTGCTGGGC	0.21424865	1.483522	0.069847	0.111027
tsrna-05896	AGAGTGGCGCAGCGGAAGCGTGCTGGG	0.14283243	1.639682	0	0.111027
tsrna-05895	AGAGTGGCGCAGCGGAAGCGTGCTGG	0.07141622	0.31232	0	0
tsrna-05887	AGAGTAAATAATAGGAGCTT	0	0	0	0
tsrna-05886	AGAGTAAATAATAGGAGCT	0	0	0	0
tsrna-05880	AGAGGTCGATGGATCGAAAC	0	0.07808	0	0
tsrna-05876	AGAGGTCCCGGGTTCAAATCCC	0	0.07808	0	0.111027
tsrna-05875	AGAGGTCCCGGGTTCAAATC	0	0.07808	0	0
tsrna-05874	AGAGGTCCCGGGTTCAAA	0	0	0	0
tsrna-05872	AGAGGCTTACGACCCCTTATTTACCCCA	0.14283243	0.15616	0.069847	0
tsrna-05871	AGAGGCTTACGACCCCTTATTTACCCC	0	0	0	0
tsrna-05863	AGAGCCTTCAAAGCCCTCAGT	0	0.07808	0	0
tsrna-05860	AGAGCCCGGTAATCGCATAAAACTTAAAAC	0.07141622	0	0.069847	0
tsrna-05859	AGAGCCCGGTAATCGCATAAAACTTA	0.07141622	0	0	0
tsrna-05856	AGAGCCCGGTAATCGCATAA	0	0	0.069847	0
tsrna-05851	AGAGCATGGGACTCTTAATCCCAG	0.14283243	0.07808	0.069847	0.111027
tsrna-05850	AGAGCATGGGACTCTTAATC	0	0	0.069847	0
tsrna-05849	AGAGCATGGGACTCTT	0	0	0	0.111027
tsrna-05848	AGAGAATAGTTTAAATTAGAATCTTAGCTT	0.07141622	0.15616	0.139694	0.111027
tsrna-05846	AGAGAACCAACACCTCTTTACAGTGACCA	0.07141622	0.07808	0	0
tsrna-05836	AGACTTTTTCTCTGACCA	0.14283243	0.624641	0.488928	0.222054
tsrna-05835	AGACTTTTTCTCTGACC	0	0.15616	0	0
tsrna-05834	AGACTTTTTCTCTGAC	0.07141622	0	0	0
tsrna-05833	AGACTTTTAATCTGAGGGTCCAGGGT	1.21407568	3.513605	0.628621	0.444109
tsrna-05832	AGACTTTTAATCTGAGGGTCCAG	0.71416216	2.732804	0.628621	0.444109
tsrna-05831	AGACTTTTAATCTGAGGGTCC	0.35708108	1.249282	0.349234	0.333081
tsrna-05830	AGACTGAAGATCTAAAGGTCCCTGGT	0.92841081	1.249282	0.419081	0.333081
tsrna-05829	AGACTCAAGTTCTGGTCTCCGGA	0.14283243	0.390401	0.069847	0
tsrna-05828	AGACTCAAGTTCTGGTCTCCGG	0.14283243	0.31232	0.069847	0
tsrna-05827	AGACTCAAGTTCTGGTCTCC	0	0.23424	0	0
tsrna-05826	AGACGGGCTCACATCACCCCATAAACACCA	1.2854919	0.546561	0.349234	1.665407
tsrna-05825	AGACGGGCTCACATCACCCCATAAACACC	0.4284973	0	0.139694	0
tsrna-05824	AGACGGGCTCACATCACCCCATAAACA	0.07141622	0	0.069847	0
tsrna-05823	AGACGGGCTCACATCACCCCATA	0	0	0.069847	0
tsrna-05822	AGACGGGCTCACATCACCCC	0	0	0.069847	0
tsrna-05819	AGACGGGCTCACATCAC	0.07141622	0	0	0
tsrna-05817	AGACCTCGTGGCGCAATGG	0.07141622	0	0	0
tsrna-05815	AGACCGGGGTTCGATTCCCCGACGGGGAGCCA	0	1.093122	0	0
tsrna-05814	AGACCGGGGTTCGATTCCCCGACGGGGAGCC	0	0.23424	0	0
tsrna-05813	AGACCGGGGTTCGATTCCCCGACGGGGAGC	0	0.07808	0	0
tsrna-05812	AGACCGGGGTTCGATTCCCCGACGGGGAG	0	0	0	0.111027
tsrna-05809	AGACCGGGGTTCGATTCCCCGACGGG	0	0	0	0

tsrna-05798	AGACCAAGAGCCTTCAAAGC	0	0	0.069847	0
tsrna-05797	AGACCAAGAGCCTTCAAAG	0.07141622	0.07808	0	0
tsrna-05795	AGACAGGGGTTAGGCCTCTTTTTACCACC	0.07141622	0	0	0
tsrna-05794	AGACAGGGGTTAGGCCTCTTTTTACCAC	0	0	0.069847	0
tsrna-05793	AGACAGGGGTTAGGCCTCTTTTTACCA	0	0.07808	0.069847	0
tsrna-05792	AGACAGGGGTTAGGCCTCTTTTTACC	0	0	0.069847	0
tsrna-05791	AGACAGGGGTTAGGCCTCTTT	0.07141622	0	0	0
tsrna-05790	AGACAGGGGTTAGGCCTCTT	0	0	0	0
tsrna-05789	AGACAGGGGTTAGGCCTCT	0.07141622	0	0	0
tsrna-05788	AGAATTCTCGCCTGCCACGCGGGAGGCCCG	0.8569946	1.015041	0.069847	0.111027
tsrna-05787	AGAATTCTCGCCTGCCACGCGGG	0.57132973	1.093122	0	0.111027
tsrna-05786	AGAATTCTCGCCTGCCACGCGG	0.35708108	0.936961	0.20954	0.222054
tsrna-05785	AGAATTCTCGCCTGCCACGCG	0.28566487	1.093122	0.20954	0.111027
tsrna-05784	AGAATTCTCGCCTGCCACGC	0.07141622	0.936961	0.139694	0.111027
tsrna-05783	AGAATTCTCGCCTGCCACG	0.4284973	0.468481	0.419081	0.111027
tsrna-05782	AGAATTCTCGCCTGCCAC	0.07141622	0.07808	0	0
tsrna-05781	AGAATTCTCGCCTGCCA	0.28566487	0	0.069847	0
tsrna-05779	AGAATTCTCGCCTCCCACG	0	0	0	0.111027
tsrna-05778	AGAATGGCCGAGTGGTTAAGGCGTTGGACTTAA	0.07141622	0	0	0
tsrna-05775	AGAATCTTAGCTTTGGGTGCTAATGGTGGAGTTAAAGACT	0.35708108	0.390401	0.139694	0.222054
tsrna-05774	AGAATCTTAGCTTTGGGTGCTAATGGTGGAGTTAAA	0.35708108	0	0.139694	0.111027
tsrna-05773	AGAATCTTAGCTTTGGGTGCTAATGGTGG	0	0.07808	0	0
tsrna-05770	AGAATCTTAGCTTTGGGTGC	0	0	0	0
tsrna-05766	AGAATAGTTTAAATTAGAATCTTAGCTTTG	0.35708108	0.23424	0.139694	0.222054
tsrna-05765	AGAAGGTTGCGTGTTCA	0	0.15616	0	0.111027
tsrna-05764	AGAAGGTCCTGGGTTCGAGCCCCAGTGGAACCACCA	2.71381622	2.420483	1.187396	1.776434
tsrna-05763	AGAAGGTCCTGGGTTCGAGCCCCAGTGGA	0.14283243	0	0	0
tsrna-05761	AGAAGGCTGCGTGTTCG	0.07141622	0	0	0
tsrna-05759	AGAAGCAGCTTCAAACCTGCCGGGGCTTCC	0	0	0.069847	0
tsrna-05758	AGAAGCAGCTTCAAACCTGCCGGGGCTT	0	0	0	0
tsrna-05757	AGAAGCAGCTTCAAACCTGCCGGGGCT	0	0	0	0
tsrna-05755	AGAAGCAGCTTCAAACCTGCCGGGG	0	0.07808	0	0
tsrna-05752	AGAAGATTGCAGGTTCGAGTCCTGCCGCGGTCGCCA	0.49991351	0.546561	0.069847	0
tsrna-05749	AGAAGATTGAGGGTTCGAGTCCCTTCGTGGTCGCCA	0.28566487	1.327362	0.20954	0.444109
tsrna-05748	AGAAGATTGAGGGTTCGAGTCCCTTCGTGGTCGCC	0.21424865	0.546561	0.349234	0.111027
tsrna-05747	AGAAGATTGAGGGTTCGAGTCCCTTCGT	0.35708108	0.390401	0.069847	0.111027
tsrna-05746	AGAAGATTGAGGGTTCG	0.07141622	0.23424	0.139694	0.222054
tsrna-05745	AGAAGATTGAGGGTTC	0.28566487	0.546561	0	0
tsrna-05744	AGAAGATTCTAGGTTCGACTCCTG	0.07141622	0.546561	0.069847	0.111027
tsrna-05743	AGAAGATTCTAGGTTC	0.07141622	0.07808	0.069847	0
tsrna-05742	AGAAGATTCCAGGTTCGACTCCTGG	0.21424865	0.15616	0	0
tsrna-05741	AGAAGATTCCAGGTTCG	0	0.23424	0	0
tsrna-05740	AGAAGATTCCAGGTTC	0.07141622	0.07808	0	0
tsrna-05739	AGAACTGCTAACTCATGCCCCCATGTCTAACAACATGGCTTT(	0.21424865	0.07808	0.558774	0.111027
tsrna-05738	AGAACTGCTAACTCATGCCCCCATGTCTAACAACATGGCTTT(	0.14283243	0.07808	0.488928	0
tsrna-05737	AGAACTGCTAACTCATGCCCCCATGTCTAACAACATGGCT	0.07141622	0.07808	0.139694	0
tsrna-05736	AGAACTGCTAACTCATGCCCCCATGTCTAACAACATGGC	0.07141622	0	0.139694	0
tsrna-05735	AGAACTGCTAACTCATGCCCCCATGTCTAACAACATGG	0	0.07808	0.20954	0
tsrna-05734	AGAACTGCTAACTCATGCCCCCATGTCTAACAACAT	0	0.07808	0	0
tsrna-05733	AGAACTGCTAACTCATGCCCCCATGTCTAACAACA	0	0	0.069847	0
tsrna-05732	AGAACTGCTAACTCATGCCCCCATGTCTAACAA	0	0	0.069847	0
tsrna-05731	AGAACTGCTAACTCATGCCCCCATGTCTAACA	0.07141622	0	0	0.111027
tsrna-05730	AGAACTGCTAACTCATGCCCCCATGTCTAAC	0	0	0	0
tsrna-05729	AGAACTGCTAACTCATGCCCCCATGTCTAA	0.07141622	0.07808	0	0
tsrna-05728	AGAACTGCTAACTCATGCCCCCATGTCTA	0	0	0	0.111027
tsrna-05727	AGAACTGCTAACTCATGCCCCCATGTCT	0	0	0.069847	0
tsrna-05726	AGAACTGCTAACTCATGCCCCCATGTC	0.07141622	0.07808	0.069847	0
tsrna-05725	AGAACTGCTAACTCATGCCCCCATGT	0	0.07808	0	0
tsrna-05724	AGAACTGCTAACTCATGCCCCCATG	0	0.07808	0	0
tsrna-05723	AGAACTGCTAACTCATGCCCCCAT	0	0	0	0
tsrna-05721	AGAACTGCTAACTCATGCCCCC	0	0	0	0
tsrna-05720	AGAACTGCTAACTCATGCCCC	0	0	0	0
tsrna-05717	AGAACTGCTAACTCATGC	0	0.07808	0	0
tsrna-05710	AGAACCAACACCTCTTTACAGTG	0	0	0	0

tsrna-05705	AGAAATTTAGGTTAAATACAGACCAAGAGC	0.07141622	0.624641	0.139694	0.222054
tsrna-05704	AGAAATTTAGGTTAAATACAGACCAAGA	0	0.702721	0	0.111027
tsrna-05703	AGAAATTTAGGTTAAATACAGACCAAG	0.07141622	0.546561	0	0
tsrna-05702	AGAAATTTAGGTTAAATACAGACC	0.07141622	0.31232	0	0
tsrna-05701	AGAAATTTAGGTTAAATACAGAC	0.21424865	0.546561	0	0
tsrna-05700	AGAAATTTAGGTTAAATACAG	0	0.468481	0	0.222054
tsrna-05699	AGAAATTTAGGTTAAATACA	0.07141622	0.624641	0	0
tsrna-05698	AGAAATTTAGGTTAAATAC	0	0.936961	0	0.222054
tsrna-05695	AGAAATATGTCTGATAAAAGAGTTACTTTGATAGAGTAAATAA	0.99982703	1.561602	1.257243	0.111027
tsrna-05694	AGAAATATGTCTGATAAAAGAGTTACTTTGATAGAG	0.35708108	0.624641	0.558774	0
tsrna-05693	AGAAATATGTCTGATAAAAGAGTTACTTTGAT	0.14283243	0.15616	0.139694	0
tsrna-05692	AGAAATATGTCTGATAAAAGAGTTACTTTG	0	0.15616	0.20954	0
tsrna-05690	AGAAATATGTCTGATAAAAGAGTTA	0	0.23424	0.139694	0
tsrna-05689	AGAAATATGTCTGATAAAAGAGT	0	0	0	0
tsrna-05681	AGAAAGCTCACAAGAACTGCTAACTCATGCCCCCATGTCTAA	1.2854919	3.201285	0.838162	1.221299
tsrna-05680	AGAAAGCTCACAAGAACTGCTAACTCATGCCCCCATGTCTAA	1.21407568	2.967044	1.047702	1.110271
tsrna-05679	AGAAAGCTCACAAGAACTGCTAACTCATGCCCCCAT	1.14265946	2.732804	1.047702	0.999244
tsrna-05678	AGAAAGCTCACAAGAACTGCTAACTCATGCC	0.78557838	1.717762	0.628621	0.555136
tsrna-05677	AGAAAGCTCACAAGAACTGCTAACTCATGC	1.35690811	1.795843	0.698468	0.888217
tsrna-05676	AGAAAGCTCACAAGAACTGCTAACTCATG	0.49991351	2.732804	1.047702	1.110271
tsrna-05675	AGAAAGCTCACAAGAACTGCTAACTCA	0.71416216	1.873923	0.628621	0.77719
tsrna-05674	AGAAAGCTCACAAGAACTGCTAACT	0.71416216	2.186243	0.768315	0.77719
tsrna-05673	AGAAAGCTCACAAGAACTGCTAAC	0.78557838	2.264323	0.419081	0.888217
tsrna-05672	AGAAAGCTCACAAGAACTGCTAA	0.28566487	0.31232	0.419081	0.111027
tsrna-05671	AGAAAGCTCACAAGAACTGCTA	0.35708108	1.093122	0.279387	0.555136
tsrna-05670	AGAAAGCTCACAAGAACTGCT	0.35708108	0.624641	0.279387	0
tsrna-05669	AGAAAGCTCACAAGAACTGC	0.07141622	0.390401	0	0
tsrna-05668	AGAAAGCTCACAAGAACTG	0	0	0.139694	0
tsrna-05667	AGAAAGCTCACAAGAACT	0	0.23424	0	0.111027
tsrna-05665	AGAAAAAGTCATGGAGGCCA	1.35690811	6.94913	0.488928	0.888217
tsrna-05664	ACTTTTTCTCTGACCA	0.07141622	0.07808	0.139694	0.111027
tsrna-05663	ACTTTTAATCTGAGGGTCCAGGGTTCAAGTCCCTGTTCGGGC	1.57115676	7.02721	1.327089	0.888217
tsrna-05662	ACTTTTAATCTGAGGGTCCAGGGT	0.99982703	3.747845	0.488928	0.444109
tsrna-05661	ACTTTTAATCTGAGGGTCCAG	0.57132973	2.342403	0.279387	0.333081
tsrna-05660	ACTTTTAATCTGAGGGTCCA	1.07124325	2.108163	0.558774	0.333081
tsrna-05659	ACTTTTAATCTGAGGGTCC	0.64274595	1.249282	0.279387	0
tsrna-05658	ACTTTTAAAGGATAACAGCTATCCATTGGTCTTAGGCCCCA	0.49991351	0.07808	0.628621	0.111027
tsrna-05657	ACTTTTAAAGGATAACAGCTATCCATTGGTCTTAGGC	0	0	0.069847	0.111027
tsrna-05656	ACTTTTAAAGGATAACAGCTATCCATTGGTCTTAGG	0.14283243	0.07808	0.069847	0.222054
tsrna-05655	ACTTTTAAAGGATAACAGCTATCCATTGGTCTT	0	0.07808	0.139694	0.333081
tsrna-05654	ACTTTTAAAGGATAACAGCTATCCATTGGTCT	0	0	0.139694	0.222054
tsrna-05653	ACTTTTAAAGGATAACAGCTATCCATTGGTC	0.07141622	0	0	0
tsrna-05652	ACTTTTAAAGGATAACAGCTATCCATTGGT	0.14283243	0	0	0.333081
tsrna-05651	ACTTTTAAAGGATAACAGCTATCCATTGG	0	0	0.069847	0.111027
tsrna-05650	ACTTTTAAAGGATAACAGCTATCCATTG	0	0.15616	0	0
tsrna-05649	ACTTTTAAAGGATAACAGCTATCCATT	0	0.07808	0	0.222054
tsrna-05648	ACTTTTAAAGGATAACAGCTATCCA	0.07141622	0.07808	0	0.111027
tsrna-05647	ACTTTTAAAGGATAACAGCTATCC	0.14283243	0	0.069847	0.111027
tsrna-05646	ACTTTTAAAGGATAACAGCTAT	0	0	0.069847	0
tsrna-05645	ACTTTTAAAGGATAACAGCTA	0	0	0.139694	0.111027
tsrna-05644	ACTTTTAAAGGATAACAGCT	0	0	0.139694	0.222054
tsrna-05639	ACTTTGTCAAAGTTAAATTATAGGCTAAATCCTATATATCTTAC	1.2854919	0.15616	1.885864	0
tsrna-05638	ACTTTGTCAAAGTTAAATTATAGGCTAA	0.28566487	0.07808	0.279387	0.111027
tsrna-05637	ACTTTGTCAAAGTTAAATTATAGGCT	0.21424865	0.07808	0.558774	0
tsrna-05636	ACTTTGTCAAAGTTAAATTATAGGC	0.21424865	0.07808	0.20954	0
tsrna-05635	ACTTTGTCAAAGTTAAATTATAGG	0	0.15616	0.139694	0
tsrna-05634	ACTTTGTCAAAGTTAAATTATAG	0.07141622	0	0.069847	0
tsrna-05633	ACTTTGTCAAAGTTAAATT	0	0	0.069847	0
tsrna-05630	ACTTTGATAGAGTAAATAATAGGAGCTTAAACCCCCT	0.49991351	0.15616	0.279387	0
tsrna-05629	ACTTTGATAGAGTAAATAATAGGAGCTTAAACCCCC	0.49991351	0	0.139694	0
tsrna-05628	ACTTTGATAGAGTAAATAATAGGAGCTTAAACCCC	0.28566487	0	0.069847	0.111027
tsrna-05627	ACTTTGATAGAGTAAATAATAGGAGCTTAAACCC	0.07141622	0	0.279387	0.111027
tsrna-05626	ACTTTGATAGAGTAAATAATAGGAGCTTAAACC	0.07141622	0	0.139694	0.111027
tsrna-05625	ACTTTGATAGAGTAAATAATAGGAGCTTA	0.07141622	0.07808	0	0

tsrna-05624	ACTTTGATAGAGTAAATAATAGGAGCTT	0	0.07808	0	0
tsrna-05623	ACTTTGATAGAGTAAATAATAGGAGCT	0	0	0.139694	0.111027
tsrna-05622	ACTTTGATAGAGTAAATAATAGGAGC	0.14283243	0	0	0
tsrna-05618	ACTTTGAATCCAGCGATCCGAGTTCAA	0.28566487	0	0.20954	0
tsrna-05617	ACTTTGAATCCAGCGATCCGAGTTC	0	0.15616	0.20954	0
tsrna-05616	ACTTTGAATCCAGCGATCCGAGTT	0.21424865	0.468481	0	0
tsrna-05615	ACTTTGAATCCAGCGATCCGAGT	0	0.07808	0.069847	0.111027
tsrna-05614	ACTTTGAATCCAGCGATCCGAG	0.07141622	0	0	0
tsrna-05613	ACTTTGAATCCAGCGATCCGA	0.35708108	0.390401	0	0
tsrna-05612	ACTTTGAATCCAGCGATCCG	0	0	0.069847	0
tsrna-05611	ACTTTGAATCCAGCGATCC	0	0.07808	0.069847	0
tsrna-05610	ACTTTGAATCCAGCGATC	0.14283243	0	0.139694	0.222054
tsrna-05609	ACTTTGAATCCAGCGA	0.07141622	0	0.139694	0
tsrna-05608	ACTTTGAATCCAGCAATCCGAGT	0.21424865	1.093122	0.419081	0.888217
tsrna-05607	ACTTTGAATCCAGCAATCCGAG	0.14283243	0	0.139694	0.111027
tsrna-05606	ACTTTGAATCCAGCAATCCGA	0	0.15616	0.069847	0.111027
tsrna-05605	ACTTTGAATCCAGCAATCCG	0.21424865	0.31232	0.139694	0.111027
tsrna-05603	ACTTTACAGTCAGAGGTTC	0.07141622	0	0	0
tsrna-05602	ACTTTACAGTCAGAGGTT	0	0.07808	0	0
tsrna-05600	ACTTGACCGCTCTGACCA	0.4284973	0.23424	0	0.111027
tsrna-05597	ACTTCTAATTCAAAGGTTCCGGGT	0	0.15616	0.139694	0
tsrna-05593	ACTTCGGATCAGAAGATTGAGGGT	0.14283243	0.390401	0.069847	0.222054
tsrna-05587	ACTTATAATGCCGAGGTTGTGAGT	1.14265946	1.327362	0.698468	1.110271
tsrna-05586	ACTTAGGAGATTTCAACTTAACTTGACCGCTCTGACCA	6.0703784	4.762887	5.308357	1.887461
tsrna-05585	ACTTAGGAGATTTCAACTTAACTTGACCGCTCTGACC	1.14265946	0.702721	0.768315	0.222054
tsrna-05584	ACTTAGGAGATTTCAACTTAACTTGACCGCTCTGAC	0.28566487	0.468481	0	0.111027
tsrna-05583	ACTTAGGAGATTTCAACTTAACTTGACCGCTCTGA	0.07141622	0.07808	0.20954	0.333081
tsrna-05582	ACTTAGGAGATTTCAACTTAACTTGACCGCTCTG	0.14283243	0.15616	0.069847	0
tsrna-05581	ACTTAGGAGATTTCAACTTAACTTGACCGCT	0.07141622	0	0	0
tsrna-05580	ACTTAGGAGATTTCAACTTAACTTGACCGC	0	0	0.069847	0
tsrna-05579	ACTTAGGAGATTTCAACTTAACTTGACCG	0.07141622	0.23424	0	0
tsrna-05578	ACTTAGGAGATTTCAACTTAACTTGACC	0	0.15616	0	0
tsrna-05576	ACTTAGGAGATTTCAACTTAACT	0	0	0	0
tsrna-05569	ACTTACACTTAGGAGATTTCAACTTAACTTGACCGCTCTGACC	5.42763245	4.294406	6.006825	1.665407
tsrna-05568	ACTTACACTTAGGAGATTTCAACTTAACTTGACCGCTCTGACC	0.99982703	0.936961	1.117549	0.111027
tsrna-05567	ACTTACACTTAGGAGATTTCAACTTAACTTGACCGCTCTGAC	0.4284973	0.23424	0.838162	0.333081
tsrna-05566	ACTTACACTTAGGAGATTTCAACTTAACTTGACCGCTCTGA	0.14283243	0.23424	0.349234	0
tsrna-05565	ACTTACACTTAGGAGATTTCAACTTAACTTGACCGCTCTG	0.14283243	0.07808	0.279387	0
tsrna-05564	ACTTACACTTAGGAGATTTCAACTTAACTTGACCGCTCT	0.28566487	0.07808	0.20954	0
tsrna-05563	ACTTACACTTAGGAGATTTCAACTTAACTTGACCGCTC	0	0	0.349234	0
tsrna-05562	ACTTACACTTAGGAGATTTCAACTTAACTTGACCGCT	0.21424865	0	0.488928	0
tsrna-05561	ACTTACACTTAGGAGATTTCAACTTAACTTGACCGC	0.21424865	0	0.139694	0
tsrna-05560	ACTTACACTTAGGAGATTTCAACTTAACTTGACCG	0.07141622	0.07808	0.279387	0
tsrna-05559	ACTTACACTTAGGAGATTTCAACTTAACTTGACC	0.14283243	0	0.139694	0
tsrna-05558	ACTTACACTTAGGAGATTTCAACTTAACTT	0.14283243	0	0.069847	0
tsrna-05557	ACTTACACTTAGGAGATTTCAACTTAACT	0	0	0	0
tsrna-05556	ACTTACACTTAGGAGATTTCAACTTA	0	0	0	0
tsrna-05555	ACTTACACTTAGGAGATTTCAACTT	0	0	0	0
tsrna-05554	ACTTACACTTAGGAGATTTCAACT	0	0	0	0
tsrna-05545	ACTTAAGATCCAATGGACATATGT	0	0	0	0
tsrna-05539	ACTTAACTTGACCGCTCTGACCA	0.64274595	1.405442	1.327089	0.333081
tsrna-05538	ACTTAACTTGACCGCTCTGACC	0.14283243	0.23424	0.069847	0
tsrna-05537	ACTTAACTTGACCGCTCTGAC	0	0.07808	0.069847	0
tsrna-05536	ACTTAACTTGACCGCTCTGA	0	0.07808	0	0
tsrna-05535	ACTTAACTTGACCGCTCTG	0	0	0	0
tsrna-05534	ACTTAACTTGACCGCTCT	0	0	0	0
tsrna-05531	ACTTAAAACTTTACAGTCAGAGGTTC	0	0.07808	0.069847	0
tsrna-05528	ACTTAAAACTTTACAGTCAGAG	0	0.07808	0	0
tsrna-05522	ACTGTAGATCCTTAGGTCGCTGGTTCGATT	0.14283243	0.07808	0.069847	0.111027
tsrna-05521	ACTGTAGATCCTTAGGTCGCTGGTT	0	0	0	0.111027
tsrna-05520	ACTGTAGATCCTTAGGTCGCTGGT	0	0.07808	0	0
tsrna-05514	ACTGTAAATCTAAAGACAGGGGTTAGGCCT	0.14283243	0	0.069847	0
tsrna-05513	ACTGTAAATCTAAAGACAGGGGTTAGGC	0	0	0	0
tsrna-05502	ACTGTAAAGCTAACTTAGCATTAACC	0	0	0	0

tsrna-05501	ACTGTAAAGCTAACTTAGCATTAAC	0	0	0	0.111027
tsrna-05499	ACTGTAAAGCTAACTTAGCATT	0	0.07808	0	0
tsrna-05498	ACTGTAAAGCTAACTTAGCA	0	0	0	0
tsrna-05493	ACTGGTGGTTCAGTGGTAGAATTCTCGCCT	0.49991351	0.624641	0.069847	0.333081
tsrna-05492	ACTGGTGGTTCAGTGGTAGAATTCTCGC	0.28566487	0.31232	0.139694	0
tsrna-05491	ACTGGTGGTTCAGTGGTAGAATTCTCG	0.28566487	0.390401	0	0
tsrna-05490	ACTGGTGGTTCAGTGGTAGAATTCTC	0.21424865	0.23424	0.139694	0
tsrna-05489	ACTGGTGGTTCAGTGGTAGAATTCT	0	0.31232	0.069847	0.111027
tsrna-05488	ACTGGGCGGAAACACCA	0.07141622	0.23424	0.069847	0
tsrna-05486	ACTGCTAACTCATGCCCCCATGTCTAACAACATGGCTTTCTC/	0.92841081	0.390401	3.003413	0
tsrna-05485	ACTGCTAACTCATGCCCCCATGTCTAACAACATGGCTTTCTC/	0.78557838	0.468481	1.74617	0.111027
tsrna-05484	ACTGCTAACTCATGCCCCCATGTCTAACAACATGGCT	0	0.07808	0	0
tsrna-05483	ACTGCTAACTCATGCCCCCATGTCTAACAA	0	0	0.069847	0
tsrna-05481	ACTGCTAACTCATGCCCCCATGTCTAAC	0	0.07808	0	0
tsrna-05478	ACTGCTAACTCATGCCCCCATGT	0	0.07808	0	0
tsrna-05475	ACTGCTAACTCATGCCCCCA	0	0	0	0
tsrna-05470	ACTGCAGATCAAGAGGTCCCCGGT	0	0.23424	0	0.111027
tsrna-05466	ACTGAAGATCTAAAGGTCCCTGGT	0.64274595	1.639682	0.558774	0
tsrna-05464	ACTGAAAATGTTTAGACGGGCTCACATCAC	5.14196758	2.498564	2.584332	0.333081
tsrna-05463	ACTGAAAATGTTTAGACGGGCTCACATCA	3.71364325	3.123204	2.025557	1.221299
tsrna-05462	ACTGAAAATGTTTAGACGGGCTCACA	2.64240001	1.795843	0.977855	0.555136
tsrna-05461	ACTGAAAATGTTTAGACGGGCTCAC	2.14248649	1.639682	0.349234	0.333081
tsrna-05460	ACTGAAAATGTTTAGACGGGCTCA	0.78557838	0.936961	0	0.222054
tsrna-05459	ACTGAAAATGTTTAGACGGGCTC	0.71416216	0.780801	0.349234	0
tsrna-05458	ACTGAAAATGTTTAGACGGGCT	0.07141622	0.23424	0	0.111027
tsrna-05457	ACTGAAAATGTTTAGACGGGC	0.07141622	0	0	0
tsrna-05456	ACTGAAAATGTTTAGACGGG	0.07141622	0.07808	0	0
tsrna-05455	ACTGAAAATGTTTAGACGG	0	0.15616	0	0
tsrna-05453	ACTCTTTTAGTATAAATAGTACCGTTAACTTCC	0.21424865	0	0.488928	0
tsrna-05452	ACTCTTTTAGTATAAATAGTACCGTTAACT	0	0	0.139694	0
tsrna-05440	ACTCTTAATCTCAGGGTCGTGGGT	0.4284973	0.858881	0.139694	0.444109
tsrna-05439	ACTCTTAATCTCAGGGTCGTGG	0.07141622	0.702721	0.139694	0.222054
tsrna-05438	ACTCTTAATCTCAGGGTCGTG	0.21424865	0.546561	0.139694	0.333081
tsrna-05436	ACTCTTAATCCCAGGGTCGTGG	0	0	0	0
tsrna-05435	ACTCTTAATCCCAGGGTCGTG	0	0.07808	0	0
tsrna-05434	ACTCTTAATCCCAGGG	0	0.15616	0	0
tsrna-05432	ACTCTGGACTTTGAATCCA	0.07141622	0.07808	0	0
tsrna-05431	ACTCTGGACTTTGAATCC	0.07141622	0.07808	0	0
tsrna-05430	ACTCTGGACTTTGAATC	0	0	0	0
tsrna-05428	ACTCTGGACTCTGAATCCGGTA	0	0.15616	0	0
tsrna-05427	ACTCTGGACTCTGAATCCGGT	0	0.23424	0	0
tsrna-05426	ACTCTGGACTCTGAATCCGG	0	0.15616	0	0
tsrna-05425	ACTCTGGACTCTGAATCCAGCGATCCGAGTTCA	0.07141622	0.23424	0	0
tsrna-05424	ACTCTGGACTCTGAATCCAGCGATCCGAGTTC	0.28566487	0.468481	0.069847	0.333081
tsrna-05423	ACTCTGGACTCTGAATCCAGCGATCCGAGT	0.28566487	0.15616	0	0
tsrna-05422	ACTCTGGACTCTGAATCCAGCGATCCGAG	0.28566487	0.15616	0	0.222054
tsrna-05421	ACTCTGGACTCTGAATCCAGCGATCCGA	0	0.07808	0	0.111027
tsrna-05420	ACTCTGGACTCTGAATCCAGCGATCCG	0.21424865	0.07808	0.069847	0.111027
tsrna-05419	ACTCTGGACTCTGAATCCAGCGATCC	0	0.23424	0	0
tsrna-05418	ACTCTGGACTCTGAATCCAGCGATC	0	0.23424	0	0
tsrna-05417	ACTCTGGACTCTGAATCCAGCGAT	0.07141622	0.31232	0	0
tsrna-05416	ACTCTGGACTCTGAATCCAGCGA	0.07141622	0.23424	0.069847	0
tsrna-05415	ACTCTGGACTCTGAATCCAGCG	0	0	0	0
tsrna-05414	ACTCTGGACTCTGAATCCAGC	0.07141622	0.23424	0	0.111027
tsrna-05413	ACTCTGGACTCTGAATCCAG	0.07141622	0.15616	0.069847	0.111027
tsrna-05412	ACTCTGGACTCTGAATCCA	0.07141622	0.07808	0	0
tsrna-05411	ACTCTGGACTCTGAATCC	0	0	0	0
tsrna-05410	ACTCTGGACTCTGAATC	0	0.07808	0	0
tsrna-05402	ACTCTGAATCCAGCGATCCGAGTTCAA	0	0.07808	0.069847	0
tsrna-05401	ACTCTGAATCCAGCGATCCGAGTTC	0	0.23424	0	0
tsrna-05400	ACTCTGAATCCAGCGATCCGAGTT	0	0	0	0
tsrna-05397	ACTCTGAATCCAGCGATCCGA	0.07141622	0.07808	0	0
tsrna-05396	ACTCTGAATCCAGCGATCCG	0	0	0	0
tsrna-05390	ACTCCTGGCTGGCTCGCCA	1.14265946	5.231367	0.558774	0.222054

[truncated: 1,718,996 more chars]
